# BUI1 coordinates actin cytoskeleton remodeling and ROS homeostasis to confer broad-spectrum disease resistance in rice

**DOI:** 10.1007/s44154-026-00321-5

**Published:** 2026-07-02

**Authors:** Hui Lin, Kaixuan Cui, Jingyi Wang, Yanlong Jin, Xi Zhang, Yiduo Lu, Xuanxuan Wu, Li Zhang, Shasha Liu, Jiyun Liu, Qun Li, Yiwen Deng, Weibing Yang, Zuhua He, Mingjun Gao

**Affiliations:** 1 State Key Laboratory of Plant Trait Design, CAS Center for Excellence in Molecular Plant Sciences, Chinese Academy of Sciences, Shanghai, 200032 China; 2 Shanghai Agricultural Technology Extension and Service Center, Shanghai, 201103 China; 3 Ministry of Education Key Laboratory for Biodiversity, Science and Ecological Engineering, National Observations and Research Station for Wetland Ecosystems of the Yangtze Estuary, School of Life Sciences, Fudan University, Shanghai, 200433 China; 4 College of Agriculture and Biotechnology, Zhejiang University, Hangzhou, 310058 China; 5 Key Laboratory of Plant Stress Biology, State Key Laboratory of Cotton Biology, School of Life Sciences, Henan University, Kaifeng, 475004 China

**Keywords:** BUI1, Actin cytoskeleton, Susceptibility, ROS, *rod1*

## Abstract

The cytoskeleton functions as a dynamic intracellular structural scaffold, enabling cells to rapidly respond to biotic and abiotic stresses. During plant innate immunity, the cytoskeleton undergoes significant actin remodeling. Using the rice*-Magnaporthe oryzae* pathosystem, we demonstrate that pattern-triggered immunity (PTI) induces a rapid and transient actin filament reorganization dependent on the Class II formin protein BENT UPPERMOST INTERNODE1 (BUI1). Disruption of actin filaments using latrunculin B significantly enhances host susceptibility to the blast fungus *M. oryzae*. Importantly, the *bui1* mutant exhibits compromised resistance not only to blast but also to the bacterial blight pathogen *Xanthomonas oryzae* pv. *oryzae* and the sheath blight fungus *Rhizoctonia solani*. We identify that this enhanced susceptibility to multiple pathogens stems from increased ROS scavenging activity in *bui1*, mediated by upregulation of peroxidase-encoding genes and consequent reduction in H₂O₂ accumulation. Furthermore, genetic analyses reveal that BUI1 acts downstream of the immunity suppressor RESISTANCE OF RICE TO DISEASES1 (ROD1), as the *bui1* mutation compromises *rod1*-mediated broad-spectrum resistance. Our findings establish BUI1 as a key regulator linking cytoskeletal reorganization to receptor-mediated immune signaling and ROS homeostasis during plant-pathogen interactions, providing new insights into the integration of actin dynamics with plant defense mechanisms.

**Supplementary Information:**

The online version contains supplementary material available at 10.1007/s44154-026-00321-5.

## Introduction

Rice (*Oryza sativa*) is a stable food crop that supports a large portion of the global population. However, its productivity and security are severely threatened by various destructive microbial pathogens, including the hemibiotrophic fungus *Magnaporthe oryzae* (*M. oryzae*) causing rice blast disease, the bacterial pathogen *Xanthomonas oryzae* pv. *oryzae* (*Xoo*) underlying bacterial blight, and the necrotrophic fungus *Rhizoctonia solani* (*R. solani*) inducing sheath blight (Nino-Liu al., 2006; Zhang et al. 2016; Senapati et al. 2022). To counter pathogen invasion, plants have evolved highly efficient immune systems capable of rapidly detecting microbial molecules such as pathogen-associated molecular patterns (PAMPs) (Dodds and Rathjen 2010). Recognition of PAMPs by cell-surface pattern recognition receptors activates pattern-triggered immunity (PTI), which initiates a cascade of defense responses against pathogen attacks (Jones & Dangl 2006; Tang et al. 2017). Given the continual threat of these diseases, increasing attention has been directed toward enhancing basal resistance by identifying PTI-related genes that confer durable and broad-spectrum resistance.

The rice-*M. oryzae* pathosystem serves as a well-established model for studying plant-pathogen interactions. During infection, the fungus deposits asexual spores (conidia) on the hydrophobic rice leaf surface. These spores develop into specialized infection structures called appressoria, which generate turgor pressure through glycerol accumulation, enabling mechanical penetration of the plant cuticle. Appressorium formation is tightly regulated by cell cycle progression, autophagy, and metabolic control mechanisms mediated by TOR kinase and the PKA pathway (de et al., 1997; Sun et al. 2019a). The host cytoskeleton and cell wall at the infection site are critical barriers to fungal entry (Hardham et al. 2007; Sinha et al. 2024). However, the specific mechanisms coordinating systematic cytoskeletal remodeling with immune signaling during pathogen invasion remain unclear. Understanding how plant defense is integrated with cytoskeletal dynamics is essential for developing effective strategies to control rice blast and other major diseases.

The cytoskeleton, a dynamic network composed of microtubules and actin filaments, plays a pivotal role in mediating cellular responses to both beneficial and pathogenic microbes (Takemoto and Hardham 2004; Basu et al. 2008; Li and Staiger 2018). In plant immunity, pathogen infection and PAMPs perception trigger rapid and extensive actin remodeling, involving changes in filament density, orientation and bundling through polymerization and depolymerization cycles (Sinha et al. 2024). Disrupting the cytoskeleton, either genetically or chemically, often increases plant susceptibility to pathogens. For instance, treatment with actin polymerization inhibitors like cytochalasin or latrunculin B (LatB) induced actin depolymerization and enhanced susceptibility to *Pseudomonas syringae* (Kang et al. 2014). Pharmacological and cytochemical studies further supported the connection between actin dynamics and defense responses, demonstrating that actin filament polymerization is critical for effective immunity (Takemoto and Hardham 2004). Notably, during PTI response, actin bundles accumulate at fungal or oomycete infection sites (Henty-Ridilla et al. 2014; Li et al. 2015). Moreover, AtPRF3, a negative regulator of formin-mediated actin assembly, was found to enhance PAMP-triggered immunity, including a substantial increase in the production of reactive oxygen species (ROS), which plays a crucial role in plant defense (Sun et al. 2018). Chitin-induced phosphorylation of CERK1 also facilitated cytoskeleton-dependent endocytosis of LYK5 (Cao et al. 2014). However, the precise mechanisms by which actin remodeling is orchestrated during host–pathogen interactions remain to be fully elucidated.

In our previous studies, the rice actin-binding protein BUI1 (BENT UPPERMOST INTERNODE1), also termed RMD or OsFH5, was identified as a class II formin that modulates actin organization and rice morphogenesis (Yang et al. 2011). Loss of RMD function results in growth defects such as bent uppermost internodes, dwarfism, wavy panicles, and abnormal seed morphology, largely attributed to impaired cell elongation and disrupted microtubule-microfilament arrays (Zhang et al. 2011). RMD also participates in an auxin-actin regulatory loop governed by the auxin response factors OsARF23 and OsARF24, thereby influencing cell growth (Li et al. 2014). Moreover, RMD also regulated crown root angle and was transcriptionally upregulated under low phosphate conditions (Huang et al. 2018). More Recently, the OsERF34-RMD cascade was shown to promote secondary cell wall thickening by enhancing cellulose and lignin biosynthesis, thereby improving stem mechanical strength and grain yield, highlighting its potential as a target for crop breeding (Zhang et al. 2022). Despite these advances, the role of BUI1/RMD/OsFH5 in plant defense, particularly whether BUI1-mediated actin remodeling contributes to immune signaling and how it interfaces with pathogen perception and downstream responses are key unresolved questions.

In this study, we demonstrate that BUI1 plays a pivotal role in rice immunity by integrating actin cytoskeleton remodeling with immune signaling. Using the rice-*M. oryzae* as a model, we show that BUI1 is essential for PAMP-triggered actin reorganization and contributes to basal resistance against multiple pathogens. Loss of *BUI1* function not only increases susceptibility to *M. oryzae*, *Xoo*, and *R. solani* but also compromises the broad-spectrum resistance conferred by *rod1*. Our findings uncover BUI1 as a regulatory hub that links cytoskeletal dynamics to receptor-mediated signaling and ROS homeostasis, providing new insights into the integration of actin remodeling and plant defense.

## Results

### Actin polymerization is required for blast resistance in rice

**Fig. 1:**
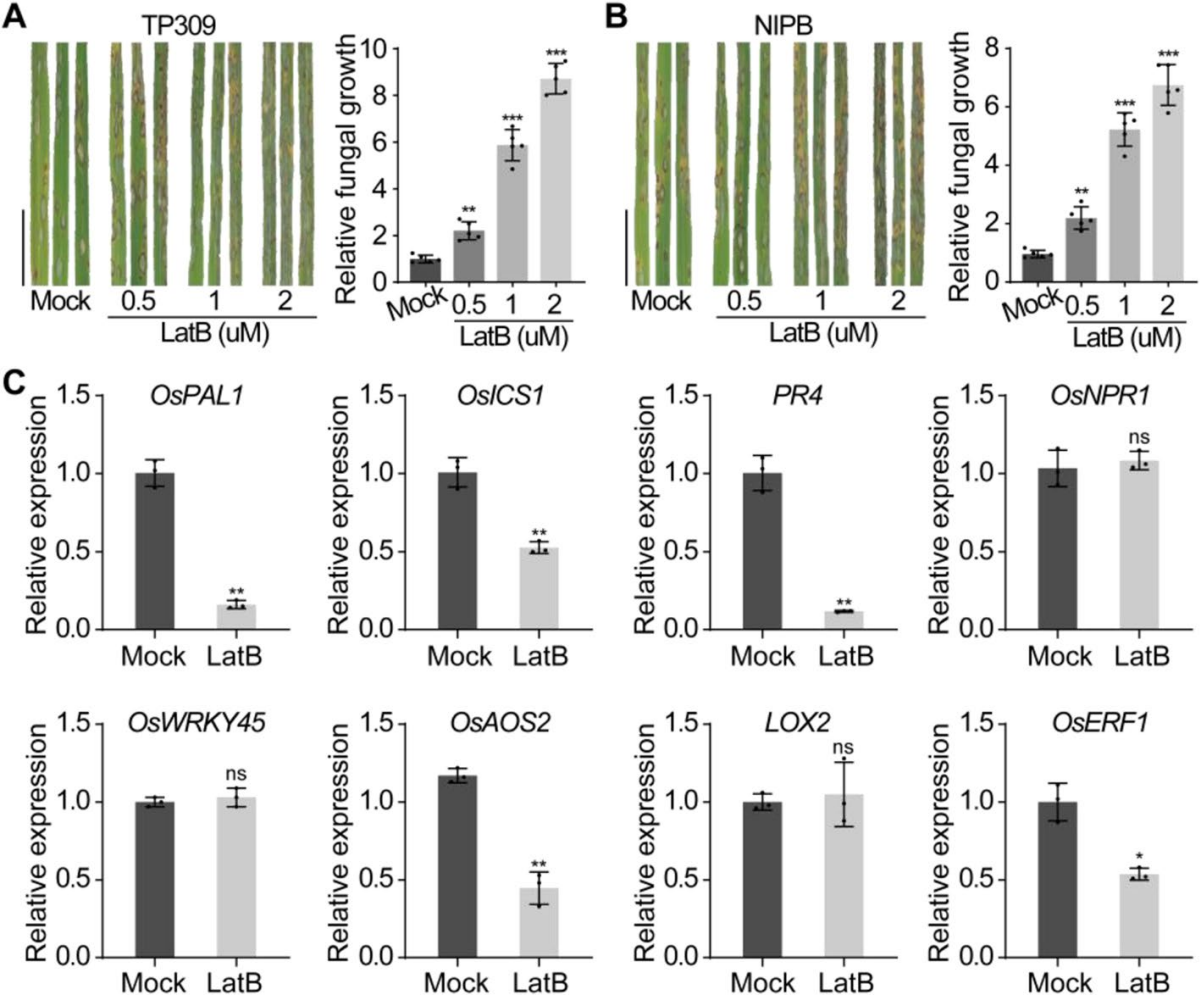
Actin polymerization regulates rice blast resistance. **A**, **B**. Blast susceptibility of *Japonica* rice varieties TP309 (**A**) and NIPB (**B**) at 7 days post-inoculation (dpi) following spray inoculation. Two-week-old seedlings were pretreated with 0.5, 1 or 2 µM latrunculin B (LatB) or mock (0.05% DMSO) for 12 h, then inoculated with *M. oryzae* isolate TH12. LatB-treated plants exhibited enhanced susceptibility compared to controls. Relative fungal growth was quantified by qRT-PCR. Scale bars, 1 cm. **C**. Treatment with 1 uM LatB regulates the expression of defense-responsive genes, including those in the salicylic acid (SA) pathway (*OsPAL1*, *OsICS1*, *PR4*) and the jasmonic acid/ethylene (JA/ET) pathway (*OsERF1*, *OsAOS2*). Data were shown as mean ± SD, *n* = 5 (**A**, **B**) and *n* = 3 (**C**). Asterisks represented statistical significance (**P < 0.01, ***P < 0.001, two-tailed Student’s t-test). ns, not significant. Experiments were independently repeated three times with similar results

The actin cytoskeleton is a key component of plant pathogen-associated molecular pattern (PAMP)-triggered immunity (PTI), particularly in reinforcing penetration resistance during pathogen attack. To assess its role in rice, we used Latrunculin B (LatB), a macrolide compound derived from marine sponges, that inhibits actin polymerization by binding to monomeric actin and preventing filament elongation (Morton et al. 2000). When rice seedlings of TP309 and Nipponbare (NIPB) were pretreated with LatB before spray-inoculation with the virulent *Magnaporthe oryzae* isolate TH12, they exhibited increased susceptibility to blast infection, with the higher concentration causing the most pronounced effect (Fig. 1A and B). These findings indicate that actin polymerization and filament abundance in host cells are essential for basal resistance.

To further explore the relationship between actin dynamics and immune signaling, we examined the effects of LatB-mediated actin depolymerization on defense responses, which typically involve hormone signaling pathways and the induction of pathogenesis-related genes. qRT-PCR analysis revealed that LatB treatment broadly suppresses multiple defense hormone pathways. Specifically, it inhibited genes in the salicylic acid (SA) pathway, including *OsPAL1* (Phenylalanine Ammonia-Lyase 1), *OsICS1* (Isochorismate Synthase 1), and *PR4* (Pathogenesis-Related Protein 4), as well as genes in the jasmonic acid/ethylene (JA/ET) pathway, such as *OsAOS2* (Allene Oxide Synthase 2), and *OsERF1* (Ethylene Response Factor 1) (Fig. 1C). Together, these results indicated that actin depolymerization compromised host immunity.

### *BUI1* mutation reduces resistance to rice blast

**Fig. 2:**
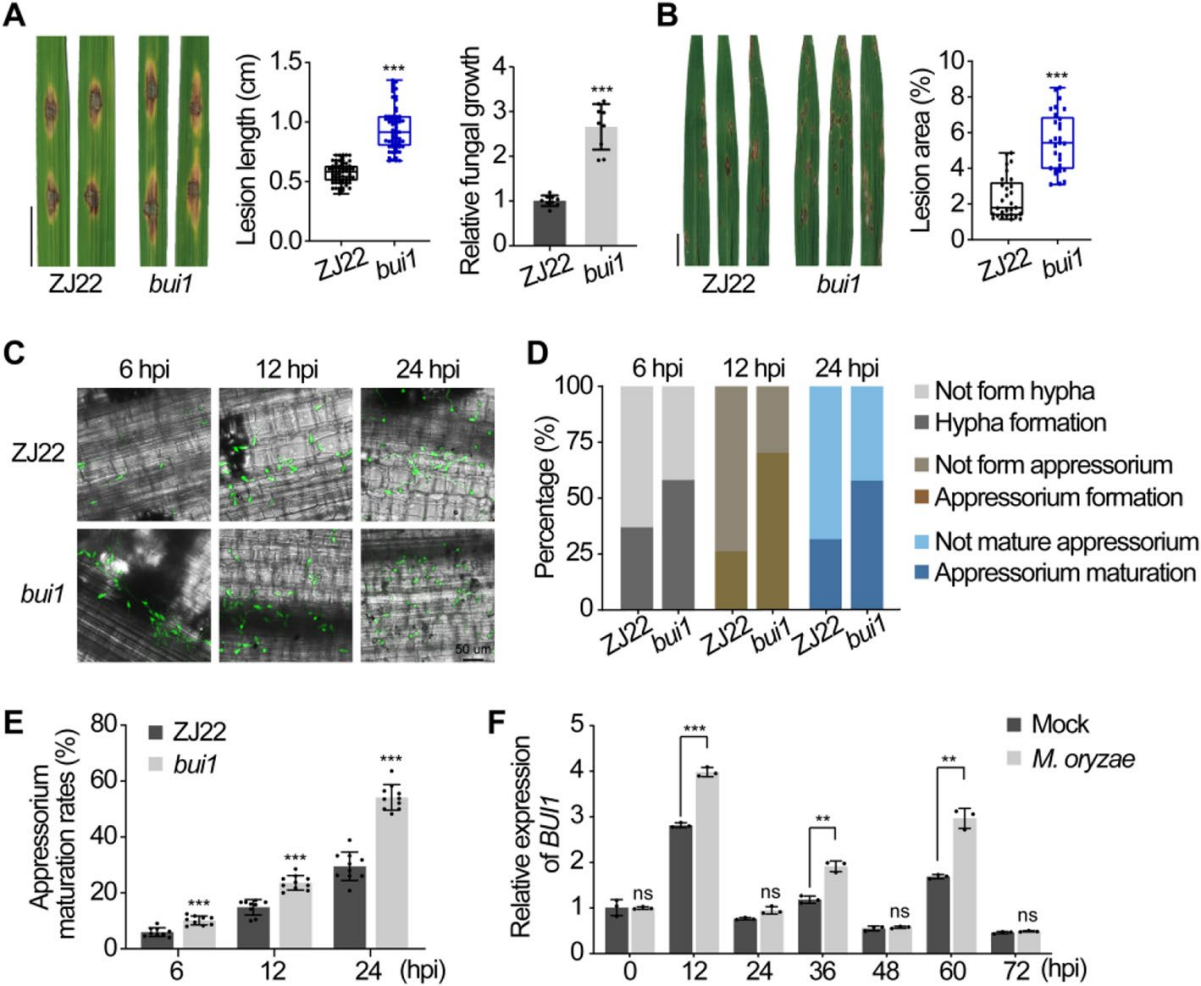
BUI1 positively regulates rice blast resistance. **A**. Disease phenotypes of the ZJ22 and *bui1* mutant at 7 dpi by punch inoculation with *M. oryzae* isolate TH12. Lesion length was measured and relative fungal growth was quantified by qRT-PCR. **B**. Field resistance evaluation in blast nursery. The *bui1* mutant showed significantly reduced resistance compared to the moderately resistant variety ZJ22, as demonstrated by increased lesion area. **C**. Infection process monitoring in leaf sheaths inoculated with GFP-tagged *M. oryzae* isolate, TH12-GFP. Fluorescent signals were tracked at 6, 12, and 24 hpi. **D**, **E**. The *bui1* mutant showed significantly higher appressorium formation (**D**) and appressorium maturation rates (mature appressoria/total spores) (**E**) compared to ZJ22, suggesting that BUI1 suppresses this critical infection step to enhance blast resistance. **F**. The expression of *BUI1* was significantly induced in NIPB upon TH12 infection compared to mock treatment. Data were shown as mean ± SD, *n* > 25 (A, B), *n* = 10 (**E**) and *n* = 3 (**F**). Scale bars, 1 cm (**A**, **B**). Asterisks represented statistical significance (**P < 0.01, ***P < 0.001, two-tailed Student’s t-test). ns, not significant. Experiments were independently repeated three times with similar results

*BUI1* encodes a class II formin FH5 previously shown to regulate actin organization and rice development (Yang et al. 2011). We found that LatB treatment not only increased susceptibility to blast, but also downregulated the expression of *BUI1* compared to the mock control (Fig. S1A). To further investigate its role in disease resistance, we tested the *bui1* mutant, derived from the moderately resistant *Japonica* cultivar Zhejing 22 (ZJ22), by punch inoculation with *M. oryzae* isolate TH12. Compared to the wild-type ZJ22, the *bui1* mutant showed significantly increased lesion size (Fig. 2A), suggesting that BUI1 contributes to blast resistance. Consistent with this result, field trials in the blast nursery revealed that *bui1* plants were markedly more susceptible to natural infection (Fig. 2B).

To assess whether BUI1 influences fungal infection structures, we visualized appressorium development using a GFP-tagged *M. oryzae* strain. Notably, appressorium formation was more efficient and appressorium maturation rates were higher on *bui1* leaf sheath cells than on wild-type plants (Fig. 2C-E), suggesting that BUI1-mediated actin organization inhibits fungal infection by impairing appressorium development. Furthermore, expression analysis showed that *BUI1* was upregulated upon *M. oryzae* inoculation compared with mock treatment (Fig. 2E), further supporting its positive role in blast resistance.

### BUI1 is essential for PAMP-triggered actin reorganization

**Fig. 3:**
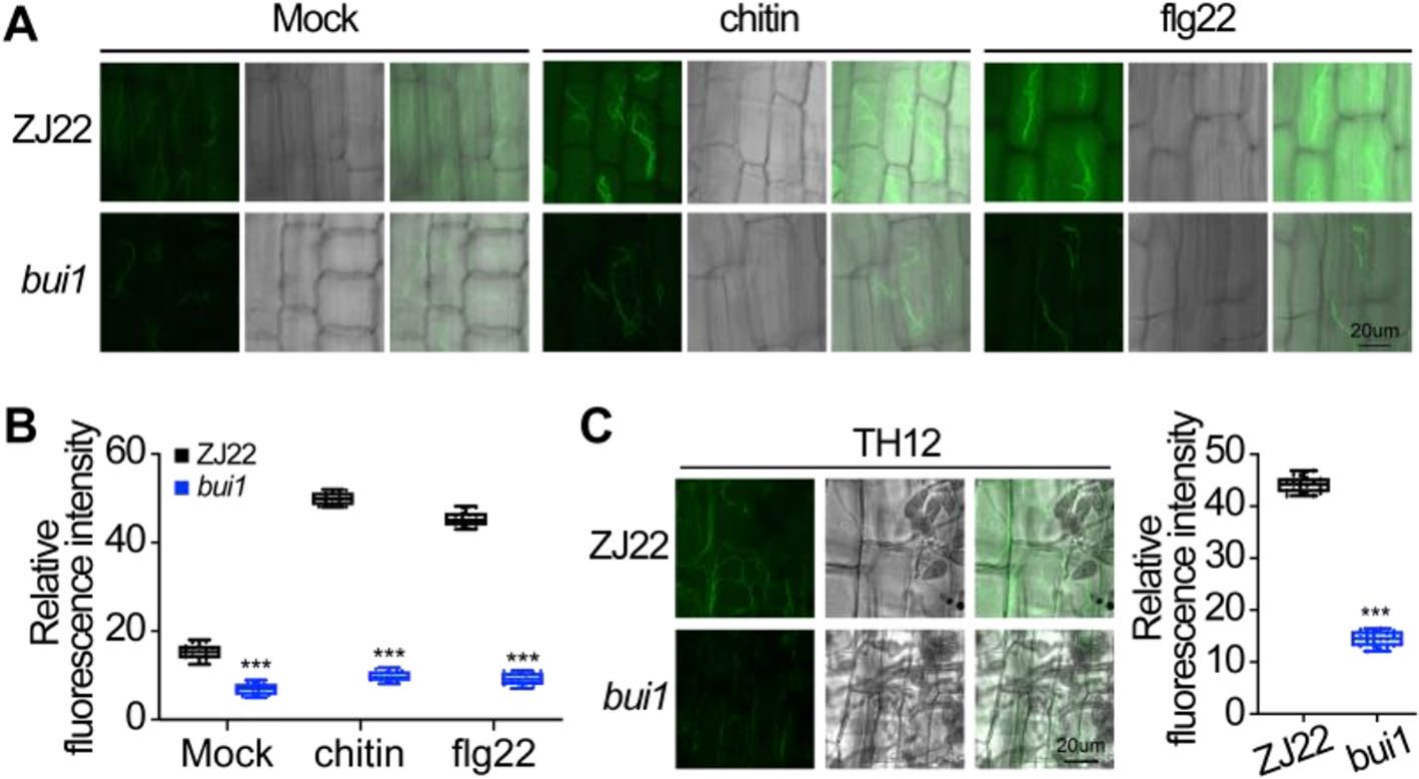
BUI1 promotes actin filament reorganization in response to PAMPs and *M. oryzae*. **A**. Actin filament organization was visualized by AlexaFluor488-phalloidin staining. Treatment with 1 µM chitin and flg22 for 5 min significantly increased actin filament density in ZJ22 leaf sheath cells but not in *bui1*, indicating that BUI1 is required for PAMP-induced actin reorganization. **B**. Quantitative analysis of actin filament levels in ZJ22 and *bui1* leaf sheath cells as detected in (**A**). **C**. *M. oryzae* isolate TH12 infection induced a greater abundance of actin filament abundance in ZJ22 compared to *bui1* at 12 hpi, further supporting BUI1’s role in actin dynamics during fungal defense. Data were shown as mean ± SD, *n* = 20 (**B**, **C**). Asterisks represented statistical significance (***P < 0.001, two-tailed Student’s t-test). Experiments were independently repeated twice with similar results

Plants perceive diverse PAMPs through pattern recognition receptors, activating immune signaling. To investigate whether BUI1 mediates cytoskeletal responses to microbial signals, we monitored actin filament organization in rice leaf sheath cells following stimulation with synthetic PAMP peptides. Phalloidin staining revealed well-organized actin filaments in wild-type ZJ22, whereas the *bui1* mutant displayed severe cytoskeletal disorganization (Fig. 3A). PAMP treatment with chitin or flg22 triggered a significant and transient increase in actin filament bundling in ZJ22, a response that was markedly attenuated in *bui1* mutant (Fig. 3A and B).

We further validated cytoskeletal responses in the rice-*M. oryzae* pathosystem. Upon inoculation with strain TH12, wild-type cells accumulated dense actin bundles around infection sites, whereas *bui1* mutant failed to mount this defense-associated remodeling (Fig. 3C). These results demonstrate that BUI1 is essential for PAMP-induced actin reorganization.

### *BUI1* mutation compromises resistance to multiple pathogens in rice

**Fig. 4:**
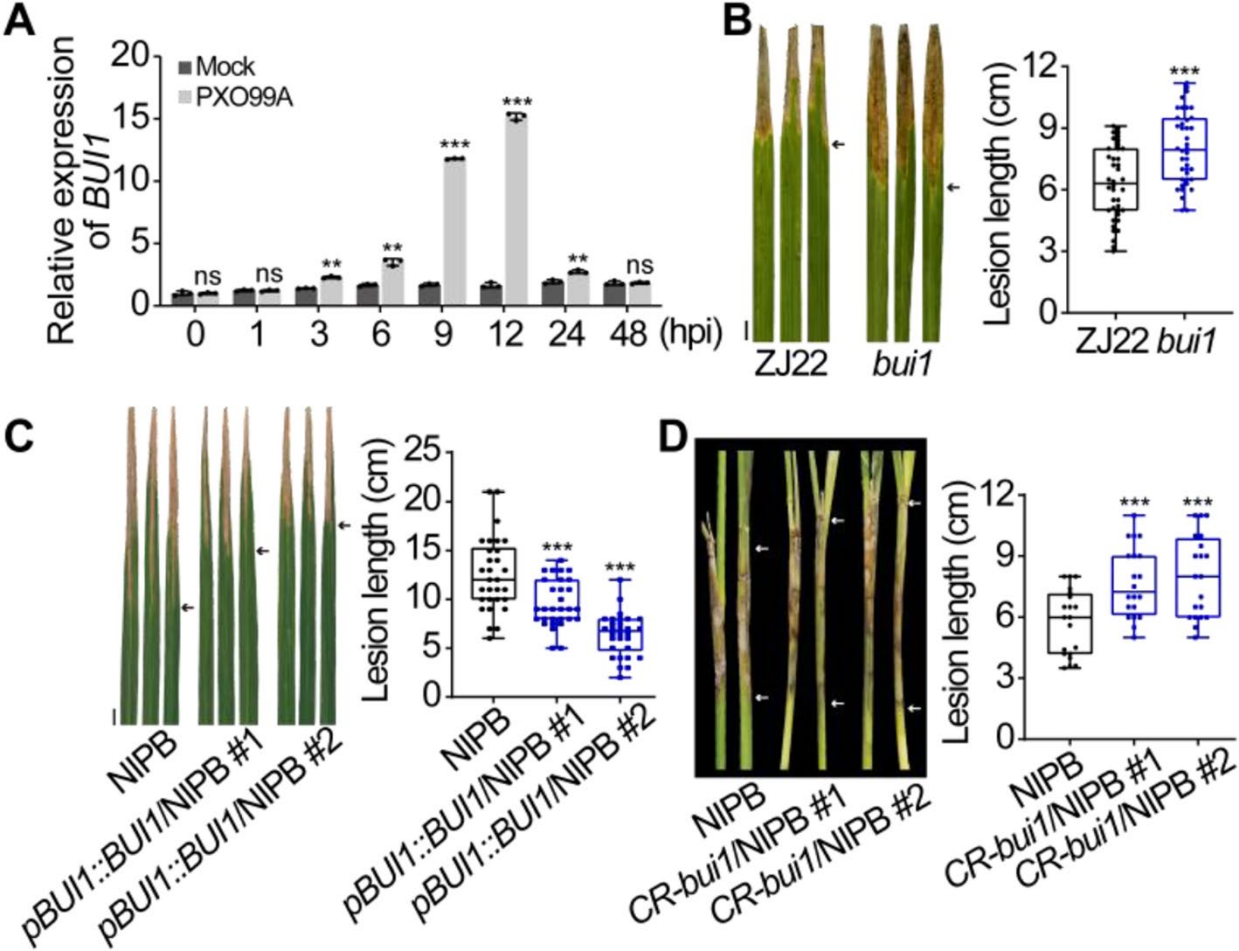
*BUI1* mutation impairs resistance to multiple pathogens. **A**. qRT-PCR analysis reveals significant upregulation of *BUI1* in NIPB upon *Xoo* PXO99A infection compared to mock-treated controls. **B**. Enhanced susceptibility of *bui1* mutant to bacterial blight compared to ZJ22. Plants were inoculated with *Xoo* strain PXO99A, and disease symptom was assessed at 14 days post-inoculation (dpi). **C**. Disease resistance to *Xoo* strain PXO99A was evaluated in WT, *BUI1*-OE in NIPB background. **D**. BUI1 regulates resistance to sheath blight. *CR-bui1*/NIPB was inoculated with *R. solani* at the tillering stage under field conditions. Lesion length was measured at 7 dpi. Data were shown as mean ± SD, *n* = 3 (**A**) and *n* ≥ 20 (**B**-**D**). Scale bars, 1 cm. Asterisks represented statistical significance (**P < 0.01, ***P < 0.001, two-tailed Student’s t-test). ns, not significant. Experiments were independently repeated three times with similar results

To determine whether BUI1 contributes to defense beyond rice blast, we assessed its role in resistance to other major pathogens. When inoculated with *Xoo* strain PXO99A, *BUI1* transcript levels were strongly induced compared with mock treatment (Fig. 4A), and the *bui1* mutant exhibited significantly longer lesions than wild-type ZJ22 (Fig. 4B), indicating a positive role in bacterial blight resistance. Moreover, *BUI1* overexpression lines (*pBUI1::BUI1*/NIPB) exhibited enhanced *Xoo* resistance compared to the wild-type NIPB, with the level of resistance increasing accordingly with its expression level (Fig. 4C and S1B). Because ZJ22 shows limited susceptibility to sheath blight, we generated *BUI1* knockout lines in the NIPB background (*CR-bui1*/NIPB) (Fig. S1C). Compared to wild type, *CR-bui1*/NIPB plants showed increased susceptibility to sheath blight (Fig. 4D). Together, these results demonstrate that BUI1 is critical for basal resistance to multiple fungal and bacterial pathogens in rice.

### Mutation in *bui1* partially abolishes enhanced disease resistance of *rod1* plants

**Fig. 5:**
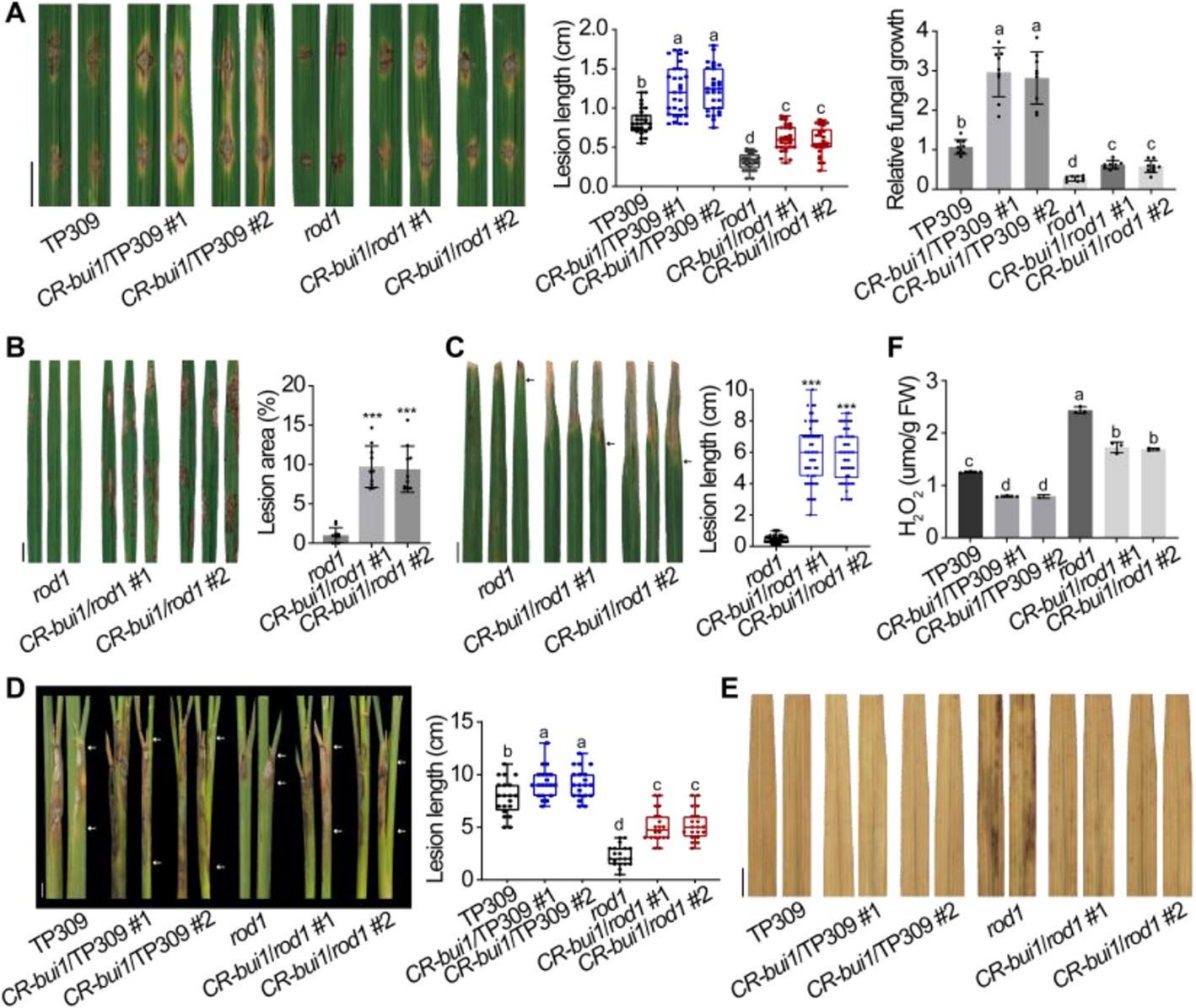
Mutation in *BUI1* abolished enhanced disease resistance of *rod1* plants. **A**. Blast resistance of TP309, *CR-bui1*/TP309, *rod1*, and *CR-bui1*/*rod1* lines after punch inoculation with the *M. oryzae* isolate TH12. Photos were taken at 7 dpi. *CR-bui1*/*rod1* exhibited reduced resistance compared to *rod1* but remained more resistant than TP309. **B**. Field evaluation of *CR-bui1*/*rod1* resistance in a blast nursery. Notably, the *BUI1* knockout lines compromised the complete resistance mediated by *rod1* to *M. oryzae*. **C**. Loss of resistance to *Xoo* strain PXO99A in *CR-bui1*/*rod1* plants, confirming that BUI1 is essential for ROD1-mediated immune control. **D**. BUI1 increased resistance to sheath blight. Lesion length was measured at 7 dpi in *CR-bui1*/TP309 and *CR-bui1*/*rod1*. Note that loss of *BUI1* function suppressed the disease resistance in *rod1*. **E**, **F**. As shown by DAB staining (**E**) and H_2_O_2_ content measurement (**F**), ROS levels were lower in *CR-bui1*/*rod1* compared to *rod1*, and lower in *CR-bui1*/TP309 compared to TP309, with TP309 serving as a negative control. These results support the role of BUI1 in regulating ROS homeostasis. Data were shown as mean ± SD, *n* = 3 (**F**), *n* = 5 (**A** right), *n* = 10 (**B**), and *n* > 20 (**A** middle, **C**, **D**). Scale bars, 1 cm. Asterisks represented statistical significance (***P < 0.001, two-tailed Student’s t-test). Letters indicate significant differences (*P* < 0.05) determined by one-way ANOVA with Tukey’s HSD test (**A**, **D**, **F**). Experiments were independently repeated three times with similar results

The Ca^2+^ sensor RESISTANCE OF RICE TO DISEASES1 (ROD1) negatively regulates rice immunity by activating catalase to enhance ROS scavenging, and loss of *ROD1* confers broad-spectrum disease resistance (Gao et al. 2021). To examine whether BUI1 contributes to this enhanced immunity, we generated *BUI1* knockout lines in both wild-type TP309 and *rod1* mutant backgrounds (CR-*bui1*/TP309 and CR-*bui1*/*rod1*) using CRISPR-Cas9 genome editing method (Fig. S1D). Compared with wild-type TP309, the *CR-bui1*/TP309 plants displayed increased susceptibility to *M. oryzae* in both spray and punch inoculation assays (Fig. 5A and S1E), further supporting the positive role of BUI1 in rice basal resistance. Notably, the strong blast resistance in the *rod1* mutant was significantly attenuated in the *CR-bui1*/*rod1* double mutant plants. This reduction in resistance was evident in spray inoculation, punch inoculation, and field tests, though the *CR-bui1*/*rod1* remained less susceptible than TP309 (Fig. 5A, B and S1E).

Similarly, loss of *BUI1* function compromised *rod1*-mediated resistance to bacterial blight and sheath blight (Fig. 5C and D). Moreover, *CR-bui1*/*rod1* accumulated lower ROS levels than *rod1*, as shown by DAB staining and H_2_O_2_ content measurement (Fig. 5E and F), supporting a functional link between BUI1 and ROS homeostasis. However, no direct interaction was detected by yeast two-hybrid (Y2H) assays between BUI1 and ROD1, or between BUI1 and the ROD1-interacting protein Catalase B (OsCatB) (Fig. S1F and S1G). Together, these findings demonstrate that BUI1 acts downstream of ROD1 to promote broad-spectrum disease resistance, likely through coordinated regulation of actin remodeling and ROS signaling.

### *BUI1* mutation attenuates immunity responses by enhancing ROS scavenging

**Fig. 6:**
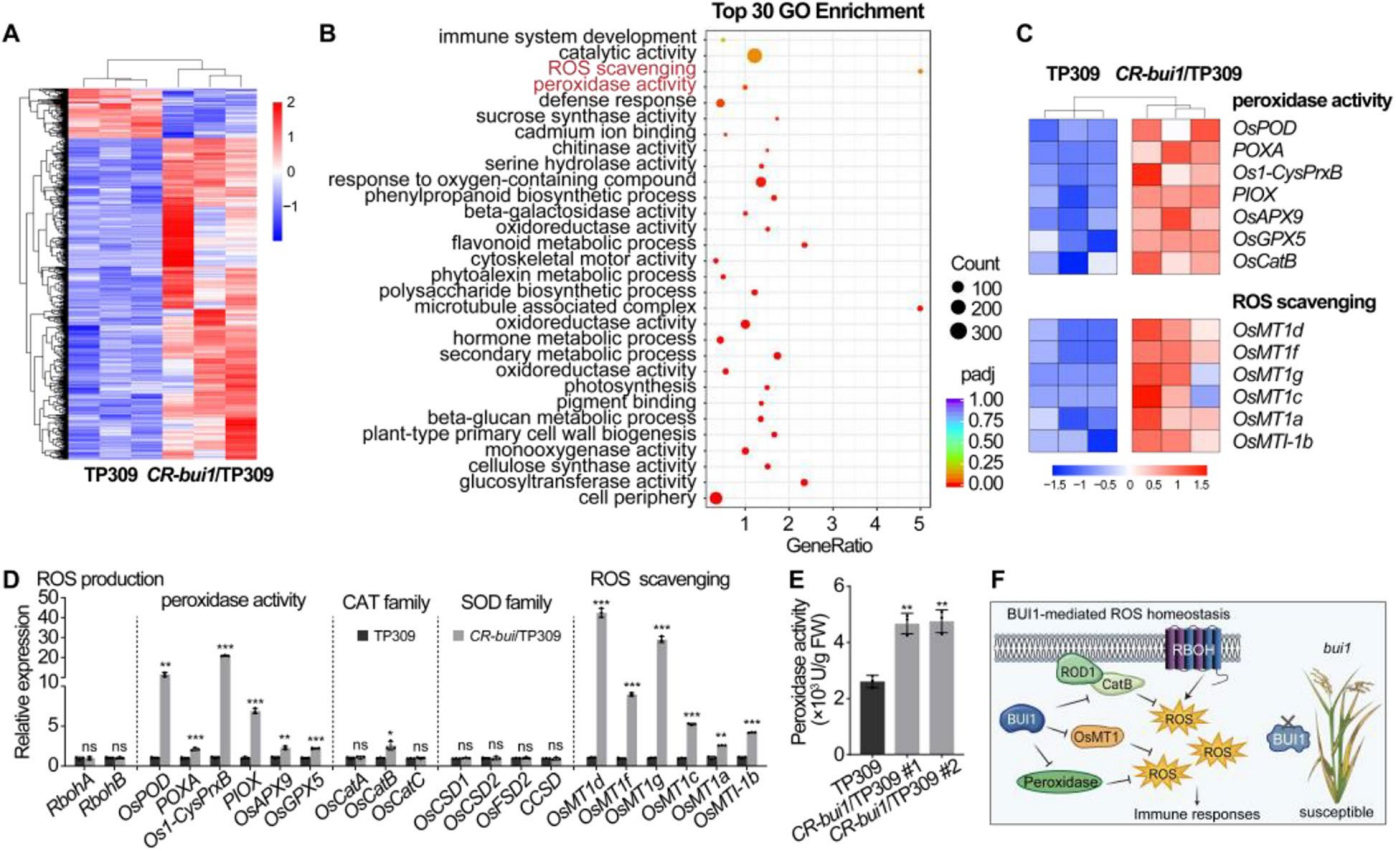
BUI1 regulates defense responses by suppressing ROS scavenging. **A**, **B**. Hierarchical clustering (**A**) and Gene ontology enrichment analysis (**B**) of differentially expressed genes (DEGs) in TP309 and *CR-bui1*/TP309 inoculated with *M. oryzae* isolate TH12, highlighting terms related to ROS scavenging. **C**, DEGs involved in ROS scavenging and peroxidase activity in *CR-bui1*/TP309 compared to TP309. **D**. qRT-PCR analysis showed that genes involved in peroxidase activity (*OsPOD*, *POXA*, *Os1-CysPrxB*, *PIOX*, *OsAPX9*, *OsGPX5*, *OsCatB*), and ROS scavenging (*OsMT1d*, *OsMT1f*, *OsMT1g*, *OsMT1c*, *OsMT1a*, *OsMTI-1b*) were upregulated in *CR-bui1*/TP309, whereas ROS production genes (*RbohA*, *RbohB*) and superoxide dismutase (SOD) family (*OsCSD1*, *OsCSD2*, *OsFSD2*, *CCSD*) were not. **E**. *CR-bui1*/TP309 exhibited higher peroxidase activity than TP309, indicating BUI1 negatively regulates peroxidase function. **F**. A working model summarizing the main regulatory mechanism of BUI1 identified in this study. Data were shown as mean ± SD, *n* = 3 (**D**, **E**). Asterisks represented statistical significance (*P < 0.05, **P < 0.01, ***P < 0.001, two-tailed Student’s t-test). ns, not significant. Experiments were independently repeated three times with similar results (**D**, **E**)

The rapid accumulation of ROS, or ROS burst, represents a hallmark of PAMP-triggered immunity, whereas excess ROS must be tightly regulated by scavenging systems. To maintain cellular immune homeostasis, plants adopted ROS-scavenging mechanisms, including peroxidase-mediated decomposition of hydrogen peroxide (H₂O₂), a key immune regulator. To investigate whether BUI1 influences ROS dynamics, we performed RNA-seq analysis on *CR-bui1*/TP309 and wild-type TP309 plants following *M. oryzae* infection. Genes associated with peroxidase activity and ROS detoxification were significantly upregulated in the mutant, including *OsPOD*, *POXA*, *OsCatB*, *OsMT1d*, and *OsMT1f* (Fig. 6A-C). qRT-PCR revealed no significant changes in the expression of ROS-producing genes (*RbohA*, *RbohB*) in the *CR-bui1*/TP309 mutant. In contrast, genes encoding ROS-scavenging enzymes, including peroxidases and *OsMT1s*, were greatly upregulated in the *bui1* mutant (Fig. 6D). These results establish that BUI1 maintains ROS homeostasis primarily through targeted inhibition of scavenging pathways, rather than through dual regulation of both production and scavenging mechanisms. This upregulation aligns with the increased susceptibility of *CR-bui1*/TP309, supporting the role of BUI1 in modulating ROS homeostasis and disease resistance.

Consistent with these transcriptional changes, *CR-bui1*/TP309 plants exhibits higher peroxidase activity than wild-type plants (Fig. 6E), which aligns with the reduced H₂O₂ accumulation observed in *CR-bui1*/TP309 (Fig. 5E and F). Together, these results indicate that BUI1 suppresses excessive ROS scavenging during immune responses, thereby facilitating effective defense activation. Loss of *BUI1*, in contrast, shifts the balance toward ROS detoxification, weakening pathogen resistance.

### BUI1 regulates transcriptional reprogramming during bacterial infection

To identify key defense-regulated genes regulated by BUI1, we performed a comprehensive time-course transcriptome analysis of ZJ22 and *bui1* plants following inoculation with *Xoo* strain PXO99A. Differential expression analysis revealed extensive transcriptional reprogramming in wild-type plants during the early stages of infection, with numerous genes showing strong induction as the infection progressed (Fig. S2A, Table S1). These genes were categorized into 20 distinct expression clusters, reflecting dynamic changes in immune responses. We focused on clusters 2, 7, 9, and 20, which contained genes strongly induced in ZJ22 upon *Xoo* infection but not in *bui1*. Kyoto Encyclopedia of Genes and Genomes (KEGG) pathway enrichment analysis of these clusters highlighted their involvement in phenylpropanoid biosynthesis, the MAPK signaling pathway, and plant-pathogen interaction pathways-key components of immune signaling networks (Fig. S2B and S2C). The failure of *bui1* to activate these pathways underscores the essential role of BUI1 in orchestrating early defense responses against bacterial infection.

## Discussion

The actin cytoskeleton is a fundamental component of plant immunity, and our findings reinforce its central role in defense activation. Pharmacological disruption of actin polymerization with latrunculin B (LatB) significantly increased the susceptibility of rice cultivars TP309 and NIPB to the fungal pathogen *M. oryzae* (Fig. 1A and B). Similarly, the *bui1* mutant, which is defective in actin organization, exhibited increased susceptibility to multiple pathogens, including the fungal blast pathogen *Magnaporthe oryzae*, sheath blight fungus *Rhizoctonia solani*, and bacterial blight pathogen *Xanthomonas oryzae* pv. *oryzae* (Figs. 2A, 4B and D). These results, together with previous reports, highlight the critical contribution of actin dynamics to basal resistance. Interestingly, previous studies have reported that the impact of cytoskeletal inhibitors on immunity varies depending on the timing and mode of application. For instance, pre-infection treatment with LatB enhanced resistance to the bacterial pathogen *Pseudomonas syringae* pv. *tomato* (*Pst* DC3000) in Arabidopsis and to the fungal pathogen *Leptosphaeria maculans* in *Brassica napus* (Leontovyčová et al. 2019), whereas pre-infection treatment with cytochalasin E induced hypersensitive response (HR)-like cell death in tobacco (Kobayashi et al., 2007). However, co-inoculation of cytochalasin D with *Pst* DC3000 in Arabidopsis had no significant effect (Shimono et al. 2016), while co-inoculation of LatB with bacterial pathogens increased susceptibility in Arabidopsis (Henty-Ridilla et al. 2013; Jelenska et al. 2014; Kang et al. 2014). These discrepancies may reflect differences in treatment strategy, species, or pathogen lifestyle. Unlike these dicot-based studies, our work provides direct evidence in a monocot system that actin polymerization is indispensable for blast resistance. Together, these findings underscore the importance of cytoskeletal integrity as a determinant of plant immunity.

The actin cytoskeleton dynamically responds to various biotic and abiotic stimuli, including microbial signals. Upon perception of pathogen-associated molecular patterns (PAMPs), rapid accumulation and reorganization of actin filaments occur. This process involves kinases such as FLS2, BAK1, and BIK1 (Henty-Ridilla et al. 2013), which have been reported to interact with actin (Bhandari and Brandizzi 2020). Moreover, the plant cytoskeleton provides microenvironments for pattern recognition receptor (PRR) complexes and regulates the endo-membrane trafficking of PRRs involved in plant immunity. Both the membrane trafficking system and the cytoskeleton are required for the delivery and proper localization of regulatory components to the plasma membrane, including the transport of PRRs and other defense-related factors (Lu et al. 2023). Consistent with this model, our study demonstrates that rice actin undergoes pronounced remodeling upon exposure to bacterial and fungal PAMPs, including flg22 and chitin (Fig. 3A). Furthermore, inoculation with the adapted fungal pathogen *M. oryzae* increased actin filament density in wild-type TP309 leaf sheath cells, whereas this response was absent in the *bui1* mutant (Fig. 3C), highlighting BUI1’s essential role in cytoskeletal dynamics during infection. Interestingly, such rapid actin remodeling was not observed in mature Arabidopsis leaf cells treated with elf26, a PAMP derived from the bacterial elongation factor EF-Tu (Henty-Ridilla et al. 2013). In contrast, cortical actin rearrangement occurred within minutes in hypocotyl cells following elf26 treatment (Henty-Ridilla et al. 2014; Li et al. 2015). These findings suggest that actin cytoskeletal responses may be PAMP-specific or depend on the spatial distribution of PAMP receptors across organs, tissues, or cell types. This complexity underscores the integrative role of the actin dynamics in plant immunity and highlights the importance of regulatory components such as BUI1 in coordinating these responses.

Reactive oxygen species (ROS) play multifaceted roles in plant biology, regulating processes such as the cell cycle, organelle movement, cytoskeleton dynamics, and immune signaling during pathogen infection (Brieger et al. 2012; Wu et al. 2023; Nakao et al. 2023). However, the mechanisms by which host-derived ROS influence cytoskeletal rearrangements during immune responses remain poorly understood. Previous studies have shown that cytoskeletal disruption suppresses key defense outputs, including callose deposition, ROS accumulation, hypersensitive response (HR), and pathogenesis-related (*PR*) gene expression (Badet et al. 2019; Sun et al. 2019b). Furthermore, growing evidence indicates a bidirectional interplay between ROS and cytoskeletal remodeling, where alterations in actin dynamics can trigger ROS release and subsequent programmed cell death (Gourlay et al. 2004; Gourlay and Ayscough 2005). Our findings establish a direct link between actin remodeling and ROS regulation in rice immunity. We show that actin polymerization contributes to enhanced disease resistance, whereas disruption of actin organization in *bui1* mutants leads to increased peroxidase activity and reduced H₂O₂ levels (Fig. 6), suggesting a key role for ROS-cytoskeleton crosstalk in immunity. Future studies should investigate the upstream signals and molecular complexes that coordinate BUI1-mediated actin dynamics with ROS homeostasis, which may reveal broader principles of cytoskeleton-ROS integration in cellular stress responses.

Phytohormones are integral regulators of cytoskeleton remodeling during plant immunity (Li and Staiger 2018; Sinha et al. 2024). Among them, salicylic acid (SA) signaling appears to be closely linked to actin dynamics. Previous studies have shown that disruption of EDS1, a key upstream regulator of SA signaling, compromises non-host resistance to wheat powdery mildew by impairing actin cytoskeletal function (Yun et al. 2003). Our findings further suggest that actin polymerization not only reinforces basal defense but also regulated SA, jasmonic acid/ethylene (JA/ET) pathway, thereby enhancing rice resistance against multiple pathogens (Fig. 1C). Although the mechanistic connection between actin dynamics and hormone signaling remains unclear, these results raise the intriguing possibility that actin remodeling functions upstream of SA/JA/ET accumulation to potentiate immune signaling. Future studies should dissect the signaling intermediates that couple actin polymerization with SA/JA/ET biosynthetic pathways, which may uncover novel regulatory nodes for engineering durable and broad-spectrum disease resistance in crops.

BUI1 contains three highly conserved domains: PTEN, proline-rich (FH1), and formin homology (FH2) (Fig. S3A). The PTEN domain mediates the BUI1 localization to the chloroplast surface, while the FH1 domain interacts with profilin, a small actin monomer-binding protein, or with the actin/profilin complex, thereby promoting actin polymerization. The FH2 domain, the most conserved region among formins, binds to the growing ends of actin filaments, modulating their elongation and regulating cytoskeletal dynamics. In addition to these canonical domains, we identified intrinsically disordered regions (IDRs; aa 1011–1171) that lack a stable tertiary structure. Computational analysis using PLAAC predicted these IDRs may mediate liquid–liquid phase separation (Fig. S3B). However, expressing full-length BUI1 protein, whether in rice in vivo or in heterologous systems such as *E. coli*, yeast, or viral-based platforms in vitro, has proven challenging, likely due to the presence of these IDRs. To overcome this, we generated a truncated BUI1 variant lacking IDRs, which failed to undergo phase separation, confirming that the IDRs are essential for this function. Future studies should further investigate BUI1 as a phase separation-competent immune regulator, particularly exploring how its IDR-mediated biomolecular condensates function as organizational hubs that integrate cytoskeletal dynamics with defense signaling pathways in plant immunity.

## Conclusion

In summary, our study demonstrates that the *bui1* mutation disrupts actin cytoskeletal remodeling, thereby compromising both basal disease resistance and the broad-spectrum resistance conferred by *rod1*. BUI1 acts as a regulatory hub in PAMP-triggered immune signaling and modulates host ROS homeostasis (Fig. 6F).

## Materials and methods

### Plants and growth conditions

Rice (*Oryza sativa*) materials used in this study included the wild-type (WT) cultivars ZJ22, Nipponbare (NIPB), and TP309, as well as the previously described mutants *bui1* and *rod1*(Yang et al. 2011; Gao et al. 2021). Transgenic lines were generated in the NIPB and TP309 backgrounds. Newly developed mutant lines included *CR-bui1*/NIPB, *CR-bui1*/TP309, *CR-bui1*/*rod1*, and *pBUI1::BUI1*/NIPB.

Rice seeds were first treated to break dormancy by heating at 42 °C for 48 h, followed by surface sterilization in 1% sodium hypochlorite for 15 min. Plants were grown in a controlled growth chamber at 26℃ with14-h light/10-h dark photoperiod. Field trials were conducted in Shanghai and Hainan during the natural growing seasons. For pathogen inoculation, two-week-old seedlings and tillering-stage plants were used for rice blast assays, while two-month-old plants were used for bacterial blight and sheath blight infection assays.

### Development of transgenic rice plants

To generate *BUI1* overexpression lines, we first constructed the vector PUN1301-BUI1-GFP, in which BUI1 was fused with GFP under the control of the maize *UBIQUITIN* (*UBI*) promoter. This construct was transformed into wild-type NIPB. However, neither GFP fluorescence nor specific GFP antibody signals were detected. We then attempted *BUI1* overexpression using its native promoter. For this purpose, the *BUI1* genomic DNA was inserted into the pCambia1300 vector to generate *pBUI1::BUI1*/NIPB.

To generate *BUI1* knockout mutants, CRISPR-Cas9 vectors were constructed following established protocols (Ma et al. 2015). The designed target sequences were cloned into the CRISPR-Cas9 vector, which was then introduced into *Agrobacterium tumefaciens* strain EHA105 for rice transformation. More than 15 independent transgenic lines were obtained in different genetic backgrounds. Mutations in the target gene were confirmed by PCR amplification and sequencing. All primers used for cloning are listed in Table S2.

### Pathogen inoculation

Rice blast assays were performed as previously described (Deng et al. 2017). *M. oryzae* isolate TH12 was cultured on complete agar medium at 25 °C for 10 days to induce sporulation. Spores were harvested in sterile water and adjusted to a concentration of 1 × 10^5^ spores/mL. Two-week-old seedlings were spray-inoculated, while leaves at the tillering and booting stages were punch-inoculated. For leaf sheath inoculation, sheaths were excised at the base and placed in Petri dishes lined with two layers of moistened filter paper, and conidial suspension was pipetted onto each sheath. Dishes were sealed and maintained under high humidity. All inoculated plants were kept in a dew chamber at 26℃ in darkness for 24 h and then transferred to growth conditions of 26℃ with 12 h/12 h (day/night) and 90% relative humidity. Disease symptom was scored at 7 days post-inoculation (dpi) by measuring lesion lengths/areas using ImageJ. Relative fungal growth was quantified using qRT-PCR to determine the abundance of *Pot2* transposon DNA, normalized to the rice *ubiquitin* gene. PCR primers are listed in Table S2.

To evaluate bacterial blight resistance, two-month-old rice plants were inoculated with *Xoo* strain PXO99A using the leaf-clip method (Lin et al. 2022). Briefly, PXO99A was cultured on peptone sucrose agar (PSA) medium at 28 °C for three days. Bacterial cells were collected and resuspended in sterile water to optical density OD_600_ of 1.0. Lesion length was measured at 14 dpi.

Sheath blight resistance was assessed at the tillering stage as described (Gao et al. 2021). *R. solani* isolate RH-9 was cultured on potato-dextrose-agar (PDA) plates at 28℃ for 2 days. Sterilized wooden toothpicks (0.8–1.0 cm in length) were co-incubated with fungal plugs for 3 days to allow colonization, and then inserted into the third leaf sheath. Disease symptoms were evaluated at 7 dpi.

### Rice blast nursery test

Field resistance to rice blast was assessed under natural infection conditions at the Lingshui blast nursery on Hainan Island, a well-established site for evaluating blast resistance under high disease pressure. Each experimental replicate consisted of more than 100 plants per genotype.

### Chemical treatments

The actin-depolymerizing drug latrunculin B (LatB; Abcam, ab144291) was used to disrupt actin cytoskeleton dynamics. A 2 mM stock solution was prepared in DMSO. For treatment, two-week-old soil-grown rice seedlings were sprayed with either 1 µM LatB or 0.05% DMSO (as control) 12 h prior to *M. oryzae* inoculation.

### RNA extraction and gene expression analysis

Total RNA was extracted from various rice tissues or infected leaves using TRIzol reagent (Invitrogen, 15596018CN) according to the manufacturer’s protocol. For gene expression analysis, 2 µg of RNA was reverse-transcribed into cDNA using the ReverTra Ace qPCR RT Master Mix with gDNA Remover kit (TOYOBO, FSQ-301C). Quantitative real-time PCR (qRT-PCR) was performed using SYBR Premix Ex Taq (TaKaRa, RR420A) on a CFX96 Real-Time PCR system, with gene-specific primers in Table S2. Rice *ACTIN1* served as the internal control. All experiments were independently repeated three times.

### Observation of actin filaments in rice leaf sheath cells

Confocal microscopy was used to visualize actin filaments in wild-type and *bui1* rice cells, following established methods (Yang et al. 2011). Approximately 1 cm segments were excised from the adaxial sheath epidermis and treated for 5 min with mock solution, 1 µM chitin, or 1 µM flg22. Samples were then incubated in PME buffer supplemented with 300 µM MBS (m-maleimidobenzoyl-N-hydroxysuccinimide ester, Invitrogen, 22,311), 1.5% glycerol, and 0.1% Triton X-100 for 30 min under gentle agitation to facilitate cross-linking. Subsequently, the tissues were fixed in PME buffer containing 2% paraformaldehyde for 30 min. For actin visualization, samples were stained with 0.66 µM Alexa Fluor™ 488-phalloidin (Invitrogen, A12379) in actin-staining buffer. Actin filament levels were imaged using a Zeiss LSM 880 confocal microscope, and leaf sheaths of wild-type and *bui1* were used to quantify the actin filament level with ImageJ software.

### DAB staining

To visualize in vivo H_2_O_2_ accumulation, leaf tissues were sectioned and infiltrated with 1 mg/mL 3,3'-diaminobenzidine (DAB, Sigma, D12384) at 25℃ for 8 h. Subsequently, samples were decolorized by boiling in 96% ethanol for 10 min to remove chlorophyll, followed by equilibration in 50% glycerol for enhanced optical clarity during imaging.

### Measurement of H_2_O_2_

H_2_O_2_ was quantified using a Hydrogen Peroxide (H_2_O_2_) Content Assay Kit (Sangon Biotech, D799774) according to the manufacturer’s protocol. Briefly, approximately 100 mg of leaf tissue rapidly frozen in liquid nitrogen and homogenized to a fine powder. The powder was then suspended in 1 mL of ice-cold H_2_O_2_ extraction buffer (20 mM sodium phosphate buffer, pH 6.5) and incubated at 4 °C for 30 min. After extraction, the samples were centrifuged at 12,000 rpm at 4℃ for 10 min. The supernatant was collected for analysis, and H_2_O_2_ concentration was determined and expressed as micromoles per gram of fresh weight (μmol/g FW).

### Peroxidase activity assay

Fresh rice leaves were immediately frozen in liquid nitrogen and homogenized to a fine powder. A 100 mg of the powdered tissue was suspended in 1 mL of extraction buffer, then centrifuged at 12, 000 rpm for 5 min at 4℃. The supernatant was collected, and peroxidase activity was measured using the Peroxidase Assay Kit (Bioss, AK098) in strict accordance with the manufacturer’s protocol.

### RNA sequencing

Total RNA was extracted from leaves of two-week-old seedlings (TP309 vs *CR-bui1*/TP309) and two-month-old plants (Zj22 vs *bui1*) using TRIzol reagent. Samples were collected after a 36-h incubation with TH12 or a 0–48-h time course following inoculation with *Xoo* strain PXO99A. For RNA-seq analysis, three independent biological replicates were prepared. Library construction and sequencing was performed by Shanghai Ruixing Bio-Tech (Shanghai, China).

### Statistical analysis

Data analysis was performed using GraphPad Prism, Excel 2016 and SPSS Statistics (https://www.ibm.com/cn-zh/spss). Results are presented as mean ± SD, with the sample or replicate number (n) specified in the respective figure legends. Statistical significance was determined as follows: two-tailed Student’s t-test for comparisons between two groups, and one-way ANOVA followed by Tukey’s honest significant differences (HSD) test for multiple-group comparisons. Detailed quantification methods and statistical results are provided in the figure legends.

## Supplementary Information

Supplementary Material 1. Fig. S1. Development of transgenic rice plants. A. qRT-PCR analysis of *BUI1* expression in leaves after 12 h treatment with 1 uM LatB or Mock. Note that, *BUI1*, an actin organization-related gene, was down-regulated following LatB treatment. B. Relative transcript accumulation of* BUI1 *determined by qRT-PCR in NIPB and OE lines. The rice ACTIN1 served as an internal control. C, D. Schematic representation of independent *BUI1* knockout (KO) mutants in NIPB, TP309, and *rod1* genetic backgrounds. E. Blast resistance of TP309, *CR-bui1*/TP309, *rod1*, and *CR-bui1*/*rod1* lines after spray inoculation with the *M. oryzae* isolate TH12. Photos were taken at 7 dpi. *CR-bui1*/*rod1*exhibited reduced resistance compared to *rod1* but remained more resistant than TP309. F. G. No direct interaction was detected between BUI1 (full-length, PTEN, FH1 or FH2 domain) and ROD1 or OsCatB by yeast two-hybrid (Y2H) assays, whereas the interaction between ROD1 and OsCatB was used as a positive control. Data were shown as mean ± SD, *n*= 3 (A, B) and *n*= 5 (E). Scale bars, 1 cm. Asterisks represented statistical significance (**P < 0.01, ***P < 0.001, two-tailed Student’s t-test). Letters indicate significant differences (*P* < 0.05) determined by one-way ANOVA with Tukey’s HSD test. Experiments were independently repeated three times with similar results (A, B, E-G). Fig. S2. BUI1 regulates transcriptional reprogramming during responses against *Xoo*. A. Hierarchical clustering of differentially expressed genes in ZJ22 and *bui1 *uponinoculation with *Xoo* strain PXO99A at 0, 6, 12, 24, and 48 hpi. Clustering was performed using a Gaussian mixture model with an empirical Bayes approach (variational Bayesian inference) to automatically determine the optimal number of clusters. B. Kyoto encyclopedia of genes and genomes (KEGG) pathway enrichment analysis of upregulated genes in ZJ22 (clusters 2, 7, 9, and 20) that were not induced in *bui1 *upon *Xoo* infection. C. Key defense-related KEGG pathways, including phenylpropanoid biosynthesis, MAPK signaling, and plant-pathogen interaction, were significantly enriched in ZJ22 but not in* bui1*, suggesting BUI1's role in activating these defense mechanisms. Fig. S3. Visualization outputs of BUI1 protein from PLAAC. A. Schematic diagram of BUI1, containing PTEN (198-336 aa), FH1 (825-1170 aa) and FH2 (1188-1588 aa) domain. B. Detailed visualization of the BUI1 protein using PLAAC (Prion-Like Amino Acid Composition) software, a web and command-line application designed to scan proteins sequences for prion-like amino acid composition. The output displays several prion-prediction scores, including those related to intrinsically disordered regions (IDRs) enrich in proline amino acid.

Supplementary Material 2. Table S1. *Xoo*-induced gene were categorized into 20 distinct clusters.

Supplementary Material 3. Table S2. Constructs and primers.

## Data Availability

All study data are included in the article and/or supporting information. Genome sequence data of *BUI1* can be found in the GenBank/EMBL libraries under the following accession numbers: LOC_Os07g40510. The RNA-seq data generated in this study have been deposited in the SRA database under accession PRJNA1298961 and GSE305236.

## Abbreviations

PTI: Pattern-triggered immunity
*M. oryzae*: *Magnaporthe oryzae*
*Xoo*: *Xanthomonas oryzae* Pv. *oryzae*
*R. solani*: *Rhizoctonia solani*
PAMPs: Pathogen-associated molecular patterns
ROS: Reactive oxygen species
H₂O₂: Hydrogen peroxide
HR: Hypersensitive response
IDRs: Intrinsically disordered regions
