# Supplementary material for: BUI1 coordinates actin cytoskeleton remodeling and ROS homeostasis to confer broad-spectrum disease resistance in rice
Source: Stress Biol. 2026 Jul 2;6(1):48. doi: 10.1007/s44154-026-00321-5 (PMC13328597; doi:10.1007/s44154-026-00321-5)
Supplement: Supplementary file 1 — Supplementary Material 1. Fig. S1. Development of transgenic rice plants. A. qRT-PCR analysis of BUI1 expression in leaves after 12 h treatment with 1 uM LatB or Mock. Note that, BUI1, an actin organization-related gene, was down-regulated following LatB treatment. B. Relative transcript accumulation of BUI1 determined by qRT-PCR in NIPB and OE lines. The rice ACTIN1 served as an internal control. C, D. Schematic representation of independent BUI1 knockout (KO) mutants in NIPB, TP309, and rod1 genetic backgrounds. E. Blast resistance of TP309, CR-bui1/TP309, rod1, and CR-bui1/rod1 lines after spray inoculation with the M. oryzae isolate TH12. Photos were taken at 7 dpi. CR-bui1/rod1exhibited reduced resistance compared to rod1 but remained more resistant than TP309. F. G. No direct interaction was detected between BUI1 (full-length, PTEN, FH1 or FH2 domain) and ROD1 or OsCatB by yeast two-hybrid (Y2H) assays, whereas the interaction between ROD1 and OsCatB was used as a positive control. Data were shown as mean ± SD, n= 3 (A, B) and n= 5 (E). Scale bars, 1 cm. Asterisks represented statistical significance (**P < 0.01, ***P < 0.001, two-tailed Student’s t-test). Letters indicate significant differences (P < 0.05) determined by one-way ANOVA with Tukey’s HSD test. Experiments were independently repeated three times with similar results (A, B, E-G). Fig. S2. BUI1 regulates transcriptional reprogramming during responses against Xoo. A. Hierarchical clustering of differentially expressed genes in ZJ22 and bui1 uponinoculation with Xoo strain PXO99A at 0, 6, 12, 24, and 48 hpi. Clustering was performed using a Gaussian mixture model with an empirical Bayes approach (variational Bayesian inference) to automatically determine the optimal number of clusters. B. Kyoto encyclopedia of genes and genomes (KEGG) pathway enrichment analysis of upregulated genes in ZJ22 (clusters 2, 7, 9, and 20) that were not induced in bui1 upon Xoo infection. C. Key defense-related KEGG pathway [file 44154_2026_321_MOESM1_ESM.docx]

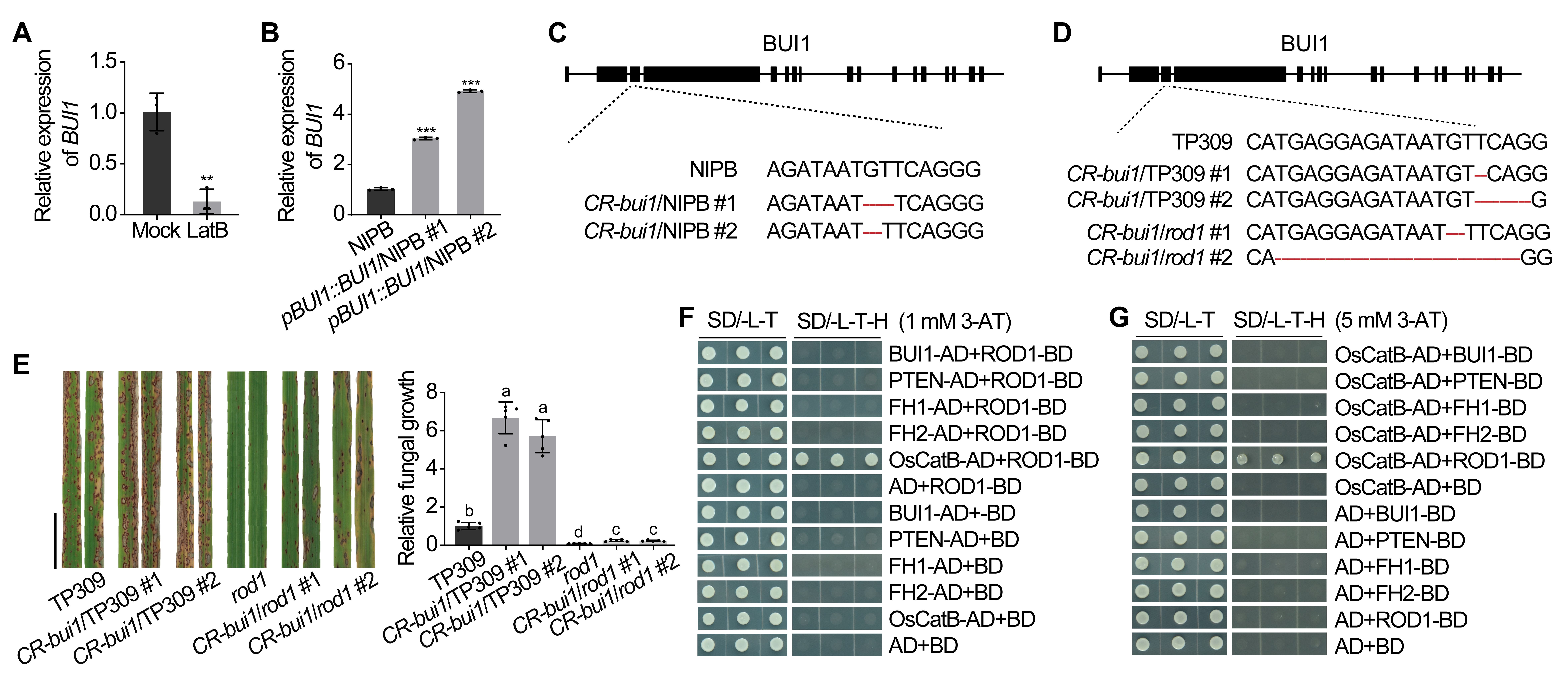


## Fig. S1. Development of transgenic rice plants. A. qRT-PCR analysis of *BUI1* expression in leaves after 12 h treatment with 1 uM LatB or Mock. Note that, *BUI1*, an actin organization-related gene, was down-regulated following LatB treatment. B. Relative transcript accumulation of *BUI1* determined by qRT-PCR in NIPB and OE lines. The rice ACTIN1 served as an internal control. C, D. Schematic representation of independent *BUI1* knockout (KO) mutants in NIPB, TP309, and *rod1* genetic backgrounds. E. Blast resistance of TP309, *CR-bui1*/TP309, *rod1*, and *CR-bui1*/*rod1* lines after spray inoculation with the *M. oryzae* isolate TH12. Photos were taken at 7 dpi. *CR-bui1*/*rod1* exhibited reduced resistance compared to *rod1* but remained more resistant than TP309. F. G. No direct interaction was detected between BUI1 (full-length, PTEN, FH1 or FH2 domain) and ROD1 or OsCatB by yeast two-hybrid (Y2H) assays, whereas the interaction between ROD1 and OsCatB was used as a positive control. Data were shown as mean ± SD, n = 3 (A, B) and n = 5 (E). Scale bars, 1 cm. Asterisks represented statistical significance (***P* < 0.01, ****P* < 0.001, two-tailed Student’s t-test). Letters indicate significant differences (*P* < 0.05) determined by one-way ANOVA with Tukey’s HSD test. Experiments were independently repeated three times with similar results (A, B, E-G).


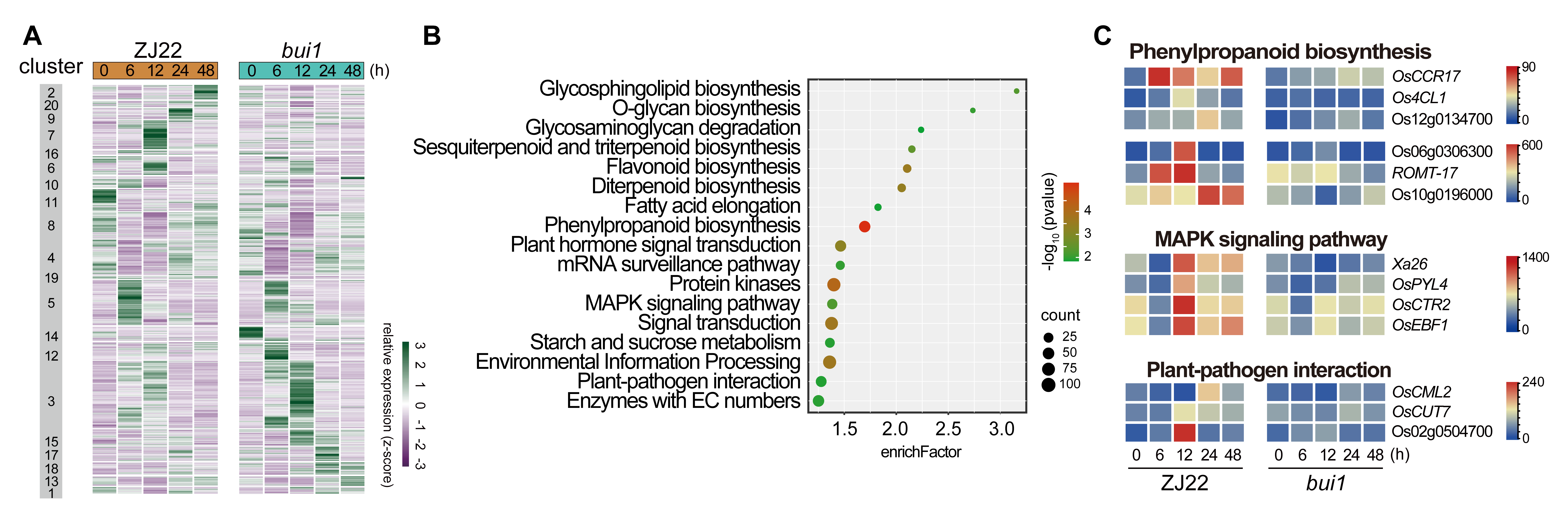


## Fig. S2. BUI1 regulates transcriptional reprogramming during responses against *Xoo*. A. Hierarchical clustering of differentially expressed genes in ZJ22 and *bui1* upon inoculation with *Xoo* strain PXO99A at 0, 6, 12, 24, and 48 hpi. Clustering was performed using a Gaussian mixture model with an empirical Bayes approach (variational Bayesian inference) to automatically determine the optimal number of clusters. B. Kyoto encyclopedia of genes and genomes (KEGG) pathway enrichment analysis of upregulated genes in ZJ22 (clusters 2, 7, 9, and 20) that were not induced in *bui1* upon *Xoo* infection. C. Key defense-related KEGG pathways, including phenylpropanoid biosynthesis, MAPK signaling, and plant-pathogen interaction, were significantly enriched in ZJ22 but not in *bui1*, suggesting BUI1's role in activating these defense mechanisms.


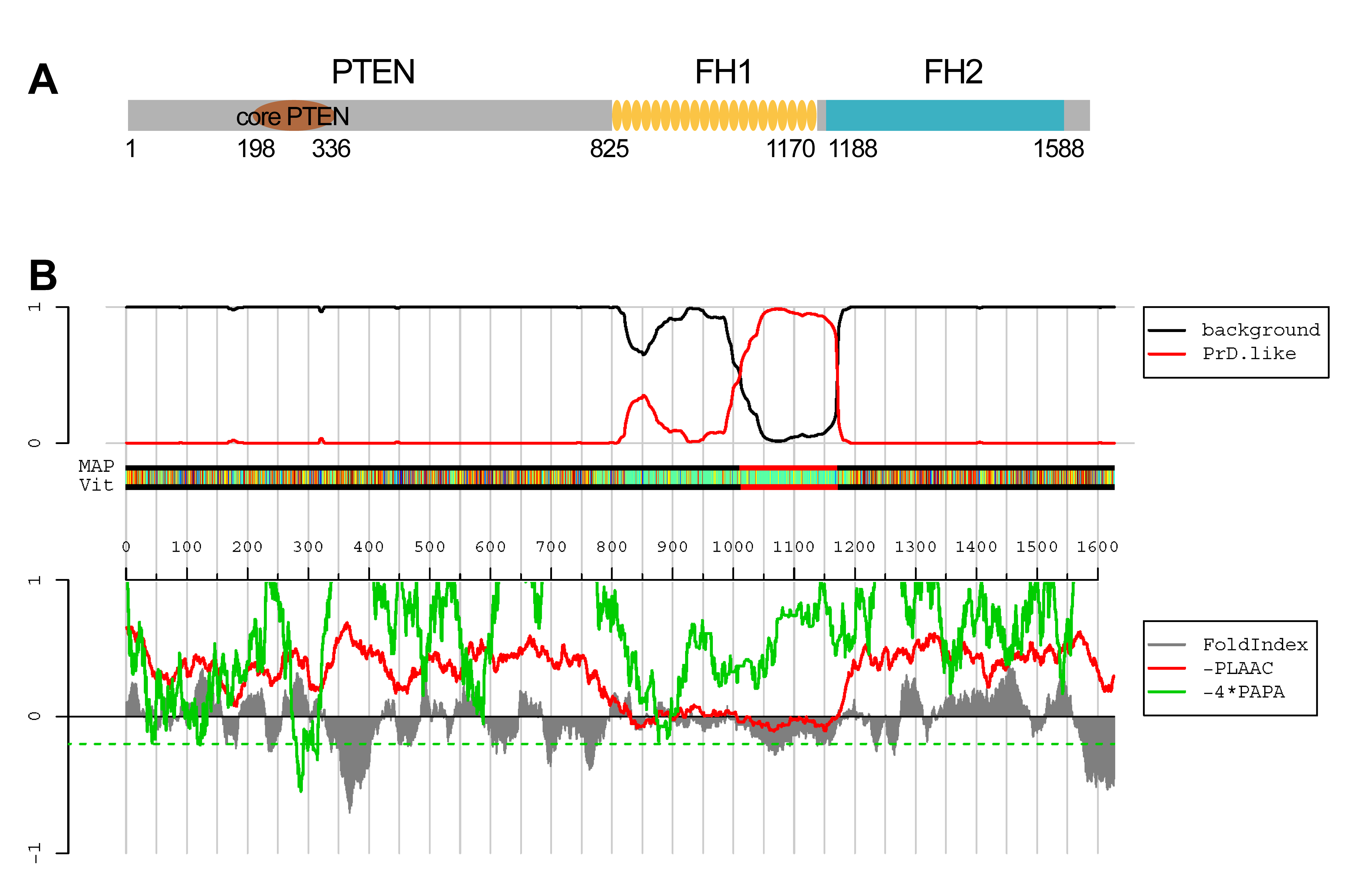


## Fig. S3. Visualization outputs of BUI1 protein from PLAAC. A. Schematic diagram of BUI1, containing PTEN (198-336 aa), FH1 (825-1170 aa) and FH2 (1188-1588 aa) domain. B. Detailed visualization of the BUI1 protein using PLAAC (Prion-Like Amino Acid Composition) software, a web and command-line application designed to scan proteins sequences for prion-like amino acid composition. The output displays several prion-prediction scores, including those related to intrinsically disordered regions (IDRs) enrich in proline amino acid.
