# Supplementary material for: BUI1 coordinates actin cytoskeleton remodeling and ROS homeostasis to confer broad-spectrum disease resistance in rice
Source: Stress Biol. 2026 Jul 2;6(1):48. doi: 10.1007/s44154-026-00321-5 (PMC13328597; doi:10.1007/s44154-026-00321-5)
Supplement: Supplementary file 3 — Supplementary Material 3. Table S2. Constructs and primers. [file 44154_2026_321_MOESM3_ESM.pdf]

Constructs and primers

Primers used for vector construction	
BUI1-U3F	ggcaCCATGAGGAGATAATGTTCA
BUI1-U3R	aaacTGAACATTATCTCCTCATGG
BUI1-crispr-JD-F	AAAAGCCTCCTATTAGCCGACC
BUI1-crispr-JD-R	CTCCATCATCGTCTACCTCCT
Cas9-F	GAAGAGGTCGTGAAGAAGATGAAGAA
Cas9-R	AGGTAGTGCTTGTGCTGTTCCA
pBUI1::BUI1/NIPB-F	tacgaattcgagctcggtaccGGATAGTACGATGGTGGGCG
pBUI1::BUI1/NIPB-R	caggtcgactctagaggatccGATCATGGAAAAGCTGCTGCA
BUI1-AD-F	CCATGGAGGCCAGTGAATTCatggcgctcttccgcaaatt
BUI1-AD-R	GCAGCTCGAGCTCGATGGATCCtcaacctacatcttttcctc
PTEN-AD-F	CCATGGAGGCCAGTGAATTCATGagccgacccctcatcttg
PTEN-AD-R	GCAGCTCGAGCTCGATGGATCCTCAagggtctgaaaagact
FH1-AD-F	GCCATGGAGGCCAGTGAATTCATGcctccacctccacctccaccacctccaccctatgctt
FH1-AD-R	CAGCTCGAGCTCGATGGATCCTCAaggattgcctcctggaggtggtggtgctcctggtcc
FH2-AD-F	GCCATGGAGGCCAGTGAATTCATGtttggggctgcagctgca
FH2-AD-R	GCAGCTCGAGCTCGATGGATCCTCActtttctgcttctgcctct
BUI1-BD-F	ATGGCCATGGAGGCCGAATTCatggcgctcttccgcaaatt
BUI1-BD-R	CCGCTGCAGGTCGACGGATCCtcaacctacatcttttcctc
PTEN-BD-F	ATGGCCATGGAGGCCGAATTCATGagccgacccctcatcttg
PTEN-BD-R	CCGCTGCAGGTCGACGGATCCTCAagggtctgaaaagact
FH1-BD-F	ATGGCCATGGAGGCCGAATTCATGcctccacctccacctccaccacctccaccctatgct
FH1-BD-R	CCGCTGCAGGTCGACGGATCCTCAaggattgcctcctggaggtggtggtgctcctggtcc
FH2-BD-F	ATGGCCATGGAGGCCGAATTCATGtttggggctgcagctgc
FH2-BD-R	GCCGCTGCAGGTCGACGGATCCTCActtttctgcttctgc

Primers used for real time qPCR	
OsACTIN-q-F	TGTATGCCAGTGGTCGTACCA
OsACTIN-q-R	CCAGCAAGGTCGAGACGAA
BUI1-q-F	CCAGCAGCTGTTCCAAAACCG
BUI1-q-R	AACACCCGCAGTTTTGACTCC
OsPAL1-q-F	AGGAGCTCGGCTGCGTATT
OsPAL1-qT-R	ATGCCGAGGAACACCTTGTT
OsICS1-q-F	CAACGAGAACTCCCTCACCT
OsICS1-q-R	TCCCACCTTTGAGCTCTTGT
PR4-q-F	AGTATGGATGGACCGCCTTCTGT
PR4-q-R	CTCGCAATTATTGTCGCACCTGTTC
WRKY45-q-F	CGGGTAAAACGATCGAAAGA
WRKY45-q-R	TTTCGAAAGCGGAAGAACAG
OsNPR1-q-F	TCATGGCGCAGGTCCTCTT
OsNPR1-q-R	TAATCCGAGGCTTAGGCGTG
OsLOX2-q-F	GTGGGAGGTGGAGAAGATGG
OsLOX2-q-R	CAGGAGTTGGCGACGAAGA
OsAOS2-q-F	CTCCAAGAAAGAACGCCGAACA
OsAOS2-q-R	CCGAAACTTTGGACGCCTAACTAC
OsERF1-q-F	CAGTGAAGCAAGCAAACCAA

OsERF1-q-R	GCTTATCGCGTTTGCAATTT
OsPOD-q-F	CTAGTCCGCCTCCTCTTCCA
OsPOD -q-R	CTTCTCCGGGTGTGGGTTC
POXA-q-F	ATGCTGCCATGAGGACTGAG
POXA -q-R	AAGGATGGAACCGTCACACC
Os1-cysPrxB-q-F	GTCGCACAAGGACTGGATCA
Os1-cysPrxB-q-R	CCTGATCGCCTCCCTGTCC
PIOX-q-F	GACCTTGCTGCCCTAGAAG
PIOX-q-R	ACTTCTCTCCCTGTCTCGGT
OsAPX9-q-F	GGGCTCTCCTGGAAGATCCT
OsAPX9-q-R	CTACGTGGAGCGAACCCAAG
OsGPX5-q-F	GTCCACGACATCTCCGTCAA
OsGPX5-q-R	ACCTTCCCCTCATACTCGCT
OsCATB-q-F	CGTGGAAGCCCTGAGACATT
OsCATB-q-R	GACAGGCATATTGTTCCCAACA
OsMT1d-q-F	GGATGCGGCGGTGGCTGCGG
OsMT1d-q-R	AGTGGTTGCAGTAGTGGTGG
OsMT1f-q-F	CGGTGGAAGTTGCAACTGTG
OsMT1f-q-R	CCCCTTCTCAGGTGCAACAC
OsMT1g-q-F	GGAAGTTGCAACTGCGGTTT
OsMT1g-q-R	GGTGGTGTTCTTCTCTGCCA
OsMT1c-q-F	AGATGTACCCTGACCTGGCT
OsMT1c-q-R	TTGCAGCTGGAGCCACAG
OsMT1a-q-F	TCAAACTGCTCCTGCGG
OsMT1a-q-R	CTTGGTGCTGCTGCTCTTCT
OsMTI-1b-q-F	GGCTCGAACTGCACGTGCGG
OsMTI-1b-q-R	GCTGCTGCTCTTCTCTTCCA

**Primers used for blast infection analysis**	
OsUbq-F	GACGGACGCACCCTGGCTGACTAC
OsUbq-R	TGCCAATTACCATATACCACGAC
MgPot2-F	ACGACCCGTCTTTACTTATTTGG
MgPot2-R	AAGTAGCGTTGGTTTTGTTGGAT
