# Supplementary material for: BUI1 coordinates actin cytoskeleton remodeling and ROS homeostasis to confer broad-spectrum disease resistance in rice
Source: Stress Biol. 2026 Jul 2;6(1):48. doi: 10.1007/s44154-026-00321-5 (PMC13328597; doi:10.1007/s44154-026-00321-5)
Supplement: Supplementary file 2 — Supplementary Material 2. Table S1. Xoo-induced gene were categorized into 20 distinct clusters. [file 44154_2026_321_MOESM2_ESM.pdf]

Xoo-induced gene were categorized into 20 distinct clusters

Gene ID	BUI_0h			BUI_6h			BUI_12h			BUI_24h			BUI_48h			ZJ22_0h			ZJ22_6h			ZJ22_12h			ZJ22_24h			ZJ22_48h			gene_cluster
Os01g0873800	412	458	430	397.4	419.4	468	293	255	281	404.4	483.5	332.1	456.09	396.72	419.74	776.2	390.6	486.9	288	370.1	326.8	427.4	344.2	344.5	471.1	450.7	401.3	476	445	503	13
Os01g0734100	308	234	268	234.8	234.1	204.2	200	178	219	248.3	250	287	253.63	294.97	252.64	477.6	261.8	256.9	312.1	255.6	294.9	207	184.6	181.3	254.6	289.5	307	214	203	197	13
Os01g0563000	258	448	283	2607	2642	3111	641	548	741	288.8	264.2	194.7	144.77	181.09	152.06	286.6	300.9	186.5	1657	1581	1648	1792	2221	1907	229.2	210.3	311.4	253	250	187	10
Os01g0973300	401	485	431	572.2	595.7	525.9	485	502	483	363.8	403.5	294.5	499.63	399.52	482.31	931.4	397.8	353.3	425.5	463.6	456.1	544.2	453.9	458.6	412	351.7	335.5	492	453	452	6
Os01g0889800	592	590	617	1025	1152	1033	1033	1068	1030	668.2	699.5	791.2	853.4	684.22	567.05	1158	534.8	593.6	1283	1447	1311	663.2	674.9	885.5	624.3	606.2	587.7	502	607	598	10
Os01g0878400	13.3	18.6	7.54	4.926	8.136	5.19	8.61	8.22	3.46	32.76	47.14	34.81	25.036	24.27	11.879	0	5.153	10.36	0.929	4.031	1.724	5.915	3.563	2.167	15.85	3.771	6.579	4.63	7.65	11.7	7
Os01g0544450	2.04	0	1.08	6.568	0	3.46	3.91	1.9	1.38	0.862	0	0	0	0	0	0	0	0	5.574	4.031	0.862	2.958	0.713	4.333	0	0	0	1.85	0	0.83	12
Os01g0810300	937	714	792	5401	4365	6929	1052	1257	1589	1117	1006	963.4	969.87	1047.3	771.37	1278	826.5	768.7	7652	6934	8293	1102	1154	1378	1049	1340	1353	880	966	1066	10
Os01g0855700	1.02	0	0	0	1.808	0	0.78	0	0.69	0	0	0	0	0	0	0	2.061	0	0	0	0	1.479	0.713	1.444	0	0	0	0	0	0	9
Os01g0923000	17.3	20.5	10.8	72.25	59.66	80.45	173	180	177	14.66	9.868	11.29	7.6196	25.203	26.135	0	14.43	18.65	64.1	51.6	52.6	147.9	188.1	157.4	14.79	7.543	17.54	13	15.3	24.2	18
Os01g0647700	109	135	105	152.7	162.7	109.9	204	156	149	86.22	104.2	87.5	96.878	93.345	98.996	167.2	123.7	144	115.2	104	103.5	158.2	196.7	174.1	98.24	92.4	83.33	137	104	127	17
Os01g0816400	1549	1817	1734	1084	1136	1175	874	944	934	1277	1365	1010	1505.4	1347	1667.1	2914	1503	1537	783.1	812.7	883.8	1164	1183	1034	1481	1383	1322	1681	1514	1688	13
Os01g0848475	2.04	3.73	1.08	4.105	2.712	0.865	12.5	10.8	5.53	9.484	4.386	3.763	3.2656	1.8669	1.5839	0	5.153	5.18	3.716	1.613	1.724	2.958	4.988	0.722	5.282	1.886	8.772	4.63	6.69	4.17	5
Os01g0688200	47.9	41	34.5	37.76	33.45	62.28	178	142	149	44.83	43.86	93.14	40.275	42.005	41.974	71.65	58.74	47.66	101.3	76.6	85.36	184.1	155.4	181.3	53.87	64.11	67.98	74	65	57.5	18
Os01g0646700	941	1063	928	747.1	718.7	791.5	803	668	670	894.1	896.9	970.9	664	735.56	775.33	2018	1056	1135	821.2	817.6	905.3	1312	987	1006	999.3	1075	1239	1247	1026	1011	13
Os01g0633900	33.6	22.4	26.9	29.56	31.64	30.28	19.6	21.5	22.1	14.66	26.31	30.11	21.77	28.004	24.551	35.82	30.92	24.86	32.51	36.28	33.63	43.62	33.49	21.67	26.41	28.29	37.28	31.5	34.4	35.8	16
Os01g0852100	111	92.2	93.7	59.93	75.03	66.61	75.9	58.2	66.3	76.73	71.27	51.75	82.728	84.011	101.37	143.3	89.66	98.42	43.66	63.7	49.15	227	233.7	163.9	128.9	106.5	100.9	145	111	120	17
Os01g0604000	2.04	8.38	1.08	4.926	4.52	3.46	18.8	17.7	22.1	13.79	6.578	12.23	2.177	1.8669	5.5438	0	5.153	4.144	13.93	12.09	6.898	10.35	14.97	20.95	4.225	8.486	6.579	5.55	3.83	5.83	18
Os01g0510500	475	510	495	437.6	372.4	381.5	403	338	357	453.5	468.2	414.9	453.91	415.39	479.93	967.2	492.6	456.9	335.4	358	367.3	350.5	365.6	353.2	461.6	480.8	372.8	522	543	508	13
Os01g0116900	1376	1683	1874	1477	1613	1248	3689	3404	3437	2003	1968	1534	2125.9	1834.2	2094	2555	1666	1510	1408	1639	1325	2430	2469	2345	1800	1471	1474	1628	1735	1738	18
Os01g0934900	52	45.6	49.6	65.68	58.76	46.71	54.8	43	49.8	84.49	53.72	94.08	54.426	70.942	49.894	83.59	57.71	45.59	68.74	59.66	88.81	38.45	35.63	37.56	47.54	56.57	61.4	48.1	56.4	38.3	16
Os01g0823951	0	0	0	0.821	0.904	0	1.57	1.27	0	0	2.193	0.941	3.2656	0	0	0	1.031	0	0	4.838	1.724	0.739	2.138	2.167	0	0.943	0	1.85	0	0	15
Os01g0661000	69.3	87.6	79.7	189.6	168.1	160	118	111	90.5	69.84	63.59	57.39	66.4	87.744	51.478	131.4	74.2	69.41	151.4	143.5	167.3	132.4	125.4	123.5	76.06	92.4	92.1	96.2	88	87.5	10
Os01g0362533	51	41.9	45.3	64.04	84.97	75.26	59.5	55	69.8	44.83	39.47	79.03	57.692	84.944	45.142	143.3	48.43	73.56	94.75	94.33	111.2	56.19	52.02	46.95	35.92	55.63	57.02	41.6	51.6	34.2	6
Os01g0229600	872	823	844	843.1	857.9	749.1	526	528	493	950.1	1099	1183	1255.1	1019.3	1087.4	1146	947.1	895.1	861.1	779.7	945	237.3	258	384.2	986.6	820.3	839.9	635	540	674	3
Os01g0833700	161	119	119	91.13	114.8	133.2	197	171	212	142.3	105.3	113.8	115.38	122.28	139.39	131.4	101	92.21	144	134.6	116.4	168.6	184.6	155.3	123.6	109.4	144.7	133	120	110	18
Os01g0329800	172	173	139	164.2	158.2	122.8	118	153	126	136.2	132.7	174.1	143.68	127.88	144.93	203	174.2	161.6	162.6	143.5	168.1	144.9	162.5	146.6	168	158.4	243.4	147	188	161	11
Os01g0205100	259	273	260	312.8	317.3	295	241	268	237	224.2	277.4	230.5	256.89	261.37	231.25	441.8	260.7	284.9	288.9	323.3	311.3	337.2	327.8	299	251.4	238.5	232.4	255	268	242	6
Os01g0127450	14.3	13	14	13.96	14.46	24.22	39.9	57.6	58	30.18	27.41	38.57	11.974	8.4011	11.879	11.94	11.34	15.54	22.29	33.86	14.66	49.54	29.22	39.72	12.68	14.14	15.35	5.55	16.3	10.8	18
Os01g0570700	812	863	661	1075	1127	1001	808	805	839	690.6	665.5	780.9	514.87	536.73	540.91	979.2	599.8	694.1	824	738.6	948.4	902.8	937.8	1009	724.7	902.3	877.2	638	581	587	12
Os01g0226600	195	228	303	13.96	5.424	19.9	0	3.16	2.07	487.1	436.4	431.8	309.14	346.31	372.22	609	294.7	320.1	34.37	20.16	18.97	2.218	6.414	3.611	584.2	460.1	410.1	468	321	369	3
Os01g0881966	185	189	190	249.6	206.1	235.3	138	144	183	161.2	222.6	191.9	268.86	198.83	234.42	406	190.6	232.1	176.5	198.3	184.5	211.5	186	187.1	222.9	202.7	171	212	241	257	13
Os01g0164800	24.5	20.5	20.5	14.78	18.98	23.36	2.35	4.43	10.4	12.93	8.771	6.586	13.062	10.268	22.967	95.53	41.22	37.3	21.37	18.54	16.38	17.75	17.82	12.28	15.85	21.69	15.35	30.5	31.6	32.5	13
Os01g0837600	613	664	600	1188	827.1	1214	294	424	439	573.4	640.3	286	466.98	456.46	626.45	1516	360.7	522.2	1381	1447	1336	130.1	231.6	208.7	363.4	251.7	320.2	504	808	642	16
Os01g0221700	530	575	558	824.3	898.5	797.6	730	834	858	462.1	549.3	607.8	556.23	422.85	346.09	513.5	439	537.7	659.6	691	722.5	826.6	744.7	871.7	436.3	434.7	436.4	318	364	429	12
Os01g0290700	2.04	0	1.08	0	1.808	0.865	0.78	1.9	3.46	1.724	5.482	1.882	1.0885	4.6673	4.7518	0	7.214	2.072	0.929	5.644	6.898	8.133	8.552	5.056	0	0	2.193	0	1.91	0	9
Os01g0200400	109	133	126	140.4	151.9	162.6	189	190	156	166.4	133.8	129.8	140.42	141.88	133.05	155.2	116.5	110.9	172.8	157.2	143.1	133.1	148.2	145.9	129.9	134.8	162.3	133	120	147	12
Os01g0976600	401	368	384	481.1	461	478.4	414	430	470	441.4	409	444.1	408.19	497.53	396.77	525.4	352.4	395.8	615	577.3	579.4	431.8	447.5	414.6	388.7	404.5	478.1	343	387	378	10
Os01g0668100	0	0	0	0.821	3.616	0.865	0	0	0	7.76	1.096	0.941	0	0	0	0	0	0	0	0	0	0	0	0	0	0.943	0	0.93	0	0	7
Os01g0676900	0	0	0	0	0	0.865	0	0	0	0	0	0	0	0.9335	0	0	0	0	0	0	0	0.739	0	0	0	0	0	0	0	0	2
Os01g0172900	17.3	13	12.9	2.463	3.616	6.055	1.57	2.53	4.84	6.035	5.482	9.408	6.5311	5.6007	5.5438	0	10.31	11.4	6.503	3.225	2.587	5.915	1.425	2.167	4.225	2.829	0	18.5	6.69	9.16	14
Os01g0217400	0	0.93	1.08	0	0	1.73	0	0	0	0	0	0	0	0	0	0	0	0	0.929	0	0	0	0.713	0	0	0	0	0	0	0	2
Os01g0764300	132	115	137	108.4	123.8	121.1	86.9	93	108	130.2	125	141.1	117.56	116.68	118	262.7	137.1	147.1	116.1	154.8	115.5	102.8	102.6	104	159.5	135.8	144.7	113	147	108	13
Os01g0514700	2.04	0	0	1.642	0	0.865	0	0	1.38	0	0	0	0	0	1.5839	0	0	0	0	0	0	0.739	1.425	0	0	0	4.386	0	0.96	0	11
Os01g0228300	545	492	557	375.2	404.1	403.1	469	510	396	575.1	621.7	522.2	714.07	709.42	717.52	979.2	622.4	634.1	358.6	428.9	382	466.6	434	380.6	602.1	600.6	598.7	683	636	783	13
Os01g0727200	20.4	27	39.9	56.65	47.91	37.2	69.7	68.3	70.5	63.8	60.3	44.22	35.921	34.538	44.35	23.88	22.67	26.94	29.73	61.28	49.15	53.24	45.61	50.56	25.35	39.6	32.89	31.5	20.1	25.8	18
Os01g0818400	120	127	94.8	192.1	170.9	206.7	198	159	189	101.7	77.84	125.1	101.23	99.879	100.58	214.9	138.1	139.9	183	177.4	238.8	146.4	173.2	205.1	101.4	124.5	133.8	112	138	132	12
Os01g0351300	414	387	381	519.7	451.1	521.6	622	686	594	338	314.7	339.6	332	305.24	323.91	394.1	391.6	390.6	306.6	390.2	372.5	617.4	616.4	610.3	388.7	383.7	381.6	375	338	348	18
Os01g0337160	16.3	30.7	15.1	36.12	58.76	33.74	86.1	92.3	91.2	33.63	38.37	36.69	16.328	21.469	22.175	11.94	16.49	22.79	44.59	47.57	43.11	46.58	33.49	55.61	22.18	16.97	21.93	13	26.8	15.8	18
Os01g0620400	0	0	1.08	1.642	0	0	0	0	0	0	0	0	0	0	0	0	0	0	0	0.806	0	3.697	0.713	0	0	0	2.193	0	0	0.83	9
Os01g0299300	34.7	36.3	29.1	76.35	88.59	83.91	36.8	67	60.8	18.11	13.16	5.645	16.328	16.802	16.631	83.59	26.79	14.5	45.52	55.63	40.52	76.9	49.88	48.39	11.62	16.03	35.09	11.1	23	15	12
Os01g0967700	1.02	7.45	0	1.642	2.712	3.46	2.35	3.8	0.69	1.724	3.289	0	1.0885	0	3.1679	0	0	0	0	1.613	1.724	1.479	0.713	3.611	0	0	2.193	2.78	0.96	3.33	2
Os01g0772900	1.02	0	0	0.821	0.904	0.865	0	0	0	0	2.193	0	0	0.9335	0.792	0	0	0	0.929	0.806	0	0	0	0	0	0	0	0.93	0	0	2
Os01g0175733	0	2.79	0	0	0	0	0	0	0	0	1.096	0	0	0.9335	0	0	0	1.036	0	0	0	0	0	0	1.056	3.771	0	0.93	0.96	0	1
Os01g0700100	1765	1637	1810	22.99	18.98	68.34	36.8	27.2	69.8	1534	2029	954.9	1404.2	1333	1427.9	4287	1680	1268	85.46	69.34	48.28	67.28	84.09	82.34	1763	1879	2243	1935	2336	2197	13
Os01g0354175	2.04	5.59	2.16	0.821	0.904	0	0	0	2.07	0	2.193	0.941	1.0885	2.8004	3.1679	0	10.31	2.072	2.787	0	0.862	0	1.425	1.444	0	1.886	2.193	0	0.96	0.83	14
Os01g0566100	104	83.8	116	825.1	908.5	774.2	714	846	736	78.46	49.34	47.04	95.79	94.279	105.33	179.1	99.96	84.96	719	701.5	680.3	727.6	698.4	698.4	89.79	75.43	50.44	109	80.3	88.3	12
Os01g0742766	29.6	8.38	17.2	18.88	18.98	16.44	25.8	20.9	12.4	18.97	17.54	32.93	18.505	21.469	22.175	23.88	10.31	18.65	23.22	16.93	31.04	22.92	19.24	11.56	30.63	19.8	26.32	21.3	21	11.7	20
Os01g0512400	8.16	10.2	9.7	2.463	6.328	2.595	7.05	5.06	8.98	19.83	6.578	12.23	3.2656	6.5342	5.5438	23.88	0	2.072	6.503	6.45	11.21	5.915	3.563	5.778	2.113	12.26	4.386	2.78	8.61	5	6
Os01g0766966	10.2	10.2	9.7	13.96	16.27	14.71	5.48	12.7	5.53	10.35	12.06	7.527	10.885	5.6007	8.7116	11.94	15.46	15.54	8.361	3.225	3.449	15.53	14.25	12.28	9.507	7.543	8.772	6.48	14.3	15.8	8
Os01g0938200	40.8	50.3	49.6	68.96	59.66	70.07	59.5	54.4	50.4	35.35	67.98	37.63	50.072	37.338	49.102	23.88	42.25	43.51	38.09	38.7	36.21	53.24	48.46	70.78	34.86	30.17	39.47	28.7	27.7	34.2	2
Os01g0327500	0	0	0	0	0	0	0	0	0	0	0	0	0	0	0	11.94	0	0	0	0	0	0	0	0.722	0	0	0	0	0	0	6
Os01g0868900	0	0	0	0	0	0	0	0	0.69	0	0	0	0	0	0	0	0	0	0	0	0	0.739	0	0	0	0	0	0	0	0	9
Os01g0911200	665	878	811	953.2	1034	994.8	600	618	597	669.9	609.6	316.1	563.85	563.8	622.49	788.1	636.9	655.8	562	557.1	517.3	971.6	883.7	712.1	560.9	515.7	495.6	648	588	705	2
Os01g0898800	1170	1330	977	102.6	83.17	112.5	162	170	84.3	1837	1965	2043	1877.7	1730.6	2093.2	1660	1409	1414	121.7	112.1	102.6	135.3	160.3	158.9	2017	2439	2498	1567	1565	1851	3
Os01g0898600	41.8	48.4	46.3	13.96	33.45	28.55	22.7	20.9	18	63.8	50.43	59.27	48.983	54.14	54.646	83.59	36.07	49.73	33.44	22.58	25	18.48	18.53	13.72	77.11	49.97	54.82	44.4	63.1	60	3
Os01g0926400	20.4	5.59	5.39	7.389	9.04	3.46	7.83	0	2.76	5.173	1.096	1.882	1.0885	5.6007	3.9598	0	5.153	5.18	1.858	3.225	4.311	6.654	12.83	7.222	3.169	2.829	4.386	6.48	5.74	5	19
Os01g0929000	10.2	12.1	10.8	30.38	23.5	17.3	60.3	39.8	22.1	8.622	5.482	4.704	6.5311	1.8669	14.255	0	4.122	6.216	18.58	12.9	8.622	22.92	32.78	27.45	9.507	4.714	6.579	10.2	5.74	5	18
Os01g0840700	389	307	292	512.3	495.4	516.4	585	460	628	409.5	384.8	654.8	367.92	438.72	308.07	991.1	343.2	307.7	635.4	536.2	585.4	468	458.9	434.1	314.8	452.6	528.5	303	371	244	6
Os01g0839500	556	529	538	791.4	774.7	545.8	933	1160	1219	555.2	547.1	509.9	425.61	355.65	415.78	979.2	468.9	470.4	722.7	578.9	628.6	668.4	824.5	775	390.9	344.1	364	362	336	397	18
Os01g0373800	10.2	14.9	1.08	5.747	2.712	6.92	1.57	8.86	0.69	0.862	2.193	8.467	2.177	0	1.5839	0	5.153	0	8.361	7.257	39.66	0.739	8.552	2.889	1.056	2.829	2.193	0	0	0.83	16
Os01g0176000	14.3	14	29.1	54.18	42.49	43.25	50.1	52.5	73.2	57.77	43.86	28.22	42.452	38.272	55.438	35.82	14.43	22.79	42.73	96.75	24.14	49.54	88.37	49.11	29.58	24.51	28.51	26.8	20.1	17.5	12
Os01g0834200	1035	1059	1050	834.1	730.4	893.6	905	892	926	921.7	887	764.9	937.22	963.32	1134.9	2125	1025	1018	753.4	719.2	788.1	1011	993.4	863.1	995.1	887.2	929.8	1131	1076	1015	13
Os01g0868200	199	204	179	169.1	153.7	171.3	325	266	299	295.7	246.7	334	223.15	277.24	270.06	214.9	239.1	265.2	246.2	228.2	244	201.1	199.5	215.2	236.6	243.3	353.1	223	217	199	5
Os01g0182100	314	351	347	377.6	433.9	410	290	357	314	378.5	325.6	338.7	336.35	441.52	393.61	394.1	389.5	344	376.2	316.1	376.8	244	316.4	320	328.5	330.9	337.7	382	355	366	16
Os01g0904200	1593	1578	1599	743.8	777.4	804.5	560	665	574	1320	1574	1519	1464.1	1585	1505.5	2615	1967	1742	1041	811.9	920.8	497.6	570.8	605.2	1661	1690	1710	1584	1562	1653	13
Os01g0825500	2551	2187	2259	575.5	614.7	561.4	14.9	13.3	7.6	1851	2105	1848	1742.7	2283.2	2295.1	5111	4175	3182	657.7	984.5	850.1	58.41	65.56	42.61	4430	4920	3925	5760	4233	4996	13
Os01g0833500	12.2	15.8	9.7	0	0	0.865	0	3.16	1.38	2.587	3.289	0.941	3.2656	3.7338	1.5839	0	5.153	13.47	0.929	0.806	3.449	0.739	9.977	0.722	1.056	1.886	2.193	11.1	8.61	6.66	14
Os01g0190400	21.4	7.45	22.6	18.88	14.46	8.65	8.61	8.22	11.1	7.76	16.45	9.408	8.7082	8.4011	7.1277	11.94	12.37	12.43	10.22	8.063	8.622	14.05	13.54	13.72	7.395	14.14	13.16	5.55	7.65	7.5	19
Os01g0901900	453	689	542	2773	3286	2664	2448	2726	2467	625.1	773	494.9	714.07	451.79	408.65	704.5	243.2	373	1619	2174	1616	1826	1868	1998	458.5	241.4	232.4	225	242	340	12
Os01g0974600	0	0	0	0	0.904	0	0	0	1.38	0	0	0	0	0	0	0	0	0	0	0	0	0	0	0.722	0	0	0	0	0.96	0	18
Os01g0653350	0	0	0	0	0.904	0	0	5.69	2.76	0	0	0	0	0	0	0	0	0	0	0	0	1.479	0	5.056	0	0	0	0	0	0	18
Os01g0561800	1.02	1.86	1.08	0	0.904	0	0	0	0	3.449	5.482	0	0	0.9335	2.3759	0	2.061	1.036	0	0	0	0	0	0	1.056	1.886	0	0.93	0.96	0	7
Os01g0134700	133	230	177	104.3	110.3	103.8	98.6	201	68.4	164.7	108.5	55.51	128.45	97.079	93.452	214.9	143.2	194.8	54.81	83.05	41.39	160.4	349.9	145.2	132	79.2	41.67	81.4	126	137	17
Os01g0784000	88.7	115	75.4	190.5	177.2	160.9	68.1	71.5	97.4	84.49	73.46	61.15	84.905	71.876	83.948	83.59	85.53	105.7	123.6	173.4	172.4	127.9	106.2	91.73	67.61	59.4	52.63	105	120	103	10
Os01g0275600	1671	1739	1762	1823	1929	1926	1765	1543	1417	1633	1516	1538	1792.8	1772.6	1751.8	2388	1504	1721	1648	1349	1606	1415	1351	1196	1460	1500	1403	1359	1516	1437	6
Os01g0829400	44.9	31.7	45.3	66.5	65.99	56.23	110	79.7	65.6	49.14	65.78	87.5	53.337	47.606	34.055	143.3	47.4	46.62	80.82	89.5	107.8	51.02	35.63	62.11	48.59	52.8	61.4	35.2	29.6	35	6
Os01g0779400	1522	1603	1469	1139	1180	1153	990	1079	1037	1154	1414	1083	1529.4	1427.2	1580	2854	1495	1551	969.8	1173	1070	1418	1406	1260	1511	1426	1094	1657	1495	1594	13
Os01g0867900	892	741	885	529.5	574	576.1	860	796	844	573.4	641.4	541	588.89	555.4	621.69	955.3	814.1	851.6	567.6	557.9	552.7	923.5	787.5	837.8	574.7	586.4	559.2	615	591	648	14
Os01g0734000	1.02	2.79	5.39	1.642	1.808	2.595	0	0	0	3.449	1.096	0	4.3541	0.9335	3.1679	0	1.031	0	5.574	0	0	0	0.713	0.722	0	0.943	0	0	1.91	2.5	4
Os01g0870500	130	162	161	103.4	116.6	125.4	130	101	111	231.9	255.5	191.9	149.13	168.95	188.49	262.7	190.6	195.8	121.7	126.6	127.6	143.4	149.7	137.2	307.4	295.1	291.7	287	189	199	3
Os01g0762300	573	582	548	251.2	205.2	268.2	1437	1464	1657	803.6	865.1	1245	845.78	797.17	845.03	1540	903.8	813.3	368.8	394.3	342.3	949.4	849.5	1272	1120	1234	1252	740	706	693	5
Os01g0689900	402	350	389	251.2	250.4	246.5	453	287	294	514.7	492.3	580.5	346.15	360.31	345.3	573.2	471	340.9	377.2	360.4	362.1	368.2	342.1	367.6	405.6	461	603.1	362	354	293	20
Os01g0720500	976	817	649	179.8	33.45	114.2	3.91	8.22	3.46	507.8	307	3207	301.52	362.18	251.85	704.5	1078	1478	900.2	598.3	1305	5.915	10.69	13.72	626.4	1558	1278	322	668	355	20
Os01g0548900	39.8	49.4	31.2	68.96	54.24	57.09	115	73.4	60.8	42.25	41.66	41.4	43.541	42.939	49.894	83.59	36.07	43.51	78.03	61.28	64.67	90.94	86.23	91	25.35	25.46	39.47	39.8	34.4	42.5	12
Os01g0677000	0	0	0	0	0	0	0	0	0	0	0	0	0	0	0	0	0	0	0	0	0	0	0	5.056	0	0	0	0	0	0	9
Os01g0504950	7.14	4.66	4.31	6.568	12.66	10.38	11	3.8	8.29	6.897	2.193	6.586	4.3541	3.7338	0.792	0	4.122	0	13.01	3.225	13.8	6.654	4.988	12.28	2.113	6.6	2.193	4.63	0	1.67	12
Os01g0668200	27.5	46.6	30.2	44.33	45.2	50.17	13.3	17.1	18	12.07	21.93	8.467	20.682	21.469	39.598	71.65	30.92	41.44	22.29	27.41	18.11	36.23	38.48	28.89	19.01	25.46	21.93	31.5	25.8	37.5	8
Os01g0698200	0	0	0	0	0	0	0.78	0	0	0	0	0	0	0	0	0	0	0	0	0	0	0.739	0	0	0	0	0	0	0	0	9
Os01g0341000	9.18	1.86	2.16	4.926	7.232	5.19	7.05	4.43	4.84	6.035	4.386	8.467	6.5311	6.5342	2.3759	0	0	6.216	0.929	4.031	9.484	2.218	1.425	2.889	2.113	5.657	6.579	3.7	4.78	0.83	15
Os01g0882400	11.2	29.8	18.3	17.24	4.52	5.19	13.3	7.59	9.67	17.24	21.93	13.17	19.593	15.869	14.255	11.94	14.43	14.5	13.01	10.48	15.52	19.22	14.25	14.44	12.68	14.14	13.16	21.3	15.3	18.3	14
Os01g0610050	53	40.1	64.7	79.63	91.3	47.58	40.7	77.2	65	60.35	62.49	32.93	71.842	70.009	45.934	107.5	50.5	53.87	45.52	50.8	43.11	23.66	43.47	25.28	58.1	20.74	39.47	51.8	18.2	50	6
Os01g0862300	600	645	651	614.1	592.1	602.1	889	794	768	684.6	700.6	639.8	668.35	660.88	741.28	1314	632.7	606.1	584.3	596.6	575.1	792.6	720.5	717.2	657.1	668.5	714.9	661	623	666	6
Os01g0957100	0	0	1.08	0	0	0	0	0.63	0	0	0	0	0	0	0	0	0	0	0	0	0	0	0	0	0	0	0	0	0	0	14
Os01g0836600	8.16	3.73	14	0	0	0.865	0	0	0	4.311	7.675	13.17	1.0885	9.3345	2.3759	0	28.85	26.94	1.858	1.613	0	0	0	0	13.73	9.428	4.386	5.55	1.91	5.83	3
Os01g0203300	113	116	128	174	141.9	142.7	164	136	149	119	120.6	143	137.15	136.28	112.46	238.8	127.8	146.1	175.6	178.2	175.9	208.5	171.7	145.9	98.24	136.7	125	145	170	142	6
Os01g0686400	304	234	283	147.8	149.2	206.7	294	261	236	287.1	299.3	319.9	246.01	316.44	277.19	250.8	275.2	267.3	219.2	214.5	211.2	206.3	227.3	236.9	219.7	307.4	280.7	233	228	236	20
Os01g0823300	583	456	441	524.6	559.6	618.5	493	404	570	571.6	484.6	971.9	589.98	533.93	416.57	1218	488.5	469.3	704.1	674.1	713.1	394.1	426.9	446.3	498.6	703.4	677.6	327	465	287	6
Os01g0216400	0	0	0	0	0	0	0	0	0	0	0	0	0	0	0	0	0	0	0	0	0	0	0	0.722	0	0	0	0	0	0	9
Os01g0571800	0	0	2.16	0	0	1.73	1.57	0	0	0	0	0	0	0	0.792	0	0	2.072	0	3.225	0	0	0	0	2.113	0	0	0	0	0.83	16
Os01g0172400	4846	4640	4051	3264	3473	3420	2807	3058	2679	3497	2976	2768	2759.4	2999.2	3361.1	4920	4077	4024	2660	2720	2842	2626	3244	2628	3997	3473	3460	3614	2829	3282	14
Os01g0664200	42.8	34.5	29.1	32.02	20.79	38.06	30.5	23.4	11.7	21.55	23.02	35.75	27.213	30.804	15.047	59.7	42.25	30.05	37.16	26.61	30.18	44.36	44.9	46.22	27.47	68.83	74.56	55.5	54.5	29.2	11
Os01g0338200	646	717	714	1247	1470	1165	731	1229	1085	582.8	637	496.8	650.93	627.28	721.48	1612	839.9	813.3	1072	1129	1210	1248	1516	988	868.3	642.1	675.4	738	830	845	12
Os01g0795300	20.4	14	19.4	14.78	22.6	19.03	20.4	29.1	22.8	31.04	14.25	18.82	18.505	17.736	13.463	0	11.34	16.58	18.58	8.869	15.52	14.05	26.37	34.67	13.73	10.37	19.74	16.7	22	12.5	18

Os01g0212700	2.04	0.93	0	1.642	2.712	10.38	0	0	1.38	0	0	0	0	0.9335	0	0	0	2.072	0	3.225	0.862	1.479	0	0	1.056	1.886	0	2.78	0	0	2
Os01g0860900	3.06	1.86	1.08	1.642	1.808	3.46	4.7	3.8	4.84	1.724	3.289	0.941	2.177	3.7338	3.9598	11.94	2.061	3.108	2.787	2.419	0.862	1.479	0	2.167	5.282	0.943	2.193	0.93	6.69	1.67	6
Os01g0760400	63.2	72.7	80.8	68.14	74.13	81.31	44.6	35.4	38.7	43.97	47.14	46.1	54.426	42.005	48.31	59.7	47.4	76.67	41.8	53.21	43.11	57.67	48.46	51.28	61.27	61.28	52.63	86	40.2	41.7	8
Os01g0532700	17.3	27	17.2	45.97	35.25	34.6	53.2	48.1	49.8	33.63	25.22	37.63	26.124	31.737	27.719	47.76	39.16	23.83	29.73	51.6	48.28	38.45	47.03	39	29.58	25.46	21.93	45.3	30.6	39.2	12
Os01g0724700	40.8	23.3	42	19.7	14.46	16.44	30.5	44.3	26.3	27.59	5.482	6.586	14.151	14.935	19.007	11.94	13.4	15.54	19.51	13.71	0.862	11.09	27.08	10.83	5.282	3.771	6.579	11.1	7.65	14.2	19
Os01g0114700	2.04	6.52	5.39	0.821	4.52	0.865	2.35	5.69	2.76	12.07	5.482	4.704	7.6196	5.6007	11.879	11.94	6.183	5.18	0.929	3.225	2.587	3.697	14.97	2.889	21.13	9.428	2.193	8.33	8.61	5.83	13
Os01g0153700	63.2	73.6	88.4	53.36	52.43	57.96	77.5	36.7	66.3	97.43	81.13	46.1	64.223	69.075	76.029	131.4	46.37	60.09	63.17	65.31	81.05	81.33	84.09	78.72	53.87	66	63.59	64.8	85.1	69.1	6
Os01g0624500	4578	3437	5100	1798	1750	1861	2219	1980	1924	6355	5303	6319	5515.5	5340.3	5531.9	4263	2797	2729	2586	2106	2166	1371	1544	1548	5360	4265	4770	3219	2998	3005	3
Os01g0279800	851	843	815	898.1	949.2	834.8	929	822	860	914.8	896.9	984.1	773.94	852.24	739.7	1218	756.4	756.3	892.7	889.3	845.8	662.5	677.7	818.3	819.7	818.4	947.3	683	706	707	6
Os01g0842200	4693	3643	4469	1342	1476	1222	1688	1780	1798	2224	2472	2360	3165.4	2991.7	3377.7	8478	4885	4402	1686	1798	2156	1936	2243	1980	3173	2972	2621	3188	3252	3684	13
Os01g0878900	1.02	0	1.08	0	0	0	1.57	0	0	0	0	0	0	0.9335	0.792	0	0	0	0.929	0	0	0	1.425	0	0	0	0	0	0	0	19
Os01g0745300	358	383	342	470.4	461	500	715	725	738	313.8	444	455.4	520.31	350.98	367.47	501.5	301.9	388.5	478.4	504.7	530.3	625.5	579.4	637.7	363.4	368.7	311.4	307	308	341	18
Os01g0392100	460	505	482	394.9	419.4	359	777	739	728	565.6	643.6	599.3	624.81	564.74	514.78	1111	552.4	474.5	555.5	584.6	521.6	436.2	501	572.7	479.6	545	581.1	521	586	500	6
Os01g0699900	0	0	0	0	0	0.865	0	0	0	0	0	0	0	0	0	0	0	0	0	0	0	0	0	0.722	0	0	0	0	0	0	9
Os01g0535400	171	205	212	161.7	119.3	144.5	101	114	131	109.5	105.3	49.86	115.38	103.61	132.26	179.1	152.5	140.9	83.61	125.8	79.32	151.6	156.8	122.8	128.9	79.2	92.1	185	158	152	8
Os01g0915350	592	491	511	356.3	369.7	359	90.8	162	176	324.2	322.3	248.4	367.92	369.65	408.65	871.7	456.5	512.8	152.3	242.7	212.1	371.2	309.3	163.2	403.5	403.5	274.1	515	427	457	13
Os01g0810200	168	157	177	220.8	234.1	227.5	68.1	81	83.6	124.2	165.6	155.2	141.51	159.62	122.75	286.6	145.3	147.1	196.9	237.9	273.3	80.59	67.7	59.22	143.7	158.4	129.4	171	146	170	16
Os01g0891300	2.04	0.93	1.08	1.642	0.904	1.73	1.57	1.27	0	0	0	0.941	2.177	0.9335	0	0	1.031	1.036	0	0.806	0.862	0.739	0	0	0	0.943	0	2.78	0	0	19
Os01g0283100	1126	1063	1005	870.2	912.1	864.2	1259	1106	1271	1148	1106	1398	977.49	1076.3	969.37	1134	1035	1062	1027	800.6	981.2	984.9	984.1	1018	1058	1091	1213	896	967	914	5
Os01g0709400	394	333	426	234.8	217	251.7	263	261	272	310.4	284	261.5	256.89	276.3	255.81	346.3	394.7	324.3	245.2	239.5	260.4	335.7	275.8	291.1	338	328.1	293.9	284	287	295	14
Os01g0511300	427	503	491	553.3	537.9	545	493	491	520	476.8	459.4	457.2	413.64	496.6	411.82	597	442.1	438.2	449.6	522.5	460.4	501.3	460.4	476.7	438.4	409.2	460.5	418	449	447	6
Os01g0829000	514	628	619	2368	2744	2181	1521	1708	1359	452.6	601.9	389.5	770.67	526.47	459.34	919.5	315.3	513.9	2177	2493	2217	1293	1400	1481	378.2	253.6	232.4	268	402	383	10
Os01g0235800	542	547	548	477.8	461.9	438.6	511	512	553	462.1	530.7	417.7	522.49	536.73	559.92	1134	566.8	530.5	470.1	416.8	447.5	561.9	501	516.4	607.4	530.8	578.9	550	547	532	6
Os01g0589500	1724	1511	1733	1473	1504	1460	1065	1088	1072	1699	1715	1925	1755.8	1952.8	1947.4	2090	1972	1794	1770	1753	1901	984.9	890.1	1011	1645	1756	1785	1592	1720	1635	3
Os01g0942900	354	814	282	692.1	538.8	711.1	87.7	143	207	275	227	164.6	628.08	341.64	226.5	262.7	344.2	346	438.5	599.1	425.9	290.6	225.9	191.4	218.7	284.7	179.8	259	697	323	2
Os01g0298400	32.6	44.7	19.4	10.67	8.136	4.325	20.4	0.63	20	12.93	3.289	2.822	1.0885	2.8004	2.3759	0	42.25	7.252	15.79	16.13	9.484	11.09	12.83	39	2.113	0	0	1.85	1.91	0	14
Os01g0519100	53	58.7	56	51.72	62.37	56.23	50.9	72.7	56	56.04	72.36	71.5	50.072	70.009	41.974	143.3	65.95	46.62	57.6	59.66	48.28	69.5	47.75	66.45	63.38	82.03	81.14	62	58.3	65.8	6
Os01g0924500	23.4	28.9	36.6	16.42	18.98	20.76	14.9	9.49	5.53	31.04	30.7	40.46	46.806	23.336	47.518	59.7	41.22	19.68	12.08	29.83	24.14	20.7	14.97	17.33	40.14	35.83	48.24	41.6	25.8	26.7	13
Os01g0281200	5.1	2.79	4.31	0	0	0	0	0	0	5.173	2.193	5.645	2.177	5.6007	0	0	8.244	3.108	0	0	0	0	0	0	3.169	1.886	4.386	0.93	2.87	4.17	3
Os01g0339400	5.1	0.93	0	1.642	1.808	0	1.57	1.9	1.38	0	0	0	1.0885	0.9335	0	0	1.031	1.036	0	0.806	1.724	1.479	0.713	0.722	1.056	0	2.193	0.93	0	0.83	19
Os01g0660900	446	469	528	897.3	895.8	798.4	294	261	229	728.5	702.8	583.3	910	813.04	705.64	644.8	566.8	459	1177	1187	1407	144.2	180.3	193.6	531.4	488.4	524.1	583	530	602	16
Os01g0843800	0	0	0	1.642	0.904	0	0	0	0	4.311	2.193	0.941	0	0	0	0	0	0	0	0	0	0	0	0	0	0	0	0	0	0	7
Os01g0372700	469	336	390	350.6	327.2	332.2	302	262	274	540.6	504.3	716	538.82	654.35	475.97	847.8	519.4	481.8	611.3	532.1	650.1	184.1	245.1	221.7	502.8	586.4	714.9	389	423	358	13
Os01g0885700	12.2	14	11.9	21.35	7.232	12.98	3.13	7.59	4.84	31.04	18.64	28.22	23.947	9.3345	19.799	35.82	15.46	13.47	33.44	21.77	19.83	8.133	7.839	7.945	21.13	18.86	24.12	16.7	22	9.16	16
Os01g0965800	5.1	4.66	4.31	8.21	18.08	13.84	7.05	6.33	6.22	11.21	13.16	5.645	14.151	4.6673	9.5036	0	3.092	9.324	26.94	11.29	29.32	4.436	5.701	8.667	4.225	9.428	17.54	7.4	4.78	8.33	16
Os01g0748300	0	0	0	0	0	0	0	0	0	0	0	0	0	0	0	0	1.031	0	0	0	0	0	0	0	0	0	0	0	0	0	1
Os01g0208600	781	809	780	588.6	550.5	645.3	723	692	653	642.3	623.8	611.5	677.06	778.5	768.21	1600	860.5	822.6	556.4	551.5	614.8	825.9	838.8	834.2	753.2	635.5	798.2	874	877	916	13
Os01g0284900	17.3	53.1	51.7	9.031	11.75	18.17	38.4	32.9	35.2	50.87	47.14	23.52	37.01	40.138	33.263	95.53	53.59	27.97	18.58	26.61	29.32	36.97	68.41	46.22	61.27	49.03	67.98	59.2	70.8	64.2	13
Os01g0612600	1776	1596	1555	888.3	936.5	889.3	1184	1324	1244	1235	1284	1152	1371.5	1325.5	1392.3	2806	1681	1592	940.1	945	794.1	1402	1481	1371	1525	1432	1305	1595	1488	1537	13
Os01g0781600	0	0	0	0	0.904	0.865	0	0	0	0	0	0	0	0	0	0	0	0	0	0	0	0	0	0	0	0	0	0	0	0	2
Os01g0820700	1.02	0	0	0	0	0.865	0	0	0	1.724	0	0	0	0	0	0	2.061	0	0	0	0	0.739	1.425	0.722	0	0	2.193	0	0	2.5	11
Os01g0754100	132	162	137	364.5	418.5	302.8	540	607	570	158.6	205	156.2	120.83	117.61	108.5	167.2	75.23	106.7	291.7	272.5	252.6	370.4	412.6	406.6	103.5	95.23	107.5	80.5	91.8	72.5	18
Os01g0150800	71.4	84.8	73.3	72.25	69.61	57.96	43.1	31	65.6	30.18	47.14	41.4	46.806	44.806	51.478	95.53	60.8	47.66	18.58	41.12	38.8	96.12	74.11	75.84	54.93	42.43	43.86	97.1	46.9	72.5	8
Os01g0916350	1.02	0	0	0.821	0.904	0	0	2.53	3.46	1.724	1.096	0	0	0	2.3759	0	3.092	0	0	1.613	0	2.218	0	0.722	0	0.943	0	0.93	1.91	1.67	5
Os01g0550300	####	####	####	10413	9812	10125	7960	7398	6937	15056	12009	11269	12308	12263	12578	32085	20744	17746	11354	13930	12206	7912	9802	9344	12714	12691	11881	####	####	####	13
Os01g0207200	26.5	21.4	16.2	29.56	20.79	62.28	37.6	13.3	20.7	20.69	19.74	27.28	6.5311	21.469	26.135	11.94	16.49	17.61	26.94	13.71	24.14	31.79	52.02	53.45	25.35	12.26	26.32	17.6	9.56	10	17
Os01g0167700	97.9	117	97	63.22	70.51	102.9	54.8	46.8	33.2	93.98	104.2	77.15	93.613	105.48	79.197	262.7	107.2	103.6	78.03	74.18	70.7	78.37	95.49	85.95	148.9	116	149.1	170	78.4	147	13
Os01g0784200	1.02	0	0	0.821	0	0	3.13	0	1.38	0	1.096	0.941	0	0	0	0	0	0	0	0	0	0	1.425	0.722	0	0	0	0.93	0	1.67	5
Os01g0331100	160	125	113	82.92	57.85	75.26	23.5	17.1	13.8	140.5	121.7	159	85.993	72.809	83.156	47.76	128.8	106.7	75.25	44.35	50.87	76.9	33.49	80.17	69.72	115	188.6	78.6	104	60	3
Os01g0844400	266	252	287	174.9	164.5	193.8	281	267	274	288	240.1	214.5	163.28	229.63	236.8	441.8	232.9	252.8	160.7	183	181.1	243.3	298.6	224.6	225	202.7	265.3	237	230	222	6
Os01g0943100	3.06	1.86	3.23	0.821	0.904	1.73	4.7	2.53	3.46	0.862	3.289	2.822	0	0.9335	2.3759	0	2.061	2.072	1.858	3.225	0.862	2.218	8.552	2.889	2.113	0.943	0	0.93	0.96	2.5	17
Os01g0313600	0	0	0	0.821	3.616	8.65	3.91	0	0.69	0	0	0	0	0	0	0	0	1.036	0	0	0	0.739	2.138	2.167	0	0	0	0.93	4.78	0	2
Os01g0619900	1178	1594	1073	1046	953.7	933.4	397	825	578	948.4	1173	1150	943.75	1849.2	1549.9	2161	1693	1993	804.5	1263	1117	2229	2460	1887	2593	2659	2581	1507	777	1769	1
Os01g0558500	350	402	380	485.2	499	395.3	481	456	433	372.5	358.5	298.2	450.65	392.05	429.25	585.1	350.4	370.9	372.5	402.3	354.4	464.3	418.3	441.3	397.2	423.3	368.4	506	423	436	6
Os01g0909150	0	0	0	1.642	0	0.865	0	0	0	0	0	0	0	0	0	0	0	0	0	0	0	0	0	0	0	0	0	0	0	0	2
Os01g0576100	3.06	6.52	2.16	1.642	1.808	0.865	3.13	3.16	2.07	1.724	1.096	5.645	2.177	5.6007	2.3759	11.94	5.153	6.216	0.929	4.031	1.724	2.218	0	2.167	6.338	11.31	10.96	15.7	11.5	10.8	13
Os01g0233400	0	0.93	0	0.821	0	0	1.57	0	0.69	0.862	0	0	2.177	0	1.5839	11.94	0	0	0	0	0.862	0	0	0	0	0	0	0	0.96	0	6
Os01g0589300	970	998	980	874.3	951	883.2	921	1002	1048	943.2	919.9	749.8	957.9	954.92	906.8	1445	994.5	1120	822.1	1026	1028	938.3	881.5	915.1	871.5	836.3	901.3	932	953	1045	6
Os01g0824000	5.1	1.86	5.39	7.389	8.136	7.785	14.9	11.4	15.2	3.449	3.289	3.763	7.6196	5.6007	0	0	3.092	8.288	8.361	8.063	2.587	22.92	27.79	15.17	4.225	2.829	0	0.93	6.69	4.17	17
Os01g0817400	0	0	0	0	0	0	0	0	0	0	0	0.941	0	0	0.792	0	0	0	0	0	0	0	0.713	0	0	0	0	0.93	0	0.83	1
Os01g0801700	640	455	585	563.2	553.2	527.7	538	493	554	655.3	634.8	963.4	852.31	774.76	743.66	668.7	627.6	620.6	639.1	611.2	689.8	407.4	470.3	414.6	724.7	832.5	782.9	551	588	602	20
Os01g0856850	0	1.86	0	0	0	2.595	0	0	0	0	0	0	0	0	0	0	0	0	0	0	0	0.739	0	0	0	0	0	0	0.96	0.83	8
Os01g0314100	751	782	801	1015	1046	910.9	824	1080	967	682	881.5	702.8	772.85	617.94	778.5	1337	644.1	684.8	848.1	828.9	756.2	1097	1020	838.5	683.5	590.2	583.3	753	824	834	6
Os01g0147900	30.6	22.4	23.7	17.24	19.89	23.36	42.3	29.7	23.5	37.94	33.99	56.45	47.895	35.471	36.43	23.88	24.73	41.44	37.16	24.19	32.76	17.01	17.82	31.78	36.97	49.03	37.28	28.7	26.8	24.2	20
Os01g0705700	48.9	41	32.3	1.642	0	0	3.13	8.86	4.15	1.724	2.193	1.882	7.6196	1.8669	0.792	0	12.37	13.47	2.787	0	0	6.654	8.552	10.11	2.113	1.886	10.96	2.78	0.96	0.83	14
Os01g0182600	####	####	####	32319	33054	33324	4401	4283	4382	10508	10690	9542	13611	12127	12931	22210	14596	13984	29716	33158	32206	3950	3503	3682	10809	10840	9383	####	####	####	10
Os01g0813400	3014	2545	2829	1678	1803	2023	1711	1532	1611	2657	2348	3061	2210.8	3287.6	2572.3	4132	3329	3158	2750	2075	2584	1468	1531	1715	2713	2906	3870	2786	2604	2521	13
Os01g0623200	61.2	19.6	67.9	32.84	15.37	58.82	84.6	64.5	60.1	25	8.771	63.03	48.983	64.408	58.605	83.59	76.26	35.23	88.25	42.73	81.05	78.37	74.83	221.7	17.96	41.49	100.9	70.3	66	45	9
Os01g0584100	1.02	0	6.47	0	0.904	0	0	0	0	3.449	1.096	0	1.0885	1.8669	0.792	0	2.061	1.036	0	0	0	2.218	1.425	0	1.056	0.943	0	2.78	0.96	0.83	1
Os01g0830300	142	146	196	158.4	147.3	152.2	226	201	240	229.3	211.6	238	212.26	247.36	199.58	214.9	223.6	218.6	249.9	246.7	231.1	162.7	173.9	198.6	229.2	216.9	274.1	187	202	167	20
Os01g0834125	1.02	0.93	0	0	0	0	0	0.63	0	0.862	0	0	0	0	0	0	0	0	0	0	0.862	0.739	0	0	0	0	0	0	0	0	19
Os01g0655400	1211	1315	1294	1316	1261	1207	1018	1128	1045	1257	1317	1085	1416.2	1263	1424.7	2627	1177	1273	963.3	1069	967.4	1216	1199	1059	1365	1203	1167	1424	1425	1508	13
Os01g0868400	0	0	0	0	0	0.865	0	0	0	0	0	0	0	0	0	0	0	0	0.929	0	0	0	0.713	0	0	0.943	0	0	0	0	16
Os01g0649200	202	156	154	156.8	155.5	185.1	325	300	340	253.5	187.5	207	140.42	134.42	203.54	143.3	131.9	138.8	199.7	191.1	225	351.9	379.8	354.6	194.4	168.8	217.1	155	150	162	18
Os01g0668500	0	1.86	1.08	0.821	0	0	0	0	0	2.587	0	0	0	0	0.792	0	0	0	0	0	0	0	0	0	0	1.886	6.579	0.93	0	0.83	11
Os01g0117500	73.4	117	120	64.86	58.76	65.74	43.1	60.7	61.5	231.9	194.1	172.2	157.84	120.42	182.15	346.3	163.9	128.5	80.82	70.15	91.4	68.76	86.23	55.61	268.3	155.6	153.5	222	115	131	13
Os01g0156300	4.08	0	4.31	1.642	0.904	3.46	4.7	1.27	2.07	0.862	0	1.882	1.0885	3.7338	0.792	0	2.061	0	4.645	0	0	0	0.713	0.722	1.056	0.943	2.193	0.93	0.96	0.83	19
Os01g0858900	83.6	107	136	75.53	75.93	54.5	355	163	281	183.6	163.4	131.7	93.613	88.678	107.71	107.5	128.8	106.7	58.52	89.5	68.12	158.2	192.4	190.7	132	81.08	109.6	97.1	119	106	5
Os01g0308200	1.02	1.86	0	0	1.808	0	0.78	0.63	3.46	6.897	6.578	1.882	2.177	4.6673	0	0	2.061	0	1.858	0	0.862	0	2.138	0.722	0	6.6	2.193	0	0.96	1.67	7
Os01g0678100	718	853	866	757.8	856.1	707.6	518	409	370	1193	876	804.4	949.19	852.24	927.39	704.5	554.4	680.7	450.5	512	571.6	269.9	290.8	312.7	721.5	534.6	519.7	697	624	651	7
Os01g0919400	2965	3948	3804	497.5	492.7	390.1	2515	2663	2627	2943	3315	2273	3642.2	2932.9	3557.5	6006	3558	3354	619.6	611.2	500.9	2444	2366	2805	2796	2726	2292	3856	4466	4549	13
Os01g0627933	719	778	997	1227	1346	1138	1375	1545	1336	753.6	847.5	785.6	1254	1027.7	1071.5	1409	749.2	826.8	1193	1365	1110	1227	1256	1229	977.1	707.1	695.2	900	941	947	12
Os01g0546900	189	157	193	127.3	107.6	173.9	222	184	176	192.3	177.6	188.2	188.31	253.9	217.79	214.9	153.5	163.7	145.8	163.7	172.4	216.6	203.1	197.9	198.6	136.7	214.9	166	167	177	5
Os01g0902200	451	416	457	435.9	429.4	466.3	331	364	353	332.8	362.9	386.7	354.86	400.45	399.94	800	467.9	436.2	477.5	509.6	490.6	371.2	306.4	312.7	360.2	355.5	309.2	423	450	422	6
Os01g0871600	0	0	0	0	1.808	0	0	0.63	0	0	0	0	0	0	0	0	0	0	0	0	0	0	0	0.722	0	0	0	0	0	0	2
Os01g0773200	773	794	759	729.8	780.1	795	604	605	564	688	799.3	725.4	961.16	839.17	916.3	1863	797.6	785.3	778.5	828.9	770	773.4	717.6	633.4	844	834.4	758.7	1020	969	1006	13
Os01g0978400	173	181	204	230.7	215.1	231.8	158	131	197	181.1	167.7	209.8	209	170.82	149.68	465.7	210.2	214.5	282.4	239.5	306.9	90.21	102.6	114.1	156.3	196.1	221.5	142	152	192	6
Os01g0718500	21.4	14.9	3.23	3.284	0.904	12.98	0	5.69	2.76	2.587	4.386	0	2.177	3.7338	7.9197	0	2.061	3.108	0.929	4.838	0	5.176	3.563	5.778	2.113	4.714	2.193	16.7	11.5	0.83	19
Os01g0223200	784	1001	762	490.9	449.3	578.7	70.5	114	117	663.9	635.9	574.8	388.6	842.91	714.35	1122	856.4	866.1	399.4	495.1	414.7	595.9	701.9	563.4	1015	1080	1208	1300	931	1127	13
Os01g0886700	1.02	1.86	1.08	2.463	5.424	4.325	0.78	0	3.46	1.724	0	2.822	1.0885	0.9335	2.3759	0	0	1.036	0.929	2.419	6.898	5.176	0	2.167	0	4.714	0	0.93	0.96	1.67	10
Os01g0235100	4785	4019	5099	229.1	291.1	291.5	336	463	296	4175	4895	5188	7789.4	7561.9	7122.9	7487	6367	5262	530.4	437.8	363.9	257.3	342.1	426.8	6287	6055	5364	5596	4743	5838	3
Os01g0649900	52	78.2	67.9	82.1	101.2	96.89	10.2	24	13.1	89.67	62.49	43.28	29.39	42.939	52.27	23.88	30.92	30.05	41.8	56.44	44.84	34.01	49.88	44.06	38.03	46.2	72.37	47.2	55.5	33.3	2
Os01g0618900	78.5	78.2	78.7	111.7	121.1	137.5	276	165	242	65.53	49.34	79.97	30.479	78.41	39.598	23.88	51.53	55.95	133.8	91.92	125.9	108.7	80.53	179.8	35.92	54.68	61.4	46.3	64.1	34.2	18
Os01g0195000	90.7	66.1	74.4	39.41	43.39	41.52	63.4	35.4	62.9	63.8	74.55	98.79	30.479	49.473	49.894	83.59	63.89	40.41	61.31	30.64	39.66	66.54	54.16	71.5	42.25	68.83	114	55.5	54.5	54.2	11
Os01g0115566	3.06	2.79	2.16	4.926	7.232	1.73	0.78	7.59	1.38	5.173	2.193	0.941	2.177	0.9335	1.5839	0	0	1.036	0.929	2.419	0	2.218	5.701	3.611	2.113	0	0	0.93	1.91	2.5	2
Os01g0975800	0	0	0	0	0	0	0	0	0	0	0	0	0	0	0	0	0	2.072	0	0	0	0	0	0	0	0	0	0	0	0	1
Os01g0749200	2921	3398	3170	5791	6155	5316	7202	6536	6807	3267	4044	4761	3251.4	2832.1	2306.2	3487	2138	2817	6740	6926	7172	4181	3590	5917	2567	2350	2491	1564	2043	1781	12
Os01g0705200	3.06	0.93	2.16	0	0.904	1.73	1.57	16.4	3.46	0	0	0	3.2656	0	0	0	6.183	1.036	3.716	1.613	0	0	2.851	0	0	0	0	0	0	0	18
Os01g0846400	62.2	67.1	55	14.78	12.66	23.36	29.7	15.2	32.5	34.49	30.7	35.75	44.629	29.87	34.055	346.3	111.3	117.1	12.08	27.41	15.52	21.44	14.25	13.61	72.89	71.66	61.4	95.3	90.9	85.8	13
Os01g0859500	4.08	7.45	6.47	2.463	3.616	11.25	7.05	6.96	2.76	6.035	6.578	3.763	3.2656	1.8669	6.3357	0	4.122	2.072	1.858	4.031	1.724	8.133	5.701	5.778	6.338	4.714	8.772	0.93	4.78	5.83	11
Os01g0528000	9.18	6.52	11.9	22.17	31.64	15.57	7.05	17.1	7.6	6.897	5.482	4.704	17.416	6.5342	11.879	23.88	6.183	12.43	10.22	10.48	13.8	14.79	15.68	8.667	9.507	8.486	4.386	17.6	8.61	16.7	2
Os01g0654450	0	2.79	0	4.926	3.616	6.055	0.78	0	0.69	0.862	1.096	0	0	0	0	0	0	0	0.929	0.806	6.898	0	0	0	0	0	0	0.93	0.96	0	10
Os01g0301000	76.5	83.8	69	252	270.3	222.3	141	147	131	111.2	81.13	79.03	99.055	81.21	76.821	203	83.47	93.24	198.8	188.7	206.1	127.9	121.9	112.7	67.61	95.23	87.72	97.1	109	80	10
Os01g0109366	6.12	6.52	5.39	1.642	2.712	0.865	3.13	5.69	8.29	6.035	0	10.35	4.3541	1.8669	0	11.94	5.153	2.072	3.716	0	0.862	2.958	2.138	8.667	0	0.943	0	1.85	0	0.83	6
Os01g0706100	52	42.8	38.8	115.8	155.5	109	68.1	94.2	80.2	24.14	19.74	27.28	26.124	30.804	28.511	71.65	28.85	17.61	95.68	116.1	137.1	92.42	89.79	57.78	26.41	26.4	28.51	35.2	49.7	22.5	10
Os01g0925400	89.7	79.2	89.4	52.54	53.33	81.31	75.9	64.5	63.6	85.36	78.94	175	42.452	112.01	67.317	107.5	106.1	80.81	78.96	86.27	81.05	63.59	70.55	72.22	79.23	90.51	136	53.7	79.4	75.8	20
Os01g0232100	0	0	1.08	0	0	0	0	0	1.38	0	0	0	1.0885	0	0.792	0	0	0	0.929	0	0	1.479	0	0.722	0	0	0	0	0	0	9
Os01g0612500	23.4	6.52	20.5	29.56	22.6	41.52	11	14.5	10.4	24.14	15.35	8.467	18.505	14.002	23.759	107.5	34.01	33.15	40.87	28.22	19.83	17.75	19.95	9.389	41.2	33.94	57.02	69.4	98.5	33.3	13
Os01g0225600	0	0	0	0	0	0	0	0	0	0	1.096	0	0	0	0	0	0	0	0	0	0	0	0	0	0	0	0	0	0	0	7
Os01g0289600	71.4	129	129	214.3	287.5	104.7	176	491	146	233.7	145.8	62.09	154.57	102.68	262.14	203	94.81	88.06	136.6	115.3	55.18	144.2	346.3	145.2	185.9	62.23	78.95	154	95.6	185	18
Os01g0788900	55.1	64.3	53.9	79.63	77.74	83.91	42.3	51.2	38.7	68.11	50.43	72.44	52.249	43.872	53.854	35.82	66.98	66.31	78.03	82.24	62.94	48.06	65.56	54.17	54.93	76.37	52.63	75.9	48.8	61.7	16
Os01g0676200	540	472	564	1394	1314	1290	1366	1285	1481	644.9	684.4	884.4	790.27	701.96	630.4	1051	590.5	549.1	1711	1717	1962	913.1	768.2	1122	644.4	609.1	526.3	501	504	534	12
Os01g0645200	3932	4646	4604	1479	1489	1311	1804	1968	1705	4580	4746	3419	4811.3	4720.5	4860.3	9768	3913	3582	1357	1359	1238	1129	1466	1383	4204	4141	3844	3866	4264	4528	13
Os01g0853800	581	593	605	782.4	782.8	698.1	485	417	434	929.4	851.9	796.9	673.79	663.68	703.27	812	599.8	615.4	929	963.5	907.1	317.9	377	387.1	783.8	721.3	739	509	589	575	16
Os01g0379101	16.3	4.66	10.8	50.08	44.29	38.06	11.7	10.8	9.67	7.76	5.482	6.586	8.7082	5.6007	5.5438	23.88	3.092	0	45.52	41.12	48.28	8.133	10.69	10.83	8.451	4.714	0	12	3.83	3.33	10
Os01g0654300	312	222	235	213.5	229.6	211.1	385	317	354	284.5	304.8	307.6	283.02	316.44	272.44	358.2	337	253.8	281.5	287	275	294.3	227.3	275.9	315.9	324.3	337.7	297	331	266	5
Os01g0144700	64.2	48.4	58.2	105.9	98.53	114.2	118	99.3	112	50.87	78.94	54.57	76.196	91.478	53.854	143.3	76.26	82.88	110.5	125	139.7	83.55	99.06	79.45	46.48	63.17	50.44	60.1	67.9	55	12

Os01g0180300	270	318	288	1842	2117	1817	3753	4251	4080	310.4	348.7	477.9	284.1	239.9	228.09	203	226.7	187.5	1372	1485	1715	2468	2230	3216	291.6	268.7	344.3	137	176	187	18
Os01g0132766	60.2	33.5	55	62.39	73.22	84.77	75.9	85.4	81.5	43.11	72.36	49.86	22.859	35.471	28.511	71.65	39.16	49.73	84.53	115.3	69.84	96.12	61.29	68.61	34.86	32.06	24.12	34.2	50.7	23.3	12
Os01g0617500	1433	1639	1578	1609	1635	1600	1094	1204	1095	1336	1434	1418	1632.8	1495.4	1496.8	2699	1627	1622	1340	1516	1343	1386	1278	1177	1390	1564	1366	1551	1505	1638	13
Os01g0121900	0	0.93	0	0	0	0	0	0	0	0	0	0	0	0	0	0	0	0	0.929	0	0	0	0	0	0	0	0	0	0	0	14
Os01g0507500	623	706	593	513.9	587.6	565.7	1051	929	924	612.2	596.4	692.4	623.72	701.02	675.55	1087	795.6	649.6	549.9	561.2	516.5	909.4	828.8	826.2	645.4	726	815.8	637	582	717	5
Os01g0882500	1196	1262	1348	1676	1886	1663	6184	5954	6345	1964	2061	2287	1701.4	1407.6	1358.2	1433	1535	1371	2318	1981	2587	3093	3056	4290	1577	1608	1408	1008	1181	1409	18
Os01g0884200	0	0	0	0	0	0	0	0	0.69	0	0	0	2.177	0	0	0	1.031	1.036	0	0	0.862	0	0	0	0	0	0	0.93	0	0.83	4
Os01g0154500	7.14	23.3	4.31	9.852	8.136	6.055	3.91	6.33	6.22	8.622	1.096	8.467	2.177	15.869	7.1277	59.7	16.49	7.252	10.22	4.031	12.93	2.958	7.839	5.056	7.395	19.8	6.579	10.2	6.69	11.7	6
Os01g0604900	1.02	0.93	0	0.821	1.808	0	0	0	0	2.587	0	0	1.0885	0	0	0	0	0	0	1.613	0.862	0	1.425	0	0	0	0	0	1.91	0	7
Os01g0147200	1581	1760	1704	1406	1455	1389	1275	1230	1280	1758	1795	1551	1866.8	1928.5	1897.6	2376	1651	1687	1467	1436	1457	1321	1376	1318	1883	1733	1829	1744	1541	1741	13
Os01g0839300	201	189	176	290.6	271.2	339.1	157	175	240	154.3	162.3	203.2	185.05	193.22	158.39	358.2	168	152.3	406	258	291.4	261	223.8	204.4	153.2	196.1	241.2	140	173	114	10
Os01g0221100	16.3	18.6	6.47	110.8	112.1	95.16	50.9	55	60.1	23.28	20.83	17.88	16.328	10.268	15.047	0	10.31	9.324	17.65	23.38	18.97	412.6	378.4	190.7	24.3	11.31	2.193	20.4	20.1	24.2	17
Os01g0286900	3.06	1.86	3.23	4.926	1.808	2.595	1.57	7.59	12.4	3.449	2.193	0.941	3.2656	5.6007	4.7518	0	1.031	2.072	2.787	1.613	8.622	40.67	16.39	28.17	2.113	0.943	17.54	4.63	3.83	5.83	9
Os01g0148700	536	515	457	531.2	635.5	550.2	512	489	515	582	586.6	675.5	517.05	606.74	473.6	668.7	411.2	473.5	638.2	542.6	682	374.1	449	439.8	418.3	505.4	541.7	366	422	384	16
Os01g0229100	435	425	398	514.8	524.3	534.6	618	588	762	403.5	453.9	453.5	448.47	468.59	437.96	895.6	377.2	388.5	422.7	433.8	482.8	637.4	614.3	561.9	460.6	452.6	399.1	345	318	321	6
Os01g0287400	35.7	28.9	45.3	34.48	29.83	21.63	50.9	43.6	31.8	32.76	17.54	46.1	33.744	28.004	38.806	59.7	31.95	32.12	48.31	24.99	31.04	25.14	54.16	51.28	22.18	22.63	35.09	32.4	25.8	20	6
Os01g0363300	247	186	178	59.11	94.01	102.1	288	334	340	278.5	287.3	460.1	216.62	250.17	236.8	155.2	179.3	205.1	51.09	44.35	47.42	191.5	166	215.2	212.3	281	335.5	209	216	163	5
Os01g0306400	0	1.86	1.08	0.821	0.904	0.865	0.78	0	4.15	0	0	0.941	5.4426	0	1.5839	0	0	1.036	6.503	4.838	12.07	2.218	0	0	0	0	0	0.93	0	0	16
Os01g0158100	155	179	206	175.7	181.7	192.9	216	238	242	309.5	312.5	309.5	238.39	212.83	209.08	274.6	180.3	190.6	236	237.9	216.4	143.4	145.4	187.1	232.4	182	149.1	134	138	177	7
Os01g0878000	17.3	27.9	38.8	149.4	177.2	136.7	0	1.9	2.76	19.83	32.89	31.05	32.656	36.405	26.927	47.76	19.58	23.83	143.1	95.95	191.4	0.739	0	0.722	14.79	25.46	26.32	20.4	30.6	20.8	10
Os01g0971700	476	550	534	1162	1104	1203	1213	1350	1365	701.8	758.7	752.7	927.42	825.17	884.63	1063	642	622.7	1486	1493	1405	1106	1209	1196	864.1	759	758.7	750	795	750	12
Os01g0199400	493	515	477	83.74	99.44	122	217	289	261	344	438.6	367.9	545.35	469.53	521.91	1111	612.1	553.2	101.3	114.5	112.1	258.8	347.1	261.5	676.1	504.4	530.7	662	618	717	13
Os01g0646400	57.1	30.7	35.6	78.81	76.84	69.2	72.8	63.3	44.9	40.52	29.6	34.81	32.656	56.007	36.43	59.7	32.98	51.8	85.46	62.08	94.84	45.84	42.76	44.78	31.69	31.11	41.67	17.6	25.8	20	10
Os01g0276600	14.3	12.1	4.31	33.66	34.35	35.47	56.4	33.5	23.5	25	25.22	32.93	11.974	34.538	15.839	71.65	18.55	17.61	22.29	26.61	35.35	24.4	35.63	38.28	20.07	32.06	26.32	28.7	19.1	15.8	6
Os01g0118066	81.6	121	139	107.5	134.7	114.2	24.3	22.8	19.3	41.38	67.98	19.76	74.019	61.608	83.156	143.3	93.78	104.6	47.38	45.96	33.63	66.54	76.96	41.17	54.93	51.86	37.28	138	103	137	8
Os01g0744000	24.5	19.6	26.9	58.29	55.14	100.3	18	8.22	11.7	13.79	16.45	17.88	9.7967	18.669	12.671	23.88	36.07	19.68	86.39	45.15	70.7	17.01	23.52	38.28	16.9	25.46	48.24	23.1	27.7	19.2	10
Os01g0864500	1662	2231	1332	158.4	77.74	89.1	79.1	139	87.8	131.1	88.81	56.45	121.91	79.343	120.38	883.6	1663	1055	87.32	93.53	175.9	159.7	233	627.6	43.31	42.43	67.98	81.4	87	105	14
Os01g0622800	0	0.93	1.08	0	0	0	3.13	1.27	2.07	1.724	0	0	0	0	0.792	0	1.031	0	0	1.613	0	1.479	0	0.722	1.056	1.886	0	1.85	0	1.67	5
Os01g0610700	192	197	181	169.9	181.7	125.4	187	216	214	195.7	210.5	180.6	194.85	172.69	196.41	334.3	189.6	187.5	171.9	169.3	162.1	219.6	189.6	190.7	198.6	211.2	151.3	197	231	202	6
Os01g0285300	24.5	19.6	10.8	16.42	10.85	45.85	18	17.7	7.6	8.622	8.771	10.35	4.3541	15.869	6.3357	11.94	18.55	10.36	19.51	25.8	32.76	22.18	41.33	53.45	9.507	18.86	63.59	13.9	24.9	7.5	11
Os01g0627800	505	224	315	168.3	124.7	159.2	10.2	10.8	5.53	181.9	183.1	187.2	591.07	570.34	426.08	1433	619.3	364.7	346.5	410.4	686.3	5.915	24.23	18.78	273.6	379	247.8	425	646	472	13
Os01g0572700	125	129	110	149.4	144.6	111.6	240	206	224	117.3	160.1	93.14	114.29	87.744	94.244	131.4	108.2	132.6	127.3	123.4	122.4	144.9	163.2	187.8	118.3	106.5	127.2	96.2	92.8	114	18
Os01g0888300	97.9	68.9	79.7	71.42	84.97	76.99	65.8	50	65	106.9	71.27	62.09	121.91	70.009	84.74	179.1	82.44	102.6	54.81	52.41	47.42	53.24	52.02	49.83	61.27	74.48	70.17	64.8	84.2	80	13
Os01g0355600	1023	1134	1054	1047	1033	976.6	893	938	957	1037	1044	877.8	1062.4	1150	1095.3	1600	1059	1040	820.3	874	933.8	1090	1113	961.3	1113	941	971.5	1087	975	1132	13
Os01g0690600	169	148	238	83.74	79.55	65.74	220	224	153	220.7	188.6	79.03	153.48	140.02	135.43	310.5	74.2	116	71.53	84.66	43.97	178.9	255.1	133.6	115.1	70.71	26.32	143	111	110	6
Os01g0151600	428	408	483	178.2	216	229.2	225	262	236	308.7	320.1	403.6	383.16	378.98	393.61	823.9	472	490	226.7	285.4	236.2	261.7	249.4	248.5	398.2	464.8	469.3	449	404	432	13
Os01g0896800	1285	1159	1053	1545	1559	1431	1394	1226	1424	1321	992.2	1844	1235.5	1506.6	973.33	1505	1037	1141	1314	1279	1560	1264	1355	1153	1154	1461	1583	906	853	861	15
Os01g0660700	0	0	0	0	0	0	0	0	1.38	0	0	0	0	0	1.5839	0	0	1.036	0	0	0	0	0	0	0	0	2.193	0	0.96	0	11
Os01g0915300	875	603	855	548.4	482.7	486.2	374	482	460	757	724.7	847.7	839.25	858.78	918.68	1457	819.3	874.4	563.9	605.5	540.6	550.8	481	352.5	814.5	853.3	706.1	689	783	686	13
Os01g0777200	152	108	105	126.4	150.1	173.9	300	299	324	88.81	73.46	93.14	70.754	84.011	81.572	95.53	141.2	116	147.7	129	142.3	265.4	257.3	271.6	94.02	96.17	131.6	95.3	87	83.3	18
Os01g0771350	286	186	264	284.9	235.9	256.1	348	281	280	350.9	281.8	379.2	313.49	265.1	282.73	214.9	281.3	265.2	406	441	373.3	224	230.9	234	294.7	288.5	274.1	287	269	259	15
Os01g0829600	0	0.93	0	0	0	0	0.78	0	0	0.862	0	0.941	0	0	1.5839	0	0	4.144	3.716	0	0	0	0	0	2.113	0.943	0	2.78	0.96	0.83	1
Os01g0804200	155	150	206	234	324.5	123.7	186	251	165	149.2	123.9	97.85	167.63	98.946	148.89	191.1	126.8	157.5	196	216.1	102.6	135.3	271.5	100.4	105.6	59.4	57.02	120	133	177	2
Os01g0648500	209	175	194	290.6	281.1	243.9	219	251	236	221.6	270.8	201.3	232.94	231.5	247.89	537.3	210.2	179.2	259.2	344.3	275.9	224.8	243	211.6	226.1	227.2	184.2	260	239	265	6
Os01g0935700	1530	1391	1505	1052	1074	1035	877	813	694	1218	1142	1502	1190.8	1501	1309.1	2520	1961	1540	1239	1162	1203	766.7	998.4	948.3	1447	1538	1833	1625	1377	1338	13
Os01g0885000	90.7	110	79.7	58.29	56.05	69.2	132	57.6	60.8	174.2	130.5	195.7	93.613	109.21	72.861	35.82	61.83	63.2	170	89.5	140.5	65.07	62	118.4	88.73	113.1	105.3	59.2	76.5	50	20
Os01g0720800	124	56.8	92.7	69.78	82.26	74.39	70.5	59.5	77.4	47.42	59.21	114.8	70.754	88.678	63.357	191.1	124.7	94.28	77.1	101.6	106.1	110.9	106.2	130	102.5	122.6	109.6	64.8	88.9	48.3	6
Os01g0218900	686	714	690	456.5	499.9	453.3	445	395	343	649.2	743.4	593.7	820.74	748.63	747.62	1385	789.4	713.8	602	519.2	590.6	309.1	335.7	346.7	693	678.8	701.7	714	707	705	13
Os01g0810100	1233	1280	1217	2346	2741	2298	498	493	439	1294	1563	1783	1823.3	1559.8	1231.5	2293	1173	1412	2762	2774	2836	376.3	380.5	437.7	1332	1298	1436	1046	1201	1116	16
Os01g0806801	0	0	0	0	0	0	0	0	0	0	0	0	0	0	0	0	0	1.036	0	0	0	0	0	0	0	0	0	0	0	0	1
Os01g0766900	108	91.3	81.9	144.5	140.1	135.8	159	202	117	85.36	121.7	74.32	82.728	58.807	83.948	47.76	91.72	69.41	79.89	91.11	115.5	108	138.3	166.8	91.9	121.6	109.6	76.8	66	61.7	18
Os01g0585200	0	0	2.16	2.463	0.904	0.865	1.57	0.63	7.6	6.897	5.482	0.941	4.3541	2.8004	3.1679	11.94	0	6.216	0	0.806	0	0	4.988	0.722	0	0.943	2.193	4.63	2.87	0.83	6
Os01g0846900	1.02	2.79	0	0	0.904	0.865	0.78	0	0	0	1.096	0.941	0	0.9335	0	0	2.061	3.108	0.929	0.806	0	0	0.713	0	0	0	0	0.93	0.96	1.67	14
Os01g0909000	0	1.86	0	0	0	0.865	0	3.16	2.07	0	0	1.882	0	0	2.3759	0	0	4.144	1.858	0	1.724	0.739	0	0	0	0	0	0	0	0	15
Os01g0784750	5.1	8.38	8.62	8.21	8.136	3.46	9.39	8.86	6.91	6.035	8.771	0.941	5.4426	1.8669	3.1679	23.88	5.153	2.072	3.716	5.644	0.862	5.176	7.839	0.722	3.169	0.943	0	12	1.91	1.67	6
Os01g0897000	210	229	242	260.2	272.1	277.7	289	214	234	217.3	209.4	217.3	179.61	206.29	194.82	322.4	186.5	180.3	246.2	256.4	289.7	268.4	233.7	294.7	173.2	218.7	208.3	189	168	157	12
Os01g0776700	2410	2942	1849	1081	1009	1020	1383	1567	1727	1140	1225	1343	1330.2	1208.8	1309.1	3176	2305	2151	1350	1623	1699	1137	1172	1693	1329	1307	1217	1122	1257	1071	14
Os01g0910200	179	183	196	263.5	233.2	197.2	188	210	196	144.8	119.5	159	201.38	180.16	167.1	441.8	225.7	236.2	247.1	266.9	322.5	227	191.7	195	184.9	177.3	157.9	189	233	202	6
Os01g0191200	1560	2344	1764	8403	9083	8508	####	####	####	3817	3803	4670	1778.6	1626.1	1647.3	2173	1582	2060	8474	8013	9995	5741	5622	9918	1597	2449	2871	671	937	1061	12
Os01g0876300	1.02	0	1.08	0	0	0.865	0	0	0	0	0	0	0	0	0	0	0	0	0	0	0	0	0	0	0	0	0	0	0	0	19
Os01g0973400	1175	1406	1434	2685	3247	2733	4139	3920	3781	1396	1873	1760	1621.9	1518.7	1291.7	2293	1214	1406	2658	2903	2990	2800	2498	3287	1474	1616	1335	1127	1280	1207	12
Os01g0142100	3087	3243	3711	3504	3926	3737	2548	2739	2667	3680	3904	4055	4756.8	4135.2	4108.7	7463	3284	3562	3056	3528	3570	2655	2253	2435	3784	3392	2925	3246	3673	3949	13
Os01g0935400	174	137	172	100.2	132	75.26	23.5	29.7	25.6	161.2	150.2	225.8	117.56	200.69	140.18	214.9	219.5	150.2	164.4	105.6	135.4	22.92	21.38	39	99.3	203.7	269.7	207	166	135	3
Os01g0186200	761	689	821	737.2	640.9	832.2	135	194	187	819.1	912.2	1116	1201.7	1010.9	1052.5	1421	975.9	995.6	887.2	947.4	1015	272.1	234.5	189.2	906.4	1017	791.6	849	885	926	13
Os01g0339600	32.6	45.6	46.3	22.17	31.64	25.95	25.1	31.6	33.9	31.04	36.18	30.11	43.541	46.673	41.182	107.5	69.05	36.26	48.31	41.12	23.28	68.02	63.42	62.84	46.48	66	48.24	58.3	70.8	55	13
Os01g0512100	734	815	848	646.1	629.2	628.9	985	922	855	797.5	862.9	900.4	880.61	954.92	887.79	1767	1032	923.1	833.3	736.9	793.2	791.1	751.1	806.7	834.5	861.8	927.6	1021	929	904	13
Os01g0350900	835	944	1008	699.5	645.4	641	1206	1151	1065	1296	1395	1361	1372.6	1197.6	1412.1	2293	969.7	1012	776.6	776.4	666.5	872.5	892.9	1019	1370	1294	1072	1139	1110	1143	13
Os01g0726700	0	1.86	0	0	0	0	0	0	0	0.862	1.096	0	0	0	0	0	0	0	0	0	0	0	0	0	1.056	0	0	0	0	0	7
Os01g0917900	1.02	0	0	0	0	0	0	0	0	0	0	0	0	0	0	0	0	0	0	0	0	0	0	0	0	2.829	0	0	0.96	0	1
Os01g0144800	15.3	10.2	10.8	4.105	7.232	6.92	10.2	6.96	15.2	15.52	7.675	3.763	11.974	17.736	19.799	0	2.061	8.288	7.432	8.869	1.724	14.05	9.264	11.56	9.507	6.6	15.35	17.6	17.2	13.3	4
Os01g0818000	2260	2489	2360	866.9	873.2	648.8	912	894	837	2243	2739	2049	1839.6	1728.8	2307.8	3057	2053	2059	749.7	789.3	648.4	868.8	899.3	962	1990	1887	1432	1984	1916	2063	3
Os01g0599550	561	515	689	767.6	960	760.4	579	765	587	343.2	446.2	314.2	612.84	653.42	527.45	1445	596.7	671.4	616.8	866.8	629.4	684.7	674.2	520.7	510.2	333.8	291.7	678	681	807	6
Os01g0605000	29.6	35.4	23.7	32.84	36.16	21.63	49.3	38	38.7	43.97	49.34	26.34	55.515	44.806	45.934	59.7	29.89	26.94	49.23	53.21	52.6	22.92	22.8	27.45	50.71	39.6	48.24	37	28.7	38.3	16
Os01g0946800	0	0	0	0	0	1.73	0	0	0	0	0	0	0	0	0	0	0	0	0	0	0	0	0	0	0	0	0	0	0	0	2
Os01g0748800	401	438	303	50.08	63.28	162.6	237	94.9	209	369.9	383.7	390.4	211.17	245.5	266.89	203	413.2	441.4	110.5	87.08	114.7	210	83.38	156.7	391.9	380.9	451.7	250	222	374	3
Os01g0717400	300	430	284	1080	1195	1379	140	102	74.6	239.7	365.1	288.8	230.77	349.11	213.83	406	528.7	362.6	1691	1938	2332	264	255.8	325	459.5	751.4	741.2	489	500	453	16
Os01g0638701	0	0	0	0	0	0	0	0	0	0	0	1.882	0	0	0	0	0	0	0	0	0	0.739	0	0	0	0	0	0	0	0	20
Os01g0704000	0	0	0	0	0	0	0	0	0.69	0	0	0	0	0	0.792	0	0	0	0	0	0	0	0	0	0	0	0	0	0	0	4
Os01g0246100	947	1028	1037	969.6	962.7	948.1	666	650	618	682.9	837.6	660.5	965.52	848.51	888.59	1851	1004	1003	714.4	807.9	807.9	908	813.8	868.1	890.5	840.1	723.7	1046	957	1108	13
Os01g0567200	2.04	1.86	1.08	0	0.904	0	0	0	1.38	0.862	0	0	0	0	0	0	0	0	0	0.806	0	0	0	0	1.056	3.771	0	1.85	0	0	19
Os01g0267300	0	0.93	1.08	1.642	0.904	0.865	0.78	2.53	0.69	0	0	0	0	0.9335	0	0	0	0	0	0	4.311	1.479	2.851	1.444	0	1.886	0	0.93	1.91	0.83	17
Os01g0866000	0	0	0	0	0	0.865	0	0	0	0	0	0	0	0	0	0	0	0	0	0	0	0	0	0	0	0	0	0	0	0	2
Os01g0121950	0	0	0	0	0	0	0	0	0	0	0	0	0	0	0	0	0	0	0	0	0	0	0	0	0	0	2.193	0	0	0	11
Os01g0727500	7.14	27	10.8	11.49	4.52	4.325	5.48	6.96	26.3	2.587	2.193	2.822	7.6196	1.8669	5.5438	0	8.244	8.288	2.787	4.031	3.449	45.84	22.09	44.78	1.056	3.771	0	0	1.91	0.83	9
Os01g0971400	0	0	0	0	0	0	0	1.27	0	0	0	0	0	0	0	0	1.031	0	0	0	0.862	0	0	0	0	0	0	0	0	0	15
Os01g0521400	658	726	687	307	311	357.3	362	403	431	728.5	786.1	702.8	768.5	667.42	782.24	1099	780.1	860.9	284.3	346.7	318.2	528.7	467.5	464.4	783.8	835.4	802.6	788	726	827	13
Os01g0964800	121	148	127	170.8	146.4	148.8	51.7	99.9	51.1	101.7	106.4	63.98	136.06	100.81	137.8	214.9	142.2	137.8	65.03	77.4	62.94	178.9	148.2	132.2	140.5	109.4	78.95	147	153	214	8
Os01g0882200	54	46.6	47.4	60.75	81.36	53.63	193	166	148	57.77	70.17	60.21	47.895	55.074	36.43	107.5	45.34	53.87	101.3	74.18	88.81	110.9	79.82	117	31.69	42.43	65.79	44.4	48.8	36.7	18
Os01g0678000	65.2	118	140	104.3	148.3	72.66	89.2	117	47.7	128.5	72.36	35.75	123	86.811	128.3	35.82	51.53	128.5	17.65	24.99	30.78	172.3	158.2	131.4	91.9	37.71	32.89	83.3	81.3	104	17
Os01g0620900	0	2.79	1.08	4.105	0.904	5.19	2.35	6.33	2.07	12.93	0	7.527	5.4426	2.8004	5.5438	11.94	1.031	1.036	5.574	2.419	1.724	0	4.988	2.889	4.225	1.886	0	3.7	3.83	5	6
Os01g0516900	0	0	0	0	0	0	0	0	0	0	0	0	0	0.9335	0	0	0	0	0	0	0	0	0	0	0	0	0	1.85	0	0.83	1
Os01g0752100	455	394	442	376.8	372.4	377.2	291	327	337	484.5	489	424.3	500.72	461.13	517.95	776.2	474	405.1	470.1	529.7	469.9	324.6	300.7	309.1	465.9	455.4	453.9	436	433	442	13
Os01g0355400	0	0	0	0	0	0	0	0	0	0	0	0	0	0	0	0	0	0	0	1.613	0	0	0	0	0	0	0	0	0	0	16
Os01g0969100	148	106	87.3	133	119.3	244.8	50.9	52.5	54.6	90.53	73.46	96.9	38.098	102.68	60.981	155.2	140.2	101.5	119.8	105.6	152.6	93.9	103.3	130.7	78.17	80.14	129.4	110	115	65.8	2
Os01g0162400	1.02	5.59	0	9.852	8.136	12.11	3.91	5.06	2.76	1.724	2.193	0	0	1.8669	0.792	0	1.031	0	4.645	15.32	13.8	4.436	4.988	0	1.056	2.829	0	1.85	3.83	2.5	10
Os01g0734200	245	208	208	142.8	120.2	174.7	75.9	67.7	29.7	267.3	265.3	244.6	151.3	253.9	149.68	131.4	143.2	159.5	178.4	170.9	164.7	40.67	75.54	69.34	131	169.7	241.2	117	134	115	20
Os01g0961300	819	786	833	844	743.1	1028	555	661	586	739.8	677.6	763	763.05	840.11	696.14	2197	849.2	996.7	878.8	887.7	971.7	789.7	680.6	713.6	1054	965.5	1044	1055	1025	1071	13
Os01g0927900	613	626	682	884.2	1019	843.4	957	893	920	662.2	723.6	643.5	757.61	742.09	720.69	1254	599.8	720	848.1	867.6	833.8	746.8	731.2	741.7	643.3	642.1	778.5	590	644	607	6
Os01g0228500	5.1	5.59	8.62	4.105	5.424	10.38	16.4	4.43	3.46	11.21	8.771	8.467	4.3541	10.268	9.5036	35.82	14.43	5.18	3.716	5.644	2.587	6.654	2.138	10.11	7.395	13.2	8.772	28.7	5.74	5.83	13
Os01g0660300	173	110	135	22.99	33.45	83.91	89.2	63.9	67.7	75.87	69.07	111	43.541	114.81	98.204	131.4	227.7	136.8	64.1	45.96	57.77	136.8	89.79	168.3	107.7	97.11	133.8	170	101	101	1
Os01g0126600	140	148	128	74.71	91.3	67.47	74.4	101	73.9	215.5	252.2	327.4	131.71	186.69	161.56	441.8	176.2	165.8	102.2	95.14	118.1	83.55	86.94	59.95	202.8	216.9	296	221	195	162	13
Os01g0533500	227	261	245	225.8	231.4	249.1	245	237	209	227.6	264.2	223.9	283.02	246.43	254.22	788.1	228.8	256.9	254.5	265.3	284.5	217.4	230.2	205.8	254.6	233.8	236.8	316	271	292	6
Os01g0660500	0	0	0	0	0	3.46	0	0	0	0	0	0	0	0	0	0	0	0	0	0	0	0	0	0	0	0	0	0	0	0	2
Os01g0853400	1257	1328	1281	855.5	787.4	773.4	1073	1090	1010	1261	1320	1170	1193	1192	1286.2	2137	1143	1154	782.2	761.9	648.4	1156	1307	1156	1337	1296	1335	1377	1235	1308	13
Os01g0588400	3.06	1.86	3.23	6.568	2.712	5.19	13.3	11.4	14.5	4.311	3.289	8.467	2.177	2.8004	3.9598	0	0	1.036	1.858	1.613	0	20.7	13.54	28.17	1.056	1.886	2.193	3.7	0.96	0	9
Os01g0196800	16.3	12.1	12.9	8.21	3.616	6.92	9.39	1.9	7.6	15.52	4.386	10.35	11.974	15.869	8.7116	35.82	10.31	16.58	13.01	10.48	12.07	7.394	12.83	4.333	17.96	25.46	13.16	23.1	26.8	35	13
Os01g0582400	553	562	506	476.2	477.3	423	550	645	560	538.9	530.7	487.3	543.17	561	528.24	740.3	517.3	537.7	422.7	377.3	442.3	527.2	551.6	551.1	525	542.1	574.5	486	444	577	6
Os01g0183850	16.3	14	6.47	1.642	2.712	0	3.13	4.43	4.15	3.449	7.675	1.882	1.0885	0.9335	4.7518	0	6.183	18.65	1.858	7.257	1.724	1.479	6.414	10.83	1.056	1.886	0	3.7	3.83	1.67	14
Os01g0870300	217	249	261	150.2	165.6	202.4	142	133	133	139.7	202.8	115.7	202.46	180.16	266.89	501.5	250.4	251.8	134.7	188.7	148.3	200.4	154.6	154.6	195.4	177.3	127.2	277	319	327	13
Os01g0220200	1947	2119	2953	915.4	1026	724	135	241	252	1682	2213	1211	3441.9	1935	3232	4681	1572	2315	479.3	651.5	332.8	449.5	379.1	295.4	2101	1399	850.9	2041	2161	2645	13
Os01g0519400	437	483	466	454.8	549.6	539.8	573	595	513	432	491.2	472.3	456.09	491	492.6	812	487.4	540.8	586.2	625.7	551.8	519	521.6	445.6	521.8	484.6	578.9	513	456	583	6
Os01g0808900	68.3	75.4	115	46.8	29.83	38.06	79.1	64.5	87.1	45.7	86.62	68.68	57.692	69.075	54.646	59.7	58.74	46.69	39.94	33.06	27.59	19.22	37.06	46.22	108.8	62.23	50.44	39.8	44.9	56.7	5
Os01g0151200	8812	8525	9738	9260	10184	9562	7152	8476	7694	8286	9349	9056	13202	12149	11777	17016	10913	10117	9135	10500	10092	5324	5318	6993	9297	9415	7169	9578	####	9761	13
Os01g0845100	45.9	44.7	32.3	8.21	7.232	12.98	9.39	10.8	10.4	14.66	14.25	16.93	9.7967	3.7338	7.1277	59.7	50.5	34.19	12.08	7.257	7.76	19.96	16.39	31.06	3.169	18.86	10.96	12	14.3	7.5	14
Os01g0923200	2.04	6.52	3.23	2.463	1.808	0.865	5.48	7.59	2.76	1.724	3.289	7.527	0	0	2.3759	0	0	4.144	0	5.644	7.76	8.873	2.138	8.667	1.056	4.714	4.386	1.85	3.83	2.5	9
Os01g0138600	57.1	65.2	67.9	85.38	99.44	89.1	83.8	67.7	53.2	84.49	75.65	32.93	55.515	66.275	79.989	191.1	58.74	66.31	87.32	90.3	103.5	89.47	72.69	33.22	61.27	44.31	43.86	47.2	89.9	57.5	6
Os01g0801800	46.9	33.5	47.4	46.8	49.72	58.82	38.4	35.4	47.7	58.63	50.43	65.86	59.869	41.072	44.35	143.3	35.04	31.08	49.23	49.99	69.84	32.53	45.61	40.45	36.97	45.26	65.79	21.3	41.1	38.3	6

Os01g0284700	579	623	654	835.8	835.3	722.3	505	499	668	805.3	872.7	1064	807.68	759.83	697.72	871.7	416.3	565.7	942.9	954.6	970	334.2	354.2	395.1	524	440.3	469.3	306	424	322	16
Os01g0571166	117	135	94.8	154.3	95.82	190.3	23.5	38.6	15.2	37.94	46.05	49.86	62.046	98.012	64.941	310.5	178.3	135.7	119.8	131.4	167.3	172.3	239.4	191.4	141.6	161.2	155.7	228	120	228	8
Os01g0887400	287	265	261	460.6	519.8	521.6	527	451	486	175.9	234.6	298.2	328.73	281.9	251.05	358.2	230.8	239.3	630.8	677.3	715.6	304.6	277.9	474.5	240.9	223.5	239	202	255	227	10
Os01g0113000	24.5	21.4	23.7	29.56	24.41	30.28	15.7	16.4	13.8	10.35	13.16	19.76	31.567	13.068	7.1277	11.94	10.31	17.61	15.79	19.35	33.63	25.88	13.54	27.45	13.73	22.63	17.54	17.6	18.2	24.2	2
Os01g0267900	623	607	554	811.1	875.9	776.8	1461	1272	1352	656.1	617.3	703.7	533.37	618.88	582.89	1385	562.7	439.3	855.6	867.6	863.9	970.1	1150	1130	573.6	611.9	723.7	530	494	479	18
Os01g0154300	0	0	0	0	0	0	0	0	0	0	0	0	0	0	0	0	0	0	0	0	0	0	0	0	1.056	0	0	0	0	0	1
Os01g0691700	1382	1313	1351	979.4	1077	1039	1225	1093	1137	1342	1479	1402	1517.4	1679.3	1427.9	2340	1752	1508	1298	993.3	1232	889.5	980.6	1020	1361	1561	1675	1453	1393	1400	13
Os01g0755100	537	550	508	793.1	810	781.1	615	700	621	438	486.8	413	365.74	407.92	516.36	871.7	469.9	451.7	687.4	631.3	687.2	697.2	682	606	524	531.8	320.2	551	484	497	12
Os01g0281400	1135	1169	1081	1282	1338	1349	1358	1273	1414	1171	992.2	931.4	951.37	1016.5	1141.2	1731	1120	931.4	1026	1046	1058	1457	1446	1405	1004	920.2	1033	1059	1008	1057	6
Os01g0973500	13.3	13	15.1	2.463	4.52	6.055	5.48	5.69	9.67	9.484	5.482	17.88	4.3541	8.4011	6.3357	0	14.43	7.252	2.787	8.869	10.35	5.176	4.988	7.222	11.62	13.2	8.772	6.48	6.69	13.3	20
Os01g0897800	2298	2330	2323	2240	2292	2314	2060	2025	1907	2072	1966	1974	2648.4	2339.2	2622.2	4932	2547	2460	2066	1938	2241	2018	2127	2016	2263	2173	2112	2457	2320	2575	6
Os01g0960900	22.4	8.38	15.1	68.96	101.2	39.79	7.83	41.7	22.1	12.93	8.771	3.763	17.416	6.5342	17.423	95.53	14.43	33.15	45.52	70.15	54.32	22.18	47.75	13	25.35	4.714	6.579	19.4	39.2	20.8	10
Os01g0169800	139	92.2	88.4	98.52	98.53	113.3	36	48.1	58.7	94.84	104.2	159	101.23	168.02	131.47	286.6	128.8	107.7	114.3	88.69	117.3	166.4	116.2	139.4	129.9	193.3	234.6	140	179	163	13
Os01g0833200	2350	2038	2201	464.7	398.7	439.4	1188	1273	1185	2412	2554	2613	3074	2529.7	2729.9	3451	2445	2383	702.3	739.4	577.7	680.2	854.4	967.1	2323	2488	2167	1991	2146	2305	3
Os01g0713800	0	0	0	3.284	0.904	2.595	0	0.63	0.69	0.862	0	0.941	0	0	0	0	0	0	0.929	1.613	0	1.479	0.713	0.722	1.056	0	0	0	0	0	2
Os01g0133700	0	0	0	0	0	0	0	0	0	0	0	0	0	0	0	0	0	0	0	0	0	0	0.713	0	0	0	0	0	0	0	17
Os01g0653500	1.02	0	0	0	0	0	0	0	0	0	0	0	0	0	0	0	0	0	0	0	0	0	0	0	0	0	0	0	0	0	19
Os01g0570601	0	0	0	0	0	0	0	0	0	0	0	0	0	0	0	0	0	0	0	0	0	0	0	0	0	0	0	0	0	3.33	1
Os01g0323500	4.08	9.31	5.39	0	2.712	5.19	0.78	3.16	2.07	5.173	3.289	0	5.4426	8.4011	1.5839	0	3.092	1.036	1.858	5.644	6.036	1.479	0	0.722	2.113	1.886	2.193	1.85	0.96	4.17	4
Os01g0136000	2.04	1.86	0	1.642	0.904	0	0	0	0	0	1.096	0.941	0	4.6673	2.3759	0	0	1.036	1.858	0	1.724	0	0	0	1.056	0	0	2.78	0	0	4
Os01g0600900	####	####	####	30334	24414	21473	5137	3078	4452	91390	98692	#####	52030	50291	56225	66129	47485	61882	26589	21628	35566	573	354.2	450.7	44769	48755	48444	####	####	####	3
Os01g0550000	1708	1728	1510	1884	2073	1977	1832	1713	1693	1309	1481	1471	1753.6	1764.2	1535.6	3618	1686	1800	1981	2062	1813	1630	1671	1512	1590	1708	1735	1495	1566	1584	6
Os01g0622033	505	453	462	491.8	426.7	571.8	23.5	60.1	29	120.7	159	136.4	161.1	224.96	217	895.6	772.9	493.2	318.6	438.6	446.6	154.5	174.6	174.8	270.4	260.2	263.2	537	393	494	8
Os01g0844700	0	0	0	0	0	0	0	0	0	0	0	0	0	0	0	0	0	0	0	0	0	0	0.713	0	0	0	0	0	0.96	0	17
Os01g0158800	0	0	0	0	0	0	0	0	0	0.862	0	0	0	0	0	0	0	0	0	0	0	0	0	1.444	0	0	0	0	0	0	9
Os01g0809450	0	0.93	1.08	0	0	0	0	0.63	0	0	0	0	0	0	0	0	0	0	0	1.613	1.724	0.739	0.713	0	2.113	0	0	0	0.96	0.83	16
Os01g0859600	479	604	491	431.8	464.6	437.7	528	485	530	507.8	475.8	433.7	569.3	451.79	565.46	609	517.3	533.6	366.9	385.4	386.3	464.3	508.8	529.4	446.8	454.4	377.2	508	481	568	13
Os01g0938400	1.02	10.2	4.31	0.821	4.52	1.73	6.26	6.33	6.91	6.897	9.868	13.17	4.3541	2.8004	3.1679	11.94	2.061	3.108	2.787	0	0	8.133	2.138	5.778	7.395	5.657	8.772	11.1	7.65	5	5
Os01g0842801	0	0	1.08	0	1.808	4.325	1.57	0	0	0.862	0	0	0	0	0	0	0	0	0	0	0	8.133	10.69	5.778	3.169	0.943	4.386	4.63	1.91	2.5	17
Os01g0255100	4.08	11.2	6.47	49.26	38.87	44.98	7.05	11.4	5.53	7.76	4.386	10.35	9.7967	0.9335	3.9598	59.7	3.092	8.288	43.66	35.48	50.87	12.57	10.69	5.778	6.338	6.6	13.16	3.7	12.4	8.33	10
Os01g0309900	661	636	629	652.7	642.7	750.9	1415	1474	1439	706.1	712.7	690.6	710.8	687.95	794.34	1624	610.1	680.7	650.3	705.5	801.9	1190	1209	1146	713	707.1	800.4	663	591	647	18
Os01g0259200	1242	1383	1361	1754	1770	1744	2241	2241	2051	1799	1702	1787	1625.2	1663.4	1656	2555	1377	1490	1837	1952	1730	2113	2122	2044	1896	1592	1715	1878	1763	1958	6
Os01g0590900	600	628	675	499.2	472.8	509.5	418	524	470	561.3	679.8	620.9	649.85	686.09	721.48	1385	772.9	715.9	485.8	497.5	540.6	576.7	509.5	478.1	663.4	718.4	607.4	836	748	798	13
Os01g0376600	1.02	0	0	0	0	0	0	0	0.69	1.724	0	0	1.0885	0.9335	0	0	0	0	0	0	0	1.479	0	2.167	0	0.943	0	0	0	0	9
Os01g0231600	6.12	2.79	1.08	3.284	3.616	3.46	1.57	1.27	5.53	3.449	5.482	9.408	4.3541	5.6007	2.3759	11.94	7.214	5.18	2.787	4.031	1.724	1.479	2.138	5.056	13.73	6.6	6.579	3.7	4.78	5	13
Os01g0869750	29.6	4.66	21.6	14.78	14.46	12.98	3.13	10.1	4.15	28.45	23.02	28.22	34.833	23.336	14.255	0	16.49	51.8	39.94	32.25	52.6	10.35	5.701	5.056	40.14	33.94	26.32	17.6	24.9	30	16
Os01g0627701	1.02	0	0	0	0	0	0	0	0	0.862	1.096	0	0	0	0	0	0	0	1.858	0	0	0	0	0	1.056	0	0	0	0	0	7
Os01g0702450	1.02	5.59	1.08	1.642	6.328	7.785	0.78	3.16	1.38	2.587	2.193	12.23	3.2656	0.9335	5.5438	0	3.092	2.072	7.432	7.257	2.587	3.697	3.563	5.778	3.169	0	4.386	2.78	1.91	6.66	16
Os01g0871800	0	0	0	392.4	381.5	306.2	24.3	24	18	0	0	0	0	0	0	0	0	0	78.96	117.7	102.6	81.33	96.21	17.33	0	0	0	0	0.96	0.83	2
Os01g0267100	687	715	725	904.7	826.2	820.1	735	757	804	757	758.7	636	881.7	840.11	811.77	1624	691.5	742.8	671.6	778.9	720	848.1	821	808.2	750	712.8	638.1	755	781	836	6
Os01g0679000	341	303	338	523	549.6	525.1	390	386	379	294.9	208.3	276.6	332	329.51	325.5	501.5	284.4	329.5	482.1	502.3	612.2	374.9	389.1	337.3	276.8	309.3	265.3	306	310	307	10
Os01g0778800	272	293	306	539.4	791.9	490.5	1119	1444	1305	460.4	292.7	314.2	174.16	175.49	237.59	465.7	169	218.6	421.7	497.5	250	1043	1313	1018	249.3	187.6	221.5	281	245	230	18
Os01g0961600	488	509	567	520.5	501.7	470.6	467	546	479	524.2	542.7	580.5	512.69	484.46	635.95	1290	491.6	614.4	471.9	531.3	500.1	638.8	629.3	538.8	616.9	623.2	559.2	702	714	615	6
Os01g0627500	587	465	497	444.1	376.1	406.6	213	211	205	509.6	510.9	557.9	625.9	712.22	620.9	800	636.9	525.3	802.6	876.4	726	144.9	198.8	163.2	531.4	499.7	614	677	619	563	16
Os01g0911800	906	988	889	799.6	739.4	836.5	632	626	764	775.1	825.6	642.6	950.28	837.31	939.27	1588	987.2	898.2	663.3	685.3	713.9	1003	868	892.7	947.6	877.8	866.2	1076	980	1116	13
Os01g0170900	6.12	3.73	1.08	1.642	0	4.325	3.91	3.8	5.53	0	0	1.882	0	3.7338	2.3759	11.94	5.153	2.072	2.787	4.838	0	4.436	2.138	1.444	1.056	3.771	6.579	0	1.91	3.33	6
Os01g0949750	691	774	983	682.2	744.9	401.4	370	511	343	531.1	486.8	509	644.4	697.29	585.26	1206	664.7	655.8	680	556.3	522.5	138.3	280.8	152.4	528.2	388.5	366.2	562	421	527	13
Os01g0931600	2.04	1.86	1.08	0	0	0	0	0	0	0.862	3.289	0	0	0.9335	0	0	0	3.108	0	0	0	0	0	0	3.169	2.829	0	0	1.91	0.83	3
Os01g0823400	37.7	23.3	26.9	19.7	22.6	38.93	7.83	17.7	6.91	26.73	21.93	38.57	25.036	42.939	20.591	71.65	56.68	39.37	30.66	17.74	25.87	30.31	29.22	28.17	31.69	46.2	28.51	50.9	33.5	43.3	13
Os01g0168300	224	223	196	296.4	294.7	327.9	402	437	470	308.7	305.9	294.5	261.24	268.83	251.05	310.5	245.3	255.9	369.7	340.3	304.4	283.9	265.8	291.1	289.4	229.1	256.6	205	224	215	18
Os01g0182900	632	583	558	515.6	536.1	475.8	580	667	568	697.5	730.2	809.1	796.8	649.68	692.97	1385	659.5	623.7	652.1	736.1	653.6	500.6	490.3	529.4	682.4	702.4	690.8	672	640	637	13
Os01g0861900	119	98.7	123	40.23	50.62	38.06	69.7	69.6	59.4	113.8	120.6	137.4	174.16	154.95	169.48	286.6	89.66	100.5	52.95	34.67	54.32	42.15	43.47	43.33	76.06	64.11	76.75	99	105	74.1	13
Os01g0209400	710	662	711	649.4	592.1	636.7	526	476	494	771.7	763.1	814.7	778.29	918.52	777.71	1015	779.1	730.4	870.4	858.7	756.2	572.3	534.5	556.9	738.4	742	912.3	851	712	776	13
Os01g0118700	140	175	150	60.75	80.45	69.2	194	165	202	68.97	82.23	84.67	79.462	51.34	74.445	107.5	130.9	134.7	74.32	73.37	52.6	292.8	210.9	224.6	103.5	78.26	103.1	126	110	101	9
Os01g0810401	14.3	16.8	32.3	14.78	14.46	17.3	1.57	1.27	6.91	7.76	3.289	0	7.6196	5.6007	15.839	23.88	15.46	16.58	2.787	10.48	4.311	14.05	9.977	3.611	11.62	11.31	2.193	21.3	15.3	25	8
Os01g0143900	20.4	40.1	29.1	58.29	84.07	64.88	55.6	51.2	62.9	41.38	31.8	38.57	18.505	23.336	35.638	11.94	16.49	23.83	50.16	33.86	31.9	168.6	195.3	137.9	28.52	18.86	39.47	21.3	18.2	28.3	17
Os01g0150400	7.14	19.6	10.8	9.031	17.18	7.785	8.61	5.69	11.1	11.21	16.45	8.467	5.4426	16.802	8.7116	0	17.52	11.4	17.65	8.869	17.24	11.09	7.126	10.83	11.62	10.37	24.12	11.1	16.3	6.66	11
Os01g0862600	22.4	12.1	12.9	40.23	49.72	60.55	27.4	31	44.2	20.69	14.25	19.76	15.239	13.068	19.007	23.88	11.34	31.08	39.02	40.31	69.84	34.75	37.06	31.78	19.01	22.63	32.89	17.6	15.3	12.5	10
Os01g0589800	1445	1066	1330	683.9	619.2	774.2	2190	2120	2133	1625	1716	2365	1725.3	1780.1	1712.2	2245	2007	1531	1324	1232	1462	1142	1249	1751	1721	2128	1978	1288	1331	1244	5
Os01g0348900	0	0	0	0	0	0.865	0.78	0	0	0	0	0	0	0	0	0	0	0	0	0.806	0	0	0	0	0	0	0	0	0	0	12
Os01g0619000	517	486	524	520.5	501.7	515.6	380	406	404	494.9	510.9	533.4	607.39	559.14	620.9	895.6	477.1	519.1	400.4	486.2	462.1	520.5	463.9	411	574.7	531.8	493.4	578	624	629	13
Os01g0184900	2077	2148	2022	1866	2013	1993	2194	2234	2387	2083	2173	1956	1922.3	1998.5	2176.3	3678	1910	2025	1737	1729	1664	2209	2358	2185	2072	2222	1989	2114	1819	1903	6
Os01g0135600	17.3	18.6	17.2	39.41	51.53	51.9	106	87.9	99.5	28.45	23.02	26.34	16.328	16.802	14.255	11.94	7.214	7.252	30.66	37.9	69.84	66.54	69.13	76.56	23.24	9.428	10.96	13.9	16.3	20	18
Os01g0548000	0	0	0	0.821	0	0	0	0	0	0	0	0	0	0	0	0	0	0	0	0	0	0	0	0	0	0	0	0.93	0	0	2
Os01g0960400	795	1031	758	1105	1034	911.8	297	388	378	332.8	400.2	346.2	372.27	394.85	393.61	800	632.7	642.3	764.5	903.8	798.4	1585	1517	907.1	556.7	729.8	589.9	840	606	724	17
Os01g0900900	762	606	645	287.3	317.3	339.1	711	823	838	907.9	1002	1229	782.65	890.51	919.47	883.6	887.3	769.8	292.6	230.6	302.6	662.5	533.1	687.6	986.6	1031	1046	789	658	752	3
Os01g0149400	6.12	3.73	1.08	0.821	0	0	1.57	5.06	2.76	3.449	3.289	5.645	0	5.6007	1.5839	0	1.031	5.18	2.787	2.419	3.449	0.739	0.713	1.444	0	2.829	0	0.93	1.91	2.5	19
Os01g0265800	1401	1338	1282	1052	1100	1049	948	1063	1014	1419	1467	1443	1488	1409.5	1402.6	2723	1329	1377	990.3	968.3	1031	997.4	1115	1080	1555	1500	1553	1402	1311	1433	13
Os01g0834800	0	0	2.16	0	0	0.865	0	0	0	0	0	0.941	0	0	0	0	0	0	0	0	0	0	0	0	0	0	0	0	0	0	14
Os01g0694050	3.06	2.79	4.31	0	0.904	4.325	6.26	1.9	0	4.311	4.386	0	3.2656	0.9335	3.1679	0	6.183	3.108	2.787	4.031	2.587	2.218	2.851	0.722	2.113	1.886	6.579	2.78	1.91	2.5	11
Os01g0880400	460	385	469	134.6	127.5	197.2	256	249	296	329.4	296	291.7	276.48	312.71	296.99	1075	517.3	506.6	188.6	243.5	229.3	420	333.5	441.3	347.5	375.3	453.9	461	398	391	13
Os01g0901500	171	159	173	114.1	76.84	112.5	85.3	128	71.9	137.1	176.5	142.1	163.28	175.49	181.36	274.6	244.2	198.9	151.4	118.5	116.4	102	138.3	98.95	156.3	178.2	236.8	177	159	186	13
Os01g0558600	2185	1968	2112	1636	1677	1669	2084	2219	2053	2262	2105	2488	2288.1	2289.8	2344.2	3797	2294	2098	2072	1601	1848	1593	2007	1819	2220	2301	2568	2053	1979	2006	13
Os01g0894300	852	771	738	355.5	366.1	607.3	371	432	435	432	503.2	511.8	496.37	750.5	709.6	1087	1109	890	484.9	376.5	441.5	529.4	540.9	541	852.5	993.8	1007	1114	749	916	13
Os01g0567400	360	307	280	135.5	128.4	105.5	294	237	258	341.4	278.5	287.9	269.95	266.97	247.89	453.8	366.9	308.7	146.8	122.6	108.6	427.4	373.4	423.2	277.8	300.8	322.4	292	249	292	9
Os01g0237000	0	3.73	1.08	7.389	1.808	5.19	0	1.27	0.69	0.862	0	0	0	2.8004	0	0	0	3.108	7.432	3.225	10.35	0.739	0.713	2.167	2.113	1.886	2.193	3.7	0	0	16
Os01g0172600	52	28.9	26.9	14.78	10.85	10.38	2.35	13.9	11.7	27.59	26.31	64.92	22.859	27.07	27.719	23.88	31.95	26.94	10.22	12.09	6.898	8.873	10.69	13	26.41	24.51	52.63	33.3	34.4	30	3
Os01g0946300	3.06	4.66	5.39	4.926	5.424	3.46	5.48	6.33	2.07	3.449	5.482	9.408	3.2656	4.6673	7.9197	35.82	5.153	4.144	1.858	4.031	3.449	4.436	7.126	3.611	5.282	3.771	0	4.63	7.65	1.67	6
Os01g0694400	63.2	38.2	62.5	44.33	78.65	67.47	54	68.9	33.2	67.25	61.4	63.03	74.019	67.209	57.022	71.65	51.53	61.13	58.52	49.18	77.6	53.98	53.45	43.33	62.33	62.23	67.98	66.6	65	75.8	4
Os01g0242200	0	0	1.08	0	0	0	0	0.63	0	0.862	0	0.941	0	0	0	0	0	0	0.929	0	0	0.739	0.713	1.444	0	0	0	0	0	0	9
Os01g0666600	2356	2354	2589	112.5	95.82	157.4	290	289	280	4044	4220	3612	4019.9	4354.6	4098.4	5170	3279	2921	286.1	219.3	250	182.6	248.7	240.5	4078	4044	3184	2924	2758	3388	3
Os01g0560000	562	636	565	910.5	1021	788.1	1102	980	1085	582	541.6	624.7	619.37	538.6	514.78	1182	453.4	449.6	806.3	670	788.1	806.7	850.2	876.1	500.7	452.6	570.2	504	471	524	12
Os01g0692200	51	45.6	47.4	160.9	105.8	124.6	36.8	46.2	46.3	89.67	97.58	107.3	113.21	72.809	78.405	95.53	45.34	55.95	161.6	150	149.2	29.58	29.93	46.95	82.4	75.43	87.72	87.9	82.2	60	16
Os01g0652600	1758	2009	1663	2068	2254	1849	3137	3001	2645	1611	1502	1921	1156	1211.6	1165	1457	1420	1364	1808	1455	1814	2045	2059	2131	1139	1375	1406	984	912	964	18
Os01g0105400	23.4	37.3	39.9	22.99	31.64	30.28	86.9	78.4	52.5	48.28	52.63	44.22	23.947	28.004	44.35	11.94	27.82	27.97	43.66	31.44	26.73	31.79	45.61	27.45	42.25	36.77	28.51	26.8	41.1	25	5
Os01g0886500	981	839	972	1263	1250	1407	957	975	964	986.3	959.3	1095	1201.7	1145.3	1135.7	1636	1208	1027	1684	1793	1730	752.7	765.4	793.7	1008	1127	1072	1143	1114	1131	16
Os01g0584200	0	0	0	0	0.904	0	0	0	0	0	0	0	0	0	0	0	0	0	0	0	0	0	0	0	0	0	0	0	0	0	2
Os01g0605300	6.12	1.86	2.16	21.35	26.22	26.82	16.4	25.9	22.1	0.862	1.096	0	0	0	2.3759	0	1.031	1.036	12.08	15.32	17.24	17.01	17.1	15.17	1.056	0.943	0	0	0.96	0.83	12
Os01g0498300	42.8	27.9	28	3.284	1.808	1.73	0	5.06	3.46	9.484	2.193	7.527	1.0885	6.5342	3.9598	23.88	23.7	21.76	4.645	2.419	1.724	3.697	3.563	15.89	8.451	8.486	0	3.7	9.56	3.33	14
Os01g0673500	1198	1244	1218	816.9	828	827.9	721	815	707	1311	1255	1134	1313.8	1324.6	1417.6	2102	1364	1158	879.7	857.1	859.6	747.5	657	764.1	1307	1235	1228	1260	1230	1349	13
Os01g0673300	54	58.7	57.1	59.93	34.35	47.58	87.7	70.8	87.8	124.2	98.68	129.8	72.931	72.809	83.156	59.7	36.07	56.98	91.04	69.34	76.74	63.59	52.02	54.89	127.8	97.11	109.6	69.4	66	56.7	20
Os01g0816100	103	94.1	88.4	77.17	23.5	78.72	40.7	52.5	197	25	17.54	14.11	10.885	9.3345	23.759	35.82	43.28	32.12	45.52	116.1	68.12	102.8	122.6	96.06	9.507	11.31	13.16	21.3	30.6	20	17
Os01g0639600	660	461	473	405.6	359.8	316.6	148	251	94.7	385.4	259.8	240.8	242.74	235.23	301.74	1111	831.6	503.5	451.5	467.6	432	236.6	420.5	294	306.3	255.5	274.1	292	292	362	13
Os01g0816500	0	0	1.08	0	0	0	0	0	0	0	0	0	0	0	0	0	0	0	0	0	0	0	0	0	0	0	0	0	0	0	14
Os01g0268900	306	275	330	495	602.9	557.1	501	478	571	261.2	301.5	268.1	481.13	478.86	319.95	776.2	354.5	357.4	678.1	797.4	691.5	417.8	352	522.9	294.7	212.1	241.2	298	324	347	10
Os01g0147250	3.06	4.66	2.16	0	0	1.73	0	5.06	0	5.173	1.096	0.941	3.2656	1.8669	3.9598	0	5.153	5.18	0.929	0.806	0	0.739	0.713	0	2.113	3.771	0	2.78	8.61	5.83	1
Os01g0219300	10.2	13	5.39	1.642	1.808	0	0	4.43	2.07	24.14	12.06	16.93	8.7082	12.135	8.7116	0	6.183	3.108	0.929	0	0	8.133	9.977	7.222	13.73	7.543	26.32	9.25	17.2	11.7	3
Os01g0342800	28.5	29.8	19.4	68.96	75.93	51.9	61.1	77.2	87.8	41.38	48.24	22.58	41.364	33.604	22.967	119.4	32.98	35.23	59.45	67.73	101.7	38.45	59.86	42.61	19.01	40.54	35.09	41.6	32.5	30.8	6
Os01g0757800	157	192	187	750.4	781.9	722.3	533	640	559	134.5	138.1	89.38	211.17	151.22	207.5	429.9	176.2	195.8	549.9	604.7	582.9	519.8	506	528	140.5	153.7	83.33	228	190	237	12
Os01g0781100	28.5	40.1	26.9	21.35	26.22	23.36	7.05	10.8	14.5	28.45	27.41	27.28	20.682	39.205	30.887	35.82	10.31	34.19	13.01	17.74	8.622	13.31	17.1	11.56	25.35	31.11	32.89	36.1	39.2	27.5	3
Os01g0926800	2212	2262	2510	1325	1409	1146	2367	2641	2094	3127	2704	2647	2653.8	2404.6	2932.6	4502	2271	2091	1580	1613	1417	1416	2053	1831	2730	2537	2377	2216	2412	2509	13
Os01g0815900	4.08	3.73	3.23	7.389	13.56	19.03	0	0	4.84	3.449	0	0	1.0885	0.9335	0	0	2.061	1.036	1.858	1.613	2.587	3.697	4.276	2.889	0	2.829	0	0	0	1.67	2
Os01g0104100	2111	2208	2235	2014	2159	2073	1968	2078	2072	2406	2548	2369	2743.1	2619.3	2636.5	4836	2587	2486	2373	2305	2238	1681	1655	1883	2720	2520	2267	2416	2455	2559	13
Os01g0631500	11.2	1.86	3.23	2.463	3.616	3.46	1.57	1.27	7.6	5.173	6.578	10.35	1.0885	7.4676	4.7518	35.82	9.275	10.36	6.503	0	1.724	2.218	0.713	4.333	7.395	5.657	13.16	0.93	10.5	2.5	13
Os01g0837100	0	0	0	0	0	0	0	0	0	0	0	0	0	0	0	0	0	0	0	0	0	0	0.713	0	0	0	0	0	0	0	17
Os01g0279400	420	452	408	1604	1691	2164	1624	1713	1717	520.8	470.4	445	459.36	666.48	562.3	2317	751.3	662	2283	2799	3307	1873	1821	1777	703.5	772.2	429.8	1086	918	883	10
Os01g0745000	0	0	0	0	0	0	0	0	15.9	0	0	0	0	0	0	0	0	0	0	0	0	7.394	7.839	6.5	0	0	0	0	0	0	17
Os01g0637500	36.7	40.1	50.6	18.06	29.83	24.22	21.1	17.1	16.6	30.18	37.28	45.16	46.806	54.14	40.39	83.59	65.95	68.38	43.66	43.54	33.63	15.53	24.94	23.11	36.97	66	37.28	84.2	99.5	65.8	13
Os01g0235632	0	0	1.08	0	0	0	0	0	0	0	1.096	0	0	0	0	0	0	1.036	0	1.613	0	0	0.713	0	0	0	0	0	0.96	0	1
Os01g0933400	385	296	364	289	366.1	322.7	254	340	221	331.9	371.7	355.6	341.8	418.19	401.53	214.9	337	404.1	306.6	373.3	338.9	369	308.6	250.6	409.9	344.1	335.5	441	552	432	1
Os01g0605400	0	0.93	0	5.747	7.232	6.055	8.61	3.8	2.76	0	0	0	1.0885	0.9335	0	0	0	0	5.574	20.16	0	2.218	0	7.222	0	0	0	0	0	0	12
Os01g0209000	821	781	875	807	790.1	846.9	680	638	679	821.7	835.5	816.6	856.67	822.37	852.16	1457	921.3	758.4	802.6	766	852.7	635.9	610.7	682.5	925.4	815.6	822.3	728	761	802	13
Os01g0873300	14.3	18.6	17.2	2.463	7.232	6.92	26.6	25.3	29	12.93	16.45	17.88	16.328	17.736	10.296	11.94	16.49	14.5	5.574	2.419	7.76	24.4	17.1	22.39	19.01	3.771	4.386	10.2	9.56	13.3	5
Os01g0658700	1.02	3.73	0	0	0.904	0.865	0	1.9	0.69	3.449	3.289	6.586	3.2656	0.9335	1.5839	0	4.122	2.072	0	1.613	1.724	3.697	1.425	2.167	2.113	3.771	8.772	5.55	4.78	2.5	11
Os01g0182400	106	105	95.9	107.5	96.72	118.5	79.1	67	85.7	88.81	103.1	89.38	133.89	164.29	148.1	155.2	108.2	85.99	78.03	98.37	144	95.38	101.2	83.78	114.1	93.34	100.9	128	109	131	13
Os01g0137300	79.5	60.5	61.4	74.71	71.41	66.61	83	65.1	65.6	55.18	65.78	43.28	67.488	57.874	50.686	59.7	57.71	76.67	66.88	65.31	79.32	73.94	60.57	63.56	57.04	60.34	39.47	87	66.9	70.8	2
Os01g0706400	1555	1544	1687	912.1	892.2	836.5	783	940	829	735.4	675.4	491.1	785.91	715.02	846.61	1815	941.9	1029	714.4	753.9	739.8	875.4	879.4	829.1	642.3	648.7	561.4	742	634	735	14

Os01g0158400	248	119	170	85.38	108.5	106.4	66.5	76.5	102	149.2	109.6	395.1	161.1	367.78	180.57	394.1	295.8	231	97.54	59.66	64.67	41.41	48.46	43.33	315.9	330.9	432	233	202	246	3
Os01g0959000	462	523	516	606.7	667.1	612.5	352	385	347	473.3	485.7	488.3	515.96	547	553.58	752.3	459.6	574	509.1	462.8	536.3	461.4	410.5	337.3	497.5	506.3	548.2	549	517	572	13
Os01g0601625	87.7	142	111	307	329	285.5	454	380	274	237.1	118.4	75.27	66.4	103.61	89.492	119.4	75.23	96.35	258.2	267.7	266.4	344.6	268	265.1	90.85	76.37	57.02	84.2	82.2	76.6	12
Os01g0694300	528	579	553	551.7	541.5	508.6	588	605	580	687.2	683.1	747	746.72	684.22	729.4	895.6	602.9	662	625.2	623.3	638	493.9	518.1	502.7	657.1	736.4	679.8	563	555	605	13
Os01g0539900	329	314	314	334.1	377	322.7	330	352	319	332.8	348.7	446.9	370.1	415.39	353.22	597	293.7	299.4	346.5	413.6	394	323.1	329.9	324.3	371.8	439.4	390.3	329	377	291	6
Os01g0773800	95.8	154	60.3	0.821	0	0	0.78	1.9	0.69	0.862	0	0	6.5311	0.9335	4.7518	11.94	28.85	24.86	2.787	0	0	0	1.425	0	3.169	1.886	15.35	2.78	0	0	14
Os01g0935000	0	0	0	0	0	0	0	0.63	0	0	0	0	0	0	0	0	0	0	0.929	0.806	0	0	0	0	0	0	0	0.93	0	0	16
Os01g0610100	1167	1001	969	947.4	1036	981.8	1261	1034	1249	1263	1123	1375	1028.7	1158.4	956.69	1588	1119	1041	1167	939.3	1047	1032	1110	1127	1210	1350	1445	1018	949	879	6
Os01g0872700	1931	2011	2009	2605	2707	2731	1128	1173	1213	1549	1677	1564	1832	1768	1883.3	3535	2096	1993	2438	2554	2813	1666	1541	1478	1786	1958	1754	2091	1921	2040	16
Os01g0113350	78.5	84.8	77.6	55.01	44.29	50.17	75.9	77.8	69.8	83.63	105.3	57.39	88.17	48.539	82.364	83.59	50.5	67.34	30.66	47.57	36.21	84.29	79.82	75.11	83.45	68.83	59.21	71.2	65	74.1	5
Os01g0740350	8.16	4.66	3.23	0	2.712	0.865	0	3.16	0	2.587	4.386	5.645	4.3541	3.7338	16.631	0	13.4	3.108	6.503	0.806	0	2.218	0	0	1.056	4.714	2.193	3.7	3.83	8.33	4
Os01g0391500	651	656	686	635.4	641.8	679.9	448	463	417	536.3	586.6	577.7	722.78	735.56	637.53	1505	701.8	679.6	548.1	653.9	612.2	592.2	523.8	489	596.8	667.5	585.5	756	688	784	13
Os01g0153800	0	0	0	0	0	0	0	0.63	0	0	0	0	0	0	0	0	0	0	0	0	0	0	0	0	0	0	0	0	0	0	18
Os01g0640000	1.02	5.59	4.31	2.463	1.808	0.865	2.35	0	1.38	8.622	3.289	12.23	1.0885	4.6673	5.5438	0	4.122	3.108	6.503	6.45	3.449	0.739	4.276	2.889	6.338	0.943	4.386	4.63	3.83	3.33	20
Os01g0178000	366	736	534	2079	2309	2133	1446	1517	1370	474.2	598.6	427.1	830.54	538.6	605.85	1182	517.3	506.6	2021	1963	1700	1409	1556	1288	670.8	564.8	598.7	711	729	818	10
Os01g0719100	8752	4686	7111	1505	1212	1445	1142	1343	1139	8443	7605	10755	10081	9168.4	8577	6233	6753	6793	2096	2191	1968	1514	1198	1118	9759	8001	9090	6702	5489	5763	3
Os01g0700300	0	0	0	0	0	0	0	0	0	0	0	0	0	0	0	0	0	1.036	0	0	0	0	0	0	0	0	0	0	0	0	1
Os01g0708201	3.06	0.93	0	0.821	0	1.73	0	2.53	2.07	0	2.193	0	1.0885	0.9335	0.792	0	1.031	2.072	0.929	1.613	8.622	0	1.425	0.722	2.113	2.829	0	2.78	2.87	0.83	16
Os01g0934901	9.18	12.1	11.9	26.27	36.16	19.9	4.7	1.27	7.6	6.897	8.771	6.586	2.177	9.3345	11.879	23.88	10.31	21.76	17.65	19.35	23.28	18.48	5.701	10.83	2.113	3.771	2.193	15.7	9.56	10	10
Os01g0174300	240	211	214	307.9	282	332.2	215	201	196	179.3	188.6	155.2	136.06	167.09	131.47	274.6	184.5	184.4	328.8	196.7	240.6	141.2	189.6	161.8	124.7	152.7	206.1	166	143	144	10
Os01g0784600	302	361	346	525.4	501.7	488.8	446	476	374	331.9	382.6	358.5	420.17	322.97	312.03	489.6	255.6	331.5	488.6	578.9	532	338.6	336.4	378.5	304.2	314.9	344.3	276	335	283	10
Os01g0248800	2.04	1.86	7.54	0.821	5.424	4.325	0.78	3.16	4.15	0.862	2.193	1.882	0	4.6673	2.3759	0	4.122	5.18	3.716	0.806	0	0	0.713	2.167	3.169	0.943	10.96	5.55	2.87	1.67	11
Os01g0184800	129	102	144	110.8	130.2	119.4	139	162	173	120.7	136	107.3	115.38	113.88	133.05	250.8	127.8	109.8	70.6	75.79	71.56	176.7	161.8	153.8	102.5	110.3	157.9	125	123	142	6
Os01g0974400	138	169	172	197.9	214.2	121.1	333	295	267	201.8	241.2	266.3	253.63	234.3	201.16	274.6	194.8	163.7	219.2	181.4	194.9	153.1	131.1	223.2	236.6	305.5	296	225	224	253	5
Os01g0763200	179	225	153	114.9	52.43	107.3	135	41.7	102	133.6	122.8	166.5	81.639	121.35	112.46	334.3	239.1	202	199.7	144.3	196.6	244	270.1	375.6	207	266.8	348.7	253	182	220	11
Os01g0259900	117	113	139	101	112.1	120.2	128	101	144	85.36	120.6	76.21	119.74	115.75	80.781	238.8	112.3	117.1	65.03	82.24	93.12	130.1	74.11	117.7	61.27	56.57	39.47	112	86.1	87.5	6
Os01g0119000	0	0	0	0	0	0.865	3.13	0.63	2.76	0	0	0	0	0	0	0	0	0	0	0	0	1.479	4.988	0	0	0	0	0	0	0	17
Os01g0783900	108	170	142	383.4	372.4	342.6	86.1	94.9	111	93.12	85.52	35.75	118.65	81.21	118	322.4	89.66	122.3	173.7	215.3	202.6	223.3	199.5	135.8	94.02	133.9	26.32	157	130	157	2
Os01g0714600	413	632	343	88.67	46.1	44.12	58.7	114	107	206.9	81.13	93.14	74.019	70.942	70.485	477.6	534.8	360.5	47.38	74.18	81.91	181.1	218.8	473.1	97.19	86.74	98.68	48.1	64.1	152	14
Os01g0757051	66.3	51.2	51.7	15.6	33.45	8.65	14.1	10.1	32.5	30.18	23.02	16.93	20.682	32.671	19.007	155.2	75.23	91.17	24.15	12.09	17.24	14.79	6.414	16.61	25.35	24.51	46.05	14.8	11.5	20	13
Os01g0674500	1176	998	1072	799.6	717.8	861.6	669	686	730	647.5	667.7	613.4	824.01	715.02	747.62	1672	887.3	983.2	768.2	751.5	766.5	792.6	816.7	743.9	684.5	628.9	671	918	813	881	6
Os01g0828200	11.2	17.7	2.16	0	1.808	0.865	0	0.63	1.38	3.449	8.771	8.467	3.2656	0.9335	2.3759	0	6.183	10.36	8.361	1.613	0	1.479	0.713	4.333	1.056	9.428	19.74	3.7	3.83	2.5	11
Os01g0588900	####	####	####	16074	11267	21178	4189	5777	6444	18181	15704	20413	18608	19692	18361	36909	24124	24442	28945	24557	35437	6254	6389	6427	25597	25000	26374	####	####	####	13
Os01g0610500	304	316	312	315.3	307.3	425.6	355	299	355	352.6	288.4	293.5	313.49	331.38	294.61	800	346.3	273.5	395.7	400.7	339.7	300.2	292.9	343.1	334.9	314.9	269.7	256	321	249	6
Os01g0575801	7.14	6.52	8.62	1.642	1.808	1.73	2.35	0	0	7.76	7.675	0	1.0885	6.5342	3.9598	11.94	3.092	3.108	0.929	3.225	2.587	2.218	0.713	0.722	5.282	7.543	10.96	3.7	1.91	4.17	13
Os01g0875000	0	0	0	10.67	15.37	9.516	0.78	1.27	0.69	1.724	0	0	2.177	0	0.792	0	0	0	12.08	6.45	16.38	2.958	0	0	0	2.829	0	0	0	0	10
Os01g0219200	13.3	10.2	4.31	7.389	6.328	13.84	6.26	3.16	12.4	8.622	6.578	6.586	5.4426	17.736	4.7518	23.88	10.31	7.252	14.86	8.063	8.622	13.31	4.988	10.83	5.282	5.657	8.772	18.5	9.56	8.33	6
Os01g0338150	256	224	210	197	191.6	203.3	178	225	193	275.9	312.5	291.7	306.96	309.91	276.4	394.1	233.9	214.5	236.9	220.1	261.3	186.3	238.7	211.6	297.9	264.9	271.9	254	219	267	13
Os01g0781700	23.4	12.1	20.5	82.1	33.45	15.57	15.7	21.5	8.98	24.14	17.54	21.64	33.744	16.802	22.967	11.94	15.46	60.09	19.51	15.32	14.66	25.88	26.37	22.39	12.68	15.09	13.16	38.9	23.9	25.8	2
Os01g0604150	0	0	0	0	0	0	0	0	0	0	0	0	0	0	0	0	0	0	0	0	0	0	0	0	0	0	0	0.93	0	0	1
Os01g0681900	386	468	458	541	556.8	593.4	640	607	558	270.7	242.3	245.6	225.32	266.97	297.78	680.6	402.9	365.7	342.8	329	328.5	1068	1266	951.9	268.3	230.1	307	463	377	395	17
Os01g0553400	203	259	212	224.9	241.4	228.4	208	230	225	213.8	274.1	234.3	322.2	302.44	318.37	298.5	261.8	278.7	203.4	198.3	204.3	224.8	223.8	208	302.1	230.1	195.2	253	215	256	4
Os01g0276800	1459	1519	1423	1525	1358	1494	1937	1855	1795	1462	1545	1428	1505.4	1382.4	1496	2484	1415	1320	1343	1356	1282	1550	1602	1695	1537	1559	1662	1422	1289	1480	6
Os01g0587000	2185	1938	2001	1694	1774	1823	1440	1391	1426	1828	1688	2086	1687.2	1994.8	1660.8	2854	2201	2053	1843	1605	1653	1370	1691	1564	1877	1927	2353	2053	1822	1815	13
Os01g0545100	0	0	0	1.642	0	0	0	0.63	0	0	0	0	0	0	0	0	0	0	0	0	0	0	0	0	0	0	0	0	0	0	2
Os01g0580500	7.14	4.66	0	2.463	2.712	3.46	0	2.53	20	1.724	0	4.704	0	4.6673	3.1679	0	6.183	2.072	10.22	0.806	3.449	24.4	2.851	12.28	1.056	0	2.193	0	3.83	0.83	9
Os01g0204000	150	155	169	216.7	217.9	195.5	213	257	199	158.6	171	90.32	174.16	161.49	185.32	310.5	135	173	153.3	201.6	167.3	215.9	215.9	197.2	148.9	140.5	103.1	175	155	179	6
Os01g0246400	237	109	208	13.96	5.424	18.17	5.48	5.69	1.38	136.2	132.7	226.7	527.93	405.12	385.69	274.6	421.5	292.2	94.75	164.5	65.53	0	6.414	5.056	233.5	173.5	175.4	387	294	293	3
Os01g0829500	0	0	0	0	0	0	0	0	0	0	0	0.941	0	0	0	0	0	0	0	0.806	0	0	0	0	0	0	0	0	0	0	20
Os01g0731800	70.3	44.7	55	59.93	48.81	65.74	78.3	75.3	69.8	48.28	40.57	56.45	29.39	32.671	36.43	23.88	48.43	61.13	55.74	60.47	75.01	118.3	125.4	106.2	54.93	58.46	70.17	57.4	41.1	33.3	17
Os01g0606050	64.2	59.6	51.7	181.4	217.9	192	165	164	138	33.63	47.14	50.8	25.036	52.273	30.887	131.4	48.43	69.41	192.3	219.3	211.2	155.3	129.7	140.8	44.37	40.54	35.09	56.4	64.1	31.7	12
Os01g0705100	0	0	1.08	0	0	0	0	0.63	0	0	0	0	0	0	0	0	0	0	0	0	0	0.739	0.713	0	0	0	0	0	0	0	17
Os01g0874100	876	1044	996	1960	2137	1617	1053	1434	1064	894.9	1097	776.2	1137.5	1130.4	997.09	1230	834.7	872.3	1700	1672	1606	724.6	1051	845.7	839.8	947.6	679.8	755	861	929	10
Os01g0738800	74.4	58.7	36.6	44.33	67.8	64.01	72	49.3	46.3	48.28	65.78	59.27	38.098	47.606	49.102	83.59	53.59	41.44	78.03	41.12	86.22	34.75	52.73	33.22	35.92	49.03	103.1	55.5	70.8	55.8	11
Os01g0783000	0	0	0	0	0.904	0	0.78	0	0	0	0	0	0	0	0	0	0	0	0	0	0	0	0.713	0.722	0	0	0	0	0	0	17
Os01g0175100	111	191	111	1172	776.5	1334	3681	3258	5449	147.4	94.29	59.27	105.59	113.88	87.908	119.4	176.2	145	1491	1317	2162	4423	3570	4555	86.62	65.06	85.52	78.6	78.4	106	18
Os01g0103700	76.5	39.1	72.2	59.11	56.05	76.12	78.3	82.2	76	52.59	41.66	66.8	48.983	60.674	52.27	71.65	60.8	62.16	86.39	77.4	73.29	80.59	111.9	65.72	55.99	64.11	61.4	73.1	67.9	45	17
Os01g0622901	2.04	0	1.08	0	0	0	0	0	0	2.587	0	0	0	0	0.792	0	3.092	1.036	0	3.225	0	0.739	0	0	1.056	0.943	2.193	0	0.96	0.83	19
Os01g0604100	24.5	35.4	25.9	59.93	48.81	51.9	65.8	53.1	78.8	29.31	38.37	36.69	35.921	33.604	23.759	83.59	54.62	32.12	57.6	71.76	70.7	76.9	75.54	88.84	45.42	44.31	43.86	36.1	42.1	41.7	12
Os01g0972000	326	471	211	110.8	99.44	71.8	96.3	94.2	76	63.8	43.86	41.4	69.665	56.007	42.766	298.5	362.7	247.6	105	108	259.5	81.33	77.68	376.3	36.97	21.69	39.47	49	43	58.3	14
Os01g0940000	4237	3030	3568	806.2	604.8	840	132	183	167	1943	2259	1805	2283.7	3872	3372.2	6388	4637	3497	959.6	1219	1431	398.5	506.7	426.1	4612	5903	5177	4554	3414	3910	13
Os01g0139900	1.02	3.73	1.08	3.284	1.808	4.325	3.91	0	0	4.311	0	2.822	0	8.4011	0	0	1.031	3.108	0.929	0	0	0.739	0	0	0	0	4.386	3.7	2.87	1.67	11
Os01g0360200	76.5	115	101	571.4	539.7	741.3	308	219	214	147.4	145.8	141.1	108.85	145.62	99.788	107.5	122.6	134.7	552.7	540.2	689.8	261	362.7	322.8	117.3	158.4	212.7	162	124	136	10
Os01g0168800	11.2	8.38	14	9.031	9.04	25.95	7.83	1.9	0.69	23.28	4.386	15.05	1.0885	14.935	5.5438	11.94	10.31	11.4	6.503	8.063	9.484	5.915	12.11	10.11	4.225	16.97	19.74	12	6.69	7.5	11
Os01g0279900	682	631	807	697.8	635.5	667.8	489	490	505	716.5	654.5	604.9	906.74	785.03	807.81	1457	803.8	880.6	657.7	732.1	671.7	542.7	506.7	492.6	700.4	766.5	609.6	832	873	942	13
Os01g0599900	0	0.93	0	1.642	0	0	0	0	0	0	0	0	0	0	0	0	0	0	0	0	0	0	0	0	0	0	0	0	0	0	2
Os01g0115533	41.8	76.4	89.4	77.17	87.69	53.63	121	211	122	100	65.78	41.4	90.347	77.476	96.62	71.65	43.28	45.59	40.87	39.51	32.76	116.1	130.4	64.28	54.93	49.03	65.79	99.9	66.9	71.6	18
Os01g0874900	1878	1713	1781	1195	1108	1121	813	887	804	1639	1764	1433	1847.2	1746.5	1983.1	2758	1961	1688	1186	1080	1105	766.7	810.3	767.7	1965	1768	1930	1567	1592	1882	13
Os01g0746200	544	544	547	779.1	901.3	793.2	584	596	555	419	469.3	347.2	448.47	497.53	449.84	859.7	427.7	495.2	629.8	643.4	726	595.9	549.4	529.4	403.5	396.9	333.3	519	450	499	10
Os01g0973000	10.2	4.66	9.7	25.45	32.54	36.33	38.4	23.4	29.7	16.38	1.096	10.35	3.2656	10.268	6.3357	0	12.37	6.216	22.29	18.54	26.73	25.88	27.79	26.72	5.282	7.543	10.96	3.7	5.74	3.33	12
Os01g0507700	3300	2901	2956	1280	1100	1563	593	699	562	2320	2211	2668	1646.9	2773.3	2067	4430	4094	3294	1650	1721	2050	1453	1870	1504	3371	4237	4153	3818	2863	2878	13
Os01g0233000	1093	1003	818	3687	4309	3230	2793	3557	2126	491.4	548.2	438.4	706.45	767.3	791.97	1170	1177	809.1	2642	2841	2485	2411	2568	3187	734.2	594.9	831.1	1123	770	1143	12
Os01g0540300	0	0	0	0	0	0	0	0.63	0	0.862	0	0	0	0	0	0	0	0	0	0.806	0	0	0	0.722	0	0	0	0	0	0	7
Os01g0779300	38.7	63.3	55	88.67	87.69	68.34	95.5	130	95.4	57.77	64.69	75.27	52.249	73.743	69.693	35.82	60.8	66.31	92.9	88.69	75.88	75.42	96.92	87.39	65.49	69.77	94.3	69.4	82.2	55	18
Os01g0653300	0	0	1.08	1.642	0	0	0	0	0	0	0	0	0	0.9335	0	0	0	0	0	1.613	2.587	1.479	3.563	1.444	0	0	0	0	0	0	17
Os01g0938100	2366	1830	2335	535.3	459.2	560.6	994	1022	858	2178	2190	3578	2668	2619.3	2405.2	4167	3587	2646	1511	1095	1256	513.1	590.1	918	2348	3098	3204	1811	2246	2043	3
Os01g0648700	2.04	1.86	2.16	3.284	1.808	2.595	7.83	3.16	3.46	0	5.482	4.704	4.3541	0	1.5839	0	4.122	4.144	3.716	4.031	1.724	5.176	15.68	16.61	6.338	0	0	8.33	2.87	1.67	17
Os01g0685200	1.02	3.73	4.31	4.105	1.808	0.865	4.7	6.96	3.46	6.035	4.386	3.763	2.177	2.8004	0	0	1.031	2.072	6.503	3.225	7.76	7.394	3.563	8.667	0	2.829	0	6.48	0	2.5	9
Os01g0166300	66.3	58.7	37.7	73.07	38.87	86.5	101	81.6	105	57.77	51.53	61.15	47.895	55.074	56.23	95.53	76.26	31.08	97.54	72.57	88.81	71.72	81.24	71.5	45.42	35.83	54.82	42.6	36.3	25.8	12
Os01g0208000	0	0	0	0	0.904	1.73	0	1.9	0	0	0	0	0	0	0	0	0	0	0	0	0	0	0	0	0	0	0	0.93	0	0	2
Os01g0290650	22.4	33.5	24.8	39.41	31.64	17.3	20.4	47.4	33.9	45.7	14.25	28.22	21.77	20.536	44.35	11.94	15.46	15.54	25.08	14.51	17.24	31.79	38.48	37.56	36.97	19.8	26.32	29.6	39.2	20	17
Os01g0155000	0	1.86	2.16	9.852	0.904	6.055	0	11.4	0	5.173	1.096	0.941	1.0885	2.8004	1.5839	11.94	0	1.036	1.858	4.031	11.21	1.479	4.276	1.444	3.169	2.829	2.193	1.85	8.61	0.83	6
Os01g0923900	412	421	411	378.5	338.1	334.8	460	459	428	419	457.2	442.2	428.88	421.92	487.85	525.4	422.5	460	328.8	351.5	343.2	407.4	407.6	405.2	437.3	487.4	414.5	415	385	493	13
Os01g0607100	2.04	0	3.23	2.463	0.904	3.46	3.91	1.27	4.15	0	2.193	0	5.4426	0.9335	0	0	1.031	0	5.574	7.257	5.173	3.697	2.138	1.444	1.056	0.943	0	0	3.83	0	12
Os01g0951000	610	577	635	645.3	688.8	654	804	772	879	728.5	677.6	724.4	701.01	656.22	666.04	1003	697.7	737.7	654	603.1	602.7	748.3	662.8	743.9	664.5	722.2	780.7	722	627	643	6
Os01g0867100	447	521	474	399	448.4	391.9	406	387	395	396.6	448.4	352.8	544.26	433.12	475.97	907.5	510.1	556.4	439.4	427.3	378.5	467.3	439.7	403.7	536.6	505.4	390.3	566	546	626	13
Os01g0695700	1682	1823	2025	380.1	439.3	412.6	148	193	159	1867	1867	1855	1658.9	2047.1	1968.8	4179	2816	2319	693.9	641	853.6	127.2	127.6	174.1	2468	2293	2171	2396	2350	2352	13
Os01g0613300	240	292	334	324.3	425.8	365.9	578	497	500	323.3	388.1	346.2	370.1	302.44	319.95	274.6	238.1	232.1	378.1	335.4	332.8	354.9	311.4	366.2	206	248	243.4	218	248	216	18
Os01g0101200	247	365	273	582.1	546	439.4	806	1106	1093	509.6	561.4	950.2	372.27	210.96	264.52	477.6	280.3	267.3	544.4	396.7	555.3	579.7	555.1	838.5	461.6	432.8	388.1	126	161	199	18
Os01g0699600	526	730	310	20.52	6.328	9.516	6.26	1.9	7.6	0.862	4.386	8.467	19.593	1.8669	6.3357	131.4	159.7	189.6	10.22	3.225	50.87	36.23	21.38	104	1.056	2.829	10.96	0.93	0.96	6.66	14
Os01g0545900	23.4	25.1	19.4	68.96	66.89	63.15	30.5	23.4	24.9	27.59	29.6	17.88	32.656	22.403	20.591	59.7	21.64	31.08	67.81	58.05	59.49	39.19	33.49	37.56	29.58	49.03	26.32	27.8	44	29.2	10
Os01g0705500	251	266	252	211	224.2	220.6	355	257	284	281.9	255.5	279.4	201.38	208.16	200.37	226.9	213.3	231	217.4	205.6	250	252.1	237.3	297.6	240.9	233.8	186.4	183	196	175	18
Os01g0759400	347	360	394	548.4	589.4	436.8	178	112	110	450.1	518.6	410.2	716.25	509.66	577.34	991.1	384.4	439.3	488.6	503.1	544.1	92.42	117.6	106.9	436.3	367.7	370.6	354	419	480	13
Os01g0572200	0	0	0	0	0	0	0	0	0	0	0	0	0	1.8669	0	0	0	0	0	0	0	0	0	0	0	0	0	0.93	0	0	4
Os01g0176700	20.4	20.5	21.6	38.59	42.49	41.52	21.9	22.1	15.9	17.24	8.771	22.58	16.328	18.669	7.1277	23.88	26.79	32.12	49.23	22.58	23.28	28.1	22.8	13.72	8.451	19.8	10.96	13.9	14.3	7.5	10
Os01g0102800	252	256	245	291.4	256.7	235.3	236	238	207	228.5	240.1	222	249.27	196.02	245.51	453.8	262.8	215.5	236.9	223.3	209.5	268.4	218.1	253.5	249.3	232.9	250	225	210	268	6
Os01g0685800	2.04	1.86	0	0.821	3.616	1.73	1.57	1.27	0.69	3.449	4.386	4.704	4.3541	1.8669	2.3759	0	1.031	0	0.929	4.031	2.587	0.739	1.425	1.444	1.056	0.943	2.193	0.93	0.96	1.67	7
Os01g0205200	61.2	119	106	437.6	464.6	410	238	359	251	120.7	159	141.1	94.701	95.212	55.438	95.53	56.68	85.99	226.7	300.7	274.2	252.9	265.1	290.3	112	116.9	50.44	58.3	76.5	96.6	12
Os01g0543400	1.02	5.59	3.23	1.642	4.52	0.865	0	3.8	2.76	0.862	3.289	0.941	4.3541	1.8669	0	0	1.031	6.216	1.858	0	0.862	1.479	0	5.056	0	0.943	0	2.78	0.96	3.33	14
Os01g0891033	7.14	3.73	7.54	6.568	0.904	0.865	10.2	13.9	5.53	3.449	9.868	18.82	7.6196	3.7338	5.5438	0	8.244	2.072	3.716	7.257	4.311	5.915	8.552	4.333	7.395	6.6	10.96	5.55	3.83	3.33	20
Os01g0863166	311	267	291	292.3	301.9	336.5	366	333	334	264.7	278.5	266.3	293.9	251.1	262.93	334.3	329.8	240.4	279.6	290.3	298.3	442.9	393.4	361.1	303.2	263.1	298.2	298	340	321	17
Os01g0209500	2.04	3.73	3.23	39.41	43.39	44.98	0	0	0.69	0.862	3.289	2.822	2.177	3.7338	3.1679	0	1.031	2.072	18.58	20.16	27.59	1.479	2.138	0.722	0	1.886	2.193	4.63	3.83	2.5	10
Os01g0772400	364	340	387	217.6	159.1	237	335	317	292	326.8	300.4	328.3	302.61	308.97	335	453.8	347.3	287	235	268.5	220.7	221.8	233.7	244.1	341.2	327.2	348.7	226	270	277	13
Os01g0851300	7.14	5.59	2.16	4.926	5.424	12.11	16.4	11.4	24.2	0.862	2.193	2.822	0	4.6673	0.792	23.88	11.34	4.144	2.787	0.806	5.173	5.176	13.54	19.5	1.056	8.486	2.193	2.78	0.96	2.5	18
Os01g0737200	7.14	6.52	19.4	11.49	7.232	10.38	1.57	6.96	4.15	5.173	4.386	2.822	8.7082	5.6007	3.1679	0	10.31	12.43	4.645	7.257	6.898	4.436	6.414	1.444	3.169	0.943	0	13	2.87	9.16	8
Os01g0254900	1621	1507	1572	1215	1137	1187	1216	1274	1239	1683	1597	1620	1681.8	1700.7	1796.2	3714	1620	1639	1390	1121	1245	1104	1203	1140	1623	1646	1700	1464	1421	1513	13
Os01g0878700	1.02	0	0	2.463	0	0.865	0	0	0	1.724	2.193	4.704	0	0	3.9598	0	0	1.036	0	1.613	0	0	1.425	0.722	0	0	4.386	0	0.96	0	20
Os01g0958400	327	339	330	413	369.7	418.7	274	338	305	348.3	337.7	302	384.25	301.5	349.26	740.3	338	347.1	310.3	468.4	373.3	386	297.2	327.9	380.3	297	298.2	385	352	372	6
Os01g0608900	3.06	1.86	2.16	4.105	6.328	6.92	0	0	1.38	2.587	0	0	0	0	0	0	0	2.072	0.929	2.419	0	1.479	2.138	2.167	0	1.886	0	0.93	0.96	0	2
Os01g0770700	313	348	406	344	348	151.4	130	192	187	541.5	445.1	414	353.77	250.17	520.32	883.6	330.8	292.2	368.8	287	363	55.45	81.95	35.39	391.9	289.5	309.2	206	239	262	13
Os01g0158900	191	257	231	1304	1140	1302	222	213	205	197.4	164.5	149.6	177.43	174.56	210.66	477.6	230.8	207.2	1042	1191	1221	279.5	266.5	213.8	183.8	202.7	162.3	282	254	288	10
Os01g0934000	308	286	359	290.6	355.3	288.1	368	416	405	348.3	335.5	366.2	371.19	331.38	380.94	585.1	329.8	269.4	326.1	287.8	310.4	321.6	372	341.6	326.4	307.4	298.2	326	313	305	6
Os01g0723600	139	134	111	110.8	47.01	59.69	41.5	72.1	72.6	43.11	14.25	31.99	32.656	30.804	57.814	71.65	58.74	71.49	37.16	58.05	62.94	24.4	36.34	21.67	25.35	32.06	48.24	25	37.3	35.8	14
Os01g0672700	489	537	548	450.7	442.9	509.5	489	517	520	514.7	521.9	613.4	682.5	677.69	617.73	812	554.4	624.7	500.7	510.4	558.7	550.1	468.2	478.8	527.1	663.8	620.6	575	583	508	13
Os01g0930200	3880	3426	3761	2780	2497	2626	2843	3214	3223	3001	2777	2377	2460.1	2192.7	2653.9	5027	3675	3086	3164	3995	3364	3152	3260	3855	2825	2761	2724	2563	2724	2866	6
Os01g0135900	1634	1772	1812	1152	1034	848.6	444	441	498	1138	1124	1114	753.26	992.26	1048.6	1254	1004	1159	1132	729.7	627.7	743.8	756.8	758.4	1184	980.6	1535	969	806	942	14
Os01g0905800	2863	2621	2731	1359	1469	1438	2586	2234	2349	3592	2775	4363	2061.7	2551.1	2308.6	3523	2832	2768	1610	1134	1625	2677	2828	2211	3068	3182	3487	2552	2080	2012	20

Os01g0336300	0	0	0	0	0	0	0	0	0	0.862	0	0	0	0	0	0	0	1.036	0	0	0.862	0	0	0	1.056	1.886	6.579	0	0	0	11
Os01g0910500	1013	739	830	629.7	720.5	637.5	187	472	308	535.4	634.8	913.5	429.97	902.65	632.78	1266	999.6	1451	586.2	308.8	206.9	411.8	429	400	653.9	1369	1550	635	855	804	13
Os01g0292900	1242	1293	1235	1258	1223	1100	1580	1430	1529	1435	1341	950.2	1220.2	1075.3	1288.5	2281	1121	1142	1029	1098	1108	1785	1810	1623	1162	1065	1136	1316	1250	1305	6
Os01g0664500	1317	1210	1142	623.9	651.8	734.4	632	581	557	1516	1246	1763	933.95	1068.8	906.8	1899	1207	1399	859.3	748.2	861.4	400.7	402.6	460.1	1125	1141	1384	1085	1292	989	13
Os01g0690200	35.7	36.3	45.3	5.747	10.85	12.98	15.7	16.4	14.5	44.83	26.31	13.17	22.859	25.203	24.551	35.82	25.76	20.72	1.858	3.225	4.311	8.873	10.69	13.72	24.3	22.63	17.54	34.2	27.7	55	3
Os01g0298301	29.6	36.3	45.3	24.63	33.45	26.82	49.3	50.6	36.6	31.9	39.47	16.93	48.983	34.538	41.182	95.53	41.22	39.37	39.02	36.28	35.35	14.05	29.93	34.67	27.47	45.26	6.579	25.9	35.4	39.2	6
Os01g0235900	2.04	8.38	7.54	0	0	1.73	6.26	11.4	4.15	14.66	12.06	11.29	6.5311	12.135	10.296	11.94	11.34	9.324	1.858	0	0.862	0	1.425	5.778	9.507	22.63	10.96	3.7	3.83	18.3	3
Os01g0207400	48.9	45.6	43.1	59.11	23.5	81.31	59.5	63.3	47.7	49.14	47.14	132.7	32.656	52.273	52.27	47.76	72.14	56.98	74.32	34.67	89.67	62.11	93.36	131.4	59.16	91.46	122.8	63.8	73.6	50	11
Os01g0966400	516	562	543	651	653.6	647.9	1286	1172	1352	741.5	715.9	842	795.71	755.16	750.78	1146	590.5	549.1	675.3	693.4	619.1	865.8	884.4	965.6	722.6	774.1	787.3	695	598	624	18
Os01g0115800	116	137	102	82.92	56.05	50.17	219	219	111	237.1	211.6	190	130.62	170.82	184.53	155.2	122.6	136.8	115.2	128.2	97.43	134.6	216.6	161.1	191.2	158.4	186.4	208	133	167	5
Os01g0744550	0	0	0	0	0	0	0	0	0	0	0	0	3.2656	0	0	0	0	0	0	0	0	0	0	0	0	0	0	0	0	0	4
Os01g0275300	347	372	316	678.9	781.9	634.9	981	880	946	365.6	483.5	506.2	382.07	333.24	273.23	417.9	309.2	326.4	805.4	773.2	888.9	763.8	659.2	830.6	310.6	314	412.3	228	261	243	12
Os01g0825700	443	445	464	518	499.9	500	448	502	473	419.9	400.2	427.1	559.5	477.93	555.96	1039	586.4	494.2	469.1	491.8	571.6	495.4	509.5	464.4	539.8	533.6	491.2	490	503	579	6
Os01g0611950	0	0	1.08	0	0	0	0	0	0	0	0	0	0	0	0	0	0	0	0	0	0	0	0	0	0	0	0	0	0	0	14
Os01g0921300	648	645	707	329.2	273	301	381	395	429	644.1	640.3	674.6	633.52	687.02	643.87	895.6	726.5	617.5	351.1	386.2	374.2	370.4	386.2	398.7	735.2	811.8	732.4	634	755	630	3
Os01g0337700	244	244	252	219.2	304.6	251.7	229	260	215	173.3	259.8	163.7	215.53	255.77	225.71	453.8	282.4	293.2	224.8	251.6	263.8	293.5	258	243.4	250.4	249.9	193	312	286	292	6
Os01g0848700	1.02	0	0	1.642	0	0	0	0.63	0	0	0	0	0	0	0	0	0	0	0	0	0	0	0.713	0	0	0	0	0	0	0	2
Os01g0742100	26.5	55.9	39.9	352.2	513.5	356.4	210	221	193	37.94	54.82	38.57	54.426	50.406	30.887	23.88	31.95	31.08	219.2	212.9	244	131.6	151.1	124.2	25.35	18.86	10.96	24.1	35.4	38.3	10
Os01g0763700	481	366	428	560.7	597.5	570.9	1189	1174	1394	438.9	530.7	690.6	499.63	393.92	391.23	823.9	439	407.2	546.2	591.8	702.7	833.3	595.1	1052	512.3	545.9	541.7	306	388	332	18
Os01g0128800	3296	4207	4013	5238	5102	4956	542	627	511	2748	2794	1961	2710.4	2669.7	3526.6	6615	4037	4056	4706	5042	4275	785.2	1049	972.9	3282	3075	3101	4841	4423	4757	16
Os01g0388200	38.7	20.5	52.8	34.48	53.33	42.39	54.8	66.4	50.4	44.83	23.02	42.34	57.692	56.007	29.303	23.88	21.64	38.33	35.3	26.61	18.97	54.71	37.06	33.22	23.24	25.46	26.32	38.9	25.8	27.5	18
Os01g0330100	9.18	7.45	4.31	10.67	10.85	19.03	14.1	14.5	22.1	10.35	8.771	6.586	5.4426	13.068	2.3759	11.94	14.43	14.5	7.432	8.063	5.173	15.53	10.69	14.44	4.225	1.886	10.96	10.2	15.3	15	18
Os01g0353600	0	0	1.08	0	0	0	1.57	1.27	1.38	0.862	0	0.941	0	1.8669	0	0	2.061	1.036	2.787	1.613	0.862	0	0.713	5.778	0	0	2.193	0	0.96	1.67	9
Os01g0773700	####	####	####	12098	11808	12057	####	####	####	30355	34793	42200	26401	24730	24805	23010	22423	22637	16786	13310	17763	16046	14494	21602	29025	32506	29006	####	####	####	20
Os01g0314300	1249	1298	1360	1112	1181	1163	1165	1153	1213	1271	1335	1400	1128.8	1375.9	1203.8	1648	1256	1273	1455	1183	1340	1016	1087	1209	1224	1212	1346	1062	1074	1023	6
Os01g0722100	262	219	239	264.4	258.5	253.5	228	239	209	135.4	161.2	170.3	200.29	229.63	186.11	501.5	201	232.1	234.1	240.3	201.8	253.6	229.5	212.3	158.5	185.7	168.9	211	176	172	6
Os01g0213800	0	0	0	0.821	0	0	0	0	0	0	0	0	0	0	0	0	0	0	0	0	0.862	0	0.713	0	0	0	0	0	0	0	2
Os01g0546800	110	97.8	115	81.28	85.88	83.04	222	175	224	138.8	84.42	112	76.196	98.946	92.66	238.8	61.83	77.7	82.68	76.6	101.7	150.1	179.6	193.6	84.51	60.34	96.49	65.7	66.9	85	18
Os01g0713900	16.3	18.6	8.62	7.389	4.52	12.98	5.48	1.27	0.69	2.587	7.675	2.822	5.4426	5.6007	3.9598	11.94	19.58	9.324	4.645	0.806	2.587	22.92	9.977	6.5	6.338	8.486	8.772	11.1	22	15	8
Os01g0708100	13.3	27.9	20.5	16.42	21.7	26.82	7.83	10.8	13.8	19.83	21.93	7.527	14.151	14.002	7.9197	11.94	10.31	14.5	18.58	8.869	10.35	15.53	15.68	13	15.85	4.714	8.772	13.9	26.8	11.7	2
Os01g0510701	45.9	36.3	34.5	32.02	56.95	51.04	61.1	57.6	45.6	37.94	44.95	58.33	38.098	49.473	31.679	35.82	39.16	34.19	57.6	64.5	69.84	48.06	47.75	48.39	49.65	43.37	46.05	38.9	56.4	49.2	15
Os01g0131600	10.2	6.52	11.9	6.568	3.616	10.38	15.7	5.69	15.2	7.76	8.771	9.408	7.6196	13.068	7.9197	23.88	17.52	12.43	17.65	12.9	12.93	5.915	9.977	7.222	10.56	16.03	28.51	4.63	4.78	9.16	11
Os01g0594900	0	0	0	0	0	0	0	0	0	0	0	0	0	0	0	0	0	0	0	0	0	0.739	0	0	0	0	0	0	0	0	9
Os01g0126401	36.7	49.4	38.8	24.63	21.7	31.14	14.9	6.96	6.22	81.91	60.3	51.75	48.983	48.539	38.014	155.2	42.25	47.66	26.94	29.03	44.84	25.14	14.97	15.89	60.21	46.2	46.05	91.6	68.9	57.5	13
Os01g0723400	787	870	828	2803	2592	2418	1384	1161	1357	1077	884.8	1064	878.44	792.5	860.87	919.5	733.7	658.9	2859	2856	2675	924.2	1154	1218	876.8	921.2	945.1	731	791	665	10
Os01g0185500	164	186	147	159.3	166.3	168.7	129	125	117	123.3	149.1	120.4	153.48	158.69	148.89	250.8	203	181.3	173.7	201.6	182.8	152.3	116.9	138.7	173.2	132.9	186.4	215	192	195	13
Os01g0102900	3609	6814	3824	#####	#####	95203	####	####	####	8919	12237	9077	4427	3203.6	3344.5	2627	2673	3018	75499	67319	79575	29677	28119	35428	6518	5820	5884	1564	1640	2443	10
Os01g0323000	3.06	0	1.08	7.389	10.85	19.03	7.05	10.8	4.15	6.035	1.096	3.763	2.177	8.4011	1.5839	0	5.153	0	0.929	4.031	6.036	1.479	1.425	2.889	0	0	8.772	0.93	3.83	0.83	2
Os01g0260100	18.4	6.52	6.47	36.12	59.66	44.98	21.9	14.5	8.29	4.311	0	1.882	8.7082	7.4676	3.1679	59.7	14.43	7.252	25.08	41.93	32.76	27.36	22.09	23.11	2.113	11.31	10.96	13	12.4	10	10
Os01g0739000	685	635	651	672.4	696.1	717.1	431	417	435	504.4	439.7	538.1	499.63	580.61	480.72	740.3	596.7	611.3	658.6	567.6	691.5	417	452.5	454.3	468	558.2	598.7	546	580	497	16
Os01g0205500	94.8	49.4	66.8	64.86	74.13	94.29	83	70.2	98.1	81.91	81.13	108.2	64.223	84.011	61.773	143.3	70.08	56.98	112.4	83.85	106.1	57.67	67.7	79.45	65.49	90.51	83.33	39.8	51.6	45.8	6
Os01g0509750	0	0	0	0	0	0	0	0	0	0	0	0	0	0	0	0	0	0	0	0	0	0	0	0.722	0	0	0	0	0	0	9
Os01g0822200	788	707	656	384.2	366.1	353.8	206	282	238	590.6	575.6	510.8	482.21	488.2	601.89	907.5	481.3	554.3	434.8	341.9	403.5	270.6	269.4	278.1	630.6	551.6	646.9	543	627	510	13
Os01g0966100	882	905	931	848.1	760.2	789.8	537	676	612	901.8	911.1	789.3	1095.1	984.79	1065.2	1552	952.2	931.4	723.7	914.3	773.4	796.3	736.9	781.5	935.9	993.8	929.8	1151	1184	1057	13
Os01g0518400	2.04	9.31	6.47	4.926	9.04	10.38	3.13	1.27	2.76	6.897	2.193	0.941	2.177	3.7338	3.9598	11.94	2.061	11.4	2.787	4.031	4.311	11.09	10.69	10.83	5.282	3.771	2.193	12	8.61	3.33	8
Os01g0355800	45.9	52.2	38.8	44.33	41.58	32.01	90	43.6	81.5	78.46	80.04	47.98	58.78	31.737	73.653	59.7	21.64	30.05	36.23	56.44	48.28	34.01	49.88	41.17	41.2	34.89	41.67	52.7	58.3	52.5	5
Os01g0917400	2031	1718	1997	559.9	568.6	634.9	1390	1433	1578	2221	2321	2991	2432.8	2338.3	2341.1	4454	2475	2214	782.2	670	682.9	1242	1056	1510	2672	3075	2680	2083	2351	2318	13
Os01g0869800	####	####	####	9186	8422	10197	9391	9280	7866	33805	33617	45508	40262	40662	35954	73281	42435	32138	28203	21545	26861	4282	4607	7816	32426	34569	32495	####	####	####	13
Os01g0559750	738	509	691	153.5	121.1	410	128	67	137	381.9	300.4	289.8	231.85	314.57	243.93	3857	2592	1758	498.8	291.9	638	85.77	101.2	169.7	632.8	1022	1985	853	977	325	13
Os01g0564950	0	0	0	0	0	0	0	0	0	0	0	0	0	0	0	0	0	0	0	0	0	0	0	0	0	0	2.193	0	0	0	11
Os01g0351100	95.8	110	99.1	102.6	111.2	90.83	102	111	133	68.11	96.48	63.03	82.728	97.079	114.04	191.1	69.05	70.45	64.1	120.9	94.84	113.1	120.4	96.78	90.85	81.08	37.28	99.9	90.9	99.1	6
Os01g0123900	585	306	434	5180	774.7	7176	1546	1385	6773	2467	2034	1008	855.58	346.31	378.56	214.9	312.3	273.5	5696	2578	5707	3934	3602	3737	778.5	890	3265	214	1002	432	12
Os01g0772100	3.06	0	1.08	0.821	1.808	3.46	2.35	2.53	2.76	1.724	0	4.704	0	0	0.792	0	2.061	0	0.929	1.613	0.862	0	4.276	2.167	2.113	3.771	0	0	0.96	0	15
Os01g0850550	1.02	23.3	26.9	18.06	39.77	10.38	35.2	118	3.46	90.53	28.51	15.99	31.567	18.669	69.693	0	5.153	8.288	20.44	24.19	0.862	21.44	53.45	1.444	38.03	1.886	4.386	10.2	37.3	24.2	7
Os01g0346900	21.4	16.8	19.4	0	0	0.865	0	0	0	5.173	20.83	9.408	1.0885	1.8669	4.7518	23.88	8.244	9.324	0	1.613	0	0	0	0	28.52	29.23	15.35	6.48	1.91	6.66	3
Os01g0147700	322	374	306	647.7	684.3	545	390	414	401	321.6	334.4	265.3	317.85	345.38	292.24	668.7	271	283.9	430.1	464.4	385.4	315	427.6	371.2	258.8	279.1	322.4	273	279	293	10
Os01g0546400	157	135	112	13.96	13.56	30.28	18	10.8	12.4	106	97.58	188.2	78.373	133.48	98.204	131.4	77.29	116	12.08	11.29	18.11	15.53	7.126	11.56	63.38	83.91	103.1	105	80.3	82.5	3
Os01g0911700	0	0	0	0	0	0	0	0	0	0	0	0	0	0	0	0	0	0	1.858	0	0	0	0	0	0	0	0	0	0	0	16
Os01g0607000	1.02	0	1.08	0	1.808	0	0	0	0	0	0	0	0	0	0	0	1.031	0	0	0	1.724	0	0.713	0	0	0	0	0	0	0	19
Os01g0896200	17.3	6.52	6.47	9.031	9.944	5.19	4.7	0.63	3.46	16.38	15.35	4.704	3.2656	7.4676	4.7518	0	7.214	1.036	16.72	1.613	7.76	9.612	6.414	6.5	8.451	3.771	13.16	7.4	0.96	3.33	7
Os01g0683700	3.06	4.66	3.23	0	1.808	2.595	17.2	9.49	6.22	7.76	2.193	5.645	5.4426	0.9335	7.9197	0	3.092	4.144	4.645	2.419	9.484	4.436	2.138	9.389	4.225	6.6	8.772	9.25	3.83	2.5	5
Os01g0814100	0	0	0	0	0	0	0	0	0	0	0	0.941	0	0	0	0	0	0	0	0	0	0.739	0	0	0	0	0	0.93	0	0	20
Os01g0256400	387	373	407	325.9	273	247.4	536	450	446	548.4	519.7	604	450.65	452.72	463.3	764.2	498.8	394.7	449.6	379	392.3	357.1	327.8	394.3	489.1	529.9	622.8	436	405	395	13
Os01g0170800	30.6	33.5	31.2	78.81	96.72	67.47	42.3	44.9	51.8	33.63	47.14	26.34	27.213	29.87	33.263	83.59	39.16	36.26	45.52	84.66	47.42	80.59	60.57	42.61	47.54	42.43	15.35	46.3	66	33.3	2
Os01g0259933	57.1	48.4	45.3	94.41	107.6	122	38.4	51.2	47	42.25	50.43	79.97	63.134	71.876	65.733	47.76	47.4	65.27	64.1	85.47	95.71	87.99	60.57	49.83	50.71	67.88	89.91	70.3	80.3	45	2
Os01g0727840	5.1	26.1	9.7	9.031	12.66	8.65	6.26	0	2.07	1.724	4.386	10.35	7.6196	10.268	3.1679	35.82	11.34	9.324	1.858	7.257	6.036	27.36	6.414	42.61	3.169	4.714	13.16	11.1	1.91	16.7	9
Os01g0957500	0	0	0	0	0	0	0	0	0	0	0	0	0	0	0	0	0	0	0	0	0	1.479	0	0.722	0	0	0	0	0	0	9
Os01g0316500	260	216	286	224.1	184.4	201.6	305	239	303	347.5	335.5	378.2	247.09	317.37	323.91	441.8	279.3	267.3	219.2	258.8	228.5	247	221.6	243.4	346.5	388.5	342.1	321	260	262	13
Os01g0355900	553	512	513	560.7	531.5	538.1	736	660	716	707	651.3	655.8	680.32	599.28	674.76	1469	574	575	510	542.6	651.8	631.4	581.5	650	793.3	694.9	765.3	646	616	644	6
Os01g0201700	5.1	4.66	4.31	3.284	0.904	2.595	0	0	0	1.724	1.096	1.882	0	3.7338	0.792	0	3.092	2.072	1.858	0	1.724	1.479	0	1.444	2.113	0.943	0	3.7	0	3.33	14
Os01g0367700	333	356	296	302.1	351.6	404	427	495	560	432.8	434.2	458.2	493.1	392.98	510.82	322.4	377.2	367.8	308.4	311.2	407.8	303.1	434.7	495.5	331.7	348.9	357.4	357	322	339	5
Os01g0901000	64.2	72.7	60.3	83.74	93.11	85.64	53.2	49.3	51.8	81.05	71.27	77.15	68.577	70.009	79.197	95.53	63.89	71.49	75.25	56.44	51.73	45.84	68.41	48.39	61.27	61.28	59.21	56.4	72.7	65	2
Os01g0588100	0	0	0	0	0	0	0	0.63	1.38	0	0	0	0	0	0.792	0	0	0	0	0	0	0	0	0	0	0	0	0	0	0	18
Os01g0748100	88.7	76.4	92.7	66.5	59.66	89.1	84.6	69.6	50.4	86.22	75.65	50.8	45.718	85.878	83.156	119.4	59.77	90.14	48.31	80.63	66.39	70.24	81.24	59.95	76.06	54.68	65.79	74.9	93.7	108	13
Os01g0185900	0	2.79	1.08	0	0.904	0	1.57	0.63	0	0	0	0	1.0885	0	3.1679	0	0	1.036	0	0	0	4.436	6.414	6.5	2.113	0	0	0	1.91	0.83	17
Os01g0908800	103	134	136	111.7	113.9	130.6	127	128	137	133.6	116.2	142.1	152.39	117.61	131.47	143.3	126.8	132.6	91.04	142.7	101.7	146.4	112.6	146.6	147.9	116	114	121	137	128	5
Os01g0906200	395	491	462	445	490.9	439.4	503	515	562	490.6	436.4	504.3	424.52	413.52	435.58	382.1	447.3	396.8	445	427.3	414.7	425.9	394.8	454.3	387.7	361.1	473.7	370	360	396	15
Os01g0176100	39.8	30.7	28	26.27	15.37	16.44	38.4	37.3	53.9	26.73	13.16	23.52	27.213	18.669	34.055	35.82	13.4	20.72	25.08	19.35	9.484	68.76	73.4	68.61	31.69	17.91	24.12	32.4	33.5	19.2	17
Os01g0802200	8.16	8.38	16.2	3.284	5.424	0.865	4.7	7.59	9.67	6.897	1.096	6.586	5.4426	2.8004	5.5438	11.94	11.34	10.36	2.787	1.613	4.311	5.915	13.54	7.945	2.113	6.6	6.579	4.63	2.87	10.8	14
Os01g0702500	5.1	8.38	2.16	0.821	0	1.73	0	0.63	0	4.311	0	9.408	2.177	3.7338	1.5839	47.76	11.34	9.324	0	0	0	0	0	0	1.056	1.886	2.193	13	12.4	5.83	13
Os01g0856900	803	663	721	261.9	255.8	205.9	539	698	581	727.7	862.9	473.2	744.55	787.83	890.96	1170	752.3	733.5	274	266.1	282.8	361.6	392.7	315.6	605.3	592.1	552.6	713	824	775	13
Os01g0254200	592	607	562	435.9	499	556.2	855	711	943	619.9	480.2	509	424.52	484.46	463.3	597	441.1	461	527.6	474.1	401.8	675.8	701.9	777.9	465.9	439.4	548.2	384	400	390	18
Os01g0776300	116	112	98.1	114.1	92.2	123.7	124	117	104	86.22	103.1	116.7	136.06	110.15	113.25	119.4	98.93	84.96	132.8	183.8	149.2	122	116.9	139.4	67.61	77.31	78.95	124	90.9	117	16
Os01g0876400	505	363	487	261.9	240.5	263	159	219	229	394.9	335.5	291.7	343.97	370.58	331.04	465.7	355.5	315	237.8	215.3	195.7	195.9	204.5	226.8	287.3	290.4	296	342	265	272	13
Os01g0299150	1.02	0	0	4.926	4.52	3.46	3.91	3.16	1.38	0.862	0	0	1.0885	0	0	0	1.031	0	1.858	0	2.587	4.436	2.851	2.889	0	0	0	0	0.96	1.67	12
Os01g0623500	509	368	467	32.84	53.33	51.9	98.6	93.6	106	442.3	332.2	305.8	305.87	224.96	273.23	370.2	305	414.4	67.81	45.15	31.9	91.68	103.3	67.89	343.3	295.1	280.7	328	318	313	3
Os01g0890001	15.3	11.2	8.62	13.96	9.04	9.516	8.61	5.06	7.6	14.66	15.35	19.76	4.3541	12.135	3.9598	35.82	12.37	16.58	28.8	14.51	14.66	7.394	10.69	8.667	10.56	16.03	19.74	6.48	12.4	5.83	6
Os01g0696600	0	1.86	0	0	0	0	0	0	0	0	0	0	2.177	0	0.792	0	0	0	0	0	0	0	0	0	2.113	0	2.193	1.85	0	0	4
Os01g0611100	104	74.5	75.4	62.39	66.89	70.07	63.4	58.8	60.8	111.2	98.68	114.8	92.524	107.35	101.37	95.53	63.89	74.59	89.18	64.5	96.57	39.19	51.31	48.39	78.17	114.1	116.2	77.7	68.9	52.5	20
Os01g0692000	13.3	11.2	17.2	31.2	32.54	13.84	11.7	6.96	13.8	20.69	29.6	5.645	20.682	15.869	15.839	11.94	15.46	7.252	13.01	28.22	18.97	5.176	19.95	0.722	10.56	7.543	17.54	14.8	18.2	2.5	2
Os01g0848550	0	0	0	0	0	0	0	0.63	0	0	0	0	0	0	0	0	0	0	0	0	0	0	0	1.444	0	0	0	0	0	0	9
Os01g0698900	0	0	0	0.821	0	0	0	0	0	0	0	0	0	0	0	0	0	0	0	0	0	0	0.713	0	1.056	0.943	2.193	0	0	0	11
Os01g0170000	93.8	49.4	61.4	32.02	21.7	46.71	37.6	23.4	58.7	83.63	85.52	91.26	92.524	101.75	67.317	59.7	126.8	95.32	62.24	90.3	78.46	66.54	62	84.5	97.19	103.7	171	76.8	81.3	66.6	11
Os01g0675500	681	659	664	863.7	871.4	865.9	1026	1254	1127	579.4	671	560.7	659.64	505	663.67	919.5	715.2	618.5	776.6	865.1	800.1	983.4	972	1001	599	578.9	521.9	593	658	698	18
Os01g0824600	531	420	510	233.2	208.8	220.6	187	142	109	315.6	335.5	318.9	474.59	476.06	407.07	1242	578.1	480.7	263.8	341.1	219	136	139.7	143	370.8	414.9	300.4	717	688	565	13
Os01g0225550	2.04	0	2.16	2.463	5.424	8.65	12.5	0.63	11.1	1.724	1.096	1.882	2.177	2.8004	3.1679	11.94	3.092	5.18	14.86	12.9	31.9	8.133	9.977	13	7.395	7.543	10.96	2.78	7.65	0	16
Os01g0951400	204	222	232	208.5	203.4	203.3	350	308	355	325.9	279.6	302	236.21	239.9	213.83	382.1	255.6	223.8	191.4	179	178.5	332.7	320.7	333	284.2	284.7	276.3	229	223	205	5
Os01g0776400	101	113	115	202.8	169	207.6	302	293	245	133.6	94.29	88.44	102.32	129.75	110.08	214.9	119.5	97.39	158.9	200	182.8	278	275.1	250.6	114.1	106.5	100.9	113	122	104	18
Os01g0871700	0	0	0	0	0.904	0.865	0	0	0	0	0	0	0	0	0	0	0	0	0	1.613	0	0	0	0	0	0	0	0	0	0	10
Os01g0168200	659	765	718	330	289.3	342.6	406	421	495	777.7	791.6	791.2	619.37	644.08	552	1039	702.8	614.4	300.1	293.5	237.1	443.6	472.5	497.6	733.1	793.9	598.7	668	543	611	13
Os01g0666950	745	633	629	871.1	928.4	986.2	1071	928	1203	686.3	600.8	1095	784.82	815.84	559.13	823.9	672.9	632	969.8	801.4	1032	841.4	771.1	833.5	504.9	717.5	782.9	415	506	438	15
Os01g0171200	1.02	0	0	0.821	0	0	2.35	3.8	0	0	0	0	0	0	0	0	0	2.072	4.645	0	0.862	2.218	0.713	0.722	1.056	2.829	0	0	0	0	15
Os01g0554400	1.02	0	4.31	1.642	0	2.595	0	1.27	0	0.862	1.096	0	2.177	0	0	0	2.061	0	0	0	0	0.739	0.713	0	1.056	0	0	0	0	1.67	8
Os01g0862800	142	166	129	13.96	1.808	14.71	11	5.69	25.6	96.56	37.28	110.1	33.744	19.602	22.175	179.1	258.7	160.6	22.29	15.32	38.8	90.94	48.46	75.84	50.71	160.3	175.4	25	8.61	32.5	14
Os01g0891700	16.3	3.73	22.6	34.48	27.12	53.63	13.3	8.86	7.6	23.28	13.16	14.11	8.7082	14.002	15.047	11.94	1.031	7.252	54.81	56.44	39.66	22.18	22.8	11.56	19.01	18.86	50.44	23.1	22	12.5	16
Os01g0119800	0	1.86	0	0	0	0.865	0	0.63	0.69	0	0	0.941	0	0	3.1679	0	1.031	1.036	0	0.806	4.311	0	0	0.722	0	1.886	2.193	1.85	0	0.83	16
Os01g0806600	0	0	0	0	0	0	0	0	0	0	0	0	0	0	0	0	0	0	0	0	0	0	0	0.722	0	0	0	0	0	0	9
Os01g0159300	1034	1196	1012	816	819	799.3	759	873	938	611.3	699.5	522.2	753.26	781.3	814.14	1731	975.9	896.2	773.8	924	803.6	845.1	879.4	782.2	768	701.5	693	818	852	916	6
Os01g0966000	3745	3353	3825	3151	3298	3266	2145	2549	2291	2863	2975	2813	3538.8	3288.5	3496.5	7606	3427	4166	2580	3328	2681	3205	2994	2396	3455	2948	2822	4351	4025	4476	13
Os01g0630900	905	881	1004	339.1	273	500.9	530	268	491	1357	1227	1092	852.31	1105.2	902.05	1516	1236	1303	669.8	599.1	641.5	701.7	504.5	671.7	1291	1382	1629	1527	1329	1068	3
Os01g0603100	8.16	10.2	4.31	0	0.904	0.865	6.26	8.86	11.1	4.311	14.25	2.822	2.177	0	7.9197	0	9.275	4.144	0	5.644	0	8.133	6.414	2.889	10.56	8.486	4.386	0	1.91	1.67	5
Os01g0864566	2.04	0.93	0	0	0	0	0	0	0	0	1.096	0	1.0885	0.9335	0	0	0	0	0	0	0	0	2.138	0	0	0	0	0.93	0.96	0	19
Os01g0224100	12.2	6.52	3.23	25.45	28.02	21.63	3.91	0	4.15	1.724	1.096	1.882	5.4426	6.5342	3.9598	23.88	16.49	3.108	35.3	49.18	60.36	0.739	4.988	3.611	2.113	0	2.193	7.4	3.83	3.33	16
Os01g0956800	0	1.86	5.39	1.642	0.904	0.865	1.57	1.9	0.69	1.724	2.193	3.763	0	1.8669	1.5839	0	1.031	1.036	1.858	0.806	2.587	3.697	5.701	0	1.056	0	0	1.85	1.91	5.83	17
Os01g0644400	0	0	0	0	0	0	0	0	0	0	0	0	0	0	0	0	0	1.036	0	0	0	0	0	0	0	0	0	0	0	0	1
Os01g0618700	5.1	21.4	3.23	1.642	0.904	0.865	7.83	5.69	22.1	14.66	23.02	18.82	25.036	10.268	12.671	0	5.153	1.036	0.929	0.806	0	11.09	4.988	5.778	4.225	5.657	4.386	5.55	6.69	6.66	5
Os01g0232300	891	897	918	1469	1597	1381	863	1071	848	850.1	912.2	852.4	996	952.12	977.29	1111	719.3	886.8	1172	1302	1222	930.1	830.2	801	800.7	736.4	629.4	752	830	873	10

Os01g0957900	274	272	246	478.6	450.2	399.7	184	170	204	254.3	229.1	284.1	287.37	281.9	230.46	668.7	240.1	298.4	365.1	398.3	364.7	283.2	249.4	217.4	236.6	286.6	217.1	263	261	212	6
Os01g0756900	0	0	1.08	0	0	0	0	0	0	0	0	4.704	0	0	0	0	3.092	0	0	0	0	0	0	1.444	0	0	0	0	0	0	20
Os01g0607200	103	91.3	103	75.53	75.93	55.36	129	163	110	54.32	65.78	80.91	83.816	67.209	64.149	119.4	115.4	79.77	81.75	52.41	41.39	140.5	154.6	127.8	69.72	79.2	92.1	112	93.7	123	17
Os01g0359600	221	325	262	709.3	680.7	589.1	251	290	214	287.1	275.2	121.4	203.55	188.56	236.8	298.5	182.4	241.4	382.7	489.4	420.8	381.5	413.3	281	219.7	191.4	171	249	225	313	10
Os01g0571300	31.6	14	26.9	27.09	14.46	30.28	13.3	11.4	13.1	10.35	8.771	3.763	26.124	14.002	24.551	59.7	35.04	22.79	14.86	27.41	37.94	11.83	14.97	22.39	33.8	6.6	21.93	28.7	23.9	19.2	6
Os01g0899100	0	0	0	0	0	0.865	0	1.9	0	0	0	0	0	0	0	0	0	0	0	0	0	1.479	0	0.722	0	0	0	0	1.91	0	9
Os01g0944800	0	0	0	0	0	0	0	0	0	0.862	0	0	0	0	0	0	1.031	0	0.929	0	0	0	0	0	0	0	0	0	0	0	7
Os01g0144200	5.1	14.9	6.47	9.852	5.424	15.57	10.2	17.7	12.4	9.484	12.06	3.763	4.3541	6.5342	8.7116	11.94	2.061	7.252	5.574	16.13	14.66	9.612	8.552	7.945	3.169	6.6	6.579	6.48	14.3	6.66	12
Os01g0556400	110	131	121	96.05	160	132.4	70.5	103	79.5	93.98	98.68	62.09	126.27	70.942	104.54	119.4	99.96	94.28	60.38	66.92	83.64	125.7	111.2	109.8	95.07	60.34	76.75	103	88.9	137	8
Os01g0865600	823	894	888	823.4	839.8	862.5	889	868	838	850.1	848.6	843	984.02	938.12	960.65	1684	959.4	1067	797	871.6	861.4	956.8	875.1	822.6	873.6	957.9	881.6	1056	1056	1102	13
Os01g0730400	1.02	0	0	0	0	0	0	0	0	0	0	0	0	0	0	0	0	0	0	0	0	0	0	0	0	0	0	0	0	0	19
Os01g0511000	93.8	74.5	75.4	11.49	0.904	4.325	155	53.8	305	22.42	9.868	10.35	5.4426	4.6673	13.463	95.53	75.23	47.66	5.574	3.225	12.93	610.7	323.5	593	17.96	10.37	13.16	8.33	12.4	8.33	9
Os01g0873900	1114	1196	1221	1650	1721	1512	1156	1154	1037	1151	1213	950.2	1114.6	955.85	1311.5	1827	1123	1061	1650	1580	1363	1227	1262	1106	1170	1079	914.4	1048	995	976	10
Os01g0728100	2.04	0.93	3.23	0.821	0	0	0	0	0	0	2.193	0.941	1.0885	0.9335	2.3759	0	3.092	0	0	2.419	0	0	0	0	0	0	0	0	0	0.83	14
Os01g0258100	1.02	0.93	0	0	0	0	0	0	0	0	0	0	0	0	0.792	35.82	18.55	20.72	7.432	6.45	14.66	29.58	15.68	28.17	7.395	16.03	17.54	16.7	19.1	14.2	9
Os01g0581900	74.4	75.4	100	124	124.7	103.8	100	104	86.4	83.63	107.4	61.15	67.488	70.942	115.63	143.3	59.77	99.46	89.18	85.47	71.56	102	100.5	75.84	89.79	99.94	63.59	73.1	91.8	96.6	6
Os01g0744300	1023	996	935	825.9	857.9	817.5	1082	1015	1118	1140	1136	1232	1180	1111.7	1132.5	955.3	1041	1046	1034	998.2	1115	913.9	957.1	1015	1106	1160	1112	1072	925	963	20
Os01g0276200	162	121	130	604.2	637.3	647.1	212	160	193	100.9	71.27	75.27	99.055	134.42	94.244	143.3	87.6	117.1	546.2	528.9	539.7	151.6	215.2	191.4	49.65	52.8	133.8	64.8	66	59.2	10
Os01g0754300	0	0.93	0	0	0	0	0	0	0	0	0	0	0	0	0	0	0	1.036	0	0	0	0	0	0	0	0	0	0	0	0	14
Os01g0638000	146	78.2	149	9.031	11.75	7.785	2.35	3.16	6.91	10.35	10.96	6.586	87.082	34.538	45.142	191.1	59.77	30.05	1.858	6.45	17.24	1.479	9.977	6.5	6.338	0.943	26.32	23.1	28.7	15.8	13
Os01g0618100	1658	1767	1712	3038	2959	2977	583	467	513	1918	1709	1865	1717.7	1635.4	1374.1	2197	1258	1428	3310	2961	3601	317.9	325.7	444.9	1482	1342	1388	1177	1196	1418	16
Os01g0337500	3.06	6.52	3.23	8.21	4.52	2.595	5.48	6.33	4.84	4.311	6.578	3.763	1.0885	6.5342	9.5036	0	2.061	2.072	1.858	4.031	3.449	14.79	7.839	13	1.056	3.771	0	2.78	2.87	1.67	9
Os01g0635550	396	322	331	396.5	339.9	380.6	24.3	19.6	21.4	283.7	214.9	414.9	352.68	336.04	333.42	716.5	474	345	570.4	547.5	581.1	46.58	35.63	44.06	319	320.6	304.8	310	254	303	16
Os01g0810800	16.3	13	14	7.389	8.136	7.785	2.35	5.06	0	5.173	2.193	1.882	5.4426	5.6007	5.5438	35.82	4.122	7.252	4.645	3.225	5.173	11.09	6.414	8.667	6.338	9.428	6.579	3.7	5.74	6.66	6
Os01g0976700	354	457	414	654.3	626.5	551.9	633	633	543	526.8	586.6	557	486.57	476.99	526.66	573.2	453.4	470.4	606.6	643.4	614.8	505.7	533.8	560.5	571.5	604.4	607.4	542	569	494	15
Os01g0121300	31.6	28.9	32.3	76.35	65.09	82.18	158	85.4	122	50.87	64.69	57.39	37.01	46.673	37.222	71.65	37.1	32.12	143.1	115.3	118.1	102	84.8	84.5	53.87	69.77	57.02	55.5	37.3	49.2	12
Os01g0264000	43.8	79.2	48.5	2.463	6.328	4.325	28.2	29.1	23.5	100	83.33	111	58.78	98.946	95.828	95.53	82.44	75.63	4.645	0	2.587	18.48	15.68	8.667	89.79	86.74	89.91	87.9	66	91.6	3
Os01g0809300	3283	2976	3155	1699	1619	1704	3685	3082	3102	3060	2400	2941	2559.1	2594.1	2699	4036	2621	2632	2053	1852	1666	3361	3760	4814	2650	2669	3123	2529	2432	2290	9
Os01g0952500	1669	1350	1541	1474	1298	1445	229	235	340	1461	1413	1681	2057.3	1893	1866.7	3749	2331	1721	1659	1849	1887	825.9	493.9	743.9	1900	2362	1735	1945	1821	2003	13
Os01g0788400	0	0.93	0	0.821	0.904	0	0	1.9	3.46	1.724	0	1.882	3.2656	0.9335	0.792	0	0	1.036	0.929	0.806	0	0.739	4.276	2.889	2.113	2.829	8.772	0	0	0.83	11
Os01g0885500	70.3	82	80.8	171.6	151	123.7	80.6	96.8	109	83.63	76.75	56.45	75.108	85.878	89.492	310.5	85.53	97.39	136.6	162.9	115.5	80.59	87.65	85.22	88.73	85.8	39.47	80.5	102	98.3	6
Os01g0672166	11.2	10.2	8.62	22.99	28.93	16.44	4.7	6.33	7.6	6.035	10.96	3.763	18.505	7.4676	9.5036	0	9.275	11.4	11.15	18.54	15.52	8.873	7.126	10.83	11.62	13.2	0	13.9	18.2	15	2
Os01g0513500	115	95	106	133.8	140.1	144.5	86.9	70.2	88.5	115.5	121.7	126.1	92.524	134.42	100.58	322.4	126.8	144	158.9	183.8	177.6	116.8	94.07	70.06	139.4	117.9	173.2	133	152	133	6
Os01g0716300	13.3	9.31	3.23	50.08	37.06	44.12	30.5	29.7	32.5	9.484	8.771	26.34	5.4426	7.4676	10.296	11.94	22.67	11.4	70.6	55.63	50.01	31.05	28.51	35.39	10.56	13.2	2.193	15.7	15.3	10	10
Os01g0273100	4.08	0.93	0	0	0	0	0	0	0	0	0	0	0	0	0	0	0	0	0	0	0	0	0.713	0	0	0	8.772	0	0	0	11
Os01g0646800	512	506	444	567.3	557.7	471.5	258	318	236	261.2	334.4	265.3	374.45	347.24	395.19	728.4	469.9	508.7	410.6	508	395.8	551.6	469.6	314.2	330.6	441.2	245.6	623	540	607	8
Os01g0736500	14.3	14.9	17.2	13.14	13.56	19.9	11.7	9.49	4.15	16.38	6.578	4.704	8.7082	12.135	10.296	11.94	14.43	9.324	21.37	18.54	12.07	8.873	5.701	9.389	6.338	6.6	8.772	11.1	6.69	4.17	16
Os01g0656900	69.3	78.2	74.4	77.17	70.51	86.5	60.3	43.6	40.1	45.7	58.11	56.45	57.692	45.739	50.686	107.5	65.95	78.74	104	76.6	95.71	45.1	69.84	58.5	52.82	78.26	142.5	64.8	50.7	85.8	11
Os01g0971200	1039	1078	1205	793.1	912.1	827	970	1128	998	844.9	826.7	601.2	867.55	924.12	926.6	1015	897.6	837.1	605.7	578.1	595.8	1013	982	1018	813.4	673.2	635.9	720	694	871	14
Os01g0844900	26.5	10.2	11.9	0	2.712	5.19	8.61	4.43	2.76	7.76	4.386	8.467	1.0885	10.268	1.5839	0	8.244	14.5	0.929	0	1.724	10.35	13.54	6.5	5.282	3.771	6.579	5.55	8.61	8.33	19
Os01g0966700	84.6	117	90.5	88.67	96.72	103.8	242	264	219	171.6	165.6	187.2	156.75	150.29	163.14	95.53	138.1	114	160.7	124.2	147.4	122.7	170.3	156.7	146.8	155.6	173.2	117	105	142	5
Os01g0109300	0	0	0	0.821	0	0	0	0.63	2.07	0	0	0	0	0	2.3759	0	0	0	0.929	0	0	0.739	0	0	0	0	0	0	0	0	4
Os01g0197450	1.02	4.66	1.08	9.852	21.7	14.71	1.57	8.86	9.67	0.862	0	0.941	1.0885	0	5.5438	0	0	0	9.29	10.48	1.724	3.697	2.851	5.056	0	0.943	6.579	1.85	5.74	5	2
Os01g0241000	68.3	48.4	58.2	36.94	26.22	34.6	78.3	75.9	122	62.94	100.9	60.21	51.16	41.072	67.317	143.3	50.5	30.05	39.02	25.8	29.32	65.81	66.99	51.28	71.83	58.46	118.4	36.1	73.6	55	6
Os01g0796000	118	78.2	107	44.33	45.2	34.6	112	156	243	37.07	10.96	10.35	10.885	22.403	16.631	35.82	82.44	78.74	53.88	63.7	52.6	147.1	141.8	251.3	29.58	12.26	17.54	15.7	19.1	27.5	18
Os01g0357200	0	0.93	0	2.463	4.52	1.73	7.05	5.06	3.46	0	2.193	0.941	0	0.9335	0.792	0	0	0	1.858	0	0.862	2.958	2.138	2.889	1.056	1.886	0	0	0	0	18
Os01g0748600	829	724	810	456.5	439.3	425.6	504	600	542	618.2	665.5	626.6	595.42	631.95	676.34	943.3	779.1	777	489.6	462.8	460.4	591.5	642.1	642.8	661.3	746.7	739	648	611	692	13
Os01g0140400	9.18	13	6.47	12.31	11.75	15.57	7.05	5.06	8.98	2.587	1.096	13.17	1.0885	2.8004	2.3759	0	11.34	3.108	9.29	6.45	6.036	8.133	5.701	15.89	4.225	1.886	2.193	7.4	1.91	9.16	2
Os01g0323950	1.02	0	0	0	0	0	0	0	0	0	0	0	1.0885	0.9335	0	0	0	0	0	0	0	0	0	0	0	0	0	0	0.96	0	4
Os01g0631700	50	42.8	28	44.33	47.01	22.49	117	163	106	13.79	14.25	36.69	15.239	20.536	15.839	0	58.74	27.97	24.15	15.32	18.11	40.67	38.48	97.5	25.35	29.23	24.12	18.5	17.2	15.8	18
Os01g0274800	0	0.93	2.16	1.642	3.616	0	10.2	1.27	20.7	0	0	1.882	0	0.9335	1.5839	11.94	0	0	0	0	0	56.93	39.91	36.11	2.113	1.886	0	0	0.96	2.5	9
Os01g0617800	180	197	172	227.4	209.7	205.9	233	226	231	210.4	197.4	228.6	204.64	217.49	197.2	513.5	194.8	218.6	290.8	305.6	279.4	241.8	188.1	234	177.5	245.1	184.2	192	221	195	6
Os01g0301300	20.4	16.8	12.9	20.52	23.5	41.52	42.3	43.6	41.5	16.38	44.95	35.75	41.364	33.604	36.43	71.65	36.07	27.97	36.23	37.9	42.25	34.01	35.63	26	29.58	40.54	59.21	36.1	37.3	44.2	6
Os01g0937500	0	0	0	0	0	0	0	0.63	0.69	0	0	0	0	0	0	0	0	0	0	0	0.862	0	0	0	0	0	0	0	0	0	15
Os01g0222600	249	568	214	1119	1317	1712	3869	3899	4007	613	679.8	1040	706.45	554.47	684.26	143.3	295.8	499.4	601	370.1	658.7	3105	2794	3226	550.4	439.4	609.6	268	151	274	18
Os01g0977600	1426	1646	1503	1380	1379	1419	1227	1141	1244	1204	1288	1283	1297.5	1325.5	1561.8	1875	1320	1588	1025	1016	1071	1599	1407	1268	1375	1374	1112	1629	1504	1711	8
Os01g0259400	7.14	16.8	22.6	17.24	16.27	17.3	17.2	14.5	10.4	12.93	14.25	29.17	21.77	26.137	12.671	47.76	18.55	22.79	5.574	15.32	14.66	22.92	21.38	7.945	23.24	12.26	28.51	13	22	19.2	13
Os01g0890300	45.9	72.7	46.3	30.38	31.64	28.55	2.35	14.5	6.91	43.97	42.76	12.23	43.541	61.608	64.149	155.2	79.35	94.28	16.72	35.48	16.38	34.01	52.02	15.17	83.45	55.63	32.89	90.7	75.5	87.5	13
Os01g0607600	0	0	0	0	0	0	0	0	0	0	0	0	1.0885	0	0	0	0	0	0	0	0	0	0	0	0	0	0	0	0	0	4
Os01g0158000	336	384	350	391.6	402.3	446.4	431	407	469	353.5	346.5	366	427.79	378.05	416.57	680.6	339	384.4	414.3	424.1	400.1	497.6	442.5	430.5	357.1	383.7	390.3	335	388	392	6
Os01g0252300	0	0	0	0	0	0	0	0	0	0	0	0	0	0	0.792	0	0	0	0.929	0	0	0	0	0	0	0	0	0	0	0	4
Os01g0522950	22.4	13	10.8	6.568	3.616	0.865	16.4	2.53	6.91	11.21	3.289	22.58	5.4426	12.135	7.1277	0	10.31	6.216	0.929	0.806	0.862	12.57	4.276	23.83	8.451	10.37	13.16	3.7	2.87	2.5	19
Os01g0846600	300	292	254	265.2	273.9	287.2	148	144	141	233.7	241.2	206	317.85	243.63	285.11	465.7	304	219.6	268.5	259.6	254.4	111.6	113.3	130	264.1	248	252.2	316	267	262	13
Os01g0722800	374	380	406	359.6	430.3	447.2	591	555	682	417.3	411.1	470.4	375.54	383.65	365.89	238.8	308.1	354.3	405	409.6	351.8	396.3	394.8	499.8	410.9	374.3	357.4	333	342	328	18
Os01g0894700	768	1081	920	899.8	1003	1039	2628	2472	2733	989.8	1105	1229	801.15	669.28	766.62	859.7	702.8	708.6	892.7	928.8	989.8	2121	1804	2139	831.4	923	925.4	472	464	569	18
Os01g0667900	652	1236	630	104.3	108.5	80.45	76.7	61.4	104	169	197.4	179.7	316.76	264.17	289.86	1218	689.4	483.8	125.4	118.5	131.1	38.45	52.73	98.23	288.4	235.7	256.6	241	230	255	13
Os01g0542700	360	441	405	110	99.44	121.1	326	295	334	471.6	536.1	344.3	409.28	477.93	599.52	943.3	462.7	411.3	134.7	164.5	106.9	371.2	511.7	464.4	813.4	590.2	554.8	753	712	816	13
Os01g0736000	1.02	0	0	0.821	5.424	1.73	0	3.16	4.15	0	2.193	5.645	3.2656	1.8669	6.3357	0	3.092	0	1.858	0	0	4.436	0	4.333	2.113	0.943	2.193	1.85	2.87	0	4
Os01g0618200	523	463	597	745.4	813.6	771.6	846	741	777	389.7	378.3	461	448.47	373.38	435.58	680.6	554.4	518	840.7	921.6	909.6	692.1	563	710	388.7	397.9	383.8	415	483	463	12
Os01g0141600	539	563	606	949	1077	897.9	869	1057	967	461.3	516.4	499.6	829.45	614.21	600.31	1349	618.3	738.7	1026	1213	1036	889.5	858	858	682.4	554.4	381.6	749	712	755	12
Os01g0858700	127	137	120	288.2	317.3	295.8	205	183	180	131.1	96.48	88.44	140.42	126.95	122.75	370.2	95.84	111.9	224.8	290.3	256.9	219.6	225.9	183.5	132	113.1	105.3	136	113	131	10
Os01g0290000	2990	3388	3180	12856	13248	13075	6328	6009	5873	3229	3004	3422	3187.2	3123.3	2859	5302	3409	3208	12697	13363	13104	5773	5443	5651	2939	2896	2774	3096	2963	3219	10
Os01g0916000	3.06	3.73	3.23	6.568	8.136	1.73	0.78	6.33	0.69	0.862	2.193	8.467	3.2656	4.6673	2.3759	11.94	4.122	1.036	6.503	1.613	7.76	0.739	17.82	2.167	0	5.657	15.35	1.85	7.65	2.5	11
Os01g0618500	211	254	192	161.7	186.2	210.2	233	214	243	175.9	212.7	220.2	186.14	198.83	203.54	262.7	196.8	210.3	196	211.2	207.8	273.6	279.4	254.2	218.7	227.2	232.4	222	223	226	9
Os01g0510200	78.5	23.3	5.39	19.7	8.136	65.74	2.35	10.1	3.46	7.76	2.193	39.51	1.0885	13.068	3.1679	0	75.23	18.65	39.02	53.21	150	2.218	10.69	16.61	0	94.28	48.24	4.63	23.9	0	16
Os01g0310800	327	327	331	384.2	388.7	366.8	270	310	319	262.1	319.1	232.4	291.72	266.97	282.73	620.9	285.5	268.3	349.3	348.3	374.2	321.6	326.4	286	331.7	252.7	230.3	327	290	285	6
Os01g0738900	37.7	37.3	20.5	31.2	30.73	44.12	60.3	50.6	72.6	31.9	39.47	30.11	53.337	38.272	49.894	59.7	36.07	31.08	45.52	40.31	44.84	70.98	53.45	86.67	49.65	39.6	67.98	38.9	34.4	37.5	9
Os01g0835700	27.5	35.4	43.1	18.88	24.41	39.79	44.6	43	51.1	7.76	23.02	16.93	22.859	16.802	25.343	59.7	45.34	36.26	25.08	28.22	36.21	25.14	28.51	38.28	29.58	18.86	24.12	15.7	23.9	22.5	6
Os01g0875700	56.1	48.4	47.4	13.96	16.27	20.76	43.1	31.6	21.4	48.28	39.47	87.5	44.629	70.942	41.182	11.94	18.55	27.97	14.86	12.9	10.35	19.22	21.38	32.5	31.69	27.34	30.7	13.9	27.7	24.2	20
Os01g0612700	151	203	145	250.4	224.2	240.5	225	156	164	123.3	122.8	104.4	153.48	138.15	130.67	238.8	141.2	135.7	190.4	194.3	183.7	205.5	179.6	187.8	110.9	122.6	120.6	128	142	142	10
Os01g0953000	7.14	2.79	1.08	4.105	5.424	6.92	4.7	8.22	15.2	9.484	6.578	4.704	4.3541	1.8669	4.7518	0	2.061	7.252	2.787	9.675	8.622	9.612	18.53	7.222	7.395	5.657	4.386	11.1	6.69	2.5	17
Os01g0843432	1.02	5.59	8.62	2.463	6.328	0	20.4	21.5	13.8	9.484	8.771	6.586	6.5311	1.8669	3.9598	0	3.092	6.216	0	1.613	0.862	4.436	1.425	6.5	5.282	6.6	2.193	3.7	2.87	2.5	5
Os01g0112100	76.5	44.7	59.3	82.1	74.13	82.18	25.8	15.2	24.9	37.94	31.8	45.16	45.718	53.207	37.222	71.65	54.62	76.67	56.67	45.15	54.32	31.05	24.94	23.11	61.27	63.17	54.82	48.1	58.3	56.7	8
Os01g0932950	0	0	0	0	0	0	0.78	0	0	0	0	0	2.177	0	0	0	0	0	0	0.806	0	0	0	0	0	0	0	0.93	0	0	4
Os01g0848900	316	183	297	135.5	111.2	165.2	40.7	38	50.4	326.8	370.6	268.1	238.39	365.91	323.12	477.6	350.4	276.6	235	191.9	256.1	48.06	66.28	52.72	502.8	319.6	296	400	296	290	3
Os01g0718300	270	271	242	155.2	157.3	141	171	187	150	176.7	172.1	144.9	165.46	146.55	179.78	358.2	229.8	262.1	170	186.3	181.9	173.8	186.7	192.1	150	195.2	173.2	171	206	172	13
Os01g0723700	63.2	43.8	51.7	65.68	46.1	55.36	14.1	18.3	38.7	27.59	27.41	35.75	39.187	48.539	27.719	95.53	43.28	47.66	18.58	40.31	41.39	30.31	24.94	21.67	39.09	38.66	28.51	42.6	44.9	30	13
Os01g0857700	877	762	788	694.5	658.1	697.2	1047	915	1084	864.8	1045	1038	892.59	1057.6	936.9	1266	939.8	828.8	789.6	678.9	720.8	944.9	830.2	1010	916.9	1011	1048	864	843	796	5
Os01g0606000	1.02	2.79	0	0	0.904	0.865	20.4	10.1	3.46	10.35	18.64	22.58	9.7967	8.4011	0.792	0	25.76	22.79	14.86	8.063	18.11	7.394	9.977	33.22	26.41	73.54	83.33	13	38.3	9.16	11
Os01g0920200	888	884	872	894.9	1157	892.7	1082	1087	1079	867.4	961.5	922.9	953.54	1260.2	1056.5	1051	893.5	950	950.3	839.3	839.8	869.5	910	912.9	957.1	807.1	984.6	905	840	922	4
Os01g0578500	98.9	93.1	97	158.4	134.7	123.7	124	124	125	106.9	78.94	107.3	111.03	108.28	110.08	107.5	68.02	97.39	148.6	175.8	150	114.6	116.2	120.6	87.68	91.46	92.1	87	76.5	83.3	10
Os01g0925300	1.02	0	2.16	0.821	0	2.595	0	0.63	0	0	0	3.763	0	0	0	0	1.031	2.072	1.858	1.613	0.862	0.739	1.425	0	0	1.886	0	0.93	3.83	0	16
Os01g0369700	524	414	476	252	273	207.6	94.7	121	198	262.1	273	229.6	543.17	510.6	566.26	322.4	378.2	357.4	237.8	232.2	188	164.1	243	185.6	328.5	282.9	256.6	457	404	494	3
Os01g0811400	181	176	163	194.6	164.5	199.8	215	201	176	238	203.9	177.8	155.66	168.02	195.62	310.5	175.2	189.6	196.9	178.2	182.8	161.9	176	157.4	200.7	215.9	252.2	219	204	202	6
Os01g0138100	1.02	3.73	0	4.105	4.52	14.71	0.78	5.06	5.53	1.724	3.289	0.941	0	2.8004	0	0	1.031	0	1.858	3.225	0	2.218	2.851	0.722	4.225	1.886	0	0	0	0	2
Os01g0766000	3.06	0	1.08	0	0	0	0	0	0	0	0	0	0	1.8669	0	0	0	0	0	0	0	1.479	0	0	2.113	1.886	0	0.93	0.96	0	19
Os01g0775400	12.2	7.45	5.39	22.17	10.85	43.25	3.91	12.7	9.67	10.35	1.096	3.763	4.3541	5.6007	10.296	23.88	7.214	1.036	12.08	10.48	12.93	34.01	27.08	17.33	3.169	5.657	48.24	11.1	13.4	10	11
Os01g0940600	293	338	328	712.6	823.5	719.7	1023	1006	976	330.2	446.2	555.1	439.76	392.98	300.16	597	244.2	326.4	847.2	993.3	939.8	635.9	692.7	834.2	365.5	347.9	361.8	202	243	252	12
Os01g0511200	272	269	237	422	478.2	466.3	297	311	314	265.6	264.2	287.9	316.76	336.31	277.98	597	250.4	296.3	467.3	458	529.4	274.3	287.9	257.1	256.7	323.4	261	257	258	229	10
Os01g0268300	1890	1552	1963	2534	2882	2803	1891	1749	1569	1216	1205	1489	2117.2	2134.8	1549.9	4144	2427	2251	2953	3048	3150	1272	1252	1549	1420	1550	1428	1808	1987	1736	6
Os01g0575000	2761	2833	2655	2300	2328	2394	2591	2649	2569	2442	2288	2607	2402.4	2413	2541.4	3475	2636	2582	2112	1904	2006	2629	2615	2575	2775	2569	2546	2516	2299	2729	13
Os01g0686000	62.2	45.6	52.8	13.96	9.944	8.65	12.5	29.1	4.84	101.7	87.71	128	44.629	37.338	31.679	107.5	129.8	66.31	17.65	16.13	15.52	14.79	32.78	23.83	79.23	82.03	114	31.5	41.1	53.3	3
Os01g0822501	1.02	0	1.08	9.031	16.27	9.516	2.35	1.27	2.07	1.724	0	0.941	3.2656	1.8669	3.1679	23.88	2.061	3.108	12.08	8.869	28.45	1.479	0	0	0	0.943	0	0.93	0.96	0	10
Os01g0748000	26.5	18.6	30.2	13.96	27.12	16.44	29.7	31.6	22.8	48.28	38.37	27.28	27.213	33.604	32.471	59.7	16.49	37.3	24.15	33.86	17.24	43.62	30.64	39.72	29.58	36.77	24.12	59.2	31.6	50.8	13
Os01g0683100	334	338	308	380.9	373.3	338.2	428	454	426	277.6	275.2	301.1	364.65	305.24	292.24	561.2	321.5	356.4	293.5	301.5	292.3	322.4	333.5	377.7	276.8	293.2	276.3	279	250	315	6
Os01g0857300	145	176	138	114.9	109.4	130.6	151	175	153	128.5	148	108.2	139.33	140.95	133.84	358.2	124.7	153.3	125.4	151.6	119.8	190.8	151.1	137.9	138.4	117.9	122.8	171	214	137	6
Os01g0887100	586	510	637	485.2	405.9	506.1	218	259	243	649.2	714.8	849.6	726.04	918.52	832.36	943.3	621.4	515.9	634.5	570	662.2	207.8	178.9	266.5	602.1	627	684.2	612	682	630	3
Os01g0744200	1.02	0	0	0	0	3.46	0.78	0.63	1.38	0	0	0	0	0	0	0	0	0	0	2.419	2.587	0.739	0.713	0	0	0	0	0	1.91	0	10
Os01g0349600	375	437	359	627.2	725.9	629.8	896	886	905	395.7	536.1	631.3	594.33	521.8	465.68	979.2	530.7	618.5	625.2	666.8	699.3	781.5	722.6	857.3	481.7	545	429.8	366	427	480	18
Os01g0306800	752	902	798	5245	6173	4598	3515	3991	3488	646.6	722.5	730.1	765.23	551.67	331.04	835.9	282.4	664.1	3669	4784	3983	2688	2503	2933	363.4	221.6	221.5	227	365	332	12
Os01g0682500	7397	7125	8004	4719	4620	4555	7401	7703	7214	9506	9602	11975	11254	10337	10773	11953	9212	8234	7371	6333	7296	4183	4196	5502	9470	10037	10177	7800	8591	8598	3
Os01g0514450	0	0	1.08	13.96	6.328	0.865	5.48	12.7	3.46	0	2.193	0	0	0.9335	0	0	0	0	6.503	7.257	4.311	0.739	0.713	2.889	0	0	0	0	0	0.83	12
Os01g0819900	1459	1664	1612	1023	1049	1090	1421	1271	1419	1368	1411	1189	1514.1	1384.3	1770.8	2615	1531	1487	879.6	816	961.4	1339	1188	1236	1438	1371	1230	1514	1596	1645	13
Os01g0677500	0	0	1.08	0	0	0	0	0	0	0	0	0	0	0	0	0	0	0	1.858	0	0	0	0	0	0	0	0	0	0	0	16
Os01g0756101	0	0	0	0	0	0	0	0	0	0	0	0	0	0	0	0	0	0	0	0	0	0	0	0	0	0	0	0	0	0.83	1
Os01g0661400	387	369	381	555	541.5	516.4	420	405	488	372.5	337.7	355.6	395.13	435.92	268.48	847.8	362.7	415.5	602	574.1	689.8	421.5	402.6	363.3	290.5	314.9	307	338	360	290	6
Os01g0254100	2618	2677	2687	2424	2415	2475	4169	4376	4263	1866	1965	1787	2637.5	2340.2	2744.2	3929	2261	2250	1926	1763	2002	5496	5445	4411	2187	2063	2344	2725	2253	2572	17

Os01g0821300	115	224	78.7	9.852	0.904	5.19	2.35	1.9	10.4	5.173	0	0.941	1.0885	0.9335	0	11.94	26.79	25.9	2.787	1.613	0	0	2.851	0.722	0	0	0	0	6.69	0	14
Os01g0626300	1.02	0.93	1.08	2.463	1.808	2.595	5.48	0.63	2.07	1.724	0	4.704	2.177	0	0	0	1.031	2.072	1.858	0	2.587	2.218	1.425	0.722	0	1.886	2.193	0	0	0	15
Os01g0651100	24.5	11.2	21.6	160.9	182.6	149.7	77.5	95.5	67	8.622	17.54	10.35	15.239	15.869	11.879	23.88	16.49	13.47	144	160.5	138	65.07	77.68	76.56	4.225	8.486	2.193	3.7	7.65	11.7	10
Os01g0364100	86.7	123	88.4	91.95	80.45	57.09	63.4	80.3	33.9	52.59	54.82	24.46	40.275	49.473	78.405	131.4	80.38	88.06	32.51	41.12	27.59	125	104.8	59.22	80.28	34.89	19.74	119	120	143	8
Os01g0727100	3.06	7.45	1.08	4.926	0	2.595	0	1.27	3.46	3.449	1.096	5.645	5.4426	5.6007	0.792	0	2.061	5.18	7.432	4.031	8.622	1.479	9.264	5.056	1.056	5.657	2.193	0.93	1.91	1.67	16
Os01g0696800	80.5	86.6	98.1	9.852	5.424	14.71	53.2	56.3	35.9	126.7	80.04	122.3	87.082	65.342	68.901	143.3	101	92.21	7.432	16.13	7.76	25.88	32.07	49.11	76.06	100.9	182	50	52.6	46.7	3
Os01g0956500	10.2	2.79	0	1.642	2.712	1.73	0	0	0.69	0	0	2.822	0	2.8004	0	11.94	7.214	1.036	1.858	0.806	3.449	2.218	4.276	6.5	13.73	7.543	8.772	5.55	1.91	3.33	13
Os01g0803200	7152	7077	7402	21239	19553	22131	5651	6259	6871	11470	11082	10055	8544.9	9026.5	8641.1	8490	6244	6899	27137	28172	28969	6768	8679	7945	9181	8670	9162	6953	7734	7294	10
Os01g0243300	14.3	8.38	9.7	3.284	0.904	3.46	2.35	1.9	0	0	0	0.941	0	1.8669	1.5839	0	9.275	7.252	3.716	0.806	2.587	0	0	2.167	0	0.943	0	0.93	1.91	0	14
Os01g0909300	10.2	9.31	7.54	1.642	1.808	2.595	4.7	3.8	8.98	4.311	5.482	4.704	6.5311	4.6673	4.7518	11.94	7.214	9.324	2.787	2.419	6.036	5.915	4.276	8.667	1.056	11.31	13.16	5.55	7.65	9.16	13
Os01g0170100	118	146	143	142	152.8	168.7	139	140	146	151.7	191.9	184.4	162.19	171.76	165.52	274.6	191.7	180.3	134.7	166.9	167.3	133.8	134.7	160.3	134.2	196.1	199.6	176	189	196	13
Os01g0824800	155	178	186	114.9	186.2	128.9	215	231	152	234.5	171	180.6	108.85	169.89	151.27	274.6	183.4	182.3	114.3	125	140.5	231.4	302.2	256.4	171.1	151.8	182	188	165	181	17
Os01g0287600	111	89.4	120	64.86	47.91	63.15	87.7	54.4	72.6	58.63	40.57	99.73	52.249	61.608	53.854	119.4	96.87	76.67	100.3	39.51	90.53	91.68	74.83	169	63.38	45.26	61.4	54.6	37.3	44.2	9
Os01g0273500	320	345	327	409.7	460.1	405.7	498	539	552	356.9	406.8	303.9	279.75	278.17	381.73	465.7	267.9	242.4	354.9	405.6	357.8	583.4	508.8	483.9	363.4	331.9	291.7	337	329	344	18
Os01g0220900	0	0	0	0	0	0	0	0	0	0	0	0	0	0	0	0	0	0	0.929	0	0	0	0	0	0	0	0	0	0	0	16
Os01g0832900	116	78.2	91.6	123.1	123.8	115.9	69.7	87.9	60.8	58.63	43.86	30.11	43.541	46.673	61.773	167.2	68.02	67.34	122.6	122.6	103.5	97.6	124	101.1	46.48	41.49	37.28	63.8	94.7	64.2	10
Os01g0238700	0	1.86	0	0.821	0.904	0	0.78	1.27	0	0.862	1.096	0	1.0885	0	2.3759	0	0	0	1.858	0	1.724	0	0	1.444	0	0.943	0	0	1.91	0	4
Os01g0113200	40.8	58.7	31.2	5.747	4.52	5.19	17.2	6.96	8.98	2.587	6.578	5.645	1.0885	0.9335	2.3759	23.88	13.4	11.4	7.432	4.031	0	8.873	17.82	18.78	1.056	0.943	0	0.93	3.83	1.67	14
Os01g0962400	1156	962	1040	802.1	797.3	918.7	965	868	937	1383	1125	1641	1226.8	1380.6	1150.7	2018	1314	1119	1129	927.2	1116	644.7	665.6	774.2	1230	1268	1447	976	1046	918	13
Os01g0114550	60.2	101	87.3	62.39	63.28	75.26	105	125	67	147.4	137	117.6	82.728	91.478	95.036	214.9	79.35	110.9	111.5	131.4	114.7	90.21	113.3	73.67	114.1	73.54	120.6	92.5	99.5	77.5	6
Os01g0191700	3739	2848	3545	848.1	727.7	748.3	750	1006	907	2118	2016	1815	1526.1	1755.8	1939.5	3379	2379	2563	730.2	736.9	682.9	1165	1303	1125	2388	2256	2193	2089	1828	1804	13
Os01g0160700	2.04	4.66	3.23	59.11	43.39	32.01	3.91	4.43	6.91	0.862	1.096	0.941	2.177	3.7338	1.5839	0	2.061	3.108	36.23	26.61	40.52	17.01	1.425	6.5	2.113	3.771	4.386	5.55	5.74	7.5	10
Os01g0900400	2.04	5.59	9.7	2.463	1.808	3.46	0	0	0.69	18.11	10.96	10.35	2.177	16.802	13.463	11.94	9.275	7.252	1.858	8.063	0	2.958	0.713	2.889	17.96	21.69	17.54	19.4	23	30.8	3
Os01g0706200	0	0.93	0	1.642	0.904	1.73	0	0.63	3.46	0	1.096	0	2.177	0.9335	0	0	0	1.036	0	1.613	1.724	0	0	0	0	0	0	0	0	0	2
Os01g0744700	0	0.93	0	0	0.904	0	1.57	1.27	0	0	0	0	0	0	0	23.88	0	1.036	0	0	0	0.739	4.988	1.444	0	0	0	0	0	0	6
Os01g0628900	7.14	3.73	9.7	22.17	27.12	14.71	20.4	8.86	2.07	18.97	13.16	6.586	13.062	13.068	13.463	11.94	3.092	6.216	20.44	31.44	15.52	0.739	5.701	1.444	2.113	2.829	6.579	5.55	5.74	3.33	10
Os01g0549500	12.2	18.6	9.7	10.67	9.944	12.98	8.61	5.06	9.67	8.622	7.675	7.527	11.974	4.6673	16.631	0	3.092	9.324	4.645	5.644	8.622	14.79	11.4	12.28	6.338	9.428	6.579	10.2	9.56	17.5	8
Os01g0366300	4.08	7.45	11.9	5.747	4.52	0	2.35	11.4	4.15	10.35	8.771	6.586	10.885	4.6673	13.463	11.94	1.031	6.216	0	11.29	0	13.31	12.11	7.945	5.282	7.543	0	16.7	9.56	10.8	1
Os01g0821600	1001	869	565	31.2	16.27	16.44	5.48	21.5	29.7	89.67	96.48	66.8	7.6196	22.403	13.463	250.8	456.5	282.8	13.01	10.48	11.21	27.36	19.95	97.5	19.01	36.77	37.28	13.9	22	21.7	14
Os01g0224400	210	214	171	114.1	126.6	103.8	142	165	133	223.3	207.2	180.6	249.27	219.36	213.83	561.2	216.4	188.6	136.6	127.4	133.6	124.2	128.3	134.3	176.4	178.2	232.4	198	219	175	13
Os01g0873200	794	629	763	174	159.1	151.4	511	711	756	719.9	890.3	1312	921.98	617.94	742.86	955.3	918.2	885.8	159.8	156.4	155.2	480.6	418.3	587.2	830.3	866.5	642.5	502	505	638	3
Os01g0320700	135	189	167	396.5	518	470.6	403	435	421	163.8	159	157.1	201.38	136.28	171.86	167.2	106.1	149.2	322.3	381.4	378.5	346.8	233	314.9	146.8	92.4	94.3	117	138	180	12
Os01g0207900	30.6	40.1	38.8	28.73	36.16	30.28	8.61	23.4	20.7	32.76	75.65	22.58	67.488	79.343	70.485	71.65	23.7	35.23	8.361	9.675	12.93	3.697	4.988	2.167	41.2	18.86	10.96	40.7	44	59.2	4
Os01g0813700	1.02	0	0	0	0	0	0.78	1.27	0	0	0	0	0	1.8669	0	11.94	2.061	0	0	0	0	0.739	0.713	2.167	0	0	4.386	0.93	0	0	6
Os01g0257100	19.4	17.7	12.9	9.031	3.616	15.57	9.39	1.9	0.69	18.97	7.675	19.76	16.328	17.736	15.047	35.82	29.89	20.72	12.08	12.09	19.83	11.83	4.988	26	9.507	12.26	21.93	13	24.9	12.5	13
Os01g0607400	2359	2376	2349	1926	1887	1810	1694	1843	1652	1951	2211	2067	2764.8	2609.9	2649.9	4060	2525	2519	1713	1780	1859	1942	1778	1779	2454	2438	2199	2683	2466	2831	13
Os01g0615200	32.6	35.4	22.6	28.73	24.41	38.93	52.5	29.7	57.4	54.32	32.89	41.4	52.249	49.473	32.471	47.76	26.79	14.5	39.02	35.48	28.45	24.4	20.67	31.78	16.9	28.29	32.89	17.6	33.5	31.7	15
Os01g0870400	93.8	137	130	86.2	94.01	102.1	32.1	22.8	15.9	156.9	125	161.8	94.701	183.89	106.92	477.6	348.3	267.3	160.7	197.5	260.4	34.75	31.36	33.22	252.5	214	142.5	259	186	201	13
Os01g0114100	7.14	5.59	3.23	0.821	1.808	3.46	0	3.8	7.6	2.587	6.578	4.704	6.5311	4.6673	5.5438	0	1.031	0	3.716	0.806	1.724	2.218	2.851	7.222	4.225	10.37	0	4.63	3.83	3.33	4
Os01g0644200	0	0	0	0	0	0	0	0	0	0	0	0	0	0	0	0	0	0	0	0	0	0	1.425	0	0	0	0	0	0	0	17
Os01g0547200	447	503	463	391.6	431.2	429.1	200	206	193	316.4	341	271	410.37	351.91	373.81	716.5	450.3	483.8	355.8	399.9	370.8	275.1	294.3	230.4	386.6	331.9	383.8	451	482	521	13
Os01g0776600	450	478	450	1133	1003	794.1	633	756	479	427.6	414.4	394.2	290.63	345.38	401.53	680.6	383.4	335.7	1094	840.1	762.2	465.1	665.6	577.8	387.7	305.5	427.6	553	603	382	10
Os01g0771900	187	190	183	169.9	161.8	160.9	236	162	215	154.3	139.2	164.6	115.38	154.95	97.412	214.9	186.5	187.5	209.9	144.3	192.3	144.2	163.9	170.4	168	180.1	190.8	154	167	138	6
Os01g0269900	82.6	58.7	77.6	96.05	83.17	88.23	114	87.3	115	88.81	92.1	115.7	74.019	73.743	83.948	107.5	91.72	70.45	134.7	115.3	118.1	78.37	86.94	79.45	109.9	106.5	67.98	79.6	73.6	74.1	15
Os01g0919700	962	1114	1141	1060	1142	1063	1230	1498	1470	888.9	1074	951.2	1029.7	1007.2	1087.4	1803	1194	1412	951.2	1107	972.6	1643	1413	1407	1150	1168	741.2	1212	1438	1316	6
Os01g0369600	755	748	752	536.9	556.8	522.5	554	608	523	559.6	812.4	589	702.1	633.81	733.36	1397	778.1	806	445.9	464.4	413.9	817.8	687	563.4	757.4	827.8	800.4	810	802	934	13
Os01g0367900	452	601	349	643.6	1059	426.5	391	435	299	701.8	563.5	699	533.37	500.33	483.89	238.8	814.1	382.3	443.1	474.1	524.2	413.3	373.4	320	897.9	480.8	410.1	437	446	663	2
Os01g0206200	126	121	134	252	235	227.5	228	225	249	131.1	120.6	99.73	151.3	164.29	129.09	214.9	155.6	134.7	283.3	293.5	263	160.4	171.7	167.6	113	89.57	105.3	108	137	114	10
Os01g0783500	285	234	261	346.5	280.2	314	644	645	638	379.4	351.9	321.8	240.56	314.57	275.6	573.2	297.8	254.9	363.2	350.7	366.4	402.2	464.6	412.4	340.1	309.3	372.8	321	323	267	18
Os01g0844300	32.6	16.8	29.1	13.14	9.944	11.25	6.26	10.8	2.76	18.11	10.96	9.408	15.239	12.135	15.047	83.59	40.19	20.72	8.361	11.29	12.07	3.697	2.851	8.667	7.395	11.31	21.93	5.55	10.5	13.3	13
Os01g0377700	577	617	626	587	584.9	551	489	577	513	525.1	531.8	668	524.67	635.68	593.97	835.9	708	652.7	661.4	556.3	677.7	494.6	511	494	571.5	631.7	633.8	638	707	617	13
Os01g0328500	119	144	152	172.4	194.4	168.7	173	182	211	120.7	114	158.1	130.62	127.88	151.27	214.9	121.6	118.1	168.1	155.6	189.7	184.1	153.9	137.9	131	99	151.3	127	140	119	12
Os01g0152950	104	95.9	94.8	137.9	197.1	149.7	185	197	200	112.9	97.58	107.3	93.613	101.75	97.412	119.4	88.63	97.39	116.1	149.2	108.6	153.8	166.8	152.4	99.3	90.51	142.5	105	118	128	18
Os01g0925000	674	727	695	715.9	732.2	626.3	546	584	498	596.6	584.4	510.9	764.14	671.15	682.67	979.2	692.5	756.3	658.6	647.4	629.4	564.9	577.2	514.2	733.1	596.8	524.1	649	703	726	13
Os01g0707500	11.2	7.45	7.54	0.821	3.616	2.595	3.91	5.69	10.4	1.724	4.386	0.941	3.2656	2.8004	2.3759	0	0	2.072	3.716	0	3.449	10.35	9.264	11.56	4.225	2.829	0	3.7	4.78	0.83	9
Os01g0692600	3.06	0.93	3.23	7.389	2.712	5.19	4.7	1.27	6.91	4.311	3.289	2.822	1.0885	5.6007	4.7518	0	3.092	3.108	7.432	8.063	15.52	6.654	1.425	0.722	2.113	6.6	8.772	3.7	4.78	2.5	16
Os01g0499300	504	544	581	565.7	541.5	508.6	546	563	609	481.1	551.5	483.6	573.65	559.14	547.25	871.7	546.2	522.2	500.7	567.6	535.4	535.3	506	571.3	537.7	525.2	480.2	534	542	540	6
Os01g0530100	16.3	6.52	16.2	12.31	10.85	14.71	9.39	3.8	4.84	15.52	7.675	14.11	10.885	15.869	3.9598	35.82	28.85	20.72	21.37	17.74	21.56	0.739	8.552	12.28	13.73	12.26	21.93	12	13.4	15	13
Os01g0626400	13.3	18.6	11.9	27.91	13.56	20.76	11	3.8	17.3	12.07	15.35	10.35	10.885	7.4676	13.463	23.88	10.31	13.47	21.37	16.13	2.587	24.4	28.51	20.95	5.282	13.2	2.193	21.3	16.3	25	17
Os01g0770500	347	338	328	361.2	403.2	325.3	230	245	221	312.1	305.9	347.2	379.89	372.45	384.9	847.8	325.6	326.4	465.4	422.5	461.3	135.3	146.8	175.5	344.4	322.5	311.4	301	333	312	6
Os01g0132050	1.02	8.38	2.16	3.284	3.616	0	10.2	4.43	1.38	15.52	6.578	1.882	4.3541	6.5342	3.1679	11.94	5.153	2.072	3.716	3.225	9.484	2.958	12.11	3.611	3.169	2.829	4.386	1.85	4.78	3.33	7
Os01g0278900	2064	2162	2369	3232	3541	2971	7320	6535	6878	2530	3066	4093	2809.5	2314	1987	2937	1885	1894	4387	4040	4124	3454	3356	5067	2184	1984	1925	1358	1806	1585	18
Os01g0896700	3537	2964	2817	3834	3902	4118	3765	3261	4134	3327	2807	4936	3365.7	3859.8	2575.5	4287	2595	2907	4140	3175	4122	3446	3637	3242	2904	3716	3941	2195	2886	2112	15
Os01g0918200	321	360	296	232.3	208.8	224.9	265	231	217	294.9	217.1	313.3	263.42	322.97	320.75	346.3	329.8	288	293.5	289.5	338	294.3	345.6	294.7	313.7	252.7	271.9	440	330	336	1
Os01g0281600	0	0	1.08	0.821	0	6.92	2.35	0	3.46	0.862	0	1.882	1.0885	0.9335	1.5839	0	5.153	0	2.787	0.806	10.35	0	2.851	5.778	0	2.829	17.54	0	2.87	0	11
Os01g0556700	1513	1206	1192	298.8	271.2	318.3	1011	533	582	2349	2141	3187	1097.2	1348.8	1061.2	1445	1611	1458	798	458	743.2	425.9	270.1	873.2	1641	2235	2414	1193	1236	867	20
Os01g0169500	877	1002	823	914.6	966.3	878.9	882	969	862	760.4	837.6	637.9	865.37	810.24	869.58	1349	702.8	830.9	591.7	633.7	590.6	1040	1128	936.8	724.7	786.3	585.5	931	787	919	6
Os01g0238850	0	0	0	0	0	0	0	0	0	0	0	0	0	0	0	0	0	1.036	0	0	0	0	0	0	0	0	0	0	0	0	1
Os01g0110500	17.3	22.4	21.6	48.44	30.73	51.04	19.6	20.2	13.1	6.897	8.771	11.29	11.974	12.135	11.879	11.94	32.98	23.83	58.52	53.21	49.15	24.4	48.46	26.72	14.79	11.31	15.35	23.1	19.1	15.8	10
Os01g0109700	2288	2838	2607	2878	2922	2720	2542	2685	2457	2230	2415	2122	2590.7	2208.5	2737.8	4573	2475	2482	2177	2351	2153	3179	3128	2817	2702	2382	2250	2925	2626	2895	6
Os01g0286000	161	189	182	129.7	120.2	116.8	333	283	281	127.6	157.9	108.2	112.12	110.15	140.18	238.8	159.7	173	152.3	136.3	107.8	466.6	348.5	377	152.1	157.5	157.9	229	227	193	9
Os01g0832300	125	92.2	81.9	73.07	66.89	94.29	111	77.2	69.8	88.81	88.81	118.5	90.347	102.68	82.364	59.7	109.2	65.27	90.11	76.6	113.8	66.54	89.79	126.4	87.68	133.9	129.4	94.4	89.9	84.1	20
Os01g0696500	1.02	0	0	1.642	2.712	0.865	6.26	3.8	4.15	0	0	0.941	0	1.8669	0	0	2.061	0	3.716	4.031	6.898	2.218	2.851	5.056	1.056	0	2.193	0.93	0	0.83	12
Os01g0183500	0	0	0	1.642	0	0	0	0	1.38	0	0	0	0	0	0	0	0	0	0	0	0	0	0	0.722	0	0	0	0	0	0	2
Os01g0759900	0	0	0	0	0	0	0.78	1.27	0	0	0	3.763	0	4.6673	0.792	0	0	0	0	0	0	0.739	0.713	0	0	0	2.193	0	0	0	20
Os01g0256300	9.18	8.38	12.9	9.852	9.944	6.92	2.35	1.27	2.07	9.484	6.578	2.822	1.0885	12.135	5.5438	0	5.153	9.324	0.929	4.031	1.724	4.436	4.276	3.611	6.338	0.943	4.386	8.33	0.96	0.83	19
Os01g0784800	42.8	68.9	65.7	63.22	67.8	61.42	39.1	54.4	50.4	37.07	39.47	16.93	30.479	16.802	48.31	191.1	63.89	64.23	39.94	49.18	36.21	70.24	95.49	58.5	43.31	33.94	46.05	74	71.7	77.5	6
Os01g0251000	313	377	332	543.5	570.4	515.6	601	571	591	343.2	360.7	408.3	411.46	353.78	284.32	537.3	359.7	342.9	536.9	494.3	509.6	428.1	359.9	447.1	271.5	282.9	307	283	320	316	12
Os01g0102000	0	0.93	0	0	0	0	0	0	0	0	0	0	1.0885	0	0	0	0	0	0	0	0	0	0.713	0	0	1.886	0	0	0	0	4
Os01g0112050	7.14	7.45	10.8	8.21	17.18	19.03	4.7	3.8	5.53	16.38	4.386	16.93	7.6196	16.802	7.9197	0	7.214	6.216	15.79	16.93	14.66	8.133	2.851	9.389	16.9	14.14	15.35	7.4	17.2	5.83	16
Os01g0661500	1532	1090	1296	435.9	486.3	450.7	463	485	447	772.5	786.1	1075	1291	1235.9	1257.6	2854	1758	1699	666.1	590.2	658.7	459.2	401.2	387.1	1172	1305	1346	1181	1069	1136	13
Os01g0127600	2.04	1.86	1.08	83.74	2.712	130.6	1.57	77.2	145	34.49	16.45	3.763	27.213	1.8669	5.5438	0	0	0	65.03	0.806	12.93	73.2	5.701	12.28	0	3.771	245.6	0	23	0	11
Os01g0137500	10.2	15.8	15.1	8.21	10.85	10.38	5.48	19.6	3.46	5.173	7.675	0.941	6.5311	7.4676	8.7116	11.94	9.275	8.288	14.86	8.063	7.76	25.88	27.79	13	12.68	15.09	4.386	24.1	21	13.3	17
Os01g0854400	0	0	1.08	0	0	0	2.35	0	0	0	0	0	0	0	0	0	1.031	0	0	0	0	0	0	0	0	0	0	0	0	0	5
Os01g0730200	69.3	52.2	73.3	59.11	75.03	57.96	114	116	133	113.8	91	91.26	83.816	88.678	109.29	119.4	59.77	70.45	92.9	74.18	81.91	93.16	89.08	68.61	85.57	85.8	76.75	106	110	88.3	5
Os01g0802000	71.4	95	65.7	75.53	89.49	95.16	128	91.7	127	68.11	87.71	73.38	70.754	44.806	56.23	143.3	58.74	63.2	68.74	73.37	86.22	70.98	80.53	95.34	72.89	68.83	39.47	51.8	52.6	47.5	6
Os01g0149800	####	####	####	5829	5454	6737	3287	3175	2819	9957	9263	9715	9213.2	14466	10414	19046	14285	12504	6847	7147	7465	3408	4089	5654	11066	10645	12199	####	####	####	13
Os01g0868000	61.2	27.9	33.4	17.24	12.66	28.55	7.05	10.8	18	23.28	19.74	21.64	42.452	31.737	26.927	59.7	46.37	37.3	20.44	17.74	25.87	38.45	25.65	36.83	25.35	29.23	50.44	25	23.9	20.8	13
Os01g0758000	0	0	0	0	0	0.865	0	0	0	0	0	0	0	0	0.792	0	0	2.072	0	0	0	0	0.713	0	1.056	0	0	0	0	0	1
Os01g0698000	388	257	355	412.1	395.9	397.9	384	338	369	424.2	370.6	525	435.41	425.65	367.47	704.5	462.7	344	552.7	538.6	455.3	325.3	315	343.8	381.3	416.7	418.8	360	442	378	6
Os01g0637800	29.6	14.9	23.7	0.821	0.904	4.325	0	0.63	0.69	7.76	4.386	10.35	26.124	7.4676	7.1277	83.59	23.7	9.324	10.22	6.45	4.311	1.479	0	1.444	6.338	10.37	10.96	13	6.69	13.3	13
Os01g0750900	89.7	69.9	78.7	41.87	43.39	43.25	32.1	35.4	35.2	92.25	91	116.7	74.019	70.942	47.518	59.7	84.5	67.34	55.74	55.63	47.42	26.62	29.22	31.78	88.73	110.3	98.68	74	72.7	80.8	3
Os01g0510800	843	952	1010	851.4	866.9	866.8	803	826	759	885.5	804.8	880.6	1075.5	861.58	941.65	1206	1036	946.9	796.1	767.6	863.1	884.3	839.5	786.5	824	821.2	796	924	877	960	13
Os01g0673700	26.5	19.6	31.2	24.63	27.12	22.49	17.2	18.3	17.3	22.42	17.54	10.35	23.947	17.736	9.5036	23.88	37.1	23.83	19.51	32.25	37.08	15.53	19.95	30.33	32.75	22.63	13.16	14.8	28.7	22.5	16
Os01g0300200	1445	1449	1432	1445	1449	1499	1623	1742	1835	1416	1264	1279	1131	1415.1	1266.4	2699	1673	1494	1488	1264	1349	1516	1606	1789	1311	1374	1482	1410	1356	1272	6
Os01g0635000	1.02	0	0	0	0	1.73	0	0	0	0	0	0	1.0885	0	0	0	0	0	0	0	0	0	0.713	0	0	0	0	0	0	0	19
Os01g0143800	656	917	737	3426	3887	3135	3034	3087	2935	882.9	1058	1259	978.58	812.1	532.99	597	381.3	551.2	3545	3469	3656	2048	2205	2271	428.9	317.7	344.3	302	345	440	12
Os01g0663800	1222	1229	1329	996.7	1103	942.9	983	1099	1012	1248	1250	1028	1235.5	1143.5	1289.3	1875	1210	1195	678.1	848.2	820.8	1199	1303	1093	1430	1286	1149	1168	1184	1350	13
Os01g0186900	2439	2958	1231	117.4	12.66	26.82	23.5	20.9	13.8	106	52.63	47.04	66.4	36.405	38.014	1170	2031	1349	33.44	29.03	466.5	28.1	44.9	767.7	28.52	43.37	65.79	25	29.6	80	14
Os01g0112500	467	655	597	536.9	550.5	572.7	1152	968	1113	875.1	894.7	626.6	792.44	779.43	897.3	955.3	651.3	685.9	647.5	653.9	589.8	939	984.9	814.7	995.1	782.6	855.2	821	822	915	5
Os01g0672500	1023	833	980	1025	938.3	1010	197	344	304	914.8	709.4	737.6	846.87	650.62	794.34	1099	743	865.1	733.9	832.1	757.9	510.9	625	354.6	761.6	759	686.4	671	626	691	8
Os01g0705300	1898	1960	2129	2483	3060	2514	2318	2401	2515	1713	2142	1950	2338.1	2011.6	1673.4	2782	1587	1767	2334	2787	2468	1958	1710	2180	1499	1358	1252	1281	1366	1263	10
Os01g0117200	210	603	579	546.8	802.7	440.3	1555	2187	1223	894.9	878.2	263.4	653.11	512.46	882.25	561.2	203	254.9	106.8	70.15	65.53	919.8	853	348.8	332.8	111.3	175.4	437	279	389	18
Os01g0271200	125	142	126	139.6	141.9	134.9	150	158	140	159.5	109.6	165.6	152.39	198.83	125.92	167.2	124.7	153.3	167.2	132.2	161.2	146.4	176.7	146.6	155.3	139.5	239	155	136	147	11
Os01g0972200	21.4	7.45	12.9	8.21	6.328	6.055	30.5	5.06	14.5	14.66	5.482	18.82	9.7967	8.4011	0.792	11.94	3.092	7.252	6.503	0	3.449	3.697	8.552	12.28	0	1.886	2.193	3.7	6.69	7.5	5
Os01g0745400	0	0	0	0	0	0	4.7	0	0	0.862	0	0	0	0	0	0	0	0	0	0	0	0	0	0.722	0	0	0	0	0	0	5
Os01g0913800	114	82.9	123	298.8	312.8	269	259	296	247	125.9	156.8	121.4	161.1	104.55	133.84	59.7	84.5	56.98	311.2	332.2	245.7	125.7	180.3	186.3	118.3	83.91	120.6	93.4	91.8	85	12
Os01g0289950	0	0	0	0	0	0	0	0	0	0	0	0	0	0	0	0	0	0	0	0	0	0	0	0	1.056	0	0	0.93	0	0	1
Os01g0166800	217	323	255	264.4	237.7	218	254	281	214	368.2	357.4	299.2	241.65	180.16	229.67	274.6	162.8	223.8	200.7	229	127.6	343.1	334.2	257.1	283.1	179.1	177.6	259	186	249	7
Os01g0162001	3.06	2.79	3.23	10.67	3.616	6.92	31.3	9.49	6.91	1.724	5.482	0	3.2656	2.8004	1.5839	0	0	1.036	1.858	4.838	2.587	8.133	4.276	7.222	2.113	0	4.386	2.78	6.69	0	18
Os01g0869300	6.12	5.59	5.39	2.463	0	6.055	3.13	5.69	1.38	0.862	0	0	13.062	3.7338	12.671	0	1.031	2.072	5.574	0.806	2.587	1.479	2.138	2.167	3.169	0.943	2.193	1.85	6.69	2.5	4
Os01g0720200	96.9	80.1	80.8	86.2	101.2	105.5	186	105	171	127.6	121.7	157.1	97.967	111.08	114.84	143.3	112.3	124.3	180.2	127.4	150	83.55	82.67	115.6	94.02	133.9	157.9	76.8	88.9	90	15
Os01g0772700	883	964	841	921.1	1013	929.1	603	749	616	740.6	803.7	560.7	734.75	788.77	896.51	1648	845	899.3	703.2	706.3	644.1	1012	910.7	775.7	865.2	776	682	964	913	979	6
Os01g0603300	519	474	483	169.1	95.82	168.7	336	715	433	356.9	432	376.3	606.31	427.52	507.65	406	459.6	406.1	169.1	118.5	72.43	173.8	227.3	252.1	308.5	327.2	296	302	289	368	5
Os01g0976200	0	0.93	1.08	0	0	0	0	0.63	0	0	0	0	0	0.9335	0	0	0	0	0	0	0.862	0	0	0	1.056	0	0	0	0	0.83	14
Os01g0951500	231	235	251	101	107.6	144.5	340	354	370	441.4	390.3	407.4	349.41	279.1	312.03	429.9	224.7	272.5	165.4	192.7	150.9	244.7	329.2	335.1	366.6	376.2	348.7	310	315	307	5
Os01g0962100	7.14	4.66	3.23	11.49	14.46	19.03	21.1	55.7	19.3	4.311	4.386	6.586	3.2656	0	8.7116	23.88	9.275	5.18	17.65	17.74	10.35	8.873	47.75	24.56	10.56	12.26	8.772	4.63	4.78	10.8	18

Os01g0883200	723	745	767	865.3	797.3	825.3	838	924	919	888.9	854.1	897.5	913.27	949.32	995.5	1612	816.2	879.6	956.8	866.8	900.2	811.1	909.3	821.2	846.1	887.2	758.7	856	903	873	6
Os01g0264900	145	154	137	137.1	127.5	139.3	237	154	202	183.6	206.1	218.3	190.49	178.29	190.86	417.9	151.5	187.5	214.6	172.5	194.9	137.5	156.1	151.7	176.4	160.3	245.6	190	184	120	6
Os01g0304200	0	0	0	0	0	0	0	0	0.69	0	0	0	0	0	0	0	0	0	0	0	0	0	0	0	0	0	0	0	0	0	18
Os01g0252200	119	174	123	188.8	160.9	167.8	280	262	296	140.5	161.2	166.5	143.68	118.55	121.17	238.8	124.7	128.5	147.7	189.5	148.3	184.8	196	214.5	134.2	132	103.1	149	126	162	18
Os01g0963000	60.2	38.2	55	192.1	73.22	193.8	221	30.4	817	75.01	41.66	63.98	39.187	41.072	61.773	83.59	23.7	40.41	320.5	202.4	188	2523	793.2	2244	34.86	57.51	67.98	62	129	50.8	9
Os01g0800950	0	0	0	0	0	0	0	0	0	0	0	0	0	0	0	0	0	0	0	0	0	0	0.713	0	0	0	0	0	0	0	17
Os01g0891100	0	0	0	0	0	0	0	0	0	0	0	0	0	0	0	0	0	0	0	0	0	0	1.425	0.722	2.113	0	0	0	0	0	17
Os01g0749100	110	108	144	119.9	95.82	110.7	132	102	171	142.3	125	142.1	115.38	83.077	96.62	131.4	114.4	111.9	145.8	120.9	118.1	140.5	109.7	161.1	108.8	117.9	109.6	94.4	91.8	107	9
Os01g0657100	0	0	0	0	0	0	0.78	0.63	2.76	0	0	0	0	0	0.792	0	0	0	0	1.613	0	0	0	2.889	1.056	0	0	0	0	0	18
Os01g0271000	178	181	200	229.1	230.5	194.6	161	168	195	200	200.6	207	239.47	215.63	175.82	274.6	159.7	194.8	239.7	262.8	259.5	165.6	188.1	160.3	178.5	158.4	219.3	190	186	166	16
Os01g0755550	0	0	0	1.642	0	0	0	0	0	0	0	0	0	0	0	0	0	0	0	0.806	0	0	0	0.722	0	0	0	0	0	0	2
Os01g0873700	641	599	627	481.9	433.9	447.2	602	674	596	665.6	691.8	697.1	664	667.42	722.27	1361	694.6	657.9	460.8	552.3	475.9	651.4	614.3	587.2	731	730.7	600.9	648	636	574	13
Os01g0768600	320	432	338	586.2	609.3	720.6	460	536	565	461.3	537.2	532.5	436.5	301.5	430.04	310.5	237	298.4	406.9	473.3	450.1	683.9	508.1	507.7	418.3	372.4	399.1	314	279	337	12
Os01g0304400	5.1	7.45	2.16	4.926	0	0	0.78	0	0	0.862	1.096	5.645	4.3541	0.9335	1.5839	0	18.55	8.288	2.787	4.031	9.484	6.654	4.276	23.83	2.113	0.943	0	0	1.91	3.33	9
Os01g0104200	0	0	0	0	0	0.865	0	0	0	0	0	0	0	0.9335	0	11.94	0	0	1.858	0	0	0	0	0.722	0	0	0	0.93	0	0	6
Os01g0871400	2.04	0.93	2.16	2.463	6.328	1.73	0	1.9	1.38	0.862	0	0	0	0	1.5839	0	1.031	2.072	1.858	3.225	2.587	1.479	0	3.611	0	3.771	0	3.7	1.91	4.17	2
Os01g0328900	250	212	225	113.3	114.8	134.9	112	111	117	145.7	172.1	153.4	240.56	243.63	208.29	644.8	225.7	245.5	115.2	100.8	131.9	126.4	124.7	137.2	248.2	236.7	155.7	204	208	247	13
Os01g0172200	176	173	159	166.7	143.7	191.2	226	187	182	150.9	167.7	148.6	124.09	117.61	133.84	214.9	146.3	168.9	151.4	150.8	147.4	231.4	238	242	144.7	156.5	228.1	156	185	177	9
Os01g0842500	0	1.86	1.08	4.926	20.79	3.46	6.26	1.27	1.38	18.97	6.578	10.35	1.0885	0.9335	0	0	0	0	0	0	0	0	0	0	0	0	0	0	0	0	7
Os01g0819233	80.5	63.3	81.9	64.04	87.69	79.58	159	129	137	130.2	144.7	143.9	95.79	129.75	116.42	286.6	98.93	102.6	108.7	88.69	112.1	108.7	92.64	96.06	134.2	109.4	140.3	108	110	94.1	6
Os01g0667700	121	259	116	66.5	56.05	43.25	32.1	38	21.4	22.42	28.51	27.28	52.249	36.405	36.43	143.3	121.6	87.03	37.16	33.06	49.15	56.19	42.76	77.28	30.63	24.51	19.74	36.1	49.7	53.3	14
Os01g0585700	1637	1635	1668	1551	1558	1490	1684	1721	1594	1500	1588	1461	1773.2	1552.3	1736.8	3129	1818	1828	1481	1519	1510	1775	1893	1905	1811	1786	1632	1808	1822	1908	6
Os01g0303600	21.4	14	7.54	4.105	3.616	5.19	3.13	8.22	2.76	2.587	1.096	4.704	6.5311	6.5342	2.3759	11.94	5.153	9.324	9.29	5.644	17.24	0	4.988	6.5	3.169	3.771	10.96	5.55	4.78	4.17	19
Os01g0682200	250	218	231	254.5	239.6	237	263	217	225	284.5	237.9	223.9	191.58	233.36	228.09	394.1	166.9	161.6	208.1	198.3	172.4	232.9	255.8	250.6	229.2	213.1	210.5	191	185	213	6
Os01g0202500	107	95	83	712.6	659.9	759.5	47	46.8	66.3	81.05	91	107.3	131.71	142.82	124.34	417.9	216.4	155.4	1080	1123	1336	97.6	78.39	88.11	100.4	130.1	151.3	130	121	120	10
Os01g0836800	846	835	811	925.2	1010	1060	1897	1655	1455	1188	837.6	872.1	582.36	743.96	791.17	919.5	684.3	682.7	849.1	897.4	863.1	1373	1603	1384	751.1	684.5	842.1	544	618	578	18
Os01g0303000	####	####	####	92809	99948	94448	####	####	####	24462	24327	24204	38252	32663	30193	44349	25074	21902	#####	#####	#####	8323	9684	10851	25369	22850	20418	####	####	####	10
Os01g0601700	16.3	35.4	25.9	34.48	61.47	35.47	154	111	144	37.94	33.99	59.27	23.947	19.602	22.175	23.88	59.77	70.45	64.1	66.12	54.32	152.3	191	257.1	33.8	75.43	100.9	60.1	62.2	44.2	9
Os01g0922700	6.12	3.73	8.62	4955	6589	5932	207	274	174	8.622	3.289	14.11	9.7967	7.4676	10.296	0	5.153	6.216	4772	4610	6231	221.1	195.3	161.8	10.56	10.37	8.772	4.63	8.61	10.8	10
Os01g0390600	191	228	172	331.7	316.4	368.5	200	249	252	183.6	152.4	109.1	145.86	163.35	159.98	334.3	134	167.8	192.3	262.8	208.7	549.4	391.2	293.2	199.7	178.2	153.5	200	160	230	17
Os01g0974500	54	81	55	120.7	138.3	98.62	155	128	106	86.22	67.98	59.27	48.983	48.539	63.357	47.76	53.59	49.73	113.3	83.85	96.57	89.47	76.25	86.67	46.48	52.8	39.47	40.7	53.6	59.2	12
Os01g0391600	37.7	32.6	20.5	45.15	31.64	28.55	22.7	35.4	22.1	44.83	27.41	46.1	29.39	42.939	26.135	47.76	44.31	63.2	50.16	74.18	33.63	54.71	34.92	26.72	48.59	44.31	50.44	45.3	48.8	57.5	1
Os01g0789100	76.5	76.4	63.6	138.7	141.9	141.9	118	102	99.5	101.7	84.42	97.85	78.373	83.077	77.613	131.4	127.8	75.63	152.3	164.5	138	113.9	121.1	116.3	91.9	104.7	103.1	98.1	118	85.8	10
Os01g0601651	1.02	3.73	3.23	18.06	18.98	26.82	13.3	11.4	8.29	4.311	5.482	5.645	2.177	0.9335	3.9598	0	2.061	2.072	2.787	17.74	2.587	14.79	12.83	5.778	8.451	0.943	2.193	6.48	5.74	9.16	2
Os01g0301900	188	119	95.9	88.67	105.8	114.2	27.4	24	43.5	95.7	63.59	121.4	66.4	163.35	53.854	71.65	51.53	134.7	26.01	16.93	9.484	34.01	19.24	14.44	76.06	43.37	103.1	43.5	53.6	75.8	19
Os01g0848766	2.04	0	0	0	0	0	2.35	0	0	0	1.096	0	1.0885	0	0	0	0	0	0.929	0	0	0	0	0	0	0	0	0	0	0	19
Os01g0604700	639	642	721	845.6	844.3	734.4	727	817	773	657	635.9	547.6	742.37	662.75	705.64	1564	646.1	590.5	731.1	758.7	760.5	537.5	585.1	658	675	627.9	666.6	557	570	612	6
Os01g0834500	411	339	551	604.2	628.3	532	687	508	702	520.8	418.8	770.5	510.52	564.74	341.34	644.8	319.5	385.4	702.3	669.2	725.1	369	439	452.1	423.6	504.4	620.6	310	379	256	15
Os01g0138900	874	1010	971	3031	2653	3398	1218	1067	2224	1448	1840	1087	1284.5	1375.9	1212.5	1851	920.3	980.1	3891	3304	4067	1611	1281	1238	1152	1508	1879	995	1378	1114	10
Os01g0195200	31.6	27.9	21.6	19.7	24.41	35.47	18.8	9.49	2.76	25	23.02	35.75	14.151	18.669	11.088	71.65	26.79	12.43	13.93	8.063	11.21	14.79	17.82	26.72	17.96	28.29	32.89	18.5	23.9	12.5	6
Os01g0516600	85.6	97.8	101	168.3	154.6	149.7	229	301	140	137.1	100.9	143.9	112.12	118.55	122.75	191.1	80.38	50.77	109.6	97.56	73.29	112.4	161.8	119.2	101.4	94.28	100.9	87	69.8	81.6	18
Os01g0728900	228	233	238	192.1	253.1	172.1	209	199	209	227.6	250	238	246.01	272.57	248.68	310.5	261.8	307.7	208.1	257.2	213	250.7	223.1	208.7	287.3	298.9	287.3	315	328	330	13
Os01g0254850	24.5	30.7	21.6	0	3.616	2.595	18	10.1	13.8	45.7	13.16	23.52	6.5311	12.135	4.7518	11.94	19.58	9.324	8.361	1.613	0.862	4.436	6.414	7.222	7.395	7.543	15.35	0.93	1.91	4.17	7
Os01g0694100	38.7	46.6	38.8	77.17	74.13	58.82	38.4	47.4	67.7	26.73	18.64	11.29	13.062	17.736	39.598	35.82	20.61	18.65	62.24	87.08	57.77	59.15	72.69	40.45	20.07	16.03	13.16	26.8	27.7	22.5	12
Os01g0841700	382	500	366	54.18	56.95	35.47	44.6	119	48.4	212.1	75.65	73.38	69.665	74.676	113.25	238.8	334.9	299.4	33.44	61.28	50.87	151.6	206	169.7	75	57.51	109.6	70.3	73.6	68.3	14
Os01g0851600	34.7	27.9	33.4	29.56	24.41	30.28	23.5	27.8	26.9	24.14	25.22	79.03	35.921	30.804	23.759	0	22.67	23.83	21.37	23.38	41.39	11.83	9.264	24.56	22.18	33	50.44	30.5	18.2	22.5	20
Os01g0168100	6660	5465	6242	2084	1718	2045	940	1719	1262	3369	3666	2631	3547.5	3853.3	4060.4	6090	4817	4506	1810	1906	1713	1510	2124	1675	3526	3769	3375	4230	3705	4222	13
Os01g0117266	6.12	41	33.4	35.3	29.83	20.76	52.5	76.5	32.5	34.49	26.31	3.763	19.593	14.002	41.974	23.88	11.34	13.47	0	3.225	3.449	49.54	63.42	14.44	14.79	8.486	15.35	29.6	21	12.5	17
Os01g0152500	0	0	0	0	0	0	0	0	0	0	1.096	0	0	0	0	0	0	0	0	0	0	0	0	0	0	0	0	0	0	0	7
Os01g0948600	628	454	548	928.5	931.1	1066	472	597	683	306.1	332.2	405.5	211.17	457.39	364.3	656.8	634.8	529.4	958.7	928	907.1	927.2	1232	1252	322.2	510.1	622.8	479	496	391	12
Os01g0616600	25.5	17.7	24.8	82.1	98.53	74.39	50.9	37.3	60.1	30.18	25.22	30.11	18.505	20.536	12.671	47.76	17.52	27.97	94.75	76.6	92.26	32.53	37.77	28.17	20.07	22.63	37.28	13	30.6	17.5	10
Os01g0137950	0	0.93	1.08	0	0	0	1.57	0	0	0	0	0	0	0.9335	0	0	0	0	0	0	0	0	0	0	0	0	0	0	0	0	14
Os01g0629500	0	1.86	0	3.284	1.808	0.865	1.57	0.63	3.46	0	2.193	0	0	0.9335	0	0	1.031	0	6.503	8.063	7.76	2.218	1.425	0.722	1.056	0.943	0	0	0.96	0.83	16
Os01g0342750	17.3	28.9	25.9	68.96	71.41	42.39	18	42.4	14.5	8.622	15.35	8.467	14.151	12.135	28.511	11.94	8.244	19.68	23.22	35.48	24.14	22.18	25.65	24.56	14.79	9.428	15.35	25.9	26.8	17.5	2
Os01g0788200	74.4	40.1	49.6	25.45	9.04	24.22	5.48	9.49	8.98	18.97	16.45	69.62	52.249	55.074	51.478	71.65	119.5	88.06	71.53	35.48	68.98	1.479	2.138	2.167	70.78	46.2	52.63	56.4	42.1	44.2	3
Os01g0343100	0	0	0	0	0	0	0	0	0	0	0	2.822	0	0.9335	0.792	0	0	0	0	0	0	0	0	0	0	0	0	1.85	0	0.83	20
Os01g0749900	414	348	418	391.6	316.4	405.7	252	321	292	361.3	339.9	339.6	358.12	345.38	410.24	501.5	384.4	399.9	331.6	340.3	302.6	376.3	421.9	337.3	391.9	362.1	348.7	375	336	382	13
Os01g0302500	0	0	0	0	0	0	0	0.63	0	0	0	0	0	0	0	0	0	0	0	0	0	0	0	0	0	0	0	0	0	0	18
Os01g0915800	567	624	666	527.1	626.5	633.2	769	640	761	740.6	675.4	587.1	561.68	541.4	513.19	847.8	531.8	437.2	657.7	594.2	507.8	590.8	640.7	578.5	529.2	453.5	576.7	469	451	446	6
Os01g0322800	481	504	518	514.8	634.6	524.2	549	469	439	454.4	451.7	545.7	452.82	504.06	445.88	1242	487.4	443.4	591.7	581.3	650.1	439.9	452.5	434.8	415.2	451.6	451.7	479	395	465	6
Os01g0136900	0	0.93	2.16	2.463	1.808	3.46	10.2	5.06	2.76	0.862	2.193	0	2.177	0.9335	2.3759	0	2.061	0	4.645	3.225	0.862	5.176	3.563	5.778	0	0.943	4.386	0	0.96	1.67	18
Os01g0101900	0	0	0	0	0	0	0	0	0	0	0	0	0	0.9335	0	0	0	0	0	0	0.862	0	0	0	0	0	0	0	0	0	16
Os01g0976500	46.9	66.1	70	107.5	105.8	116.8	97.9	103	99.5	62.94	53.72	31.05	31.567	44.806	38.806	83.59	42.25	44.55	99.4	105.6	65.53	101.3	116.9	81.61	49.65	27.34	37.28	35.2	41.1	33.3	12
Os01g0688000	10.2	5.59	10.8	8.21	7.232	10.38	5.48	3.16	12.4	3.449	5.482	8.467	10.885	8.4011	10.296	11.94	4.122	13.47	8.361	8.063	14.66	13.31	22.8	10.11	13.73	5.657	4.386	8.33	10.5	8.33	17
Os01g0361800	283	332	282	355.5	349.8	343.4	539	578	531	304.4	332.2	285.1	279.75	254.83	293.03	406	305	348.1	338.1	383.8	291.4	707.6	620	569.9	381.3	358.3	390.3	422	417	349	17
Os01g0704400	1381	1331	1523	1387	1404	1518	1147	1228	1226	1263	1308	1216	1151.7	1164.9	1285.4	2830	1596	1497	1350	1487	1451	1470	1159	1093	1469	1426	1421	1288	1301	1376	6
Os01g0142800	52	85.7	72.2	266.8	271.2	223.2	505	517	413	157.8	126.1	94.08	132.8	130.68	119.59	107.5	58.74	63.2	270.3	293.5	191.4	317.2	368.4	320	92.96	59.4	67.98	96.2	135	108	18
Os01g0756300	5.1	0.93	0	1.642	0.904	0	3.13	1.27	11.7	0	0	0	0	0	0	11.94	5.153	3.108	0	0	0	21.44	15.68	26.72	0	0	0	0	2.87	0	9
Os01g0919900	1312	1102	1287	477.8	435.7	418.7	1556	1412	1468	1525	1749	2470	1654.6	1674.6	1545.9	1194	1552	1408	901.1	806.3	804.4	760.1	756.8	1148	1588	2021	1991	1160	1481	1234	20
Os01g0680700	1425	1317	1475	821	808.1	798.4	1517	1489	1550	1511	1593	1464	1662.2	1400.2	1488.9	2125	1478	1426	841.6	950.6	815.7	946.4	1027	1096	1678	1433	1393	1425	1436	1492	13
Os01g0837500	529	665	580	1137	1109	1051	830	941	869	549.2	523	511.8	555.15	634.75	593.97	979.2	556.5	633	1216	1062	1076	729.8	793.9	707.8	447.9	439.4	379.4	594	524	622	10
Os01g0756200	1.02	2.79	0	0	0	4.325	0	2.53	0	0.862	1.096	0.941	0	1.8669	0	0	0	0	0	0.806	0	5.915	0.713	4.333	0	0	0	0	0	0	9
Os01g0362100	4500	3185	4565	1456	1489	1802	1556	1752	1363	3651	3797	4202	7064.5	8082.8	6657.3	14449	7820	5608	3196	2959	3060	922	1035	1356	5103	5353	4862	6153	5907	6199	13
Os01g0227100	2276	2301	2254	9476	10018	11246	4183	4596	4612	2194	2415	2674	3432.1	3058.9	2919.2	6424	3065	2710	9640	11724	13789	3490	3677	4183	3004	2986	2625	2907	2933	3108	10
Os01g0764700	41.8	57.7	69	44.33	43.39	46.71	36.8	39.8	57.4	65.53	40.57	51.75	69.665	45.739	60.189	131.4	54.62	63.2	53.88	45.15	52.6	42.15	28.51	28.89	29.58	53.74	24.12	40.7	57.4	50	6
Os01g0968700	614	634	669	549.2	565.9	625.4	566	603	606	675.1	637	544.7	758.7	703.82	772.96	1218	648.2	640.3	598.2	671.6	596.7	736.4	676.3	576.4	629.6	613.8	583.3	804	837	705	13
Os01g0253900	142	213	206	298	284.8	276	93.9	168	122	96.56	94.29	70.56	139.33	108.28	132.26	250.8	139.1	118.1	184.9	204	207.8	113.9	166	106.2	104.6	84.86	78.95	131	133	153	10
Os01g0761000	5575	5201	6222	2805	2864	2656	2897	3182	2888	6076	6360	8085	6471.3	6942.1	6434.7	10150	7373	7003	4590	3939	5055	1860	1544	2334	6847	7262	7338	5397	6037	5781	3
Os01g0841600	182	187	190	196.2	262.2	304.5	189	171	224	195.7	174.3	165.6	197.02	198.83	166.31	250.8	236	172	268.5	286.2	265.6	193.7	223.8	220.3	214.4	198	241.2	176	235	164	10
Os01g0951100	40.8	49.4	33.4	29.56	18.98	31.14	11	8.86	26.3	25.87	29.6	18.82	11.974	14.002	12.671	11.94	34.01	13.47	15.79	5.644	18.11	28.84	19.24	37.56	19.01	38.66	54.82	28.7	20.1	20	11
Os01g0556800	98.9	70.8	95.9	31.2	45.2	32.01	12.5	22.1	13.1	68.11	61.4	50.8	114.29	76.543	110.08	167.2	53.59	59.05	34.37	12.09	10.35	17.01	20.67	7.945	68.66	47.14	30.7	81.4	60.2	84.1	13
Os01g0125550	89.7	71.7	73.3	110.8	70.51	110.7	117	101	73.2	66.39	92.1	96.9	67.488	76.543	70.485	59.7	86.56	70.45	151.4	104	131.1	129.4	133.3	145.2	80.28	76.37	83.33	80.5	81.3	45	12
Os01g0777900	3.06	2.79	3.23	9.031	9.944	11.25	3.91	8.22	3.46	26.73	21.93	25.4	10.885	37.338	17.423	0	7.214	12.43	10.22	1.613	2.587	7.394	14.97	18.78	20.07	33	61.4	23.1	28.7	27.5	11
Os01g0797800	42.8	21.4	32.3	14.78	11.75	12.98	39.9	25.3	30.4	37.94	31.8	32.93	18.505	18.669	30.887	71.65	35.04	46.62	16.72	13.71	12.07	48.8	33.49	35.39	26.41	28.29	43.86	32.4	17.2	31.7	13
Os01g0633500	0	2.79	0	0	0	0	0	0	0	0	0	0	0	1.8669	0	0	0	0	0	0.806	0.862	0	0	0	0	0	0	0	0	0	14
Os01g0911000	270	251	219	218.4	226.9	243.9	207	229	267	217.3	244.5	325.5	252.54	217.49	190.86	453.8	282.4	214.5	211.8	236.2	246.6	232.2	253.7	251.3	230.3	346	300.4	231	249	209	6
Os01g0954100	0	1.86	0	0	0	0	0	0	0	0	0	0	0	0	0	0	0	0	0	0	0	0	0	0	0	0	0	0	0	0	14
Os01g0349400	610	525	667	350.6	316.4	381.5	309	277	269	418.2	432	398	501.81	449.92	457.76	895.6	478.2	634.1	303.8	297.5	369.9	380.8	371.3	310.6	429.9	413.9	412.3	463	441	457	13
Os01g0594300	1.02	0	0	0	0	1.73	0	0	0	1.724	0	0.941	0	1.8669	0	0	0	1.036	0	0	0	0.739	0	0	0	0	0	0	0	0	7
Os01g0945300	34.7	51.2	25.9	212.6	258.5	223.2	110	149	120	25.87	33.99	47.04	14.151	21.469	24.551	47.76	30.92	33.15	201.6	249.9	256.1	60.63	59.86	80.89	25.35	54.68	43.86	40.7	21	37.5	10
Os01g0916200	643	697	644	816.9	821.7	776.8	725	784	823	730.3	688.5	641.6	619.37	591.81	631.99	1063	622.4	605	606.6	625.7	600.1	799.3	768.2	768.5	708.8	671.3	664.5	778	695	721	6
Os01g0691050	26.5	55.9	71.1	18.88	5.424	22.49	93.2	67.7	50.4	123.3	117.3	66.8	102.32	78.41	94.244	83.59	17.52	41.44	7.432	19.35	3.449	38.45	37.77	23.11	53.87	39.6	67.98	45.3	85.1	54.2	7
Os01g0228600	951	911	945	571.4	592.1	551	651	750	829	1035	1115	1286	1562	1286.3	1411.3	1743	1162	1060	859.3	686.1	807	598.2	604.3	762.7	1156	1170	1011	925	960	1138	13
Os01g0356951	0	1.86	0	4.926	9.944	0	0	1.9	5.53	2.587	0	0	2.177	3.7338	2.3759	0	1.031	0	0	0	0	0	0	0	0	1.886	0	0	0	0	2
Os01g0145000	1.02	0	0	0.821	2.712	1.73	0.78	3.16	6.22	0	0	0	0	0.9335	0	0	0	0	0	3.225	0	2.218	4.988	5.056	1.056	0	0	0.93	0	0	18
Os01g0120400	393	389	423	354.7	386	325.3	269	308	236	338.8	310.3	324.6	327.64	347.24	352.42	597	345.2	345	413.4	309.6	369.9	242.5	269.4	239.8	358.1	354.5	361.8	353	308	350	13
Os01g0911900	162	115	124	240.5	229.6	224	233	178	257	83.63	78.94	96.9	115.38	129.75	125.13	214.9	124.7	114	222.9	226.6	206.1	201.9	214.5	203.7	104.6	131.1	76.75	116	134	119	12
Os01g0116100	1.02	3.73	5.39	2.463	1.808	3.46	0	0	1.38	6.035	0	1.882	0	0	1.5839	0	6.183	1.036	0.929	0.806	3.449	0.739	0.713	0.722	0	2.829	0	5.55	2.87	1.67	1
Os01g0720400	2429	1583	1786	161.7	83.17	211.9	64.2	101	50.4	1626	1881	1559	996	1236.8	1260.8	2030	2287	1548	697.6	356.4	430.2	159.7	270.1	377	1534	2346	2574	1550	1924	1774	3
Os01g0812000	2.04	2.79	6.47	1.642	7.232	1.73	3.13	5.06	3.46	4.311	2.193	2.822	4.3541	3.7338	8.7116	0	6.183	9.324	0	3.225	1.724	10.35	9.977	5.778	2.113	2.829	6.579	6.48	9.56	15.8	1
Os01g0643800	115	90.4	117	113.3	144.6	122.8	146	114	106	100.9	106.4	127	80.55	119.48	99.788	226.9	140.2	122.3	158.9	160.5	178.5	125	112.6	114.8	133.1	116.9	114	144	127	104	6
Os01g0847600	71.4	82.9	64.7	32.02	24.41	19.9	23.5	5.06	15.2	40.52	25.22	46.1	43.541	73.743	40.39	59.7	97.9	84.96	32.51	13.71	24.14	18.48	14.97	19.5	54.93	66.94	65.79	79.6	58.3	71.6	3
Os01g0948100	118	141	121	227.4	282.9	191.2	117	148	132	85.36	117.3	83.73	95.79	90.545	107.71	167.2	76.26	123.3	144.9	162.9	169.9	178.9	158.9	163.9	126.8	99.94	78.95	105	120	128	2
Os01g0836900	0	3.73	0	0	0	0.865	0	0	0	0	0	0	0	0	0	0	0	0	0	0	0	0.739	0	0	0	0	0	0	0	0	14
Os01g0243400	10.2	5.59	9.7	9.031	6.328	6.92	19.6	9.49	11.1	6.035	12.06	9.408	10.885	10.268	6.3357	0	20.61	8.288	13.01	14.51	14.66	11.83	8.552	14.44	6.338	5.657	8.772	5.55	1.91	9.16	15
Os01g0531000	165	147	128	179	165.4	167	276	250	296	187.1	196.3	211.7	191.58	164.29	213.83	406	214.4	163.7	188.6	217.7	226.8	215.2	245.1	212.3	263	236.7	195.2	167	181	179	6
Os01g0978000	174	228	193	411.3	426.7	425.6	396	400	465	166.4	210.5	148.6	154.57	167.09	201.16	346.3	224.7	184.4	370.7	368.5	281.9	393.4	458.9	398.7	204.9	158.4	203.9	234	186	206	12
Os01g0225300	519	522	501	642.8	644.5	581.3	528	646	568	463.9	541.6	447.8	657.47	621.68	582.89	1075	621.4	553.2	585.2	645.8	586.3	516.1	501.7	563.4	543	512.9	625	538	535	613	6
Os01g0907300	470	467	505	511.5	524.3	491.3	519	526	526	545.8	565.7	460.1	523.58	510.6	495.77	919.5	444.2	409.2	497	491	493.2	485	414.8	509.9	546.1	429.9	410.1	420	440	462	6
Os01g0526200	506	496	484	525.4	646.3	518.2	489	495	532	405.2	481.3	503.3	555.15	500.33	475.97	728.4	508.1	510.8	617.8	529.7	556.1	487.3	527.3	429.7	483.8	496.9	543.8	476	502	490	6
Os01g0287700	355	368	431	458.9	513.5	404	427	452	368	362.1	370.6	346.2	370.1	388.32	349.26	429.9	297.8	359.5	321.4	391.9	364.7	380	272.9	340.2	331.7	297.9	335.5	335	381	360	2
Os01g0859400	248	289	253	356.3	390.5	377.2	461	450	431	280.2	251.1	227.7	254.71	252.03	289.86	704.5	250.4	210.3	309.3	301.5	329.4	386.7	389.1	356.1	238.7	238.5	291.7	255	277	285	6
Os01g0608400	30.6	33.5	52.8	14.78	20.79	16.44	15.7	21.5	11.7	16.38	7.675	8.467	2.177	10.268	22.967	35.82	15.46	17.61	13.01	9.675	8.622	34.75	68.41	22.39	14.79	9.428	4.386	27.8	27.7	21.7	17
Os01g0931000	97.9	85.7	74.4	44.33	53.33	34.6	128	125	110	164.7	166.7	97.85	114.29	67.209	90.284	107.5	109.2	116	77.1	87.88	63.8	87.99	136.8	106.9	104.6	145.2	83.33	124	94.7	97.5	5
Os01g0359400	1124	1078	1179	1189	1265	973.2	444	497	424	902.7	991.1	758.3	985.11	860.64	1138.8	1839	975.9	1108	724.6	875.6	914	625.5	486.7	470.2	1006	891	598.7	1101	1276	1156	13
Os01g0107000	553	620	591	748.7	768.4	704.1	633	836	817	539.7	530.7	484.5	703.18	673.95	654.96	1290	602.9	603	628.9	622.4	723.4	720.2	654.2	651.5	624.3	561	506.6	643	625	665	6
Os01g0871900	0	0	1.08	591.1	632.8	490.5	99.4	141	122	0	0	0	0	0	0	0	0	0	452.4	454.7	422.5	190	171	111.9	0	0	0	0.93	0	0	10

Os01g0647800	158	161	166	181.4	147.3	124.6	135	154	125	136.2	131.6	205.1	158.92	164.29	149.68	191.1	176.2	191.7	146.8	205.6	204.3	181.1	144.7	161.1	196.5	185.7	166.7	189	198	173	1
Os01g0635200	1283	942	1157	445	412.2	654	294	300	152	832	803.7	1452	752.17	1061.3	678.71	704.5	1728	1615	548.1	343.5	458.7	211.5	124	427.6	919	1191	1645	792	754	762	3
Os01g0824700	74.4	97.8	85.1	73.07	57.85	73.53	53.2	61.4	49.8	70.7	55.92	33.87	80.55	55.074	80.781	71.65	49.47	56.98	60.38	52.41	59.49	66.54	57.01	79.45	85.57	69.77	74.56	99	73.6	74.1	8
Os01g0965300	4.08	5.59	4.31	5.747	2.712	1.73	3.13	3.8	8.29	3.449	2.193	0.941	4.3541	0.9335	2.3759	0	3.092	6.216	1.858	2.419	4.311	3.697	6.414	2.167	6.338	1.886	0	2.78	0.96	4.17	17
Os01g0795100	0	0	0	0	0	1.73	0	0	0	0	0	0	1.0885	0	0	0	0	0	0	0	0	0.739	2.138	1.444	0	0	0	0	0	0	17
Os01g0210400	3.06	0	0	0	0	0.865	0	0	0	2.587	1.096	0.941	0	0.9335	0	0	0	0	0	0.806	0	2.218	0.713	1.444	0	0	0	3.7	1.91	0	19
Os01g0872500	30.6	31.7	28	125.6	125.7	116.8	133	75.9	84.3	26.73	21.93	46.1	31.567	30.804	42.766	11.94	16.49	15.54	223.9	157.2	163.8	77.64	57.01	63.56	25.35	22.63	24.12	27.8	25.8	25	10
Os01g0249800	0	0	8.62	0	0	0	0	0	0	0	0	0	0	0	0	0	0	1.036	0	0	0	0.739	3.563	0	0	0	0	0	0	0	14
Os01g0725400	7.14	6.52	18.3	4.926	9.944	11.25	17.2	19.6	18	9.484	12.06	14.11	6.5311	15.869	7.9197	11.94	14.43	21.76	12.08	5.644	6.898	19.22	18.53	11.56	9.507	17.91	0	24.1	34.4	14.2	1
Os01g0265900	195	143	136	382.6	345.3	333.9	317	271	309	160.4	169.9	143.9	183.96	180.16	171.06	286.6	128.8	164.7	347.4	395.9	347.5	218.9	238	207.3	132	140.5	114	133	137	170	10
Os01g0909500	352	442	416	888.3	825.3	876.3	503	514	511	326.8	294.9	224.9	400.58	391.12	393.61	931.4	371	373	935.5	986.1	966.5	449.5	440.4	450	303.2	315.9	271.9	382	399	371	10
Os01g0226500	307	258	263	319.4	282.9	302.8	371	294	331	275.9	222.6	261.5	281.93	283.77	296.99	382.1	287.5	290.1	327.9	310.4	324.2	289.8	295	320	269.4	344.1	287.3	258	327	322	6
Os01g0916600	359	342	385	4369	4677	4737	629	550	694	326.8	210.5	396.1	347.24	426.59	263.72	513.5	356.6	321.2	5112	4458	4897	604.1	649.2	559	219.7	321.5	287.3	262	322	232	10
Os01g0659900	14.3	15.8	15.1	8.21	6.328	3.46	3.13	4.43	4.15	16.38	4.386	20.7	8.7082	20.536	15.047	35.82	19.58	19.68	3.716	6.45	7.76	9.612	12.11	28.89	29.58	35.83	37.28	15.7	25.8	20.8	13
Os01g0221800	458	441	468	593.6	665.3	660.9	1240	1197	1222	504.4	600.8	981.3	543.17	420.99	392.82	1194	492.6	537.7	1057	1215	1101	670.6	810.3	944.7	557.8	565.7	695.2	311	427	357	15
Os01g0896300	1.02	2.79	0	3.284	0.904	2.595	1.57	4.43	8.98	0	1.096	0	1.0885	0	1.5839	0	0	2.072	0	0.806	3.449	5.915	7.839	6.5	0	0	6.579	0	0.96	1.67	17
Os01g0656300	1.02	0	0	0	0	0	0.78	1.27	0	0	0	0	0	0	0	0	0	0	0	0.806	0	0	0	0	0	0	0	0	0	0	19
Os01g0730600	1.02	1.86	2.16	0	4.52	2.595	0.78	0	0	1.724	1.096	0	1.0885	0	0	11.94	4.122	2.072	3.716	0.806	0	0	0.713	1.444	0	2.829	0	2.78	0	0.83	6
Os01g0152687	4.08	5.59	0	4.105	6.328	1.73	1.57	0	0	2.587	1.096	3.763	4.3541	4.6673	3.9598	11.94	2.061	3.108	0.929	1.613	0.862	0.739	4.988	0	3.169	0.943	0	4.63	1.91	0	6
Os01g0293200	184	224	202	353.8	294.7	345.2	188	223	196	217.3	196.3	168.4	199.2	236.16	220.96	609	177.3	211.4	269.4	358.8	316.4	239.6	265.8	185.6	210.2	203.7	173.2	270	249	276	6
Os01g0347000	0	3.73	0	0	0	0	0	0	0	0	0	0	0	0	0	0	0	1.036	0	0.806	0	0	2.851	0	0	2.829	0	0	0	0	14
Os01g0805975	1.02	2.79	3.23	0.821	9.04	4.325	13.3	9.49	1.38	0.862	2.193	15.05	8.7082	5.6007	4.7518	0	1.031	3.108	4.645	6.45	2.587	0	4.988	10.11	2.113	0.943	4.386	3.7	2.87	0	15
Os01g0901700	382	253	327	324.3	336.3	340.8	128	202	98.8	194	176.5	175.9	317.85	344.44	297.78	609	526.6	367.8	327	447.5	398.3	208.5	270.8	155.3	278.9	248.9	228.1	422	325	285	13
Os01g0343200	306	302	361	542.7	528.8	529.4	266	291	273	344	319.1	334.9	332	346.31	388.86	632.9	340.1	332.6	537.9	637	552.7	238.1	266.5	240.5	339.1	380	318	344	400	390	16
Os01g0498802	0	0	0	1.642	0	3.46	0	0	2.07	0	0	0	0	0	0	0	0	0	0.929	0	0	0	0	1.444	0	0	0	0	0	0	2
Os01g0926600	85.6	49.4	33.4	22.17	27.12	53.63	15.7	8.22	22.8	35.35	20.83	47.04	18.505	32.671	19.007	47.76	41.22	38.33	32.51	12.09	24.14	34.01	41.33	27.45	17.96	53.74	87.72	18.5	36.3	23.3	11
Os01g0260800	469	245	258	133.8	111.2	133.2	135	89.2	40.8	421.6	300.4	827.9	268.86	451.79	180.57	298.5	312.3	346	192.3	123.4	197.4	69.5	39.19	109.1	201.8	403.5	546	256	292	199	20
Os01g0936100	944	736	829	425.3	396.8	423	510	500	520	775.1	737.9	732.9	770.67	752.36	799.89	1421	932.6	780.1	482.1	462	448.4	604.8	639.2	580	814.5	726	824.5	949	795	830	13
Os01g0118100	1349	1259	1400	1218	1220	1159	771	843	753	1265	1272	1410	1693.7	1469.3	1557.8	3308	1667	1477	1353	1381	1291	821.5	828.1	823.4	1432	1547	1329	1677	1744	1614	13
Os01g0278000	227	163	107	12.31	5.424	8.65	26.6	25.3	28.3	14.66	7.675	31.05	34.833	14.935	15.839	346.3	273.1	149.2	7.432	17.74	47.42	24.4	18.53	113.4	17.96	27.34	30.7	23.1	17.2	12.5	13
Os01g0757200	36.7	19.6	11.9	38.59	19.89	71.8	8.61	10.1	53.9	7.76	1.096	10.35	2.177	1.8669	0.792	0	12.37	22.79	60.38	7.257	40.52	4.436	5.701	12.28	0	0	0	4.63	0	2.5	10
Os01g0858350	162	73.6	109	51.72	28.93	24.22	24.3	58.2	44.2	110.4	85.52	71.5	34.833	40.138	35.638	143.3	91.72	62.16	45.52	19.35	18.11	18.48	33.49	23.83	68.66	49.03	74.56	47.2	48.8	37.5	13
Os01g0837900	1395	1341	1432	1110	1063	1131	871	950	1092	1355	1402	1214	1306.2	1326.4	1568.9	2663	1433	1475	992.1	1121	1054	1136	1203	1116	1613	1469	1408	1579	1610	1541	13
Os01g0592900	126	150	139	390	388.7	354.7	200	246	261	106.9	123.9	88.44	171.99	140.95	128.3	191.1	103.1	136.8	288	331.4	269.9	298	259.4	228.2	118.3	98.06	125	112	151	125	10
Os01g0727800	21.4	17.7	7.54	25.45	18.08	13.84	54.8	17.1	40.1	37.94	25.22	62.09	11.974	13.068	11.088	0	18.55	14.5	32.51	27.41	22.42	43.62	18.53	67.89	17.96	19.8	41.67	20.4	7.65	10.8	15
Os01g0973600	379	330	309	261.9	223.3	257.8	202	180	213	348.3	321.2	349	329.82	317.37	280.36	573.2	426.6	358.5	281.5	295.1	314.7	282.4	202.4	256.4	396.1	491.2	456.1	369	446	442	13
Os01g0684301	1.02	0	2.16	0.821	0	2.595	0	0.63	0	0.862	0	0	1.0885	0	0.792	0	1.031	0	3.716	0	0	0	0	0	5.282	0.943	0	4.63	1.91	0.83	1
Os01g0663051	320	160	141	45.97	21.7	52.77	470	318	25.6	16.38	52.63	231.4	312.41	150.29	72.861	167.2	513.2	93.24	508.1	208	376.8	34.01	15.68	381.3	79.23	524.2	743.4	225	461	102	11
Os01g0142500	351	436	358	195.4	189.8	181.7	337	361	217	536.3	523	538.1	477.86	400.45	515.57	788.1	333.9	342.9	178.4	159.6	151.8	234.4	240.9	216.7	456.3	399.8	414.5	415	521	452	13
Os01g0500500	627	637	651	578	550.5	562.3	675	664	650	794.9	718.1	871.2	726.04	681.42	679.51	967.2	617.3	588.5	604.7	559.6	528.5	521.3	546.6	500.5	662.3	685.4	653.5	709	690	648	13
Os01g0177400	6.12	3.73	3.23	0.821	3.616	3.46	0	0	0	1.724	3.289	4.704	0	1.8669	3.9598	35.82	15.46	6.216	0.929	6.45	2.587	0.739	0	0	9.507	9.428	10.96	6.48	7.65	15	13
Os01g0955550	0	0	0	0	0	0	0	0	0	0	0	0	0	0.9335	0	0	0	0	0	0	0	0	0	0	0	0	0	0	0	0	4
Os01g0235300	157	170	153	224.9	233.2	200.7	379	397	327	229.3	196.3	208.9	201.38	154.95	178.19	298.5	105.1	116	207.2	176.6	189.7	283.9	272.9	320	189.1	180.1	208.3	211	158	180	18
Os01g0738100	72.4	88.5	66.8	47.62	32.54	58.82	21.9	32.3	29.7	69.84	51.53	43.28	53.337	64.408	79.989	155.2	45.34	41.44	26.94	33.86	25	34.01	49.17	41.89	53.87	35.83	65.79	84.2	67.9	51.7	13
Os01g0875400	396	428	353	384.2	409.5	415.2	524	516	525	469.9	421	460.1	432.14	420.05	403.9	668.7	428.7	379.2	398.5	407.2	430.2	454.7	402.6	473.8	429.9	455.4	425.4	319	397	365	6
Os01g0963600	8819	5920	5825	6666	6988	6990	3952	5630	3426	9241	7775	11402	5056.2	14617	8684.7	11284	7562	9973	6144	4712	4416	4697	5067	4515	9896	9242	13462	####	####	9458	3
Os01g0866700	409	323	352	314.4	333.6	365.1	373	302	357	386.3	388.1	456.3	389.69	422.85	381.73	477.6	365.8	356.4	426.4	473.3	449.2	326.8	330.7	338	419.4	446	548.2	344	357	350	20
Os01g0647850	426	348	436	344.8	329.9	327	286	342	330	338.8	347.6	302.9	306.96	280.04	335	704.5	429.7	476.6	278.7	345.1	313	510.2	417.6	327.9	361.3	396	372.8	431	442	424	13
Os01g0195300	63.2	69.9	51.7	1.642	0.904	0.865	4.7	2.53	3.46	22.42	4.386	12.23	18.505	13.068	17.423	47.76	49.47	44.55	1.858	3.225	3.449	7.394	1.425	12.28	15.85	5.657	15.35	3.7	9.56	8.33	14
Os01g0359800	10.2	9.31	11.9	99.34	81.36	74.39	57.9	37.3	43.5	1.724	4.386	5.645	9.7967	5.6007	3.9598	0	16.49	2.072	39.94	57.25	52.6	68.02	50.6	49.11	12.68	4.714	6.579	6.48	10.5	5	12
Os01g0302800	140	141	175	190.5	208.8	188.6	203	259	236	122.4	150.2	139.2	109.94	132.55	102.96	203	152.5	137.8	196	166.1	150	163.4	156.8	183.5	148.9	111.3	105.3	97.1	131	142	12
Os01g0718900	151	181	196	187.2	220.6	217.1	269	216	272	198.3	236.8	231.4	255.8	182.02	216.21	453.8	192.7	218.6	221.1	237	202.6	207.8	196.7	213.1	202.8	197.1	234.6	190	211	240	6
Os01g0262600	259	281	246	270.1	245	257.8	320	308	313	259.5	266.4	319.9	290.63	259.5	342.13	513.5	284.4	329.5	285.2	283.8	248.3	308.3	264.4	307	272.5	296.1	289.5	377	347	317	6
Os01g0777101	156	160	148	201.1	197.1	193.8	275	288	272	246.6	168.8	213.6	158.92	170.82	189.28	226.9	166.9	146.1	247.1	258	251.8	193	215.9	216.7	173.2	192.3	197.4	155	174	168	15
Os01g0737900	308	309	356	302.1	328.1	282	175	181	211	219	230.2	257.8	330.91	350.98	299.36	680.6	336	288	260.1	310.4	287.1	237.3	235.9	192.1	293.7	295.1	269.7	320	313	328	13
Os01g0243450	6.12	3.73	7.54	4.105	9.944	8.65	11	10.8	18.7	2.587	0	0	4.3541	4.6673	1.5839	47.76	4.122	9.324	4.645	4.838	12.07	8.133	7.126	5.056	6.338	5.657	6.579	4.63	3.83	1.67	6
Os01g0798900	14.3	11.2	12.9	15.6	20.79	12.98	27.4	12.7	22.8	15.52	9.868	9.408	14.151	15.869	15.047	47.76	13.4	9.324	9.29	20.16	16.38	20.7	24.23	29.61	8.451	7.543	26.32	12	11.5	12.5	6
Os01g0691600	941	909	963	952.3	940.1	928.2	1167	1129	1263	1201	1254	1243	1144	1180.8	1232.3	1349	884.2	926.2	894.6	849	882	1116	1135	1043	1133	1119	1134	987	964	959	5
Os01g0919600	3331	3862	3878	2368	2396	2471	4326	4540	4043	3772	4304	3300	3087	2790.1	3267.7	6162	3378	3440	2021	2512	2061	4065	3720	4276	3005	3512	2996	3376	4164	3977	6
Os01g0889400	12.2	5.59	8.62	1.642	0.904	0.865	3.91	0.63	2.07	0.862	5.482	4.704	5.4426	6.5342	2.3759	23.88	13.4	5.18	2.787	2.419	6.036	4.436	7.839	4.333	2.113	5.657	8.772	8.33	5.74	5.83	13
Os01g0912600	109	82	97	87.84	74.13	87.37	113	95.5	126	110.4	115.1	103.5	118.65	146.55	126.71	191.1	122.6	130.5	122.6	112.9	110.4	108.7	116.9	90.28	138.4	171.6	127.2	130	124	141	13
Os01g0289100	0	0	0	1.642	0	0	0	0	0	0	0	0	0	0	0	0	0	0	0	0	0	0	1.425	0	0	0	0	0	0	0	17
Os01g0954400	780	765	829	494.2	419.4	467.1	605	728	567	592.3	714.8	429	671.62	705.69	820.48	1911	747.1	838.2	488.6	530.5	469	842.2	873.7	774.2	844	734.5	697.3	995	958	1012	13
Os01g0950300	1.02	0.93	0	3.284	0.904	4.325	0.78	8.22	5.53	2.587	1.096	0.941	0	1.8669	1.5839	0	0	6.216	2.787	5.644	4.311	2.958	3.563	4.333	0	0.943	4.386	2.78	0	1.67	12
Os01g0783700	18.4	17.7	22.6	0.821	0.904	8.65	5.48	0.63	5.53	5.173	0	3.763	4.3541	5.6007	3.9598	23.88	26.79	8.288	2.787	4.031	11.21	8.133	10.69	11.56	5.282	2.829	0	1.85	2.87	0.83	14
Os01g0708400	463	463	459	949.9	988.9	1019	759	786	759	584.6	672.1	627.5	609.57	590.87	540.12	441.8	551.3	497.3	1215	1297	1322	611.5	605.7	723	597.9	579.8	607.4	498	474	496	10
Os01g0937100	0	0	0	0	0	0	0	0	0	0	0	0.941	0	0	0	0	0	0	0	0	0	0	0	0	0	0	0	0	0.96	0	20
Os01g0711500	272	717	264	169.9	147.3	76.12	188	226	215	226.8	254.4	216.4	241.65	238.03	343.71	429.9	315.3	293.2	159.8	133.8	141.4	172.3	157.5	167.6	243	261.2	269.7	282	323	312	14
Os01g0331900	373	389	345	646.1	740.4	657.4	490	457	456	366.4	377.2	282.2	453.91	476.99	432.41	776.2	362.7	353.3	671.6	590.2	771.7	420	410.5	434.1	371.8	302.7	348.7	448	427	452	10
Os01g0327000	0	0	0	2.463	4.52	0.865	2.35	9.49	5.53	0	0	0	0	0	0.792	0	0	0	1.858	4.031	0	0.739	12.11	2.167	1.056	0	0	0	0	0	17
Os01g0919200	0	0	0	0	0	0	0	0	0	0	0	0	0	0	0	0	0	0	0	0	0	0	0	0	0	0	0	0	0	0.83	1
Os01g0886300	1821	1707	1853	1337	1329	1327	555	608	563	1571	1619	1432	1763.4	1602.7	1915.8	3666	1970	1727	1377	1465	1493	733.5	680.6	725.9	1693	1624	1403	1754	1923	1929	13
Os01g0908500	1055	1162	1125	941.7	941.9	897.1	1379	1559	1550	1135	1248	1128	1132.1	1068.8	1189.5	1958	1153	1174	897.4	866.8	988.1	1303	1231	1408	1095	1020	1134	1049	1182	1041	6
Os01g0880200	27.5	31.7	17.2	7.389	10.85	13.84	60.3	43	49.8	9.484	7.675	14.11	11.974	7.4676	10.296	11.94	8.244	5.18	1.858	5.644	6.898	85.03	45.61	72.22	12.68	11.31	17.54	6.48	12.4	15	9
Os01g0686600	0	0	0	0	0	0	0	0	0	0	0	0	0	1.8669	0	0	0	0	0	0	0	0	0	0	0	0	0	0	0	0.83	4
Os01g0784900	69.3	48.4	34.5	52.54	52.43	31.14	32.1	31	29.7	68.11	47.14	82.79	42.452	47.606	50.686	59.7	85.53	56.98	56.67	37.9	50.87	42.88	26.37	36.11	63.38	72.6	87.72	37	61.2	32.5	20
Os01g0243200	335	299	311	179.8	204.3	200.7	202	216	182	299.2	216	296.4	257.98	258.57	294.61	501.5	323.6	285.9	251.7	226.6	229.3	249.2	261.5	199.3	281	297.9	307	402	339	329	13
Os01g0250600	385	319	328	395.7	424.9	446.4	380	307	395	394	341	474.2	449.56	463.93	327.87	573.2	351.4	346	536.9	499.1	533.7	386	347.1	333.7	378.2	422.4	519.7	342	336	323	16
Os01g0865700	13.3	29.8	11.9	6.568	3.616	6.055	5.48	1.27	6.22	12.07	10.96	12.23	5.4426	6.5342	4.7518	23.88	9.275	11.4	9.29	2.419	6.898	12.57	8.552	13.72	12.68	7.543	4.386	10.2	11.5	4.17	14
Os01g0286600	744	768	797	1403	1804	1251	1177	1422	1026	562.1	690.7	769.6	752.17	755.16	627.24	1015	667.8	862	1333	1286	1722	757.1	691.3	913.6	473.3	437.5	475.9	413	530	511	10
Os01g0785300	280	303	317	465.5	545.1	544.1	278	373	332	189.7	265.3	172.2	461.53	336.04	365.89	274.6	353.5	414.4	326.1	423.3	364.7	487.3	354.9	299.7	345.4	231	195.2	395	369	528	2
Os01g0583100	1300	896	1078	651.9	479.1	1004	865	525	815	709.6	580	666.1	854.49	951.19	788.01	2806	1615	1200	1160	1357	1780	451	540.9	569.9	703.5	1087	1118	1168	1498	995	13
Os01g0634600	0	0	0	0	0	0	0	0	0	0	0	0	0	0	0	0	0	0	0	0	0	0.739	0	0	0	0	0	0	0	0	9
Os01g0906425	39.8	43.8	46.3	19.7	15.37	22.49	50.1	32.3	37.3	49.14	57.01	86.56	47.895	65.342	68.901	83.59	52.56	67.34	33.44	48.38	38.8	39.19	32.07	49.11	60.21	59.4	61.4	61.1	51.6	35.8	3
Os01g0975300	871	904	685	385	575.8	334.8	993	1238	490	1228	1167	3153	680.32	728.09	405.49	537.3	542.1	755.3	158.9	99.17	131.1	1402	835.2	1117	645.4	890	1515	585	379	529	20
Os01g0621700	294	280	242	259.4	247.7	260.4	493	423	429	313	281.8	302	239.47	266.97	292.24	382.1	292.7	328.4	293.5	289.5	325.9	277.3	368.4	365.5	301.1	298.9	322.4	308	257	231	18
Os01g0922800	652	640	596	787.3	876.9	780.3	650	697	569	609.6	608.5	770.5	655.29	675.82	645.45	1373	726.5	702.4	927.1	842.6	849.3	425.1	535.9	515	637	646.8	782.9	609	606	626	6
Os01g0970700	4931	4799	5792	4331	5010	4170	2800	4100	2702	6747	7202	6115	11468	8977	10817	13696	7279	5932	5074	5220	4833	2190	2816	2401	7176	6878	5359	7375	7313	8000	13
Os01g0622300	681	722	684	1369	1666	1190	1020	1111	984	555.2	645.8	704.7	660.73	549.8	515.57	1122	529.7	592.6	1221	1081	1277	746	694.1	882.6	487	466.7	379.4	376	416	471	10
Os01g0354700	29.6	27.9	32.3	34.48	45.2	41.52	45.4	36.7	31.8	28.45	35.08	38.57	27.213	28.004	34.055	47.76	38.13	44.55	50.16	49.99	37.94	53.24	49.17	50.56	24.3	51.86	28.51	38.9	48.8	31.7	9
Os01g0720700	454	382	339	73.07	49.72	59.69	189	240	245	482	433.1	604.9	497.45	381.78	417.37	453.8	429.7	490	82.68	110.5	112.1	85.77	83.38	91	400.4	444.1	416.7	299	274	307	3
Os01g0897600	1711	2470	2402	1340	1458	1452	1803	1547	1406	2018	2177	1416	2634.2	3001	2484.4	4609	1634	2090	1190	1103	1123	1051	1050	1098	1526	1375	1382	2522	2927	2474	13
Os01g0174500	7.14	14.9	16.2	13.96	5.424	4.325	0.78	5.06	4.15	5.173	5.482	2.822	4.3541	2.8004	7.9197	35.82	7.214	6.216	2.787	5.644	4.311	5.915	4.276	5.056	8.451	5.657	2.193	8.33	9.56	25	13
Os01g0772150	26.5	21.4	33.4	39.41	39.77	37.2	26.6	38.6	39.4	39.66	44.95	33.87	41.364	45.739	41.974	59.7	31.95	42.48	38.09	41.12	36.21	37.71	42.76	28.89	47.54	45.26	43.86	31.5	31.6	32.5	6
Os01g0149900	886	898	1078	416.2	345.3	353.8	262	287	333	826	891.4	744.2	1005.8	1088.4	1075.5	1934	1188	1056	447.8	405.6	509.6	456.2	417.6	392.2	995.1	963.6	771.9	993	1014	1130	13
Os01g0779100	522	649	639	1011	1135	878.9	665	705	589	562.1	570.1	426.2	554.06	640.35	628.03	1003	460.6	638.2	767.3	1002	751.9	874	673.4	629.8	584.2	528	401.3	711	691	639	10
Os01g0152800	27.5	14.9	19.4	8.21	17.18	10.38	3.13	3.8	8.29	14.66	16.45	12.23	25.036	11.201	28.511	83.59	25.76	21.76	14.86	13.71	27.59	5.915	4.276	2.167	27.47	18.86	17.54	38.9	28.7	26.7	13
Os01g0764600	828	870	855	825.1	853.3	945.5	1302	1139	1275	988.9	1121	985	1226.8	1124.8	1181.6	1564	889.3	952.1	992.1	1074	1091	1026	857.3	1047	978.2	943.8	888.1	1096	950	972	6
Os01g0786800	153	150	123	140.4	171.8	142.7	215	144	137	197.4	188.6	325.5	114.29	162.42	120.38	143.3	126.8	104.6	128.2	71.76	131.1	187.8	176	250.6	150	183.9	254.4	155	154	135	20
Os01g0111900	108	70.8	56	71.42	79.55	78.72	307	154	157	101.7	96.48	147.7	76.196	92.412	72.069	155.2	126.8	84.96	128.2	74.18	113	104.3	79.1	178.4	90.85	132	184.2	71.2	82.2	65	5
Os01g0846500	112	157	140	168.3	184.4	188.6	233	231	191	112.9	115.1	99.73	94.701	93.345	70.485	143.3	49.47	92.21	173.7	141.9	138	232.9	227.3	185.6	73.95	71.66	105.3	103	120	63.3	12
Os01g0209200	59.1	50.3	51.7	83.74	107.6	91.7	112	83.5	69.8	81.05	61.4	68.68	76.196	40.138	59.397	83.59	71.11	55.95	120.8	105.6	133.6	70.98	89.79	59.22	62.33	52.8	87.72	78.6	44.9	70.8	10
Os01g0742200	1.02	0.93	1.08	4.105	1.808	4.325	1.57	0	2.76	0	0	0	1.0885	2.8004	0	0	0	0	2.787	0	0	0.739	0	0.722	0	0	0	0.93	0.96	1.67	2
Os01g0902300	400	323	397	187.2	183.5	134.9	203	230	220	146.6	188.6	161.8	154.57	142.82	210.66	358.2	266.9	275.6	191.4	177.4	184.5	172.3	176.7	200.1	176.4	175.4	142.5	154	177	172	14
Os01g0714800	0	0	0	1.642	0	0	0	0	11.7	0	0	0	0	0	1.5839	0	0	0	0	0	0	0	0.713	0	0	0	0	0	0	0	18
Os01g0826400	1167	1340	847	74.71	48.81	64.88	117	154	124	265.6	152.4	124.2	113.21	129.75	213.83	800	893.5	676.5	65.96	106.4	99.15	435.5	308.6	465.1	119.4	101.8	149.1	112	119	106	14
Os01g0332100	1569	1391	1205	5960	4165	8433	1440	1959	2723	1110	816.8	1148	2879.1	2809.7	2223	1445	1052	1382	8827	10533	12825	2043	2461	2087	1175	1331	1695	1901	1831	1810	10
Os01g0596100	0	0	0	0	0	0	0.78	0	0	0	0	0	0	0	0	0	0	0	0	0	0	0	0	0	0	0	0	0	0	0	5
Os01g0523401	0	0	0	0	0	0	0	0	0	0	0	0	0	0	0	0	0	0	0	0	0	0	0.713	0	0	0	0	0	0	0	17
Os01g0923800	3.06	4.66	3.23	2.463	12.66	1.73	5.48	10.1	8.98	5.173	5.482	0.941	3.2656	1.8669	0	11.94	3.092	4.144	7.432	6.45	2.587	5.915	9.264	5.778	6.338	4.714	2.193	3.7	4.78	5.83	6
Os01g0888700	8.16	2.79	5.39	8.21	5.424	9.516	1.57	6.96	4.84	10.35	1.096	9.408	9.7967	11.201	7.9197	23.88	9.275	5.18	8.361	1.613	4.311	8.133	7.126	7.222	3.169	3.771	4.386	9.25	5.74	10.8	6
Os01g0839100	2.04	0	1.08	1.642	0	3.46	0	0.63	1.38	0.862	2.193	1.882	1.0885	3.7338	0.792	0	4.122	1.036	0.929	0	0	0	1.425	4.333	0	0	0	0	1.91	2.5	19
Os01g0796700	224	238	276	223.3	223.3	207.6	261	235	229	166.4	188.6	114.8	205.73	205.36	201.95	513.5	263.8	226.9	271.3	246.7	252.6	313.5	242.3	274.5	247.2	179.1	199.6	230	227	242	6
Os01g0675933	23.4	30.7	39.9	0.821	0.904	0	0	1.9	1.38	61.22	54.82	81.85	40.275	52.273	41.974	59.7	51.53	59.05	0	1.613	0.862	0.739	0.713	0	60.21	58.46	76.75	36.1	43	37.5	3
Os01g0270300	3.06	0	4.31	0.821	0.904	25.09	1.57	0	0.69	0	0	0	0	2.8004	0	0	7.214	9.324	0.929	0.806	0.862	0	0	0	0	1.886	0	2.78	0.96	0	2
Os01g0798100	1377	1386	1463	986.8	1039	1048	530	614	643	926	1014	860.9	990.55	978.26	902.84	2006	1200	1456	670.7	801.4	801	1230	977.7	780.7	1066	1166	1092	1146	1142	1160	13
Os01g0286500	5.1	5.59	1.08	9.852	18.08	12.98	7.05	8.22	8.98	4.311	2.193	1.882	5.4426	4.6673	3.1679	23.88	2.061	3.108	5.574	10.48	11.21	6.654	2.138	3.611	1.056	5.657	0	1.85	3.83	10.8	6

Os01g0649400	0	0	0	0	2.712	0.865	0	0	0	0	0	1.882	0	0	0	0	3.092	1.036	0	0	0	0.739	0	0	0	0	0	0	0.96	0	2
Os01g0119050	10.2	1.86	2.16	6.568	3.616	17.3	11	1.9	5.53	10.35	5.482	7.527	1.0885	4.6673	0	0	7.214	4.144	12.08	12.09	29.32	7.394	11.4	20.22	4.225	13.2	41.67	3.7	7.65	3.33	11
Os01g0680200	1.02	2.79	0	0	0	0	0	0	0	7.76	3.289	4.704	3.2656	0	0.792	0	0	4.144	1.858	0	0	0	0.713	0	0	3.771	2.193	0	0	0	7
Os01g0330650	0	0	2.16	1.642	0	0.865	0	0	0	0	0	0	0	0	0	0	0	0	0	0	0	0	0	0	0	0	0	0	0.96	1.67	8
Os01g0270100	1272	1188	1351	1004	1186	1230	1464	1357	1294	1492	1352	1479	1363.9	1272.3	1260.8	2400	1190	1122	1340	1317	1290	989.3	1106	1031	1262	1174	1436	1098	1268	1056	6
Os01g0577600	0	0	0	0	0	0	0	0	0	0	0	0	0	0	0	0	0	0	0	0	0	0	0	0	0	0	0	0	0	0.83	1
Os01g0508300	156	162	155	76.35	111.2	83.91	86.9	102	94.7	181.9	190.8	186.3	138.24	171.76	182.15	298.5	161.8	159.5	95.68	109.7	116.4	96.86	106.2	72.95	160.6	202.7	250	184	201	158	13
Os01g0223900	0	0	0	0	0.904	0.865	11.7	0.63	22.8	0.862	0	0	0	0	0	0	0	0	0.929	0	0	11.83	12.11	10.83	1.056	0	0	0	0	1.67	18
Os01g0584800	295	302	292	427.7	450.2	481.8	253	295	252	251.8	260.9	275.7	332	326.71	282.73	537.3	306.1	381.3	414.3	452.3	513.9	266.2	256.5	272.3	272.5	297.9	271.9	323	290	287	10
Os01g0181300	54	39.1	39.9	44.33	66.89	60.55	57.2	45.5	53.2	51.73	49.34	74.32	59.869	68.142	47.518	107.5	62.86	53.87	80.82	70.15	98.29	50.28	43.47	49.83	65.49	71.66	85.52	37.9	56.4	51.7	16
Os01g0820800	262	279	238	276.7	234.1	231.8	251	234	244	236.2	247.8	249.3	301.52	270.7	304.11	525.4	257.6	249.7	235	238.7	268.1	243.3	210.9	247	260.9	256.5	247.8	267	245	306	6
Os01g0290901	0	0	3.23	0	0	0.865	0	0.63	0	0	0	0	0	0	0	0	0	0	0	0	0	0	0	0	0	1.886	0	0	0	0	14
Os01g0253000	2.04	0	0	0	0	0	0	0	0	0	2.193	0	1.0885	0	0	0	0	0	0	0	0	0	0	0	2.113	0	0	2.78	1.91	0	1
Os01g0383100	0	0	0	0	1.808	1.73	0	0	0	0	0	0	1.0885	0	0	0	0	0	0	0	0	0.739	0	0	0	0	0	0	2.87	0.83	2
Os01g0551400	33.6	14	18.3	35.3	32.54	34.6	41.5	24	31.8	27.59	21.93	40.46	13.062	34.538	20.591	83.59	24.73	38.33	56.67	42.73	46.56	22.92	22.09	26.72	12.68	33.94	63.59	30.5	25.8	25.8	6
Os01g0827300	0	2.79	1.08	1.642	0	2.595	0	1.27	0	0	0	0	4.3541	0	0.792	0	0	0	0.929	1.613	0	0	0.713	0	1.056	0	0	0	1.91	2.5	4
Os01g0346400	43.8	39.1	47.4	69.78	59.66	73.53	54	45.5	50.4	61.22	49.34	55.51	53.337	53.207	38.806	83.59	40.19	59.05	94.75	100.8	98.29	26.62	51.31	53.45	70.78	44.31	35.09	37	36.3	37.5	16
Os01g0670200	15.3	14	22.6	31.2	34.35	32.01	39.9	29.1	29.7	14.66	12.06	10.35	9.7967	29.87	14.255	47.76	20.61	11.4	43.66	54.02	20.69	45.1	32.78	42.61	13.73	4.714	6.579	16.7	17.2	5.83	12
Os01g0675100	8929	8179	9391	4202	4508	4428	3644	3559	3318	8804	8269	9317	8778.9	9349.5	8581.7	14460	8613	8173	5575	4216	5579	2007	2277	2533	8361	8278	8649	7127	7467	7341	13
Os01g0133100	0	0	0	0	0	0	0	0	0.69	0	0	0	0	0	0	0	0	2.072	0	0	0	0	0	0	0	0	0	0	2.87	0.83	1
Os01g0279700	7297	6885	7886	6766	7327	7080	1841	2240	1668	7185	7718	7166	9872.9	9260.8	9361	19703	9813	8885	7745	8179	9371	1335	1333	1502	8723	8676	6947	8071	7999	8769	13
Os01g0683550	0	0.93	2.16	0	4.52	5.19	0.78	0.63	1.38	0	0	0	0	1.8669	0.792	0	0	0	2.787	1.613	1.724	0	2.138	0.722	1.056	0.943	0	0.93	2.87	0	2
Os01g0341750	200	265	203	235.6	256.7	238.8	39.9	219	87.8	262.1	361.8	98.79	223.15	232.43	464.88	334.3	243.2	174.1	138.4	163.7	101.7	491	713.3	367.6	570.4	399.8	368.4	455	527	618	1
Os01g0721900	1674	1394	1517	2049	2260	2091	1848	1777	1889	1636	1539	2622	1827.6	1915.4	1418.4	2006	1630	1571	2346	2139	2261	1343	1484	1530	1536	1901	1686	1261	1436	1204	16
Os01g0741200	1.02	2.79	4.31	9.031	17.18	8.65	18.8	6.33	18	1.724	8.771	0.941	2.177	3.7338	5.5438	23.88	1.031	2.072	4.645	8.869	15.52	6.654	6.414	6.5	0	3.771	4.386	1.85	0	1.67	6
Os01g0764950	9.18	18.6	15.1	62.39	64.18	44.12	13.3	20.9	17.3	1.724	9.868	1.882	17.416	0	8.7116	11.94	9.275	12.43	26.94	35.48	27.59	19.22	23.52	13.72	2.113	4.714	6.579	9.25	4.78	19.2	10
Os01g0933900	59.1	64.3	60.3	41.87	46.1	61.42	43.1	60.7	117	61.22	40.57	36.69	58.78	68.142	54.646	95.53	76.26	39.37	52.02	46.76	37.08	125.7	129	92.45	52.82	64.11	74.56	65.7	74.6	56.7	17
Os01g0881900	444	595	544	610	625.5	725.8	371	392	402	393.2	447.3	625.6	589.98	553.54	505.27	967.2	585.3	564.6	765.5	724	1011	398.5	305.7	440.8	394	485.6	399.1	515	553	618	16
Os01g0593100	0	0	0	2.463	0	2.595	6.26	13.3	15.2	0	0	0.941	0	3.7338	0.792	11.94	1.031	3.108	2.787	4.838	1.724	8.133	4.276	11.56	0	0	0	3.7	0.96	0.83	18
Os01g0970400	930	877	914	954.8	1036	1042	809	794	880	951	939.6	1173	1100.5	1192	1013.7	1194	974.9	838.2	1285	918.4	1122	764.5	813.8	792.3	983.5	985.3	1281	908	827	897	16
Os01g0725000	610	483	466	308.7	278.4	276	80.6	73.4	82.9	231.9	243.4	211.7	316.76	260.43	254.22	871.7	512.2	464.1	365.1	382.2	594.1	69.5	71.98	177.7	196.5	169.7	164.5	193	198	209	13
Os01g0665900	5.1	3.73	7.54	96.88	112.1	134.1	59.5	80.3	78.8	5.173	2.193	1.882	5.4426	17.736	3.9598	11.94	8.244	8.288	134.7	104.8	150	59.89	59.86	74.39	4.225	1.886	6.579	2.78	10.5	7.5	10
Os01g0392800	352	343	420	597.7	655.4	586.5	411	479	498	307.8	387	328.3	403.84	372.45	352.42	644.8	381.3	436.2	519.3	668.4	584.6	572.3	498.8	437.7	319	375.3	300.4	417	523	461	10
Os01g0323100	1.02	0	2.16	0.821	0	0.865	2.35	0	5.53	4.311	0	1.882	1.0885	0.9335	0.792	0	5.153	2.072	2.787	3.225	0	0.739	0.713	0	0	0	6.579	5.55	5.74	1.67	11
Os01g0809000	5.1	0.93	2.16	0	3.616	0.865	8.61	0	2.07	0.862	4.386	1.882	2.177	5.6007	1.5839	0	6.183	9.324	0.929	0.806	1.724	0	0	0.722	8.451	8.486	0	3.7	1.91	3.33	1
Os01g0948200	375	427	407	305.4	240.5	230.1	174	90.4	117	569	649.1	732.9	407.11	281.9	342.13	1397	402.9	350.2	234.1	218.5	213.8	127.2	116.2	116.3	499.7	527	368.4	312	418	389	13
Os01g0748900	1275	1014	1085	584.5	611.1	601.2	494	572	583	438.9	432	414.9	545.35	461.13	620.11	1039	776	789.5	529.5	576.5	538	652.1	668.5	621.1	463.7	493.1	620.6	503	505	527	14
Os01g0830700	238	183	182	169.9	146.4	140.1	434	479	455	173.3	156.8	111	123	137.22	160.77	238.8	103.1	116	113.3	125	105.2	663.2	662	606	114.1	116	125	159	134	138	17
Os01g0532300	103	139	121	271.7	293.8	499.1	210	278	171	77.6	63.59	142.1	279.75	299.64	273.23	167.2	255.6	284.9	515.6	306.4	561.3	383	240.2	377	135.2	186.7	239	249	183	191	10
Os01g0557500	2454	2274	2731	306.2	232.3	347.7	921	1091	994	1916	2138	1815	2283.7	1818.4	2129.6	4776	2995	2674	369.7	455.5	371.6	708.3	884.4	950.5	2520	2960	2627	2870	2496	2429	13
Os01g0872650	0	0	0	0.821	0	0.865	0	0	0	1.724	1.096	0	0	0	2.3759	0	0	0	1.858	0	0	0	0	0	3.169	0	4.386	0	0.96	0	11
Os01g0265200	649	659	704	1518	1578	1475	675	696	701	534.6	515.3	430	809.86	630.08	586.85	1087	657.5	676.5	1332	1563	1418	763.8	706.2	792.3	487	465.8	596.5	610	608	654	10
Os01g0934300	535	573	550	640.4	578.5	585.6	539	562	498	508.7	570.1	525	622.63	494.73	648.62	979.2	566.8	612.3	479.3	592.6	561.3	647	654.2	575.6	602.1	581.7	515.3	577	580	681	6
Os01g0615100	3.06	2.79	4.31	22.17	27.12	10.38	7.05	19.6	18	20.69	12.06	6.586	16.328	7.4676	19.007	0	1.031	0	6.503	7.257	0	1.479	24.94	4.333	5.282	0	0	5.55	5.74	11.7	2
Os01g0784450	1.02	0	0	0	0.904	0	0	0	0	0	0	0	0	1.8669	0	0	0	3.108	0	0	0.862	8.133	9.264	5.778	1.056	0	0	1.85	0	0	17
Os01g0819500	690	622	601	537.7	505.3	557.1	1310	1024	1120	998.4	911.1	1138	756.52	740.23	785.63	1003	645.1	627.8	850.9	814.3	766.5	584.1	604.3	824.1	827.1	810.8	929.8	627	664	512	5
Os01g0175850	1.02	3.73	1.08	3.284	4.52	3.46	1.57	4.43	4.15	0.862	2.193	1.882	1.0885	2.8004	2.3759	0	0	0	1.858	1.613	0.862	3.697	5.701	2.167	1.056	2.829	0	0	0.96	0	17
Os01g0956600	12.2	8.38	10.8	17.24	24.41	25.09	35.2	27.8	18	3.449	10.96	20.7	3.2656	11.201	10.296	47.76	11.34	23.83	25.08	21.77	10.35	28.84	39.19	33.22	12.68	7.543	4.386	9.25	21	10	6
Os01g0144500	7.14	5.59	6.47	3.284	1.808	6.92	0.78	2.53	0.69	4.311	5.482	3.763	4.3541	5.6007	5.5438	23.88	7.214	9.324	3.716	3.225	0	3.697	7.126	2.167	5.282	5.657	4.386	6.48	2.87	5	13
Os01g0152900	980	586	859	699.5	524.3	570.9	974	1090	837	872.5	833.3	993.5	701.01	815.84	720.69	1278	812.1	642.3	725.5	616.8	576.8	501.3	607.9	626.2	1110	721.3	833.3	599	697	656	6
Os01g0869200	478	509	477	298	292.9	237.9	237	249	219	538.9	554.8	477.9	534.46	477.93	645.45	764.2	441.1	452.7	331.6	299.9	262.1	219.6	253	197.9	441.6	451.6	447.4	444	449	520	13
Os01g0723100	23.4	34.5	21.6	17.24	18.98	17.3	16.4	17.7	27.6	26.73	14.25	21.64	7.6196	22.403	24.551	35.82	17.52	38.33	13.01	8.063	3.449	39.93	39.91	18.78	16.9	39.6	39.47	24.1	23	21.7	11
Os01g0765300	495	499	509	387.5	421.2	408.3	449	466	475	500.1	502.1	532.5	536.64	495.66	554.38	979.2	494.7	500.4	456.1	434.6	365.6	498.3	511	416.7	533.5	506.3	519.7	553	550	536	13
Os01g0236700	4409	4904	4900	1165	1090	1132	691	852	914	5321	6187	5384	5647.2	4146.4	5494.7	7809	4535	4899	1003	1063	934.6	913.9	889.4	997.4	6212	6732	5059	4130	3617	4435	3
Os01g0929800	12.2	10.2	16.2	32.84	35.25	23.36	3.91	8.86	7.6	7.76	2.193	12.23	9.7967	8.4011	8.7116	0	10.31	13.47	28.8	40.31	32.76	5.915	4.988	10.83	2.113	1.886	0	17.6	16.3	11.7	10
Os01g0960300	892	1053	994	1496	1780	1317	1089	1501	1046	920	994.4	859	1262.7	869.04	984.41	1373	746.1	879.6	1070	1319	1156	1061	975.6	1019	787	643	550.4	751	818	967	10
Os01g0372350	132	147	130	104.3	92.2	101.2	45.4	74.6	49.8	68.11	84.42	58.33	87.082	88.678	87.908	143.3	108.2	135.7	62.24	87.88	74.15	133.8	101.2	72.95	102.5	82.03	85.52	145	122	161	8
Os01g0806200	55.1	65.2	50.6	79.63	100.3	79.58	178	120	185	38.8	64.69	61.15	48.983	82.144	75.237	59.7	64.92	49.73	55.74	62.89	68.98	205.5	153.2	224.6	29.58	66.94	52.63	67.5	79.4	52.5	18
Os01g0523100	0	0	0	0	0	0	0	0	0	0	0	0	0	0	0	0	0	0	0.929	0	3.449	0	0.713	0	1.056	0	0	0	0	0	16
Os01g0695300	4017	3498	4100	1215	1192	1277	1673	1553	1596	3737	4158	3861	4147.3	4609.4	4446.9	6090	4897	4463	2065	1731	2144	1542	1149	1477	4383	5023	5186	4536	4249	4392	3
Os01g0571100	6.12	9.31	4.31	3.284	4.52	5.19	6.26	0	0.69	1.724	0	6.586	0	7.4676	7.1277	11.94	1.031	2.072	6.503	1.613	5.173	5.176	1.425	7.222	6.338	4.714	0	6.48	1.91	7.5	6
Os01g0185250	1.02	1.86	2.16	0	0	0	0	0.63	0	0	0	3.763	0	0.9335	1.5839	11.94	3.092	2.072	0	0	0	0.739	0	0	2.113	0	0	0	0	0	6
Os01g0753000	744	824	739	720.8	772	758.6	1048	999	1087	764.8	885.9	741.4	687.94	812.1	836.32	1134	857.4	872.3	782.2	710.3	873.4	975.3	804.6	1169	849.3	846.7	793.8	880	755	821	18
Os01g0716450	26.5	34.5	33.4	15.6	7.232	12.98	11.7	6.96	1.38	27.59	27.41	30.11	59.869	37.338	38.806	23.88	31.95	25.9	8.361	16.93	14.66	5.915	4.988	13.72	39.09	31.11	32.89	33.3	32.5	31.7	3
Os01g0820000	19.4	9.31	22.6	0	0.904	0.865	0	1.27	0	31.04	20.83	11.29	32.656	20.536	45.934	59.7	8.244	12.43	0.929	0.806	0	0	0	0	31.69	12.26	6.579	20.4	25.8	25	13
Os01g0715400	259	285	303	879.3	938.3	949	926	1198	1191	223.3	262	176.9	363.57	421.92	338.96	835.9	284.4	288	732	841	863.9	1125	1040	1110	276.8	243.3	250	444	504	421	12
Os01g0222800	5.1	4.66	11.9	49.26	65.09	47.58	152	81.6	77.4	17.24	9.868	28.22	6.5311	8.4011	3.9598	11.94	4.122	4.144	67.81	54.02	56.91	95.38	97.63	116.3	8.451	10.37	4.386	1.85	5.74	0.83	12
Os01g0283400	4.08	0.93	3.23	3.284	0	0.865	0.78	0	1.38	0.862	6.578	4.704	8.7082	3.7338	3.1679	0	4.122	7.252	2.787	0.806	0	5.915	3.563	1.444	5.282	3.771	0	12	4.78	0	1
Os01g0300400	4.08	5.59	2.16	3.284	1.808	0.865	0	1.27	0.69	1.724	1.096	2.822	3.2656	4.6673	3.1679	0	3.092	1.036	0.929	4.838	1.724	2.958	0.713	2.167	2.113	4.714	0	7.4	1.91	5	1
Os01g0232400	907	944	958	527.9	530.6	534.6	584	601	652	908.7	893.6	871.2	817.48	818.64	987.58	1039	812.1	809.1	595.5	638.6	600.1	662.5	611.4	686.9	983.5	1025	993.4	791	782	801	3
Os01g0595850	0	0	0	0	0	0	0	0	0	0	0	0	0	0	0	0	0	0	0.929	0	0	0	0	0	0	0	0	0	0	0	16
Os01g0148400	473	529	504	825.9	840.7	816.6	519	559	500	476.8	490.1	401.7	483.3	529.27	477.56	1003	551.3	511.8	784	976.4	771.7	599.6	505.3	532.3	497.5	481.8	467.1	645	546	680	10
Os01g0280200	0	0	0	0	0.904	0.865	1.57	0	0	0	0	0.941	0	1.8669	0	0	0	0	2.787	1.613	2.587	0	0.713	0	0	0	0	0.93	0	0	16
Os01g0615500	0	0	0	0	0	2.595	0	0.63	0.69	0	0	0	1.0885	0	0	0	0	0	0	4.031	0	2.218	2.851	5.056	0	0	0	0	0	0	9
Os01g0382700	1022	1384	1235	2121	2390	2252	3060	3325	3358	1455	2009	1882	1583.8	1224.7	1220.4	1266	993.4	1095	1546	1846	1819	3205	2575	2955	1488	1424	1287	892	1050	1261	18
Os01g0113600	24.5	16.8	10.8	0.821	1.808	1.73	11	8.22	9.67	6.035	5.482	0	3.2656	1.8669	3.1679	0	13.4	11.4	3.716	9.675	0	9.612	9.264	10.11	4.225	1.886	6.579	6.48	5.74	5.83	14
Os01g0229300	130	92.2	100	88.67	92.2	76.12	90	96.8	104	99.15	105.3	97.85	136.06	135.35	129.88	107.5	114.4	102.6	74.32	66.92	85.36	89.47	124	90.28	109.9	98.06	94.3	134	118	119	4
Os01g0276000	365	270	287	361.2	374.2	382.4	799	560	660	458.7	413.3	740.4	401.66	437.79	276.4	155.2	271	316	634.5	527.3	582	375.6	352.8	398	352.8	491.2	506.6	237	295	193	15
Os01g0665750	30.6	27	28	52.54	59.66	39.79	18.8	42.4	30.4	16.38	24.12	5.645	10.885	6.5342	20.591	35.82	26.79	19.68	17.65	16.93	9.484	41.41	82.67	37.56	17.96	27.34	41.67	31.5	21	38.3	17
Os01g0695900	0	0	0	0.821	0	0.865	0.78	1.27	2.07	0	0	0	0	0	0	0	0	0	3.716	1.613	1.724	0.739	2.851	0	0	0	0	0	0	0	12
Os01g0707300	1682	1717	1676	1492	1567	1473	1646	1882	1674	1767	1702	1667	1632.8	1883.7	1862.7	2042	1658	1619	1583	1315	1483	1628	1871	1688	1747	1512	1941	1576	1451	1747	5
Os01g0728300	87.7	74.5	84.1	66.5	79.55	68.34	193	152	214	185.4	108.5	98.79	123	88.678	118	59.7	80.38	79.77	95.68	121.7	97.43	105.7	166	135.1	80.28	68.83	70.17	84.2	94.7	107	18
Os01g0894500	742	720	800	637.9	692.4	636.7	853	817	950	886.3	824.5	777.1	782.65	848.51	788.8	1899	665.7	670.3	709.7	660.3	730.3	750.5	835.9	839.2	820.8	789.2	822.3	695	756	634	6
Os01g0251100	229	181	199	340.7	341.7	375.4	226	182	233	196.6	151.3	227.7	217.7	252.97	157.6	250.8	175.2	206.2	329.8	296.7	324.2	236.6	218.8	210.2	169	204.6	197.4	157	186	162	10
Os01g0236000	71.4	72.7	57.1	17.24	19.89	22.49	18.8	37.3	29	65.53	63.59	62.09	80.55	66.275	64.149	95.53	90.69	69.41	17.65	29.03	28.45	28.84	19.24	26.72	76.06	82.97	50.44	87.9	77.5	75	3
Os01g0804150	2.04	6.52	8.62	0.821	0	0.865	0	2.53	2.07	3.449	1.096	1.882	8.7082	1.8669	3.1679	0	1.031	8.288	0	0	2.587	0	1.425	0	1.056	3.771	4.386	1.85	4.78	8.33	3
Os01g0121100	699	691	662	701.9	688.8	656.6	1476	1188	1354	950.1	888.1	893.8	871.9	750.5	867.2	1075	686.3	665.1	667	660.3	692.4	1020	937.1	979.4	796.5	806.1	760.9	709	644	697	18
Os01g0886200	0	0.93	1.08	0	0	0	0	0	0	0	0	2.822	0	0	0	0	0	0	1.858	0	0	0	0.713	0	0	0	0	0	0	0	20
Os01g0812050	136	113	149	86.2	68.7	82.18	64.2	68.3	92.6	49.14	72.36	95.02	63.134	66.275	86.324	71.65	117.5	124.3	48.31	55.63	49.15	207.8	162.5	158.2	112	124.5	105.3	139	85.1	116	9
Os01g0534700	703	711	871	605.9	584.9	589.1	442	520	417	1519	1260	879.7	1045	997.86	1257.6	2376	686.3	952.1	414.3	572.5	391.4	362.3	593.6	283.1	1266	918.3	842.1	1153	1015	1017	13
Os01g0948133	0	0.93	0	2.463	4.52	3.46	3.13	2.53	4.15	3.449	0	3.763	2.177	0	0.792	0	4.122	0	9.29	8.869	2.587	2.218	4.276	2.167	0	1.886	2.193	0	2.87	1.67	10
Os01g0144300	3.06	0.93	0	0	3.616	0	0	0	0	3.449	0	0	0	0.9335	2.3759	0	0	1.036	0	0	0	0.739	0	0	0	0	0	2.78	5.74	1.67	1
Os01g0205700	222	206	245	254.5	264.9	283.7	535	407	384	305.2	301.5	316.1	244.92	258.57	266.89	453.8	218.5	216.5	328.8	309.6	354.4	236.6	284.3	324.3	245.1	264.9	280.7	214	261	226	18
Os01g0132500	65.2	63.3	69	35.3	25.31	33.74	76.7	63.9	73.9	64.66	52.63	42.34	75.108	56.941	64.941	23.88	72.14	48.69	52.95	35.48	63.8	53.24	69.84	75.11	75	77.31	81.14	73.1	59.3	78.3	5
Os01g0227800	419	337	458	463.9	463.7	500.9	1279	1465	1456	619.9	576.7	664.2	711.89	593.68	696.93	1994	554.4	538.7	644.7	752.3	683.7	788.9	870.8	1008	616.9	640.2	513.1	611	622	651	6
Os01g0127300	1140	1165	1293	1588	1660	1581	1310	1517	1664	1463	1563	1490	1790.6	1536.5	1481.3	1672	1072	1262	1673	1659	1816	1072	1208	1271	1292	1270	1318	871	993	1019	10
Os01g0747400	1023	839	833	873.5	966.3	855.5	719	777	836	633.7	731.3	719.7	788.09	633.81	611.4	1421	904.8	817.4	980	982.1	1186	416.3	425.4	656.5	693	713.7	730.2	554	726	672	6
Os01g0590300	1.02	0.93	0	3.284	6.328	6.055	16.4	11.4	5.53	0.862	3.289	0.941	0	0.9335	1.5839	0	1.031	0	0	0.806	0.862	10.35	2.851	1.444	0	0	0	0	0.96	1.67	18
Os01g0129500	11.2	3.73	2.16	13.14	18.08	23.36	22.7	25.3	14.5	6.035	3.289	0	7.6196	14.935	3.9598	0	6.183	9.324	13.93	4.031	6.898	5.915	10.69	15.89	6.338	9.428	15.35	5.55	8.61	1.67	18
Os01g0900800	457	344	307	324.3	280.2	337.4	1592	900	1864	425.1	369.5	435.6	358.12	396.72	392.02	465.7	412.2	390.6	513.7	390.2	407	1594	1550	1509	503.9	558.2	609.6	380	377	413	18
Os01g0275200	0	0	0	0	0	0	2.35	0	0	0	0	0	0	1.8669	0	0	0	0	0	1.613	0.862	0	0	0	1.056	0	0	1.85	0.96	0	1
Os01g0871350	124	122	151	243.8	254.9	290.7	200	192	205	200	143.6	137.4	108.85	132.55	155.23	143.3	113.4	133.6	262	241.9	259.5	198.2	214.5	221	144.7	151.8	109.6	171	151	163	10
Os01g0881400	117	152	168	128.1	120.2	117.6	156	167	180	150	140.3	112	154.57	116.68	161.56	155.2	147.4	137.8	118.9	147.5	131.9	184.1	148.9	141.6	151.1	141.4	151.3	186	141	168	5
Os01g0108700	0	8.38	0	0	0	0	0	0	2.07	1.724	0	0	0	0	0	0	1.031	0	0	0	0	0	0	0.722	0	0.943	0	0.93	0	0	14
Os01g0806300	43.8	42.8	38.8	83.74	69.61	77.85	59.5	60.1	80.8	28.45	24.12	47.98	41.364	33.604	29.303	11.94	35.04	42.48	93.82	107.2	75.88	64.33	44.9	69.34	44.37	43.37	26.32	19.4	32.5	19.2	12
Os01g0800400	41.8	32.6	31.2	120.7	122	90.83	65	76.5	64.3	31.9	36.18	12.23	17.416	20.536	46.726	35.82	18.55	22.79	88.25	70.15	104.8	61.37	49.88	50.56	24.3	31.11	24.12	23.1	27.7	28.3	10
Os01g0967900	776	833	857	1136	1324	1188	1181	1346	1323	799.2	925.4	840.2	938.3	959.59	917.1	1600	910	907.6	1176	1265	1337	1177	1060	1195	819.7	887.2	769.7	885	970	899	16
Os01g0928300	196	223	165	267.6	276.6	249.1	227	233	188	175	166.7	138.3	200.29	202.56	186.9	334.3	173.1	179.2	209.9	239.5	236.2	271.4	267.2	191.4	173.2	180.1	146.9	204	184	217	6
Os01g0179700	37.7	22.4	17.2	40.23	28.93	40.66	41.5	29.1	27.6	19.83	26.31	32.93	17.416	22.403	21.383	47.76	25.76	25.9	24.15	25.8	29.32	25.88	26.37	25.28	20.07	32.06	37.28	23.1	20.1	31.7	6
Os01g0934100	2406	2513	1687	357.9	229.6	306.2	268	351	280	458.7	307	381	426.7	375.25	435.58	1767	2183	1581	335.4	371.7	625.1	408.1	429	1234	419.4	390.3	379.4	459	442	550	14
Os01g0358700	1.02	1.86	2.16	0	0	0.865	0.78	1.27	0.69	0	0	2.822	1.0885	2.8004	1.5839	0	0	3.108	0	0	0.862	1.479	0.713	0	2.113	1.886	2.193	4.63	0	2.5	1
Os01g0884300	4164	4524	3288	1906	1633	1863	2287	2618	2351	1502	1584	2019	2851.9	2404.6	2713.3	7857	4671	3448	3866	3934	3666	2152	2607	2634	2353	2332	2445	2732	3034	2860	6
Os01g0631800	91.8	58.7	43.1	7.389	1.808	9.516	6.26	6.33	3.46	27.59	7.675	6.586	4.3541	11.201	8.7116	71.65	106.1	42.48	4.645	11.29	14.66	8.873	9.264	51.28	8.451	2.829	2.193	10.2	8.61	8.33	14
Os01g0144900	35.7	32.6	22.6	15.6	14.46	34.6	40.7	28.5	46.3	44.83	38.37	31.05	37.01	44.806	49.894	23.88	28.85	20.72	18.58	25.8	32.76	48.8	29.93	46.95	32.75	18.86	15.35	27.8	37.3	22.5	5
Os01g0354200	23.4	21.4	22.6	14.78	14.46	22.49	19.6	5.06	12.4	22.42	17.54	14.11	8.7082	17.736	23.759	35.82	31.95	15.54	25.08	26.61	38.8	22.18	34.21	30.33	22.18	16.97	17.54	26.8	21	30	6
Os01g0757700	1350	1537	1623	3323	3592	3357	2133	2201	2070	1384	1599	1669	1949.5	1531.8	1393.1	3367	1241	1408	3281	3559	3334	1755	1658	1912	1420	1312	1191	1267	1428	1428	10
Os01g0530366	370	357	327	339.9	317.3	350.3	485	412	441	536.3	504.3	679.3	400.58	505	369.06	597	333.9	347.1	438.5	463.6	475.9	324.6	355.6	420.3	459.5	488.4	515.3	376	335	359	20

Os01g0639200	474	344	360	94.41	66.89	128.9	95.5	84.8	74.6	353.5	264.2	372.6	312.41	462.99	338.17	573.2	688.4	455.9	209	173.4	205.2	159	182.4	291.8	400.4	485.6	546	528	457	410	3
Os01g0167800	155	143	167	86.2	69.61	80.45	45.4	46.2	44.2	100.9	91	86.56	108.85	114.81	95.828	274.6	121.6	140.9	77.1	81.43	80.19	79.85	55.59	67.89	107.7	116	87.72	139	96.6	122	13
Os01g0329400	749	724	797	294.7	242.3	293.3	624	629	762	989.8	744.5	693.4	766.32	771.96	829.98	1457	854.3	808.1	364.1	337	349.2	524.2	553	542.4	850.4	893.8	831.1	708	660	680	13
Os01g0598200	3.06	8.38	8.62	4.926	5.424	0.865	15.7	13.9	11.1	3.449	1.096	0.941	4.3541	1.8669	11.088	0	0	2.072	1.858	1.613	0.862	34.75	34.92	28.17	2.113	8.486	2.193	5.55	7.65	2.5	17
Os01g0513900	4.08	0.93	4.31	3.284	3.616	0	2.35	0.63	0	2.587	2.193	4.704	2.177	4.6673	3.1679	0	2.061	0	0.929	4.031	2.587	0.739	4.988	2.167	0	0.943	0	4.63	0.96	1.67	4
Os01g0760800	0	0	0	2.463	2.712	0	0.78	0.63	6.91	0	1.096	0	0	0	0.792	0	1.031	1.036	1.858	2.419	1.724	0.739	0	1.444	2.113	0	0	0	4.78	1.67	12
Os01g0944100	284	298	380	197.9	173.6	331.3	170	133	200	379.4	266.4	378.2	274.31	262.3	204.33	1779	833.7	767.7	655.8	912.7	882.9	216.6	246.6	157.4	1070	369.6	449.5	884	506	522	13
Os01g0812200	233	153	189	77.99	65.99	75.26	139	183	141	188.8	213.8	192.9	190.49	126.95	174.23	214.9	211.3	179.2	72.46	72.57	68.12	182.6	173.2	191.4	183.8	190.5	155.7	148	150	190	3
Os01g0800300	349	272	306	163.4	181.7	167.8	217	209	238	213	209.4	267.2	188.31	195.09	182.94	286.6	282.4	285.9	162.6	177.4	183.7	156.7	183.9	205.1	204.9	159.3	166.7	162	199	158	14
Os01g0233900	36.7	45.6	46.3	47.62	49.72	48.44	64.2	66.4	38.7	43.11	78.94	52.69	91.436	96.146	83.948	143.3	70.08	44.55	46.45	70.95	56.04	49.54	81.95	65	61.27	49.97	63.59	97.1	76.5	75.8	13
Os01g0692300	991	1053	1144	1655	1422	1672	1589	1946	1633	1553	1664	1532	1802.6	1388	1606.9	2328	1024	1206	1572	1713	1455	1254	1360	1439	1390	1252	1156	1205	1375	1387	6
Os01g0269500	58.1	54	62.5	31.2	43.39	31.14	26.6	14.5	35.9	36.21	59.21	26.34	31.567	38.272	30.095	131.4	70.08	74.59	35.3	39.51	34.49	76.16	42.76	42.61	53.87	50.91	41.67	84.2	99.5	82.5	13
Os01g0154000	1.02	1.86	0	0	0	0	0	0	0	0	0	0	0	0.9335	0	0	0	0	0	0	0	0	1.425	0	1.056	0	0	0	0	0	14
Os01g0569800	4.08	1.86	6.47	6.568	5.424	0.865	7.83	6.96	4.84	13.79	6.578	0	11.974	4.6673	1.5839	11.94	1.031	4.144	3.716	4.031	0	5.176	7.839	9.389	5.282	1.886	0	5.55	1.91	5	7
Os01g0672300	573	719	658	628	605.7	606.4	596	769	701	649.2	563.5	523.1	708.63	607.68	696.14	1099	585.3	649.6	523.9	632.9	419	763	746.8	666.6	772.2	629.8	436.4	804	860	836	6
Os01g0974800	432	467	474	575.5	629.2	518.2	644	697	585	464.7	502.1	406.4	457.18	411.65	507.65	752.3	427.7	473.5	536	554.7	472.5	922.8	763.2	789.4	440.5	408.3	462.7	572	529	476	17
Os01g0242300	127	102	107	115.8	108.5	104.7	114	142	135	113.8	129.4	108.2	90.347	105.48	92.66	167.2	110.3	116	155.1	153.2	129.3	96.12	127.6	132.9	99.3	139.5	116.2	99.9	140	81.6	6
Os01g0974900	3.06	5.59	4.31	4.926	5.424	3.46	10.2	5.69	9.67	2.587	2.193	6.586	7.6196	2.8004	8.7116	11.94	3.092	6.216	0.929	4.031	12.07	17.01	17.1	16.61	5.282	12.26	4.386	7.4	13.4	9.16	9
Os01g0536000	1019	1083	1020	418.7	392.3	467.1	484	519	525	905.3	985.7	1119	1386.8	1475.8	1317.8	2221	1313	1230	448.7	385.4	461.3	468	452.5	572.7	1294	1451	1248	1427	1191	1360	13
Os01g0666900	6.12	6.52	6.47	4.105	6.328	11.25	9.39	8.86	10.4	6.897	8.771	14.11	4.3541	8.4011	4.7518	0	5.153	2.072	4.645	3.225	12.93	4.436	6.414	6.5	7.395	12.26	6.579	0.93	6.69	5	15
Os01g0120800	1937	1813	1758	2208	2420	2199	1794	1929	1870	1817	1616	2255	1937.6	1917.3	1755.8	3630	1697	1843	1772	1654	1716	2013	1997	1668	1845	2088	1958	1589	1782	1598	6
Os01g0688700	1.02	0	0	0	0	0	0	0	0	0	0	0	0	0	0	0	0	0	0	0	0	0	0	0	0	0	0	0	0	0	19
Os01g0976800	64.2	70.8	62.5	97.7	103.1	79.58	79.1	72.7	89.1	89.67	74.55	89.38	84.905	98.012	90.284	71.65	64.92	77.7	79.89	114.5	103.5	57.67	59.86	57.06	65.49	68.83	83.33	65.7	66.9	44.2	16
Os01g0313300	16.3	5.59	19.4	78.81	103.1	59.69	18	29.1	26.9	1.724	1.096	0	0	1.8669	2.3759	0	13.4	12.43	46.45	39.51	51.73	67.28	37.77	35.39	0	2.829	0	2.78	0.96	0.83	10
Os01g0866800	955	624	765	454	439.3	438.6	593	602	486	862.2	684.2	1042	715.16	612.34	791.17	1409	796.6	826.8	569.4	487.8	573.4	506.5	491.7	635.6	899	910.8	1046	700	694	771	13
Os01g0876650	105	102	111	61.57	50.62	71.8	65	91.1	65	87.08	89.9	65.86	111.03	94.279	101.37	179.1	126.8	117.1	68.74	91.92	79.32	51.02	49.17	60.67	53.87	54.68	41.67	98.1	121	116	13
Os01g0310100	264	319	268	194.6	177.2	192.9	209	237	151	204.3	287.3	189.1	204.64	181.09	258.18	441.8	361.7	293.2	207.2	252.4	227.6	246.2	257.3	282.4	219.7	195.2	252.2	426	477	320	13
Os01g0278200	77.5	73.6	61.4	103.4	129.3	117.6	193	214	205	83.63	75.65	95.02	112.12	72.809	95.828	95.53	66.98	83.92	83.61	87.88	82.77	176	159.6	130.7	115.1	97.11	78.95	54.6	68.9	77.5	18
Os01g0335700	234	279	263	226.6	236.8	206.7	323	266	264	275.9	269.7	262.5	226.41	243.63	207.5	394.1	193.7	238.3	158.9	170.1	199.2	317.2	291.5	285.3	281	249.9	212.7	221	204	230	6
Os01g0541600	166	197	172	181.4	154.6	199	329	281	357	229.3	217.1	221.1	256.89	219.36	229.67	370.2	187.6	174.1	217.4	162.1	200	290.6	295.7	285.3	256.7	262.1	309.2	242	213	241	5
Os01g0511400	0	0	0	0	0	0	0	0	0	0	0	0	0	0	0	0	0	0	0	0	0	0	0	0	0	0	0	0	0.96	0	1
Os01g0935500	533	685	528	595.2	638.2	529.4	560	663	689	492.3	652.4	412.1	296.08	300.57	429.25	620.9	345.2	491.1	321.4	428.9	411.3	724.6	625.7	588.6	435.2	443.1	346.5	401	404	408	17
Os01g0365000	26.5	35.4	31.2	46.8	39.77	49.31	39.9	29.1	33.9	45.7	37.28	41.4	17.416	59.741	36.43	35.82	30.92	61.13	27.87	48.38	38.8	58.41	114.7	70.06	38.03	18.86	28.51	56.4	34.4	35	17
Os01g0788700	0	0.93	0	0	0	0	0	0	0	0.862	0	0	0	0	0	0	3.092	1.036	0	0	0	0	0	0	2.113	0	2.193	5.55	0	2.5	1
Os01g0171800	1630	1489	1759	202	181.7	186.9	349	494	374	1367	1488	1178	1605.6	1502.9	1646.5	3642	1767	1525	244.3	199.2	200.9	287.6	337.8	358.2	1520	1464	1564	1484	1465	1734	13
Os01g0856000	184	135	177	217.6	186.2	282	57.9	61.4	42.2	133.6	144.7	178.8	252.54	252.03	159.19	453.8	134	259	213.7	221.7	240.6	102.8	102.6	92.45	165.8	181	210.5	182	205	149	13
Os01g0704300	463	415	351	531.2	541.5	529.4	314	307	298	377.6	351.9	360.3	337.44	313.64	381.73	214.9	444.2	430	421.7	383.8	623.4	231.4	244.4	255.7	256.7	297	355.3	232	242	275	16
Os01g0623000	0	0	0	0	0	0	0	0	0.69	0	0	0	0	0	0	0	0	0	0	0	0	0	0	0	0	0	0	0	0	0	18
Os01g0117600	33.6	35.4	43.1	77.17	65.99	44.12	74.4	72.1	67	62.94	41.66	50.8	33.744	34.538	53.854	35.82	26.79	49.73	94.75	70.95	88.81	61.37	46.32	62.84	44.37	48.08	72.37	37.9	33.5	29.2	15
Os01g0867200	216	246	189	473.7	433.9	431.7	389	363	350	230.2	216	177.8	241.65	272.57	239.97	382.1	193.7	256.9	367.9	368.5	418.2	383.7	363.4	305.5	232.4	154.6	153.5	215	214	241	12
Os01g0930950	61.2	60.5	85.1	26.27	37.97	32.01	39.9	50.6	42.2	50.01	52.63	45.16	44.629	48.539	52.27	35.82	72.14	82.88	18.58	26.61	9.484	72.46	77.68	31.78	45.42	69.77	48.24	70.3	52.6	77.5	1
Os01g0942200	0	0	0	0.821	3.616	0.865	3.13	0	0	0	0	0	0	0	0	0	0	0	0	1.613	0	0.739	0	0	0	0	0	0	0	0	2
Os01g0536400	12.2	7.45	8.62	13.14	11.75	12.11	7.83	6.33	6.22	12.07	8.771	9.408	14.151	16.802	10.296	0	5.153	6.216	9.29	12.09	7.76	6.654	7.839	7.945	6.338	4.714	15.35	4.63	2.87	6.66	4
Os01g0176500	0	0.93	0	0	0	0	0	0	0	0	0	0	0	0	0	0	0	0	0	0	0	0.739	0	0.722	0	0	2.193	0	0	0	11
Os01g0802100	510	664	669	855.5	969.1	882.3	1455	1405	1424	684.6	770.8	528.7	569.3	653.42	548.83	991.1	432.8	510.8	825.8	773.2	657	1001	945.7	976.5	494.4	410.1	486.8	365	516	464	18
Os01g0178200	178	203	193	174	155.5	205	214	154	185	196.6	224.8	202.3	292.81	210.96	251.85	370.2	169	207.2	181.1	158	147.4	193.7	190.3	165.4	251.4	228.2	173.2	220	256	242	13
Os01g0857600	1.02	0	1.08	0.821	0.904	0.865	1.57	1.27	0	5.173	1.096	7.527	0	0	0	0	0	0	0.929	0	0.862	2.958	0	0	0	0.943	2.193	0.93	0	0	20
Os01g0851400	123	148	136	159.3	194.4	172.1	201	214	209	105.2	97.58	119.5	116.47	100.81	117.21	143.3	146.3	106.7	205.3	205.6	228.5	165.6	180.3	197.9	109.9	146.1	140.3	128	146	94.1	12
Os01g0625900	32.6	24.2	17.2	4.926	1.808	10.38	7.05	4.43	4.15	6.035	7.675	25.4	9.7967	6.5342	1.5839	0	12.37	18.65	5.574	5.644	15.52	14.05	9.264	15.17	11.62	7.543	26.32	5.55	15.3	5.83	19
Os01g0639900	####	####	####	18851	17382	19498	####	####	8190	#####	#####	#####	160581	146028	130605	#####	#####	#####	50409	34864	40474	8521	7845	27373	#####	#####	#####	####	####	####	3
Os01g0595725	3.06	0.93	1.08	1.642	6.328	2.595	3.13	8.86	4.15	3.449	1.096	0	5.4426	1.8669	7.9197	23.88	3.092	1.036	1.858	4.031	4.311	6.654	4.988	3.611	5.282	2.829	4.386	6.48	1.91	2.5	6
Os01g0650000	67.3	72.7	51.7	50.08	29.83	40.66	25.1	8.86	31.1	60.35	51.53	34.81	43.541	37.338	44.35	71.65	36.07	37.3	15.79	31.44	28.45	42.88	35.63	42.61	39.09	48.08	37.28	38.9	57.4	31.7	14
Os01g0589900	35.7	37.3	29.1	162.6	196.2	153.1	33.7	44.3	22.8	13.79	16.45	9.408	18.505	13.068	14.255	47.76	34.01	23.83	72.46	99.17	85.36	50.28	68.41	53.45	21.13	12.26	10.96	21.3	44	24.2	10
Os01g0835500	117	115	74.4	192.1	108.5	239.6	9.39	12	13.1	55.18	52.63	34.81	81.639	70.942	107.71	167.2	91.72	104.6	157	114.5	147.4	59.15	44.18	51.28	85.57	74.48	83.33	159	134	148	16
Os01g0834900	802	476	360	89.49	51.53	109.9	162	172	164	112.9	100.9	80.91	66.4	69.075	49.894	131.4	122.6	170.9	96.61	60.47	133.6	147.1	151.1	179.8	31.69	62.23	136	35.2	83.2	35.8	19
Os01g0567600	0	0	0	0	0	4.325	0	0	0	0	0	0	0	0	0	0	0	0	0	0	0	0	0	0	0	0	0	0	0	0	2
Os01g0700900	0	0	0	0.821	0	0	0	0	0	0	0	0	0	0	0	0	0	0	0	0	0	0	0	0	0	0	0	0	0	0	2
Os01g0640600	0	0.93	0	0	0	0	0	0	0	0	0	2.822	0	0	0	0	0	0	0	0	0	0	0.713	0	0	2.829	0	0.93	0	0	20
Os01g0723000	0	0	0	0	0	0	0	0	0	0	0	0.941	0	0.9335	0	0	0	0	0	0	1.724	0	0	0	0	0.943	0	0	0	0	16
Os01g0252900	151	158	122	127.3	127.5	109.9	82.2	85.4	94	117.3	116.2	123.2	97.967	92.412	123.55	203	105.1	125.4	91.97	99.98	97.43	122.7	98.34	104.7	117.3	95.23	83.33	101	93.7	135	13
Os01g0888800	6.12	7.45	4.31	7.389	0.904	0.865	0.78	1.27	14.5	0.862	0	2.822	2.177	0	0.792	0	3.092	1.036	0	0.806	0	19.22	14.25	20.95	6.338	0	0	2.78	1.91	1.67	9
Os01g0679700	1606	1369	1361	1601	1775	1933	3190	2255	3301	1715	1648	2770	1801.5	1920.1	1324.2	3809	1182	1489	2294	1883	2293	1704	1722	1997	1518	1788	2068	1050	1291	931	6
Os01g0616100	963	931	1003	967.9	885.9	945.5	803	798	800	959.6	869.4	844.9	1026.5	947.45	1071.5	1445	971.8	895.1	798	809.5	760.5	826.6	843	897	934.9	909.8	835.5	972	882	1003	13
Os01g0810600	1.02	0	0	1.642	0	0.865	3.91	0.63	0	0.862	0	3.763	0	1.8669	0	0	0	1.036	0.929	0.806	2.587	0	0	1.444	2.113	0	0	0	0.96	0.83	15
Os01g0672400	2571	2425	2375	1225	1207	1265	827	877	916	1168	1049	1081	1538.1	1312.4	1426.3	2185	1914	1872	1144	1174	1225	978.9	1008	911.5	1200	1140	1070	1180	1178	1335	14
Os01g0842400	1.02	0.93	3.23	0.821	1.808	0.865	0	0	1.38	4.311	0	0	3.2656	0.9335	0	0	0	0	0.929	0	0	0	0.713	0	0	0	0	0	0	0	7
Os01g0736100	25.5	21.4	34.5	10.67	6.328	23.36	0	1.9	0	11.21	17.54	17.88	26.124	25.203	22.175	143.3	43.28	41.44	18.58	23.38	6.036	0.739	1.425	0.722	62.33	16.03	28.51	28.7	26.8	34.2	13
Os01g0805900	77.5	70.8	69	266.8	291.1	461.1	48.5	32.3	41.5	100	66.88	45.16	43.541	69.075	42.766	131.4	37.1	47.66	222.9	234.6	200	36.97	28.51	45.5	33.8	43.37	72.37	44.4	33.5	26.7	10
Os01g0206600	66.3	78.2	70	68.96	54.24	70.07	124	87.3	103	108.6	120.6	105.4	119.74	70.009	96.62	95.53	74.2	60.09	80.82	80.63	79.32	69.5	59.86	98.23	84.51	75.43	65.79	84.2	90.9	71.6	5
Os01g0202601	8.16	11.2	10.8	4.926	8.136	9.516	11.7	6.33	6.91	8.622	6.578	23.52	4.3541	19.602	9.5036	11.94	18.55	6.216	3.716	4.031	11.21	15.53	11.4	23.83	11.62	16.03	13.16	15.7	10.5	10.8	9
Os01g0102700	736	869	914	903.1	945.6	1049	1105	1127	1204	982.9	926.5	848.6	712.98	804.64	951.94	1230	776	767.7	1006	997.4	871.7	1355	1337	1237	953.9	932.5	1033	892	987	831	17
Os01g0562600	26.5	33.5	20.5	18.88	15.37	19.9	25.1	26.6	31.1	41.38	31.8	28.22	18.505	21.469	12.671	35.82	16.49	15.54	19.51	11.29	17.24	51.76	52.73	44.06	21.13	26.4	39.47	30.5	27.7	21.7	9
Os01g0301700	58.1	58.7	59.3	150.2	150.1	141	75.2	70.8	67.7	41.38	41.66	21.64	67.488	66.275	57.814	131.4	24.73	43.51	148.6	157.2	144	68.02	52.73	64.28	43.31	23.57	30.7	50	63.1	54.2	10
Os01g0107801	0	0	0	0	0	0	0	0	0	0	0	0	0	0	0	0	1.031	0	0	0	1.724	0	0	1.444	0	0	0	0	0.96	0	9
Os01g0736300	9.18	9.31	8.62	13.96	8.136	4.325	8.61	7.59	7.6	11.21	3.289	17.88	9.7967	17.736	3.9598	0	14.43	13.47	9.29	16.93	5.173	5.176	4.988	9.389	10.56	18.86	21.93	14.8	11.5	13.3	20
Os01g0687300	1081	804	983	1545	1718	1936	626	911	887	452.6	426.5	447.8	607.39	784.1	667.63	2854	1711	1174	2021	2317	2212	1394	1553	1518	679.2	829.7	635.9	1121	961	935	10
Os01g0117400	5.1	13	3.23	0.821	1.808	5.19	11	4.43	6.91	25.87	20.83	31.99	16.328	11.201	24.551	0	18.55	14.5	5.574	11.29	16.38	5.176	2.138	7.945	31.69	15.09	32.89	16.7	10.5	3.33	20
Os01g0763000	311	299	309	406.4	423.1	423.9	330	271	341	311.2	264.2	372.6	288.46	411.65	315.2	513.5	285.5	292.2	552.7	401.5	478.5	377.8	339.9	319.2	285.2	348.9	324.6	249	322	272	10
Os01g0126100	7.14	17.7	4.31	0	4.52	4.325	7.05	1.27	7.6	13.79	10.96	17.88	7.6196	3.7338	8.7116	0	9.275	16.58	5.574	5.644	5.173	17.01	12.83	17.33	9.507	5.657	6.579	12	7.65	11.7	9
Os01g0717650	4.08	0	0	6.568	4.52	5.19	5.48	4.43	0	1.724	1.096	0	1.0885	2.8004	4.7518	0	2.061	2.072	8.361	4.838	2.587	2.218	3.563	12.28	5.282	1.886	4.386	3.7	0	1.67	9
Os01g0721300	26.5	25.1	10.8	10.67	11.75	3.46	7.83	10.8	0	10.35	14.25	31.05	8.7082	14.935	8.7116	0	17.52	10.36	19.51	2.419	6.036	5.176	4.988	1.444	5.282	10.37	8.772	22.2	23	13.3	19
Os01g0925100	38.7	27	31.2	67.32	51.53	63.15	29.7	27.2	29.7	13.79	10.96	7.527	10.885	14.002	30.887	23.88	20.61	13.47	73.39	66.12	46.56	56.19	54.87	36.11	20.07	7.543	24.12	28.7	26.8	12.5	10
Os01g0342500	330	306	503	265.2	242.3	268.2	157	206	131	330.2	370.6	323.6	416.9	807.44	662.88	609	720.3	556.4	547.2	398.3	506.1	72.46	89.08	105.4	538.7	538.4	844.3	488	613	583	3
Os01g0773000	38.7	27.9	31.2	175.7	186.2	190.3	50.9	46.8	33.2	7.76	16.45	0	19.593	16.802	17.423	83.59	16.49	21.76	79.89	142.7	125	70.98	79.82	48.39	16.9	4.714	10.96	16.7	33.5	25.8	10
Os01g0701700	19.4	13	4.31	22.17	28.93	23.36	60.3	35.4	54.6	19.83	10.96	25.4	13.062	6.5342	9.5036	23.88	17.52	12.43	24.15	18.54	9.484	45.1	49.88	33.95	14.79	47.14	125	21.3	30.6	25	11
Os01g0870201	94.8	131	93.7	114.1	126.6	118.5	118	140	154	117.3	140.3	78.09	125.18	132.55	129.09	262.7	111.3	105.7	97.54	121.7	124.2	197.4	173.9	148.1	129.9	105.6	72.37	148	140	135	6
Os01g0802900	14.3	11.2	3.23	13.14	7.232	12.11	43.1	23.4	16.6	16.38	15.35	14.11	2.177	11.201	11.088	11.94	8.244	11.4	19.51	29.03	30.18	17.01	22.8	18.06	7.395	22.63	8.772	17.6	9.56	5	15
Os01g0107900	1908	2163	2027	2880	2956	2787	3315	3337	3628	2256	2718	2908	2345.8	2317.8	2324.4	2973	2091	2316	2498	2707	2656	3433	2915	3459	2568	2685	2517	2034	2155	2183	18
Os01g0805800	492	392	474	625.6	597.5	683.4	705	653	706	503.5	518.6	520.3	362.48	444.32	416.57	585.1	409.1	399.9	665.1	669.2	672.5	797.1	652.8	722.2	367.6	426.2	585.5	471	493	502	12
Os01g0615050	41.8	58.7	56	194.6	203.4	76.99	32.9	131	361	253.5	200.6	77.15	136.06	26.137	286.69	0	2.061	4.144	50.16	123.4	6.036	19.22	289.3	20.22	52.82	16.03	43.86	33.3	191	70	7
Os01g0782200	723	502	792	998.3	895.8	888.4	724	832	648	629.4	532.8	500.5	513.78	436.86	449.84	752.3	557.5	672.4	1057	934.5	1085	397.8	441.8	521.5	403.5	386.6	344.3	389	380	432	10
Os01g0678600	2059	2194	2032	3180	3413	3109	6519	5797	6695	2520	3184	3974	2391.5	1813.7	1607.7	2436	1661	1769	3858	4060	4217	3764	3343	5403	2034	2067	2048	1009	1182	1140	18
Os01g0777400	110	82.9	100	96.05	91.3	106.4	140	148	108	106	148	103.5	85.993	104.55	93.452	0	128.8	92.21	141.2	173.4	219	89.47	114	137.2	92.96	109.4	114	103	92.8	122	15
Os01g0879100	0	0	1.08	0	0	0.865	0	0	2.07	0	0	0	0	0	0	0	0	1.036	0	1.613	0.862	0	0	0	0	0	0	0.93	0	0	16
Os01g0183600	8.16	13	6.47	12.31	9.944	16.44	9.39	13.9	2.76	12.93	6.578	10.35	1.0885	23.336	3.1679	0	11.34	3.108	9.29	3.225	7.76	10.35	9.264	14.44	1.056	0	0	4.63	1.91	4.17	2
Os01g0877600	387	336	434	269.3	303.7	264.7	182	238	214	281.9	282.9	255.9	308.05	293.1	354.01	704.5	421.5	465.2	218.3	270.9	225	596.7	412.6	294.7	353.9	382.8	322.4	451	440	443	13
Os01g0131250	0	0	0	0	0	0	0	0	0.69	0	0	0	0	0	0	0	1.031	0	0	0	0	0	0.713	0	0	0	0	0	0	0.83	1
Os01g0346250	68.3	69.9	42	56.65	84.07	60.55	91.6	68.9	51.1	103.5	81.13	153.4	55.515	42.005	26.927	23.88	52.56	52.84	38.09	9.675	15.52	32.53	42.05	54.17	44.37	48.08	50.44	21.3	39.2	28.3	20
Os01g0318202	1.02	0	0	0	0	0	3.91	0.63	4.15	0	0	0	0	0	0	0	0	0	0	0	0	56.93	18.53	15.17	0	0	0	0	0	0	9
Os01g0826800	5.1	1.86	7.54	2.463	1.808	2.595	8.61	4.43	1.38	1.724	0	9.408	2.177	8.4011	0.792	0	7.214	7.252	1.858	2.419	1.724	4.436	1.425	4.333	2.113	2.829	6.579	0.93	1.91	4.17	20
Os01g0832100	0	0	0	0	0	0	0	0	0	0	0	0	0	0	0	0	0	0	0	0	0	1.479	0	0.722	0	0	0	0	0	0	9
Os01g0636400	2.04	15.8	24.8	3.284	1.808	6.055	0	0	0	2.587	0	0	4.3541	1.8669	4.7518	0	1.031	1.036	11.15	10.48	0	2.218	0.713	0	2.113	1.886	0	0.93	2.87	9.16	14
Os01g0105900	1182	1232	1018	1474	1281	1478	1183	1331	1578	1247	1233	1123	1274.7	1168.7	1291.7	2066	1107	1057	1272	1284	1337	1865	2235	1842	1278	1203	1329	1044	1206	1345	17
Os01g0889000	815	846	942	894	1021	937.7	1068	1093	1179	888.1	884.8	1007	939.39	882.11	852.95	1481	809	893.1	840.7	824	807	808.1	859.4	779.3	820.8	774.1	682	756	777	832	6
Os01g0765200	107	124	115	106.7	124.7	95.16	29.7	45.5	50.4	59.49	75.65	27.28	75.108	69.075	102.16	286.6	112.3	98.42	20.44	47.57	28.45	94.64	85.52	60.67	57.04	53.74	59.21	116	93.7	130	13
Os01g0672100	1.02	0.93	1.08	4.926	2.712	6.055	0.78	1.27	0	4.311	1.096	0	0	0	0	0	6.183	0	3.716	1.613	21.56	2.958	6.414	0.722	0	0	0	1.85	0.96	0	16
Os01g0236800	530	478	530	424.4	436.6	437.7	767	720	826	708.7	755.4	719.7	917.62	911.98	929.77	1349	647.2	559.5	516.5	435.4	557	415.5	439.7	462.2	756.4	703.4	614	646	671	661	13
Os01g0912900	2.04	10.2	2.16	37.76	58.76	51.9	27.4	24	14.5	6.897	8.771	3.763	9.7967	7.4676	0.792	0	1.031	2.072	31.58	54.02	46.56	19.22	15.68	21.67	5.282	0.943	2.193	2.78	4.78	5.83	10
Os01g0195100	0	0	0	0	2.712	0	0	0	0.69	2.587	0	0.941	1.0885	0	0	0	0	0	0.929	0	0	0	0.713	0.722	0	0	0	0	0.96	0	7
Os01g0629400	5198	4873	5380	1647	1651	1609	1725	2155	1728	3401	3496	2561	4039.5	4172.5	4497.6	10735	5285	5122	1717	2167	1920	1920	2292	1879	4207	4118	3515	5376	4544	5074	13
Os01g0806101	2.04	0.93	0	0	1.808	0.865	6.26	3.8	14.5	0	0	0.941	1.0885	0	3.1679	0	0	2.072	6.503	0.806	0.862	6.654	3.563	11.56	0	0.943	4.386	0.93	0	0	18
Os01g0258700	1237	1089	1123	988.5	850.6	928.2	1273	1230	1326	1361	1174	1193	1273.6	1392.7	1331.3	1946	1359	1287	1098	975.6	1008	1213	1074	1241	1292	1191	1364	1430	1455	1366	13
Os01g0647150	0	0	0	0	0	0	0	0	0	0	0	1.882	0	0	0	0	0	0	0	0	0	0	0.713	0	0	0	0	0	0	0	20
Os01g0547133	10.2	9.31	7.54	4.105	4.52	6.055	10.2	2.53	2.07	5.173	7.675	15.99	4.3541	6.5342	10.296	0	10.31	6.216	6.503	8.869	5.173	6.654	9.264	9.389	3.169	12.26	6.579	4.63	13.4	10.8	20
Os01g0621900	4453	3042	4130	1117	911.2	1030	468	545	594	3570	2909	5063	4471.6	3339	3677.9	8132	4115	3945	1441	1421	1303	875.4	911.5	711.4	3717	4228	3285	2953	2923	2777	13
Os01g0694900	251	261	249	175.7	200.7	225.8	163	145	148	136.2	145.8	134.5	125.18	154.95	132.26	203	231.9	232.1	144	149.2	156.1	166.4	169.6	162.5	134.2	116.9	142.5	179	159	188	14
Os01g0165600	0	0	0	0	0	0	0.78	0	0.69	0	0	0	0	0	0	0	1.031	0	1.858	0.806	0	1.479	0.713	0	0	0.943	0	0	0	0	15

Os01g0953400	61.2	72.7	63.6	77.99	65.09	47.58	53.2	59.5	69.1	62.08	66.88	37.63	64.223	36.405	69.693	119.4	53.59	53.87	37.16	56.44	51.73	87.99	76.96	67.89	75	63.17	46.05	62	64.1	74.1	6
Os01g0777600	50	61.5	94.8	69.78	43.39	64.88	128	76.5	69.1	78.46	87.71	66.8	70.754	56.007	60.189	0	119.5	63.2	70.6	150	129.3	45.1	69.13	72.95	81.34	72.6	46.05	51.8	57.4	48.3	15
Os01g0200000	166	167	209	220	282	245.7	257	223	224	214.7	193	265.3	215.53	259.5	205.12	238.8	184.5	189.6	236.9	303.2	294.9	172.3	202.4	208.7	264.1	223.5	274.1	257	221	233	16
Os01g0860700	0	1.86	1.08	0	0.904	0	3.13	1.9	1.38	1.724	2.193	0.941	0	0	0	0	0	0	0	1.613	1.724	2.218	3.563	5.056	2.113	0	0	0	0	0.83	9
Os01g0687400	188	153	216	62.39	66.89	71.8	114	153	161	423.3	264.2	334.9	67.488	106.41	87.908	203	96.87	112.9	68.74	79.02	68.12	171.5	224.5	171.9	133.1	129.2	140.3	50.9	62.2	32.5	7
Os01g0906450	75.4	74.5	62.5	45.15	36.16	36.33	114	90.4	115	110.4	117.3	110.1	96.878	90.545	94.244	214.9	89.66	90.14	51.09	50.8	46.56	66.54	57.72	72.22	82.4	101.8	98.68	68.5	68.9	81.6	13
Os01g0209700	0	0	0	0	0	3.46	0	0.63	0.69	0	0	0	0	0	0	0	0	0	0	0.806	0	0.739	0.713	0	0	0	4.386	0	0	0	11
Os01g0955500	1.02	1.86	0	0	0	0	0	0	0	0.862	0	0	2.177	0.9335	0	0	0	2.072	0	0	0	0	0	0	0	0	0	0	0	0	14
Os01g0723200	850	547	665	891.6	885	981	1037	749	952	889.8	729.1	1486	779.38	993.19	667.63	1528	691.5	719	1393	1234	1461	609.3	643.5	751.9	659.2	1048	1281	594	604	422	16
Os01g0683400	29.6	18.6	14	68.96	51.53	57.09	54.8	35.4	46.3	18.97	13.16	14.11	11.974	16.802	11.088	35.82	18.55	20.72	53.88	47.57	63.8	42.15	35.63	41.89	11.62	24.51	10.96	15.7	14.3	17.5	12
Os01g0502400	881	859	789	890.8	963.6	826.1	799	964	806	849.3	914.4	903.2	935.04	903.58	897.3	1122	897.6	851.6	938.2	891.7	932.1	736.4	809.6	767	821.9	904.2	732.4	826	864	919	6
Os01g0768700	472	435	476	671.6	762	644.5	208	230	194	335.4	333.3	353.7	446.29	418.19	346.09	620.9	378.2	526.3	523.9	703.9	656.1	329.8	353.5	272.3	286.3	363	245.6	429	460	455	16
Os01g0946500	0	0	0	0	0	0	3.91	0	0	0.862	2.193	2.822	0	0	0	0	0	0	0	0	0	0	0	0.722	0	0.943	0	0	0	1.67	5
Os01g0977400	7.14	3.73	6.47	82.92	84.07	80.45	55.6	58.2	42.8	4.311	0	4.704	5.4426	8.4011	3.9598	11.94	0	7.252	43.66	63.7	52.6	42.88	44.18	39	3.169	0.943	8.772	2.78	4.78	1.67	12
Os01g0610400	151	156	136	197	213.3	199	153	145	132	181.9	91	151.5	117.56	168.95	139.39	513.5	121.6	139.9	238.7	212.9	212.1	116.1	140.4	147.3	121.5	135.8	136	125	133	96.6	6
Os01g0641100	243	299	278	305.4	270.3	257.8	381	349	366	293.1	320.1	314.2	324.38	335.11	349.26	609	292.7	251.8	285.2	311.2	319.9	292.8	325.7	271.6	293.7	302.7	388.1	302	374	304	6
Os01g0801100	44.9	41.9	29.1	109.2	120.2	88.23	82.2	106	89.8	36.21	35.08	41.4	55.515	41.072	38.014	47.76	36.07	58.02	91.97	116.1	96.57	76.9	83.38	62.11	30.63	25.46	32.89	37.9	44	55	12
Os01g0928800	467	591	437	811.9	734	581.3	1220	1280	1156	607	611.8	477	466.98	407.92	498.15	406	338	407.2	582.5	578.9	510.4	1309	1326	1203	601.1	531.8	583.3	429	413	489	18
Os01g0740150	0	0	0	0	0	0	0	0	0	0	0	0	0	0	0	0	0	0	0	0	0	0	0	0.722	0	0	0	0	0	0	9
Os01g0938600	142	186	167	134.6	116.6	143.6	211	157	192	224.2	168.8	154.3	120.83	93.345	134.63	47.76	89.66	105.7	131.9	100.8	106.9	220.3	191.7	223.2	112	146.1	140.3	98.1	118	112	9
Os01g0355700	0	7.45	1.08	3.284	3.616	9.516	8.61	4.43	13.1	10.35	26.31	2.822	13.062	9.3345	13.463	0	0	1.036	0	0.806	2.587	14.79	6.414	2.167	1.056	0.943	8.772	6.48	5.74	4.17	7
Os01g0664000	820	915	995	1494	1636	1369	921	1192	1034	745.8	985.7	738.5	1242	861.58	933.73	2030	919.2	1119	1131	1385	1262	1013	954.2	1030	1006	858	625	884	960	1085	6
Os01g0924933	1.02	3.73	0	0	0	2.595	0	0.63	2.07	1.724	4.386	0	0	0	0	0	0	2.072	0	0	0	2.218	2.851	1.444	0	0	0	0	1.91	0	17
Os01g0552850	15.3	19.6	20.5	28.73	28.02	23.36	33.7	39.2	31.8	33.63	24.12	47.98	47.895	27.07	38.806	71.65	38.13	17.61	33.44	37.09	24.14	14.05	24.23	20.95	28.52	45.26	28.51	45.3	31.6	33.3	6
Os01g0212400	552	314	328	291.4	274.8	398.8	168	164	155	187.1	250	202.3	138.24	242.7	201.95	788.1	466.8	358.5	190.4	198.3	301.8	281	294.3	347.4	269.4	343.2	289.5	277	245	257	6
Os01g0282251	0	0	1.08	0	0	0	0	0	0	0	0	0	0	0	0	0	0	0	0	0	0	0	0	0	0	0	0	0	0	0	14
Os01g0860800	216	119	117	491.8	450.2	677.3	146	177	282	81.05	103.1	148.6	101.23	141.88	93.452	143.3	149.4	133.6	473.8	419.3	515.6	460.6	441.8	322.8	134.2	188.6	168.9	114	148	116	10
Os01g0708300	197	183	153	427.7	337.2	382.4	325	318	327	155.2	173.2	142.1	213.35	186.69	201.95	501.5	206.1	180.3	384.6	395.9	428.5	299.5	289.3	296.8	168	194.2	162.3	229	191	198	10
Os01g0823500	33.6	26.1	20.5	2.463	2.712	2.595	0	1.27	2.76	20.69	26.31	19.76	9.7967	24.27	17.423	35.82	48.43	47.66	11.15	11.29	11.21	9.612	15.68	11.56	41.2	65.06	70.17	44.4	53.6	31.7	3
Os01g0302900	8.16	5.59	9.7	1.642	5.424	5.19	7.05	8.86	4.15	3.449	8.771	7.527	2.177	3.7338	6.3357	11.94	4.122	8.288	2.787	0	10.35	1.479	2.851	2.889	2.113	7.543	6.579	7.4	7.65	3.33	13
Os01g0944700	20.4	11.2	3.23	8.21	23.5	3.46	2.35	5.69	15.2	124.2	28.51	78.09	0	12.135	19.799	0	2.061	2.072	0	0	2.587	22.18	0	1.444	4.225	9.428	2.193	0	0	3.33	7
Os01g0829900	2.04	0.93	0	0	0	0	3.13	2.53	2.07	1.724	1.096	0	0	0.9335	0	0	0	2.072	1.858	0	0	0	2.138	0.722	4.225	3.771	2.193	2.78	0	4.17	5
Os01g0889200	2689	2846	3044	1607	1654	1727	1570	1679	1462	1992	2251	2115	2369.7	2376.6	2764.8	4526	2961	2816	1529	1700	1566	2081	1752	1650	2644	2360	2388	2906	2738	3057	13
Os01g0754500	589	712	714	822.6	913	763	864	879	793	763	729.1	696.2	691.21	683.29	692.97	1182	654.4	664.1	798	750.6	723.4	715.7	821.7	743.9	717.3	622.3	725.9	648	649	680	6
Os01g0846000	1053	1134	1143	1240	1319	1209	812	945	767	883.7	1009	877.8	1096.1	860.64	995.5	2054	1032	1109	848.1	1182	976	899.8	835.9	769.9	897.9	831.6	686.4	1063	1185	1171	6
Os01g0834400	248	247	262	259.4	260.3	263	244	248	245	217.3	205	225.8	259.07	247.36	293.03	764.2	291.6	230	319.6	318.5	271.6	235.1	250.8	270.8	248.2	198	307	326	274	300	6
Os01g0600400	137	169	168	220.8	226	190.3	152	105	117	129.3	148	132.7	191.58	119.48	163.14	250.8	132.9	150.2	168.1	169.3	124.2	130.9	119	110.5	136.3	119.7	81.14	128	170	177	6
Os01g0841200	918	740	995	746.3	631.9	703.3	636	688	759	797.5	797.1	804.4	853.4	770.1	827.6	1302	1094	977	765.5	870.8	794.1	712	651.3	756.2	811.3	907	844.3	804	798	822	13
Os01g0595400	922	750	878	734	675.3	737	550	546	524	1017	948.4	896.6	872.99	828.9	936.1	1194	764.7	770.8	714.4	736.9	718.2	479.1	577.2	569.1	956	757.1	686.4	887	766	715	13
Os01g0103600	619	646	654	908	1015	815.7	1218	1384	1232	793.2	683.1	665.2	670.53	576.87	635.16	1063	526.6	527.3	936.4	925.6	838.9	910.9	1132	1049	747.9	595.9	635.9	551	536	532	18
Os01g0952300	523	617	555	713.4	656.3	685.1	745	742	713	626.8	675.4	557.9	714.07	583.41	691.39	740.3	525.6	597.8	463.5	521.7	443.2	774.9	771.8	759.8	680.3	568.5	436.4	612	534	676	17
Os01g0186700	996	1080	1016	834.9	800	765.6	804	833	796	1150	1162	1009	1099.4	1172.4	1198.2	2257	949.1	1089	763.6	809.5	785.5	968.6	954.2	892	1267	1111	1145	1119	1168	1075	13
Os01g0884850	0	0	0	0	0	0	0	0	0	0	0	0	0	0	0	0	3.092	1.036	0	0	0	0	0	0	0	0	0	0	1.91	0	1
Os01g0743300	356	368	355	526.2	589.4	429.9	748	751	771	427.6	356.3	424.3	366.83	384.58	320.75	489.6	311.2	365.7	478.4	463.6	423.3	634.4	737.6	603.8	381.3	303.6	401.3	307	271	317	18
Os01g0957200	56.1	46.6	52.8	64.86	34.35	51.9	142	86	108	81.05	51.53	76.21	77.285	72.809	59.397	119.4	103.1	40.41	106.8	71.76	90.53	117.6	106.9	158.2	68.66	55.63	114	101	95.6	55.8	9
Os01g0741300	0	0	0	0	0	0	0	0	0	0	0	0	0	0	0	0	0	0	0.929	0	0	0	0	0	0	0	0	0	0	0	16
Os01g0973200	8.16	14.9	11.9	36.94	44.29	57.09	81.4	55	71.2	6.897	16.45	2.822	6.5311	2.8004	8.7116	0	6.183	9.324	47.38	50.8	47.42	51.02	37.06	71.5	9.507	8.486	13.16	3.7	12.4	7.5	12
Os01g0924600	760	767	699	882.5	918.4	934.3	1077	1051	1082	956.2	899	859	1010.1	826.1	985.21	1672	890.4	847.5	974.5	1047	945.9	965.6	967	1039	997.2	946.6	894.7	920	935	921	6
Os01g0190000	1250	1410	1461	2732	2895	2215	2798	2526	2294	2115	2282	2224	2596.1	2458.7	2621.4	2221	1491	1360	3075	2937	2933	1270	1097	1312	2234	1673	1739	1533	1894	2212	16
Os01g0205900	0	0	0	0	0	0	0	1.9	0	0	0	0	0	0	0	0	0	0	0	0	0	0	0	0	0	0	0	0	0	0	18
Os01g0834250	535	391	502	327.6	367	297.6	202	295	175	293.1	312.5	302.9	359.21	358.45	358.76	812	569.9	518	326.1	402.3	359.5	358.6	353.5	256.4	404.6	371.5	364	604	533	549	13
Os01g0117050	48.9	84.8	76.5	49.26	47.01	66.61	337	349	286	320.7	239	161.8	105.59	127.88	137.8	47.76	92.75	65.27	52.02	58.05	65.53	224.8	127.6	126.4	124.7	136.7	138.2	99	103	75	5
Os01g0505600	6.12	0	6.47	0	1.808	0	0	0	8.98	4.311	3.289	11.29	2.177	1.8669	5.5438	11.94	8.244	2.072	0	0	3.449	0.739	4.276	1.444	2.113	6.6	10.96	2.78	5.74	1.67	13
Os01g0687600	178	183	181	221.7	230.5	199.8	208	202	209	179.3	175.4	173.1	232.94	222.16	214.62	382.1	269	204.1	265.7	219.3	269	199.6	217.4	197.9	193.3	238.5	184.2	204	235	186	6
Os01g0747800	849	587	789	133.8	130.2	112.5	182	187	227	473.3	451.7	320.8	316.76	336.04	349.26	489.6	578.1	490	144	112.9	134.5	244.7	260.8	328.6	472.2	413	407.9	362	374	426	14
Os01g0768100	19.4	24.2	21.6	9.031	3.616	9.516	10.2	12	12.4	15.52	7.675	6.586	9.7967	15.869	4.7518	0	22.67	15.54	5.574	4.838	12.93	9.612	12.11	15.89	9.507	7.543	15.35	2.78	7.65	5.83	14
Os01g0198200	1084	986	1066	1122	1137	1086	1116	1018	963	1436	1355	1573	1683.9	1625.1	1412.9	3117	1298	1228	1627	1530	1499	728.3	804.6	901.4	1374	1454	1417	1329	1230	1292	13
Os01g0805300	1710	1230	1508	1110	1103	1284	1329	1123	1460	1715	1631	3478	1429.2	1724.1	1165.8	1887	2302	1732	2630	1997	2628	918.3	846.6	1265	1794	2708	2778	1172	1543	1124	20
Os01g0246500	152	112	128	112.5	111.2	133.2	83.8	71.5	78.8	125	102	144.9	163.28	148.42	161.56	203	172.1	167.8	125.4	119.3	112.1	88.73	67.7	59.95	156.3	157.5	177.6	151	132	114	13
Os01g0661700	4.08	0	1.08	2.463	0	1.73	1.57	0	0	0	1.096	0	2.177	0	0.792	0	2.061	1.036	0	1.613	0	0	0	0	0	0	0	0.93	0.96	0	19
Os01g0111200	822	975	900	1062	962.7	938.6	809	900	780	861.3	910	891	880.61	793.43	903.63	1839	815.2	887.9	698.6	716.8	738.9	977.5	998.4	902.8	1029	811.8	932	862	934	942	6
Os01g0559600	1975	3825	2458	5074	7081	3321	5365	7848	5023	2480	3075	2038	2576.5	2054.5	2264.2	4191	2361	2599	4006	2771	2859	4799	4866	4435	2805	2094	2118	2956	3084	3672	12
Os01g0880800	0	0	0	0	0	0	0	0	0	0	0	0	0	0	0	0	0	0	0	0	0	0	0	0	0	0	0	0	0	0.83	1
Os01g0684200	627	693	698	741.3	735.8	685.1	667	745	639	628.5	601.9	602.1	681.41	581.54	675.55	1003	722.4	726.3	771	749	657	740.9	751.8	740.3	668.7	671.3	736.8	782	699	730	6
Os01g0173000	1541	1223	1793	854.6	955.5	883.2	663	858	637	1810	1914	1656	2930.3	2946.9	2797.2	3224	2345	1744	1363	1256	1303	382.3	553.7	507.7	1923	1761	1436	1934	1654	2136	13
Os01g0810700	0	0.93	2.16	0	0	0.865	3.91	5.06	0	0	0	0	1.0885	0.9335	0.792	0	1.031	3.108	0	2.419	0	2.958	2.138	2.167	0	0	0	8.33	0.96	1.67	1
Os01g0633000	6376	5724	7067	5686	6442	5336	9668	9072	8934	6838	7573	8816	9982.8	7855	7819.1	13983	7752	6360	6831	6825	6104	4856	5091	6661	6776	6303	5592	5617	5212	6150	6
Os01g0157400	0	0	0	0	0	0	0	0	0	0	0	0	0	0	0	0	0	0	0	0	0	0	2.138	0	0	0	0	0	0	0	17
Os01g0162500	17.3	17.7	16.2	23.81	27.12	10.38	11.7	8.22	5.53	9.484	8.771	3.763	9.7967	8.4011	13.463	71.65	16.49	9.324	13.01	11.29	7.76	12.57	9.977	5.778	12.68	2.829	2.193	36.1	25.8	15.8	6
Os01g0321900	9.18	14	16.2	4.105	0.904	0	3.13	2.53	0.69	6.035	3.289	1.882	10.885	6.5342	5.5438	11.94	9.275	7.252	1.858	4.031	0.862	26.62	13.54	20.95	6.338	5.657	2.193	6.48	7.65	9.16	9
Os01g0227200	439	400	376	123.1	93.11	150.5	18.8	35.4	23.5	82.77	88.81	68.68	125.18	154.95	152.06	406	406	353.3	60.38	74.98	106.1	96.86	125.4	56.34	177.5	226.3	258.8	476	401	431	13
Os01g0146101	1.02	0	0	0	0	0	0	0	2.07	0	0	0	0	0	1.5839	0	0	0	0	0	0	0	0	0	0	0	0	0	0	0	4
Os01g0594650	1.02	0	0	0	0	2.595	0.78	0	0	0	0	0	0	0	0	0	1.031	1.036	0	0.806	0	0	0	2.889	1.056	0	2.193	0	0	0	11
Os01g0939200	62.2	99.7	90.5	245.5	284.8	187.7	93.2	136	107	93.12	73.46	83.73	90.347	83.077	92.66	131.4	83.47	101.5	156.1	179.8	193.1	98.34	114.7	99.67	129.9	61.28	85.52	84.2	99.5	85	10
Os01g0368900	2.04	2.79	2.16	0.821	0	0	0	1.27	0	0	1.096	4.704	1.0885	0.9335	2.3759	0	6.183	6.216	0	0	0	0	0	0	6.338	2.829	2.193	2.78	1.91	0	3
Os01g0373400	129	106	105	115.8	114.8	121.1	96.3	121	135	121.6	145.8	119.5	131.71	122.28	130.67	155.2	110.3	114	116.1	116.1	110.4	91.68	87.65	93.89	117.3	131.1	100.9	119	124	95	13
Os01g0327900	566	469	512	318.5	337.2	359.9	290	280	290	439.7	474.7	484.5	571.47	595.54	531.41	728.4	669.8	705.5	359.5	408.8	418.2	379.3	349.2	322.1	502.8	619.4	541.7	582	526	517	13
Os01g0746400	194	193	281	45.97	64.18	49.31	42.3	33.5	18	256.1	291.6	244.6	128.45	180.16	171.86	549.3	316.4	215.5	53.88	62.89	82.77	5.915	19.24	23.11	283.1	229.1	206.1	200	186	227	13
Os01g0637600	2082	1567	2149	2061	2094	2045	874	797	770	2116	2170	2217	3237.3	3160.7	2657	3415	2425	2276	2467	2438	2820	729	678.4	801	2135	2318	2085	2008	2022	2077	13
Os01g0591500	1.02	4.66	4.31	2.463	5.424	1.73	4.7	6.96	7.6	8.622	18.64	9.408	4.3541	1.8669	2.3759	0	6.183	5.18	0	0.806	0	12.57	9.264	9.389	6.338	6.6	10.96	0.93	0.96	4.17	5
Os01g0358300	1169	1134	1269	1171	1241	1253	1780	1688	1752	1350	1464	1817	1651.3	1474.9	1326.5	1684	1224	1284	1474	1374	1486	1296	1187	1462	1473	1438	1500	951	1154	1119	15
Os01g0343300	293	221	317	241.4	248.6	239.6	60.3	59.5	63.6	272.5	301.5	507.1	390.78	322.97	310.45	465.7	451.4	320.1	381.8	461.2	396.6	36.23	38.48	44.06	345.4	371.5	361.8	272	305	314	16
Os01g0257900	13.3	14.9	10.8	2.463	9.944	8.65	5.48	7.59	4.84	6.897	9.868	7.527	11.974	14.002	5.5438	47.76	18.55	9.324	13.93	14.51	9.484	31.05	19.24	26	12.68	12.26	8.772	14.8	3.83	15.8	6
Os01g0631100	350	345	314	414.6	376.1	437.7	352	323	375	316.4	328.9	294.5	247.09	308.97	275.6	453.8	281.3	280.8	391.1	358	393.2	357.9	350.6	374.8	300	311.1	313.6	297	288	276	10
Os01g0205300	192	236	207	91.13	94.01	89.96	472	499	567	282.8	381.5	274.7	180.69	199.76	203.54	143.3	204	210.3	73.39	107.2	86.22	251.4	204.5	382.8	221.8	281.9	210.5	162	173	192	5
Os01g0180000	15.3	20.5	28	0	0	0	0	1.27	0	10.35	6.578	3.763	3.2656	10.268	5.5438	11.94	15.46	11.4	0	0	0	0.739	0	0.722	3.169	5.657	0	2.78	3.83	3.33	14
Os01g0898900	345	371	353	212.6	180.8	208.5	400	349	301	348.3	370.6	285.1	275.4	364.05	439.54	1003	385.4	311.8	274	290.3	242.3	317.2	418.3	416.7	327.5	383.7	473.7	500	489	422	13
Os01g0601675	0	0.93	0	4.105	0.904	1.73	0	0	1.38	2.587	0	0	2.177	3.7338	5.5438	0	4.122	0	2.787	6.45	2.587	1.479	2.138	1.444	1.056	6.6	4.386	1.85	5.74	0	16
Os01g0513800	116	115	130	142.8	118.4	136.7	160	131	154	118.1	139.2	218.3	138.24	131.62	124.34	83.59	142.2	115	218.3	167.7	163.8	140.5	131.1	120.6	140.5	163.1	168.9	114	102	112	15
Os01g0252400	2.04	0	0	0	0	0	0	0.63	0	0	0	0	0	0	0	0	0	0	0	0	0	0	0	0	0	0	0	0.93	0	0	19
Os01g0863100	57.1	50.3	50.6	58.29	36.16	72.66	47	49.3	76.7	30.18	41.66	32.93	31.567	17.736	31.679	59.7	50.5	41.44	25.08	24.19	37.94	132.4	100.5	80.17	44.37	52.8	43.86	37.9	40.2	43.3	17
Os01g0391800	29.6	31.7	33.4	28.73	23.5	23.36	26.6	25.3	18	47.42	26.31	45.16	22.859	17.736	21.383	107.5	38.13	30.05	28.8	35.48	36.21	28.84	37.77	26	30.63	33	32.89	37	36.3	34.2	6
Os01g0884500	462	423	483	342.3	270.3	322.7	241	273	236	429.4	428.7	414.9	501.81	489.13	448.25	919.5	529.7	480.7	372.5	396.7	367.3	254.3	258	242.7	447.9	449.7	484.6	506	485	522	13
Os01g0115600	624	922	823	877.6	969.1	621.1	1599	2874	1510	1349	945.1	632.2	761.96	731.83	967.78	871.7	428.7	612.3	376.2	421.7	290.6	1562	1559	1032	684.5	430.9	396.9	878	570	797	18
Os01g0221600	402	220	131	147.8	131.1	100.3	24.3	36.7	35.9	115.5	177.6	70.56	138.24	313.64	304.91	429.9	219.5	415.5	114.3	109.7	48.28	88.73	133.3	65.72	171.1	363.9	390.3	457	535	642	13
Os01g0609000	0	0	0	0	0.904	0	0	0	0	0	0	0	0	0	0	0	0	0	0	0.806	0	0	0	0	0	0	0	0	0	0	10
Os01g0788950	254	203	242	284.9	321.8	292.4	265	253	292	257.8	264.2	345.3	264.51	298.7	217	609	213.3	256.9	240.6	241.9	242.3	311.3	242.3	257.1	237.7	291.3	302.6	159	234	203	6
Os01g0300600	886	905	835	962.2	1034	939.4	895	889	882	790.6	796	716.9	760.88	757.03	822.85	1254	805.9	686.9	858.4	820.8	821.7	835.5	945	866	724.7	706.2	785.1	748	794	745	6
Os01g0892800	228	196	268	5.747	0	21.63	3.13	3.8	2.76	43.11	9.868	31.99	25.036	11.201	14.255	95.53	244.2	200	0.929	2.419	0	36.97	25.65	57.06	25.35	56.57	67.98	37.9	30.6	15.8	14
Os01g0672800	251	220	218	175.7	113.9	231.8	71.2	120	142	213	189.7	189.1	161.1	216.56	241.55	191.1	178.3	187.5	211.8	184.6	174.2	202.6	215.2	187.1	238.7	215.9	230.3	266	241	248	1
Os01g0875500	41.8	15.8	22.6	20.52	24.41	19.03	11.7	15.8	9.67	6.035	8.771	15.05	7.6196	11.201	3.9598	0	15.46	20.72	14.86	20.96	20.69	11.83	7.839	12.28	8.451	5.657	6.579	1.85	17.2	6.66	19
Os01g0218032	1495	1715	1598	2290	2095	2286	1725	2061	1891	1098	1169	969	1274.7	1302.2	1317	2830	1539	1463	1879	1995	2056	2771	2745	2457	1377	1326	1491	1672	1634	1670	17
Os01g0760600	241	265	264	175.7	165.4	192.9	109	118	122	125	111.8	116.7	116.47	128.82	131.47	334.3	184.5	224.8	84.53	97.56	94.84	284.7	307.9	244.8	175.4	151.8	197.4	269	174	256	8
Os01g0210500	2166	1418	1975	1339	1385	1328	828	906	862	2050	1873	2566	2087.8	2094.7	1779.5	2770	1782	1503	2018	1989	1574	542.7	653.5	754	1492	1561	1500	1288	1447	1310	13
Os01g0224166	41.8	27.9	52.8	61.57	41.58	45.85	121	127	95.4	38.8	38.37	55.51	45.718	42.939	53.062	95.53	44.31	33.15	124.5	114.5	132.8	65.81	63.42	117.7	39.09	60.34	74.56	47.2	77.5	31.7	15
Os01g0836400	763	736	812	907.2	999.8	1068	928	801	1002	604.4	649.1	547.6	939.39	791.57	874.33	1636	814.1	904.5	785	1005	1077	1415	1054	1157	749	759.9	666.6	886	810	887	6
Os01g0713600	1.02	4.66	10.8	2.463	4.52	1.73	2.35	1.27	0	12.07	13.16	0	6.5311	2.8004	15.839	11.94	1.031	0	0.929	0	0	0	0	0.722	16.9	0.943	4.386	0	3.83	5	7
Os01g0892300	105	89.4	122	27.09	28.93	43.25	66.5	28.5	60.1	158.6	108.5	81.85	60.957	98.012	56.23	47.76	79.35	97.39	23.22	17.74	27.59	69.5	52.73	87.39	94.02	63.17	105.3	69.4	54.5	57.5	7
Os01g0821700	1079	1014	1016	697.8	698.8	847.7	475	373	476	696.6	594.2	648.2	876.26	1052	744.45	1385	815.2	811.2	837	759.5	985.5	362.3	337.8	387.8	643.3	630.8	703.9	748	971	722	13
Os01g0594800	25.5	18.6	26.9	34.48	47.01	33.74	75.2	46.2	69.8	34.49	42.76	44.22	28.302	38.272	16.631	23.88	17.52	27.97	28.8	30.64	24.14	64.33	31.36	78.72	31.69	57.51	78.95	16.7	27.7	11.7	18
Os01g0549700	1226	1284	1270	1473	1601	1544	1119	1123	1061	1042	1196	1041	1361.7	1477.7	1268.7	2627	1399	1376	1707	1663	1812	1217	1257	1154	1237	1246	1228	1332	1371	1288	6
Os01g0666800	466	522	509	422.8	507.1	487	500	527	532	529.4	519.7	459.1	564.94	544.2	519.53	895.6	518.4	427.9	416.2	429.7	419	542	505.3	535.2	527.1	553.4	423.2	555	483	507	6
Os01g0833400	269	176	207	145.3	83.17	117.6	183	197	157	118.1	112.9	95.02	121.91	81.21	123.55	286.6	218.5	193.7	129.1	150	126.7	172.3	215.2	244.8	119.4	113.1	122.8	111	144	132	14
Os01g0791033	0	0.93	1.08	1.642	3.616	0.865	3.91	5.06	5.53	7.76	1.096	2.822	6.5311	3.7338	7.9197	11.94	4.122	3.108	7.432	3.225	3.449	8.873	2.138	1.444	2.113	1.886	6.579	1.85	4.78	5	6
Os01g0193400	8.16	12.1	10.8	14.78	9.944	12.11	11.7	16.4	24.9	8.622	23.02	15.99	14.151	12.135	13.463	0	11.34	12.43	13.93	13.71	7.76	28.1	7.126	10.11	12.68	12.26	19.74	11.1	8.61	5	15
Os01g0343500	177	193	182	709.3	757.5	673	1172	1273	1198	170.7	203.9	296.4	177.43	196.96	137.8	179.1	217.4	183.4	680	696.6	715.6	805.2	794.6	992.4	194.4	201.8	186.4	134	164	140	12
Os01g0648000	33.6	20.5	25.9	17.24	10.85	11.25	10.2	1.9	15.2	12.93	13.16	5.645	7.6196	6.5342	7.9197	23.88	13.4	25.9	10.22	12.9	5.173	7.394	5.701	15.89	6.338	7.543	17.54	6.48	5.74	6.66	14

Os01g0857250	0	0	0	0	0	0	0	1.27	0	0	0	0	0	0	0	0	0	1.036	0.929	0	0	0.739	0	0	0	0	0	0	0	0	18
Os01g0500600	479	499	509	614.1	642.7	617.6	379	409	380	419.9	478	515.6	461.53	505.93	492.6	1051	529.7	520.1	561.1	703.9	598.4	557.5	436.1	414.6	533.5	510.1	460.5	587	539	586	6
Os01g0543600	7.14	21.4	8.62	72.25	89.49	70.93	24.3	31.6	18	12.07	12.06	6.586	23.947	17.736	31.679	0	0	6.216	9.29	9.675	6.898	22.92	25.65	13	5.282	1.886	6.579	0	3.83	5.83	2
Os01g0348700	1052	877	887	1213	1281	1343	2215	1827	2469	1186	1020	1896	1099.4	1190.2	876.71	1122	871.8	885.8	1432	1220	1400	1369	1365	1405	906.4	1267	1346	593	808	607	15
Os01g0858200	17.3	8.38	8.62	3.284	0.904	6.055	2.35	1.27	3.46	6.897	7.675	2.822	5.4426	2.8004	0	0	4.122	2.072	0.929	1.613	0.862	1.479	1.425	0.722	12.68	0.943	2.193	4.63	5.74	0	19
Os01g0115500	0	0	1.08	0	0	0	1.57	1.27	1.38	0	0	0	0	0	0.792	0	0	0	0	0	0	0	0	0	0	0	0	0	0.96	0	5
Os01g0310032	0	1.86	0	5.747	2.712	2.595	6.26	1.27	2.76	2.587	0	2.822	1.0885	3.7338	1.5839	0	2.061	4.144	1.858	8.063	1.724	10.35	1.425	11.56	3.169	0	6.579	4.63	2.87	0.83	9
Os01g0155500	860	708	783	565.7	521.6	554.5	242	204	254	740.6	702.8	601.2	775.03	698.22	697.72	1254	692.5	754.2	667	842.6	785.5	354.9	402.6	341.6	588.4	689.2	657.9	855	925	944	13
Os01g0159500	0	0	0	0	0	0	0	0	0	0	0	0	0	0	0	0	0	0	0	0	0	0	0	0	2.113	0	0	0	0	0	1
Os01g0110700	62.2	70.8	36.6	18.88	21.7	33.74	16.4	15.2	8.98	31.04	32.89	49.86	29.39	43.872	33.263	83.59	70.08	62.16	19.51	18.54	17.24	29.58	48.46	47.67	54.93	91.46	61.4	68.5	50.7	80	13
Os01g0869500	10.2	14.9	6.47	9.031	14.46	14.71	17.2	13.3	6.91	13.79	14.25	3.763	17.416	5.6007	8.7116	0	13.4	11.4	10.22	9.675	4.311	8.133	12.11	11.56	13.73	7.543	19.74	7.4	6.69	6.66	11
Os01g0686500	0	2.79	0	0	0	0	1.57	1.27	0	0	2.193	0	0	0	0	0	0	1.036	0	0	0	0	0	0	0	2.829	2.193	0	0	0.83	11
Os01g0819100	32.6	24.2	23.7	27.09	27.12	29.41	18.8	15.8	22.1	26.73	23.02	29.17	25.036	15.869	10.296	23.88	15.46	23.83	19.51	26.61	36.21	14.79	18.53	25.28	14.79	23.57	28.51	15.7	18.2	16.7	16
Os01g0920000	294	246	268	238.1	231.4	237.9	375	386	392	389.7	391.4	471.4	303.7	287.5	312.83	346.3	281.3	247.6	310.3	258.8	235.4	330.5	374.8	350.3	377.1	348.9	375	332	293	329	5
Os01g0831200	726	610	783	573.9	579.4	641	752	825	791	713	698.4	695.3	677.06	714.09	696.14	991.1	787.3	666.2	633.5	602.3	682	605.6	651.3	633.4	623.3	652.4	765.3	688	605	691	6
Os01g0949500	3863	2242	3553	308.7	232.3	251.7	108	133	142	2168	1903	2225	990.55	908.25	867.99	2448	3134	2089	588	641	691.5	130.1	151.1	265.8	1733	1847	1728	1082	1292	1467	3
Os01g0369900	19.4	18.6	9.7	3.284	18.98	6.055	13.3	12.7	8.29	11.21	19.74	26.34	22.859	16.802	12.671	0	17.52	11.4	4.645	3.225	6.898	11.09	6.414	10.83	6.338	16.97	28.51	15.7	3.83	7.5	20
Os01g0541900	1032	926	1122	736.4	748.5	723.2	835	903	882	749.2	786.1	671.7	1041.7	1074.4	1148.4	1946	1289	1087	906.7	945	889.8	1061	1033	1015	979.2	851.4	778.5	1288	1109	1254	13
Os01g0913100	54	48.4	31.2	42.69	23.5	19.03	106	70.8	94	43.97	50.43	74.32	32.656	42.005	35.638	59.7	43.28	61.13	38.09	28.22	31.04	48.06	52.73	75.11	29.58	65.06	57.02	38.9	46.9	35.8	5
Os01g0374200	5.1	6.52	3.23	29.56	43.39	32.87	13.3	19.6	9.67	1.724	4.386	3.763	4.3541	5.6007	1.5839	0	6.183	6.216	8.361	33.06	14.66	13.31	14.25	3.611	3.169	0	0	1.85	0.96	0.83	10
Os01g0191400	0	3.73	1.08	4.926	8.136	7.785	3.13	3.8	2.07	0	0	0	0	0.9335	0.792	11.94	0	0	4.645	0	0.862	7.394	7.839	5.056	0	0	0	0.93	0	0	6
Os01g0571500	0	0	0	0	1.808	0	0	0	0.69	0	0	0	0	0	0	0	0	0	0	0	0	0	0	0	0	0	0	0	0	0	2
Os01g0358400	14.3	6.52	3.23	14.78	25.31	17.3	13.3	19.6	25.6	30.18	17.54	35.75	14.151	22.403	8.7116	23.88	14.43	10.36	25.08	29.03	32.76	11.09	14.97	9.389	10.56	16.03	15.35	11.1	14.3	10.8	15
Os01g0106800	757	808	818	1239	1365	1205	1190	1140	1053	961.3	887	950.2	871.9	822.37	826.02	1934	759.5	788.4	1265	1272	1284	859.9	848.7	1037	827.1	804.2	859.6	714	807	699	6
Os01g0603500	209	170	153	234.8	237.7	202.4	84.6	125	74.6	201.8	173.2	156.2	143.68	153.09	163.14	214.9	189.6	210.3	221.1	246.7	205.2	79.85	102.6	80.17	155.3	151.8	144.7	191	161	146	16
Os01g0246601	0	0	4.31	3.284	0.904	0.865	2.35	3.8	9.67	2.587	1.096	0	0	0	0	11.94	3.092	0	1.858	2.419	0.862	0.739	3.563	2.889	1.056	0	0	0.93	0.96	0	6
Os01g0750600	31.6	43.8	22.6	22.99	19.89	34.6	12.5	8.86	15.9	18.11	2.193	20.7	1.0885	9.3345	3.9598	0	19.58	13.47	25.08	16.93	18.97	24.4	23.52	34.67	11.62	14.14	28.51	8.33	12.4	6.66	19
Os01g0201000	186	155	152	139.6	137.4	129.8	202	170	155	174.2	218.2	177.8	193.76	205.36	201.16	155.2	198.9	134.7	187.6	195.9	161.2	116.8	146.1	132.9	203.9	232.9	184.2	161	177	149	20
Os01g0119100	24.5	12.1	16.2	10.67	3.616	44.98	11	2.53	1.38	13.79	7.675	5.645	6.5311	10.268	3.9598	35.82	24.73	13.47	20.44	17.74	37.08	24.4	43.47	53.45	9.507	27.34	76.75	27.8	53.6	4.17	11
Os01g0377000	0	0	0	0	3.616	1.73	2.35	4.43	2.76	0	0	0	0	0	0	0	1.031	0	0	0.806	0.862	1.479	0	0	0	0	0	0	0	1.67	18
Os01g0304300	113	139	98.1	133.8	120.2	117.6	157	163	159	104.3	107.4	128.9	93.613	66.275	106.92	119.4	121.6	97.39	146.8	123.4	127.6	148.6	161.1	132.9	101.4	99.94	118.4	127	106	93.3	18
Os01g0934500	320	287	323	218.4	217	246.5	152	179	120	175	263.1	175.9	191.58	186.69	234.42	441.8	239.1	308.7	108.7	210.4	165.5	292.8	230.2	222.5	292.6	259.3	241.2	371	320	377	8
Os01g0751600	21.4	33.5	53.9	70.6	56.95	57.09	55.6	63.9	57.4	48.28	44.95	37.63	28.302	37.338	39.598	0	42.25	31.08	51.09	66.12	62.94	71.72	73.4	62.84	50.71	55.63	50.44	38.9	56.4	35	12
Os01g0194200	36.7	27	28	32.84	24.41	24.22	31.3	46.8	29	28.45	23.02	22.58	10.885	21.469	31.679	23.88	13.4	18.65	15.79	26.61	19.83	29.58	31.36	37.56	15.85	12.26	24.12	20.4	22	35.8	18
Os01g0966200	123	210	148	536.9	661.7	545	1549	1383	1449	245.7	287.3	453.5	139.33	104.55	95.036	119.4	109.2	127.4	529.5	628.9	607	709.8	732.6	967.8	158.5	151.8	100.9	66.6	63.1	99.1	18
Os01g0800500	1013	674	963	186.4	141.9	316.6	393	288	343	488	655.6	470.4	1134.2	1271.4	1184.8	2806	1413	873.4	482.1	367.7	432	168.6	179.6	298.3	765.9	730.7	802.6	1176	1416	995	13
Os01g0179500	636	667	676	605.1	598.4	561.4	514	638	533	676	729.1	584.2	695.56	538.6	700.1	1481	587.4	739.7	448.7	549.9	518.2	573.8	639.2	584.3	808.1	703.4	625	874	781	837	13
Os01g0252100	1441	1298	1486	1218	1193	1195	1266	1106	1169	1365	1300	1162	1260.5	1252.7	1297.2	2328	1388	1282	1303	1471	1368	1009	1115	1112	1208	1186	1200	1264	1365	1196	6
Os01g0151400	130	154	125	285.7	332.7	256.1	25.1	45.5	18	88.81	76.75	75.27	87.082	105.48	77.613	441.8	170	118.1	333.5	304	256.9	37.71	52.73	34.67	102.5	89.57	96.49	142	145	167	16
Os01g0674000	237	246	245	200.3	222.4	241.3	213	210	228	274.2	239	194.7	251.45	242.7	277.98	489.6	236	198.9	258.2	248.3	299.2	217.4	198.1	259.3	199.7	245.1	225.9	242	225	243	6
Os01g0352100	94.8	142	155	36.94	32.54	1.73	59.5	167	56	363	358.5	193.8	317.85	230.56	464.88	346.3	73.17	131.6	13.93	4.031	7.76	13.31	40.62	13	252.5	109.4	89.91	93.4	149	219	3
Os01g0683600	1397	1416	1449	1510	1496	1567	1681	1529	1600	1455	1433	1286	1492.4	1413.2	1482.6	2305	1317	1333	1570	1524	1584	1674	1685	1598	1465	1424	1375	1408	1314	1415	6
Os01g0602075	9.18	2.79	4.31	10.67	6.328	6.055	14.1	13.3	16.6	10.35	10.96	5.645	15.239	6.5342	7.9197	0	0	0	0	0	0	0	0	0	0	0	0	0	0	0	5
Os01g0955000	264	122	107	104.3	58.76	134.1	18	15.8	15.9	89.67	66.88	116.7	82.728	113.88	76.029	274.6	304	139.9	136.6	120.1	181.9	36.23	53.45	91.73	145.8	179.1	278.5	189	182	171	13
Os01g0727820	12.2	16.8	18.3	19.7	9.04	6.92	19.6	5.69	8.29	18.11	12.06	23.52	13.062	12.135	7.1277	11.94	23.7	20.72	14.86	3.225	12.07	27.36	9.264	52.72	10.56	22.63	17.54	25.9	29.6	20	9
Os01g0661601	181	211	173	105.1	132.9	107.3	164	185	193	286.2	358.5	306.7	375.54	420.99	411.82	453.8	237	238.3	130.1	137.1	188.8	181.9	143.2	203	306.3	330.9	263.2	179	236	268	13
Os01g0733600	182	194	199	232.3	200.7	211.1	314	297	327	275.9	207.2	274.7	214.44	216.56	234.42	226.9	199.9	196.8	268.5	297.5	263	290.6	262.2	296.1	217.6	270.6	239	256	221	165	18
Os01g0307500	8.16	7.45	5.39	3.284	0	1.73	6.26	3.8	0.69	14.66	23.02	43.28	7.6196	10.268	6.3357	0	5.153	15.54	0	0	4.311	0	3.563	2.889	10.56	18.86	10.96	5.55	9.56	4.17	20
Os01g0881800	88.7	111	67.9	221.7	207.9	167.8	943	884	1028	175	178.7	175.9	191.58	90.545	135.43	250.8	53.59	75.63	167.2	151.6	163.8	476.9	365.6	482.5	105.6	64.11	61.4	58.3	51.6	73.3	18
Os01g0139700	9.18	7.45	10.8	23.81	22.6	7.785	14.1	1.27	3.46	9.484	18.64	11.29	1.0885	3.7338	10.296	59.7	14.43	11.4	9.29	10.48	3.449	8.873	14.25	16.61	14.79	8.486	15.35	7.4	8.61	13.3	6
Os01g0346600	12.2	53.1	15.1	19.7	24.41	18.17	18	13.3	14.5	43.11	21.93	15.99	23.947	17.736	10.296	23.88	22.67	14.5	13.93	21.77	15.52	18.48	47.75	28.17	26.41	26.4	19.74	30.5	27.7	23.3	7
Os01g0353900	222	264	226	295.6	326.3	271.6	460	463	410	303.5	296	296.4	252.54	252.97	275.6	394.1	257.6	258	355.8	395.9	392.3	493.2	379.8	456.5	328.5	370.5	350.9	292	234	253	18
Os01g0777650	4.08	8.38	8.62	5.747	13.56	16.44	18	14.5	22.8	1.724	9.868	2.822	5.4426	11.201	12.671	0	7.214	3.108	10.22	23.38	5.173	22.92	9.264	10.11	12.68	7.543	10.96	16.7	4.78	20.8	18
Os01g0712500	3.06	9.31	3.23	0.821	3.616	2.595	2.35	4.43	2.76	1.724	0	0.941	5.4426	3.7338	1.5839	0	4.122	2.072	5.574	0	0	0	0	2.889	2.113	3.771	2.193	0	0	4.17	14
Os01g0558850	203	200	259	284.1	282	253.5	55.6	72.1	51.8	87.08	116.2	48.92	213.35	124.15	167.9	477.6	152.5	225.9	95.68	133	85.36	195.9	173.9	108.3	170.1	122.6	120.6	266	199	261	8
Os01g0613500	1.02	0	0	3.284	1.808	19.03	0	0	3.46	0	0	0	0	0	0	0	0	0	2.787	0	1.724	2.958	6.414	0.722	0	0.943	0	0	0	0	2
Os01g0850700	4.08	3.73	1.08	0	0	0	11	3.8	1.38	1.724	5.482	16.93	1.0885	0	0	0	4.122	2.072	0	0	0	0	0	11.56	2.113	3.771	8.772	0	0	0	20
Os01g0182500	144	103	109	49.26	64.18	44.98	76.7	82.9	83.6	62.08	74.55	86.56	84.905	95.212	104.54	119.4	83.47	102.6	42.73	41.93	45.7	90.94	87.65	70.78	82.4	78.26	118.4	62.9	71.7	66.6	14
Os01g0543100	5.1	1.86	5.39	2.463	5.424	6.055	5.48	3.8	3.46	1.724	2.193	5.645	0	3.7338	1.5839	0	4.122	3.108	4.645	4.838	7.76	6.654	5.701	18.78	3.169	0.943	0	3.7	3.83	2.5	9
Os01g0199300	0	0	0	0	0	0	0	0	0	0	0	0.941	0	0	0	0	0	0	0	0	0	0.739	0	0	0	0	0	0	0	0	20
Os01g0855000	13.3	15.8	14	25.45	22.6	18.17	25.1	31.6	35.2	4.311	4.386	13.17	4.3541	14.002	4.7518	155.2	7.214	16.58	21.37	20.16	31.9	10.35	22.09	14.44	7.395	13.2	8.772	16.7	23.9	6.66	6
Os01g0227700	1.02	4.66	6.47	3.284	2.712	0	22.7	25.3	34.6	7.76	0	0	2.177	0.9335	4.7518	11.94	0	0	0.929	0	0	42.15	25.65	38.28	6.338	0	0	4.63	5.74	2.5	18
Os01g0218100	89.7	99.7	84.1	110.8	96.72	76.99	104	114	120	84.49	60.3	89.38	104.5	63.475	112.46	71.65	68.02	67.34	63.17	66.12	51.73	153.8	296.5	150.9	64.44	80.14	81.14	124	119	75	17
Os01g0641750	0	0.93	0	0	0	0	0	0	0.69	0	0	0.941	0	0	0	0	2.061	0	0	0	0.862	0	0	0	0	0	2.193	0	0	0	11
Os01g0272600	26.5	41	20.5	37.76	27.12	34.6	37.6	34.2	31.8	48.28	33.99	47.98	13.062	36.405	26.927	23.88	26.79	25.9	34.37	34.67	16.38	50.28	37.06	52.72	11.62	23.57	26.32	17.6	26.8	9.16	9
Os01g0167750	18.4	22.4	16.2	25.45	31.64	26.82	49.3	29.7	20	43.11	37.28	34.81	25.036	27.07	28.511	35.82	14.43	26.94	27.87	17.74	19.83	17.75	24.23	23.11	35.92	12.26	21.93	14.8	23	17.5	7
Os01g0631200	303	274	409	122.3	121.1	135.8	50.1	68.3	103	292.3	337.7	461.9	131.71	121.35	250.26	513.5	370	270.4	162.6	138.7	161.2	126.4	124	198.6	248.2	477.1	418.8	170	215	194	13
Os01g0239000	2242	2317	2465	1085	1064	931.7	1210	1373	1673	3278	3579	3143	3728.2	2628.6	3353.2	4120	1921	2266	935.5	981.2	834.6	1152	1211	1093	2828	2746	2270	1882	2332	2487	3
Os01g0278600	250	212	264	175.7	203.4	173.9	172	157	145	194	201.7	200.4	202.46	220.29	233.63	417.9	347.3	322.2	226.7	191.1	238	165.6	161.8	127.1	203.9	260.2	199.6	266	311	292	13
Os01g0797600	791	771	645	181.4	95.82	96.89	248	149	319	612.2	653.5	596.5	371.19	331.38	289.86	1349	1114	619.5	167.2	162.9	257.8	555.3	223.8	919.4	648.6	611	734.6	410	399	604	13
Os01g0609700	0	0	0	0	1.808	0	0	0	1.38	0.862	0	0.941	1.0885	0.9335	0	0	0	1.036	0	1.613	1.724	1.479	0.713	2.167	1.056	2.829	0	0.93	0.96	0.83	9
Os01g0879500	73.4	60.5	52.8	89.49	91.3	71.8	82.2	87.9	96.1	65.53	41.66	50.8	81.639	65.342	86.324	95.53	53.59	70.45	70.6	97.56	100	99.08	74.11	83.06	58.1	75.43	46.05	62.9	65	38.3	12
Os01g0144340	228	229	258	252.9	268.5	249.1	198	204	216	260.4	290.5	233.3	280.84	287.5	279.56	477.6	225.7	223.8	243.4	278.2	256.1	161.9	181.7	174.8	263	278.1	239	241	265	253	13
Os01g0703600	3.06	0	4.31	0.821	2.712	0.865	3.91	5.69	2.07	11.21	7.675	3.763	1.0885	1.8669	5.5438	0	2.061	1.036	5.574	6.45	2.587	1.479	4.276	0.722	2.113	0	0	0.93	0.96	0.83	7
Os01g0513700	364	348	307	426.9	460.1	436.8	617	628	657	389.7	362.9	313.3	402.75	355.65	346.09	441.8	370	278.7	522.1	449.1	556.1	384.5	434	516.4	311.6	288.5	331.1	291	322	313	12
Os01g0268000	0	1.86	2.16	0	0	0	0.78	0	0.69	1.724	1.096	0	2.177	0.9335	3.9598	23.88	2.061	1.036	0.929	0	0	0	1.425	0.722	1.056	4.714	0	3.7	2.87	8.33	6
Os01g0537250	230	352	233	559.1	686.1	420.4	95.5	196	171	160.4	244.5	96.9	235.12	164.29	235.21	346.3	241.1	250.7	268.5	341.9	347.5	287.6	367.7	205.1	350.7	231	175.4	282	140	225	2
Os01g0133600	3.06	8.38	1.08	1.642	0	1.73	0.78	0	0.69	2.587	4.386	2.822	11.974	2.8004	10.296	11.94	1.031	8.288	0	0	1.724	0.739	0	1.444	4.225	0.943	4.386	3.7	2.87	3.33	13
Os01g0209150	0	0	0	0	0	0	1.57	0	0	0	0	0	0	0	0	0	0	0	0	0	3.449	0	0.713	0	0	0	2.193	0	0	0.83	11
Os01g0215000	43.8	14.9	49.6	57.47	32.54	86.5	57.2	19.6	23.5	23.28	9.868	29.17	18.505	36.405	26.135	71.65	26.79	29.01	101.3	29.83	50.87	100.6	59.15	227.5	11.62	20.74	26.32	40.7	20.1	19.2	9
Os01g0882800	7.14	1.86	7.54	13.14	24.41	17.3	86.1	53.1	56.7	5.173	0	5.645	4.3541	3.7338	2.3759	0	5.153	0	17.65	10.48	31.04	43.62	44.9	65	1.056	3.771	17.54	3.7	5.74	3.33	18
Os01g0110800	0	0	2.16	16.42	9.04	9.516	13.3	13.3	9.67	0	0	4.704	3.2656	1.8669	3.9598	0	0	1.036	12.08	6.45	8.622	6.654	10.69	17.33	1.056	0	0	3.7	1.91	5	12
Os01g0970600	1327	1254	1482	1133	1324	1212	895	1043	904	1136	1285	1250	1608.8	1527.1	1551.5	2484	1461	1487	1445	1315	1547	783.7	719	806	1313	1219	1276	1438	1450	1439	13
Os01g0503400	359	332	312	423.6	386.9	337.4	882	923	1090	412.1	427.6	530.6	220.97	199.76	197.99	226.9	214.4	258	264.8	203.2	208.7	1276	1280	905	371.8	441.2	583.3	204	156	195	18
Os01g0885300	29.6	21.4	16.2	41.05	27.12	65.74	37.6	46.2	44.9	25	36.18	17.88	10.885	17.736	15.047	59.7	29.89	16.58	19.51	27.41	34.49	69.5	105.5	146.6	30.63	40.54	81.14	45.3	47.8	19.2	9
Os01g0225100	2099	2187	2139	1335	1317	1425	1438	1435	1540	2179	2445	2310	2774.6	2572.6	2648.3	3988	2679	2477	1410	1542	1493	1490	1437	1390	2687	2655	2274	2648	2454	2889	13
Os01g0819300	24.5	14.9	43.1	23.81	38.87	19.03	13.3	9.49	11.7	21.55	7.675	6.586	14.151	15.869	15.047	11.94	34.01	12.43	13.01	19.35	12.93	19.96	27.79	13.72	13.73	10.37	19.74	33.3	25.8	33.3	8
Os01g0819800	7.14	4.66	17.2	32.84	41.58	42.39	40.7	24	41.5	11.21	15.35	4.704	19.593	14.935	11.088	0	11.34	7.252	40.87	48.38	37.94	29.58	29.93	31.78	11.62	10.37	13.16	13.9	12.4	15	12
Os01g0328400	2142	1737	1744	2172	2316	2292	2601	2032	2454	2239	1934	3699	2238	2317.8	1614.8	2782	1553	1671	2624	2305	2582	1754	1807	2013	1768	2350	2632	1356	1569	1231	15
Os01g0115100	446	539	633	270.9	275.7	256.1	324	378	248	811.3	657.8	581.4	537.73	617.01	651	716.5	533.8	554.3	275.9	391	287.1	369.7	424	294	600	610	712.7	809	638	670	3
Os01g0274300	0	0	1.08	0	1.808	1.73	3.13	1.27	0	0	0	0	0	0	0	0	0	0	2.787	0.806	0	1.479	0.713	0	0	0	0	0	0	0.83	12
Os01g0260500	21.4	34.5	25.9	64.04	43.39	41.52	10.2	25.3	14.5	13.79	15.35	8.467	22.859	17.736	20.591	23.88	22.67	23.83	12.08	24.19	23.28	62.11	47.75	45.5	14.79	10.37	4.386	31.5	17.2	32.5	2
Os01g0117900	249	334	295	218.4	192.5	250.9	229	175	189	233.7	326.7	241.8	382.07	299.64	354.8	358.2	207.1	244.5	229.5	226.6	165.5	217.4	163.9	214.5	189.1	178.2	186.4	262	314	292	4
Os01g0243000	150	114	88.4	28.73	27.12	38.06	39.1	62	44.2	51.73	41.66	27.28	35.921	34.538	43.558	83.59	85.53	67.34	29.73	35.48	38.8	45.1	52.02	46.22	40.14	30.17	26.32	52.7	33.5	33.3	14
Os01g0236300	208	251	194	197	205.2	194.6	478	493	358	144	198.4	176.9	203.55	209.09	283.52	537.3	253.5	206.2	207.2	164.5	188	553.8	563.7	504.8	237.7	254.6	230.3	336	292	335	17
Os01g0742500	1375	1332	1307	564.8	666.2	656.6	673	624	690	1383	1399	1568	1448.8	1508.5	1503.9	2627	1551	1377	659.6	559.6	691.5	469.5	526.6	541.7	1499	1821	1548	1324	1456	1343	13
Os01g0778700	741	720	633	610	565.9	558	706	704	603	734.6	707.2	684.9	685.77	715.02	776.92	883.6	716.2	708.6	575	633.7	653.6	683.2	629.3	644.2	799.7	732.6	703.9	700	825	799	13
Os01g0214800	530	547	486	247.1	295.6	237.9	597	487	498	421.6	418.8	411.1	448.47	465.79	476.76	489.6	494.7	417.5	327.9	290.3	283.7	365.3	496	424.7	360.2	368.7	515.3	571	651	570	5
Os01g0850050	19.4	15.8	9.7	31.2	40.68	20.76	15.7	11.4	13.8	12.93	12.06	15.05	27.213	23.336	17.423	0	18.55	3.108	19.51	15.32	18.11	2.958	2.851	5.056	11.62	13.2	2.193	11.1	4.78	13.3	2
Os01g0375500	6.12	1.86	3.23	10.67	10.85	5.19	46.2	32.9	58	2.587	0	1.882	2.177	1.8669	3.9598	11.94	0	2.072	18.58	16.93	4.311	31.79	28.51	41.89	1.056	0	2.193	0	0.96	5	18
Os01g0293000	70.3	51.2	49.6	68.96	59.66	51.04	11.7	39.8	40.8	54.32	42.76	39.51	18.505	42.005	45.934	59.7	27.82	71.49	26.01	22.58	15.52	33.27	47.03	18.06	39.09	67.88	105.3	40.7	54.5	78.3	11
Os01g0174900	318	292	303	394.9	430.3	426.5	478	467	515	401.8	364	372.6	400.58	329.51	376.18	513.5	262.8	276.6	470.1	409.6	381.1	422.9	383.4	433.3	316.9	319.6	309.2	276	311	294	12
Os01g0616300	2.04	2.79	2.16	4.105	4.52	2.595	7.05	10.8	4.84	0.862	1.096	1.882	1.0885	1.8669	3.9598	0	0	1.036	0	1.613	4.311	0	4.988	2.889	2.113	1.886	2.193	0	7.65	0	18
Os01g0773350	1.02	0	0	0	0	0.865	0	0	0	0	0	0	0	0	0	0	0	0	0	0	0	0	0	0	0	0	0	0	0.96	0	19
Os01g0798500	1045	1284	1356	1593	1699	1532	2135	2245	2218	1146	1379	1344	1420.5	1251.8	1254.5	1696	1055	1165	1636	1811	1866	1414	1400	1958	1075	1000	916.6	874	1106	1032	12
Os01g0901200	213	210	184	794.7	922	761.2	401	381	261	213	189.7	141.1	207.91	318.31	179.78	358.2	218.5	280.8	576	670	632	334.9	312.8	221.7	142.6	184.8	160.1	172	163	204	10
Os01g0558100	74.4	51.2	62.5	205.2	172.7	171.3	20.4	75.9	45.6	39.66	48.24	30.11	55.515	42.939	53.854	119.4	42.25	50.77	252.7	250.8	161.2	42.15	76.96	42.61	41.2	24.51	59.21	56.4	75.5	30.8	10
Os01g0688300	2.04	0	0	0	0.904	0.865	0	1.27	0	0	0	4.704	0	0	0	0	0	2.072	0	0.806	2.587	0	1.425	0.722	0	0	0	0	0	0	20
Os01g0801600	102	112	80.8	35.3	33.45	55.36	47	63.3	51.8	68.97	70.17	88.44	76.196	89.611	83.948	155.2	89.66	72.52	61.31	41.93	53.46	80.59	95.49	75.84	126.8	124.5	203.9	124	85.1	93.3	11
Os01g0102500	485	328	418	136.3	131.1	128	133	161	142	371.6	382.6	516.5	241.65	227.76	254.22	585.1	417.4	322.2	143.1	200	167.3	147.1	158.2	203	399.3	396	355.3	236	303	267	13
Os01g0832000	872	917	1010	1275	1357	1205	1412	1340	1289	1137	1196	1092	1089.6	996.93	1029.6	1314	754.3	826.8	1333	1276	1378	921.3	924.3	1059	918	833.5	833.3	689	794	845	12
Os01g0861700	2.04	0	3.23	13.14	14.46	11.25	16.4	24.7	11.7	4.311	5.482	1.882	1.0885	3.7338	2.3759	0	0	1.036	12.08	8.869	18.97	17.75	17.1	20.22	0	1.886	0	0	1.91	1.67	12
Os01g0136400	1252	1137	1120	1190	990.8	1275	957	971	916	912.2	869.4	1060	863.2	893.31	970.16	1481	1158	1190	1730	1524	1974	1253	1081	1248	1147	991.9	1134	1034	887	930	16
Os01g0756501	167	229	183	203.6	229.6	221.5	145	214	236	156.9	159	145.8	234.03	173.62	242.34	286.6	196.8	205.1	132.8	163.7	133.6	277.3	281.5	260.7	234.5	175.4	107.5	215	212	242	17
Os01g0758300	0	0	0	0	0	0	0	0	0	1.724	0	0	0	0	0	0	0	0	0	0	0	0	0	0	0	0	0	0	0	0	7
Os01g0609200	1.02	0	0	4.926	2.712	0.865	0	0	0	0	1.096	0	0	0	0	0	0	0	0.929	4.031	0.862	2.218	4.988	2.889	0	0	0	0	0	0	17
Os01g0730800	1236	995	1176	1205	1209	1227	1397	1639	1493	1230	1403	1686	1911.4	1727.8	1646.5	3678	1669	1472	1788	1820	1814	944.9	954.2	1257	1718	1613	1469	1431	1434	1416	6

Os01g0264500	541	453	585	403.1	393.2	450.7	900	789	744	490.6	446.2	614.4	618.28	679.55	591.6	1027	783.2	591.6	621.5	649.1	683.7	640.3	617.1	651.5	552.5	579.8	539.5	627	623	572	6
Os01g0189700	418	504	564	624.8	698.8	752.6	578	681	632	350	479.1	303.9	520.31	454.59	483.1	1003	381.3	447.6	531.4	580.5	497.5	678	586.5	497.6	278.9	207.4	199.6	358	391	433	6
Os01g0831300	0	0	0	1.642	0	0	0	1.9	0	0	0	0	0	0	0	0	0	0	0	0.806	0	0.739	0	0	0	0	0	0	0	0	12
Os01g0788500	160	188	212	279.1	258.5	197.2	313	357	261	175.9	144.7	108.2	144.77	145.62	176.61	250.8	165.9	119.1	175.6	226.6	155.2	334.2	352	295.4	161.6	111.3	133.8	179	166	160	18
Os01g0780600	0	2.79	0	1.642	0.904	2.595	0	0.63	0	0	0	1.882	0	0	0	0	0	0	0	0.806	0	0	0	3.611	0	0	0	0	0	0	2
Os01g0752300	7.14	9.31	12.9	10.67	8.136	6.055	3.91	12.7	5.53	4.311	3.289	8.467	7.6196	5.6007	11.879	11.94	5.153	7.252	1.858	13.71	12.07	5.176	4.988	5.778	12.68	4.714	6.579	12	0.96	10.8	4
Os01g0935800	0	0	0	0	0	0	0	0	0	1.724	0	0	1.0885	0	0.792	0	0	1.036	0	0	0	0	0	0.722	0	0.943	0	0	3.83	0.83	1
Os01g0867700	236	170	205	240.5	199.8	279.4	312	264	264	263	241.2	213.6	202.46	238.03	215.41	238.8	236	204.1	259.2	235.4	282.8	231.4	236.6	270.1	202.8	203.7	210.5	168	161	150	15
Os01g0769900	685	1013	1029	4249	5108	3296	1971	3032	1960	749.2	901.2	541	1310.6	837.31	838.69	1672	386.4	705.5	1986	2156	1708	1432	1539	1400	670.8	370.5	291.7	531	652	721	10
Os01g0291500	2.04	16.8	18.3	2.463	0.904	2.595	0.78	0.63	2.76	22.42	6.578	1.882	4.3541	10.268	3.9598	0	4.122	7.252	0	2.419	0	0.739	0.713	0	7.395	2.829	6.579	11.1	5.74	0.83	7
Os01g0233500	7.14	7.45	9.7	6.568	4.52	12.98	6.26	6.96	3.46	3.449	10.96	8.467	8.7082	3.7338	7.9197	11.94	2.061	8.288	6.503	8.869	7.76	10.35	8.552	16.61	4.225	11.31	6.579	1.85	6.69	5.83	9
Os01g0954000	492	407	552	50.08	41.58	51.9	42.3	38	81.5	368.2	264.2	367.9	351.59	373.38	410.24	740.3	500.8	438.2	97.54	83.85	77.6	47.32	69.13	66.45	283.1	402.6	517.5	455	577	467	13
Os01g0371200	58.1	22.4	39.9	8.21	5.424	6.92	7.05	5.69	7.6	47.42	42.76	45.16	69.665	74.676	59.397	95.53	37.1	50.77	14.86	12.09	16.38	5.915	10.69	7.222	16.9	41.49	96.49	52.7	46.9	55.8	13
Os01g0214600	23.4	13	8.62	4.926	0	8.65	3.13	1.27	0	12.93	6.578	21.64	9.7967	9.3345	2.3759	23.88	17.52	10.36	13.01	3.225	12.93	3.697	8.552	6.5	11.62	24.51	39.47	16.7	24.9	17.5	11
Os01g0939300	2.04	0	0	5.747	1.808	6.92	0	1.27	4.84	1.724	1.096	2.822	2.177	0	0.792	0	1.031	5.18	1.858	2.419	1.724	2.958	4.988	1.444	0	0	0	1.85	2.87	0.83	2
Os01g0852800	0	1.86	0	0.821	0	0.865	0	0	0	1.724	1.096	0	2.177	0.9335	0.792	0	0	2.072	3.716	2.419	1.724	0	0	0	0	0	2.193	0	0	0	16
Os01g0318600	97.9	87.6	93.7	85.38	82.26	76.99	32.1	55	33.9	53.46	62.49	53.63	72.931	98.946	90.284	191.1	132.9	125.4	37.16	63.7	37.94	84.29	94.07	56.34	97.19	73.54	39.47	173	105	140	13
Os01g0763750	689	628	796	550.1	553.2	512.1	1060	1030	876	716.5	570.1	505.2	500.72	506.86	574.18	1385	501.9	553.2	467.3	554.7	551.8	1393	1317	1129	642.3	587.4	574.5	575	534	575	17
Os01g0687500	281	216	260	310.3	364.3	319.2	428	491	426	188.8	221.5	261.5	251.45	255.77	228.09	453.8	230.8	296.3	523.9	503.1	486.3	343.8	291.5	369.1	217.6	210.3	199.6	216	215	199	12
Os01g0855200	1148	847	1092	2649	2662	2042	178	236	376	1514	1885	873.1	1963.7	1284.4	1399.4	2926	1209	1002	3252	3480	2833	206.3	240.2	121.3	1406	1380	1151	1940	2509	1880	16
Os01g0679600	229	237	254	261.9	327.2	289.8	418	393	439	260.4	303.7	249.3	259.07	286.57	258.97	573.2	202	226.9	290.8	262.8	211.2	289.1	312.8	313.5	202.8	179.1	173.2	172	198	174	6
Os01g0266100	0	2.79	0	3.284	4.52	3.46	5.48	1.9	0.69	0.862	1.096	0	0	0	0	0	0	0	1.858	0.806	1.724	1.479	2.138	7.945	0	0.943	0	0.93	0	0.83	12
Os01g0849100	0	0	0	0	0	0	0	0	0	0	0	1.882	0	0	0	0	0	0	0	0	0	0	0	0	0	0	0	0	0	0	20
Os01g0152600	72.4	96.9	100	53.36	70.51	50.17	19.6	46.2	37.3	68.97	57.01	58.33	65.311	55.074	58.605	214.9	79.35	93.24	43.66	54.02	56.04	37.71	43.47	25.28	61.27	48.08	39.47	79.6	61.2	65.8	13
Os01g0210600	2522	2213	2179	1215	1031	1240	1257	1281	1821	2639	2185	4001	1630.6	2091.9	1942.7	3021	2039	1927	1680	1242	1189	1365	1846	1734	2431	2473	2660	1972	1412	1777	20
Os01g0283000	63.2	45.6	50.6	93.59	77.74	66.61	68.1	59.5	73.2	31.9	50.43	36.69	39.187	47.606	49.102	119.4	23.7	45.59	53.88	66.92	43.97	72.46	63.42	55.61	41.2	32.06	26.32	50.9	39.2	55	6
Os01g0842600	160	147	169	128.9	153.7	132.4	75.9	96.8	62.2	109.5	96.48	87.5	69.665	83.077	79.197	203	132.9	182.3	97.54	127.4	156.9	102	136.8	84.5	95.07	156.5	149.1	113	107	103	14
Os01g0248300	0	0	0	0	0	0.865	1.57	0	0	0	0	0	0	0	0	0	0	0	0	0	0	0.739	0	0	0	0.943	0	0	0	0	5
Os01g0502800	1368	1304	1469	789	902.2	866.8	776	945	879	1021	1119	1095	1244.2	1235	1325.8	2627	1614	1608	893.7	969.2	995.9	1039	885.8	893.4	1163	1255	1292	1410	1470	1453	13
Os01g0727700	2.04	6.52	6.47	9.852	8.136	18.17	16.4	20.2	8.98	4.311	1.096	11.29	4.3541	1.8669	2.3759	0	7.214	5.18	13.01	14.51	33.63	13.31	15.68	10.83	3.169	3.771	6.579	4.63	1.91	10.8	12
Os01g0369200	58.1	54	52.8	33.66	58.76	29.41	23.5	15.2	15.2	25.87	51.53	47.98	33.744	43.872	56.23	23.88	29.89	35.23	28.8	17.74	12.93	14.79	22.8	13.72	39.09	27.34	26.32	21.3	22	25	19
Os01g0217500	52	66.1	59.3	13.14	38.87	19.9	90.8	83.5	93.3	144.8	96.48	64.92	20.682	42.005	67.317	0	28.85	40.41	24.15	33.86	13.8	204.8	242.3	224.6	75	67.88	74.56	47.2	29.6	42.5	9
Os01g0115700	79.5	108	100	23.81	32.54	32.01	61.8	67	35.9	172.4	143.6	237.1	102.32	139.08	156.81	83.59	104.1	79.77	58.52	41.12	54.32	84.29	106.2	96.06	157.4	165	182	150	66	103	20
Os01g0871300	256	338	276	256.1	232.3	206.7	125	175	156	288.8	220.4	247.4	264.51	280.97	261.35	465.7	277.2	284.9	259.2	212.9	219	159	238	182	190.1	241.4	252.2	285	258	269	13
Os01g0654900	0	0.93	0	0	0	0	0	0	0.69	0	0	0	0	0	0	0	0	0	0	0	0	0	0	0.722	0	0	0	0	0	0	14
Os01g0847900	245	190	237	101	87.69	110.7	72	61.4	69.1	224.2	195.2	271	218.79	213.76	257.39	310.5	202	176.1	120.8	99.17	104.3	83.55	76.96	83.06	251.4	280	293.9	211	173	202	3
Os01g0234001	45.9	41	48.5	10.67	16.27	15.57	22.7	28.5	24.9	18.97	37.28	46.1	25.036	60.674	41.182	191.1	81.41	58.02	25.08	29.83	29.32	16.27	14.25	18.06	48.59	48.08	48.24	48.1	73.6	65.8	13
Os01g0699100	121	94.1	115	5.747	1.808	11.25	0.78	5.69	7.6	46.56	40.57	79.03	47.895	46.673	68.901	59.7	145.3	99.46	2.787	4.031	3.449	17.01	27.79	19.5	96.13	93.34	111.8	70.3	56.4	83.3	3
Os01g0916800	1309	1469	1491	1337	1291	1290	985	1157	1019	1224	1332	1030	1694.8	1297.5	1564.1	2412	1412	1287	1127	1172	1043	1120	1288	1228	1442	1397	1195	1517	1427	1575	13
Os01g0147600	10.2	15.8	9.7	21.35	18.08	33.74	27.4	15.2	24.2	17.24	18.64	12.23	8.7082	10.268	8.7116	47.76	7.214	11.4	27.87	20.16	14.66	10.35	32.07	14.44	12.68	18.86	28.51	18.5	26.8	16.7	6
Os01g0881950	0	0.93	0	0	0	0	0	0	0	0	0	0.941	0	0	0	0	0	0	0	1.613	0	0.739	0	0	0	0	0	0	0	0.83	16
Os01g0260700	1107	1057	977	1399	1419	1286	1278	1359	1198	1302	1128	1494	1119	1178.9	1129.3	1946	990.3	1085	1604	1338	1534	1062	1045	1089	1098	1134	1241	989	970	912	6
Os01g0584300	78.5	70.8	75.4	215.9	218.8	216.3	92.4	63.9	80.2	106	82.23	118.5	96.878	85.878	89.492	71.65	69.05	74.59	246.2	245.9	278.5	72.46	63.42	89.56	76.06	69.77	63.59	76.8	88	109	10
Os01g0655300	1049	1011	1061	1008	972.7	975.8	771	813	768	1017	1094	984.1	1186.5	981.99	1104.8	2173	1125	1015	837.9	1009	870	961.2	947.8	884	1063	1030	940.8	1177	1165	1264	13
Os01g0807900	643	792	704	1148	1219	1113	1185	1273	1200	546.6	667.7	445.9	523.58	460.19	582.89	1099	421.5	531.5	723.7	849	652.7	1183	1211	1172	586.3	462.9	548.2	530	537	552	12
Os01g0350100	0	0.93	3.23	0	0	1.73	0	0	0.69	1.724	0	1.882	0	0	0	0	0	1.036	0	0	0	0	2.851	0.722	3.169	0	0	0.93	0.96	0	7
Os01g0886900	3720	3371	3720	711.8	528.8	688.6	99.4	120	135	3189	3601	2819	2942.3	3018.8	3234.4	5875	4027	3729	732.9	875.6	926.9	300.9	246.6	238.3	3339	3839	2925	3516	3488	3694	13
Os01g0178700	34.7	40.1	35.6	37.76	38.87	44.12	50.9	48.1	33.2	50.01	27.41	36.69	39.187	31.737	39.598	11.94	37.1	42.48	38.09	47.57	44.84	56.93	91.93	61.39	36.97	36.77	41.67	37	39.2	30.8	17
Os01g0822800	191	149	163	1436	1313	1268	32.1	8.86	21.4	158.6	130.5	84.67	173.07	172.69	126.71	656.8	254.5	204.1	1264	1380	1581	29.58	20.67	25.28	175.4	133.9	103.1	232	244	235	10
Os01g0794100	88.7	44.7	100	22.99	14.46	17.3	1.57	6.96	4.15	23.28	5.482	13.17	21.77	21.469	27.719	95.53	10.3	69.41	64.1	64.5	68.12	5.176	11.4	21.67	23.24	9.428	19.74	23.1	33.5	25	14
Os01g0694000	23.4	31.7	17.2	57.47	54.24	82.18	24.3	38	31.1	31.04	39.47	37.63	19.593	26.137	23.759	71.65	14.43	19.68	52.95	63.7	68.12	31.05	17.1	10.83	22.18	22.63	39.47	13.9	33.5	28.3	10
Os01g0152675	0	0	0	0.821	0	0	0	0	0	0	0	0.941	0	0.9335	1.5839	11.94	0	0	0	0	0	0	0.713	0	0	0	0	0	1.91	0	6
Os01g0136100	7.14	7.45	8.62	8.21	21.7	2.595	1.57	0.63	0	5.173	3.289	1.882	14.151	14.935	3.1679	0	0	0	0.929	0.806	2.587	0	0	0.722	0	0.943	4.386	2.78	0.96	0	4
Os01g0172100	896	741	690	348.9	317.3	378	723	431	479	913.1	992.2	1476	472.42	673.95	518.74	752.3	983.1	671.4	634.5	341.9	521.6	533.1	374.8	637	798.6	1091	1669	635	618	615	20
Os01g0157500	0	0	0	0	0	0	0	0	0	0	0	0	0	0	0	0	0	1.036	0	0	0	0	0	0	0	0	0	0	0	0	1
Os01g0561600	0	0	0	0	4.52	0.865	4.7	4.43	0.69	0.862	0	0	2.177	0.9335	1.5839	0	1.031	2.072	0	3.225	0	14.05	7.839	10.11	0	0	0	1.85	0	0.83	9
Os01g0685100	95.8	83.8	98.1	84.56	96.72	96.02	174	176	109	127.6	118.4	102.5	128.45	126.95	114.04	167.2	92.75	125.4	124.5	113.7	135.4	150.1	158.9	194.3	96.13	82.03	96.49	132	130	136	18
Os01g0243600	3.06	11.2	1.08	0	0	0	2.35	1.9	0	0	0	0	0	0	0	0	0	0	0.929	0	0.862	1.479	1.425	2.167	0	0	0	0	0	1.67	14
Os01g0737401	53	42.8	48.5	51.72	65.99	74.39	64.2	51.2	53.2	53.46	30.7	48.92	75.108	63.475	68.109	131.4	60.8	53.87	124.5	133.8	161.2	38.45	43.47	40.45	50.71	59.4	59.21	44.4	55.5	59.2	16
Os01g0265400	424	365	454	283.2	310.1	260.4	410	417	516	325	343.2	284.1	299.34	224.96	312.03	465.7	434.9	430	239.7	301.5	293.2	469.5	349.9	426.8	282	363.9	291.7	280	367	329	9
Os01g0602466	24.5	41	36.6	93.59	111.2	52.77	653	642	602	100.9	62.49	59.27	45.718	21.469	39.598	59.7	26.79	26.94	87.32	74.18	56.04	329.8	468.9	351	52.82	50.91	67.98	9.25	30.6	22.5	18
Os01g0296000	6.12	10.2	20.5	87.84	77.74	66.61	89.2	79.1	98.1	11.21	28.51	13.17	9.7967	8.4011	12.671	0	5.153	16.58	69.67	58.86	59.49	64.33	84.8	65.72	5.282	9.428	15.35	14.8	9.56	13.3	12
Os01g0372400	5.1	6.52	1.08	1.642	0.904	6.92	8.61	15.8	4.84	6.897	2.193	3.763	1.0885	2.8004	7.9197	0	6.183	0	1.858	1.613	0.862	8.133	11.4	6.5	2.113	3.771	10.96	3.7	0.96	4.17	5
Os01g0805700	724	688	649	513.1	442	513.8	456	447	506	569	549.3	557	597.6	695.42	624.86	1015	708	761.5	461.7	428.9	423.3	542	644.2	525.8	660.2	654.3	739	834	590	694	13
Os01g0881500	1822	2091	2037	2155	1950	2263	1628	1891	1858	1750	1709	1675	2007.2	1780.1	2167.6	2830	1730	1907	1823	1821	1999	2142	2374	2154	1942	1807	1588	1770	1578	1825	6
Os01g0849500	83.6	56.8	72.2	32.02	26.22	34.6	25.1	34.8	42.8	26.73	52.63	79.03	38.098	106.41	48.31	23.88	108.2	95.32	57.6	39.51	46.56	25.88	27.79	20.22	67.61	96.17	98.68	52.7	49.7	62.5	3
Os01g0837800	2774	3130	3281	889.1	804.5	811.4	1618	1614	2270	3074	3403	2524	3761.9	2982.4	4027.1	6532	2679	2748	865.8	964.3	905.3	2168	2084	1886	3499	2889	2732	3225	3415	3647	13
Os01g0739500	1416	1664	1195	2176	1998	3116	444	687	529	554.4	483.5	730.1	2222.8	1461.8	1927.6	1421	2035	1595	1711	1534	2615	1276	949.2	1392	709.9	793.9	638.1	847	410	835	10
Os01g0953600	683	652	744	549.2	500.8	582.2	564	593	582	844.1	753.2	669.9	610.66	701.02	654.16	1600	642	599.9	587.1	620	604.4	485	601.5	590.8	796.5	644.9	631.6	685	641	697	13
Os01g0238200	99.9	74.5	83	126.4	126.6	127.2	255	165	191	123.3	166.7	157.1	104.5	112.01	124.34	59.7	102	107.7	127.3	176.6	181.9	130.9	79.1	154.6	133.1	136.7	109.6	116	103	104	15
Os01g0227400	229	241	266	139.6	126.6	161.8	139	112	92.6	444.9	380.4	423.4	370.1	350.04	322.33	955.3	312.3	269.4	246.2	196.7	226.8	85.77	104	127.8	385.6	420.5	521.9	318	321	271	13
Os01g0538000	1784	1621	1638	1598	1520	1657	1397	1389	1515	1917	1693	2102	1867.9	1977.1	1745.5	4155	1746	1771	1874	1687	1918	1531	1520	1434	1871	1912	2020	1630	1690	1555	6
Os01g0706000	446	414	428	367.8	451.1	413.5	424	394	471	408.7	466	430	473.51	441.52	439.54	585.1	372	388.5	369.7	376.5	383.7	447.3	388.4	398.7	440.5	413	462.7	391	412	429	6
Os01g0197700	71.4	14	34.5	18.06	9.944	19.9	0	6.33	2.07	40.52	19.74	47.98	130.62	118.55	76.029	59.7	25.76	16.58	13.93	0.806	0.862	4.436	21.38	3.611	23.24	32.06	2.193	20.4	17.2	48.3	4
Os01g0589700	76.5	72.7	69	104.3	79.55	88.23	125	104	113	96.56	76.75	93.14	69.665	70.009	74.445	155.2	76.26	68.38	61.31	66.12	51.73	90.94	104	91	66.55	78.26	83.33	95.3	75.5	51.7	6
Os01g0583525	2.04	0	0	0.821	0	0	1.57	0	0	1.724	0	0	0	0.9335	0	0	0	1.036	0.929	0	0	0	0.713	0	2.113	0	0	0	0	0	19
Os01g0622500	730	562	625	302.1	303.7	320.9	195	175	179	375.9	337.7	509	476.77	511.53	471.22	1254	715.2	779.1	281.5	389.4	357.8	308.3	268.7	234.7	554.6	571.4	530.7	648	643	620	13
Os01g0614500	572	595	529	578	565	564.9	289	283	262	422.5	424.3	443.1	549.7	452.72	502.9	1314	531.8	582.3	438.5	578.1	554.4	367.5	353.5	262.2	515.5	528	504.4	589	544	649	13
Os01g0328300	236	198	263	333.3	370.6	326.1	213	246	200	263.8	274.1	240.8	284.1	256.7	293.82	382.1	201	219.6	262	397.5	334.5	214.4	242.3	190.7	221.8	240.4	149.1	236	228	212	10
Os01g0229400	196	156	179	128.9	135.6	155.7	106	84.8	115	215.5	232.4	214.5	185.05	195.09	155.23	370.2	142.2	133.6	143.1	112.1	145.7	36.97	32.78	59.95	120.4	154.6	188.6	77.7	90.9	90.8	13
Os01g0221500	959	960	1093	1952	1951	2228	2201	2593	2250	1083	1067	1105	1184.3	1319.9	1373.3	2734	1261	1155	2532	2434	2706	1551	1770	1892	1339	1383	1241	1184	1223	1309	12
Os01g0164500	180	225	173	218.4	238.6	215.4	150	186	178	143.1	179.8	162.8	181.78	167.09	155.23	429.9	159.7	178.2	184.9	192.7	173.3	182.6	199.5	161.8	157.4	206.5	138.2	179	188	184	6
Os01g0606133	0	1.86	0	0	0	0	0	0	0	0	0	0	0	0	0	0	0	1.036	0	0	2.587	0	0	0	0	0	0	0	0	0	16
Os01g0731100	0	4.66	5.39	2.463	0.904	6.055	3.91	3.16	17.3	6.035	6.578	5.645	6.5311	8.4011	3.9598	23.88	1.031	1.036	4.645	4.838	6.036	182.6	23.52	173.3	9.507	6.6	6.579	12	10.5	11.7	9
Os01g0892600	2409	2334	2433	137.1	111.2	128	222	265	176	2912	3047	3161	2272.8	2259	2479.6	3451	2523	2340	207.2	120.1	196.6	125	124	208	2769	3082	2645	2374	2582	2281	3
Os01g0327400	119	175	148	114.1	98.53	88.23	300	306	413	219.9	155.7	192.9	81.639	111.08	166.31	95.53	102	87.03	157	121.7	87.08	412.6	677	540.2	131	106.5	155.7	115	125	98.3	17
Os01g0373700	13.3	2.79	4.31	6.568	4.52	12.11	0	3.16	0	0	6.578	6.586	0	4.6673	1.5839	0	6.183	0	9.29	0.806	18.97	0	5.701	1.444	10.56	3.771	4.386	0.93	2.87	0.83	16
Os01g0866600	1866	1567	1689	820.2	818.1	964.5	1547	1283	1131	2325	2154	3357	2106.3	2597.8	2183.5	2926	2690	1916	1665	1081	1536	805.2	782.5	1058	2552	2567	2899	2126	1857	2124	3
Os01g0853700	261	248	286	170.8	170.9	149.7	359	306	234	368.2	372.8	472.3	259.07	337.91	216.21	620.9	423.5	355.4	221.1	237	217.3	326.8	265.8	398.7	447.9	546.8	578.9	337	361	430	13
Os01g0231700	478	362	427	489.3	490.9	499.1	449	381	475	528.5	458.3	748.9	632.43	559.14	417.37	513.5	386.4	398.9	584.3	545	632.9	383	376.3	394.3	465.9	559.1	600.9	375	421	297	20
Os01g0141900	143	155	144	229.9	192.5	204.2	189	188	193	166.4	145.8	164.6	166.54	168.95	146.51	214.9	174.2	132.6	192.3	195.1	251.8	184.1	142.5	202.2	160.6	171.6	199.6	140	144	173	10
Os01g0956300	4.08	1.86	0	0	3.616	0	3.13	6.96	12.4	0.862	6.578	1.882	0	3.7338	4.7518	0	1.031	0	0.929	2.419	1.724	1.479	1.425	1.444	0	4.714	0	0.93	1.91	1.67	5
Os01g0186400	0	1.86	0	6.568	4.52	0	5.48	5.06	5.53	9.484	1.096	0.941	3.2656	1.8669	5.5438	0	0	1.036	9.29	0	2.587	1.479	2.138	1.444	1.056	0	0	0	0.96	1.67	7
Os01g0649000	372	410	381	454.8	467.4	447.2	279	271	229	351.8	385.9	380.5	438.67	415.39	398.36	967.2	408.1	492.1	485.8	500.7	485.4	337.9	333.5	292.5	382.4	413.9	414.5	403	361	397	6
Os01g0121200	20.4	21.4	30.2	70.6	58.76	44.12	76.7	107	42.2	31.04	33.99	29.17	9.7967	24.27	30.095	11.94	38.13	20.72	48.31	29.83	35.35	50.28	99.06	41.17	27.47	33	30.7	20.4	14.3	25	18
Os01g0703400	7119	6765	7130	1176	1068	1222	1477	2267	1796	7669	7426	7512	7757.9	7498.4	7671.8	11487	6781	6749	1693	1146	1244	1237	1092	1233	6544	6240	6533	5865	6163	6229	3
Os01g0283300	933	671	747	1068	752.1	1708	446	506	609	745.8	706.1	952.1	1235.5	918.52	906.8	2687	2882	1134	1329	1001	2233	937.5	1183	1179	1256	1162	1241	1488	889	1050	13
Os01g0581300	814	1191	867	1813	1925	1485	2883	2888	2511	1701	2001	2374	1078.7	1075.3	1100.8	1552	889.3	1150	1587	1528	1944	1610	1484	1754	1397	1437	1283	599	670	751	18
Os01g0952100	0	0	0	0	0	0	0	0	0	0	0	0	0	0	0	0	0	1.036	0	0	0	0	0	0	0	0	0	0	0	0	1
Os01g0728250	0	0	1.08	0.821	0.904	0.865	0.78	0.63	2.76	0.862	3.289	0	0	0	0	0	1.031	0	0	0.806	0	5.176	0.713	3.611	0	0	0	0	0	0	9
Os01g0622066	13.3	11.2	15.1	14.78	6.328	14.71	0	0	0	0	0	0.941	1.0885	1.8669	0	0	18.55	10.36	0.929	4.031	5.173	3.697	3.563	9.389	6.338	6.6	4.386	14.8	14.3	11.7	8
Os01g0535900	107	159	97	700.3	844.3	652.2	658	828	639	167.3	239	215.4	165.46	135.35	124.34	167.2	75.23	97.39	495.1	528.9	448.4	417.8	475.3	473.1	137.3	144.3	107.5	88.8	89.9	105	12
Os01g0315600	0	1.86	0	0.821	0	0.865	0	0	0.69	0.862	0	1.882	1.0885	0	0	0	0	6.216	0	0	0	0	0	0	0	0	0	0	0	0.83	14
Os01g0533800	17.3	30.7	25.9	17.24	26.22	22.49	3.13	3.16	1.38	8.622	9.868	1.882	15.239	14.935	15.839	23.88	10.31	21.76	0.929	6.45	6.036	22.92	18.53	15.89	9.507	4.714	8.772	15.7	17.2	27.5	8
Os01g0933500	7.14	3.73	7.54	14.78	0.904	4.325	6.26	5.69	14.5	4.311	2.193	3.763	4.3541	3.7338	5.5438	0	1.031	11.4	0.929	4.838	0	9.612	11.4	11.56	0	5.657	4.386	0.93	3.83	3.33	17
Os01g0815400	1481	1283	1678	450.7	370.6	359.9	326	182	265	1046	1095	1544	778.29	1005.3	839.48	1481	1352	1221	581.5	427.3	488.9	556	406.2	944.7	1076	1884	1610	1254	971	881	3
Os01g0809900	160	176	161	184.7	205.2	162.6	178	209	185	202.6	214.9	200.4	209	246.43	201.95	489.6	221.6	192.7	152.3	192.7	199.2	222.6	187.4	197.2	253.5	231	223.7	247	251	237	6
Os01g0160100	45.9	41	28	19.7	18.98	33.74	36.8	27.2	32.5	43.11	25.22	62.09	32.656	39.205	40.39	35.82	28.85	22.79	40.87	36.28	42.25	19.22	23.52	25.28	51.76	29.23	28.51	24.1	24.9	13.3	20
Os01g0128700	145	148	149	138.7	119.3	119.4	8.61	19.6	11.1	106.9	94.29	81.85	94.701	104.55	130.67	238.8	154.6	157.5	104	95.14	93.98	22.18	25.65	31.06	109.9	84.86	98.68	165	149	177	13
Os01g0170051	282	268	246	97.7	85.88	143.6	22.7	39.2	70.5	159.5	166.7	100.7	156.75	155.89	158.39	370.2	286.5	279.7	76.17	91.92	94.84	266.9	243.7	190	230.3	174.4	239	263	282	274	13
Os01g0253800	1.02	3.73	4.31	1.642	0.904	1.73	0	0	0	0.862	5.482	0	1.0885	0	0	0	1.031	0	7.432	3.225	0.862	1.479	0	0.722	0	0	0	0.93	0	0.83	16
Os01g0771200	677	508	623	337.4	321.8	338.2	386	424	363	796.7	714.8	838.3	715.16	661.82	726.23	1146	634.8	588.5	445	402.3	411.3	319.4	303.6	312	658.1	725	793.8	551	572	501	13
Os01g0721800	0	0	2.16	30.38	8.136	13.84	3.91	5.06	5.53	6.035	0	4.704	2.177	1.8669	3.9598	0	0	1.036	29.73	15.32	14.66	5.915	19.95	10.11	1.056	0	0	1.85	6.69	4.17	10
Os01g0843300	12.2	22.4	12.9	48.44	68.7	50.17	57.9	74	44.9	16.38	23.02	10.35	8.7082	16.802	20.591	23.88	15.46	21.76	23.22	29.03	13.8	57.67	79.1	41.17	17.96	14.14	8.772	29.6	17.2	20	12
Os01g0692400	0	1.86	0	12.31	8.136	2.595	9.39	10.8	15.2	1.724	3.289	8.467	10.885	3.7338	0.792	23.88	4.122	14.5	8.361	15.32	9.484	7.394	11.4	11.56	10.56	1.886	10.96	11.1	9.56	1.67	6
Os01g0810500	1.02	0.93	0	0	0	0	0	0	0	0	0	0.941	0	0	0	0	0	0	0	0	0	0.739	0	0	0	0	0	0.93	0	1.67	1
Os01g0227500	583	721	601	703.6	711.4	578.7	195	287	206	516.4	487.9	386.7	629.16	504.06	624.86	847.8	553.4	641.3	599.2	516	482.8	314.2	347.1	212.3	532.4	528.9	526.3	731	837	936	8

Os01g0158200	495	523	589	288.2	296.5	323.5	219	280	293	515.6	449.5	435.6	554.06	570.34	527.45	1230	703.9	469.3	322.3	363.6	312.1	239.6	330.7	255	532.4	462	502.2	563	551	595	13
Os01g0559300	159	170	147	171.6	197.1	177.3	205	164	193	206.9	195.2	245.6	161.1	172.69	194.82	274.6	204	124.3	253.6	211.2	239.7	159.7	161.1	184.9	214.4	217.8	228.1	151	180	157	6
Os01g0734501	2.04	5.59	2.16	5.747	5.424	5.19	11	35.4	34.6	1.724	3.289	1.882	2.177	8.4011	3.1679	0	4.122	5.18	3.716	5.644	13.8	17.75	19.95	18.78	6.338	3.771	2.193	13	5.74	5.83	18
Os01g0132700	132	98.7	100	112.5	94.92	112.5	147	147	129	111.2	174.3	167.5	121.91	127.88	131.47	179.1	144.3	133.6	125.4	146.7	126.7	118.3	124	107.6	139.4	176.3	140.3	129	142	116	20
Os01g0101700	1989	2332	2121	318.5	271.2	321.8	585	579	642	3660	4218	3152	3311.3	3297	3661.3	5457	2212	2164	516.5	463.6	454.4	399.3	466.1	531.6	3038	3205	2857	2508	2800	2829	3
Os01g0362800	18.4	20.5	21.6	17.24	19.89	23.36	21.9	17.1	20.7	21.55	21.93	21.64	27.213	21.469	16.631	95.53	23.7	26.94	18.58	24.99	23.28	29.58	29.93	22.39	31.69	43.37	24.12	30.5	29.6	30	6
Os01g0362400	38.7	31.7	49.6	57.47	79.55	45.85	141	124	120	45.7	65.78	75.27	55.515	51.34	40.39	59.7	73.17	35.23	95.68	108	90.53	88.73	81.95	119.2	40.14	41.49	43.86	28.7	20.1	27.5	18
Os01g0280000	475	579	513	482.7	484.5	383.2	615	610	647	507	529.6	585.2	513.78	512.46	517.95	823.9	508.1	480.7	432.9	419.3	432	530.9	523.1	582.9	559.9	509.1	548.2	527	472	521	6
Os01g0155400	1185	1162	1253	1377	1401	1344	770	884	811	1139	1114	1003	1531.5	1469.3	1465.1	1946	1168	1227	1245	1529	1308	851	783.2	817.6	1197	1194	1057	1485	1590	1521	13
Os01g0311800	0	0	0	33.66	0	42.39	2.35	0.63	6.91	4.311	0	3.763	3.2656	0	0	0	0	0	4.645	0	1.724	5.915	57.72	7.222	1.056	3.771	21.93	0.93	8.61	2.5	17
Os01g0847100	13.3	42.8	19.4	49.26	56.95	51.04	86.1	114	68.4	27.59	20.83	15.99	22.859	24.27	33.263	0	17.52	23.83	26.01	28.22	25	244	312.1	160.3	11.62	25.46	24.12	15.7	15.3	16.7	17
Os01g0217100	0	0	1.08	0	0.904	0	2.35	0.63	0	0	1.096	1.882	0	0	0	0	0	0	0	0.806	0	1.479	0	0.722	1.056	0	0	0	0.96	3.33	5
Os01g0928000	612	583	587	259.4	198	318.3	164	166	227	846.7	764.2	861.8	770.67	925.05	901.26	1218	956.3	836.1	553.7	460.4	595.8	258.8	322.8	240.5	1237	1230	1156	1220	922	1023	3
Os01g0253300	2562	2575	2377	2442	2400	2401	3369	3191	3321	2765	2525	2794	2693	2695.8	2712.5	4466	2517	2527	2220	2204	2326	3084	2788	2834	2594	2608	2895	2322	2119	2206	6
Os01g0166200	1.02	2.79	2.16	0.821	3.616	3.46	3.91	1.9	2.76	2.587	0	1.882	3.2656	3.7338	0	0	0	2.072	0.929	0	2.587	3.697	3.563	3.611	2.113	2.829	0	0.93	5.74	0.83	9
Os01g0629750	310	304	293	300.5	277.5	330.4	621	486	642	294	250	391.4	288.46	277.24	272.44	394.1	344.2	340.9	335.4	337.8	407.8	404.4	336.4	529.4	307.4	285.7	324.6	253	210	244	18
Os01g0848300	123	71.7	135	134.6	132.9	153.1	32.9	25.3	39.4	98.29	96.48	158.1	168.72	108.28	133.05	143.3	125.7	146.1	128.2	172.5	166.4	31.79	32.07	32.5	153.2	131.1	59.21	123	129	102	16
Os01g0752500	219	115	194	88.67	99.44	77.85	43.8	43.6	69.1	108.6	99.77	143	131.71	142.82	150.47	358.2	223.6	151.3	209	112.1	181.9	22.92	50.6	22.39	104.6	99.94	118.4	115	121	96.6	13
Os01g0317800	125	127	125	182.3	209.7	139.3	72	79.1	95.4	155.2	146.9	164.6	129.53	114.81	96.62	83.59	117.5	94.28	235	159.6	216.4	64.33	70.55	80.17	105.6	108.4	78.95	115	99.5	112	16
Os01g0947700	145	144	170	230.7	240.5	230.1	286	246	352	143.1	166.7	129.8	137.15	129.75	176.61	238.8	125.7	145	169.1	205.6	175	295.8	265.1	309.1	133.1	142.4	118.4	156	186	142	18
Os01g0504100	280	275	333	560.7	579.4	486.2	440	297	350	585.4	572.3	457.2	488.75	514.33	449.04	1122	306.1	369.9	823.1	700.7	748.4	203.3	277.9	281	541.9	414.9	326.7	483	540	540	6
Os01g0361700	1.02	0	0	0	0	0.865	0	0.63	0	0	0	0	0	0	0.792	0	0	0	0	0	0.862	0	0	0	0	1.886	0	0.93	0.96	0	1
Os01g0254000	1780	1530	1828	803.7	827.1	833.9	522	616	584	1767	1738	1465	1996.3	1869.7	2279.3	2746	1595	1502	1051	1077	1002	496.9	520.9	590.1	1690	1553	1693	1758	1697	1540	13
Os01g0772500	1.02	0	1.08	0	0	1.73	0	0	0	0	0	0	0	0.9335	0	0	0	0	0	0	0	0	0	0.722	0	0	0	0	0	0	19
Os01g0149500	14.3	12.1	15.1	137.1	152.8	116.8	51.7	59.5	34.6	7.76	5.482	0	4.3541	3.7338	3.1679	11.94	8.244	8.288	78.03	112.1	94.84	115.3	71.26	79.45	6.338	0	2.193	6.48	2.87	7.5	10
Os01g0530300	8.16	0.93	3.23	47.62	67.8	77.85	23.5	23.4	16.6	6.035	1.096	0.941	3.2656	5.6007	3.9598	11.94	0	7.252	21.37	35.48	17.24	19.96	19.24	19.5	1.056	0	0	10.2	10.5	6.66	10
Os01g0962300	0	0	1.08	0	0	0	0	0	0	0	0	0	0	0	0	0	0	0	0	1.613	0	0	2.138	0	0	0	0	0	0	0	17
Os01g0778400	0	0	0	0	0	0	0	0	0	0	0	0	0	0	0	0	0	0	3.716	0.806	0	0	0.713	0.722	0	0	2.193	0.93	0.96	0.83	11
Os01g0197350	70.3	58.7	92.7	112.5	88.59	83.91	127	169	167	94.84	89.9	69.62	77.285	61.608	67.317	155.2	72.14	82.88	56.67	66.92	52.6	154.5	129	122.8	65.49	65.06	52.63	80.5	66	89.1	18
Os01g0674450	5.1	3.73	1.08	5.747	1.808	3.46	0.78	3.8	0.69	6.035	9.868	1.882	1.0885	0	2.3759	0	2.061	2.072	4.645	4.838	0	2.218	0.713	0	2.113	0.943	0	0.93	2.87	0.83	7
Os01g0323600	72.4	55	61.4	14.78	7.232	9.516	0.78	5.06	4.15	53.46	29.6	28.22	44.629	26.137	50.686	23.88	64.92	61.13	4.645	3.225	6.898	25.14	22.8	14.44	77.11	57.51	67.98	63.8	86.1	73.3	3
Os01g0841800	797	937	911	962.2	1012	869.4	359	561	442	523.3	640.3	477	1012.3	750.5	864.03	1516	700.8	854.7	398.5	455.5	485.4	638.8	573	498.3	664.5	495	370.6	745	746	801	8
Os01g0680400	839	845	925	719.2	660.8	724.9	1082	1058	1116	1253	1218	1247	1492.4	1288.2	1325	1660	973.9	926.2	839.8	733.7	858.8	851	741.9	850.1	1100	1107	1077	1040	915	1015	13
Os01g0917300	22.4	42.8	37.7	144.5	188.9	163.5	218	163	143	36.21	31.8	45.16	28.302	47.606	26.135	71.65	24.73	35.23	188.6	179	181.9	122	124	165.4	45.42	25.46	30.7	26.8	37.3	29.2	12
Os01g0756600	17.3	14	8.62	10.67	12.66	27.68	8.61	7.59	12.4	3.449	7.675	4.704	5.4426	16.802	3.9598	11.94	4.122	11.4	17.65	3.225	7.76	5.176	8.552	7.945	3.169	10.37	6.579	4.63	3.83	4.17	2
Os01g0857500	281	279	341	373.5	310.1	302.8	532	596	527	127.6	173.2	260.6	165.46	143.75	110.88	310.5	247.3	273.5	398.5	346.7	356.1	351.9	307.1	517.8	117.3	165.9	184.2	94.4	130	145	12
Os01g0814700	330	353	350	257	264.9	285.5	416	379	370	351.8	357.4	382.9	375.54	364.98	424.49	788.1	433.9	383.3	297.3	300.7	361.3	391.9	322.8	352.5	394	429	473.7	421	419	468	13
Os01g0347300	6.12	0.93	3.23	0	1.808	0	5.48	4.43	0.69	6.035	3.289	10.35	4.3541	0.9335	8.7116	0	9.275	7.252	3.716	0.806	1.724	0	0.713	6.5	6.338	5.657	10.96	0.93	10.5	1.67	20
Os01g0286550	0	0	1.08	1.642	0.904	0	0	0	0	0	0	0	0	0	0	0	0	0	0	0	0	0	0.713	1.444	0	0	0	0	0.96	0	2
Os01g0835000	63.2	58.7	61.4	96.88	104	124.6	159	144	150	50.87	69.07	68.68	69.665	70.942	54.646	71.65	65.95	39.37	124.5	125	164.7	87.99	90.5	100.4	79.23	50.91	89.91	54.6	61.2	51.7	12
Os01g0306200	0	0	0	1.642	0	0	2.35	0	0	0	0	0	0	0	0	0	0	0	0	0	0	0	0	0.722	0	0	0	0	0	0	18
Os01g0507000	698	523	673	394.1	467.4	327	502	698	377	604.4	601.9	725.4	564.94	574.07	654.96	1242	890.4	786.4	476.6	620.8	489.7	429.6	574.4	381.3	801.8	891	785.1	826	893	642	13
Os01g0688900	0	5.59	0	0	0.904	1.73	3.13	0	0	4.311	2.193	5.645	0	0.9335	0	0	0	0	0	0.806	0	0	0	1.444	1.056	0	10.96	0.93	0.96	0	11
Os01g0852500	336	339	278	220.8	241.4	256.1	363	297	335	338.8	361.8	325.5	458.27	455.52	424.49	632.9	333.9	321.2	221.1	277.4	271.6	332.7	275.1	328.6	365.5	429	403.5	415	383	345	13
Os01g0959200	8.16	22.4	23.7	9.031	2.712	25.09	2.35	5.06	3.46	2.587	7.675	17.88	11.974	6.5342	5.5438	35.82	101	34.19	16.72	0.806	1.724	4.436	16.39	4.333	9.507	0.943	4.386	1.85	0.96	3.33	14
Os01g0170500	11.2	16.8	10.8	1.642	9.04	5.19	1.57	2.53	1.38	5.173	7.675	22.58	5.4426	5.6007	0.792	0	9.275	12.43	2.787	8.869	8.622	4.436	0.713	1.444	9.507	13.2	13.16	9.25	8.61	3.33	20
Os01g0120700	1069	1105	992	879.3	889.5	916.1	1529	1612	1505	981.2	869.4	872.1	1025.4	827.97	983.62	1505	947.1	946.9	923.4	971.6	909.6	1299	1113	1224	948.6	878.7	929.8	874	903	957	18
Os01g0733001	59.1	52.2	51.7	16.42	15.37	13.84	63.4	58.2	69.8	43.11	42.76	24.46	46.806	29.87	31.679	119.4	46.37	38.33	17.65	13.71	14.66	57.67	34.21	95.34	19.01	22.63	28.51	31.5	26.8	37.5	6
Os01g0231000	587	584	467	625.6	654.5	611.6	588	517	514	542.3	592.1	653.9	434.32	589.94	486.27	991.1	589.5	522.2	729.2	792.6	843.2	572.3	641.4	811.1	502.8	529.9	574.5	527	477	500	6
Os01g0872566	4.08	4.66	5.39	9.852	8.136	14.71	3.91	5.06	6.22	4.311	3.289	1.882	1.0885	4.6673	6.3357	0	7.214	7.252	12.08	7.257	9.484	14.05	9.977	10.11	3.169	4.714	8.772	6.48	1.91	5.83	2
Os01g0143300	48.9	33.5	28	105.9	123.8	132.4	50.9	56.3	49.1	60.35	58.11	59.27	57.692	83.077	46.726	23.88	39.16	64.23	183.9	198.3	207.8	30.31	42.76	37.56	36.97	59.4	35.09	46.3	40.2	33.3	10
Os01g0121800	260	284	265	225.8	220.6	207.6	280	205	279	267.3	287.3	216.4	243.83	205.36	228.88	394.1	249.4	214.5	197.9	172.5	212.1	215.9	253	269.4	233.5	249.9	195.2	242	252	281	6
Os01g0924400	1225	1675	1378	1066	994.4	1061	884	817	891	2059	2287	2418	1816.7	1432.8	1770.8	3427	1665	1539	1128	1046	994.1	916.1	832.4	874.6	2050	2030	1732	1545	1606	1691	13
Os01g0905300	105	105	60.3	2.463	7.232	5.19	1.57	1.27	2.76	20.69	8.771	6.586	2.177	6.5342	0	0	30.92	32.12	6.503	0	6.036	4.436	6.414	5.056	6.338	13.2	6.579	3.7	0.96	7.5	14
Os01g0167400	38.7	71.7	39.9	82.92	68.7	57.09	123	93	133	62.08	80.04	99.73	60.957	53.207	57.022	131.4	61.83	44.55	85.46	67.73	48.28	137.5	117.6	126.4	57.04	69.77	87.72	37	45.9	42.5	18
Os01g0621600	2.04	6.52	5.39	2.463	4.52	4.325	2.35	3.16	6.22	2.587	2.193	0.941	5.4426	0	0	11.94	6.183	4.144	0.929	7.257	1.724	10.35	13.54	16.61	5.282	1.886	0	8.33	1.91	5	9
Os01g0750100	1066	958	901	1025	799.1	908.3	750	932	1041	705.3	711.6	650.1	818.57	577.81	860.08	895.6	643.1	707.6	877.9	873.2	995	1029	1273	1082	652.8	654.3	570.2	568	645	667	17
Os01g0897700	22.4	15.8	12.9	9.031	8.136	29.41	13.3	13.9	37.3	11.21	15.35	15.05	5.4426	4.6673	5.5438	11.94	17.52	17.61	13.01	4.031	8.622	12.57	12.11	39	5.282	12.26	8.772	10.2	23	15.8	9
Os01g0911300	2823	2425	2998	874.3	715	932.5	944	1085	1165	3170	3356	4333	3936.1	3922.4	3980.4	6412	4010	3395	1539	1286	1325	932.4	894.4	1167	4118	4452	3985	3391	3533	3227	13
Os01g0551000	1782	1580	1541	413.8	199.8	274.2	488	525	498	1521	1386	1225	999.26	864.38	1020.1	2567	2000	1555	536	412	425.9	607	692.7	853.7	1256	1188	1439	1106	1183	1151	13
Os01g0170300	0	0	0	0	0	0	0	0	0	1.724	0	0	0	0	0	0	0	0	0	0.806	0	0	0	0	0	2.829	0	0	3.83	0	1
Os01g0187600	3.06	0	0	0	0	0	0	0	0	0	0	0	1.0885	0	0	0	0	0	0	0	0	0	0.713	0	0	0	0	0	0	0	19
Os01g0310500	891	1039	1004	918.7	880.5	851.2	398	524	451	549.2	694	534.4	673.79	656.22	790.38	1731	978	1201	630.8	774	759.6	1055	870.1	706.4	668.7	741.1	530.7	1105	1141	1088	8
Os01g0850400	18.4	6.52	12.9	14.78	24.41	24.22	1.57	6.33	6.22	1.724	3.289	4.704	8.7082	8.4011	8.7116	0	1.031	2.072	22.29	18.54	13.8	4.436	5.701	7.945	5.282	4.714	0	4.63	0.96	2.5	10
Os01g0701900	207	219	216	1554	1330	1753	178	280	166	112.9	140.3	140.2	137.15	143.75	163.14	262.7	220.5	215.5	1471	1843	1406	334.9	340.6	231.1	122.5	149	144.7	272	253	242	10
Os01g0955100	675	1729	452	136.3	36.16	38.93	8.61	10.8	15.2	69.84	33.99	47.98	75.108	23.336	45.142	680.6	1119	603	25.08	38.7	189.7	22.18	31.36	276.6	27.47	23.57	35.09	25.9	37.3	89.1	14
Os01g0282800	5.1	4.66	4.31	25.45	32.54	53.63	7.83	10.1	7.6	18.97	12.06	14.11	5.4426	12.135	9.5036	0	14.43	9.324	33.44	9.675	37.08	6.654	3.563	15.89	8.451	13.2	19.74	9.25	10.5	2.5	10
Os01g0376700	1037	985	1208	1066	1119	1039	1422	1686	1442	913.1	1021	893.8	964.43	1157.5	1134.1	1863	1105	1027	1074	1056	1039	917.6	938.5	1153	834.5	759	782.9	1148	1253	1064	6
Os01g0506200	310	297	327	325.9	289.3	315.7	337	334	358	252.6	297.1	316.1	372.27	323.91	316.79	609	309.2	307.7	283.3	362	322.5	337.2	270.1	348.8	273.6	282.9	232.4	321	300	331	6
Os01g0389200	4.08	25.1	10.8	4.926	6.328	2.595	0	3.8	2.07	6.035	0	1.882	0	0	0	11.94	1.031	8.288	2.787	4.838	0	26.62	14.25	22.39	0	0	0	0	2.87	0	9
Os01g0897100	61.2	31.7	70	44.33	46.1	55.36	65	76.5	69.1	71.56	95.39	66.8	70.754	59.741	74.445	143.3	83.47	36.26	51.09	57.25	52.6	57.67	64.85	48.39	71.83	82.03	87.72	80.5	70.8	65	13
Os01g0768300	4.08	0	2.16	0.821	0	2.595	4.7	1.27	1.38	0	1.096	0	0	0	1.5839	0	3.092	0	0.929	2.419	0	2.958	3.563	0.722	0	0	0	0	0	0	17
Os01g0948500	478	353	389	109.2	104	118.5	101	99.3	93.3	101.7	89.9	88.44	107.76	126.02	128.3	274.6	218.5	288	107.8	130.6	109.5	139	158.9	132.9	65.49	116.9	118.4	120	116	142	14
Os01g0234800	179	203	199	172.4	207.9	194.6	293	260	296	279.3	246.7	263.4	239.47	252.97	252.64	274.6	230.8	230	183	195.1	183.7	253.6	269.4	247.7	204.9	259.3	271.9	253	252	270	5
Os01g0927300	15.3	5.59	4.31	27.91	12.66	64.01	18	22.8	6.91	6.035	9.868	6.586	19.593	8.4011	6.3357	0	13.4	16.58	85.46	41.93	122.4	17.75	24.23	35.39	8.451	14.14	32.89	26.8	35.4	6.66	16
Os01g0738600	1211	1231	1259	900.6	1016	936	788	920	751	972.5	998.8	830.7	969.87	896.11	1014.5	1994	997.6	1271	768.2	1022	893.3	893.2	992.7	754.7	922.2	817.4	673.2	1071	1056	1178	13
Os01g0728150	3.06	1.86	0	0	0	0	0	1.9	0	0	0	0	7.6196	0	0	0	0	0	0.929	0	0	0	0	0	0	2.829	4.386	0	0.96	0	4
Os01g0326000	3.06	0	4.31	2.463	0.904	17.3	3.13	5.06	4.15	0	3.289	4.704	2.177	8.4011	0.792	0	2.061	2.072	3.716	0	12.07	4.436	3.563	1.444	0	4.714	21.93	2.78	0.96	0	11
Os01g0633800	0	4.66	10.8	0	3.616	0.865	0	0	0	0	2.193	1.882	0	0	0	0	2.061	4.144	0	3.225	0	1.479	3.563	0	0	0.943	0	0.93	1.91	3.33	14
Os01g0754000	37.7	37.3	22.6	96.05	132	107.3	62.6	44.3	71.2	39.66	13.16	28.22	26.124	29.87	11.879	35.82	17.52	32.12	87.32	116.9	125.9	42.15	41.33	26	24.3	25.46	10.96	20.4	25.8	17.5	10
Os01g0948900	112	89.4	120	131.4	133.8	100.3	118	149	106	169	130.5	130.8	148.04	108.28	166.31	226.9	75.23	94.28	105	74.18	44.84	73.2	66.28	65	90.85	66	61.4	82.3	108	102	6
Os01g0225000	19.4	17.7	19.4	4.105	16.27	12.11	71.2	75.9	54.6	19.83	20.83	4.704	44.629	27.07	30.887	11.94	37.1	21.76	33.44	5.644	15.52	17.75	29.93	38.28	16.9	25.46	43.86	75.9	73.6	45.8	5
Os01g0700200	9.18	14.9	3.23	4.105	0	3.46	12.5	1.9	8.29	0.862	7.675	9.408	1.0885	3.7338	0.792	47.76	10.31	9.324	1.858	8.063	0.862	8.873	5.701	19.5	2.113	2.829	6.579	7.4	12.4	4.17	6
Os01g0960500	1187	1405	1303	1035	1051	968.9	1856	1965	1716	1415	1567	1505	1439	1300.3	1578.4	2854	1447	1357	1148	1177	1139	1463	1586	1415	1553	1462	1382	1504	1463	1535	6
Os01g0533450	0	0.93	0	0	0	0	0	0	0	0	0	0	0	0	0	0	0	0	0	0	0.862	0	0	0	0	0	0	0	0	0	14
Os01g0691500	1382	1231	1281	1302	1485	1195	1623	1647	1663	1422	1557	1674	1664.3	1488.9	1514.2	2221	1173	1310	1267	1400	1336	1411	1171	1455	1623	1426	1346	1274	1204	1288	6
Os01g0685300	1.02	2.79	1.08	2.463	0	3.46	3.91	2.53	0.69	0	3.289	0	1.0885	1.8669	0.792	0	3.092	0	1.858	0.806	1.724	2.958	2.138	0.722	0	0	2.193	0	0.96	0.83	12
Os01g0670800	396	373	361	596	623.7	479.2	336	428	380	281.9	322.3	321.8	303.7	302.44	281.15	477.6	344.2	346	507.2	453.9	411.3	439.2	495.3	483.9	323.2	264.9	214.9	320	291	377	10
Os01g0963400	1000	1102	1131	1327	1299	1256	1859	1837	1896	1280	1422	1676	1393.3	1360	1256.8	2293	1269	1328	1954	1816	2029	1298	1322	1687	1331	1542	1474	978	1117	1103	6
Os01g0226250	14.3	5.59	5.39	4.926	6.328	8.65	2.35	4.43	0.69	1.724	2.193	5.645	11.974	7.4676	5.5438	0	6.183	1.036	9.29	15.32	5.173	2.958	3.563	5.778	6.338	7.543	4.386	5.55	11.5	9.16	16
Os01g0534800	870	974	836	409.7	377.9	358.1	409	335	422	481.1	504.3	474.2	533.37	532.07	605.06	1027	685.3	640.3	344.6	347.5	418.2	430.3	427.6	464.4	533.5	509.1	589.9	554	481	498	13
Os01g0505500	25.5	26.1	24.8	77.17	76.84	64.88	20.4	28.5	29	18.97	10.96	15.99	5.4426	19.602	13.463	11.94	12.37	12.43	47.38	49.99	49.15	26.62	37.77	33.22	20.07	12.26	43.86	16.7	24.9	10.8	10
Os01g0750300	5.1	4.66	4.31	22.99	37.97	39.79	7.05	6.33	9.67	25.87	7.675	2.822	0	11.201	0	11.94	11.34	3.108	6.503	7.257	11.21	10.35	6.414	4.333	2.113	3.771	0	32.4	31.6	27.5	2
Os01g0112600	11.2	21.4	5.39	0	0	0	0	0	0	0	0	0	0	0	0	11.94	6.183	3.108	0	0	0	0.739	0	0.722	0	0	0	0	0	0	14
Os01g0933200	645	657	818	1130	1341	1011	1742	2080	1325	869.9	728	744.2	861.02	883.05	856.12	1839	778.1	743.9	732.9	1043	888.1	782.3	1047	696.2	716.2	589.3	635.9	1346	1241	955	6
Os01g0715700	2.04	7.45	4.31	34.48	24.41	28.55	22.7	13.9	10.4	4.311	4.386	0	4.3541	2.8004	3.9598	0	0	0	24.15	25.8	31.9	16.27	14.97	13	3.169	0	6.579	1.85	2.87	2.5	10
Os01g0110200	4.08	6.52	1.08	9.852	15.37	25.09	3.91	2.53	2.76	31.9	5.482	12.23	0	4.6673	0.792	35.82	18.55	1.036	2.787	3.225	11.21	0	0	0	3.169	4.714	19.74	5.55	12.4	4.17	6
Os01g0144600	511	414	567	373.5	358.9	382.4	526	552	564	503.5	482.4	532.5	532.29	486.33	472.8	1433	487.4	473.5	497	429.7	478.5	476.2	449	437	501.8	561	484.6	461	452	473	6
Os01g0770100	76.5	85.7	52.8	59.93	48.81	53.63	33.7	43.6	24.2	66.39	99.77	55.51	76.196	44.806	53.854	107.5	56.68	49.73	61.31	42.73	43.11	28.1	43.47	43.33	79.23	116.9	92.1	116	81.3	91.6	13
Os01g0255700	12.2	14.9	11.9	11.49	8.136	9.516	18.8	9.49	6.22	14.66	16.45	13.17	17.416	11.201	11.088	23.88	17.52	17.61	7.432	5.644	11.21	12.57	10.69	23.83	17.96	18.86	13.16	24.1	12.4	14.2	13
Os01g0923600	670	770	710	731.5	717.8	664.4	700	790	651	732.9	620.6	557	578	585.27	692.18	1302	647.2	676.5	565.7	613.6	513.9	851.8	1013	763.4	665.5	613.8	662.3	838	817	752	6
Os01g0931200	2.04	0.93	1.08	0	0.904	0	0.78	0	0	2.587	1.096	0.941	2.177	0	0	0	0	0	0	0.806	0	0.739	0	0.722	0	2.829	0	0	0	0	7
Os01g0654100	205	317	262	289	254	372	66.5	49.3	65.6	496.6	399.1	239	519.22	798.1	660.5	334.3	401.9	293.2	372.5	419.3	498.4	59.15	58.44	50.56	490.2	530.8	425.4	427	762	683	3
Os01g0566900	2750	3004	2750	2590	2603	2232	4309	4187	3993	3588	3480	3447	2820.4	2744.3	3220.9	3487	2353	2543	2426	2159	2178	3097	3114	3310	2999	2898	2958	2363	2379	2242	5
Os01g0769000	936	955	1015	1279	1236	1332	526	544	586	508.7	566.8	363.2	635.7	596.48	677.13	1719	915.1	822.6	932.7	1068	1104	826.6	737.6	797.4	639.1	558.2	585.5	799	735	728	6
Os01g0776800	379	361	366	392.4	368.8	336.5	461	509	388	399.2	362.9	298.2	346.15	325.77	345.3	489.6	360.7	331.5	353.9	379.8	361.3	383.7	412.6	364.7	320.1	283.8	366.2	328	376	323	6
Os01g0125900	909	915	890	1020	1018	968.9	787	906	899	839.8	910	745.1	868.64	861.58	868.79	1528	921.3	809.1	841.6	809.5	776	1061	1085	923	899	826.9	714.9	887	880	971	6
Os01g0638600	425	459	417	246.3	199.8	268.2	58.7	92.3	44.9	321.6	346.5	349	259.07	533	426.08	967.2	731.7	600.9	258.2	308	371.6	238.1	325.7	283.1	632.8	771.2	596.5	1016	756	719	13
Os01g0743800	338	346	324	264.4	290.2	341.7	316	354	297	363	347.6	387.6	392.96	434.99	361.14	489.6	425.6	445.5	452.4	557.1	666.5	587.1	574.4	495.5	413	475.2	432	502	528	454	1
Os01g0539400	3100	3305	3363	3488	3698	3506	4456	4514	4217	3232	3619	3797	3722.7	3504.2	3478.3	5851	4179	4024	3855	4547	3825	5321	3999	4011	4121	4093	3945	3909	4273	4254	6
Os01g0857000	1023	911	1002	671.6	761.1	801	449	562	457	903.6	893.6	790.3	979.67	886.78	974.12	2149	865.6	931.4	590.8	735.3	635.5	762.3	734.7	670.2	928.5	884.4	855.2	1001	992	1021	13
Os01g0183000	105	107	123	86.2	95.82	78.72	122	165	124	112.1	116.2	98.79	94.701	105.48	121.96	107.5	77.29	110.9	106.8	96.75	72.43	81.33	98.34	83.78	105.6	69.77	83.33	74	89.9	112	5
Os01g0667750	0	0	0	0	0	0	0	0.63	0	0	0	0	0	0	0	0	0	2.072	0	0	0	0	0	0	0	0	0	0	0	0.83	1
Os01g0959600	8.16	15.8	11.9	63.22	54.24	43.25	22.7	40.5	26.9	5.173	5.482	1.882	17.416	2.8004	11.879	23.88	5.153	6.216	26.01	33.86	41.39	29.58	41.33	34.67	10.56	0.943	0	1.85	12.4	5.83	12
Os01g0750800	852	746	864	647.7	693.3	698.1	858	629	824	923.4	975.8	1157	904.56	1042.7	824.44	919.5	874.9	815.4	977.3	861.1	906.2	617.4	605.7	750.4	930.7	912.7	1059	796	762	704	20
Os01g0950700	1.02	1.86	5.39	1.642	6.328	3.46	0	0.63	0	0.862	0	0	0	0	0	0	1.031	1.036	1.858	4.031	1.724	2.958	2.138	1.444	0	0.943	0	0	0	0.83	2
Os01g0174200	11.2	11.2	14	8.21	0	8.65	11.7	15.2	11.7	9.484	9.868	11.29	9.7967	10.268	7.1277	23.88	7.214	18.65	6.503	8.063	6.898	11.83	13.54	9.389	12.68	16.03	6.579	17.6	25.8	5.83	13

Os01g0184050	0	0	0	0	0	0	0	0	0	0.862	0	0	0	0	0	0	0	0	0	0	0	0	0	0	0	0	0	0	0	0	7
Os01g0100400	35.7	29.8	30.2	57.47	64.18	59.69	106	78.4	96.7	30.18	26.31	31.05	29.39	21.469	17.423	47.76	19.58	36.26	56.67	64.5	76.74	67.28	59.86	70.06	29.58	65.06	35.09	25	36.3	19.2	12
Os01g0327600	353	361	359	389.1	442	388.4	300	331	336	288.8	345.4	290.7	357.03	310.84	349.26	537.3	309.2	357.4	269.4	315.3	326.8	425.9	383.4	296.8	308.5	341.3	256.6	369	342	329	6
Os01g0678950	1.02	2.79	1.08	0	0	0	0	0	0	0	0	2.822	0	2.8004	1.5839	0	1.031	0	0	0	0	1.479	1.425	0	0	1.886	0	0.93	0	0.83	14
Os01g0549400	1088	1082	1053	1162	1182	1150	953	967	1017	1179	1071	969	1291	1043.6	1233.1	2114	1015	1080	941	1013	978.6	1091	1027	949	1167	1110	1031	1168	1204	1171	6
Os01g0725800	6164	5444	6566	1357	1275	1377	2595	3305	3017	5460	5996	6305	7997.4	6617.2	7298	14902	7563	6934	1652	1955	1438	2479	2666	2923	6993	6732	5713	6601	6424	7092	13
Os01g0186600	25.5	82	72.2	96.88	152.8	50.17	459	553	252	258.7	217.1	28.22	102.32	113.88	183.74	238.8	24.73	21.76	153.3	108.8	50.01	136	228.8	54.89	169	11.31	8.772	32.4	60.2	81.6	18
Os01g0695800	817	641	822	258.6	201.6	338.2	236	420	366	558.7	323.4	347.2	277.57	330.44	451.42	656.8	371	398.9	323.3	446.7	373.3	350.5	732.6	287.5	269.4	291.3	425.4	308	349	252	14
Os01g0582600	1774	1673	1333	215.1	89.49	179.1	137	190	191	398.3	286.2	305.8	374.45	257.63	330.25	1755	1693	1121	191.4	188.7	404.4	193.7	193.8	620.4	348.6	347	302.6	284	297	500	14
Os01g0113300	5.1	4.66	0	0	0	0	4.7	0.63	1.38	0	0	0	0	0	1.5839	0	1.031	1.036	0	0	0	4.436	2.851	2.167	0	0	0	0	0	0	9
Os01g0787400	327	289	272	110	121.1	113.3	121	109	77.4	283.7	269.7	284.1	323.29	278.17	281.15	406	364.8	323.2	196	155.6	151.8	78.37	81.95	122.1	281	268.7	236.8	296	250	307	3
Os01g0622700	2447	1970	2240	881.7	897.6	977.5	1283	1328	1198	2128	1953	2123	2391.5	2359.8	2303.8	3009	2586	2178	1229	1287	1315	1302	1545	1533	2273	2615	2408	2268	2040	1958	3
Os01g0137232	0	1.86	0	0.821	1.808	0	0	0.63	0	0.862	0	0	0	0.9335	0.792	0	0	0	0	3.225	3.449	0	0	0	0	0	0	0	0	0.83	16
Os01g0273800	2.04	5.59	10.8	7.389	17.18	9.516	75.9	45.5	19.3	31.04	8.771	6.586	4.3541	2.8004	6.3357	35.82	5.153	10.36	5.574	29.83	12.93	14.79	17.82	8.667	5.282	7.543	4.386	13.9	5.74	5	18
Os01g0309451	0	0	0	0	0	0	0.78	5.69	0	0	0	0	1.0885	0	0	0	0	0	0	0	0	0	0	0	1.056	0	0	0.93	0.96	0.83	5
Os01g0104600	1218	1126	1182	762.7	754.8	723.2	830	975	917	1191	1312	1383	1343.2	1295.6	1263.2	2161	1510	1301	811	823.2	945.9	792.6	761.8	733.8	1508	1547	1379	1190	1234	1365	13
Os01g0570500	1926	2165	2005	1542	1571	1417	1732	1572	1529	1793	1947	1921	1847.2	1776.4	1924.5	2866	2147	2073	1478	1422	1568	1809	1765	1923	2014	2017	1919	1976	1895	2118	13
Os01g0517900	0	0	0	0	0	0	0	0	0	0	0	0	0	0	0.792	0	1.031	1.036	0	0	0	0	0	0	0	0	0	0	0	0	1
Os01g0864200	644	582	657	241.4	293.8	207.6	374	326	283	432	224.8	210.7	265.6	248.3	292.24	609	415.3	375	234.1	266.1	220.7	281.7	310.7	370.5	202.8	122.6	186.4	204	225	189	14
Os01g0681200	115	151	144	146.1	165.4	200.7	241	186	214	167.3	143.6	139.2	126.27	154.95	144.14	179.1	120.6	111.9	147.7	129.8	146.6	179.7	208.1	197.9	146.8	110.3	116.2	167	121	108	18
Os01g0566200	4.08	4.66	3.23	11.49	16.27	6.92	6.26	5.06	10.4	4.311	4.386	1.882	1.0885	6.5342	2.3759	11.94	6.183	5.18	13.93	6.45	9.484	6.654	9.264	5.778	5.282	3.771	4.386	0.93	2.87	2.5	10
Os01g0783400	1.02	1.86	2.16	0	0	1.73	1.57	0.63	2.07	0	1.096	0	0	0	0.792	11.94	4.122	2.072	0.929	1.613	6.898	0.739	2.138	0	2.113	0.943	4.386	5.55	4.78	0.83	6
Os01g0634500	0	0	0	0	0	0	0	0.63	0	0	0	0	0	0	0	0	0	0	0	0	0	0	0	0	0	0	0	0	0	0	18
Os01g0328700	2638	2718	2619	2364	2570	2157	4776	5195	5425	2239	2505	3871	2453.5	2423.2	2133.6	4657	3100	2796	2722	2268	2473	3741	3337	5246	2709	3255	3044	1889	2455	2262	18
Os01g0508000	568	491	676	203.6	207	167.8	350	266	314	623.4	550.4	511.8	655.29	653.42	521.11	1194	432.8	493.2	207.2	244.3	250.9	159	147.5	203	498.6	382.8	407.9	419	528	433	13
Os01g0385400	5.1	3.73	2.16	1.642	1.808	3.46	0	1.27	0	1.724	1.096	0.941	0	0	1.5839	11.94	1.031	3.108	0	1.613	0.862	0	1.425	0	1.056	0.943	0	0.93	0.96	1.67	6
Os01g0206800	2.04	2.79	0	1.642	0	0	0	1.9	0	0	0	0.941	1.0885	1.8669	0.792	0	0	0	0	0.806	0.862	2.958	0.713	1.444	1.056	3.771	0	0.93	2.87	0.83	1
Os01g0767900	867	869	943	789.8	761.1	784.6	775	653	823	891.5	979.1	949.3	865.37	839.17	807.81	1457	975.9	834	738.5	864.3	938.1	751.2	671.3	679.6	887.3	989	1055	923	797	896	13
Os01g0825900	135	143	147	97.7	91.3	91.7	73.6	57.6	77.4	125	142.5	116.7	144.77	95.212	127.51	286.6	117.5	128.5	68.74	97.56	69.84	85.77	106.2	96.06	122.5	93.34	120.6	153	163	145	13
Os01g0879000	0	0.93	0	0.821	0	1.73	0	1.9	1.38	0	0	0	0	0	1.5839	0	2.061	0	0	0	1.724	1.479	0	0	0	0	0	0	0	0	2
Os01g0200350	15.3	14.9	10.8	0	0	1.73	0.78	0.63	2.07	4.311	3.289	1.882	6.5311	8.4011	5.5438	23.88	12.37	7.252	0	2.419	0	5.176	5.701	10.11	1.056	9.428	0	14.8	9.56	21.7	13
Os01g0273000	548	513	566	499.2	527.9	588.2	686	689	698	583.7	627.1	668	524.67	593.68	502.11	764.2	519.4	535.6	637.3	555.5	656.1	495.4	476.8	521.5	563	622.3	671	477	539	457	15
Os01g0586600	526	494	495	559.9	584	474	666	667	524	423.3	502.1	401.7	365.74	428.45	407.07	609	477.1	479.7	514.6	450.7	435.4	691.3	654.9	602.4	455.3	411.1	418.8	465	484	439	17
Os01g0687900	0	0	1.08	0	0	0	0	0	0	1.724	0	0	0	0.9335	0	0	0	1.036	0	0.806	0	0.739	0	0.722	0	0	0	0.93	0.96	0.83	1
Os01g0139600	0	0	0	0	0	0	0	0	0	0	0	0	0	0	0.792	0	0	0	0	0	0	0	0	0	0	0	0	0	0	0	4
Os01g0223300	1.02	4.66	1.08	3.284	0	3.46	0	0	2.76	3.449	5.482	3.763	1.0885	0.9335	1.5839	0	5.153	2.072	0	1.613	2.587	2.218	2.851	2.889	9.507	9.428	13.16	0.93	3.83	5	11
Os01g0752200	411	421	461	348.9	416.7	431.7	388	452	598	494	432	366	375.54	439.66	414.2	394.1	396.8	342.9	380.9	382.2	326.8	391.1	520.2	484.6	350.7	345.1	475.9	388	392	373	18
Os01g0837300	185	208	189	233.2	207	286.3	261	304	297	191.4	209.4	153.4	163.28	189.49	178.19	143.3	249.4	222.7	310.3	325.7	272.5	265.4	268.7	291.8	162.7	205.5	245.6	166	191	152	12
Os01g0904500	70.3	95	92.7	116.6	107.6	122.8	93.2	74	80.8	60.35	55.92	50.8	52.249	49.473	64.941	143.3	51.53	67.34	69.67	91.11	96.57	72.46	84.8	74.39	50.71	79.2	81.14	70.3	71.7	48.3	6
Os01g0308700	0	0	0	0	0	0	1.57	0	0	0	0	0	0	0.9335	0	0	1.031	0	0	0	0	0	0	0	0	0	0	0	0	0	5
Os01g0338401	0	0	0	0	0	0	0	0	0	0	0	0	1.0885	0	0	0	0	0	0	0	0	0	0	0	0	0	0	0	0	0	4
Os01g0190500	0	0	0	0	0	0	0	0	1.38	1.724	0	0	0	0	0	0	0	0	0	0	0	0	0	0	0	0	0	0	0	0	7
Os01g0311300	1.02	1.86	4.31	12.31	11.75	3.46	10.2	8.22	1.38	0.862	3.289	0.941	1.0885	3.7338	3.1679	11.94	2.061	1.036	4.645	9.675	3.449	1.479	2.851	4.333	0	0.943	0	0.93	0	0	10
Os01g0239200	234	298	357	403.1	321.8	287.2	290	370	404	447.5	461.6	414.9	360.3	278.17	335.79	525.4	370	441.4	305.6	174.2	308.7	299.5	277.2	294	438.4	304.5	182	318	267	448	13
Os01g0114000	5.1	3.73	5.39	0.821	0	3.46	0	0.63	0.69	0.862	5.482	1.882	1.0885	5.6007	7.9197	0	3.092	4.144	0.929	4.031	4.311	1.479	2.851	0.722	6.338	0.943	2.193	13	3.83	6.66	1
Os01g0935900	704	726	753	658.4	631	654.8	1390	1111	1400	1044	939.6	1145	791.35	894.25	797.51	1146	698.7	660	872.3	729.7	833.8	865.1	922.9	1003	806	854.2	1028	649	631	636	5
Os01g0698300	217	209	197	183.9	225.1	216.3	225	266	259	226.8	245.6	223	210.08	224.03	251.85	286.6	229.8	223.8	168.1	158.8	207.8	259.5	262.2	296.1	248.2	231.9	219.3	192	220	231	5
Os01g0167100	868	1244	1151	457.3	626.5	348.6	1084	1050	717	2668	2142	1544	1590.3	1024.9	1490.5	1099	557.5	779.1	451.5	434.6	251.8	1040	1309	749.7	1352	908.9	892.5	1251	1060	1175	7
Os01g0666200	195	162	183	187.2	230.5	232.7	380	412	413	242.3	245.6	266.3	293.9	270.7	261.35	298.5	215.4	218.6	295.4	266.1	256.1	272.1	284.3	286	201.8	210.3	239	174	183	187	18
Os01g0356400	6406	6154	7026	2518	2542	2356	4193	5056	4679	4890	5401	4309	7522.8	6235.5	7549	14699	7303	6374	2908	2961	2600	4145	4647	4112	5791	5670	4498	7225	6942	7272	13
Os01g0843500	12.2	17.7	7.54	18.88	16.27	9.516	28.2	32.3	29	26.73	30.7	22.58	18.505	25.203	25.343	11.94	12.37	33.15	12.08	6.45	6.036	19.22	18.53	15.17	17.96	14.14	10.96	11.1	15.3	18.3	5
Os01g0138000	0	0	0	0.821	0	0	0	0.63	0	0	0	0	0	0	0	0	0	0	0	0	0	0	0	0	0	0	0	0	0	0	2
Os01g0118600	4.08	0.93	4.31	10.67	2.712	12.98	0.78	3.16	8.98	4.311	3.289	0.941	0	3.7338	1.5839	0	6.183	1.036	2.787	4.031	6.036	0.739	3.563	6.5	4.225	4.714	0	10.2	9.56	0.83	2
Os01g0208400	0	0	0	3.284	3.616	2.595	3.13	1.9	6.22	0	0	0	0	0	0	0	0	0	0	1.613	4.311	7.394	4.276	4.333	0	0	0	0.93	0	0.83	12
Os01g0640200	0	0	0	0	0	0	0	0	0	0	0	0	0	0	0	0	0	0	0	0	0	0	0	0	0	0.943	0	0	0	0	1
Os01g0650700	458	525	484	484.4	491.8	493.9	580	676	592	527.7	552.6	509	621.54	521.8	552.79	704.5	494.7	505.6	560.2	566.8	578.5	566.4	531.6	561.9	614.8	605.3	480.2	455	528	516	6
Os01g0339500	371	385	398	311.1	340.8	373.7	677	623	592	503.5	569	561.7	350.5	371.51	422.91	453.8	418.4	398.9	303.8	342.7	352.6	664.7	509.5	533	399.3	432.8	576.7	404	427	370	5
Os01g0376200	68.3	80.1	77.6	68.96	61.47	70.07	54.8	50.6	54.6	54.32	83.33	75.27	59.869	63.475	53.854	107.5	84.5	72.52	60.38	59.66	57.77	59.89	44.9	56.34	69.72	72.6	92.1	62	77.5	64.2	13
Os01g0709100	0	0	0	0	0.904	0	0	0	0	0	0	0	0	0	0	0	0	0	0	0	0	0	0	0	0	0	0	0	0	0	2
Os01g0888500	575	512	516	2283	2386	2170	832	1023	1007	447.5	396.9	572	472.42	517.13	448.25	979.2	426.6	559.5	1561	1584	1685	942	914.3	900.6	450	533.6	458.3	465	495	441	10
Os01g0369000	1747	1993	1944	2052	2116	2040	2341	2582	2279	1465	1544	1470	1825.4	1830.5	1863.5	3821	1998	1782	1785	1617	1806	2541	2489	2458	1838	1625	1647	2218	1846	2069	6
Os01g0305200	739	548	486	255.3	170.9	295	188	201	272	325	341	273.8	281.93	275.37	372.22	740.3	586.4	406.1	503.5	322.5	517.3	376.3	474.6	322.1	378.2	437.5	552.6	422	379	296	13
Os01g0827500	700	578	711	439.2	420.3	418.7	363	468	408	610.4	634.8	537.2	710.8	603.94	654.16	1027	592.6	575	332.6	332.2	367.3	405.9	411.2	424	680.3	539.3	552.6	654	577	596	13
Os01g0730100	14.3	14.9	7.54	35.3	25.31	20.76	21.9	5.06	11.7	16.38	21.93	20.7	6.5311	10.268	15.047	11.94	11.34	15.54	15.79	16.93	18.97	12.57	8.552	8.667	3.169	10.37	35.09	6.48	16.3	8.33	11
Os01g0642700	6.12	16.8	16.2	28.73	11.75	30.28	24.3	10.8	15.2	6.897	2.193	18.82	19.593	3.7338	9.5036	35.82	13.4	11.4	13.01	21.77	9.484	73.94	51.31	53.45	12.68	4.714	4.386	25.9	34.4	10	9
Os01g0877900	6.12	2.79	1.08	6.568	4.52	2.595	4.7	4.43	8.29	8.622	4.386	12.23	2.177	5.6007	4.7518	11.94	3.092	4.144	5.574	0.806	0.862	2.958	2.851	2.889	1.056	3.771	4.386	7.4	3.83	2.5	6
Os01g0595800	0	0.93	1.08	0	0	0.865	0	0	0	0	0	0	3.2656	0.9335	0	23.88	2.061	1.036	0	0	0	1.479	0	0	0	0	4.386	0.93	0.96	0	6
Os01g0534900	322	316	248	504.9	401.4	538.9	232	171	151	239.7	292.7	322.7	211.17	299.64	206.7	310.5	174.2	264.2	424.5	413.6	560.4	281.7	289.3	309.1	217.6	210.3	285.1	315	248	240	10
Os01g0234300	6.12	9.31	7.54	9.852	10.85	12.98	4.7	1.9	1.38	8.622	1.096	0	4.3541	3.7338	4.7518	11.94	2.061	6.216	3.716	0	2.587	0.739	3.563	0.722	6.338	6.6	8.772	5.55	7.65	10.8	8
Os01g0731000	401	360	384	344.8	320	333	458	445	406	451.8	471.4	531.6	411.46	460.19	527.45	609	490.5	437.2	436.6	393.5	470.8	283.9	336.4	330.1	487	445	445.2	371	399	397	13
Os01g0940800	193	168	158	157.6	192.5	92.56	362	177	616	158.6	105.3	283.2	112.12	34.538	99.788	119.4	32.98	53.87	123.6	71.76	31.9	150.1	475.3	348.1	28.52	121.6	223.7	67.5	215	38.3	18
Os01g0370000	2.04	2.79	7.54	2.463	0	0.865	1.57	0.63	0	0	6.578	4.704	1.0885	0.9335	3.1679	11.94	4.122	7.252	0	0.806	0	4.436	2.851	0.722	6.338	4.714	4.386	6.48	11.5	2.5	13
Os01g0912700	8805	7690	8859	3455	3178	3676	5968	6019	6444	12495	13497	16062	13850	12397	13416	15153	11869	10659	5791	5387	6430	4029	3810	5470	13373	14859	12166	9680	####	####	3
Os01g0288200	0	0.93	0	0	0	0	2.35	0	0.69	0	0	2.822	1.0885	0.9335	0	0	0	0	0	0.806	0.862	0	0	0.722	0	0	0	0	0	0	15
Os01g0513100	408	386	434	174.9	174.5	214.5	279	244	273	290.6	262	255	243.83	293.1	257.39	394.1	409.1	393.7	198.8	173.4	195.7	297.2	267.2	288.2	270.4	281.9	328.9	263	269	304	14
Os01g0799500	1.02	0	0	0.821	0	0	0	0	8.98	0	0	0.941	0	0	0	0	0	0	0	0	0	0	0	0	0	0	0	0	0	0	18
Os01g0814000	273	294	261	771.7	792.8	722.3	706	658	609	368.2	307	261.5	369.01	451.79	351.63	967.2	300.9	327.4	858.4	818.4	918.3	434.8	395.5	369.8	262	264	230.3	303	340	322	10
Os01g0802600	0	0	0	0.821	0	0	0	1.27	0	0	0	0	0	0	0	0	0	0	0	0	0	0	0	0	0	0	0	0	0.96	0	2
Os01g0720300	150	150	199	178.2	177.2	183.4	193	203	216	182.8	171	203.2	174.16	150.29	182.94	214.9	218.5	247.6	222	151.6	251.8	179.7	168.2	204.4	176.4	210.3	188.6	191	193	170	16
Os01g0114600	1.02	0	1.08	0	0	0	0	0	0.69	0.862	0	0	1.0885	0.9335	1.5839	0	0	1.036	0	0	0	0.739	0.713	0	1.056	0.943	2.193	4.63	1.91	2.5	1
Os01g0559525	272	287	261	305.4	342.6	273.4	415	436	417	300	334.4	309.5	401.66	382.72	353.22	298.5	306.1	282.8	455.2	479.7	523.4	272.1	298.6	340.9	249.3	360.2	396.9	294	273	276	15
Os01g0755600	0	0	0	0.821	0	0	3.91	1.27	2.07	0	0	0	0	1.8669	0	0	0	1.036	2.787	0	2.587	0.739	0	0.722	2.113	1.886	4.386	0	0.96	0.83	15
Os01g0175700	716	475	613	21.35	43.39	20.76	3.13	1.27	3.46	953.6	1136	910.7	1643.7	1358.2	1374.1	776.2	1050	892	47.38	66.92	25.87	0	0.713	2.889	1607	1263	947.3	1289	1289	1325	3
Os01g0248500	118	137	119	177.3	162.7	131.5	369	570	471	169	130.5	89.38	106.67	116.68	140.18	119.4	83.47	114	144	160.5	114.7	326.1	487.4	291.1	126.8	70.71	83.33	90.7	100	126	18
Os01g0678700	101	135	140	291.4	332.7	272.5	166	170	206	108.6	96.48	73.38	95.79	119.48	99.788	537.3	137.1	134.7	181.1	221.7	179.3	180.4	150.4	131.4	136.3	102.8	57.02	105	142	120	6
Os01g0742000	72.4	80.1	76.5	554.2	725.9	541.5	523	477	416	91.39	85.52	72.44	119.74	123.22	104.54	310.5	66.98	84.96	652.1	653.1	707	291.3	327.8	297.6	70.78	67.88	63.59	60.1	72.7	44.2	10
Os01g0124700	0	0.93	0	1.642	0.904	0.865	0	0	0	0	0	0.941	0	0	0	0	0	0	0	0	0	0	3.563	0	0	0	0	0	0	0	17
Os01g0775500	201	120	173	92.77	97.63	186.9	21.9	38	30.4	68.11	54.82	75.27	75.108	147.49	101.37	453.8	173.1	220.7	56.67	33.86	41.39	56.93	59.86	62.84	105.6	129.2	114	244	285	193	13
Os01g0230200	150	143	139	62.39	81.36	57.96	97.9	87.3	71.9	106	74.55	167.5	65.311	103.61	84.74	226.9	135	98.42	61.31	37.09	53.46	99.82	96.92	114.1	89.79	116.9	136	114	104	121	13
Os01g0213100	1.02	0	0	0	0	0	0	0	0	0	0	0	0	0	0	0	0	0	0	0	0	0	0	0	0	0	0	0	0	0	19
Os01g0571700	1606	1704	1762	1124	1143	1120	854	923	791	1397	1726	1388	1426	1429.1	1765.3	3917	1786	1740	939.2	1026	1022	1318	1082	975	1717	1652	1460	1712	1732	1940	13
Os01g0850100	393	308	316	252	243.2	286.3	146	207	198	191.4	168.8	158.1	218.79	200.69	215.41	453.8	349.4	280.8	199.7	245.9	213.8	288.4	330.7	251.3	163.7	155.6	131.6	214	210	197	14
Os01g0922600	73.4	93.1	83	125.6	190.7	157.4	68.9	34.8	32.5	64.66	89.9	75.27	69.665	60.674	64.149	107.5	69.05	42.48	170.9	136.3	191.4	35.49	47.03	62.11	38.03	51.86	46.05	62	42.1	46.7	10
Os01g0920400	987	1086	1044	793.1	824.4	800.2	902	868	948	1034	1004	952.1	927.42	1067.9	1003.4	1875	1100	994.6	784	809.5	865.7	1017	979.9	1057	1197	1212	1210	1089	1114	1129	13
Os01g0916100	20.4	76.4	63.6	25.45	32.54	31.14	27.4	54.4	14.5	60.35	86.62	53.63	55.515	106.41	79.989	83.59	97.9	45.59	28.8	16.13	36.21	27.36	78.39	28.89	62.33	64.11	70.17	72.2	81.3	70.8	3
Os01g0773400	2.04	4.66	3.23	3.284	1.808	1.73	3.13	2.53	0	0	1.096	0	0	1.8669	1.5839	0	0	2.072	0.929	5.644	0.862	7.394	7.839	0.722	1.056	1.886	0	0.93	1.91	0.83	17
Os01g0593000	384	424	387	350.6	363.4	327	310	300	339	319	362.9	321.8	407.11	347.24	401.53	704.5	440	427.9	267.5	308	280.2	388.2	393.4	377	403.5	439.4	311.4	467	452	449	13
Os01g0566000	4.08	0.93	2.16	0.821	0.904	0	0	0	0	0	0	0	4.3541	0	1.5839	0	0	0	0.929	0	0.862	0	0.713	0	1.056	0	2.193	0	0.96	2.5	19
Os01g0658500	998	834	912	485.2	459.2	484.4	821	741	833	1184	1043	1364	1101.6	1119.2	1163.4	1755	958.4	870.3	786.8	679.7	687.2	579.7	535.2	672.4	1043	1006	1123	896	757	804	13
Os01g0739700	24.5	14.9	19.4	9.031	17.18	26.82	11	20.2	5.53	3.449	12.06	5.645	3.2656	14.002	7.9197	0	9.275	11.4	19.51	7.257	15.52	7.394	7.839	7.945	5.282	13.2	13.16	7.4	15.3	6.66	19
Os01g0872600	1.02	0	0	0.821	1.808	4.325	5.48	3.8	8.98	0.862	2.193	0	1.0885	0.9335	0	11.94	0	1.036	7.432	1.613	5.173	3.697	2.851	4.333	4.225	0	0	0.93	0	0.83	6
Os01g0730300	899	931	855	1243	1264	1167	1361	1487	1442	698.4	797.1	804.4	756.52	790.63	738.11	1469	735.8	869.2	1083	1048	1159	1241	1225	1180	758.5	704.3	653.5	843	878	942	12
Os01g0183950	0	0	2.16	2.463	4.52	3.46	2.35	13.9	2.07	6.035	2.193	8.467	1.0885	3.7338	4.7518	0	1.031	2.072	1.858	2.419	0.862	3.697	4.276	0.722	4.225	3.771	4.386	0.93	3.83	0.83	5
Os01g0706850	58.1	27.9	32.3	4.105	6.328	8.65	9.39	6.96	6.91	43.97	21.93	70.56	18.505	17.736	6.3357	23.88	35.04	48.69	6.503	1.613	6.898	6.654	1.425	9.389	17.96	44.31	46.05	17.6	8.61	11.7	20
Os01g0722700	92.8	99.7	116	85.38	88.59	109	78.3	63.3	97.4	144.8	130.5	127	92.524	89.611	72.069	191.1	81.41	78.74	65.96	58.05	50.87	87.99	87.65	91	83.45	88.63	92.1	85.1	83.2	60.8	6
Os01g0850900	3090	2071	3067	661.7	746.7	462.8	581	1128	424	2043	1574	2063	2558	2255.2	2227	3702	3305	2357	822.1	961.1	560.4	337.9	716.2	632	1861	1915	2007	1899	2025	1925	13
Os01g0135700	743	877	450	92.77	49.72	98.62	89.2	84.1	69.1	165.5	78.94	135.5	120.83	107.35	83.948	573.2	865.6	528.4	82.68	58.05	106.9	179.7	182.4	639.2	108.8	123.5	144.7	91.6	75.5	136	14
Os01g0757400	1.02	9.31	4.31	1.642	1.808	6.055	9.39	3.8	0	3.449	0	4.704	2.177	0.9335	2.3759	0	4.122	7.252	6.503	0.806	1.724	0.739	0	0.722	2.113	4.714	8.772	3.7	1.91	0	11
Os01g0133400	602	680	671	724.9	708.7	667.8	790	865	921	638	607.4	598.4	530.11	486.33	590.01	1075	516.3	542.9	472.8	551.5	528.5	956	939.3	876.8	637	586.4	565.8	600	626	638	17
Os01g0195066	0	0	0	0.821	0	0	0	0.63	0	0.862	0	0	0	0	0	0	0	0	0	0	0	0.739	2.138	0.722	0	0	0	0	0	0	17
Os01g0777800	290	303	303	415.4	469.2	419.5	286	288	260	307.8	362.9	321.8	365.74	348.18	357.18	382.1	285.5	279.7	378.1	498.3	415.6	287.6	260.8	241.2	330.6	281.9	245.6	223	284	298	10
Os01g0836700	3.06	2.79	1.08	0	0	0	0	0	0	0	0	0	0	0	0.792	0	0	1.036	0	2.419	0	0	0	0.722	0	0	0	0	0	0.83	14
Os01g0578800	0	0	0	0	0	0	0	0	0	0	0	0	0	0	0	0	0	0	0	0	0	0.739	0	0	0	0	0	0	0	0	9
Os01g0565600	964	873	934	741.3	680.7	775.9	831	703	727	1287	1185	1236	1035.2	1103.3	1031.1	1075	905.8	838.2	929.9	906.3	904.5	584.1	704.8	660.1	1072	1081	1061	814	893	826	3
Os01g0943700	0	0	0	0	0	0	0	0	0	0	0	0	0	0	0	0	0	0	0	0	0	0	0	0	0	0	0	0	1.91	0	1
Os01g0946850	3.06	0.93	12.9	4.926	8.136	14.71	4.7	3.16	2.76	2.587	3.289	0.941	20.682	9.3345	10.296	0	5.153	1.036	13.01	2.419	2.587	5.915	3.563	1.444	5.282	5.657	10.96	4.63	6.69	4.17	4
Os01g0716550	0	0	0	0	0	0	0	0.63	0.69	0.862	0	2.822	0	0	0	0	0	0	0	0	0	0	0.713	0	0	0	0	0	0	0	20

Os01g0971500	75.4	120	107	116.6	131.1	107.3	135	130	135	101.7	94.29	36.69	82.728	70.009	92.66	47.76	73.17	73.56	76.17	61.28	43.97	110.2	124.7	96.78	77.11	59.4	41.67	80.5	61.2	85	18
Os01g0580200	15.3	8.38	10.8	48.44	34.35	34.6	68.9	61.4	68.4	12.07	9.868	10.35	10.885	5.6007	9.5036	23.88	14.43	2.072	30.66	26.61	22.42	61.37	56.3	89.56	8.451	4.714	4.386	7.4	9.56	5	18
Os01g0187200	0	0	0	0.821	0.904	0	0	0.63	0	0	0	0	0	0	0	0	0	0	0	0	0	0	0	0	0	0	0	0	0	0	2
Os01g0706500	111	87.6	85.1	95.23	116.6	111.6	42.3	81	75.3	73.29	69.07	91.26	40.275	72.809	37.222	203	62.86	65.27	96.61	94.33	119.8	67.28	96.92	82.34	64.44	84.86	92.1	70.3	45.9	50.8	6
Os01g0930900	4.08	3.73	2.16	10.67	3.616	1.73	0	1.27	2.07	4.311	0	1.882	0	1.8669	2.3759	11.94	3.092	3.108	3.716	4.031	4.311	0.739	1.425	0	3.169	0.943	0	3.7	0	1.67	6
Os01g0978100	####	####	####	7080	7475	7491	####	####	####	12832	14220	18109	13797	15033	12730	18723	15889	14491	11460	10008	11844	9713	10268	12965	14959	16203	17679	9449	####	####	20
Os01g0818300	60.2	67.1	76.5	419.5	532.4	287.2	210	252	236	71.56	66.88	35.75	90.347	66.275	60.981	95.53	36.07	44.55	229.5	337	232.8	166.4	185.3	149.5	34.86	22.63	28.51	36.1	49.7	50	10
Os01g0814800	61.2	56.8	48.5	605.1	775.6	468	387	486	308	62.94	76.75	63.98	58.78	30.804	19.007	47.76	16.49	43.51	358.6	516	410.4	240.3	219.5	221	42.25	16.03	28.51	16.7	21	35.8	12
Os01g0618400	1006	1271	1057	3035	3507	3059	2901	3272	3083	795.8	1045	1093	1542.4	1169.6	810.18	1946	886.3	1041	2884	3168	3522	2659	2143	2589	662.3	565.7	506.6	634	833	844	12
Os01g0154200	0	0	0	0	0	0	0	0	0	0	0	0	0	0	0	0	0	0	0	0	0	0	0	0	0	0	0	0	0	0.83	1
Os01g0810000	400	300	316	518.9	602	563.1	368	407	373	294.9	311.4	326.5	353.77	336.98	306.49	609	331.8	329.5	566.7	530.5	612.2	373.4	418.3	331.5	246.1	296.1	309.2	315	322	350	10
Os01g0641800	39.8	15.8	29.1	25.45	30.73	28.55	30.5	32.9	33.9	73.29	42.76	85.61	31.567	21.469	17.423	35.82	19.58	16.58	23.22	29.83	16.38	20.7	23.52	13.72	28.52	21.69	19.74	26.8	33.5	15.8	7
Os01g0943000	11.2	29.8	24.8	9.031	13.56	13.84	8.61	10.8	4.84	7.76	9.868	235.2	5.4426	109.21	7.1277	0	11.34	20.72	29.73	69.34	12.07	9.612	4.276	7.945	4.225	39.6	2.193	8.33	9.56	15	20
Os01g0714900	279	301	309	271.7	224.2	279.4	344	339	364	331.9	292.7	308.6	244.92	234.3	236.8	537.3	316.4	310.8	288	220.1	245.7	361.6	349.9	375.6	295.8	297.9	263.2	327	314	301	6
Os01g0266200	557	683	556	825.9	858.8	686.8	629	718	612	523.3	676.5	485.5	539.91	443.39	658.92	1015	484.3	590.5	493.3	738.6	551.8	867.3	702.7	561.2	582.1	614.7	489	615	711	819	6
Os01g0190300	257	118	135	88.67	65.99	115.9	53.2	68.3	37.3	303.5	231.3	669.9	189.4	244.56	213.04	155.2	146.3	163.7	135.6	73.37	108.6	40.67	39.19	80.89	273.6	369.6	412.3	170	192	253	20
Os01g0555300	357	362	406	509	487.2	513.8	382	397	419	399.2	399.1	402.7	490.92	408.85	414.99	704.5	349.4	385.4	537.9	416	475.1	353.4	404.1	428.3	351.8	367.7	361.8	342	346	390	6
Os01g0921600	879	921	879	1218	1332	1236	1086	995	1127	1009	873.8	873.1	947.01	899.85	879.08	1337	840.9	724.2	1306	1280	1261	909.4	892.9	930.3	775.4	890	881.6	737	804	826	10
Os01g0908400	797	646	659	612.4	646.3	618.5	841	670	802	686.3	703.9	792.2	622.63	785.97	635.95	1182	762.6	648.6	764.5	571.7	651.8	601.1	629.3	622.6	745.8	752.4	859.6	581	588	587	6
Os01g0908001	6.12	4.66	2.16	5.747	3.616	6.055	5.48	5.06	11.1	15.52	8.771	10.35	1.0885	6.5342	1.5839	23.88	9.275	3.108	7.432	4.031	6.036	1.479	4.276	7.945	13.73	15.09	8.772	7.4	1.91	3.33	6
Os01g0728000	65.2	59.6	66.8	69.78	50.62	65.74	80.6	79.1	71.2	93.12	92.1	55.51	46.806	60.674	67.317	71.65	43.28	63.2	56.67	69.34	73.29	73.2	63.42	51.28	52.82	52.8	43.86	50	51.6	42.5	7
Os01g0110400	453	459	460	411.3	421.2	436	616	654	827	437.1	447.3	412.1	581.27	625.41	616.15	823.9	481.3	471.4	472.8	440.2	425.1	672.1	724.7	645	457.4	475.2	644.7	525	515	474	18
Os01g0620301	520	654	524	908	962.7	990.5	58.7	94.9	56	388	435.3	380.1	390.78	623.55	542.5	1134	683.2	721.1	548.1	839.3	655.3	249.2	323.5	192.8	824	614.7	510.9	1018	602	937	8
Os01g0511600	1173	1013	1064	1602	1666	1384	3543	3284	3414	873.4	929.7	1492	888.23	1011.9	806.22	2352	1442	1286	2543	1784	2364	2084	1722	3139	994	1173	1379	928	1138	986	18
Os01g0147800	7.14	10.2	6.47	9.852	8.136	6.92	3.91	8.22	8.29	4.311	5.482	0.941	14.151	9.3345	4.7518	0	2.061	6.216	8.361	4.031	12.93	5.915	2.138	3.611	6.338	0	6.579	5.55	7.65	6.66	4
Os01g0654500	633	750	714	1827	2060	1683	1112	947	1311	931.2	836.5	757.4	624.81	844.77	756.33	585.1	648.2	620.6	1853	2058	1917	715.7	868.7	834.2	743.7	692	725.9	699	697	632	10
Os01g0917500	209	194	268	123.1	119.3	94.29	258	231	252	209.5	182	210.7	198.11	224.03	171.86	573.2	208.2	182.3	106.8	112.9	105.2	201.9	193.8	218.1	164.8	191.4	203.9	197	239	211	6
Os01g0102600	4080	4105	4583	3812	4330	3805	3605	3745	3717	4111	4971	5238	5735.4	5460.7	5095.5	5959	4880	4747	4520	4704	5116	2830	2397	3454	4776	4437	3945	4136	4182	4406	13
Os01g0584032	5.1	10.2	5.39	0.821	5.424	5.19	0	0	1.38	2.587	0	0	2.177	7.4676	1.5839	11.94	12.37	6.216	2.787	3.225	3.449	2.218	1.425	4.333	6.338	3.771	8.772	16.7	13.4	16.7	1
Os01g0263300	38.7	17.7	28	84.56	85.88	70.93	63.4	43.6	37.3	33.63	21.93	36.69	18.505	22.403	23.759	59.7	22.67	43.51	88.25	97.56	82.77	54.71	54.87	57.78	26.41	39.6	21.93	37	34.4	24.2	10
Os01g0306100	1.02	0	0	0	0	1.73	0	0	0	0	0	0	0	0	0	0	0	0	0	0	0	0	0	0	0	0	0	0	0.96	0	19
Os01g0833800	1139	1343	1249	740.5	764.8	727.5	1353	1281	1531	1270	1347	1171	1146.2	1124.8	1274.3	2328	1234	1217	699.5	716.8	690.6	1552	1396	1436	1537	1570	1265	1289	1197	1408	13
Os01g0547500	70.3	78.2	65.7	89.49	100.3	93.43	60.3	67	56.7	92.25	125	91.26	92.524	80.277	82.364	179.1	76.26	66.31	96.61	84.66	112.1	70.98	59.86	55.61	86.62	65.06	67.98	89.7	77.5	93.3	6
Os01g0976000	697	711	682	518.9	526.1	467.1	550	534	572	673.4	703.9	707.5	689.03	607.68	659.71	1493	683.2	681.7	642.8	614.4	635.5	516.1	525.9	578.5	745.8	785.4	820.2	708	759	708	13
Os01g0615300	30.6	27	22.6	41.87	37.97	36.33	51.7	29.1	35.2	27.59	32.89	30.11	32.656	32.671	11.088	71.65	19.58	29.01	35.3	35.48	40.52	31.79	28.51	38.28	22.18	19.8	19.74	9.25	14.3	25.8	6
Os01g0364000	154	154	172	132.2	89.49	122	122	142	131	144.8	151.3	154.3	170.9	175.49	199.58	417.9	265.9	223.8	99.4	128.2	139.7	204.8	163.2	156.7	170.1	259.3	223.7	228	242	273	13
Os01g0729600	0	0	0	0	0	0	0	0	0	0	0	0	0	0	0	0	0	0	0	0	0	0	2.851	0	0	0	0	0	0	0	17
Os01g0103100	13.3	10.2	11.9	3.284	2.712	4.325	5.48	6.33	8.29	11.21	6.578	13.17	6.5311	14.002	12.671	11.94	8.244	8.288	4.645	9.675	6.036	5.176	17.82	9.389	16.9	12.26	2.193	11.1	6.69	6.66	3
Os01g0559825	0	0	0	0	0	0	0	0	0	0	0	0	0	0.9335	0	0	0	0	0	0.806	0	0	0	0	0	0	0	0	0	0	16
Os01g0700400	0	0	0	0	0	0	0	0	0	0	0	0	0	0.9335	0	0	0	0	0	0	0	0	0	0	0	0	0	0	0	0	4
Os01g0832200	723	633	650	604.2	563.2	606.4	912	941	922	782	722.5	810	759.79	631.95	740.49	1087	666.8	625.8	668.8	707.9	676.8	771.2	821.7	839.2	797.6	672.2	804.8	679	640	575	5
Os01g0808100	304	374	351	307.9	322.7	251.7	918	1400	962	310.4	319.1	284.1	416.9	340.71	376.98	609	347.3	371.9	182.1	185.4	165.5	818.5	672.7	720.1	258.8	212.1	219.3	290	301	351	18
Os01g0668300	316	356	336	391.6	394.1	358.1	272	311	258	290.6	343.2	220.2	357.03	313.64	380.14	429.9	322.6	382.3	292.6	340.3	334.5	318.7	315	355.3	331.7	316.8	276.3	392	360	393	8
Os01g0243700	15.3	7.45	11.9	16.42	9.04	8.65	2.35	6.33	6.22	6.035	8.771	1.882	19.593	2.8004	7.9197	11.94	5.153	7.252	1.858	6.45	0	11.09	10.69	0.722	7.395	5.657	6.579	13.9	8.61	10	8
Os01g0192550	4.08	11.2	15.1	6.568	4.52	12.11	18.8	3.8	8.98	13.79	9.868	19.76	7.6196	4.6673	6.3357	0	15.46	12.43	5.574	2.419	15.52	24.4	13.54	15.17	14.79	15.09	13.16	4.63	15.3	2.5	9
Os01g0172800	211	149	175	87.84	74.13	82.18	72.8	56.9	51.8	211.2	206.1	235.2	113.21	145.62	103.75	191.1	180.3	193.7	109.6	85.47	111.2	77.64	89.79	104.7	168	235.7	256.6	197	160	151	3
Os01g0761500	3.06	5.59	4.31	3.284	4.52	0.865	0.78	2.53	0	3.449	3.289	4.704	0	8.4011	0	0	1.031	1.036	2.787	4.031	2.587	0	0	0	0	3.771	0	0.93	0	0	7
Os01g0179400	667	748	720	811.9	881.4	756.9	563	695	602	528.5	544.9	411.1	825.1	627.28	825.23	1385	633.8	625.8	573.2	650.7	616.5	768.2	766.8	696.2	670.8	545	460.5	742	747	816	6
Os01g0128300	84.6	62.4	70	70.6	48.81	73.53	91.6	74	77.4	117.3	94.29	94.08	75.108	69.075	61.773	214.9	96.87	90.14	113.3	83.05	151.8	39.19	44.18	49.83	71.83	91.46	109.6	77.7	69.8	61.7	6
Os01g0823700	116	240	159	399	374.2	137.5	179	247	134	106.9	105.3	64.92	180.69	70.009	121.96	346.3	79.35	168.9	201.6	204	80.19	327.5	168.9	142.3	87.68	36.77	46.05	126	162	209	2
Os01g0117000	2.04	1.86	1.08	1.642	2.712	1.73	3.91	4.43	3.46	6.897	0	3.763	2.177	8.4011	5.5438	0	4.122	7.252	2.787	2.419	0.862	1.479	2.851	2.889	3.169	2.829	6.579	0.93	2.87	5	4
Os01g0100900	1507	1598	1470	1627	1685	1534	1235	1372	1247	1296	1225	1298	1046.1	1084.7	1248.1	1588	1311	1239	1443	1187	1210	1429	1799	1546	1399	1405	1366	1317	1183	1390	17
Os01g0947000	0	0	0	0	0	0	0	0	0	0	0	0	0	0	0	0	0	0	0	0	0	0.739	0	2.167	0	0	0	0	0	0	9
Os01g0116400	36.7	53.1	36.6	59.11	52.43	53.63	258	208	253	87.94	120.6	99.73	76.196	93.345	106.12	11.94	35.04	29.01	70.6	74.18	68.98	108	106.9	129.3	86.62	82.03	92.1	62	63.1	53.3	18
Os01g0898500	32.6	35.4	24.8	39.41	30.73	29.41	18	15.8	11.1	17.24	27.41	31.05	23.947	26.137	22.175	59.7	32.98	19.68	20.44	19.35	28.45	28.1	29.22	19.5	31.69	36.77	28.51	38.9	37.3	25.8	13
Os01g0924300	537	489	516	555.8	576.7	576.1	458	510	486	638	536.1	742.3	543.17	605.81	554.38	919.5	551.3	533.6	629.8	580.5	564.8	540.5	449.7	478.1	550.4	655.3	635.9	549	562	517	6
Os01g0863300	360	358	317	37.76	28.93	51.9	18.8	24	23.5	75.87	61.4	91.26	66.4	48.539	68.901	298.5	299.9	260	80.82	78.21	156.9	17.01	39.91	35.39	102.5	58.46	127.2	64.8	51.6	63.3	14
Os01g0533900	6.12	5.59	3.23	9.852	5.424	6.055	4.7	2.53	3.46	0.862	0	0.941	2.177	0	0.792	0	0	4.144	3.716	9.675	1.724	8.133	3.563	7.945	1.056	1.886	2.193	1.85	6.69	2.5	2
Os01g0303300	1.02	0	0	0.821	0.904	0.865	0	0	0.69	1.724	0	0	0	0	0	0	1.031	0	1.858	0	0	1.479	0	0	0	0	0	0	0	0	2
Os01g0847300	58.1	58.7	55	22.99	11.75	19.9	10.2	8.22	15.9	24.14	35.08	37.63	42.452	28.937	30.887	131.4	65.95	46.62	21.37	16.93	16.38	26.62	25.65	17.33	41.2	26.4	52.63	75.9	40.2	32.5	13
Os01g0719800	1636	1719	1934	443.3	422.2	413.5	944	1429	1092	1840	2169	1834	2256.5	2166.5	2387.8	2102	1863	1870	405	424.1	406.1	976	967.8	1013	1864	1999	1445	1941	2060	2093	3
Os01g0715000	572	543	536	626.4	689.7	596	992	1037	930	700.1	767.5	803.5	904.56	789.7	740.49	1433	732.7	729.4	767.3	847.4	838.1	723.9	697	756.9	741.6	775	725.9	729	752	741	6
Os01g0679300	1.02	0	0	0	0	0	0	0	0	0	0	0	0	0	0	0	0	0	1.858	0	0	0	0	0	0	0	2.193	0	0	0	11
Os01g0965000	57.1	64.3	56	49.26	54.24	81.31	37.6	52.5	84.3	56.9	77.84	65.86	52.249	63.475	50.686	35.82	57.71	51.8	29.73	54.02	49.15	80.59	70.55	41.89	70.78	75.43	78.95	65.7	52.6	66.6	1
Os01g0645900	30.6	21.4	21.6	1211	1285	1259	103	125	98.1	35.35	29.6	53.63	23.947	38.272	35.638	35.82	26.79	32.12	1010	1114	1049	99.82	122.6	70.06	17.96	25.46	13.16	35.2	29.6	21.7	10
Os01g0969200	195	244	212	378.5	338.1	304.5	189	226	167	213.8	270.8	242.7	269.95	243.63	242.34	286.6	239.1	242.4	365.1	466	352.6	168.6	179.6	176.2	244	219.7	254.4	265	287	228	16
Os01g0865400	38.7	29.8	25.9	74.71	94.92	33.74	54.8	112	100	55.18	61.4	24.46	50.072	28.004	50.686	35.82	15.46	18.65	32.51	45.15	44.84	50.28	47.75	50.56	26.41	16.97	13.16	17.6	23	47.5	18
Os01g0235850	0	0	0	0	0	0	0	1.27	0	0	0	0	0	0	0	0	0	0	0	0	0	0	0	0	0	0	0	0	0	0	18
Os01g0795700	2.04	3.73	0	0.821	3.616	3.46	10.2	5.06	16.6	1.724	3.289	1.882	1.0885	0	1.5839	0	0	0	0	2.419	0	4.436	8.552	13.72	1.056	0.943	0	0	2.87	0.83	18
Os01g0695600	252	227	277	169.9	151.9	141	145	133	142	199.2	206.1	179.7	279.75	229.63	275.6	501.5	221.6	246.6	126.3	125.8	143.1	136.8	118.3	147.3	247.2	212.1	173.2	259	258	274	13
Os01g0234900	469	480	541	215.9	216	151.4	1077	746	803	618.2	495.6	526.9	512.69	455.52	552	417.9	340.1	436.2	198.8	237	213	632.9	598.6	610.3	465.9	399.8	403.5	354	331	337	5
Os01g0882300	494	493	469	286.5	275.7	278.5	468	405	405	378.5	441.8	352.8	445.2	361.25	495.77	776.2	444.2	420.6	301.9	293.5	301.8	397.1	380.5	382.1	405.6	429.9	453.9	443	416	427	13
Os01g0651300	0	0	0	0	0	0	2.35	0	0	0.862	0	0	0	0.9335	0	0	0	0	0	0	0	0	0	0	0	0	0	0	0	0	5
Os01g0370750	0	0	1.08	0	0	0	0	0	0	0	0	0	0	0.9335	0	0	0	0	0	0	0	0	0	0	2.113	0	0	0	0	0	4
Os01g0770400	878	794	834	473.7	491.8	471.5	436	563	548	768.2	844.2	795	970.96	829.84	941.65	1373	1085	931.4	529.5	674.1	632	434	411.9	466.6	1006	1033	877.2	942	953	956	13
Os01g0198900	555	735	724	186.4	111.2	164.4	186	173	294	1692	1649	1391	724.95	794.37	1057.3	1528	824.4	863	173.7	202.4	204.3	364.5	479.6	473.1	2042	2128	2017	1353	1040	1097	3
Os01g0732100	175	150	210	159.3	173.6	176.5	182	138	159	220.7	183.1	218.3	168.72	175.49	167.9	274.6	198.9	163.7	173.7	160.5	181.9	136	96.21	127.1	189.1	183.9	182	177	161	168	13
Os01g0886350	135	116	144	316.9	339.9	315.7	209	252	207	58.63	95.39	105.4	105.59	62.541	69.693	95.53	93.78	127.4	355.8	383.8	421.6	238.1	196.7	214.5	75	70.71	118.4	74.9	99.5	100	10
Os01g0841050	9.18	5.59	1.08	1.642	4.52	3.46	2.35	4.43	0.69	5.173	0	0	2.177	0	5.5438	11.94	4.122	3.108	0.929	8.063	12.07	3.697	5.701	3.611	3.169	0.943	0	8.33	0.96	2.5	6
Os01g0351800	24.5	25.1	33.4	25.45	26.22	18.17	23.5	12	31.1	21.55	13.16	32.93	4.3541	17.736	19.799	23.88	4.122	11.4	32.51	19.35	21.56	29.58	44.18	46.95	5.282	6.6	17.54	7.4	9.56	11.7	9
Os01g0902700	7.14	9.31	4.31	0	1.808	1.73	168	58.8	114	31.04	41.66	5.645	6.5311	11.201	13.463	11.94	6.183	1.036	0	0	2.587	24.4	34.21	39	9.507	7.543	15.35	7.4	8.61	5.83	18
Os01g0853600	170	153	212	128.9	155.5	122	65.8	89.8	49.8	62.08	59.21	72.44	68.577	98.946	83.156	191.1	183.4	161.6	71.53	81.43	51.73	107.2	73.4	49.11	76.06	66	63.59	161	166	152	8
Os01g0660000	2.04	5.59	3.23	0	0	0	0	1.9	2.07	2.587	1.096	2.822	2.177	1.8669	0.792	0	2.061	6.216	0	0	0	0	0.713	0	0	0.943	0	0.93	0.96	0.83	14
Os01g0207500	0	0	0	0	0	0	0	0	0	0	0	0	0	0	0	0	0	0	0	0	0	0	0	1.444	0	0	0	0	0	0	9
Os01g0511700	268	274	324	305.4	312.8	259.5	305	390	283	256.1	285.1	243.7	291.72	325.77	290.65	680.6	322.6	347.1	224.8	279	267.3	322.4	300.7	273	356	370.5	357.4	358	379	418	6
Os01g0710100	0	0	0	0	0	0	0	0	0	0	0	0	0	0	0	0	0	0	0	0	0	0	0	0	0	0	0	0.93	0	0	1
Os01g0602400	12.2	2.79	11.9	1.642	2.712	6.055	21.9	13.3	13.1	13.79	5.482	15.99	7.6196	8.4011	8.7116	0	16.49	21.76	10.22	14.51	10.35	13.31	22.8	20.95	27.47	29.23	39.47	11.1	12.4	8.33	11
Os01g0278100	0	0	0	0	0	0	0	0	0	0	0	0	0	0	0	0	0	0	0	0	0	0.739	0	0	0	0	0	0	0	0	9
Os01g0276700	426	328	338	357.1	306.4	406.6	90.8	110	110	251.8	205	293.5	209	248.3	258.18	286.6	367.9	347.1	251.7	274.1	306.1	161.9	233	181.3	275.7	340.4	353.1	335	261	288	19
Os01g0388101	0	0.93	0	0	0	0	0	0	1.38	0	0	0	0	0	0	0	0	0	0	0	0	0.739	0.713	3.611	0	0	0	0	0	0	9
Os01g0266500	688	534	532	193.8	168.1	232.7	326	230	254	439.7	365.1	545.7	604.13	667.42	521.91	752.3	590.5	536.7	389.2	341.9	355.2	270.6	282.2	284.6	410.9	415.8	543.8	536	467	472	13
Os01g0214400	1110	993	1127	955.6	1023	825.3	1313	1341	1182	1254	1376	1259	1529.4	1285.4	1274.3	1899	1141	1093	1105	1176	1226	709.8	772.5	870.3	1245	1171	1013	1087	1048	1186	13
Os01g0908200	1110	956	1019	1007	978.1	979.2	238	164	225	810.5	922.1	934.2	989.46	772.9	838.69	1672	1314	920	1362	1484	1399	215.2	284.3	278.1	780.7	866.5	778.5	1008	1217	1181	16
Os01g0245600	0	0	0	0.821	0	0	0	0	0	0	0	0	0	0	0	0	0	0	0	0	0	0.739	0	0	0	0	0	0	0	0	2
Os01g0852900	62.2	43.8	31.2	125.6	142.8	127.2	34.4	54.4	33.2	30.18	27.41	25.4	28.302	21.469	26.135	47.76	25.76	35.23	91.04	88.69	86.22	68.76	59.86	47.67	20.07	44.31	28.51	34.2	37.3	50	10
Os01g0229500	134	101	156	78.81	84.07	90.83	116	109	138	194	187.5	191.9	183.96	167.09	158.39	334.3	128.8	106.7	147.7	117.7	112.1	98.34	91.93	111.9	166.9	157.5	232.4	139	118	123	13
Os01g0878101	0	1.86	0	4.926	3.616	1.73	11	7.59	11.7	5.173	2.193	1.882	2.177	1.8669	1.5839	11.94	1.031	3.108	0.929	12.09	7.76	5.176	4.276	8.667	1.056	1.886	0	0.93	0	5	18
Os01g0187350	0	0	0	0	0	0	1.57	0	0	0	0	0	0	0	0	0	0	0	1.858	0	0	0	0	0	0	0	0	0	0	0	15
Os01g0621300	2070	1379	1730	514.8	472.8	640.1	651	666	746	1038	1066	1096	1255.1	1194.8	1291.7	2794	1791	1836	560.2	471.7	578.5	475.4	590.1	767	1276	1179	1094	1164	1179	1158	13
Os01g0860450	10.2	10.2	5.39	5.747	1.808	5.19	14.9	7.59	13.8	6.897	8.771	7.527	4.3541	4.6673	5.5438	0	1.031	6.216	5.574	4.838	6.898	11.83	10.69	13	1.056	5.657	6.579	5.55	10.5	0	18
Os01g0234499	0	0	0	0	0	0	0	0	0	0	0	0	0	0	0	0	0	0	0	0	0	0	0	0	0	0	0	0	0	0.83	1
Os01g0101600	2506	2657	2484	2282	2352	2124	1796	2043	1791	2351	2719	2229	1973.5	2141.3	2590.5	3881	2217	2766	1778	2120	1751	2565	2130	1910	2781	2858	2307	2878	2946	3054	13
Os01g0201100	41.8	34.5	37.7	16.42	16.27	8.65	43.8	48.7	20.7	14.66	12.06	0.941	11.974	17.736	19.799	23.88	15.46	15.54	11.15	9.675	5.173	23.66	51.31	17.33	7.395	3.771	10.96	14.8	7.65	9.16	17
Os01g0711200	67.3	67.1	61.4	8.21	2.712	15.57	0	2.53	0.69	27.59	44.95	10.35	50.072	50.406	41.182	59.7	68.02	49.73	12.08	11.29	8.622	0.739	0	0	22.18	47.14	28.51	85.1	71.7	98.3	3
Os01g0100800	572	547	595	498.3	540.6	509.5	583	526	543	633.7	565.7	714.1	620.46	638.48	621.69	1218	680.2	618.5	554.6	599.1	501.8	550.8	475.3	493.3	672.9	746.7	627.2	661	705	640	13
Os01g0224200	6.12	0	9.7	3.284	2.712	3.46	2.35	5.06	3.46	6.035	9.868	6.586	8.7082	10.268	8.7116	0	3.092	1.036	1.858	3.225	1.724	0.739	0	0	3.169	1.886	0	8.33	0.96	6.66	4
Os01g0698500	2.04	1.86	1.08	0	0	2.595	3.13	1.27	1.38	0	1.096	4.704	0	3.7338	0	0	12.37	1.036	0	4.031	0.862	1.479	0.713	3.611	0	5.657	4.386	0	2.87	0	20
Os01g0728200	13.3	27	11.9	12.31	6.328	18.17	43.1	36.7	60.1	18.97	15.35	6.586	11.974	18.669	13.463	11.94	14.43	13.47	23.22	20.96	18.11	54.71	54.16	64.28	13.73	3.771	28.51	6.48	23	5.83	18
Os01g0958500	149	115	128	90.31	94.92	121.1	59.5	53.1	65	102.6	111.8	164.6	79.462	117.61	101.37	262.7	155.6	100.5	109.6	79.02	85.36	69.5	101.2	78.72	101.4	113.1	136	111	93.7	91.6	13
Os01g0642900	97.9	99.7	106	313.6	348.9	301.9	173	173	207	164.7	107.4	116.7	107.76	131.62	72.861	179.1	93.78	109.8	358.6	343.5	344	160.4	176	173.3	96.13	82.97	87.72	78.6	88.9	86.6	10
Os01g0171000	231	231	208	142	175.4	196.4	385	306	375	151.7	195.2	189.1	128.45	176.42	125.92	382.1	188.6	158.5	156.1	107.2	202.6	199.6	121.9	360.4	114.1	157.5	250	121	179	106	18
Os01g0764500	1134	1060	1136	765.1	747.6	885.8	1279	1478	1412	1254	1534	1395	1237.6	1319	1359	2663	1632	1417	1141	1231	1399	1119	1007	1119	1526	1597	1421	1415	1393	1509	13
Os01g0839700	21.4	15.8	11.9	6.568	8.136	6.055	29	21.5	25.6	20.69	25.22	27.28	29.39	26.137	22.967	23.88	14.43	22.79	13.01	9.675	19.83	14.79	21.38	21.67	28.52	17.91	43.86	13.9	15.3	13.3	5
Os01g0102400	23.4	33.5	38.8	22.99	37.97	25.09	38.4	31	19.3	37.07	25.22	32.93	47.895	38.272	38.014	11.94	29.89	30.05	35.3	47.57	18.11	25.14	17.1	28.89	34.86	17.91	17.54	31.5	33.5	33.3	4
Os01g0195400	24.5	5.59	15.1	3.284	5.424	13.84	3.13	4.43	1.38	21.55	10.96	11.29	11.974	15.869	3.9598	35.82	27.82	10.36	2.787	4.838	2.587	1.479	4.276	13	1.056	38.66	85.52	10.2	29.6	7.5	11
Os01g0721200	3.06	0	0	3.284	1.808	0	0	6.96	0.69	3.449	1.096	1.882	0	0	7.1277	0	1.031	0	0.929	0.806	0	0	3.563	0	2.113	0	2.193	0.93	0	0	7
Os01g0807532	61.2	70.8	43.1	52.54	84.07	66.61	56.4	64.5	38.7	70.7	57.01	66.8	47.895	66.275	48.31	131.4	89.66	96.35	70.6	54.83	70.7	87.25	97.63	78	80.28	69.77	52.63	90.7	80.3	78.3	6

Os01g0950833	12.2	14	7.54	6.568	3.616	7.785	15.7	9.49	14.5	10.35	9.868	11.29	14.151	14.002	17.423	0	21.64	10.36	18.58	13.71	17.24	10.35	9.264	8.667	16.9	15.09	21.93	9.25	16.3	6.66	11
Os01g0159200	0	0.93	0	0	3.616	1.73	0.78	0	0	3.449	0	3.763	0	0	0	0	0	0	0	0	0	0	0	0	0	0	0	0	0	0	7
Os01g0271400	1407	1497	1548	2320	2266	2068	3368	3357	3368	1883	2021	2445	2299	1872.5	1510.3	2842	1476	1562	3007	2966	2594	2025	1861	2612	1632	1685	1546	1306	1510	1475	12
Os01g0730500	6729	5907	7130	7145	7416	7576	1148	1307	1184	10169	10746	12061	11629	11397	10998	14043	9280	8121	9988	9606	10987	643.3	763.9	890.5	11354	11072	9107	7635	8134	8759	3
Os01g0859200	745	985	914	997.5	1047	720.6	1148	1378	1349	1217	1438	1188	1472.8	1112.7	1466.7	1516	631.7	747	888.1	780.5	611.3	1241	1136	1041	1251	876.8	723.7	870	960	1204	5
Os01g0328600	1227	1144	1274	574.7	546	630.6	780	898	888	1391	1514	1410	1658.9	1722.2	1879.3	2233	1634	1339	682.8	746.6	735.5	891.7	964.2	881.1	1779	1824	1614	1648	1478	1767	3
Os01g0585300	3551	3205	3395	1232	1091	966.3	2414	2448	1976	4873	5355	7563	5052.9	4391	4604.5	4514	3897	3875	2016	1556	2218	1011	940.7	1580	4401	5476	4221	2691	3157	3416	3
Os01g0220300	318	324	309	376	485.4	420.4	407	383	432	328.5	279.6	266.3	411.46	403.25	375.39	716.5	298.9	301.5	509.1	379	374.2	283.2	329.9	314.9	285.2	242.3	254.4	313	311	325	6
Os01g0127400	252	272	300	550.1	631.9	480.1	525	579	494	321.6	446.2	562.6	407.11	275.37	259.76	226.9	161.8	232.1	455.2	614.4	507	343.8	357	443.5	329.6	287.6	206.1	159	174	162	12
Os01g0697400	4.08	0.93	8.62	2.463	0.904	0	3.13	2.53	4.15	5.173	0	0.941	0	2.8004	2.3759	0	2.061	5.18	3.716	2.419	0.862	2.218	0.713	7.222	0	0.943	0	0.93	0	4.17	9
Os01g0595100	702	749	763	769.3	751.2	733.6	418	431	457	774.2	688.5	697.1	773.94	818.64	828.4	1445	757.4	756.3	682.8	699	686.3	490.2	503.8	459.3	799.7	765.6	758.7	810	783	810	13
Os01g0838900	37.7	52.2	37.7	57.47	54.24	64.88	88.5	98.7	94	41.38	40.57	42.34	37.01	51.34	49.894	119.4	40.19	42.48	44.59	74.98	56.91	84.29	79.82	81.61	45.42	34.89	43.86	46.3	52.6	55	18
Os01g0691400	7.14	16.8	21.6	9.852	17.18	6.92	20.4	26.6	15.9	17.24	15.35	13.17	7.6196	12.135	18.215	47.76	6.183	16.58	22.29	12.9	15.52	6.654	12.11	10.83	11.62	5.657	10.96	6.48	13.4	10.8	6
Os01g0669100	23.4	39.1	28	15.6	18.08	25.09	61.8	41.1	58	58.63	55.92	20.7	59.869	30.804	57.022	35.82	14.43	13.47	13.93	14.51	4.311	51.76	34.92	48.39	42.25	20.74	4.386	24.1	44	30	5
Os01g0677900	89.7	103	119	96.05	99.44	83.91	251	222	195	205.2	137	133.6	92.524	93.345	112.46	83.59	54.62	66.31	71.53	58.05	68.12	247	324.2	307	80.28	58.46	74.56	83.3	73.6	65.8	18
Os01g0558200	735	710	716	637.1	575.8	605.5	652	676	657	715.6	745.5	640.7	837.07	701.96	913.93	1218	809	740.8	623.3	645.8	632	702.4	708.4	758.4	859.9	842.9	719.3	907	776	951	13
Os01g0681400	488	517	474	488.5	467.4	504.3	667	627	655	477.7	471.4	398.9	411.46	477.93	516.36	812	542.1	491.1	463.5	437	463	791.1	749.7	708.5	463.7	463.9	403.5	489	421	527	17
Os01g0174700	40.8	44.7	36.6	50.08	51.53	42.39	77.5	106	76.7	50.01	52.63	75.27	29.39	56.941	53.854	35.82	54.62	51.8	61.31	65.31	64.67	64.33	87.65	71.5	40.14	60.34	52.63	50	61.2	46.7	18
Os01g0833100	446	388	416	415.4	385.1	359	521	659	480	438.9	385.9	318.9	438.67	417.25	441.92	441.8	373.1	380.2	404.1	382.2	410.4	411.8	446.8	481.7	477.5	347	399.1	422	453	450	18
Os01g0108400	0	1.86	0	1.642	0	0	1.57	1.9	4.84	0.862	6.578	0	0	1.8669	0	11.94	0	1.036	0	0	0	0	0.713	0	3.169	0	0	0	0	0	6
Os01g0630300	0	0	0	0	0	0	0	0	0	0	0	0	0	0	0	0	0	0	0	0	0	0	0	0.722	0	0	0	0.93	0	0	9
Os01g0872300	0	0	0	0.821	3.616	0	0	0	0	0.862	0	0	0	0	0	0	0	0	0	0	0	0	0	0	0	0	0	0	0	0	2
Os01g0805500	375	232	370	86.2	113	99.48	86.9	110	90.5	243.1	286.2	368.8	473.51	449.92	368.26	644.8	546.2	392.7	202.5	165.3	195.7	59.89	59.15	71.5	340.1	440.3	335.5	334	321	345	13
Os01g0116200	0	0	0	0	0	0	0	0	0	0	0	0	0	0	0	0	0	0	0	0	0	0	0	0	0	0	2.193	0.93	0.96	0	11
Os01g0629600	6.12	24.2	6.47	4.926	11.75	12.11	6.26	15.2	22.1	14.66	7.675	0	1.0885	4.6673	11.879	11.94	2.061	15.54	9.29	5.644	12.93	45.1	36.34	28.17	1.056	2.829	4.386	14.8	12.4	10	17
Os01g0546500	52	49.4	38.8	170.8	186.2	161.8	63.4	46.2	53.9	63.8	42.76	66.8	65.311	57.874	42.766	107.5	37.1	37.3	188.6	166.1	211.2	48.8	45.61	37.56	52.82	29.23	61.4	39.8	30.6	35.8	10
Os01g0849950	9.18	7.45	2.16	12.31	20.79	11.25	3.13	2.53	2.07	0.862	1.096	1.882	0	0.9335	5.5438	0	12.37	4.144	9.29	3.225	2.587	2.958	3.563	4.333	2.113	0.943	0	4.63	4.78	4.17	2
Os01g0588000	1.02	0.93	0	0	0	0.865	0	0	1.38	0	0	0	0	0.9335	0	0	0	0	0	0	0	0	0	0	0	0	0	0	0	0	19
Os01g0612900	1082	803	827	258.6	188	301.9	158	197	169	596.6	506.5	686.8	476.77	624.48	586.05	1552	976.9	1072	298.2	261.2	302.6	471.7	568	588.6	744.7	1035	971.5	881	704	801	13
Os01g0773100	303	279	274	399	405.9	494.8	337	375	301	288.8	255.5	221.1	237.3	229.63	248.68	406	208.2	245.5	324.2	343.5	349.2	369	453.2	351.7	195.4	195.2	252.2	242	258	247	12
Os01g0974100	5.1	6.52	6.47	4.105	6.328	6.055	7.83	3.16	8.29	12.93	9.868	5.645	17.416	2.8004	12.671	0	3.092	6.216	11.15	12.09	16.38	7.394	7.839	11.56	13.73	7.543	15.35	11.1	10.5	17.5	4
Os01g0872400	0	0	0	0	0	0	0.78	0	0	0	0	0	0	0	0	0	0	1.036	0	0	0	0	0	0	0	0.943	0	0	0	0	5
Os01g0883900	64.2	66.1	52.8	136.3	127.5	169.5	115	112	127	41.38	36.18	27.28	43.541	60.674	26.927	59.7	41.22	60.09	75.25	70.95	104.3	94.64	182.4	114.8	40.14	21.69	63.59	53.7	52.6	39.2	12
Os01g0104000	389	427	364	399	441.1	429.9	406	403	405	343.2	403.5	369.7	448.47	418.19	428.45	680.6	434.9	381.3	332.6	374.1	393.2	502	488.9	457.9	475.4	431.8	394.7	562	461	513	6
Os01g0732801	169	138	129	36.94	33.45	24.22	86.1	76.5	93.3	51.73	38.37	35.75	63.134	42.005	33.263	119.4	105.1	76.67	23.22	22.58	43.97	81.33	80.53	149.5	38.03	30.17	32.89	44.4	46.9	50	14
Os01g0183400	0	0	0	0	0.904	0	0	0	0	0	0	0	0	0	0	0	0	0	0	0	0	0	0	0	0	0	0	0	0	0	2
Os01g0693900	610	537	693	589.5	609.3	672.1	453	446	558	857	788.3	843.9	936.13	897.05	825.23	2293	830.6	665.1	793.3	733.7	838.9	522.7	444	533	772.2	850.4	721.5	624	704	699	6
Os01g0572100	203	211	177	220.8	226.9	199	132	149	133	231.1	239	208.9	263.42	210.03	250.26	214.9	190.6	192.7	196	235.4	214.7	159	148.2	123.5	229.2	231.9	166.7	179	232	247	4
Os01g0357800	1638	1769	1800	1863	1996	1729	1688	1748	1735	1337	1697	1290	1435.8	1270.4	1488.1	3152	1514	1827	1435	1853	1530	2537	1980	1920	1520	1378	1261	1694	1768	1925	6
Os01g0753500	391	477	455	355.5	388.7	288.9	353	364	352	678.5	586.6	585.2	462.62	549.8	592.39	764.2	495.7	469.3	314.9	291.1	362.1	365.3	409.8	445.6	742.6	514.8	570.2	677	471	541	13
Os01g0367100	10.2	69.9	7.54	7.389	8.136	11.25	4.7	6.33	16.6	5.173	13.16	3.763	5.4426	3.7338	18.215	23.88	9.275	7.252	9.29	8.869	0.862	5.915	7.839	6.5	7.395	33	2.193	26.8	9.56	9.16	14
Os01g0276300	0	0.93	1.08	4.926	3.616	0.865	3.91	5.06	4.84	1.724	0	1.882	0	1.8669	0.792	0	0	0	13.01	4.031	6.036	1.479	2.851	1.444	2.113	4.714	4.386	0	4.78	0	15
Os01g0797900	471	362	488	138.7	124.7	122	82.2	87.9	105	322.5	294.9	339.6	329.82	350.04	352.42	907.5	519.4	497.3	175.6	156.4	175	130.1	111.9	106.9	329.6	390.3	484.6	421	431	415	13
Os01g0924200	38.7	51.2	63.6	65.68	75.03	36.33	11.7	10.8	18.7	103.5	89.9	66.8	60.957	91.478	83.156	71.65	47.4	90.14	83.61	64.5	33.63	31.05	42.76	31.78	62.33	86.74	186.4	49	142	100	11
Os01g0230800	1.02	0	4.31	1.642	0	3.46	5.48	0	3.46	0	2.193	0	0	2.8004	0.792	11.94	0	3.108	1.858	1.613	1.724	6.654	3.563	11.56	0	0	0	0.93	0	4.17	6
Os01g0590100	2.04	0.93	0	0.821	0	0.865	0	3.8	0	1.724	1.096	0.941	0	0.9335	3.9598	0	3.092	3.108	0	0.806	0	0.739	1.425	2.889	0	0	0	0	0.96	0	19
Os01g0154600	166	218	177	105.9	108.5	101.2	25.1	44.3	40.1	179.3	175.4	119.5	117.56	227.76	199.58	191.1	198.9	164.7	61.31	126.6	123.3	74.68	106.2	70.78	278.9	300.8	372.8	294	228	242	3
Os01g0814300	34.7	31.7	40.9	202.8	209.7	167.8	116	91.1	89.1	27.59	30.7	20.7	52.249	44.806	32.471	23.88	39.16	44.55	173.7	208.8	159.5	113.1	110.5	96.78	21.13	32.06	26.32	52.7	38.3	39.2	10
Os01g0819700	129	136	98.1	6.568	3.616	9.516	0	8.22	8.29	23.28	19.74	12.23	10.885	8.4011	12.671	95.53	102	49.73	6.503	2.419	12.93	31.79	34.92	42.61	23.24	45.26	24.12	24.1	30.6	16.7	14
Os01g0651150	2.04	0	3.23	0	0.904	0.865	7.05	4.43	4.84	2.587	2.193	0	2.177	4.6673	0	0	3.092	2.072	1.858	0.806	6.036	2.218	3.563	0.722	1.056	0	0	1.85	0.96	0.83	18
Os01g0611300	68.3	52.2	61.4	119.9	144.6	97.75	74.4	68.3	53.9	63.8	78.94	53.63	63.134	57.874	37.222	71.65	51.53	56.98	127.3	112.1	174.2	37.71	37.77	73.67	47.54	42.43	54.82	39.8	40.2	45	10
Os01g0839900	4.08	2.79	4.31	0	0	0	0	0	0	0	3.289	1.882	0	2.8004	0	0	16.49	2.072	0.929	0	8.622	0	0	0	2.113	0.943	15.35	4.63	2.87	3.33	11
Os01g0145200	11.2	7.45	9.7	9.031	9.944	6.055	7.83	5.06	7.6	12.93	10.96	5.645	6.5311	3.7338	7.9197	11.94	2.061	7.252	3.716	5.644	9.484	8.133	14.25	9.389	8.451	1.886	6.579	13	14.3	8.33	8
Os01g0106600	9.18	4.66	2.16	10.67	9.944	9.516	2.35	4.43	12.4	3.449	1.096	15.05	7.6196	9.3345	4.7518	0	7.214	9.324	8.361	3.225	2.587	6.654	4.988	0.722	4.225	16.03	10.96	10.2	1.91	5	20
Os01g0883800	421	311	395	385.9	539.7	221.5	223	248	129	1024	1331	1060	1159.3	727.16	997.88	895.6	350.4	359.5	209.9	287.8	258.7	25.88	57.72	23.83	829.2	535.5	429.8	445	417	458	3
Os01g0317500	1.02	0.93	0	1.642	1.808	6.055	2.35	2.53	1.38	0.862	0	2.822	0	0	0.792	0	2.061	0	7.432	3.225	9.484	2.958	2.138	2.889	0	0	0	0.93	0.96	0	10
Os01g0872800	751	984	679	1204	1250	1095	2698	2994	2802	1085	1191	1340	980.76	802.77	898.09	1075	807.9	799.8	931.7	1062	919.1	2325	2096	2379	1101	1007	964.9	676	690	794	18
Os01g0963300	166	175	165	207.7	192.5	177.3	363	306	299	235.4	217.1	293.5	238.39	234.3	239.17	143.3	192.7	203.1	237.8	187.9	236.2	232.9	200.3	218.8	201.8	216.9	219.3	199	185	232	15
Os01g0662600	6765	6626	6997	3826	3767	3794	3061	2892	2824	11558	12377	14247	10639	10237	10707	13469	8162	7536	5584	5621	5429	1712	2045	2531	10999	11690	11800	7361	7686	7536	3
Os01g0743100	13.3	2.79	1.08	0.821	0.904	0	1.57	1.9	0	3.449	1.096	0	1.0885	0.9335	0.792	0	2.061	5.18	1.858	0.806	0	0.739	0	1.444	1.056	0.943	0	1.85	2.87	0	19
Os01g0679500	0	0	0	0	0	0	0	0	0	0	0	0	0	0	0	0	0	1.036	0	0	0	0	0	0	0	0	0	0	0	0	1
Os01g0953500	421	497	524	333.3	303.7	299.3	240	312	248	750.1	631.5	631.3	522.49	594.61	662.88	1158	631.7	528.4	392	387	404.4	230.7	374.8	319.2	683.5	574.2	517.5	661	668	651	13
Os01g0192300	212	366	318	375.2	377	312.3	728	674	614	381.1	402.4	379.2	443.03	385.52	392.82	489.6	276.2	318.1	253.6	276.6	346.6	287.6	367	354.6	322.2	356.4	339.9	263	354	302	18
Os01g0170600	3262	2534	2712	2161	2257	2779	1731	1747	2253	3282	2618	5196	3634.6	6400.7	4181.6	5493	4161	3654	4329	2960	3639	2050	2421	1919	5474	5063	7070	4810	4325	4998	3
Os01g0333001	21.4	32.6	26.9	41.87	59.66	30.28	32.9	22.1	34.6	12.93	19.74	16.93	23.947	14.935	15.047	11.94	6.183	18.65	17.65	26.61	18.97	39.19	59.86	29.61	13.73	11.31	21.93	18.5	18.2	20	17
Os01g0299500	1794	1782	1780	1448	1381	1392	1894	2022	1968	1925	2152	1925	1579.4	1622.3	1997.3	2830	1924	1967	1458	1520	1414	2187	2050	1701	2121	2057	1928	1943	2031	2118	13
Os01g0650100	75.4	65.2	36.6	21.35	21.7	28.55	24.3	7.59	20.7	56.9	57.01	42.34	38.098	65.342	42.766	35.82	29.89	40.41	10.22	20.16	25.87	36.23	19.24	46.22	57.04	58.46	100.9	29.6	38.3	32.5	11
Os01g0921100	20.4	14	19.4	13.96	12.66	13.84	20.4	23.4	21.4	18.11	35.08	26.34	17.416	30.804	29.303	59.7	17.52	16.58	14.86	3.225	9.484	40.67	56.3	64.28	40.14	59.4	70.17	39.8	70.8	29.2	11
Os01g0772800	1032	1083	1066	1134	1257	1162	1197	1213	1272	1218	1235	1212	1085.3	1121.1	1143.6	1731	1033	1050	1134	1093	1041	1023	1184	1013	1124	1122	1053	1025	1030	974	6
Os01g0913400	40.8	31.7	31.2	96.88	111.2	96.89	62.6	56.3	45.6	16.38	32.89	29.17	27.213	35.471	18.215	47.76	27.82	24.86	98.47	94.33	82.77	68.02	92.64	46.22	25.35	14.14	19.74	32.4	40.2	25.8	10
Os01g0675000	0	6.52	0	0	0	0	0	0	0	0	0	0	0	0	0	0	0	0	0	0	0	0	0	0	0	0	0	0	0	0	14
Os01g0283600	0	0.93	0	0	0	0	0	0	0	0	0	0	0	0	0	0	0	0	0	0	0	0	0	0	0	1.886	0	0	0	0	1
Os01g0262500	1094	1313	1087	394.1	285.7	462.8	222	245	370	1292	1308	1293	1078.7	1195.8	1209.3	1887	1268	1096	565.7	435.4	461.3	267.7	374.8	401.6	1957	1549	1362	1907	1291	1888	3
Os01g0133866	0	0	0	0	0	0	0	0	0	0	0	0	0	0	0	0	0	1.036	0	0	0	0	0	0	0	0	0	0	0	1.67	1
Os01g0633200	1634	1695	1651	1555	1678	1478	1861	2035	1700	1538	1627	1432	1688.3	1590.6	1663.9	3546	1622	1530	1225	1237	1210	1529	1610	1482	1665	1601	1614	1786	1663	1738	6
Os01g0559550	117	121	109	107.5	128.4	125.4	164	208	161	167.3	157.9	207.9	132.8	145.62	118	119.4	153.5	154.4	177.4	180.6	194	109.4	141.1	163.2	195.4	235.7	221.5	138	131	128	20
Os01g0936200	338	438	338	497.5	638.2	572.7	562	624	493	457.8	403.5	407.4	389.69	424.72	445.88	680.6	391.6	433.1	419.9	464.4	475.9	524.2	549.4	479.6	457.4	404.5	429.8	396	369	437	6
Os01g0276100	823	847	775	756.9	751.2	694.6	1071	1042	1110	877.7	764.2	855.2	715.16	799.03	761.08	1099	942.9	810.2	851.9	707.9	788.9	1081	1055	1095	790.2	841	815.8	692	796	747	18
Os01g0387133	13.3	21.4	10.8	24.63	33.45	20.76	29	27.8	26.3	20.69	24.12	14.11	10.885	8.4011	9.5036	11.94	19.58	12.43	13.93	10.48	25	21.44	21.38	24.56	9.507	19.8	30.7	7.4	9.56	6.66	12
Os01g0247500	108	187	181	182.3	132	244.8	233	364	264	104.3	105.3	93.14	87.082	82.144	91.868	441.8	211.3	193.7	229.5	287	424.2	161.2	268.7	203.7	159.5	121.6	175.4	169	164	111	6
Os01g0183100	265	229	236	323.5	409.5	316.6	315	311	325	231.9	194.1	337.8	252.54	261.37	201.16	382.1	192.7	209.3	353.9	378.1	339.7	264	290	257.1	204.9	278.1	282.9	155	220	177	10
Os01g0686100	718	876	805	1268	1343	1296	1065	1141	961	817.4	961.5	931.4	938.3	756.1	750.78	1099	758.5	772.9	969.8	1246	995	1209	979.2	997.4	903.2	837.2	699.5	667	737	879	12
Os01g0855600	21.4	19.6	19.4	4.926	4.52	0.865	10.2	8.86	9.67	12.07	4.386	8.467	3.2656	1.8669	5.5438	23.88	25.76	11.4	3.716	8.869	1.724	18.48	8.552	18.06	3.169	1.886	0	0.93	0.96	3.33	14
Os01g0597701	0	0	0	0	0	0	0	0	0	0	0	0	0	0	0.792	0	0	0	0	0	0	0	0	0	0	0	0	0	0	0	4
Os01g0649100	1232	1136	1137	1612	1617	1798	1926	1428	2016	1409	1121	1645	1180	1402	1151.5	2161	1124	1041	2003	1542	1824	1437	1355	1630	1101	1225	1491	1053	1038	981	12
Os01g0635400	135	130	128	257.8	200.7	296.7	39.1	64.5	51.8	121.6	104.2	154.3	131.71	199.76	125.13	131.4	150.5	126.4	337.2	266.1	376.8	51.76	46.32	59.95	156.3	154.6	122.8	128	106	119	16
Os01g0735450	0	0	1.08	3.284	1.808	0.865	3.91	3.8	6.91	1.724	1.096	0	0	3.7338	1.5839	0	1.031	4.144	4.645	0	0.862	2.958	1.425	5.056	1.056	0	0	0.93	0.96	0.83	18
Os01g0956075	16.3	4.66	15.1	15.6	15.37	11.25	6.26	11.4	16.6	5.173	3.289	15.99	3.2656	14.002	9.5036	11.94	19.58	6.216	6.503	4.838	3.449	37.71	28.51	40.45	6.338	5.657	8.772	17.6	11.5	12.5	9
Os01g0187400	1818	1900	1824	1483	1460	1510	1257	1209	1332	1681	1673	1515	1925.6	1807.2	1942.7	3057	2010	1936	1361	1346	1318	1846	1698	1593	2037	1847	1557	2133	1915	2184	13
Os01g0580150	0	0.93	0	0	0	0	0	0	0	0	0	0	0	0	0	0	0	0	0	0.806	0.862	0	0	0	0	0	0	0	0	0	16
Os01g0777700	304	420	394	440	499.9	393.6	167	226	187	161.2	180.9	110.1	255.8	252.97	268.48	692.6	260.7	341.9	177.4	316.9	182.8	535.3	446.8	300.5	239.8	214	149.1	397	392	404	8
Os01g0716200	0	2.79	2.16	10.67	6.328	8.65	5.48	4.43	8.29	2.587	1.096	0.941	0	3.7338	0	0	0	0	2.787	3.225	6.898	2.958	2.851	2.167	1.056	3.771	2.193	2.78	1.91	1.67	12
Os01g0184500	824	771	918	1804	1957	1511	1433	1590	1358	739.8	813.5	945.5	1137.5	1058.5	827.6	1731	787.3	829.9	1694	1650	1560	1024	930	1005	682.4	688.3	616.2	687	753	779	10
Os01g0197400	301	312	333	362	384.2	398.8	784	824	797	484.5	451.7	484.5	448.47	363.11	357.97	800	322.6	354.3	478.4	509.6	363.9	602.6	514.5	578.5	436.3	420.5	307	335	328	336	18
Os01g0248701	13.3	7.45	5.39	40.23	6.328	36.33	20.4	133	115	39.66	52.63	7.527	28.302	3.7338	7.9197	0	3.092	7.252	27.87	4.838	6.036	70.24	34.21	11.56	5.282	1.886	54.82	0	10.5	3.33	18
Os01g0509400	0	0.93	0	0	0	0	0	0	0	0	0	2.822	0	0	0	0	2.061	0	0	0	0	0	0	0.722	0	1.886	0	0.93	0	0	20
Os01g0930300	3.06	14.9	8.62	55.01	48.81	44.98	39.9	44.3	51.1	9.484	7.675	1.882	3.2656	7.4676	3.1679	0	5.153	2.072	41.8	58.05	27.59	40.67	34.21	37.56	3.169	7.543	2.193	12	10.5	10	12
Os01g0127500	0	0	0	3.284	4.52	0.865	5.48	1.27	0	2.587	0	0	0	0	0	0	0	0	1.858	1.613	0.862	0	0.713	2.167	0	0	2.193	0	0.96	0	12
Os01g0151900	13.3	20.5	8.62	108.4	112.1	85.64	80.6	77.8	56.7	12.07	16.45	10.35	10.885	13.068	19.007	23.88	7.214	13.47	98.47	96.75	89.67	58.41	57.01	46.22	5.282	5.657	17.54	6.48	11.5	7.5	12
Os01g0128100	0	1.86	0	0.821	0	0	0.78	0.63	0	0	0	0	1.0885	0.9335	0	0	0	0	0	0	0	2.218	0.713	0	0	0	0	0	0.96	0	17
Os01g0949060	177	167	186	379.3	397.7	320.9	241	256	199	174.2	127.2	123.2	132.8	205.36	162.35	250.8	165.9	191.7	396.7	392.7	424.2	211.5	210.2	184.2	107.7	143.3	193	135	142	137	10
Os01g0702700	9.18	8.38	6.47	19.7	26.22	25.09	29	19	10.4	13.79	16.45	5.645	3.2656	10.268	9.5036	23.88	17.52	9.324	9.29	15.32	13.8	26.62	34.21	39	9.507	4.714	8.772	12	23.9	10	17
Os01g0923950	1.02	3.73	1.08	2.463	2.712	0	1.57	0.63	0.69	0.862	2.193	0	0	1.8669	0	11.94	5.153	5.18	1.858	1.613	2.587	0	3.563	0	0	0	0	5.55	6.69	3.33	6
Os01g0871000	0	0	0	0	0	0.865	0	0	0	0	0	1.882	0	0	0	0	0	0	0	0	0	0	0	0	0	0	0	0	0	0.83	20
Os01g0660200	317	543	522	285.7	443.8	263.8	436	557	222	1270	632.6	681.2	294.99	413.52	468.84	597	228.8	369.9	133.8	239.5	129.3	264.7	413.3	322.1	398.2	615.7	390.3	307	465	232	7
Os01g0841000	223	190	226	76.35	66.89	52.77	250	140	159	238	193	278.5	182.87	148.42	117.21	441.8	168	170.9	103.1	74.98	67.25	249.9	151.1	244.1	97.19	138.6	131.6	144	142	130	6
Os01g0891066	0	0	0	0	0	2.595	0	0	0	0	0	0	0	0	0	0	0	0	0	0	0	0	0.713	0	2.113	0	0	0	0.96	0	2
Os01g0187000	17.3	15.8	16.2	11.49	8.136	11.25	18.8	12	15.2	28.45	9.868	31.05	17.416	8.4011	13.463	0	15.46	14.5	25.08	12.09	19.83	17.75	19.95	25.28	15.85	16.03	17.54	12	19.1	25.8	20
Os01g0387865	0	0.93	0	0	0	0	0.78	0	0	0	0	0	0	0	0	0	0	0	0	0	1.724	0.739	0	0	0	0	0	0	0	0	16
Os01g0235400	314	269	287	436.8	455.6	393.6	324	348	352	407.8	332.2	438.4	403.84	318.31	335	537.3	273.1	276.6	423.6	391	432.8	244	317.8	271.6	278.9	319.6	291.7	290	340	273	6
Os01g0890200	0	0	0	0	0.904	0	0	0	0	0	0	0	0	0	1.5839	0	0	0	0	0	0	0	0.713	0.722	0	0	0	0	0	0	4
Os01g0616400	406	417	427	435.9	499	475.8	531	560	560	445.8	445.1	503.3	423.43	441.52	445.88	955.3	479.2	435.1	448.7	426.5	463.9	511.7	464.6	468	399.3	462	438.6	462	438	444	6
Os01g0558800	715	754	832	1321	1709	1366	936	1098	918	814.8	1087	995.4	1185.4	909.18	915.51	1505	649.2	743.9	1286	1617	1386	664.7	677.7	819.7	750	720.3	594.3	542	698	629	10
Os01g0269100	416	395	423	402.3	392.3	417	500	551	533	411.3	426.5	387.6	398.4	384.58	423.7	740.3	396.8	367.8	399.4	372.5	378.5	448.1	493.1	478.8	376.1	344.1	403.5	410	419	405	6
Os01g0918500	411	353	371	218.4	241.4	261.2	211	235	240	251.8	225.9	237.1	287.37	255.77	266.1	525.4	306.1	335.7	219.2	255.6	244.9	275.8	280.1	252.1	312.7	263.1	344.3	306	292	313	13
Os01g0807000	0	0	0	0	0	0	1.57	0	0.69	0	0	0	0	0	0	0	0	2.072	0	0	0	0	0	0	0	0	0	0	0	0	5
Os01g0310600	61.2	98.7	77.6	96.05	109.4	96.02	217	202	228	143.1	161.2	200.4	138.24	104.55	136.22	23.88	98.93	81.85	115.2	93.53	90.53	126.4	104	143	107.7	116.9	116.2	65.7	89.9	65	5

Os01g0651932	0	0.93	0	0	0	0	1.57	0	0	0	0	0	0	0	0	0	0	0	0	0	0	0	0	0	0	0	0	0	0	0	5
Os01g0591000	332	326	359	140.4	112.1	128.9	171	250	190	193.1	160.1	173.1	234.03	269.77	220.96	632.9	518.4	332.6	134.7	88.69	92.26	163.4	208.1	205.1	231.3	193.3	210.5	294	257	292	13
Os01g0803300	2.04	3.73	1.08	0.821	0.904	0	0	0.63	0	32.76	19.74	23.52	4.3541	6.5342	5.5438	11.94	0	4.144	0	0	0	0	0	0	2.113	4.714	0	3.7	10.5	2.5	7
Os01g0357900	210	144	139	19.7	14.46	53.63	28.2	20.2	26.9	64.66	35.08	45.16	43.541	53.207	45.934	167.2	158.7	158.5	29.73	29.83	31.04	61.37	56.3	89.56	45.42	75.43	120.6	53.7	86.1	52.5	14
Os01g0736400	73.4	65.2	59.3	99.34	125.7	160.9	150	93.6	110	64.66	48.24	41.4	58.78	67.209	60.981	167.2	81.41	98.42	115.2	116.9	113.8	143.4	128.3	163.9	69.72	61.28	39.47	90.7	87	94.1	12
Os01g0949900	1.02	0.93	4.31	17.24	25.31	18.17	11.7	21.5	2.76	1.724	1.096	0.941	2.177	4.6673	5.5438	11.94	6.183	0	17.65	20.96	2.587	18.48	35.63	12.28	1.056	0	6.579	1.85	2.87	4.17	12
Os01g0167900	91.8	86.6	84.1	53.36	43.39	89.1	25.8	25.9	40.1	72.42	54.82	48.92	43.541	52.273	45.934	35.82	106.1	64.23	61.31	58.86	51.73	99.82	97.63	76.56	85.57	47.14	59.21	79.6	101	68.3	1
Os01g0108800	7.14	22.4	12.9	9.031	6.328	7.785	21.1	11.4	26.9	18.11	29.6	11.29	10.885	28.004	15.839	23.88	16.49	21.76	2.787	5.644	5.173	18.48	13.54	12.28	28.52	34.89	19.74	26.8	24.9	23.3	1
Os01g0509700	103	103	115	57.47	90.4	70.07	163	110	128	118.1	120.6	77.15	93.613	95.212	72.861	83.59	93.78	90.14	53.88	59.66	65.53	136	65.56	132.2	84.51	103.7	100.9	85.1	76.5	78.3	5
Os01g0780400	639	691	624	406.4	405	442.9	309	366	303	421.6	433.1	333.1	426.7	468.59	490.23	1170	551.3	539.8	346.5	333.8	300.9	443.6	387.7	432.6	378.2	466.7	530.7	598	516	557	13
Os01g0775100	1583	1682	1505	335.8	327.2	327.9	352	435	444	1093	1174	1023	1151.7	1085.6	1271.9	2376	1762	1676	335.4	439.4	328.5	778.6	870.8	808.2	1655	1750	1443	2220	1646	2096	13
Os01g0812600	0	0	0	0.821	0	0.865	0	0	0.69	0	0	0	0	0	0	0	0	0	0	0	0	0.739	0	0	0	0.943	0	0	1.91	0	2
Os01g0670100	17.3	16.8	20.5	141.2	156.4	82.18	63.4	75.3	95.4	12.07	8.771	8.467	21.77	5.6007	14.255	0	14.43	10.36	103.1	80.63	56.91	217.4	90.5	216.7	10.56	4.714	10.96	18.5	21	4.17	12
Os01g0686800	1547	1194	1228	1609	1630	1573	1329	1168	1522	1675	1420	2473	1463	1809	1167.4	2114	1153	1465	1852	1461	1634	1189	1298	1204	1249	1631	1754	903	1165	950	20
Os01g0310700	3.06	0	17.2	27.09	37.97	29.41	23.5	70.8	56	12.07	9.868	1.882	7.6196	4.6673	15.047	35.82	6.183	4.144	11.15	20.16	5.173	8.873	11.4	15.89	14.79	7.543	2.193	7.4	4.78	9.16	18
Os01g0267050	0	0.93	1.08	0	0.904	0	0	0	0	0	0	0	0	0	0.792	0	0	0	0	0	1.724	0	0.713	0	0	0	0	0	0	0	16
Os01g0338600	1249	1227	1385	704.4	759.3	641.9	1086	1178	1180	1342	1397	1796	1463	1293.8	1513.4	2376	1635	1472	907.6	868.4	882	908.7	860.2	931	1492	1631	1651	1341	1507	1469	13
Os01g0593600	94.8	69.9	69	71.42	65.09	77.85	128	125	118	106.9	84.42	107.3	90.347	111.08	66.525	107.5	56.68	75.63	104	104	62.08	79.85	84.09	94.61	82.4	80.14	89.91	80.5	60.2	69.1	5
Os01g0965900	1806	1515	1947	902.3	952.8	848.6	516	978	473	1499	1313	1290	1821.1	2021.9	1957.7	4406	2133	1625	1195	1440	863.1	819.2	1484	839.2	1909	1371	1585	2443	1960	2049	13
Os01g0963800	6.12	0	6.47	0.821	3.616	1.73	0.78	0	0	3.449	1.096	1.882	4.3541	4.6673	1.5839	0	3.092	1.036	1.858	0	0	0	0	0	0	0	2.193	0	1.91	0.83	19
Os01g0904400	36.7	52.2	39.9	102.6	94.92	102.9	90	82.2	74.6	59.49	37.28	38.57	53.337	37.338	56.23	143.3	45.34	68.38	98.47	91.92	105.2	129.4	123.3	98.95	66.55	88.63	57.02	87	85.1	87.5	6
Os01g0717601	2.04	1.86	2.16	0	3.616	3.46	0	1.9	0	0.862	1.096	0	2.177	0.9335	1.5839	0	4.122	3.108	0.929	2.419	0	0.739	0	2.167	6.338	4.714	4.386	0.93	3.83	2.5	1
Os01g0533400	0	0	1.08	0	0	0	0	0	0	0	0	0	0	0	0	0	0	0	0	0	0	0	1.425	0.722	0	0	0	0	0	0	17
Os01g0126000	1.02	0.93	0	0	0	2.595	0	0	0	3.449	0	0.941	0	0.9335	0.792	0	0	1.036	0	0	0	3.697	2.138	1.444	4.225	0	2.193	0.93	0.96	0	9
Os01g0770200	125	138	88.4	69.78	84.97	97.75	93.2	86	115	178.5	224.8	139.2	158.92	123.22	145.72	274.6	172.1	149.2	90.11	110.5	127.6	79.85	119	115.6	253.5	165	140.3	262	195	215	13
Os01g0248600	319	331	339	288.2	296.5	307.1	474	481	492	324.2	338.8	333.1	370.1	385.52	350.05	489.6	341.1	358.5	338.1	344.3	370.8	456.2	402.6	420.3	278.9	348.9	353.1	343	342	373	18
Os01g0666400	13.3	2.79	8.62	9.852	11.75	18.17	14.9	13.3	14.5	12.07	10.96	17.88	25.036	19.602	16.631	47.76	34.01	24.86	12.08	12.09	7.76	14.05	15.68	18.06	14.79	23.57	32.89	23.1	25.8	20.8	13
Os01g0736551	52	57.7	37.7	12.31	13.56	7.785	21.9	14.5	42.8	37.94	21.93	47.04	18.505	35.471	48.31	95.53	52.56	46.62	11.15	12.09	7.76	62.85	44.18	46.22	57.04	53.74	41.67	50	25.8	40	13
Os01g0500100	2.04	11.2	7.54	4.105	6.328	4.325	9.39	4.43	8.98	6.897	1.096	1.882	2.177	2.8004	3.9598	0	3.092	3.108	0.929	4.838	11.21	8.873	6.414	5.056	7.395	7.543	10.96	13	1.91	8.33	1
Os01g0785700	314	263	269	435.1	419.4	434.3	323	274	305	276.8	255.5	257.8	247.09	366.85	223.33	465.7	209.2	261.1	395.7	434.6	456.1	264.7	340.6	280.2	197.5	203.7	309.2	192	250	224	10
Os01g0920100	1583	1909	1739	3650	3905	2924	3504	3179	2771	2049	2123	1200	1719.9	1671.8	1894.4	2902	1348	1570	2382	2591	1743	2702	2871	2235	1625	1501	1496	2044	1965	2125	12
Os01g0817700	3394	3312	3477	8032	8572	8504	2982	2608	3191	2290	2002	2512	1798.2	2659.4	2097.9	4848	3311	3163	8233	6985	8562	3593	3539	3863	2416	2361	2546	3099	2647	2567	10
Os01g0929500	230	167	165	245.5	200.7	207.6	235	254	274	224.2	216	238	217.7	209.09	221.75	346.3	140.2	139.9	206.2	201.6	228.5	237.3	246.6	207.3	198.6	193.3	182	197	177	172	6
Os01g0801200	196	183	182	185.5	160	161.8	224	225	224	162.1	163.4	205.1	173.07	217.49	174.23	250.8	153.5	177.2	187.6	192.7	219	228.5	215.9	224.6	171.1	215.9	214.9	165	209	146	18
Os01g0302250	14.3	21.4	14	55.83	60.57	40.66	42.3	43	49.1	17.24	24.12	17.88	42.452	23.336	21.383	11.94	10.31	17.61	41.8	40.31	44.84	14.05	11.4	28.17	14.79	18.86	15.35	10.2	13.4	11.7	12
Os01g0144100	348	393	349	628.9	659	547.6	526	571	530	435.4	449.5	794.1	242.74	142.82	143.35	250.8	168	259	515.6	444.3	532	427.4	272.2	481	315.9	277.2	193	73.1	114	125	12
Os01g0198702	3.06	4.66	1.08	0.821	2.712	0.865	2.35	1.27	4.15	1.724	4.386	0	1.0885	1.8669	1.5839	0	6.183	0	0.929	5.644	3.449	3.697	4.276	6.5	0	0.943	0	2.78	1.91	1.67	9
Os01g0725900	0	1.86	0	0	0	0.865	0	0	0	0	0	0	0	0	0	0	0	0	0	0	0	0	0	0	0	0	0	0	0	0.83	14
Os01g0252600	239	266	270	322.6	304.6	288.9	1108	960	1115	379.4	459.4	540	426.7	350.98	338.96	752.3	358.6	303.6	531.4	503.9	629.4	510.2	473.2	701.3	364.4	347	278.5	260	332	292	18
Os01g0706900	88.7	85.7	76.5	23.81	21.7	19.9	27.4	27.8	38.7	100	87.71	87.5	142.6	74.676	87.908	143.3	75.23	90.14	24.15	20.16	21.56	74.68	75.54	54.89	107.7	71.66	24.12	63.8	78.4	79.1	13
Os01g0277100	0	3.73	2.16	3.284	6.328	0.865	23.5	8.86	8.29	0	8.771	1.882	1.0885	1.8669	0.792	0	3.092	0	0.929	0	0.862	9.612	2.851	8.667	2.113	1.886	0	0	1.91	0	18
Os01g0140500	320	268	259	366.2	384.2	374.6	701	517	639	413	310.3	590.8	362.48	394.85	258.97	776.2	254.5	272.5	469.1	326.5	438	327.5	379.8	412.4	301.1	325.3	346.5	190	262	202	6
Os01g0303100	0	0	0	0.821	3.616	0.865	0	0	0	0	0	0	0	0	0	0	0	0	6.503	4.031	6.898	0	0	1.444	0	0	0	0	0	0	16
Os01g0185200	2036	2230	2075	2601	2788	2471	1878	1870	1541	1775	1734	1611	1973.5	2101.2	1968	2317	2251	1940	2434	2125	2349	1774	1980	1831	1847	1836	1903	1900	1687	2012	10
Os01g0899000	346	404	345	265.2	270.3	261.2	210	213	223	262.1	285.1	245.6	284.1	210.03	258.74	525.4	325.6	290.1	191.4	214.5	239.7	283.9	263	289.6	281	271.5	285.1	268	239	267	13
Os01g0192000	175	279	253	310.3	241.4	373.7	254	159	121	240.6	330	214.5	182.87	143.75	205.91	262.7	184.5	144	223.9	389.4	347.5	222.6	196.7	221	159.5	172.5	195.2	167	156	147	10
Os01g0540800	478	505	533	449.9	447.5	493.1	173	227	199	197.4	275.2	163.7	339.62	319.24	361.93	979.2	441.1	519.1	213.7	314.4	265.6	543.4	465.4	258.6	347.5	324.3	267.5	486	495	551	8
Os01g0276400	168	131	169	87.02	95.82	115.1	121	86.7	82.2	125.9	112.9	121.4	115.38	114.81	91.076	179.1	134	146.1	86.39	116.9	87.95	95.38	79.1	93.89	105.6	99	83.33	91.6	89.9	104	14
Os01g0718000	139	97.8	112	51.72	48.81	46.71	75.2	68.9	74.6	61.22	48.24	47.98	97.967	93.345	77.613	310.5	120.6	104.6	102.2	67.73	115.5	76.9	67.7	73.67	79.23	77.31	72.37	74	80.3	60.8	6
Os01g0884900	3205	2717	3391	2067	2026	2106	2373	2271	2010	3554	3760	4035	4747	4526.3	4900.7	5553	4392	3404	3557	3723	3802	1102	1298	1711	3765	3824	3746	3748	3824	3677	13
Os01g0838350	481	336	560	231.5	97.63	175.6	427	330	186	562.1	548.2	448.8	345.06	535.8	699.31	716.5	681.2	423.7	834.2	532.1	830.3	373.4	481.7	1112	593.7	594.9	973.7	712	687	425	11
Os01g0299200	24.5	15.8	17.2	87.84	84.07	61.42	28.2	40.5	43.5	7.76	10.96	4.704	4.3541	6.5342	10.296	0	2.061	10.36	36.23	58.86	31.9	50.28	55.59	23.11	5.282	0	15.35	13.9	19.1	19.2	2
Os01g0964133	308	277	333	128.9	148.3	118.5	272	280	258	339.7	362.9	396.1	461.53	480.73	426.87	465.7	428.7	374	177.4	198.3	144	166.4	186.7	177.7	346.5	413.9	449.5	459	445	442	3
Os01g0811100	1055	1100	1082	1168	1284	1183	1477	1387	1521	1078	1178	1220	1190.8	1302.2	1212.5	1552	1110	1082	1308	1070	1186	1013	1162	1139	1131	969.2	1011	1029	939	1037	6
Os01g0314800	####	####	####	211.8	76.84	334.8	128	257	1066	12844	10468	23286	8059.4	8167.7	7718.5	25745	15720	12160	275.9	325.7	122.4	346.8	338.5	350.3	11782	19015	14245	5969	7155	5676	13
Os01g0330200	0	0	0	0	0	0	0	0	0	0	0	0	0	0	0	0	0	0	0.929	0	0	0	0	0	0	0	0	0	0	0	16
Os01g0580100	144	142	169	163.4	212.4	173	128	137	95.4	117.3	118.4	78.09	92.524	118.55	84.74	262.7	125.7	130.5	122.6	122.6	117.3	155.3	162.5	138.7	115.1	102.8	87.72	127	126	137	6
Os01g0201800	0	0	0	0	0	0	0	0	0	3.449	1.096	0	0	0	0.792	0	1.031	0	0	0	0.862	0	0	0.722	1.056	0.943	0	0	0.96	0	7
Os01g0627600	2.04	1.86	1.08	2.463	1.808	1.73	0	2.53	0	2.587	0	0	4.3541	2.8004	4.7518	0	0	2.072	2.787	0.806	4.311	0	1.425	0	1.056	0	2.193	0	0	1.67	4
Os01g0909100	116	84.8	87.3	195.4	214.2	198.1	139	112	114	92.25	80.04	72.44	83.816	104.55	80.781	214.9	80.38	92.21	222	236.2	244	116.8	127.6	106.9	81.34	93.34	129.4	112	88.9	111	10
Os01g0142300	1462	1420	1790	435.9	371.5	514.7	613	671	406	1649	2046	1469	1712.2	2077.9	2073.4	3797	2064	1806	993.1	813.5	902.7	700.2	698.4	853	2162	1998	1871	2840	3162	2544	13
Os01g0935200	0	2.79	1.08	4.105	2.712	3.46	0.78	0.63	0.69	2.587	4.386	2.822	1.0885	0	1.5839	0	5.153	4.144	0.929	5.644	4.311	11.09	9.264	13	2.113	2.829	0	1.85	3.83	2.5	9
Os01g0665500	33.6	26.1	7.54	40.23	28.93	33.74	41.5	58.2	47.7	25.87	19.74	28.22	26.124	21.469	19.007	47.76	30.92	27.97	37.16	38.7	27.59	48.06	50.6	43.33	25.35	25.46	15.35	34.2	36.3	25	18
Os01g0117700	54	53.1	47.4	95.23	106.7	110.7	114	82.9	71.9	39.66	55.92	57.39	37.01	52.273	45.142	95.53	40.19	63.2	73.39	73.37	90.53	90.21	91.93	83.78	45.42	44.31	41.67	58.3	56.4	45	12
Os01g0184200	834	825	847	903.1	988	918.7	597	565	493	612.2	646.9	557	638.96	794.37	689.01	2102	753.3	872.3	776.6	906.3	872.6	856.9	714.1	567	607.4	663.8	583.3	832	843	860	6
Os01g0249700	0	0	0	0	0	0	0	0	0	0	0	0	0	0	0	0	0	0	0	0	0	1.479	0	0	0	0	0	0	0	0	9
Os01g0751400	0	0	0	0	0	0	0	0	1.38	0	0	0	0	0	0	0	0	0	0	0	0	0	0	0	0	0	0	0	0	0	18
Os01g0600000	629	511	582	533.6	593	520.8	595	533	623	548.4	515.3	748.9	526.84	547.94	474.8	979.2	554.4	558.4	857.4	631.3	744.1	414.1	453.9	587.9	554.6	614.7	684.2	479	499	436	6
Os01g0526100	308	270	306	325.1	301	282	343	307	319	288.8	354.1	365	374.45	328.57	339.75	585.1	352.4	305.6	312.1	320.1	331.1	278.7	306.4	286	408.8	316.8	350.9	354	356	339	6
Os01g0670500	8.16	2.79	8.62	12.31	6.328	5.19	17.2	13.3	26.9	5.173	3.289	5.645	4.3541	2.8004	2.3759	23.88	9.275	3.108	8.361	8.063	2.587	53.98	42.76	30.33	1.056	4.714	4.386	7.4	6.69	3.33	17
Os01g0793300	21.4	7.45	7.54	19.7	1.808	30.28	0	6.96	152	30.18	60.3	1.882	5.4426	1.8669	3.1679	23.88	35.04	16.58	17.65	4.031	10.35	0	10.69	5.056	3.169	1.886	32.89	3.7	22	10	5
Os01g0245700	0	0	0	0	0	0	0	0	0	0	0	0	0	0	0.792	0	0	0	0	0	0	0	0	0	0	0	0	0	0	0	4
Os01g0928100	470	461	462	478.6	480.9	488.8	636	622	688	431.1	514.2	426.2	486.57	427.52	564.67	931.4	432.8	436.2	406.9	420.1	440.6	604.1	540.2	580	437.3	447.8	478.1	498	510	516	6
Os01g0214500	50	60.5	48.5	17.24	22.6	20.76	61.1	62.6	82.2	50.01	24.12	39.51	32.656	52.273	40.39	23.88	41.22	40.41	26.01	23.38	22.42	25.88	38.48	32.5	30.63	22.63	32.89	32.4	34.4	30.8	5
Os01g0238375	1.02	1.86	1.08	1.642	3.616	1.73	0	0.63	0.69	1.724	1.096	1.882	1.0885	0	1.5839	0	0	0	0.929	0	0	0	0	0	3.169	1.886	0	2.78	0.96	0	2
Os01g0659200	2914	2553	2580	2342	2546	2418	2146	2051	2142	2656	2218	2984	2265.2	2811.6	2198.5	3797	2882	2514	2502	2145	2508	1635	1781	1957	2426	2533	2890	2329	2278	2179	13
Os01g0708900	285	198	309	1955	1677	2066	270	340	311	133.6	141.4	91.26	145.86	141.88	149.68	394.1	145.3	145	2091	2516	2847	346	331.4	326.5	90.85	88.63	81.14	183	203	132	10
Os01g0373500	0	0	0	0	0	0	0	0	0	0	0	0	0	0	0	0	0	1.036	0	0	0	0	0	0	0	0	0	0.93	0.96	0	1
Os01g0332900	1588	1778	1511	2453	2546	2221	4650	4374	4294	2606	2736	3034	2362.1	1966.8	2413.1	2484	1483	1621	3317	2951	2907	2802	3471	2941	2276	2512	2153	1795	2075	2157	18
Os01g0781200	278	292	287	438.4	522.5	414.4	200	285	220	100.9	156.8	65.86	198.11	187.62	170.27	489.6	197.9	226.9	213.7	318.5	282.8	431.8	381.3	271.6	125.7	123.5	72.37	276	268	242	2
Os01g0898300	772	914	736	677.3	624.6	648.8	932	1032	922	660.4	703.9	486.4	605.22	592.74	754.74	1254	785.3	683.8	483.1	549.9	432.8	1062	1204	997.4	782.8	691.1	638.1	1003	799	824	17
Os01g0605700	78.5	81	95.9	50.08	48.81	83.91	132	112	79.5	101.7	108.5	143.9	62.046	109.21	68.901	286.6	205.1	174.1	131.9	91.11	180.2	85.03	98.34	123.5	178.5	259.3	208.3	141	116	107	13
Os01g0764900	3107	4346	3434	5181	5444	3817	2490	2175	2564	2725	3025	2539	2131.3	1888.4	2503.4	3666	2360	2436	3856	2786	3517	2399	1825	2620	1889	2127	2081	1715	1646	1994	2
Os01g0275500	0	0.93	1.08	0	0	0	0.78	0	0	0	0	1.882	0	0	0	0	0	0	0	0	0	0	0.713	0	0	0	0	0	0	0	20
Os01g0754200	647	839	669	793.9	801.8	735.3	622	598	572	755.3	720.3	696.2	592.15	553.54	692.97	1122	675	683.8	536	562.8	507	694.3	673.4	619	743.7	738.2	668.8	715	667	766	6
Os01g0552100	259	169	232	135.5	150.1	125.4	153	184	160	249.2	251.1	373.5	256.89	290.3	289.07	179.1	281.3	245.5	268.5	217.7	226.8	139.7	131.1	138.7	272.5	297	357.4	240	250	263	3
Os01g0726801	9.18	21.4	14	68.96	78.65	65.74	150	106	169	26.73	31.8	28.22	20.682	16.802	26.927	11.94	18.55	27.97	32.51	30.64	50.01	42.88	26.37	52	10.56	14.14	10.96	3.7	9.56	5	18
Os01g0219000	31.6	28.9	26.9	41.87	38.87	33.74	51.7	27.2	56.7	50.01	41.66	47.98	51.16	25.203	33.263	71.65	36.07	29.01	60.38	49.99	65.53	28.1	30.64	30.33	27.47	42.43	30.7	24.1	25.8	20	6
Os01g0659800	11.2	6.52	2.16	6.568	11.75	8.65	17.2	13.9	10.4	6.897	6.578	20.7	5.4426	4.6673	13.463	11.94	13.4	11.4	16.72	11.29	22.42	19.96	12.11	24.56	12.68	8.486	2.193	17.6	11.5	5.83	9
Os01g0228901	5.1	7.45	6.47	13.96	4.52	10.38	0	3.16	2.07	2.587	3.289	0	1.0885	0	8.7116	11.94	9.275	11.4	7.432	4.031	6.036	14.05	6.414	5.056	6.338	5.657	2.193	18.5	7.65	7.5	8
Os01g0384300	101	80.1	81.9	80.46	58.76	55.36	111	130	130	85.36	69.07	93.14	88.17	98.012	84.74	143.3	137.1	94.28	83.61	79.02	73.29	99.82	94.07	123.5	89.79	118.8	87.72	102	86.1	110	5
Os01g0559900	360	248	288	163.4	178.1	282.9	135	99.3	115	375.1	287.3	425.3	198.11	258.57	239.17	226.9	352.4	253.8	404.1	351.5	456.1	73.94	99.06	102.6	309.5	395.1	598.7	234	306	215	20
Os01g0868300	0	0	0	0.821	0	1.73	0	0	0.69	0	0	0	0	0	1.5839	0	1.031	2.072	0	1.613	0	0	1.425	0.722	0	1.886	0	0.93	1.91	0.83	1
Os01g0939100	11.2	6.52	3.23	10.67	13.56	14.71	10.2	24	17.3	4.311	3.289	7.527	5.4426	6.5342	6.3357	11.94	15.46	11.4	11.15	17.74	14.66	28.1	23.52	6.5	9.507	4.714	10.96	8.33	12.4	14.2	17
Os01g0770000	1337	1138	1041	439.2	405.9	397.1	121	137	180	883.7	982.4	892.8	810.95	731.83	691.39	776.2	693.5	632	355.8	281.4	245.7	221.8	335.7	285.3	1166	849.5	739	1032	619	915	3
Os01g0793800	2.04	2.79	4.31	3.284	5.424	3.46	0.78	4.43	6.22	2.587	2.193	0	2.177	0	1.5839	0	3.092	2.072	6.503	7.257	2.587	1.479	5.701	2.889	1.056	0	0	0	0	2.5	12
Os01g0249300	1447	1549	1593	1668	1714	1591	2123	2094	2305	1883	1929	1682	1631.7	1661.5	1693.2	3200	1488	1476	1839	1755	1849	1604	1807	1861	1563	1652	1684	1550	1678	1531	6
Os01g0180700	98.9	61.5	70	136.3	124.7	128.9	146	117	104	87.08	96.48	97.85	62.046	107.35	107.71	286.6	90.69	98.42	147.7	166.1	138.8	103.5	101.9	125.7	109.9	113.1	120.6	74.9	98.5	102	6
Os01g0122000	0	2.79	1.08	0.821	0.904	0	0	0	0	0.862	0	0	1.0885	0	0	0	4.122	1.036	0.929	0	0	0	0	0	0	0	0	0.93	0	0	14
Os01g0555100	29.6	12.1	14	11.49	9.944	5.19	14.9	19.6	20	16.38	35.08	15.99	19.593	21.469	61.773	23.88	11.34	16.58	6.503	7.257	5.173	5.915	18.53	10.83	24.3	20.74	32.89	12	58.3	40	4
Os01g0919800	359	258	230	137.9	132.9	215.4	129	117	126	77.6	119.5	164.6	99.055	165.22	151.27	417.9	253.5	513.9	173.7	130.6	126.7	118.3	153.9	129.3	176.4	194.2	243.4	304	318	350	13
Os01g0612350	0	0	0	0	0	0	0	0	0	0	0	0	0	0	0	0	0	0	0	0	0	0	0	0	0	0	0	0	0	0.83	1
Os01g0644600	29.6	24.2	24.8	4.926	2.712	0.865	3.91	2.53	3.46	27.59	24.12	36.69	14.151	20.536	22.175	47.76	27.82	15.54	11.15	4.031	8.622	2.218	5.701	4.333	19.01	39.6	24.12	27.8	18.2	23.3	13
Os01g0211600	40.8	37.3	46.3	53.36	40.68	22.49	13.3	33.5	23.5	30.18	15.35	48.92	32.656	34.538	30.095	11.94	38.13	33.15	19.51	19.35	32.76	11.09	29.93	16.61	25.35	16.03	13.16	20.4	11.5	25	19
Os01g0150500	5.1	0.93	1.08	1.642	2.712	3.46	0.78	0	1.38	5.173	2.193	1.882	2.177	5.6007	0.792	0	2.061	1.036	1.858	1.613	7.76	2.218	0.713	1.444	4.225	4.714	4.386	0.93	1.91	1.67	16
Os01g0899500	713	795	670	682.2	508	650.5	1412	1549	1311	541.5	668.8	636.9	412.55	628.21	653.37	1146	877	825.7	833.3	872.4	672.5	1646	1430	2000	733.1	764.6	789.5	1026	974	844	9
Os01g0681100	4.08	8.38	5.39	4.926	1.808	3.46	5.48	6.96	9.67	6.035	0	1.882	0	4.6673	3.9598	0	5.153	3.108	1.858	5.644	1.724	8.873	4.276	3.611	6.338	2.829	8.772	1.85	2.87	0.83	18
Os01g0165200	22.4	9.31	7.54	18.88	16.27	25.09	17.2	8.86	19.3	20.69	14.25	9.408	8.7082	9.3345	17.423	11.94	19.58	20.72	18.58	17.74	18.11	32.53	29.93	36.11	22.18	12.26	28.51	22.2	5.74	17.5	9
Os01g0753150	0	1.86	0	3.284	7.232	6.92	3.91	8.22	0.69	6.897	0	2.822	1.0885	2.8004	3.1679	11.94	8.244	1.036	2.787	6.45	6.898	5.176	4.276	5.778	0	2.829	8.772	2.78	3.83	6.66	8
Os01g0730900	24.5	36.3	10.8	22.17	10.85	12.98	4.7	0.63	4.84	3.449	6.578	4.704	6.5311	4.6673	5.5438	11.94	29.89	36.26	7.432	8.869	1.724	19.96	18.53	21.67	7.395	13.2	10.96	30.5	22	24.2	8
Os01g0386500	0	1.86	0	0	0.904	0	0	0	0	0	0	0	0	0	0	0	0	1.036	0	0	1.724	0.739	2.851	0	1.056	0	0	0.93	1.91	0	17
Os01g0678500	1145	1174	1152	1020	1091	1094	1067	1144	1169	1072	1252	1136	730.4	741.16	715.94	1505	1105	997.7	775.7	885.3	789.2	1094	1192	1039	1315	1335	1169	825	765	853	6
Os01g0223800	9.18	2.79	5.39	11.49	12.66	16.44	6.26	6.96	21.4	4.311	10.96	5.645	3.2656	5.6007	6.3357	11.94	3.092	2.072	7.432	4.031	0.862	11.09	10.69	9.389	7.395	1.886	0	4.63	6.69	7.5	2
Os01g0121600	115	102	75.4	50.9	34.35	40.66	20.4	56.9	22.8	118.1	107.4	99.73	72.931	118.55	124.34	179.1	163.9	100.5	70.6	65.31	56.91	52.5	54.87	59.22	101.4	82.03	85.52	85.1	88.9	113	13
Os01g0667200	546	456	490	463.9	497.2	424.7	425	545	538	392.3	300.4	382	524.67	499.4	504.48	692.6	420.5	411.3	495.1	412	413.9	337.2	433.3	405.9	380.3	248.9	298.2	405	423	402	6
Os01g0180800	768	495	662	248.8	160.9	293.3	246	178	205	865.6	752.1	1194	953.54	893.31	921.06	1278	633.8	617.5	527.6	428.9	463.9	167.8	129.7	201.5	809.2	861.8	943	663	711	577	3
Os01g0152951	47.9	58.7	43.1	45.97	28.02	31.14	28.2	38	26.3	75.87	60.3	80.91	51.16	49.473	38.014	35.82	44.31	50.77	39.02	52.41	54.32	22.92	19.95	18.06	44.37	38.66	59.21	35.2	51.6	50	20

Os01g0113400	30.6	29.8	24.8	21.35	17.18	14.71	29	15.2	13.8	24.14	19.74	9.408	22.859	15.869	18.215	35.82	18.55	33.15	23.22	24.19	24.14	22.92	20.67	20.95	22.18	18.86	21.93	19.4	22	20.8	14
Os01g0321800	232	195	192	231.5	219.7	226.6	312	314	286	246.6	262	300.1	257.98	294.97	300.16	226.9	216.4	211.4	273.1	234.6	233.7	238.1	251.6	244.8	294.7	225.3	225.9	248	212	267	5
Os01g0964400	0	2.79	0	0.821	1.808	2.595	0	0	0	0	3.289	0	2.177	3.7338	3.9598	0	1.031	6.216	0.929	4.838	0.862	3.697	3.563	2.889	2.113	1.886	0	0.93	5.74	5	1
Os01g0778500	0	0	0	0	0	3.46	1.57	0	0.69	1.724	0	0	0	0	0	0	0	0	0.929	0	3.449	0	0	0	0	0	0	0	0	0	16
Os01g0647200	27.5	43.8	21.6	19.7	29.83	6.92	38.4	8.22	4.15	155.2	162.3	350.9	3.2656	2.8004	12.671	0	29.89	39.37	127.3	120.1	49.15	27.36	19.95	45.5	95.07	558.2	934.2	15.7	91.8	247	11
Os01g0720600	471	433	388	367.8	386	381.5	316	390	370	348.3	457.2	444.1	394.04	342.58	338.96	501.5	332.9	375	268.5	272.5	269	312	355.6	343.1	469	492.2	379.4	291	286	264	13
Os01g0915600	479	532	613	587.8	596.6	481.8	816	786	670	411.3	508.7	459.1	483.3	495.66	487.06	1075	590.5	590.5	521.1	545	617.3	553.8	740.4	761.2	396.1	461	478.1	495	504	478	6
Os01g0205600	21.4	14	26.9	17.24	14.46	6.92	36	19	26.9	19.83	32.89	29.17	26.124	35.471	18.215	47.76	24.73	17.61	19.51	8.063	13.8	15.53	19.24	30.33	6.338	23.57	28.51	25	18.2	9.16	6
Os01g0626100	48.9	59.6	48.5	38.59	59.66	44.12	13.3	24.7	20	25	16.45	10.35	44.629	17.736	21.383	47.76	45.34	42.48	7.432	16.13	9.484	59.89	54.87	31.06	25.35	12.26	8.772	52.7	44	67.5	8
Os01g0962500	80.5	54	76.5	83.74	93.11	76.12	50.9	47.4	57.4	61.22	65.78	88.44	69.665	53.207	53.854	119.4	96.87	88.06	83.61	94.33	81.91	76.9	59.15	69.34	82.4	83.91	74.56	61.1	60.2	66.6	6
Os01g0748950	0	1.86	1.08	0	0	0.865	0	0	1.38	0	0	0	0	0	0	0	0	0	0	0	0	0.739	0.713	0	1.056	0	0	0	0	0	14
Os01g0767100	477	522	432	625.6	713.2	583.9	1170	1102	1130	515.6	471.4	581.4	306.96	353.78	393.61	358.2	391.6	426.8	614	595.8	659.6	919.1	878.7	1030	400.4	487.4	506.6	376	312	339	18
Os01g0346700	559	526	639	280.8	194.4	258.6	146	175	142	764.8	731.3	574.8	778.29	737.43	772.17	1337	746.1	759.4	435.7	437	510.4	108.7	143.2	148.1	821.9	770.3	734.6	755	755	831	13
Os01g0755500	0	0.93	0	1.642	1.808	0.865	1.57	1.9	0	0	0	0.941	0	0	0	0	0	0	0.929	0	6.898	2.218	2.138	2.889	0	0	0	1.85	1.91	0	12
Os01g0388500	755	768	819	734	793.7	719.7	667	715	666	745.8	826.7	732	760.88	794.37	839.48	1719	854.3	915.9	806.3	917.5	934.6	826.6	724.7	665.2	766.9	839.1	682	882	852	817	6
Os01g0135800	3.06	0	0	0	0.904	1.73	3.91	1.27	2.07	0	0	0	0	0.9335	0.792	0	0	2.072	0.929	1.613	0.862	4.436	0	5.056	0	0	0	0	0	0.83	9
Os01g0627400	35.7	36.3	47.4	14.78	27.12	17.3	8.61	20.9	15.9	37.07	42.76	44.22	51.16	67.209	53.854	95.53	59.77	70.45	32.51	37.9	33.63	7.394	7.839	7.222	38.03	47.14	46.05	42.6	52.6	43.3	13
Os01g0943900	0	0.93	0	0.821	2.712	1.73	1.57	0.63	1.38	1.724	0	1.882	0	0	0.792	0	0	0	1.858	3.225	0	0	0.713	1.444	2.113	0	0	0.93	0	2.5	2
Os01g0592500	1.02	0	0	0	0	0.865	0	0	1.38	0	2.193	0	1.0885	0.9335	0	0	0	0	0	0	0	0.739	8.552	1.444	0	0	0	0	6.69	1.67	17
Os01g0933600	927	1011	843	915.4	844.3	941.2	1829	1480	1665	1208	1312	1412	1169.1	1152.8	1229.1	1588	1132	1133	1186	1325	1309	1213	1100	1405	1262	1252	1314	1040	1101	1154	5
Os01g0844800	708	727	595	871.9	875	911.8	884	806	792	825.1	778.4	829.8	696.65	572.21	669.21	585.1	559.6	667.2	855.6	864.3	745	774.9	810.3	731.6	689.8	713.7	576.7	592	557	556	12
Os01g0617900	41.8	34.5	45.3	90.31	113.9	95.16	61.8	70.2	45.6	28.45	47.14	44.22	59.869	38.272	27.719	59.7	35.04	31.08	87.32	86.27	103.5	58.41	47.03	46.95	32.75	25.46	17.54	28.7	33.5	45.8	10
Os01g0606900	91.8	40.1	63.6	1812	1575	1924	1.57	3.8	0	15.52	9.868	9.408	4.3541	7.4676	7.1277	0	25.76	25.9	1875	2474	2756	2.958	4.988	4.333	8.451	3.771	4.386	6.48	9.56	6.66	10
Os01g0929200	13.3	25.1	16.2	20.52	18.98	23.36	36.8	27.8	28.3	17.24	1.096	36.69	18.505	23.336	14.255	0	20.61	20.72	18.58	20.96	31.04	16.27	31.36	39.72	11.62	14.14	35.09	15.7	21	9.16	15
Os01g0918400	191	124	187	34.48	28.02	32.01	18.8	12.7	16.6	169	224.8	143.9	248.18	219.36	265.31	274.6	99.96	119.1	52.02	46.76	43.11	11.09	9.264	16.61	165.8	163.1	214.9	160	147	101	3
Os01g0234433	0	0	0	0	0	0.865	0	0	0	1.724	0	0.941	0	0	0	0	0	0	0.929	0	0	0	0	0	0	0	0	0	0	1.67	7
Os01g0256800	79.5	71.7	80.8	54.18	51.53	77.85	63.4	44.9	51.1	74.15	58.11	56.45	64.223	95.212	60.189	119.4	106.1	80.81	51.09	63.7	55.18	59.15	73.4	57.78	68.66	56.57	92.1	99	81.3	71.6	13
Os01g0272850	3.06	6.52	7.54	362	423.1	365.9	121	166	126	1.724	5.482	0	3.2656	8.4011	7.1277	0	7.214	2.072	200.7	171.7	250.9	110.2	126.1	88.84	2.113	0	6.579	10.2	1.91	10.8	10
Os01g0108600	1110	1005	870	552.5	371.5	619.4	882	1020	1242	530.2	523	465.7	459.36	483.53	416.57	1791	867.7	712.8	806.3	653.9	830.3	777.1	887.2	1081	558.8	672.2	870.6	529	591	546	6
Os01g0290600	17.3	14.9	12.9	11.49	16.27	9.516	9.39	6.33	19.3	12.93	9.868	12.23	2.177	4.6673	3.9598	0	3.092	14.5	14.86	19.35	6.898	19.96	10.69	18.78	11.62	5.657	6.579	4.63	5.74	7.5	9
Os01g0715800	339	288	312	349.7	378.8	367.6	348	314	347	316.4	376.1	296.4	343.97	364.98	346.88	513.5	304	290.1	443.1	447.5	406.1	301.7	313.6	385.7	316.9	305.5	357.4	291	292	269	6
Os01g0720801	34.7	24.2	52.8	29.56	21.7	18.17	54	34.8	47.7	29.31	17.54	32.93	38.098	38.272	35.638	35.82	29.89	32.12	19.51	22.58	29.32	45.1	50.6	30.33	39.09	30.17	21.93	37.9	40.2	26.7	5
Os01g0391100	275	247	274	277.5	289.3	279.4	215	263	227	295.7	234.6	230.5	329.82	284.7	301.74	346.3	244.2	237.3	248	292.7	225	309.8	246.6	213.8	275.7	275.3	280.7	303	287	326	13
Os01g0393400	0	0	0	0.821	0	0	2.35	0	0	0	0	0	0	0	0	0	1.031	0	0	0	0	0	0.713	0	0	0	2.193	0	0	0	11
Os01g0680100	1.02	0	0	0	0	0	0	0	0	0	0	1.882	0	0	0	0	0	0	0	0	0	0.739	0	0	0	0	0	0	0	0	20
Os01g0864700	165	106	137	45.97	64.18	52.77	75.9	75.3	91.9	113.8	93.19	112	117.56	172.69	109.29	322.4	155.6	132.6	85.46	66.92	110.4	77.64	57.72	99.67	112	146.1	166.7	102	101	116	13
Os01g0128200	26.5	31.7	22.6	189.6	167.2	180.8	33.7	34.2	49.8	8.622	20.83	10.35	20.682	20.536	29.303	23.88	12.37	22.79	174.6	173.4	130.2	36.97	58.44	46.95	19.01	16.03	15.35	37	36.3	25.8	10
Os01g0829266	0	0.93	0	4.926	2.712	0.865	7.05	8.86	8.29	3.449	0	0.941	1.0885	1.8669	0	0	5.153	1.036	2.787	0.806	4.311	2.218	1.425	12.28	0	0	0	0	3.83	0	18
Os01g0153600	0	0	0	0	0	0	0	0	0	0.862	0	0	0	0	0	0	0	0	0	0	0	0	0	0	0	0	0	0	0	0	7
Os01g0743200	0	0	0	0	0	0.865	0	0	0	0	0	0	0	0	0.792	0	0	0	0	0	0	0	0	0	0	0	0	0	0	0	4
Os01g0307562	1.02	0	0	0	0	0	0	1.27	1.38	0	0	0	2.177	0	0	0	0	0	0	0	1.724	0.739	0.713	0	0	0.943	0	0	0	0	4
Os01g0383001	0	0.93	0	0	0	0	0	0	0	0.862	0	0	2.177	2.8004	0	0	0	0	0	0	1.724	0	0	0	0	0	0	0	0	0	4
Os01g0274100	38.7	44.7	35.6	13.96	15.37	19.03	0	0	0	26.73	14.25	22.58	21.77	41.072	31.679	35.82	41.22	19.68	18.58	8.869	8.622	0	0	0	25.35	16.03	17.54	80.5	52.6	45	3
Os01g0364900	5.1	5.59	1.08	7.389	5.424	12.98	7.05	1.27	0.69	6.035	6.578	6.586	2.177	0	3.9598	0	2.061	2.072	16.72	8.063	9.484	1.479	2.138	0	2.113	2.829	6.579	0	2.87	4.17	16
Os01g0584250	31.6	26.1	25.9	45.15	26.22	27.68	36	18.3	32.5	46.56	43.86	40.46	22.859	55.074	24.551	35.82	34.01	34.19	42.73	29.03	31.9	25.14	27.79	46.95	42.25	36.77	37.28	46.3	39.2	37.5	20
Os01g0349800	20.4	34.5	24.8	41.87	31.64	54.5	10.2	17.1	18	35.35	18.64	25.4	14.151	27.07	20.591	47.76	16.49	17.61	10.22	11.29	9.484	32.53	29.93	21.67	8.451	10.37	4.386	46.3	23.9	35	8
Os01g0310550	17.3	34.5	31.2	24.63	29.83	36.33	93.2	93.6	85.7	60.35	48.24	110.1	67.488	55.074	49.102	71.65	28.85	36.26	46.45	33.86	50.01	68.02	46.32	77.28	33.8	43.37	57.02	28.7	50.7	38.3	5
Os01g0208700	1937	2222	2348	2060	2172	2023	4340	4516	4512	2753	2862	2983	2750.7	2239.4	2374.3	3690	2307	2604	2619	2504	2723	2881	2530	3447	2321	2473	2085	2043	2179	2255	18
Os01g0515300	291	289	343	116.6	154.6	137.5	122	149	105	376.8	313.6	201.3	171.99	210.03	224.13	609	314.3	337.7	110.5	174.2	144	187.1	211.7	205.1	258.8	333.8	300.4	290	237	245	13
Os01g0771300	61.2	43.8	48.5	45.15	51.53	58.82	121	84.8	144	71.56	76.75	131.7	87.082	48.539	72.861	95.53	54.62	45.59	103.1	57.25	78.46	68.02	59.86	75.84	67.61	72.6	65.79	50.9	52.6	47.5	15
Os01g0223600	173	142	184	181.4	195.3	174.7	132	113	150	150	153.5	162.8	165.46	153.09	123.55	226.9	142.2	131.6	122.6	171.7	150.9	134.6	125.4	177.7	140.5	184.8	201.7	142	166	132	6
Os01g0735632	6.12	13	11.9	18.06	10.85	5.19	25.1	34.8	15.9	12.07	14.25	15.99	9.7967	13.068	7.1277	11.94	12.37	9.324	13.01	24.19	14.66	11.83	21.38	17.33	14.79	6.6	4.386	10.2	6.69	8.33	18
Os01g0116300	38.7	58.7	53.9	43.51	48.81	57.09	54.8	56.3	57.4	52.59	58.11	51.75	34.833	56.941	49.894	71.65	39.16	50.77	42.73	65.31	62.94	64.33	54.87	57.78	58.1	51.86	70.17	72.2	64.1	82.5	1
Os01g0237500	0	0	0	4.926	1.808	1.73	0.78	3.16	0	0.862	1.096	6.586	1.0885	0	1.5839	0	1.031	0	9.29	2.419	0	2.218	1.425	0.722	0	0	0	0	0	0.83	15
Os01g0832600	0	0	0	70.6	9.944	91.7	0	2.53	12.4	0	0	0	0	0	0	0	0	0	30.66	0	3.449	1.479	5.701	30.33	0	0.943	2.193	0	0.96	0	2
Os01g0914800	949	968	1073	1050	1037	1015	1754	1901	1678	837.2	940.7	802.5	834.89	761.7	875.91	1433	1016	990.5	919.7	974	989.8	1403	1564	1495	923.3	837.2	785.1	872	918	877	18
Os01g0725600	393	340	403	377.6	428.5	390.1	460	562	473	386.3	366.2	347.2	451.74	420.99	365.89	609	432.8	392.7	366.9	409.6	374.2	504.3	518.8	457.2	444.7	356.4	348.7	483	449	405	6
Os01g0100500	892	917	866	725.7	772.9	763	795	703	690	906.2	895.8	979.4	916.53	966.12	918.68	1576	907.9	938.6	830.5	806.3	767.4	783	736.2	873.2	937	1020	914.4	926	887	881	13
Os01g0837200	3.06	1.86	5.39	2.463	4.52	3.46	0	1.9	2.07	0	8.771	2.822	4.3541	3.7338	0.792	0	6.183	3.108	0.929	0.806	1.724	4.436	2.851	2.167	1.056	5.657	10.96	4.63	0	4.17	11
Os01g0968800	0	1.86	1.08	0	0	0	0	0	0	0	0	0	1.0885	0	0	0	0	1.036	0	0	0	0	0	0	0	0	0	0	0	0	14
Os01g0690800	658	632	752	277.5	334.5	221.5	698	602	564	983.8	708.3	481.7	667.26	600.21	639.12	823.9	451.4	552.2	234.1	264.5	265.6	555.3	608.6	465.8	761.6	409.2	438.6	597	522	597	7
Os01g0937300	133	99.7	137	171.6	158.2	128	76.7	93.6	86.4	68.11	59.21	85.61	70.754	88.678	60.981	274.6	111.3	132.6	117	116.9	97.43	94.64	99.77	59.95	107.7	66.94	50.44	117	120	111	6
Os01g0197200	556	567	520	587	556.8	623.7	714	651	702	615.6	645.8	686.8	556.23	625.41	619.32	835.9	576.1	549.1	648.4	622.4	758.8	648.4	685.6	675.3	646.5	722.2	721.5	602	521	572	6
Os01g0272901	0	0	0	0	0	0	0	0	0	0	0	0	0	0	0	0	1.031	0	0	0	0	0	0	0	0	0	0	0	0	0	1
Os01g0355100	2.04	1.86	2.16	5.747	6.328	7.785	3.13	4.43	0	2.587	0	1.882	1.0885	0.9335	2.3759	0	3.092	1.036	7.432	3.225	6.036	1.479	4.276	3.611	4.225	4.714	0	0	0.96	1.67	10
Os01g0745700	0	0	0	0	0	0	0	0	0	0	0	0	1.0885	0	0	0	0	0	0	0	0	0	0	0	0	0	0	0	0	0	4
Os01g0729100	322	299	322	281.6	320.9	313.1	384	381	431	374.2	361.8	432.8	392.96	378.98	338.96	477.6	372	327.4	378.1	381.4	335.4	327.5	344.2	315.6	349.7	386.6	372.8	327	348	317	6
Os01g0217800	332	350	316	404.7	326.3	344.3	391	394	390	325.9	336.6	303.9	255.8	286.57	279.56	453.8	316.4	315	375.3	379	364.7	542.7	607.2	564.1	386.6	358.3	379.4	361	333	347	17
Os01g0976900	3.06	1.86	2.16	2.463	3.616	2.595	0	1.9	0	6.035	0	2.822	3.2656	0.9335	0.792	0	1.031	1.036	0.929	0	0.862	0.739	0	1.444	0	3.771	0	0.93	2.87	2.5	7
Os01g0812900	207	190	183	200.3	249.5	277.7	144	95.5	208	222.4	203.9	299.2	189.4	260.43	138.59	238.8	220.5	172	407.8	281.4	400.9	109.4	117.6	120.6	159.5	179.1	344.3	140	165	138	16
Os01g0253100	780	753	718	730.7	706	670.4	701	745	692	728.5	721.4	691.5	789.18	777.57	775.33	1218	838.9	760.5	552.7	612.8	638.9	792.6	786.7	710.7	733.1	824	721.5	870	749	841	13
Os01g0617650	0	0	0	2.463	7.232	5.19	8.61	1.9	2.76	0.862	3.289	0	1.0885	0	0.792	0	0	0	3.716	8.063	6.898	3.697	2.851	5.056	0	0.943	0	0	0	0	12
Os01g0666000	13.3	11.2	11.9	2.463	3.616	4.325	3.13	5.69	10.4	25	31.8	22.58	2.177	10.268	8.7116	35.82	23.7	22.79	15.79	2.419	3.449	14.05	19.95	23.11	20.07	72.6	59.21	13	11.5	13.3	11
Os01g0874300	382	281	219	102.6	67.8	115.1	32.1	43	42.2	62.94	61.4	78.09	95.79	89.879	109.29	203	540	302.5	184.9	133	200	119.8	148.9	294	122.5	138.6	157.9	145	117	160	14
Os01g0744400	19.4	11.2	12.9	18.88	8.136	19.9	31.3	18.3	24.2	18.97	15.35	21.64	8.7082	9.3345	8.7116	35.82	13.4	5.18	17.65	18.54	19.83	9.612	8.552	15.89	8.451	14.14	8.772	12	9.56	4.17	6
Os01g0885200	553	442	571	352.2	352.5	344.3	380	404	377	403.5	385.9	389.5	440.85	426.59	445.08	1003	536.9	494.2	330.7	371.7	363.9	313.5	364.2	396.5	400.4	414.9	495.6	389	476	467	13
Os01g0968600	198	256	237	200.3	225.1	193.8	72.8	74	56	98.29	119.5	43.28	125.18	121.35	148.1	358.2	130.9	195.8	73.39	108	74.15	184.1	202.4	105.4	135.2	94.28	50.44	241	230	261	8
Os01g0258600	92.8	93.1	79.7	107.5	124.7	116.8	90	84.8	104	101.7	83.33	75.27	70.754	117.61	72.069	203	81.41	76.67	119.8	131.4	109.5	76.9	88.37	94.61	50.71	99	94.3	69.4	66.9	80.8	6
Os01g0316800	955	958	962	1013	1100	1106	1300	1139	1269	957.9	964.8	859	832.72	954.92	811.77	1660	876	808.1	1122	1073	1028	1038	1085	1161	890.5	939.1	916.6	965	815	779	6
Os01g0925700	1040	753	1053	609.2	535.1	651.4	1001	1098	990	1191	593.1	503.3	569.3	694.49	895.71	1899	914.1	882.7	785	806.3	929.5	1070	1281	1028	709.9	645.8	785.1	895	1029	777	6
Os01g0511800	4.08	4.66	2.16	2.463	0.904	4.325	0.78	5.06	2.07	2.587	3.289	1.882	2.177	0.9335	0.792	0	5.153	5.18	0	2.419	1.724	3.697	0	5.056	2.113	7.543	8.772	0	1.91	1.67	11
Os01g0719400	59.1	41	42	15.6	18.08	22.49	3.91	6.33	2.76	21.55	26.31	16.93	27.213	63.475	41.974	95.53	39.16	31.08	14.86	12.9	12.93	8.133	0.713	10.11	22.18	16.03	13.16	28.7	32.5	24.2	13
Os01g0747300	305	305	256	290.6	240.5	365.1	25.1	62	43.5	110.4	94.29	96.9	64.223	82.144	108.5	191.1	238.1	194.8	345.6	372.5	564.8	122.7	163.9	148.8	70.78	149.9	228.1	124	149	122	16
Os01g0754700	6.12	11.2	14	101.8	122.9	127.2	43.8	38.6	44.2	5.173	8.771	2.822	15.239	2.8004	3.9598	23.88	5.153	9.324	69.67	83.85	97.43	32.53	37.77	36.11	8.451	2.829	6.579	1.85	7.65	0	10
Os01g0743600	61.2	57.7	34.5	65.68	67.8	74.39	38.4	67	45.6	33.63	31.8	31.99	56.603	47.606	56.23	71.65	49.47	50.77	59.45	49.99	62.08	65.81	62.71	72.95	59.16	56.57	59.21	68.5	64.1	60	2
Os01g0115300	155	198	222	109.2	122	139.3	75.2	111	60.1	150	169.9	83.73	144.77	165.22	203.54	250.8	143.2	150.2	65.03	104.8	68.98	190	175.3	105.4	201.8	169.7	149.1	254	249	262	8
Os01g0750000	11.2	0.93	6.47	14.78	16.27	26.82	4.7	3.8	7.6	4.311	5.482	0.941	6.5311	2.8004	1.5839	11.94	9.275	1.036	8.361	12.9	9.484	4.436	4.276	10.11	2.113	0.943	8.772	5.55	0.96	1.67	10
Os01g0735500	30.6	38.2	28	34.48	37.97	29.41	162	185	135	68.97	42.76	47.98	20.682	39.205	35.638	35.82	21.64	22.79	61.31	42.73	27.59	59.15	81.95	100.4	34.86	17.91	28.51	31.5	25.8	30.8	18
Os01g0147400	0	0	0	0	0	0	0	0	0	0	0	0	0	0	0	0	0	1.036	0	0	0	1.479	0	0	0	0	0	0	0	0	9
Os01g0262700	597	669	636	1345	1483	1371	1163	1424	1291	445.8	609.6	436.5	683.59	559.14	462.51	991.1	441.1	605	1069	1218	1144	1216	912.2	962	479.6	386.6	311.4	445	504	595	12
Os01g0168400	0	5.59	6.47	10.67	23.5	18.17	7.05	6.33	14.5	0.862	3.289	1.882	3.2656	0.9335	0.792	0	3.092	2.072	18.58	33.86	25	4.436	7.839	7.222	7.395	1.886	4.386	2.78	0.96	1.67	10
Os01g0901600	53	22.4	37.7	99.34	62.37	142.7	88.5	67	153	75.87	80.04	35.75	74.019	39.205	43.558	95.53	18.55	15.54	147.7	74.98	79.32	116.1	81.95	82.34	54.93	35.83	160.1	37.9	91.8	60.8	12
Os01g0755700	10.2	3.73	4.31	22.99	24.41	14.71	19.6	20.9	38.7	9.484	9.868	23.52	3.2656	8.4011	7.1277	0	7.214	11.4	20.44	4.031	7.76	75.42	30.64	31.78	7.395	13.2	6.579	6.48	0.96	2.5	9
Os01g0266000	649	594	600	863.7	809.1	682.5	571	639	641	512.1	464.9	572	610.66	533.93	474.39	991.1	474	536.7	748.7	651.5	626	549.4	544.5	534.4	426.8	419.6	425.4	392	423	372	6
Os01g0726400	2.04	2.79	2.16	0	0.904	0.865	2.35	1.27	1.38	3.449	1.096	6.586	0	0	3.1679	0	1.031	1.036	5.574	0.806	1.724	1.479	2.138	0.722	0	0.943	4.386	0.93	1.91	4.17	20
Os01g0629900	176	155	137	173.2	139.2	206.7	209	182	260	219	189.7	208.9	154.57	189.49	232.84	107.5	179.3	181.3	218.3	203.2	269.9	264.7	238.7	291.1	236.6	227.2	274.1	197	193	177	9
Os01g0750500	27.5	23.3	22.6	13.14	6.328	16.44	25.1	61.4	6.91	18.11	15.35	31.05	14.151	12.135	9.5036	11.94	10.31	24.86	5.574	4.031	4.311	22.92	19.95	21.67	13.73	6.6	8.772	10.2	3.83	10.8	5
Os01g0649732	0	0	0	0	0	0	0	0	0	0	0	0	0	0	0	0	0	0	0	0	0	0.739	0	0	0	0	0	0	0	0	9
Os01g0971800	0	2.79	6.47	4475	4701	4927	875	1061	989	8.622	6.578	10.35	7.6196	14.935	7.1277	0	7.214	4.144	6856	6941	7534	961.9	1194	998.8	5.282	7.543	2.193	5.55	8.61	7.5	10
Os01g0873100	237	169	183	31.2	35.25	40.66	265	252	277	269.9	280.7	702.8	283.02	219.36	224.13	226.9	367.9	260	118	104	102.6	109.4	97.63	207.3	316.9	399.8	473.7	139	143	154	20
Os01g0850200	171	109	134	280	304.6	260.4	51.7	56.3	47.7	93.98	99.77	112.9	109.94	98.946	101.37	262.7	160.8	143	353.9	302.4	400.9	61.37	71.98	65.72	124.7	114.1	109.6	142	141	151	16
Os01g0263500	262	265	284	270.9	232.3	257.8	205	218	231	267.3	239	213.6	365.74	316.44	327.87	441.8	301.9	298.4	271.3	279	294.9	263.2	223.8	236.2	288.4	296.1	232.4	289	277	290	13
Os01g0506100	125	112	145	128.1	104.9	126.3	79.9	79.7	87.1	108.6	112.9	123.2	140.42	110.15	125.92	406	121.6	141.9	131	158.8	169	100.6	106.9	91.73	126.8	114.1	100.9	112	143	109	6
Os01g0303200	202	181	198	257	246.8	251.7	249	196	229	179.3	187.5	145.8	133.89	171.76	206.7	250.8	182.4	194.8	217.4	221.7	207.8	247.7	254.4	256.4	172.2	194.2	164.5	209	186	184	12
Os01g0974701	81.6	85.7	65.7	83.74	65.99	71.8	73.6	74.6	95.4	62.08	71.27	57.39	59.869	75.61	60.189	131.4	53.59	55.95	77.1	79.82	56.04	51.02	43.47	88.11	59.16	51.86	78.95	26.8	41.1	35.8	6
Os01g0124401	0	0	0	0	0	0.865	0	0	0	0	0	0	0	0	0	0	0	0	0	0.806	0.862	0	2.138	0	0	0	0	0	0	0	17
Os01g0215500	0	0	1.08	1.642	0.904	3.46	3.91	1.9	0.69	0	1.096	0	0	0	0	0	0	0	0.929	8.063	2.587	0.739	0	0.722	0	0	0	1.85	0.96	0	15
Os01g0881300	557	799	772	511.5	611.1	499.1	1351	1168	1222	541.5	720.3	597.4	672.71	685.15	555.96	1242	682.2	514.9	397.6	390.2	532	664.7	488.9	971.4	582.1	604.4	489	432	498	522	18
Os01g0866300	1255	962	1204	736.4	705.1	781.1	1340	1323	1370	1186	992.1	1115	1063.5	1189.2	1129.3	1982	1107	1095	1000	773.2	855.3	1037	1051	1041	1022	948.5	1092	960	862	829	6
Os01g0969000	140	139	155	424.4	485.4	352.1	548	424	377	150.9	112.9	157.1	155.66	118.55	122.75	203	105.1	128.5	390.2	445.1	288	258.8	260.8	276.6	120.4	117.9	131.6	120	138	136	12
Os01g0807266	1.02	8.38	2.16	1.642	2.712	3.46	2.35	1.9	1.38	1.724	1.096	0.941	4.3541	7.4676	5.5438	0	7.214	5.18	4.645	6.45	2.587	2.218	3.563	2.889	4.225	3.771	0	4.63	3.83	1.67	4
Os01g0249900	6.12	2.79	3.23	2.463	3.616	1.73	0	1.9	0	4.311	0	1.882	1.0885	3.7338	2.3759	0	2.061	5.18	3.716	3.225	3.449	0.739	4.276	5.056	1.056	2.829	13.16	1.85	1.91	1.67	11
Os01g0921000	0	0.93	0	0.821	0	0	0.78	0	0	0	1.096	0	1.0885	0	0	0	0	0	0	0	0	0	3.563	0	1.056	0	0	0	0	0	17
Os01g0680650	0	0	1.08	7.389	0	5.19	3.91	5.06	7.6	2.587	6.578	0	1.0885	3.7338	3.1679	23.88	3.092	3.108	2.787	0.806	7.76	4.436	6.414	10.83	4.225	0.943	0	1.85	2.87	0	6
Os01g0905700	476	565	623	663.3	678.9	757.8	536	616	503	775.1	698.4	800.6	791.35	822.37	834.73	764.2	550.3	696.2	628.9	579.7	603.6	556.8	516.7	547.5	844	811.8	688.6	730	602	800	4
Os01g0117300	103	331	318	221.7	307.3	181.7	1548	2034	962	1114	744.5	198.5	426.7	436.86	723.06	322.4	143.2	173	93.82	37.9	44.84	430.3	559.4	275.2	237.7	125.4	186.4	298	198	243	5
Os01g0795200	2.04	9.31	6.47	13.96	25.31	6.92	6.26	8.22	5.53	0.862	0	0	1.0885	0	5.5438	0	1.031	5.18	6.503	4.838	0.862	6.654	13.54	5.778	0	1.886	4.386	6.48	3.83	1.67	2

Os01g0650800	93.8	157	140	145.3	175.4	148.8	43.1	50.6	51.8	50.87	78.94	32.93	74.019	58.807	101.37	286.6	86.56	82.88	29.73	45.96	50.01	160.4	129.7	71.5	87.68	49.97	59.21	117	90.9	162	8
Os01g0908600	9.18	10.2	0	0	1.808	3.46	0.78	3.16	0	6.897	3.289	19.76	13.062	3.7338	3.1679	23.88	9.275	5.18	4.645	0	0.862	0.739	0.713	3.611	2.113	7.543	8.772	3.7	11.5	5.83	13
Os01g0871733	0	0	0	1.642	0	1.73	0	0	0	0	0	0	0	0	0	0	0	0	0	0	0	0.739	0.713	0	0	0	0	0	0	0	2
Os01g0706600	12.2	10.2	7.54	21.35	15.37	20.76	16.4	15.2	18	13.79	13.16	8.467	7.6196	10.268	10.296	11.94	13.4	20.72	31.58	14.51	15.52	5.176	14.25	9.389	5.282	8.486	19.74	6.48	5.74	12.5	10
Os01g0851100	98.9	77.3	110	28.73	37.06	31.14	50.9	37.3	49.1	41.38	35.08	20.7	30.479	30.804	41.974	47.76	31.95	64.23	5.574	23.38	15.52	56.93	34.92	43.33	22.18	23.57	30.7	44.4	40.2	42.5	14
Os01g0612200	407	363	425	331.7	333.6	434.3	405	326	337	613.9	599.7	671.7	525.76	581.54	484.68	931.4	512.2	534.6	516.5	560.4	475.1	399.3	366.3	429.7	502.8	648.7	675.4	537	512	482	13
Os01g0124000	89.7	12.1	4.31	4194	60.57	6712	489	154	7449	910.5	753.2	370.7	301.52	75.61	46.726	11.94	17.52	9.324	3852	874.8	3100	1975	3389	2959	608.5	1500	4726	25	643	90	12
Os01g0840100	3699	1732	3709	199.5	163.6	253.5	83	104	40.8	3607	3303	3333	2534.1	1988.3	2868.5	1552	1081	1248	408.7	311.2	248.3	49.54	64.85	51.28	2758	1840	2239	1593	1282	1106	3
Os01g0711600	121	122	148	127.3	161.8	117.6	170	159	211	111.2	191.9	103.5	179.61	112.01	126.71	143.3	149.4	132.6	117	133	143.1	295.8	229.5	210.9	166.9	138.6	118.4	170	164	157	17
Os01g0239700	702	717	700	1364	1209	1267	2031	1547	2519	1232	1181	1310	757.61	820.5	719.11	597	420.5	444.5	1365	1044	1229	2257	1632	2058	875.7	834.4	1046	623	693	697	18
Os01g0625200	26.5	17.7	20.5	4.105	4.52	15.57	11.7	18.3	9.67	9.484	13.16	21.64	8.7082	14.935	7.9197	11.94	62.86	34.19	6.503	8.869	16.38	14.05	11.4	33.22	7.395	25.46	24.12	15.7	18.2	8.33	14
Os01g0636100	5.1	0.93	5.39	4.105	7.232	3.46	6.26	0.63	2.07	2.587	2.193	4.704	6.5311	8.4011	8.7116	0	2.061	3.108	8.361	9.675	2.587	0.739	1.425	4.333	3.169	0.943	4.386	0.93	4.78	4.17	4
Os01g0542000	355	318	337	667.5	702.4	690.3	366	401	318	380.2	260.9	384.8	355.95	439.66	267.68	322.4	377.2	344	927.1	740.2	931.2	198.9	245.9	276.6	356	290.4	304.8	338	286	341	10
Os01g0377500	740	757	765	864.5	779.2	780.3	788	778	734	819.9	849.7	750.8	930.68	865.31	1031.1	1409	890.4	721.1	809.1	794.2	697.5	670.6	664.9	699.9	855.7	829.7	721.5	757	894	862	13
Os01g0959900	2018	1983	2078	2768	2537	2692	5401	5180	5619	2111	2348	2870	2479.6	2181.5	2014.8	2914	2060	2026	3293	3350	3685	3824	3367	5217	2337	2577	2311	1811	1983	1925	18
Os01g0307686	57.1	28.9	61.4	46.8	13.56	38.93	10.2	50.6	115	17.24	14.25	15.05	17.416	7.4676	32.471	47.76	31.95	11.4	28.8	33.86	8.622	48.06	82.67	35.39	17.96	13.2	39.47	31.5	37.3	16.7	17
Os01g0660400	0	0	0	0.821	0	0	0	0	0	0	0	0	0	0	0	0	0	0	0	2.419	0	0	0	0	0	0	0	0	0	0	16
Os01g0527700	360	371	401	359.6	379.7	362.5	550	475	522	473.3	495.6	511.8	489.83	488.2	451.42	597	356.6	344	446.8	365.2	391.4	437.7	441.1	486.8	445.8	398.8	368.4	359	364	332	5
Os01g0148500	154	134	135	110	153.7	144.5	97.1	90.4	115	126.7	133.8	122.3	128.45	163.35	99.788	131.4	79.35	118.1	115.2	59.66	104.3	113.1	94.78	93.89	79.23	87.68	87.72	72.2	76.5	71.6	19
Os01g0673900	193	224	195	179	163.6	155.7	193	192	173	256.9	311.4	262.5	305.87	252.97	254.22	286.6	287.5	227.9	203.4	219.3	206.1	167.1	173.9	170.4	278.9	260.2	241.2	260	267	242	3
Os01g0210900	0	0	0	0	0	0	0	0	0	0	0	0	0	0	0	0	0	0	0	0	0	0.739	0.713	0	0	0	0	0	0	0	17
Os01g0575500	1078	1170	1032	1162	1200	1114	1026	953	1034	1017	980.2	987.9	1054.8	948.39	1000.3	2066	1054	997.7	872.3	902.2	884.6	1242	1247	1158	1035	928.7	1000	1083	1038	1132	6
Os01g0763800	1.02	0	2.16	0	0	0	1.57	0.63	0.69	2.587	0	1.882	0	0	0	0	1.031	0	0.929	0	0	0.739	0.713	2.889	0	0.943	0	0.93	1.91	0	9
Os01g0835900	605	540	626	837.4	883.2	727.5	973	1022	831	788	687.4	752.7	610.66	570.34	527.45	871.7	616.3	686.9	1191	980.4	1048	569.3	651.3	695.5	564.1	580.8	620.6	537	527	622	12
Os01g0233100	0	0	0	0.821	4.52	1.73	2.35	0	0	0	0	1.882	0	0	0.792	0	3.092	0	0	3.225	0.862	0	0	3.611	3.169	0	0	0.93	0.96	4.17	2
Os01g0553600	904	971	1081	922.8	1033	949	1145	1194	1072	872.5	997.7	902.2	1070	979.19	989.17	2555	1028	998.7	895.5	1028	836.4	1190	898.6	953.4	981.4	882.5	771.9	1092	1235	1247	6
Os01g0815800	1003	711	764	917.8	894.9	955.9	1138	911	1212	1049	893.6	1603	984.02	1195.8	822.85	895.6	799.7	834	1272	1158	1116	827.4	892.2	851.5	900	1043	1281	735	786	558	15
Os01g0259300	142	106	140	44.33	45.2	35.47	29	17.7	18.7	204.3	112.9	269.1	57.692	122.28	64.941	155.2	111.3	108.8	79.89	62.08	62.08	88.73	68.41	49.11	150	245.1	230.3	129	104	111	20
Os01g0660800	1.02	0	0	7.389	9.944	10.38	9.39	1.27	3.46	0.862	2.193	0	0	0.9335	0	0	0	0	7.432	16.13	6.036	2.218	2.138	1.444	2.113	0	0	0	0	0	10
Os01g0103900	9.18	8.38	11.9	11.49	6.328	9.516	7.83	3.16	15.9	5.173	3.289	8.467	4.3541	12.135	6.3357	0	7.214	7.252	3.716	9.675	3.449	14.79	12.83	12.28	17.96	12.26	15.35	11.1	3.83	19.2	1
Os01g0173100	568	524	596	413	447.5	471.5	537	552	493	482.8	500	568.3	600.86	601.14	569.42	1003	656.4	556.4	571.3	512.8	493.2	411.8	453.2	465.1	518.7	593	631.6	523	531	533	13
Os01g0783200	1621	1569	1655	981.9	888.6	1003	535	718	636	2165	1931	1981	1908.2	2048.9	2109.8	4442	2273	2037	1447	1525	1679	647	653.5	608.9	2837	2198	2215	2573	2163	2345	13
Os01g0136200	1.02	0	1.08	0	2.712	0	0	0	0	2.587	0	0.941	3.2656	0	0	0	0	0	0	0	0	0	0	0	0	0	0	0	0	0	7
Os01g0921500	4.08	2.79	0	0.821	3.616	0.865	0	0	0	2.587	0	0.941	1.0885	0.9335	0.792	0	1.031	0	0.929	2.419	0	0.739	0	0.722	2.113	0.943	2.193	0	0.96	0.83	19
Os01g0910300	50	26.1	46.3	36.94	32.54	31.14	58.7	41.7	47	33.63	55.92	49.86	57.692	41.072	44.35	95.53	45.34	52.84	40.87	39.51	31.04	56.19	51.31	43.33	41.2	44.31	41.67	63.8	50.7	57.5	6
Os01g0620800	0	0	0	1.642	0	0.865	0	0	0	1.724	0	0	0	0	0	0	0	0	0	0	1.724	0	0.713	0.722	0	2.829	2.193	0	1.91	0.83	11
Os01g0936600	53	70.8	61.4	37.76	29.83	33.74	79.1	38	37.3	114.7	89.9	86.56	57.692	55.074	41.974	35.82	42.25	19.68	24.15	31.44	24.14	56.93	40.62	53.45	40.14	31.11	72.37	29.6	42.1	29.2	7
Os01g0233800	0	0	0	0	0.904	3.46	0.78	1.27	0	0	3.289	0	0	0	0	0	0	0	0.929	0	0.862	1.479	1.425	0.722	0	0	2.193	1.85	0	0.83	2
Os01g0558300	959	808	806	793.9	875.9	764.7	743	780	765	813.9	873.8	908.8	766.32	859.71	788.01	1325	869.8	868.2	791.5	824	783.8	846.6	829.5	835.6	906.4	882.5	859.6	811	793	808	6
Os01g0192900	0	0	1.08	0	1.808	4.325	43.1	0	258	0	0	0	1.0885	0	0.792	0	0	0	0.929	0	0	712.8	301.4	537.4	0	0	0	0	0	0	9
Os01g0893400	1769	1239	1657	1088	1165	1249	1214	1401	1362	1245	1203	1374	1330.2	1653.1	1635.4	3391	1915	2099	1641	1807	1597	1616	1307	1520	1501	1638	1375	1900	1853	1905	13
Os01g0531300	0	0.93	0	0	0	0	0.78	0	0	0.862	1.096	0	0	0	0.792	0	1.031	1.036	0	0	0	0	0.713	0	0	0.943	0	0	0	0	7
Os01g0923400	1073	959	1173	756.1	857.9	865.9	600	749	644	955.3	1152	1086	1234.4	1252.7	1187.2	2400	1472	1304	924.3	1097	1006	946.4	806	676	1139	1375	1278	1354	1352	1257	13
Os01g0689800	316	339	345	490.9	452.9	471.5	799	665	770	491.4	491.2	502.4	455	460.19	380.14	573.2	390.6	368.8	546.2	595.8	649.2	529.4	511	638.5	462.7	470.5	467.1	430	369	353	18
Os01g0692700	804	622	724	933.4	900.4	1165	876	965	890	719.9	682	684.9	857.75	912.92	814.93	1755	800.7	786.4	851.9	773.2	917.4	576	639.9	649.3	760.6	762.8	745.6	754	813	826	6
Os01g0960800	266	238	272	312.8	318.2	322.7	189	197	208	274.2	246.7	288.8	403.84	351.91	322.33	704.5	368.9	303.6	391.1	399.9	347.5	186.3	221.6	190	274.7	244.2	276.3	294	281	291	6
Os01g0105800	302	244	287	162.6	175.4	187.7	156	154	152	321.6	274.1	334.9	466.98	462.06	437.96	417.9	285.5	281.8	202.5	172.5	186.2	172.3	101.2	140.8	326.4	245.1	269.7	315	265	327	3
Os01g0835800	94.8	87.6	106	88.67	84.97	118.5	100	104	135	69.84	83.33	63.98	94.701	94.279	77.613	119.4	127.8	132.6	98.47	110.5	94.84	93.9	126.8	124.9	97.19	92.4	127.2	105	100	78.3	9
Os01g0935600	1573	1680	1706	1119	1239	1010	1189	1192	1054	1519	1742	1730	2187.9	1841.7	2017.1	2914	1935	1769	1096	1117	1175	842.9	912.9	931	1797	1738	1537	1904	1755	1885	13
Os01g0111800	23.4	34.5	31.2	27.09	38.87	13.84	67.3	31	34.6	27.59	19.74	35.75	26.124	31.737	22.967	11.94	29.89	17.61	22.29	23.38	13.8	41.41	38.48	52.72	17.96	22.63	28.51	23.1	21	27.5	18
Os01g0368700	5.1	7.45	7.54	13.14	12.66	28.55	48.5	79.7	81.5	25	12.06	27.28	11.974	25.203	15.047	0	9.275	6.216	11.15	19.35	21.56	47.32	42.05	52.72	26.41	25.46	26.32	23.1	13.4	18.3	18
Os01g0710000	1414	1366	1428	1636	1705	1707	1849	1639	1611	1385	1262	1326	1301.9	1464.6	1394.7	2066	1446	1434	1833	1545	1848	1364	1467	1444	1333	1451	1476	1254	1295	1266	6
Os01g0507900	1275	1502	1447	2554	3144	2500	2721	2682	2407	1217	1486	1295	1652.4	1540.2	1215.7	1839	1088	1304	2424	2626	2269	1828	1862	2116	1124	855.2	929.8	1001	979	1234	12
Os01g0835651	2.04	0	0	0	0	0	0	0	0	1.724	0	0	0	0	0	0	0	0	0	0	0	0	0	0.722	0	0	0	0	0	0	19
Os01g0861400	0	0	0	0	0.904	0	0.78	0	0	0	0	0	0	0	0	0	0	0	0	0	0	0	0	0	0	0	0	0	0	0	2
Os01g0153300	1377	1195	1426	1369	1481	1217	1560	1613	1546	1635	1626	1642	1151.7	967.06	1156.3	2734	1427	1323	1776	1521	1686	930.9	1238	1096	1255	1321	1278	1101	1134	1256	6
Os01g0822900	2.04	0	0	0	0	0	0	0	0	0	0	0	0	0	0	0	1.031	1.036	0	0	0	0	0	0	0	0	0	0	0	0.83	19
Os01g0257300	1.02	0.93	5.39	1.642	0	6.055	0	0	0	6.035	1.096	5.645	3.2656	10.268	0	0	1.031	0	1.858	3.225	0	0.739	3.563	0.722	1.056	5.657	4.386	0.93	0	3.33	20
Os01g0621200	12.2	11.2	8.62	8.21	24.41	16.44	29	31.6	18	5.173	6.578	4.704	6.5311	8.4011	0	23.88	14.43	10.36	15.79	3.225	9.484	17.75	6.414	24.56	1.056	2.829	6.579	6.48	3.83	2.5	18
Os01g0587400	73.4	56.8	94.8	105.1	115.7	100.3	47.8	62	44.9	95.7	58.11	89.38	85.993	64.408	86.324	203	105.1	84.96	137.5	111.3	105.2	68.76	76.96	54.17	77.11	35.83	41.67	108	96.6	107	6
Os01g0130200	48.9	77.3	108	12.31	7.232	7.785	18.8	19	30.4	143.1	167.7	142.1	51.16	70.942	63.357	441.8	119.5	141.9	17.65	8.063	25.87	2.218	4.276	6.5	155.3	166.9	140.3	111	76.5	89.1	13
Os01g0268700	0	0	0	0	0	0	0	0	0	0	0	0	1.0885	0	0	0	0	0	0	0	0	0	0	0	0	0	0	0	0	0	4
Os01g0658400	956	864	903	191.3	178.1	224.9	310	307	402	926	1003	1055	1023.2	1136	1062	1445	957.4	901.4	261	295.9	292.3	355.6	379.8	433.3	953.9	930.6	1083	915	872	891	3
Os01g0193600	134	113	133	260.2	248.6	250	254	238	287	135.4	163.4	229.6	187.23	226.83	160.77	310.5	170	177.2	471	458	507.8	195.9	203.8	223.9	140.5	149.9	221.5	139	159	147	10
Os01g0800200	0	0	0	0	0	0	0	1.27	0	0	0	0	0	0	0	0	0	0	0	0	0	0	0	0	0	0	0	0	0	0	18
Os01g0616900	4.08	5.59	7.54	10.67	16.27	14.71	1.57	1.9	2.07	2.587	5.482	8.467	5.4426	2.8004	7.9197	0	5.153	4.144	3.716	7.257	5.173	17.01	7.839	1.444	9.507	3.771	0	4.63	8.61	14.2	2
Os01g0719300	20.4	55	40.9	98.52	117.5	19.03	46.2	68.3	37.3	102.6	121.7	8.467	62.046	47.606	161.56	262.7	37.1	192.7	177.4	122.6	91.4	69.5	96.92	43.33	183.8	21.69	37.28	154	431	284	1
Os01g0895600	7112	6863	7761	10421	11097	10679	####	9407	9099	11348	8220	8134	8423	8902.3	8376.6	12765	5545	5559	11128	10714	8993	7015	8245	7603	8980	7171	7335	6182	5897	5963	10
Os01g0358800	1.02	0	0	0	0	0	0.78	1.27	2.76	6.897	0	0.941	4.3541	0.9335	0.792	0	2.061	0	0	0	0.862	0.739	0	2.167	0	0.943	0	0	0.96	2.5	7
Os01g0512200	180	216	182	322.6	314.6	348.6	391	411	429	194	282.9	185.3	217.7	189.49	206.7	406	199.9	220.7	287	281.4	275	345.3	328.5	397.2	215.5	200.8	223.7	175	199	201	18
Os01g0194300	325	324	490	340.7	313.7	360.7	705	680	717	644.1	456.1	230.5	560.59	476.06	549.62	812	196.8	278.7	347.4	366.1	223.3	884.3	870.1	941.8	398.2	282.9	247.8	348	328	399	17
Os01g0787600	5.1	9.31	7.54	6.568	3.616	2.595	4.7	1.27	12.4	6.897	1.096	5.645	1.0885	5.6007	1.5839	0	4.122	4.144	5.574	1.613	4.311	13.31	28.51	13.72	2.113	5.657	6.579	0.93	6.69	4.17	17
Os01g0805100	42.8	37.3	38.8	46.8	51.53	44.12	59.5	48.7	42.8	50.01	51.53	42.34	48.983	50.406	36.43	11.94	40.19	33.15	62.24	38.7	47.42	45.1	34.21	58.5	29.58	40.54	63.59	30.5	41.1	45	15
Os01g0556201	1.02	0	0	0	0.904	0	0.78	0	0	0	0	0	0	0	0	0	0	0	0	0	0	0	1.425	1.444	0	0	0	0	0	0	17
Os01g0231800	396	300	323	284.1	327.2	340	227	142	120	336.3	260.9	567.3	256.89	325.77	189.28	441.8	252.5	317	350.2	345.1	367.3	168.6	148.9	210.2	220.8	312.1	359.6	278	241	224	20
Os01g0566701	2.04	0.93	0	1.642	0	0	10.2	0.63	4.15	0.862	2.193	6.586	0	1.8669	1.5839	0	1.031	3.108	1.858	0	0	17.01	1.425	16.61	4.225	2.829	4.386	0.93	1.91	0	9
Os01g0765500	2.04	1.86	0	2.463	1.808	1.73	0	0	0.69	3.449	0	1.882	0	0	0	0	1.031	2.072	0.929	2.419	0.862	1.479	0.713	0.722	0	1.886	4.386	1.85	0	0.83	11
Os01g0150700	0	0	0	0	0	0	0	0	0.69	0	0	0	0	0	0	0	0	0	0	0	0	0	0	0	0	0	0	0	0	0	18
Os01g0309800	1174	1620	1275	3292	2953	3856	4809	5403	5276	3115	2833	3902	2823.6	2732.2	2827.3	2508	2070	2150	5515	4262	4986	4191	3383	4413	3467	4276	3603	2535	2552	2654	15
Os01g0641700	1316	1195	1332	403.9	424.9	312.3	550	591	501	1169	1366	1092	1225.7	1473	1423.2	2364	1625	1421	471.9	419.3	464.7	492.4	538	470.9	1328	1360	1406	1352	1334	1440	13
Os01g0667600	274	302	212	52.54	56.05	63.15	182	187	169	100	62.49	80.91	75.108	84.944	76.029	95.53	225.7	194.8	81.75	74.98	119	113.1	114	183.5	55.99	51.86	50.44	56.4	53.6	65.8	14
Os01g0931300	290	382	343	739.7	702.4	586.5	239	280	205	450.9	385.9	299.2	272.13	307.11	338.17	513.5	250.4	265.2	575	524.9	576	252.9	280.8	242	314.8	297.9	280.7	288	313	284	10
Os01g0614900	591	798	665	740.5	640	774.2	809	862	873	744.1	832.2	713.1	831.63	746.76	907.59	1743	759.5	709.7	682.8	649.9	694.1	986.3	847.3	827	868.3	842	798.2	833	768	849	6
Os01g0916300	417	398	406	402.3	425.8	394.5	438	409	413	399.2	369.5	360.3	440.85	433.12	453	537.3	441.1	427.9	455.2	437.8	444.9	391.1	372.7	405.2	451.1	454.4	436.4	469	397	472	13
Os01g0500825	67.3	66.1	80.8	50.08	54.24	63.15	13.3	25.3	13.8	61.22	70.17	75.27	67.488	61.608	65.733	83.59	59.77	61.13	26.94	54.02	46.56	36.97	34.92	26.72	58.1	50.91	70.17	77.7	72.7	106	3
Os01g0661750	0	0	0	0	0	2.595	0	0	0.69	0	0	0	0	0	0	0	0	0	0	0	0	0	0.713	0.722	1.056	0	0	0	0	0.83	2
Os01g0694200	290	268	275	369.4	301.9	327	260	245	312	371.6	383.7	280.4	383.16	322.97	409.45	501.5	306.1	347.1	275	361.2	311.3	365.3	357.7	353.9	362.3	381.9	291.7	373	387	392	13
Os01g0970900	106	102	94.8	281.6	345.3	316.6	75.9	105	93.3	76.73	72.36	88.44	119.74	127.88	76.029	71.65	103.1	92.21	307.5	248.3	297.5	91.68	121.1	96.06	51.76	99	54.82	110	75.5	97.5	10
Os01g0771100	109	82.9	103	227.4	245	232.7	54.8	51.9	64.3	99.15	109.6	118.5	104.5	121.35	93.452	131.4	102	118.1	275	260.4	260.4	87.25	73.4	72.95	90.85	101.8	140.3	90.7	97.5	108	10
Os01g0109750	157	205	202	239.7	247.7	224	289	287	280	205.2	189.7	192.9	165.46	145.62	148.1	203	187.6	163.7	209	203.2	200.9	261	233.7	303.3	169	172.5	173.2	159	171	172	18
Os01g0546700	24.5	33.5	46.3	50.9	59.66	57.96	14.1	19	13.1	32.76	37.28	24.46	30.479	37.338	30.887	59.7	24.73	19.68	24.15	34.67	41.39	37.71	27.79	36.83	35.92	32.06	28.51	43.5	18.2	39.2	2
Os01g0610300	831	808	909	941.7	952.8	891.9	483	610	490	588	641.4	620	870.82	661.82	775.33	1254	766.7	865.1	753.4	988.5	841.5	469.5	427.6	444.2	715.2	574.2	594.3	767	847	841	8
Os01g0189800	417	465	459	733.1	681.6	640.1	1156	1023	1045	638.9	637	617.2	645.49	617.01	639.91	907.5	428.7	439.3	721.8	620.8	621.7	803	717.6	724.4	588.4	444.1	539.5	544	490	515	18
Os01g0921550	0	0.93	0	0	0	0.865	0	0	0	0	0	0	0	0	0	0	2.061	0	0	0	0	0	0	0	0	0	0	0	0	0	14
Os01g0906600	652	656	639	624.8	691.5	640.1	528	491	485	513	591	417.7	697.74	636.61	695.35	1218	678.1	622.7	521.1	567.6	515.6	711.3	618.6	496.2	654.9	648.7	611.8	742	718	793	13
Os01g0843700	7.14	0.93	2.16	0	2.712	0	0	0	0	0	0	1.882	3.2656	0	0.792	0	3.092	4.144	0	0	0	0	0	0	2.113	5.657	4.386	0	2.87	1.67	19
Os01g0219100	77.5	54	56	59.93	69.61	71.8	63.4	71.5	65.6	59.49	76.75	63.03	42.452	60.674	57.814	47.76	79.35	45.59	73.39	80.63	83.64	61.37	61.29	66.45	70.78	52.8	59.21	39.8	47.8	51.7	15
Os01g0508500	173	230	240	32.84	26.22	49.31	47.8	18.3	178	303.5	372.8	258.7	176.34	392.05	183.74	549.3	308.1	230	57.6	41.12	72.43	12.57	25.65	24.56	268.3	300.8	269.7	154	158	148	13
Os01g0782100	1.02	0	1.08	0.821	0.904	0.865	0	0	0	0	1.096	0	0	2.8004	0	0	0	2.072	0	0	0.862	0.739	2.138	0	0	0.943	0	0	0.96	0.83	1
Os01g0951800	0	0	0	0	0.904	0	0	0	0	0.862	0	0	0	0	0	0	0	0	0	1.613	0	0	0	0	0	0	0	2.78	0	0	1
Os01g0555600	16.3	26.1	30.2	50.9	48.81	55.36	52.5	56.3	37.3	24.14	33.99	15.99	20.682	18.669	13.463	0	14.43	16.58	52.95	64.5	52.6	52.5	22.09	56.34	8.451	6.6	6.579	9.25	21	10.8	12
Os01g0702400	576	633	672	724.9	659.9	731.8	424	486	452	457.8	433.1	392.3	571.47	534.87	531.41	991.1	502.9	595.7	539.7	720.8	599.2	607	601.5	530.1	456.3	463.9	364	529	596	597	6
Os01g0632700	389	364	403	370.3	348	303.6	379	405	314	325.9	316.9	299.2	384.25	336.04	350.84	764.2	421.5	358.5	352.1	386.2	394	357.9	372.7	395.8	396.1	395.1	381.6	405	415	442	6
Os01g0136300	80.5	60.5	59.3	73.89	56.95	60.55	43.8	17.7	32.5	60.35	49.34	94.08	48.983	59.741	22.967	35.82	54.62	44.55	57.6	36.28	77.6	42.15	18.53	68.61	25.35	42.43	46.05	31.5	31.6	40	19
Os01g0194000	1116	1217	721	131.4	131.1	158.3	168	142	138	344	299.3	414	204.64	219.36	209.87	453.8	613.2	580.2	177.4	191.1	208.7	176	197.4	248.5	293.7	370.5	462.7	278	340	325	14
Os01g0531500	1696	1636	1607	1488	1472	1504	1516	1331	1419	1727	1492	2166	1409.6	1806.2	1356.6	2054	1740	1679	1634	1302	1724	991.5	1159	1253	1616	1949	2044	1447	1449	1326	20
Os01g0133832	2.04	0	0	0	0	0.865	0	0	0	1.724	0	0	1.0885	0	3.1679	0	4.122	0	0	0	0	0	0.713	0.722	1.056	0	0	0	0.96	3.33	1
Os01g0831250	6.12	11.2	8.62	8.21	1.808	1.73	8.61	17.7	9.67	6.897	5.482	0.941	1.0885	0.9335	3.1679	0	10.31	5.18	5.574	4.031	3.449	3.697	19.95	5.778	4.225	0	2.193	8.33	3.83	0.83	17
Os01g0653700	205	246	197	360.4	357.1	314	407	434	423	231.9	213.8	221.1	278.66	231.5	283.52	346.3	221.6	204.1	311.2	328.2	290.6	309.8	333.5	366.9	249.3	206.5	190.8	250	246	241	12
Os01g0721000	105	82	103	82.92	74.13	77.85	218	154	232	127.6	140.3	118.5	108.85	120.42	87.908	179.1	115.4	84.96	94.75	105.6	79.32	173.8	222.3	190.7	135.2	126.3	120.6	158	141	137	5
Os01g0220400	553	586	668	563.2	440.2	430.8	870	614	665	1570	1134	1239	981.84	766.36	850.57	1731	828.5	862	915.9	649.9	757	369.7	546.6	437	1009	829.7	1059	894	985	764	13
Os01g0952200	652	764	664	889.9	875	820.9	527	574	501	657.8	660	562.6	696.65	564.74	682.67	1469	677.1	651.7	705.1	831.3	728.6	635.1	657	554	625.4	574.2	541.7	652	756	749	6
Os01g0856500	115	93.1	52.8	59.11	44.29	52.77	97.1	72.7	47	128.5	81.13	202.3	46.806	62.541	42.766	35.82	124.7	92.21	68.74	33.86	66.39	35.49	22.09	36.83	70.78	144.3	171	56.4	65	49.2	20
Os01g0765400	16.3	24.2	14	32.84	32.54	30.28	68.9	34.8	31.1	22.42	17.54	15.99	14.151	10.268	12.671	35.82	8.244	7.252	36.23	54.83	37.94	36.23	39.91	33.95	11.62	18.86	8.772	12	14.3	15	12
Os01g0689600	200	189	154	197	185.3	188.6	170	185	174	151.7	208.3	143.9	190.49	230.56	189.28	358.2	161.8	182.3	177.4	212.1	183.7	240.3	208.1	190	198.6	168.8	203.9	167	181	236	6
Os01g0694800	2.04	7.45	0	4.926	1.808	4.325	15.7	13.9	14.5	3.449	7.675	8.467	1.0885	1.8669	3.9598	11.94	2.061	3.108	3.716	0	5.173	5.176	2.851	6.5	0	8.486	4.386	0.93	1.91	3.33	18
Os01g0874800	467	596	504	1358	1340	1112	1144	1152	1130	776.8	874.9	925.8	626.99	490.06	462.51	597	308.1	455.9	1015	1263	1036	1018	836.6	931	657.1	501.6	418.8	290	326	353	12

Os01g0866400	####	####	####	2203	1919	2278	####	####	####	9087	10678	9203	12221	10432	10775	15117	10972	10759	3754	2725	3261	15581	13869	18875	11193	10160	9910	9896	####	####	5
Os01g0747600	10.2	4.66	11.9	32.02	30.73	23.36	36	31	28.3	9.484	5.482	8.467	17.416	12.135	11.879	23.88	9.275	7.252	42.73	25.8	30.18	18.48	25.65	36.83	10.56	13.2	6.579	6.48	14.3	13.3	12
Os01g0811300	261	282	232	270.1	264	258.6	301	285	322	256.1	251.1	265.3	226.41	252.97	292.24	298.5	248.4	252.8	210.9	240.3	234.5	326.8	317.1	317.1	300	249.9	291.7	275	280	276	17
Os01g0222300	0	0.93	3.23	1.642	2.712	3.46	1.57	1.27	2.07	3.449	3.289	5.645	0	0.9335	0	0	1.031	0	0.929	2.419	1.724	2.958	3.563	2.167	0	0	0	0.93	0.96	0	7
Os01g0950200	1.02	0	0	0.821	0.904	4.325	3.13	1.9	2.76	1.724	0	1.882	1.0885	0.9335	0.792	0	0	3.108	0	3.225	1.724	0.739	2.851	0.722	2.113	0.943	0	0	0.96	0	15
Os01g0834700	956	793	940	547.6	499	519.9	277	310	312	848.4	812.4	860.9	1045	936.25	1086.6	1612	709	737.7	606.6	534.6	541.5	328.3	340.6	333.7	878.9	946.6	938.6	813	801	746	13
Os01g0229900	102	118	112	220	226	197.2	116	118	117	63.8	74.55	82.79	68.577	64.408	83.948	107.5	81.41	89.1	148.6	208	179.3	93.16	104.8	113.4	95.07	75.43	81.14	66.6	67.9	68.3	10
Os01g0966500	661	638	682	697.8	717.8	652.2	770	684	679	645.8	732.4	702.8	721.69	792.5	754.74	740.3	647.2	620.6	786.8	607.9	647.5	668.4	674.2	652.9	704.6	625.1	688.6	658	647	699	4
Os01g0114800	15.3	16.8	22.6	13.96	18.08	19.03	50.9	51.2	22.8	52.59	36.18	25.4	25.036	17.736	39.598	23.88	18.55	34.19	18.58	25.8	13.8	36.97	65.56	26	49.65	37.71	28.51	56.4	47.8	41.7	5
Os01g0830100	1675	1437	1768	496.7	477.3	538.9	1329	1395	1444	1611	1589	1743	1837.4	1993.9	1780.3	2949	1753	1655	747.8	641.8	582	922	1035	1277	1733	1642	1693	1724	1724	1665	13
Os01g0374501	1.02	0	0	0	0	0	0	0	0	0	0	0	0	0	0	0	0	0	0	0	0	0	0	0	0	0	2.193	0	0	0	11
Os01g0551800	1.02	0	0	0	0	0	0	1.9	0	0	0	0	0	0	0	0	0	0	0	0	0	1.479	0	0.722	1.056	0.943	0	0	0	0	9
Os01g0155600	897	836	866	886.7	930.2	833.9	1228	1250	1287	1027	1021	1163	947.01	922.25	844.24	1540	854.3	916.9	956.8	984.5	945.9	1096	1094	1070	1058	1234	1081	918	865	896	6
Os01g0972900	1.02	1.86	0	4.105	10.85	2.595	7.83	17.7	10.4	0	0	0	0	0	0	0	3.092	0	0.929	15.32	9.484	6.654	4.276	11.56	0	0	2.193	0	0.96	0.83	12
Os01g0607800	371	411	432	734	804.5	737.9	651	855	692	297.5	356.3	367.9	563.85	495.66	387.27	537.3	404	490	720.9	818.4	880.3	571.5	564.4	738.9	460.6	382.8	399.1	371	346	428	12
Os01g0260000	7.14	3.73	6.47	50.9	48.81	46.71	15.7	27.2	22.1	0.862	2.193	2.822	3.2656	2.8004	2.3759	23.88	2.061	3.108	31.58	37.9	32.76	28.84	22.09	17.33	3.169	0.943	2.193	2.78	2.87	7.5	10
Os01g0753300	0	0	0	0	0	0	0	0	0	0	0	0	0	0	0	0	0	0	0	0	0	0	0	0	0	0.943	0	0	0	0	1
Os01g0268600	15.3	13	17.2	12.31	8.136	6.92	5.48	24	8.98	19.83	13.16	4.704	4.3541	10.268	18.215	0	16.49	13.47	5.574	9.675	0	45.84	38.48	30.33	6.338	4.714	10.96	7.4	8.61	5	17
Os01g0823200	278	285	321	121.5	150.1	140.1	74.4	94.2	99.5	234.5	288.4	300.1	375.54	391.12	355.59	597	356.6	318.1	95.68	104	114.7	136	130.4	135.8	359.2	364.9	256.6	285	345	341	13
Os01g0225200	273	273	269	388.3	395	387.5	407	384	370	255.2	275.2	257.8	261.24	243.63	276.4	549.3	262.8	300.5	344.6	398.3	383.7	350.5	328.5	305.5	239.8	246.1	250	306	268	287	6
Os01g0730450	0	0	0	0	0	0	1.57	0.63	0	0	0	0.941	0	0	0	0	0	0	0	0	0	0	0	0	0	0	0	0	0	0	5
Os01g0692050	0	0	0	0	0	0.865	0	0	0	0	0	0	0	0	0	0	0	0	0	0.806	0	0	0	0	0	0	0	0	0	0	10
Os01g0886000	688	760	804	980.2	981.7	1003	682	844	817	877.7	913.3	608.7	1001.4	947.45	1148.4	1827	749.2	722.1	1001	1001	963.1	658.1	700.5	716.5	840.9	746.7	657.9	871	811	996	6
Os01g0657000	0	0.93	1.08	0	0	0	0	0	0	0	0	0	0	0	0	0	0	0	0	0	0	0	0	0	0	0	0	0	0	0	14
Os01g0295900	931	843	984	1117	1133	1183	973	1144	1102	659.6	856.3	789.3	1207.2	913.85	928.98	1934	990.3	1024	1121	1276	1311	1327	1087	1022	961.3	722.2	725.9	874	883	1079	6
Os01g0976300	16.3	8.38	12.9	10.67	5.424	12.11	13.3	8.22	5.53	12.07	13.16	32.93	8.7082	26.137	3.9598	11.94	16.49	15.54	10.22	0.806	4.311	8.873	12.11	16.61	15.85	22.63	19.74	8.33	5.74	10	20
Os01g0174000	681	465	579	278.3	233.2	282.9	208	183	178	532	524.1	589.9	617.19	533	567.84	1051	734.8	595.7	536.9	497.5	342.3	170.8	208.1	197.9	666.6	662.8	673.2	682	529	605	13
Os01g0121500	27.5	28.9	18.3	4.926	2.712	20.76	0	0	2.76	1.724	2.193	5.645	11.974	7.4676	2.3759	47.76	75.23	44.55	0.929	2.419	6.036	4.436	6.414	7.945	4.225	2.829	2.193	0.93	2.87	5.83	14
Os01g0183300	23.4	14	24.8	23.81	25.31	32.87	5.48	15.8	16.6	21.55	13.16	16.93	11.974	22.403	15.047	23.88	10.31	13.47	10.22	8.869	9.484	26.62	34.92	10.11	12.68	31.11	41.67	18.5	23.9	25.8	11
Os01g0794800	7.14	3.73	3.23	5.747	3.616	11.25	3.13	2.53	0.69	2.587	3.289	1.882	0	0.9335	6.3357	0	8.244	6.216	1.858	0	3.449	11.09	7.126	2.889	1.056	2.829	13.16	1.85	3.83	1.67	11
Os01g0182200	684	754	788	850.5	982.6	810.6	767	789	900	704.4	834.4	691.5	969.87	845.71	796.72	1815	785.3	806	996.8	961.9	851.9	683.2	762.5	809.6	814.5	645.8	649.1	809	818	891	6
Os01g0323300	1349	1289	1428	2587	2757	2591	853	1030	1008	1439	1706	1405	2382.8	2438.2	2009.2	2328	1510	1545	2728	2711	3049	746.8	727.6	853.7	1577	1458	1210	1596	1531	1713	16
Os01g0817800	96.9	82.9	74.4	78.81	72.32	90.83	334	215	171	160.4	161.2	240.8	119.74	141.88	122.75	47.76	89.66	69.41	140.3	138.7	115.5	77.64	94.78	145.9	134.2	148	164.5	92.5	112	58.3	15
Os01g0187900	287	317	342	777.5	713.2	764.7	418	479	405	233.7	232.4	312.4	388.6	455.52	345.3	644.8	374.1	336.7	723.7	670.8	790.7	296.5	306.4	287.5	251.4	323.4	228.1	415	465	412	10
Os01g0195500	873	834	953	927.7	916.6	831.3	801	736	746	1047	1047	1255	969.87	939.05	940.06	919.5	937.8	898.2	1181	1192	1167	631.4	596.5	658.7	980.3	1040	1138	869	875	838	16
Os01g0797700	5.1	10.2	12.9	1.642	2.712	3.46	0.78	1.9	3.46	8.622	8.771	2.822	20.682	9.3345	10.296	11.94	12.37	4.144	1.858	0.806	0	0	3.563	3.611	9.507	4.714	8.772	11.1	1.91	5	3
Os01g0598400	35.7	33.5	33.4	27.09	28.02	27.68	72.8	59.5	60.8	34.49	31.8	31.05	30.479	29.87	35.638	47.76	25.76	38.33	29.73	27.41	35.35	44.36	52.02	46.22	29.58	20.74	4.386	18.5	22	13.3	18
Os01g0276500	282	267	237	367	445.7	391	199	273	215	172.4	182	223	148.04	176.42	156.81	417.9	198.9	220.7	336.3	294.3	339.7	260.3	204.5	247	182.8	210.3	225.9	179	197	184	10
Os01g0863400	816	688	746	480.3	527.9	473.2	596	596	542	694.9	763.1	860.9	850.13	953.99	902.84	1063	850.2	811.2	514.6	483	551.8	558.2	491.7	478.8	859.9	859.9	824.5	799	736	771	3
Os01g0136700	1.02	0.93	4.31	3.284	5.424	3.46	0.78	6.96	2.07	0.862	4.386	2.822	1.0885	0	4.7518	0	2.061	4.144	1.858	1.613	0	14.05	4.988	7.945	8.451	0.943	4.386	0.93	2.87	3.33	9
Os01g0179300	1098	972	1030	703.6	687	773.4	775	812	985	850.1	843.1	924.8	912.18	835.44	796.72	2352	978	929.3	717.2	717.6	782	819.2	804.6	808.9	890.5	952.3	804.8	742	737	829	6
Os01g0303800	11.2	9.31	7.54	18.06	20.79	18.17	193	66.4	257	7.76	8.771	19.76	4.3541	6.5342	5.5438	0	4.122	5.18	7.432	4.031	8.622	485	193.1	372	2.113	0.943	8.772	2.78	2.87	0.83	9
Os01g0774200	91.8	96.9	105	151.1	151	115.1	157	152	120	79.32	119.5	87.5	119.74	83.077	86.324	262.7	113.4	109.8	118	143.5	146.6	133.1	103.3	96.06	77.11	92.4	83.33	73.1	140	132	6
Os01g0860400	62.2	31.7	75.4	9.852	7.232	20.76	35.2	24	33.2	18.11	10.96	8.467	11.974	24.27	23.759	23.88	63.89	20.72	9.29	0	4.311	36.23	17.1	75.84	23.24	18.86	32.89	24.1	17.2	7.5	14
Os01g0668000	835	796	815	755.3	784.6	847.7	848	795	795	1010	944	1144	1134.2	1110.8	1123	1887	1049	945.9	916.9	938.5	957.9	602.6	672.7	682.5	1079	1075	1066	1057	1087	1004	13
Os01g0242900	250	183	186	193.8	205.2	228.4	190	154	182	200.9	179.8	215.4	169.81	195.09	179.78	441.8	162.8	146.1	165.4	179.8	186.2	171.5	216.6	226.8	198.6	198	234.6	197	180	166	6
Os01g0166400	14.3	8.38	12.9	14.78	15.37	18.17	22.7	10.1	15.9	10.35	13.16	22.58	7.6196	8.4011	8.7116	0	14.43	6.216	10.22	7.257	18.97	11.83	19.24	9.389	2.113	2.829	6.579	7.4	10.5	3.33	15
Os01g0235350	298	264	305	119.9	141.9	109	116	120	112	231.1	257.7	213.6	290.63	273.5	366.68	489.6	412.2	378.2	117	152.4	108.6	145.7	166	135.1	299	329.1	313.6	382	328	356	13
Os01g0210700	5.1	2.79	1.08	5.747	6.328	1.73	3.91	4.43	3.46	8.622	6.578	6.586	6.5311	11.201	5.5438	0	10.31	7.252	13.01	3.225	7.76	5.915	12.11	6.5	7.395	15.09	6.579	13	8.61	7.5	1
Os01g0816000	18.4	14.9	12.9	87.84	125.7	85.64	54.8	65.1	36.6	3.449	10.96	3.763	7.6196	12.135	4.7518	23.88	5.153	5.18	69.67	85.47	62.94	30.31	59.15	53.45	2.113	2.829	6.579	8.33	2.87	5	10
Os01g0685650	47.9	39.1	44.2	45.15	56.95	51.9	12.5	31	15.9	33.63	18.64	2.822	44.629	16.802	29.303	23.88	42.25	40.41	23.22	30.64	13.8	51.02	45.61	18.06	33.8	21.69	13.16	43.5	44	43.3	8
Os01g0185100	0	0	0	0	0	0	0	1.27	0	0	0	0	0	0	0	0	1.031	0	0	1.613	0	1.479	0	0	0	0	0	0	0	0.83	9
Os01g0360100	56.1	60.5	56	80.46	82.26	87.37	100	115	100	80.18	76.75	64.92	70.754	83.077	91.868	83.59	65.95	73.56	93.82	113.7	100	77.64	49.17	83.78	83.45	101.8	96.49	70.3	120	82.5	15
Os01g0612425	2.04	3.73	0	0	0	0	0	0	0	0.862	2.193	4.704	0	0.9335	0	0	0	0	0	0	0	1.479	0	0	2.113	0	2.193	0.93	0	0	20
Os01g0974300	567	672	625	457.3	548.7	499.1	590	513	560	576.8	571.2	493.9	623.72	499.4	642.28	955.3	652.3	616.4	383.7	493.4	435.4	681	627.1	663.7	612.7	676	638.1	682	601	612	13
Os01g0256600	229	163	187	211.8	162.7	196.4	251	252	340	287.1	257.7	348.1	248.18	212.83	249.47	441.8	210.2	197.9	216.4	236.2	210.4	222.6	223.8	241.2	237.7	254.6	256.6	208	208	177	6
Os01g0109500	11.2	14.9	14	12.31	17.18	15.57	21.1	20.9	16.6	15.52	8.771	15.05	9.7967	19.602	3.1679	23.88	9.275	13.47	27.87	12.09	26.73	16.27	10.69	20.95	12.68	7.543	8.772	8.33	15.3	13.3	12
Os01g0930000	440	499	494	757.8	823.5	828.7	975	954	948	564.7	672.1	680.2	570.38	468.59	384.9	871.7	438	492.1	988.4	1113	1064	770.4	682.7	775	490.2	432.8	427.6	391	411	505	12
Os01g0363500	470	547	525	535.3	495.4	565.7	864	834	864	678.5	618.4	585.2	582.36	648.75	728.61	1349	547.2	529.4	558.3	607.9	501.8	786	727.6	702.7	623.3	663.8	587.7	647	651	701	6
Os01g0501700	3262	3322	3710	4621	5163	4729	2726	2646	2441	4267	4500	5305	5003.9	4523.5	4401	5517	3578	3555	4954	4771	5925	1640	1477	1890	4238	4544	3914	2963	3185	3452	16
Os01g0100600	316	403	373	528.7	617.4	572.7	489	509	405	351.8	457.2	387.6	424.52	383.65	365.89	406	380.3	357.4	517.4	600.7	544.9	539	495.3	470.9	367.6	396	344.3	377	372	443	10
Os01g0643300	14.3	2.79	12.9	0	0.904	6.055	3.13	0.63	1.38	6.897	3.289	11.29	2.177	20.536	10.296	11.94	15.46	11.4	8.361	3.225	6.036	2.958	2.138	1.444	24.3	9.428	19.74	4.63	4.78	8.33	3
Os01g0901300	9.18	18.6	10.8	70.6	74.13	91.7	91.6	59.5	104	4.311	6.578	14.11	3.2656	7.4676	1.5839	0	9.275	7.252	37.16	45.96	31.9	71.72	46.32	66.45	1.056	3.771	4.386	7.4	6.69	3.33	12
Os01g0639100	845	786	829	866.9	1011	917.8	1197	1112	1172	864.8	842	980.3	908.91	855.04	800.68	1349	782.2	800.9	882.5	882.9	989	878.4	976.3	1054	914.8	810.8	907.9	792	735	791	6
Os01g0681800	45.9	54	52.8	18.88	25.31	23.36	368	214	156	157.8	94.29	76.21	75.108	56.007	89.492	35.82	78.32	78.74	26.94	29.03	15.52	152.3	160.3	174.8	58.1	68.83	98.68	27.8	49.7	38.3	5
Os01g0294500	0	0	1.08	3.284	1.808	2.595	0	1.9	3.46	0	0	1.882	3.2656	0	4.7518	11.94	0	0	3.716	6.45	3.449	4.436	0.713	2.167	0	1.886	0	2.78	1.91	4.17	6
Os01g0180400	44.9	38.2	26.9	41.05	24.41	24.22	56.4	68.9	59.4	29.31	44.95	22.58	44.629	26.137	32.471	95.53	38.13	33.15	29.73	42.73	39.66	48.06	42.05	38.28	32.75	33.94	28.51	34.2	31.6	22.5	6
Os01g0319000	0	0	0	0	0	0	0	0	0	0	0	0	0	0	0.792	0	0	0	0	0	0	0	0	0	1.056	0	0	0	0	0.83	4
Os01g0789000	0	0	0	0	0	0	0	0	0	0	0	0	0	0	0	0	0	1.036	3.716	0	0	0.739	0	0	0	0	0	0	0.96	0	16
Os01g0219500	0	0	0	0	0	0	0	0	0	0	0	0	0	0	0	0	0	0	1.858	0	0	0	0	0	0	0	0	0	0	0	16
Os01g0201200	1742	2141	2102	1066	1277	732.7	1802	1885	1683	2969	3705	2176	3499.6	2974.9	3898.1	4693	2085	2348	930.8	1188	860.5	1359	1714	1208	3472	2949	2011	3578	3922	4316	13
Os01g0179800	4393	3836	4798	307.9	264	355.5	148	195	175	4759	5620	5624	6877.3	6130	6544	9374	6442	5332	693	596.6	551	139	173.9	192.1	6634	6383	4577	5596	5737	6484	3
Os01g0915900	187	161	95.9	111.7	119.3	109.9	82.2	160	47.7	160.4	134.9	240.8	115.38	326.71	186.9	298.5	316.4	169.9	168.1	83.05	324.2	59.89	197.4	65	157.4	336.6	307	233	258	208	13
Os01g0143100	436	364	370	311.1	292	272.5	245	292	266	388	458.3	540	500.72	476.06	394.4	883.6	545.2	422.7	380.9	366.1	345.7	288.4	230.9	254.2	474.3	502.5	423.2	461	472	433	13
Os01g0181000	244	269	241	332.5	355.3	288.1	269	339	348	300.9	266.4	312.4	309.14	317.37	335.79	346.3	266.9	266.3	326.1	329.8	303.5	215.9	258.7	232.6	306.3	339.4	344.3	260	288	267	16
Os01g0856800	433	407	501	465.5	463.7	429.9	636	567	561	432.8	507.6	434.7	482.21	454.59	491.81	692.6	529.7	442.4	572.2	508	633.7	344.6	382.7	476.7	394	399.8	475.9	406	404	427	6
Os01g0247600	122	149	145	240.5	264	250.9	524	586	506	186.2	196.3	334	164.37	117.61	133.05	167.2	160.8	141.9	366.9	332.2	332	305.4	280.1	409.5	161.6	172.5	153.5	84.2	140	132	18
Os01g0620100	398	397	343	411.3	473.7	471.5	573	498	490	338	377.2	394.2	371.19	399.52	354.8	382.1	262.8	368.8	425.5	413.6	413.9	445.9	406.2	488.2	283.1	270.6	280.7	262	282	295	12
Os01g0299100	0	0	0	0	0	0	0	0	0	0	0	0.941	0	0	0	0	0	0	0	0	0	0	0	0	0	0	0	0	0	0	20
Os01g0808400	0	0	0	0	0	0	0	0.63	0	0	0	0	1.0885	0	0	0	0	0	0	2.419	0.862	0	0	0	0	0.943	0	0	0	0	16
Os01g0909400	6.12	9.31	9.7	13.14	18.98	27.68	132	156	162	8.622	5.482	6.586	5.4426	3.7338	3.1679	0	9.275	10.36	14.86	4.838	12.07	90.21	89.79	91.73	4.225	14.14	13.16	1.85	2.87	1.67	18
Os01g0356900	0	0	1.08	6.568	3.616	0.865	11.7	6.33	21.4	2.587	0	4.704	0	0.9335	2.3759	0	0	0	0	0	0	0.739	0	0	0	0	0	0	0	0	18
Os01g0969700	32.6	20.5	28	19.7	18.08	23.36	9.39	15.8	20.7	17.24	17.54	10.35	11.974	9.3345	5.5438	0	10.31	16.58	22.29	24.99	13.8	11.83	20.67	26	14.79	12.26	19.74	13.9	17.2	5	19
Os01g0619800	221	244	152	238.1	225.1	207.6	265	357	301	326.8	259.8	415.8	197.02	359.38	270.06	203	242.2	272.5	231.3	255.6	275.9	408.9	366.3	330.1	398.2	529.9	484.6	225	168	246	11
Os01g0946100	2.04	0	1.08	0	0.904	0.865	0	0	0	0	0	3.763	0	1.8669	0	0	0	0	0	0	0	0	0	0	0	0	0	0	0	0.83	20
Os01g0937200	84.6	68.9	52.8	38.59	28.93	36.33	47	56.9	33.9	89.67	81.13	174.1	68.577	71.876	52.27	107.5	78.32	87.03	79.89	45.96	43.97	42.15	42.76	65	87.68	97.11	125	78.6	80.3	60	20
Os01g0335600	0	0	0	1.642	0.904	0	0	0	0	3.449	0	0	0	0	0	0	0	1.036	0	0	1.724	0.739	0.713	0.722	0	3.771	0	0	0	0	7
Os01g0698100	820	986	894	833.3	829.8	899.6	500	661	566	664.7	842	549.4	777.2	731.83	919.47	1445	871.8	1018	505.4	692.6	549.2	1178	991.3	730.9	860.9	772.2	620.6	1134	1048	1138	8
Os01g0767700	1469	1313	1356	1329	1191	1284	708	872	754	981.2	1225	1067	1764.5	1355.4	1465.1	2436	1264	1498	1083	1262	1091	998.9	949.9	847.9	1294	1113	1000	1568	1531	1623	13
Os01g0266400	14.3	14.9	6.47	6.568	9.04	4.325	10.2	1.9	7.6	7.76	8.771	22.58	0	6.5342	3.9598	0	7.214	9.324	6.503	5.644	6.898	22.18	9.264	13	5.282	3.771	6.579	7.4	3.83	1.67	9
Os01g0913300	0	0.93	0	0	0	0	0	0	0	0	2.193	0	0	0	0	0	0	0	0	0	0.862	0	0	0	0	0	0	0	0	0	7
Os01g0202700	40.8	21.4	21.6	64.04	18.98	71.8	14.1	15.2	38.7	53.46	52.63	45.16	25.036	34.538	17.423	35.82	34.01	26.94	55.74	12.09	24.14	18.48	18.53	10.11	26.41	27.34	50.44	26.8	37.3	35	7
Os01g0625300	66.3	48.4	36.6	11.49	12.66	6.92	12.5	10.8	6.91	79.32	97.58	85.61	52.249	85.878	72.861	47.76	73.17	61.13	6.503	12.09	16.38	19.96	32.07	39	185.9	132.9	103.1	127	93.7	91.6	3
Os01g0144000	401	359	405	433.5	377	449.8	326	279	344	516.4	385.9	551.3	304.79	384.58	364.3	597	419.4	411.3	535.1	453.9	441.5	342.3	339.2	378.5	438.4	439.4	572.4	418	364	384	16
Os01g0100700	666	466	483	588.6	611.1	699	688	569	760	695.8	572.3	1358	668.35	693.55	453	931.4	565.8	562.6	812.8	687.8	848.4	603.3	516.7	533.7	533.5	817.4	756.6	442	555	356	20
Os01g0732200	841	964	925	2057	2298	1931	2332	2469	2357	1209	1426	1710	1175.6	859.71	855.32	1433	721.4	830.9	2016	2198	2267	1521	1434	1944	1018	1052	888.1	523	614	661	12
Os01g0878800	309	330	316	229.9	236.8	238.8	354	427	404	469	351.9	359.4	412.55	378.05	362.72	597	279.3	277.7	280.5	331.4	205.2	295	401.2	363.3	330.6	293.2	254.4	270	281	322	6
Os01g0386700	3677	3655	4027	3016	3215	3336	4612	4634	4875	3815	4192	4468	5086.7	4773.7	4878.5	7594	4927	4708	3525	3841	3890	5457	4351	4323	4983	5140	5147	4655	5162	4962	13
Os01g0849000	35.7	6.52	4.31	61.57	28.02	295	24.3	11.4	4.84	25	5.482	25.4	84.905	25.203	0.792	0	2.061	2.072	22.29	3.225	97.43	2.218	17.1	4.333	3.169	20.74	15.35	6.48	3.83	5	2
Os01g0952600	548	682	554	1469	1576	1337	1690	1791	1910	568.2	616.2	405.5	487.66	397.65	540.12	979.2	448.3	480.7	671.6	861.9	646.7	1772	1558	1531	445.8	331.9	392.5	424	427	520	18
Os01g0847700	3909	2340	4006	873.5	858.8	733.6	213	271	227	1123	1083	1471	2148.7	2661.3	2220.7	6484	4393	2935	1488	1102	984.7	207.8	354.9	437	2098	2071	2629	2980	2419	2698	13
Os01g0705000	10.2	14.9	17.2	24.63	26.22	13.84	14.9	19	13.1	11.21	2.193	2.822	5.4426	9.3345	4.7518	35.82	16.49	14.5	26.01	15.32	29.32	17.01	14.97	28.17	8.451	6.6	13.16	3.7	2.87	3.33	6
Os01g0609900	61.2	98.7	84.1	321.8	278.4	201.6	142	207	258	43.11	28.51	31.05	37.01	30.804	79.989	131.4	46.37	46.62	105.9	186.3	70.7	667.7	693.4	383.5	23.24	19.8	28.51	52.7	70.8	50	17
Os01g0838300	12.2	14.9	23.7	9.031	19.89	12.11	19.6	29.1	19.3	15.52	8.771	22.58	15.239	12.135	18.215	119.4	24.73	9.324	32.51	16.93	12.93	8.133	25.65	31.06	12.68	16.03	21.93	20.4	16.3	17.5	6
Os01g0939700	1361	1065	1388	1069	1114	982.7	1046	982	950	1066	934.1	1352	1590.3	1475.8	1207	1576	1013	1122	1110	1020	1202	667.7	652.1	850.8	1140	966.4	1114	1053	1017	1064	13
Os01g0131800	98.9	135	121	161.7	229.6	159.2	192	229	202	153.5	150.2	128	177.43	132.55	144.93	203	116.5	95.32	145.8	199.2	160.4	157.5	155.4	154.6	127.8	139.5	140.3	113	120	137	12
Os01g0106300	0	3.73	0	1.642	0.904	0.865	0	0	0	0	0	0	1.0885	0	1.5839	0	0	0	0	0	0	0	0	0	0	0	2.193	0	0.96	0	14
Os01g0930500	1.02	1.86	0	3.284	0.904	1.73	1.57	0	0	6.897	5.482	3.763	2.177	2.8004	2.3759	0	2.061	0	2.787	0	0	0	0	0	0	0.943	8.772	0	0	3.33	11
Os01g0739300	87.7	117	84.1	163.4	152.8	154	286	335	337	108.6	96.48	117.6	165.46	124.15	164.73	250.8	124.7	120.2	183.9	149.2	194.9	158.2	157.5	210.2	116.2	96.17	100.9	97.1	87	102	18
Os01g0756000	0	0	0	0	0	0	0	0	0	0	0	0	1.0885	0	0	0	0	0	0	0	0	0.739	0	0	2.113	0	0	0	0	0	4
Os01g0760000	68.3	47.5	53.9	12.31	7.232	10.38	7.05	6.33	9.67	15.52	12.06	16.93	9.7967	1.8669	3.1679	0	39.16	27.97	26.01	7.257	15.52	9.612	9.977	18.06	5.282	8.486	13.16	2.78	2.87	4.17	14
Os01g0382400	5.1	9.31	1.08	3.284	0	13.84	1.57	0.63	2.76	0	1.096	7.527	0	2.8004	2.3759	0	0	0	0	0	0	0	0	1.444	0	0	6.579	0.93	0	0	19
Os01g0654200	630	648	635	523.8	581.3	568.3	568	523	570	603.5	623.8	601.2	610.66	659.02	639.12	1684	675	712.8	448.7	564.4	562.2	761.6	667.7	621.9	651.8	659	655.7	760	817	792	6
Os01g0747700	586	616	677	547.6	601.1	545.8	545	579	547	600.9	719.2	592.7	807.68	784.1	833.15	1600	724.5	661	654.9	715.2	760.5	468	446.8	498.3	680.3	740.1	583.3	587	714	638	13
Os01g0150100	318	266	390	273.4	247.7	240.5	195	208	200	315.6	395.8	294.5	362.48	392.98	405.49	453.8	362.7	349.1	264.8	297.5	303.5	261.7	196.7	206.6	376.1	356.4	342.1	426	361	375	13
Os01g0577450	0	0.93	0	2.463	0.904	0.865	1.57	3.16	2.76	1.724	5.482	2.822	0	0.9335	0.792	0	0	1.036	0.929	0	0	0.739	1.425	1.444	0	0.943	0	0.93	0	0	7
Os01g0970500	62.2	82.9	48.5	92.77	130.2	93.43	117	119	124	103.5	77.84	82.79	59.869	79.343	63.357	119.4	60.8	55.95	130.1	116.9	102.6	62.85	120.4	75.11	86.62	54.68	57.02	98.1	118	96.6	12

Os01g0908100	244	289	219	335	382.4	351.2	524	559	587	337.1	344.3	333.1	244.92	258.57	336.59	334.3	246.3	266.3	271.3	324.9	256.1	500.6	471.8	449.2	310.6	291.3	287.3	254	233	270	18
Os01g0658300	0	0.93	0	0	0	0	0	0	0	0	0	0	0	0	0.792	0	0	1.036	0	0	0	0	0	0	1.056	0	0	1.85	1.91	0	1
Os01g0971100	1399	1500	1755	711	712.3	783.7	1995	2276	2055	1693	1585	1718	1900.6	2086.3	1960.9	4514	2145	2039	1014	1107	1066	1468	1484	1686	1912	2181	2132	1987	2259	2375	13
Os01g0710700	2259	2045	2169	2405	2325	2469	3395	3734	4077	1815	1670	2272	2060.6	1599	1467.5	2018	1750	1961	2267	2207	2312	3548	3426	3873	1794	1778	1787	1079	1183	1302	18
Os01g0826000	506	303	420	144.5	131.1	375.4	109	155	120	216.4	151.3	389.5	354.86	745.83	447.46	561.2	476.1	402	178.4	92.72	165.5	99.82	92.64	94.61	316.9	304.5	445.2	483	422	482	13
Os01g0633400	30.6	55.9	45.3	29.56	45.2	21.63	85.3	132	69.8	55.18	50.43	18.82	44.629	44.806	68.109	95.53	26.79	41.44	18.58	14.51	12.07	46.58	83.38	24.56	53.87	13.2	35.09	47.2	53.6	45.8	5
Os01g0740650	143	213	202	339.9	322.7	289.8	52.5	72.1	58	88.81	86.62	31.99	76.196	68.142	101.37	47.76	147.4	123.3	159.8	208.8	178.5	183.4	156.8	100.4	88.73	86.74	118.4	218	184	181	2
Os01g0367400	25.5	47.5	46.3	68.14	57.85	87.37	93.9	53.8	58.7	12.07	13.16	10.35	38.098	11.201	26.927	11.94	11.34	13.47	22.29	35.48	25.87	62.85	42.05	91	20.07	2.829	13.16	8.33	25.8	24.2	12
Os01g0878200	0	0	0	0	0	0	2.35	1.27	0	0	0	0	0	0	0	0	0	0	0.929	0.806	0	0	0	0	0	0	0	0	0	0	15
Os01g0607300	28.5	22.4	26.9	32.02	33.45	42.39	18	29.1	41.5	21.55	13.16	22.58	23.947	24.27	19.799	47.76	19.58	21.76	60.38	54.83	47.42	34.75	34.92	49.11	25.35	33.94	50.44	26.8	25.8	35	10
Os01g0564300	182	333	270	237.3	523.4	165.2	378	673	339	405.2	359.6	344.3	169.81	282.84	233.63	465.7	108.2	218.6	221.1	126.6	50.87	745.3	1014	530.1	276.8	68.83	100.9	238	249	276	17
Os01g0934400	424	419	394	64.86	59.66	47.58	155	98	76.7	344.9	313.6	623.8	303.7	300.57	310.45	704.5	520.4	459	38.09	80.63	56.91	126.4	101.9	136.5	380.3	586.4	449.5	253	320	262	13
Os01g0161200	0	0	0	1.642	0	0	0	0	0.69	2.587	0	0	0	0	0.792	0	0	0	1.858	0	0.862	1.479	0	1.444	0	0.943	0	0	0	0	7
Os01g0289900	1.02	0	0	0	0	0.865	0.78	1.27	0	4.311	0	0	0	0.9335	0	0	1.031	1.036	0	0	0	0	0	0	2.113	0.943	0	1.85	0	0	7
Os01g0242800	43.8	49.4	24.8	29.56	34.35	28.55	18	21.5	15.2	29.31	24.12	25.4	30.479	37.338	18.215	23.88	38.13	35.23	34.37	26.61	19.83	37.71	41.33	46.22	54.93	36.77	19.74	49	36.3	35	1
Os01g0920700	2.04	0	4.31	0	0	0.865	0.78	0.63	2.76	0	1.096	0	1.0885	0.9335	0	0	0	0	1.858	0	0	6.654	7.126	7.222	0	0	2.193	0.93	0	3.33	9
Os01g0206650	5.1	14.9	14	9.852	7.232	3.46	3.91	0	2.07	10.35	3.289	4.704	14.151	14.935	10.296	23.88	12.37	12.43	2.787	4.031	0	7.394	6.414	2.167	11.62	7.543	6.579	4.63	6.69	10	13
Os01g0622600	3616	2604	2914	1245	1307	1343	742	847	917	1686	1545	1527	1309.5	1415.1	1435.8	1576	1748	2117	1445	1403	1402	1108	1175	986.6	1430	1443	1397	1250	1173	1217	14
Os01g0519050	6.12	5.59	4.31	5.747	6.328	9.516	3.91	1.9	4.15	9.484	13.16	9.408	10.885	4.6673	5.5438	0	8.244	9.324	2.787	4.838	6.898	5.176	4.988	8.667	11.62	7.543	6.579	12	6.69	4.17	1
Os01g0304000	1292	973	1088	1236	1298	1374	1221	980	1426	1437	1275	2175	1360.6	1627.9	1105.6	2531	1035	1080	1672	1438	1604	1051	1147	1104	1300	1612	1767	940	1128	772	6
Os01g0684800	8.16	1.86	7.54	9.852	9.944	14.71	11.7	10.8	10.4	4.311	6.578	0.941	7.6196	1.8669	9.5036	0	4.122	1.036	3.716	13.71	12.07	10.35	19.95	10.11	4.225	5.657	0	6.48	5.74	7.5	17
Os01g0314000	21.4	14	17.2	25.45	28.93	19.03	14.9	20.2	20.7	23.28	30.7	36.69	15.239	14.002	16.631	11.94	10.31	17.61	28.8	24.19	26.73	12.57	22.8	31.06	19.01	21.69	21.93	19.4	17.2	15	15
Os01g0674150	11.2	13	20.5	19.7	9.04	25.09	26.6	22.1	31.8	21.55	15.35	28.22	25.036	21.469	13.463	11.94	14.43	20.72	24.15	16.93	42.25	25.14	29.93	25.28	28.52	25.46	30.7	25	17.2	18.3	15
Os01g0769100	177	176	209	252	288.4	294.1	400	403	375	269.9	324.5	314.2	290.63	331.38	289.86	179.1	273.1	298.4	386.4	431.4	497.5	298.7	282.9	344.5	282	326.2	453.9	248	263	248	15
Os01g0775750	68.3	56.8	57.1	42.69	73.22	46.71	90	96.8	81.5	59.49	67.98	68.68	75.108	44.806	58.605	119.4	76.26	62.16	56.67	66.92	48.28	51.76	51.31	77.28	46.48	60.34	46.05	51.8	49.7	60	6
Os01g0846450	452	512	498	440.9	407.7	407.4	436	461	490	485.4	498.9	389.5	449.56	432.19	498.15	1314	617.3	598.8	362.3	450.7	381.1	439.9	481.7	374.1	499.7	482.7	578.9	650	723	594	13
Os01g0271700	502	379	413	453.2	452	519.9	661	471	702	657.8	597.5	915.4	525.76	637.55	469.64	656.8	455.5	441.4	689.3	535.4	685.5	450.3	431.1	487.5	524	654.3	640.3	395	502	334	20
Os01g0922000	679	619	676	478.6	373.3	459.3	335	405	316	557.8	514.2	448.8	556.23	489.13	563.09	1349	669.8	704.5	379.9	502.3	397.5	522.7	449.7	398	546.1	498.8	423.2	636	592	610	13
Os01g0600300	815	768	886	995.8	901.3	954.1	603	539	483	945	976.9	1088	1057	1087.5	1115.1	1994	1020	1082	1088	1198	1065	700.9	692	619.7	1111	1031	973.7	1091	1089	1105	13
Os01g0225400	43.8	60.5	93.7	98.52	104.9	106.4	139	207	186	66.39	70.17	74.32	91.436	59.741	85.532	59.7	49.47	51.8	130.1	86.27	102.6	169.3	174.6	148.8	48.59	24.51	52.63	33.3	47.8	35.8	18
Os01g0716500	30.6	10.2	18.3	21.35	8.136	15.57	46.2	41.7	48.4	39.66	46.05	49.86	53.337	40.138	34.847	11.94	26.79	12.43	26.94	21.77	20.69	15.53	40.62	23.83	31.69	37.71	50.44	29.6	17.2	19.2	5
Os01g0712700	80.5	79.2	134	18.06	18.08	22.49	48.5	90.4	126	133.6	155.7	67.74	141.51	152.15	136.22	214.9	84.5	73.56	21.37	25.8	12.93	25.88	34.21	38.28	125.7	60.34	78.95	105	212	129	13
Os01g0777300	7.14	11.2	2.16	0.821	3.616	4.325	6.26	4.43	2.76	3.449	3.289	1.882	5.4426	1.8669	1.5839	23.88	10.31	6.216	1.858	2.419	2.587	4.436	6.414	5.056	3.169	6.6	6.579	9.25	2.87	10	6
Os01g0799900	510	530	452	450.7	496.3	493.9	509	528	482	502.7	442.9	481.7	385.34	467.66	425.29	943.3	481.3	485.9	376.2	375.7	346.6	553.1	549.4	621.1	512.3	475.2	625	458	405	474	6
Os01g0949700	413	375	452	229.1	230.5	204.2	48.5	42.4	37.3	202.6	223.7	236.1	156.75	244.56	186.11	573.2	330.8	282.8	214.6	221.7	167.3	45.1	75.54	62.84	229.2	182.9	217.1	269	196	308	13
Os01g0646000	3.06	7.45	11.9	339.1	366.1	287.2	47.8	68.9	44.9	5.173	4.386	2.822	1.0885	5.6007	1.5839	11.94	1.031	9.324	201.6	260.4	263.8	43.62	57.72	40.45	2.113	1.886	0	2.78	2.87	5	10
Os01g0614300	7.14	12.1	1.08	6.568	12.66	9.516	5.48	11.4	0	3.449	2.193	2.822	15.239	11.201	9.5036	0	7.214	15.54	6.503	7.257	8.622	6.654	0.713	9.389	8.451	8.486	4.386	2.78	3.83	5	4
Os01g0827400	89.7	64.3	83	158.4	133.8	179.9	103	104	100	107.8	80.04	103.5	95.79	112.01	82.364	203	74.2	83.92	209	175	165.5	79.85	68.41	97.5	89.79	79.2	78.95	70.3	86.1	66.6	10
Os01g0891400	0	0.93	1.08	0.821	1.808	0.865	2.35	6.96	2.07	0	2.193	0	0	1.8669	0.792	0	2.061	0	3.716	4.838	5.173	2.218	1.425	2.889	0	3.771	0	0	0	0	12
Os01g0764000	56.1	66.1	65.7	35.3	27.12	45.85	16.4	23.4	22.8	59.49	42.76	31.05	56.603	56.007	64.941	23.88	39.16	46.62	47.38	27.41	20.69	23.66	24.94	28.89	40.14	37.71	37.28	50	47.8	50	3
Os01g0218800	459	542	519	626.4	648.1	678.2	581	679	624	476.8	539.4	594.6	569.3	539.53	606.65	1063	597.7	567.7	549.9	645	646.7	716.5	714.1	647.1	656	595.9	587.7	575	578	612	6
Os01g0361000	2623	2721	2788	2451	2556	2522	2556	2744	2633	2112	2504	2682	3093.6	2806.9	2677.6	6305	3711	3370	2463	2698	2529	2744	2358	2271	2813	2994	2746	3289	3702	3662	13
Os01g0975000	107	99.7	66.8	7.389	7.232	11.25	1.57	7.59	11.7	18.97	16.45	1.882	10.885	18.669	23.759	47.76	60.8	51.8	18.58	7.257	7.76	12.57	8.552	22.39	13.73	6.6	4.386	13	23.9	18.3	14
Os01g0779201	2.04	0.93	1.08	0.821	3.616	0.865	0	0	0	0	0	0	3.2656	7.4676	0	0	1.031	1.036	0.929	0.806	0	1.479	1.425	1.444	0	1.886	0	1.85	1.91	1.67	4
Os01g0580800	329	319	339	234	169	215.4	218	181	267	349.2	296	373.5	275.4	279.1	270.85	788.1	389.5	390.6	279.6	177.4	190.5	383	399.8	349.6	330.6	380.9	458.3	428	473	427	13
Os01g0724450	13.3	16.8	26.9	21.35	19.89	13.84	3.91	14.5	3.46	6.035	8.771	0	13.062	5.6007	6.3357	0	11.34	10.36	3.716	8.869	0	26.62	25.65	7.222	8.451	8.486	2.193	16.7	23	15.8	8
Os01g0321300	3500	3686	3751	4089	4738	4045	4663	5288	4827	3624	4155	3960	4895.1	4378.8	4331.3	6006	3683	3772	3982	4235	4082	3728	3477	4227	4112	3852	3381	3714	4144	4051	6
Os01g0964333	72.4	80.1	97	30.38	37.97	28.55	32.9	53.1	35.9	77.6	80.04	16.93	117.56	70.942	90.284	191.1	101	110.9	13.93	29.83	12.07	59.89	61.29	44.78	61.27	62.23	63.59	110	135	137	13
Os01g0253500	0	0.93	0	0.821	4.52	0.865	1.57	0	2.07	1.724	0	0	0	0	0	0	1.031	0	0	0	0	5.915	0	8.667	1.056	0	0	0	2.87	0.83	9
Os01g0723800	361	308	293	485.2	503.5	508.6	416	557	518	319	207.2	466.6	231.85	268.83	210.66	238.8	220.5	219.6	475.6	298.3	422.5	838.5	630.7	613.2	216.6	331.9	486.8	208	235	167	12
Os01g0347200	50	31.7	39.9	53.36	60.57	41.52	103	82.2	74.6	86.22	78.94	70.56	38.098	39.205	37.222	35.82	27.82	26.94	40.87	31.44	36.21	73.2	111.9	101.8	20.07	40.54	41.67	32.4	32.5	25.8	18
Os01g0973100	106	181	135	180.6	317.3	220.6	1988	792	715	504.4	247.8	207.9	70.754	169.89	132.26	107.5	94.81	98.42	122.6	388.6	342.3	454	294.3	478.1	73.95	91.46	232.4	18.5	36.3	36.7	18
Os01g0799600	0	0	0	0	0	0	0	0	0	0	0	0	0	0	0	0	0	0	0	0	0	0	0	0	0	0	0	0	0	0.83	1
Os01g0650200	646	632	620	390	377.9	331.3	396	476	343	446.6	608.5	552.3	550.79	561.94	714.35	883.6	492.6	532.5	369.7	374.1	209.5	775.6	779.6	533	693	528.9	517.5	789	779	879	13
Os01g0665200	1397	1437	1228	1336	971.8	1445	655	1390	1038	1560	1529	1141	1073.3	1678.3	1919.7	2579	1438	1559	1172	1823	1571	2324	3166	2257	2070	2168	2156	2503	2289	2251	1
Os01g0114200	4.08	2.79	8.62	0.821	0	3.46	2.35	1.27	0	0.862	2.193	0	5.4426	7.4676	0.792	0	0	1.036	0	2.419	0	4.436	4.276	2.167	7.395	13.2	2.193	12	3.83	2.5	1
Os01g0683525	0	0.93	0	0.821	0	0.865	0	1.9	0	0	0	0	0	0	0	0	0	0	0	0	0	0	1.425	0	0	0	2.193	0	1.91	0.83	11
Os01g0553901	0	0	0	0	0	0	0	0	0	0.862	0	0	0	0.9335	0	0	0	1.036	1.858	0	0	0	0	0	1.056	0	0	0	0	0	16
Os01g0686200	71.4	73.6	84.1	64.04	67.8	43.25	261	314	271	143.1	159	83.73	176.34	93.345	153.64	143.3	55.65	42.48	61.31	67.73	63.8	102	139	132.2	102.5	50.91	67.98	74.9	58.3	77.5	18
Os01g0141300	0	0	0	0	0	0	0	0	0	0	0	0	0	0	0	0	0	0	0	1.613	0	0	0	0	0	0	0	0	0	0	16
Os01g0626200	495	426	413	398.2	408.6	484.4	622	562	684	480.2	550.4	414.9	439.76	491	459.34	859.7	457.6	436.2	495.1	514.4	502.7	478.4	442.5	502	383.5	480.8	557	413	409	417	6
Os01g0627967	0	2.79	0	2.463	0.904	1.73	1.57	0	0	0	0	0	0	0	0	0	0	0	0.929	0.806	2.587	0.739	2.138	2.889	0	0	0	0	0	0	12
Os01g0847200	936	873	1214	171.6	159.1	181.7	214	238	225	899.3	985.7	1007	974.23	896.11	1006.6	1146	1141	906.5	160.7	186.3	148.3	175.2	194.5	210.2	1007	975.8	1068	797	826	1002	3
Os01g0693300	396	369	494	308.7	283.8	289.8	159	195	234	415.6	237.9	181.6	259.07	280.97	395.98	549.3	301.9	374	275	315.3	326.8	241	293.6	197.9	228.2	164.1	171	268	339	290	13
Os01g0716400	1273	1090	1405	509.8	515.3	469.7	273	360	230	1130	1134	1193	1874.4	1758.6	1806.5	2066	1161	1253	497	443.5	474.2	232.2	229.5	221.7	1338	1148	1134	1385	1263	1340	3
Os01g0624000	74.4	57.7	61.4	107.5	103.1	106.4	125	86.7	76.7	63.8	69.07	112.9	68.577	82.144	76.029	47.76	60.8	66.31	157.9	170.9	144	47.32	49.88	66.45	58.1	73.54	76.75	62.9	45.9	51.7	10
Os01g0795000	207	185	175	174	120.2	198.1	31.3	46.2	37.3	25	26.31	17.88	37.01	28.937	53.854	226.9	209.2	181.3	47.38	77.4	68.98	473.2	731.9	299.7	119.4	60.34	46.05	156	143	152	17
Os01g0155200	79.5	112	101	12.31	9.04	6.055	3.13	5.06	0	158.6	93.19	79.03	64.223	52.273	80.781	71.65	87.6	88.06	13.01	19.35	21.56	17.75	13.54	26	123.6	77.31	76.75	98.1	84.2	111	3
Os01g0960200	44.9	61.5	62.5	45.15	50.62	41.52	29.7	46.8	40.1	43.11	31.8	8.467	41.364	39.205	54.646	47.76	39.16	35.23	37.16	44.35	40.52	24.4	54.16	33.95	40.14	26.4	26.32	33.3	33.5	51.7	8
Os01g0166500	5.1	4.66	3.23	0.821	0	0	0	0	0	1.724	1.096	4.704	2.177	9.3345	1.5839	11.94	6.183	6.216	1.858	0.806	1.724	3.697	0	0	4.225	14.14	2.193	7.4	6.69	17.5	13
Os01g0660850	0	0	0	0	0	0	0	0	0	0	0	0	0	0	0.792	0	0	0	0	0	0	0	0	0	0	0	0	0	0	0	4
Os01g0157700	2.04	0.93	1.08	0.821	0	0	0	0	0	0.862	0	0	0	2.8004	0	0	0	2.072	0	0	0	0	0.713	0	0	0	0	0	0	0	19
Os01g0738000	63.2	104	60.3	144.5	151.9	148.8	175	156	153	92.25	85.52	109.1	53.337	97.079	83.156	167.2	89.66	73.56	182.1	185.4	150	137.5	143.2	162.5	80.28	86.74	78.95	64.8	92.8	67.5	12
Os01g0908700	1023	1022	1015	349.7	289.3	331.3	377	487	419	286.2	233.5	210.7	289.55	237.1	293.82	429.9	490.5	493.2	259.2	320.1	344.9	572.3	592.9	468.7	232.4	244.2	274.1	269	296	344	14
Os01g0210200	0	0	0	0	0	0	0	1.9	0.69	0	0	0	2.177	0	0	0	0	0	0	0	0	0	0	0	0	0	0	0.93	0	0	4
Os01g0772200	784	780	823	759.4	736.7	787.2	756	735	746	846.7	840.9	905.1	813.12	817.7	824.44	1433	864.6	802.9	805.4	812.7	834.6	848.1	805.3	800.2	1008	1042	1013	891	922	956	13
Os01g0749300	368	325	415	248.8	259.4	236.2	209	223	236	434.5	377.2	421.5	444.12	382.72	471.22	609	366.9	329.5	269.4	279.8	287.1	230.7	227.3	227.5	401.4	364.9	372.8	370	311	356	13
Os01g0741900	560	344	484	259.4	179	218	813	1161	995	175	178.7	179.7	294.99	299.64	385.69	214.9	312.3	340.9	186.7	120.9	169.9	1102	1011	1302	226.1	251.7	370.6	271	274	247	18
Os01g0739400	450	442	547	885	976.3	891.9	480	538	572	363	387	312.4	505.07	450.86	406.28	871.7	373.1	454.8	833.3	804.7	884.6	480.6	476.8	559.7	377.1	311.1	350.9	363	423	345	10
Os01g0305900	195	201	159	179.8	130.2	177.3	64.2	93.6	91.9	164.7	169.9	112.9	95.79	155.89	164.73	537.3	292.7	215.5	111.5	203.2	192.3	327.5	468.9	342.3	239.8	404.5	407.9	347	270	290	13
Os01g0311500	133	123	119	142	113.9	128.9	151	127	91.2	125	117.3	135.5	91.436	118.55	118.79	155.2	126.8	145	131	95.95	80.19	137.5	133.3	137.9	117.3	103.7	179.8	126	100	116	11
Os01g0589200	15.3	13	22.6	33.66	37.97	15.57	41.5	41.1	25.6	18.97	27.41	11.29	22.859	17.736	19.799	11.94	11.34	23.83	38.09	19.35	12.93	44.36	79.82	27.45	28.52	21.69	21.93	21.3	23	27.5	17
Os01g0967100	1562	1469	1596	1476	1407	1378	1466	1590	1513	1436	1442	1338	1859.2	1616.7	1847.7	2520	1573	1556	1215	1159	1317	1517	1479	1443	1651	1561	1531	1722	1537	1777	13
Os01g0825800	229	186	179	241.4	279.3	221.5	78.3	90.4	139	249.2	315.8	183.5	461.53	449.92	706.43	322.4	129.8	140.9	250.8	234.6	260.4	87.99	77.68	74.39	306.3	191.4	289.5	252	339	490	4
Os01g0139200	921	992	941	2008	1953	1919	1343	1351	1432	1060	956.1	852.4	918.71	843.84	963.03	1624	845	758.4	2019	2024	2064	1431	1710	1478	941.2	893.8	940.8	999	971	906	10
Os01g0161000	1.02	5.59	7.54	11.49	20.79	15.57	63.4	47.4	18	98.29	26.31	4.704	15.239	11.201	11.879	11.94	2.061	4.144	0	1.613	3.449	5.176	7.839	0.722	9.507	0	4.386	0	3.83	2.5	7
Os01g0721100	157	174	198	197	178.1	199	225	196	205	181.1	177.6	147.7	167.63	174.56	192.45	406	160.8	159.5	176.5	206.4	175	222.6	220.2	192.8	209.2	197.1	151.3	221	254	255	6
Os01g0642600	0	0	1.08	0	0	0	0	0	0	0.862	2.193	0.941	0	0	3.1679	0	1.031	0	0	0	0	0	0	0	0	0.943	0	0.93	0.96	0	4
Os01g0590200	560	456	497	551.7	542.4	581.3	479	472	517	454.4	482.4	604	424.52	499.4	441.13	680.6	423.5	431	580.6	448.3	580.3	496.1	503.1	496.2	527.1	530.8	519.7	403	466	363	6
Os01g0268100	58.1	65.2	49.6	27.09	31.64	25.95	29	59.5	53.2	25.87	47.14	31.99	46.806	69.075	46.726	95.53	55.65	47.66	14.86	22.58	6.036	70.98	85.52	51.28	54.93	36.77	41.67	58.3	40.2	70.8	13
Os01g0209800	15.3	21.4	25.9	24.63	27.12	24.22	103	98	84.3	62.94	38.37	63.03	38.098	35.471	40.39	11.94	24.73	44.55	78.96	34.67	44.84	34.75	76.96	38.28	32.75	17.91	37.28	43.5	24.9	31.7	18
Os01g0881000	5.1	0.93	1.08	3.284	10.85	0.865	8.61	37.3	10.4	3.449	5.482	2.822	0	1.8669	3.1679	11.94	2.061	1.036	1.858	1.613	6.036	4.436	6.414	2.889	7.395	1.886	4.386	13.9	4.78	2.5	18
Os01g0644000	254	412	454	234.8	311	205.9	292	311	601	110.4	196.3	18.82	673.79	245.5	240.76	429.9	18.55	23.83	142.1	50.8	9.484	21.44	100.5	54.17	42.25	12.26	15.35	77.7	214	91.6	4
Os01g0221400	1067	1159	1290	431.8	461.9	762.1	86.9	114	96.1	544.9	497.8	399.8	527.93	869.04	456.17	800	1567	1317	738.5	466.8	812.2	54.71	81.95	91	545.1	599.6	585.5	823	558	597	14
Os01g0120500	2431	2478	2815	881.7	960	936.8	1013	1167	1155	3546	3780	4102	4127.7	3999.8	4232.3	6006	3569	3166	1273	1299	1305	698.7	713.3	866	3927	3681	3568	3024	3360	3310	3
Os01g0817100	838	910	788	415.4	428.5	361.6	523	694	376	764.8	811.3	673.6	769.58	777.57	973.33	1636	982.1	766.7	347.4	379	325.9	485.8	702.7	470.2	1140	946.6	984.6	1291	896	1094	13
Os01g0878300	3396	2480	3748	651.9	591.2	586.5	290	247	191	1367	1418	939.8	2862.8	2206.7	2936.6	3212	1872	2215	729.2	567.6	658.7	312	219.5	231.8	1362	1235	850.9	1515	1686	2119	13
Os01g0101800	2.04	0.93	0	0	0	0	0.78	0.63	0.69	1.724	1.096	0.941	0	0	1.5839	0	0	0	0	0	1.724	1.479	0.713	0	5.282	5.657	0	0.93	0.96	6.66	1
Os01g0934800	23.4	20.5	21.6	22.99	64.18	27.68	9.39	16.4	20	29.31	28.51	11.29	18.505	22.403	20.591	23.88	20.61	11.4	30.66	30.64	27.59	32.53	20.67	14.44	27.47	31.11	15.35	21.3	23.9	13.3	2
Os01g0727900	22.4	24.2	17.2	29.56	28.93	43.25	50.1	53.1	59.4	36.21	27.41	25.4	26.124	21.469	23.759	35.82	21.64	20.72	33.44	52.41	45.7	36.23	29.93	32.5	21.13	19.8	19.74	17.6	27.7	10.8	12
Os01g0761900	0	0	0	2.463	0	0	0.78	0	0	0	0	0	0	0	0	0	0	0	0	0	0	0.739	0.713	0	0	0	0	0	0	0	2
Os01g0178400	103	124	97	30.38	25.31	30.28	71.2	46.2	54.6	110.4	125	95.96	95.79	60.674	103.75	107.5	80.38	115	16.72	18.54	30.18	75.42	37.77	57.78	95.07	98.06	50.44	64.8	58.3	70	3
Os01g0805600	1.02	13	5.39	0	0.904	6.92	1.57	0	1.38	0	0	6.586	0	3.7338	4.7518	0	6.183	3.108	0.929	1.613	6.036	2.958	4.276	0	2.113	9.428	2.193	0	1.91	2.5	14
Os01g0784500	53	41.9	56	57.47	39.77	44.12	39.9	48.1	65.6	41.38	29.6	43.28	35.921	44.806	45.142	59.7	42.25	27.97	55.74	47.57	37.08	89.47	100.5	89.58	28.52	36.77	59.21	58.3	47.8	54.2	17
Os01g0241100	147	168	131	193.8	186.2	203.3	113	130	108	108.6	133.8	116.7	95.79	114.81	117.21	406	152.5	141.9	159.8	154	187.1	117.6	160.3	103.3	126.8	148	151.3	170	177	142	6
Os01g0113900	3.06	6.52	2.16	1.642	0	3.46	0	1.27	0	3.449	5.482	2.822	3.2656	0.9335	8.7116	23.88	3.092	5.18	4.645	4.031	5.173	1.479	2.138	0.722	3.169	0.943	0	2.78	6.69	8.33	6
Os01g0152300	118	99.7	105	50.9	66.89	43.25	146	125	179	109.5	84.42	101.6	70.754	70.009	75.237	71.65	128.8	101.5	105.9	88.69	79.32	129.4	124.7	142.3	64.44	66.94	87.72	70.3	58.3	67.5	18
Os01g0142200	253	224	178	209.3	198.9	187.7	143	123	118	191.4	232.4	262.5	243.83	253.9	220.17	585.1	230.8	232.1	201.6	192.7	253.5	142	140.4	127.1	257.8	261.2	223.7	193	222	229	13
Os01g0304500	0	0	0	0	0	0	0	0	0	0	0	0	0	0	0	0	1.031	0	0.929	0	0	0	0	0	4.225	0	0	0	0	1.67	1
Os01g0521500	76.5	65.2	63.6	67.32	45.2	46.71	120	94.2	103	87.08	102	89.38	81.639	106.41	70.485	47.76	77.29	96.35	64.1	79.82	92.26	66.54	66.28	78.72	77.11	84.86	175.4	71.2	77.5	83.3	11
Os01g0531200	336	277	310	330.9	380.6	314.9	207	253	256	306.1	284	281.3	371.19	254.83	309.66	632.9	320.5	312.9	289.8	284.6	241.4	310.5	302.2	277.3	282	272.5	263.2	316	301	294	6
Os01g0760300	0	0	0	0	0	0	0	0	0	0	0	0	0	0	0	0	1.031	0	1.858	0.806	0	0	0	0.722	0	0	0	0	0.96	0	16
Os01g0631400	6.12	11.2	5.39	3.284	1.808	0.865	3.13	5.06	4.15	2.587	1.096	5.645	2.177	4.6673	4.7518	11.94	4.122	5.18	1.858	0	0	10.35	12.83	9.389	12.68	16.03	19.74	22.2	16.3	6.66	1
Os01g0530400	545	482	559	592.7	621.9	613.3	682	739	658	590.6	573.4	682.1	689.03	738.36	598.73	859.7	528.7	550.1	759.9	661.2	770.8	537.5	568.7	577.8	526.1	582.7	589.9	482	535	545	6
Os01g0919500	457	363	391	278.3	241.4	289.8	243	225	272	352.6	322.3	344.3	427.79	372.45	380.94	561.2	445.2	451.7	280.5	308.8	275	291.3	283.6	291.1	395.1	401.7	412.3	396	392	410	13
Os01g0855900	129	59.6	92.7	143.7	123.8	147.1	114	79.7	96.1	92.25	86.62	130.8	93.613	122.28	108.5	226.9	116.5	117.1	143.1	140.3	144	105	91.93	88.84	86.62	99.94	92.1	123	146	81.6	6
Os01g0588700	503	446	498	442.5	514.4	477.5	623	592	690	557.8	542.7	580.5	481.13	509.66	494.19	800	490.5	440.3	534.1	503.9	559.6	414.8	424	458.6	462.7	518.6	524.1	485	503	428	6

Os01g0708350	433	564	564	1202	1234	1142	302	377	371	329.4	327.8	192.9	408.19	372.45	431.62	728.4	332.9	415.5	518.4	591	537.2	620.3	592.9	490.4	390.9	290.4	328.9	439	379	486	2
Os01g0295600	0	0	0	0	0	0.865	0	0.63	0	0	0	2.822	0	0	0	0	0	1.036	0	0	0	1.479	2.138	0	0	0	2.193	0	0	0	11
Os01g0799000	40.8	31.7	51.7	15.6	15.37	9.516	14.1	13.3	6.22	14.66	16.45	3.763	14.151	11.201	21.383	23.88	17.52	41.44	4.645	17.74	18.11	23.66	26.37	11.56	21.13	10.37	6.579	15.7	17.2	27.5	14
Os01g0869600	1448	1136	1376	660.9	687.9	724	928	1029	864	836.3	890.3	974.7	1119	1097.7	1065.2	2305	1453	1372	818.4	1015	851	1039	1138	1099	1140	1078	945.1	1301	1063	1318	13
Os01g0767600	1004	918	913	407.2	337.2	527.7	50.1	26.6	30.4	1273	1244	1106	1030.8	913.85	1047.8	1051	819.3	799.8	462.6	381.4	375.9	26.62	19.24	23.83	967.6	825.9	914.4	710	647	707	3
Os01g0131300	2410	2029	2352	1622	1575	1477	1877	2068	2211	3471	3422	2967	2464.4	2721	2747.3	4705	2235	2305	1743	1680	1791	2169	2175	2108	2524	2564	2088	2404	2440	2577	13
Os01g0822001	1.02	0	0	0	0	0	1.57	0.63	0	0	0	0	0	0	0	0	0	2.072	0	0	1.724	2.218	1.425	2.167	1.056	0	0	0	0	0	9
Os01g0572966	0	0	0	0	0.904	0	0	0	0	0	0	0	0	0	0	0	0	0	0	0	0	0	0	0	0	0	0	0	0	0	2
Os01g0286100	1038	904	973	1090	1210	721.5	693	602	788	1386	1755	808.2	2110.6	1061.3	1753.4	2699	840.9	1084	914.1	1055	611.3	537.5	536.6	382.1	1260	807.1	513.1	840	1414	1183	13
Os01g0642801	1.02	5.59	3.23	9.031	9.04	4.325	11.7	6.33	8.29	10.35	2.193	1.882	9.7967	3.7338	12.671	0	3.092	3.108	13.01	9.675	0	19.96	21.38	19.5	5.282	3.771	0	13.9	12.4	6.66	17
Os01g0203800	545	501	629	133.8	104	121.1	35.2	54.4	31.1	281.9	297.1	180.6	391.87	444.32	651.79	991.1	450.3	495.2	75.25	100.8	75.88	127.2	159.6	71.5	455.3	468.6	357.4	931	826	895	13
Os01g0175000	1571	1535	1531	1309	1463	1245	1841	1756	1619	1944	1852	2429	1559.8	1563.5	1454	2520	1661	1449	1625	1532	1449	1213	1174	1447	1782	1836	1921	1359	1325	1346	20
Os01g0673400	0	0	0	0	0	0	0	0	0	0	0	0	0	0	0	0	2.061	0	0	0	0	0	0	0	0	0	0	0	0	0	1
Os01g0894000	298	318	293	291.4	274.8	276.8	221	213	225	230.2	239	236.1	218.79	256.7	300.16	561.2	282.4	251.8	278.7	244.3	255.2	251.4	253.7	290.3	250.4	250.8	210.5	315	265	312	6
Os01g0724500	199	215	235	288.2	284.8	254.3	268	254	253	237.1	216	147.7	217.7	191.36	247.09	417.9	176.2	214.5	222	244.3	228.5	324.6	270.1	226.8	207	179.1	197.4	264	251	282	6
Os01g0756400	1007	1018	985	922	827.1	923.9	1568	1445	1532	1164	1174	1136	1268.1	1205.1	1168.1	1851	1193	1072	1045	1097	1027	1329	1259	1357	1167	1157	1162	1106	1024	1001	6
Os01g0104900	5.1	4.66	4.31	1.642	4.52	1.73	3.91	1.9	0	0.862	0	2.822	6.5311	10.268	4.7518	11.94	4.122	2.072	0.929	0	1.724	0	0	0	1.056	3.771	4.386	8.33	2.87	1.67	13
Os01g0142600	0	11.2	6.47	10.67	20.79	6.92	6.26	22.1	11.7	4.311	0	0	2.177	1.8669	3.1679	11.94	1.031	2.072	3.716	4.031	0.862	14.05	20.67	10.83	1.056	0.943	2.193	5.55	7.65	7.5	17
Os01g0139000	3.06	4.66	1.08	1.642	0	3.46	0	8.86	2.07	0.862	8.771	9.408	6.5311	1.8669	1.5839	0	5.153	6.216	2.787	7.257	1.724	1.479	8.552	2.167	4.225	5.657	13.16	0	0.96	1.67	11
Os01g0601687	0	0	0	0	0	0	0	0	0	0	0	0	0	0	0	0	0	0	0	0	0	0	0	0	0	0	0	0	0.96	0	1
Os01g0518300	0	0	0	0.821	0.904	0.865	0	1.9	0	1.724	0	2.822	0	0	0.792	11.94	1.031	0	0.929	0	0	0	2.851	0	0	0.943	4.386	0.93	1.91	0	6
Os01g0821800	658	731	668	656	750.3	682.5	733	732	743	608.7	595.3	701.9	583.45	677.69	566.26	991.1	559.6	615.4	684.6	562.8	586.3	585.6	670.6	618.2	630.6	547.8	675.4	544	509	543	6
Os01g0867600	332	334	337	266.8	309.2	253.5	396	364	337	273.3	254.4	250.3	361.39	323.91	373.02	453.8	346.3	366.8	228.5	222.5	227.6	278.7	299.3	249.2	307.4	263.1	302.6	327	303	311	13
Os01g0369500	1.02	0	0	0	0	0	0	0	0	0	0	0	0	0	0	0	0	0	0	0	0	0	0.713	0	0	0	0	0	0	0	19
Os01g0521600	0	0	0	0	0.904	0	0	0	0	0	0	0.941	0	0	0	0	0	0	0	0	0	0	0	0	0	0	0	0	0	0	20
Os01g0300000	0	0	0	0	0	0	0	0	0	0	0	0	0	0	0	0	0	0	2.787	0	0	0	0	0	0	0	0	0	0	0	16
Os01g0134900	5.1	0	2.16	0.821	0	0.865	4.7	1.9	6.22	0.862	6.578	13.17	4.3541	0	0.792	0	3.092	0	1.858	0.806	0	0.739	2.138	2.167	0	5.657	2.193	5.55	3.83	1.67	20
Os01g0533000	36.7	31.7	28	82.1	71.41	76.99	93.9	132	80.2	36.21	36.18	34.81	32.656	22.403	16.631	71.65	26.79	33.15	38.09	44.35	33.63	125.7	91.93	111.2	31.69	35.83	32.89	46.3	42.1	46.7	18
Os01g0246700	976	1010	950	688	609.3	519.9	853	691	842	427.6	313.6	253.1	312.41	320.17	384.1	1230	668.8	562.6	699.5	745.8	751	715.7	813.1	665.2	281	312.1	364	389	431	433	6
Os01g0125600	26.5	41.9	29.1	47.62	33.45	39.79	33.7	29.7	24.9	20.69	27.41	11.29	21.77	20.536	16.631	35.82	26.79	36.26	39.94	16.13	24.14	75.42	59.15	44.78	19.01	26.4	26.32	57.4	35.4	34.2	17
Os01g0192600	0	3.73	2.16	0.821	1.808	5.19	3.91	1.9	0.69	0.862	2.193	0.941	0	4.6673	0.792	0	2.061	0	3.716	1.613	0	2.218	0.713	1.444	7.395	0.943	8.772	0.93	6.69	0.83	11
Os01g0977901	0	0	0	0	0	0	0.78	0	0	0.862	0	0	0	0	0	0	0	0	0.929	0	0	0	0	0	0	0	0	0	0	0	7
Os01g0533100	2.04	1.86	0	9.852	12.66	9.516	17.2	11.4	15.9	0.862	2.193	0	2.177	1.8669	1.5839	0	2.061	3.108	10.22	7.257	11.21	15.53	22.8	13	1.056	3.771	13.16	4.63	3.83	6.66	12
Os01g0642200	3247	2310	2987	3933	3506	3066	3413	4344	2582	6013	4790	7249	4875.5	6526.7	5924.7	3952	3984	4182	4668	1545	1526	2820	3354	2929	5036	10759	16328	4006	5918	4792	11
Os01g0872100	0	0	1.08	102.6	97.63	89.96	8.61	3.8	8.29	0	0	0	0	1.8669	0	0	0	0	103.1	95.95	94.84	7.394	5.701	7.945	0	0	0	0	0	0	10
Os01g0871200	####	####	####	7265	6193	6783	5455	6851	5715	16483	18870	14872	14997	11787	18892	21183	12462	13531	5221	5305	5958	6581	6649	7697	15254	15220	12530	####	####	####	3
Os01g0585600	102	127	129	92.77	122	82.18	42.3	71.5	44.9	61.22	61.4	34.81	74.019	69.075	72.861	143.3	74.2	111.9	49.23	54.02	31.9	127.2	101.9	70.78	76.06	71.66	46.05	114	79.4	146	8
Os01g0266600	823	781	838	862.8	1069	834.8	1420	1343	1165	770.8	700.6	906	791.35	775.7	618.53	1027	725.5	769.8	1038	1057	996.7	659.5	751.1	993.8	522.9	603.4	662.3	624	582	613	12
Os01g0562400	6.12	6.52	2.16	4.105	2.712	6.92	0.78	1.9	1.38	2.587	9.868	0	4.3541	0	3.1679	11.94	3.092	3.108	3.716	8.869	6.036	11.83	14.25	13	5.282	2.829	4.386	6.48	0.96	0.83	9
Os01g0130400	32.6	45.6	31.2	49.26	47.91	63.15	11.7	8.86	7.6	13.79	19.74	31.99	11.974	14.002	5.5438	23.88	34.01	33.15	78.96	72.57	80.19	7.394	3.563	4.333	12.68	33.94	17.54	11.1	9.56	8.33	16
Os01g0248400	207	201	213	128.9	121.1	141.9	154	159	175	201.8	202.8	179.7	160.01	202.56	213.83	83.59	195.8	212.4	138.4	141.9	113.8	191.5	198.8	187.8	194.4	186.7	201.7	225	208	189	1
Os01g0847800	390	382	442	413.8	446.6	415.2	404	373	428	515.6	378.3	439.4	333.09	503.13	372.22	644.8	404	415.5	653.1	503.9	530.3	269.1	340.6	301.9	344.4	366.8	403.5	420	357	402	16
Os01g0243100	117	81	100	110.8	120.2	109	177	264	225	111.2	89.9	168.4	180.69	131.62	151.27	179.1	90.69	125.4	176.5	151.6	144	172.3	145.4	177.7	132	83.91	98.68	115	116	120	18
Os01g0674700	2050	2229	2185	3176	3617	3074	2007	2475	2101	1664	2095	1819	2664.7	2189.9	1971.2	2782	1707	1984	2583	2531	2713	1682	1764	1811	1788	1474	1513	1527	1556	1744	10
Os01g0893700	1.02	0	0	0	0	0	0	0	0	0	0	0	0	0	0	0	0	0	0.929	0	0	0.739	0	0	0	0	0	0	0	0	19
Os01g0618300	0	0	0	0	0	0	0	0	0	0	0	0	0	0	0	0	0	0	0	0	0	1.479	0	0	0	0	2.193	0	0	0	11
Os01g0958000	1200	1169	1205	1009	932.9	996.5	1285	1290	1316	1097	1105	1098	1057	1112.7	1205.4	1612	1055	1185	994	907.9	1003	1337	1253	1282	1178	1291	1243	1237	1086	1060	6
Os01g0778100	4.08	2.79	4.31	35.3	53.33	38.93	46.2	25.3	19.3	7.76	6.578	10.35	9.7967	13.068	4.7518	0	5.153	2.072	57.6	81.43	109.5	49.54	29.93	34.67	8.451	8.486	4.386	15.7	8.61	5	10
Os01g0938900	1058	1030	894	1401	1285	1430	1614	1267	1366	1245	995.5	1507	1025.4	1119.2	1007.4	1731	1036	956.3	1554	1286	1534	1272	1172	1213	1039	1180	1368	908	907	943	15
Os01g0950800	257	265	245	248.8	220.6	254.3	329	323	345	281.1	271.9	365	246.01	292.17	274.02	334.3	271	268.3	284.3	330.6	317.3	360.8	357	329.3	304.2	344.1	315.8	258	259	261	5
Os01g0148050	2302	2594	2517	2678	2645	2446	2468	2631	2257	2130	2297	1899	2493.8	2269.2	2602.4	3738	2140	2469	1791	2133	1931	2847	2619	2406	2357	2121	2017	2523	2434	2748	6
Os01g0610800	8.16	10.2	2.16	9.852	8.136	17.3	6.26	5.69	9.67	10.35	3.289	10.35	4.3541	9.3345	11.879	35.82	12.37	6.216	16.72	12.9	22.42	2.958	13.54	6.5	7.395	7.543	10.96	6.48	4.78	5.83	6
Os01g0917200	21.4	29.8	23.7	37.76	18.98	44.12	15.7	20.2	2.76	18.11	13.16	31.99	15.239	19.602	14.255	59.7	37.1	33.15	28.8	22.58	49.15	33.27	37.06	52	41.2	47.14	48.24	43.5	35.4	17.5	11
Os01g0504500	495	643	633	360.4	353.5	269	582	574	734	729.4	726.9	470.4	661.82	462.06	687.43	907.5	418.4	410.3	237.8	238.7	119	508.7	656.3	397.2	449	348.9	339.9	476	509	633	5
Os01g0856600	250	245	291	451.5	484.5	496.5	243	192	233	244.9	214.9	226.7	332	363.11	266.1	549.3	280.3	295.3	471.9	522.5	501.8	194.5	210.2	195	194.4	215.9	182	251	268	255	10
Os01g0199900	491	499	538	913.7	1071	891	537	643	528	434.5	435.3	381	531.2	581.54	513.99	1230	636.9	604	750.6	887.7	823.4	515.4	498.8	434.8	398.2	389.4	355.3	534	587	552	10
Os01g0697250	0	0	0	0	0.904	0	0	1.27	0	0	0	0	0	0	0	0	0	0	0	0	0	0	0	0	0	0	0	0	0	0	2
Os01g0167500	1053	1295	1153	499.2	464.6	673	783	574	737	1431	1288	1247	1047.2	1094	1118.3	1887	947.1	987.3	511.9	511.2	485.4	668.4	746.1	809.6	1380	1230	1217	1056	1201	1238	13
Os01g0153100	879	745	690	620.7	777.4	560.6	1491	1329	1258	1280	1141	1186	849.05	663.68	696.93	1540	839.9	679.6	1038	990.1	912.2	681.7	963.5	882.6	790.2	810.8	828.9	657	743	650	5
Os01g0213350	0	0	0	0	0	0	0	0	0	0	0	0	0	0	0	0	0	0	0	0	0	0	0	0	0	0	0	0	0	0.83	1
Os01g0332800	4582	5061	5166	3751	3756	3670	1718	1858	1940	6022	6755	8684	8438.2	6854.3	7031.1	11714	5447	5523	6253	5373	6448	1587	1152	2511	8300	7407	6506	6081	5659	6812	13
Os01g0198000	154	200	157	133	184.4	116.8	135	133	107	127.6	125	127	105.59	119.48	121.96	155.2	129.8	162.7	69.67	135.5	78.46	110.9	112.6	100.4	121.5	117.9	116.2	147	161	132	8
Os01g0841100	13.3	9.31	10.8	18.88	14.46	15.57	12.5	7.59	2.76	1.724	8.771	17.88	11.974	8.4011	6.3357	23.88	11.34	16.58	26.94	12.9	16.38	18.48	16.39	17.33	2.113	3.771	13.16	4.63	5.74	4.17	10
Os01g0919100	11.2	4.66	4.31	5.747	0	0	0	1.27	0	0.862	3.289	0	0	5.6007	1.5839	0	0	1.036	0	4.838	0	4.436	0.713	1.444	2.113	2.829	2.193	0	3.83	2.5	19
Os01g0109432	7.14	2.79	2.16	6.568	2.712	2.595	4.7	2.53	0	0	4.386	1.882	0	6.5342	2.3759	0	1.031	6.216	3.716	2.419	1.724	2.958	4.276	2.889	2.113	1.886	4.386	4.63	1.91	1.67	19
Os01g0663500	336	276	303	561.5	605.7	586.5	493	546	513	405.2	335.5	334	341.8	357.51	345.3	477.6	295.8	302.5	602	603.9	542.3	468.8	460.4	412.4	337	310.2	287.3	315	295	262	12
Os01g0215100	27.5	20.5	28	34.48	23.5	15.57	76.7	68.3	80.8	41.38	40.57	50.8	90.347	40.138	37.222	71.65	29.89	40.41	38.09	33.86	43.11	42.15	45.61	53.45	46.48	30.17	26.32	24.1	36.3	32.5	5
Os01g0169400	0	0	0	0	0	0	0	0	0	0	1.096	0	0	0	0	0	0	1.036	0	0	0	0.739	0	0	0	0	0	0	0	0	7
Os01g0780800	301	291	355	249.6	298.3	188.6	246	319	294	246.6	271.9	266.3	301.52	219.36	251.85	465.7	223.6	222.7	162.6	222.5	199.2	243.3	267.2	193.6	202.8	195.2	162.3	182	238	216	6
Os01g0893300	14.3	13	14	13.14	12.66	15.57	11.7	15.2	22.1	12.93	18.64	15.05	6.5311	16.802	11.088	11.94	23.7	6.216	18.58	12.09	12.93	31.05	27.08	14.44	22.18	19.8	21.93	12	22	7.5	17
Os01g0948300	1197	1286	1179	1188	1225	1151	1747	1626	1550	1347	1422	1438	1309.5	1364.7	1355.1	1827	1234	1203	1331	1247	1284	1328	1378	1540	1354	1274	1436	1238	1063	1183	5
Os01g0942300	0	1.86	1.08	1.642	1.808	5.19	4.7	0	4.84	5.173	2.193	0	0	0	0	0	0	0	0.929	0.806	4.311	4.436	9.977	3.611	0	2.829	0	0	0.96	0	17
Os01g0872450	0	0	0	0	0	0	0.78	0	0	0	0	0	0	0	0	0	0	0	0	0	0	0	0	0	0	0.943	0	0.93	0	0	1
Os01g0927600	5976	5832	6149	4567	4398	4266	6379	5840	5816	5737	5500	5128	4840.6	4720.5	5124	9553	5530	5839	4274	4567	4680	5760	5064	5788	4922	5378	5109	5280	5249	5124	6
Os01g0510100	2187	2093	2182	1548	1586	1480	1019	1156	1104	1642	1437	1278	1402	1642.9	1606.1	3188	1927	1978	1318	1345	1364	1150	1176	992.4	1473	1381	1430	1617	1664	1586	13
Os01g0761100	172	175	165	230.7	196.2	208.5	236	261	336	221.6	201.7	179.7	205.73	179.22	225.71	298.5	162.8	188.6	178.4	189.5	201.8	326.8	342.1	330.1	212.3	187.6	201.7	167	155	167	18
Os01g0326300	106	113	200	10.67	0.904	11.25	15.7	3.8	81.5	25	39.47	12.23	27.213	18.669	21.383	71.65	19.58	15.54	7.432	1.613	1.724	202.6	36.34	242.7	29.58	8.486	4.386	60.1	44	23.3	9
Os01g0141000	16.3	20.5	30.2	8.21	5.424	6.055	50.1	3.16	182	18.97	35.08	26.34	26.124	21.469	10.296	47.76	11.34	6.216	15.79	20.96	7.76	607	295	550.4	28.52	2.829	2.193	25.9	30.6	32.5	9
Os01g0524500	171	344	273	423.6	393.2	352.1	696	666	717	470.8	510.9	608.7	701.01	472.33	489.43	680.6	336	369.9	539.7	539.4	563	505	357	600.9	435.2	537.4	390.3	359	379	432	15
Os01g0583200	182	166	172	222.5	230.5	204.2	231	245	286	177.6	218.2	213.6	204.64	218.43	182.15	274.6	148.4	231	215.5	208.8	262.1	232.2	205.2	174.1	163.7	193.3	179.8	138	154	164	12
Os01g0603000	665	633	665	1068	1061	1075	792	769	755	716.5	650.2	813.8	780.47	768.23	654.16	1230	581.2	665.1	979.1	884.5	878.6	675.1	649.9	622.6	625.4	720.3	633.8	587	675	696	10
Os01g0237750	0	0	0	3.284	2.712	1.73	0	0.63	0	0	2.193	0	1.0885	0	1.5839	0	0	1.036	0	0.806	0	1.479	2.138	0	1.056	0	0	0.93	0.96	0.83	2
Os01g0147300	0	0	0	0	0	0	0	0	0	0	0	0	0	0	0	0	0	0	0	0	0	0	0	0	0	0	0	0.93	0	0	1
Os01g0950000	1.02	1.86	3.23	3.284	0.904	2.595	3.91	2.53	1.38	0	0	0	3.2656	0	0.792	0	3.092	0	1.858	0.806	0.862	6.654	9.264	2.167	0	0.943	0	0.93	0	1.67	17
Os01g0913900	2.04	1.86	0	0.821	0.904	0	1.57	0	0.69	0	0	1.882	0	0	0	0	0	0	0	0	0	0	0	0	0	0	0	0	0.96	0	19
Os01g0945800	1529	1429	1464	1338	1397	1332	1146	1197	1035	1411	1391	1350	1486.9	1520.6	1544.3	2352	1572	1549	1462	1429	1531	1237	1281	1267	1523	1546	1485	1627	1562	1570	13
Os01g0256900	375	397	442	392.4	386.9	461.1	435	386	426	437.1	410.1	467.6	449.56	428.45	399.15	585.1	432.8	349.1	523	426.5	419.9	334.9	305	382.1	454.2	457.3	421	373	337	373	6
Os01g0366100	462	472	512	554.2	666.2	654	309	278	328	367.3	405.7	391.4	481.13	615.14	416.57	835.9	402.9	553.2	513.7	533	421.6	454	407.6	313.5	343.3	422.4	443	484	533	447	6
Os01g0818700	229	266	269	654.3	654.5	659.2	26.6	29.7	24.2	87.08	76.75	25.4	55.515	78.41	111.67	394.1	294.7	223.8	199.7	341.9	294	161.9	165.3	104.7	156.3	215.9	153.5	285	228	277	2
Os01g0897500	311	284	280	326.7	283.8	274.2	337	339	408	309.5	328.9	300.1	336.35	316.44	303.32	501.5	316.4	305.6	320.5	367.7	364.7	320.9	321.4	357.5	315.9	374.3	394.7	326	325	298	6
Os01g0830500	314	275	291	137.9	127.5	99.48	168	259	214	158.6	130.5	221.1	220.97	208.16	214.62	322.4	273.1	243.5	75.25	87.88	76.74	281.7	275.1	242.7	218.7	192.3	221.5	240	227	262	14
Os01g0944200	9854	####	####	9018	8982	9430	9926	####	####	10648	11714	10006	11538	9731.2	11799	18114	10773	10634	8513	9862	8229	12771	11270	10293	12863	11329	10726	####	####	####	13
Os01g0383700	####	8549	####	4270	3875	4180	1956	2354	2138	5402	6075	5710	10405	9881.5	11175	24610	12479	10617	5939	5796	5610	2507	2459	2280	7587	7236	7166	####	####	####	13
Os01g0222700	158	119	137	186.4	153.7	241.3	244	256	294	196.6	237.9	187.2	253.63	206.29	251.85	167.2	108.2	116	123.6	109.7	108.6	410.4	346.3	359.7	284.2	220.6	234.6	166	166	190	9
Os01g0390300	386	433	418	394.1	425.8	411.8	459	470	422	376.8	442.9	467.6	537.73	520.87	616.15	931.4	550.3	463.1	356.7	481.4	486.3	496.1	540.2	436.2	432.1	479	429.8	516	625	618	13
Os01g0721500	110	112	106	124	132	120.2	111	140	120	92.25	111.8	84.67	119.74	113.88	108.5	203	120.6	127.4	118.9	135.5	133.6	140.5	155.4	123.5	123.6	112.2	125	163	177	151	6
Os01g0201850	2.04	0	0	0	0	0	0	0	0	0	0	0	0	0	0	0	1.031	0	1.858	0.806	0	0	0	0	0	0	0	0	0	0	19
Os01g0877450	4.08	1.86	0	4.105	0.904	1.73	0.78	6.96	4.84	0	0	5.645	0	1.8669	3.9598	0	1.031	2.072	0	3.225	6.036	2.958	4.276	5.778	5.282	3.771	0	4.63	5.74	7.5	1
Os01g0925200	803	829	824	1298	1231	1165	1437	1340	1245	1236	1106	1240	1049.3	1005.3	1002.6	1493	782.2	881.7	1761	1912	1799	970.8	1105	1101	1050	1047	1149	865	838	764	10
Os01g0201400	0	0	0	0.821	1.808	1.73	0	0	0	0	0	0	0	0	0	0	0	0	0.929	1.613	0	0	0	0	0	0	0	0	0	0	10
Os01g0901800	179	183	207	124	114.8	147.1	304	359	303	190.5	208.3	159.9	189.4	166.15	215.41	453.8	159.7	176.1	160.7	203.2	156.1	332.7	355.6	366.9	236.6	168.8	133.8	241	246	237	6
Os01g0860300	339	289	287	348.1	380.6	389.3	299	308	353	253.5	221.5	409.3	301.52	355.65	212.25	561.2	269	281.8	459.8	392.7	472.5	323.9	312.8	256.4	272.5	345.1	403.5	235	274	192	6
Os01g0730700	7.14	7.45	3.23	50.08	63.28	25.09	25.8	67	29.7	13.79	10.96	15.99	7.6196	9.3345	13.463	11.94	4.122	6.216	59.45	45.15	20.69	26.62	71.26	15.89	7.395	2.829	4.386	6.48	12.4	15.8	12
Os01g0574600	449	449	477	575.5	707.8	594.3	833	832	843	466.4	525.2	557.9	472.42	463.93	437.96	597	360.7	436.2	605.7	682.1	731.2	587.1	515.9	688.3	393	402.6	331.1	340	401	427	12
Os01g0373200	2.04	0	1.08	0	0	0	0	1.27	0	0	2.193	0.941	2.177	2.8004	2.3759	0	1.031	2.072	1.858	3.225	0	2.218	1.425	0	0	0	2.193	3.7	3.83	2.5	1
Os01g0910900	####	####	####	35048	34905	25085	####	####	####	25891	31452	24597	40012	55639	68462	#####	33677	39731	26015	18677	19470	28984	33030	22407	45513	26655	27304	####	####	####	13
Os01g0834600	1070	932	892	1110	1150	1148	1409	1064	1522	1112	934.1	1771	1021	1256.4	825.23	1051	852.2	915.9	1420	1087	1471	913.1	1063	1068	1073	1292	1627	661	886	558	15
Os01g0762000	211	277	217	408	444.8	337.4	551	707	681	288	275.2	284.1	236.21	242.7	293.82	429.9	198.9	223.8	295.4	256.4	279.4	604.8	564.4	502.7	248.2	271.5	203.9	246	211	260	18
Os01g0691200	0	4.66	0	1.642	1.808	0.865	6.26	0.63	2.07	0.862	0	1.882	0	0.9335	2.3759	0	2.061	0	0	1.613	0	1.479	1.425	1.444	0	0	0	0.93	0	0	18
Os01g0196300	1.02	0	1.08	4.926	6.328	0.865	5.48	3.16	6.22	3.449	1.096	10.35	4.3541	5.6007	7.1277	11.94	0	0	1.858	5.644	0	0	4.988	2.167	2.113	1.886	0	1.85	1.91	2.5	6
Os01g0719000	18.4	25.1	22.6	7.389	11.75	9.516	21.1	8.22	18.7	19.83	33.99	17.88	19.593	43.872	26.135	47.76	42.25	30.05	8.361	17.74	10.35	17.75	7.126	7.222	54.93	25.46	10.96	23.1	25.8	28.3	13
Os01g0180600	177	176	196	292.3	348.9	303.6	126	149	115	98.29	128.3	97.85	120.83	145.62	148.1	238.8	121.6	206.2	209	250.8	279.4	182.6	139.7	157.4	136.3	113.1	105.3	190	191	197	10
Os01g0541800	60.2	39.1	35.6	66.5	18.98	99.48	22.7	31	32.5	31.9	43.86	38.57	20.682	38.272	30.887	11.94	30.92	33.15	52.02	55.63	131.9	306.8	297.2	278.1	89.79	98.06	193	52.7	46.9	39.2	9
Os01g0269800	0	0	1.08	1.642	0	0	0	0.63	0	0	0	0	1.0885	0	0	0	0	2.072	0	0	0	0	0	0	0	0	0	0	0.96	0.83	8
Os01g0913600	0	0	0	0.821	0	0	0	0	0	0	0	0	0	0	0	0	0	0	0	0	0	0	0	0	0	0	0	0	0	0	2
Os01g0769200	1.02	1.86	0	0	0.904	2.595	0	0.63	0	0	0	0	0	0	0	0	0	0	0	0	0	0.739	0	0.722	0	0	0	0.93	0	0.83	2
Os01g0232800	900	1007	927	1136	1114	1086	1473	1213	1346	997.5	1031	1007	793.53	894.25	878.29	1290	712.1	723.2	1135	1001	1080	1157	1167	1374	852.5	813.7	991.2	809	815	756	18

Os01g0153400	476	483	514	646.1	782.8	753.5	485	490	473	487.1	523	559.8	544.26	458.32	436.37	871.7	417.4	505.6	565.7	665.2	592.3	524.2	422.6	491.8	476.4	487.4	364	414	426	447	6
Os01g0393300	0	0	0	0	0.904	0	0	0	0	0	0	0	0	0	0	0	0	0	0	0	0	0	0	0	0	0	0	0	0	0	2
Os01g0759700	461	560	528	538.6	602.9	495.7	531	479	507	562.1	587.7	593.7	617.19	664.62	578.14	1015	505	530.5	545.3	480.5	537.2	449.5	426.9	437	603.2	560	614	526	495	564	6
Os01g0275800	215	179	218	179	206.1	231.8	75.2	78.4	91.9	101.7	134.9	72.44	107.76	168.02	178.19	561.2	233.9	242.4	81.75	146.7	153.5	254.3	225.2	134.3	169	178.2	105.3	290	301	342	13
Os01g0613800	69.3	69.9	119	30.38	40.68	30.28	141	86	72.6	359.5	141.4	114.8	106.67	83.077	121.17	131.4	65.95	79.77	20.44	35.48	18.97	54.71	48.46	54.17	113	121.6	162.3	51.8	89.9	70.8	7
Os01g0813100	89.7	111	103	204.4	241.4	204.2	147	91.1	87.1	90.53	91	194.7	140.42	147.49	94.244	95.53	106.1	149.2	210.9	196.7	179.3	79.11	59.86	85.95	128.9	135.8	149.1	75.9	82.2	102	10
Os01g0332500	52	44.7	52.8	61.57	53.33	72.66	37.6	30.4	39.4	52.59	42.76	47.04	58.78	63.475	53.062	107.5	45.34	44.55	108.7	103.2	102.6	41.41	29.22	40.45	40.14	56.57	32.89	64.8	53.6	57.5	16
Os01g0914200	17.3	13	9.7	85.38	82.26	66.61	43.1	39.8	38.7	5.173	6.578	2.822	4.3541	2.8004	2.3759	0	6.183	4.144	44.59	74.98	56.91	39.19	23.52	15.89	10.56	6.6	2.193	4.63	3.83	10.8	10
Os01g0347100	179	129	137	114.9	112.1	63.15	468	340	343	100.9	99.77	107.3	111.03	119.48	142.55	83.59	159.7	103.6	79.89	87.88	76.74	450.3	433.3	393.6	90.85	79.2	76.75	135	107	117	18
Os01g0777500	3.06	0	1.08	3.284	5.424	2.595	2.35	4.43	0.69	0	5.482	0	7.6196	2.8004	0.792	0	3.092	2.072	3.716	0.806	1.724	5.176	1.425	2.167	3.169	1.886	0	0.93	2.87	1.67	4
Os01g0117450	32.6	65.2	52.8	28.73	24.41	16.44	18	13.9	11.7	54.32	70.17	18.82	53.337	43.872	143.35	47.76	83.47	92.21	11.15	22.58	8.622	35.49	33.49	15.17	84.51	46.2	28.51	96.2	121	93.3	1
Os01g0922100	0	0	0	0	0	0	0	1.27	0	0	0	0	0	0	0	0	0	0	0	0	0	0	0	0	0	0.943	0	0	0	0	5
Os01g0598600	112	105	89.4	91.95	76.84	125.4	174	127	178	231.1	167.7	185.3	137.15	169.89	154.43	143.3	206.1	151.3	193.2	156.4	169	88.73	80.53	112.7	259.9	202.7	190.8	161	150	167	20
Os01g0280500	19.4	2.79	0	3.284	0	8.65	0	0	3.46	0.862	0	2.822	0	0	0	0	2.061	5.18	15.79	4.031	31.04	0	4.988	8.667	0	10.37	8.772	0	1.91	0	16
Os01g0498200	6505	6202	6773	2004	1819	2138	15.7	32.3	35.9	6768	6680	6839	7853.7	6871.1	7537.9	14783	7532	7308	2658	2770	2573	14.79	12.83	18.06	7259	7233	6294	7094	6898	6966	13
Os01g0759200	0	0.93	0	0	0	0	0	0	0	0.862	0	0	0	0	0	0	0	1.036	0	0	0	0.739	0	0	1.056	0	0	0	0	0	7
Os01g0164600	1836	1702	1914	1127	1206	1164	1510	1866	1504	2149	2304	2635	2351.2	2438.2	2719.6	4346	2585	2197	1447	1558	1623	1089	1113	1333	2494	2696	2333	2015	2203	2368	13
Os01g0145600	4.08	4.66	3.23	1.642	0	0.865	0	0	0.69	0.862	0	1.882	1.0885	1.8669	1.5839	0	0	2.072	0	0	0.862	2.958	1.425	0	2.113	0	4.386	4.63	1.91	5	1
Os01g0733100	1.02	0	1.08	4.105	2.712	1.73	5.48	3.16	4.15	0	0	1.882	1.0885	0	0	0	1.031	1.036	3.716	4.031	5.173	1.479	1.425	2.889	0	0.943	0	0	0	0	12
Os01g0761300	685	707	713	841.5	916.6	849.5	1185	1121	1147	716.5	774.1	731	703.18	756.1	772.17	991.1	733.7	698.3	898.3	895.8	985.7	1014	953.5	1049	752.1	831.6	868.4	660	642	676	18
Os01g0200300	4.08	0	0	0	0.904	0	1.57	0.63	2.07	0.862	0	11.29	2.177	4.6673	0.792	0	1.031	1.036	2.787	0.806	0.862	1.479	1.425	0.722	3.169	4.714	6.579	2.78	0.96	3.33	20
Os01g0188200	285	288	248	81.28	78.65	76.99	31.3	38.6	58.7	141.4	203.9	117.6	181.78	204.43	245.51	561.2	306.1	265.2	46.45	45.15	48.24	96.86	104.8	55.61	269.4	222.5	245.6	341	296	393	13
Os01g0231900	999	1030	1045	1004	1041	878	870	978	889	907.9	1053	925.8	1105.9	952.12	1069.2	1672	852.2	865.1	741.3	848.2	820	1003	796	819.7	893.7	923	857.4	852	909	1027	6
Os01g0866500	137	115	147	21.35	6.328	19.03	96.3	127	124	119	93.19	106.3	131.71	121.35	96.62	131.4	165.9	108.8	52.95	41.93	35.35	66.54	83.38	92.45	107.7	79.2	98.68	92.5	119	105	3
Os01g0368500	0	0.93	0	0	0	0.865	0	0.63	0	0	0	0	1.0885	0.9335	0	0	1.031	0	0	0	0.862	0	0	0	0	0	0	0	0	0	4
Os01g0684900	404	355	406	513.9	514.4	533.7	639	719	784	404.4	392.5	482.6	423.43	406.05	385.69	656.8	432.8	460	682.8	624.1	671.7	604.1	611.4	647.9	414.1	406.4	453.9	423	382	392	12
Os01g0638800	0	0	0	0	0	0	0	0	0	0	0	0	0	0	0	0	0	0	0	0	0	0	0.713	0	0	0	0	0	0	0	17
Os01g0563700	0	0	0	1.642	1.808	0	0	0	0	2.587	0	0	2.177	0.9335	0.792	0	0	0	1.858	1.613	0	1.479	1.425	1.444	1.056	1.886	2.193	0	0	1.67	7
Os01g0636500	2.04	2.79	1.08	7.389	3.616	6.055	1.57	1.9	1.38	0.862	3.289	1.882	2.177	1.8669	1.5839	0	2.061	3.108	4.645	4.838	2.587	0	0	0	0	0.943	2.193	2.78	2.87	0	10
Os01g0552050	31.6	30.7	21.6	31.2	29.83	31.14	14.1	17.7	13.1	8.622	21.93	6.586	11.974	5.6007	22.175	11.94	21.64	24.86	7.432	11.29	12.93	41.41	35.63	20.22	11.62	14.14	6.579	30.5	39.2	37.5	8
Os01g0757600	6.12	11.2	11.9	6.568	4.52	21.63	3.13	19	11.7	2.587	8.771	7.527	3.2656	5.6007	3.1679	11.94	10.31	9.324	12.08	20.96	20.69	11.83	12.11	17.33	12.68	5.657	10.96	9.25	9.56	10	16
Os01g0960600	5.1	1.86	9.7	3.284	5.424	5.19	1.57	3.8	0.69	1.724	6.578	3.763	6.5311	0.9335	13.463	0	1.031	4.144	0	0.806	0.862	2.218	1.425	3.611	2.113	3.771	2.193	3.7	4.78	6.66	4
Os01g0185300	93.8	95	98.1	24.63	23.5	25.95	32.9	32.9	33.2	19.83	15.35	24.46	16.328	24.27	27.719	71.65	60.8	68.38	29.73	33.06	22.42	39.19	49.88	57.78	16.9	29.23	30.7	25.9	31.6	27.5	14
Os01g0356500	169	136	144	113.3	102.1	89.1	163	160	246	133.6	137	130.8	166.54	148.42	156.81	286.6	162.8	152.3	120.8	97.56	100	168.6	160.3	163.9	143.7	157.5	144.7	154	199	162	6
Os01g0382000	2315	797	829	3011	3537	1045	1830	1431	3020	1582	891.4	602.1	599.77	1810.9	3605.8	1039	287.5	238.3	1114	1979	191.4	645.5	2168	230.4	146.8	312.1	464.9	52.7	678	284	2
Os01g0719200	12.2	3.73	14	12.31	8.136	6.055	6.26	5.06	6.22	7.76	15.35	9.408	1.0885	11.201	11.088	23.88	10.31	12.43	19.51	17.74	25.87	2.958	11.4	5.056	6.338	11.31	21.93	7.4	7.65	5	16
Os01g0738300	0	0	0	1.642	0	1.73	0	0	0	1.724	0	0	0	0	0	0	0	1.036	0	0	1.724	0.739	2.138	2.167	0	0	4.386	0	4.78	0	11
Os01g0621400	0	0.93	0	0	0	2.595	0	0	0	0	0	0	0	0	1.5839	0	0	0	0	0	0	0	0	0	0	0	0	0	0	0.83	2
Os01g0693400	222	244	294	199.5	178.1	115.9	303	267	353	250.9	247.8	169.3	237.3	159.62	225.71	322.4	135	130.5	222	220.9	169.9	272.1	311.4	260	196.5	141.4	87.72	99	151	157	18
Os01g0731201	0	0	0	0.821	0	1.73	0.78	0	1.38	0.862	7.675	3.763	4.3541	0.9335	2.3759	0	2.061	0	4.645	2.419	2.587	24.4	7.839	10.83	0	0	2.193	3.7	3.83	0.83	9
Os01g0319200	0	0	0	0	0	0	0	0	0	0	1.096	0	0	0	0	0	0	0	0	0	0	0	0	0	0	0	0	0	0	0	7
Os01g0617700	114	129	108	217.6	156.4	212.8	223	220	198	111.2	134.9	140.2	130.62	104.55	126.71	370.2	126.8	141.9	159.8	133	160.4	165.6	191	183.5	125.7	137.7	153.5	110	125	129	6
Os01g0304050	0	0.93	0	3.284	1.808	2.595	3.13	0.63	0.69	0.862	1.096	0.941	2.177	1.8669	2.3759	23.88	11.34	14.5	10.22	14.51	7.76	10.35	10.69	2.167	4.225	5.657	0	7.4	5.74	7.5	6
Os01g0255000	1779	1545	1825	1962	1993	2249	1220	1177	1312	1906	1993	1934	2620.1	2651.9	2132	3212	2063	1933	2844	2364	2981	834	807.4	940.4	1906	1700	1746	1652	1703	1709	16
Os01g0286200	8.16	4.66	9.7	15.6	15.37	13.84	13.3	6.33	10.4	12.07	9.868	8.467	18.505	9.3345	10.296	23.88	4.122	8.288	4.645	4.838	3.449	11.09	6.414	12.28	11.62	10.37	10.96	16.7	11.5	12.5	6
Os01g0297200	15.3	30.7	38.8	35.3	28.02	15.57	25.8	16.4	30.4	122.4	36.18	44.22	40.275	33.604	28.511	11.94	12.37	10.36	12.08	51.6	9.484	19.22	22.8	7.222	29.58	20.74	19.74	41.6	47.8	16.7	7
Os01g0823600	####	9570	####	1781	1944	1723	265	490	169	14447	14983	15766	20764	19110	18785	31058	19615	13053	4058	3224	2218	174.5	364.9	229	16177	17486	16401	####	####	####	13
Os01g0735900	15.3	9.31	6.47	5.747	5.424	9.516	12.5	10.1	16.6	6.897	8.771	20.7	9.7967	8.4011	3.9598	0	4.122	12.43	12.08	4.838	7.76	14.79	19.95	20.95	16.9	7.543	8.772	9.25	18.2	13.3	9
Os01g0177100	1.02	0.93	2.16	2.463	1.808	5.19	0.78	0	0.69	0.862	0	1.882	2.177	0.9335	0.792	0	2.061	5.18	9.29	0.806	0	0	2.138	2.889	5.282	2.829	0	1.85	0.96	0.83	16
Os01g0306900	1120	1047	953	495.9	516.2	462.8	413	464	453	1541	1658	1939	1261.6	1262	1303.6	1540	1293	1201	649.3	634.5	750.1	695	539.5	700.6	1341	1813	1735	1281	1168	1278	3
Os01g0817000	1113	721	712	67.32	39.77	59.69	235	214	223	602.7	606.3	541	542.08	520.87	558.34	919.5	851.2	851.6	90.11	89.5	106.1	239.6	225.9	318.5	735.2	735.4	750	571	585	562	3
Os01g0543000	376	381	357	230.7	211.5	232.7	137	132	153	303.5	246.7	433.7	313.49	345.38	258.18	573.2	311.2	346	233.2	208.8	223.3	279.5	241.6	221.7	394	429.9	510.9	403	350	369	13
Os01g0341200	13.3	10.2	15.1	8.21	10.85	7.785	16.4	10.8	8.98	16.38	2.193	15.99	11.974	6.5342	5.5438	23.88	6.183	4.144	7.432	6.45	14.66	12.57	9.977	12.28	4.225	10.37	26.32	8.33	6.69	10	11
Os01g0845000	0	0.93	4.31	1.642	0	3.46	0	0	1.38	1.724	0	0	0	2.8004	0.792	0	0	0	0	0	0.862	0	3.563	1.444	0	0	0	0	0	0	17
Os01g0610600	962	897	969	866.1	863.3	937.7	634	720	656	876.8	928.6	888.1	957.9	911.98	915.51	1684	985.2	925.2	865.8	936.9	805.3	872.5	723.3	722.2	961.3	1030	1013	1020	1041	1088	13
Os01g0200950	158	197	157	179	173.6	156.6	128	137	126	124.2	134.9	108.2	182.87	144.68	160.77	226.9	125.7	162.7	106.8	112.9	91.4	134.6	110.5	128.6	140.5	149	92.1	142	139	182	8
Os01g0800266	2.04	0	0	0.821	0	0	2.35	0	2.76	1.724	0	0	0	0	0	0	1.031	0	3.716	0	0	0.739	0.713	0	0	0	0	0	0.96	2.5	15
Os01g0106400	24.5	27	25.9	149.4	198.9	49.31	19.6	15.8	11.7	105.2	31.8	21.64	42.452	61.608	319.95	23.88	6.183	8.288	155.1	74.88	15.52	5.915	6.414	5.056	25.35	41.49	105.3	13	62.2	90	4
Os01g0580300	91.8	97.8	81.9	135.5	139.2	134.1	211	194	209	135.4	142.5	110.1	119.74	107.35	114.04	155.2	90.69	94.28	131.9	136.3	135.4	170.8	148.2	182	136.3	115	100.9	114	91.8	95.8	18
Os01g0136800	6.12	11.2	5.39	9.852	9.04	7.785	4.7	8.86	4.15	3.449	9.868	6.586	3.2656	3.7338	5.5438	11.94	12.37	6.216	4.645	0.806	6.036	10.35	8.552	12.28	5.282	0.943	13.16	8.33	6.69	5.83	9
Os01g0921400	231	194	207	193.8	176.3	211.1	260	321	281	231.9	197.4	195.7	216.62	219.36	265.31	501.5	265.9	245.5	231.3	228.2	256.1	260.3	257.3	281	212.3	227.2	236.8	216	235	230	6
Os01g0732300	12.2	5.59	1.08	1.642	0	0.865	0	5.06	0	0.862	2.193	4.704	0	1.8669	0.792	0	0	2.072	2.787	0.806	0.862	0.739	0.713	2.167	3.169	7.543	0	2.78	1.91	2.5	19
Os01g0953801	0	0	0	0	0	0	0	0	0	1.724	0	0	0	0.9335	0	0	0	0	0	0	0	0	0	0	0	0	0	0	0	0	7
Os01g0544700	761	741	831	717.5	682.5	728.4	595	514	492	662.2	599.7	788.4	653.11	687.02	634.36	931.4	718.3	806	714.4	674.9	745.8	638.8	520.2	555.4	605.3	758	835.5	638	758	625	13
Os01g0740700	5.1	10.2	4.31	41.05	23.5	46.71	19.6	12	10.4	6.035	1.096	2.822	16.328	4.6673	12.671	0	5.153	6.216	19.51	21.77	17.24	48.06	49.17	26	2.113	8.486	4.386	15.7	14.3	10.8	17
Os01g0183633	233	231	258	234	278.4	246.5	263	280	241	265.6	258.7	263.4	289.55	295.9	293.03	406	288.5	235.2	297.3	259.6	235.4	217.4	226.6	226.8	279.9	281.9	269.7	278	261	278	13
Os01g0894600	247	260	276	260.2	264.9	308.8	178	220	191	294.9	268.6	313.3	298.25	428.45	312.83	668.7	399.8	351.2	347.4	308.8	458.7	210.7	220.9	217.4	337	495.9	443	481	419	451	13
Os01g0660100	0	0.93	0	0	0	0	0	0	0	0.862	0	0	0	0.9335	0	0	0	0	0	0	0	0	0	0	0	0.943	0	0.93	0.96	0	1
Os01g0864300	0	0	0	0	0	0	0.78	0	0.69	0	0	2.822	0	0	0	0	0	0	0	0	0	2.218	3.563	0	0	0	0	0	0	0	17
Os01g0277500	52	67.1	46.3	139.6	145.5	116.8	326	295	299	58.63	52.63	83.73	45.718	51.34	44.35	59.7	59.77	65.27	116.1	123.4	136.2	235.1	195.3	261.5	51.76	65.06	43.86	47.2	47.8	44.2	18
Os01g0552000	101	118	140	122.3	131.1	106.4	33.7	44.9	29.7	37.07	58.11	18.82	69.665	50.406	83.156	119.4	63.89	84.96	22.29	37.09	35.35	122	99.06	75.84	60.21	45.26	35.09	105	92.8	115	8
Os01g0968000	401	391	400	402.3	405	356.4	276	293	257	391.4	400.2	485.5	552.97	463.93	517.15	382.1	402.9	422.7	376.2	368.5	354.4	301.7	296.5	309.1	456.3	412	432	389	424	457	3
Os01g0351500	1.02	0	0	0	0	0	0	0	0	0	0	0	0	0	0	0	0	0	0	0	0	0	0	0	0	0	0	0	0	0	19
Os01g0231500	1586	1391	1553	981.9	1061	957.6	356	494	373	1090	1180	987.9	1459.7	1568.2	1523.7	2364	1761	1609	1028	1199	1122	516.8	566.5	474.5	1587	1466	1145	1750	1735	1830	13
Os01g0622000	0	0	0	0	0	0	0	0	0	0	0	0	0	0	0	0	0	0	0	0	0	0	0	0	2.113	0	0	0	0	0	1
Os01g0276900	616	304	586	120.7	54.24	166.1	3.13	2.53	6.22	518.2	358.5	999.2	477.86	558.2	432.41	919.5	676	448.6	277.8	125.8	187.1	24.4	55.59	35.39	324.3	693	1042	486	558	345	3
Os01g0778900	63.2	55	66.8	21.35	7.232	13.84	20.4	22.1	28.3	55.18	66.88	66.8	88.17	71.876	78.405	179.1	71.11	76.67	13.01	10.48	14.66	8.133	4.276	23.83	73.95	101.8	70.17	62.9	65	79.1	13
Os01g0838800	337	354	379	421.2	441.1	406.6	575	559	536	405.2	394.7	327.4	416.9	353.78	414.99	549.3	358.6	370.9	501.6	420.9	489.7	465.8	471.1	420.3	332.8	347.9	361.8	331	438	412	18
Os01g0702000	408	535	634	22628	26230	23185	####	####	####	331.9	344.3	112	594.33	495.66	464.88	1433	328.7	623.7	21097	20987	23727	13514	13716	14776	262	86.74	52.63	484	517	688	12
Os01g0194600	435	298	395	128.9	97.63	124.6	92.4	46.8	29	625.9	387	943.6	574.74	669.28	495.77	501.5	418.4	419.6	317.7	128.2	221.6	20.7	15.68	31.78	332.8	334.7	440.8	396	216	352	3
Os01g0558900	530	518	533	541.8	553.2	545	719	674	671	586.3	603	736.7	707.54	620.75	580.51	991.1	624.5	551.2	715.3	707.1	665.6	429.6	539.5	525.8	637	681.7	677.6	610	536	545	6
Os01g0531400	24.5	12.1	18.3	9.852	11.75	14.71	0.78	17.1	8.98	9.484	4.386	13.17	5.4426	13.068	3.1679	35.82	7.214	15.54	2.787	5.644	8.622	11.83	9.977	18.78	10.56	13.2	6.579	10.2	6.69	10	6
Os01g0104800	480	450	482	885.8	894.9	788.1	342	362	352	420.7	349.7	476.1	523.58	509.66	395.19	632.9	428.7	414.4	693.9	708.7	657.9	377.8	435.4	366.9	365.5	401.7	414.5	454	428	400	10
Os01g0550200	72.4	70.8	53.9	59.93	73.22	95.16	50.1	72.1	66.3	100	75.6	106.3	70.754	71.876	78.405	119.4	66.98	67.34	53.88	74.98	76.74	64.33	68.41	72.95	78.17	61.28	81.14	65.7	72.7	90	6
Os01g0197900	80.5	113	92.7	46.8	55.14	52.77	64.2	68.9	38	67.25	64.69	44.22	84.905	69.075	101.37	214.9	128.8	148.2	61.31	70.15	43.11	122	116.9	75.84	133.1	62.23	98.68	150	90.9	154	13
Os01g0527400	663	659	638	719.2	697	683.4	804	805	826	778.6	848.6	848.6	764.14	778.5	807.01	1158	624.5	634.1	670.7	649.1	642.4	700.2	645.6	656.5	770.1	740.1	725.9	737	741	706	6
Os01g0732000	239	265	264	302.1	285.7	323.5	310	287	298	353.5	349.7	390.4	289.55	295.9	258.18	298.5	257.6	220.7	353	330.6	380.2	227	224.5	288.2	289.4	265.9	320.2	227	260	242	15
Os01g0972300	0	0	0	0.821	0	0.865	1.57	3.8	0	0	3.289	3.763	0	0	0	0	0	0	0	0	0	2.218	0	1.444	0	0	0	0	0.96	0.83	5
Os01g0125700	59.1	73.6	76.5	74.71	75.03	90.83	95.5	77.2	54.6	86.22	74.55	90.32	74.019	70.942	63.357	59.7	57.71	76.67	81.75	97.56	86.22	62.85	69.84	65	70.78	65.06	76.75	55.5	60.2	64.2	15
Os01g0104400	4224	3703	3553	3006	2837	3347	1029	996	911	2805	2786	3633	2042.1	3531.2	2578.6	5481	4423	4013	3278	3469	3931	1155	1239	1474	3565	4430	4438	3676	3367	3271	13
Os01g0375200	109	143	120	210.2	179	187.7	145	174	180	121.6	107.4	81.85	81.639	113.88	91.868	83.59	83.47	80.81	150.5	143.5	100.9	133.8	226.6	151.7	117.3	94.28	133.8	87.9	96.6	102	12
Os01g0301100	0	0	0	0	0	0	0.78	0	0	0	0	0	0	0	0	0	1.031	0	0	0	0	0	0.713	0.722	0	0.943	0	0	0	0	9
Os01g0151100	708	609	730	1209	1258	1083	703	745	708	817.4	702.8	697.1	969.87	858.78	921.06	1875	892.4	693.1	1092	1358	1282	729	860.2	691.9	863	827.8	785.1	921	911	891	6
Os01g0945200	2.04	0	0	9.031	4.52	4.325	0.78	3.16	2.07	0	1.096	0	3.2656	0	0	0	0	1.036	11.15	7.257	0.862	0	0	0	0	0	0	0	0.96	1.67	10
Os01g0371500	1.02	1.86	3.23	1.642	4.52	1.73	0.78	0	0	1.724	3.289	2.822	0	0	0.792	0	0	4.144	0	0.806	0	0.739	0.713	0	0	0	0	0	0	0	2
Os01g0525701	0	0	0	0	0	0	0	0	1.38	0	0	0	0	0	0	0	0	0	0	0	0	0	0	0	0	0	0	0	0	0	18
Os01g0118300	7.14	0.93	7.54	4.926	6.328	1.73	11.7	20.9	14.5	5.173	4.386	0	1.0885	3.7338	7.9197	0	0	4.144	7.432	3.225	0	9.612	14.97	4.333	4.225	1.886	4.386	7.4	4.78	4.17	18
Os01g0833000	191	218	219	518	637.3	540.7	261	323	314	197.4	274.1	232.4	371.19	218.43	190.07	429.9	182.4	214.5	551.8	641	724.3	162.7	163.9	223.2	214.4	174.4	140.3	124	158	164	10
Os01g0713200	321	448	397	198.7	168.1	174.7	821	383	1092	707	505.4	607.8	197.02	214.69	250.26	167.2	121.6	120.2	143.1	249.9	170.7	773.4	396.2	1029	198.6	654.3	587.7	53.7	138	83.3	5
Os01g0814150	0	0	0	0	0.904	0	0	0	0.69	1.724	0	0	0	0	0	0	0	0	0	0	0	0	0	1.444	0	0	0	0	0	0	7
Os01g0782500	639	740	612	1295	1337	1268	1170	1221	1127	745.8	740.1	779.9	830.54	759.83	736.53	812	645.1	671.4	1272	1241	1394	1051	1036	1097	715.2	690.2	732.4	616	663	711	12
Os01g0351200	27.5	9.31	24.8	110.8	94.92	80.45	42.3	70.2	42.8	15.52	12.06	11.29	13.062	30.804	26.135	71.65	18.55	29.01	67.81	74.18	50.87	49.54	54.87	37.56	11.62	9.428	4.386	18.5	22	22.5	10
Os01g0742400	421	270	407	133.8	154.6	132.4	189	340	198	341.4	127.2	147.7	124.09	169.89	238.38	191.1	182.4	169.9	117	141.1	93.12	258	282.9	240.5	129.9	69.77	122.8	78.6	120	82.5	14
Os01g0505700	66.3	60.5	88.4	121.5	89.49	117.6	107	82.9	109	109.5	84.42	147.7	90.347	114.81	78.405	131.4	86.56	109.8	207.2	175.8	212.1	85.03	91.22	88.11	76.06	116	98.68	73.1	84.2	70	16
Os01g0758200	1.02	0.93	0	4.105	1.808	0.865	11	13.9	22.8	0	1.096	0.941	0	0	2.3759	0	0	0	4.645	4.031	0.862	8.873	9.977	7.222	1.056	0	0	0	0	0	18
Os01g0124200	0	0	3.23	30.38	0.904	37.2	2.35	0.63	38.7	0	12.06	0	0	0	0	0	0	0	0.929	0	0	0	6.414	0.722	0	0	0	0	0	0	2
Os01g0645400	0	2.79	5.39	1.642	4.52	2.595	7.05	3.16	4.84	3.449	9.868	15.05	8.7082	1.8669	8.7116	0	4.122	1.036	3.716	0.806	0.862	5.176	6.414	8.667	13.73	8.486	2.193	16.7	3.83	9.16	1
Os01g0613900	604	518	567	581.3	606.6	583	615	624	577	567.3	589.9	675.5	699.92	739.29	651.79	1266	696.6	639.2	656.8	636.2	719.1	472.5	445.4	517.8	551.4	627	585.5	603	626	579	6
Os01g0193900	325	335	359	285.7	395.9	371.1	307	278	274	262.1	299.3	238	324.38	327.64	349.26	668.7	300.9	352.3	256.4	312	244	367.5	406.2	321.4	309.5	295.1	225.9	381	287	351	6
Os01g0873000	20.4	9.31	9.7	2.463	0	7.785	1.57	3.8	2.07	18.97	32.89	45.16	28.302	22.403	23.759	0	31.95	20.72	3.716	4.031	4.311	1.479	0.713	2.167	35.92	54.68	26.32	26.8	17.2	40	3
Os01g0163000	75.4	74.5	98.1	91.95	59.66	33.74	21.9	82.2	26.3	67.25	63.59	31.05	84.905	47.606	83.948	35.82	25.76	47.66	64.1	27.41	4.311	14.79	26.37	15.89	44.37	18.86	15.35	55.5	52.6	51.7	4
Os01g0667000	0	2.79	1.08	0	0	0.865	0	0	1.38	0	0	0	0	0.9335	0	0	0	1.036	0	0	0	0.739	0	1.444	0	0	0	0	0	0	14
Os01g0261100	1405	1463	1474	1056	1013	1042	1076	1078	1109	1372	1463	1343	1604.5	1495.4	1579.2	2663	1524	1564	1067	1070	1035	1221	1174	1177	1571	1675	1535	1606	1565	1660	13
Os01g0753100	1291	1045	1136	720.8	796.4	804.5	724	787	643	1178	1226	1411	1455.4	1445	1382	1612	1418	1207	945.7	940.1	826.9	583.4	667.7	760.5	1364	1323	1145	1358	1113	1268	3
Os01g0296200	0	0	2.16	0	0	0	0	0	0	0	0	0	0	0	0	0	1.031	0	0	0	0	0	0	0	1.056	1.886	4.386	0	2.87	0.83	11
Os01g0532800	40.8	54	51.7	76.35	84.07	96.89	54.8	60.7	58	62.94	65.78	90.32	60.957	66.275	64.149	155.2	75.23	83.92	83.61	90.3	89.67	84.29	88.37	81.61	82.4	83.91	89.91	67.5	44.9	72.5	6
Os01g0221000	4.08	7.45	6.47	42.69	55.14	44.12	77.5	57.6	57.4	20.69	14.25	29.17	9.7967	11.201	10.296	0	2.061	3.108	23.22	20.96	16.38	133.1	153.9	106.9	13.73	13.2	19.74	7.4	10.5	8.33	17

Os01g0896400	2.04	2.79	0	0	0	0	0	0	0	0	0	2.822	0	0	0	0	3.092	1.036	0	0	0	0.739	0	0	1.056	0.943	2.193	0	0	0	14
Os01g0812800	159	74.5	106	191.3	182.6	219.7	160	129	171	133.6	80.04	194.7	139.33	144.68	79.197	131.4	72.14	116	289.8	244.3	280.2	137.5	138.3	128.6	80.28	141.4	122.8	71.2	114	51.7	10
Os01g0799100	344	337	391	422	407.7	396.2	425	514	425	340.6	327.8	231.4	299.34	254.83	318.37	513.5	325.6	412.3	338.1	350.7	319	539.8	549.4	473.1	333.8	256.5	313.6	340	383	353	17
Os01g0223500	11.2	14	16.2	13.14	19.89	16.44	30.5	30.4	29	6.897	14.25	9.408	18.505	13.068	7.9197	0	15.46	10.36	4.645	15.32	13.8	20.7	16.39	23.11	9.507	5.657	8.772	15.7	19.1	5.83	18
Os01g0284500	0	0	0	0.821	0	0.865	0	0	0	0	0	0	0	0	0	0	0	0	0	0.806	0	0.739	0.713	1.444	0	0	0	0	0	0	9
Os01g0670600	0	0	0	1.642	0	0	1.57	1.27	0	0	0	0.941	0	0	0	0	0	1.036	0	0.806	0	2.218	0.713	2.889	0	0	0	0	0	0	9
Os01g0285200	0	0.93	0	0.821	0	1.73	0.78	0	2.76	0	0	0.941	2.177	2.8004	0	0	1.031	0	2.787	1.613	1.724	1.479	0.713	2.167	2.113	0.943	2.193	0	0	0.83	15
Os01g0284300	0	0	0	0	0	0	0	0	0	0	0	0	1.0885	0	0	0	0	0	0	0	0	0	0	0	0	0	2.193	0	0	0	11
Os01g0721400	59.1	47.5	64.7	24.63	27.12	32.01	44.6	43.6	25.6	74.15	54.82	118.5	51.16	57.874	51.478	59.7	43.28	36.26	23.22	10.48	26.73	30.31	25.65	42.61	46.48	38.66	52.63	58.3	44	43.3	20
Os01g0518800	101	61.5	62.5	64.04	50.62	68.34	29	32.3	27.6	66.39	60.3	95.02	72.931	70.942	64.941	59.7	73.17	66.31	96.61	78.21	62.08	48.06	50.6	44.78	65.49	80.14	81.14	97.1	100	70	3
Os01g0703300	0	0	0	1.642	0.904	0	0.78	0	0	5.173	4.386	0	1.0885	0	0	0	0	0	0	0	0	0	0	0	0	0	0	0	5.74	0	7
Os01g0318700	0	0	0	1.642	0	0	0	0	0	0	0	0	0	1.8669	0	0	0	0	0	0	0	0	0	0	0	0	0	0	0	0	4
Os01g0953100	43.8	44.7	49.6	67.32	37.06	59.69	218	185	193	111.2	60.3	89.38	51.16	35.471	47.518	47.76	47.4	38.33	61.31	55.63	56.04	144.9	148.9	135.1	69.72	57.51	63.59	34.2	44	35.8	18
Os01g0133500	805	649	862	382.6	373.3	391.9	297	267	282	813.9	858.5	798.8	1113.6	1188.3	1243.4	1218	647.2	710.7	446.8	370.9	432	174.5	217.4	218.1	829.2	695.8	793.8	1013	1003	961	3
Os01g0221900	28.5	55	40.9	157.6	167.2	127.2	220	264	225	56.9	62.49	61.15	39.187	34.538	48.31	23.88	52.56	56.98	112.4	160.5	117.3	248.4	188.8	221	43.31	38.66	17.54	45.3	34.4	61.7	12
Os01g0793701	6.12	0	0	5.747	2.712	0	0	0.63	0	1.724	1.096	0	3.2656	0.9335	0.792	0	1.031	0	0.929	1.613	0.862	1.479	2.851	0	2.113	0.943	0	4.63	0	1.67	19
Os01g0561500	26.5	13	33.4	22.17	12.66	10.38	16.4	19	24.2	23.28	23.02	15.99	14.151	10.268	10.296	23.88	25.76	12.43	37.16	18.54	17.24	19.96	22.09	17.33	29.58	31.11	19.74	14.8	15.3	17.5	19
Os01g0886600	298	350	327	492.6	436.6	529.4	474	493	430	244	336.6	348.1	261.24	243.63	229.67	370.2	224.7	227.9	485.8	590.2	513	360.1	316.4	469.5	229.2	292.3	247.8	217	250	227	12
Os01g0885900	17.3	14	11.9	0	0	0	0	0	0	2.587	1.096	0	0	2.8004	4.7518	0	4.122	4.144	1.858	2.419	1.724	0	0.713	0.722	1.056	0.943	0	0.93	1.91	0.83	14
Os01g0149200	1232	1014	1024	463.9	468.3	587.4	74.4	198	127	502.7	419.9	462.9	536.64	812.1	685.05	883.6	1302	1191	320.5	262	350.1	306.1	405.5	337.3	872.6	858.9	1037	1178	768	746	3
Os01g0612800	164	144	159	137.1	120.2	134.1	360	280	310	244	227	295.4	214.44	225.9	187.7	262.7	188.6	184.4	231.3	212.1	233.7	196.7	207.4	216.7	238.7	259.3	214.9	182	145	148	5
Os01g0575200	407	329	448	82.1	106.7	66.61	128	151	132	170.7	125	98.79	95.79	122.28	108.5	167.2	134	112.9	93.82	75.79	56.04	251.4	252.3	268	64.44	87.68	100.9	74	109	74.1	14
Os01g0636700	1039	1022	1056	963	980.8	998.3	868	940	794	963.1	1067	1044	1262.7	1088.4	1147.6	1911	1218	1149	918.7	1036	953.6	1061	1033	865.2	1280	1156	1101	1326	1252	1370	13
Os01g0642000	227	145	207	10.67	6.328	5.19	0.78	2.53	1.38	166.4	167.7	133.6	292.81	295.9	297.78	561.2	253.5	275.6	5.574	7.257	8.622	5.176	17.1	8.667	169	175.4	111.8	259	212	268	13
Os01g0603700	37.7	27.9	42	46.8	36.16	38.93	35.2	26.6	20.7	43.11	47.14	34.81	40.275	33.604	22.967	59.7	41.22	33.15	42.73	54.83	51.73	36.23	19.95	29.61	36.97	38.66	43.86	31.5	30.6	24.2	16
Os01g0862000	376	324	409	336.6	339	342.6	501	537	554	474.2	470.4	450.7	397.31	415.39	491.02	823.9	515.3	430	387.4	431.4	439.7	590.8	518.1	478.1	508.1	559.1	451.7	498	502	481	6
Os01g0814200	19.4	22.4	12.9	34.48	46.1	25.09	25.1	32.3	21.4	2.587	6.578	6.586	4.3541	4.6673	8.7116	11.94	5.153	5.18	14.86	33.86	23.28	26.62	44.9	28.89	11.62	3.771	2.193	4.63	7.65	7.5	12
Os01g0769700	371	183	298	85.38	76.84	102.9	24.3	24	28.3	81.91	70.17	58.33	62.046	98.012	51.478	238.8	217.4	231	71.53	55.63	77.6	24.4	17.82	26	44.37	56.57	54.82	50.9	72.7	50	14
Os01g0518500	11.2	7.45	14	18.06	24.41	8.65	23.5	31.6	29.7	32.76	18.64	11.29	22.859	21.469	9.5036	11.94	16.49	12.43	26.94	33.86	17.24	5.915	17.1	24.56	29.58	13.2	13.16	13.9	17.2	13.3	15
Os01g0513200	416	488	399	230.7	245.9	200.7	236	276	225	285.4	411.1	294.5	336.35	349.11	396.77	943.3	431.8	328.4	185.8	249.1	213.8	224.8	227.3	244.1	297.9	336.6	307	419	399	410	13
Os01g0501000	370	454	446	435.9	457.4	444.6	378	397	367	365.6	378.3	379.2	383.16	381.78	345.3	728.4	388.5	423.7	452.4	429.7	379.4	385.2	391.2	359	375	349.8	394.7	394	419	454	6
Os01g0150000	3110	3230	3135	1727	1861	1716	1397	1366	1366	3319	3379	3392	3204.6	3313.8	3121.9	4358	2780	2637	1885	1825	1875	886.5	912.9	1099	3741	3168	2903	3256	2568	3191	3
Os01g0788800	6.12	1.86	5.39	2.463	4.52	2.595	1.57	5.06	2.76	11.21	5.482	10.35	1.0885	4.6673	1.5839	0	4.122	5.18	7.432	4.838	2.587	4.436	5.701	5.778	6.338	5.657	6.579	6.48	0.96	3.33	20
Os01g0675800	308	241	194	30.38	18.98	17.3	25.8	29.1	13.8	62.94	44.95	56.45	58.78	36.405	60.189	394.1	209.2	165.8	16.72	24.19	61.22	36.97	40.62	97.5	52.82	65.06	61.4	72.2	59.3	82.5	13
Os01g0904300	255	318	294	358.8	395.9	292.4	296	409	394	358.7	452.8	229.6	429.97	380.85	438.75	597	310.2	301.5	330.7	383.8	390.6	232.9	225.2	190.7	328.5	262.1	208.3	339	363	432	6
Os01g0372100	10.2	5.59	6.47	2.463	0	2.595	5.48	1.9	3.46	1.724	6.578	17.88	4.3541	5.6007	1.5839	23.88	12.37	8.288	1.858	5.644	1.724	4.436	6.414	5.056	9.507	15.09	4.386	11.1	4.78	11.7	13
Os01g0251200	462	473	483	388.3	368.8	359.9	260	288	292	563.9	521.9	632.2	524.67	512.46	549.62	680.6	465.8	457.9	386.4	344.3	369	301.7	285.1	317.1	556.7	509.1	462.7	468	450	504	3
Os01g0729900	257	254	227	407.2	326.3	493.1	432	436	509	278.5	262	273.8	328.73	312.71	333.42	250.8	213.3	250.7	487.7	513.6	672.5	360.1	400.5	353.9	286.3	218.7	346.5	191	186	173	12
Os01g0364800	185	211	185	142	129.3	153.1	47	85.4	144	138.8	151.3	86.56	87.082	158.69	216.21	262.7	144.3	145	73.39	112.9	98.29	512.4	510.2	343.1	147.9	121.6	140.3	229	147	155	17
Os01g0238500	3737	3327	3499	776.6	266.7	1072	429	355	1229	6772	6874	11081	3065.3	3088.8	2653.1	4478	3735	4274	1309	949.8	2000	659.5	600.8	887.6	5114	7184	6555	1749	2681	1968	20
Os01g0136050	3.06	0	0	1.642	3.616	1.73	0	0	0	2.587	0	0	0	0	0	0	0	0	0	0.806	4.311	0	0	0	1.056	0	6.579	0	1.91	0	11
Os01g0787000	37.7	32.6	28	7.389	5.424	9.516	3.91	6.96	12.4	20.69	9.868	11.29	3.2656	8.4011	11.088	23.88	21.64	16.58	7.432	4.031	2.587	16.27	15.68	19.5	3.169	7.543	17.54	9.25	4.78	6.66	14
Os01g0776500	2.04	5.59	5.39	13.96	8.136	13.84	1.57	14.5	8.98	0	2.193	3.763	0	1.8669	2.3759	0	2.061	0	17.65	7.257	6.898	7.394	4.988	2.889	4.225	1.886	0	0	3.83	0	12
Os01g0607900	48.9	42.8	45.3	22.99	19.89	19.03	11	11.4	13.8	112.9	112.9	146.8	79.462	69.075	78.405	23.88	62.86	39.37	28.8	11.29	35.35	4.436	10.69	9.389	106.7	118.8	140.3	74.9	53.6	80.8	3
Os01g0876800	83.6	68	113	105.1	143.7	119.4	6.26	15.2	11.7	117.3	52.63	79.03	82.728	89.611	58.605	59.7	43.28	55.95	111.5	66.12	82.77	8.133	19.24	7.945	53.87	19.8	21.93	62	70.8	72.5	16
Os01g0238600	475	465	434	550.9	550.5	566.6	790	659	674	554.4	546	707.5	440.85	531.13	469.64	549.3	446.2	413.4	748.7	627.3	699.3	413.3	469.6	607.4	517.6	489.3	594.3	349	411	346	15
Os01g0833150	467	502	553	83.74	97.63	116.8	217	279	244	494.9	532.8	461.9	719.51	509.66	698.51	740.3	482.3	495.2	99.4	88.69	80.19	140.5	184.6	166.8	488	423.3	276.3	458	455	626	3
Os01g0685400	10.2	5.59	5.39	0.821	3.616	0	7.05	6.33	8.29	0	0	0.941	4.3541	5.6007	1.5839	0	7.214	6.216	1.858	0	0.862	0.739	4.276	4.333	0	1.886	13.16	2.78	0.96	0	11
Os01g0838600	21.4	16.8	12.9	6.568	4.52	16.44	10.2	4.43	13.1	17.24	21.93	17.88	6.5311	11.201	23.759	23.88	14.43	17.61	3.716	13.71	10.35	18.48	14.97	11.56	19.01	13.2	13.16	21.3	24.9	15.8	13
Os01g0815700	1453	1314	1391	2217	2211	2114	700	713	671	1613	1527	2111	2085.6	1926.6	1806.5	2388	1678	1579	2624	2217	2669	539.8	560.1	534.5	1542	1769	1596	1424	1329	1291	16
Os01g0509800	0	0	0	0.821	3.616	0	3.91	0	5.53	0.862	0	0	0	0	0	0	0	0	1.858	0	0	0	0	0	1.056	0	0	0	0	0	18
Os01g0680500	33.6	34.5	38.8	24.63	26.22	23.36	69.7	65.8	64.3	38.8	30.7	53.63	30.479	22.403	30.887	35.82	29.89	46.62	36.23	44.35	25	51.02	57.72	54.89	36.97	46.2	13.16	57.4	40.2	60	18
Os01g0296100	974	1085	946	1128	1003	1066	1969	1713	2117	1093	1138	1018	1166.9	1240.6	1203.8	1684	1147	1145	1226	1197	1310	1669	1470	1776	1199	1166	1235	1214	1093	1176	18
Os01g0626500	587	472	572	576.3	566.8	634.9	297	262	322	427.6	462.7	556	488.75	563.8	484.68	740.3	547.2	630.9	571.3	677.3	607	673.6	565.1	441.3	511.3	645.8	649.1	565	660	545	16
Os01g0565900	505	413	444	512.3	426.7	667.8	539	422	500	598.4	407.9	487.3	448.47	497.53	407.07	656.8	372	346	709.7	498.3	492.3	349	379.1	421.8	429.9	373.4	664.5	328	346	240	16
Os01g0111000	0	0	0	1.642	0	1.73	2.35	2.53	2.76	0	0	3.763	0	0.9335	0	0	0	0	0	0	0	2.218	0	1.444	0	0	0	0	0	0	18
Os01g0514000	419	356	332	359.6	391.4	394.5	719	662	693	406.1	412.2	514.6	360.3	441.52	331.83	382.1	297.8	308.7	484	404.8	480.3	428.8	491.7	491.8	293.7	369.6	486.8	235	293	217	18
Os01g0810866	0	0.93	2.16	0	0.904	0.865	2.35	0	0	0	0	0.941	0	0.9335	0	0	2.061	0	0	0	0	0	0	0.722	0	1.886	0	0.93	0	0.83	14
Os01g0696701	15.3	10.2	11.9	9.031	3.616	9.516	7.83	6.33	8.29	12.93	10.96	11.29	9.7967	15.869	5.5438	0	13.4	14.5	7.432	12.09	4.311	10.35	7.126	8.667	7.395	16.97	15.35	13.9	14.3	19.2	1
Os01g0238800	34.7	19.6	33.4	41.05	46.1	38.06	51.7	51.2	70.5	56.04	33.99	69.62	54.426	70.942	35.638	35.82	37.1	34.19	82.68	95.14	69.84	25.88	38.48	30.33	40.14	54.68	30.7	46.3	45.9	48.3	15
Os01g0654800	3.06	1.86	3.23	1.642	1.808	1.73	2.35	2.53	2.76	10.35	5.482	5.645	3.2656	7.4676	5.5438	11.94	1.031	2.072	0.929	0.806	1.724	4.436	2.138	2.167	6.338	3.771	0	3.7	6.69	5	13
Os01g0119500	466	529	482	606.7	693.3	565.7	240	228	211	350	416.6	351.9	390.78	393.92	401.53	513.5	478.2	430	458.9	391	411.3	292.8	282.2	239.1	444.7	414.9	322.4	497	413	528	8
Os01g0667100	212	164	217	202	205.2	187.7	213	200	212	201.8	188.6	202.3	182.87	161.49	171.86	370.2	209.2	201	253.6	229.8	238	165.6	173.2	214.5	178.5	194.2	206.1	176	144	183	6
Os01g0179000	1.02	0.93	1.08	3.284	0	1.73	0.78	3.16	3.46	0	2.193	0	1.0885	1.8669	0.792	11.94	0	0	0	0	0	14.05	17.82	15.89	0	0	2.193	0	0.96	0	17
Os01g0532600	54	60.5	78.7	17.24	13.56	12.11	2.35	0.63	4.15	168.1	187.5	130.8	120.83	133.48	129.09	95.53	78.32	95.32	19.51	26.61	12.07	13.31	8.552	4.333	187	129.2	142.5	146	136	138	3
Os01g0875300	2.04	7.45	3.23	2.463	5.424	4.325	9.39	2.53	4.15	3.449	2.193	2.822	1.0885	2.8004	1.5839	0	11.34	9.324	0	1.613	6.898	2.218	3.563	2.167	4.225	12.26	0	3.7	9.56	8.33	1
Os01g0928400	0	1.86	0	0	0	0	1.57	0.63	0	0.862	1.096	4.704	2.177	0.9335	0	23.88	1.031	1.036	0	1.613	0	0.739	2.138	12.28	0	0	2.193	0	0	1.67	6
Os01g0259966	2.04	0	0	1.642	0.904	2.595	0.78	1.9	5.53	2.587	1.096	0.941	1.0885	4.6673	1.5839	0	0	4.144	1.858	0	0	0	4.276	1.444	4.225	1.886	2.193	0.93	0	0	5
Os01g0501800	####	####	####	34359	32015	29147	####	####	####	77735	82804	#####	49519	46402	49058	78715	44210	53652	42824	36350	46170	25484	22532	33522	64682	70550	61647	####	####	####	20
Os01g0126200	1373	1268	1394	666.6	687	648.8	428	366	308	1188	1135	1557	1238.7	1229.4	1143.6	2746	1635	1456	882.5	837.7	797.6	466.6	396.9	603.1	1327	1395	1316	1556	1573	1603	13
Os01g0837700	2.04	3.73	1.08	1.642	1.808	3.46	3.13	3.8	2.76	2.587	5.482	3.763	2.177	0	1.5839	0	2.061	1.036	1.858	0.806	0.862	0.739	2.138	1.444	8.451	0.943	6.579	0	1.91	1.67	11
Os01g0914700	0	0	0	0.821	0	0	0	0	0	0	0	0	0	0	0	0	0	0	0	0	0.862	0	0	0	0	0	0	0	0	0	16
Os01g0552500	433	397	422	468	484.5	436	582	578	555	420.7	444	436.5	425.61	426.59	417.37	668.7	475.1	473.5	474.7	578.1	481.1	593	583.6	578.5	461.6	464.8	412.3	494	497	513	6
Os01g0153000	5.1	4.66	2.16	1.642	4.52	2.595	2.35	0	4.15	3.449	4.386	5.645	2.177	2.8004	3.1679	0	5.153	5.18	5.574	4.838	4.311	2.958	0	2.889	3.169	1.886	0	3.7	1.91	6.66	16
Os01g0211800	20.4	13	6.47	4.926	1.808	0.865	0.78	0	0	4.311	7.675	5.645	9.7967	13.068	5.5438	47.76	34.01	11.4	0.929	0.806	5.173	1.479	0	0	9.507	21.69	13.16	8.33	9.56	9.16	13
Os01g0500750	31.6	19.6	24.8	23.81	19.89	18.17	3.91	20.2	6.22	9.484	14.25	8.467	14.151	15.869	21.383	71.65	35.04	18.65	7.432	9.675	7.76	21.44	32.78	30.33	25.35	22.63	13.16	32.4	31.6	31.7	13
Os01g0709500	1594	1440	1760	504.1	435.7	583.9	471	514	589	1714	1976	1976	1962.6	1783.8	2394.9	3690	2112	2243	583.4	577.3	499.2	1045	1044	754	2172	2401	2384	2701	2611	2539	13
Os01g0879600	0	0	0	0	0	0	0	0	0	0	0	0	0	0	0	0	0	0	0.929	0	0	0	0	0	0	0	0	0	0	0	16
Os01g0618000	28.5	34.5	26.9	17.24	12.66	13.84	7.05	17.1	13.1	25.87	12.06	16.93	13.062	14.002	14.255	83.59	53.59	23.83	14.86	16.93	30.18	8.133	12.83	10.11	19.01	19.8	15.35	22.2	24.9	13.3	13
Os01g0280400	63.2	71.7	94.8	728.2	743.1	526.8	55.6	45.5	42.2	42.25	38.37	29.17	66.4	99.879	43.558	203	99.96	103.6	472.8	456.4	563	22.92	45.61	23.11	42.25	45.26	35.09	55.5	70.8	52.5	10
Os01g0127900	26.5	41	36.6	36.12	26.22	42.39	29.7	36.1	30.4	28.45	25.22	19.76	31.567	22.403	36.43	23.88	19.58	37.3	29.73	29.03	28.45	68.76	89.08	66.45	19.01	19.8	32.89	17.6	28.7	21.7	17
Os01g0685900	0	3.73	0	0.821	0	3.46	0	0	0.69	1.724	3.289	3.763	1.0885	2.8004	2.3759	0	1.031	1.036	0	0	0	0	3.563	0	4.225	0.943	4.386	1.85	3.83	0	20
Os01g0811001	1.02	1.86	12.9	1.642	0.904	5.19	3.91	1.27	4.15	1.724	1.096	0	1.0885	0.9335	2.3759	0	6.183	1.036	2.787	2.419	2.587	8.873	1.425	5.056	2.113	2.829	2.193	6.48	2.87	2.5	9
Os01g0132800	798	753	847	1520	1679	1495	866	1059	1048	673.4	840.9	676.4	1258.3	975.46	822.06	1302	703.9	786.4	1409	1693	1615	826.6	793.2	877.5	757.4	657.2	600.9	809	772	821	10
Os01g0375000	530	416	408	642	659	646.2	355	332	383	525.1	375	672.7	438.67	538.6	388.06	1099	436.9	497.3	633.5	610.4	625.1	383.7	357.7	350.3	345.4	525.2	482.4	414	503	345	6
Os01g0277700	262	211	207	232.3	259.4	246.5	348	257	358	273.3	286.2	303.9	215.53	266.97	244.72	370.2	202	264.2	270.3	235.4	264.7	265.4	213.8	298.3	248.2	264.9	269.7	208	230	190	6
Os01g0112300	196	216	173	243	230.5	282	319	276	310	256.1	284	290.7	246.01	227.76	258.18	382.1	191.7	181.3	240.6	230.6	216.4	325.3	265.8	258.6	218.7	235.7	217.1	211	228	210	6
Os01g0598000	0	0	0	0.821	0	0	0	0	0	0	0	0	0	0	2.3759	0	0	0	0	2.419	0	0	0.713	0	0	0	0	0	0	0	4
Os01g0772000	227	260	253	309.5	341.7	313.1	358	398	355	244	237.9	244.6	274.31	267.9	270.06	346.3	231.9	230	249	270.9	269.9	360.1	307.1	349.6	270.4	280	247.8	250	243	232	18
Os01g0699400	8.16	5.59	3.23	9.852	2.712	21.63	0	1.9	2.07	5.173	3.289	3.763	14.151	0.9335	0.792	11.94	3.092	3.108	51.09	20.16	20.69	3.697	3.563	8.667	2.113	4.714	2.193	4.63	3.83	3.33	16
Os01g0626900	1685	1711	1816	2002	1953	1804	1198	1420	1349	1442	1472	1345	1729.7	1392.7	1675.8	3988	1820	1877	1641	1984	1815	2096	2131	2017	1818	1542	1382	2045	1974	2175	6
Os01g0967600	524	539	541	559.9	499.9	570.9	532	497	463	598.4	594.2	661.4	569.3	541.4	588.43	1218	582.3	584.3	601	593.4	644.1	513.9	439.7	477.4	541.9	651.5	620.6	617	647	597	6
Os01g0945001	144	143	144	260.2	305.5	333	261	201	230	81.91	83.33	103.5	142.6	133.48	109.29	143.3	156.6	127.4	374.4	335.4	392.3	218.1	207.4	224.6	112	109.4	87.72	103	148	111	10
Os01g0857200	192	177	209	269.3	273	249.1	339	324	304	173.3	228.1	187.2	237.3	286.57	224.92	489.6	237	210.3	311.2	315.3	327.6	250.7	298.6	255.7	217.6	161.2	254.4	210	218	230	6
Os01g0580000	1005	1070	1203	1069	1222	1045	1154	1196	1131	723.4	919.9	761.1	970.96	903.58	850.57	1373	976.9	1035	1130	1167	1055	925.7	889.4	1151	945.4	773.1	712.7	833	914	972	6
Os01g0945250	0	0	1.08	7.389	11.75	3.46	3.91	3.8	0	0	0	0	0	0	0	0	0	0	1.858	1.613	0	0.739	0.713	0	0	0.943	0	3.7	2.87	0.83	2
Os01g0604600	1.02	0	9.7	2.463	1.808	0	7.05	8.86	8.29	1.724	2.193	12.23	4.3541	4.6673	0.792	0	2.061	1.036	0	0	2.587	4.436	2.851	2.889	6.338	2.829	10.96	2.78	2.87	4.17	5
Os01g0555200	424	628	692	317.7	403.2	158.3	247	475	146	550.1	612.9	386.7	561.68	391.12	729.4	764.2	304	406.1	371.6	258	144	149.4	238	155.3	436.3	241.4	285.1	327	532	496	13
Os01g0224300	52	28.9	51.7	32.84	43.39	30.28	40.7	41.1	29.7	60.35	52.63	60.21	72.931	68.142	46.726	83.59	35.04	45.59	50.16	45.96	38.8	34.01	37.77	28.17	47.54	40.54	41.67	35.2	50.7	55.8	13
Os01g0959750	580	401	477	1402	1523	1165	1758	2937	1529	789.8	576.7	527.8	439.76	488.2	733.36	382.1	445.2	470.4	1105	1228	1173	1065	1764	1130	409.9	442.2	502.2	345	282	253	12
Os01g0746700	8.16	7.45	12.9	14.78	9.04	15.57	7.83	9.49	8.98	4.311	8.771	2.822	1.0885	9.3345	3.1679	11.94	3.092	4.144	4.645	3.225	9.484	9.612	5.701	12.28	1.056	15.09	15.35	5.55	17.2	2.5	11
Os01g0898400	52	64.3	40.9	87.84	82.26	88.23	85.3	55	62.9	40.52	53.72	40.46	54.426	56.941	52.27	59.7	46.37	61.13	73.39	73.37	94.84	57.67	62.71	87.39	59.16	62.23	35.09	64.8	49.7	54.2	10
Os01g0159000	0	0.93	0	0	0	0	0	0	0	0.862	2.193	0	0	0	1.5839	0	0	0	0	0	0	0	0	0.722	1.056	0	0	1.85	4.78	0	1
Os01g0939000	42.8	46.6	45.3	101.8	105.8	89.96	148	94.9	74.6	38.8	58.11	39.51	50.072	55.074	51.478	107.5	46.37	42.48	111.5	108	121.6	85.03	86.23	93.89	32.75	39.6	24.12	44.4	47.8	40	12
Os01g0965500	103	102	115	111.7	113.9	102.9	153	154	126	92.25	81.13	102.5	100.14	105.48	112.46	203	98.93	118.1	124.5	129	138	112.4	121.9	114.8	85.57	82.97	105.3	82.3	101	102	6
Os01g0574500	730	700	795	689.6	709.6	749.1	564	427	469	907	1017	935.2	952.45	1019.3	945.61	919.5	999.6	780.1	1054	973.2	1089	355.6	369.1	424.7	865.2	986.2	934.2	806	753	741	16
Os01g0121700	1721	1837	1883	991.7	988.9	1029	1146	1124	1269	1540	1576	1439	2127	2118	2209.6	3415	1874	1697	1061	1109	1109	1145	1013	1123	1698	1758	1627	1828	1917	1865	13
Os01g0224700	32.6	60.5	29.1	41.05	39.77	11.25	16.4	28.5	25.6	14.66	18.64	19.76	29.39	29.87	49.894	143.3	65.95	53.87	39.94	45.96	38.8	45.84	58.44	31.78	52.82	40.54	32.89	50	76.5	80	13
Os01g0743500	0	0	0	0	1.808	1.73	0	1.27	0.69	0	0	0	0	0	0	0	4.122	0	0	0	0	0	0	0.722	0	0	0	0.93	0.96	0	2
Os01g0585100	380	254	391	36.12	32.54	44.12	39.9	66.4	49.8	313.8	262	352.8	701.01	603.01	497.35	835.9	345.2	311.8	41.8	34.67	21.56	35.49	46.32	32.5	361.3	266.8	254.4	370	296	324	13
Os01g0159800	14.3	16.8	12.9	46.8	54.24	13.84	46.2	142	49.8	27.59	23.02	5.645	17.416	7.4676	24.551	47.76	8.244	8.288	10.22	18.54	0	23.66	52.73	17.33	11.62	0.943	10.96	9.25	19.1	22.5	18
Os01g0850800	0	0	1.08	0	0	0	0	0	0	0	0	0	0	0	0	0	0	0	0	0	0	0	0	0	0	0	0	0	0	0	14
Os01g0571766	25.5	21.4	18.3	39.41	30.73	46.71	57.9	56.3	38.7	34.49	27.41	74.32	28.302	46.673	42.766	95.53	17.52	38.33	49.23	46.76	56.91	15.53	21.38	28.89	27.47	12.26	43.86	20.4	28.7	15.8	6
Os01g0912400	1.02	0	3.23	0	0	1.73	0	0	0	0.862	0	0	0	0	0	0	0	0	0.929	0	0	0	0	0	0	0	0	0	0	0	19
Os01g0787300	0	0	0	0	0.904	0	0	0	0	0	0	0	0	0	0	0	0	0	0	0	0.862	0	0	0	0	0	0	0	0	0	10
Os01g0668600	26.5	41	39.9	54.18	59.66	25.09	103	91.1	54.6	66.39	62.49	53.63	37.01	48.539	34.847	11.94	25.76	33.15	36.23	26.61	29.32	74.68	76.25	83.78	36.97	31.11	32.89	30.5	29.6	27.5	18
Os01g0837000	363	314	373	313.6	281.1	210.2	265	322	497	238	150.2	118.5	168.72	109.21	181.36	417.9	150.5	191.7	228.5	292.7	190.5	482.1	497.4	458.6	116.2	132	175.4	105	111	87.5	17

Os01g0924800	141	139	114	163.4	190.7	200.7	277	242	300	167.3	156.8	260.6	197.02	196.02	197.2	346.3	177.3	158.5	247.1	208	241.4	264	214.5	229.7	195.4	217.8	201.7	167	160	151	6
Os01g0653601	0	0	0	0	0	0	0	0	0	0	0	0	0	0	0	0	0	0	0	0	0	0	0.713	0	0	0	0	0	0	0	17
Os01g0818800	0	0	0	0	0	0.865	0	0	0	0	0	0	0	0	0	0	0	0	0.929	0.806	5.173	0.739	0	0	0	0	0	0	0.96	0	16
Os01g0697100	24.5	12.1	17.2	5.747	7.232	3.46	11	5.69	4.84	14.66	20.83	18.82	10.885	13.068	7.1277	11.94	28.85	13.47	0	6.45	1.724	8.133	7.126	20.95	10.56	18.86	17.54	11.1	9.56	7.5	3
Os01g0762400	192	185	185	260.2	293.8	249.1	184	192	222	151.7	132.7	147.7	217.7	184.82	190.86	406	146.3	179.2	234.1	277.4	263	200.4	192.4	158.2	140.5	145.2	146.9	154	217	168	6
Os01g0955700	727	454	532	262.7	229.6	357.3	132	169	290	365.6	297.1	540	556.23	471.39	423.7	1445	614.2	533.6	539.7	441	561.3	425.9	343.5	346.7	444.7	456.3	453.9	522	500	404	13
Os01g0869000	1561	1676	1752	2551	2821	2177	1897	2097	1858	2030	2291	1625	2942.3	2448.4	2632.5	2830	1229	1421	1466	1430	1476	1667	1802	1571	1894	1480	1653	1449	1409	1721	4
Os01g0902000	224	260	237	694.5	737.6	601.2	452	764	692	122.4	137	139.2	119.74	97.079	188.49	871.7	282.4	329.5	402.2	500.7	650.1	502.8	679.1	468.7	409.9	165.9	131.6	168	253	227	12
Os01g0550600	4.08	4.66	0	4.926	1.808	6.055	25.1	13.3	21.4	3.449	0	0	3.2656	3.7338	0.792	0	0	2.072	4.645	1.613	3.449	14.05	12.11	20.95	0	1.886	2.193	4.63	0	0.83	18
Os01g0389700	199	319	198	662.5	476.4	506.9	116	129	267	68.97	30.7	29.17	51.16	42.005	73.653	203	130.9	153.3	525.8	528.1	649.2	503.5	355.6	675.3	28.52	49.03	39.47	36.1	72.7	60	10
Os01g0775600	389	437	420	432.7	482.7	426.5	682	664	593	497.5	480.2	505.2	374.45	431.25	412.61	573.2	398.8	430	448.7	483	459.6	401.5	440.4	396.5	414.1	416.7	447.4	443	384	373	18
Os01g0182700	8.16	6.52	3.23	6.568	10.85	6.055	26.6	15.2	6.91	6.035	14.25	11.29	6.5311	5.6007	0.792	11.94	10.31	11.4	14.86	17.74	14.66	34.01	12.83	23.83	8.451	16.03	30.7	13.9	17.2	22.5	9
Os01g0242500	196	196	223	194.6	188.9	207.6	300	283	340	203.5	221.5	242.7	234.03	249.23	220.96	513.5	211.3	220.7	281.5	270.9	243.1	284.7	268	262.2	232.4	247	258.8	210	203	233	6
Os01g0221300	108	109	98.1	156.8	148.3	141.9	134	130	130	96.56	91	97.85	107.76	125.08	91.076	131.4	88.63	111.9	125.4	114.5	106.9	111.6	106.2	130	79.23	84.86	100.9	112	99.5	84.1	12
Os01g0237366	0	0.93	0	0	0	0	0	0	0	0	0	0	0	0	0	0	0	0	0	0	0	0	0	0.722	0	0	0	0	0	0	14
Os01g0923300	1140	1182	1102	1007	1014	1029	1712	1801	1731	1175	1297	1158	1259.4	1221.9	1260	2054	1310	1082	988.4	947.4	1038	1413	1208	1426	1292	1205	1077	1208	1184	1331	6
Os01g0772600	1358	1400	1395	1435	1402	1355	1172	1289	1332	1442	1454	1424	1422.7	1461.8	1392.3	1576	1564	1371	1358	1465	1224	1077	1135	1166	1390	1357	1333	1402	1347	1365	13
Os01g0563500	65.2	64.3	39.9	76.35	69.61	72.66	79.9	74.6	47.7	53.46	60.3	30.11	48.983	58.807	53.854	47.76	46.37	54.91	47.38	50.8	49.15	70.98	79.82	78.72	53.87	43.37	48.24	67.5	66.9	49.2	17
Os01g0110100	77.5	62.4	49.6	27.09	27.12	19.03	3.13	7.59	0.69	33.63	37.28	63.98	16.328	44.806	45.142	71.65	52.56	101.5	17.65	2.419	0.862	11.09	15.68	5.056	24.3	57.51	94.3	56.4	65	69.1	3
Os01g0277600	759	625	710	569.8	565.9	604.7	433	426	415	568.2	706.1	605.9	827.28	702.89	799.89	1242	620.4	632	555.5	581.3	569.1	509.4	480.3	476	693	677	559.2	628	610	658	13
Os01g0555800	46.9	65.2	58.2	220	293.8	160.9	274	284	240	43.11	42.76	71.5	31.567	31.737	28.511	59.7	25.76	41.44	184.9	197.5	215.6	147.9	127.6	206.6	34.86	16.97	15.35	19.4	16.3	27.5	12
Os01g0664100	508	471	592	380.1	355.3	418.7	439	451	455	510.4	559.2	546.6	628.08	715.96	662.88	1325	776	635.1	567.6	474.9	425.9	429.6	398.4	387.1	517.6	586.4	574.5	695	689	621	13
Os01g0652100	0	0	0	0	0	2.595	1.57	0	0	0	0	0	0	0	0	0	0	0	0	0	0.862	0	0	0.722	0	0	0	0	0	0	2
Os01g0958100	1605	1631	1698	2024	2263	2022	2566	2514	2534	1468	1765	2005	1959.3	1766.1	1542	2902	1602	1785	2173	2168	2296	2050	1908	2264	1643	1625	1399	1424	1489	1641	12
Os01g0883000	38.7	39.1	56	45.15	75.03	69.2	136	161	155	37.07	53.72	27.28	51.16	28.004	50.686	71.65	36.07	48.69	53.88	53.21	56.91	102.8	125.4	116.3	44.37	26.4	41.67	31.5	41.1	36.7	18
Os01g0943800	138	209	189	336.6	261.2	338.2	38.4	76.5	65.6	85.36	76.75	56.45	146.95	138.15	131.47	214.9	150.5	149.2	115.2	125	148.3	113.1	124.7	83.06	114.1	94.28	41.67	212	161	157	8
Os01g0924700	0	0	0	0	0	0	0	0	0	0	0	0	0	0	0	0	1.031	0	0.929	0	0	0	0	0	0	0	0	0	0	0	16
Os01g0651350	5.1	2.79	5.39	0	0	2.595	5.48	3.16	4.15	2.587	0	2.822	7.6196	5.6007	9.5036	11.94	6.183	1.036	1.858	0	1.724	0.739	8.552	1.444	2.113	1.886	0	5.55	11.5	0.83	13
Os01g0882100	8.16	1.86	0	1.642	0.904	0.865	0	4.43	1.38	2.587	1.096	0	1.0885	2.8004	0	0	4.122	2.072	5.574	0	0	1.479	1.425	0.722	2.113	0	0	1.85	0	0	19
Os01g0609300	333	306	421	518.9	286.6	464.5	297	338	460	368.2	241.2	315.2	207.91	262.3	293.82	358.2	365.8	336.7	335.4	255.6	294.9	387.4	660.6	404.5	338	376.2	315.8	357	318	397	17
Os01g0595900	0	0	0	2.463	0	0	1.57	0.63	0	0	2.193	0	0	0	0.792	0	0	0	0	0	0.862	0.739	2.851	0	2.113	0.943	2.193	0.93	2.87	0	17
Os01g0765000	248	209	259	308.7	287.5	280.3	197	180	167	156.1	126.1	77.15	195.93	151.22	127.51	191.1	143.2	192.7	335.4	378.1	272.5	197.4	226.6	210.2	113	107.5	83.33	144	161	127	10
Os01g0805000	1011	1219	1140	2449	2553	2106	5022	4373	4804	1576	1802	2666	1384.6	1054.8	832.36	1588	728.6	884.8	2661	2786	2863	2285	2211	3664	1088	1040	1085	556	662	559	18
Os01g0665300	941	953	935	813.6	753	798.4	1512	1305	1437	990.7	946.2	742.3	1006.9	1008.1	1151.5	1743	896.6	868.2	810	864.3	862.2	1470	1352	1249	1052	995.6	894.7	1022	891	1055	6
Os01g0358100	0	2.79	3.23	1.642	0.904	0.865	6.26	0	3.46	3.449	2.193	5.645	2.177	0	0	0	4.122	4.144	1.858	0	0.862	1.479	0.713	3.611	3.169	2.829	0	2.78	0.96	0.83	5
Os01g0896500	####	####	####	3625	3405	3136	3069	4207	4123	11884	14012	12691	14974	12752	14426	18413	10501	11820	3602	3557	4318	3045	2788	3318	13486	12446	10397	9725	####	####	3
Os01g0229200	659	658	673	637.9	609.3	609.9	1358	1382	1466	685.4	660	768.6	751.08	690.75	768.21	1158	668.8	610.2	645.6	610.4	621.7	847.3	798.2	1021	664.5	697.7	756.6	573	577	639	18
Os01g0710800	379	393	423	475.3	465.5	485.3	591	654	600	397.5	412.2	446.9	451.74	355.65	432.41	609	477.1	443.4	554.6	580.5	539.7	580.4	498.8	608.9	446.8	451.6	429.8	464	525	497	18
Os01g0763300	592	537	559	449.9	448.4	456.7	679	512	652	688	606.3	780.9	708.63	637.55	536.95	931.4	631.7	591.6	669.8	622.4	601.8	445.1	474.6	494	666.6	701.5	657.9	558	574	552	13
Os01g0254400	0	0	0	0	0	0	0	0	0	0	0	0	0	0	0	0	0	0	0	0	0	0	2.138	0	0	0	0	0	0	0	17
Os01g0131900	38.7	28.9	15.1	35.3	37.06	44.98	57.2	48.7	52.5	31.04	59.21	42.34	51.16	46.673	24.551	23.88	30.92	26.94	33.44	54.02	50.01	34.01	37.06	39	21.13	33	35.09	28.7	24.9	28.3	15
Os01g0337600	1629	1708	1737	1577	1526	1490	1127	1359	1221	1375	1542	1164	1865.7	1645.7	1953	3415	1703	1765	1265	1466	1284	1562	1410	1216	1530	1441	1441	1972	1822	2087	13
Os01g0748200	50	61.5	56	64.04	62.37	62.28	81.4	72.7	69.8	50.01	42.76	47.98	47.895	44.806	49.894	59.7	61.83	47.66	96.61	73.37	81.05	58.41	71.98	66.45	44.37	32.06	46.05	62.9	51.6	53.3	12
Os01g0296166	2.04	0.93	1.08	0.821	0	4.325	2.35	1.9	5.53	1.724	3.289	1.882	1.0885	0	0.792	0	1.031	9.324	6.503	1.613	1.724	4.436	0.713	1.444	3.169	2.829	4.386	0.93	5.74	3.33	1
Os01g0524700	1534	1267	1788	1149	1282	1099	447	512	412	1061	1184	1041	3187.2	2583.8	2449.6	2782	1261	1414	952.2	1163	1107	464.3	430.4	481	1230	869.3	798.2	1717	1505	1730	13
Os01g0838100	807	606	802	633.8	664.4	687.7	367	362	457	594.9	601.9	800.6	863.2	831.71	700.89	1911	864.6	910.7	693	734.5	773.4	697.2	483.2	474.5	677.1	754.3	796	820	875	790	13
Os01g0723500	0	0	1.08	0	0	0	0	0	1.38	0.862	0	0	0	0	0	0	0	0	0	0	0	0	0	0	0	0	0	0	0	0	7
Os01g0220100	0	0.93	0	0	0	0	0	0	0	0	0	0	1.0885	0.9335	0	0	0	0	0	0	0	0	0	0	0	0	0	0	0	0	4
Os01g0827600	196	222	232	107.5	92.2	91.7	93.9	84.1	81.5	200	174.3	213.6	152.39	154.02	159.98	191.1	171.1	172	72.46	69.34	110.4	177.5	211.7	191.4	151.1	165.9	199.6	207	143	178	3
Os01g0595201	0	0.93	0	0.821	0	0	0	0	0	0	0	0	0	2.8004	0	0	2.061	0	0	0.806	0	0	0	0	0	0	0	0	0	0	4
Os01g0823900	98.9	80.1	123	59.93	65.09	64.88	101	76.5	102	111.2	88.81	81.85	72.931	59.741	94.244	274.6	130.9	89.1	78.03	80.63	44.84	195.9	144	143.7	135.2	120.7	92.1	128	85.1	115	6
Os01g0283500	530	338	504	128.1	109.4	128.9	196	149	157	524.2	502.1	652.9	453.91	400.45	507.65	1027	510.1	373	181.1	216.1	197.4	137.5	163.2	176.9	585.2	478	517.5	455	350	379	13
Os01g0292200	4060	4106	3656	2481	2010	3316	1295	1075	1213	6015	7168	6825	5386	5211.5	5563.6	5290	5512	4292	4080	3977	4167	819.2	990.6	850.1	4834	5320	4818	4018	3997	3654	3
Os01g0128000	0	0	0	0.821	0	0	0	1.9	0.69	0.862	0	0.941	0	0	1.5839	0	0	0	0	0	0	0.739	0.713	0	1.056	0.943	0	0	0	0	5
Os01g0775200	597	590	554	342.3	289.3	285.5	724	618	677	419.9	411.1	518.4	431.05	511.53	540.91	656.8	636.9	579.1	312.1	262	308.7	812.6	741.1	760.5	694	602.5	758.7	607	455	563	5
Os01g0130900	33.6	15.8	17.2	23.81	37.06	34.6	73.6	51.9	126	28.45	40.57	44.22	28.302	13.068	19.799	11.94	13.4	22.79	69.67	43.54	65.53	253.6	116.9	171.2	34.86	40.54	52.63	15.7	16.3	18.3	9
Os01g0921200	624	744	705	692.9	705.1	717.1	672	639	753	619	638.1	461.9	665.09	681.42	642.28	1349	618.3	649.6	606.6	676.5	647.5	746	875.8	751.9	631.7	561.9	530.7	716	669	651	6
Os01g0656400	119	119	191	68.14	78.65	77.85	212	172	149	210.4	72.36	71.5	71.842	88.678	106.92	191.1	163.9	112.9	64.1	99.98	76.74	92.42	107.6	116.3	55.99	99.94	127.2	69.4	71.7	43.3	5
Os01g0133900	1.02	0	0	0	0	0	0	0	0.69	0	0	0	1.0885	0	0	11.94	6.183	0	0	0	0	0	0	0.722	0	1.886	2.193	1.85	0.96	0.83	6
Os01g0710250	147	137	141	132.2	125.7	102.9	91.6	116	90.5	94.84	112.9	79.97	113.21	102.68	94.244	214.9	117.5	115	144	117.7	106.9	142	117.6	121.3	113	99	131.6	127	138	136	6
Os01g0879200	1.02	6.52	4.31	11.49	11.75	17.3	11.7	7.59	12.4	22.42	12.06	11.29	4.3541	7.4676	4.7518	35.82	6.183	8.288	13.01	15.32	20.69	5.915	9.977	6.5	2.113	5.657	6.579	7.4	18.2	8.33	6
Os01g0794400	272	204	301	89.49	103.1	79.58	184	119	108	328.5	212.7	335.9	193.76	224.03	165.52	119.4	216.4	218.6	104	74.18	71.56	128.7	124.7	116.3	169	253.6	234.6	237	237	166	3
Os01g0199700	17.3	27	11.9	10.67	9.04	5.19	61.8	113	71.9	43.11	42.76	37.63	35.921	24.27	28.511	119.4	55.65	41.44	3.716	8.063	13.8	44.36	44.18	44.06	31.69	34.89	32.89	33.3	27.7	45	6
Os01g0222500	28.5	49.4	28	32.02	29.83	37.2	57.9	65.8	49.1	42.25	24.12	30.11	16.328	22.403	19.007	35.82	22.67	22.79	18.58	21.77	13.8	42.88	61.29	70.78	40.14	42.43	37.28	13	5.74	20	18
Os01g0176400	6.12	0	0	0.821	0	0	1.57	0	0	1.724	2.193	11.29	0	1.8669	1.5839	0	3.092	2.072	1.858	0	0	0	0.713	0.722	3.169	10.37	19.74	3.7	4.78	2.5	11
Os01g0600200	1066	1093	1103	885	889.5	808.8	968	1007	923	1031	1051	1123	1043.9	1107.1	1091.3	2209	1033	1014	745	774	723.4	877.7	899.3	865.2	999.3	966.4	1000	1041	996	1016	13
Os01g0554150	4.08	4.66	3.23	4.926	0	0	2.35	9.49	1.38	4.311	1.096	4.704	4.3541	3.7338	7.1277	0	4.122	1.036	10.22	4.838	4.311	2.218	3.563	1.444	2.113	6.6	10.96	4.63	4.78	3.33	11
Os01g0103400	28.5	37.3	32.3	29.56	25.31	37.2	47.8	60.1	67	57.77	65.78	80.91	35.921	47.606	46.726	59.7	46.37	44.55	66.88	57.25	81.05	42.88	34.92	44.78	54.93	55.63	59.21	24.1	42.1	28.3	15
Os01g0601000	89.7	98.7	101	16.42	28.02	25.95	234	93.6	135	81.91	112.9	40.46	153.48	161.49	81.572	191.1	103.1	110.9	32.51	47.57	56.91	42.88	32.07	25.28	89.79	43.37	39.47	104	131	93.3	5
Os01g0812850	0	0	0	0	0	0	0	0	0	0	0	0	0	0	0	0	0	0	0.929	0.806	0	0	0	0	0	0	0	0	0	0	16
Os01g0151700	749	586	710	27.91	37.97	49.31	20.4	21.5	8.98	600.1	687.4	987.9	916.53	1153.7	929.77	1027	1207	852.7	146.8	78.21	157.8	2.958	10.69	18.06	878.9	899.5	925.4	829	846	815	3
Os01g0839200	44.9	3.73	21.6	8.21	0.904	11.25	23.5	8.22	7.6	18.11	10.96	13.17	1.0885	17.736	15.047	35.82	53.59	27.97	11.15	13.71	25	51.76	59.15	121.3	35.92	97.11	131.6	22.2	44	25.8	11
Os01g0738500	140	135	121	153.5	141.9	130.6	103	107	129	155.2	143.6	143.9	137.15	123.22	151.27	262.7	105.1	158.5	150.5	117.7	150	160.4	166	144.4	133.1	130.1	136	178	178	170	6
Os01g0225500	0	0	0	0	0	0	0.78	0	0.69	0	0	0	0	0	0	0	0	0	0	0	0	1.479	0	0	0	0	0	0	0	0.83	9
Os01g0652800	7.14	6.52	2.16	0.821	2.712	5.19	7.05	1.27	0.69	0	1.096	4.704	1.0885	4.6673	1.5839	0	6.183	6.216	6.503	1.613	6.898	0.739	3.563	5.056	0	4.714	24.12	1.85	8.61	0.83	11
Os01g0795050	23.4	21.4	17.2	25.45	40.68	25.95	15.7	31	11.7	12.93	21.93	16.93	32.656	21.469	16.631	11.94	23.7	31.08	32.51	41.12	28.45	11.83	16.39	27.45	14.79	8.486	24.12	17.6	15.3	15.8	10
Os01g0907400	153	200	197	305.4	291.1	271.6	216	182	160	180.2	193	165.6	176.34	169.89	217	441.8	158.7	145	232.2	280.6	233.7	285.4	295.7	258.6	200.7	162.2	136	232	227	230	6
Os01g0944900	30.6	72.7	74.4	67.32	75.03	57.09	30.5	36.1	31.1	113.8	70.17	69.62	40.275	41.072	89.492	71.65	35.04	54.91	52.02	119.3	50.87	27.36	42.05	29.61	35.92	117.9	65.79	89.7	66	37.5	7
Os01g0551900	432	469	444	441.7	454.7	401.4	406	453	393	474.2	525.2	466.6	522.49	449.92	491.81	871.7	446.2	450.7	346.5	413.6	408.7	449.5	504.5	478.8	506	481.8	388.1	461	464	540	6
Os01g0824900	1.02	0.93	3.23	0	0.904	0	0.78	0.63	0	3.449	1.096	3.763	1.0885	0	0	23.88	3.092	3.108	1.858	5.644	2.587	2.218	2.138	4.333	1.056	11.31	24.12	6.48	10.5	0.83	11
Os01g0780500	962	901	922	925.2	982.6	1001	985	856	823	1023	903.4	928.6	814.21	940.92	917.1	2125	1055	971.8	904.8	848.2	977.8	879.9	926.4	889.8	1029	990	969.3	928	841	891	6
Os01g0966300	331	285	232	523.8	631.9	624.6	443	460	408	251.8	225.9	334.9	301.52	386.45	255.81	525.4	300.9	339.8	601	569.2	690.6	394.1	455.4	407.3	216.6	284.7	337.7	242	288	244	10
Os01g0634300	282	419	347	445.8	489	429.9	205	236	182	181.1	201.7	128	232.94	211.89	281.94	573.2	288.5	250.7	165.4	263.7	242.3	355.6	391.9	269.4	253.5	169.7	184.2	353	290	353	8
Os01g0917801	0	0	0	0	0	0	0	0	0	0	0	0.941	0	0	0	0	0	0	0	0	1.724	0	0	0	0	0	2.193	0	0	0	11
Os01g0279200	1209	1213	1204	1085	1194	1073	1167	1220	1178	1233	1244	1309	1322.6	1468.3	1389.1	1887	1293	1205	1196	1032	1074	923.5	1086	1049	1198	1298	1382	1231	1160	1206	13
Os01g0604550	1.02	0	0	0.821	0.904	2.595	3.91	3.16	2.07	3.449	1.096	0	1.0885	0	3.9598	11.94	0	2.072	0.929	0	2.587	5.176	1.425	2.889	3.169	5.657	2.193	2.78	0	1.67	6
Os01g0301500	0	0	2.16	0	2.712	0	0	1.27	0	0	0	0	0	1.8669	0	0	0	1.036	2.787	0	0	0	0	1.444	1.056	0	2.193	0.93	0.96	0	11
Os01g0833600	742	557	734	42.69	31.64	57.96	26.6	31	24.9	588	540.5	674.6	796.8	760.76	767.42	1564	884.2	696.2	106.8	98.37	101.7	17.01	20.67	23.83	623.3	653.4	528.5	709	754	723	13
Os01g0269000	429	412	394	321.8	373.3	306.2	341	347	346	495.8	508.7	411.1	535.55	503.13	600.31	609	343.2	342.9	396.7	319.3	331.1	292.8	322.1	304.1	413	388.5	368.4	406	396	407	13
Os01g0267800	2.04	1.86	0	4.105	4.52	2.595	5.48	13.3	13.1	0.862	0	3.763	0	0.9335	0	0	1.031	0	6.503	3.225	6.036	17.75	11.4	13	1.056	0.943	6.579	1.85	0	0	18
Os01g0866100	2454	1723	1732	871.1	862.4	810.6	1364	1419	1285	1418	1467	1899	1240.9	1671.8	1515	1648	1746	1612	993.1	1058	1008	2053	1799	1923	1743	1849	2276	1979	1437	1470	3
Os01g0164400	502	437	515	413	391.4	397.1	365	361	422	501.8	523	620.9	558.41	664.62	521.91	859.7	505	575	454.3	450.7	463.9	354.9	384.8	372	550.4	632.6	684.2	480	467	471	13
Os01g0879400	397	404	409	431	424.9	346.9	444	453	402	438.9	419.9	397	378.8	379.91	383.31	704.5	371	371.9	417.1	428.9	393.2	398.5	387	366.9	434.2	381.9	418.8	358	421	367	6
Os01g0937400	294	363	322	296.4	330.9	292.4	262	191	229	206.9	253.3	225.8	217.7	206.29	230.46	453.8	297.8	283.9	195.1	212.9	195.7	321.6	325	279.5	230.3	233.8	182	259	321	312	8
Os01g0140100	1.02	1.86	1.08	0.821	0	0.865	2.35	0	4.15	2.587	1.096	0.941	0	0	0.792	0	2.061	6.216	2.787	2.419	0	2.218	5.701	1.444	2.113	4.714	0	2.78	0.96	0	17
Os01g0976100	31.6	53.1	43.1	47.62	67.8	53.63	12.5	19.6	16.6	7.76	5.482	7.527	19.593	13.068	15.839	59.7	18.55	22.79	24.15	16.93	12.07	66.54	36.34	24.56	14.79	18.86	2.193	29.6	30.6	27.5	8
Os01g0588200	33.6	27	15.1	32.84	37.97	51.04	79.1	63.3	68.4	45.7	19.74	33.87	30.479	42.939	36.43	35.82	36.07	34.19	57.6	41.93	65.53	34.01	47.03	65.72	19.01	28.29	50.44	38.9	31.6	22.5	15
Os01g0214200	135	182	180	489.3	584	326.1	633	778	621	145.7	136	118.5	162.19	141.88	152.85	143.3	109.2	117.1	402.2	308	261.3	451.8	597.9	415.3	122.5	92.4	83.33	177	172	167	12
Os01g0137875	0	0	0	0	0	0	0	0	0	0	0	0	0	0	0	0	0	0	0	0	0	0.739	0	0	0	0	0	0	0	0	9
Os01g0102850	90.7	91.3	83	764.3	706.9	1100	142	82.2	118	70.7	95.39	82.79	92.524	117.61	85.532	131.4	97.9	92.21	964.3	1065	1219	132.4	99.06	100.4	83.45	84.86	74.56	76.8	72.7	92.5	10
Os01g0308600	0	0	0	0	0	0	0	0	0	0	0	0	0	0.9335	0	0	0	0	0.929	0.806	0.862	1.479	3.563	0	0	0	2.193	0	0.96	0	17
Os01g0819000	525	487	594	394.1	482.7	423.9	445	402	413	607	573.4	646.3	512.69	581.54	664.46	895.6	515.3	514.9	371.6	421.7	402.7	385.2	313.6	336.6	658.1	654.3	572.4	565	573	549	13
Os01g0751300	838	786	791	563.2	587.6	544.1	802	744	736	796.7	792.7	850.5	890.41	845.71	852.95	1433	903.8	869.2	746.9	657.9	719.1	526.4	525.9	625.5	839.8	877.8	750	787	836	805	13
Os01g0550100	952	971	1015	1096	1040	1008	819	899	928	920	796	859	904.56	1009.1	964.61	1827	931.6	818.5	954	867.6	907.9	925.7	957.8	901.4	957.1	961.7	1009	938	872	980	6
Os01g0795832	59.1	41	55	23.81	30.73	33.74	75.9	67	95.4	30.18	33.99	21.64	42.452	35.471	41.182	35.82	71.11	50.77	35.3	33.06	36.21	76.16	62	83.06	29.58	29.23	28.51	28.7	30.6	22.5	18
Os01g0646300	1.02	2.79	1.08	1.642	0.904	1.73	0.78	0	0.69	0.862	0	0	1.0885	0	0	0	1.031	1.036	2.787	3.225	0.862	2.958	0	4.333	0	0	0	0	0	0	9
Os01g0196600	194	134	148	156.8	153.7	154.8	72.8	98	108	124.2	137	115.7	80.55	124.15	105.33	286.6	142.2	123.3	173.7	146.7	140.5	110.9	131.8	137.9	114.1	116.9	157.9	116	123	157	6
Os01g0149350	6.12	5.59	2.16	40.23	41.58	48.44	28.2	19.6	18.7	0.862	1.096	0.941	5.4426	1.8669	0	0	2.061	1.036	26.94	44.35	27.59	33.27	27.79	32.5	0	0	0	1.85	1.91	1.67	12
Os01g0165800	930	846	986	352.2	353.5	371.1	251	266	234	728.5	850.8	746.1	1087.4	1047.3	1047	2352	1258	1195	397.6	432.2	390.6	332.7	324.2	229.7	856.7	999.4	883.7	1167	1135	1208	13
Os01g0920300	2650	3204	3297	7771	8289	7196	5560	6281	5740	3356	3992	3704	3664	3114	2881.2	4657	2098	2634	7479	8106	8137	3800	3377	4724	2777	2418	1987	1954	2232	2414	10
Os01g0176300	18.4	17.7	15.1	119	122.9	89.1	68.1	45.5	43.5	16.38	13.16	1.882	19.593	17.736	14.255	0	4.122	8.288	49.23	65.31	42.25	67.28	64.85	41.89	4.225	14.14	17.54	15.7	5.74	14.2	12
Os01g0940100	378	339	367	331.7	332.7	308	210	216	261	294	231.3	268.1	268.86	357.51	363.51	453.8	345.2	327.4	270.3	233.8	293.2	207.8	192.4	160.3	326.4	251.7	337.7	272	246	267	13
Os01g0265100	3.06	6.52	3.23	10.67	6.328	34.6	8.61	0	7.6	15.52	6.578	10.35	2.177	5.6007	3.9598	0	6.183	8.288	10.22	4.031	12.07	4.436	9.264	20.22	3.169	8.486	39.47	6.48	12.4	2.5	11
Os01g0921800	325	270	306	179	178.1	169.5	213	242	169	430.2	447.3	670.8	472.42	324.84	392.02	740.3	352.4	395.8	220.2	347.5	250	176.7	178.2	215.2	466.9	508.2	440.8	343	384	364	13
Os01g0257400	120	74.5	75.4	54.18	50.62	54.5	117	115	76.7	88.81	73.46	127	63.134	92.412	68.901	119.4	89.66	121.2	67.81	70.95	60.36	119	116.2	116.3	81.34	107.5	114	74	91.8	63.3	5
Os01g0895000	26.5	19.6	11.9	8.21	3.616	3.46	3.13	4.43	8.29	12.93	13.16	15.99	5.4426	7.4676	22.175	0	22.67	17.61	6.503	8.869	13.8	46.58	37.77	40.45	22.18	15.09	21.93	37.9	15.3	9.16	9
Os01g0253400	2.04	0.93	0	3.284	0.904	0	0	0	0	0	2.193	0	0	1.8669	0	11.94	2.061	0	0	0	0.862	0.739	0	0	1.056	0.943	0	2.78	0.96	0	6

Os01g0863800	0	0	0	0.821	0	0	0	0	0	0	0	0	0	0	0	0	0	0	0.929	0.806	0	0	0	0	0	0	0	0	0	0	16
Os01g0227350	228	258	261	101	116.6	127.2	46.2	79.1	58	230.2	266.4	219.2	250.36	221.23	258.18	382.1	203	260	112.4	117.7	103.5	65.81	99.06	90.28	265.1	268.7	280.7	312	279	312	13
Os01g0140700	1.02	0	0	3.284	0	2.595	0	0	2.07	0	0	0	0	0	0	0	0	0	1.858	0	0	11.83	2.138	5.778	0	0	0	0	1.91	0	9
Os01g0292700	156	232	155	123.1	125.7	122	899	1070	949	285.4	328.9	331.2	181.78	186.69	223.33	143.3	277.2	320.1	131.9	108.8	154.3	781.5	678.4	734.5	320.1	332.8	298.2	247	226	263	18
Os01g0357100	18.4	14	21.6	51.72	63.28	43.25	25.1	26.6	33.2	17.24	21.93	15.99	35.921	24.27	27.719	47.76	17.52	23.83	21.37	46.76	23.28	59.15	40.62	36.83	25.35	15.09	21.93	33.3	29.6	41.7	2
Os01g0106500	96.9	119	61.4	264.4	253.1	217.1	301	240	276	117.3	112.9	171.2	143.68	163.35	129.88	119.4	125.7	114	249	216.9	226.8	153.1	166	159.6	90.85	138.6	155.7	125	131	109	12
Os01g0852200	468	499	457	1009	1039	776.8	1026	1064	994	396.6	495.6	558.8	563.85	446.19	448.25	740.3	381.3	405.1	1011	1090	1110	828.9	823.1	879	391.9	330.9	348.7	394	451	428	12
Os01g0232500	364	396	405	257	245	243.1	182	250	201	235.4	231.3	156.2	262.33	245.5	256.6	465.7	318.4	347.1	129.1	154	140.5	382.3	386.2	331.5	256.7	257.4	214.9	348	317	409	8
Os01g0934350	16.3	20.5	20.5	4.926	2.712	1.73	11	1.27	11.1	16.38	6.578	21.64	6.5311	5.6007	16.631	11.94	42.25	34.19	1.858	0	3.449	5.176	4.276	2.889	19.01	29.23	24.12	22.2	21	12.5	3
Os01g0752600	230	215	240	160.1	183.5	204.2	129	151	133	166.4	197.4	175	227.5	206.29	232.05	465.7	226.7	250.7	204.4	197.5	209.5	170.1	138.3	155.3	182.8	191.4	149.1	269	265	243	13
Os01g0753400	0	0	0	0	0.904	0	0	0	0	0	0	0	0	0	0	0	0	0	0	0	0	0	0	0	0	0	0	0	0	0	2
Os01g0618800	4625	4962	5440	3382	3444	3296	5618	5846	5720	5180	5896	5204	7042.7	6767.5	6536.9	9457	5650	5459	4096	3916	4084	4246	4081	4742	5771	5779	5342	5611	6109	5945	13
Os01g0784700	106	112	131	302.1	261.2	333.9	38.4	51.9	44.2	167.3	131.6	175.9	199.2	194.16	129.09	119.4	165.9	154.4	437.5	444.3	564.8	49.54	69.13	85.22	192.3	165	289.5	137	193	167	16
Os01g0924000	3840	3853	4275	3413	3703	3598	6976	6590	7175	4716	5011	6475	5617.9	4549.6	4727.2	5672	4279	3935	4529	4131	4682	3917	3697	5318	4191	4469	4070	3274	4060	3577	5
Os01g0513400	902	840	843	483.6	448.4	540.7	461	490	620	807	868.3	883.4	795.71	780.37	835.52	1302	838.9	831.9	508.1	597.5	524.2	606.3	555.1	534.5	820.8	867.4	771.9	774	723	846	13
Os01g0278466	41.8	34.5	31.2	59.11	59.66	51.04	108	89.8	127	54.32	54.82	73.38	52.249	45.739	48.31	11.94	48.43	32.12	62.24	58.05	67.25	77.64	72.69	72.22	58.1	50.91	74.56	32.4	33.5	31.7	18
Os01g0175400	3.06	17.7	7.54	119.9	122	102.1	78.3	63.3	91.2	20.69	10.96	8.467	14.151	24.27	14.255	23.88	7.214	5.18	55.74	89.5	59.49	116.1	84.8	106.9	7.395	4.714	6.579	7.4	11.5	8.33	12
Os01g0698800	17.3	24.2	11.9	3.284	0	2.595	0.78	0.63	0	5.173	0	4.704	4.3541	1.8669	0	0	6.183	2.072	2.787	0.806	0	0	2.138	1.444	0	0	6.579	0	4.78	1.67	14
Os01g0121000	0	0	2.16	0	0	1.73	0	0	0	0.862	0	0	0	0	0	0	0	0	0	0	0	0	0	0	0	0	0	0	0	0	7
Os01g0130000	237	232	205	140.4	130.2	131.5	68.1	54.4	50.4	135.4	125	213.6	145.86	136.28	148.89	167.2	242.2	210.3	96.61	83.85	87.95	148.6	147.5	137.9	176.4	183.9	166.7	165	125	201	14
Os01g0178500	149	243	130	75.53	96.72	44.98	408	216	290	248.3	358.5	320.8	246.01	218.43	293.03	286.6	155.6	170.9	37.16	37.09	42.25	424.4	235.2	309.8	210.2	302.7	245.6	156	193	194	5
Os01g0337900	558	511	596	840.7	956.4	865	784	689	660	618.2	552.6	690.6	619.37	545.14	503.69	704.5	382.3	432	903.9	773.2	852.7	497.6	458.2	570.6	436.3	478	497.8	319	369	355	10
Os01g0517500	0	0	0	0	0	0	0	0	0	1.724	0	1.882	0	0	0	0	0	0	0	0	0	0	0	0	0	0.943	0	0	0	0	20
Os01g0133200	1.02	0	1.08	0	0	0	0	0	0	0	0	0	0	0	0	0	1.031	0	0	0	0.862	0	0	0	0	0	0	0	0	0	19
Os01g0201250	6.12	8.38	7.54	10.67	9.04	11.25	19.6	24.7	22.8	11.21	16.45	26.34	10.885	11.201	11.879	11.94	7.214	2.072	13.01	12.09	25	8.873	10.69	12.28	12.68	13.2	10.96	9.25	9.56	7.5	15
Os01g0344620	2.04	0.93	5.39	1.642	1.808	1.73	6.26	1.9	0	2.587	0	4.704	0	0	1.5839	0	4.122	2.072	4.645	0	1.724	4.436	9.264	1.444	4.225	0	2.193	5.55	7.65	0.83	17
Os01g0679900	663	561	606	226.6	193.4	237.9	492	426	344	682	731.3	974.7	637.87	730.89	617.73	1218	848.1	723.2	454.3	358.8	419.9	350.5	322.1	386.4	627.5	809	839.9	757	754	625	13
Os01g0571000	386	304	378	231.5	225.1	288.1	168	157	159	353.5	237.9	314.2	287.37	394.85	330.25	692.6	376.1	388.5	278.7	266.9	291.4	212.2	233.7	203.7	339.1	380.9	458.3	513	373	422	13
Os01g0884700	7.14	3.73	1.08	1.642	1.808	0.865	0	0	0	0.862	12.06	0.941	1.0885	3.7338	3.9598	0	6.183	1.036	1.858	4.031	3.449	0	0.713	1.444	5.282	4.714	4.386	1.85	3.83	9.16	3
Os01g0691100	0	0	0	1.642	0	0	0.78	0	0	0	0	1.882	0	0	1.5839	0	0	0	0	0	0	0	0	0	0	0	0	0	0	0	20
Os01g0116000	0	0	0	0	0	0	1.57	1.27	0.69	0.862	0	0	0	0.9335	0.792	0	0	1.036	0	0	0	3.697	1.425	0.722	0	0	0	0	0	0	17
Os01g0758400	0	1.86	0	0	0	0	0	0	0	0	0	0	0	0.9335	0	0	1.031	0	3.716	0	0	0	0	0	0	0	0	0	0	0	16
Os01g0210550	4.08	7.45	5.39	0.821	3.616	1.73	0.78	0	1.38	1.724	3.289	3.763	8.7082	1.8669	3.1679	0	1.031	3.108	4.645	1.613	0.862	2.958	1.425	1.444	2.113	5.657	6.579	1.85	5.74	0	4
Os01g0281100	1569	1409	1513	12.31	16.27	21.63	0	0.63	0.69	1618	1997	1677	1686.1	1771.7	1887.3	2734	1894	1798	16.72	36.28	28.45	1.479	4.988	1.444	2088	2100	1941	1823	1674	1976	3
Os01g0693700	3.06	3.73	5.39	3.284	1.808	1.73	4.7	5.06	2.76	5.173	2.193	0	8.7082	6.5342	7.9197	0	2.061	8.288	0.929	1.613	0.862	4.436	7.126	5.056	8.451	0	0	3.7	0.96	4.17	4
Os01g0371400	0	0.93	0	0.821	0.904	0	3.13	1.27	0.69	0	0	0.941	0	0	0	0	0	0	0	1.613	1.724	1.479	1.425	1.444	0	0	0	0	0	1.67	18
Os01g0708000	921	914	925	873.5	939.2	858.1	787	731	773	920	891.4	950.2	1003.6	967.99	981.25	1481	1065	1001	961.5	833.7	851.9	853.3	833.8	772.8	945.4	977.7	1044	953	931	939	13
Os01g0687150	0	0	0	0	1.808	0	5.48	1.27	3.46	0	1.096	0	0	0.9335	0	0	0	0	1.858	0.806	1.724	2.218	2.851	0.722	0	0	0	0	0	0	18
Os01g0111700	42.8	52.2	62.5	38.59	31.64	46.71	115	98	125	206.1	125	77.15	35.921	75.61	83.156	11.94	46.37	34.19	38.09	56.44	40.52	87.99	102.6	111.2	64.44	39.6	63.59	26.8	46.9	30.8	7
Os01g0586800	0	0	0	0	0	0	0	0	0	0	0	0	0	0	0	0	0	0	0	0	0	0	0.713	0	0	0	0	0	0	0	17
Os01g0757900	99.9	55	61.4	64.04	32.54	82.18	50.9	62	49.8	27.59	25.22	33.87	27.213	35.471	49.102	59.7	112.3	99.46	114.3	62.89	50.87	214.4	185.3	122.8	36.97	91.46	151.3	87	103	109	17
Os01g0641000	438	368	316	372.7	356.2	411.8	379	409	426	544	490.1	541	358.12	401.38	342.13	513.5	423.5	363.6	522.1	349.9	508.7	467.3	508.1	573.5	506	556.3	653.5	474	343	400	11
Os01g0500400	5.1	4.66	5.39	4.105	0.904	4.325	11	7.59	9.67	2.587	2.193	13.17	2.177	7.4676	2.3759	0	3.092	1.036	2.787	4.031	0.862	3.697	9.264	9.389	2.113	3.771	8.772	1.85	6.69	2.5	5
Os01g0579800	62.2	54	72.2	94.41	72.32	70.07	154	95.5	160	71.56	88.81	112	95.79	70.009	67.317	191.1	50.5	64.23	100.3	77.4	119.8	119.8	86.94	87.39	75	88.63	118.4	56.4	69.8	61.7	6
Os01g0759800	4.08	0.93	1.08	0.821	1.808	2.595	0	4.43	0.69	0.862	1.096	0	4.3541	0.9335	4.7518	0	0	9.324	0	0	0	7.394	2.851	3.611	5.282	0	2.193	0.93	1.91	6.66	1
Os01g0176200	70.3	83.8	84.1	59.93	50.62	54.5	50.1	67.7	82.9	64.66	43.86	48.92	47.895	58.807	79.197	71.65	50.5	46.62	44.59	58.05	39.66	56.19	86.94	69.34	46.48	32.06	59.21	58.3	27.7	37.5	14
Os01g0141100	765	681	794	770.9	710.5	752.6	1190	1097	1246	882.9	874.9	989.7	776.11	923.18	834.73	895.6	829.6	765.6	963.3	843.4	794.1	881.3	1033	945.4	812.3	815.6	918.8	738	719	730	18
Os01g0528800	4412	3894	4446	2577	2890	2444	3042	3447	3115	3237	2927	3288	4876.6	4643.9	4355.8	7129	4539	4138	3171	2853	2724	2111	2300	2175	3915	3080	3311	4030	3558	4411	13
Os01g0892500	398	346	503	32.02	25.31	66.61	84.6	74.6	111	174.2	117.3	184.4	94.701	146.55	108.5	465.7	220.5	258	42.73	24.99	21.56	241.8	171.7	232.6	118.3	212.1	225.9	169	140	100	14
Os01g0681700	31.6	29.8	31.2	47.62	75.93	95.16	765	787	716	37.07	33.99	41.4	34.833	45.739	53.854	47.76	44.31	52.84	33.44	42.73	37.08	638.8	578.7	555.4	32.75	33	52.63	31.5	32.5	34.2	18
Os01g0813500	546	555	547	481.9	497.2	501.7	478	467	485	487.1	591	499.6	587.8	575.01	587.64	1015	578.1	615.4	448.7	587	483.7	654.4	554.4	604.5	635.9	592.1	679.8	704	625	693	13
Os01g0382900	140	154	161	168.3	178.1	209.3	218	183	150	114.7	98.68	143.9	119.74	182.02	156.02	453.8	183.4	148.2	193.2	212.1	240.6	221.8	198.1	174.8	194.4	146.1	120.6	207	216	177	6
Os01g0329000	1.02	2.79	4.31	0	0	0.865	0	0	0	1.724	3.289	1.882	1.0885	4.6673	7.9197	23.88	2.061	5.18	1.858	0.806	0	0	0	1.444	2.113	2.829	0	3.7	5.74	3.33	13
Os01g0673600	784	757	861	678.1	791.9	791.5	449	571	554	530.2	437.5	479.8	493.1	538.6	531.41	573.2	698.7	651.7	591.7	453.9	564.8	584.9	678.4	598	553.5	550.6	559.2	645	557	655	8
Os01g0716800	389	205	337	344.8	279.3	378.9	274	387	306	148.3	152.4	251.2	243.83	257.63	197.2	262.7	303	248.6	680.9	621.6	617.3	198.2	163.9	192.1	142.6	219.7	197.4	190	216	214	16
Os01g0733500	775	448	323	209.3	113	147.1	37.6	15.2	33.2	106	106.4	258.7	128.45	87.744	91.076	322.4	324.6	350.2	58.52	118.5	59.49	121.3	215.2	216	469	260.2	221.5	513	305	408	19
Os01g0299400	164	139	119	151.9	144.6	162.6	176	185	190	153.5	166.7	135.5	173.07	175.49	126.71	250.8	138.1	151.3	197.9	187.9	206.1	159	147.5	130.7	115.1	133.9	136	145	141	127	6
Os01g0264100	0	0	0	0	0.904	0	0	0	1.38	0	0	0	0	0	0	0	0	0	0	2.419	0	0	0	0	0	0	0	0	0	0.83	16
Os01g0528300	1.02	4.66	1.08	1.642	1.808	0	0	0.63	0	7.76	2.193	0	2.177	2.8004	0	11.94	2.061	3.108	0.929	2.419	0	0.739	0.713	0.722	4.225	2.829	13.16	18.5	10.5	9.16	13
Os01g0760900	10.2	5.59	14	12.31	9.04	17.3	10.2	6.96	3.46	12.07	4.386	11.29	7.6196	13.068	5.5438	23.88	15.46	13.47	13.01	15.32	27.59	8.133	13.54	7.945	8.451	20.74	26.32	15.7	15.3	14.2	16
Os01g0672201	45.9	36.3	30.2	28.73	28.02	35.47	7.05	11.4	6.91	18.97	15.35	11.29	28.302	11.201	18.215	47.76	17.52	26.94	15.79	10.48	11.21	25.14	29.93	14.44	20.07	18.86	19.74	41.6	36.3	36.7	8
Os01g0191000	17.3	21.4	35.6	35.3	24.41	23.36	45.4	44.9	42.8	25.87	48.24	30.11	18.505	25.203	28.511	47.76	30.92	27.97	33.44	42.73	26.73	39.93	64.85	46.95	36.97	16.97	28.51	36.1	37.3	30.8	17
Os01g0593500	119	106	93.7	202.8	191.6	196.4	105	115	106	89.67	64.69	88.44	134.98	127.88	106.12	214.9	104.1	110.9	239.7	216.9	235.4	102	79.82	109.8	68.66	117.9	87.72	87.9	107	86.6	10
Os01g0262401	1.02	0	0	0	0	0.865	0	0.63	0	0	0	0.941	0	0	0.792	0	0	0	2.787	0.806	0	0	0	0	0	0	0	0.93	0	0.83	16
Os01g0589000	446	532	492	825.1	931.1	715.4	843	946	894	538	638.1	664.2	666.17	515.27	514.78	1158	465.8	476.6	734.8	795	828.6	652.1	691.3	762	540.9	464.8	416.7	370	420	431	12
Os01g0178100	373	564	489	277.5	311	210.2	357	373	320	675.1	780.6	543.8	745.64	549.8	746.82	1063	478.2	495.2	235	236.2	206.9	339.4	330.7	302.6	752.1	601.5	600.9	588	651	740	13
Os01g0762900	26.5	7.45	16.2	14.78	12.66	12.11	18	7.59	12.4	18.97	16.45	36.69	13.062	31.737	17.423	35.82	31.95	20.72	13.01	8.063	25.87	3.697	2.138	7.222	13.73	30.17	46.05	9.25	25.8	15	20
Os01g0687700	252	282	238	326.7	348.9	374.6	446	464	450	258.7	247.8	216.4	226.41	273.5	235.21	417.9	267.9	239.3	313.1	335.4	338	394.8	387	379.2	229.2	223.5	193	261	265	277	18
Os01g0241400	0	1.86	0	0	0	0	0	0	0	0.862	3.289	1.882	0	0	0	0	1.031	1.036	0	0	0	0	0.713	0.722	0	0	2.193	0.93	0.96	0	20
Os01g0295700	1572	1440	1116	544.3	480.9	647.1	743	755	657	562.1	531.8	636	746.72	701.96	725.44	1660	1604	1203	818.4	790.2	886.4	746.8	827.4	866.7	715.2	786.3	835.5	833	791	749	14
Os01g0742300	3.06	0.93	3.23	1.642	5.424	4.325	3.91	5.69	2.76	8.622	2.193	0	1.0885	5.6007	3.9598	0	2.061	5.18	2.787	7.257	5.173	4.436	6.414	8.667	3.169	0	0	2.78	0	0.83	9
Os01g0828900	0	0	0	0	0.904	0	1.57	1.27	2.07	0.862	0	0	0	0	0.792	0	0	0	0	0	0.862	0.739	0	4.333	0	0	2.193	0	0	0	9
Os01g0704250	2.04	2.79	4.31	3.284	0	3.46	4.7	6.33	2.76	4.311	2.193	1.882	1.0885	0.9335	3.1679	23.88	4.122	3.108	5.574	8.063	3.449	3.697	3.563	2.889	0	0.943	4.386	0.93	9.56	0	6
Os01g0816550	19.4	28.9	28	36.94	33.45	36.33	26.6	29.7	51.8	12.07	19.74	16.93	28.302	16.802	22.175	11.94	14.43	20.72	32.51	24.99	36.21	32.53	29.22	28.89	19.01	17.91	2.193	25	33.5	22.5	12
Os01g0637300	1.02	0.93	0	0	0.904	0	0	0.63	0.69	0	1.096	0	0	0	0	0	0	0	2.787	1.613	1.724	0.739	0	0.722	1.056	0	4.386	0	0	0	11
Os01g0795900	0	2.79	0	3.284	1.808	0.865	2.35	2.53	2.07	4.311	2.193	0	3.2656	5.6007	0.792	0	7.214	0	0.929	0.806	1.724	0	5.701	4.333	0	1.886	2.193	5.55	1.91	2.5	1
Os01g0578700	463	480	433	449.9	445.7	455	319	299	346	399.2	373.9	364.1	391.87	396.72	472.8	823.9	450.3	456.9	340.9	380.6	347.5	422.9	401.2	370.5	428.9	401.7	414.5	472	458	477	13
Os01g0203000	965	949	809	587	503.5	479.2	888	891	1061	705.3	904.5	496.8	523.58	448.99	543.29	1015	649.2	652.7	413.4	478.9	386.3	817.8	699.8	777.1	703.5	550.6	570.2	456	472	578	5
Os01g0102300	739	843	960	1166	1174	1020	4554	4173	4384	1274	1363	1138	1571.8	1031.5	1186.4	2293	853.3	919	1492	1478	1252	1840	1881	3034	1009	910.8	901.3	774	833	1005	18
Os01g0830200	467	442	500	408.8	395	378	426	446	357	426.8	429.8	453.5	475.68	473.26	481.52	680.6	473	457.9	329.8	413.6	340.6	504.3	392.7	429.7	501.8	484.6	504.4	490	557	559	13
Os01g0181166	4.08	3.73	6.47	21.35	23.5	13.84	25.1	28.5	30.4	7.76	12.06	10.35	9.7967	3.7338	7.1277	23.88	12.37	7.252	29.73	14.51	31.04	23.66	13.54	20.95	7.395	15.09	2.193	5.55	15.3	3.33	12
Os01g0914000	0	0	1.08	0.821	0	1.73	4.7	4.43	3.46	0.862	0	1.882	1.0885	0.9335	0	0	0	0	0	0	0	0.739	0.713	3.611	1.056	0	0	0	1.91	0	18
Os01g0153250	12.2	7.45	4.31	13.96	24.41	13.84	7.05	10.1	4.84	5.173	2.193	1.882	6.5311	7.4676	12.671	0	6.183	13.47	9.29	15.32	13.8	8.873	9.264	17.33	12.68	0.943	15.35	9.25	7.65	6.66	2
Os01g0192500	1.02	0	0	1.642	1.808	1.73	5.48	1.27	14.5	0	1.096	0.941	1.0885	0.9335	0	11.94	1.031	1.036	1.858	2.419	0.862	0.739	0.713	2.167	2.113	2.829	0	0	0	0	6
Os01g0242400	1.02	5.59	0	0	0	0	0	0	0	0	0	2.822	0	0.9335	0	0	0	0	0	0	0	0.739	0	1.444	2.113	0	0	0	0	1.67	14
Os01g0127700	701	483	681	440	530.6	415.2	222	315	193	256.1	173.2	100.7	289.55	252.97	275.6	334.3	361.7	444.5	361.4	445.9	333.7	283.9	391.9	249.9	181.7	125.4	149.1	234	245	190	14
Os01g0852400	0	0	0	0	0	0	0	1.27	1.38	2.587	0	3.763	1.0885	0	0.792	11.94	1.031	2.072	0	0	0.862	0.739	0	0.722	4.225	0	0	2.78	0	0.83	6
Os01g0924966	11.2	15.8	14	4.926	1.808	3.46	10.2	5.06	1.38	13.79	10.96	10.35	11.974	10.268	7.9197	0	10.31	11.4	3.716	0.806	6.036	20.7	19.95	60.67	15.85	16.03	6.579	17.6	6.69	10.8	9
Os01g0185400	434	466	463	498.3	466.4	445.5	431	464	513	381.1	447.3	417.7	507.25	359.38	481.52	728.4	465.8	386.4	396.7	371.7	395.8	489.5	496.7	479.6	429.9	455.4	456.1	465	398	467	6
Os01g0890950	0	0	0	0	0.904	0	0	0	0	0	0	0	0	0	0	0	0	0	0.929	0	0	1.479	0	0	0	0	2.193	0	0.96	1.67	11
Os01g0752400	437	434	537	371.9	429.4	383.2	481	454	533	557.8	577.8	526.9	530.11	605.81	540.12	537.3	423.5	416.5	407.8	463.6	373.3	420	453.2	462.2	586.3	488.4	528.5	514	422	466	3
Os01g0763100	62.2	48.4	78.7	160.9	182.6	155.7	151	157	160	64.66	47.14	72.44	71.842	65.342	57.022	59.7	59.77	52.84	151.4	176.6	189.7	104.3	115.4	123.5	72.89	61.28	43.86	59.2	65	60	12
Os01g0210800	87.7	52.2	50.6	57.47	56.05	51.04	23.5	31	40.8	57.77	43.86	53.63	22.859	48.539	36.43	59.7	76.26	58.02	26.94	62.89	59.49	80.59	66.99	57.06	54.93	58.46	37.28	76.8	66	78.3	8
Os01g0962200	290	363	343	485.2	426.7	384.9	682	660	614	409.5	345.4	377.3	335.26	364.05	367.47	477.6	345.2	292.2	402.2	375.7	432	568.6	488.2	503.4	339.1	369.6	335.5	369	378	411	18
Os01g0184700	21.4	19.6	25.9	30.38	46.1	35.47	31.3	37.3	51.8	29.31	38.37	35.75	32.656	28.004	23.759	59.7	26.79	27.97	29.73	42.73	36.21	32.53	42.76	26.72	20.07	28.29	41.67	31.5	25.8	15	6
Os01g0108000	376	489	494	472.9	537.9	470.6	373	492	455	405.2	462.7	367.9	432.14	396.72	451.42	776.2	451.4	403	338.1	408	372.5	504.3	502.4	496.2	470.1	423.3	423.2	465	438	467	6
Os01g0906000	46.9	24.2	34.5	15.6	18.98	19.9	59.5	66.4	71.2	26.73	16.45	27.28	21.77	22.403	35.638	23.88	46.37	32.12	13.01	24.99	32.76	28.84	40.62	26	40.14	31.11	21.93	25	18.2	23.3	5
Os01g0232000	29.6	75.4	37.7	15.6	14.46	16.44	75.9	161	118	62.08	88.81	127	75.108	57.874	37.222	0	36.07	40.41	13.01	7.257	14.66	49.54	43.47	77.28	47.54	58.46	61.4	20.4	19.1	36.7	5
Os01g0939600	46.9	47.5	50.6	83.74	79.55	57.96	42.3	26.6	30.4	47.42	38.37	76.21	10.885	22.403	13.463	0	17.52	23.83	47.38	62.08	55.18	34.01	19.24	36.83	12.68	14.14	19.74	14.8	11.5	9.16	10
Os01g0867300	273	179	278	170.8	223.3	202.4	729	636	782	297.5	209.4	253.1	333.09	259.5	313.62	394.1	225.7	218.6	138.4	174.2	141.4	850.3	796.7	697	236.6	162.2	239	253	212	187	18
Os01g0615000	50	61.5	59.3	122.3	110.3	104.7	212	161	179	59.49	74.55	97.85	108.85	74.676	95.036	179.1	74.2	80.81	145.8	140.3	164.7	143.4	100.5	193.6	76.06	63.17	65.79	48.1	53.6	66.6	12
Os01g0289750	0	0.93	0	0	0	0	0	0	0.69	0	0	0.941	0	0	0	0	0	0	0	0	0	0	0	0.722	0	0	0	0	0.96	0	20
Os01g0524400	11.2	13	16.2	32.02	32.54	28.55	11	12	18	16.38	15.35	11.29	20.682	32.671	19.007	71.65	6.183	17.61	26.94	20.96	27.59	9.612	7.839	7.222	9.507	16.03	6.579	7.4	8.61	11.7	6
Os01g0945150	2.04	0.93	1.08	0	2.712	0.865	0	0	2.76	0	2.193	0	0	0	3.1679	11.94	0	0	0	0	0.862	2.218	7.839	10.11	0	0	0	5.55	0.96	1.67	6
Os01g0877500	0	0.93	2.16	0	0	0	0	1.27	2.76	0	0	0	0	3.7338	0	0	0	0	0.929	1.613	0	0	3.563	1.444	1.056	0	0	0.93	0	0.83	17
Os01g0801901	136	96.9	84.1	68.14	60.57	74.39	43.1	64.5	46.3	77.6	110.7	118.5	67.488	126.95	93.452	83.59	90.69	147.1	83.61	37.09	27.59	55.45	50.6	57.78	84.51	191.4	197.4	95.3	149	137	3
Os01g0214300	408	452	419	354.7	428.5	379.8	514	538	570	502.7	512	477	502.9	441.52	523.49	561.2	406	382.3	469.1	453.9	457.8	493.2	517.4	500.5	482.8	502.5	429.8	478	503	464	5
Os01g0710200	10.2	6.52	6.47	12.31	9.944	5.19	4.7	3.16	3.46	7.76	12.06	16.93	13.062	16.802	11.879	23.88	14.43	10.36	5.574	2.419	0.862	5.176	7.126	1.444	6.338	12.26	17.54	19.4	3.83	15	13
Os01g0737800	595	591	659	607.5	514.4	641	815	723	824	600.1	725.8	667	657.47	797.17	734.94	1027	642	638.2	592.7	593.4	580.3	784.5	688.4	703.5	641.2	645.8	625	728	674	634	6
Os01g0597800	82.6	61.5	62.5	16.42	12.66	6.055	13.3	31.6	58.7	23.28	13.16	12.23	28.302	16.802	21.383	0	27.82	16.58	13.93	12.09	14.66	25.88	52.02	62.11	7.395	7.543	15.35	22.2	15.3	7.5	14
Os01g0840200	1239	1055	1084	837.4	759.3	648.8	804	843	758	1135	1101	1010	960.07	983.86	1167.4	2245	1396	1260	844.4	911.9	869.1	997.4	1216	1173	1269	1418	1340	1520	1753	1636	13
Os01g0168850	0	0	0	0.821	0	0	0	0	0	0	0	0	0	0	0	0	1.031	0	0	0	0	0	0	0	0	0	0	0	0	0	2
Os01g0191900	59.1	61.5	55	87.02	97.63	74.39	88.5	65.1	71.2	38.8	43.86	56.45	33.744	44.806	23.759	59.7	45.34	36.26	94.75	77.4	78.46	108	104	122.1	40.14	53.74	72.37	37	28.7	43.3	12
Os01g0172300	246	255	230	257.8	247.7	298.4	354	351	343	327.6	325.6	330.2	322.2	286.57	306.49	513.5	290.6	251.8	284.3	276.6	265.6	448.1	426.9	475.2	379.2	383.7	381.6	282	248	277	9
Os01g0124900	0	0	0	0	0	0.865	0	0	0	0	0	0	0	0	0	0	0	1.036	0	0	0	0	0	0	0	0	2.193	0	0	0	11
Os01g0752800	4630	4280	4482	3721	3912	4148	4286	3648	3994	4331	4345	5507	3890.4	5206.8	4176.8	7176	5502	5023	5693	4393	5209	3105	3153	4020	4959	5646	6539	4271	4410	4106	13
Os01g0268800	711	609	733	526.2	467.4	505.2	508	463	470	779.4	720.3	699	818.57	708.49	852.16	1051	725.5	591.6	598.2	603.9	651	512.4	506.7	478.8	739.5	760.9	754.4	670	686	642	13

Os01g0226400	1.02	0	0	0	0	0.865	0.78	0	2.07	2.587	1.096	3.763	0	0.9335	4.7518	0	0	1.036	0.929	2.419	3.449	0	2.138	1.444	1.056	0	2.193	0	1.91	3.33	20
Os01g0174100	222	268	171	179.8	169.9	188.6	109	175	119	190.5	257.7	239	287.37	226.83	285.9	334.3	193.7	231	131.9	107.2	100	122	103.3	118.4	282	188.6	241.2	228	165	288	13
Os01g0916950	0	0	1.08	0.821	0	0	0	0	0	0	0	0	1.0885	0	0	0	0	0	0.929	0	0.862	0.739	0	0	0	0	0	0	0.96	1.67	4
Os01g0579600	12.2	16.8	5.39	22.17	39.77	39.79	20.4	22.1	18.7	9.484	20.83	14.11	4.3541	15.869	14.255	23.88	19.58	41.44	22.29	22.58	16.38	30.31	38.48	26	26.41	19.8	35.09	25	15.3	23.3	2
Os01g0125800	87.7	73.6	83	48.44	57.85	59.69	79.9	108	80.2	81.05	84.42	118.5	70.754	84.944	83.948	238.8	86.56	72.52	92.9	80.63	73.29	52.5	62	67.17	57.04	67.88	70.17	58.3	70.8	63.3	6
Os01g0686700	313	366	336	22.99	27.12	10.38	10.2	20.9	1.38	354.4	372.8	296.4	260.16	378.05	426.08	859.7	417.4	384.4	24.15	49.99	28.45	10.35	8.552	13.72	431	416.7	425.4	542	473	527	13
Os01g0885600	298	241	274	64.86	40.68	52.77	43.1	43	85	281.1	291.6	316.1	240.56	282.84	290.65	453.8	254.5	289.1	64.1	75.79	65.53	66.54	69.84	52	249.3	330	375	284	295	251	3
Os01g0848400	0	4.66	3.23	0	0.904	2.595	1.57	0	0.69	0	2.193	0.941	1.0885	0	1.5839	0	3.092	3.108	0	2.419	0	2.958	4.276	6.5	1.056	0	0	0.93	5.74	5	9
Os01g0549200	567	560	712	689.6	655.4	599.5	487	583	541	610.4	575.6	519.3	1065.7	741.16	792.76	895.6	628.6	656.8	537.9	546.7	612.2	542.7	551.6	486.8	620.1	566.6	594.3	742	702	733	4
Os01g0680950	1.02	0	0	0	2.712	2.595	3.13	0	4.15	0.862	0	0.941	0	0.9335	0	0	0	0	1.858	0.806	4.311	1.479	0.713	0	1.056	0	0	0	0	0	15
Os01g0243800	24.5	15.8	21.6	9.852	20.79	16.44	6.26	25.9	6.22	7.76	10.96	9.408	18.505	14.935	19.799	35.82	12.37	6.216	8.361	11.29	9.484	17.01	29.22	9.389	16.9	8.486	2.193	18.5	23.9	11.7	6
Os01g0166100	412	362	380	309.5	265.8	324.4	344	392	261	459.5	419.9	534.4	359.21	403.25	456.96	764.2	581.2	484.9	344.6	315.3	362.1	317.2	338.5	377	550.4	660	578.9	538	460	523	13
Os01g0877400	4.08	2.79	0	0	0.904	0	2.35	0.63	1.38	0.862	2.193	1.882	1.0885	0.9335	0	0	0	1.036	6.503	1.613	1.724	0.739	0	1.444	2.113	4.714	0	0.93	0.96	2.5	19
Os01g0572300	28.5	39.1	22.6	50.08	51.53	29.41	164	140	139	56.9	59.21	61.15	55.515	34.538	53.062	71.65	39.16	35.23	54.81	49.18	56.91	104.3	96.21	109.8	55.99	32.06	32.89	36.1	42.1	52.5	18
Os01g0261200	2894	2565	2543	1159	1247	1046	1208	1662	1156	1124	959.3	916.4	1242	1221	1229.1	2006	1882	1872	850	949	781.2	1133	1552	1047	1066	1156	1039	1214	976	1186	14
Os01g0734800	887	913	1125	846.4	1012	664.4	708	944	509	1300	851.9	918.2	947.01	799.03	1088.2	1469	878	863	934.5	1420	826	482.8	798.2	439.8	859.9	701.5	594.3	809	830	771	13
Os01g0956200	20.4	23.3	19.4	22.99	20.79	11.25	47	31.6	28.3	17.24	20.83	24.46	18.505	29.87	22.967	0	45.34	31.08	27.87	16.13	22.42	12.57	10.69	18.06	10.56	26.4	37.28	17.6	8.61	15	15
Os01g0932600	803	893	789	806.2	757.5	722.3	837	870	749	818.2	924.3	738.5	709.71	797.17	904.43	1576	843	811.2	666.1	613.6	707	802.2	884.4	886.2	886.3	844.8	883.7	921	905	831	6
Os01g0183800	611	727	578	663.3	631	622.8	1140	987	1087	882	806.9	852.4	791.35	725.29	917.1	1194	640	691	633.5	711.9	680.3	1071	987.7	1065	867.3	771.2	719.3	855	707	826	5
Os01g0315800	325	310	268	427.7	368.8	497.4	958	725	950	487.1	457.2	439.4	447.38	478.86	399.94	835.9	416.3	361.6	703.2	633.7	613	480.6	648.5	611.7	441.6	387.5	486.8	410	356	289	18
Os01g0807800	98.9	103	93.7	128.1	128.4	105.5	127	159	102	105.2	95.39	105.4	96.878	119.48	111.67	143.3	91.72	114	106.8	126.6	113.8	154.5	168.2	111.2	137.3	103.7	100.9	116	125	101	17
Os01g0965201	40.8	71.7	47.4	147	179.9	140.1	93.2	134	119	59.49	94.29	66.8	74.019	72.809	93.452	71.65	60.8	41.44	76.17	112.9	97.43	110.9	103.3	93.89	68.66	56.57	50.44	44.4	54.5	58.3	12
Os01g0911100	7647	6367	7408	4561	4385	5254	3939	4213	4260	5169	5112	4852	5875.8	5362.7	5845.5	8562	6650	7423	3971	4403	4259	5845	4931	4505	5983	5629	5169	6716	6184	6798	13
Os01g0226300	69.3	39.1	60.3	55.83	49.72	101.2	63.4	46.8	39.4	66.39	29.6	109.1	40.275	63.475	61.773	59.7	73.17	51.8	101.3	81.43	93.98	48.06	54.87	44.78	70.78	80.14	125	82.3	60.2	56.7	11
Os01g0929100	2231	3316	2698	6844	7313	6490	####	####	####	3163	3909	4682	2841	2497.9	2123.3	3117	2550	2651	8373	7381	8790	10634	11166	14654	3356	3233	3151	1691	2087	2215	18
Os01g0708700	0	0	0	0.821	0	1.73	0.78	0	0	0	0	0	0	0	0	0	0	0	0	0	0	0	0	0	0	0	0	0	0	0	2
Os01g0958800	406	403	417	359.6	398.7	350.3	507	562	623	400.1	463.8	404.6	409.28	392.05	422.91	597	409.1	418.6	339.1	343.5	386.3	483.6	417.6	464.4	366.6	396	368.4	323	449	379	18
Os01g0601200	1.02	1.86	1.08	11.49	8.136	12.98	7.05	6.96	4.15	3.449	1.096	0.941	0	0	0.792	0	0	3.108	10.22	5.644	8.622	5.915	4.276	8.667	0	0.943	0	0.93	0.96	1.67	12
Os01g0272700	1.02	0.93	0	0	0	0	0	0	0	0	0	1.882	0	0	0	0	0	0	0	0	0	0	0	0	0	0	0	0	0	0	20
Os01g0508100	13.3	6.52	14	3.284	2.712	0.865	5.48	13.9	15.2	6.035	4.386	7.527	3.2656	10.268	6.3357	23.88	7.214	9.324	4.645	2.419	3.449	0	0.713	2.889	3.169	10.37	2.193	5.55	2.87	4.17	6
Os01g0215700	2.04	0	0	0	0	0	0	0	0	0	0	0	0	0	0	0	0	0	0	0	0	0	0	0	0	0	0	0	0	0	19
Os01g0706800	26.5	13	7.54	1.642	0.904	1.73	0	2.53	2.07	10.35	10.96	22.58	3.2656	3.7338	7.1277	0	11.34	8.288	0.929	0	0	0	0.713	0	1.056	7.543	4.386	4.63	0.96	1.67	19
Os01g0648600	2.04	1.86	12.9	0	0	0	7.05	1.27	17.3	12.07	12.06	15.99	6.5311	7.4676	3.1679	0	2.061	3.108	1.858	0	0.862	13.31	17.1	7.945	14.79	6.6	21.93	5.55	5.74	9.16	5
Os01g0962600	72.4	35.4	42	41.05	59.66	88.23	60.3	43.6	66.3	66.39	48.24	79.03	31.567	44.806	26.927	95.53	41.22	68.38	85.46	91.11	93.12	36.97	50.6	39.72	31.69	54.68	70.17	22.2	44	21.7	16
Os01g0162300	0	0	0	1.642	7.232	3.46	3.91	3.8	0	0.862	0	0.941	1.0885	1.8669	0.792	0	2.061	0	6.503	3.225	7.76	0	1.425	1.444	2.113	2.829	2.193	0.93	0	1.67	10
Os01g0764800	87.7	117	102	32.84	18.98	14.71	11.7	13.3	13.1	50.87	115.1	27.28	138.24	97.079	151.27	179.1	56.68	69.41	26.01	17.74	12.93	59.15	20.67	20.95	72.89	16.97	24.12	101	148	84.1	13
Os01g0814900	831	710	822	688	744.9	838.2	650	645	691	689.7	559.2	708.4	635.7	785.03	639.12	967.2	1053	862	927.1	842.6	889.8	672.1	704.1	845	677.1	712.8	850.9	829	699	741	16
Os01g0279000	13.3	10.2	6.47	4.926	9.04	13.84	0	0.63	2.07	20.69	31.8	16.93	16.328	16.802	21.383	23.88	15.46	20.72	2.787	10.48	6.036	1.479	0.713	3.611	12.68	22.63	28.51	13.9	22	15	3
Os01g0623900	10.2	2.79	4.31	3.284	0.904	1.73	3.13	7.59	2.07	12.93	3.289	3.763	5.4426	4.6673	7.1277	23.88	2.061	0	0.929	1.613	1.724	0	2.851	1.444	5.282	2.829	4.386	0.93	1.91	0	6
Os01g0742350	2.04	3.73	3.23	1.642	1.808	1.73	3.91	6.33	2.76	2.587	0	4.704	0	0.9335	5.5438	0	4.122	1.036	0	1.613	1.724	2.218	2.851	0	1.056	1.886	4.386	1.85	3.83	0	5
Os01g0651400	4.08	1.86	7.54	8.21	1.808	4.325	11	10.8	11.1	11.21	4.386	4.704	10.885	15.869	11.879	47.76	10.31	15.54	7.432	6.45	5.173	5.176	5.701	10.11	4.225	11.31	8.772	17.6	23	12.5	6
Os01g0867800	1049	1367	1230	1690	1934	1592	1667	1859	1602	1125	1262	1098	1295.3	1300.3	1302	1743	1244	1241	1551	1789	1779	1722	1616	1464	1292	1117	1077	1367	1347	1439	12
Os01g0884400	32.6	40.1	75.4	43.51	45.2	38.93	145	117	140	51.73	32.89	24.46	23.947	46.673	25.343	59.7	32.98	42.48	26.94	34.67	28.45	148.6	170.3	146.6	24.3	13.2	21.93	32.4	32.5	26.7	18
Os01g0805200	6792	6582	7761	10955	11679	11028	####	####	####	8971	9855	9046	13112	10888	10190	14150	8457	7378	13326	13731	13390	8228	8040	9158	9527	8467	7570	7225	6945	8478	12
Os01g0120300	1466	1895	1869	2770	3052	2549	2137	2822	2401	1707	2052	1845	2428.5	1824	1900.7	2448	1415	1716	2071	2452	2011	2226	2060	2259	1997	1487	1410	1447	1681	1847	12
Os01g0720000	0	0.93	0	0	0	0	0.78	0	0	0	0	0	0	0	0.792	0	0	0	0	0	0	0	0	0	0	0	0	0	0.96	1.67	1
Os01g0927400	88.7	81	78.7	97.7	76.84	85.64	71.2	78.4	65.6	70.7	32.89	47.04	51.16	68.142	79.989	59.7	54.62	69.41	87.32	83.85	70.7	102.8	89.08	72.22	66.55	51.86	78.95	73.1	61.2	51.7	2
Os01g0736900	5116	4127	5213	2895	2655	3465	1839	1584	1681	4313	3897	3908	3665	4212.7	4009.7	6663	4110	3879	3773	4074	4011	1765	1972	2019	4089	4005	4542	4381	4241	3573	13
Os01g0153150	22.4	27	30.2	58.29	68.7	63.15	28.2	44.3	35.9	19.83	27.41	25.4	28.302	29.87	29.303	47.76	21.64	26.94	64.1	74.18	81.05	25.14	45.61	29.61	11.62	21.69	24.12	22.2	33.5	25.8	10
Os01g0106200	517	405	448	624.8	491.8	618.5	1039	826	818	653.5	603	537.2	572.56	589.94	563.88	859.7	421.5	451.7	732.9	746.6	766.5	508	575.1	624	565.2	512.9	631.6	426	531	419	15
Os01g0530500	28.5	21.4	21.6	151.1	176.3	126.3	139	137	143	18.97	17.54	20.7	19.593	26.137	25.343	0	22.67	19.68	119.8	122.6	113	108	93.36	105.4	15.85	19.8	10.96	26.8	38.3	14.2	12
Os01g0137700	0	0	3.23	1.642	1.808	0	0	1.27	0	0	0	0	0	2.8004	0	0	0	0	0	0	0	5.176	5.701	1.444	0	0	2.193	0.93	1.91	0	17
Os01g0910400	4.08	4.66	4.31	0	3.616	1.73	3.13	1.9	0	0.862	0	6.586	1.0885	0	3.1679	0	8.244	0	0.929	2.419	4.311	3.697	7.126	4.333	7.395	8.486	10.96	1.85	1.91	4.17	11
Os01g0188400	1814	1637	1423	146.1	171.8	185.1	329	254	197	2003	1879	2767	1334.5	1574.7	1627.5	1827	1664	1531	231.3	271.7	346.6	214.4	221.6	250.6	1708	2070	2403	1615	1306	1407	3
Os01g0124650	0	0	0	0	0	1.73	0.78	0	0.69	0	0	3.763	2.177	0	0	0	0	0	0	0	0	0	0	2.889	1.056	0	0	0	0	0	20
Os01g0617600	63.2	66.1	65.7	199.5	193.4	175.6	72.8	59.5	82.9	65.53	69.07	41.4	67.488	68.142	45.934	95.53	43.28	62.16	182.1	209.6	170.7	67.28	86.94	54.17	61.27	39.6	63.59	42.6	54.5	38.3	10
Os01g0367300	452	442	541	784	728.6	785.5	623	559	616	473.3	653.5	573.9	668.35	489.13	631.99	585.1	659.5	433.1	809.1	753.1	1061	369.7	544.5	498.3	444.7	622.3	609.6	493	641	617	16
Os01g0193250	0	0	0	0	0.904	0	0	0	0	0	0	0	0	0	0	0	0	0	0	0	0	0	0	0	0	0	0	0	0.96	0	2
Os01g0880900	0	0	2.16	33.66	40.68	32.01	58.7	19	51.8	2.587	3.289	0	1.0885	0.9335	0.792	0	0	0	11.15	25.8	32.76	19.96	18.53	21.67	5.282	1.886	0	4.63	0	0.83	12
Os01g0134500	451	420	516	766.8	894.9	661.8	740	899	665	342.3	396.9	420.5	433.23	456.46	376.98	656.8	479.2	507.7	762.7	699	757	291.3	305.7	518.6	347.5	363	287.3	379	439	426	10
Os01g0816700	0	0	1.08	0	0	0	0	0	0	0	0	0	0	0	0	0	0	0	0	0	0	0	0	0	0	0	0	0	0	0	14
Os01g0829800	72.4	48.4	57.1	48.44	60.57	69.2	63.4	60.1	67	61.22	37.28	68.68	35.921	83.077	47.518	59.7	50.5	45.59	58.52	27.41	63.8	56.19	52.73	65.72	34.86	41.49	67.98	60.1	56.4	43.3	19
Os01g0737500	2.04	1.86	1.08	0.821	4.52	0	1.57	0.63	0	3.449	5.482	1.882	0	0.9335	3.9598	0	3.092	3.108	5.574	0.806	0.862	0.739	10.69	6.5	5.282	6.6	0	1.85	6.69	17.5	1
Os01g0182300	4.08	2.79	7.54	1.642	3.616	1.73	6.26	3.8	7.6	12.07	16.45	17.88	5.4426	3.7338	9.5036	0	4.122	1.036	8.361	3.225	3.449	3.697	2.851	10.83	15.85	10.37	17.54	6.48	6.69	7.5	20
Os01g0343400	1.02	5.59	0	1.642	8.136	12.98	11.7	9.49	4.84	1.724	1.096	2.822	0	0	0.792	0	1.031	0	8.361	3.225	2.587	1.479	9.264	4.333	0	0.943	0	1.85	0	0	12
Os01g0749400	1091	1384	1349	825.9	800.9	917.8	613	769	627	898.4	984.6	808.2	2043.2	1198.6	1633	1946	784.2	1135	606.6	610.4	632	720.9	736.9	694.8	1015	673.2	620.6	759	661	977	13
Os01g0828300	1.02	6.52	19.4	9.031	7.232	5.19	13.3	21.5	21.4	7.76	12.06	7.527	11.974	13.068	15.047	0	11.34	9.324	15.79	11.29	13.8	19.22	13.54	12.28	8.451	12.26	8.772	8.33	14.3	5	18
Os01g0628700	409	582	469	1284	1586	1074	165	154	153	459.5	495.6	261.5	548.61	399.52	530.62	632.9	184.5	296.3	861.1	948.2	849.3	252.9	278.6	206.6	326.4	231.9	265.3	291	320	463	10
Os01g0390900	1086	1074	1169	549.2	573.1	549.3	829	984	945	1085	1181	1041	1307.3	1405.8	1383.6	2973	1251	1169	609.4	570	588.9	722.4	694.8	620.4	1162	1027	1057	1165	1201	1423	13
Os01g0238900	704	748	733	631.3	722.3	572.7	783	843	769	728.5	674.3	803.5	789.18	747.69	709.6	1648	688.4	691	725.5	638.6	630.3	653.6	600.8	638.5	702.5	770.3	736.8	609	668	631	6
Os01g0200200	0	0	0	0	0	0	0.78	0	9.67	3.449	1.096	0.941	0	0.9335	0	0	0	3.108	0	0	0	34.01	21.38	25.28	0	0	0	0	0	0.83	9
Os01g0264700	882	849	836	651	661.7	723.2	752	644	744	1165	929.7	1179	777.2	912.92	767.42	1170	885.2	853.7	745	553.9	651	594.5	685.6	660.9	826.1	904.2	991.2	802	786	726	3
Os01g0870100	383	473	413	769.3	781.9	681.7	604	660	695	437.1	502.1	350	438.67	323.91	456.17	991.1	366.9	451.7	560.2	679.7	600.1	869.5	853.7	733.1	503.9	400.7	458.3	436	452	459	12
Os01g0290100	936	1022	1060	865.3	991.7	811.4	1105	1106	1100	1104	1064	1125	1183.2	1059.5	1098.5	1624	1034	1095	944.7	941.7	876	740.1	819.5	866.7	947.6	1007	826.7	982	931	911	6
Os01g0644900	0	0	0	0	0	0	0	0	0	0.862	0	0	1.0885	0.9335	0	0	0	0	0	0	0	0	0	0	0	0	0	0.93	0	0	4
Os01g0247700	0	4.66	0	0	0	0	0	0	0	0	0	1.882	0	0	0	11.94	0	0	0	0	0	0	0	0	0	0	0	0	1.91	0.83	6
Os01g0589100	90.7	78.2	90.5	93.59	103.1	96.89	159	138	123	103.5	104.2	148.6	102.32	117.61	79.197	119.4	81.41	96.35	121.7	104.8	112.1	82.81	88.37	93.17	90.85	112.2	109.6	73.1	78.4	74.1	15
Os01g0827200	449	371	472	163.4	184.4	123.7	155	165	176	490.6	495.6	414.9	456.09	436.86	495.77	537.3	462.7	440.3	211.8	232.2	189.7	175.2	204.5	163.2	491.2	470.5	480.2	470	464	468	3
Os01g0818200	3.06	10.2	12.9	40.23	57.85	47.58	32.1	29.7	10.4	1.724	4.386	3.763	1.0885	1.8669	1.5839	11.94	3.092	1.036	39.94	34.67	28.45	14.05	24.23	28.17	3.169	6.6	0	1.85	0.96	2.5	10
Os01g0678800	6.12	13	7.54	5.747	9.04	6.055	2.35	12	1.38	12.07	1.096	1.882	0	1.8669	15.047	0	1.031	4.144	8.361	6.45	6.898	4.436	11.4	5.778	19.01	0	6.579	2.78	5.74	4.17	7
Os01g0916700	166	171	183	248.8	228.7	255.2	279	255	249	265.6	199.5	254	278.66	205.36	254.22	382.1	270	217.6	374.4	303.2	350.1	204.1	199.5	220.3	236.6	236.7	258.8	241	206	231	16
Os01g0166700	565	740	665	1114	1373	1161	1123	1361	1282	600.9	687.4	537.2	540.99	532.07	525.07	835.9	576.1	559.5	1074	1123	993.3	1095	1085	1247	625.4	535.5	616.2	507	535	568	12
Os01g0909200	520	673	672	665.8	733.1	638.4	368	455	406	494.9	480.2	373.5	512.69	488.2	571.8	788.1	519.4	576	466.3	474.1	431.1	515.4	566.5	442.7	442.6	422.4	471.5	557	579	575	8
Os01g0965700	0	0.93	0	0	0	0	0	0	0	0	0	0	0	0	0	0	0	0	0	0	0.862	0	0	0	0	0	0	0	0	0	14
Os01g0348600	1540	1715	1625	1914	1991	1670	1617	1757	1408	1642	1585	1570	1381.3	1402	1476.2	2078	1395	1427	1684	1486	1352	1272	1625	1530	1576	1509	1480	1590	1480	1492	6
Os01g0964000	317	256	308	221.7	198.9	177.3	184	213	158	254.3	274.1	283.2	272.13	332.31	312.83	370.2	330.8	248.6	264.8	267.7	214.7	142.7	173.2	140.8	311.6	277.2	298.2	289	303	318	3
Os01g0200500	257	221	220	373.5	415.8	365.9	318	268	337	233.7	241.2	278.5	209	359.38	246.3	358.2	229.8	221.7	448.7	396.7	458.7	244.7	287.2	262.9	221.8	265.9	271.9	239	247	217	10
Os01g0959100	15.3	20.5	6.47	22.17	5.424	48.44	0.78	8.86	4.84	5.173	1.096	4.704	27.213	9.3345	4.7518	23.88	159.7	19.68	6.503	2.419	0.862	6.654	14.97	9.389	7.395	3.771	4.386	0.93	6.69	2.5	14
Os01g0138500	208	192	219	72.25	61.47	134.9	30.5	13.3	29	225	191.9	175	173.07	208.16	187.7	1600	550.3	359.5	160.7	222.5	288	25.14	21.38	23.83	385.6	202.7	153.5	347	258	233	13
Os01g0766400	6.12	3.73	5.39	13.96	9.944	6.92	7.05	10.8	7.6	3.449	4.386	5.645	5.4426	1.8669	0.792	11.94	4.122	9.324	13.01	9.675	9.484	16.27	16.39	5.056	9.507	1.886	2.193	7.4	7.65	1.67	17
Os01g0574400	137	179	158	211	244.1	202.4	171	190	167	199.2	172.1	164.6	182.87	164.29	201.16	226.9	120.6	154.4	230.4	218.5	187.1	180.4	196.7	171.2	138.4	137.7	228.1	168	154	170	10
Os01g0202800	19.4	28.9	35.6	2.463	1.808	8.65	0.78	3.8	6.22	31.04	24.12	11.29	19.593	15.869	22.175	0	20.61	26.94	5.574	7.257	8.622	11.09	28.51	7.945	21.13	17.91	21.93	35.2	24.9	38.3	1
Os01g0835600	487	625	536	527.1	598.4	500	965	987	986	554.4	527.4	485.5	557.32	489.13	581.3	823.9	469.9	529.4	406.9	424.9	432	802.2	801	820.5	569.4	584.6	473.7	463	471	543	18
Os01g0964900	89.7	97.8	62.5	107.5	105.8	111.6	156	154	193	124.2	108.5	92.2	104.5	85.878	83.156	179.1	97.9	66.31	126.3	112.9	87.95	100.6	89.08	126.4	77.11	87.68	74.56	86	82.2	71.6	18
Os01g0265000	11.2	13	12.9	11.49	17.18	17.3	36	19	30.4	19.83	17.54	12.23	9.7967	17.736	14.255	11.94	11.34	13.47	19.51	12.9	12.93	29.58	24.94	18.06	14.79	14.14	10.96	17.6	20.1	15	18
Os01g0602800	463	416	358	220	162.7	256.9	176	128	132	324.2	305.9	389.5	249.27	345.38	319.16	931.4	581.2	450.7	216.4	264.5	261.3	386	412.6	382.1	535.6	565.7	570.2	634	443	538	13
Os01g0170700	42.8	72.7	45.3	61.57	77.74	60.55	42.3	46.8	58.7	60.35	50.43	79.97	56.603	66.275	64.149	95.53	69.05	62.16	80.82	79.02	71.56	48.06	55.59	40.45	64.44	43.37	43.86	68.5	85.1	66.6	16
Os01g0598900	594	754	622	866.9	894	813.1	1394	1608	1635	701.8	838.7	595.5	649.85	596.48	633.57	991.1	633.8	627.8	854.6	937.7	860.5	1768	1420	1547	668.7	641.1	592.1	615	683	640	18
Os01g0311400	43.8	72.7	67.9	33.66	39.77	37.2	55.6	50	48.4	68.97	70.17	61.15	66.4	50.406	54.646	83.59	53.59	45.59	22.29	38.7	25.87	33.27	29.22	21.67	46.48	62.23	46.05	64.8	44.9	38.3	13
Os01g0737300	122	113	120	119	187.1	154.8	72.8	84.1	71.2	49.14	75.65	59.27	120.83	101.75	110.88	131.4	83.47	93.24	136.6	124.2	155.2	68.76	66.99	75.11	58.1	82.03	59.21	97.1	101	95	10
Os01g0747500	39.8	38.2	34.5	210.2	191.6	152.2	172	180	172	41.38	36.18	21.64	23.947	29.87	27.719	23.88	34.01	19.68	186.7	194.3	169.9	150.1	151.8	182	25.35	14.14	24.12	24.1	36.3	36.7	12
Os01g0926300	2.04	0.93	1.08	5.747	4.52	6.92	6.26	5.69	4.84	3.449	5.482	2.822	4.3541	3.7338	0	0	2.061	2.072	6.503	4.838	6.898	1.479	2.138	2.167	2.113	1.886	4.386	1.85	6.69	1.67	15
Os01g0945600	0	0.93	0	0	0.904	0	0	0	0	0	0	0	0	0	0	0	0	0	0	0	0	0.739	0	0	0	0	0	0	0.96	0	8
Os01g0718700	67.3	42.8	86.2	9.031	5.424	2.595	4.7	3.8	0.69	20.69	9.868	19.76	41.364	56.007	69.693	143.3	110.3	58.02	4.645	13.71	4.311	1.479	4.988	2.167	53.87	45.26	28.51	68.5	44.9	59.2	13
Os01g0104350	133	143	195	183.1	131.1	147.9	255	140	211	194.9	200.6	255	182.87	177.36	143.35	214.9	224.7	231	263.8	262.8	326.8	122.7	106.2	166.8	180.6	182.9	206.1	134	162	180	16
Os01g0254950	5.1	2.79	3.23	4.926	1.808	4.325	0	2.53	0.69	3.449	3.289	0	4.3541	1.8669	1.5839	0	6.183	0	0.929	2.419	1.724	2.958	4.276	0.722	1.056	0.943	8.772	0.93	3.83	3.33	11
Os01g0899200	188	171	110	335.8	274.8	391	947	606	509	234.5	174.3	432.8	140.42	212.83	140.97	226.9	239.1	167.8	565.7	424.1	588.9	527.2	411.2	793.7	187	320.6	482.4	240	212	177	18
Os01g0954900	0	0	0	0	0	0	0	0	0	0	0	0	0	0	0	0	0	0	0	0	0	0	0	0	0	0.943	0	0	0	0	1
Os01g0888900	4.08	1.86	4.31	4.105	1.808	2.595	10.2	6.96	29	3.449	4.386	1.882	6.5311	3.7338	5.5438	0	1.031	4.144	0	4.838	1.724	27.36	19.24	24.56	2.113	4.714	0	3.7	0.96	5	9
Os01g0372500	0	0	0	0	0	0	0	0	0	0	0	0	0	0	0	11.94	0	0	0	0.806	0	0	1.425	0	0	0	0	0	0	0	6
Os01g0311600	0	0	0	0	0.904	0	0	0	0	0	0	0	0	0	0	0	0	0	0	0	0	0	0	0	0	0	0	0	0	0	2
Os01g0974000	1073	1080	1125	863.7	851.5	779.4	944	1066	1083	1280	1230	1341	1183.2	1023.1	1175.3	1815	1072	1042	1129	989.3	913.1	825.9	888.7	946.1	1243	1262	1300	957	1106	1078	13
Os01g0173900	1142	1089	1192	845.6	790.1	812.3	1197	1098	1634	1267	1171	1513	1526.1	1267.6	1528.5	1672	1139	1123	1032	986.1	1153	1291	1467	1344	1506	1403	1289	1368	1201	1246	5
Os01g0721700	18.4	23.3	22.6	33.66	28.02	22.49	18.8	13.9	24.9	27.59	25.22	16.93	25.036	18.669	19.799	0	15.46	18.65	26.01	27.41	19.83	13.31	22.8	30.33	17.96	19.8	19.74	25.9	20.1	32.5	2
Os01g0936700	0	0.93	0	0	0	0	0	0	0	0	2.193	0	0	0	0.792	0	1.031	0	0	0	0	0.739	0	0	1.056	0.943	0	0	0	0	7
Os01g0940700	453	657	623	513.9	565	474	835	335	1721	1822	876	935.2	347.24	473.26	1142	967.2	210.2	317	301	1458	327.6	566.4	374.1	738.1	341.2	848.6	883.7	119	474	181	7
Os01g0378100	0	0.93	2.16	3.284	0.904	14.71	1.57	1.9	0	0.862	1.096	7.527	3.2656	0.9335	0.792	0	0	0	12.08	4.838	19.83	2.218	8.552	6.5	3.169	13.2	15.35	2.78	8.61	2.5	16

Os01g0392600	8.16	8.38	5.39	23.81	21.7	27.68	23.5	32.9	31.8	9.484	12.06	8.467	10.885	3.7338	2.3759	23.88	9.275	4.144	14.86	12.9	15.52	50.28	39.91	23.83	7.395	4.714	6.579	4.63	14.3	10	17
Os01g0560200	412	401	367	400.6	417.6	350.3	566	550	544	405.2	406.8	472.3	387.51	403.25	360.34	668.7	367.9	328.4	461.7	354	392.3	442.2	441.1	486.8	341.2	435.6	473.7	361	356	397	6
Os01g0941800	0	0	0	0	0.904	1.73	0	0	1.38	1.724	0	0	1.0885	0	0	0	0	0	0	0	0	0	0.713	0.722	0	0	2.193	1.85	0	0	11
Os01g0653800	678	799	663	963	945.6	923	667	689	643	549.2	473.6	454.4	510.52	542.34	624.07	823.9	588.4	656.8	634.5	604.7	571.6	713.5	778.9	659.4	565.2	482.7	418.8	679	638	689	2
Os01g0793200	217	212	206	574.7	555.9	524.2	397	341	334	256.9	218.2	215.4	176.34	238.03	224.92	406	182.4	188.6	507.2	451.5	486.3	261.7	317.1	284.6	142.6	196.1	212.7	194	192	197	10
Os01g0952800	1.02	1.86	4.31	10.67	2.712	6.92	3.13	4.43	0	0.862	1.096	0	0	2.8004	0	0	0	0	0	0	0	0	0	0	0	0	2.193	0	0	0	2
Os01g0958900	42.8	61.5	56	115.8	90.4	101.2	124	74.6	122	67.25	50.43	47.04	37.01	68.142	45.934	83.59	57.71	37.3	109.6	114.5	91.4	75.42	75.54	65.72	33.8	37.71	59.21	88.8	66	44.2	12
Os01g0624700	21.4	37.3	18.3	4.105	5.424	4.325	3.91	6.96	5.53	2.587	0	0.941	2.177	0	5.5438	47.76	13.4	10.36	0.929	1.613	0	28.84	11.4	41.89	3.169	10.37	0	0	0.96	3.33	14
Os01g0518651	0	0	0	0	0	0	0	0	0	0	0	0	0	0	0	0	0	0	0	0	0	0	0	0	6.338	0	0	0	0	0	1
Os01g0223700	409	451	441	754.5	902.2	820.9	361	373	439	312.1	418.8	373.5	329.82	430.32	388.86	776.2	531.8	406.1	513.7	662	656.1	669.1	506	623.3	481.7	463.9	405.7	482	374	417	10
Os01g0232700	1197	1231	1119	1906	2057	1849	1425	1387	1607	1271	1360	1280	1471.7	1458.1	1279	2149	994.5	1271	1889	1865	1991	1227	1205	1252	1276	1116	1094	982	1032	1021	10
Os01g0877300	590	550	553	553.3	530.6	584.8	466	479	468	506.1	575.6	548.5	549.7	512.46	551.21	1218	694.6	594.7	514.6	558.8	554.4	614.4	476	460.8	616.9	572.3	585.5	579	630	653	13
Os01g0783800	249	236	239	202	313.7	254.3	229	161	176	172.4	168.8	99.73	134.98	167.09	181.36	370.2	178.3	210.3	90.11	171.7	153.5	334.9	285.1	194.3	155.3	141.4	149.1	202	204	195	8
Os01g0332200	2.04	1.86	1.08	64.86	83.17	38.93	0.78	0.63	0.69	0	0	0.941	3.2656	4.6673	9.5036	23.88	4.122	2.072	95.68	76.6	87.95	0.739	0.713	2.167	0	0.943	0	10.2	6.69	1.67	10
Os01g0106700	945	973	835	1571	1574	1420	519	615	511	538.9	615.1	536.3	837.07	722.49	603.48	1194	834.7	939.7	1002	1198	1014	797.1	812.4	611	634.9	685.4	627.2	843	860	875	2
Os01g0960000	400	398	413	342.3	405	358.1	387	338	357	399.2	333.3	363.2	361.39	404.18	422.12	680.6	420.5	387.5	451.5	394.3	390.6	289.1	313.6	332.2	371.8	352.6	399.1	383	384	374	6
Os01g0819466	1422	1664	1655	1377	1558	1400	1870	2111	2016	1437	1258	1392	1502.2	1475.8	1414.5	1863	1384	1350	1174	961.9	1120	1556	1536	1686	1206	1204	1349	1155	1203	1376	18
Os01g0740500	239	303	173	1149	1350	674.7	364	1243	393	145.7	164.5	125.1	115.38	138.15	211.45	441.8	255.6	280.8	504.4	472.5	468.2	607.8	1012	387.8	306.3	181	197.4	228	230	301	12
Os01g0530650	7.14	4.66	11.9	15.6	18.98	13.84	18	8.86	11.7	9.484	18.64	11.29	5.4426	3.7338	4.7518	11.94	8.244	8.288	11.15	8.063	11.21	7.394	7.126	15.17	6.338	3.771	0	8.33	7.65	8.33	12
Os01g0167600	2.04	6.52	5.39	10.67	9.04	11.25	15.7	15.2	11.7	7.76	4.386	13.17	2.177	5.6007	4.7518	35.82	5.153	6.216	9.29	6.45	6.898	17.75	10.69	20.22	3.169	13.2	2.193	8.33	1.91	8.33	6
Os01g0577300	51	19.6	38.8	15.6	3.616	27.68	2.35	0.63	0	16.38	7.675	14.11	15.239	34.538	30.887	226.9	59.77	35.23	9.29	24.19	10.35	3.697	9.977	11.56	24.3	34.89	30.7	93.4	50.7	41.7	13
Os01g0315700	0	0	1.08	0	0.904	5.19	2.35	0	1.38	2.587	2.193	10.35	0	0	0	0	3.092	2.072	3.716	3.225	0	0	1.425	0.722	0	0	4.386	0	0	0.83	20
Os01g0739200	1791	1139	1370	280.8	235.9	212.8	230	237	218	721.7	692.9	844.9	637.87	665.55	628.03	1051	988.3	989.4	303.8	304	341.4	206.3	254.4	218.1	672.9	740.1	778.5	521	530	532	14
Os01g0978500	275	299	234	249.6	234.1	205.9	496	462	464	263	263.1	318.9	305.87	218.43	209.08	262.7	524.5	336.7	332.6	289.5	313.8	363	315.7	463	304.2	335.7	344.3	343	270	327	18
Os01g0955400	1.02	2.79	2.16	0	0	0	0	0	0	1.724	1.096	0.941	3.2656	7.4676	2.3759	0	2.061	1.036	0	0	0.862	0.739	0	0	0	0.943	0	1.85	0	0	4
Os01g0786700	0	0.93	0	0	0.904	0	0	0	1.38	1.724	0	2.822	0	0	0	0	0	0	0	0.806	0	2.218	0	0	0	0.943	2.193	0.93	0	0	20
Os01g0838400	228	279	279	179	189.8	186	138	211	115	204.3	212.7	197.6	212.26	232.43	216.21	322.4	217.4	249.7	183.9	246.7	152.6	135.3	144	125.7	243	223.5	182	200	216	239	13
Os01g0777000	260	330	254	353.8	377.9	319.2	186	262	198	167.3	184.2	98.79	213.35	148.42	207.5	358.2	197.9	215.5	205.3	202.4	165.5	315.7	300	311.3	176.4	136.7	157.9	265	249	284	8
Os01g0912850	5.1	6.52	3.23	4.105	2.712	6.92	11	1.27	8.29	6.897	4.386	4.704	6.5311	5.6007	3.9598	0	6.183	4.144	10.22	8.063	8.622	13.31	17.82	17.33	9.507	3.771	0	8.33	6.69	15	9
Os01g0242600	525	587	466	878.4	800.9	957.6	661	635	615	389.7	395.8	298.2	367.92	435.92	475.18	859.7	565.8	510.8	747.8	752.3	671.7	927.9	937.1	827.7	602.1	502.5	508.8	709	472	596	12
Os01g0660550	579	597	628	481.9	462.8	560.6	543	430	477	576.8	492.3	359.4	545.35	533.93	662.08	1063	616.3	584.3	485.8	564.4	495.8	857.7	701.9	567	613.7	546.8	475.9	683	709	679	6
Os01g0101150	12.2	3.73	3.23	28.73	31.64	26.82	5.48	7.59	10.4	0.862	1.096	0	3.2656	4.6673	3.9598	23.88	2.061	4.144	3.716	16.13	4.311	17.01	14.25	11.56	3.169	3.771	0	2.78	7.65	4.17	2
Os01g0272800	0	0	0	1.642	0	3.46	0.78	1.27	0.69	0	0	0	0	0	0	0	0	0	2.787	2.419	2.587	0	0	0	0	0	0	0	0	0	10
Os01g0559200	139	183	156	200.3	197.1	224	168	153	178	215.5	197.4	273.8	197.02	222.16	217.79	620.9	191.7	193.7	227.6	216.1	203.5	175.2	170.3	144.4	187	270.6	247.8	181	208	188	6
Os01g0149600	426	293	445	463.9	457.4	402.2	489	431	460	415.6	410.1	483.6	397.31	294.97	348.47	406	421.5	354.3	587.1	511.2	527.7	322.4	375.6	315.6	370.8	316.8	313.6	310	341	377	10
Os01g0126900	0	0	0	0	0	0	0	0	0	0	0	0	0	0	0	0	0	0	0	0	0	0	2.138	0	0	0	0	0	0	0	17
Os01g0702900	0	0	0	0.821	0	0	0	0.63	0	0	0	0	0	0	0	0	0	0	0	0	0	0	0.713	0	0	0	0	0	1.91	0	1
Os01g0596700	1.02	0	0	0	0	0	0	0	2.76	0	0	0.941	0	0	0	0	0	0	0	0	0	0	0	0	0	0	0	0	0	0	5
Os01g0611900	153	175	169	217.6	307.3	300.2	193	166	182	130.2	164.5	79.03	141.51	174.56	159.19	322.4	232.9	160.6	227.6	265.3	219.9	211.5	176.7	155.3	158.5	152.7	188.6	170	160	176	10
Os01g0148000	209	276	237	313.6	294.7	255.2	69.7	95.5	51.1	65.53	84.42	44.22	99.055	75.61	147.31	358.2	132.9	185.5	66.88	58.05	70.7	278.7	228.8	118.4	114.1	66.94	52.63	216	176	235	8
Os01g0962700	108	86.6	87.3	341.5	329	221.5	543	677	1044	171.6	121.7	175.9	68.577	90.545	98.996	83.59	46.37	59.05	273.1	269.3	101.7	1309	2094	1138	125.7	67.88	122.8	89.7	187	76.6	17
Os01g0804900	13.3	23.3	11.9	15.6	19.89	17.3	17.2	13.9	7.6	19.83	14.25	14.11	15.239	17.736	22.175	35.82	10.31	14.5	18.58	7.257	9.484	10.35	26.37	14.44	13.73	5.657	13.16	23.1	21	19.2	6
Os01g0636600	196	131	222	188	152.8	210.2	57.2	56.9	46.3	125.9	140.3	179.7	177.43	205.36	171.86	417.9	199.9	162.7	225.7	249.9	256.1	54.71	64.85	62.84	159.5	163.1	120.6	172	185	152	13
Os01g0390400	1400	1376	1585	978.6	1076	1112	723	903	956	1094	1297	1134	1846.1	1507.5	1662.3	2985	1780	1548	1081	1263	1045	1117	1112	873.2	1503	1376	1320	1891	1818	2079	13
Os01g0882000	8.16	7.45	7.54	0	0.904	0.865	1.57	0	0	4.311	0	0	1.0885	2.8004	3.1679	0	10.31	10.36	4.645	0	0.862	0	0.713	1.444	2.113	1.886	2.193	5.55	3.83	2.5	14
Os01g0947800	3.06	1.86	0	0	0	0.865	0.78	0.63	0.69	0	1.096	0.941	1.0885	0.9335	0	0	0	0	0.929	0	0	1.479	1.425	2.889	0	0	0	0	0.96	0.83	9
Os01g0203400	14.3	21.4	24.8	15.6	7.232	7.785	42.3	49.3	63.6	95.7	127.2	113.8	59.869	46.673	41.182	83.59	14.43	10.36	16.72	8.063	16.38	125.7	109.7	80.89	55.99	42.43	52.63	24.1	57.4	72.5	5
Os01g0604500	89.7	56.8	93.7	9.852	6.328	6.92	17.2	15.8	17.3	35.35	28.51	29.17	31.567	24.27	23.759	23.88	52.56	62.16	8.361	7.257	4.311	17.75	15.68	26.72	24.3	28.29	17.54	29.6	23.9	27.5	14
Os01g0859100	15.3	15.8	24.8	11.49	6.328	30.28	8.61	1.27	19.3	5.173	5.482	2.822	13.062	5.6007	5.5438	0	9.275	9.324	7.432	2.419	9.484	10.35	22.8	13	5.282	10.37	15.35	14.8	26.8	9.16	8
Os01g0584900	85.6	81	114	25.45	42.49	34.6	61.1	50	56.7	189.7	92.1	73.38	82.728	68.142	122.75	167.2	74.2	84.96	24.15	82.24	48.28	33.27	57.72	41.17	35.92	34.89	72.37	41.6	66	76.6	13
Os01g0496900	335	327	342	382.6	339.9	328.7	219	298	277	326.8	290.5	297.3	287.37	267.9	347.67	704.5	296.8	349.1	336.3	341.9	341.4	269.1	299.3	205.8	316.9	259.3	311.4	277	338	318	6
Os01g0566800	8.16	6.52	0	0	0	1.73	7.83	0	14.5	3.449	15.35	2.822	0	4.6673	0	0	10.31	6.216	0	1.613	0.862	39.19	7.126	48.39	6.338	3.771	17.54	6.48	7.65	0.83	9
Os01g0954500	138	109	143	111.7	69.61	109.9	142	140	152	126.7	112.9	110.1	167.63	112.95	174.23	358.2	113.4	89.1	115.2	97.56	125	82.81	192.4	148.8	81.34	55.63	103.1	125	208	153	6
Os01g0704200	332	279	321	314.4	335.4	314	274	325	279	306.9	349.7	290.7	378.8	378.05	341.34	561.2	331.8	359.5	414.3	374.1	434.6	237.3	233	272.3	299	358.3	346.5	358	372	378	13
Os01g0637100	0	0	0	0	0	0	0	0	0	0	0	0	0	0	0	0	0	0	0	0	0	0	0	0	0	0	2.193	1.85	0	0	11
Os01g0929967	1.02	0	0	0	0.904	0	0	0	0	0	0	0	0	0	0	0	0	0	0.929	0	0	0.739	0.713	0.722	0	0	0	0	0	0	9
Os01g0510600	820	1080	1031	1930	2329	1645	1409	1680	1493	1060	1275	1296	1208.3	853.17	896.51	1588	607	948	1577	1768	1569	1128	1085	1220	946.5	660.9	609.6	611	617	721	10
Os01g0168600	225	256	213	190.5	136.5	204.2	119	130	133	171.6	168.8	143.9	176.34	194.16	201.16	214.9	300.9	225.9	120.8	180.6	165.5	184.1	188.8	210.9	231.3	208.4	203.9	232	220	247	1
Os01g0977250	56.1	33.5	59.3	32.02	46.1	47.58	76.7	66.4	69.1	85.36	67.98	102.5	88.17	70.942	83.948	203	52.56	45.59	69.67	69.34	64.67	39.93	39.19	41.17	42.25	76.37	67.98	51.8	48.8	61.7	6
Os01g0708600	470	496	452	508.2	480	571.8	605	591	656	420.7	422.1	436.5	531.2	588.07	480.72	1218	515.3	407.2	586.2	483.8	526	516.8	478.9	569.9	407.8	433.7	587.7	419	419	453	6
Os01g0895500	109	114	103	101	94.01	107.3	103	120	118	107.8	125	72.44	89.259	101.75	88.7	250.8	103.1	89.1	84.53	97.56	75.88	122	124.7	117.7	120.4	102.8	96.49	88.8	82.2	100	6
Os01g0686300	0	0	0	0	0	0	0	0	0	0	0	0	0	0	0	0	0	0	0	0	0	0.739	0	0.722	0	0	0	0	0	0	9
Os01g0805400	35.7	61.5	33.4	107.5	91.3	76.99	157	216	211	66.39	49.34	36.69	39.187	47.606	60.981	23.88	29.89	40.41	89.18	101.6	50.01	127.9	178.9	113.4	28.52	47.14	19.74	35.2	35.4	51.7	18
Os01g0663400	566	359	517	397.4	402.3	436.8	187	189	218	308.7	269.7	372.6	220.97	480.73	312.83	1003	674	590.5	549.9	452.3	569.1	286.9	378.4	416.7	439.4	582.7	651.3	602	575	452	13
Os01g0915732	0	0	0	0	0.904	0	0	0	0	0	0	0	1.0885	3.7338	0	0	0	1.036	0	0	0	2.218	0	0	1.056	0	0	0.93	0	0	4
Os01g0138400	9.18	6.52	9.7	41.87	41.58	31.14	0.78	8.86	6.91	4.311	3.289	6.586	1.0885	4.6673	6.3357	35.82	5.153	8.288	39.02	61.28	31.04	25.14	42.05	17.33	4.225	3.771	8.772	9.25	2.87	8.33	10
Os01g0939550	73.4	62.4	56	63.22	94.01	64.88	90	91.1	95.4	98.29	75.65	104.4	70.754	62.541	51.478	155.2	39.16	61.13	87.32	109.7	86.22	46.58	49.17	34.67	67.61	64.11	57.02	64.8	48.8	36.7	6
Os01g0184100	5.1	14	11.9	20.52	14.46	23.36	7.83	3.16	7.6	12.07	5.482	8.467	7.6196	13.068	15.839	35.82	8.244	3.108	13.01	16.93	15.52	8.133	8.552	3.611	1.056	10.37	21.93	13	5.74	4.17	6
Os01g0283700	3.06	2.79	4.31	7.389	2.712	12.11	0	2.53	1.38	1.724	3.289	0.941	2.177	6.5342	3.1679	0	3.092	8.288	0.929	2.419	0	2.218	2.851	0	2.113	1.886	6.579	8.33	7.65	7.5	8
Os01g0794900	63.2	82	64.7	58.29	58.76	43.25	39.1	51.9	29.7	29.31	27.41	25.4	10.885	16.802	25.343	119.4	94.81	69.41	39.02	37.9	31.04	122	142.5	75.84	63.38	31.11	43.86	75.9	76.5	78.3	17
Os01g0311100	59.1	64.3	88.4	66.5	74.13	50.17	104	78.4	57.4	126.7	73.46	87.5	47.895	40.138	57.022	119.4	99.96	78.74	54.81	58.05	80.19	36.97	23.52	71.5	54.93	43.37	50.44	24.1	32.5	34.2	7
Os01g0814400	1137	917	1011	410.5	444.8	481.8	427	562	533	394	423.2	384.8	349.41	398.58	434.79	812	792.5	808.1	333.5	426.5	423.3	578.2	532.3	665.9	342.3	454.4	464.9	521	590	531	14
Os01g0933075	62.2	59.6	56	108.4	112.1	88.23	179	186	138	44.83	43.86	51.75	46.806	48.539	51.478	47.76	39.16	61.13	90.11	95.91	81.05	70.24	119	93.17	51.76	53.74	17.54	44.4	44.9	47.5	12
Os01g0589400	6.12	8.38	4.31	78.81	84.97	69.2	55.6	50.6	40.1	3.449	4.386	8.467	14.151	7.4676	3.1679	11.94	10.31	20.72	66.88	88.69	72.43	29.58	40.62	33.95	3.169	5.657	2.193	7.4	13.4	5.83	10
Os01g0862500	0	0	0	2.463	0	0.865	0.78	3.16	0.69	0.862	0	0	0	0	0	0	0	0	0.929	0	1.724	4.436	4.988	1.444	0	0	0	0	0	0	17
Os01g0633100	1609	1671	1832	935.9	1093	962.8	811	980	808	1114	1309	1142	1403.1	1272.3	1439.8	3797	1979	2045	1038	1088	925.2	1308	1030	889.8	1530	1483	1210	2045	1955	2239	13
Os01g0899700	2.04	0.93	0	0.821	0	0	3.13	6.96	2.76	0	0	0	1.0885	0	0.792	0	2.061	1.036	0	0	4.311	0.739	0	9.389	3.169	4.714	4.386	0	4.78	3.33	9
Os01g0263600	305	280	277	273.4	245.9	269.9	125	127	136	257.8	209.4	249.3	338.53	259.5	330.25	525.4	289.6	331.5	194.2	269.3	238.8	134.6	169.6	152.4	269.4	244.2	228.1	311	290	341	13
Os01g0549300	0	0	0	0	0	0	0	0	0	0	1.096	0	0	0	0	0	0	0	0	0	0	0	0	0	0	0	0	0	0	0	7
Os01g0640700	410	346	277	214.3	222.4	274.2	227	247	193	336.3	324.5	328.3	280.84	435.92	389.65	406	557.5	365.7	304.7	304.8	411.3	355.6	449.7	413.8	391.9	472.4	486.8	536	339	401	1
Os01g0783600	187	174	199	233.2	244.1	280.3	237	213	305	219.9	202.8	230.5	277.57	229.63	200.37	250.8	211.3	190.6	288.9	256.4	303.5	201.1	210.2	234	253.5	192.3	276.3	206	190	178	15
Os01g0350300	0	0.93	1.08	0.821	0	0	0	0.63	0	0	0	0	0	0	0	0	0	0	0	0	0	0	0	0.722	0	0	0	0	0.96	0	14
Os01g0962000	120	90.4	105	105.1	81.36	109.9	178	170	168	170.7	180.9	216.4	103.41	160.55	139.39	155.2	151.5	169.9	187.6	148.4	146.6	166.4	126.1	143	169	226.3	241.2	167	189	146	20
Os01g0768333	59.1	76.4	47.4	11.49	12.66	5.19	1.57	16.4	16.6	2.587	5.482	1.882	2.177	1.8669	1.5839	11.94	20.61	17.61	20.44	7.257	5.173	15.53	22.8	40.45	0	0.943	4.386	0	0	0.83	14
Os01g0217000	1.02	0	1.08	0	2.712	0	7.05	1.27	0	5.173	4.386	2.822	1.0885	0.9335	1.5839	11.94	0	0	0.929	1.613	1.724	2.958	4.988	7.945	4.225	1.886	13.16	2.78	3.83	0.83	11
Os01g0157600	0	2.79	0	0	0	0	0	0	0	0	0	0	0	0	0	0	0	0	0.929	0	0	0	0	0	1.056	0	0	0	0	1.67	14
Os01g0273300	263	277	291	509.8	513.5	475.8	459	478	489	319	285.1	279.4	335.26	280.97	349.26	417.9	258.7	251.8	518.4	463.6	498.4	378.6	409.8	406.6	305.3	221.6	254.4	237	307	282	12
Os01g0581800	4.08	1.86	1.08	0.821	6.328	2.595	0	0.63	1.38	3.449	0	1.882	0	0	5.5438	0	0	0	0.929	0.806	0	0.739	2.138	1.444	0	4.714	0	0	4.78	0	2
Os01g0668700	118	121	147	214.3	206.1	159.2	259	230	198	137.1	118.4	131.7	131.71	135.35	125.92	143.3	84.5	110.9	191.4	197.5	178.5	210.7	166	187.8	120.4	101.8	98.68	146	164	117	12
Os01g0758500	13.3	3.73	4.31	4.926	2.712	4.325	1.57	0.63	0.69	4.311	7.675	10.35	10.885	10.268	3.9598	11.94	7.214	4.144	2.787	0.806	1.724	0.739	0	0	0	9.428	6.579	8.33	1.91	3.33	13
Os01g0583900	0	0	0	0	0.904	0	0	0	0	0	0	0	0	0	0	0	0	0	0	0	0	0	0	0.722	0	0	0	0	0	0	2
Os01g0222001	3.06	1.86	1.08	9.031	4.52	4.325	0	6.96	10.4	6.897	1.096	0.941	0	1.8669	0	11.94	0	1.036	0	0.806	2.587	0.739	4.988	0.722	0	0	2.193	0	0.96	0	6
Os01g0225700	4.08	5.59	2.16	12.31	7.232	21.63	3.91	13.3	14.5	3.449	3.289	4.704	11.974	7.4676	1.5839	0	6.183	5.18	11.15	12.9	24.14	6.654	4.276	7.222	2.113	2.829	13.16	2.78	11.5	1.67	10
Os01g0148100	559	623	547	659.2	728.6	681.7	621	657	657	598.4	778.4	667	634.61	556.73	609.02	1087	616.3	570.9	577.8	637.8	663.9	817.8	768.2	655.8	685.6	709	679.8	665	732	666	6
Os01g0965400	9.18	9.31	20.5	24.63	39.77	25.95	6.26	9.49	9.67	10.35	8.771	10.35	13.062	4.6673	6.3357	0	4.122	3.108	8.361	3.225	11.21	11.83	7.126	10.11	11.62	3.771	2.193	6.48	4.78	15.8	2
Os01g0759000	5.1	1.86	2.16	4.926	5.424	7.785	6.26	1.9	1.38	6.035	2.193	4.704	3.2656	6.5342	4.7518	0	5.153	8.288	8.361	5.644	5.173	0.739	2.138	3.611	0	8.486	2.193	0.93	0.96	3.33	16
Os01g0869900	956	1134	884	504.1	547.8	464.5	773	606	548	782.9	849.7	748	910	761.7	806.22	1278	1013	910.7	483.1	451.5	508.7	428.8	478.2	462.2	799.7	720.3	760.9	867	851	825	13
Os01g0923700	1083	1264	1032	1511	1536	1471	2434	2699	2193	946.7	1118	865.6	1395.5	1182.7	1476.2	1767	1124	1147	1262	1328	1205	2200	2508	2265	1112	1039	1123	1221	1280	1473	18
Os01g0689000	88.7	73.6	66.8	28.73	37.06	32.01	50.9	40.5	45.6	64.66	50.43	44.22	28.302	49.473	37.222	35.82	73.17	42.48	36.23	25.8	39.66	51.02	33.49	47.67	31.69	37.71	37.28	24.1	34.4	35.8	14
Os01g0897300	67.3	74.5	83	41.05	39.77	66.61	50.9	46.8	51.1	55.18	60.3	59.27	48.983	42.939	32.471	179.1	93.78	72.52	31.58	41.93	41.39	78.37	65.56	77.28	48.59	64.11	48.24	63.8	64.1	73.3	6
Os01g0595300	109	115	129	169.1	169	162.6	146	149	134	113.8	95.39	96.9	130.62	112.95	151.27	214.9	98.93	120.2	105	132.2	138	137.5	140.4	157.4	103.5	99.94	127.2	136	124	144	6
Os01g0936800	852	746	874	513.9	550.5	532	496	436	465	795.8	627.1	617.2	704.27	717.82	719.9	1182	760.5	841.3	531.4	532.1	440.6	452.5	542.3	414.6	758.5	714.7	714.9	789	739	775	13
Os01g0115750	5.1	10.2	7.54	6.568	9.944	1.73	32.1	19	13.8	69.84	43.86	48.92	29.39	23.336	32.471	0	3.092	12.43	9.29	16.13	8.622	16.27	9.264	13	26.41	26.4	32.89	19.4	15.3	6.66	7
Os01g0100100	839	970	964	936.7	1010	900.5	963	1126	936	1088	1072	1130	1067.8	962.39	1039.1	1588	991.4	954.2	1004	973.2	906.2	780.8	830.9	850.1	1110	980.6	912.3	942	984	1046	6
Os01g0103800	233	290	249	366.2	371.5	385.8	429	349	406	313.8	310.3	273.8	314.58	266.97	300.95	716.5	284.4	243.5	385.5	333	416.5	303.1	373.4	382.8	323.2	288.5	276.3	296	342	297	6
Os01g0841500	329	325	343	323.5	313.7	383.2	237	316	328	309.5	287.3	235.2	358.12	353.78	377.77	823.9	378.2	398.9	263.8	333	313	431.8	438.3	370.5	359.2	325.3	333.3	409	337	391	6
Os01g0865100	158	170	100	486	596.6	403.1	2583	2304	2729	291.4	237.9	329.3	124.09	107.35	129.09	47.76	87.6	105.7	287	249.9	177.6	1190	1702	1148	124.7	168.8	195.2	68.5	90.9	55.8	18
Os01g0583300	10.2	2.79	7.54	40.23	25.31	13.84	5.48	22.1	11.1	6.035	7.675	6.586	0	12.135	8.7116	35.82	8.244	9.324	22.29	16.93	19.83	5.176	4.276	7.222	6.338	2.829	0	3.7	5.74	0.83	10
Os01g0717000	1690	1955	1575	3733	3732	3741	949	1261	1033	789.8	771.9	661.4	893.67	986.66	1011.3	1815	1579	1488	3015	3653	3955	1397	1190	1044	975	1093	1397	1502	1175	1310	10
Os01g0265700	184	216	218	298.8	320.9	299.3	249	245	245	249.2	286.2	259.7	311.32	251.1	243.13	632.9	255.6	220.7	326.1	374.9	325.9	215.2	218.8	223.9	270.4	269.7	250	279	252	274	6
Os01g0883600	0	0	0	0	0	0	0	0	0	0	0	0	0	0	0	0	0	0	0	0	0	0	0	0	0	0.943	0	0	0	0	1
Os01g0590800	2.04	0	0	0	0	0	0	0	0	1.724	0	9.408	0	0	0	0	0	0	0	0	0	0.739	0	0	1.056	0	0	0	0	0.83	20
Os01g0771000	16.3	6.52	14	3.284	3.616	12.11	3.13	0.63	2.76	17.24	6.578	24.46	11.974	6.5342	13.463	11.94	16.49	6.216	20.44	12.9	13.8	2.218	4.276	10.11	7.395	11.31	43.86	7.4	16.3	5	11
Os01g0544200	1.02	8.38	0	4.926	1.808	3.46	9.39	1.27	8.9	8.622	3.289	15.05	6.5311	3.7338	3.9598	0	3.092	3.108	7.432	4.031	0	9.612	8.552	19.5	8.451	5.657	15.35	4.63	3.83	2.5	9
Os01g0188900	1590	1441	1606	2512	2763	2482	2418	2584	2061	1037	1011	1099	1369.4	1226.6	1054.9	2902	1185	1501	2461	2938	2490	2196	1846	1783	863	891	903.5	1052	1320	1129	12
Os01g0279300	872	1090	1120	1115	1223	968	1066	1240	1189	1022	959.3	785.6	1018.9	899.85	1006.6	1791	859.5	829.9	957.8	793.4	752.7	794.1	984.9	886.2	862	755.2	1013	945	924	936	6
Os01g0267600	56.1	64.3	85.1	85.38	60.57	78.72	46.2	60.7	72.6	56.04	58.11	65.86	53.337	47.606	56.23	131.4	54.62	59.05	63.17	87.88	60.36	55.45	70.55	50.56	67.61	58.46	63.59	45.3	57.4	53.3	6

Os01g0975900	####	####	####	8431	4219	13768	1091	389	385	37256	33057	36736	32490	32460	28772	29900	22459	21011	22001	14310	17485	370.4	250.8	754.7	29260	24117	31922	####	####	####	3
Os01g0977100	1.02	0	1.08	0	0.904	3.46	0	0.63	0	0	4.386	1.882	0	0	0	0	2.061	0	0	0	0	0.739	0	0	1.056	1.886	2.193	0	0	0	20
Os01g0735300	197	94.1	139	13.14	2.712	19.9	27.4	21.5	4.84	16.38	16.45	55.51	37.01	126.95	65.733	131.4	491.6	182.3	39.02	31.44	43.97	25.88	77.68	187.1	78.17	83.91	197.4	189	132	127	1
Os01g0612000	409	447	415	414.6	437.5	411.8	379	392	415	388	414.4	399.8	429.97	420.05	468.84	764.2	424.6	425.8	391.1	395.1	468.2	393.4	441.1	390.7	477.5	386.6	508.8	507	397	442	6
Os01g0813800	40.8	57.7	49.6	55.83	37.97	36.33	67.3	25.9	110	21.55	16.45	8.467	28.302	16.802	9.5036	23.88	23.7	7.252	36.23	54.83	13.8	143.4	57.72	113.4	39.09	8.486	6.579	38.9	72.7	60	9
Os01g0750400	0	0	0	0	0	0	0	0	0	0	0	0	0	0	0	0	0	0	0	0	0.862	0	0	0	0	0	0	0	0	0	16
Os01g0574800	107	77.3	74.4	50.08	41.58	67.47	162	156	126	219	157.9	369.7	138.24	150.29	149.68	179.1	72.14	119.1	58.52	35.48	70.7	86.51	95.49	92.45	131	172.5	157.9	117	103	134	20
Os01g0678400	0	0	1.08	0	0	0	2.35	0	0	0	0	0	0	0	0	0	0	0	0	0	0.862	0	0	0	0	0	0	0	0	0	5
Os01g0795766	23.4	34.5	34.5	17.24	23.5	30.28	55.6	66.4	63.6	18.11	26.31	24.46	19.593	23.336	17.423	35.82	29.89	25.9	27.87	34.67	52.6	45.84	39.91	72.22	16.9	16.03	26.32	15.7	22	14.2	18
Os01g0251400	0	0	0	0	1.808	0	0	0	0	0	0	0	0	0	0	0	0	0	0	0	0	0	0	0	0	0	0	0	0	0	2
Os01g0783100	248	224	245	510.6	546	484.4	594	701	741	184.5	198.4	290.7	209	144.68	145.72	286.6	196.8	232.1	414.3	514.4	486.3	598.9	543	569.1	182.8	216.9	140.3	167	211	177	12
Os01g0656200	139	94.1	136	196.2	182.6	191.2	25.1	17.1	24.9	50.01	49.34	31.05	51.16	70.009	46.726	71.65	81.41	128.5	197.9	253.2	260.4	22.92	19.24	17.33	21.13	39.6	17.54	59.2	34.4	43.3	10
Os01g0256500	3010	3173	2405	728.2	543.3	709.3	2191	1744	3991	4142	3226	5494	2735.5	1924.8	2294.3	2687	2646	2305	893.7	700.7	785.5	2802	2356	2971	2546	2541	2487	1442	1122	1369	20
Os01g0388000	0	6.52	2.16	4.926	4.52	0	7.05	6.33	10.4	2.587	7.675	5.645	7.6196	6.5342	5.5438	23.88	2.061	0	0.929	1.613	0.862	22.18	11.4	20.22	0	6.6	0	1.85	8.61	8.33	9
Os01g0212100	951	962	949	901.4	872.3	847.7	549	636	619	709.6	827.8	618.1	1071.1	924.12	1030.3	1636	873.9	921	698.6	760.3	726.8	955.3	818.8	680.4	894.7	866.5	756.6	1079	957	1024	13
Os01g0355500	762	659	726	623.1	667.1	601.2	468	521	509	574.2	582.2	547.6	729.31	606.74	656.54	1302	743	758.4	495.1	551.5	553.5	601.1	646.4	533.7	687.7	557.2	570.2	661	738	773	13
Os01g0904700	275	240	220	267.6	305.5	276.8	281	306	303	232.8	229.1	257.8	248.18	261.37	213.04	298.5	213.3	285.9	225.7	249.1	271.6	266.2	203.8	287.5	185.9	303.6	346.5	223	257	291	11
Os01g0821900	39.8	36.3	39.9	13.96	15.37	29.41	41.5	23.4	44.9	29.31	31.8	42.34	31.567	35.471	28.511	0	38.13	34.19	33.44	28.22	32.76	67.28	42.76	44.78	40.14	56.57	50.44	52.7	36.3	33.3	9
Os01g0654400	0	0	0	0.821	0.904	0	0	0	0	0	0	0	0	0	0	0	0	0	0	0.806	0	0	0	0	0	0	0	0	0	0	2
Os01g0239300	7.14	6.52	7.54	52.54	47.91	27.68	40.7	29.1	18.7	11.21	9.868	11.29	8.7082	1.8669	4.7518	11.94	2.061	5.18	30.66	21.77	25	28.1	16.39	16.61	7.395	4.714	8.772	4.63	4.78	9.16	12
Os01g0266800	3692	3007	4033	60.75	58.76	103.8	67.3	78.4	54.6	3536	4228	3886	4590.3	3981.2	4397	8944	5051	4083	220.2	137.1	138	56.19	66.99	83.78	4335	4820	3686	4389	4478	4336	13
Os01g0237100	1085	1253	1204	5345	5409	5041	1464	1648	1648	1617	1877	1855	2013.8	1546.7	1433.5	1863	930.6	983.2	4854	4626	5455	1221	1302	1588	1722	1608	1375	898	847	1046	10
Os01g0118400	102	65.2	87.3	57.47	33.45	156.6	175	84.1	271	132.8	66.88	71.5	63.134	75.61	95.036	47.76	70.08	46.62	99.4	116.1	106.1	200.4	129	273.7	50.71	72.6	173.2	36.1	85.1	51.7	9
Os01g0902800	0	0	0	0	0	0	0	0	0	0	0	0	0	0	0	0	0	0	0	0	0	0	1.425	0	1.056	0	0	0	0	0	17
Os01g0149700	0	1.86	0	0.821	0.904	1.73	5.48	0	0.69	0	3.289	2.822	1.0885	3.7338	0.792	0	4.122	0	4.645	1.613	0	2.218	0.713	2.889	2.113	0	0	3.7	0	0	15
Os01g0340600	0	0	0	0	0	0	0	0	0	0	0	0.941	0	0	0	0	0	0	0	0	0	0	0.713	0	0	0	0	0	0	0	20
Os01g0623600	1.02	0	1.08	0	0	0	0	0	0	0.862	0	0	1.0885	0	0	0	0	2.072	0	0	0	0	0	0	1.056	0	0	0	0	0	19
Os01g0189100	87.7	98.7	95.9	371.9	438.4	391	132	132	119	44.83	55.92	47.04	63.134	68.142	43.558	131.4	80.38	45.59	196	308	219	108	119	91.73	48.59	44.31	41.67	56.4	64.1	55	10
Os01g0644500	0	0	0	0	0.904	0	3.13	0.63	2.07	0.862	1.096	0	1.0885	0	0	0	0	0	0	0	0	0	0	0	1.056	0.943	0	0	0	1.67	5
Os01g0815100	215	208	204	105.9	87.69	106.4	276	215	238	284.5	213.8	247.4	181.78	193.22	183.74	334.3	246.3	219.6	162.6	133.8	153.5	184.1	192.4	224.6	173.2	232.9	269.7	181	165	167	5
Os01g0273150	7.14	1.86	10.8	5.747	7.232	10.38	14.1	3.16	2.07	4.311	1.096	6.586	4.3541	4.6673	5.5438	11.94	4.122	4.144	6.503	6.45	2.587	12.57	9.977	13.72	0	1.886	4.386	3.7	3.83	4.17	9
Os01g0578000	215	222	178	243	276.6	250	412	354	397	249.2	245.6	211.7	220.97	245.5	200.37	406	211.3	226.9	271.3	285.4	313.8	367.5	320	360.4	206	205.5	243.4	229	169	234	18
Os01g0767000	1916	2181	1939	2411	2504	2599	2038	2488	2357	1598	1639	1750	2076.9	1917.3	1927.6	5158	2350	2352	2059	2113	2103	2748	2705	2667	2133	2195	1765	2414	2359	2517	6
Os01g0128400	1207	1155	1145	1188	1052	1202	605	592	659	1199	1158	959.6	976.4	1108.9	1115.9	1684	1106	1011	1223	1222	1239	703.2	720.5	679.6	1117	1004	993.4	1098	1061	1059	13
Os01g0253050	0	2.79	2.16	0	0.904	0	0.78	1.27	0	0.862	2.193	0	2.177	0	1.5839	0	2.061	1.036	0	0	0	0	0.713	0	0	0	0	0.93	0	3.33	4
Os01g0369300	0	0	0	0	0	0	1.57	0	0	0	1.096	0	0	0	0	0	0	0	0	1.613	0.862	0	0	0	1.056	0	0	0.93	0.96	0	16
Os01g0213400	29.6	46.6	45.3	73.07	75.03	46.71	31.3	17.7	41.5	18.97	6.578	4.704	7.6196	13.068	24.551	0	26.79	18.65	63.17	41.93	36.21	102.8	80.53	108.3	8.451	5.657	6.579	6.48	5.74	7.5	9
Os01g0952700	62.2	154	106	187.2	208.8	205	539	536	635	104.3	109.6	126.1	66.4	95.212	72.069	262.7	89.66	87.03	185.8	205.6	179.3	544.9	460.4	477.4	120.4	116	149.1	74	105	92.5	18
Os01g0645000	0	0	0	6.568	11.75	0	0	0.63	2.07	6.035	6.578	0	0	0	0	0	0	0	0	0	0	0	0	0	0	0	0	0	0	0	2
Os01g0155800	0	0	0	27.91	27.12	1.73	4.7	0	0	0.862	0	0	4.3541	0.9335	0	0	0	0	0	1.613	0	0.739	0	0	0	0	0	4.63	0	0	2
Os01g0685500	267	282	282	336.6	260.3	257.8	238	256	207	213	267.5	206	253.63	290.3	245.51	429.9	261.8	278.7	280.5	284.6	263.8	227.7	265.1	218.1	268.3	231.9	282.9	276	287	269	6
Os01g0647000	225	244	258	133	132.9	127.2	334	312	238	469.9	439.7	414.9	343.97	352.84	391.23	417.9	262.8	289.1	138.4	172.5	194.9	186.3	297.2	244.8	433.1	355.5	285.1	421	338	325	3
Os01g0839400	0	1.86	1.08	8.21	9.04	8.65	3.13	3.16	2.07	0.862	1.096	0	0	0	0	0	0	1.036	5.574	8.869	7.76	9.612	5.701	2.167	0	0	0	0	0	0	10
Os01g0785600	0	0	0	0.821	0	0	0	0	0.69	0	0	0	0	0	0	0	0	0	0	0	0	0	0	0.722	0	0	0	0	0	0	9
Os01g0106900	7921	8274	8735	3811	3706	4261	5354	6089	6301	9307	11028	8510	10315	11141	11337	16586	9214	8707	3801	3444	4265	5390	4825	5363	11193	11522	10333	9502	9877	####	13
Os01g0971900	129	129	123	394.1	416.7	412.6	182	199	173	113.8	73.46	54.57	97.967	111.08	91.076	191.1	102	122.3	320.5	400.7	363.9	238.8	276.5	191.4	76.06	75.43	50.44	115	95.6	119	10
Os01g0374600	2.04	0.93	0	0	0	0	0	0	0	0	0	0	0	0	0	0	0	0	0	0	0	0	0	0	0	0	0	0	0	0	19
Os01g0704100	308	199	268	25.45	8.136	21.63	37.6	14.5	16.6	153.5	95.39	381	19.593	70.009	41.182	23.88	124.7	89.1	43.66	30.64	29.32	35.49	19.95	69.34	27.47	144.3	133.8	48.1	44.9	24.2	20
Os01g0840900	7.14	11.2	8.62	7.389	7.232	3.46	42.3	27.2	42.8	12.07	7.675	5.645	3.2656	7.4676	11.088	23.88	7.214	11.4	4.645	4.838	5.173	43.62	34.21	26.72	1.056	5.657	0	7.4	6.69	5	18
Os01g0926700	84.6	68	69	80.46	79.55	65.74	58.7	55	49.8	108.6	66.88	43.28	52.249	52.273	55.438	83.59	51.53	43.51	67.81	62.89	70.7	75.42	84.8	75.84	52.82	62.23	61.4	65.7	86.1	60	7
Os01g0872000	1.02	2.79	1.08	58.29	47.01	25.95	21.1	10.8	13.1	3.449	3.289	2.822	3.2656	0	0.792	11.94	1.031	2.072	31.58	29.03	15.52	28.1	20.67	31.78	2.113	5.657	4.386	0	3.83	4.17	10
Os01g0681600	116	185	147	147.8	144.6	140.1	291	391	360	121.6	179.8	95.96	119.74	100.81	158.39	119.4	116.5	126.4	53.88	62.89	81.05	355.6	326.4	277.3	123.6	112.2	89.91	106	79.4	134	18
Os01g0813900	1106	1329	1210	810.3	825.3	753.5	1282	1340	1347	1232	1329	1091	1197.4	1159.3	1379.6	1731	1166	1156	738.5	775.6	744.1	1259	1252	1266	1301	1293	1239	1308	1222	1390	13
Os01g0312500	0	0	0	0	0	0	0	0	0	0	0	0	0	1.8669	0	0	0	0	0	0	0	0	0	1.444	0	0	0	0	0	0	9
Os01g0616500	239	214	179	339.9	372.4	387.5	301	308	346	200.9	188.6	175.9	232.94	261.37	211.45	429.9	178.3	206.2	308.4	320.9	330.2	307.6	318.5	296.8	183.8	171.6	195.2	203	194	182	12
Os01g0224000	1.02	3.73	6.47	4.926	4.52	4.325	8.61	6.96	13.8	6.897	8.771	4.704	2.177	0.9335	0.792	0	3.092	0	3.716	4.031	3.449	7.394	7.126	13	5.282	1.886	4.386	4.63	3.83	1.67	18
Os01g0895100	4056	4418	4706	4095	4250	3907	4864	5252	5322	5215	5596	5660	6102.2	5347.7	5204.8	6878	4373	4264	4002	3858	4145	3608	3451	4351	5096	4851	4618	3842	4191	4398	13
Os01g0717700	897	1357	1121	2570	3135	2116	1838	1532	1279	1285	1352	824.2	1457.5	1109.9	1325	1934	1177	1001	2672	2857	2061	1173	1467	1372	1008	987.2	1110	1447	1413	1439	10
Os01g0773600	485	487	502	557.4	591.2	602.9	608	602	575	469	501.1	417.7	448.47	435.92	503.69	1015	477.1	480.7	555.5	589.4	543.2	532.4	488.2	455	471.1	422.4	410.1	476	527	490	6
Os01g0548600	26.5	17.7	25.9	36.12	18.08	32.87	13.3	16.4	65	18.97	15.35	8.467	20.682	15.869	10.296	47.76	15.46	16.58	30.66	17.74	27.59	41.41	79.1	51.28	28.52	22.63	17.54	28.7	23.9	19.2	17
Os01g0858500	354	373	372	386.7	368.8	365.9	492	434	548	485.4	537.2	435.6	616.1	501.26	551.21	716.5	452.4	395.8	419.9	449.1	453.5	394.8	403.4	417.5	489.1	494	460.5	454	440	456	6
Os01g0175600	0	2.79	0	2.463	1.808	3.46	5.48	3.8	8.98	10.35	4.386	5.645	4.3541	0.9335	0.792	0	0	3.108	8.361	8.869	6.036	8.133	7.126	5.778	0	0.943	6.579	0.93	0.96	0	15
Os01g0689451	1408	1463	1399	1174	1257	1082	1242	1375	1329	1214	1362	1228	1479.3	1271.4	1625.9	2579	1577	1515	855.6	1002	900.2	1358	1421	1452	1500	1608	1403	1549	1442	1567	13
Os01g0931100	911	1036	891	1236	1380	981.8	431	567	440	621.6	664.4	559.8	571.47	614.21	639.12	1337	846.1	721.1	696.7	693.4	594.9	547.1	463.9	537.4	665.5	693.9	888.1	810	760	799	8
Os01g0897200	713	974	791	853	984.4	632.4	1617	1956	1621	915.6	972.5	964.3	754.34	718.76	895.71	752.3	735.8	793.6	767.3	725.7	668.2	1158	1171	1356	841.9	734.5	815.8	641	696	711	18
Os01g0861000	54	37.3	53.9	53.36	50.62	50.17	64.2	53.8	79.5	50.87	54.82	64.92	41.364	49.473	53.854	107.5	35.04	45.59	55.74	64.5	50.01	67.28	54.87	49.83	48.59	48.08	50.44	55.5	35.4	34.2	6
Os01g0559100	172	130	108	151.9	160.9	178.2	222	189	256	145.7	175.4	191.9	131.71	183.89	149.68	95.53	171.1	162.7	227.6	237	231.1	174.5	182.4	203	144.7	155.6	188.6	130	115	121	15
Os01g0674100	220	246	228	265.2	250.4	283.7	103	130	113	152.6	174.3	71.5	192.67	153.09	205.91	370.2	263.8	231	139.3	162.9	136.2	239.6	295	216	218.7	156.5	151.3	290	241	281	8
Os01g0588800	477	537	481	495.9	599.3	541.5	440	514	506	499.2	573.4	541	619.37	572.21	601.89	907.5	524.5	492.1	497.9	533.8	558.7	530.9	506.7	476	464.8	505.4	561.4	513	529	525	6
Os01g0642100	289	148	262	6.568	15.37	18.17	6.26	12.7	9.67	240.6	277.4	316.1	480.04	407.92	509.23	692.6	457.6	358.5	25.08	21.77	13.8	2.218	12.11	10.11	325.4	357.3	247.8	368	315	357	13
Os01g0803800	8.16	8.38	4.31	0.821	0	0	0.78	0.63	0	6.035	3.289	9.408	1.0885	6.5342	1.5839	0	1.031	1.036	3.716	1.613	0.862	2.218	0	2.889	1.056	1.886	0	3.7	2.87	0.83	20
Os01g0640800	439	501	466	789	806.3	825.3	546	508	528	562.1	530.7	682.1	630.25	626.35	563.88	656.8	485.4	502.5	954	980.4	1054	434	409.1	544.6	589.5	548.7	502.2	469	483	488	10
Os01g0888600	147	169	173	89.49	104	126.3	143	116	138	277.6	193	266.3	163.28	222.16	231.25	406	260.7	258	163.5	140.3	246.6	180.4	215.9	168.3	292.6	314.9	258.8	277	202	213	13
Os01g0548750	1.02	0	3.23	1.642	0	0.865	0	0.63	1.38	0	0	0	0	0	0	0	0	2.072	0	1.613	1.724	2.218	6.414	3.611	1.056	0	0	0	0	2.5	17
Os01g0339851	0	0.93	0	0	0	0	2.35	3.16	0	0	1.096	0	1.0885	0.9335	0	0	1.031	2.072	1.858	0.806	0	0	0	0	1.056	0	0	0	0	0	5
Os01g0934200	516	467	506	205.2	199.8	229.2	215	238	274	403.5	464.9	580.5	537.73	500.33	544.08	1146	562.7	558.4	257.3	269.3	256.1	326.1	286.5	252.1	512.3	583.6	629.4	570	530	527	13
Os01g0726100	0	2.79	0	0.821	0	0	0.78	0	0	0	0	0	0	0	0	0	1.031	1.036	0	0.806	0	0	2.138	0	2.113	0	0	0	0	0.83	14
Os01g0571200	159	177	147	196.2	207.9	198.1	282	265	290	250	225.9	272.8	176.34	207.23	190.86	191.1	138.1	145	243.4	227.4	236.2	163.4	173.2	158.2	177.5	198	208.3	164	128	158	15
Os01g0682700	0	2.79	2.16	0.821	0	1.73	0.78	2.53	1.38	0	0	0	2.177	3.7338	0.792	0	2.061	0	0	0.806	0.862	1.479	0	0	2.113	0	2.193	0	0	0	4
Os01g0224500	331	320	341	394.9	413.1	517.3	573	479	558	381.1	414.4	459.1	524.67	439.66	435.58	406	345.2	407.2	547.2	549.9	532.9	394.1	388.4	395.1	361.3	462	475.9	377	390	382	15
Os01g0239100	345	251	370	196.2	166.3	197.2	137	152	144	339.7	425.4	452.5	473.51	411.65	453	429.9	299.9	319.1	296.3	261.2	296.6	82.07	89.79	85.22	328.5	403.5	416.7	321	322	320	3
Os01g0279100	####	####	####	7842	7063	6863	5057	5470	5431	42036	44331	58286	35776	35787	38499	53507	28300	32020	11540	9761	11519	4924	3592	5403	42433	44823	34120	####	####	####	3
Os01g0361500	2406	2158	2705	1085	1003	1157	933	1608	1288	2509	2873	2725	3476.7	2938.5	3251.8	5123	3455	2985	1332	1486	1090	1179	1283	1203	3175	3185	2789	3231	3508	3452	13
Os01g0155300	0	0	0	0	0	0	0	0	0	0	0	0	0	0	0	0	0	0	0	0	0	0	0	0	0	0	2.193	0	0	0	11
Os01g0844500	98.9	61.5	46.3	3.284	8.136	14.71	25.8	1.27	15.2	49.14	53.72	58.33	22.859	24.27	22.175	59.7	52.56	63.2	13.93	9.675	17.24	8.133	5.701	17.33	44.37	41.49	57.02	40.7	27.7	30.8	3
Os01g0719150	1.02	0	1.08	2.463	0	0	0	0	0	0	0	0	0	0	0	0	1.031	2.072	0	0	0	0	0	0	0	0	2.193	0.93	4.78	0	1
Os01g0758900	4.08	8.38	11.9	2.463	0	0	2.35	0	0	0	5.482	0.941	0	0	0	0	2.061	5.18	0	0	1.724	1.479	1.425	0.722	1.056	0.943	0	3.7	0	3.33	14
Os01g0502700	5.1	4.66	2.16	13.14	2.712	5.19	11	12.7	16.6	8.622	8.771	4.704	6.5311	4.6673	5.5438	11.94	7.214	5.18	9.29	14.51	8.622	22.18	17.82	25.28	4.225	2.829	0	2.78	5.74	7.5	9
Os01g0806000	5.1	14.9	11.9	10.67	13.56	13.84	7.83	3.16	5.53	1.724	12.06	0	8.7082	9.3345	0.792	11.94	4.122	3.108	1.858	5.644	15.52	4.436	5.701	16.61	10.56	3.771	6.579	6.48	8.61	1.67	2
Os01g0165000	229	182	222	153.5	132	190.3	171	172	206	158.6	146.9	134.5	197.02	159.62	173.44	226.9	150.5	188.6	199.7	176.6	169	177.5	225.9	156	143.7	142.4	175.4	154	165	137	14
Os01g0191500	1364	1278	1148	963.8	1010	1031	1064	1003	1108	1135	1088	1348	1041.7	1254.6	968.57	1815	1204	1142	1131	901.4	1045	1114	1073	1071	1083	1256	1423	1082	1062	1031	13
Os01g0497400	0	0	0	0	0	2.595	0	0.63	0.69	2.587	0	0	0	0	0	0	0	0	0	0	0	0	0	0	0	0	0	0	0	0	7
Os01g0611000	3.06	1.86	0	0	0	6.055	3.91	0	2.07	0	0	0	1.0885	0.9335	0.792	0	1.031	0	0.929	0	0	4.436	2.851	10.11	0	0	0	1.85	0	0.83	9
Os01g0708500	38.7	29.8	30.2	132.2	143.7	107.3	72	64.5	113	27.59	16.45	15.99	14.151	28.937	38.806	59.7	16.49	25.9	72.46	83.05	109.5	82.07	76.96	62.84	19.01	13.2	4.386	14.8	22	15	12
Os01g0786900	1259	1393	1355	648.6	639.1	630.6	610	801	655	1120	1322	827.9	1448.8	1201.4	1465.1	2078	1389	1467	573.2	669.2	579.4	779.3	841.6	904.3	1278	1313	997.8	1519	1531	1662	13
Os01g0184000	138	145	135	179	201.6	216.3	206	219	196	127.6	119.5	147.7	154.57	169.89	139.39	322.4	137.1	160.6	217.4	212.1	246.6	219.6	187.4	169.7	127.8	162.2	133.8	118	153	124	6
Os01g0546000	1.02	0	2.16	0	0	0	0	0	0	0	0	0	0	0	0	0	0	0	0	0	0	0	0	0	3.169	0	2.193	0	2.87	1.67	1
Os01g0524100	0	12.1	5.39	7.389	1.808	9.516	2.35	0.63	0.69	1.724	0	0	0	1.8669	0.792	11.94	3.092	4.144	0	2.419	2.587	5.915	4.988	4.333	3.169	0.943	4.386	7.4	8.61	0.83	8
Os01g0907200	1.02	0	0	0.821	0	0	0.78	0	2.07	0	0	2.822	0	0.9335	0	0	1.031	2.072	0	0	0	8.873	2.138	4.333	0	0	0	0	0	0	9
Os01g0752700	1064	1002	1005	1459	1444	1542	874	944	927	843.2	887	894.7	1287.7	1166.8	1080.2	1719	998.6	993.6	1301	1311	1461	970.8	919.3	908.6	928.5	1002	857.4	968	1015	896	10
Os01g0965600	261	279	279	244.7	259.4	228.4	511	433	429	375.9	356.3	398.9	296.08	350.04	338.17	764.2	303	302.5	242.5	274.1	221.6	331.2	334.2	374.1	331.7	337.5	315.8	340	318	331	6
Os01g0254800	217	181	213	163.4	149.2	157.4	77.5	137	115	168.1	137	124.2	183.96	124.15	150.47	262.7	197.9	204.1	116.1	144.3	124.2	152.3	147.5	117	159.5	143.3	114	204	226	207	8
Os01g0854000	386	624	375	610.8	673.5	555.4	274	159	348	613.9	890.3	634.1	741.28	793.43	860.08	788.1	1051	776	624.3	557.1	644.9	972.3	797.4	694.8	1386	884.4	899.1	706	417	957	1
Os01g0191300	201	211	195	192.9	150.1	214.5	62.6	90.4	76	129.3	159	139.2	117.56	152.15	166.31	358.2	240.1	197.9	138.4	179.8	202.6	197.4	262.2	210.9	266.2	245.1	291.7	293	223	227	13
Os01g0675700	1365	1452	1279	1114	1139	1083	916	885	864	1636	1535	2047	1546.8	1734.4	1534.8	2090	1636	1409	1683	1443	1834	626.3	653.5	888.4	1635	1816	1912	1291	1301	1330	3
Os01g0753200	330	374	333	499.2	554.1	480.1	644	713	713	381.9	399.1	429	309.14	338.84	333.42	453.8	301.9	323.2	470.1	541.8	455.3	698	657.8	699.9	394	380.9	421	295	289	278	18
Os01g0516700	2.04	0.93	2.16	0	0.904	0.865	0	0	0	0	0	0	0	0	1.5839	0	3.092	0	0	0	0	0.739	3.563	0	0	2.829	6.579	4.63	0.96	1.67	11
Os01g0939400	331	290	324	328.4	289.3	279.4	305	331	328	387.1	362.9	431.8	429.97	425.65	384.1	668.7	343.2	304.6	379	366.9	407.8	261.7	273.7	310.6	450	396	421	369	380	337	13
Os01g0889900	145	107	90.5	182.3	101.2	172.1	61.8	39.8	56.7	84.49	85.52	104.4	44.629	94.279	55.438	286.6	84.5	112.9	100.3	178.2	150	150.8	163.2	180.6	118.3	159.3	153.5	141	114	107	6
Os01g0105700	28.5	24.2	36.6	9.852	16.27	10.38	3.91	9.49	5.53	36.21	52.63	54.57	46.806	36.405	61.773	35.82	28.85	18.65	9.29	21.77	18.97	14.05	10.69	12.28	38.03	27.34	19.74	36.1	35.4	35	3
Os01g0246200	2.04	2.79	2.16	0	0	0	0	0	0	2.587	3.289	0.941	4.3541	0.9335	0.792	0	0	1.036	1.858	0	0	0	0	0	3.169	1.886	0	0	0.96	3.33	3
Os01g0379400	241	264	239	266.8	297.4	206.7	191	201	187	203.5	217.1	198.5	217.7	203.49	228.09	549.3	219.5	220.7	251.7	266.9	289.7	157.5	145.4	184.9	174.3	175.4	155.7	234	207	242	6
Os01g0766200	615	493	559	156.8	197.1	173	250	226	179	433.7	419.9	388.6	452.82	471.39	443.5	1027	490.5	560.5	275	244.3	251.8	200.4	234.5	189.2	475.4	406.4	508.8	559	511	526	13
Os01g0113700	1.02	0	1.08	1.642	0	0	3.91	0	0.69	0	3.289	1.882	0	0.9335	2.3759	0	0	0	1.858	5.644	0.862	0	1.425	2.167	1.056	0.943	0	1.85	3.83	2.5	16
Os01g0864401	217	272	244	302.1	349.8	273.4	251	288	237	275.9	213.8	213.6	171.99	172.69	154.43	406	229.8	290.1	281.5	316.1	317.3	254.3	225.2	256.4	192.3	210.3	203.9	192	248	236	6
Os01g0103050	0	0	0	2.463	0.904	1.73	0	0	0	0	0	0	0	0	0	0	0	0	0	0.806	0	0	0	0	0	0	0	0	0	0	2
Os01g0828100	1054	1004	1082	601	519.8	766.4	610	593	693	1665	1395	1417	1394.4	1385.2	1275.9	2806	1178	1106	886.2	715.2	945.9	624	499.6	661.6	1401	1433	1351	1154	1231	1244	13

Os01g0851000	163	169	151	325.1	418.5	288.1	153	190	164	168.1	177.6	131.7	198.11	122.28	122.75	155.2	76.26	122.3	152.3	167.7	165.5	125.7	148.2	163.2	133.1	115	85.52	53.7	67.9	104	2
Os01g0329900	16.3	11.2	20.5	4.105	8.136	12.11	21.9	6.96	20.7	25.87	8.771	15.05	10.885	11.201	19.799	11.94	32.98	18.65	0.929	1.613	12.93	39.93	37.06	52.72	4.225	24.51	41.67	25.9	28.7	14.2	9
Os01g0168500	300	315	333	261.1	311	294.1	344	352	267	336.3	339.9	257.8	324.38	320.17	353.22	501.5	311.2	268.3	293.5	275.7	287.1	322.4	326.4	325.7	370.8	320.6	339.9	387	348	373	13
Os01g0813300	0	0	0	0	0	0	0	0	0	0	0	0	0	0	0	0	0	0	0.929	0	0	0	0	0	0	0	0	0	0	0	16
Os01g0228400	292	280	361	44.33	45.2	37.2	171	216	187	206.1	294.9	334	400.58	378.05	376.18	931.4	401.9	355.4	52.02	52.41	37.08	149.4	156.1	122.1	343.3	387.5	333.3	377	326	307	13
Os01g0712800	0	1.86	0	1.642	0	0	0	0	0	0	0	0	0	0	0	0	0	0	0	1.613	0	0	0.713	0	0	0	0	0	0	0	2
Os01g0663300	47.9	42.8	57.1	17.24	23.5	20.76	7.05	0	0	25.87	10.96	8.467	25.036	42.005	20.591	119.4	79.35	49.73	45.52	40.31	40.52	3.697	5.701	4.333	26.41	5.657	2.193	22.2	18.2	21.7	13
Os01g0535100	0	0	0	0.821	0	0	0	0	0	0	0	0	0	0	0	0	0	0	0	0	0	0	0	0	0	0	0	0	0	0	2
Os01g0971600	28.5	35.4	20.5	138.7	145.5	180.8	145	142	102	10.35	30.7	18.82	29.39	25.203	15.047	83.59	18.55	26.94	137.5	161.3	169.9	140.5	98.34	135.1	24.3	28.29	10.96	14.8	33.5	18.3	12
Os01g0587300	8.16	16.8	5.39	29.56	22.6	16.44	19.6	11.4	12.4	4.311	0	3.763	6.5311	8.4011	7.1277	23.88	18.55	18.65	27.87	22.58	28.45	12.57	21.38	16.61	7.395	11.31	0	6.48	11.5	15.8	10
Os01g0753800	0	0	0	0	0	0	0	0	0	0.862	0	0	0	0	0	0	0	0	3.716	0	0	0	2.138	0	0	0	0	0	0	0	16
Os01g0495701	5.1	8.38	2.16	7.389	2.712	1.73	3.91	2.53	2.76	6.897	18.64	3.763	8.7082	9.3345	4.7518	0	3.092	3.108	1.858	0.806	0	2.218	1.425	0.722	2.113	8.486	19.74	2.78	7.65	8.33	11
Os01g0927000	59.1	67.1	38.8	68.14	67.8	98.62	39.9	44.9	34.6	33.63	33.99	58.33	37.01	39.205	35.638	143.3	43.28	64.23	52.02	55.63	74.15	49.54	57.01	44.06	34.86	23.57	54.82	48.1	46.9	37.5	6
Os01g0253600	261	264	232	167.5	205.2	162.6	264	248	240	268.1	255.5	244.6	198.11	226.83	254.22	214.9	170	181.3	215.5	195.1	226.8	230.7	220.2	244.1	244	226.3	293.9	195	193	191	5
Os01g0798800	34.7	41.9	42	41.05	37.06	42.39	83.8	63.9	54.6	100	65.78	65.86	55.515	50.406	56.23	59.7	32.98	41.44	30.66	45.96	25	63.59	69.84	62.11	40.14	37.71	41.67	28.7	52.6	30.8	5
Os01g0507300	587	571	571	580.4	640.9	579.6	529	572	569	578.5	586.6	604.9	544.26	580.61	599.52	1122	575	527.3	654.9	702.3	713.9	471	498.1	424.7	546.1	560	589.9	612	640	597	6
Os01g0910800	1.02	0	0	3.284	3.616	0	0	0	0	0	0	0	0	0	0	0	0	0	0	0	0	0	0	0	0	0	0	0.93	0	0	2
Os01g0151500	54	47.5	40.9	66.5	56.95	70.93	26.6	12.7	13.1	56.9	44.95	72.44	26.124	33.604	34.847	107.5	65.95	51.8	86.39	34.67	62.94	8.873	21.38	33.95	30.63	47.14	35.09	43.5	39.2	40	16
Os01g0113800	40.8	60.5	35.6	49.26	53.33	57.96	53.2	39.8	43.5	30.18	30.7	15.05	25.036	29.87	34.055	0	36.07	31.08	26.01	37.9	35.35	63.59	80.53	57.78	26.41	33	10.96	52.7	56.4	56.7	17
Os01g0924900	1137	1124	1216	1277	1453	1228	1092	1137	945	994.1	918.8	780.9	1070	1091.2	1069.9	1218	1269	1157	1141	1123	1183	1006	1128	982.3	1027	1031	1044	1201	1019	1242	8
Os01g0876500	455	554	490	674	574	707.6	496	361	383	439.7	379.4	434.7	397.31	430.32	395.19	561.2	428.7	500.4	609.4	623.3	619.1	636.6	450.4	490.4	374	408.3	489	515	454	488	10
Os01g0191800	0	0.93	3.23	0	0.904	1.73	0	3.8	1.38	0.862	1.096	0.941	4.3541	0	0.792	0	1.031	1.036	0.929	0	0.862	1.479	0	0	1.056	1.886	2.193	0	0	2.5	4
Os01g0218500	10.2	15.8	10.8	8.21	6.328	7.785	3.13	1.9	16.6	12.07	10.96	14.11	14.151	4.6673	3.9598	23.88	21.64	42.48	10.22	9.675	17.24	13.31	4.276	6.5	20.07	29.23	26.32	13.9	23.9	19.2	13
Os01g0200700	####	####	####	#####	#####	#####	####	####	####	#####	#####	#####	364719	452765	418506	#####	#####	#####	#####	#####	#####	74697	70805	80951	#####	#####	#####	####	####	####	13
Os01g0111300	32.6	28.9	37.7	29.56	20.79	27.68	53.2	47.4	37.3	50.87	47.14	36.69	23.947	23.336	34.055	71.65	48.43	44.55	36.23	37.09	30.18	52.5	64.85	59.95	43.31	46.2	59.21	50	32.5	44.2	6
Os01g0844000	118	99.7	95.9	101	99.44	142.7	336	292	301	125	128.3	206	186.14	111.08	129.88	83.59	168	143	179.3	212.1	233.7	150.1	134.7	281.7	147.9	165	151.3	110	91.8	90.8	15
Os01g0179200	356	284	336	325.9	350.7	368.5	431	433	379	372.5	359.6	353.7	348.33	337.91	308.57	525.4	341.1	374	357.6	380.6	332.8	336.4	373.4	340.9	282	314.9	372.8	301	311	344	6
Os01g0879300	3.06	4.66	2.16	2.463	1.808	1.73	0.78	3.16	0	0	1.096	1.882	2.177	1.8669	1.5839	0	4.122	1.036	1.858	3.225	6.898	4.436	5.701	2.889	3.169	3.771	2.193	3.7	0	4.17	1
Os01g0169900	539	597	614	588.6	555	557.1	814	744	741	591.5	599.7	627.5	621.54	612.34	624.86	823.9	520.4	566.7	587.1	530.5	613.9	607.8	592.9	690.5	591.6	568.5	633.8	604	575	561	18
Os01g0947833	6.12	0.93	5.39	1.642	1.808	7.785	3.91	1.9	11.7	1.724	4.386	2.822	4.3541	2.8004	7.9197	0	0	2.072	0.929	0.806	3.449	2.218	4.276	10.83	1.056	0	0	0.93	4.78	0	9
Os01g0107400	4.08	7.45	3.23	80.46	78.65	83.91	131	138	128	14.66	6.578	15.05	6.5311	6.5342	7.9197	0	4.122	11.4	93.82	83.85	118.1	145.7	144.7	157.4	8.451	9.428	4.386	1.85	2.87	5.83	12
Os01g0704700	1159	1291	1284	1271	1404	1272	1242	1657	1428	1298	1498	1277	1449.9	1381.5	1459.6	2818	1258	1263	1179	1460	1396	1253	1216	1210	1294	1260	1011	1242	1314	1415	6
Os01g0667800	215	163	166	142.8	196.2	155.7	118	161	140	145.7	142.5	142.1	168.72	154.95	163.94	298.5	214.4	250.7	165.4	197.5	163	135.3	161.8	153.8	184.9	216.9	175.4	228	194	167	13
Os01g0300900	1.02	2.79	3.23	0	1.808	1.73	3.13	0.63	4.15	13.79	8.771	0	0	1.8669	1.5839	0	3.092	2.072	0	0	0.862	4.436	4.988	12.28	2.113	4.714	0	6.48	2.87	2.5	7
Os01g0278950	10.2	3.73	3.23	4.105	2.712	3.46	5.48	0.63	0.69	0.862	0	5.645	1.0885	0.9335	1.5839	11.94	6.183	3.108	3.716	2.419	0.862	2.218	0	2.889	0	1.886	0	5.55	1.91	5.83	6
Os01g0175500	32.6	57.7	40.9	66.5	65.09	45.85	112	94.9	140	51.73	57.01	80.91	38.098	49.473	45.142	23.88	32.98	40.41	42.73	45.96	37.94	93.9	76.25	91	30.63	29.23	61.4	28.7	23	31.7	18
Os01g0138800	40.8	61.5	57.1	19.7	18.98	19.9	3.91	13.9	1.38	22.42	14.25	19.76	13.062	30.804	9.5036	47.76	58.74	60.09	31.58	23.38	40.52	1.479	1.425	2.889	23.24	16.97	17.54	37.9	28.7	16.7	14
Os01g0918100	228	294	241	267.6	215.1	274.2	572	408	483	179.3	205	241.8	166.54	209.09	217	394.1	168	204.1	166.3	216.1	199.2	456.9	470.3	523.6	231.3	267.8	396.9	302	223	239	18
Os01g0229700	36.7	29.8	29.1	17.24	10.85	10.38	15.7	17.7	15.2	36.21	28.51	36.69	26.124	34.538	22.175	47.76	30.92	36.26	21.37	17.74	22.42	29.58	24.94	28.17	41.2	36.77	41.67	22.2	20.1	25	13
Os01g0147001	0	0	0	0.821	0.904	0	0.78	0	0	0	0	0	1.0885	0	0	0	0	0	0	0	0	0	0	0.722	0	0	0	0	0	0	2
Os01g0220700	191	173	109	19.7	13.56	23.36	5.48	10.8	18	43.97	57.01	119.5	39.187	114.81	44.35	47.76	111.3	163.7	13.01	12.09	4.311	17.01	10.69	23.83	45.42	130.1	217.1	94.4	131	117	3
Os01g0302000	570	333	294	233.2	281.1	359	75.9	94.2	93.3	303.5	293.8	387.6	225.32	423.79	192.45	298.5	194.8	378.2	124.5	96.75	46.56	93.9	71.26	70.06	202.8	225.3	392.5	146	121	199	19
Os01g0871500	109	204	150	849.7	918.4	937.7	674	631	547	272.5	288.4	460.1	343.97	249.23	319.16	155.2	192.7	200	841.6	851.4	867.4	437	400.5	516.4	247.2	366.8	368.4	247	185	282	10
Os01g0157900	785	593	706	413.8	433	473.2	538	600	511	716.5	513.1	672.7	596.51	604.88	553.58	740.3	584.3	607.1	487.7	456.4	493.2	406.7	535.9	421.1	559.9	495	508.8	428	469	406	14
Os01g0236400	43.8	16.8	28	19.7	20.79	35.47	14.1	15.8	15.9	20.69	28.51	37.63	20.682	28.937	28.511	59.7	39.16	46.62	22.29	36.28	55.18	21.44	24.23	19.5	24.3	17.91	21.93	47.2	29.6	21.7	13
Os01g0360600	707	788	806	1070	1225	1074	1344	1397	1321	697.5	827.8	825.1	965.52	846.64	860.08	1648	807.9	773.9	1070	1034	1042	1192	1043	1158	804.9	717.5	638.1	708	736	822	12
Os01g0178900	76.5	95	92.7	119	101.2	74.39	198	234	230	164.7	137	65.86	103.41	51.34	106.92	143.3	62.86	66.31	86.39	91.92	60.36	287.6	337.1	252.1	103.5	65.06	35.09	105	131	100	17
Os01g0327100	0	0	0	1.642	0	0	0	11.4	0.69	0	0	0.941	0	0	0.792	0	0	0	0	0	0	0	7.126	0	0	0.943	0	0	0	0	17
Os01g0823100	20.4	9.31	0	6.568	0	8.65	0.78	1.27	8.29	9.484	5.482	7.527	1.0885	6.5342	0.792	0	0	3.108	1.858	0	0	8.133	4.988	13.72	5.282	1.886	0	5.55	5.74	3.33	19
Os01g0774500	0	0	0	0	0	0	3.13	0	0	0	0	0.941	0	0	0	0	0	0	0	0	0	1.479	0.713	1.444	0	0	0	0	0	0	9
Os01g0226200	0	0.93	0	1.642	0	0	0	0.63	0	0	0	0	0	0	0	0	0	0	0	0	0	0	0	0.722	1.056	0	0	0	0	0	2
Os01g0341300	0	0.93	1.08	0	0.904	0	0	0	0	0.862	0	0.941	0	0	0	0	0	0	0	0	0	0	0	0	1.056	0	0	0	0	0	7
Os01g0834100	2.04	4.66	3.23	17.24	19.89	19.03	16.4	10.8	8.29	13.79	20.83	2.822	6.5311	12.135	8.7116	11.94	1.031	0	10.22	16.93	17.24	11.83	7.839	10.83	4.225	6.6	6.579	3.7	4.78	4.17	10
Os01g0884800	11.2	11.2	6.47	10.67	14.46	6.92	4.7	9.49	3.46	10.35	10.96	10.35	10.885	9.3345	13.463	0	9.275	8.288	7.432	3.225	7.76	0	6.414	1.444	8.451	1.886	6.579	6.48	7.65	6.66	4
Os01g0588600	0	0	0	0	0	0.865	3.13	0	0.69	0	0	0	0	0.9335	0	0	0	0	0	0	0.862	0	0	0	0	0	0	0	0	0	15
Os01g0662700	256	236	227	623.9	791	598.6	1060	1249	1126	197.4	222.6	302	176.34	200.69	174.23	286.6	301.9	288	682.8	878	853.6	818.5	739.7	1125	252.5	267.8	241.2	191	187	192	12
Os01g0927500	94.8	71.7	66.8	57.47	62.37	58.82	58.7	70.2	65	31.9	58.11	84.67	37.01	66.275	29.303	83.59	62.86	73.56	60.38	54.02	50.87	56.93	44.18	53.45	57.04	74.48	78.95	67.5	79.4	69.1	19
Os01g0339950	0	0	0	0	0	0	0.78	0	0	0	0	0	0	0	0	0	0	0	0	0	0	0	0	0	0	0	0	0	0	0	5
Os01g0672600	1.02	3.73	1.08	8.21	9.04	0.865	0	5.69	8.29	0	5.482	0	0	0	7.1277	35.82	12.37	5.18	5.574	0	0	0	6.414	8.667	0	8.486	0	5.55	0	11.7	6
Os01g0547600	401	342	380	33.66	50.62	57.09	0	1.27	0	433.7	509.8	492	210.08	357.51	315.2	573.2	660.6	481.8	148.6	162.9	131.9	0	4.988	0.722	563	678.8	982.4	728	628	552	3
Os01g0714100	239	266	293	301.3	278.4	309.7	296	367	319	330.2	361.8	273.8	380.98	279.1	342.92	752.3	304	291.1	293.5	303.2	282.8	307.6	292.9	340.9	310.6	337.5	331.1	304	299	294	6
Os01g0527900	1.02	0.93	0	0.821	0	1.73	0	0	0	1.724	0	0	1.0885	4.6673	0	0	2.061	2.072	3.716	0.806	0	0	0.713	0	0	0	0	16.7	0	2.5	1
Os01g0570800	2879	3335	2260	391.6	302.8	368.5	481	503	581	523.3	490.1	427.1	520.31	391.12	573.38	1970	2179	1627	305.6	388.6	475.9	1060	819.5	1379	474.3	540.2	500	612	533	554	14
Os01g0700000	1150	1351	1223	253.7	213.3	280.3	465	452	641	1517	1679	1164	1393.3	1172.4	1494.4	2627	1109	1008	306.6	274.1	201.8	514.6	581.5	530.1	1555	1501	1561	1551	1589	1505	13
Os01g0780900	2878	2611	2912	2699	2771	2599	2176	1729	1843	4005	3510	3124	3835.9	3254.9	3571.8	3260	2283	2486	1904	2052	2070	1270	1642	1293	2876	2407	2513	2697	2495	2598	3
Os01g0951200	339	333	312	1300	1412	1305	820	638	713	290.6	270.8	303.9	280.84	392.98	266.1	537.3	290.6	350.2	1319	1125	1350	570.1	647.8	567	213.4	208.4	287.3	284	300	241	10
Os01g0931400	158	138	173	220.8	266.7	222.3	166	147	153	189.7	189.7	174.1	176.34	148.42	165.52	238.8	129.8	115	228.5	275.7	269	157.5	136.1	170.4	148.9	184.8	201.7	119	118	130	10
Os01g0363600	17.3	19.6	28	18.06	17.18	10.38	29	44.9	29.7	23.28	28.51	29.17	37.01	30.804	35.638	11.94	24.74	39.37	9.29	20.16	18.97	29.58	29.93	33.95	20.07	36.77	21.93	19.4	12.4	26.7	5
Os01g0197500	46.9	42.8	55	30.38	24.41	31.14	32.9	27.2	23.5	48.28	42.76	51.75	51.16	55.074	47.518	155.2	51.53	49.73	35.3	29.83	43.11	46.58	41.33	33.22	85.57	73.54	48.24	70.3	56.4	65	13
Os01g0764400	1291	1159	1402	399.8	392.3	378.9	597	764	684	1631	1375	1413	1323.6	1684.9	1523.7	1887	1571	1433	521.1	441	448.4	523.5	903.6	652.2	1427	1434	1570	1357	1246	1281	3
Os01g0871100	136	128	154	62.39	89.49	88.23	59.5	62	45.6	106	125	71.5	142.6	176.42	133.05	394.1	156.6	201	76.17	64.5	81.91	64.33	83.38	63.56	156.3	131.1	149.1	178	169	197	13
Os01g0182832	45.9	68	47.4	50.08	56.95	54.5	10.2	12.7	10.4	18.11	20.83	11.29	30.479	21.469	28.511	47.76	28.85	44.55	13.01	26.61	12.07	39.19	32.78	18.06	21.13	18.86	8.772	42.6	29.6	52.5	8
Os01g0944000	2119	2330	2389	1325	1483	1433	1479	1818	1665	1380	1690	1251	1579.4	1493.5	1918.9	4012	2050	2284	874.1	1257	1086	2998	2241	1934	1843	1845	1432	2070	2072	2484	13
Os01g0234700	6.12	0.93	6.47	4.926	1.808	6.055	10.2	5.06	2.76	3.449	6.578	4.704	3.2656	3.7338	2.3759	0	2.061	9.324	7.432	1.613	0.862	2.218	2.851	1.444	2.113	7.543	4.386	0.93	2.87	4.17	19
Os01g0915000	5.1	2.79	0	4.105	2.712	0.865	0	2.53	1.38	0	0	0.941	1.0885	0.9335	1.5839	0	0	0	1.858	3.225	9.484	2.958	3.563	2.889	4.225	0	0	0	1.91	0	16
Os01g0967200	1.02	1.86	0	0	2.712	3.46	0	0	1.38	0	0	1.882	3.2656	0	0	0	2.061	1.036	0	0	0	1.479	2.138	0	0	2.829	0	1.85	7.65	0	1
Os01g0781000	260	227	254	208.5	188.9	229.2	442	377	444	263	248.9	295.4	269.95	252.97	282.73	525.4	294.7	281.8	242.5	263.7	300.9	277.3	270.8	345.2	279.9	298.9	245.6	200	210	235	6
Os01g0874700	289	245	294	220	246.8	179.9	183	249	227	153.5	145.8	181.6	118.65	112.95	166.31	274.6	314.3	213.4	221.1	183	167.3	208.5	225.9	213.1	132	137.7	162.3	154	124	169	14
Os01g0830900	26.5	15.8	14	0	0	0	1.57	0	0	28.45	19.74	66.8	7.6196	3.7338	0.792	11.94	18.55	10.36	0	0	0	0	0	0	8.451	31.11	32.89	0.93	5.74	3.33	20
Os01g0223000	117	98.7	74.4	114.1	72.32	99.48	148	104	97.4	94.84	100.9	105.4	81.639	140.02	162.35	131.4	114.4	100.5	87.32	135.5	106.1	199.6	212.4	179.8	147.9	189.5	188.6	195	115	140	9
Os01g0267200	976	917	1020	1071	1087	1153	1576	1345	1418	1339	1163	1217	1138.6	1179.9	1121.4	1576	973.9	941.8	1264	1082	1073	1019	1159	1071	1041	1038	1138	811	915	874	6
Os01g0948400	359	297	302	545.9	546.9	490.5	235	220	293	336.3	245.6	398.9	280.84	369.65	306.49	477.6	303	347.1	590.8	458	507	216.6	248.7	229	321.1	340.4	322.4	250	243	259	10
Os01g0766300	21.4	20.5	18.3	34.48	41.58	19.9	89.2	110	56	57.77	54.82	45.16	27.213	14.935	36.43	11.94	20.61	17.61	28.8	26.61	19.83	79.11	105.5	72.95	33.8	32.06	21.93	27.8	26.8	15.8	18
Os01g0228800	244	341	264	246.3	213.3	262.1	427	386	448	459.5	401.3	390.4	303.7	340.71	322.33	656.8	303	327.4	267.5	264.5	263.8	415.5	541.6	418.2	400.4	391.3	383.8	478	364	441	6
Os01g0681000	22.4	23.3	12.9	551.7	593.9	525.9	258	218	212	30.18	30.7	40.46	18.505	25.203	23.759	11.94	16.49	16.58	399.4	446.7	457	191.5	149.7	162.5	26.41	19.8	21.93	25	28.7	22.5	10
Os01g0196500	82.6	71.7	80.8	74.71	87.69	92.56	138	119	96.7	106	108.5	132.7	83.816	112.01	92.66	191.1	73.17	112.9	89.18	115.3	113.8	103.5	88.37	83.78	99.3	99	92.1	114	104	91.6	6
Os01g0316600	4782	3922	5305	499.2	442	602.9	246	209	258	3932	4765	3656	5598.3	5100.4	6242.3	9051	4691	4863	811.9	892.6	779.4	301.7	327.1	243.4	5097	4942	4432	5934	5548	5657	13
Os01g0200600	1.02	0.93	0	0.821	0	0	0	0	0	0	0	0	0	0.9335	1.5839	11.94	1.031	1.036	0.929	0	0.862	0	0	0	2.113	0	0	2.78	0	0.83	6
Os01g0950900	127	102	121	65.68	70.51	76.99	40.7	66.4	45.6	84.49	75.65	124.2	60.957	86.811	102.96	238.8	117.5	115	61.31	79.82	66.45	76.9	93.36	66.45	120.4	83.91	92.1	174	120	112	13
Os01g0967400	439	415	444	313.6	276.6	378	343	378	403	475.1	457.2	438.4	575.83	495.66	487.06	979.2	528.7	462.1	376.3	437	456.1	403.7	342.1	365.5	441.6	481.8	500	586	595	530	13
Os01g0857950	136	187	115	199.5	223.3	184.3	171	219	175	114.7	156.8	162.8	198.11	174.56	123.55	11.94	153.5	168.9	175.6	187.1	164.7	144.2	163.9	134.3	150	149	131.6	144	143	156	2
Os01g0933300	150	112	112	106.7	130.2	105.5	150	119	184	104.3	78.94	61.15	78.373	64.408	87.908	262.7	130.9	128.5	128.2	124.2	144	264	188.8	211.6	114.1	139.5	155.7	99.9	142	82.5	9
Os01g0830000	5140	4912	5412	1754	1617	1913	3258	3471	3415	6920	6730	6584	7126.5	7026.1	7409.6	8848	5802	5756	2497	2337	2414	2143	2383	2521	6673	6919	6193	5770	5953	6086	3
Os01g0218700	2079	2058	2104	1505	1567	1520	1973	2406	2123	2217	2353	2361	2757.2	2454	2845.5	4669	2225	2306	1484	1592	1518	1922	1934	1862	2692	2310	2072	2221	2326	2387	13
Os01g0525800	2.04	0	4.31	4.105	5.424	4.325	3.91	1.9	0.69	4.311	2.193	2.822	2.177	1.8669	0	0	2.061	3.108	0	0.806	0	0	1.425	1.444	1.056	2.829	2.193	0	0	4.17	2
Os01g0212500	1.02	0	0	1.642	0	0	2.35	0	0	0	0	0	0	0	1.5839	0	0	0	0	0	0	0	0	0.722	0	0	0	0	0.96	0	5
Os01g0914600	4.08	4.66	5.39	24.63	59.66	30.28	7.83	1.9	6.91	0	1.096	0	2.177	3.7338	3.9598	0	2.061	3.108	4.645	17.74	14.66	11.83	12.11	15.89	6.338	1.886	8.772	3.7	1.91	1.67	2
Os01g0887700	345	399	336	440.9	377	361.6	798	735	752	440.6	438.6	520.3	462.62	445.26	463.3	406	338	319.1	430.1	322.5	386.3	491	429	494.7	371.8	355.5	405.7	293	383	353	18
Os01g0197100	12.2	6.52	4.31	8.21	8.136	5.19	50.9	51.2	38	13.79	6.578	14.11	11.974	8.4011	11.088	35.82	18.55	8.288	27.87	20.16	11.21	35.49	28.51	34.67	15.85	4.714	21.93	7.4	11.5	14.2	18
Os01g0267400	57.1	87.6	61.4	92.77	89.49	100.3	179	167	124	81.05	71.27	59.27	67.488	84.944	82.364	119.4	76.26	66.31	93.82	105.6	147.4	130.1	106.2	124.9	78.17	64.11	59.21	85.1	73.6	75	18
Os01g0250200	0	0	0	0	0	0	0	0	0	0	0	0	0	0	0	0	0	2.072	0	0	0	0	0	0	0	0.943	0	0	0	0	1
Os01g0684400	0	0	0	0	0	0	0	0	0	0	0	0	0	0	0	0	0	0	0	0	0	0	0.713	0	0	0	0	0	0	0	17
Os01g0339900	68.3	76.4	77.6	125.6	166.3	138.4	65	71.5	104	43.97	54.82	37.63	57.692	71.876	54.646	143.3	62.86	49.73	80.82	63.7	73.29	105.7	95.49	83.06	47.54	27.34	48.24	61.1	46.9	72.5	2
Os01g0234100	184	203	224	534.5	578.5	490.5	105	121	78.8	213.8	212.7	126.1	237.3	213.76	245.51	561.2	229.8	195.8	375.3	424.1	419.9	159.7	171	124.9	263	207.4	171	325	260	322	10
Os01g0269550	20.4	25.1	29.1	15.6	6.328	12.98	7.83	5.06	9.67	17.24	21.93	22.58	27.213	12.135	13.463	47.76	47.4	26.94	13.93	7.257	8.622	28.1	17.82	27.45	28.52	36.77	39.47	38.9	33.5	46.7	13
Os01g0261500	335	383	337	419.5	429.4	403.1	679	707	578	331.1	427.6	411.1	374.45	385.52	407.86	465.7	425.6	446.5	430.1	417.7	480.3	540.5	548	541	436.3	471.4	491.2	426	364	392	18
Os01g0100200	3.06	0	0	0	0.904	0	0	1.9	0	0	0	0.941	1.0885	0	0	0	1.031	0	0.929	0.806	2.587	0	0	0.722	1.056	0	0	0.93	1.91	1.67	19
Os01g0794500	3251	2449	3037	960.5	963.6	1082	1668	1584	1726	2225	2193	1952	1909.3	2409.2	2182.7	2770	2766	2780	1510	1241	1568	1406	1572	1572	2083	1825	2480	1867	1758	1826	3
Os01g0264400	153	115	121	47.62	20.79	53.63	65.8	43.6	67.7	93.98	77.84	124.2	35.921	50.406	49.894	71.65	112.3	63.2	56.67	76.6	167.3	29.58	26.37	71.5	66.55	155.6	67.98	36.1	78.4	30.8	19
Os01g0590700	322	268	281	292.3	286.6	285.5	182	170	146	125.9	188.6	159	226.41	256.7	248.68	382.1	340.1	270.4	289.8	317.7	318.2	188.5	197.4	183.5	189.1	217.8	206.1	275	265	231	16
Os01g0201900	821	727	681	463	450.2	513.8	428	392	406	486.3	573.4	592.7	468.06	503.13	567.05	680.6	850.2	725.2	400.4	434.6	423.3	857.7	706.2	738.1	722.6	822.2	811.4	890	736	841	1
Os01g0547000	433	500	476	474.5	462.8	483.6	328	431	377	351.8	368.4	222	279.75	342.58	394.4	692.6	344.2	405.1	266.6	396.7	343.2	656.6	570.1	428.3	382.4	329.1	324.6	490	459	501	8
Os01g0829100	46.9	41	39.9	15.6	18.08	21.63	28.2	17.1	21.4	34.49	29.6	29.17	28.302	49.473	27.719	95.53	35.04	25.9	12.08	20.16	40.52	24.4	23.52	24.56	33.8	55.63	26.32	48.1	23.9	21.7	13
Os01g0857400	960	1095	1060	588.6	599.3	329.6	813	950	779	1004	979.1	1059	525.76	595.54	667.63	1325	691.5	685.9	532.3	377.3	289.7	1305	1261	1305	867.3	1009	1042	813	728	718	9
Os01g0514600	5.1	0.93	0	0.821	5.424	3.46	7.05	5.69	9.67	0	1.096	2.822	3.2656	0	0.792	0	2.061	2.072	0	2.419	0	3.697	4.276	6.5	3.169	0	2.193	0.93	0.96	0	18
Os01g0949260	1.02	1.86	2.16	0	0	1.73	0	2.53	0	0	1.096	0	1.0885	2.8004	0	0	1.031	0	1.858	0.806	1.724	2.958	2.851	0.722	0	4.714	0	0	0	0.83	17
Os01g0757500	696	416	652	177.3	175.4	200.7	43.8	45.5	44.9	519.9	530.7	555.1	961.16	787.83	871.16	752.3	342.1	323.2	203.4	156.4	189.7	30.31	55.59	44.78	475.4	369.6	510.9	429	415	493	3

Os01g0662300	841	807	894	1580	1705	1490	3427	2966	3193	1005	1157	1617	1058	741.16	708.81	1433	654.4	809.1	2245	2651	2406	1432	1358	2278	734.2	595.9	574.5	402	580	436	12
Os01g0656600	677	681	732	701.9	786.5	676.5	533	741	755	586.3	601.9	586.6	523.58	683.29	682.67	1254	724.5	652.7	657.7	663.6	775.1	577.5	729	684	631.7	600.6	673.2	692	593	582	6
Os01g0551100	626	671	526	429.4	475.5	432.5	754	694	700	605.3	683.1	1008	526.84	518.07	434	561.2	602.9	507.7	295.4	194.3	201.8	970.1	691.3	889.1	813.4	594	699.5	421	318	452	5
Os01g0692100	20.4	14.9	18.3	18.06	12.66	25.95	4.7	2.53	4.84	6.897	4.386	0.941	11.974	9.3345	10.296	0	4.122	17.61	18.58	20.96	12.07	7.394	12.83	6.5	0	7.543	13.16	15.7	11.5	9.16	2
Os01g0588500	107	108	97	79.63	92.2	56.23	261	234	237	135.4	127.2	211.7	141.51	112.01	125.92	155.2	99.96	106.7	115.2	83.85	99.15	92.42	115.4	134.3	116.2	110.3	157.9	79.6	57.4	70.8	5
Os01g0350000	43.8	51.2	45.3	55.01	30.73	76.12	27.4	19.6	13.8	54.32	48.24	32.93	28.302	39.205	39.598	59.7	24.73	27.97	9.29	22.58	24.14	20.7	20.67	18.06	20.07	16.97	10.96	41.6	30.6	50	8
Os01g0384901	2.04	3.73	0	0	0	0	0	0	0	0	0	0	0	0	0	0	0	0	0	0	0	0	0	0	0	0	0	0	0	0	14
Os01g0338000	1173	1028	1145	1143	1276	1241	1066	1118	1255	1002	885.9	855.2	854.49	928.78	984.41	1445	1089	1009	1111	1159	1020	966.4	1013	1067	932.8	836.3	1033	945	929	861	6
Os01g0605650	287	317	296	327.6	383.3	342.6	505	484	495	300.9	330	261.5	317.85	278.17	409.45	632.9	250.4	316	316.8	310.4	288	372.7	391.2	359	320.1	302.7	280.7	286	280	290	6
Os01g0349000	4.08	7.45	5.39	3.284	8.136	7.785	13.3	13.9	6.91	4.311	5.482	12.23	2.177	9.3345	6.3357	11.94	16.49	10.36	5.574	8.063	10.35	9.612	13.54	16.61	7.395	1.886	6.579	7.4	7.65	2.5	9
Os01g0134200	2.04	4.66	11.9	59.93	75.03	47.58	23.5	19	24.9	2.587	2.193	0	2.177	2.8004	6.3357	11.94	8.244	3.108	32.51	49.18	50.01	21.44	31.36	20.95	2.113	0.943	0	6.48	6.69	2.5	10
Os01g0527600	187	210	164	471.2	426.7	340	302	331	284	187.1	161.2	207.9	169.81	198.83	176.61	214.9	170	178.2	293.5	351.5	252.6	215.2	241.6	220.3	173.2	159.3	188.6	176	172	170	10
Os01g0137400	9.18	14.9	5.39	3.284	4.52	11.25	2.35	3.16	9.67	2.587	6.578	9.408	4.3541	5.6007	13.463	0	7.214	5.18	7.432	5.644	3.449	5.176	4.276	7.222	8.451	5.657	4.386	16.7	11.5	4.17	1
Os01g0862400	1.02	0	0	0.821	0.904	5.19	3.13	2.53	0.69	0	0	2.822	2.177	0.9335	0	0	1.031	3.108	11.15	4.838	5.173	2.958	5.701	5.056	1.056	1.886	2.193	0	1.91	0	15
Os01g0945650	2.04	0.93	0	10.67	16.27	5.19	1.57	5.06	21.4	1.724	3.289	2.822	1.0885	0	3.1679	0	0	1.036	2.787	4.031	1.724	2.958	24.23	0	0	0	0	1.85	1.91	1.67	17
Os01g0559000	775	573	738	604.2	659	570.9	656	760	717	665.6	565.7	843.9	775.03	681.42	700.1	1242	718.3	754.2	766.4	678.1	627.7	573	513.1	537.4	769	658.1	653.5	614	533	592	6
Os01g0849600	0	0	1.08	0	1.808	0	0	0	0	0	0	3.763	1.0885	0	0	0	3.092	1.036	1.858	0	0	0	0	0	1.056	0	2.193	1.85	0	0.83	20
Os01g0150200	271	286	269	270.9	264.9	308.8	354	293	403	275	291.6	279.4	253.63	285.64	255.81	668.7	277.2	274.5	308.4	257.2	254.4	426.6	456.8	435.5	307.4	322.5	342.1	302	307	260	6
Os01g0530316	9.18	2.79	3.23	46.8	47.91	50.17	34.4	22.8	25.6	6.897	8.771	6.586	9.7967	6.5342	3.1679	0	3.092	3.108	26.94	56.44	34.49	18.48	22.8	15.89	3.169	4.714	2.193	2.78	11.5	3.33	10
Os01g0696000	441	529	498	446.6	474.6	497.4	384	430	423	442.3	491.2	398.9	444.12	537.67	487.85	943.3	455.5	444.5	491.4	496.7	505.3	450.3	390.5	472.3	464.8	452.6	346.5	439	500	507	6
Os01g0187500	582	626	570	529.5	477.3	512.1	586	638	539	778.6	828.9	675.5	796.8	701.96	779.29	1588	691.5	658.9	469.1	582.9	515.6	444.4	538.8	499.8	834.5	776.9	675.4	755	697	720	13
Os01g0763600	844	821	914	638.7	670.7	631.5	860	908	692	665.6	744.5	567.3	658.55	680.49	689.8	1588	783.2	667.2	694.9	631.3	676	656.6	600	741.7	642.3	632.6	688.6	723	792	794	6
Os01g0959800	1248	1152	1124	796.3	868.7	756.9	630	678	662	777.7	622.8	636	443.03	603.01	593.18	788.1	887.3	1002	793.3	712.8	701	1009	905	897	661.3	695.8	877.2	712	735	746	14
Os01g0794650	0	0.93	1.08	0.821	0	0	0	0	1.38	0	0	0	0	0.9335	1.5839	0	0	1.036	0	0.806	0	1.479	0	0.722	1.056	0.943	0	0	0	0.83	4
Os01g0495900	2937	3241	3222	4149	4806	3997	4520	5199	4862	2939	3339	3229	3188.3	2770.5	2708.5	3976	2452	2715	3554	3540	3697	2977	2801	3772	2467	2381	2265	1806	2039	2163	12
Os01g0711400	####	####	####	10481	8873	12441	7654	####	####	65844	66887	57546	108198	82885	91037	#####	71902	61268	23800	19323	20028	4962	6335	8806	62896	60259	51080	####	####	####	13
Os01g0108200	1052	1144	1081	882.5	846.1	872.8	697	669	652	900.1	987.9	851.4	1078.7	1046.4	1092.9	2102	1086	994.6	779.4	770.8	821.7	869.5	868.7	730.2	1010	1051	899.1	1122	1049	1144	13
Os01g0766600	333	349	303	550.9	574	520.8	610	553	580	424.2	419.9	373.5	421.26	401.38	471.22	537.3	317.4	305.6	575	562	647.5	461.4	396.2	446.3	461.6	404.5	339.9	320	365	370	12
Os01g0939450	21.4	21.4	22.6	24.63	40.68	31.14	19.6	21.5	25.6	30.18	18.64	30.11	21.77	24.27	17.423	0	20.61	20.72	19.51	24.19	37.94	10.35	15.68	23.11	20.07	12.26	10.96	21.3	18.2	16.7	2
Os01g0186000	1.02	6.52	0	9.031	0	9.516	3.13	5.69	17.3	5.173	5.482	0.941	4.3541	0	1.5839	23.88	0	2.072	6.503	7.257	3.449	19.96	11.4	22.39	2.113	0	0	0.93	0	5	9
Os01g0177200	849	858	820	1002	1078	1022	790	880	819	856.2	833.3	741.4	926.33	821.44	896.51	1493	820.3	754.2	824.9	896.6	876.9	835.5	858.7	765.6	836.6	829.7	756.6	899	870	846	6
Os01g0342900	0	0	1.08	0	3.616	0	0.78	3.16	2.76	0	3.289	1.882	5.4426	0	2.3759	0	1.031	1.036	0	1.613	0.862	1.479	2.851	2.167	3.169	0.943	0	3.7	0.96	2.5	4
Os01g0743400	609	516	653	468.8	532.4	481	535	570	650	444.9	441.8	680.2	581.27	490.06	440.33	1230	632.7	618.5	523	610.4	626	477.6	463.2	550.4	435.2	463.9	486.8	377	452	408	6
Os01g0118000	621	494	543	472.1	468.3	436	236	243	209	516.4	396.9	557.9	455	488.2	435.58	991.1	517.3	448.6	403.2	290.3	372.5	250.7	357.7	334.4	418.3	381.9	500	488	425	377	13
Os01g0299700	0	0	0	0	0	0	0	0	0	0	0	0.941	0	0	0	0	0	0	0	0	0	0	0	0	0	0	0	0	0	0	20
Os01g0661650	0	0	7.54	0.821	0.904	0	0.78	1.9	0	0	0	0	9.7967	0.9335	3.1679	0	0	2.072	1.858	5.644	4.311	0	0	0	2.113	0	0	0	2.87	0.83	4
Os01g0711100	794	747	790	1291	1200	1183	546	633	543	726	744.5	734.8	1083.1	985.72	961.45	1839	840.9	788.4	1235	1278	1442	564.9	534.5	514.2	763.8	707.1	703.9	861	872	919	16
Os01g0665400	113	139	140	327.6	358	215.4	500	462	492	269.9	177.6	217.3	107.76	137.22	128.3	131.4	91.72	143	275	311.2	249.2	298.7	327.8	366.2	165.8	144.3	190.8	83.3	111	106	18
Os01g0362000	162	187	144	212.6	245.9	250	262	239	276	164.7	217.1	138.3	163.28	160.55	188.49	274.6	140.2	158.5	206.2	218.5	201.8	269.9	252.3	237.6	168	155.6	177.6	166	150	182	12
Os01g0925800	698	683	702	396.5	457.4	485.3	644	615	679	749.2	739	979.4	699.92	843.84	769.79	1242	807.9	723.2	592.7	442.6	455.3	444.4	444	540.2	781.7	768.4	875	764	732	780	13
Os01g0941500	0	0	0	0.821	0	0	0	0	0	0	0	0	0	0	0	0	0	0	0	0	0	0	0	0	0	0	0	0	0	0	2
Os01g0848200	143	113	116	55.01	61.47	82.18	11.7	20.9	24.2	93.12	61.4	73.38	96.878	87.744	102.16	83.59	187.6	151.3	53.88	65.31	49.15	41.41	59.15	31.78	156.3	168.8	98.68	162	105	125	3
Os01g0818600	346	404	397	885	877.8	945.5	103	101	122	264.7	253.3	222	198.11	255.77	266.89	441.8	482.3	392.7	746.9	1011	939	238.8	235.2	223.2	363.4	385.6	407.9	398	348	333	10
Os01g0846300	230	275	231	136.3	131.1	181.7	116	72.1	136	155.2	195.2	121.4	186.14	193.22	200.37	597	337	297.3	91.04	118.5	119.8	96.86	106.2	97.5	223.9	190.5	193	294	287	349	13
Os01g0525500	2.04	1.86	0	0.821	0	0	0	0	0	6.035	0	0	0	1.8669	0	0	2.061	1.036	0	0.806	0	0	0	1.444	1.056	0.943	2.193	0.93	0.96	0	7
Os01g0888200	48.9	55.9	58.2	134.6	125.7	106.4	175	134	150	116.4	104.2	156.2	133.89	77.476	75.237	131.4	79.35	69.41	153.3	123.4	122.4	102	70.55	121.3	75	92.4	85.52	50	51.6	81.6	15
Os01g0851700	4549	5136	5896	8884	9192	9221	2353	1886	2081	3666	4406	3090	6250.3	6893.5	5859.8	10663	4131	4496	9841	9170	9215	1836	1735	1874	3565	3160	2941	6129	7492	5274	16
Os01g0727400	40.8	41.9	36.6	27.91	33.45	47.58	20.4	16.4	30.4	14.66	21.93	22.58	22.859	28.004	31.679	35.82	30.92	41.44	18.58	19.35	22.42	34.01	34.21	33.95	42.25	32.06	21.93	40.7	27.7	45.8	8
Os01g0695200	1515	1139	1440	600.1	480.9	500.9	279	312	357	1571	1558	1327	1629.5	1326.4	1551.5	2427	1507	1472	617.8	688.6	581.1	365.3	317.8	286	1398	1387	1178	1396	1350	1519	13
Os01g0820400	0	0	0	0	0	0	0.78	1.27	0	0	0	0	0	0	0	0	0	0	0	0	0	0	0.713	0	0	0	0	0	0	0	18
Os01g0159400	326	399	320	894.9	928.4	800.2	990	1142	1285	421.6	435.3	439.4	384.25	328.57	365.89	394.1	253.5	331.5	617.8	533.8	538	1152	1082	962	418.3	414.9	462.7	330	252	287	18
Os01g0172000	645	612	666	454	466.4	501.7	626	571	600	595.8	603	629.4	631.34	523.67	592.39	1051	573	521.1	476.6	487.8	466.5	482.8	533.1	606.7	564.1	570.4	625	550	538	600	6
Os01g0655500	1514	1708	1659	2261	2222	1933	548	615	607	1833	1812	1512	2324	2247.8	2468.6	3033	1794	1785	1790	1772	2126	533.1	524.5	411	1909	1674	1414	1880	1893	2077	13
Os01g0109000	47.9	56.8	33.4	44.33	34.35	36.33	120	102	131	44.83	84.42	41.4	39.187	56.941	57.814	131.4	55.65	45.59	42.73	27.41	37.94	105	99.06	101.1	72.89	71.66	72.37	62.9	59.3	52.5	18
Os01g0511100	132	170	135	198.7	255.8	151.4	455	519	385	143.1	132.7	123.2	90.347	114.81	115.63	119.4	77.29	90.14	164.4	165.3	119.8	198.2	242.3	188.5	106.7	56.57	111.8	112	81.3	109	18
Os01g0974200	2990	1662	1991	824.3	1082	1134	767	960	445	4154	3306	7087	2951	4841.8	2413.1	1099	1897	2646	1273	769.2	1098	247.7	199.5	455	2706	6123	7335	1815	2611	2053	20
Os01g0383900	77.5	66.1	50.6	48.44	47.91	56.23	48.5	41.7	35.2	47.42	44.95	54.57	20.682	40.138	30.887	71.65	71.11	62.16	73.39	46.76	57.77	62.11	59.86	66.45	49.65	74.48	30.7	53.7	53.6	32.5	19
Os01g0719600	38.7	10.2	18.3	31.2	30.73	25.09	61.8	30.4	15.2	25	36.18	58.33	29.39	50.406	22.175	11.94	40.19	30.05	36.23	27.41	30.18	16.27	10.69	49.11	17.96	39.6	39.47	17.6	35.4	20.8	20
Os01g0157800	124	121	98.1	108.4	92.2	82.18	82.2	117	84.3	87.94	123.9	116.7	84.905	118.55	94.244	155.2	113.4	110.9	125.4	105.6	108.6	100.6	104	73.67	86.62	93.34	74.56	90.7	97.5	84.1	6
Os01g0227300	962	964	1137	633	561.4	643.6	251	214	225	676.8	747.7	555.1	798.97	820.5	901.26	1791	1014	951.1	601	534.6	514.7	339.4	378.4	371.2	812.3	843.8	782.9	1069	1043	1131	13
Os01g0958200	0	0	0	0	0	0	0	0	0	0	0	0	0	0	0	0	0	0	0	0	0	0	0	0	1.056	2.829	0	0.93	0	0	1
Os01g0120600	194	165	191	118.2	109.4	119.4	157	186	187	266.4	208.3	254	213.35	199.76	212.25	429.9	316.4	194.8	149.6	150.8	133.6	133.8	153.2	145.2	243	243.3	276.3	215	219	232	13
Os01g0388700	0	3.73	0	1.642	0.904	1.73	1.57	0.63	2.07	2.587	7.675	7.527	2.177	5.6007	2.3759	0	5.153	5.18	3.716	2.419	0.862	4.436	2.138	9.389	10.56	15.09	8.772	3.7	4.78	9.16	20
Os01g0631900	0	0	0	0	0	0	2.35	0	0	0.862	0	0	0	0	1.5839	0	0	0	0.929	0	0	0	0.713	0	0	0	0	0	0	1.67	5
Os01g0294700	50	15.8	5.39	4.926	0	18.17	1.57	3.16	0.69	3.449	2.193	21.64	30.479	9.3345	0.792	11.94	41.22	3.108	7.432	4.031	37.08	0.739	1.425	17.33	0	68.83	122.8	5.55	45.9	3.33	11
Os01g0918300	####	8660	####	4752	4956	5799	8977	8337	8937	8244	8601	11411	10687	13424	10559	20849	12484	10485	9066	7273	7924	6831	6107	6806	10211	10201	11054	####	9593	####	13
Os01g0552300	4716	4882	5771	4467	4287	4140	5127	5844	5782	6353	7039	6284	7779.7	7397.6	7503.1	9517	6611	6071	5483	4945	5898	3494	3696	4391	7368	7550	6835	6190	6168	6964	13
Os01g0732700	38.7	24.2	38.8	23.81	39.77	32.01	25.1	19	14.5	16.38	25.22	21.64	25.036	19.602	23.759	11.94	24.73	17.61	36.23	37.09	26.73	19.96	30.64	28.89	10.56	31.11	24.12	24.1	23	17.5	19
Os01g0655800	2219	2616	2483	2525	2730	2401	2464	2413	2327	2394	2350	2190	2120.4	2318.7	2470.1	3702	2037	2292	2110	2053	1952	2335	3079	2400	2369	2186	2195	2439	2132	2242	6
Os01g0859300	0	0	0	0.821	0.904	0	0.78	1.27	0	1.724	0	0	0	1.8669	0	0	0	0	0	0	0	0.739	2.851	1.444	0	0	0	0	0.96	0	17
Os01g0949400	640	505	610	399	433	355.5	359	324	290	488	497.8	493.9	477.86	491.93	445.88	823.9	592.6	608.2	551.8	580.5	600.1	282.4	253	281	513.4	518.6	510.9	489	519	479	13
Os01g0680900	46.9	27.9	39.9	56.65	67.8	109.9	33.7	49.3	47.7	42.25	32.89	79.97	27.213	40.138	20.591	23.88	40.19	22.79	67.81	54.83	75.01	34.75	32.78	41.17	45.42	41.49	50.44	42.6	22	13.3	10
Os01g0826900	4171	4035	4370	593.6	690.6	560.6	241	366	227	5080	5346	5687	5973.8	5873.3	6315.9	8574	5447	4788	953.1	821.6	1014	149.4	196.7	214.5	5690	6096	4620	4771	4382	4777	3
Os01g0691000	10.2	20.5	12.9	3.284	5.424	18.17	13.3	10.8	7.6	20.69	10.96	4.704	10.885	11.201	19.799	47.76	3.092	7.252	1.858	3.225	0.862	10.35	11.4	5.056	9.507	5.657	4.386	27.8	21	9.16	6
Os01g0112400	0	0	0	0	0	0	0.78	1.27	1.38	0	0	0.941	0	0	0.792	0	0	0	0	0	0	0	1.425	0	0	0	0	0	0	0	18
Os01g0905200	349	411	321	32.02	18.98	16.44	41.5	69.6	30.4	82.77	37.28	20.7	41.364	29.87	38.014	167.2	201	185.5	20.44	28.22	31.04	46.58	37.77	96.78	19.01	24.51	15.35	34.2	23	20.8	14
Os01g0900200	135	119	111	27.91	24.41	33.74	25.8	23.4	30.4	62.08	84.42	73.38	71.842	94.279	95.036	119.4	93.78	95.32	26.01	25.8	23.28	65.81	68.41	45.5	92.96	51.86	76.75	115	66.9	80.8	13
Os01g0532200	1247	1264	1356	1346	1414	1327	1147	1135	1113	1212	1287	1229	1461.9	1349.8	1445.3	2651	1248	1295	1326	1242	1254	922	1037	1045	1325	1328	1083	1322	1324	1324	6
Os01g0579900	902	971	890	954.8	966.3	866.8	945	1035	963	738	718.1	764.9	597.6	808.37	581.3	1111	823.4	833	768.2	769.2	829.5	1123	979.9	998.1	793.3	708.1	793.8	843	734	781	17
Os01g0652300	0	0	0	0	0	0	0	1.27	0	0	0	0	0	0	0	0	0	0	0	0	0	0	0	0	0	0	0	0	0	0	18
Os01g0519700	250	245	235	410.5	396.8	359	367	354	305	258.7	282.9	295.4	226.41	225.9	258.18	501.5	233.9	276.6	400.4	378.1	328.5	328.3	258	305.5	215.5	249.9	186.4	296	274	235	6
Os01g0593200	348	272	344	133.8	109.4	154	255	295	475	286.2	225.9	331.2	370.1	276.3	323.12	847.8	414.3	427.9	169.1	213.7	135.4	844.4	662	678.9	402.5	384.7	285.1	420	375	473	9
Os01g0259600	2288	2365	2367	2578	2797	2599	3696	3991	4141	2433	2774	2665	3002.1	2850.8	2883.5	6281	2664	2490	2569	2834	2783	3629	3034	3717	2993	2611	2487	2456	2567	2701	6
Os01g0541000	1135	1097	1156	848.9	834.4	956.7	1167	1070	1103	1201	1061	1297	1245.3	1188.3	1180.8	1648	1091	1208	1042	1083	1166	1101	931.4	900.6	1129	1257	1193	1029	1175	971	13
Os01g0307624	31.6	30.7	30.2	51.72	6.328	25.09	4.7	21.5	41.5	12.93	9.868	15.05	32.656	15.869	21.383	23.88	15.46	21.76	25.08	46.76	13.8	22.92	41.33	15.17	10.56	7.543	19.74	25.9	16.3	11.7	2
Os01g0734600	1247	1068	1350	570.6	612	561.4	1159	1506	1327	959.6	1186	1565	1948.5	1394.6	1474.6	2914	1790	1433	1008	924	1111	715	741.9	1056	1487	1446	1274	1243	1405	1544	13
Os01g0729400	1.02	0	2.16	0.821	0.904	1.73	1.57	0	0	0.862	0	0	2.177	0.9335	0	0	0	0	0	0	1.724	1.479	0	0	0	0	2.193	0	0	2.5	4
Os01g0887550	3.06	4.66	4.31	16.42	41.58	10.38	38.4	24	17.3	5.173	2.193	2.822	10.885	5.6007	6.3357	0	2.061	3.108	5.574	15.32	4.311	2.218	9.264	9.389	4.225	0	0	0	1.91	2.5	12
Os01g0141700	348	352	365	339.9	308.3	389.3	181	205	193	291.4	308.1	308.6	348.33	422.85	350.84	764.2	493.6	440.3	359.5	387	317.3	265.4	313.6	257.8	416.2	406.4	322.4	591	477	544	13
Os01g0191100	1108	855	801	1153	1155	1308	1075	832	1243	1173	1047	1892	973.14	1185.5	777.71	1505	696.6	873.4	1383	1321	1565	961.9	922.1	920.9	952.8	1274	1270	621	840	502	15
Os01g0872900	9.18	5.59	1.08	0.821	1.808	2.595	2.35	6.96	2.07	24.14	18.64	17.88	18.505	24.27	10.296	23.88	15.46	13.47	2.787	0	5.173	1.479	0.713	1.444	24.3	23.57	8.772	12	12.4	15.8	3
Os01g0930800	1487	2105	1775	3789	4309	3369	3963	4976	4260	2624	2979	2814	2135.7	1440.3	1684.5	2472	1402	1691	2540	2864	2666	3273	3099	3254	2123	2197	1724	1051	1300	1589	12
Os01g0290800	54	90.4	89.4	106.7	117.5	116.8	52.5	93.6	114	101.7	62.49	63.03	54.426	64.408	101.37	107.5	58.74	91.17	68.74	74.98	51.73	173	320	160.3	69.72	73.54	67.98	98.1	79.4	80.8	17
Os01g0949800	1.02	1.86	4.31	7.389	9.944	1.73	1.57	6.33	7.6	0	3.289	3.763	2.177	2.8004	0.792	0	0	1.036	3.716	4.838	0	4.436	9.977	2.889	0	0.943	0	1.85	0	1.67	12
Os01g0606400	444	508	526	679.8	734	743.9	674	767	640	415.6	562.5	554.1	605.22	473.26	542.5	656.8	569.9	605	700.4	831.3	735.5	709.8	572.2	691.2	641.2	608.1	552.6	475	643	653	12
Os01g0966900	0	0	0	0	0	0	0	0	0	1.724	0	0	0	0	0	0	0	0	0	0	0	0	0	1.444	0	1.886	0	0	0	0	7
Os01g0935300	25.5	42.8	18.3	110	121.1	88.23	48.5	46.8	53.9	42.25	46.05	19.76	28.302	42.005	39.598	23.88	32.98	23.83	66.88	70.15	71.56	58.41	54.87	39.72	27.47	28.29	15.35	39.8	26.8	44.2	10
Os01g0298500	52	36.3	43.1	26.27	28.93	28.55	19.6	13.9	11.7	12.07	17.54	19.76	28.302	16.802	18.215	59.7	44.31	42.48	23.22	35.48	19.83	29.58	13.54	10.83	27.47	17.91	26.32	28.7	26.8	38.3	13
Os01g0111400	165	184	114	143.7	150.1	136.7	246	198	247	172.4	187.5	192.9	142.6	185.76	181.36	298.5	180.3	188.6	160.7	150	160.4	207.8	198.1	214.5	214.4	188.6	219.3	160	151	190	6
Os01g0129800	0	0	0	0	0.904	0	0	0	0	0	0	0	0	0	0	0	0	0	0	1.613	0	0	0	0	0	0	0	0	0	0	16
Os01g0558700	13.3	24.2	12.9	17.24	9.04	7.785	32.9	29.7	22.1	21.55	14.25	32.93	20.682	15.869	19.799	59.7	30.92	14.5	19.51	16.93	19.83	17.01	14.97	36.83	8.451	25.46	28.51	10.2	10.5	10.8	6
Os01g0566500	0	2.79	1.08	53.36	11.75	59.69	0	1.27	3.46	4.311	3.289	0	0	0.9335	0	0	1.031	2.072	4.645	2.419	3.449	0.739	0.713	0.722	0	0	0	0.93	0	0.83	2
Os01g0844200	0	0	0	0	0	0	0	0	0	0	0	0	0	0	0	0	0	0	0	0	0	0	0	0	0	0	0	0.93	0	0	1
Os01g0636200	0	0	0	0	0	0	0	0	0	0	0	0	0	0.9335	0	0	0	0	0	0	0	0	0	0	0	0	0	0	0	0	4
Os01g0793900	629	549	548	122.3	91.3	87.37	47	61.4	37.3	136.2	89.9	113.8	108.85	117.61	132.26	585.1	588.4	431	177.4	177.4	142.3	53.98	69.84	122.1	106.7	48.08	76.75	98.1	117	114	14
Os01g0597600	1664	2201	1093	9.852	2.712	4.325	25.8	19.6	24.9	18.11	15.35	1.882	72.931	6.5342	8.7116	155.2	277.2	346	15.79	5.644	30.18	45.1	38.48	208	3.169	4.714	10.96	5.55	8.61	20	14
Os01g0775300	629	583	605	627.2	629.2	590.8	636	689	730	698.4	662.2	625.6	748.9	642.21	739.7	1516	623.5	680.7	585.2	648.3	587.2	693.5	682	706.4	693	636.4	600.9	670	666	647	6
Os01g0666500	146	147	144	139.6	141	146.2	273	270	300	194	178.7	192.9	216.62	232.43	189.28	250.8	162.8	150.2	160.7	154	134.5	214.4	193.8	228.2	178.5	175.4	206.1	149	171	190	5
Os01g0899387	0	2.79	5.39	3.284	4.52	0.865	1.57	3.8	6.91	1.724	1.096	1.882	4.3541	3.7338	3.1679	0	2.061	4.144	0.929	12.9	3.449	2.958	10.69	0.722	1.056	0.943	0	2.78	1.91	1.67	17
Os01g0846700	767	511	845	5.747	8.136	7.785	0.78	0	0	432.8	492.3	366.9	633.52	612.34	788.8	1397	793.5	673.4	11.15	8.869	4.311	0.739	0	1.444	626.4	608.1	506.6	731	781	814	13
Os01g0393000	650	757	812	883.4	917.5	754.3	1527	1751	1714	907.9	1031	919.2	1151.7	848.51	932.94	1039	747.1	755.3	894.6	1141	977.8	999.7	846.6	1239	741.6	713.7	583.3	609	663	668	18
Os01g0961200	520	439	531	320.2	350.7	334.8	276	366	234	395.7	383.7	279.4	432.14	511.53	441.92	967.2	658.5	612.3	289.8	347.5	350.1	303.9	337.8	282.4	445.8	375.3	462.7	695	643	616	13
Os01g0693800	1078	863	1033	1262	1033	1430	682	555	848	1671	1447	1782	1215.9	1215.4	1274.3	1672	834.7	1124	1219	1234	1348	780.1	659.2	694.8	1235	1460	1013	669	812	679	20
Os01g0111100	114	124	91.6	246.3	253.1	220.6	395	484	511	177.6	208.3	563.6	82.728	43.872	59.397	59.7	123.7	130.5	254.5	216.1	244	198.9	156.8	344.5	95.07	139.5	184.2	25	40.2	32.5	15
Os01g0819200	0	0	1.08	0	0	1.73	0	2.53	0	0	0	0	0	0	0	0	0	0	0	0	0	0.739	0.713	0	0	0	0	0	0	0	18
Os01g0101300	9.18	7.45	0	12.31	1.808	4.325	8.61	4.43	5.53	8.622	3.289	2.822	0	5.6007	12.671	0	13.4	6.216	10.22	4.838	9.484	13.31	12.83	7.945	11.62	9.428	2.193	5.55	10.5	12.5	1
Os01g0946200	155	141	166	128.9	96.72	93.43	193	191	194	181.9	180.9	136.4	170.9	160.55	167.1	298.5	120.6	115	112.4	104.8	114.7	130.1	148.9	135.1	161.6	122.6	120.6	117	135	150	6

Os01g0706700	224	158	131	15.6	13.56	34.6	40.7	39.2	38.7	122.4	116.2	105.4	87.082	103.61	88.7	226.9	161.8	151.3	27.87	33.86	34.49	50.28	52.02	47.67	77.11	106.5	127.2	102	114	76.6	13
Os01g0162200	0	2.79	1.08	4.926	2.712	0	0	0.63	0	3.449	0	0	2.177	0.9335	0	0	1.031	2.072	0.929	2.419	0.862	1.479	3.563	0.722	3.169	1.886	0	6.48	3.83	1.67	1
Os01g0616800	41.8	25.1	48.5	27.91	28.93	32.01	66.5	60.7	52.5	56.9	63.59	62.09	40.275	79.343	46.726	107.5	34.01	52.84	86.39	55.63	58.63	29.58	45.61	44.06	49.65	64.11	41.67	47.2	38.3	34.2	6
Os01g0557100	1218	1178	1413	293.1	180.8	314.9	433	529	463	1396	1454	1187	1391.1	1251.8	1492.1	4155	1855	1599	758	719.2	704.4	332	479.6	431.2	1707	1507	1482	1812	1778	1534	13
Os01g0796400	741	885	849	953.2	1003	923.9	1164	1243	1390	1011	1092	846.7	1172.3	950.25	1119.8	1290	711.1	726.3	961.5	920.8	732.9	1120	1270	1091	965.5	703.4	828.9	854	974	1005	18
Os01g0235200	148	167	122	114.9	91.3	126.3	116	125	101	131.9	174.3	120.4	130.62	136.28	137.01	238.8	162.8	196.8	126.3	126.6	135.4	167.1	168.2	149.5	177.5	198.9	171	222	207	197	13
Os01g0249200	34.7	18.6	22.6	35.3	30.73	30.28	31.3	18.3	23.5	65.53	46.05	33.87	7.6196	17.736	17.423	0	21.64	35.23	40.87	20.16	14.66	28.84	32.78	35.39	22.18	26.4	32.89	15.7	24.9	23.3	7
Os01g0759100	0	0	0	1.642	0.904	0.865	1.57	0	0	0	0	0	0	0	0	0	0	0	1.858	0.806	0	1.479	0	0.722	0	0	0	0	0	0	12
Os01g0652700	1.02	0.93	2.16	0.821	1.808	0.865	4.7	0.63	0.69	4.311	2.193	1.882	2.177	0	0	0	8.244	0	5.574	3.225	0.862	2.958	0	3.611	1.056	3.771	0	3.7	1.91	1.67	1
Os01g0326100	15.3	31.7	22.6	4.926	0	1.73	0	0.63	7.6	1.724	2.193	0	3.2656	1.8669	0.792	0	1.031	0	0	1.613	0	31.79	14.25	29.61	4.225	3.771	0	7.4	0.96	12.5	9
Os01g0862200	1703	1841	1929	2081	2394	2170	3525	3648	3518	1759	2075	2355	1883.1	2223.5	1872.2	3057	2400	2180	3048	2639	3407	2405	1886	2918	1816	1976	2050	1584	1758	1974	18
Os01g0322700	148	206	225	422	254	147.1	188	395	363	606.1	548.2	656.7	138.24	145.62	507.65	406	134	197.9	259.2	198.3	254.4	202.6	210.2	194.3	419.4	633.6	690.8	164	168	154	20
Os01g0768200	1079	1164	1236	950.7	909.4	940.3	1267	1592	1339	1256	1241	1085	1350.9	1295.6	1271.1	1719	1100	1160	1044	1030	992.4	1299	1328	1210	1245	1150	1112	1143	1161	1268	6
Os01g0358900	0	0	0	0	0	0	0	0	0	0.862	0	0	0	0	0	0	1.031	0	0	0	0	0	0	0	0	0	0	0	0	0	7
Os01g0715900	4.08	8.38	2.16	48.44	79.55	53.63	20.4	17.1	15.2	1.724	8.771	1.882	4.3541	0	4.7518	0	1.031	2.072	38.09	44.35	36.21	17.01	22.09	14.44	6.338	5.657	4.386	4.63	3.83	2.5	10
Os01g0585400	6.12	3.73	6.47	4.105	8.136	2.595	10.2	8.86	13.8	12.07	13.16	3.763	3.2656	2.8004	3.9598	0	9.275	4.144	2.787	8.063	8.622	12.57	15.68	18.06	6.338	4.714	2.193	1.85	9.56	1.67	9
Os01g0688600	200	165	152	148.6	145.5	134.9	217	240	275	167.3	144.7	191	152.39	141.88	133.84	131.4	158.7	168.9	178.4	207.2	216.4	229.2	216.6	320	143.7	153.7	168.9	107	124	123	18
Os01g0866200	157	71.7	105	33.66	33.45	28.55	151	179	149	81.05	87.71	47.98	52.249	42.005	53.854	143.3	110.3	95.32	41.8	34.67	33.63	136.8	152.5	134.3	45.42	66	72.37	78.6	64.1	62.5	18
Os01g0132000	89.7	51.2	12.9	579.6	98.53	1105	61.1	72.7	732	181.9	186.4	79.03	90.347	21.469	44.35	0	13.4	20.72	687.4	86.27	182.8	318.7	335.7	298.3	66.55	212.1	1083	7.4	190	16.7	11
Os01g0658900	128	175	113	124.8	110.3	111.6	36.8	42.4	19.3	80.18	93.19	78.09	90.347	126.02	84.74	226.9	181.4	102.6	97.54	110.5	106.1	38.45	47.75	50.56	104.6	90.51	103.1	126	154	140	13
Os01g0880300	10.2	0	2.16	1.642	0.904	6.92	7.05	4.43	11.7	1.724	3.289	9.408	0	1.8669	2.3759	0	1.031	3.108	9.29	2.419	4.311	8.873	12.83	7.222	2.113	0.943	17.54	2.78	6.69	3.33	11
Os01g0595600	211	179	207	197.9	141.9	188.6	25.1	42.4	52.5	147.4	144.7	145.8	199.2	122.28	160.77	752.3	150.5	160.6	259.2	166.9	194	46.58	73.4	49.11	219.7	149	127.2	158	137	203	6
Os01g0164700	302	311	282	260.2	282.9	268.2	219	290	256	265.6	321.2	277.5	296.08	265.1	310.45	406	328.7	309.8	241.5	268.5	243.1	291.3	240.9	268.7	339.1	307.4	320.2	308	315	316	13
Os01g0110050	7.14	3.73	2.16	4.105	4.52	2.595	0	0.63	3.46	0.862	2.193	9.408	0	1.8669	6.3357	11.94	7.214	3.108	1.858	0	2.587	2.218	1.425	0.722	3.169	3.771	10.96	4.63	6.69	9.16	13
Os01g0741250	0	0	0	0	0	0	0	0	0	0	0	0	0	0	0.792	0	0	0	0	0	1.724	0	0.713	0	0	0	0	0	0	0	16
Os01g0712501	10.2	7.45	6.47	5.747	2.712	0.865	2.35	3.16	6.22	3.449	1.096	1.882	4.3541	4.6673	2.3759	0	4.122	3.108	4.645	7.257	3.449	1.479	2.138	2.889	3.169	0.943	0	4.63	1.91	3.33	19
Os01g0103000	27.5	27.9	22.6	205.2	247.7	187.7	54.8	43	53.2	19.83	16.45	31.05	37.01	41.072	27.719	47.76	19.58	25.9	171.9	166.9	181.9	67.28	53.45	51.28	29.58	28.29	21.93	28.7	19.1	23.3	10
Os01g0957000	860	918	973	495.9	501.7	446.4	836	891	775	808.7	870.5	794.1	772.85	775.7	840.28	1170	1056	1056	510	561.2	500.9	809.6	795.3	824.8	839.8	900.4	710.5	952	821	950	13
Os01g0565800	0	3.73	0	0	0.904	0	2.35	5.06	2.76	0.862	7.675	0	0	0	0	0	2.061	1.036	0	0	0	0	1.425	2.889	0	0	0	1.85	0	1.67	5
Os01g0581400	17.3	22.4	32.3	32.02	41.58	45.85	18	19.6	21.4	43.97	29.6	32.93	27.213	28.937	30.095	35.82	19.58	23.83	26.01	38.7	25	32.53	22.09	37.56	30.63	20.74	6.579	46.3	27.7	35	2
Os01g0729500	6.12	3.73	14	5.747	13.56	11.25	11.7	5.69	19.3	2.587	5.482	9.408	7.6196	5.6007	7.1277	11.94	3.092	8.288	9.29	16.93	13.8	8.133	10.69	13.72	4.225	1.886	2.193	1.85	3.83	1.67	12
Os01g0674400	0	0	0	0	0	0	0	0	0	0	0	2.822	0	0	0	0	0	0	0	0	0	0	0	0	0	0	0	0	0	0	20
Os01g0668400	39.8	22.4	34.5	53.36	58.76	44.98	14.1	25.3	26.9	25.87	17.54	42.34	40.275	41.072	33.263	119.4	30.92	35.23	47.38	46.76	33.63	15.53	12.83	20.22	45.42	40.54	50.44	15.7	31.6	27.5	6
Os01g0154950	5.1	9.31	12.9	9.852	16.27	5.19	0.78	16.4	5.53	9.484	8.771	3.763	17.416	9.3345	34.847	11.94	7.214	13.47	7.432	4.031	6.898	21.44	32.07	14.44	21.13	8.486	6.579	25.9	20.1	26.7	1
Os01g0961000	156	137	135	469.6	555.9	384.9	159	445	284	91.39	75.65	78.09	133.89	121.35	191.66	513.5	174.2	213.4	345.6	433	340.6	188.5	287.9	131.4	247.2	62.23	76.75	164	219	187	10
Os01g0834150	7.14	1.86	2.16	0.821	0.904	3.46	3.13	3.8	0.69	1.724	1.096	0	2.177	0.9335	2.3759	0	5.153	10.36	4.645	1.613	0	2.218	2.138	3.611	1.056	2.829	2.193	2.78	0	3.33	19
Os01g0817633	0	3.73	0	2.463	3.616	2.595	0.78	2.53	2.07	0.862	2.193	2.822	3.2656	0	0	0	0	0	2.787	5.644	0	0.739	2.851	2.889	0	0	2.193	0.93	0	0.83	12
Os01g0542100	10.2	6.52	9.7	11.49	23.5	30.28	5.48	10.1	4.15	12.07	2.193	11.29	10.885	7.4676	4.7518	11.94	7.214	5.18	22.29	18.54	20.69	8.133	6.414	7.222	6.338	9.428	8.772	13.9	6.69	14.2	10
Os01g0135000	0	0	0	0	0	0	0	0	0	0	0	0	0	0	0	0	0	0	0	0	0	0	0	0	0	0	0	0.93	0	0	1
Os01g0281000	3224	3406	3451	522.1	507.1	560.6	2160	2622	2311	4709	5073	5401	5823.6	5439.2	5728.3	8920	4886	4599	981.9	1071	795	1455	1603	1711	5592	5916	5535	4787	5014	5305	13
Os01g0384375	0	0	0	0	0	0	0	0	0	0	0	0	1.0885	0	0	0	0	0	0	0	0	0	0	0	0	0	0	0	0	0	4
Os01g0727600	1.02	3.73	6.47	10.67	9.944	6.055	8.61	5.69	8.29	1.724	3.289	0	8.7082	3.7338	1.5839	0	8.244	2.072	4.645	4.838	19.83	3.697	4.988	1.444	4.225	2.829	0	4.63	1.91	6.66	10
Os01g0179901	6.12	6.52	5.39	4.105	5.424	9.516	4.7	1.9	1.38	1.724	5.482	9.408	6.5311	3.7338	5.5438	11.94	3.092	6.216	2.787	4.838	1.724	1.479	1.425	6.5	8.451	4.714	8.772	6.48	10.5	5	13
Os01g0711000	159	148	140	182.3	179	193.8	124	142	160	150	131.6	137.4	118.65	156.82	133.84	250.8	174.2	130.5	118	133	164.7	116.1	129	116.3	120.4	123.5	138.2	150	148	158	6
Os01g0384800	1103	1523	853	277.5	135.6	172.1	182	176	243	164.7	110.7	125.1	173.07	112.01	176.61	788.1	1425	940.7	182.1	195.1	451.8	526.4	374.1	1554	143.7	140.5	162.3	118	113	244	14
Os01g0179600	2200	1813	2706	164.2	113.9	109	193	300	253	1928	2442	2684	6328.7	4235.1	4903.1	6591	3918	2674	565.7	388.6	400.1	41.41	112.6	112.7	2650	2209	1647	3027	2836	3362	13
Os01g0801000	15.3	17.7	17.2	18.88	16.27	18.17	30.5	20.9	20	19.83	25.22	25.4	21.77	21.469	19.007	0	22.67	15.54	36.23	41.93	45.7	29.58	11.4	20.22	9.507	18.86	19.74	13.9	19.1	20	15
Os01g0524251	0	0.93	0	1.642	0	4.325	0	0	0	0	0	7.527	0	0.9335	1.5839	0	1.031	2.072	0.929	4.838	0.862	2.958	4.276	1.444	0	1.886	15.35	9.25	0.96	0	11
Os01g0694500	0	1.86	0	0	0	0	0	0	0	0	0	0	0	0.9335	0	0	0	0	0	0	0	0.739	0	0	0	0	0	0	0	0	14
Os01g0375100	389	425	425	359.6	433.9	327	279	317	289	338	351.9	267.2	389.69	345.38	354.8	501.5	314.3	326.4	275	322.5	291.4	347.5	307.1	281.7	348.6	321.5	269.7	400	396	424	8
Os01g0695100	697	582	679	899.8	1069	1113	113	98.7	85	494	415.5	484.5	697.74	665.55	586.85	871.7	727.6	620.6	994	928.8	1061	79.11	90.5	67.89	534.5	452.6	443	552	501	669	16
Os01g0177900	23.4	23.3	32.3	19.7	9.944	16.44	14.1	16.4	11.7	29.31	36.18	39.51	42.452	43.872	34.055	0	44.31	53.87	13.93	13.71	12.07	30.31	24.23	22.39	59.16	57.51	57.02	75.9	66	67.5	1
Os01g0250900	1323	1493	1522	2702	2852	2952	2289	2300	2591	1202	1472	1485	1486.9	1419.8	1248.1	2711	1284	1322	2446	2664	2887	2281	2039	2540	1494	1416	1421	1204	1284	1237	12
Os01g0556650	2162	2333	1977	270.9	312.8	371.1	373	230	236	1332	1527	2091	687.94	906.38	583.68	1421	1790	1686	441.3	214.5	308.7	414.8	308.6	805.3	1072	1646	1555	1060	1076	1337	3
Os01g0890600	60.2	63.3	74.4	126.4	153.7	161.8	389	352	443	89.67	129.4	101.6	91.436	69.075	76.029	143.3	54.62	100.5	105	118.5	163.8	345.3	255.8	355.3	79.23	124.5	67.98	75.9	70.8	81.6	18
Os01g0178300	85.6	87.6	95.9	107.5	109.4	124.6	57.9	77.2	124	139.7	103.1	126.1	116.47	163.35	76.029	191.1	148.4	112.9	131	123.4	161.2	95.38	100.5	95.34	115.1	127.3	133.8	117	86.1	97.5	6
Os01g0324400	256	253	250	375.2	405	369.4	160	201	180	171.6	194.1	225.8	200.29	266.97	198.78	489.6	238.1	320.1	375.3	428.9	425.1	238.8	240.2	192.8	219.7	225.3	223.7	254	292	212	10
Os01g0321700	339	349	344	336.6	368.8	335.6	261	259	299	327.6	325.6	319.9	365.74	325.77	320.75	704.5	314.3	307.7	268.5	273.3	234.5	329	265.8	304.1	340.1	304.5	331.1	297	328	314	6
Os01g0795600	84.6	97.8	80.8	36.94	44.29	35.47	69.7	77.2	89.1	37.07	44.95	38.57	30.479	46.673	53.062	155.2	140.2	78.74	31.58	54.83	48.28	66.54	46.32	197.2	26.41	30.17	41.67	20.4	25.8	23.3	9
Os01g0958700	974	1049	1094	785.7	888.6	794.1	723	841	835	833.7	844.2	668	908.91	927.85	944.02	1302	1008	869.2	682.8	668.4	614.8	1020	1118	910.8	835.6	808	903.5	1072	1068	1021	8
Os01g0857900	638	591	581	248.8	236.8	274.2	272	283	230	448.3	566.8	518.4	379.89	445.26	494.19	895.6	738.9	671.4	301	292.7	313.8	502	374.8	377	632.8	724.1	765.3	730	527	634	13
Os01g0687800	7.14	5.59	3.23	4.926	8.136	10.38	2.35	0.63	2.07	4.311	7.675	2.822	3.2656	6.5342	3.1679	83.59	13.4	13.47	2.787	9.675	11.21	5.176	7.839	5.778	5.282	1.886	10.96	8.33	9.56	6.66	6
Os01g0870750	0	0.93	0	0.821	0	0	0	0	0	0.862	0	0	0	0	0	0	0	0	0	0	0	0	1.425	0	1.056	0	0	0	0	0	17
Os01g0905500	11.2	5.59	18.3	1.642	1.808	2.595	5.48	8.22	3.46	24.14	14.25	39.51	6.5311	7.4676	12.671	0	8.244	11.4	9.29	3.225	8.622	3.697	4.276	8.667	13.73	21.69	28.51	5.55	3.83	4.17	20
Os01g0796500	406	401	469	422.8	562.3	489.6	608	576	601	537.1	578.9	711.3	445.2	430.32	381.73	800	559.6	526.3	700.4	594.2	695.8	458.4	430.4	466.6	513.4	610	552.6	451	490	447	6
Os01g0827800	4.08	1.86	0	0	0	0	0	0	2.07	0	1.096	0	0	0.9335	2.3759	0	0	0	0	0	0	7.394	3.563	8.667	0	0	0	0	0	0	9
Os01g0263400	44.9	51.2	48.5	139.6	141	134.9	45.4	48.7	48.4	15.52	25.22	3.763	26.124	22.403	15.047	107.5	50.5	38.33	65.03	82.24	81.05	85.77	83.38	66.45	29.58	23.57	24.12	40.7	38.3	40.8	10
Os01g0107700	10.2	6.52	6.47	6.568	3.616	7.785	9.39	6.96	8.98	10.35	12.06	13.17	10.885	12.135	11.879	11.94	13.4	15.54	15.79	13.71	13.8	12.57	10.69	13	7.395	15.09	13.16	7.4	0.96	16.7	20
Os01g0386600	7.14	1.86	11.9	11.49	5.424	9.516	25.1	15.8	20.7	7.76	8.771	17.88	1.0885	7.4676	7.9197	11.94	5.153	10.36	10.22	5.644	10.35	22.92	17.82	26.72	15.85	11.31	6.579	13.9	3.83	6.66	18
Os01g0571133	10.2	14	4.31	7.389	3.616	7.785	0	0.63	1.38	1.724	0	0.941	2.177	5.6007	5.5438	23.88	17.52	11.4	2.787	3.225	5.173	14.79	23.52	8.667	5.282	7.543	0	12	10.5	11.7	8
Os01g0117100	287	373	400	353.8	445.7	321.8	592	784	398	530.2	415.5	236.1	341.8	350.04	447.46	859.7	426.6	410.3	271.3	408.8	335.4	526.4	471.8	277.3	366.6	307.4	201.7	476	385	438	6
Os01g0949300	1112	917	1046	454	325.4	399.7	462	594	672	525.1	428.7	487.3	244.92	194.16	255.81	823.9	1191	750.1	409.7	470.9	633.7	797.1	770.4	1248	325.4	363	445.2	189	173	408	14
Os01g0237400	2.04	2.79	0	5.747	4.52	1.73	9.39	6.96	10.4	4.311	1.096	1.882	1.0885	5.6007	4.7518	0	5.153	1.036	4.645	4.031	3.449	2.958	3.563	4.333	4.225	1.886	4.386	0	0	2.5	18
Os01g0683500	0	0	0	1.642	0.904	0	0	0	0	0	2.193	0.941	1.0885	0	3.9598	0	0	0	0	0.806	0.862	0	0	0	1.056	0.943	0	0.93	0	0	4
Os01g0509900	5.1	8.38	15.1	10.67	12.66	10.38	29	28.5	13.8	25.87	13.16	26.34	9.7967	9.3345	5.5438	23.88	9.275	8.288	0.929	9.675	6.898	10.35	10.69	15.17	9.507	7.543	6.579	6.48	7.65	5.83	5
Os01g0634900	23.4	14	5.39	4.926	0.904	6.055	1.57	3.16	0.69	6.897	8.771	14.11	7.6196	13.068	8.7116	0	14.43	17.61	0.929	2.419	1.724	8.133	2.138	11.56	6.338	18.86	50.44	10.2	9.56	11.7	11
Os01g0368000	6.12	7.45	2.16	27.09	18.98	25.09	0	0	0	0.862	0	3.763	1.0885	0.9335	0.792	0	3.092	3.108	10.22	9.675	25	3.697	2.138	0	1.056	0.943	4.386	1.85	4.78	5	10
Os01g0674800	223	215	222	305.4	225.1	308.8	141	149	269	232.8	242.3	152.4	141.51	175.49	137.8	214.9	213.3	180.3	232.2	195.1	216.4	317.2	275.8	293.2	161.6	179.1	230.3	210	236	187	9
Os01g0271500	922	896	934	1156	1186	1091	943	927	876	856.2	864	875.9	1033	990.39	978.87	1827	858.4	847.5	1143	1255	1093	932.4	923.6	881.1	884.2	908.9	872.8	896	908	927	6
Os01g0514300	804	803	846	963.8	912.1	856.4	471	443	424	614.7	689.6	512.7	757.61	708.49	837.11	1230	751.3	757.3	719	670	728.6	494.6	487.4	442	751.1	549.7	622.8	823	705	774	8
Os01g0237200	1.02	1.86	1.08	26.27	29.83	28.55	4.7	9.49	6.91	0.862	0	0	0	2.8004	1.5839	0	0	0	21.37	29.83	22.42	5.176	6.414	3.611	1.056	0	0	6.48	0.96	0	10
Os01g0188100	1408	1382	1439	555	677.1	693.8	2075	2620	2207	1368	1149	1682	1855.9	2240.3	2071	3630	2186	1767	700.4	636.2	639.8	1732	1919	2085	1968	1788	2042	2106	2048	2089	13
Os01g0850000	123	140	115	174	173.6	100.3	264	262	198	92.25	102	110.1	121.91	108.28	110.08	83.59	173.1	118.1	270.3	233.8	263.8	111.6	132.6	211.6	106.7	149.9	116.2	105	122	148	12
Os01g0529800	59.1	57.7	107	21.35	10.85	11.25	5.48	3.8	51.8	18.11	13.16	7.527	7.6196	4.6673	9.5036	0	15.46	13.47	4.645	4.031	0	4.436	15.68	15.89	19.01	3.771	2.193	8.33	8.61	6.66	14
Os01g0926200	399	355	332	208.5	192.5	207.6	204	212	160	333.7	262	309.5	383.16	365.91	373.81	525.4	273.1	261.1	209	180.6	189.7	125	155.4	155.3	270.4	240.4	241.2	303	277	239	13
Os01g0363900	516	578	538	799.6	876.9	756.1	414	558	448	428.5	441.8	325.5	469.15	439.66	449.04	859.7	532.8	482.8	550.9	654.7	612.2	567.8	580.8	470.2	421.5	462	414.5	560	480	551	2
Os01g0891601	16.3	20.5	38.8	4.105	8.136	8.65	25.1	24	13.8	27.59	17.54	8.467	10.885	15.869	12.671	11.94	16.49	27.97	2.787	7.257	2.587	53.24	64.85	28.89	13.73	9.428	8.772	20.4	12.4	10.8	17
Os01g0949220	1.02	0	0	0.821	0	0	0	1.27	0	0	0	0	0	0	0	0	0	1.036	0	2.419	0	0	0.713	0	0	0	0	0	0	0	19
Os01g0869400	4.08	4.66	6.47	1.642	0.904	0	4.7	6.33	4.15	1.724	3.289	0	6.5311	5.6007	9.5036	0	0	1.036	4.645	1.613	2.587	0	4.988	0	1.056	0	0	0	1.91	3.33	4
Os01g0841400	0	0	0	16.42	4.52	10.38	3.13	3.16	1.38	0	0	1.882	0	0	1.5839	0	1.031	0	4.645	6.45	9.484	4.436	4.988	2.167	0	0	0	0	0.96	0	10
Os01g0374900	319	359	318	358.8	358.9	348.6	552	523	476	382.8	355.2	337.8	340.71	402.32	324.71	250.8	274.1	294.2	376.2	395.9	363.9	377.1	404.8	463	284.2	248	390.3	306	297	280	18
Os01g0812100	0	0	0	0	0	0	0.78	0	0	0	0	0	0	0	0	0	0	0	0	0	0	0.739	0	0	0	0	0	0	0	0	9
Os01g0813000	322	392	363	356.3	399.6	316.6	509	480	497	370.7	356.3	493.9	291.72	282.84	244.72	382.1	292.7	285.9	335.4	370.1	332.8	478.4	353.5	445.6	329.6	336.6	276.3	237	255	286	18
Os01g0302100	108	92.2	65.7	128.9	122	125.4	124	95.5	98.1	101.7	78.94	118.5	67.488	75.61	66.525	107.5	115.4	71.49	102.2	73.37	149.2	147.1	125.4	153.1	92.96	128.2	85.52	105	104	80.8	9
Os01g0316900	295	294	311	265.2	250.4	247.4	283	321	272	213	291.6	210.7	303.7	262.3	330.25	406	261.8	255.9	241.5	238.7	215.6	283.9	241.6	247	262	252.7	245.6	305	324	343	13
Os01g0605500	0	0	0	0	0	0.865	0.78	0	0	0	0	0	0	0	0	0	0	0	0	1.613	0	0.739	1.425	2.889	0	0	0	0	0.96	0	9
Os01g0774400	0	0.93	0	0	1.808	1.73	2.35	0	0.69	0.862	0	0	3.2656	0.9335	0.792	0	0	0	0	0	0	0	0	0	1.056	0	0	0.93	1.91	0	4
Os01g0278050	622	342	454	110.8	83.17	102.9	244	176	189	352.6	368.4	386.7	457.18	485.39	414.2	537.3	556.5	506.6	203.4	192.7	238	153.1	129	216	440.5	470.5	515.3	405	408	310	3
Os01g0629000	1.02	0.93	1.08	4.105	5.424	5.19	0.78	0.63	0	1.724	0	1.882	3.2656	0	1.5839	0	5.153	4.144	5.574	2.419	4.311	2.218	0	0.722	4.225	0	4.386	4.63	4.78	5.83	16
Os01g0740300	672	524	621	201.1	181.7	449.8	84.6	285	126	207.8	276.3	374.4	236.21	392.98	412.61	573.2	443.1	493.2	144	70.95	93.98	122	174.6	117.7	391.9	314	353.1	431	442	385	13
Os01g0726001	234	211	272	258.6	226	205.9	221	221	220	286.2	271.9	318	261.24	245.5	303.32	465.7	313.3	335.7	242.5	232.2	290.6	215.9	235.9	197.9	277.8	358.3	342.1	308	311	293	13
Os01g0583600	1.02	3.73	4.31	0.821	0.904	0	0	0	0	0	0	2.822	0	0	0	0	3.092	0	0.929	0	0	0	2.138	0.722	0	0	0	0.93	0	2.5	14
Os01g0925600	4.08	4.66	2.16	0.821	1.808	0	0	3.8	3.46	5.173	0	0	0	0.9335	0.792	0	0	2.072	0	2.419	0.862	4.436	2.138	0	0	0	0	1.85	0.96	1.67	14
Os01g0777450	9.18	4.66	12.9	5.747	6.328	3.46	5.48	4.43	3.46	0.862	1.096	19.76	1.0885	1.8669	0.792	0	1.031	0	1.858	8.063	5.173	6.654	8.552	1.444	1.056	6.6	8.772	3.7	1.91	5.83	20
Os01g0956700	219	239	241	325.9	290.2	222.3	207	263	229	225.9	235.7	199.5	251.45	240.83	251.05	489.6	197.9	226.9	227.6	204	188.8	278.7	250.1	221	246.1	215	247.8	249	265	260	6
Os01g0152700	670	504	543	501.6	517.1	511.2	879	627	672	723.4	674.3	900.4	608.48	604.88	536.95	859.7	573	517	685.6	689.4	779.4	432.5	480.3	583.6	577.8	627.9	607.4	503	514	517	15
Os01g0364400	655	902	847	889.1	1075	693.8	410	476	295	764.8	620.6	461.9	579.09	654.35	616.94	871.7	677.1	776	517.4	573.3	568.2	462.1	523.1	250.6	683.5	517.6	453.9	740	518	712	8
Os01g0520600	279	306	245	295.6	300.1	312.3	245	277	313	284.5	280.7	265.3	342.88	299.64	313.62	692.6	355.5	307.7	197.9	257.2	246.6	259.5	292.2	279.5	263	286.6	252.2	308	295	328	6
Os01g0976450	63.2	84.8	73.3	160.1	153.7	167.8	94.7	131	151	54.32	48.24	34.81	47.895	42.939	48.31	131.4	37.1	67.34	92.9	124.2	89.67	172.3	171	115.6	49.65	23.57	28.51	34.2	60.2	43.3	12
Os01g0773500	0	0.93	0	3.284	1.808	1.73	8.61	2.53	0	0.862	7.675	4.704	0	2.8004	4.7518	0	0	0	0	0.806	3.449	6.654	6.414	4.333	6.338	0.943	4.386	3.7	2.87	3.33	5
Os01g0763900	1.02	0	0	0.821	0	0.865	3.91	1.9	2.07	0	0	0	0	0.9335	0	0	0	0	0	1.613	0.862	0.739	0	2.889	0	0	0	0	0	0	18
Os01g0967800	1793	1821	1884	2306	2188	2188	2106	2356	2219	1885	1760	1882	2085.6	1711	1816	3690	1726	1853	2101	2149	1978	2121	2157	2227	1817	1667	1500	1648	1706	1804	6
ENSRNA04947380	0	0	0	0.821	0	0	0	0	0	0	0	0	0	0	0	0	0	0	0	0	0	0	0	0	0	0	0	0	0	0	2
ENSRNA04947387[illegible]	11.2	1.86	3.23	1.642	0.904	0.865	0	0	2.07	0.862	5.482	0.941	6.5311	4.6673	3.1679	0	2.061	6.216	0	0.806	6.036	3.697	1.425	1.444	1.056	5.657	4.386	1.85	1.91	5	19
ENSRNA04947387[illegible]	5.1	2.79	4.31	7.389	9.04	4.325	6.26	4.43	2.07	1.724	2.193	8.467	5.4426	7.4676	7.1277	0	2.061	0	10.22	10.48	10.35	5.915	2.851	4.333	6.338	5.657	2.193	5.55	6.69	0.83	16
ENSRNA04947388[illegible]	4.08	0.93	2.16	0	0	0.865	0.78	0.63	2.07	2.587	1.096	6.586	2.177	0.9335	0	0	0	4.144	2.787	0.806	0	1.479	2.851	1.444	3.169	6.6	4.386	7.4	0	2.5	20
ENSRNA04947389[illegible]	2.04	0.93	0	0.821	0	0.865	0	0	4.15	5.173	2.193	2.822	0	2.8004	1.5839	0	4.122	0	0	0	1.724	0	0.713	0.722	7.395	3.771	2.193	1.85	2.87	3.33	20

ENSRNA04947389	0	0.93	0	0	0	1.73	0	0	0.69	0	0	0	0	1.8669	0	23.88	0	0	0	0	0	0	0.713	2.889	0	0	0	0	0	0	6
ENSRNA04947394	0	0	0	0	0.904	0	0	0	0.69	0	0	0	0	0.9335	0	23.88	0	0	0	0	0	0	0	0	1.056	0	0	0	0	3.33	6
ENSRNA04947289	1.02	2.79	2.16	0.821	0	0	0	0	0	1.724	0	0	0	2.8004	0.792	0	0	1.036	0	0	0	0	0	0.722	1.056	0	0	1.85	0.96	3.33	1
ENSRNA04947290	3.06	0	3.23	0.821	2.712	6.055	2.35	0	5.53	4.311	9.868	3.763	8.7082	8.4011	10.296	23.88	10.31	7.252	2.787	0.806	12.07	2.218	0.713	0.722	10.56	11.31	4.386	6.48	6.69	18.3	13
ENSRNA04947290	0	0.93	2.16	0	0	0	0	0	0	0	0	0	0	0	0	0	3.092	0	0	0	0	0	0	0.722	5.282	2.829	0	0.93	0.96	3.33	1
ENSRNA04947297	0	0	0	0	0.904	0	0	0	0.69	0.862	0	0	0	2.8004	0	0	0	0	0	0	1.724	0	0.713	0	0	0	0	0.93	0	0	4
ENSRNA04947298	0	0.93	1.08	0	0	0	0	0	0	0	1.096	0	2.177	0	1.5839	0	1.031	0	0.929	0	0	0	0	0	0	0.943	0	1.85	5.74	2.5	1
ENSRNA04947299	2.04	0.93	6.47	0	0.904	0.865	0	1.27	0	2.587	3.289	0.941	0	1.8669	1.5839	0	0	2.072	0	0.806	0	1.479	2.851	0	4.225	1.886	2.193	1.85	3.83	6.66	1
ENSRNA04947304	2.04	0	0	0	0	0	0	0	0	0	1.096	1.882	2.177	0	0.792	0	1.031	0	0	0	0	0	0	0	0	0	0	0	0	0	19
ENSRNA04947307	1.02	0.93	1.08	0	0	0	0	0	0	0	1.096	1.882	0	0	0	0	0	0	0	0	0.862	0	0	0	0	0	0	0.93	1.91	0	20
ENSRNA04947308	0	0	0	0	0	0	0	0	0	0	0	0	0	0	0	0	0	0	0	0	0	0	0	0	0	0	0	0	0	2.5	1
ENSRNA04947310	0	0	0	0	0	0	0	0	0	0	0	0	0	0	0	0	0	0	0	0	0	0.739	0	0	0	0	0	0	0.96	0.83	1
ENSRNA04947312	0	0	0	0.821	0	0	0	0	0	0	0	0	0	0.9335	0	0	1.031	0	0	0	1.724	0	0	0	0	0	0	0	0	0	16
ENSRNA04947312	2.04	0.93	2.16	0.821	1.808	3.46	2.35	2.53	0	1.724	0	2.822	7.6196	3.7338	3.1679	0	3.092	3.108	0.929	0.806	0	0.739	0.713	0	1.056	3.771	0	1.85	1.91	2.5	4
ENSRNA04947316	0	0	0	0	0	0	0	0	0	0	0	0	0	0	0.792	0	0	0	0	0	0	0	0	0	0	0	0	0	0	0.83	4
ENSRNA04947317	0	0	1.08	0	0	0	0	0	0	0	0	2.822	0	0	0.792	0	0	0	0	0	0	0	0.713	0	0	0.943	0	1.85	2.87	0	1
ENSRNA04947318	2.04	0.93	2.16	0.821	4.52	2.595	0	0.63	0	0.862	4.386	2.822	0	3.7338	2.3759	11.94	2.061	0	0	0	3.449	0.739	0.713	0	3.169	9.428	10.96	1.85	5.74	0.83	13
ENSRNA04947321	6.12	2.79	4.31	0	0	2.595	0	0	2.07	0	0	0	1.0885	1.8669	0	0	2.061	6.216	0.929	0	0.862	2.218	0	0	3.169	7.543	0	2.78	0.96	4.17	19
ENSRNA04947322	1.02	0	0	0	0	0.865	0	0	0	0	0	0	0	0	0.792	0	1.031	0	0.929	0	0	0.739	0	1.444	2.113	0.943	0	0	0	5	1
ENSRNA04947323	2.04	0	0	1.642	0.904	0.865	0.78	0	0	0.862	0	3.763	0	0	0.792	0	0	0	0	0.806	0	0	2.138	0.722	3.169	2.829	0	0.93	2.87	1.67	20
ENSRNA04947597	0	0	0	0	0	0	0	0	0	0	0	0	0	0	0	0	0	0	0	1.613	0	0	0	0	0	0	0	0	0	0	16
ENSRNA04947596	0	0	0	0	0	0	0	0	0	0	0	0	0	0	1.5839	0	0	0	0	0	0	0	0	0	0	0	0	0	0	0	4
ENSRNA04947595	1.02	0.93	1.08	4.105	13.56	3.46	0.78	0	4.84	0	1.096	0	1.0885	5.6007	4.7518	0	2.061	2.072	10.22	10.48	8.622	5.176	3.563	0	1.056	0.943	4.386	1.85	2.87	0.83	10
ENSRNA04947594	0	0	1.08	0	0	0	0	0.63	0.69	0	0	0	1.0885	0	0	47.76	0	0	0	0	0	0	0	0	0	0	0	0.93	0.96	0	6
ENSRNA04947593	2.04	0	0	5.747	14.46	9.516	3.13	1.27	3.46	3.449	0	0	0	0	0	0	0	4.144	3.716	6.45	12.07	7.394	2.138	0.722	0	0	6.579	0.93	1.91	0	10
ENSRNA04947401	2.04	4.66	1.08	0	0.904	0	0	0	0	1.724	3.289	0	1.0885	0.9335	1.5839	0	2.061	1.036	1.858	0	0	0	0	0.722	1.056	0.943	0	2.78	0	4.17	14
ENSRNA04947402	0	0	1.08	0	0	0	0	0	0	0	0	0	0	0	0	0	1.031	0	0	0	0	0	0	0	0	0.943	0	1.85	0	0	1
Os03g0782300	0	0	0	0	0	0	0	0	0	0	0	0	0	0	0	0	2.061	0	0	0	0	0	0	0	0	0.943	0	0	0	0	1
Os03g0729000	632	416	820	82.1	65.09	153.1	32.9	50.6	26.3	1880	1346	973.7	1950.6	1360	1559.4	692.6	204	297.3	108.7	71.76	106.9	17.75	20.67	10.83	650.7	297	576.7	456	471	532	4
Os03g0744650	328	335	315	296.4	252.2	270.8	223	248	216	329.4	255.5	336.8	311.32	263.23	335	513.5	304	347.1	262	298.3	227.6	283.9	274.4	291.8	379.2	307.4	342.1	242	320	318	13
Os03g0142900	1695	1610	1788	1769	1459	1500	2110	2244	2484	1730	1771	1500	1483.7	1534.6	1953	2328	1539	1517	2182	2008	2046	2478	2441	2474	1689	1616	1638	1602	1672	1572	18
Os03g0744200	9.18	10.2	6.47	45.15	37.06	49.31	21.1	16.4	9.67	10.35	23.02	5.645	14.151	15.869	21.383	35.82	13.4	13.47	46.45	37.09	56.91	23.66	16.39	19.5	15.85	11.31	17.54	30.5	21	15.8	10
Os03g0799300	2.04	0	0	0	0	0	0	0.63	2.76	0.862	0	0	0	0.9335	0	0	1.031	4.144	0.929	0	0.862	0	0	0	1.056	0.943	8.772	0	0	0	11
Os03g0780600	362	198	261	133	82.26	104.7	122	98	97.4	303.5	279.6	339.6	259.07	336.98	262.93	191.1	339	239.3	117	116.9	152.6	83.55	62.71	97.5	276.8	313	293.9	193	208	197	3
Os03g0626800	32.6	59.6	31.2	48.44	60.57	38.06	18.8	36.7	29	19.83	25.22	14.11	32.656	29.87	22.967	0	37.1	33.15	14.86	20.96	30.18	34.01	25.65	32.5	26.41	12.26	19.74	30.5	38.3	39.2	2
Os03g0818000	84.6	110	98.1	123.1	93.11	89.96	120	173	130	84.49	89.9	85.61	111.03	106.41	117.21	214.9	96.87	103.6	105	84.66	80.19	119	115.4	104	105.6	77.31	92.1	116	102	117	6
Os03g0575200	1235	1233	1201	389.1	454.7	342.6	623	1049	661	1426	1263	987.9	1064.6	1204.2	1152.3	1648	938.8	1070	347.4	424.1	368.2	703.2	927.9	699.1	1439	1258	1020	1046	1009	1022	3
Os03g0765100	337	408	407	391.6	405	407.4	712	757	746	448.3	453.9	443.1	459.36	435.92	500.52	382.1	346.3	322.2	475.6	422.5	419	509.4	502.4	608.1	466.9	448.8	460.5	379	372	367	18
Os03g0341000	0	0.93	0	4.105	1.808	0	0	0	0	0	0	0	0	0	0	0	0	0	4.645	0.806	0.862	0	0	0	0	0	0	0	0.96	0	10
Os03g0647500	37.7	39.1	40.9	124.8	137.4	123.7	133	105	102	16.38	27.41	33.87	29.39	36.405	24.551	71.65	34.01	33.15	115.2	126.6	153.5	65.81	70.55	93.89	24.3	23.57	32.89	19.4	24.9	30	12
Os03g0777100	31.6	27	47.4	69.78	66.89	60.55	116	142	122	34.49	55.92	55.51	54.426	52.273	49.894	95.53	29.89	30.05	64.1	75.79	61.22	115.3	124	92.45	57.04	63.17	41.67	31.5	34.4	27.5	18
Os03g0194300	24.5	15.8	11.9	12.31	17.18	6.055	22.7	13.3	6.91	13.79	17.54	31.05	17.416	12.135	15.047	11.94	22.67	6.216	15.79	11.29	17.24	6.654	7.839	17.33	8.451	13.2	21.93	14.8	15.3	6.66	20
Os03g0107100	0	0	0	0	0	0	0.78	0	0	0	0	0	0	0	0	0	0	0	0	0	0	0.739	0	0.722	0	0	0	0	0	0	9
Os03g0796700	4.08	12.1	11.9	9.852	9.944	13.84	4.7	6.96	9.67	11.21	6.578	2.822	1.0885	2.8004	8.7116	11.94	11.34	1.036	7.432	8.869	9.484	13.31	16.39	10.11	7.395	0	2.193	9.25	0.96	13.3	17
Os03g0405100	554	457	580	368.6	395.9	375.4	382	392	373	360.4	423.2	375.4	532.29	646.88	475.97	1111	566.8	495.2	460.8	440.2	406.1	254.3	281.5	322.1	409.9	463.9	526.3	414	440	492	13
Os03g0727800	12.2	7.45	6.47	2.463	0.904	1.73	0.78	0.63	0.69	14.66	16.45	7.527	10.885	14.002	22.175	0	0	2.072	0	0	0.862	0	0.713	2.167	23.24	6.6	8.772	10.2	6.69	3.33	3
Os03g0403501	0	0	0	0	0	0.865	0	0	0	0	0	0	0	0	0	0	0	0	0	0	0	0	0	0	0	0	0	0	0	0	2
Os03g0577000	2113	1720	1784	2148	2219	2207	2266	2004	2501	2266	2123	3284	2216.2	2010.7	1765.3	4335	1636	1879	2571	2131	2505	1843	1995	2048	1893	2555	2610	1446	1719	1271	6
Os03g0379100	207	203	184	155.2	120.2	205	144	121	140	299.2	282.9	394.2	183.96	192.29	175.82	167.2	163.9	194.8	209	190.3	259.5	99.08	114.7	132.9	153.2	202.7	250	181	219	159	20
Os03g0143000	358	326	350	388.3	389.6	358.1	239	254	252	238.8	288.4	248.4	314.58	363.11	349.26	776.2	384.4	384.4	280.5	324.1	388	381.5	347.1	311.3	327.5	305.5	293.9	439	412	450	6
Os03g0196900	298	246	334	182.3	206.1	192.9	201	172	198	401.8	415.5	464.8	419.08	427.52	459.34	394.1	305	300.5	229.5	249.9	208.7	160.4	175.3	166.1	461.6	398.8	451.7	340	372	322	3
Os03g0182700	688	605	665	777.5	813.6	868.5	826	704	860	838	634.8	1103	650.93	756.1	614.57	943.3	648.2	658.9	866.7	774	900.2	752	763.9	704.2	721.5	810.8	857.4	521	609	468	15
Os03g0804500	33.6	28.9	44.2	32.02	29.83	41.52	120	63.9	150	49.14	38.37	57.39	19.593	24.27	21.383	23.88	34.01	40.41	43.66	16.93	25.87	261	251.6	241.2	19.01	28.29	37.28	28.7	35.4	13.3	9
Os03g0158000	1.02	0	1.08	13.96	9.04	11.25	6.26	6.96	13.1	4.311	2.193	7.527	0	1.8669	1.5839	11.94	1.031	0	9.29	12.9	6.898	6.654	12.11	7.222	3.169	1.886	0	1.85	2.87	1.67	12
Os03g0701000	970	1073	1097	1102	1302	1125	709	843	704	831.1	998.8	786.5	1103.8	883.05	1056.5	1934	1095	1082	852.8	969.2	942.4	958.2	814.5	827.7	1022	857	752.2	1008	1018	1115	6
Os03g0756300	4.08	3.73	14	9.031	5.424	12.11	5.48	5.69	13.1	14.66	10.96	21.64	8.7082	16.802	9.5036	0	13.4	8.288	7.432	4.838	7.76	42.15	19.24	18.06	7.395	7.543	8.772	6.48	3.83	7.5	9
Os03g0756000	1478	1325	1307	1673	1795	1773	2173	1770	2306	1646	1522	2384	1724.2	1713.8	1369.3	2269	1186	1373	1880	1695	1805	1548	1522	1646	1364	1793	1822	1104	1244	967	15
Os03g0803000	2216	2359	2120	1814	1817	1963	2374	2462	2539	2224	2055	2041	1935.4	2076.9	2173.9	2209	2125	1948	1494	1519	1436	2475	2503	2488	2263	2114	2324	2169	1799	2202	5
Os03g0790500	7.14	1.86	1.08	19.7	4.52	29.41	15.7	22.8	44.9	6.035	5.482	10.35	8.7082	0	3.1679	0	6.183	3.108	8.361	4.031	46.56	19.22	21.38	19.5	2.113	2.829	2.193	0	1.91	0.83	12
Os03g0639233	1.02	2.79	5.39	1.642	1.808	0	0	0	0	4.311	0	0	0	6.5342	1.5839	0	2.061	1.036	1.858	3.225	0.862	0.739	2.138	1.444	4.225	1.886	0	2.78	3.83	5	1
Os03g0267100	6.12	4.66	6.47	10.67	2.712	5.19	21.9	19.6	24.9	22.42	12.06	5.645	9.7967	14.935	15.047	0	10.31	8.288	10.22	10.48	15.52	3.697	12.11	10.11	8.451	5.657	8.772	2.78	6.69	8.33	15
Os03g0178200	439	505	478	793.1	881.4	744.8	1080	1075	1028	456.1	626	685.9	600.86	468.59	434.79	776.2	446.2	496.3	937.3	973.2	968.3	613.7	595.1	851.5	503.9	414.9	311.4	332	446	399	12
Os03g0193700	0	0	0	0	0	0	0	0	0	0	0	0	0	0	0	0	0	0	0	0	0	0.739	0	0	0	0	0	0	0	0	9
Os03g0644000	528	540	499	454.8	526.1	561.4	480	540	470	539.7	530.7	466.6	513.78	580.61	556.75	859.7	561.6	524.2	537.9	516.8	563	516.8	528.8	470.9	565.2	470.5	543.8	589	563	552	6
Os03g0113000	5.1	6.52	2.16	0.821	1.808	7.785	4.7	3.16	3.46	7.76	10.96	10.35	3.2656	4.6673	3.1679	0	7.214	7.252	1.858	1.613	2.587	2.958	4.276	10.11	7.395	10.37	19.74	0.93	4.78	4.17	11
Os03g0348200	7124	6586	3460	1427	1277	2177	4744	7334	7205	11597	12021	12857	2688.6	4264.9	2990.5	2328	7403	5574	4001	2104	2700	12487	11923	11854	7840	15960	24521	5896	7524	5666	11
Os03g0321000	353	291	337	211.8	202.5	219.7	284	241	263	319	279.6	286	252.54	264.17	269.27	501.5	320.5	300.5	257.3	263.7	238.8	229.9	243.7	236.9	231.3	306.4	350.9	237	233	222	13
Os03g0128500	20.4	19.6	20.5	103.4	67.8	104.7	63.4	55	76.7	13.79	27.41	32.93	16.328	35.471	16.631	23.88	16.49	34.19	78.03	64.5	80.19	68.76	67.7	58.5	14.79	26.4	15.35	33.3	28.7	21.7	12
Os03g0253100	2013	2235	2177	1853	1836	2004	1581	1706	1435	1656	1785	1457	2100.8	2294.4	2289.6	4084	2339	2353	1791	2133	1791	2252	2041	1743	2112	1923	1932	2901	2506	2654	13
Os03g0821250	993	1061	1079	652.7	723.2	788.9	2147	2204	2336	1297	1663	1930	1390	1385.2	1245	1731	1395	1299	907.6	781.3	868.3	1953	1660	1969	1617	1803	1914	927	1120	1125	5
Os03g0139100	2457	2036	2010	3149	3120	3374	3745	3066	4280	2820	2543	4113	2921.6	2989.8	2121.7	2830	1954	2107	3909	3305	3731	2620	2693	2849	2474	2971	3298	1618	2159	1496	15
Os03g0806500	466	505	454	627.2	694.2	529.4	366	354	350	416.4	453.9	442.2	537.73	438.72	491.02	740.3	569.9	508.7	521.1	567.6	509.6	425.9	342.8	383.5	441.6	403.5	425.4	448	419	544	6
Os03g0762800	0	7.45	1.08	5.747	8.136	6.055	12.5	14.5	10.4	0	0	2.822	0	1.8669	6.3357	35.82	3.092	4.144	2.787	12.09	9.484	7.394	9.977	8.667	4.225	2.829	0	1.85	5.74	4.17	6
Os03g0226700	330	335	377	325.9	377	355.5	517	481	477	494.9	573.4	625.6	502.9	426.59	427.66	609	399.8	454.8	490.5	455.5	518.2	255.8	298.6	312	435.2	433.7	425.4	356	369	350	20
Os03g0110700	0	0.93	0	0	0	0	0	0	0	0	0	0	0	0	0	0	0	0	0	0	0	0	0	0	0	0	0	0	0	0	14
Os03g0185500	217	181	197	253.7	198.9	227.5	115	157	168	188.8	186.4	170.3	267.78	232.43	217	286.6	211.3	218.6	217.4	299.1	273.3	241	203.8	164.7	198.6	201.8	225.9	222	190	198	16
Os03g0770800	8.16	5.59	2.16	10.67	3.616	13.84	15.7	23.4	17.3	1.724	0	0.941	2.177	6.5342	0	0	6.183	6.216	1.858	6.45	12.07	2.218	9.977	9.389	4.225	1.886	0	0.93	2.87	0	18
Os03g0338800	0	0	1.08	1.642	0	0	2.35	3.16	3.46	0	0	0	0	0	0.792	0	0	1.036	0	0.806	0	0.739	0.713	2.889	0	0	0	0	0	0	18
Os03g0191700	0	0	0	0.821	0.904	1.73	0	0	0	0	0	0	0	0	0	0	0	0	0	0	0	0	0	0	0	0	0	0	0	0	2
Os03g0167700	267	293	292	219.2	218.8	225.8	236	223	218	224.2	223.7	223	267.78	278.17	281.94	310.5	314.3	285.9	172.8	162.9	158.6	220.3	218.8	218.8	296.8	278.1	265.3	300	283	323	13
Os03g0712300	645	635	589	559.1	620.1	645.3	556	509	461	713	584.4	634.1	512.69	573.14	574.97	800	547.2	540.8	737.6	593.4	653.6	352.7	416.2	446.3	531.4	545.9	679.8	477	560	462	16
Os03g0801600	1481	1905	1649	2142	2252	1974	1936	1863	1822	1779	1608	1627	1540.3	1781	1669.5	2125	1538	1570	1717	1647	1758	1857	1849	1914	1729	1483	1612	1550	1455	1669	2
Os03g0261900	420	388	392	577.1	586.7	581.3	216	227	236	260.4	241.2	228.6	369.01	368.71	332.63	668.7	358.6	384.4	510	592.6	593.2	348.3	331.4	256.4	285.2	368.7	291.7	410	370	376	10
Os03g0232800	479	309	342	181.4	197.1	167.8	7.05	17.1	11.1	407	280.7	635.1	277.57	296.84	233.63	823.9	476.1	450.7	226.7	179.8	183.7	7.394	11.4	14.44	376.1	472.4	526.3	407	413	290	13
Os03g0220700	1123	1056	1016	752	801.8	766.4	839	820	884	849.3	827.8	819.5	810.95	820.5	994.71	1719	1023	965.6	589	711.9	657.9	1458	1167	977.9	928.5	982.4	982.4	1180	1200	1182	13
Os03g0151500	0	1.86	0	3.284	9.04	5.19	0.78	1.27	2.76	0.862	0	0	0	5.6007	2.3759	0	0	0	2.787	3.225	1.724	3.697	3.563	2.167	1.056	0	0	0	2.87	0	2
Os03g0810300	202	145	237	98.52	108.5	98.62	121	129	113	180.2	221.5	219.2	253.63	295.9	260.56	549.3	318.4	290.1	127.3	123.4	138	130.9	114	95.34	290.5	252.7	188.6	271	221	321	13
Os03g0274800	182	183	172	143.7	125.7	143.6	275	292	225	284.5	265.3	233.3	167.63	231.5	259.76	214.9	182.4	170.9	154.2	175.8	159.5	227	327.1	234.7	299	233.8	285.1	294	209	224	5
Os03g0158600	33.6	31.7	33.4	54.18	56.95	51.04	7.83	13.9	22.8	11.21	24.12	11.29	32.656	27.07	29.303	35.82	25.76	32.12	26.94	20.96	29.32	19.22	23.52	14.44	20.07	16.97	19.74	21.3	23	19.2	2
Os03g0636300	4.08	4.66	5.39	3.284	5.424	2.595	6.26	4.43	2.76	6.035	1.096	12.23	5.4426	5.6007	4.7518	0	4.122	3.108	4.645	2.419	6.898	0.739	7.126	2.167	3.169	3.771	2.193	7.4	3.83	3.33	20
Os03g0140900	74.4	54	70	38.59	52.43	38.06	39.9	88.6	63.6	59.49	37.28	20.7	50.072	78.41	35.638	95.53	55.65	93.24	25.08	38.7	36.21	68.02	67.7	51.28	38.03	35.83	46.05	75.9	74.6	58.3	8
Os03g0262300	214	266	205	263.5	247.7	260.4	149	144	155	147.4	148	133.6	171.99	144.68	254.22	370.2	236	252.8	177.4	229.8	233.7	220.3	208.8	210.9	184.9	187.6	153.5	248	264	297	8
Os03g0591600	0	0	1.08	0.821	0	0	0.78	0	0	1.724	1.096	0	0	0	2.3759	11.94	2.061	3.108	0	0	0	0	0	3.611	0	2.829	0	0.93	0	0	6
Os03g0708500	290	373	284	2136	2217	2119	4136	3767	4795	350	410.1	362.2	399.49	294.04	304.11	382.1	311.2	339.8	3064	2652	3164	2174	2262	3306	304.2	298.9	223.7	307	462	370	12
Os03g0803700	710	769	778	630.5	629.2	687.7	425	488	441	744.1	678.7	695.3	939.39	721.56	812.56	1087	714.2	742.8	546.2	687.8	685.5	559.7	555.1	577.1	733.1	786.3	627.2	819	762	813	13
Os03g0240900	382	418	374	378.5	424.9	365.1	413	433	381	329.4	402.4	341.5	396.22	346.31	392.02	764.2	371	336.7	352.1	398.3	354.4	394.8	406.2	383.5	389.8	424.3	379.4	387	366	375	6
Os03g0196250	16.3	24.2	12.9	3.284	9.04	7.785	14.9	12.7	11.7	13.79	33.99	40.46	5.4426	12.135	6.3357	0	17.52	9.324	8.361	7.257	15.52	11.83	0	13.72	9.507	28.29	35.09	8.33	2.87	7.5	20
Os03g0168200	1551	1278	1636	3076	3261	3370	1359	1483	1733	1913	1717	1256	3065.3	3243.7	3010.3	3105	1927	1576	3370	3471	3569	1037	1370	892.7	1855	1344	1331	2221	2010	2109	16
Os03g0240700	1150	834	1072	508.2	507.1	547.6	634	612	565	916.5	834.4	847.7	900.21	885.85	860.87	1170	843	859.9	696.7	643.4	557.9	482.1	569.4	546	933.8	783.5	958.3	801	806	646	13
Os03g0777000	40.8	47.5	42	77.99	74.13	65.74	71.2	112	71.2	47.42	33.99	32.93	33.744	35.471	67.317	59.7	61.83	48.69	60.38	45.15	25	85.77	123.3	61.39	25.35	32.06	39.47	52.7	42.1	44.2	17
Os03g0178550	11.2	21.4	24.8	14.78	30.73	26.82	25.8	26.6	24.2	13.79	20.83	11.29	8.7082	5.6007	9.5036	0	10.31	20.72	21.37	34.67	18.97	24.4	31.36	21.67	13.73	13.2	32.89	8.33	23.9	7.5	12
Os03g0577300	1.02	12.1	11.9	8.21	1.808	6.92	19.6	14.5	13.8	0.862	9.868	8.467	2.177	4.6673	4.7518	0	9.275	5.18	13.93	6.45	2.587	3.697	9.977	10.11	5.282	3.771	8.772	4.63	5.74	5.83	18
Os03g0752400	45.9	37.3	63.6	3.284	0.904	0.865	12.5	3.8	13.1	13.79	33.99	29.17	58.78	43.872	30.887	83.59	68.02	36.26	0	0	0	9.612	2.138	20.22	29.58	49.97	32.89	87	70.8	77.5	13
Os03g0271100	853	932	879	1524	1652	1551	2790	2922	3076	1242	1267	1629	1288.8	1085.6	1002.6	895.6	960.5	954.2	1489	1591	1728	2124	1946	2504	1299	1254	1153	784	848	891	18
Os03g0730400	1.02	1.86	2.16	0	0	0	0	0	0	0	0	1.882	0	0.9335	0	0	0	0	4.645	0	0	0	0.713	7.222	1.056	0.943	6.579	0.93	2.87	0	11
Os03g0235700	69.3	77.3	70	36.12	28.93	42.39	22.7	18.3	26.9	49.14	40.57	54.57	25.036	26.137	31.679	47.76	55.65	55.95	22.29	45.96	23.28	44.36	52.02	35.39	41.2	45.26	65.79	58.3	64.1	40.8	14
Os03g0781700	37.7	60.5	40.9	237.3	207	317.5	43.8	42.4	41.5	36.21	25.22	37.63	22.859	35.471	23.759	119.4	55.65	47.66	190.4	195.9	224.2	76.16	58.44	56.34	29.58	53.74	78.95	38.9	47.8	43.3	10
Os03g0230500	3117	3160	3404	585.4	554.1	624.6	211	261	225	2823	3296	2833	3924.1	3352	3743.6	8096	3376	3644	757.1	769.2	639.8	251.4	258	239.1	3852	3507	2956	3912	4059	4416	13
Os03g0808900	0	0	1.08	0.821	0	2.595	7.05	0.63	0	0	0	1.882	1.0885	0	0	0	0	0	6.503	5.644	11.21	0	0.713	3.611	0	0	0	3.7	0	0	16
Os03g0102700	2.04	1.86	0	0	0	0	0	0	0	0	0	0	0	0	0	0	0	0	0	0	0	0	0	0	0	0	0	0	0	0	14
Os03g0112700	1245	1182	1234	927.7	786.5	824.4	999	958	1117	1115	1043	958.7	1063.5	1032.4	1160.2	1743	1253	1169	803.5	869.2	717.4	1315	1266	1248	1236	1147	1121	1299	1115	1102	13
Os03g0805500	923	855	879	546.8	518	582.2	543	698	728	887.2	961.5	861.8	869.73	1051.1	1142.8	1827	1120	992.5	628.9	649.1	661.3	695	934.3	841.4	1087	1000	1200	1211	1229	1166	13
Os03g0102366	6.12	14.9	11.9	16.42	18.98	21.63	5.48	18.3	8.29	11.21	17.54	10.35	8.7082	4.6673	8.7116	35.82	11.34	9.324	24.15	28.22	25	19.22	12.83	20.22	15.85	11.31	10.96	10.2	12.4	12.5	6
Os03g0655400	####	####	####	8664	8890	8024	5872	9109	8151	14909	12946	20569	7116.7	12279	11950	16455	22267	20511	12807	6911	8781	5563	6109	9366	18496	19918	18688	####	####	####	3
Os03g0807600	0	0	0	0.821	0	0	0	0	0	0	0	0	0	0	0	0	0	0	0	0	0	0	0	0	0	0	0	0	0	0	2
Os03g0271500	446	376	393	445.8	537	407.4	669	632	661	283.7	398	493	445.2	474.19	362.72	417.9	473	480.7	617.8	624.9	603.6	439.9	382.7	577.1	422.5	372.4	353.1	306	314	299	12
Os03g0148950	0	0	0	0	0	0	0	0	0.69	0	0	0	0	0	0	0	0	0	0	0	0	0	0	0	0	0	0	0	0	0	18

Os03g0105700	153	152	164	212.6	260.3	204.2	189	178	189	163	161.2	152.4	165.46	169.89	171.06	203	148.4	119.1	217.4	200	233.7	147.9	171	168.3	183.8	137.7	153.5	147	163	151	10
Os03g0269700	38.7	41	29.1	6.568	17.18	16.44	9.39	5.06	7.6	60.35	67.98	81.85	44.629	44.806	45.934	131.4	95.84	69.41	57.6	53.21	83.64	5.915	7.126	10.11	77.11	120.7	111.8	68.5	76.5	55.8	13
Os03g0266900	302	137	206	15.6	12.66	35.47	11.7	7.59	8.98	173.3	116.2	241.8	253.63	252.03	236.01	203	82.44	77.7	32.51	19.35	27.59	6.654	16.39	9.389	144.7	109.4	142.5	89.7	134	86.6	3
Os03g0303100	68.3	54	52.8	41.05	44.29	25.95	3.13	4.43	0	72.42	70.17	61.15	75.108	43.872	35.638	47.76	49.47	33.15	17.65	25.8	25.87	5.176	1.425	2.889	57.04	67.88	50.44	24.1	34.4	35.8	3
Os03g0792500	1106	1102	1141	980.2	998.9	891.9	918	960	893	1108	1056	1017	1441.2	1206	1263.2	1624	1121	1127	941	958.7	919.1	812.6	855.2	886.9	1154	993.8	1055	1181	1183	1241	13
Os03g0372900	3.06	0	0	0	0	0	0	3.16	0	6.035	1.096	4.704	1.0885	0.9335	0	0	3.092	1.036	0.929	0	0	1.479	0	0	2.113	1.886	4.386	0	0.96	0.83	20
Os03g0401300	8.16	2.79	6.47	17.24	21.7	32.01	14.9	10.8	20	14.66	26.31	12.23	2.177	9.3345	6.3357	0	1.031	2.072	12.08	30.64	33.63	5.915	14.97	7.222	1.056	2.829	2.193	3.7	0.96	2.5	10
Os03g0292800	530	458	466	344.8	359.8	436.8	428	405	379	518.2	410.1	597.4	459.36	630.08	475.97	943.3	623.5	451.7	488.6	424.1	445.8	319.4	337.1	378.5	460.6	463.9	598.7	444	469	444	13
Os03g0589100	6.12	3.73	7.54	0	0	0.865	1.57	0	0	4.311	1.096	1.882	1.0885	3.7338	7.1277	0	13.4	4.144	0.929	1.613	0	0	0	0	2.113	1.886	0	0	1.91	2.5	14
Os03g0666100	324	391	351	354.7	369.7	281.1	312	304	315	311.2	335.5	273.8	262.33	252.03	361.93	561.2	282.4	300.5	264.8	252.4	227.6	342.3	387.7	333.7	310.6	347	300.4	295	347	270	6
Os03g0670100	1212	1285	1379	1286	1360	1153	946	1147	1139	1525	1670	1389	1360.6	1353.5	1458	1684	1209	1191	1445	1428	1580	963.4	976.3	1139	1314	1429	1528	1215	1359	1357	16
Os03g0376600	491	516	505	2564	2961	2311	1805	1750	1474	467.3	464.9	502.4	695.56	578.74	327.87	632.9	388.5	499.4	2767	3227	3013	862.9	930.7	1207	297.9	248	245.6	323	351	309	10
Os03g0357500	0	0	0	0	0	0	0	0	0	0	0	0	0	0	0	0	0	0	0	0	0	1.479	1.425	0.722	0	0	2.193	0.93	0	0	11
Os03g0689300	2.04	2.79	1.08	3.284	0	3.46	0.78	0.63	2.07	1.724	4.386	0	0	2.8004	4.7518	0	3.092	3.108	3.716	2.419	0	0.739	0.713	0.722	2.113	0.943	17.54	2.78	0.96	3.33	11
Os03g0146400	1108	1240	1403	1763	1484	2021	1004	1169	1262	1667	1744	1389	2330.5	2083.5	2203.2	2949	1354	1190	1984	1272	1733	804.5	832.4	777.1	1508	1313	1550	1451	1449	1556	13
Os03g0211800	203	173	181	206.9	145.5	296.7	126	135	139	122.4	126.1	121.4	106.67	143.75	112.46	501.5	225.7	169.9	228.5	161.3	216.4	232.9	243.7	275.2	121.5	188.6	287.3	156	211	177	6
Os03g0392050	264	251	298	403.1	418.5	429.9	509	484	536	302.6	326.7	367.9	265.6	330.44	259.76	429.9	283.4	219.6	492.3	449.1	440.6	347.5	387	346.7	265.1	252.7	344.3	233	207	203	12
Os03g0650200	61.2	64.3	84.1	36.12	47.01	12.11	0	5.69	1.38	253.5	199.5	138.3	171.99	104.55	114.04	155.2	73.17	85.99	44.59	33.86	12.93	2.218	2.138	2.167	90.85	55.63	76.75	77.7	63.1	72.5	3
Os03g0142800	722	811	817	500	477.3	507.8	575	547	574	591.5	658.9	681.2	862.11	673.02	746.03	1158	727.6	668.2	453.3	399.9	430.2	488	596.5	509.9	717.3	564.8	666.6	829	751	790	13
Os03g0757600	20.4	23.3	21.6	32.84	31.64	28.55	11.7	18.3	13.8	29.31	19.74	7.527	31.567	17.736	25.343	71.65	18.55	17.61	15.79	31.44	49.15	21.44	12.11	14.44	17.96	15.09	21.93	21.3	27.7	40	6
Os03g0307333	8.16	8.38	8.62	5.747	9.04	6.055	37.6	23.4	23.5	18.97	12.06	18.82	16.328	8.4011	15.839	35.82	12.37	9.324	25.08	12.09	21.56	12.57	22.09	10.83	8.451	12.26	8.772	15.7	17.2	17.5	6
Os03g0109700	1163	1070	1192	770.1	810	791.5	988	1090	1076	1330	1398	1696	1400.9	1327.4	1399.4	1755	1303	1309	898.3	844.2	909.6	728.3	708.4	868.1	1499	1607	1445	1129	1300	1310	3
Os03g0726200	483	457	449	1326	963.6	1689	644	1005	1115	452.6	449.5	436.5	589.98	488.2	537.74	644.8	506	416.5	1112	995.8	1199	1302	997	1114	573.6	502.5	574.5	456	494	525	12
Os03g0400700	14.3	6.52	3.23	21.35	6.328	32.01	8.61	10.1	22.8	8.622	6.578	35.75	7.6196	5.6007	4.7518	0	2.061	7.252	48.31	10.48	20.69	19.96	17.1	26.72	10.56	41.49	100.9	7.4	4.78	3.33	11
Os03g0376000	30.6	28.9	19.4	22.99	32.54	24.22	24.3	31.6	20.7	21.55	20.83	16.93	17.416	17.736	23.759	23.88	22.67	22.79	33.44	20.16	35.35	22.92	25.65	20.95	23.24	28.29	26.32	18.5	10.5	17.5	10
Os03g0638900	16.3	20.5	14	14.78	9.944	10.38	35.2	42.4	24.9	53.46	29.6	77.15	29.39	16.802	26.135	0	19.58	12.43	22.29	7.257	26.73	34.01	19.95	33.22	23.24	64.11	116.2	13	23.9	24.2	11
Os03g0441601	192	197	256	220	302.8	269	215	198	220	181.1	222.6	207	217.7	209.09	201.95	310.5	238.1	247.6	270.3	245.1	354.4	195.9	190.3	203	210.2	215.9	232.4	215	166	202	16
Os03g0302100	15.3	10.2	15.1	11.49	6.328	6.92	2.35	1.9	2.07	7.76	3.289	9.408	5.4426	6.5342	9.5036	11.94	12.37	10.36	2.787	4.838	9.484	14.05	5.701	2.167	2.113	8.486	6.579	13	11.5	9.16	8
Os03g0594600	3.06	0.93	1.08	1.642	0.904	0.865	4.7	0.63	7.6	0	7.675	0.941	1.0885	0.9335	0.792	0	2.061	3.108	1.858	4.031	3.449	5.176	6.414	5.778	3.169	0.943	4.386	0.93	1.91	3.33	9
Os03g0840800	0	0	0	0	0	0	3.91	0.63	0.69	0	0	0	0	0	0	0	0	1.036	0.929	0	0	2.218	3.563	1.444	0	0.943	0	1.85	0	0	17
Os03g0772800	1107	1036	1026	1239	1282	1187	1542	1202	1504	1370	1317	1393	1168	1217.2	1062.8	1493	1017	1059	1655	1478	1620	1024	1181	1407	1164	1021	1270	1001	1021	951	15
Os03g0818400	585	574	539	830	888.6	893.6	1284	1104	1164	616.5	483.5	705.6	422.35	451.79	331.83	776.2	352.4	417.5	814.7	749.8	805.3	683.2	687	846.5	334.9	441.2	434.2	289	289	266	12
Os03g0576400	38.7	42.8	38.8	50.08	54.24	48.44	13.3	15.2	23.5	38.8	33.99	31.99	46.806	27.07	49.894	35.82	51.53	50.77	17.65	20.96	31.04	39.93	47.75	24.56	49.65	30.17	26.32	61.1	39.2	50	8
Os03g0741000	38.7	15.8	23.7	37.76	20.79	38.06	1.57	8.22	6.22	8.622	12.06	0	2.177	3.7338	26.135	23.88	14.43	16.58	7.432	19.35	6.898	82.07	57.01	21.67	7.395	16.03	19.74	50	36.3	35	17
Os03g0335500	0	0	0	0	0	0	0	0	0	0	0	2.822	0	0.9335	0	0	0	0	0	0	0	1.479	0	0	0	0	0	0	0	0	20
Os03g0708400	3192	3043	3227	1813	2054	1956	1661	2180	1542	2537	2625	3170	3582.3	3671.3	3206.7	4681	4051	3408	2156	2316	1998	1458	1928	1874	3254	3307	3105	3237	2946	3362	13
Os03g0201700	46.9	64.3	44.2	109.2	146.4	108.1	43.1	41.7	33.2	40.52	23.02	38.57	35.921	32.671	26.135	0	38.13	56.98	78.96	80.63	81.91	62.85	74.83	62.84	17.96	33.94	39.47	42.6	29.6	40	10
Os03g0837700	0	0	1.08	12.31	7.232	11.25	0.78	1.27	2.07	3.449	0	0.941	5.4426	2.8004	1.5839	0	1.031	4.144	17.65	17.74	16.38	0	0	0.722	0	0	0	0.93	0.96	1.67	10
Os03g0389000	13.3	20.5	16.2	6.568	2.712	12.98	4.7	3.8	6.22	16.38	12.06	15.05	14.151	10.268	11.088	23.88	12.37	13.47	11.15	10.48	0.862	11.09	3.563	6.5	22.18	12.26	19.74	9.25	12.4	10	13
Os03g0788200	449	308	369	171.6	164.5	236.2	342	178	196	469.9	450.6	554.1	480.04	523.67	371.43	740.3	379.2	328.4	286.1	240.3	294	108.7	117.6	180.6	449	462.9	471.5	351	330	308	13
Os03g0792800	93.8	50.3	60.3	14.78	15.37	33.74	13.3	13.9	16.6	21.55	29.6	26.34	31.567	39.205	15.047	83.59	102	74.59	28.8	24.99	37.08	22.18	17.1	30.33	27.47	44.31	19.74	38.9	32.5	35.8	13
Os03g0180400	1207	1278	1195	1612	1710	1747	2335	2087	2237	1501	1355	1614	1380.2	1585.9	1352.7	1588	1324	1102	1990	1591	1721	1483	1599	1563	1347	1310	1557	1184	1243	1150	12
Os03g0322600	3408	3249	3568	5253	5502	5464	2248	2257	2262	3330	3647	3802	4329	4645.8	3778.5	5290	4276	3528	6469	5567	7486	1734	1648	1911	3759	3698	3800	3213	3330	3681	16
Os03g0170100	76.5	55	70	13.14	2.712	11.25	9.39	6.33	24.2	9.484	3.289	1.882	14.151	1.8669	4.7518	23.88	57.71	49.73	8.361	9.675	4.311	19.22	19.24	60.67	5.282	3.771	0	5.55	3.83	4.17	14
Os03g0262100	275	218	262	257	264.9	256.9	326	306	361	283.7	278.5	248.4	273.22	269.77	266.89	513.5	222.6	279.7	286.1	290.3	286.3	246.2	247.3	327.9	271.5	297.9	217.1	240	261	233	6
Os03g0247100	524	489	525	505.7	493.6	500.9	393	435	445	714.8	705	771.5	624.81	603.01	679.51	967.2	541	583.3	518.4	532.1	497.5	340.1	426.2	402.3	768	715.6	587.7	513	580	597	13
Os03g0314750	0	0.93	2.16	0	0.904	0	0	0	1.38	0	3.289	0	2.177	0	0	0	0	0	0	0	0	0	2.138	0	0	0	0	0	1.91	1.67	4
Os03g0785800	6.12	3.73	3.23	6.568	7.232	11.25	30.5	7.59	8.29	0.862	2.193	8.467	1.0885	3.7338	1.5839	0	7.214	5.18	5.574	11.29	6.036	10.35	5.701	10.83	6.338	3.771	8.772	5.55	6.69	1.67	15
Os03g0300100	0	0	0	0	0	0	1.57	0	2.07	0	0	0	0	0	0.792	0	0	0	0	0	0	0.739	2.851	5.778	0	0	0	1.85	0	0.83	9
Os03g0718600	315	201	279	245.5	232.3	263	396	354	471	297.5	358.5	348.1	353.77	384.58	402.32	525.4	336	239.3	294.5	281.4	374.2	338.6	286.5	327.2	346.5	350.7	405.7	263	291	277	6
Os03g0583800	6865	7121	8211	858.7	539.7	1073	1703	2275	2198	10936	13486	10256	13490	11722	14175	10317	9303	8053	1565	1032	1177	1670	1687	1829	11697	11748	10140	9100	8008	####	3
Os03g0196800	2383	2514	2496	3679	4190	3497	6388	5967	6078	3064	3454	5152	3054.4	2554.9	2268.2	3785	2123	2293	4285	3714	4494	3151	2938	4290	2522	2593	2513	1343	1703	1728	18
Os03g0201000	16.3	20.5	16.2	22.17	16.27	25.95	11.7	6.33	10.4	22.42	10.96	14.11	10.885	28.937	15.047	83.59	23.7	38.33	26.01	16.93	13.8	18.48	19.95	21.67	24.3	21.69	28.51	18.5	17.2	15.8	6
Os03g0104100	179	223	218	211.8	232.3	205	413	523	359	176.7	193	266.3	198.11	168.02	194.82	310.5	251.4	191.7	278.7	327.4	282.8	382.3	349.2	457.2	208.1	234.8	228.1	247	196	197	18
Os03g0556600	16.3	15.8	16.2	13.14	7.232	6.055	16.4	9.49	18.7	9.484	5.482	0	7.6196	3.7338	9.5036	11.94	6.183	7.252	2.787	3.225	2.587	43.62	33.49	52.72	3.169	5.657	0	11.1	1.91	9.16	9
Os03g0744501	0	0	0	0.821	0	0	0	0	0	0	0	0	0	0	0	0	0	0	0	0	0	0	0	0	0	0	0	0	0	0	2
Os03g0291500	7.14	16.8	4.31	2.463	4.52	0.865	13.3	22.8	11.7	12.93	5.482	0.941	0	11.201	3.1679	11.94	2.061	2.072	0.929	4.031	1.724	11.83	12.11	3.611	7.395	0	6.579	0	7.65	2.5	18
Os03g0220300	177	141	180	165.8	148.3	153.1	313	240	218	208.6	199.5	189.1	182.87	180.16	175.82	346.3	157.7	202	157	191.1	190.5	253.6	244.4	247	176.4	198.9	199.6	204	154	219	6
Os03g0821200	39.8	29.8	34.5	21.35	18.08	18.17	25.1	29.7	18.7	49.14	33.99	68.68	43.541	28.004	25.343	35.82	32.98	38.33	35.3	8.063	16.38	12.57	23.52	13.72	40.14	54.68	63.59	36.1	30.6	33.3	20
Os03g0415750	61.2	46.6	48.5	125.6	150.1	147.1	10.2	16.4	15.9	31.04	31.8	31.05	64.223	93.345	57.814	23.88	53.59	48.69	108.7	118.5	154.3	8.873	8.552	3.611	32.75	20.74	13.16	43.5	49.7	48.3	10
Os03g0691050	0	0.93	2.16	0	0	0	0	0	0	0	0	0	0	0	0	0	0	0	0	0	0	0	0.713	0.722	0	0.943	0	0	0	0.83	14
Os03g0790900	4411	4720	5065	2449	2783	2071	3921	4598	3602	3087	3335	3061	4585.9	4320	4239.4	7368	4499	4434	1962	2174	1685	3072	3738	3666	3939	3860	3528	5354	4534	5011	13
Os03g0832500	36.7	34.5	28	45.15	30.73	51.9	41.5	33.5	19.3	31.04	38.37	37.63	18.505	45.739	36.43	47.76	26.79	36.26	31.58	29.03	37.94	34.75	33.49	30.33	30.63	51.86	41.67	25.9	43	37.5	11
Os03g0575950	0	0	0	0	0	0	0	0	0	0	0	0	0	0	0	11.94	1.031	0	0	0	0	0.739	2.138	2.167	0	0	0	0	0	2.5	6
Os03g0217900	161	120	102	56.65	80.45	54.5	148	137	149	106.9	107.4	103.5	89.259	98.012	76.821	549.3	201	125.4	95.68	67.73	80.19	142.7	114	146.6	90.85	156.5	125	117	120	146	6
Os03g0119300	464	156	373	6.568	5.424	43.25	7.05	5.06	6.22	172.4	71.27	313.3	247.09	608.61	323.91	346.3	485.4	473.5	19.51	12.09	12.07	0.739	0	6.5	194.4	176.3	254.4	195	167	211	3
Os03g0213100	132	90.4	111	300.5	264.9	340.8	335	235	410	232.8	159	166.5	150.22	214.69	201.16	47.76	115.4	92.21	454.3	400.7	341.4	244	217.4	231.1	143.7	129.2	208.3	84.2	83.2	76.6	12
Os03g0855500	28.5	23.3	28	40.23	39.77	44.12	147	109	116	50.87	47.14	40.46	63.134	42.939	39.598	11.94	22.67	33.15	37.16	37.09	40.52	119	129	124.9	53.87	43.37	30.7	48.1	44	39.2	18
Os03g0843200	1827	1530	2064	243.8	273	285.5	525	703	522	1502	1938	1916	1715.5	2003.2	2010	3475	2626	2139	465.4	449.1	353.5	532.4	487.4	528.7	2104	2285	1919	2234	2105	2164	13
Os03g0713500	0	0	0	0.821	0	0	0	0	0	0	2.193	0	0	0.9335	1.5839	0	0	0	0	0	0	0.739	0	0	0	0	0	0	0	0	4
Os03g0703100	16.3	22.4	18.3	33.66	29.83	51.9	33.7	25.9	29	26.73	23.02	28.22	18.505	26.137	18.215	23.88	19.58	19.68	42.73	28.22	56.91	39.19	32.78	33.95	11.62	23.57	37.28	36.1	28.7	25	10
Os03g0301550	0	0	0	0	0.904	0.865	0	0	0	0	0	0	0	0	0	0	0	4.144	0	0.806	0	0	0	0	0	0	0	0	0	0	1
Os03g0280750	4.08	3.73	3.23	9.852	0.904	6.92	0.78	3.16	0	2.587	12.06	2.822	3.2656	5.6007	1.5839	0	6.183	0	6.503	3.225	9.484	1.479	0	3.611	1.056	1.886	0	6.48	0	5	16
Os03g0158300	1133	1141	1284	2503	2620	2474	4577	4357	4620	1245	1404	1543	1698.1	1543	1218.8	1719	1212	1217	3764	3567	3722	2213	2437	3369	1166	1145	1162	999	1270	1300	12
Os03g0717200	0	0	0	3.284	5.424	0	3.91	7.59	4.15	5.173	0	0.941	0	0.9335	0	0	2.061	1.036	1.858	1.613	3.449	2.218	0	4.333	2.113	0.943	2.193	0	0.96	2.5	18
Os03g0144300	0	0	0	0	0	0	0	0	0	0.862	0	0	0	0	0	0	2.061	0	0	0	0	0	0	0	0	0	0	0	0	0	1
Os03g0281300	0	0	0	0.821	0	0	3.91	0	0	0	0	0	0	0	0	0	0	0	0	0.806	0	0	0	0.722	0	0.943	0	0.93	0.96	1.67	5
Os03g0125700	144	132	133	144.5	135.6	104.7	184	190	191	150.9	167.7	140.2	139.33	160.55	172.65	107.5	95.84	154.4	117	113.7	87.95	139	118.3	117	132	114.1	98.68	96.2	115	138	5
Os03g0163000	7.14	14	5.39	4.105	8.136	9.516	17.2	13.3	13.8	10.35	12.06	19.76	5.4426	8.4011	7.1277	11.94	7.214	11.4	9.29	8.063	18.11	8.133	14.97	8.667	15.85	8.486	15.35	9.25	12.4	6.66	15
Os03g0128700	600	647	501	950.7	637.3	1119	403	564	506	464.7	555.9	467.6	419.08	611.41	542.5	632.9	570.9	536.7	624.3	737.7	883.8	1634	2011	1523	579.9	840.1	875	778	838	757	17
Os03g0632666	0	0	0	0.821	0	0	0	0	0	0	0	0	0	0	0	0	0	0	0	0	0	0	0	0	0	0	0	0	0	0	2
Os03g0293400	58.1	85.7	62.5	113.3	119.3	71.8	117	89.8	82.2	69.84	74.55	94.08	71.842	70.009	73.653	83.59	64.92	104.6	70.6	80.63	96.57	85.77	83.38	76.56	75	90.51	61.4	62	89.9	45.8	12
Os03g0377300	325	344	358	360.4	338.1	336.5	354	350	366	338.8	381.5	375.4	383.16	363.11	410.24	525.4	331.8	355.4	329.8	371.7	338.9	351.2	394.1	330.8	389.8	388.5	333.3	357	338	387	6
Os03g0577100	0	0	0	1.642	0	0.865	1.57	1.27	0	0	0	0	0	0	0.792	0	0	0	1.858	3.225	0	0.739	0.713	1.444	0	0.943	0	0	0	0	12
Os03g0187100	782	929	821	807.8	764.8	831.3	685	767	755	819.9	815.7	652	866.46	760.76	977.29	1469	778.1	820.5	605.7	654.7	552.7	1004	892.9	902.8	813.4	762.8	655.7	982	843	991	6
Os03g0831550	2.04	1.86	3.23	5.747	15.37	2.595	5.48	5.69	6.91	3.449	1.096	0.941	1.0885	0.9335	3.9598	0	1.031	3.108	2.787	4.031	3.449	2.218	4.988	2.167	2.113	0	2.193	1.85	0.96	0.83	12
Os03g0411600	135	87.6	114	105.9	79.55	121.1	110	105	106	127.6	141.4	126.1	153.48	128.82	121.17	143.3	80.38	92.21	131.9	72.57	93.12	110.2	137.5	103.3	94.02	75.43	61.4	89.7	110	95.8	7
Os03g0743900	1816	1563	1553	2061	2079	1284	1071	1737	936	2112	2013	2513	1617.5	1818.4	3155.2	1982	1489	1517	2042	1774	1904	1162	1289	1350	2241	2276	2833	1586	1752	1828	20
Os03g0146000	741	564	653	216.7	216	335.6	291	361	300	470.8	453.9	538.1	581.27	722.49	604.27	1230	702.8	672.4	352.1	243.5	415.6	219.6	260.8	359	654.9	639.2	708.3	659	542	511	13
Os03g0176800	166	167	176	137.9	151.9	146.2	119	190	161	187.1	149.1	142.1	224.24	159.62	172.65	286.6	129.8	180.3	118.9	157.2	97.43	156.7	155.4	131.4	169	138.6	164.5	209	201	172	13
Os03g0222600	17.3	20.5	1.08	7.389	1.808	26.82	7.83	3.8	3.46	2.587	4.386	15.99	2.177	0	4.7518	23.88	31.95	5.18	6.503	4.031	3.449	12.57	3.563	20.22	8.451	18.86	103.1	24.1	29.6	7.5	11
Os03g0750700	893	688	603	491.8	540.6	633.2	449	419	380	379.4	364	425.3	347.24	659.02	418.16	1421	1276	783.2	723.7	640.2	711.3	328.3	515.9	548.2	484.9	597.8	800.4	763	699	575	13
Os03g0212300	3.06	2.79	0	1.642	0.904	1.73	0.78	0	0	1.724	1.096	0	0	2.8004	0.792	0	0	2.072	1.858	1.613	0	0	2.138	0.722	0	0.943	0	0	0	0.83	19
Os03g0803600	55.1	77.3	49.6	6.568	5.424	20.76	26.6	10.8	14.5	18.97	13.16	18.82	13.062	14.002	12.671	11.94	24.73	25.9	15.79	15.32	18.11	22.18	24.23	20.95	12.68	26.4	13.16	7.4	16.3	7.5	14
Os03g0796301	0	0	1.08	0	0	0	0	0	0.69	0	1.096	0.941	0	0	1.5839	0	0	2.072	0.929	0	0.862	0	1.425	0	0	0	0	0.93	0.96	1.67	1
Os03g0673000	2.04	0.93	2.16	4.105	5.424	0.865	1.57	3.8	0.69	2.587	5.482	7.527	1.0885	7.4676	1.5839	11.94	4.122	14.5	1.858	3.225	3.449	3.697	2.851	2.889	5.282	7.543	10.96	7.4	5.74	3.33	13
Os03g0650000	140	117	116	21.35	44.29	27.68	50.1	49.3	19.3	120.7	132.7	242.7	71.842	101.75	49.102	95.53	186.5	175.1	36.23	41.93	46.56	28.1	24.23	48.39	84.51	113.1	182	87	136	102	3
Os03g0816400	27.5	21.4	29.1	102.6	104	96.02	84.6	73.4	80.2	19.83	24.12	22.58	9.7967	17.736	18.215	47.76	12.37	14.5	88.25	66.12	54.32	59.89	41.33	70.78	14.79	13.2	26.32	15.7	30.6	11.7	12
Os03g0604700	58.1	66.1	58.2	189.6	149.2	148.8	101	144	107	68.97	58.11	72.44	39.187	48.539	87.116	47.76	47.4	42.48	122.6	98.37	87.08	99.82	113.3	111.9	49.65	43.37	21.93	41.6	65	58.3	12
Os03g0625900	174	143	144	284.9	276.6	242.2	139	102	112	169.9	130.5	219.2	148.04	164.29	127.51	179.1	148.4	170.9	320.5	248.3	296.6	142.7	110.5	135.1	105.6	132.9	175.4	147	150	117	10
Os03g0278200	129	103	125	60.75	47.01	98.62	57.9	65.1	63.6	63.8	53.72	52.69	47.895	101.75	74.445	131.4	219.5	102.6	58.52	56.44	85.36	71.72	86.94	116.3	73.95	99	107.5	99	118	65.8	14
Os03g0101800	656	751	758	756.9	756.6	735.3	1412	1309	1405	1000	858.5	859.9	768.5	709.42	735.74	1182	672.9	597.8	826.8	738.6	752.7	1034	1084	1164	773.3	670.4	732.4	642	575	661	18
Os03g0160200	2675	2854	2853	3001	2866	2928	2865	2726	2712	3112	2968	2554	2751.8	2701.4	2879.6	5027	3001	2926	2815	3177	3223	3497	3034	2862	2888	2914	2906	3106	3324	3161	6
Os03g0275400	0	0.93	0	0	0	0	0	0	0.69	0	0	0	0	0	0.792	0	0	0	0	0	0	0.739	0	0	0	0	0	0.93	0	0	1
Os03g0348500	32.6	37.3	14	36.94	29.83	51.04	78.3	105	79.5	18.97	46.05	28.22	35.921	30.804	22.967	107.5	21.64	17.61	28.8	46.76	34.49	76.9	78.39	73.67	50.71	30.17	32.89	26.8	44.9	51.7	18
Os03g0320400	0	0	0	0	0	0	0	0	0	0.862	0	0	0	0	0	0	0	0	0	0	0	0.739	0	0	0	0	0	0	0	0	7
Os03g0610650	844	648	768	605.1	577.6	636.7	683	593	672	830.3	640.3	785.6	570.38	788.77	596.35	644.8	620.4	678.6	683.7	504.7	628.6	507.2	606.5	564.8	613.7	636.4	833.3	551	619	554	20
Os03g0828300	0	0	0	0	0	0	0	0	0	0	0	0	0	0	0	0	0	0	0	0	0	0	0.713	0	0	0	0	0	0	0	17
Os03g0311300	####	####	####	4162	3973	4624	6106	6930	6450	13047	14995	16169	17825	18404	17554	30628	18798	17500	6777	6062	7318	5084	4787	5683	16379	17116	15532	####	####	####	13
Os03g0304800	84.6	30.7	65.7	124.8	60.57	175.6	12.5	12	11.7	52.59	53.72	53.63	45.718	80.277	37.222	47.76	87.6	55.95	213.7	118.5	196.6	17.75	7.839	36.11	59.16	96.17	103.1	26.8	55.5	25	16
Os03g0388432	85.6	83.8	143	105.9	133.8	96.89	25.1	27.8	17.3	32.76	33.99	14.11	25.036	34.538	33.263	23.88	58.74	66.31	25.08	47.57	33.63	79.11	85.52	43.33	24.3	36.77	17.54	71.2	51.6	79.1	8
Os03g0437200	36.7	108	35.6	6.568	0	0	0	1.9	4.15	0	0	0	5.4426	0.9335	0	11.94	24.73	26.94	1.858	0	0	19.22	2.851	14.44	0	0	0	0.93	4.78	0	14
Os03g0793900	1.02	3.73	5.39	3.284	2.712	3.46	0.78	0	0.69	0	0	0	0	0.9335	0.792	0	0	0	4.645	2.419	0	0	0	0	1.056	0	0	0	0	1.67	2
Os03g0139500	0	1.86	0	1.642	0	0	0	0	0	0	0	3.763	0	0.9335	0	0	0	0	0	0.806	0	1.479	0.713	0	0	0	0	1.85	0	2.5	20
Os03g0685500	2754	2181	2544	1934	1897	2289	2086	1615	1836	2846	2694	3570	3013	3228.8	2946.9	4239	2854	2575	3303	3070	3238	1432	1797	2109	3015	3130	3333	2755	2855	2322	13
Os03g0324700	13.3	3.73	8.62	9.031	10.85	16.44	18.8	22.1	25.6	12.93	15.35	15.05	3.2656	8.4011	10.296	11.94	7.214	10.36	15.79	9.675	5.173	11.83	22.8	13.72	4.225	8.486	4.386	7.4	4.78	5	18
Os03g0819600	1149	840	866	1395	1261	1722	440	296	236	857	658.9	1105	672.71	1263	829.98	2173	1324	1111	2026	1553	1954	275.1	669.9	793.7	858.8	1107	1399	1448	1084	1041	16

Os03g0595200	230	278	207	215.9	241.4	215.4	511	411	377	294	277.4	348.1	240.56	245.5	218.58	250.8	220.5	221.7	260.1	254	273.3	288.4	285.1	348.8	214.4	269.7	282.9	229	238	237	18
Os03g0328900	336	278	303	188.8	253.1	225.8	194	178	211	218.1	211.6	234.3	173.07	213.76	211.45	310.5	263.8	348.1	265.7	211.2	310.4	269.1	250.1	318.5	291.6	326.2	311.4	306	275	302	1
Os03g0376800	1364	1348	1405	1576	1620	1522	1296	1315	1174	1270	1293	1040	1285.5	1374	1381.2	2591	1585	1530	1151	1324	1305	1396	1298	1205	1449	1508	1232	1711	1763	1695	6
Os03g0253600	214	272	213	117.4	159.1	76.99	32.9	34.8	22.8	220.7	205	129.8	125.18	170.82	134.63	167.2	166.9	128.5	119.8	89.5	92.26	73.94	57.72	82.34	114.1	107.5	107.5	174	169	176	3
Os03g0622200	0	1.86	0	0	0	0	0	0	0	0	0	0	0	0	0	0	0	0	0	0	0	0	0	0	0	0	0	0	0	0	14
Os03g0591700	305	279	281	318.5	292.9	265.6	106	142	158	179.3	193	223	200.29	245.5	230.46	465.7	254.5	324.3	236.9	254.8	287.1	265.4	238	191.4	208.1	261.2	230.3	251	292	252	13
Os03g0826200	4219	3309	3947	650.2	594.8	647.1	2822	2948	3313	3522	3834	4857	4145.1	4327.5	4338.4	6985	5851	4391	1202	926.4	948.4	2392	2302	3444	5096	5173	4574	3569	3715	3947	13
Os03g0755700	43.8	34.5	45.3	13.96	27.12	14.71	21.1	27.2	26.3	84.49	84.42	119.5	84.905	48.539	64.941	119.4	44.31	45.59	14.86	17.74	25	38.45	26.37	22.39	84.51	96.17	129.4	58.3	59.3	57.5	3
Os03g0566800	743	718	668	845.6	781.9	768.2	780	679	717	707	651.3	820.4	593.24	693.55	653.37	1051	713.1	695.2	761.7	733.7	847.6	731.3	728.3	727.3	669.7	750.5	741.2	631	601	633	6
Os03g0128400	278	352	365	328.4	366.1	304.5	561	705	554	449.2	498.9	395.1	587.8	409.79	467.26	1182	256.6	325.3	314.9	344.3	294.9	320.2	298.6	435.5	384.5	295.1	282.9	291	313	363	6
Os03g0764450	553	447	504	364.5	376.1	346	382	377	453	509.6	461.6	522.2	665.09	541.4	565.46	788.1	599.8	530.5	390.2	400.7	374.2	343.1	386.2	347.4	581	608.1	554.8	525	489	547	13
Os03g0685000	1932	1792	1931	2210	2152	2035	3983	3863	4069	2468	2683	3337	2802.9	2582.9	2265	2603	2267	2208	3321	3240	3660	2293	2216	2981	2565	2592	2550	1742	1979	2008	15
Os03g0155000	0	0	0	0	0	0	0	0	0	0	0	0	0	0	0	0	0	0	0	0	0	0	0	0	0	0	0	0.93	0	0	1
Os03g0140300	2.04	1.86	1.08	0	1.808	4.325	5.48	8.22	6.22	0.862	5.482	1.882	2.177	0	3.1679	0	0	0	2.787	0	9.484	1.479	2.138	6.5	3.169	0.943	2.193	0	4.78	3.33	18
Os03g0704200	81.6	105	81.9	87.02	107.6	67.47	91.6	99.9	87.8	60.35	69.07	59.27	64.223	94.279	69.693	59.7	113.4	73.56	78.96	108.8	75.01	96.86	68.41	84.5	62.33	33.94	81.14	59.2	61.2	52.5	12
Os03g0414400	457	546	485	820.2	915.7	835.6	1210	1160	1206	482.8	627.1	763	797.89	755.16	612.98	609	566.8	529.4	1018	870.8	965.7	856.9	781	954.1	763.8	662.8	686.4	510	547	632	12
Os03g0800800	321	392	322	541.8	620.1	522.5	321	280	262	279.3	301.5	281.3	328.73	326.71	348.47	465.7	304	318.1	409.7	482.2	457	290.6	332.1	267.2	300	290.4	267.5	345	381	327	10
Os03g0207900	296	344	315	380.1	438.4	360.7	315	266	257	294.9	275.2	271	313.49	294.97	338.96	620.9	341.1	330.5	377.2	402.3	377.7	322.4	325.7	386.4	346.5	328.1	280.7	338	399	377	6
Os03g0808200	0	0	0	0	0	6.055	7.05	4.43	2.07	0.862	0	0	2.177	0	2.3759	11.94	0	0	4.645	0.806	0	0	0	2.889	0	0	0	0	0	0	6
Os03g0819300	1.02	0	0	2.463	3.616	2.595	0	6.33	1.38	1.724	0	1.882	2.177	2.8004	0.792	11.94	2.061	1.036	0.929	1.613	1.724	2.958	0.713	2.889	3.169	0.943	13.16	2.78	0.96	2.5	6
Os03g0588200	472	562	552	447.4	471	482.7	406	395	457	480.2	483.5	448.8	566.03	495.66	534.58	812	560.6	474.5	365.1	311.2	312.1	478.4	409.1	453.6	613.7	549.7	583.3	545	462	530	13
Os03g0597450	296	293	324	308.7	348.9	323.5	280	350	350	281.9	320.1	298.2	339.62	349.11	335	573.2	399.8	409.2	314.9	389.4	395.8	346.8	316.4	301.2	372.9	453.5	337.7	417	415	463	13
Os03g0587200	0	0	0	0	0	1.73	0	0	0	0	0	0	0	0	0	0	0	0	0	0	0	0	0	0	0	0	0	0	0	0	2
Os03g0259500	0	1.86	0	0	0	0.865	0	0	0	0	0	0	0	0	0	0	0	0	0	1.613	4.311	0	0	0	0	7.543	2.193	0	1.91	0	16
Os03g0665200	77.5	83.8	62.5	45.97	52.43	59.69	102	94.2	133	129.3	211.6	115.7	100.14	85.878	94.244	191.1	71.11	71.49	21.37	46.76	37.08	84.29	94.78	114.1	95.07	119.7	96.49	73.1	120	78.3	5
Os03g0307200	4.08	0.93	1.08	1.642	0.904	1.73	3.13	1.9	0.69	0.862	0	1.882	1.0885	1.8669	0	0	1.031	2.072	0.929	0	0	0.739	0	0	0	0	0	0	1.91	1.67	19
Os03g0162300	0	0	0	0	0	0	0	0	0	0	0	0	3.2656	1.8669	0	0	0	0	0	0	0	0	0	0	0	0	0	0.93	0	0	4
Os03g0860700	0	0	0	0	0	0	0	0	0	0	0	0	0	0	0	0	0	0	0	0	0	0	0.713	0	0	0	0	0	0	0	17
Os03g0594100	12.2	13	14	3.284	5.424	4.325	3.91	8.86	2.76	7.76	12.06	8.467	11.974	8.4011	19.799	11.94	7.214	15.54	9.29	3.225	4.311	5.176	17.1	7.945	12.68	5.657	21.93	28.7	15.3	14.2	1
Os03g0211400	93.8	61.5	55	64.04	48.81	73.53	31.3	41.1	55.3	51.73	42.76	64.92	34.833	51.34	55.438	95.53	96.87	81.85	57.6	44.35	57.77	70.98	71.26	74.39	55.99	77.31	48.24	81.4	60.2	64.2	13
Os03g0691800	119	124	100	165	145.5	139.3	65	85.4	80.2	81.05	104.2	122.3	106.67	135.35	141.76	71.65	140.2	121.2	170.9	151.6	130.2	197.4	178.9	205.1	129.9	98.06	107.5	144	110	131	9
Os03g0800500	256	306	275	367	382.4	316.6	407	406	409	413.8	399.1	501.5	264.51	210.96	247.09	179.1	221.6	245.5	302.8	276.6	292.3	252.1	264.4	317.1	299	265.9	300.4	177	171	198	15
Os03g0231400	67.3	58.7	64.7	41.87	36.16	36.33	203	92.3	107	98.29	62.49	63.98	85.993	83.077	92.66	83.59	106.1	68.38	75.25	68.53	67.25	48.06	90.5	76.56	54.93	65.06	81.14	87	92.8	78.3	5
Os03g0330300	1062	1052	932	1091	798.2	1251	574	581	847	607.8	656.7	527.8	487.66	682.35	651	1505	1070	846.4	929.6	830.5	844.1	1102	1428	1113	833.5	1072	1434	1205	1280	974	8
Os03g0306700	12.2	1.86	4.31	14.78	23.5	4.325	1.57	3.16	0	21.55	10.96	2.822	14.151	10.268	14.255	23.88	4.122	2.072	6.503	10.48	1.724	0	2.851	0	9.507	0	0	3.7	6.69	8.33	6
Os03g0175200	63.2	103	106	49.26	51.53	65.74	200	223	245	275.9	236.8	238	128.45	146.55	161.56	191.1	119.5	114	51.09	50.8	45.7	123.5	118.3	157.4	151.1	150.9	149.1	101	120	90.8	5
Os03g0267700	416	343	335	478.6	446.6	552.8	287	205	211	463	447.3	456.3	427.79	457.39	469.64	573.2	564.7	434.1	740.4	748.2	775.1	249.2	234.5	228.2	479.6	496.9	585.5	426	453	449	16
Os03g0161800	4932	4633	4967	4144	4241	4143	5203	5102	5020	5576	5202	6263	5260.8	6362.4	5192.1	5839	5358	4775	5927	4998	5848	3250	3529	3584	5287	5179	5217	4276	4401	4421	20
Os03g0257000	6.12	10.2	10.8	21.35	12.66	34.6	25.1	12.7	29.7	1.724	7.675	6.586	0	5.6007	2.3759	0	2.061	3.108	23.22	27.41	15.52	36.23	27.08	46.95	4.225	7.543	0	10.2	4.78	2.5	6
Os03g0264400	1139	1033	1239	1220	1163	1002	821	773	1218	909.6	616.2	601.2	570.38	563.8	679.51	835.9	599.8	671.4	925.2	941.7	1004	1082	823.1	898.5	407.8	438.4	543.8	443	545	397	12
Os03g0192300	0	0	0	0	0	0	0	0	2.07	0	0	0	0	0	0	0	0	0	1.858	0	0	0	0	0.722	1.056	2.829	0	0	0	0	15
Os03g0733800	1008	720	881	354.7	293.8	414.4	312	266	324	536.3	526.3	464.8	452.82	556.34	613.77	537.3	486.4	547	424.5	416.8	325.1	398.5	326.4	335.8	440.5	419.6	587.7	448	396	350	14
Os03g0258200	87.7	68	65.7	22.17	12.66	41.52	61.1	55	69.1	9.484	10.96	23.52	26.124	26.137	33.263	35.82	55.65	55.95	55.74	14.51	36.21	141.2	69.84	252.8	15.85	27.34	30.7	12	9.56	15	9
Os03g0233700	8.16	1.86	1.08	9.031	8.136	12.98	11	2.53	4.84	1.724	8.771	4.704	0	2.8004	3.1679	0	5.153	3.108	2.787	8.063	0.862	14.05	1.425	9.389	6.338	4.714	2.193	3.7	3.83	6.66	2
Os03g0562400	0	0	1.08	0	0	0	0.78	0	0	0	0	0	0	0	0	0	0	0	0	0.806	0	0	0.713	0	0	0	0	0	0	0	17
Os03g0311600	9.18	4.66	2.16	1.642	1.808	3.46	0.78	0	0	4.311	6.578	12.23	1.0885	0	0.792	11.94	9.275	1.036	0	2.419	3.449	0	0	0	2.113	6.6	4.386	0.93	2.87	3.33	13
Os03g0301950	0	0	0	0	0.904	0	0	0	0	0	0	0	1.0885	0	0	0	0	0	0	0	0	0.739	0	0	2.113	0	0	0	0	0	4
Os03g0100900	352	434	336	380.9	376.1	384.9	462	500	499	327.6	367.3	334.9	456.09	297.77	410.24	573.2	436.9	383.3	362.3	409.6	394	565.6	520.9	518.6	394	356.4	386	383	415	457	17
Os03g0842900	0	0	0	1.642	0	0.865	0	0	0	0	0	0	0	0	0	0	1.031	0	0	0	0	0	0	0	0	0	0	0.93	0	0	2
Os03g0297600	384	304	363	206.9	203.4	161.8	211	180	369	503.5	394.7	676.4	526.84	442.46	515.57	370.2	380.3	301.5	280.5	193.5	195.7	1094	662.8	847.2	681.4	612.8	410.1	479	405	547	9
Os03g0251500	1726	1614	1650	1175	1492	1357	497	769	445	758.7	823.4	947.4	1063.5	1229.4	1138.8	2711	1870	1619	1059	1180	1007	831.8	910	680.4	1319	1208	1105	1551	1254	1525	13
Os03g0847600	1201	1131	1065	773.4	631	917.8	599	731	746	1406	1181	1364	782.65	810.24	983.62	1445	913.1	1171	613.1	649.9	687.2	1000	1197	919.4	1377	1475	1353	970	828	972	3
Os03g0215000	370	417	367	579.6	602.9	495.7	260	301	408	321.6	281.8	284.1	261.24	267.9	289.86	346.3	258.7	229	444	453.1	386.3	404.4	535.2	416	271.5	254.6	364	296	294	287	2
Os03g0582800	0	0	2.16	0	0	0.865	0	0	0	3.449	0	0	0	0	0	0	0	0	0	0	0	0.739	0	0	0	0	2.193	0	0.96	0	7
Os03g0794800	1003	1166	1025	1009	1156	1080	929	1043	862	964.8	1042	967.2	1337.8	1018.4	1179.2	1672	1108	1073	1000	1053	961.4	1025	1020	962.8	1140	1101	1134	1174	1116	1203	13
Os03g0266100	126	126	136	351.4	303.7	349.5	388	345	390	145.7	111.8	189.1	102.32	151.22	108.5	83.59	138.1	94.28	317.7	324.9	398.3	342.3	353.5	364.7	136.3	162.2	162.3	92.5	160	112	12
Os03g0352800	304	313	332	463.9	494.5	459.3	485	464	504	357.8	309.2	446.9	359.21	374.31	285.9	238.8	284.4	299.4	566.7	490.2	592.3	355.6	352	414.6	243	300.8	337.7	249	278	256	12
Os03g0754800	541	455	501	645.3	627.4	613.3	504	399	418	464.7	512	419.6	522.49	555.4	572.59	1015	568.9	479.7	637.3	685.3	618.2	603.3	535.2	514.2	439.4	491.2	530.7	480	530	504	6
Os03g0135400	361	304	375	444.1	439.3	364.2	529	701	610	306.9	321.2	275.7	288.46	243.63	253.43	286.6	344.2	288	352.1	310.4	350.9	471.7	481.7	517.1	299	339.4	361.8	306	294	261	18
Os03g0139400	702	558	619	409.7	375.1	372.8	269	275	297	776	695.1	820.4	710.8	629.15	609.02	1588	907.9	810.2	745.9	807.1	741.5	174.5	219.5	236.2	729.9	829.7	879.4	645	621	568	13
Os03g0661800	200	243	209	264.4	259.4	259.5	274	272	258	235.4	242.3	207	273.22	235.23	249.47	406	243.2	235.2	246.2	232.2	254.4	245.5	272.9	241.2	252.5	248	190.8	261	241	239	6
Os03g0848600	416	350	364	302.9	321.8	280.3	703	688	731	385.4	355.2	398.9	329.82	363.11	300.16	274.6	272.1	271.4	262.9	261.2	245.7	502	487.4	541	328.5	348.9	412.3	229	273	232	18
Os03g0265600	6790	4527	6505	1189	996.2	1337	628	609	603	5665	5836	5543	6426.6	5325.3	6898.8	7129	4901	4780	1639	1821	1690	907.2	818.8	712.9	6874	6140	6202	5990	5585	5308	3
Os03g0701550	72.4	90.4	78.7	68.14	78.65	87.37	88.5	56.9	89.8	112.1	105.3	135.5	78.373	94.279	86.324	143.3	74.2	66.31	81.75	99.98	103.5	63.59	33.49	54.17	73.95	101.8	92.1	69.4	80.3	64.2	20
Os03g0176900	87.7	64.3	73.3	66.5	58.76	80.45	68.9	72.1	62.9	84.49	73.46	140.2	79.462	109.21	80.781	131.4	85.53	116	43.66	36.28	40.52	38.45	12.83	48.39	86.62	113.1	85.52	68.5	63.1	54.2	13
Os03g0747400	0	0	0	0	0	0	1.57	1.9	0	0	2.193	0	0	0	0	0	0	0	0	0	0	0	0	0	1.056	0	0	0	0	0	5
Os03g0618900	48.9	39.1	48.5	13.96	18.08	24.22	45.4	32.9	30.4	73.29	72.36	91.26	63.134	58.807	54.646	59.7	50.5	59.05	24.15	18.54	28.45	17.01	14.25	32.5	61.27	83.91	74.56	33.3	63.1	52.5	3
Os03g0819201	0	0	0	0	0	0	0	0	0	0	0	0	2.177	0	0	0	0	0	0	0	0	0	0.713	0.722	1.056	0.943	0	0.93	0	0	4
Os03g0698350	204	267	262	241.4	248.6	232.7	116	137	124	157.8	159	100.7	227.5	200.69	197.99	477.6	219.5	223.8	157	219.3	153.5	181.9	217.4	175.5	245.1	156.5	160.1	236	272	281	13
Os03g0847851	134	122	106	81.28	64.18	76.12	177	146	154	177.6	162.3	210.7	152.39	130.68	163.14	95.53	134	119.1	139.3	147.5	107.8	114.6	99.77	122.1	191.2	191.4	171	109	109	107	20
Os03g0207700	13.3	14	9.7	21.35	14.46	20.76	8.61	14.5	14.5	11.21	14.25	18.82	9.7967	5.6007	15.047	23.88	19.58	11.4	11.15	8.869	12.07	15.53	10.69	13.72	5.282	19.8	13.16	16.7	16.3	14.2	6
Os03g0255200	540	571	605	708.5	884.1	559.7	586	720	632	476.8	555.9	504.3	490.92	426.59	379.35	644.8	320.5	398.9	551.8	649.1	563.9	480.6	505.3	500.5	337	349.8	379.4	277	257	292	12
Os03g0235000	9.18	3.73	3.23	3.284	7.232	4.325	13.3	13.3	87.8	3.449	1.096	5.645	2.177	0.9335	3.9598	0	1.031	1.036	16.72	0.806	1.724	90.94	109	202.2	1.056	2.829	0	0	1.91	0.83	9
Os03g0181100	2107	2148	1533	247.1	63.28	289.8	103	137	140	95.7	77.84	60.21	154.57	63.475	56.23	597	742	934.5	353	319.3	600.1	345.3	367	464.4	99.3	148	287.3	104	147	183	14
Os03g0279700	6.12	0.93	0	1.642	0.904	2.595	0.78	0	0	2.587	0	5.645	4.3541	0	0	0	2.061	4.144	0.929	0	2.587	2.218	1.425	0.722	1.056	0	0	0	1.91	0	19
Os03g0344300	526	555	462	461.4	480.9	506.1	496	488	489	541.5	574.5	615.3	623.72	547.94	620.9	1087	561.6	577.1	513.7	506.3	527.7	533.8	465.4	463	653.9	743.9	679.8	612	602	655	13
Os03g0797500	9.18	0	1.08	74.71	3.616	2.595	7.83	305	567	59.49	123.9	0	44.629	0.9335	8.7116	0	1.031	0	3.716	9.675	0	4.436	32.07	3.611	0	0	2.193	0.93	1.91	0	18
Os03g0696150	2.04	0	0	1.642	0.904	0	2.35	0.63	0	0.862	0	0.941	2.177	0.9335	0	0	1.031	0	1.858	0	3.449	0.739	0	2.889	2.113	0.943	0	1.85	0	0	15
Os03g0268100	771	786	804	723.3	715.9	802.8	889	910	967	704.4	799.3	741.4	886.06	957.72	921.85	1230	972.8	854.7	869.5	924	910.5	818.5	771.1	791.6	880	806.1	785.1	967	860	1036	6
Os03g0198400	123	123	125	139.6	158.2	173.9	180	166	161	106	95.39	121.4	111.03	104.55	100.58	83.59	97.9	112.9	223.9	218.5	208.7	149.4	159.6	154.6	84.51	86.74	131.6	98.1	113	92.5	12
Os03g0425900	0	5.59	0	0	0	0	0	0	0	0	0	0	0	0	0	0	0	0	0	0	0	0	0	0	0	0	0	0	0	0	14
Os03g0197175	63.2	96.9	127	52.54	63.28	20.76	153	176	75.3	135.4	107.4	79.97	65.311	40.138	102.96	59.7	56.68	50.77	26.01	33.06	7.76	56.19	124	71.5	70.78	28.29	28.51	59.2	74.6	86.6	5
Os03g0859600	165	233	251	134.6	98.53	121.1	47	45.5	31.8	151.7	102	112.9	171.99	158.69	163.94	429.9	113.4	158.5	88.25	45.96	69.84	64.33	64.85	45.5	132	103.7	94.3	101	122	137	13
Os03g0664650	5.1	3.73	4.31	5.747	2.712	12.98	4.7	1.9	8.29	4.311	7.675	3.763	2.177	6.5342	3.9598	11.94	4.122	1.036	1.858	6.45	3.449	9.612	2.851	3.611	2.113	4.714	0	1.85	2.87	4.17	6
Os03g0170701	0	2.79	1.08	0	0	1.73	0	0	0	0.862	0	0	0	0	0	0	2.061	0	0	0.806	0	0	0	0	0	0	0	0.93	0	0	14
Os03g0254400	77.5	74.5	86.2	46.8	44.29	44.12	75.2	49.3	65	106	88.81	128	74.019	96.146	67.317	167.2	108.2	72.52	75.25	51.6	52.6	84.29	86.94	78	92.96	116.9	107.5	107	88	101	13
Os03g0413400	324	393	343	388.3	397.7	378	274	250	337	361.3	348.7	279.4	335.26	276.3	297.78	537.3	379.2	329.5	409.7	409.6	539.7	321.6	312.8	355.3	390.9	395.1	372.8	371	449	421	16
Os03g0736900	308	360	258	169.9	148.3	114.2	214	121	225	142.3	244.5	290.7	84.905	73.743	98.204	561.2	525.6	393.7	251.7	293.5	309.5	235.1	168.9	572.7	246.1	304.5	298.2	117	128	211	6
Os03g0266000	598	550	586	912.9	1120	1071	788	741	800	488.9	531.8	673.6	708.63	667.42	551.21	776.2	717.2	681.7	1334	1147	1356	604.1	539.5	723.7	582.1	652.4	668.8	554	565	602	10
Os03g0138500	12.2	7.45	2.16	3.284	1.808	4.325	0.78	0	2.07	2.587	3.289	3.763	7.6196	1.8669	5.5438	11.94	1.031	1.036	2.787	2.419	1.724	4.436	2.138	1.444	2.113	1.886	0	0	0.96	0.83	6
Os03g0649000	156	147	134	31.2	22.6	32.01	68.1	56.9	61.5	168.1	142.5	189.1	82.728	128.82	130.67	131.4	116.5	124.3	40.87	30.64	37.94	76.9	76.25	97.5	151.1	161.2	219.3	166	140	155	3
Os03g0650350	1.02	0	0	0	0	0	0	0	0	0	0	0	0	0	0	0	0	0	0	0	0	0	0	0	1.056	0	0	0	0	0.83	19
Os03g0640400	327	333	336	318.5	376.1	284.6	496	430	396	317.3	368.4	354.7	371.19	378.05	310.45	417.9	312.3	272.5	383.7	331.4	396.6	478.4	399.1	424	302.1	316.8	342.1	278	255	317	18
Os03g0853350	0	0	0	0	0	1.73	0	0	0	0	0	0	0	0	0	0	0	0	0	0	0	0	0	0	0	0	0	0	1.91	0	2
Os03g0120100	429	305	355	176.5	177.2	269.9	516	383	589	341.4	423.2	513.7	460.44	686.09	557.54	716.5	694.6	479.7	510	349.9	499.2	704.6	456.8	777.1	597.9	769.4	947.3	674	678	597	11
Os03g0288600	514	536	462	472.9	482.7	513.8	528	574	595	525.9	599.7	653.9	569.3	556.34	587.64	1182	520.4	547	562	548.3	553.5	544.2	506	508.5	557.8	658.1	679.8	548	586	496	6
Os03g0396500	0	2.79	3.23	0	0.904	0.865	3.91	3.16	2.76	4.311	7.675	0	5.4426	2.8004	1.5839	0	5.153	3.108	0.929	1.613	0.862	1.479	2.851	2.167	3.169	0	2.193	0.93	0	5	7
Os03g0650300	0	0	0	0	0	0	0	0	0	0	2.193	0	0	0.9335	1.5839	0	0	0	0	0	0	0	0	0	1.056	0.943	0	0	0	0	4
Os03g0181800	1159	966	1036	139.6	106.7	159.2	322	358	344	527.7	516.4	601.2	531.2	543.27	528.24	1230	786.3	782.2	170	241.9	208.7	302.4	282.9	357.5	563	582.7	554.8	562	619	649	13
Os03g0626200	339	336	339	390.8	419.4	417.8	386	361	393	408.7	331.1	565.4	432.14	390.18	293.82	477.6	324.6	304.6	471.9	399.1	449.2	315.7	285.1	325	311.6	348.9	396.9	283	275	269	15
Os03g0741600	331	265	337	302.9	343.5	316.6	253	293	238	289.7	277.4	260.6	238.39	245.5	250.26	585.1	386.4	277.7	345.6	300.7	312.1	190.8	213.8	221.7	252.5	239.5	256.6	273	305	347	6
Os03g0295500	677	612	623	527.9	493.6	486.2	817	636	727	821.7	697.3	935.2	608.48	769.16	587.64	835.9	641	676.5	732.9	674.1	676.8	453.2	463.9	580.7	658.1	743.9	807	525	568	502	20
Os03g0292200	956	816	886	179	151.9	171.3	225	215	250	841.5	804.8	848.6	1015.6	1003.5	1002.6	1815	1084	872.3	264.8	233.8	253.5	266.9	314.3	236.9	878.9	890	864	881	782	783	13
Os03g0689900	379	394	367	367.8	379.7	378	694	562	672	503.5	480.2	612.5	396.22	382.72	394.4	644.8	331.8	335.7	382.7	334.6	364.7	533.1	429.7	522.2	429.9	464.8	546	305	321	310	5
Os03g0825700	697	768	717	759.4	810	803.6	674	773	732	684.6	713.8	577.7	692.3	678.62	691.39	1170	637.9	647.5	608.5	591.8	626.8	800.8	807.4	684.7	699.3	687.3	614	729	647	718	6
Os03g0814600	0	0	0	0	0	0.865	0	0	1.38	0	0	0	0	2.8004	0	0	0	0	0	0	0.862	0	0	0	0	0.943	2.193	1.85	2.87	0	11
Os03g0817500	0	0	0	0	0	0	2.35	0.63	0.69	0	0	0	0	0	0	0	0	0	0	0	0	0	0	0	0	0	0	0	0	0	18
Os03g0194600	84.6	77.3	86.2	34.48	39.77	68.34	38.4	48.7	56	62.08	63.59	94.08	64.223	56.007	68.109	262.7	152.5	137.8	117	74.98	87.08	45.1	71.98	48.39	91.9	78.26	78.95	95.3	56.4	69.1	13
Os03g0333500	0	0	0	0	0	0	0	0	0	0	0	0	0	0	0	0	0	0	0	0	0	0.739	0	0	1.056	0.943	0	0	0	0	1
Os03g0728200	59.1	69.9	53.9	77.17	93.11	93.43	102	67.7	73.2	72.42	99.77	98.79	90.347	79.343	79.197	119.4	69.05	67.34	78.96	99.17	103.5	86.51	73.4	85.22	77.11	114.1	100.9	71.2	64.1	72.5	6
Os03g0736600	2161	1500	2117	1328	1178	1130	2043	2378	2662	3593	3602	5212	2334.9	2104.9	2115.3	3761	3414	2933	2412	2349	2307	1695	1691	1898	3839	4541	4004	2073	2420	2074	20
Os03g0811600	858	714	745	623.1	664.4	666.1	759	656	719	970.8	949.5	1051	1171.2	1193	1085	1385	792.5	827.8	831.4	746.6	772.5	607.8	554.4	569.1	844	960.8	894.7	883	790	933	13
Os03g0229551	2.04	6.52	4.31	4.105	4.52	0.865	0	0	2.07	4.311	2.193	0.941	0	0.9335	7.9197	11.94	2.061	10.36	2.787	1.613	1.724	2.958	0	0.722	5.282	2.829	2.193	10.2	10.5	3.33	13
Os03g0389175	1.02	0	0	0	0	0	0	0	0	0	0	0	0	0	0.792	0	0	0	0	0	0	0.739	0	0	1.056	0	0	0	0	0	19
Os03g0190900	334	337	370	185.5	143.7	164.4	267	274	287	300.9	282.9	266.3	290.63	295.9	300.95	358.2	332.9	302.5	176.5	174.2	171.6	278.7	258.7	244.1	321.1	314	282.9	328	295	349	3
Os03g0132900	571	840	962	295.6	443.8	375.4	596	531	327	1668	894.7	987.9	415.81	643.15	587.64	1063	540	743.9	225.7	333	254.4	614.4	545.9	569.9	535.6	1400	804.8	552	499	297	7
Os03g0784400	935	1040	1027	917	1103	983.6	1309	1221	1205	1057	975.8	1052	933.95	969.86	916.3	1612	812.1	760.5	973.5	908.7	848.4	1060	1092	1018	968.7	927.8	903.5	829	862	886	6

Os03g0832600	695	747	700	756.1	776.5	626.3	1079	981	968	879.4	854.1	924.8	583.45	603.01	660.5	740.3	612.1	563.6	545.3	596.6	441.5	693.5	874.4	807.5	699.3	637.4	690.8	513	446	525	18
Os03g0143700	432	543	546	546.8	507.1	501.7	517	469	418	625.9	585.5	476.1	536.64	536.73	646.24	740.3	494.7	533.6	588	608.7	557	413.3	510.2	420.3	622.2	522.3	578.9	655	657	610	13
Os03g0155632	0	0	1.08	0	0	0	0	0	0	0	0	0	0	0	0	0	0	0	0	0	0	0	0	0	0	0	0	0	0	0	14
Os03g0101900	317	315	278	569.8	644.5	508.6	600	587	515	273.3	311.4	290.7	290.63	284.7	298.57	489.6	253.5	335.7	494.2	547.5	463.9	491.7	538	455.7	237.7	277.2	243.4	264	282	333	12
Os03g0751100	221	191	172	69.78	39.77	52.77	240	108	112	445.8	401.3	643.5	228.59	243.63	194.03	59.7	292.7	223.8	159.8	133	155.2	95.38	114	178.4	345.4	436.5	500	204	257	203	20
Os03g0770900	0	0	0	0	0	0.865	0	1.9	1.38	0.862	0	0	0	0	0	0	1.031	3.108	0	1.613	0	0	0	0	1.056	0	0	0	0	0	1
Os03g0807700	0	0	0	0	0.904	0	0	0	0	0	0	0	0	0	0	0	0	0	0	0	0	0	0	0	0	0	0	0	0	0	2
Os03g0788250	12.2	8.38	4.31	9.031	12.66	6.92	1.57	0.63	3.46	1.724	7.675	0	3.2656	5.6007	9.5036	11.94	4.122	3.108	2.787	3.225	2.587	11.09	5.701	4.333	10.56	8.486	4.386	9.25	1.91	10	8
Os03g0577500	342	420	397	320.2	276.6	295.8	215	170	239	394.9	356.3	271.9	340.71	450.86	346.88	537.3	445.2	363.6	283.3	293.5	284.5	372.7	490.3	353.2	465.9	428.1	425.4	553	419	511	13
Os03g0687000	149	211	169	269.3	245.9	229.2	583	380	562	360.4	301.5	375.4	256.89	241.76	207.5	143.3	146.3	146.1	196.9	187.1	197.4	346	341.4	387.8	215.5	264.9	293.9	196	189	183	18
Os03g0673800	25.5	27	18.3	7.389	12.66	11.25	7.83	9.49	9.67	18.11	12.06	17.88	5.4426	12.135	6.3357	35.82	26.79	13.47	13.01	14.51	8.622	5.176	8.552	15.89	12.68	21.69	24.12	15.7	20.1	14.2	13
Os03g0118200	205	207	223	101.8	113	98.62	249	309	309	164.7	186.4	182.5	143.68	189.49	186.9	334.3	286.5	222.7	88.25	77.4	112.1	108	81.95	130.7	192.3	207.4	195.2	142	166	177	5
Os03g0218100	1.02	0	1.08	0	0	0	0	1.27	0	0	0	0	0	0	0	0	0	0	0	0.806	0.862	2.218	0	1.444	1.056	0	2.193	0	0.96	0	9
Os03g0395100	40.8	60.5	49.6	45.15	54.24	39.79	74.4	61.4	67	48.28	41.66	44.22	62.046	34.538	37.222	95.53	42.25	40.41	60.38	33.86	43.97	45.84	37.77	80.17	38.03	39.6	46.05	44.4	54.5	39.2	6
Os03g0193901	3.06	12.1	6.47	11.49	13.56	15.57	7.83	3.8	8.29	0.862	5.482	5.645	13.062	7.4676	3.1679	0	9.275	5.18	19.51	17.74	17.24	8.133	5.701	13	3.169	4.714	8.772	1.85	9.56	11.7	10
Os03g0626500	284	269	217	131.4	115.7	99.48	72	192	106	300	284	275.7	285.19	234.3	422.91	191.1	179.3	220.7	103.1	97.56	75.88	149.4	305.7	165.4	433.1	318.7	495.6	458	295	318	3
Os03g0295600	24.5	26.1	22.6	23.81	12.66	25.95	29.7	26.6	13.8	23.28	31.8	31.99	9.7967	22.403	16.631	35.82	31.95	24.86	14.86	11.29	23.28	28.84	12.11	31.06	25.35	33.94	28.51	24.1	39.2	34.2	1
Os03g0120400	3.06	6.52	3.23	43.51	71.41	44.98	72.8	58.8	85.7	12.07	9.868	26.34	11.974	6.5342	2.3759	0	2.061	8.288	49.23	32.25	43.11	97.6	69.84	80.17	5.282	7.543	15.35	0.93	1.91	1.67	12
Os03g0862000	1.02	3.73	3.23	0	1.808	2.595	0	0.63	0.69	0	2.193	0.941	5.4426	2.8004	0.792	0	2.061	1.036	0	4.838	6.898	4.436	4.276	2.167	0	5.657	2.193	2.78	3.83	0	16
Os03g0816950	242	224	252	339.1	340.8	286.3	54	99.3	94	129.3	150.2	86.56	224.24	183.89	186.11	214.9	181.4	237.3	139.3	147.5	159.5	125	136.8	123.5	165.8	156.5	136	236	175	263	8
Os03g0303500	166	70.8	70	17.24	8.136	32.87	31.3	25.3	14.5	45.7	28.51	49.86	72.931	85.878	44.35	394.1	300.9	159.5	31.58	33.86	52.6	69.5	108.3	163.2	69.72	165.9	182	131	125	97.5	13
Os03g0262000	3.06	4.66	3.23	6.568	7.232	10.38	9.39	2.53	12.4	0.862	1.096	0	0	0.9335	0	0	0	0	6.503	7.257	9.484	2.958	3.563	14.44	0	0	0	3.7	0.96	3.33	12
Os03g0149400	138	117	108	241.4	255.8	190.3	87.7	108	91.9	77.6	92.1	45.16	163.28	131.62	107.71	274.6	101	108.8	153.3	173.4	167.3	133.1	144.7	106.2	75	54.68	72.37	99	104	130	10
Os03g0586300	315	251	316	460.6	427.6	434.3	533	545	592	365.6	318	344.3	335.26	336.04	375.39	620.9	300.9	298.4	484.9	434.6	530.3	487.3	492.4	543.9	319	325.3	357.4	318	265	282	12
Os03g0672300	39.8	51.2	31.2	79.63	75.93	82.18	76.7	67	108	60.35	40.57	29.17	33.744	37.338	30.887	23.88	40.19	23.83	55.74	49.18	61.22	79.11	90.5	85.22	26.41	35.83	65.79	30.5	28.7	27.5	12
Os03g0356526	76.5	112	91.6	25.45	20.79	35.47	43.8	39.2	73.2	82.77	86.62	64.92	65.311	50.406	56.23	155.2	105.1	78.74	37.16	41.12	37.94	81.33	99.06	86.67	91.9	45.26	46.05	84.2	65	83.3	13
Os03g0185600	112	97.8	119	222.5	282.9	290.7	160	151	165	170.7	159	135.5	143.68	120.42	100.58	226.9	111.3	115	304.7	240.3	294	148.6	131.8	135.8	107.7	76.37	89.91	86	123	129	10
Os03g0809300	1426	1299	1399	1205	1185	1214	1293	1336	1283	1547	1482	1662	1712.2	1435.6	1555.4	1922	1378	1346	1315	1259	1268	1176	1214	1239	1463	1578	1335	1242	1270	1479	13
Os03g0589300	470	473	500	609.2	612	654	507	439	431	319.9	343.2	351.9	437.59	419.12	407.86	883.6	438	509.7	601	624.1	456.1	676.5	622.8	618.2	453.2	368.7	368.4	599	450	553	6
Os03g0762300	8.16	7.45	11.9	4.926	7.232	6.92	12.5	10.1	11.7	11.21	6.578	12.23	5.4426	9.3345	7.9197	0	13.4	6.216	10.22	8.063	5.173	9.612	4.988	9.389	6.338	10.37	8.772	8.33	1.91	1.67	15
Os03g0116900	137	164	150	103.4	116.6	118.5	139	123	123	76.73	103.1	113.8	100.14	96.146	92.66	310.5	107.2	138.8	102.2	118.5	105.2	181.1	145.4	150.2	90.85	116	127.2	116	111	120	6
Os03g0703300	####	9160	####	1105	1067	1386	3557	5686	4385	8670	9940	9645	12710	9398.9	10428	14652	10435	10100	1511	1489	1023	3740	3611	4300	10795	10538	8938	9625	8691	####	3
Os03g0345700	110	125	90.5	73.07	74.13	79.58	145	144	207	131.9	144.7	154.3	107.76	149.35	114.84	35.82	125.7	103.6	113.3	124.2	104.3	145.7	148.2	200.1	140.5	178.2	160.1	130	131	119	5
Os03g0316200	1126	566	629	426.9	133.8	620.2	43.1	13.9	46.3	426.8	378.3	606.8	201.38	279.1	233.63	1314	1130	509.7	256.4	310.4	422.5	127.2	134	249.2	488	492.2	769.7	440	334	281	13
Os03g0761100	2200	1901	1886	348.9	320.9	455	555	591	618	818.2	734.6	761.1	558.41	526.47	624.86	1266	1382	1212	432	499.1	478.5	674.3	700.5	764.9	798.6	726.9	750	546	607	547	14
Os03g0795300	10.2	7.45	9.7	12.31	16.27	12.11	7.05	12.7	8.29	6.897	9.868	12.23	10.885	11.201	24.551	0	4.122	7.252	6.503	8.063	6.898	21.44	15.68	11.56	16.9	9.428	15.35	10.2	15.3	20	4
Os03g0295400	430	327	336	390.8	331.8	365.9	313	298	325	378.5	321.2	495.8	458.27	433.12	357.18	597	355.5	352.3	430.1	405.6	407	320.9	294.3	268	402.5	464.8	429.8	381	342	300	13
Os03g0252100	1348	1144	1088	492.6	371.5	744.8	371	310	504	1191	1107	1327	829.45	1105.2	942.44	3033	1540	1304	837.9	955.4	1061	730.5	840.2	1004	1510	1627	1476	1511	1050	1286	13
Os03g0728800	424	429	419	499.2	502.6	486.2	384	403	365	358.7	388.1	462.9	388.6	519	449.84	776.2	454.5	395.8	442.2	407.2	459.6	423.7	416.9	398	397.2	512	506.6	440	441	431	6
Os03g0174500	481	319	358	1105	1115	1285	835	769	965	378.5	263.1	341.5	330.91	497.53	245.51	394.1	282.4	322.2	1308	961.9	1365	766	843	738.1	248.2	380	502.2	312	289	262	10
Os03g0106800	0	0	0	0	0	0	0	0	0	0	0	0	0	0	0	0	0	0	0	0	0	1.479	0	0	0	0	0	0	0	0	9
Os03g0822800	508	489	573	733.1	684.3	688.6	1037	1141	1157	570.8	729.1	776.2	634.61	682.35	628.03	1314	650.3	635.1	896.4	920.8	988.1	742.3	838.1	939.6	693	746.7	725.9	515	638	583	6
Os03g0801900	78.5	96.9	83	75.53	53.33	62.28	178	141	164	94.84	71.27	49.86	46.806	65.342	49.102	47.76	57.71	42.48	54.81	36.28	42.25	165.6	144.7	176.2	39.09	56.57	48.24	48.1	34.4	33.3	18
Os03g0377100	0	0	0	0	0	0	0	0	0	0	0	0	0	0	0	0	1.031	0	0	0	0	0	0	0	2.113	0	0	0	0	0	1
Os03g0617900	1506	1382	1383	1222	1290	1183	556	620	750	920	971.4	1178	1040.6	1188.3	871.16	1015	1134	1298	1470	1308	1271	759.3	694.8	762.7	899	1026	1103	853	1001	849	16
Os03g0640800	11.2	6.52	7.54	1.642	5.424	7.785	5.48	6.33	8.29	16.38	13.16	14.11	8.7082	14.935	8.7116	0	19.58	5.18	9.29	4.838	0.862	1.479	3.563	10.11	6.338	9.428	21.93	10.2	7.65	10	20
Os03g0755800	181	94.1	241	48.44	77.74	51.9	92.4	75.9	80.8	125.9	149.1	116.7	149.13	155.89	111.67	214.9	158.7	112.9	75.25	115.3	99.15	93.16	59.86	80.89	136.3	156.5	160.1	127	144	119	13
Os03g0133900	1068	984	1038	359.6	387.8	342.6	584	676	776	995.8	1134	1265	1160.4	1193.9	1321	1911	1282	1250	476.6	411.2	506.1	629.2	501	678.2	1318	1408	1265	1133	1053	1167	13
Os03g0190100	516	443	419	912.9	987.1	881.5	985	1035	1032	512.1	559.2	988.8	622.63	430.32	375.39	716.5	409.1	521.1	949.4	1008	993.3	1062	1003	964.2	549.3	594	403.5	340	336	416	12
Os03g0614901	233	248	251	216.7	176.3	220.6	168	171	180	179.3	156.8	204.2	195.93	213.76	186.11	406	199.9	244.5	184.9	208	178.5	303.9	241.6	251.3	212.3	212.1	239	265	297	248	13
Os03g0150800	0	0	1.08	0	0	0.865	0.78	2.53	2.76	0.862	1.096	2.822	0	0	0	0	0	0	0	0	0	0.739	0	0	0	0.943	0	0	0	0	5
Os03g0828701	29.6	36.3	39.9	29.56	42.49	39.79	45.4	50	50.4	23.28	42.76	33.87	45.718	19.602	34.847	83.59	29.89	48.69	25.08	49.99	24.14	74.68	62	61.39	39.09	49.97	39.47	47.2	27.7	38.3	6
Os03g0576700	615	591	531	688	834.4	744.8	825	725	880	607	548.2	872.1	643.32	698.22	449.84	513.5	436.9	550.1	715.3	648.3	706.2	593.7	609.3	696.2	518.7	607.2	640.3	379	427	353	15
Os03g0175600	788	808	881	722.5	808.1	717.1	942	936	938	776	825.6	768.6	936.13	875.58	866.41	1182	913.1	750.1	841.6	866.8	867.4	856.2	801.7	944.7	787	823.1	813.6	775	847	818	6
Os03g0152700	595	784	652	1399	1808	1308	1459	1714	1435	751	962.6	1056	947.01	774.76	624.86	1111	560.6	671.4	1421	1646	1543	902.1	956.4	1171	776.4	686.4	609.6	438	528	560	12
Os03g0192900	412	423	440	508.2	476.4	446.4	323	386	324	530.2	524.1	476.1	410.37	439.66	435.58	955.3	480.2	393.7	619.6	577.3	601.8	273.6	261.5	294.7	479.6	483.7	447.4	486	532	437	6
Os03g0580200	28.5	34.5	44.2	124	101.2	123.7	92.4	108	101	140.5	67.98	38.57	42.452	83.077	45.142	119.4	19.58	34.19	288	122.6	113	47.32	46.32	49.11	49.65	18.86	15.35	32.4	45.9	30	10
Os03g0107900	115	139	123	137.9	125.7	145.3	171	139	179	117.3	131.6	103.5	151.3	103.61	112.46	394.1	131.9	135.7	170.9	154	115.5	157.5	131.8	154.6	145.8	142.4	120.6	141	158	152	6
Os03g0829000	285	258	250	272.6	248.6	224.9	345	341	312	291.4	235.7	293.5	215.53	289.37	207.5	334.3	228.8	217.6	237.8	214.5	263	235.9	275.1	273.7	248.2	269.7	287.3	246	200	203	5
Os03g0738900	28.5	27.9	39.9	73.89	60.57	64.88	54.8	63.9	66.3	47.42	55.92	31.05	43.541	37.338	44.35	47.76	35.04	32.12	51.09	58.86	54.32	68.02	77.68	72.22	61.27	33.94	35.09	45.3	37.3	56.7	12
Os03g0103200	145	83.8	120	385	209.7	42.39	127	194	317	249.2	316.9	117.6	244.92	84.011	103.75	167.2	45.34	53.87	240.6	14.51	31.9	40.67	39.91	40.45	70.78	50.91	6.579	25	53.6	91.6	7
Os03g0570200	110	114	101	110	76.84	108.1	123	131	131	139.7	146.9	187.2	109.94	108.28	118	119.4	106.1	137.8	96.61	110.5	83.64	170.8	153.2	146.6	196.5	167.8	190.8	138	142	149	20
Os03g0594400	64.2	62.4	78.7	89.49	92.2	72.66	147	120	158	55.18	42.76	39.51	65.311	28.937	47.518	71.65	40.19	39.37	78.96	89.5	57.77	190.8	161.1	200.1	66.55	58.46	74.56	46.3	41.1	67.5	18
Os03g0689866	29.6	15.8	15.1	40.23	18.98	29.41	25.1	24	44.9	34.49	35.08	42.34	18.505	27.07	26.135	47.76	19.58	15.54	30.66	23.38	31.9	46.58	47.75	62.11	24.3	21.69	8.772	15.7	24.9	25.8	9
Os03g0329500	287	265	248	460.6	588.5	428.2	327	318	338	250.9	243.4	265.3	274.31	266.03	211.45	274.6	210.2	205.1	339.1	359.6	376.8	232.9	210.2	252.8	237.7	225.3	287.3	142	145	190	10
Os03g0365000	7.14	6.52	6.47	8.21	13.56	10.38	13.3	18.3	15.2	1.724	7.675	4.704	6.5311	0.9335	3.1679	11.94	2.061	2.072	7.432	7.257	6.898	4.436	4.988	7.945	2.113	1.886	0	3.7	6.69	6.66	12
Os03g0101000	33.6	50.3	34.5	70.6	76.84	57.96	116	129	128	54.32	51.53	75.27	42.452	41.072	36.43	59.7	31.95	44.55	45.52	95.95	77.6	93.9	82.67	100.4	31.69	32.06	32.89	43.5	32.5	24.2	18
Os03g0327700	27.5	37.3	29.1	47.62	66.89	35.47	75.2	89.2	82.2	30.18	39.47	35.75	32.656	30.804	37.222	11.94	9.275	41.44	33.44	24.99	35.35	39.19	53.45	39.72	23.24	17.91	8.772	15.7	9.56	27.5	18
Os03g0310000	25.5	30.7	29.1	8.21	3.616	2.595	7.05	7.59	6.91	11.21	8.771	18.82	11.974	11.201	9.5036	71.65	22.67	17.61	13.93	8.869	4.311	11.09	19.24	17.33	16.9	8.486	4.386	15.7	17.2	15.8	13
Os03g0351200	851	591	834	584.5	550.5	533.7	521	469	413	1061	1141	1541	1386.8	1080	1159.4	1576	1098	1036	881.6	937.7	962.2	229.9	268	358.2	1124	1141	1024	847	819	905	3
Os03g0783051	0	0	0	0.821	0	1.73	0	0	0	0	0	0	0	0	0	0	0	0	0	0	0	0	0	0	0	0	2.193	0.93	0	0	11
Os03g0375933	1.02	0.93	0	4.105	2.712	2.595	0	6.33	0.69	0	0	0	0	2.8004	1.5839	0	3.092	0	0	0	0	3.697	7.839	1.444	2.113	1.886	0	1.85	1.91	0.83	17
Os03g0834000	836	878	856	1110	1082	1034	802	996	954	722.5	822.3	821.3	994.91	769.16	795.93	1134	666.8	891	933.6	1155	1000	1122	942.8	971.2	784.9	734.5	546	711	738	781	10
Os03g0265700	1021	1088	997	1078	1116	1202	835	771	896	869.1	838.7	746.1	963.34	942.79	896.51	1385	1040	972.8	915	942.5	979.5	1034	920	878.2	952.8	891.9	890.3	1081	1001	1080	8
Os03g0448600	225	180	208	197	232.3	230.1	178	204	212	155.2	210.5	189.1	197.02	201.63	184.53	274.6	226.7	187.5	289.8	250.8	224.2	214.4	253	218.1	184.9	165	162.3	192	203	165	6
Os03g0594500	0	1.86	0	1.642	0	2.595	5.48	0.63	0	0	1.096	0	0	0	0	0	0	2.072	2.787	1.613	2.587	3.697	2.851	5.056	2.113	0.943	2.193	0	0	0.83	9
Os03g0149800	6.12	4.66	2.16	13.14	7.232	9.516	6.26	0	4.84	2.587	2.193	2.822	4.3541	2.8004	5.5438	0	3.092	2.072	13.01	9.675	6.036	48.8	14.25	29.61	2.113	1.886	0	2.78	4.78	2.5	9
Os03g0811100	1711	2077	2005	3005	3127	2870	2905	3355	3272	1723	2039	2193	1710.1	1627	1401.8	2137	1351	1838	2281	2513	2507	2752	2208	2923	1543	1452	1300	1144	1286	1304	12
Os03g0308850	86.7	88.5	72.2	141.2	164.5	179.9	91.6	113	98.8	106.9	94.29	77.15	113.21	136.28	66.525	119.4	99.96	119.1	140.3	176.6	210.4	77.64	72.69	70.78	76.06	97.11	78.95	113	88	71.6	10
Os03g0184300	8.16	12.1	4.31	8.21	23.5	18.17	14.1	8.22	15.9	6.897	8.771	7.527	0	14.002	1.5839	0	4.122	4.144	16.72	7.257	3.449	2.958	7.839	14.44	3.169	6.6	13.16	0	2.87	5.83	12
Os03g0694000	18.4	8.38	2.16	6.568	10.85	13.84	6.26	4.43	6.5	12.93	13.16	19.76	13.062	9.3345	2.3759	0	0	2.072	3.716	1.613	2.587	4.436	0	6.5	6.338	22.63	21.93	0	8.61	1.67	15
Os03g0643900	0	0	1.08	2.463	0.904	1.73	3.13	0.63	0.69	0.862	0	1.882	1.0885	0	0.792	0	1.031	0	0	1.613	3.449	6.654	2.138	10.83	0	2.829	0	1.85	0.96	0.83	9
Os03g0198300	4425	4422	4583	1722	1624	1516	2431	2901	2669	5419	5865	5060	6557.2	5995.6	7168.9	7320	5234	5039	2148	1877	2195	2182	2291	2583	6084	6278	5351	5186	5573	5744	3
Os03g0267800	95.8	53.1	73.3	37.76	45.2	48.44	40.7	34.2	38.7	59.49	51.53	63.98	34.833	96.146	50.686	35.82	77.29	76.67	57.6	45.15	32.76	73.94	57.01	62.84	68.66	85.8	72.37	79.6	88	86.6	1
Os03g0284500	686	548	685	181.4	187.1	221.5	163	189	164	462.1	504.3	528.7	748.9	813.97	715.15	1863	936.8	858.9	276.8	296.7	250	161.9	252.3	205.1	749	690.2	682	807	716	786	13
Os03g0130900	466	539	514	509.8	440.2	463.7	312	376	256	332.8	347.6	308.6	487.66	461.13	414.2	1230	667.8	545	432	496.7	494.9	249.2	282.2	278.1	415.2	466.7	445.2	564	565	560	13
Os03g0200500	2577	2199	2342	2956	2946	3214	2488	2299	2826	2394	2279	3476	2604.8	2889	1855.6	4000	1972	2337	3046	2590	3010	2423	2537	2345	2133	2868	2939	1675	2041	1508	6
Os03g0221100	174	215	210	210.2	222.4	179.9	110	141	158	155.2	207.2	128	217.7	173.62	197.2	250.8	242.2	216.5	122.6	167.7	150	176.7	162.5	140.8	171.1	161.2	107.5	325	243	237	8
Os03g0564600	0	0	0	0	0.904	0	0.78	0	0	0	0	0	0	0.9335	0	0	0	0	0	0	0	0.739	0	0.722	0	0	2.193	0	0	0	11
Os03g0235200	0	8.38	5.39	51.72	49.72	34.6	20.4	29.1	15.2	3.449	2.193	2.822	3.2656	0.9335	1.5839	0	2.061	3.108	39.02	48.38	37.08	13.31	12.83	17.33	1.056	0.943	0	0.93	4.78	5	10
Os03g0358800	4.08	0	0	0	0	0	0	0	0	0	0	0	2.177	0.9335	0	0	0	0	0	0	0	0	0.713	0	0	0	0	0	0	0	19
Os03g0783100	0	0	0	2.463	0	0	0	0	0	0.862	0	0	0	1.8669	0.792	0	0	0	0	0	0	0	0	0	1.056	0.943	0	0	0.96	0	4
Os03g0335100	0	0.93	0	0	0.904	0	0	0	0	0	0	0	0	0	0	0	0	1.036	0	0	0	0	0	0	0	0	0	0	0	0	14
Os03g0732000	0	0	0	0	0	0	0	0	0	0	1.096	0	0	0	0	0	0	0	0	0	0	0	0	0	0	0	0	0	0.96	0	7
Os03g0754900	248	264	258	334.1	335.4	308.8	318	359	334	241.4	281.8	213.6	272.13	272.57	312.03	465.7	299.9	300.5	315.8	353.2	342.3	295	263	294	333.8	259.3	293.9	288	306	294	6
Os03g0624300	2.04	1.86	1.08	28.73	43.39	16.44	119	55	76.7	21.55	4.386	4.704	7.6196	2.8004	3.1679	0	1.031	1.036	13.01	16.93	8.622	41.41	39.19	29.61	0	0	0	0.93	0	0.83	18
Os03g0206900	402	370	390	321.8	311	340.8	491	490	572	438	320.1	407.4	498.54	445.26	364.3	991.1	371	368.8	366.9	409.6	404.4	387.4	367	461.5	365.5	398.8	399.1	337	354	354	6
Os03g0667100	987	1202	1117	408.8	438.4	398.8	526	701	568	637.2	608.5	617.2	559.5	856.91	578.14	740.3	678.1	648.6	426.4	312.8	338	218.9	338.5	268.7	562	595.9	633.8	501	446	497	14
Os03g0165800	1.02	0	0	0	0	0	0	0	0	0	0	0	1.0885	0	0	0	0	0	0	0	0	0	0	0	0	0	0	0	0	0	19
Os03g0751600	133	83.8	78.7	18.88	12.66	27.68	66.5	70.8	33.9	102.6	91	182.5	80.55	100.81	70.485	179.1	151.5	111.9	31.58	16.13	30.18	36.97	20.67	44.06	112	134.8	175.4	74	78.4	71.6	3
Os03g0137700	2.04	4.66	2.16	2.463	5.424	8.65	14.1	7.59	4.84	3.449	5.482	0.941	6.5311	9.3345	6.3357	0	6.183	0	4.645	7.257	9.484	0.739	4.988	7.945	2.113	2.829	4.386	1.85	2.87	7.5	15
Os03g0806300	6.12	0	1.08	0.821	4.52	1.73	1.57	0.63	2.07	0.862	0	0	0	0.9335	2.3759	0	0	4.144	2.787	1.613	6.898	4.436	0	1.444	5.282	0.943	0	0	3.83	0	19
Os03g0786800	942	870	928	960.5	972.7	958.5	1287	1280	1205	1138	1124	1345	1160.4	1264.8	1147.6	1385	932.6	1014	1244	1098	1215	876.2	901.5	1015	1213	1197	1175	993	998	959	15
Os03g0715600	0	0	0	0	0	0	0	0	1.38	0	0	0	0	0	0	0	0	0	0	0	0	0	0	0	0	0	0	0	0	0	18
Os03g0823900	247	253	219	169.9	190.7	204.2	138	132	98.8	280.2	247.8	303.9	277.57	280.97	260.56	382.1	249.4	243.5	183	164.5	199.2	159	151.1	140.1	256.7	307.4	287.3	276	269	262	13
Os03g0410300	454	507	533	532.8	547.8	500	711	717	683	657.8	599.7	635.1	641.14	634.75	603.48	668.7	653.4	519.1	669.8	621.6	657.9	522.7	542.3	556.1	685.6	682.6	576.7	517	622	652	5
Os03g0195100	42.8	14	2.16	7.389	14.46	7.785	284	31.6	561	7.76	7.675	10.35	3.2656	6.5342	8.7116	0	0	3.108	11.15	33.06	5.173	39.19	65.56	109.1	1.056	5.657	17.54	4.63	8.61	5.83	18
Os03g0806600	254	279	367	398.2	490	281.1	421	436	895	425.1	309.2	141.1	269.95	349.11	403.9	394.1	349.4	204.1	379	430.6	191.4	1097	915	827	253.5	204.6	261	323	339	429	17
Os03g0252900	1.02	1.86	4.31	0	0	0	0	0	0	0	3.289	1.882	4.3541	21.469	3.1679	0	3.092	2.072	0	0	0	0	0	0	1.056	0	0	4.63	2.87	2.5	4
Os03g0294032	8.16	11.2	4.31	9.031	7.232	17.3	3.91	0.63	0.69	4.311	5.482	0.941	0	1.8669	4.7518	23.88	10.31	8.288	3.716	6.45	12.07	19.22	19.24	31.78	7.395	15.09	24.12	12	11.5	3.33	9
Os03g0160300	0	1.86	2.16	0.821	0	1.73	0	0	1.38	0.862	0	0.941	3.2656	0	0	0	0	1.036	1.858	0	0	2.958	0.713	0	0	0	0	2.78	0.96	1.67	8
Os03g0346200	0	0	0	0	0	0.865	0	0	0	0	0	0	0	0	0	0	0	0	0.929	0	0	0	0	0	0	0	0	0	0	0	16
Os03g0695700	998	1111	1057	788.1	775.6	790.7	1074	1170	1005	1096	1046	1088	998.17	1056.7	1099.2	1958	1125	1053	943.8	893.4	887.2	1168	1107	1160	1151	1157	1217	1154	1010	1127	13
Os03g0638000	23.4	28.9	12.9	9.031	16.27	12.11	2.35	0.63	6.91	6.035	9.868	10.35	5.4426	13.068	8.7116	71.65	29.89	18.65	6.503	5.644	6.898	12.57	17.82	19.5	23.24	19.8	8.772	46.3	31.6	29.2	13
Os03g0107400	133	126	90.5	78.81	72.32	76.99	17.2	25.9	10.4	30.18	21.93	13.17	25.036	22.403	41.182	191.1	109.2	79.77	30.66	27.41	19.83	79.85	135.4	106.9	66.55	56.57	48.24	155	94.7	116	8
Os03g0306900	745	791	807	641.2	749.4	620.2	421	426	321	797.5	651.3	557.9	586.71	548.87	653.37	800	611.1	634.1	538.8	543.4	533.7	230.7	344.9	273.7	528.2	460.1	515.3	501	500	554	14
Os03g0638200	360	346	422	14.78	13.56	8.65	15.7	33.5	42.8	344	365.1	445	422.35	314.57	390.44	406	337	500.4	8.361	20.16	8.622	51.76	39.19	37.56	454.2	383.7	344.3	270	340	371	3

Os03g0747600	531	614	575	614.1	627.4	620.2	489	474	443	486.3	562.5	371.6	584.54	472.33	607.44	1122	496.7	462.1	485.8	470.9	460.4	518.3	508.8	450.7	556.7	448.8	464.9	584	562	612	6
Os03g0379500	0	0	0	0	0	0	0	0	0	0	0	0	0	0	0	0	0	0	1.858	0.806	0	0.739	0	0	0	0	0	0	0	0	16
Os03g0157600	3.06	0	0	0	0	0.865	1.57	4.43	0	0	0	2.822	0	0	0	0	4.122	1.036	2.787	0.806	0.862	0	2.851	0	3.169	0.943	10.96	0	0	0	11
Os03g0601000	66.3	47.5	48.5	55.01	29.83	49.31	37.6	15.8	18.7	56.9	33.99	41.4	30.479	30.804	26.927	59.7	46.37	43.51	32.51	35.48	32.76	34.01	53.45	40.45	60.21	44.31	41.67	51.8	43	64.2	8
Os03g0608600	38.7	34.5	42	50.9	39.77	37.2	50.9	58.2	60.1	56.9	47.14	29.17	20.682	42.005	36.43	83.59	39.16	24.86	44.59	42.73	61.22	33.27	34.21	60.67	47.54	45.26	26.32	37	45.9	23.3	6
Os03g0656500	247	205	190	66.5	51.53	69.2	83	58.8	67.7	131.1	123.9	114.8	132.8	121.35	120.38	477.6	265.9	193.7	115.2	112.9	135.4	65.07	101.2	101.1	110.9	150.9	153.5	147	159	137	13
Os03g0297800	0	0.93	0	0	0	0	0	0.63	0	0	0	0	0	0	0	0	0	0	0	0	0	0	1.425	1.444	0	0	0	0	1.91	0	17
Os03g0395300	266	306	360	310.3	351.6	253.5	362	404	294	285.4	281.8	223	217.7	232.43	287.48	632.9	231.9	278.7	297.3	304.8	220.7	397.8	543	437	260.9	257.4	267.5	283	253	274	6
Os03g0367900	559	523	475	614.1	515.3	711.9	632	681	798	488.9	447.3	602.1	499.63	525.53	452.21	537.3	542.1	522.2	567.6	623.3	705.3	896.9	1077	1130	683.5	696.8	673.2	661	487	656	9
Os03g0729600	0	0	0	0.821	3.616	0	1.57	5.06	0.69	0	2.193	0.941	0	0	0	0	0	0	0	0	0	0	3.563	0.722	0	0	0	0	0	0	18
Os03g0284900	1124	1134	1105	2245	2366	2235	1713	1899	1702	1229	1364	1325	1308.4	1217.2	1299.6	2723	1167	1205	2307	2399	2374	1408	1388	1491	1440	1386	1077	1180	1222	1207	10
Os03g0271300	1.02	1.86	0	7.389	7.232	16.44	10.2	10.8	12.4	0.862	2.193	0	0	0.9335	0.792	0	2.061	3.108	13.93	8.063	10.35	5.915	7.126	6.5	0	0	0	0	0.96	0.83	12
Os03g0277600	0	1.86	0	0	0	0	0	0	4.15	0	0	0	0	0	0	0	0	0	0	0	0	1.479	20.67	1.444	0	0	0	1.85	0.96	0	17
Os03g0102400	812	875	800	644.5	596.6	649.7	816	758	831	819.1	806.9	677.4	702.1	694.49	753.16	1349	721.4	697.3	572.2	566.8	582	977.5	927.1	871	725.7	749.6	675.4	726	808	809	6
Os03g0108700	137	146	136	137.1	141	138.4	320	266	230	182.8	154.6	193.8	139.33	167.09	133.84	226.9	144.3	110.9	199.7	200	183.7	232.9	263	240.5	152.1	130.1	193	175	146	147	18
Os03g0756700	388	415	408	574.7	531.5	562.3	559	562	544	363.8	385.9	313.3	434.32	350.04	424.49	597	372	439.3	440.3	478.9	499.2	510.9	552.3	470.9	383.5	351.7	309.2	377	377	434	12
Os03g0562200	89.7	118	117	104.3	111.2	93.43	222	215	227	139.7	122.8	158.1	120.83	114.81	141.76	131.4	105.1	105.7	126.3	118.5	91.4	162.7	148.9	160.3	113	99	107.5	96.2	95.6	84.1	18
Os03g0851200	1607	1595	1777	814.4	810.9	875.4	873	941	1067	2599	2717	3076	2627.7	2421.4	2779	3033	2057	1919	1296	1313	1261	619.6	798.9	918	2887	2697	2544	1693	2143	2002	3
Os03g0815700	166	339	255	555.8	539.7	493.9	430	556	394	289.7	332.2	240.8	364.65	284.7	376.98	561.2	186.5	230	405	444.3	357	402.2	473.2	372.7	323.2	233.8	217.1	308	331	367	12
Os03g0826300	4.08	2.79	3.23	0.821	0	0	0	0	0	0	0	0	4.3541	0	1.5839	0	2.061	2.072	0	0	0	1.479	5.701	2.889	1.056	2.829	0	1.85	2.87	0	14
Os03g0192550	31.6	61.5	31.2	0.821	0	0.865	0	0	0	21.55	9.868	23.52	23.947	22.403	19.007	35.82	41.22	26.94	2.787	2.419	1.724	1.479	0	2.889	21.13	6.6	2.193	25.9	25.8	40.8	14
Os03g0565200	7257	6854	7706	2835	2708	2718	6102	6022	6694	8419	8770	12571	8869.3	9973.9	8809.8	11069	9314	8315	4805	3484	5191	3959	3629	6048	9491	10989	10480	6160	7129	6384	3
Os03g0308700	5.1	0.93	1.08	0.821	1.808	0	6.26	1.27	1.38	0.862	2.193	4.704	3.2656	1.8669	3.9598	11.94	1.031	1.036	0	0	0	1.479	0	3.611	0	1.886	2.193	3.7	0	2.5	6
Os03g0249900	204	197	191	273.4	250.4	248.3	287	283	282	237.1	259.8	262.5	251.45	224.96	243.13	704.5	232.9	262.1	280.5	285.4	252.6	183.4	190.3	169.7	191.2	231	219.3	191	234	212	6
Os03g0619700	1.02	0	0	0.821	0.904	2.595	0	0	0	0.862	1.096	0	1.0885	0.9335	0	107.5	71.11	45.59	42.73	62.89	61.22	71.72	74.83	78.72	57.04	42.43	59.21	69.4	66	72.5	1
Os03g0417700	13.3	20.5	14	15.6	10.85	19.03	14.1	17.1	10.4	79.32	37.28	52.69	47.895	30.804	54.646	0	9.275	19.68	2.787	7.257	5.173	15.53	15.68	5.778	6.338	8.486	15.35	16.7	4.78	16.7	7
Os03g0697466	3.06	2.79	0	0.821	0.904	0.865	0	0.63	2.76	0.862	0	2.822	0	0	0	0	0	1.036	3.716	0	0	5.176	0.713	2.889	3.169	1.886	2.193	1.85	0	1.67	9
Os03g0711400	373	366	365	393.2	365.2	446.4	529	479	508	388.8	364	397	360.3	384.58	338.17	501.5	345.2	318.1	387.4	366.9	375.1	386.7	420.5	398.7	358.1	371.5	390.3	314	351	289	18
Os03g0846900	99.9	122	129	200.3	202.5	173.9	128	161	161	100.9	103.1	125.1	136.06	104.55	102.16	155.2	89.66	135.7	179.3	191.9	180.2	144.2	125.4	177.7	95.07	122.6	89.91	108	95.6	109	10
Os03g0330200	6.12	5.59	0	8.21	16.27	13.84	6.26	2.53	8.29	6.897	24.12	12.23	9.7967	17.736	11.088	35.82	12.37	10.36	3.716	4.031	1.724	2.958	6.414	0.722	7.395	7.543	6.579	11.1	0.96	16.7	6
Os03g0321900	153	183	156	249.6	241.4	249.1	163	168	172	150	171	182.5	209	177.36	144.14	406	202	194.8	273.1	234.6	261.3	252.9	211.7	218.8	180.6	196.1	184.2	188	187	186	6
Os03g0129850	2.04	1.86	1.08	0	0	0	0	1.27	0.69	3.449	1.096	0	1.0885	0	0.792	0	0	5.18	0	1.613	0	0.739	1.425	0	1.056	1.886	4.386	2.78	0	0	11
Os03g0351700	9.18	4.66	8.62	17.24	7.232	12.98	25.8	13.3	35.2	12.07	3.289	12.23	17.416	8.4011	15.047	0	12.37	4.144	15.79	16.93	6.898	10.35	22.8	15.17	11.62	12.26	13.16	15.7	9.56	17.5	18
Os03g0816600	3.06	8.38	4.31	64.04	51.53	46.71	14.9	25.9	17.3	1.724	1.096	3.763	5.4426	1.8669	3.1679	11.94	2.061	1.036	29.73	58.86	36.21	19.96	17.82	19.5	0	3.771	2.193	0.93	4.78	1.67	10
Os03g0695600	695	755	748	753.7	841.6	809.7	983	972	975	807.9	718.1	795.9	644.4	834.51	728.61	1003	760.5	682.7	873.2	682.9	795.8	720.2	845.2	823.4	741.6	735.4	828.9	663	748	625	18
Os03g0651800	0	1.86	1.08	0	0	0	2.35	1.27	4.84	0.862	0	0	0	0	0.792	0	0	0	0	1.613	0	2.958	0.713	1.444	0	0	0	0	0	0	18
Os03g0771500	0	0	1.08	0	0	0	0	0	0	0	0	0	0	0	0	0	0	0	0	0	0	0	0	0	0	0	0	0	0	0	14
Os03g0734300	17.3	15.8	8.62	19.7	15.37	30.28	15.7	6.96	10.4	27.59	13.16	22.58	14.151	5.6007	5.5438	0	3.092	13.47	7.432	4.838	6.898	3.697	46.32	16.61	6.338	12.26	10.96	16.7	25.8	12.5	17
Os03g0835900	211	177	166	175.7	166.3	179.9	209	194	185	221.6	194.1	265.3	182.87	201.63	208.29	286.6	122.6	154.4	192.3	220.1	181.1	156.7	142.5	168.3	174.3	198	206.1	166	179	113	6
Os03g0177500	4031	3406	3301	4009	4508	4582	4788	4184	5214	4443	3544	6319	3895.8	4204.3	2940.6	2233	3189	3513	4258	3906	4497	3714	3736	3975	3398	4353	4261	2659	3374	2371	15
Os03g0401200	301	319	288	320.2	301.9	290.7	301	248	252	316.4	343.2	271	236.21	194.16	274.02	406	253.5	284.9	317.7	294.3	276.8	236.6	283.6	246.3	289.4	269.7	324.6	233	303	268	6
Os03g0410900	161	141	123	332.5	348	441.2	264	199	311	124.2	107.4	190	118.65	167.09	104.54	214.9	119.5	127.4	415.2	392.7	429.4	264	234.5	220.3	108.8	133.9	171	108	117	98.3	10
Os03g0184500	0	0.93	0	1.642	0	0	0	0	1.38	0	0	0	0	0	0	0	0	0	1.858	0	0.862	0	0.713	0	0	0	0	0	0.96	0	16
Os03g0668400	125	152	152	119	140.1	149.7	173	170	248	140.5	185.3	146.8	126.27	98.012	121.17	143.3	126.8	149.2	163.5	157.2	181.1	269.1	274.4	212.3	129.9	159.3	151.3	125	159	123	17
Os03g0850400	872	762	625	889.1	630.1	750	40.7	101	168	1110	945.1	1800	370.1	387.38	358.76	895.6	351.4	826.8	652.1	546.7	884.6	170.1	139	116.3	509.2	1278	861.8	203	262	159	20
Os03g0259600	7.14	0.93	4.31	0	0.904	0	15.7	2.53	4.84	1.724	2.193	2.822	1.0885	2.8004	3.9598	0	8.244	2.072	4.645	4.031	9.484	4.436	6.414	7.945	8.451	0.943	0	0.93	3.83	4.17	5
Os03g0196500	1.02	0.93	1.08	0	0	0	0.78	0	0	0.862	2.193	4.704	0	3.7338	0	11.94	3.092	4.144	0.929	2.419	1.724	0	0	7.222	0	4.714	2.193	0.93	3.83	1.67	6
Os03g0796900	150	108	120	120.7	128.4	163.5	166	194	191	136.2	146.9	135.5	173.07	134.42	144.93	274.6	142.2	125.4	113.3	129	132.8	134.6	171	149.5	151.1	116.9	164.5	141	98.5	137	6
Os03g0351400	306	344	318	160.9	159.1	143.6	314	331	294	309.5	346.5	308.6	428.88	377.11	422.91	823.9	447.3	389.5	135.6	170.9	169.9	312	302.9	301.2	422.5	463.9	407.9	378	355	402	13
Os03g0752200	70.3	83.8	81.9	77.17	56.95	73.53	126	97.4	125	106	89.9	67.74	74.019	95.212	72.861	95.53	93.78	82.88	74.32	81.43	103.5	118.3	136.8	124.2	90.85	95.23	114	89.7	91.8	109	9
Os03g0264800	28.5	30.7	19.4	24.63	23.5	28.55	25.8	27.8	19.3	31.04	17.54	16.93	10.885	11.201	14.255	47.76	41.22	34.19	33.44	20.16	28.45	29.58	27.79	18.78	20.07	26.4	19.74	26.8	31.6	19.2	6
Os03g0397600	0	1.86	0	0	0	0.865	1.57	0	0.69	0	0	0	0	0	0	0	0	0	0	1.613	1.724	1.479	0.713	0	0	0	0	0.93	1.91	0	17
Os03g0426900	6083	5115	6594	3577	3845	3631	3168	3613	3445	7237	7155	7665	9887	9907.7	8917.5	10293	5315	5775	3492	3232	3683	2337	2275	2478	6092	5372	5002	5068	5378	5324	13
Os03g0701400	742	545	612	346.5	340.8	341.7	240	246	231	654.4	604.1	676.4	543.17	479.79	522.7	871.7	557.5	651.7	357.6	395.1	372.5	215.2	242.3	224.6	534.5	492.2	475.9	514	491	525	13
Os03g0201500	0	0	0	0	0	0	0	0	0	0	0	0	0	0	0	0	0	0	0.929	0	0	0	0	0	0	0	0	0	0	0	16
Os03g0431100	836	961	867	756.1	755.7	754.3	1204	1459	1513	913.9	999.9	1358	982.93	918.52	909.97	847.8	900.7	905.5	844.4	738.6	798.4	1619	1483	1932	1036	1206	1224	748	887	964	9
Os03g0199500	0	0	0	0.821	0.904	0	0	0	0	0	0	0	0	0	0	0	0	0	0.929	0	0	0	0	0	0	0	0	0	0	0	10
Os03g0740900	42.8	40.1	42	60.75	25.31	69.2	3.91	19	8.98	12.93	8.771	0.941	7.6196	8.4011	21.383	35.82	24.73	24.86	21.37	39.51	8.622	154.5	119	33.22	19.01	20.74	32.89	64.8	50.7	55.8	17
Os03g0862200	26.5	19.6	9.7	16.42	8.136	14.71	18.8	17.7	18.7	26.73	26.31	24.46	13.062	24.27	20.591	0	15.46	10.36	19.51	14.51	33.63	25.14	19.95	20.95	15.85	24.51	17.54	14.8	12.4	9.16	15
Os03g0366800	19.4	14	18.3	14.78	15.37	15.57	21.1	11.4	18	18.97	17.54	15.05	15.239	8.4011	17.423	11.94	14.43	8.288	18.58	4.031	8.622	17.01	14.97	16.61	22.18	15.09	15.35	14.8	23.9	16.7	7
Os03g0749300	34.7	12.1	14	35.3	20.79	74.39	21.1	24	17.3	21.55	16.45	58.33	28.302	17.736	17.423	23.88	41.22	16.58	58.52	42.73	63.8	51.76	67.7	52.72	15.85	55.63	144.7	23.1	43	15.8	11
Os03g0103300	13.3	13	18.3	129.7	48.81	34.6	40.7	22.8	68.4	36.21	41.66	21.64	27.213	14.935	13.463	59.7	8.244	8.288	60.38	29.03	14.66	45.84	28.51	30.33	14.79	19.8	2.193	13	10.5	15.8	2
Os03g0331700	1058	737	838	54.18	43.39	46.71	40.7	58.2	73.2	550.1	389.2	404.6	340.71	345.38	374.6	489.6	693.5	640.3	59.45	68.53	64.67	78.37	119.7	106.9	349.7	353.6	418.8	248	289	279	14
Os03g0431200	1084	1055	970	362	213.3	383.2	73.6	140	205	1591	1358	1761	704.27	861.58	1023.2	859.7	923.4	1123	366.9	278.2	413.9	357.1	257.3	168.3	984.5	1573	1333	543	730	722	3
Os03g0228200	4043	4652	5022	284.1	262.2	332.2	6.26	5.06	4.84	5210	6294	5380	7098.2	5862.1	6072.8	10866	5670	5126	644.7	592.6	563	2.958	7.839	2.167	6616	6652	5171	5433	5745	6432	13
Os03g0616400	247	401	259	3283	3649	3219	896	1090	1025	378.5	494.5	264.4	350.5	257.63	258.97	334.3	208.2	268.3	2051	2331	2135	1445	1142	996	320.1	272.5	175.4	206	202	312	10
Os03g0596900	5085	4844	5168	4726	4765	4743	3640	3848	3290	5540	5310	5476	7387.8	6789	6482.2	9326	5697	5288	4925	4523	4720	2852	3235	3096	5791	5967	5454	6181	5788	6067	13
Os03g0299600	1458	1021	976	1062	872.3	817.5	564	619	434	1041	737.9	1020	701.01	599.28	701.68	609	783.2	788.4	1232	1054	1324	773.4	853	974.3	432.1	649.6	943	373	492	337	19
Os03g0163400	4.08	5.59	7.54	8.21	13.56	8.65	10.2	6.33	7.6	10.35	8.771	15.05	16.328	7.4676	11.088	0	8.244	15.54	8.361	7.257	12.93	5.915	18.53	15.17	16.9	6.6	10.96	20.4	17.2	11.7	1
Os03g0181500	45.9	31.7	34.5	12.31	10.85	24.22	31.3	20.9	48.4	33.63	62.49	64.92	50.072	31.737	36.43	11.94	26.79	15.54	15.79	13.71	17.24	26.62	36.34	49.11	59.16	42.43	78.95	42.6	23	20.8	20
Os03g0129400	573	950	678	973.7	744	444.6	421	584	654	1562	1930	1943	675.97	735.56	1700.4	680.6	760.5	868.2	866.7	591	1167	364.5	377	342.3	1226	2105	2044	486	704	726	20
Os03g0130950	28.5	27	25.9	18.88	28.93	23.36	25.1	47.4	35.2	12.07	16.45	1.882	23.947	16.802	22.175	0	12.37	20.72	29.73	19.35	10.35	27.36	39.19	20.22	13.73	10.37	8.772	19.4	11.5	21.7	18
Os03g0737400	439	472	472	536.1	660.8	535.5	913	975	985	513	529.6	479.8	554.06	560.07	504.48	537.3	458.6	449.6	678.1	652.3	711.3	737.9	693.4	811.1	493.3	509.1	521.9	440	471	478	18
Os03g0789200	349	364	349	173.2	180.8	194.6	169	196	209	275.9	322.3	302.9	387.51	329.51	369.06	585.1	432.8	337.7	190.4	220.9	216.4	219.6	223.8	231.1	412	355.5	344.3	385	455	459	13
Os03g0597400	4.08	8.38	9.7	9.852	13.56	6.92	9.39	10.8	13.1	8.622	8.771	12.23	6.5311	9.3345	4.7518	11.94	18.55	5.18	13.93	10.48	6.036	6.654	14.25	12.28	8.451	8.486	28.51	8.33	4.78	5.83	11
Os03g0356477	0	0	1.08	0.821	0	0	1.57	0.63	1.38	0	0	0.941	2.177	0	0.792	0	0	0	0.929	0	0	0	2.851	3.611	1.056	4.714	0	1.85	3.83	0	1
Os03g0577350	0	0	0	0	0	0	0	0	0	0	0	0	0	0	0	0	0	0	0.929	0	0	0.739	0	0	0	0	0	0	0	0	9
Os03g0816500	238	170	173	189.6	127.5	228.4	63.4	57.6	47.7	141.4	111.8	139.2	83.816	133.48	130.67	310.5	321.5	137.8	154.2	161.3	186.2	135.3	119.7	130	152.1	197.1	203.9	220	178	140	13
Os03g0127500	29.6	6.52	5.39	3.284	0	18.17	0.78	0	2.07	4.311	0	10.35	0	6.5342	1.5839	0	21.64	13.47	6.503	4.838	16.38	1.479	2.851	5.056	4.225	16.97	21.93	11.1	9.56	2.5	19
Os03g0423800	460	507	501	418.7	397.7	420.4	355	428	381	432.8	459.4	396.1	477.86	468.59	510.03	465.7	533.8	599.9	360.4	385.4	369	549.4	472.5	437	490.2	504.4	456.1	555	565	638	1
Os03g0372700	268	224	252	242.2	238.6	242.2	289	209	268	374.2	355.2	431.8	303.7	318.31	266.89	513.5	235	241.4	339.1	300.7	329.4	203.3	213.1	203	304.2	362.1	350.9	213	274	212	20
Os03g0849700	72.4	71.7	84.1	138.7	116.6	135.8	68.9	70.8	58	30.18	32.89	38.57	62.046	69.075	45.934	167.2	56.68	71.49	123.6	140.3	106.9	105.7	97.63	70.06	38.03	35.83	26.32	74	59.3	69.1	10
Os03g0761700	211	263	232	329.2	308.3	277.7	277	252	308	230.2	251.1	221.1	216.62	216.56	248.68	358.2	227.7	208.2	252.7	318.5	281.1	279.5	238.7	257.1	215.5	231	247.8	225	235	260	6
Os03g0740200	8.16	0	1.08	0	0	0	0	0	0	0.862	0	3.763	0	0.9335	1.5839	0	1.031	2.072	0	0	0.862	0	0	2.889	5.282	0	2.193	0	0.96	0.83	19
Os03g0231700	70.3	69.9	65.7	83.74	93.11	62.28	38.4	20.2	24.9	43.11	48.24	36.69	42.452	38.272	45.142	47.76	73.17	56.98	53.88	67.73	92.26	33.27	30.64	26.72	35.92	50.91	39.47	71.2	58.3	68.3	16
Os03g0674300	195	153	164	163.4	157.3	158.3	189	169	167	163	155.7	222	161.1	191.36	186.11	107.5	199.9	170.9	203.4	197.5	191.4	104.3	144	158.9	191.2	175.4	201.7	182	164	167	20
Os03g0164300	28.5	72.7	39.9	42.69	25.31	50.17	97.1	97.4	121	65.53	93.19	77.15	67.488	64.408	69.693	0	23.7	26.94	31.58	24.99	15.52	79.11	78.39	91.73	98.24	37.71	50.44	52.7	20.1	46.7	5
Os03g0598100	83.6	92.2	128	9.852	29.83	13.84	51.7	31.6	52.5	150.9	200.6	178.8	180.69	224.96	165.52	203	236	179.2	42.73	40.31	27.59	25.14	28.51	35.39	181.7	198.9	179.8	211	167	223	3
Os03g0709533	1.02	0.93	1.08	2.463	0.904	0.865	0	1.27	0	2.587	1.096	4.704	3.2656	0	2.3759	0	1.031	2.072	3.716	0.806	1.724	0	2.138	0.722	5.282	0.943	0	0	1.91	0.83	20
Os03g0796800	169	250	245	261.1	245	278.5	232	238	236	205.2	196.3	156.2	193.76	205.36	205.12	394.1	210.2	195.8	181.1	216.1	215.6	257.3	268.7	208	177.5	177.3	136	252	185	237	6
Os03g0246800	937	1029	866	1133	1295	1159	882	850	803	654.4	631.5	516.5	585.62	551.67	640.7	1039	862.6	805	711.6	842.6	691.5	984.1	1069	955.5	706.7	627	576.7	850	724	807	2
Os03g0859100	0	0	0	0	0	0	0	0	0	0	0	0	0	0	0	0	0	0	0	0	0	0	2.138	0	0	0	0	0	0	0	17
Os03g0101300	107	122	75.4	118.2	141.9	87.37	247	220	117	154.3	126.1	235.2	76.196	76.543	60.981	11.94	59.77	101.5	71.53	58.05	50.01	88.73	103.3	156.7	81.34	107.5	151.3	57.4	76.5	47.5	5
Os03g0391400	1.02	0	2.16	0	0.904	1.73	2.35	1.27	0	3.449	1.096	0.941	1.0885	1.8669	0.792	0	2.061	0	0	0.806	0	0.739	1.425	1.444	1.056	0	4.386	5.55	4.78	0.83	1
Os03g0751000	641	699	779	757.8	722.3	686.8	706	752	831	800.1	690.7	703.7	729.31	694.49	704.85	704.5	510.1	562.6	796.1	667.6	657.9	583.4	681.3	715.7	691.9	561.9	666.6	587	539	607	7
Os03g0164700	570	566	619	912.1	855.2	847.7	700	738	737	646.6	742.3	800.6	714.07	786.9	733.36	1230	732.7	683.8	1022	965.9	952.7	655.8	595.1	659.4	685.6	764.6	782.9	697	730	640	6
Os03g0816900	23.4	39.1	30.2	55.01	40.68	39.79	2.35	3.16	7.6	4.311	8.771	9.408	4.3541	7.4676	15.047	71.65	17.52	17.61	10.22	15.32	12.07	15.53	7.839	2.889	13.73	13.2	2.193	15.7	20.1	48.3	8
Os03g0336000	126	120	136	211	214.2	199	373	408	424	141.4	165.6	207.9	218.79	174.56	168.69	238.8	141.2	164.7	229.5	260.4	282.8	269.9	224.5	309.1	168	152.7	157.9	152	174	167	18
Os03g0245700	216	239	250	150.2	96.72	195.5	137	109	188	238	184.2	297.3	141.51	110.15	119.59	322.4	170	138.8	197.9	228.2	184.5	158.2	209.5	218.1	233.5	148	188.6	212	153	200	6
Os03g0132200	41.8	52.2	71.1	78.81	68.7	121.1	144	135	143	117.3	81.13	166.5	108.85	101.75	93.452	35.82	76.26	90.14	132.8	137.1	113	153.1	115.4	151.7	122.5	116.9	111.8	79.6	41.1	70	15
Os03g0224200	974	1075	1096	890.8	890.4	835.6	1271	1586	1489	802.7	752.1	813.8	878.44	781.3	943.23	1588	932.6	987.3	753.4	704.7	812.2	1360	1327	1377	904.2	953.2	960.5	921	866	948	18
Os03g0436300	95.8	97.8	123	136.3	164.5	128.9	99.4	96.8	87.8	75.87	87.71	81.85	121.91	82.144	140.18	83.59	108.2	119.1	76.17	87.08	90.53	110.2	116.2	101.1	129.9	117.9	67.98	87	102	181	8
Os03g0285900	653	629	603	1115	1004	1152	820	753	813	732.9	709.4	726.3	752.17	672.09	701.68	1099	670.9	741.8	1167	1259	1125	646.2	644.9	616.8	643.3	774.1	708.3	771	693	751	10
Os03g0762000	1006	1100	968	1124	1110	1011	1177	1187	1298	1019	1115	1109	949.19	1245.2	1018.5	1469	1106	993.6	1128	974	1145	1142	1202	1163	1136	1204	1250	989	956	1101	6
Os03g0750400	1.02	1.86	2.16	0	0	0	0	0	0	1.724	1.096	0	1.0885	0.9335	0	0	0	1.036	0	1.613	0	0	0	0.722	1.056	0	0	0.93	0	0	14
Os03g0679300	125	124	114	144.5	144.6	134.9	114	145	126	103.5	82.23	89.38	90.347	81.21	100.58	167.2	98.93	114	127.3	130.6	91.4	127.9	124	147.3	119.4	99	103.1	114	105	96.6	6
Os03g0179700	0	0	0	0	0	0	0	0	0	0	0	0	0	0	0	0	0	2.072	0	0	0	0.739	0.713	0	0	0	0	0	0.96	0	1
Os03g0269100	1695	1594	1690	2708	2959	2445	3073	3455	3225	2105	2131	1943	2457.9	2138.5	2273.7	3152	1652	1665	3302	3171	2847	1860	1880	1920	1689	1422	1384	1701	1616	1794	12
Os03g0820300	34.7	55	15.1	4.926	0	19.03	2.35	5.69	1.38	11.21	7.675	7.527	6.5311	2.8004	7.1277	11.94	31.95	21.76	0.929	0.806	5.173	11.09	8.552	7.222	7.395	1.886	0	2.78	4.78	4.17	14
Os03g0719000	194	167	153	24.63	18.08	44.98	2.35	1.27	0	74.15	106.4	99.73	95.79	107.35	111.67	298.5	277.2	154.4	39.94	40.31	44.84	4.436	4.276	9.389	158.5	206.5	155.7	228	165	202	13
Os03g0388000	35.7	36.3	51.7	88.67	87.69	86.5	58.7	58.2	47	29.31	44.95	55.51	55.515	47.606	32.471	179.1	49.47	40.41	105	87.88	101.7	56.93	47.03	49.11	33.8	51.86	46.05	40.7	38.3	45.8	6
Os03g0194450	0	0	0	0	0	0	0.78	0	0	0	0	0	0	0	0	0	0	0	0	0	0	0	0	0	0	0	0	0	0	0	5
Os03g0360200	0	1.86	0	0.821	1.808	0	0	0	0	0	0	0.941	0	0	0	0	1.031	0	0	0	0	0	0	0	0	0	0	0.93	0	0	2
Os03g0205700	0	0	1.08	0	0	0	0	0	0	0	3.289	4.704	0	0	0	0	0	1.036	0	0	0	0	0.713	0	0	0	2.193	0	0	1.67	20
Os03g0727900	344	322	307	345.6	334.5	276.8	259	278	311	359.5	391.4	422.4	436.5	402.32	376.18	417.9	325.6	326.4	277.8	319.3	320.7	272.8	280.8	281.7	350.7	405.4	405.7	363	328	366	3
Os03g0760700	257	182	197	490.9	576.7	438.6	215	238	211	142.3	115.1	197.6	179.61	266.97	146.51	417.9	215.4	217.6	587.1	428.1	676.8	169.3	177.4	170.4	91.9	143.3	162.3	121	136	132	10
Os03g0196000	24.5	14	21.6	27.09	27.12	14.71	5.48	8.86	5.53	14.66	12.06	10.35	20.682	16.802	15.839	0	15.46	16.58	21.37	12.9	18.97	9.612	17.82	17.33	28.52	15.09	10.96	24.1	19.1	16.7	2

Os03g0572083	0	0	0	0	0	0	0	0	0	1.724	0	0	0	0	0	0	0	0	0	0	0	0.739	0	0	0	0	0	0	0	0	7
Os03g0233800	53	53.1	63.6	75.53	74.13	72.66	142	94.9	146	52.59	64.69	68.68	50.072	63.475	56.23	143.3	52.56	59.05	77.1	107.2	106.9	109.4	71.26	111.9	36.97	47.14	54.82	43.5	66.9	31.7	18
Os03g0169800	571	526	567	637.1	613.8	604.7	871	765	798	701	797.1	1025	914.36	769.16	686.63	1063	646.1	644.4	870.4	811.1	926.9	482.1	463.9	569.1	871.5	825	774.1	622	588	606	20
Os03g0147900	73.4	44.7	48.5	57.47	61.47	51.9	35.2	44.9	38.7	35.35	54.82	38.57	56.603	46.673	62.565	35.82	47.4	54.91	45.52	76.6	59.49	47.32	35.63	46.95	49.65	53.74	24.12	68.5	64.1	58.3	8
Os03g0783800	28.5	29.8	25.9	20.52	20.79	12.98	29.7	17.1	12.4	50.87	47.14	49.86	34.833	41.072	25.343	59.7	47.4	32.12	38.09	14.51	32.76	31.05	36.34	58.5	35.92	34.89	46.05	40.7	26.8	25.8	13
Os03g0708100	434	411	445	695.4	697.9	735.3	651	669	712	479.4	485.7	523.1	421.26	420.05	430.04	406	368.9	414.4	715.3	753.1	757.9	528.7	607.9	615.4	492.3	473.3	375	396	381	387	12
Os03g0718200	109	112	131	144.5	202.5	118.5	224	214	220	140.5	177.6	173.1	167.63	128.82	156.02	167.2	96.87	87.03	161.6	164.5	200	143.4	159.6	153.8	114.1	124.5	87.72	102	102	103	18
Os03g0695425	286	250	266	358.8	397.7	349.5	191	141	238	256.9	268.6	279.4	257.98	201.63	220.96	513.5	332.9	294.2	362.3	367.7	446.6	130.9	148.2	192.1	294.7	235.7	228.1	251	240	240	16
Os03g0798600	1161	941	1057	1332	1334	1321	1587	1347	1743	1598	1164	2455	1387.9	1430	897.3	1719	988.3	1156	1789	1430	1704	1098	1150	1185	1175	1505	1517	807	1068	801	15
Os03g0760300	114	69.9	45.3	41.05	28.93	44.98	21.9	19	40.1	41.38	48.24	33.87	25.036	32.671	36.43	23.88	50.5	45.59	26.94	12.09	22.42	107.2	62	75.84	29.58	51.86	83.33	51.8	66	60.8	19
Os03g0805400	448	367	485	112.5	90.4	119.4	192	148	209	316.4	222.6	248.4	236.21	232.43	293.03	334.3	273.1	264.2	117	132.2	77.6	300.9	285.8	268.7	218.7	198	280.7	206	200	196	14
Os03g0711700	241	168	197	253.7	248.6	258.6	219	199	260	213.8	221.5	202.3	216.62	224.96	194.82	394.1	213.3	195.8	231.3	236.2	266.4	253.6	224.5	215.2	178.5	203.7	214.9	200	201	209	6
Os03g0654600	440	623	523	848.9	975.4	739.6	729	923	773	525.1	687.4	546.6	540.99	489.13	452.21	680.6	333.9	464.1	709.7	836.1	727.7	600.4	601.5	642.1	458.5	337.5	368.4	333	339	441	12
Os03g0271900	0	0	0	0.821	0	0	0	0	0	0	0	0	0	0	0	0	0	0	0	0	0	0	0	0	0	0	0	0	0	0	2
Os03g0844850	12.2	22.4	6.47	2.463	8.136	12.11	17.2	22.8	17.3	7.76	7.675	9.408	13.062	13.068	8.7116	11.94	12.37	10.36	7.432	6.45	2.587	10.35	10.69	9.389	6.338	8.486	2.193	4.63	20.1	11.7	5
Os03g0833200	79.5	87.6	88.4	126.4	136.5	127.2	150	120	133	109.5	117.3	101.6	117.56	114.81	114.04	167.2	117.5	126.4	150.5	148.4	152.6	139.7	127.6	137.9	99.3	116.9	142.5	120	152	117	6
Os03g0370900	454	417	468	592.7	547.8	512.1	823	856	764	508.7	474.7	499.6	446.29	446.19	460.13	656.8	443.1	454.8	510	487	510.4	733.5	728.3	668.8	422.5	441.2	489	509	493	497	18
Os03g0807800	1706	1230	1353	1548	1569	1630	2065	1605	2132	1871	1525	2882	1709	1782	1259.2	1970	1355	1561	2035	1661	2168	1423	1370	1565	1459	1906	2110	1037	1217	919	15
Os03g0576500	53	31.7	28	29.56	27.12	28.55	33.7	25.3	29.7	38.8	31.8	46.1	52.249	48.539	38.806	59.7	36.07	48.69	45.52	42.73	42.25	31.05	22.09	41.17	46.48	45.26	70.17	57.4	51.6	50.8	13
Os03g0298700	81.6	117	70	108.4	122.9	151.4	77.5	47.4	90.5	143.1	106.4	114.8	72.931	82.144	68.901	83.59	81.41	108.8	50.16	66.12	83.78	70.24	62	83.78	129.9	124.5	103.1	92.5	86.1	103	7
Os03g0698400	140	134	127	136.3	134.7	144.5	144	161	162	149.2	167.7	137.4	160.01	176.42	166.31	417.9	168	156.4	233.2	195.9	226.8	136	151.1	126.4	166.9	140.5	197.4	144	181	143	6
Os03g0161600	1.02	0	0	1.642	0	0.865	7.05	3.16	4.84	0	0	0	0	0	0.792	0	0	0	0	0	0	0	0.713	2.889	0	0	2.193	0	0	2.5	18
Os03g0609000	4.08	5.59	3.23	1.642	4.52	3.46	1.57	0	2.76	5.173	4.386	12.23	1.0885	1.8669	2.3759	0	0	3.108	0.929	1.613	6.898	1.479	0	7.222	1.056	1.886	2.193	1.85	0.96	0	20
Os03g0356638	552	657	602	791.4	823.5	729.2	771	851	694	407	446.2	393.3	583.45	519.93	533.79	919.5	500.8	576	543.4	645	701.8	768.2	699.1	681.8	426.8	394.1	364	490	520	563	12
Os03g0711650	1.02	0.93	0	2.463	0	1.73	1.57	3.16	5.53	0.862	0	0	0	0.9335	0	0	0	0	1.858	0	0	2.958	1.425	3.611	0	0.943	2.193	0	2.87	0.83	18
Os03g0828800	141	163	225	252.9	329	186	287	431	290	214.7	212.7	128.9	289.55	209.09	268.48	406	169	219.6	169.1	293.5	178.5	362.3	389.8	256.4	193.3	157.5	131.6	244	223	271	17
Os03g0302900	0	1.86	3.23	6.568	15.37	10.38	4.7	1.9	6.91	0	0	6.586	0	5.6007	4.7518	0	6.183	4.144	7.432	9.675	23.28	3.697	2.138	3.611	2.113	1.886	0	6.48	0	0	10
Os03g0111400	91.8	36.3	55	139.6	87.69	58.82	38.4	161	138	29.31	30.7	45.16	26.124	18.669	49.894	0	14.43	9.324	34.37	46.76	7.76	17.01	45.61	28.17	45.42	22.63	4.386	25	41.1	42.5	2
Os03g0297850	0	0	0	0	0	0	0	0	0	0	0	0	0	0	0	0	0	0	0	0	0	0	0.713	0	0	0	0	0	0	1.67	1
Os03g0323350	47.9	33.5	28	76.35	62.37	65.74	215	209	163	50.87	58.11	31.05	44.629	51.34	57.022	47.76	38.13	42.48	69.67	87.88	68.98	160.4	173.2	148.1	71.83	54.68	39.47	62.9	56.4	60.8	18
Os03g0673500	0	0	0	0	0	0	0	0	0.69	0	0	0	0	0	0	0	0	0	0	0	0	0	0	0	0	0	0	0	0.96	0	1
Os03g0313600	1076	981	1081	1589	1577	1578	805	959	917	1191	1169	1248	1353	1567.3	1221.2	1875	1256	1343	1827	1671	2044	837	881.5	824.1	1066	1163	1057	1353	1574	1191	16
Os03g0195300	45.9	52.2	58.2	39.41	17.18	38.06	49.3	46.8	41.5	31.9	57.01	33.87	44.629	64.408	47.518	23.88	36.07	38.33	26.94	34.67	34.49	46.58	38.48	31.06	41.2	38.66	19.74	76.8	45.9	67.5	1
Os03g0230000	30.6	25.1	15.1	2.463	3.616	6.92	0	0.63	1.38	6.897	10.96	18.82	6.5311	10.268	5.5438	11.94	24.73	24.86	1.858	0.806	7.76	3.697	4.988	0.722	7.395	2.829	21.93	8.33	7.65	9.16	14
Os03g0810700	28.5	29.8	25.9	89.49	107.6	51.9	29	81	31.8	33.63	32.89	26.34	29.39	28.004	32.471	95.53	25.76	32.12	49.23	86.27	61.22	45.1	83.38	40.45	24.3	12.26	17.54	46.3	32.5	30	10
Os03g0262400	722	620	747	634.6	700.6	684.3	685	639	659	637.2	536.1	639.8	719.51	714.09	643.87	1027	547.2	612.3	744.1	663.6	678.6	574.5	504.5	502	558.8	482.7	576.7	562	510	577	6
Os03g0428800	6609	6209	6630	6648	6584	6608	4668	4394	5177	8868	9319	10113	11396	10267	10201	12084	8737	7700	9568	9266	9840	3248	3502	3818	9795	9895	8787	7909	8106	8362	3
Os03g0127000	212	221	207	150.2	129.3	156.6	189	161	169	187.1	199.5	182.5	177.43	196.02	219.37	465.7	229.8	188.6	186.7	229.8	205.2	241.8	198.1	183.5	231.3	215	267.5	293	267	274	13
Os03g0141100	3.06	1.86	2.16	0	0	0	0	0.63	0	0.862	1.096	0	8.7082	7.4676	4.7518	0	0	0	0	0	0	0.739	1.425	0.722	0	0.943	0	0.93	0	0	4
Os03g0306200	577	643	697	573.9	549.6	590	680	617	666	636.3	671	652.9	822.92	750.5	803.05	823.9	743	690	622.4	761.9	683.7	632.2	598.6	592.2	757.4	766.5	671	686	695	741	13
Os03g0799200	0	0	0	0	0	0	0	0	0	0	0	0	0	0	0	0	0	0	0	0	0.862	0	0	0.722	0	0	0	0	0	0	9
Os03g0375200	498	423	539	18.88	32.54	33.74	53.2	125	75.3	598.4	661.1	588	810.95	589.01	730.98	1099	615.2	543.9	65.96	48.38	64.67	52.5	81.95	135.1	762.7	585.5	543.8	562	564	633	3
Os03g0718000	211	204	231	224.9	307.1	136.7	153	232	116	324.2	196.3	213.6	214.44	227.76	279.56	310.5	157.7	189.6	166.3	141.9	88.81	121.3	230.2	120.6	219.7	131.1	261	182	209	207	7
Os03g0832400	310	252	290	81.28	68.7	103.8	139	85.4	95.4	203.5	229.1	209.8	169.81	181.09	176.61	322.4	245.3	268.3	129.1	108.8	129.3	126.4	110.5	136.5	165.8	229.1	208.3	185	244	192	13
Os03g0296700	240	197	219	99.34	92.2	102.1	91.6	80.3	73.9	110.4	128.3	129.2	128.45	168.02	101.37	214.9	212.3	200	113.3	112.9	138.8	62.85	44.9	69.34	94.02	128.2	107.5	133	144	144	13
Os03g0248400	0	0.93	3.23	18.06	36.16	15.57	52.5	43.6	17.3	0.862	2.193	0.941	2.177	2.8004	0.792	11.94	1.031	4.144	22.29	13.71	26.73	15.53	14.97	7.945	2.113	2.829	0	3.7	1.91	1.67	12
Os03g0143600	488	443	449	481.9	471	466.3	580	686	556	473.3	548.2	485.5	611.75	624.48	629.61	823.9	507	566.7	524.9	649.1	513.9	505	505.3	449.2	530.3	510.1	453.9	565	593	572	6
Os03g0244950	269	257	291	138.7	129.3	122.8	265	251	264	591.5	531.8	532.5	473.51	354.71	494.19	525.4	275.2	362.6	264.8	189.5	196.6	176	213.1	226.8	372.9	392.2	333.3	267	314	316	3
Os03g0305600	15.3	23.3	37.7	17.24	10.85	26.82	34.4	18.3	33.9	37.94	23.02	29.17	17.416	19.602	28.511	59.7	39.16	38.33	28.8	21.77	34.49	23.66	29.93	43.33	22.18	30.17	28.51	43.5	44.9	26.7	6
Os03g0278000	786	795	762	686.3	701.5	917.8	1103	858	892	958.8	785	838.3	712.98	819.57	731.78	716.5	908.9	706.6	729.2	617.6	624.2	653.6	788.9	793.7	663.4	721.3	951.7	714	814	728	5
Os03g0161950	41.8	44.7	33.4	10.67	7.232	12.11	7.05	15.2	8.29	35.35	24.12	25.4	39.187	35.471	40.39	35.82	36.07	35.23	12.08	14.51	14.66	5.176	31.36	18.06	32.75	34.89	61.4	62	43	38.3	3
Os03g0736200	38.7	83.8	46.3	82.1	82.26	68.34	69.7	63.3	65	79.32	80.04	47.04	63.134	53.207	69.693	119.4	45.34	44.55	71.53	71.76	106.9	78.37	66.28	75.84	65.49	67.88	39.47	51.8	64.1	57.5	6
Os03g0143400	197	163	183	479.4	501.7	445.5	98.6	109	87.1	144	98.68	122.3	262.33	266.97	196.41	238.8	155.6	116	311.2	390.2	331.1	85.03	117.6	89.56	127.8	117.9	89.91	115	147	129	10
Os03g0314500	1.02	0	0	0	0	0	0	0	0	0	0	0	0	0	0	0	0	0	0	0	0	0	0	0	0	0	2.193	0	0	0	11
Os03g0685100	134	144	128	225.8	383.3	243.9	170	198	159	126.7	164.5	160.9	191.58	148.42	137.01	119.4	60.8	127.4	196.9	267.7	239.7	124.2	112.6	108.3	137.3	73.54	92.1	72.2	67.9	104	10
Os03g0654800	266	333	344	409.7	451.1	404	572	526	628	273.3	311.4	347.2	280.84	286.57	251.85	477.6	329.8	283.9	443.1	444.3	499.2	363	308.6	513.5	259.9	256.5	250	238	268	257	12
Os03g0137300	16.3	16.8	12.9	19.7	21.7	23.36	3.13	6.96	13.1	9.484	17.54	12.23	22.859	26.137	13.463	11.94	23.7	11.4	20.44	25.8	31.9	5.915	1.425	5.056	9.507	16.03	15.35	14.8	19.1	22.5	16
Os03g0788800	754	1047	911	894.9	976.3	877.2	813	935	892	937.2	1102	920.1	924.15	933.45	978.87	1337	760.5	877.5	712.5	834.5	701.8	948.6	935.7	937.5	1026	1043	811.4	851	917	1026	6
Os03g0328501	1.02	0.93	1.08	0	3.616	0.865	3.13	6.96	2.76	2.587	1.096	1.882	3.2656	0	0	0	0	0	0.929	0.806	1.724	4.436	5.701	3.611	0	0	0	0.93	0	1.67	18
Os03g0784600	253	398	267	295.6	325.4	287.2	177	235	192	118.1	145.8	65.86	148.04	144.68	251.05	513.5	339	290.1	90.11	126.6	120.7	569.3	726.2	459.3	385.6	355.5	234.6	577	396	630	8
Os03g0735300	670	586	606	1090	1019	1042	773	607	651	683.7	546	735.7	681.41	771.96	593.18	955.3	696.6	624.7	1124	1084	1066	470.2	556.6	582.9	563	732.6	732.4	567	578	539	10
Os03g0165600	1247	1264	1173	1171	1154	1087	988	1073	1011	1205	1259	1271	1366.1	1143.5	1302.8	2161	1124	1147	1022	1160	1087	1188	1227	1065	1310	1165	1114	1279	1246	1201	13
Os03g0424600	305	266	258	299.7	329.9	303.6	273	238	243	275.9	274.1	300.1	248.18	308.97	288.28	644.8	310.2	296.3	266.6	307.2	280.2	320.9	283.6	282.4	305.3	333.8	230.3	329	277	305	6
Os03g0850700	374	360	435	302.9	322.7	311.4	97.9	55	65.6	535.4	379.4	388.6	346.15	349.11	361.14	597	304	328.4	288.9	311.2	294.9	76.16	156.8	150.9	408.8	415.8	438.6	385	388	375	13
Os03g0851100	0	0	0	0	0	0	0	0	0	0	0	0.941	0	0	0	0	0	0	0	0	0	0	0	0	0	0	0	0	0	0	20
Os03g0375966	2.04	2.79	4.31	15.6	8.136	5.19	9.39	6.96	4.84	4.311	5.482	1.882	1.0885	1.8669	2.3759	0	6.183	1.036	11.15	7.257	5.173	8.133	17.1	7.945	11.62	0.943	4.386	10.2	2.87	9.16	17
Os03g0255400	878	976	989	1577	1583	1636	1926	2147	1953	1237	1329	1325	1313.8	989.46	1195.1	1290	917.2	997.7	1352	1524	1433	2075	2082	2087	1407	1190	1090	1095	975	1216	18
Os03g0825900	2.04	0	1.08	0.821	0.904	2.595	0.78	0	0	2.587	0	1.882	1.0885	0.9335	0	0	0	0	0	0	2.587	3.697	4.988	2.889	2.113	0	0	2.78	0.96	0.83	17
Os03g0336200	15.3	14.9	16.2	3.284	19.89	12.11	19.6	31	19.3	30.18	17.54	25.4	16.328	9.3345	14.255	11.94	18.55	17.61	4.645	8.869	12.07	12.57	13.54	10.11	28.52	18.86	15.35	18.5	7.65	13.3	5
Os03g0174900	838	837	917	688	801.8	722.3	895	763	838	794.1	789.4	1027	1082	1022.1	957.49	1194	726.5	697.3	778.5	757.9	685.5	1003	865.1	857.3	717.3	701.5	866.2	773	717	812	6
Os03g0846400	1746	1759	1808	2181	2035	2034	1777	1906	2040	2025	1944	2074	1855.9	1991.1	1912.6	3367	1556	1683	1812	1628	2015	1921	1893	1717	1817	1899	1818	1499	1674	1544	6
Os03g0270000	27.5	40.1	24.8	25.45	20.79	13.84	47	44.3	62.2	22.42	27.41	12.23	20.682	16.802	28.511	47.76	20.61	12.43	11.15	11.29	12.07	111.6	176	146.6	17.96	12.26	6.579	18.5	14.3	20	17
Os03g0793000	70.3	55	58.2	45.15	34.35	39.79	86.1	101	62.9	44.83	48.24	50.8	34.833	53.207	42.766	35.82	65.95	64.23	56.67	53.21	44.84	142	74.11	110.5	59.16	55.63	63.59	51.8	42.1	40	9
Os03g0360500	613	594	600	592.7	545.1	622.8	968	787	865	776.8	648	866.5	642.23	650.62	651.79	597	597.7	540.8	717.2	611.2	687.2	744.6	726.2	748.2	607.4	780.7	789.5	642	581	557	5
Os03g0302200	3457	3954	2834	1181	1046	1078	824	1032	1036	728.5	680.9	575.8	782.65	687.95	866.41	1397	1560	1680	811.9	1055	848.4	1389	1484	1213	817.6	684.5	769.7	880	912	986	14
Os03g0112101	176	231	231	206.1	208.8	186.9	51.7	78.4	60.1	67.25	74.55	49.86	119.74	123.22	125.13	203	136	187.5	48.31	84.66	74.15	169.3	190.3	111.9	129.9	120.7	78.95	164	137	217	8
Os03g0635000	13.3	41.9	21.6	52.54	68.7	64.01	70.5	72.7	92.6	44.83	55.92	54.57	44.629	52.273	30.887	59.7	27.82	38.33	69.67	61.28	90.53	45.1	49.88	59.95	25.35	36.77	15.35	30.5	40.2	35.8	12
Os03g0432277	3.06	3.73	1.08	4.105	2.712	1.73	9.39	5.69	6.91	0.862	6.578	3.763	3.2656	0.9335	2.3759	11.94	4.122	5.18	6.503	14.51	6.898	2.218	5.701	8.667	2.113	0.943	2.193	0.93	0.96	0.83	6
Os03g0644850	3.06	0.93	0	2.463	2.712	5.19	1.57	3.8	8.98	0.862	0	0	0	2.8004	1.5839	0	3.092	1.036	1.858	0.806	4.311	5.176	0	6.5	1.056	1.886	2.193	0.93	0	4.17	9
Os03g0708700	108	97.8	86.2	44.33	34.35	47.58	31.3	34.8	30.4	72.42	71.27	107.3	66.4	74.676	72.069	250.8	148.4	107.7	43.66	33.06	43.97	42.88	45.61	36.83	89.79	130.1	155.7	149	106	85.8	13
Os03g0367800	111	108	88.4	128.9	141	128.9	201	152	168	107.8	110.7	129.8	100.14	132.55	110.08	143.3	84.5	104.6	170	158.8	175	90.94	95.49	124.2	112	88.63	111.8	74	90.9	73.3	15
Os03g0600400	80.5	82.9	70	121.5	121.1	151.4	179	132	155	122.4	114	123.2	96.878	113.88	110.08	95.53	80.38	80.81	176.5	182.2	156.9	93.9	112.6	135.1	89.79	117.9	94.3	84.2	80.3	67.5	15
Os03g0157300	107	88.5	100	73.89	92.2	127.2	144	115	137	57.77	49.34	51.75	46.806	54.14	39.598	131.4	90.69	77.7	77.1	77.4	100.9	90.21	111.2	150.9	43.31	58.46	61.4	60.1	64.1	41.7	18
Os03g0295300	37.7	25.1	31.2	139.6	149.2	128.9	79.9	58.2	55.3	56.04	25.22	41.4	44.629	40.138	40.39	0	27.82	35.23	120.8	162.9	114.7	62.11	59.15	57.06	30.63	38.66	28.51	34.2	33.5	23.3	10
Os03g0376900	391	410	433	362.9	343.5	363.3	490	536	477	499.2	496.7	535.3	507.25	479.79	491.81	561.2	455.5	381.3	381.8	345.1	376.8	473.2	471.1	463.7	559.9	598.7	546	419	492	470	5
Os03g0187600	941	675	883	363.7	339	352.9	568	633	501	564.7	415.5	440.3	519.22	539.53	565.46	1134	802.8	808.1	508.1	572.5	500.1	346.8	464.4	447.1	551.4	363	357.4	412	494	474	13
Os03g0247000	1606	1610	1710	2345	2522	2188	2464	2720	2736	1706	2108	1902	2241.3	1683.9	1774	2281	1127	1415	2401	2738	2270	1939	1945	2459	1517	1384	1239	1169	1347	1372	12
Os03g0234200	509	336	348	512.3	528.8	495.7	1028	743	828	631.1	595.3	956.8	527.93	614.21	384.9	561.2	391.6	396.8	840.7	733.7	835.5	550.8	481.7	577.8	477.5	589.3	725.9	319	405	262	15
Os03g0414100	0	0	4.31	0	0	0	0	1.9	0	0	0	0	0	2.8004	0	0	0	0	0	0	1.724	0	0	0	1.056	0	0	0.93	0	0	4
Os03g0308100	756	825	860	2088	2278	1990	2145	2216	2106	862.2	927.5	842	1122.3	860.64	734.15	1170	514.2	676.5	1839	1944	1830	1599	1529	1749	678.2	519.5	480.2	480	584	622	12
Os03g0859800	650	673	614	706	770.2	638.4	965	968	928	584.6	607.4	566.4	638.96	589.01	702.47	597	541	523.2	603.8	691	590.6	1008	907.2	743.2	696.1	532.7	642.5	612	654	639	18
Os03g0332201	0	0	0	0	0	0.865	0	0	0	0	0	0	0	0	0	0	0	0	0	0	0	0	0	0	0	1.886	0	0	0	0	1
Os03g0238000	90.7	89.4	76.5	84.56	66.89	69.2	65.8	83.5	53.2	97.43	77.84	74.32	77.285	78.41	93.452	47.76	76.26	71.49	95.68	74.18	75.01	112.4	83.38	91.73	83.45	85.8	57.02	66.6	74.6	66.6	9
Os03g0644600	27.5	10.2	25.9	2.463	0	3.46	0	0	0	25	17.54	8.467	13.062	28.937	24.551	83.59	40.19	31.08	9.29	12.9	15.52	0	0	0	41.2	33	39.47	43.5	35.4	33.3	13
Os03g0270200	2005	1923	1911	2245	2241	2138	1902	1924	1921	1914	1861	2038	1715.5	1967.7	1972.8	3761	2011	2180	1872	1999	1893	2343	2152	2014	1876	2212	2072	2004	2326	2086	6
Os03g0245901	0	0	0	0	0	0	0	0	0	0	0	0	0	0	0	0	1.031	0	0	0	0.862	0	0	2.167	0	0	0	2.78	0	0	1
Os03g0282800	739	623	790	532	504.4	526.8	586	564	609	681.1	806.9	696.2	807.68	767.3	736.53	1170	858.4	763.6	603.8	615.2	609.6	585.6	543	595.1	780.7	743	750	761	760	811	13
Os03g0375900	13.3	12.1	11.9	55.01	20.79	32.01	61.8	55	51.8	7.76	9.868	9.408	9.7967	11.201	6.3357	0	8.244	5.18	52.95	45.96	37.08	36.23	38.48	45.5	10.56	9.428	13.16	13.9	12.4	9.16	12
Os03g0554900	1347	1437	1392	1394	1484	1370	1208	1380	1270	1201	1352	1154	1660	1472.1	1412.1	2114	1389	1412	1142	1192	1259	1391	1325	1260	1444	1359	1294	1540	1440	1569	13
Os03g0245200	0	0	0	0	0	0	0	0	0	0	0	0	0	1.8669	0	0	0	0	0	0	0	0	0	0	0	0	0	0	0	0	4
Os03g0360600	283	288	336	274.2	277.5	259.5	651	581	571	810.5	860.7	1184	620.46	617.01	598.73	907.5	512.2	577.1	624.3	519.2	695.8	220.3	228.8	347.4	752.1	800.5	668.8	459	453	426	20
Os03g0353900	2.04	1.86	7.54	12.31	23.5	10.38	7.05	10.1	18	3.449	8.771	1.882	2.177	1.8669	4.7518	0	6.183	2.072	3.716	4.031	0.862	15.53	8.552	7.945	1.056	3.771	0	4.63	2.87	0.83	2
Os03g0818100	218	268	238	207.7	204.3	238.8	157	137	169	264.7	228.1	216.4	248.18	258.57	242.34	417.9	279.3	242.4	211.8	171.7	220.7	143.4	132.6	132.2	198.6	205.5	219.3	250	266	207	13
Os03g0652000	0	0	0	0	0	0	0	0	0	0	0	0.941	0	0	0	0	0	0	0	0	0	0	0	0	0	0	0	0.93	0.96	0	1
Os03g0777600	40.8	54	35.6	71.42	76.84	86.5	41.5	58.2	35.9	20.69	28.51	26.34	30.479	26.137	33.263	47.76	44.31	60.09	39.02	43.54	30.18	110.9	86.94	67.17	34.86	32.06	21.93	50.9	43	50.8	17
Os03g0853200	7.14	10.2	9.7	0.821	4.52	6.055	1.57	3.16	12.4	25	15.35	8.467	5.4426	17.736	17.423	59.7	22.67	18.65	1.858	8.063	0.862	1.479	4.988	0.722	9.507	7.543	2.193	7.4	8.61	3.33	13
Os03g0100200	2710	2113	3441	537.7	562.3	517.3	671	662	592	3892	4179	5531	5847.5	4151.1	4420.8	1469	1981	1958	3761	561.2	623.4	315.7	307.9	621.9	2383	2221	2296	1639	2042	2263	3
Os03g0669000	658	618	622	784	756.6	753.5	688	650	666	661.3	634.8	827.9	893.67	835.44	753.95	1087	649.2	645.5	842.6	794.2	813.1	639.6	600	518.6	683.5	753.3	745.6	579	690	632	6
Os03g0347800	110	99.7	112	163.4	176.3	146.2	117	136	113	109.5	117.3	120.4	129.53	121.35	118.79	203	105.1	117.1	171.9	222.5	178.5	131.6	122.6	122.8	108.8	119.7	171	105	120	130	10
Os03g0122200	1030	1162	1144	2079	2267	1924	3128	2935	2826	947.5	1125	1454	1013.4	799.03	593.97	1624	691.5	868.2	2227	2404	2471	1793	1665	2640	680.3	663.8	644.7	447	658	578	12
Os03g0695500	2682	2451	2691	4163	4141	4126	2731	2923	2427	3352	3267	3694	4312.7	3895.3	3674.7	5075	3427	3068	5551	5249	6179	1443	1814	1843	3864	3440	3228	3031	2860	3088	16
Os03g0283400	38.7	42.8	43.1	62.39	48.81	87.37	15.7	21.5	20.7	18.97	35.08	37.63	25.036	32.671	23.759	47.76	50.5	40.41	57.6	72.57	53.46	39.19	43.47	32.5	29.58	37.71	32.89	37	44	44.2	10
Os03g0819700	211	238	183	121.5	103.1	118.5	78.3	75.3	63.6	113.8	125	121.4	78.373	120.42	109.29	119.4	147.4	152.3	90.11	101.6	98.29	116.1	114	127.1	150	122.6	153.5	149	136	130	14
Os03g0202001	404	269	366	63.22	49.72	96.89	57.2	46.8	60.1	346.6	331.1	314.2	397.31	436.86	390.44	728.4	358.6	297.3	98.47	104.8	100	45.1	58.44	67.17	326.4	398.8	453.9	349	416	351	13
Os03g0272400	294	397	290	224.9	211.5	155.7	398	388	339	357.8	398	272.8	309.14	350.04	377.77	561.2	288.5	267.3	203.4	187.1	191.4	247	258.7	250.6	307.4	350.7	241.2	246	249	307	13
Os03g0288101	242	256	276	138.7	191.6	173	72	85.4	89.8	150.9	164.5	142.1	223.15	210.03	224.13	382.1	278.2	307.7	103.1	161.3	107.8	160.4	136.8	78.72	237.7	239.5	179.8	313	311	338	13
Os03g0402800	5758	4489	4304	2674	1386	3174	1457	2441	3032	2299	1593	1239	2084.5	1859.4	2120.1	3451	2571	2860	3018	2693	3259	3677	3944	2988	1585	1708	2577	1756	1969	2025	14

Os03g0606200	2202	2011	2057	1829	1872	1982	2726	2416	2760	2484	1994	2888	1799.3	2017.2	1658.4	2997	2028	1744	2313	2114	2337	1826	1975	2423	1906	2104	2274	1551	1716	1570	15
Os03g0168100	0	0	0	1.642	0.904	0.865	0	0	0	0	0	0	0	0	0	0	0	0	0.929	0	0	1.479	0.713	0	0	0	0	0	0	0	2
Os03g0744700	9620	####	####	2098	2074	2171	1259	1658	1138	10064	11822	10566	14994	14536	15854	19762	13575	12232	3167	3331	2844	771.2	1018	1218	13006	12759	11243	####	####	####	3
Os03g0682900	0	0	0	0.821	0	1.73	0.78	0	0.69	2.587	0	0	0	0	0	0	0	0	0.929	0	0	0	5.701	2.167	0	1.886	4.386	0	0	0.83	11
Os03g0328300	4.08	1.86	9.7	5.747	9.944	6.055	5.48	3.16	3.46	7.76	7.675	2.822	10.885	8.4011	5.5438	0	4.122	7.252	4.645	4.838	8.622	5.915	3.563	10.83	7.395	5.657	0	2.78	15.3	5.83	4
Os03g0286800	213	168	194	172.4	215.1	195.5	161	149	155	170.7	150.2	169.3	174.16	204.43	156.81	203	232.9	190.6	186.7	186.3	206.9	96.12	137.5	171.2	148.9	170.7	173.2	197	149	167	16
Os03g0241800	38.7	59.6	46.3	35.3	57.85	52.77	14.9	25.3	23.5	24.14	31.8	23.52	70.754	42.939	48.31	47.76	39.16	41.44	35.3	31.44	30.18	34.01	23.52	23.83	55.99	37.71	15.35	57.4	40.2	45	8
Os03g0746000	2.04	2.79	6.47	2.463	4.52	6.055	3.91	2.53	6.22	0.862	1.096	1.882	2.177	1.8669	3.9598	0	5.153	0	3.716	8.869	3.449	7.394	4.988	2.889	5.282	2.829	6.579	3.7	0.96	2.5	17
Os03g0242200	276	198	218	301.3	289.3	371.1	142	177	126	199.2	137	235.2	207.91	225.9	206.7	370.2	295.8	262.1	319.6	326.5	270.7	125	158.9	164.7	222.9	242.3	241.2	246	210	239	16
Os03g0249500	151	124	148	142.8	117.5	147.1	101	105	85	137.9	112.9	131.7	97.967	144.68	122.75	131.4	134	151.3	137.5	103.2	131.9	91.68	103.3	105.4	91.9	164.1	164.5	129	129	124	11
Os03g0110300	1228	1140	1213	834.9	716.8	876.3	1475	1283	1399	1606	1295	1680	1024.3	1181.7	1151.5	1922	1168	1086	1150	1043	1140	1146	1284	1436	1588	1364	1607	1020	1053	1006	5
Os03g0656000	1.02	0	0	0	0	0	0.78	0	0	0	0	0	0	0	0	0	0	1.036	0	0	0	0	0.713	0	2.113	0.943	0	0	0	0	19
Os03g0848300	259	328	265	317.7	371.5	320.1	626	548	609	337.1	369.5	363.2	326.56	307.11	311.24	417.9	283.4	272.5	371.6	323.3	338.9	509.4	449.7	446.3	370.8	277.2	318	336	255	300	18
Os03g0589500	0	0	0	0	0	0.865	0	0	0	0	0	0	0	0	0	0	0	0	0	0	0	0	0	0	0	0	0	0	0	0	2
Os03g0722100	615	696	658	632.2	612.9	613.3	752	843	856	663.9	639.2	573	704.27	676.75	753.95	1051	684.3	667.2	674.4	608.7	607.9	747.5	736.2	794.5	722.6	688.3	646.9	659	711	705	6
Os03g0243200	0	3.73	0	0	1.808	0.865	0	0	0.69	0	2.193	0	1.0885	0	0	0	1.031	1.036	0	2.419	0.862	0	0	0.722	1.056	0	2.193	1.85	0.96	0	11
Os03g0115500	768	708	732	563.2	654.5	627.2	763	963	715	618.2	689.6	669.9	803.33	807.44	761.87	1648	1016	851.6	740.4	811.1	703.6	729	833.1	734.5	822.9	823.1	758.7	970	840	911	6
Os03g0730500	1.02	0	0	0	0	0	0	0	0	1.724	0	3.763	1.0885	0	0	0	0	0	0	1.613	0	0	0.713	0.722	2.113	0.943	0	0	0	0	20
Os03g0288900	0	2.79	0	1.642	0.904	0	0	0.63	0.69	0.862	1.096	1.882	0	0	1.5839	0	1.031	1.036	0	0.806	0	0.739	0	0.722	0	5.657	2.193	0	1.91	0	20
Os03g0243100	551	397	483	333.3	378.8	330.4	240	422	399	410.4	361.8	939.9	521.4	485.39	492.6	656.8	494.7	490	346.5	280.6	306.1	350.5	387.7	325	458.5	957.9	631.6	436	513	447	20
Os03g0699600	710	623	700	477.8	446.6	435.1	950	1007	937	783.7	842	846.7	845.78	726.23	863.24	1134	919.2	710.7	645.6	741.8	626.8	624	712.6	769.9	876.8	922.1	859.6	806	858	876	5
Os03g0860600	111	179	163	572.2	643.6	452.4	597	658	678	186.2	172.1	271.9	180.69	123.22	144.14	83.59	114.4	129.5	543.4	462	477.7	363.8	344.2	540.2	114.1	109.4	103.1	99	95.6	127	12
Os03g0325900	1.02	0	0	0	0.904	0	0	0	0	0.862	0	0	0	0	0	0	2.061	0	0	0	0	0	0.713	0	0	0	0	0	1.91	0	1
Os03g0180800	113	148	110	14.78	0	7.785	6.26	7.59	2.07	0	0	0	26.124	0	1.5839	11.94	22.67	35.23	0	0.806	2.587	5.915	14.25	9.389	2.113	2.829	8.772	0.93	2.87	1.67	14
Os03g0811900	3628	3530	3546	2345	2339	2525	4357	3923	4041	3653	3464	4209	3415.8	3460.3	3288.2	4012	3537	3139	2881	2433	2898	3231	3114	3637	3025	3219	3454	2945	2963	3276	5
Os03g0823100	1040	1012	1037	979.4	945.6	1020	1156	1313	1360	1150	1185	1344	1117.9	1122	1132.5	1194	939.8	1155	872.3	917.5	1017	1179	1125	1169	1071	1210	1305	869	934	995	5
Os03g0734700	271	295	301	264.4	250.4	286.3	258	217	262	276.8	273	322.7	348.33	348.18	399.15	668.7	327.7	319.1	307.5	304	329.4	238.8	245.9	244.8	305.3	335.7	370.6	314	332	337	13
Os03g0626400	6.12	2.79	6.47	0.821	0	0.865	4.7	0	5.53	4.311	4.386	4.704	3.2656	0	5.5438	0	8.244	7.252	1.858	1.613	5.173	5.915	2.851	2.889	0	7.543	8.772	6.48	1.91	0.83	11
Os03g0766800	2674	3404	1904	8735	9688	7663	1486	918	1260	564.7	793.8	349	367.92	372.45	367.47	1911	2620	1599	8268	8206	12457	1302	696.2	2882	292.6	439.4	519.7	332	623	422	10
Os03g0730100	6.12	23.3	11.9	22.99	16.27	9.516	21.1	15.2	19.3	2.587	14.25	17.88	14.151	6.5342	13.463	23.88	17.52	14.5	4.645	8.063	2.587	28.84	23.52	16.61	9.507	13.2	4.386	14.8	12.4	13.3	17
Os03g0106850	0	0	0	0	0	0	1.57	0	0	0.862	0	0	0	0	0	0	0	1.036	2.787	0	0	0	0	0	1.056	0	0	0	0	0	15
Os03g0686500	118	116	93.7	156.8	151	186	288	299	381	163	127.2	214.5	183.96	179.22	171.86	155.2	105.1	128.5	143.1	114.5	153.5	237.3	223.8	301.2	225	116.9	151.3	124	122	145	18
Os03g0412800	44.9	80.1	56	250.4	254.9	182.5	25.1	52.5	53.9	37.94	52.63	30.11	58.78	43.872	37.222	47.76	21.64	44.55	74.32	74.98	91.4	74.68	61.29	49.83	46.48	17.91	28.51	37	27.7	39.2	2
Os03g0170500	312	247	317	127.3	132	178.2	699	479	716	657.8	700.6	1088	674.88	603.01	521.11	1230	742	703.5	499.8	380.6	496.6	220.3	254.4	456.5	714.1	836.3	986.8	523	565	392	20
Os03g0377500	236	238	231	262.7	273	279.4	234	280	284	272.5	253.3	246.5	183.96	228.7	241.55	286.6	197.9	221.7	228.5	229	273.3	317.2	268	273	217.6	258.3	203.9	225	202	216	12
Os03g0224300	146	165	189	330	361.6	314.9	298	201	225	134.5	129.4	132.7	201.38	191.36	194.03	501.5	213.3	218.6	336.3	345.1	355.2	203.3	187.4	227.5	142.6	156.5	131.6	344	371	237	6
Os03g0293850	3475	3647	3873	4103	4675	4512	3547	3763	3311	2723	3302	2659	2683.2	2676.2	2897.8	5720	3176	3794	3076	3943	3658	5761	4080	3572	3116	2901	3149	3277	3055	3667	6
Os03g0285700	6038	4864	5238	4134	4011	4907	4795	4677	5135	5484	4651	7138	5359.9	6519.2	4681.3	7200	5575	5296	5352	4281	5591	4659	5098	5257	5821	5759	6802	5293	4707	4892	20
Os03g0666500	179	209	222	201.1	214.2	202.4	149	188	181	128.5	145.8	154.3	170.9	175.49	167.1	107.5	158.7	127.4	139.3	121.7	130.2	176	191	192.1	166.9	162.2	166.7	170	122	176	2
Os03g0752800	27.5	17.7	12.9	7.389	9.944	7.785	50.9	20.9	46.3	32.76	33.99	52.69	13.062	28.937	15.047	0	21.64	44.55	39.94	15.32	56.04	82.81	14.97	59.22	10.56	57.51	96.49	39.8	23.9	84.1	11
Os03g0200800	665	692	712	614.1	560.5	494.8	797	1000	851	801.8	791.6	810	664	702.89	754.74	871.7	667.8	670.3	544.4	483	522.5	815.5	819.5	736.7	806	739.2	780.7	662	624	682	5
Os03g0282300	1.02	0.93	0	1.642	0.904	2.595	0	0	0	2.587	0	0.941	0	2.8004	0	0	0	0	0	0	0	2.218	2.138	0	0	0	4.386	0	0.96	1.67	11
Os03g0859900	8.16	6.52	4.31	14.78	26.22	6.055	15.7	10.8	29	11.21	17.54	2.822	3.2656	3.7338	3.9598	11.94	5.153	4.144	0	7.257	4.311	10.35	9.264	9.389	2.113	4.714	6.579	9.25	8.61	2.5	18
Os03g0353600	58.1	80.1	46.3	87.02	93.11	50.17	17.2	56.3	33.9	22.42	51.53	20.7	42.452	30.804	49.894	131.4	41.22	41.44	22.29	50.8	27.59	75.42	81.95	37.56	45.42	29.23	15.35	60.1	48.8	77.5	8
Os03g0780200	54	26.1	45.3	27.91	37.97	16.44	12.5	9.49	5.53	56.04	49.34	29.17	29.39	50.406	41.974	59.7	25.76	41.44	5.574	5.644	7.76	5.915	8.552	7.945	25.35	37.71	83.33	32.4	42.1	50	3
Os03g0648100	306	286	324	145.3	139.2	205	228	190	204	432.8	371.7	426.2	235.12	267.9	316.79	453.8	339	269.4	174.6	195.9	172.4	164.1	223.8	226.8	451.1	416.7	432	340	336	284	3
Os03g0194900	179	249	155	475.3	365.2	260.4	619	638	1099	156.9	134.9	135.5	103.41	66.275	177.4	203	180.3	146.1	207.2	173.4	156.9	840.7	806	854.4	99.3	72.6	61.4	72.2	74.6	88.3	18
Os03g0167100	191	154	158	213.5	220.6	250	528	450	505	260.4	218.2	303.9	226.41	185.76	148.89	382.1	166.9	153.3	296.3	264.5	243.1	272.8	248	275.2	190.1	193.3	265.3	159	143	120	18
Os03g0843700	555	670	654	555.8	473.7	525.1	506	464	468	595.8	583.3	520.3	741.28	645.02	724.65	1660	681.2	650.6	495.1	523.3	568.2	544.2	497.4	522.9	725.7	641.1	695.2	656	718	765	13
Os03g0148800	218	183	272	137.9	137.4	130.6	135	139	119	206.9	234.6	244.6	215.53	195.09	230.46	358.2	229.8	167.8	112.4	150	153.5	133.1	113.3	119.2	226.1	231	228.1	181	155	205	13
Os03g0861900	47.9	29.8	35.6	80.46	91.3	70.93	22.7	46.2	37.3	41.38	36.18	29.17	41.364	33.604	26.135	35.82	48.43	37.3	54.81	46.76	47.42	54.71	69.84	58.5	46.48	28.29	39.47	44.4	54.5	60	2
Os03g0706900	1557	1671	1739	1742	1878	1707	550	591	589	1236	1319	1065	1555.5	1513.1	1482.6	1755	1651	1764	1548	1715	1762	711.3	756.1	692.6	1539	1412	1285	1656	1467	1784	16
Os03g0418766	0	0.93	2.16	3.284	0	0	0.78	1.27	0	0	0	0.941	1.0885	0	0.792	0	0	1.036	0	0.806	3.449	0	1.425	0	0	0	0	0	0	0.83	16
Os03g0298900	1.02	0	1.08	1.642	0	0	3.13	2.53	8.98	0.862	4.386	0	1.0885	0	0.792	0	0	1.036	0.929	0.806	2.587	0.739	0.713	0.722	1.056	0.943	0	0	0.96	0.83	18
Os03g0265400	997	1298	1238	2781	3042	2747	4620	4245	4385	1318	1524	2202	1316	1015.6	738.11	1337	708	991.5	3282	3451	3349	2450	2224	3690	959.2	826.9	771.9	474	755	642	12
Os03g0730000	1107	1043	1155	332.5	347.1	307.1	754	765	652	1030	1125	1183	1207.2	1249	1205.4	2102	1580	1409	356.7	358.8	394	506.5	599.3	666.6	1261	1260	1145	1070	1122	1255	13
Os03g0836500	519	530	532	577.1	659	579.6	618	657	652	406.1	463.8	392.3	486.57	456.46	559.13	955.3	594.6	605	577.8	506.3	608.7	822.9	759.7	767.7	605.3	550.6	497.8	521	509	536	6
Os03g0310400	422	443	494	777.5	847.9	696.4	1036	1135	1116	600.1	672.1	565.4	604.13	505	586.05	489.6	498.8	473.5	777.5	753.9	715.6	775.6	789.6	768.5	478.5	462.9	377.2	403	420	448	12
Os03g0185700	0	0.93	3.23	3.284	1.808	1.73	1.57	1.27	0.69	0	1.096	1.882	4.3541	3.7338	7.1277	11.94	1.031	4.144	0.929	0	1.724	0	0	0	1.056	1.886	4.386	2.78	0	5	13
Os03g0366350	255	267	294	212.6	200.7	228.4	103	126	149	387.1	418.8	303.9	588.89	502.2	511.61	907.5	350.4	307.7	189.5	329	222.5	119	125.4	117	424.7	330	184.2	478	498	520	13
Os03g0801000	0	1.86	1.08	2.463	1.808	2.595	0.78	1.9	0.69	1.724	1.096	1.882	2.177	0.9335	0	0	0	1.036	1.858	5.644	6.898	0.739	2.138	0	0	0	0	2.78	0.96	1.67	16
Os03g0182900	0	0.93	1.08	0	0	0	2.35	0	0	0.862	1.096	0	0	0	0	0	1.031	2.072	2.787	0	0	7.394	0	0.722	0	0	0	2.78	0	0	9
Os03g0854000	368	408	419	299.7	337.2	361.6	1007	1023	1397	393.2	468.2	397	476.77	525.53	529.03	585.1	365.8	306.7	421.7	397.5	366.4	783.7	866.6	964.9	435.2	424.3	458.3	575	720	689	18
Os03g0835150	396	453	344	409.7	414.9	383.2	597	519	446	456.1	453.9	882.5	325.47	497.53	327.87	226.9	406	553.2	331.6	282.2	313	473.9	490.3	534.5	335.9	511	682	400	382	257	20
Os03g0853600	24.5	27.9	16.2	41.05	37.97	32.87	70.5	47.4	52.5	18.11	8.771	31.99	21.77	28.937	29.303	59.7	19.58	23.83	36.23	49.99	53.46	76.9	54.16	50.56	19.01	25.46	21.93	21.3	16.3	29.2	12
Os03g0182800	20.4	20.5	19.4	45.97	43.39	26.82	121	186	156	31.04	25.22	13.17	27.213	19.602	36.43	11.94	10.31	12.43	28.8	33.06	10.35	240.3	272.9	140.1	24.3	13.2	13.16	26.8	23	29.2	17
Os03g0114000	14.3	9.31	4.31	0	0	0	0	2.53	1.38	6.897	4.386	4.704	4.3541	15.869	11.088	11.94	11.34	6.216	0.929	0	0	1.479	1.425	0.722	6.338	12.26	0	6.48	7.65	7.5	3
Os03g0347300	716	806	748	832.5	857	790.7	623	655	670	695.8	763.1	605.9	740.19	732.76	765.83	1290	709	788.4	650.3	720.8	779.4	758.6	747.6	699.1	687.7	748.6	723.7	813	829	867	6
Os03g0336300	820	901	786	781.6	830.7	760.4	582	564	679	556.1	677.6	569.2	726.04	726.23	740.49	1397	840.9	899.3	544.4	658.7	661.3	809.6	812.4	671.7	712	752.4	666.6	872	726	828	13
Os03g0787200	50	77.3	62.5	77.17	94.01	115.1	131	88.6	64.3	67.25	75.65	60.21	41.364	55.074	48.31	119.4	54.62	59.05	56.67	79.82	53.46	122.7	94.07	127.1	63.38	50.91	52.63	78.6	87	85	9
Os03g0418600	454	442	428	405.6	430.3	458.5	600	572	674	452.6	508.7	692.4	526.84	453.66	498.94	728.4	548.2	408.2	490.5	544.2	564.8	845.9	550.9	656.5	553.5	551.6	539.5	483	466	480	9
Os03g0646800	931	1003	1010	986	979	960.2	720	748	797	805.3	850.8	820.4	930.68	1003.5	1035.1	1314	981.1	973.9	868.6	823.2	873.4	1033	889.4	869.6	976.1	986.2	1048	980	895	941	13
Os03g0249200	1023	948	1048	518.9	582.2	599.5	748	739	749	1204	1340	1586	1741.6	1437.5	1355.1	2699	1390	1230	799.8	719.2	732	539	468.9	562.6	1424	1497	1316	1094	1188	1239	13
Os03g0675500	0	0	0	0	0.904	0	0	0	0	6.897	0	1.882	1.0885	0.9335	0	0	1.031	0	0	0.806	0	0	0	0	0	0.943	0	0	0	0	7
Os03g0645000	0	0.93	0	0	0	0	0.78	0	0	0	1.096	2.822	0	0	0	0	0	0	0	1.613	0	0	0.713	1.444	0	0	0	0	0	0	20
Os03g0163600	0	0	0	0	0	0	0	0	0	0	0	0	0	0	0	0	0	0	0	0	1.724	0	0	0	0	0	0	0	0	0	16
Os03g0122600	590	672	635	651	774.7	605.5	852	980	783	597.5	615.1	519.3	500.72	508.73	614.57	752.3	604.9	650.6	498.8	497.5	487.2	881.3	984.1	791.6	597.9	608.1	570.2	573	629	640	17
Os03g0798500	32.6	36.3	43.1	4.926	0.904	6.92	3.91	3.8	2.76	83.63	75.65	33.87	10.885	51.34	34.055	83.59	28.85	24.86	4.645	4.838	6.036	5.915	22.09	14.44	67.61	83.91	81.14	75.9	112	73.3	3
Os03g0714100	0	0	0	0	0.904	0	0	0	0	0	0	0	0	0	0	0	0	0	0	0	0	0	2.138	0	0	0	0	0	7.65	0	1
Os03g0781200	16.3	48.4	19.4	39.41	40.68	40.66	23.5	19.6	19.3	21.55	25.22	6.586	19.593	18.669	17.423	35.82	10.31	18.65	22.29	40.31	20.69	35.49	33.49	22.39	15.85	10.37	13.16	31.5	15.3	20	2
Os03g0194500	52	52.2	47.4	39.41	51.53	57.09	64.2	63.3	62.2	50.87	64.69	59.27	53.337	58.807	40.39	47.76	51.53	45.59	61.31	66.12	98.29	46.58	38.48	59.95	57.04	52.8	43.86	41.6	33.5	53.3	15
Os03g0796501	984	798	856	1271	1199	1328	2416	2049	2674	1175	1044	1759	1157.1	1184.6	841.86	1206	783.2	921	1486	1175	1504	1378	1463	1600	996.2	1251	1228	651	801	661	18
Os03g0648500	1.02	9.31	3.23	1.642	0.904	7.785	3.91	3.16	6.22	1.724	3.289	0	0	0.9335	3.1679	0	2.061	4.144	1.858	0	2.587	4.436	8.552	5.778	4.225	0.943	6.579	2.78	0	3.33	17
Os03g0355600	1112	1346	1150	1497	1643	1512	1462	1623	1484	1005	1020	977.5	949.19	1042.7	1029.6	1719	1146	1200	1129	1246	1201	1417	1554	1471	1057	1055	1252	1114	1095	1128	12
Os03g0581800	1892	1882	1711	1566	1669	1720	2272	1629	1808	1916	1711	2580	1633.9	1895.8	1450.1	2281	1718	1728	2343	1984	2311	1295	1341	1636	1788	1952	2226	1487	1500	1340	20
Os03g0210500	55.1	48.4	64.7	43.51	60.57	120.2	16.4	20.2	18	18.11	16.45	44.22	46.806	85.878	44.35	286.6	75.23	45.59	52.95	52.41	68.12	5.176	5.701	4.333	35.92	35.83	24.12	55.5	81.3	82.5	6
Os03g0748800	363	400	331	487.7	508	500.9	233	336	241	344.9	392.5	350	468.06	377.11	447.46	728.4	328.7	445.5	373.4	383.8	373.3	378.6	380.5	310.6	433.1	369.6	361.8	385	439	495	6
Os03g0105500	0	3.73	1.08	0	0	0	0	0	1.38	0	2.193	0.941	3.2656	0	0.792	0	0	0	0	0.806	0	0.739	0.713	0.722	1.056	0	6.579	2.78	0.96	0	11
Os03g0249700	1820	1624	1512	523	546	513	1154	1168	863	1347	1199	1259	1025.4	1061.3	1057.3	1684	1699	1530	461.7	545.9	524.2	675.1	708.4	922.3	1196	1212	1153	1448	1121	1254	3
Os03g0309200	1233	1451	1234	1892	1898	1699	2248	2135	2326	1221	1373	1596	1233.3	1132.3	1040.6	2269	1325	1449	1720	1741	1913	2110	1883	2115	1274	1368	1182	1133	1240	1203	12
Os03g0364700	201	244	189	214.3	186.2	171.3	137	164	156	272.5	307	352.8	211.17	178.29	229.67	47.76	213.3	294.2	133.8	170.1	131.1	142	101.2	119.2	221.8	203.7	195.2	188	159	195	20
Os03g0155700	0	0	0	0	0	0	0	0	0	0	0	0	0	0	0	0	0	0	0	0.806	0	0	0	0	0	0	0	0	0	0	16
Os03g0144800	156	116	107	113.3	87.69	83.04	105	68.9	82.2	95.7	71.27	96.9	74.019	68.142	83.156	167.2	92.75	132.6	95.68	67.73	91.4	77.64	108.3	98.95	75	93.34	118.4	92.5	102	58.3	6
Os03g0680100	0	0	0	0	0	0	0	0	0	0	0	0	0	0	0	0	0	0	0	0	0	0	0	0.722	0	0	0	0	0	0	9
Os03g0642600	0	0	0	0	0	0	0	0	0	0	0	0	0	0	0	0	0	0	0	0	0	0	0	0.722	0	0	0	0	0	0	9
Os03g0799700	961	1100	1156	1520	1823	1348	1333	1756	1334	1065	1309	1053	1555.5	1372.2	1208.5	2173	1034	1098	1479	1651	1547	874	880.1	1022	1056	943.8	697.3	936	1089	1161	6
Os03g0216766	0	0	0	0	0	0	0	0	0	0	1.096	0	0	0	0	0	0	0	0	0	0	0.739	0	0	0	0	0	0	0	0	7
Os03g0262150	1.02	1.86	0	0.821	0	0	1.57	0	0	0	0	0	0	0	0.792	0	0	0	0.929	0	0	0	0	0.722	0	0	0	0	0	0	14
Os03g0169500	2.04	3.73	4.31	15.6	22.6	31.14	32.9	24.7	27.6	5.173	7.675	1.882	1.0885	3.7338	5.5438	0	1.031	2.072	16.72	32.25	23.28	19.96	15.68	22.39	19.01	5.657	2.193	5.55	7.65	4.17	12
Os03g0602500	44.9	48.4	36.6	73.89	80.45	45.85	57.2	87.9	42.8	32.76	39.47	21.64	28.302	30.804	37.222	35.82	18.55	33.15	39.02	62.08	31.04	42.88	42.05	50.56	20.07	16.03	4.386	17.6	33.5	28.3	12
Os03g0109300	432	207	150	50.08	65.99	127.2	289	209	55.3	338.8	312.5	529.7	144.77	369.65	60.981	119.4	309.2	426.8	76.17	26.61	62.94	103.5	66.99	231.8	214.4	212.1	506.6	193	211	154	20
Os03g0390700	498	422	449	387.5	417.6	380.6	467	419	434	384.5	380.4	412.1	386.42	404.18	448.25	597	446.2	415.5	413.4	416.8	399.2	387.4	419.7	451.4	400.4	373.4	447.4	431	446	472	6
Os03g0101600	1061	1254	1169	1042	1017	1011	1317	1089	1337	1414	1453	1668	1522.8	1271.4	1364.6	1457	1094	1019	1097	865.9	921.7	848.8	919.3	931.7	1327	1263	1340	1037	1150	1163	20
Os03g0118100	0	0	0	0	0	0	0	0	0	0	2.193	0	0	0	0	0	0	0	0	0	0	0	0	1.444	0	0.943	0	0	1.91	0	1
Os03g0223200	18.4	18.6	14	0.821	4.52	4.325	2.35	1.9	0	32.76	25.22	34.81	7.6196	14.002	18.215	23.88	6.183	17.61	0.929	0.806	0	1.479	1.425	0.722	14.79	11.31	6.579	8.33	13.4	5	3
Os03g0119100	0	0	2.16	1.642	0	0	0	1.27	2.76	0.862	0	0	0	0	0	0	0	0	4.645	0	0.862	0.739	0.713	2.167	0	0.943	0	4.63	0.96	1.67	1
Os03g0116366	0	1.86	0	0	0	0	0	0	0	0	0	0	0	0	0	0	0	0	0	0	0	0	0	0	0	0	0	0	0	0	14
Os03g0131000	3.06	0	0	0	0	0	4.7	3.16	5.53	2.587	0	0	1.0885	0	0.792	0	0	0	1.858	0.806	0	2.218	4.988	2.889	0	0.943	2.193	1.85	0	0.83	18
Os03g0839600	50	50.3	56	133	158.2	100.3	139	149	89.1	47.42	71.27	82.79	69.665	42.939	37.222	35.82	54.62	61.13	195.1	252.4	166.4	98.34	84.09	105.4	48.59	39.6	28.51	51.8	49.7	31.7	10
Os03g0823500	25.5	20.5	6.47	27.91	25.31	27.68	5.48	6.96	4.84	36.21	24.12	28.22	10.885	28.004	13.463	11.94	10.31	14.5	23.22	23.38	8.622	11.83	6.414	7.945	16.9	14.14	32.89	13.9	3.83	16.7	7
Os03g0789000	14.3	10.2	11.9	8.21	4.52	8.65	6.26	7.59	4.15	6.897	14.25	5.645	6.5311	2.8004	3.1679	0	4.122	13.47	2.787	1.613	0	22.92	17.82	18.78	1.056	6.6	4.386	11.1	5.74	6.66	9
Os03g0243900	1.02	0.93	2.16	1.642	0.904	0.865	7.05	4.43	6.91	0	1.096	0	1.0885	0	0.792	0	1.031	1.036	1.858	0	4.311	2.218	0	3.611	2.113	0	0	0.93	0	0	18
Os03g0607100	0	0	0	0	0	0	0	0	0	0	0	2.822	0	0	0	0	0	0	0	0.806	0	0	0	0	0	0.943	0	0	0	0	20
Os03g0135550	5.1	11.2	7.54	8.21	2.712	7.785	16.4	6.96	11.7	10.35	6.578	5.645	6.5311	2.8004	5.5438	0	12.37	4.144	9.29	13.71	6.036	7.394	14.97	9.389	1.056	2.829	8.772	6.48	8.61	5.83	17
Os03g0197200	191	227	247	99.34	120.2	38.06	506	418	268	402.6	308.1	300.1	229.68	215.63	319.95	119.4	145.3	193.7	116.1	145.9	55.18	108.7	277.9	156.7	233.5	129.2	144.7	197	269	217	5
Os03g0369800	619	670	556	756.9	816.3	814.9	1055	1024	1026	718.2	644.7	835.4	664	646.88	563.88	1206	717.2	607.1	788.7	739.4	788.1	1064	912.2	1044	693	796.7	809.2	644	630	648	18
Os03g0840200	690	637	702	548.4	446.6	563.1	483	339	460	1407	1222	1167	1149.5	1125.7	1048.6	991.1	831.6	761.5	811	911.1	790.7	210	265.8	304.1	1016	968.3	969.3	814	926	702	3

Os03g0822300	129	115	92.7	223.3	220.6	256.1	118	109	109	138.8	105.3	151.5	152.39	149.35	110.08	214.9	110.3	116	283.3	267.7	335.4	130.9	134	101.1	108.8	122.6	131.6	111	130	103	10
Os03g0197900	1.02	1.86	3.23	0.821	0	0	3.13	6.96	2.76	7.76	0	0.941	0	0.9335	0.792	0	0	2.072	0	0	0	2.218	1.425	4.333	0	0	0	0	0	0	7
Os03g0752300	1185	1122	1265	200.3	196.2	235.3	507	311	390	760.4	1019	1172	1184.3	1200.4	1031.1	2197	1934	1201	239.7	208.8	238.8	334.2	338.5	546	1180	2021	2057	1456	2033	1681	13
Os03g0356470	1.02	0.93	8.62	0.821	0	2.595	0	0	2.76	0.862	4.386	4.704	0	0	3.1679	0	4.122	2.072	0.929	0	0.862	3.697	4.276	2.889	2.113	0	0	0	0.96	2.5	14
Os03g0565900	30.6	17.7	29.1	27.91	28.02	21.63	21.9	43.6	22.8	19.83	29.6	22.58	32.656	38.272	35.638	35.82	28.85	32.12	26.01	41.12	21.56	46.58	30.64	16.61	36.97	29.23	21.93	28.7	25.8	35	4
Os03g0701100	416	143	453	31.2	30.73	25.95	10.2	10.8	11.1	225.9	263.1	247.4	557.32	345.38	580.51	155.2	103.1	129.5	37.16	29.83	51.73	12.57	3.563	4.333	331.7	114.1	203.9	229	187	185	3
Os03g0421400	671	702	689	810.3	867.8	659.2	816	970	846	879.4	743.4	720.7	759.79	756.1	771.37	1182	622.4	609.2	762.7	828.9	613.9	710.6	916.4	735.2	685.6	626	693	713	724	632	6
Os03g0261950	11.2	9.31	14	27.91	25.31	19.9	1.57	3.16	3.46	6.035	4.386	10.35	6.5311	6.5342	13.463	0	5.153	8.288	1.858	4.031	16.38	15.53	9.264	7.945	11.62	1.886	0	14.8	9.56	24.2	2
Os03g0255100	557	472	637	326.7	273.9	341.7	535	813	488	665.6	540.5	750.8	906.74	802.77	699.31	609	591.5	542.9	285.2	216.1	162.1	485.8	434	523.6	478.5	616.6	706.1	536	563	580	3
Os03g0213300	1746	1883	1666	1820	1765	1815	1618	1653	1580	1867	1727	1693	1812.4	1546.7	1915.8	2794	1757	1867	1406	1774	1727	1792	1913	1700	2037	1970	1726	2090	1853	2034	13
Os03g0225200	57.1	43.8	36.6	45.15	29.83	41.52	46.2	33.5	29.7	65.53	54.82	68.68	64.223	41.072	53.854	59.7	35.04	66.31	42.73	53.21	31.04	68.02	48.46	40.45	69.72	66	39.47	45.3	35.4	43.3	20
Os03g0603100	1.02	1.86	1.08	2.463	0	2.595	0	0.63	1.38	0	1.096	3.763	0	3.7338	0	0	0	0	3.716	2.419	0	0	0	0	0	0	2.193	0	0	1.67	16
Os03g0351300	391	386	356	19.7	15.37	23.36	132	140	193	228.5	235.7	274.7	342.88	256.7	300.16	561.2	386.4	378.2	6.503	16.93	5.173	173.8	233.7	205.1	252.5	262.1	195.2	285	189	292	13
Os03g0210400	383	472	481	405.6	468.3	483.6	177	183	205	269	297.1	273.8	383.16	311.77	366.68	764.2	396.8	444.5	223.9	271.7	219	473.9	396.9	286.7	341.2	347	280.7	504	482	522	8
Os03g0435300	0	0	0	1.642	0	2.595	0.78	2.53	0	0	0	0	0	0	0	0	0	0	2.787	1.613	0	0.739	0.713	1.444	0	0.943	0	0	0	0	12
Os03g0791700	59.1	64.3	64.7	50.08	74.13	47.58	96.3	116	91.9	59.49	87.71	64.92	75.108	54.14	82.364	167.2	73.17	70.45	41.8	59.66	30.18	68.02	87.65	67.17	61.27	53.74	54.82	86	91.8	70	6
Os03g0289800	3.06	4.66	0	119	11.75	73.53	5.48	16.4	31.1	10.35	2.193	4.704	5.4426	0.9335	7.9197	0	3.092	0	28.8	33.86	39.66	82.07	299.3	72.95	35.92	11.31	41.67	13	28.7	30	17
Os03g0170200	461	490	484	456.5	500.8	423.9	377	421	365	455.2	526.3	408.3	480.04	437.79	468.84	561.2	505	521.1	438.5	490.2	448.4	507.2	431.1	434.1	517.6	480.8	438.6	514	551	552	8
Os03g0685700	86.7	111	91.6	101.8	109.4	108.1	40.7	30.4	23.5	49.14	30.7	8.467	47.895	57.874	52.27	131.4	26.79	85.99	32.51	27.41	26.73	116.1	91.22	66.45	38.03	40.54	4.386	72.2	57.4	89.1	8
Os03g0439000	39.8	32.6	33.4	24.63	28.93	27.68	14.1	10.8	26.9	13.79	8.771	26.34	26.124	20.536	19.007	47.76	46.37	30.05	25.08	24.19	29.32	28.1	39.91	15.17	24.3	26.4	21.93	31.5	19.1	23.3	14
Os03g0658000	0	0	0	0	0	0	0	0	1.38	0	3.289	0	0	0	0	0	0	0	0	0	0.862	0	0	0	0	0	0	0	0	0	7
Os03g0195800	3.06	7.45	5.39	1.642	0	0	1.57	0.63	2.76	5.173	1.096	5.645	2.177	0.9335	2.3759	0	1.031	0	2.787	2.419	4.311	1.479	0.713	0	0	0	4.386	0	0	2.5	14
Os03g0578300	776	739	756	697.8	733.1	636.7	560	725	743	673.4	914.4	823.2	807.68	739.29	867.2	788.1	667.8	733.5	636.3	742.6	718.2	910.2	775.3	693.4	825	843.8	793.8	831	825	835	1
Os03g0718100	318	255	248	183.9	156.4	205.9	262	201	231	350	268.6	402.7	236.21	260.43	220.96	143.3	316.4	214.5	263.8	228.2	217.3	130.1	165.3	215.2	260.9	293.2	322.4	210	291	170	20
Os03g0238800	719	530	724	157.6	128.4	174.7	43.8	44.3	66.3	394	401.3	517.5	618.28	608.61	632.78	871.7	717.2	542.9	249.9	200	240.6	83.55	89.79	90.28	552.5	498.8	526.3	621	603	534	3
Os03g0845800	158	183	191	302.1	314.6	304.5	424	384	414	194	209.4	171.2	165.46	146.55	129.88	214.9	117.5	172	261	332.2	264.7	363	338.5	372.7	150	187.6	109.6	155	140	166	12
Os03g0289400	77.5	94.1	91.6	196.2	266.7	161.8	243	268	243	123.3	145.8	179.7	109.94	80.277	82.364	83.59	56.68	94.28	249.9	229.8	255.2	133.8	126.1	193.6	79.23	83.91	105.3	28.7	48.8	46.7	12
Os03g0152300	0	0	0	0	0	0	0	0	0	0	0	0	0	0	0	0	0	0	0	0	0	0	0	0	0	0	0	0	0	0.83	1
Os03g0285200	55.1	72.7	44.2	13.96	12.66	18.17	32.9	53.8	26.9	25	7.675	23.52	10.885	28.004	26.927	35.82	24.73	20.72	22.29	21.77	36.21	16.27	47.03	18.78	13.73	16.97	17.54	15.7	21	17.5	14
Os03g0813700	266	375	319	506.5	564.1	352.1	437	505	516	372.5	390.3	251.2	378.8	334.18	336.59	262.7	223.6	216.5	362.3	307.2	373.3	435.5	425.4	385	269.4	205.5	195.2	184	223	285	12
Os03g0688200	854	672	830	688	740.4	693.8	595	595	648	837.2	740.1	887.2	817.48	777.57	788.01	1409	820.3	751.1	670.7	648.3	680.3	513.1	496	537.4	871.5	838.2	912.3	620	676	650	13
Os03g0313800	107	108	165	67.32	37.06	55.36	39.1	35.4	35.2	178.5	174.3	136.4	133.89	111.08	154.43	417.9	176.2	147.1	109.6	90.3	153.5	27.36	27.79	31.06	128.9	212.1	162.3	145	175	200	13
Os03g0363500	1249	1289	1419	1027	1101	919.5	1675	1845	1745	1483	1561	1565	1643.7	1496.3	1782.7	2400	1413	1194	1144	1113	1174	1075	998.4	1247	1400	1289	1153	1152	1248	1276	6
Os03g0641300	34.7	63.3	50.6	48.44	23.5	37.2	4.7	6.33	4.15	12.07	18.64	3.763	20.682	29.87	38.014	23.88	78.32	73.56	20.44	32.25	37.08	31.79	71.98	38.28	81.34	70.71	57.02	113	71.7	85.8	1
Os03g0781600	0	0.93	0	0	0	0	0	0	0	0	0	0	0	0	0	0	0	0	0	0	0	0	0	0	0	0	0	0	0	0	14
Os03g0659800	2.04	0	0	2.463	2.712	1.73	0	3.8	0.69	0.862	2.193	3.763	0	0	0	11.94	1.031	1.036	1.858	6.45	0	0.739	4.988	4.333	0	1.886	0	0.93	0.96	0	6
Os03g0796502	1.02	0	3.23	8.21	1.808	7.785	10.2	8.86	8.98	3.449	5.482	0	3.2656	0	3.1679	11.94	2.061	4.144	7.432	0.806	2.587	5.915	3.563	4.333	1.056	2.829	6.579	3.7	0.96	2.5	6
Os03g0670000	218	173	190	208.5	205.2	266.4	251	211	278	222.4	237.9	233.3	238.39	214.69	235.21	549.3	204	188.6	318.6	295.1	316.4	181.9	195.3	205.8	214.4	239.5	221.5	172	195	157	6
Os03g0694700	120	112	99.1	194.6	200.7	200.7	288	218	226	154.3	121.7	172.2	153.48	132.55	137.8	298.5	134	99.46	254.5	213.7	243.1	153.8	175.3	179.8	138.4	143.3	162.3	110	137	98.3	12
Os03g0713600	37.7	36.3	53.9	32.84	33.45	39.79	14.9	22.1	18.7	18.11	10.96	15.05	22.859	15.869	19.799	47.76	38.13	38.33	31.58	20.16	33.63	14.79	18.53	23.83	4.225	18.86	30.7	16.7	17.2	18.3	14
Os03g0356414	72.4	53.1	73.3	114.1	99.44	80.45	218	242	191	94.84	65.78	65.86	51.16	64.408	72.069	59.7	57.71	59.05	109.6	103.2	105.2	282.4	280.1	218.1	76.06	74.48	41.67	67.5	60.2	48.3	18
Os03g0657100	104	80.1	67.9	110	100.3	96.02	91.6	132	120	61.22	59.21	48.92	78.373	33.604	55.438	107.5	91.72	61.13	71.53	98.37	67.25	135.3	142.5	132.2	38.03	54.68	30.7	60.1	53.6	65	17
Os03g0833300	1536	1576	1486	1117	1133	1152	795	869	837	1158	1218	1110	1311.7	1228.4	1381.2	2125	1408	1395	948.5	1032	1064	1179	1167	1137	1299	1257	1351	1481	1450	1486	13
Os03g0848100	448	485	506	885	1144	997.4	600	677	717	337.1	494.5	410.2	497.45	380.85	411.82	549.3	325.6	431	696.7	945	788.1	723.1	622.8	625.5	340.1	318.7	186.4	314	395	409	10
Os03g0688300	256	257	252	41.87	31.64	38.06	49.3	50	49.8	227.6	257.7	303.9	243.83	213.76	252.64	226.9	197.9	196.8	53.88	64.5	63.8	70.24	100.5	74.39	294.7	267.8	263.2	291	240	286	3
Os03g0332900	16.3	20.5	15.1	95.23	69.61	162.6	28.2	13.3	17.3	10.35	6.578	13.17	7.6196	27.07	13.463	0	11.34	15.54	75.25	66.92	53.46	19.96	17.82	23.83	3.169	16.03	15.35	17.6	21	15.8	10
Os03g0339900	1362	1539	1612	8706	8907	8395	131	144	168	1152	1152	840.2	1101.6	1371.2	1000.3	1946	1547	1304	7986	8427	9583	168.6	212.4	178.4	950.7	944.7	739	1098	1149	1216	10
Os03g0285100	512	511	550	270.9	277.5	295.8	102	135	149	436.3	526.3	422.4	611.75	630.08	537.74	668.7	571.9	504.5	331.6	292.7	367.3	71.72	93.36	103.3	484.9	456.3	517.5	389	506	532	3
Os03g0706500	1.02	0	0	2.463	0.904	0.865	0	0.63	0	0.862	0	0	0	0	3.1679	0	0	1.036	0	0.806	0	0	0	0	0	0	0	0	0	0	2
Os03g0149300	218	116	107	104.3	77.74	185.1	35.2	68.3	84.3	84.49	85.52	223	68.577	130.68	61.773	179.1	223.6	224.8	158.9	49.18	35.35	173	187.4	156	126.8	334.7	513.1	149	164	156	11
Os03g0859550	4.08	2.79	5.39	7.389	17.18	13.84	10.2	7.59	15.2	1.724	0	0	0	0	0.792	0	1.031	1.036	8.361	12.9	2.587	11.83	6.414	17.33	1.056	2.829	0	1.85	0.96	1.67	12
Os03g0116600	1.02	0	0	3.284	2.712	6.92	3.13	11.4	6.22	0.862	0	1.882	0	0.9335	3.9598	0	1.031	0	0	1.613	5.173	2.218	1.425	1.444	1.056	2.829	2.193	0	0	0.83	18
Os03g0595800	72.4	57.7	50.6	64.86	65.99	85.64	21.9	56.9	37.3	49.14	54.82	59.27	78.373	77.476	49.894	179.1	58.74	58.02	77.1	86.27	56.04	36.23	53.45	39.72	49.65	62.23	59.21	38.9	70.8	46.7	6
Os03g0645100	633	736	744	1040	1175	1066	1316	1388	1504	558.7	711.6	625.6	575.83	587.14	623.28	1325	668.8	615.4	1093	1068	1066	1163	1140	1352	577.8	600.6	635.9	538	567	562	12
Os03g0650400	223	156	222	133	140.1	145.3	141	97.4	98.1	131.9	162.3	119.5	157.84	160.55	149.68	191.1	213.3	162.7	112.4	155.6	138	91.68	89.08	80.89	152.1	121.6	153.5	167	189	183	13
Os03g0684400	4428	5288	5267	298.8	311.9	233.6	871	923	921	7073	8250	6780	7715.4	6104.8	7924.4	7344	4974	5038	326.1	264.5	275.9	473.9	574.4	634.9	7103	6513	5566	4771	4957	6054	3
Os03g0584500	0	0	0	0	0	0	0	0	0	0	0	0	0	0	0	0	0	0	0	0	0	0	0	0	0	0	2.193	0	0	0	11
Os03g0277100	2441	2145	2028	837.4	722.3	863.3	842	1053	935	1419	1631	1593	1620.8	1262	1424.7	2233	2249	1794	768.2	744.2	888.1	1014	1261	1268	1336	1439	1278	1308	1213	1220	14
Os03g0445700	9.18	5.59	4.31	0	0	1.73	0	0	2.07	4.311	7.675	14.11	3.2656	0	0	0	3.092	5.18	0	0	0	0.739	0.713	0.722	1.056	2.829	15.35	1.85	1.91	0	20
Os03g0205150	242	224	223	213.5	211.5	215.4	429	328	390	325	319.1	368.8	336.35	264.17	256.6	310.5	254.5	220.7	288.9	308	331.1	312	309.3	343.1	290.5	346	344.3	275	270	267	5
Os03g0617101	94.8	94.1	92.7	92.77	92.2	116.8	160	140	146	143.1	128.3	138.3	141.51	126.02	112.46	83.59	82.44	98.42	105	76.6	100	120.5	90.5	121.3	115.1	141.4	129.4	91.6	74.6	102	5
Os03g0750000	2.04	0.93	4.31	0	0	4.325	0	1.27	0	0	0	0	0	1.8669	0	0	2.061	1.036	0	0	0	0	0.713	0	0	0	0	0	0	0	14
Os03g0598200	592	577	517	855.5	828.9	866.8	616	655	676	455.2	503.2	538.1	573.65	549.8	521.91	1087	473	578.1	674.4	727.3	748.4	581.2	610	517.8	471.1	550.6	480.2	529	504	505	6
Os03g0801700	319	300	324	376.8	448.4	395.3	294	343	254	293.1	409	399.8	402.75	452.72	374.6	692.6	339	348.1	416.2	414.4	466.5	241	232.3	293.2	351.8	334.7	346.5	311	312	327	6
Os03g0789800	277	295	267	459.7	535.1	470.6	652	772	706	300.9	337.7	318.9	346.15	294.97	332.63	250.8	290.6	333.6	410.6	462	480.3	676.5	550.9	611	293.7	298.9	320.2	294	333	329	18
Os03g0569900	436	393	391	947.4	980.8	894.5	838	769	786	525.9	414.4	574.8	409.28	447.12	366.68	597	344.2	399.9	860.2	789.3	915.7	664	639.9	645	387.7	431.8	438.6	342	336	333	12
Os03g0433200	2.04	0.93	1.08	1.642	0.904	5.19	3.13	3.16	6.91	1.724	2.193	5.645	2.177	3.7338	3.9598	0	3.092	3.108	2.787	0.806	2.587	5.176	3.563	5.778	0	0	0	0	1.91	0.83	18
Os03g0735900	2.04	0	0	0	0	0	0	0	0	0	0	0	0	0	0	0	0	0	0	0	2.587	0	0	0.722	0	0	0	0	0	0	19
Os03g0388500	1865	1705	2088	2104	2038	1687	1935	1938	1522	1679	1441	1140	1275.7	1269.5	1678.2	2484	1383	1438	1826	2212	1528	1529	1865	1403	1106	1176	1110	1572	1826	1524	6
Os03g0145200	6.12	6.52	2.16	2.463	3.616	3.46	13.3	5.69	9.67	2.587	8.771	4.704	4.3541	6.5342	6.3357	0	7.214	3.108	3.716	10.48	16.38	5.915	4.276	5.056	3.169	11.31	6.579	6.48	9.56	2.5	15
Os03g0692000	8.16	14	6.47	1.642	1.808	3.46	0	0.63	2.07	1.724	7.675	9.408	8.7082	2.8004	7.9197	0	12.37	9.324	3.716	3.225	2.587	4.436	1.425	2.889	13.73	13.2	15.35	14.8	7.65	5	3
Os03g0184600	495	499	500	620.7	539.7	620.2	773	750	810	669.9	650.2	679.3	581.27	634.75	662.08	1099	494.7	490	642.8	637	698.4	819.2	871.6	825.5	651.8	589.3	642.5	492	480	505	6
Os03g0757000	68.3	191	149	283.2	285.7	147.9	438	713	550	323.3	205	169.3	288.46	196.02	352.42	226.9	40.19	95.32	206.2	149.2	81.91	337.9	450.4	317.1	104.6	77.31	46.05	156	189	214	18
Os03g0852900	712	802	717	897.3	883.2	924.7	887	936	955	910.5	869.4	913.5	936.13	905.45	931.35	1170	792.5	840.2	858.4	840.1	863.1	804.5	847.3	830.6	929.6	933.4	896.9	755	837	866	6
Os03g0278366	0	5.59	0	3.284	1.808	6.055	7.05	0	2.07	1.724	1.096	2.822	10.885	0.9335	1.5839	0	1.031	2.072	0.929	1.613	4.311	2.218	0	5.778	0	1.886	0	0	0	0	4
Os03g0188900	209	177	172	39.41	35.25	33.74	92.4	62.6	58.7	105.2	99.77	112.9	78.373	56.941	73.653	95.53	86.56	89.1	16.72	12.09	14.66	40.67	105.5	62.11	58.1	55.63	76.75	50.9	91.8	44.2	14
Os03g0324800	268	247	237	162.6	142.8	197.2	231	192	233	302.6	292.7	333.1	314.58	300.57	283.52	453.8	259.7	236.2	189.5	165.3	150	181.1	172.5	186.3	300	278.1	254.4	257	274	257	13
Os03g0821550	2.04	2.79	2.16	11.49	3.616	12.98	8.61	3.16	10.4	4.311	1.096	0	1.0885	0	0.792	0	4.122	1.036	9.29	16.13	18.11	6.654	7.126	5.778	2.113	2.829	2.193	4.63	0	0.83	10
Os03g0290900	0	0	0	0	0	0	1.57	0	0	0	0	0	0	0	0	0	2.061	1.036	0	0	0	0	0	0	0	0	0	0	0	0	1
Os03g0713000	269	278	296	1722	1947	1702	986	960	1017	245.7	262	336.8	257.98	269.77	202.74	453.8	308.1	318.1	1642	1329	1561	1067	870.1	1026	293.7	333.8	381.6	285	274	265	10
Os03g0356498	23.4	15.8	11.9	5.747	1.808	0.865	0	0	0	21.55	7.675	27.28	18.505	22.403	21.383	23.88	21.64	11.4	2.787	3.225	0	0	0.713	2.167	23.24	13.2	41.67	8.33	8.61	12.5	3
Os03g0118700	0	0	0	1.642	0.904	0.865	0	0	0	0	0	0	0	0	0	0	0	0	0	0	0	0	0.713	0	0	0	0	0	0	0	2
Os03g0146800	1.02	1.86	1.08	4.105	2.712	3.46	1.57	6.96	3.46	11.21	8.771	0	6.5311	7.4676	16.631	11.94	11.34	7.252	12.08	16.93	34.49	5.915	9.977	24.56	9.507	16.03	52.63	88.8	37.3	18.3	1
Os03g0419100	465	412	487	253.7	243.2	209.3	208	283	301	247.4	299.3	212.6	365.74	316.44	376.18	537.3	408.1	347.1	175.6	234.6	222.5	444.4	401.9	335.1	320.1	287.6	245.6	370	373	446	8
Os03g0642450	78.5	74.5	73.3	67.32	94.01	76.12	66.5	128	82.2	48.28	61.4	31.05	88.17	61.608	52.27	59.7	70.08	82.88	56.67	42.73	73.29	89.47	86.94	88.11	55.99	48.08	35.09	53.7	70.8	95.8	17
Os03g0115400	506	520	481	543.5	536.1	396.2	463	638	537	460.4	415.5	393.3	401.66	412.59	468.84	835.9	472	479.7	441.3	491	419	661.8	648.5	619	577.8	400.7	412.3	561	574	566	6
Os03g0856100	294	319	349	3588	3759	3334	1066	1050	996	245.7	270.8	407.4	505.07	375.25	275.6	835.9	361.7	389.5	3562	3630	3732	873.2	786.7	1062	218.7	198	219.3	288	395	383	10
Os03g0179950	45.9	27.9	22.6	59.93	38.87	45.85	53.2	51.9	56.7	62.94	53.72	58.33	41.364	38.272	44.35	23.88	46.37	27.97	69.67	67.73	69.84	31.79	49.17	57.78	32.75	39.6	54.82	56.4	33.5	29.2	15
Os03g0598002	7.14	7.45	4.31	4.105	4.52	3.46	2.35	1.9	6.22	5.173	6.578	6.586	2.177	5.6007	3.9598	11.94	1.031	3.108	10.22	2.419	2.587	2.958	1.425	2.889	3.169	2.829	4.386	2.78	0.96	1.67	6
Os03g0567600	38.7	44.7	44.2	118.2	132	70.93	43.1	54.4	53.9	15.52	31.8	14.11	54.426	23.336	32.471	83.59	32.98	24.86	48.31	33.86	29.32	62.11	49.88	51.28	27.47	15.09	21.93	30.5	33.5	31.7	2
Os03g0178500	295	277	295	110	104	96.89	125	137	163	360.4	404.6	378.2	199.2	248.3	283.52	692.6	356.6	289.1	168.1	127.4	95.71	99.82	112.6	145.9	269.4	397.9	510.9	366	340	267	13
Os03g0431500	815	829	860	649.4	709.6	678.2	788	851	835	890.6	1045	922.9	936.13	977.32	1014.5	1982	1060	1074	842.6	811.9	792.4	1002	788.9	749.7	1102	1107	1283	1224	1111	1148	13
Os03g0171600	1750	1189	1955	674.8	588.5	675.6	680	638	860	779.4	718.1	558.8	678.15	803.7	735.74	1194	1176	1227	562	604.7	596.7	680.2	930.7	795.9	761.6	768.4	883.7	807	963	811	14
Os03g0213900	3317	3244	3460	2817	3121	3078	2531	2799	2550	2339	2788	2690	2992.3	2792	2918.4	5827	3273	3515	2650	2989	2740	4061	3127	2653	3076	3090	2746	3466	3526	3667	6
Os03g0559700	273	276	190	284.1	198	231.8	370	459	283	272.5	273	316.1	218.79	220.29	217	358.2	276.2	258	177.4	155.6	144.9	275.8	445.4	286	356	342.3	309.2	299	219	261	5
Os03g0673400	15.3	14.9	17.2	9.031	7.232	12.11	3.13	3.16	3.46	9.484	4.386	5.645	18.505	18.669	25.343	35.82	26.79	13.47	1.858	7.257	0.862	1.479	12.83	5.778	12.68	5.657	6.579	26.8	15.3	15.8	13
Os03g0336400	0	0	0	0	0	0	0	0	0	0	0	0	0	0	0	11.94	1.031	0	0	0	0.862	2.218	0	0	0	0	0	0	0	2.5	6
Os03g0666200	1160	1216	1116	1336	1394	1327	1050	1162	1031	1307	1247	1298	1354.1	1333.9	1351.9	1899	1269	1206	1469	1425	1399	1079	1061	1031	1369	1328	1386	1443	1402	1371	13
Os03g0105600	13.3	8.38	12.9	12.31	21.7	32.01	23.5	15.2	17.3	19.83	17.54	27.28	7.6196	16.802	5.5438	0	12.37	13.47	32.51	22.58	39.66	22.92	12.11	26	9.507	7.543	15.35	4.63	18.2	6.66	15
Os03g0178000	4907	3805	4126	4808	4960	5370	6879	5889	7150	5872	4796	9072	5010.5	5799.5	4298	5170	4076	4382	6741	5526	6453	4558	4470	5018	4503	5977	6322	3354	4129	3028	15
Os03g0797600	588	652	575	521.3	462.8	501.7	1228	997	922	1021	769.7	1129	710.8	739.29	721.48	716.5	592.6	621.6	583.4	487	570.8	888	720.5	1138	750	821.2	918.8	688	710	668	5
Os03g0817000	186	176	178	159.3	179.9	207.6	163	125	136	183.6	195.2	202.3	220.97	179.22	156.81	262.7	161.8	196.8	292.6	287	311.3	110.2	97.63	124.2	175.4	206.5	160.1	148	164	145	16
Os03g0815100	4622	5455	3399	437.6	211.5	334.8	110	238	301	1048	960.4	1073	793.53	623.55	832.36	4717	3814	2541	524.9	681.3	1032	593.7	538.8	1431	1110	1017	1059	712	793	930	14
Os03g0732100	1687	1709	1435	1782	1545	1742	581	428	482	1265	1662	1529	1181	1264.8	1135.7	2926	1997	1740	2014	2162	2309	725.3	706.9	676.7	1437	1773	1697	1478	1431	1700	16
Os03g0157800	730	870	827	2092	2045	2011	887	826	860	987.2	1004	885.3	1005.8	1012.8	1024	1457	880.1	881.7	1760	1813	1967	826.6	838.8	792.3	1053	827.8	945.1	944	870	958	10
Os03g0797300	291	259	181	55.83	9.04	54.5	388	1538	482	683.7	525.2	78.09	183.96	135.35	79.197	107.5	164.9	131.6	87.32	35.48	56.91	818.5	113.3	298.3	90.85	114.1	379.4	39.8	44	39.2	18
Os03g0128100	2534	2705	2445	3251	3692	3253	2482	2881	2730	1690	1702	1245	2233.6	2334.6	2277.7	4203	2552	2411	2382	3052	2569	4363	4174	3579	2445	2076	2204	3269	2769	2880	17
Os03g0176700	2331	1574	2420	406.4	471	616.8	373	676	442	1933	2219	2342	2739.8	2950.6	2602.4	3546	2915	2236	884.4	716.8	520.8	340.1	427.6	427.6	2462	2423	2265	2096	1977	1960	3
Os03g0699100	0	0	0	0	0	0	0	0	0	0	0	0	0	0	0	0	0	0	0	0.806	0	0	0	0	0	0	0	0	0	0	16
Os03g0120900	89.7	68.9	115	68.14	29.83	84.77	41.5	27.8	50.4	90.53	58.11	95.96	45.718	53.207	46.726	47.76	86.56	36.26	48.31	31.44	43.97	172.3	110.5	195.7	41.2	86.74	118.4	57.4	41.1	40	9
Os03g0133100	0	0	0	0	0	0	0	0	0	0	0	0	0	0	0	0	0	0	0	0	0	0	1.425	0.722	0	0	0	0	0	0	17
Os03g0454300	1305	1279	1496	1290	1422	1408	1596	1778	1630	1146	1392	1225	1337.8	1237.8	1165.8	2842	1642	1699	1472	1775	1581	1759	1469	1339	1325	1287	1270	1471	1580	1675	6
Os03g0305400	19.4	31.7	17.2	3.284	13.56	12.11	9.39	18.3	32.5	4.311	5.482	5.645	1.0885	8.4011	3.1679	59.7	17.52	5.18	1.858	4.031	4.311	151.6	41.33	62.84	7.395	5.657	2.193	3.7	0	5	9
Os03g0358100	1398	1320	1365	1926	2005	1778	1043	1200	1028	1147	1080	1136	1094	1299.4	1248.1	3272	1636	1348	1859	1712	1875	1193	1424	1378	1279	1196	1346	1519	1289	1415	6
Os03g0760100	67.3	61.5	73.3	15.6	30.73	10.38	106	86.7	72.6	92.25	67.98	107.3	82.728	81.21	102.96	71.65	81.41	99.46	24.15	16.13	30.18	37.71	60.57	31.06	68.66	91.46	67.98	60.1	60.2	58.3	5
Os03g0363950	13.3	12.1	3.23	11.49	17.18	8.65	11.7	10.1	12.4	10.35	10.96	0.941	4.3541	6.5342	19.007	47.76	21.64	10.36	10.22	11.29	9.484	5.176	12.83	15.89	5.282	5.657	28.51	12	21	8.33	6
Os03g0771100	25.5	16.8	11.9	0.821	9.04	4.325	4.7	2.53	0	12.07	12.06	10.35	6.5311	6.5342	4.7518	0	7.214	14.5	4.645	2.419	7.76	0.739	2.851	6.5	7.395	3.771	6.579	6.48	6.69	3.33	19

Os03g0233300	0	0	0	0.821	0	0.865	1.57	0	0	0	0	0	0	0	0.792	0	0	0	0	0	0	1.479	0	2.167	0	0	0	0	0	1.67	9
Os03g0802700	425	433	443	669.1	629.2	657.4	382	380	484	415.6	305.9	539.1	421.26	480.73	354.8	525.4	364.8	434.1	579.7	500.7	535.4	451.8	417.6	369.1	367.6	435.6	396.9	365	377	327	10
Os03g0334300	3.06	0	0	4.105	1.808	0	0	1.9	1.38	0	0	2.822	0	0	0.792	11.94	4.122	0	0.929	0	0.862	2.958	3.563	0.722	0	0	0	0.93	0	0	6
Os03g0292700	70.3	32.6	40.9	0.821	0	4.325	3.91	1.27	2.07	111.2	88.81	140.2	74.019	46.673	54.646	59.7	69.05	49.73	16.72	14.51	5.173	5.915	12.83	9.389	105.6	104.7	72.37	37	63.1	76.6	3
Os03g0268600	65.2	99.7	60.3	80.46	60.57	81.31	83	77.8	79.5	37.07	31.8	29.17	29.39	32.671	27.719	119.4	99.96	70.45	68.74	119.3	156.1	25.88	29.93	36.11	26.41	37.71	35.09	25.9	44.9	29.2	16
Os03g0100100	33.6	31.7	43.1	9.852	1.808	4.325	3.13	7.59	6.22	57.77	67.98	70.56	48.983	55.074	46.726	47.76	32.98	46.62	7.432	4.031	3.449	4.436	2.138	5.056	41.2	31.11	35.09	34.2	35.4	35.8	3
Os03g0272650	3.06	1.86	1.08	0	4.52	0	5.48	7.59	7.6	6.035	1.096	1.882	0	0	2.3759	0	3.092	4.144	0.929	2.419	1.724	3.697	1.425	8.667	2.113	2.829	6.579	0.93	0.96	0.83	18
Os03g0145300	74.4	77.3	92.7	98.52	100.3	111.6	146	104	140	85.36	62.49	98.79	67.488	104.55	75.237	119.4	74.2	81.85	118	131.4	139.7	84.29	79.82	104	79.23	82.97	103.1	83.3	60.2	67.5	15
Os03g0227300	558	610	623	559.1	612.9	540.7	614	603	540	663	655.6	569.2	805.5	741.16	788.8	1075	624.5	651.7	539.7	596.6	573.4	484.3	401.9	468	566.2	570.4	504.4	661	642	699	13
Os03g0264700	229	182	205	247.1	255.8	229.2	380	277	323	311.2	245.6	309.5	230.77	254.83	242.34	286.6	203	182.3	272.2	216.9	220.7	220.3	210.2	275.2	236.6	204.6	184.2	199	195	187	15
Os03g0179000	174	156	112	608.3	710.5	558.8	261	281	265	72.42	86.62	91.26	88.17	149.35	77.613	298.5	119.5	141.9	464.5	454.7	504.4	227.7	275.8	187.1	49.65	81.08	74.56	90.7	80.3	80.8	10
Os03g0367150	44.9	25.1	53.9	16.42	23.5	12.98	27.4	27.8	17.3	73.29	52.63	69.62	44.629	28.004	43.558	47.76	10.31	24.86	16.72	12.9	6.036	28.84	22.09	17.33	21.13	38.66	32.89	18.5	21	20.8	7
Os03g0424300	2.04	0	0	7.389	6.328	7.785	1.57	3.8	0	0	2.193	0	0	0	0	0	0	1.036	1.858	2.419	5.173	1.479	2.138	2.167	0	0	0	0	0	0	10
Os03g0116800	75.4	91.3	120	62.39	56.05	59.69	65	72.1	85.7	63.8	74.55	64.92	117.56	129.75	114.84	226.9	118.5	93.24	53.88	35.48	38.8	85.77	66.28	41.17	97.19	82.03	57.02	120	73.6	118	13
Os03g0370000	1205	961	1086	626.4	643.6	634.9	872	929	1029	1060	928.6	1149	815.3	828.9	806.22	967.2	968.7	1039	649.3	768.4	810.5	1062	833.1	1082	869.4	1060	1035	673	737	659	5
Os03g0204100	22.4	19.6	16.2	17.24	11.75	19.9	24.3	10.8	16.6	15.52	2.193	15.99	8.7082	20.536	10.296	23.88	39.16	17.61	13.93	8.869	14.66	9.612	12.11	15.89	9.507	21.69	28.51	14.8	20.1	5	11
Os03g0150600	1338	1143	1398	655.1	684.3	547.6	2746	3075	2327	1186	765.3	567.3	488.75	672.09	718.31	835.9	871.8	721.1	465.4	413.6	377.7	2042	2620	2345	596.8	385.6	489	342	342	326	18
Os03g0660000	102	159	79.7	284.1	396.8	293.3	416	438	533	106.9	144.7	85.61	188.31	168.02	173.44	95.53	121.6	175.1	203.4	322.5	290.6	627	447.5	442	154.2	108.4	87.72	116	177	147	12
Os03g0268400	4.08	3.73	4.31	13.14	7.232	7.785	79.9	83.5	60.8	4.311	3.289	9.408	4.3541	9.3345	3.9598	0	2.061	3.108	7.432	7.257	12.07	26.62	24.94	38.28	3.169	8.486	8.772	4.63	4.78	2.5	18
Os03g0704100	4948	4559	5529	824.3	802.7	931.7	1134	1509	1165	6566	7394	7941	7505.3	8207.8	8037.7	8359	7074	5659	1385	1332	1618	807.4	932.8	1398	7511	8050	7028	5901	6019	5889	3
Os03g0661300	51	28.9	18.3	26.27	18.08	57.09	29.7	19	24.9	30.18	29.6	34.81	25.036	37.338	26.135	83.59	52.56	36.26	49.23	24.19	49.15	14.05	19.95	44.78	26.41	60.34	96.49	38.9	43	25.8	11
Os03g0823400	115	140	125	430.2	329.9	564.9	196	466	756	294	202.8	535.3	171.99	95.212	194.03	167.2	91.72	174.1	350.2	351.5	514.7	501.3	577.2	324.3	172.2	260.2	346.5	48.1	64.1	36.7	12
Os03g0736000	1083	1122	1172	1123	1196	1100	930	1032	849	966.5	1209	1106	1033	1095.9	1075.5	1982	1144	1145	1163	1138	1264	1053	921.4	965.6	1067	1281	1116	1196	1212	1196	6
Os03g0128200	557	591	578	786.5	890.4	788.1	271	275	306	280.2	253.3	164.6	469.15	401.38	435.58	764.2	465.8	484.9	450.5	593.4	481.1	755.7	709.8	413.8	360.2	269.7	300.4	673	585	647	8
Os03g0809400	330	346	350	319.4	337.2	327.9	317	426	384	309.5	281.8	259.7	348.33	318.31	384.9	513.5	220.5	271.4	251.7	249.1	266.4	365.3	418.3	309.8	377.1	262.1	241.2	341	280	332	6
Os03g0688000	18.4	9.31	18.3	4.105	4.52	3.46	0	0.63	0	5.173	1.096	3.763	25.036	15.869	7.1277	23.88	12.37	13.47	0.929	1.613	1.724	0.739	0	0	10.56	3.771	0	13.9	18.2	14.2	13
Os03g0692400	34.7	27	22.6	22.17	15.37	20.76	40.7	10.1	20	53.46	32.89	50.8	10.885	35.471	15.047	23.88	28.85	49.73	23.22	36.28	31.04	40.67	24.23	26	39.09	33.94	37.28	28.7	27.7	22.5	20
Os03g0587000	468	390	407	400.6	432.1	365.1	400	441	401	330.2	365.1	423.4	371.19	385.52	341.34	632.9	433.9	394.7	456.1	464.4	417.3	390.4	400.5	411.7	359.2	381.9	394.7	377	416	427	6
Os03g0772900	6.12	1.86	2.16	0	3.616	2.595	3.91	3.8	1.38	1.724	1.096	4.704	0	0.9335	0.792	0	2.061	4.144	3.716	0.806	6.898	2.218	0	5.056	0	0.943	0	1.85	2.87	1.67	19
Os03g0711500	272	298	282	312.8	311.9	327	474	417	557	325.9	304.8	360.3	292.81	319.24	300.95	298.5	304	270.4	269.4	295.1	390.6	357.1	386.2	426.1	326.4	305.5	423.2	266	266	243	18
Os03g0156700	322	278	361	96.88	75.93	94.29	110	128	168	225.9	184.2	221.1	202.46	243.63	246.3	394.1	366.9	265.2	102.2	99.17	91.4	118.3	156.8	135.8	212.3	265.9	348.7	238	215	238	13
Os03g0733500	0	0	0	0	1.808	0	0	0	0	0	0	0	0	0	0	0	2.061	0	0	0	0	2.958	0	0.722	2.113	0	0	0.93	0	0.83	9
Os03g0676100	13.3	8.38	15.1	13.96	22.6	23.36	30.5	17.7	17.3	12.93	24.12	15.99	15.239	13.068	12.671	23.88	16.49	11.4	11.15	22.58	13.8	23.66	24.94	13	10.56	25.46	15.35	11.1	14.3	17.5	18
Os03g0613100	11.2	15.8	14	16.42	23.5	22.49	28.2	44.3	24.9	22.42	24.12	24.46	7.6196	9.3345	17.423	0	7.214	9.324	25.08	19.35	25	34.01	24.94	26	21.13	15.09	17.54	10.2	21	5	18
Os03g0647600	466	470	455	292.3	303.7	340	657	425	504	602.7	510.9	723.5	512.69	592.74	538.54	441.8	528.7	500.4	330.7	392.7	412.1	535.3	655.6	582.1	564.1	640.2	684.2	657	521	655	5
Os03g0157000	1.02	1.86	0	0	0	0	0	0.63	0.69	0	1.096	6.586	1.0885	0.9335	0	0	0	0	0.929	0	0.862	0.739	0	2.889	1.056	2.829	0	0.93	1.91	0.83	20
Os03g0844600	0	1.86	0	0	0.904	0	0	0	0.69	0	0	0	0	0	0	0	0	0	0	0	0	0	0	0.722	0	0	0	0	0	0	14
Os03g0314400	31.6	20.5	17.2	60.75	71.41	89.1	79.1	58.8	68.4	18.11	10.96	16.93	25.036	30.804	16.631	71.65	22.67	20.72	55.74	127.4	100	56.93	54.87	53.45	22.18	20.74	13.16	13	27.7	15	12
Os03g0127700	0	0.93	0	0	0	0.865	0	0	0	0.862	1.096	0	0	0	0	0	1.031	0	0	0	4.311	0	0	0	0	0	0	0	0	1.67	16
Os03g0170900	587	349	404	1035	1151	834.8	604	693	814	538	520.8	537.2	337.44	518.07	552	429.9	360.7	577.1	726.4	487	419	950.9	902.2	704.2	439.4	521.4	921	386	604	442	2
Os03g0392300	447	440	448	414.6	387.8	447.2	695	553	563	587.1	523	645.4	544.26	495.66	543.29	573.2	466.8	447.6	622.4	590.2	516.5	434.8	424.7	475.2	496.5	484.6	506.6	443	467	439	15
Os03g0758700	0	0	0	4.926	0	0.865	0.78	0.63	1.38	0	0	0	0	0	0	0	0	0	2.787	0	0.862	0	0	0	0	0	2.193	1.85	0	0	2
Os03g0215700	9.18	23.3	9.7	76.35	56.95	57.96	137	195	144	29.31	39.47	29.17	14.151	17.736	14.255	11.94	8.244	17.61	26.94	31.44	25	171.5	143.2	150.2	31.69	22.63	26.32	5.55	12.4	13.3	18
Os03g0300700	140	93.1	108	178.2	188.9	140.1	108	157	105	110.4	76.75	148.6	90.347	80.277	86.324	119.4	102	114	158.9	157.2	154.3	96.12	106.9	98.95	72.89	76.37	109.6	87.9	78.4	117	10
Os03g0776000	2002	2203	2004	2584	2729	2412	2630	2903	2571	2081	2217	2058	1732.9	2038.7	1944.3	2663	1762	1870	2283	2338	2290	2198	2334	2342	1995	1895	2031	1784	1694	1725	12
Os03g0166200	15.3	17.7	17.2	13.14	8.136	11.25	13.3	3.8	6.22	18.11	13.16	21.64	21.77	7.4676	14.255	35.82	8.244	18.65	5.574	8.063	18.11	19.96	9.264	18.06	20.07	19.8	21.93	18.5	20.1	14.2	13
Os03g0779300	24.5	36.3	19.4	14.78	11.75	11.25	30.5	13.9	13.8	32.76	33.99	22.58	8.7082	18.669	20.591	11.94	34.01	21.76	14.86	13.71	20.69	22.18	21.38	22.39	22.18	20.74	17.54	18.5	23	23.3	7
Os03g0241900	1641	1157	1447	244.7	253.1	306.2	110	110	106	338.8	364	557.9	364.65	395.78	378.56	1385	1576	1248	233.2	267.7	247.5	162.7	191.7	197.9	634.9	555.3	598.7	464	401	402	14
Os03g0848200	0	0	0	0	0	1.73	0.78	0.63	0.69	0.862	0	0.941	2.177	0	0	0	1.031	0	0	0	0.862	2.958	1.425	0.722	0	0.943	4.386	1.85	0	0	11
Os03g0150700	72.4	68	69	121.5	107.6	89.96	139	165	149	90.53	95.39	61.15	129.53	78.41	132.26	83.59	69.05	46.62	80.82	70.95	55.18	114.6	121.1	88.84	81.34	72.6	52.63	68.5	92.8	79.1	18
Os03g0407050	280	346	290	289.8	272.1	259.5	154	198	202	131.9	139.2	63.03	218.79	161.49	176.61	191.1	147.4	225.9	93.82	117.7	76.74	431.8	449	298.3	194.4	132.9	155.7	249	183	282	17
Os03g0178400	434	401	391	23.81	26.22	39.79	13.3	31.6	31.8	450.9	469.3	499.6	321.11	420.05	387.27	800	433.9	403	69.67	60.47	39.66	27.36	37.06	38.28	491.2	575.1	576.7	600	474	446	3
Os03g0191000	1742	2184	1862	1739	1854	1695	2028	2530	1990	1845	1843	1513	1948.5	1659.7	2189.8	3415	1831	2065	1577	1655	1404	2512	2480	2084	1987	1740	1746	2366	2313	2341	6
Os03g0633800	3.06	0	1.08	0.821	0.904	0	0.78	1.27	6.91	0	0	0	0	0	0	0	0	1.036	0	0	0	0.739	0.713	0.722	0	0	0	0	0	0	18
Os03g0408401	0	0	1.08	10.67	16.27	8.65	0.78	1.9	2.76	2.587	2.193	0.941	2.177	6.5342	4.7518	0	2.061	0	9.29	8.063	6.036	1.479	4.988	0.722	1.056	5.657	0	5.55	6.69	2.5	10
Os03g0795900	984	1067	982	1153	1027	989.6	814	913	766	1079	1059	953.1	1105.9	1007.2	1157.1	1122	968.7	992.5	1077	1280	1208	916.1	765.4	915.8	1054	962.6	850.9	1085	1041	1088	16
Os03g0291700	3.06	3.73	1.08	0.821	0.904	0.865	0.78	0.63	2.07	0.862	1.096	1.882	1.0885	1.8669	1.5839	0	2.061	1.036	0	0	1.724	3.697	0.713	2.167	3.169	0.943	2.193	3.7	3.83	8.33	1
Os03g0186100	485	464	570	311.1	337.2	369.4	349	318	326	517.3	536.1	594.6	532.29	505.93	524.28	1242	487.4	481.8	369.7	449.9	419	301.7	246.6	333.7	521.8	527	502.2	455	479	428	13
Os03g0684800	22.4	18.6	20.5	16.42	12.66	10.38	20.4	28.5	15.9	34.49	23.02	22.58	15.239	27.07	21.383	0	15.46	10.36	20.44	17.74	8.622	17.75	20.67	28.17	39.09	27.34	32.89	19.4	23	25.8	6
Os03g0621700	121	106	141	105.1	99.44	79.58	70.5	76.5	47	55.18	82.23	67.74	77.285	114.81	128.3	298.5	215.4	170.9	120.8	104	80.19	79.11	72.69	86.67	100.4	99	76.75	146	141	111	13
Os03g0149200	180	156	156	103.4	95.82	181.7	172	122	131	134.5	118.4	143.9	115.38	166.15	141.76	298.5	138.1	136.8	117	116.1	119.8	130.9	152.5	140.8	129.9	158.4	201.7	130	132	122	6
Os03g0780400	2848	2657	2850	2043	2151	2187	2447	2210	2402	3600	3038	3963	2799.7	3206.4	2920	3475	3062	2765	2789	2329	2515	1560	1720	1994	3330	3354	3577	2708	2820	2731	3
Os03g0241200	1687	1157	1364	1419	1477	1410	1335	1051	1298	1722	1470	2315	1914.7	2150.7	1562.5	2424	1316	1364	1911	1583	1857	1057	974.9	1054	1373	1609	2132	1215	1347	1056	20
Os03g0278400	104	93.1	102	147	134.7	129.8	54	74.6	68.4	81.91	62.49	48.92	60.957	52.273	60.189	167.2	93.78	84.96	64.1	78.21	75.88	108.7	93.36	78	51.76	65.06	63.59	74.9	64.1	117	8
Os03g0206600	247	218	207	124	101.2	115.9	160	213	221	244	244.5	249.3	265.6	243.63	244.72	585.1	181.4	226.9	152.3	126.6	125.9	166.4	163.9	183.5	238.7	228.2	265.3	183	178	186	13
Os03g0604566	20.4	11.2	6.47	27.09	22.6	18.17	0.78	2.53	6.22	12.93	7.675	26.34	4.3541	0	3.9598	0	4.122	8.288	8.361	2.419	8.622	5.176	3.563	6.5	8.451	9.428	2.193	9.25	0	5.83	2
Os03g0207800	0	0	0	0	0	0	0	0	0.69	0	1.096	0.941	2.177	0.9335	0.792	0	0	1.036	0	0	0	2.958	1.425	1.444	0	0	0	0	0	0	9
Os03g0193400	33.6	16.8	39.9	10.67	6.328	42.39	3.91	4.43	0.69	6.897	28.51	11.29	3.2656	14.002	21.383	0	24.73	22.79	9.29	8.063	7.76	8.873	4.276	5.056	36.97	19.8	32.89	9.25	7.65	15.8	19
Os03g0389100	204	161	126	115.8	131.1	116.8	142	144	126	175.9	152.4	244.6	75.108	154.02	59.397	95.53	115.4	148.2	105.9	41.93	53.46	79.85	87.65	67.17	157.4	255.5	403.5	46.3	103	105	11
Os03g0610800	368	315	282	263.5	242.3	282	230	276	217	342.3	303.7	482.6	251.45	349.11	260.56	406	238.1	353.3	257.3	164.5	215.6	278.7	228	191.4	311.6	371.5	557	228	294	297	20
Os03g0825800	29.6	26.1	24.8	31.2	24.41	21.63	83.8	67.7	81.5	48.28	50.43	63.03	26.124	42.005	44.35	35.82	26.79	22.79	26.94	37.9	33.63	55.45	76.25	63.56	46.48	38.66	48.24	25.9	43	28.3	18
Os03g0690500	105	101	89.4	132.2	131.1	102.9	261	259	212	133.6	118.4	163.7	115.38	112.95	95.036	167.2	71.11	79.77	121.7	104.8	96.57	113.9	114.7	143.7	78.17	70.71	109.6	67.5	71.7	89.1	18
Os03g0753600	199	161	195	292.3	307.3	271.6	229	237	238	148.3	174.3	331.2	193.76	173.62	121.17	489.6	179.3	248.6	389.2	382.2	420.8	224.8	212.4	221	137.3	206.5	199.6	195	188	170	6
Os03g0804900	0	0	0	0	0	2.595	5.48	1.9	0.69	0	0	0.941	0	0	0	0	0	0	0	2.419	0.862	0.739	0.713	0	0	0	0	0	0	0	15
Os03g0237950	16.3	2.79	3.23	19.7	7.232	13.84	31.3	10.1	29	20.69	19.74	27.28	15.239	9.3345	11.088	59.7	7.214	2.072	38.09	17.74	18.11	14.05	18.53	23.11	25.35	12.26	15.35	14.8	10.5	10	6
Os03g0634400	80.5	86.6	123	240.5	279.3	250	9.39	14.5	8.98	150	128.3	149.6	132.8	117.61	105.33	167.2	247.3	136.8	352.1	366.9	437.1	7.394	11.4	13.72	152.1	132.9	146.9	117	109	150	16
Os03g0187525	3.06	0.93	1.08	0	3.616	0	0	0	0	0	0	0	0	0	0.792	0	1.031	2.072	0	0.806	0	0	0.713	2.167	0	0	0	1.85	2.87	0	19
Os03g0744675	32.6	49.4	23.7	21.35	10.85	20.76	1.57	6.96	2.76	6.035	13.16	7.527	10.885	5.6007	10.296	11.94	34.01	22.79	5.574	5.644	13.8	26.62	23.52	11.56	8.451	15.09	19.74	22.2	23	27.5	14
Os03g0108400	540	684	614	550.1	566.8	602.9	1163	975	987	738	640.3	647.3	494.19	658.08	730.98	835.9	586.4	476.6	712.5	628.1	572.5	698.7	728.3	745.4	512.3	513.8	611.8	552	578	587	18
Os03g0563200	3.06	10.2	3.23	5.747	7.232	6.055	6.26	9.49	4.15	3.449	6.578	5.645	2.177	2.8004	4.7518	11.94	3.092	1.036	8.361	3.225	0.862	10.35	4.276	5.778	2.113	2.829	0	7.4	7.65	0.83	6
Os03g0125400	251	212	255	310.3	329	282	370	367	447	298.3	286.2	324.6	234.03	238.96	255.01	250.8	208.2	216.5	252.7	293.5	279.4	360.1	315	404.5	221.8	202.7	210.5	194	215	221	18
Os03g0232000	15.3	13	11.9	14.78	16.27	21.63	17.2	12	20	21.55	14.25	28.22	14.151	20.536	18.215	23.88	11.34	19.68	10.22	16.13	18.97	7.394	13.54	21.67	22.18	17.91	21.93	10.2	13.4	15.8	20
Os03g0860100	0	0.93	0	0	0	0	3.13	2.53	24.9	0	0	0	0	0	0	0	0	0	1.858	0	0.862	1.479	4.276	2.889	0	0	0	0	0	0	18
Os03g0573750	328	349	415	409.7	395	425.6	382	392	372	362.1	442.9	342.5	382.07	478.86	471.22	406	415.3	386.4	343.7	428.9	395.8	465.8	364.2	319.2	380.3	482.7	438.6	511	515	517	1
Os03g0412400	281	201	269	87.02	90.4	73.53	378	244	316	403.5	225.9	252.1	124.09	160.55	127.51	406	206.1	181.3	74.32	112.9	95.71	205.5	229.5	294.7	148.9	95.23	144.7	70.3	95.6	72.5	5
Os03g0293500	3.06	1.86	14	0.821	0	1.73	4.7	0	1.38	0.862	4.386	1.882	2.177	11.201	1.5839	0	2.061	4.144	0.929	0	0.862	27.36	15.68	6.5	6.338	1.886	4.386	9.25	3.83	9.16	17
Os03g0792400	720	879	751	3297	3627	3382	1740	1707	1833	764.8	855.2	1092	759.79	624.48	677.92	764.2	558.5	656.8	2969	3249	3841	1532	1348	1550	637	733.5	543.8	537	562	633	10
Os03g0408500	240	247	224	240.5	271.2	276	377	299	337	303.5	222.6	262.5	260.16	261.37	249.47	346.3	232.9	267.3	388.3	321.7	335.4	279.5	275.1	314.2	281	263.1	241.2	279	240	225	15
Os03g0348800	664	821	880	700.3	724.1	668.7	921	1019	999	557.8	698.4	357.5	603.04	554.47	641.49	1433	613.2	661	505.4	670.8	532.9	1111	1076	851.5	597.9	483.7	403.5	602	747	862	6
Os03g0100040	39.8	68	61.4	147	165.4	122.8	269	263	256	72.42	85.52	64.92	67.488	70.009	71.277	83.59	63.89	56.98	105.9	115.3	95.71	187.8	164.6	216	84.51	66.94	35.09	63.8	81.3	83.3	18
Os03g0277000	2.04	1.86	5.39	0	1.808	1.73	0	0	1.38	0	0	1.882	0	0	0	0	2.061	1.036	1.858	0	0	0	1.425	0	0	0	4.386	0	0.96	0	14
Os03g0346700	640	707	691	596.8	540.6	584.8	731	810	720	690.6	813.5	863.7	776.11	630.08	713.56	1039	723.4	660	634.5	613.6	589.8	861.4	900.1	861.6	807.1	834.4	831.1	740	669	770	6
Os03g0644200	471	446	564	654.3	646.3	590	245	237	210	435.4	479.1	425.3	608.48	473.26	506.86	644.8	484.3	470.4	573.2	634.5	701.8	221.8	201	236.2	474.3	415.8	298.2	446	367	464	16
Os03g0232600	783	854	891	457.3	427.6	376.3	940	926	861	1065	1239	1050	1083.1	980.12	1077.9	1457	960.5	925.2	419	493.4	488	583.4	542.3	697.7	1055	1080	912.3	810	883	903	13
Os03g0788100	1.02	0	0	0.821	0	0	0	1.27	0	0	0	0	0	0	0	0	0	0	0	0	0	1.479	0	0	0	0	0	0	0	0	19
Os03g0665700	35.7	31.7	12.9	33.66	28.93	28.55	3.13	10.1	4.84	75.87	46.05	74.32	52.249	67.209	32.471	95.53	76.26	61.13	26.01	20.16	19.83	2.218	5.701	8.667	114.1	105.6	81.14	85.1	36.3	56.7	3
Os03g0162500	0	0	0	0	0	0	2.35	2.53	0	0	0	0.941	0	0	1.5839	0	0	0	0	0.806	0	0.739	0.713	0.722	0	0	4.386	0	0.96	0	11
Os03g0773150	1399	1091	1216	1260	1256	1432	1320	1077	1346	1294	1207	1739	1309.5	1378.7	1084.2	2376	1057	1206	1465	1346	1524	1193	1085	1197	1274	1380	1618	975	1000	862	6
Os03g0280400	181	448	198	908	772	647.1	138	97.4	237	318.1	399.1	143.9	154.57	133.48	166.31	119.4	123.7	229	117	74.98	233.7	45.1	14.97	13.72	135.2	165.9	96.49	55.5	51.6	46.7	2
Os03g0187500	84.6	60.5	69	64.86	42.49	52.77	102	91.1	111	107.8	58.11	128	83.816	102.68	69.693	119.4	139.1	77.7	104	102.4	131.9	64.33	63.42	98.23	103.5	132.9	142.5	67.5	91.8	68.3	20
Os03g0106550	0	0.93	0	0	0.904	0	0.78	0	0	0	0	0.941	0	0	0	0	0	2.072	0	0	0.862	2.218	0	2.167	0	0	0	0	0	0	9
Os03g0851700	909	855	964	495	481.8	505.2	1082	956	936	1170	1224	1518	1065.7	1132.3	1045.4	1111	1066	950	885.3	870.8	866.5	601.1	672	815.4	1109	1137	1248	816	927	830	20
Os03g0126000	193	199	208	371.9	290.2	325.3	125	222	246	181.9	186.4	185.3	275.4	165.22	261.35	406	184.5	184.4	299.1	295.9	379.4	192.2	141.8	172.6	184.9	221.6	223.7	139	161	156	10
Os03g0295932	0	0	0	1.642	0.904	0	0	0	0	2.587	0	0	0	0	0.792	0	0	0	1.858	0	0	0	0	0	0	0.943	0	0	0	0	7
Os03g0166000	74.4	59.6	61.4	227.4	273.9	215.4	160	134	150	40.52	52.63	49.86	48.983	47.606	24.551	59.7	50.5	37.3	170	134.6	167.3	93.9	93.36	80.89	22.18	37.71	26.32	35.2	22	32.5	10
Os03g0744300	677	618	634	606.7	666.2	582.2	584	571	593	694.1	654.5	776.2	739.11	728.09	701.68	776.2	761.6	673.4	598.2	541	661.3	556	592.2	593.7	687.7	734.5	596.5	680	616	633	3
Os03g0702000	0	0	0	0	0	0	0	0	2.76	0	0	0	0	0	0	0	0	0	0	0	0	2.218	0.713	1.444	0	0	0	0	0	0	9
Os03g0836900	0	0	0	0	0.904	0	0	0	0	0	0	0	0	0	0	0	0	0	0	0	0	0	0	0	0	0	0	0	0	0	2
Os03g0395600	1159	1262	1180	1101	1028	1053	962	1133	1009	1123	1153	1141	1342.1	1142.5	1325	1815	1209	1197	945.7	1008	1017	1118	1217	1132	1326	1350	1204	1277	1230	1305	13
Os03g0684500	61.2	79.2	64.7	61.57	69.61	52.77	50.9	77.8	58	29.31	44.95	40.46	32.656	29.87	44.35	71.65	52.56	49.73	38.09	37.09	24.14	85.03	83.38	72.22	50.71	54.68	35.09	62.9	54.5	40.8	17
Os03g0854500	1404	1445	1478	1726	1685	1545	2550	2548	2579	1982	1881	1854	1724.2	1789.4	1826.3	2281	1706	1659	1760	1777	1911	1906	1995	2131	1914	1870	1785	1671	1667	1605	18
Os03g0692700	2385	2062	1904	1286	1165	1145	667	745	853	1555	1527	1844	1282.3	1441.2	1295.7	2209	1805	1868	1130	1049	954.5	888.7	1111	1075	2176	2214	2035	2360	1977	2130	3
Os03g0662000	189	193	209	125.6	171.8	151.4	143	111	109	200.9	152.4	139.2	152.39	162.42	161.56	358.2	174.2	212.4	132.8	136.3	156.1	149.4	132.6	115.6	133.1	123.5	173.2	233	228	218	13
Os03g0237900	164	144	133	129.7	132	133.2	111	147	165	114.7	175.4	165.6	148.04	148.42	163.14	203	137.1	146.1	144	165.3	174.2	160.4	146.1	147.3	153.2	120.7	186.4	148	134	157	6
Os03g0756800	1483	1661	1476	1538	1828	1568	2227	2044	2305	1626	1742	1722	1600.1	1601.8	1589.5	3117	1376	1381	1413	1288	1242	1758	1620	1676	1493	1459	1460	1185	1113	1312	6
Os03g0299800	45.9	49.4	66.8	64.04	63.28	59.69	55.6	45.5	46.3	81.05	38.37	51.75	53.337	50.406	42.766	107.5	20.61	39.37	50.16	39.51	36.21	33.27	71.98	48.39	30.63	33	32.89	47.2	53.6	18.3	6
Os03g0119000	336	282	308	285.7	320	393.6	395	414	451	343.2	328.9	366.9	286.28	310.84	332.63	441.8	304	306.7	423.6	322.5	362.1	408.1	382	421.1	348.6	322.5	401.3	274	277	297	18

Os03g0101700	2240	2252	2315	2135	1868	2355	510	664	379	976.9	1867	2226	2288.1	1915.4	2089.2	2675	1390	1280	1096	1384	1529	845.9	594.3	449.2	1824	1520	822.3	1373	1607	1808	13
Os03g0113800	448	405	429	341.5	377	464.5	418	370	409	384.5	356.3	398.9	317.85	395.78	385.69	513.5	407.1	374	363.2	426.5	355.2	340.9	329.9	401.6	367.6	333.8	497.8	382	369	381	6
Os03g0372950	39.8	34.5	35.6	47.62	39.77	59.69	69.7	39.8	42.8	26.73	30.7	30.11	18.505	36.405	26.927	59.7	43.28	41.44	68.74	53.21	35.35	31.79	23.52	67.89	15.85	14.14	30.7	23.1	30.6	20	12
Os03g0793600	19.4	19.6	16.2	0.821	5.424	6.055	0.78	1.9	0.69	25	12.06	14.11	9.7967	7.4676	9.5036	23.88	11.34	6.216	4.645	15.32	6.036	10.35	6.414	8.667	14.79	13.2	19.74	23.1	24.9	16.7	13
Os03g0214400	159	247	194	125.6	141.9	122	170	146	140	270.7	282.9	266.3	199.2	285.64	282.73	262.7	233.9	220.7	114.3	113.7	125	176.7	212.4	169.7	289.4	319.6	293.9	273	269	308	3
Os03g0319100	447	586	523	510.6	541.5	517.3	1051	919	937	682	648	624.7	661.82	550.74	681.09	489.6	468.9	502.5	432.9	409.6	436.3	788.2	660.6	768.5	583.1	489.3	521.9	476	430	508	18
Os03g0669900	249	257	248	265.2	238.6	290.7	339	328	304	295.7	262	266.3	287.37	250.17	308.07	334.3	279.3	297.3	335.4	265.3	361.3	269.9	287.2	310.6	238.7	274.4	276.3	206	290	263	15
Os03g0721200	1608	1794	1664	2289	2408	2234	1665	1711	1629	1158	1262	1140	1385.7	1321.8	1195.1	2734	1347	1412	1695	1953	1769	2191	2056	1851	1170	1140	1305	1350	1393	1283	6
Os03g0830900	217	205	172	307.9	235	241.3	701	750	856	274.2	289.4	210.7	227.5	249.23	345.3	238.8	183.4	163.7	214.6	228.2	234.5	712	698.4	680.4	252.5	255.5	355.3	247	228	242	18
Os03g0279200	751	515	567	530.3	593.9	454.2	710	713	607	371.6	406.8	379.2	376.63	247.36	220.96	1469	783.2	634.1	996.8	864.3	958.8	370.4	483.9	499.8	278.9	433.7	326.7	428	543	498	6
Os03g0349600	279	381	311	204.4	198.9	219.7	225	219	207	296.6	376.1	178.8	231.85	198.83	273.23	382.1	205.1	253.8	139.3	150	129.3	244	293.6	245.6	264.1	212.1	219.3	308	316	316	13
Os03g0226400	251	272	211	71.42	90.4	70.07	301	221	190	538	616.2	780.9	229.68	222.16	198.78	191.1	230.8	189.6	43.66	68.53	68.12	166.4	90.5	265.1	414.1	527	486.8	217	216	226	20
Os03g0582300	0	0	0	0	0	0	0	0.63	0.69	2.587	0	0	0	0	0	0	0	0	3.716	3.225	0	0	1.425	0.722	1.056	0	0	0	0	0	16
Os03g0708600	605	553	612	660.9	630.1	618.5	510	535	589	622.5	631.5	636.9	580.18	595.54	567.84	740.3	587.4	579.1	578.7	587.8	593.2	633.7	622.8	579.2	593.7	615.7	635.9	630	645	642	6
Os03g0838500	5.1	4.66	4.31	4.926	4.52	0.865	4.7	6.96	2.07	6.897	3.289	4.704	7.6196	8.4011	5.5438	23.88	4.122	8.288	3.716	3.225	2.587	8.133	5.701	3.611	3.169	0.943	2.193	2.78	8.61	3.33	6
Os03g0453800	771	620	804	289.8	268.5	285.5	276	278	184	535.4	592.1	590.8	693.39	759.83	715.15	1111	737.9	755.3	353	412	395.8	181.9	220.2	205.8	622.2	639.2	598.7	730	727	689	13
Os03g0174600	462	462	477	688	785.5	590.8	321	392	406	448.3	504.3	492	717.33	596.48	597.93	835.9	495.7	547	573.2	615.2	558.7	328.3	341.4	387.8	446.8	397.9	403.5	460	410	486	16
Os03g0241300	0	4.66	0	0	0	1.73	0	0	0	0.862	0	1.882	0	0.9335	3.9598	0	2.061	0	0	0	2.587	0	0	2.167	0	0.943	2.193	0.93	0	0.83	14
Os03g0125900	295	374	269	141.2	140.1	145.3	186	175	186	347.5	429.8	338.7	353.77	364.98	395.98	716.5	421.5	366.8	172.8	230.6	191.4	139.7	211.7	162.5	368.7	430.9	353.1	369	333	381	13
Os03g0306302	17.3	25.1	18.3	17.24	17.18	24.22	6.26	10.1	9.67	3.449	3.289	2.822	18.505	7.4676	12.671	47.76	7.214	16.58	4.645	8.063	9.484	24.4	16.39	11.56	16.9	6.6	4.386	25	23.9	24.2	8
Os03g0323800	729	657	619	761.9	796.4	805.4	921	698	797	600.1	644.7	593.7	689.03	716.89	628.03	1612	645.1	644.4	763.6	761.9	717.4	763.8	602.9	751.1	604.2	626	587.7	581	616	619	6
Os03g0117050	3.06	7.45	7.54	4.105	1.808	2.595	7.05	5.69	9.67	6.897	1.096	6.586	7.6196	2.8004	11.879	0	6.183	5.18	11.15	2.419	0.862	4.436	7.126	7.222	6.338	6.6	17.54	11.1	6.69	3.33	11
Os03g0317300	3.06	0.93	1.08	14.78	7.232	9.516	8.61	15.2	24.2	1.724	4.386	5.645	0	4.6673	5.5438	11.94	4.122	1.036	6.503	7.257	2.587	66.54	84.09	40.45	3.169	0.943	2.193	2.78	0.96	0.83	17
Os03g0767700	8.16	3.73	0	88.67	123.8	68.34	75.2	58.8	38.7	0	0	3.763	1.0885	0.9335	0.792	0	2.061	4.144	52.95	77.4	80.19	20.7	20.67	36.83	0	0	0	0.93	0	0.83	10
Os03g0331600	3237	2972	3620	4423	4275	3955	7217	6889	7169	4283	4740	5365	4894	3858.9	3829.9	6066	3840	4160	6202	6472	6710	3812	4109	5569	4271	3904	3678	3348	3519	3511	15
Os03g0286200	1886	1376	1796	2778	2845	3194	492	584	636	1676	1589	1711	2493.8	2185.2	2036.1	3582	1924	1995	3592	3750	4149	581.2	540.9	546.7	1966	1922	1682	1751	1765	1590	16
Os03g0432666	37.7	33.5	32.3	52.54	50.62	51.04	28.2	28.5	20.7	26.73	29.6	10.35	38.098	29.87	26.135	11.94	34.01	30.05	16.72	11.29	20.69	56.93	50.6	23.11	31.69	21.69	24.12	24.1	23.9	36.7	2
Os03g0774300	2337	2382	2393	1383	1147	1526	994	1185	1173	2535	2918	2654	2549.3	2489.5	2855.8	3702	2313	2493	1447	1297	1590	1066	1009	1065	2436	2521	2322	2262	2576	2557	13
Os03g0749900	153	152	170	188	195.3	214.5	150	136	134	150	148	155.2	182.87	149.35	167.1	298.5	144.3	186.5	196.9	199.2	207.8	123.5	149.7	165.4	180.6	206.5	153.5	164	160	170	6
Os03g0626600	156	157	153	64.86	88.59	75.26	180	132	144	123.3	120.6	105.4	180.69	164.29	181.36	238.8	137.1	141.9	73.39	82.24	81.05	153.8	194.5	117	128.9	95.23	105.3	160	152	157	13
Os03g0689544	1.02	0	0	0	0	0	0	0	0	0	0	0	0	0	0	0	0	0	0	0	0	0.739	0	0	0	0.943	0	0	0	0.83	19
Os03g0187700	633	530	506	215.9	211.5	239.6	378	397	350	341.4	297.1	249.3	268.86	254.83	289.86	561.2	432.8	400.9	265.7	237.9	265.6	299.5	334.2	337.3	278.9	281	274.1	249	262	233	14
Os03g0355800	87.7	115	78.7	278.3	323.6	291.5	186	226	193	56.9	53.72	39.51	54.426	86.811	57.814	107.5	68.02	79.77	255.5	258	283.7	189.3	191	220.3	53.87	49.03	54.82	59.2	70.8	60	12
Os03g0113900	0	0	0	1.642	0	0.865	13.3	3.8	4.84	2.587	0	0.941	0	0	0	0	0	0	1.858	4.838	1.724	11.09	11.4	15.89	1.056	0	0	0.93	1.91	1.67	9
Os03g0701600	714	711	716	654.3	631.9	573.5	902	872	827	754.4	832.2	795.9	761.96	802.77	826.81	1027	734.8	731.4	617.8	631.3	652.7	722.4	706.2	738.9	803.9	852.3	831.1	768	743	886	5
Os03g0694900	1272	1287	1187	1438	1616	1497	1120	1197	1230	1053	1033	1101	1076.5	1185.5	1060.4	1660	1057	1156	1343	1235	1310	1261	1286	1118	987.7	1194	1011	1020	1080	1076	10
Os03g0724600	16.3	6.52	10.8	6.568	6.328	6.92	5.48	1.9	2.76	21.55	6.578	22.58	5.4426	6.5342	3.9598	23.88	2.061	4.144	5.574	5.644	8.622	0.739	1.425	3.611	11.62	7.543	8.772	5.55	8.61	2.5	20
Os03g0333100	565	522	573	864.5	924.8	840	351	381	326	405.2	365.1	493.9	576.92	492.86	419.74	859.7	508.1	547	689.3	805.5	704.4	385.2	478.9	389.3	326.4	443.1	392.5	421	534	488	10
Os03g0326200	7.14	5.59	12.9	15.6	8.136	3.46	8.61	10.1	4.84	10.35	1.096	3.763	6.5311	2.8004	7.1277	0	7.214	7.252	8.361	5.644	0.862	22.18	27.79	13	8.451	3.771	6.579	9.25	7.65	10	17
Os03g0189600	1015	1105	985	1493	1257	881.5	1692	2248	1825	850.1	928.6	656.7	704.27	845.71	919.47	1087	1116	944.9	916.9	592.6	590.6	669.9	1350	893.4	996.2	959.8	995.6	1108	974	1051	18
Os03g0312500	0	0	0	0.821	0	0	0	0	0	0	0	0	0	0	0	0	0	0	0	0	0	0	0	0	0	0	0	0	0	0.83	2
Os03g0105401	88.7	84.8	97	161.7	201.6	154.8	186	188	162	128.5	141.4	137.4	149.13	142.82	133.84	203	102	150.2	184.9	241.9	238	139.7	147.5	138.7	169	173.5	214.9	139	131	140	10
Os03g0367200	10.2	14	12.9	3.284	12.66	3.46	6.26	7.59	5.53	26.73	18.64	26.34	19.593	14.002	11.879	11.94	6.183	8.288	5.574	5.644	2.587	5.915	9.264	5.056	13.73	9.428	19.74	14.8	9.56	5.83	20
Os03g0163900	0	0	0	0	0	0	0.78	0	0	1.724	0	0	0	0	0	0	0	0	0	0	0	0	0	0	0	0	4.386	0	0	0	11
Os03g0411300	79.5	103	76.5	1267	1291	1500	113	115	130	106	114	77.15	102.32	88.678	57.022	83.59	65.95	92.21	1704	1427	1666	136	111.2	150.2	50.71	64.11	105.3	80.5	93.7	95	10
Os03g0352300	600	564	534	1339	1379	1302	769	652	815	645.8	557	902.2	653.11	763.56	421.33	931.4	455.5	556.4	1379	1254	1261	659.5	657	576.4	427.8	611.9	537.3	426	531	358	10
Os03g0339700	24.5	22.4	12.9	21.35	22.6	36.33	1.57	4.43	3.46	27.59	28.51	27.28	10.885	23.336	15.839	23.88	24.73	17.61	46.45	59.66	77.6	8.133	6.414	2.889	23.24	21.69	32.89	19.4	18.2	17.5	16
Os03g0359700	648	686	680	648.6	675.3	637.5	819	827	951	706.1	747.7	626.6	753.26	758.9	893.34	823.9	709	633	636.3	630.5	631.1	944.9	870.1	764.1	695.1	656.2	763.1	743	709	791	5
Os03g0826700	362	340	384	628.9	662.6	636.7	561	514	502	425.1	319.1	391.4	403.84	432.19	420.53	429.9	357.6	390.6	701.4	766.8	658.7	430.3	498.8	464.4	399.3	360.2	339.9	368	363	394	10
Os03g0279816	5.1	7.45	6.47	10.67	21.7	9.516	23.5	18.3	10.4	4.311	3.289	1.882	1.0885	4.6673	9.5036	0	5.153	4.144	16.72	9.675	13.8	10.35	11.4	9.389	5.282	5.657	8.772	2.78	10.5	2.5	12
Os03g0259300	696	698	659	636.3	699.7	614.2	601	555	654	646.6	700.6	689.6	665.09	626.35	673.96	680.6	620.4	623.7	606.6	579.7	543.2	614.4	573	719.4	614.8	673.2	609.6	630	625	701	14
Os03g0799400	0	0	0	0	0	0.865	0	0	0	0	0	0	0	0	0	0	0	0	0	0	0	0	0	0	0	0	0	0	0	0	2
Os03g0274700	177	132	147	194.6	213.3	137.5	125	161	133	144	116.2	120.4	132.8	141.88	102.16	155.2	150.5	136.8	162.6	191.9	169	108.7	100.5	130.7	105.6	150.9	151.3	130	125	119	10
Os03g0259700	404	360	398	276.7	206.1	231.8	445	467	437	325	369.5	302	392.96	317.37	380.94	477.6	408.1	369.9	224.8	236.2	225	356.4	333.5	389.3	335.9	363	307	311	327	316	5
Os03g0361500	3.06	0	0	0	0	0.865	0	0	0.69	0.862	0	0	3.2656	0	0.792	11.94	0	1.036	0	0	0	0.739	1.425	0.722	0	0	0	0.93	0.96	0	6
Os03g0308600	1.02	2.79	0	0	0.904	1.73	1.57	0.63	4.84	0	0	0	0	1.8669	0.792	0	1.031	2.072	0.929	2.419	1.724	4.436	2.851	1.444	3.169	2.829	0	0	4.78	0	9
Os03g0377600	6.12	10.2	6.47	6.568	3.616	10.38	3.13	6.33	5.53	2.587	8.771	8.467	4.3541	10.268	3.1679	0	8.244	9.324	3.716	2.419	4.311	5.915	3.563	8.667	9.507	8.486	13.16	6.48	5.74	10	11
Os03g0399000	3.06	0	2.16	0	0	0	0.78	0.63	0	0	0	0	0	0	0	0	0	0	5.574	3.225	0	1.479	1.425	1.444	0	0	0	1.85	0	0	19
Os03g0807200	251	217	234	274.2	263.1	286.3	187	176	189	179.3	209.4	184.4	216.62	210.03	236.8	370.2	224.7	222.7	268.5	276.6	295.7	244.7	209.5	210.9	248.2	214	236.8	206	227	195	6
Os03g0400800	82.6	106	88.4	51.72	47.01	52.77	25.8	59.5	94.7	64.66	61.4	39.51	46.806	62.541	66.525	143.3	52.56	84.96	22.29	41.93	35.35	205.5	215.2	116.3	86.62	98.06	92.1	138	73.6	113	17
Os03g0190800	347	353	378	394.9	399.6	338.2	472	602	486	420.7	366.2	295.4	317.85	286.57	334.21	656.8	379.2	340.9	399.4	426.5	419.9	397.1	476	503.4	345.4	352.6	403.5	343	417	376	6
Os03g0191200	113	115	117	73.89	64.18	119.4	130	114	123	111.2	108.5	138.3	78.373	115.75	71.277	59.7	144.3	114	114.3	127.4	193.1	110.2	146.8	125.7	137.3	150.9	155.7	141	97.5	110	11
Os03g0837300	289	221	284	110.8	106.7	131.5	283	226	283	438.9	412.2	590.8	427.79	553.54	380.14	310.5	354.5	290.1	247.1	190.3	230.2	108.7	105.5	185.6	396.1	494	524.1	293	322	256	20
Os03g0802800	754	741	748	698.6	708.7	640.1	750	791	809	789.8	872.7	672.7	935.04	916.65	962.24	1015	885.2	847.5	686.5	660.3	671.7	750.5	739	699.1	824	787.3	708.3	880	887	842	13
Os03g0157900	396	352	402	813.6	761.1	716.3	355	378	951	481.1	364	404.6	411.46	503.13	547.25	871.7	426.6	383.3	775.7	662	638	328.3	409.8	290.3	334.9	393.2	528.5	370	519	424	10
Os03g0750500	15.3	14.9	6.47	0	0	5.19	1.57	0	2.07	5.173	9.868	6.586	9.7967	7.4676	15.047	11.94	20.61	11.4	3.716	2.419	8.622	0.739	0.713	0	7.395	9.428	17.54	7.4	14.3	10	3
Os03g0777700	923	1156	961	1218	1117	1013	1027	1302	1084	1110	1124	1009	1111.4	1010	1241	1576	947.1	1038	805.4	805.5	843.2	1196	1215	1075	1292	1138	910.1	1118	1041	1221	6
Os03g0167500	422	377	406	470.4	523.4	467.1	563	607	638	448.3	453.9	496.8	488.75	437.79	448.25	346.3	392.6	410.3	416.2	463.6	519.9	468	436.8	537.4	487	465.8	396.9	357	368	395	18
Os03g0184100	1.02	2.79	2.16	4035	4390	4374	316	329	290	0	1.096	0	0	0	1.5839	0	1.031	0	4766	4832	5574	382.3	362	323.6	0	2.829	0	3.7	3.83	0	10
Os03g0127900	2163	1926	2262	754.5	624.6	816.6	358	320	366	2138	1922	1836	1866.8	1918.2	2193	2484	2478	2324	1090	940.9	982.1	337.9	477.5	416.7	1949	2235	2379	2054	2294	2176	3
Os03g0281000	4052	3853	3622	1763	1985	1825	2436	3030	2864	2516	2538	1878	2746.3	2511.9	3107.7	4406	2734	3368	1475	1398	1476	2924	3163	2708	2570	2466	2068	2754	2578	2798	14
Os03g0312600	532	512	547	682.2	743.1	725.8	485	541	511	599.2	538.3	656.7	692.3	527.4	590.01	788.1	580.2	560.5	740.4	650.7	652.7	494.6	555.9	441.3	588.4	604.4	600.9	546	533	560	16
Os03g0587100	380	445	433	494.2	475.5	430.8	568	703	650	359.5	452.8	383.9	350.5	398.58	433.21	453.8	430.8	472.4	444	494.3	455.3	675.8	526.6	635.6	360.2	422.4	453.9	469	525	481	18
Os03g0180700	311	251	254	227.4	260.3	275.1	294	287	283	277.6	299.3	229.6	224.24	263.23	293.03	370.2	282.4	306.7	239.7	276.6	225.9	290.6	274.4	264.3	243	256.5	250	316	299	300	6
Os03g0207250	137	120	133	242.2	296.5	288.1	225	178	220	150	141.4	195.7	142.6	150.29	117.21	107.5	128.8	118.1	360.4	312	323.3	156.7	166	161.1	144.7	156.5	162.3	96.2	123	104	10
Os03g0806400	63.2	46.6	53.9	102.6	119.3	134.1	65.8	61.4	66.3	31.9	17.54	34.81	39.187	63.475	34.055	119.4	42.25	44.55	174.6	120.9	153.5	68.02	83.38	65.72	30.63	54.68	37.28	59.2	56.4	31.7	10
Os03g0776900	324	226	322	166.7	208.8	178.2	186	198	203	381.1	348.7	423.4	654.2	535.8	548.04	597	333.9	267.3	249.9	264.5	255.2	133.1	181.7	153.8	404.6	354.5	414.5	283	354	246	13
Os03g0648300	166	151	105	131.4	101.2	131.5	260	204	170	140.5	118.4	119.5	123	139.08	117.21	214.9	135	149.2	135.6	116.1	168.1	184.8	215.9	179.1	134.2	137.7	199.6	138	132	134	18
Os03g0179750	1.02	3.73	0	2.463	0	1.73	6.26	0	0.69	0.862	0	0	1.0885	0.9335	3.1679	11.94	1.031	2.072	0.929	8.869	6.036	5.915	4.988	3.611	4.225	1.886	4.386	4.63	7.65	1.67	6
Os03g0277500	0	3.73	0	0	0	0	1.57	0	0.69	0	0	0	1.0885	0	0	0	0	0	0	0	0.862	0	1.425	1.444	0	0.943	0	0	0.96	2.5	14
Os03g0116300	1068	700	917	242.2	165.4	286.3	60.3	72.1	55.3	438	404.6	280.4	361.39	404.18	475.97	823.9	674	638.2	304.7	412.8	273.3	156	245.9	192.1	396.1	386.6	497.8	457	554	452	13
Os03g0223400	247	177	139	97.7	65.09	93.43	57.2	68.9	132	73.29	63.59	128	70.754	158.69	135.43	226.9	142.2	247.6	80.82	36.28	16.38	154.5	190.3	117	94.02	283.8	263.2	142	155	166	13
Os03g0736500	2.04	3.73	1.08	0	1.808	0	0	0	0	0	0	8.467	0	0	0	0	1.031	0	1.858	0	0	0.739	0	0	0	0	2.193	0	0	0	20
Os03g0438900	3.06	0.93	2.16	0.821	0	1.73	3.13	2.53	2.07	0	3.289	1.882	1.0885	0.9335	0	0	0	2.072	3.716	6.45	1.724	0.739	0.713	2.167	5.282	6.6	0	0	2.87	4.17	16
Os03g0786500	98.9	95.9	84.1	94.41	64.18	89.96	33.7	27.8	17.3	90.53	93.19	128	79.462	85.878	100.58	334.3	102	93.24	94.75	89.5	142.3	22.92	27.08	22.39	113	116.9	136	63.8	72.7	75.8	13
Os03g0780800	10.2	10.2	11.9	33.66	30.73	28.55	36	37.3	40.1	6.035	20.83	16.93	6.5311	2.8004	7.1277	0	6.183	3.108	19.51	25.8	24.14	13.31	10.69	23.83	8.451	11.31	2.193	1.85	15.3	6.66	12
Os03g0822700	2131	1474	1838	366.2	326.3	407.4	328	345	456	1046	843.1	972.8	1064.6	1018.4	1082.6	1242	984.2	882.7	393.9	352.3	425.1	356.4	403.4	355.3	911.6	844.8	940.8	686	676	648	14
Os03g0358000	1209	1230	1199	1184	1181	1145	799	887	837	1067	1020	842	1177.8	1008.1	1281.4	1863	1180	1282	801.7	906.3	770	1116	1107	1075	1117	957.9	782.9	1305	1209	1544	8
Os03g0393700	182	200	154	198.7	217	157.4	208	142	196	213.8	222.6	260.6	112.12	142.82	135.43	155.2	126.8	131.6	121.7	136.3	128.5	316.5	327.1	346.7	148.9	191.4	219.3	150	181	122	9
Os03g0729100	3528	3028	4315	2290	1996	2369	1664	1427	1573	5071	5117	6027	5994.5	5892.9	5798.8	9039	5592	4980	3827	3340	4199	791.1	780.3	1119	4950	5251	4901	4755	5554	4615	13
Os03g0421700	0	0.93	0	0	0.904	1.73	1.57	0	1.38	0.862	0	2.822	2.177	2.8004	1.5839	0	1.031	1.036	0	2.419	0.862	1.479	0.713	2.167	0	0	0	0	0	0	15
Os03g0825850	5.1	1.86	1.08	2.463	3.616	5.19	3.91	4.43	2.76	1.724	6.578	7.527	2.177	0	3.9598	0	9.275	11.4	5.574	7.257	3.449	2.218	4.276	0.722	2.113	9.428	15.35	1.85	4.78	0.83	11
Os03g0667700	329	358	328	362.9	353.5	320.1	337	453	414	313.8	394.7	305.8	322.2	299.64	333.42	477.6	352.4	308.7	310.3	299.9	319.9	411.1	399.1	443.5	371.8	341.3	285.1	355	343	416	6
Os03g0227400	839	839	808	311.1	244.1	344.3	213	249	253	1006	998.8	781.8	424.52	840.11	598.73	1122	649.2	655.8	338.1	382.2	415.6	579.7	715.5	604.5	1161	1362	1491	1091	951	886	3
Os03g0421000	2.04	4.66	0	1.642	2.712	0.865	0	0	0	1.724	0	0.941	4.3541	2.8004	0	0	2.061	1.036	0.929	5.644	5.173	5.176	9.264	0.722	7.395	7.543	0	2.78	7.65	2.5	1
Os03g0405500	689	804	727	223.3	218.8	110.7	348	347	199	911.3	766.4	1075	427.79	574.07	434.79	919.5	362.7	296.3	152.3	96.75	71.56	99.82	181	189.2	407.8	364.9	484.6	243	203	286	20
Os03g0845000	1262	1234	1544	985.2	891.3	968	585	637	661	1274	1246	1252	1308.4	1236.8	1717.8	2221	1364	1340	1176	1105	1052	589.3	595.8	637	1473	1177	1189	1175	1220	1432	13
Os03g0372500	317	364	316	321.8	376.1	362.5	305	298	269	286.2	255.5	216.4	253.63	225.9	290.65	597	291.6	299.4	269.4	262	225	316.5	362	330.8	284.2	237.6	186.4	315	296	364	6
Os03g0356400	111	147	129	87.02	128.4	107.3	265	251	223	175.9	223.7	215.4	163.28	173.62	152.06	203	92.75	131.6	111.5	118.5	149.2	132.4	177.4	174.8	155.3	144.3	208.3	153	107	127	5
Os03g0804000	1.02	2.79	0	0	0	0	0	0	0	0	0	0	0	0	0	0	0	0	0	0	0	0	0	0	0	0.943	0	0	0	0	14
Os03g0637600	0	0	0	0.821	2.712	1.73	1.57	0	1.38	3.449	0	0.941	0	0	0	0	0	0	0	0	0	0	0	0	0	0	0	0	0	0	7
Os03g0851800	426	512	452	393.2	364.3	359	435	451	546	596.6	648	619.1	557.32	488.2	597.93	955.3	583.3	478.6	436.6	383.8	413.9	456.9	399.1	478.8	634.9	660.9	537.3	561	543	581	13
Os03g0577200	87.7	55	65.7	41.05	63.28	86.5	239	145	201	125	117.3	137.4	87.082	139.08	90.284	95.53	70.08	88.06	122.6	97.56	135.4	96.12	109.7	119.9	112	105.6	171	87	70.8	67.5	15
Os03g0586600	1175	1110	1221	828.4	783.7	761.2	1058	1002	1063	1409	1452	1544	1374.8	1485.1	1241.8	1457	1250	1115	1005	828.1	883.8	845.9	835.2	870.3	1250	1265	1476	1100	985	1040	3
Os03g0283900	23.4	22.4	29.1	19.7	35.25	27.68	25.1	29.7	21.4	19.83	36.18	24.46	14.151	35.471	16.631	47.76	14.43	32.12	33.44	15.32	18.97	24.4	32.07	20.22	21.13	29.23	17.54	19.4	12.4	21.7	6
Os03g0235100	579	539	553	642	596.6	617.6	712	736	639	653.5	624.9	583.3	642.23	712.22	711.98	1445	616.3	576	608.5	638.6	563	539	496	580.7	569.4	528.9	598.7	645	598	582	6
Os03g0795800	627	596	634	872.7	896.7	802.8	1151	1065	1182	700.1	682	620	569.3	648.75	641.49	1743	522.5	572.9	866.7	811.9	842.4	896.1	1004	923	615.9	524.2	627.2	525	586	577	6
Os03g0273200	0	0	0	7.389	6.328	7.785	0.78	4.43	1.38	12.07	6.578	5.645	0	0	0	0	0	0	0	0	0	0	0	0	0	0	0	0	0	0	7
Os03g0854300	233	231	186	274.2	294.7	255.2	306	336	323	216.4	233.5	284.1	260.16	240.83	228.88	310.5	188.6	183.4	298.2	269.3	360.4	246.2	274.4	228.2	215.5	229.1	247.8	192	213	157	12
Os03g0442900	0	0	0	0	0.904	0	0.78	0	0	0	0	0.941	0	0	0	0	0	0	0.929	0	0	0	1.425	0	0	0	0	0.93	0	0	17
Os03g0794700	1028	858	825	1195	1216	1317	2282	1712	2250	1073	1022	1762	1207.2	1174.3	915.51	1719	818.2	877.5	1534	1209	1545	1366	1343	1413	895.8	1190	1307	626	772	591	18
Os03g0647800	58.1	59.6	44.2	81.28	79.55	89.1	72	65.8	52.5	77.6	52.63	73.38	38.098	70.009	27.719	71.65	72.14	48.69	84.53	54.83	93.98	46.58	47.75	75.11	30.63	51.86	74.56	59.2	42.1	25	10
Os03g0199300	0	0	0	0	0	0	0	0	1.38	0	0	0.941	0	0	0	0	0	1.036	0	0	0	0	0	0	0	0	0	0	0	0	20
Os03g0702800	380	344	386	302.1	287.5	254.3	294	412	379	342.3	384.8	331.2	290.63	287.5	324.71	597	368.9	352.3	321.4	302.4	311.3	352.7	388.4	325	326.4	391.3	366.2	320	289	265	6
Os03g0803800	41.8	29.8	26.9	32.84	27.12	18.17	10.2	11.4	4.15	6.035	9.868	6.586	18.505	13.068	13.463	11.94	23.7	26.94	4.645	11.29	13.8	28.84	14.97	10.83	15.85	14.14	6.579	37.9	31.6	34.2	8
Os03g0218400	5983	4716	6074	822.6	602.9	787.2	1841	2591	2776	7045	5655	6351	3599.7	3881.3	4745.5	5911	4419	4991	1277	1404	1003	2095	2216	2150	5021	5774	5601	4305	4955	5075	3
Os03g0769600	682	1042	791	4764	4720	4514	3753	4044	3758	965.6	1229	1464	1114.6	659.02	669.21	823.9	556.5	645.5	4267	5252	5034	3058	3014	3676	815.5	726	686.4	487	575	642	12

Os03g0774600	459	517	528	578	620.1	542.4	736	658	617	518.2	533.9	600.2	457.18	525.53	441.13	429.9	494.7	418.6	605.7	547.5	663.9	498.3	463.9	619	491.2	485.6	598.7	386	382	394	15
Os03g0113500	11.2	6.52	2.16	17.24	10.85	23.36	193	70.2	253	6.897	9.868	6.586	6.5311	10.268	2.3759	35.82	13.4	3.108	25.08	11.29	15.52	298.7	126.1	362.6	10.56	6.6	6.579	7.4	12.4	5	9
Os03g0134800	0	0	0	0	0	0	0	0	0	1.724	0	1.882	0	0	0	0	0	1.036	0	0	0.862	0	1.425	0	0	0.943	0	0	0	0.83	20
Os03g0691500	2.04	0	0	0	0	0	0	0	0	0	0	0	0	0	0	0	0	0	0	0	0	0	0	0	1.056	0	0	0	0	0	19
Os03g0111200	2.04	0	0	1.642	3.616	1.73	0	0	0.69	0	0	0	0	0	0	0	0	0	0	0	0	0.739	0	0	0	0	0	0	0	0	2
Os03g0823700	60.2	50.3	55	47.62	47.01	62.28	84.6	111	77.4	81.05	74.55	120.4	55.515	70.009	68.109	23.88	48.43	36.26	75.25	66.92	54.32	69.5	66.99	73.67	65.49	59.4	76.75	59.2	51.6	43.3	15
Os03g0651900	393	308	393	149.4	167.2	159.2	384	384	361	333.7	418.8	440.3	432.14	372.45	411.82	417.9	426.6	445.5	225.7	205.6	207.8	300.9	245.9	346	413	473.3	396.9	318	307	347	3
Os03g0344900	1591	1906	1746	3125	3433	2894	3366	3665	3614	1930	2480	2228	2514.5	1981.7	2010	2794	1519	1687	2915	3486	2923	2418	2500	3176	1928	1882	1629	1473	1751	1664	12
Os03g0223900	38.7	31.7	23.7	54.18	64.18	33.74	75.9	70.2	58.7	37.07	24.12	36.69	22.859	33.604	26.927	23.88	26.79	27.97	46.45	33.86	53.46	68.02	60.57	67.89	30.63	26.4	43.86	27.8	33.5	48.3	18
Os03g0603600	276	263	282	81.28	78.65	75.26	131	183	102	243.1	151.3	207.9	85.993	152.15	131.47	191.1	211.3	191.7	91.97	93.53	68.12	289.8	473.9	282.4	176.4	177.3	265.3	220	255	118	17
Os03g0301900	0	0	0	0	0	0	0	0	0	0	0	0	0	0	0	0	4.122	0	0.929	0	0	0	0	1.444	0	0	0	0	0	0	1
Os03g0754300	821	623	772	482.7	480.9	481.8	320	392	328	569	686.3	479.8	957.9	869.98	986	1552	856.4	840.2	387.4	462	448.4	512.4	435.4	335.8	849.3	726	699.5	1056	952	1019	13
Os03g0396200	0	0.93	0	1.642	1.808	0	0.78	0	1.38	0	1.096	0	1.0885	0	0	0	0	1.036	0	0	1.724	3.697	2.138	2.167	0	0	0	0	0.96	0	9
Os03g0295800	2343	2214	2612	2273	2245	2247	3714	3482	3476	2567	2513	2698	2903.1	2707	2513.7	4466	2679	2341	2738	2675	2489	2475	2829	2975	2662	2411	2605	2556	2451	2549	6
Os03g0237100	37.7	58.7	75.4	97.7	113	74.39	380	250	231	145.7	143.6	118.5	114.29	143.75	83.948	95.53	45.34	46.62	104	89.5	106.9	132.4	147.5	138.7	97.19	72.6	65.79	61.1	41.1	55	18
Os03g0216600	1288	1231	1138	1288	1347	1401	1376	1337	1388	1230	1155	1182	1128.8	1149.1	1253.7	1552	974.9	1007	1170	1123	1103	1354	1434	1369	1090	1124	1143	1027	1035	1002	18
Os03g0821350	2.04	0	0	0	0	0	0	0	0	0	0	0	0	0	0	0	0	0	0	0	0	0	0	0	0	0	0	0	1.91	0	19
Os03g0746400	208	198	184	335	421.2	341.7	384	379	372	229.3	265.3	229.6	323.29	222.16	275.6	262.7	188.6	206.2	365.1	386.2	331.1	314.2	318.5	292.5	245.1	209.3	179.8	266	183	238	12
Os03g0278700	398	387	431	301.3	306.4	288.9	254	235	281	340.6	398	279.4	483.3	442.46	436.37	609	484.3	467.3	291.7	342.7	330.2	365.3	287.9	304.1	461.6	386.6	383.8	460	461	498	13
Os03g0119900	57.1	44.7	37.7	43.51	31.64	25.09	120	138	119	38.8	42.76	56.45	26.124	29.87	35.638	191.1	104.1	74.59	50.16	58.05	41.39	58.41	82.67	69.34	52.82	58.46	63.59	31.5	59.3	82.5	6
Os03g0128000	13.3	8.38	4.31	13.96	1.808	25.95	2.35	1.27	1.38	11.21	2.193	12.23	3.2656	8.4011	2.3759	23.88	14.43	0	12.08	4.031	9.484	0.739	1.425	12.28	5.282	13.2	83.33	14.8	23.9	3.33	11
Os03g0845600	0	0.93	5.39	0	1.808	0.865	3.13	0.63	0.69	0	0	0	2.177	0	0.792	0	0	3.108	1.858	0.806	0.862	1.479	4.988	0	3.169	3.771	2.193	0.93	0	0	17
Os03g0753500	1508	1730	1475	1945	2051	1694	2439	2421	2279	1865	1682	1628	1458.6	1539.3	1502.4	2902	1893	1811	1984	1790	2060	1548	1767	1556	1511	1811	1526	1538	1710	1627	6
Os03g0719700	8.16	7.45	14	9.852	7.232	13.84	4.7	12.7	4.84	6.035	9.868	2.822	4.3541	13.068	4.7518	0	10.31	6.216	6.503	11.29	16.38	7.394	7.126	9.389	8.451	6.6	4.386	8.33	9.56	8.33	16
Os03g0121700	479	496	578	806.2	915.7	720.6	951	1016	1137	605.3	542.7	731	624.81	444.32	338.17	429.9	418.4	501.4	761.7	699	727.7	791.9	718.3	851.5	507.1	409.2	390.3	313	313	416	12
Os03g0603500	81.6	89.4	88.4	85.38	124.7	145.3	54	39.8	50.4	71.56	67.98	61.15	43.541	52.273	48.31	107.5	81.41	95.32	111.5	107.2	101.7	74.68	87.65	60.67	61.27	64.11	81.14	98.1	68.9	70.8	10
Os03g0669600	138	169	135	136.3	134.7	161.8	146	156	180	135.4	83.33	140.2	133.89	141.88	130.67	238.8	125.7	98.42	139.3	154.8	164.7	156	163.9	173.3	119.4	126.3	160.1	112	109	141	6
Os03g0415700	0	0	0	0.821	1.808	0	1.57	0	0	0.862	0	0	0	0	0	0	0	0	0	0	0	0.739	0	0	0	0	0	0	0	0	2
Os03g0159700	103	79.2	75.4	76.35	78.65	90.83	114	99.3	126	105.2	80.04	57.39	74.019	74.676	58.605	155.2	96.87	53.87	74.32	90.3	92.26	135.3	121.1	114.8	80.28	82.03	96.49	80.5	75.5	87.5	6
Os03g0300600	560	280	478	1827	1837	1763	121	159	119	344.9	364	387.6	428.88	269.77	354.8	632.9	275.2	275.6	2087	2363	2088	166.4	200.3	150.2	419.4	281	368.4	301	290	274	10
Os03g0836400	0	0	0	0	0	0	0	0	0	0	0	0	1.0885	0	0	0	0	0	0	0	0	0	0	0	0	0.943	0	0	0	0	4
Os03g0375300	3.06	7.45	7.54	1.642	2.712	0.865	0	0	0	3.449	7.675	6.586	7.6196	3.7338	9.5036	47.76	7.214	8.288	0	0	2.587	0	0.713	0	8.451	4.714	2.193	10.2	4.78	12.5	13
Os03g0146100	3176	2204	2768	656	375.1	872.8	20.4	20.2	8.29	1692	1613	1514	1900.6	1872.5	1953.8	1469	2119	2088	836.1	785.3	940.7	91.68	93.36	99.67	2266	1885	2673	2175	1713	2257	3
Os03g0643522	3.06	3.73	2.16	5.747	8.136	5.19	35.2	25.9	22.1	5.173	3.289	15.05	5.4426	2.8004	0.792	0	0	4.144	15.79	19.35	11.21	8.873	10.69	12.28	6.338	2.829	6.579	3.7	0.96	1.67	18
Os03g0305950	6.12	1.86	0	3.284	0.904	2.595	0	0.63	0	0	0	0.941	1.0885	0.9335	6.3357	0	2.061	4.144	0.929	0	5.173	0.739	0	1.444	1.056	2.829	2.193	2.78	3.83	0.83	19
Os03g0310600	11.2	13	19.4	5.747	8.136	1.73	9.39	8.22	17.3	12.07	6.578	7.527	7.6196	13.068	15.839	23.88	5.153	4.144	3.716	6.45	4.311	18.48	31.36	17.33	6.338	6.6	4.386	15.7	14.3	8.33	17
Os03g0195500	0	0	0	0	0	0	0	0	0	0	0	0	0	0	0	0	0	0	0	0	0.862	0	0	0	0	0	0	0	0	0	16
Os03g0663100	130	169	131	103.4	115.7	248.3	139	127	159	74.15	55.92	54.57	71.842	162.42	73.653	95.53	144.3	100.5	132.8	154	91.4	168.6	227.3	176.9	80.28	77.31	87.72	91.6	140	102	17
Os03g0196400	151	229	260	238.1	291.1	211.1	958	819	1013	460.4	403.5	148.6	428.88	259.5	483.89	800	137.1	205.1	352.1	437	274.2	451.8	704.8	483.2	218.7	100.9	212.7	261	489	326	18
Os03g0783000	658	630	672	587	563.2	615	452	454	486	676	685.2	608.7	677.06	665.55	806.22	859.7	715.2	715.9	565.7	492.6	551.8	433.3	458.9	424	689.8	654.3	682	730	690	773	13
Os03g0857100	73.4	102	106	215.9	180.8	289.8	675	626	792	181.9	157.9	231.4	265.6	203.49	211.45	179.1	162.8	116	290.8	240.3	348.3	419.2	335.7	535.9	155.3	156.5	212.7	143	147	170	18
Os03g0233000	34.7	23.3	26.9	6.568	4.52	4.325	4.7	6.96	0.69	11.21	13.16	40.46	23.947	20.536	4.7518	59.7	32.98	37.3	13.01	4.838	1.724	1.479	17.1	21.67	14.79	122.6	175.4	45.3	59.3	66.6	11
Os03g0696000	366	307	320	247.9	233.2	231.8	97.9	120	117	307.8	285.1	302.9	317.85	292.17	303.32	871.7	376.1	424.8	301	292.7	239.7	82.81	117.6	92.45	315.9	311.1	348.7	364	377	422	13
Os03g0833500	0	0	0	0	0	0	0	0	0.69	0	0	0	0	0	0	0	0	0	0	0	0	0	0	0	0	0	0	0	0	0	18
Os03g0161400	18.4	22.4	6.47	0.821	0.904	2.595	7.05	0.63	1.38	15.52	28.51	17.88	10.885	12.135	9.5036	35.82	26.79	7.252	0	0	0	1.479	2.851	1.444	15.85	22.63	28.51	9.25	6.69	10	13
Os03g0828500	264	253	246	485.2	507.1	464.5	334	363	299	150.9	159	147.7	272.13	239.9	232.05	489.6	354.5	352.3	484.9	542.6	533.7	343.1	309.3	312.7	173.2	184.8	241.2	316	352	359	10
Os03g0117800	658	696	698	902.3	953.7	819.2	1048	1089	1050	575.1	561.4	666.1	726.04	666.48	605.85	1015	660.6	664.1	955	1044	1040	698	573	981.5	546.1	617.6	535.1	480	530	551	12
Os03g0434400	1.02	5.59	1.08	21.35	24.41	19.9	18	31.6	26.3	1.724	0	0.941	4.3541	0	0	0	0	0	11.15	12.9	8.622	31.79	45.61	20.95	0	1.886	0	2.78	0	0	12
Os03g0448500	828	769	722	695.4	725	686	564	600	594	619	598.6	789.3	649.85	638.48	652.58	1218	810	924.1	549.9	710.3	691.5	962.7	833.1	749	723.6	904.2	785.1	822	837	790	13
Os03g0810600	825	867	861	991.7	1095	933.4	941	918	946	769.1	775.2	757.4	824.01	792.5	832.36	1290	945	783.2	919.7	938.5	1014	942.7	862.3	871	779.6	894.8	857.4	735	737	735	6
Os03g0200700	116	138	105	130.5	167.2	134.9	211	183	203	166.4	144.7	135.5	87.082	123.22	156.81	179.1	107.2	97.39	111.5	133.8	153.5	216.6	186.7	167.6	120.4	127.3	105.3	141	129	127	18
Os03g0721400	139	149	135	188	172.7	160.9	319	286	361	229.3	220.4	156.2	157.84	183.89	193.24	203	121.6	133.6	213.7	183	212.1	218.1	211.7	220.3	162.7	170.7	162.3	139	174	130	18
Os03g0342400	137	88.5	154	16.42	14.46	11.25	43.8	34.2	57.4	93.12	82.23	115.7	94.701	42.005	78.405	107.5	139.1	150.2	24.15	25.8	27.59	42.15	59.86	83.78	108.8	107.5	96.49	40.7	62.2	101	3
Os03g0830600	0	0	0	0	0	0	0	0	2.07	0	0	0	0	0	0	0	0	0	0	0	0	0	0	0	0	0	0	0	0	0	18
Os03g0294650	2.04	9.31	1.08	6.568	9.944	5.19	1.57	3.16	1.38	0.862	1.096	0	1.0885	0.9335	2.3759	0	1.031	4.144	0	3.225	2.587	1.479	7.126	4.333	5.282	5.657	6.579	3.7	2.87	5.83	2
Os03g0132000	735	591	545	279.1	240.5	292.4	441	512	649	665.6	597.5	644.5	391.87	462.06	516.36	788.1	492.6	494.2	302.8	337	318.2	505	472.5	461.5	503.9	478	675.4	382	357	431	13
Os03g0221300	493	547	483	688	682.5	641.9	562	646	590	544	578.9	387.6	546.44	551.67	632.78	752.3	568.9	543.9	675.3	652.3	630.3	559.7	596.5	584.3	487	551.6	359.6	595	552	616	10
Os03g0388900	4613	3556	3705	1712	1600	2150	968	1201	1710	3256	3730	7783	2365.4	3315.6	2520.8	4442	5246	4426	3434	2452	2979	1082	1089	1330	5281	5901	6289	2840	2963	2586	20
Os03g0210000	3.06	8.38	5.39	6.568	5.424	0.865	19.6	26.6	17.3	6.897	17.54	6.586	6.5311	11.201	17.423	0	9.275	3.108	0.929	0	0	2.958	4.276	7.222	6.338	7.543	2.193	0	0.96	7.5	5
Os03g0799100	685	646	692	416.2	462.8	431.7	622	674	600	674.2	706.1	682.1	727.13	691.69	677.92	1516	820.3	696.2	481.2	402.3	446.6	465.1	470.3	496.9	694	668.5	616.2	715	742	716	13
Os03g0219300	1135	1145	1072	693.7	830.7	774.2	729	652	692	1047	1026	907	1026.5	936.25	917.89	991.1	955.3	1064	566.7	554.7	663	662.5	646.4	587.9	1149	899.5	853	870	910	1123	3
Os03g0794200	168	118	151	31.2	19.89	38.06	72	65.1	114	180.2	171	199.5	136.06	194.16	173.44	262.7	136	118.1	59.45	48.38	68.12	86.51	76.96	81.61	177.5	219.7	175.4	146	121	182	3
Os03g0203000	313	326	345	395.7	366.1	385.8	214	265	264	234.5	292.7	313.3	378.8	336.98	327.87	274.6	256.6	299.4	329.8	347.5	298.3	224.8	240.2	184.9	265.1	281	217.1	306	316	315	2
Os03g0289300	2.04	3.73	0	2.463	1.808	2.595	1.57	1.9	8.98	0	1.096	0.941	3.2656	0	2.3759	23.88	2.061	1.036	1.858	3.225	2.587	2.218	1.425	5.056	2.113	0	4.386	0.93	0.96	2.5	6
Os03g0738200	443	482	446	402.3	460.1	441.2	330	400	355	460.4	471.4	310.5	537.73	512.46	574.18	835.9	453.4	453.8	302.8	392.7	373.3	428.1	459.6	386.4	484.9	446.9	392.5	549	481	557	13
Os03g0263800	821	809	794	621.5	655.4	692.9	1018	941	1066	1034	1045	1091	916.53	1097.7	1028	1230	966.6	908.6	765.5	691	805.3	885.8	873.7	914.4	1095	1208	1243	903	934	906	5
Os03g0800400	69.3	76.4	43.1	100.2	113.9	39.79	135	184	94.7	75.87	69.07	46.1	66.4	55.074	84.74	226.9	110.3	137.8	121.7	99.17	81.05	138.3	171	95.34	96.13	84.86	67.98	112	88	141	6
Os03g0705300	249	389	267	297.2	273	311.4	319	331	319	359.5	320.1	306.7	280.84	263.23	280.36	406	379.2	303.6	233.2	270.1	243.1	376.3	398.4	386.4	328.5	325.3	328.9	328	341	319	9
Os03g0150200	2.04	3.73	1.08	0.821	4.52	0.865	0	0	1.38	2.587	9.868	1.882	0	2.8004	11.088	0	11.34	22.79	3.716	4.031	2.587	1.479	0.713	1.444	32.75	12.26	10.96	4.63	1.91	15.8	1
Os03g0178100	619	657	717	110.8	75.03	92.56	60.3	104	71.2	438.9	626	375.4	645.49	596.48	794.34	609	569.9	663.1	43.66	46.76	51.73	144.2	150.4	148.8	572.5	620.4	410.1	601	551	738	3
Os03g0674600	53	59.6	62.5	33.66	39.77	41.52	21.9	27.2	22.8	52.59	58.11	53.63	62.046	50.406	75.237	95.53	71.11	81.85	32.51	35.48	38.8	43.62	45.61	41.17	61.27	88.63	63.59	99.9	92.8	105	13
Os03g0407900	10.2	38.2	20.5	106.7	115.7	112.5	36	53.1	34.6	5.173	15.35	13.17	17.416	21.469	21.383	23.88	11.34	16.58	65.96	75.79	51.73	110.9	204.5	88.84	15.85	16.97	13.16	13.9	17.2	20	17
Os03g0160400	77.5	79.2	77.6	102.6	102.1	101.2	70.5	50.6	64.3	43.97	41.66	36.69	32.656	40.138	36.43	83.59	90.69	97.39	82.68	60.47	93.12	114.6	100.5	91	49.65	40.54	52.63	59.2	106	62.5	2
Os03g0403100	312	345	338	564	598.4	558	339	368	409	273.3	275.2	244.6	321.11	358.45	296.2	501.5	253.5	318.1	407.8	445.9	494.1	519.8	490.3	395.1	275.7	280	287.3	343	371	355	10
Os03g0293700	1.02	3.73	1.08	0	0	0	1.57	0	0.69	3.449	1.096	0.941	0	3.7338	3.1679	0	2.061	1.036	0	1.613	0	0	0	0	0	0	0	0	0.96	0.83	7
Os03g0162900	172	170	202	263.5	290.2	226.6	126	173	144	119.8	194.1	132.7	154.57	123.22	149.68	226.9	156.6	177.2	253.6	241.1	224.2	145.7	141.1	147.3	179.6	133.9	120.6	134	150	175	10
Os03g0764800	1009	1052	974	1006	1039	905.7	1138	1131	1104	1035	1097	1141	1169.1	1032.4	1166.6	1302	855.3	937.6	866.7	832.1	822.6	998.9	989.1	1031	1024	1025	1009	971	965	1072	5
Os03g0818800	27.5	24.2	31.2	128.1	122	123.7	95.5	199	115	17.24	18.64	19.76	22.859	17.736	41.182	59.7	26.79	36.26	72.46	74.18	41.39	111.6	173.2	96.78	23.24	24.51	35.09	19.4	29.6	25.8	12
Os03g0261500	2855	2547	2929	2038	2122	2189	2807	2730	2624	3369	3562	3555	4267	4011	3839.5	4215	3052	2562	2610	2574	2230	1726	2111	2212	3553	3201	3298	3232	2976	3106	3
Os03g0765500	0	0	0	0	0	0	0.78	0	1.38	0	0	0	0	0	0	0	0	0	0	0	0	0.739	1.425	0.722	0	0	0	0	0	0	17
Os03g0108500	0	1.86	1.08	0	0.904	1.73	0.78	3.16	0	0	5.482	0.941	0	0	0	0	4.122	4.144	0	2.419	0	0	0.713	0	1.056	0	6.579	0.93	0	0	11
Os03g0277300	88.7	41	88.4	6.568	0.904	16.44	2.35	7.59	1.38	98.29	71.27	122.3	68.577	47.606	53.854	83.59	66.98	69.41	6.503	6.45	4.311	5.915	14.97	5.778	116.2	69.77	138.2	123	41.1	49.2	3
Os03g0618300	1456	1333	1474	270.9	285.7	144.5	420	411	382	1311	1452	1609	1477.1	1687.7	1617.2	1242	1666	1433	602	340.3	352.6	190	224.5	254.2	1689	1529	1958	1212	1430	1473	3
Os03g0770000	3.06	0	0	0.821	0	0	0	0	0	2.587	0	0	0	0	0	0	0	0	0	0	0	0	0	0	0	0	0	0	0	0	19
Os03g0137500	374	365	384	352.2	346.2	390.1	600	539	619	446.6	467.1	484.5	363.57	364.05	376.18	597	385.4	345	400.4	362	337.1	564.2	502.4	547.5	361.3	375.3	383.8	352	361	328	18
Os03g0788000	4.08	6.52	3.23	4.926	5.424	3.46	12.5	8.86	2.07	2.587	4.386	0.941	0	2.8004	2.3759	0	11.34	5.18	12.08	9.675	5.173	11.83	17.1	11.56	3.169	4.714	2.193	1.85	3.83	1.67	17
Os03g0355400	0	0	0	0	1.808	0	0.78	0	0	0	0	0	0	0.9335	0	0	0	0	0	0.806	0.862	0	0	0	0	0	0	0	0	0	10
Os03g0596600	2.04	0	0	0	0.904	0	4.7	2.53	0	0	0	0	0	0.9335	0	0	0	0	0	0	0	0	0	0	0	0	0	0	0	0	5
Os03g0803066	4.08	3.73	1.08	0.821	0.904	0	0.78	0	0.69	16.38	16.45	2.822	30.479	26.137	33.263	0	3.092	0	0.929	0	0	0.739	0	0	16.9	5.657	2.193	2.78	4.78	2.5	4
Os03g0611700	245	234	214	229.1	290.2	254.3	325	282	301	272.5	278.5	262.5	246.01	244.56	247.09	489.6	215.4	292.2	299.1	295.9	300.1	277.3	235.9	257.8	244	258.3	258.8	208	269	245	6
Os03g0759500	8.16	4.66	6.47	25.45	18.08	11.25	5.48	10.1	8.98	11.21	4.386	9.408	10.885	11.201	7.1277	0	0	12.43	10.22	13.71	15.52	8.133	9.977	11.56	2.113	8.486	8.772	4.63	7.65	6.66	10
Os03g0654400	411	470	377	296.4	299.2	250.9	547	452	489	338	497.8	281.3	273.22	311.77	389.65	513.5	402.9	365.7	370.7	281.4	328.5	422.2	363.4	467.3	314.8	369.6	557	359	391	377	5
Os03g0167000	0	0	0	0	0	0	0	0	0.69	0	0	0	0	0	0	0	0	0	0	0.806	0	0	0	0.722	0	0	0	0.93	0	0	9
Os03g0123800	420	397	311	114.9	71.41	77.85	59.5	68.3	53.9	292.3	275.2	196.6	152.39	128.82	217.79	394.1	223.6	213.4	156.1	210.4	160.4	108.7	106.9	73.67	260.9	222.5	245.6	172	217	197	13
Os03g0854200	138	106	110	228.2	237.7	186	120	113	137	128.5	129.4	179.7	167.63	157.75	110.08	226.9	150.5	127.4	191.4	237	234.5	108.7	115.4	111.2	109.9	130.1	157.9	132	125	115	10
Os03g0179400	87.7	106	147	57.47	56.95	58.82	47	70.8	52.5	84.49	83.33	58.33	62.046	78.41	90.284	214.9	80.38	78.74	53.88	41.93	32.76	112.4	155.4	110.5	115.1	77.31	111.8	150	116	132	13
Os03g0739700	554	565	383	35.3	23.5	155.7	62.6	30.4	173	95.7	75.65	47.98	89.259	47.606	71.277	298.5	340.1	336.7	91.04	112.1	106.1	406.7	171.7	467.3	79.23	92.4	70.17	143	193	114	14
Os03g0221500	1.02	0	0	0.821	0	1.73	0	0	0	0	0	0	1.0885	8.4011	1.5839	0	2.061	0	0	0	3.449	0	0	0	0	0	0	0.93	0	0	4
Os03g0432200	176	182	141	216.7	226	218	238	266	262	144	177.6	156.2	140.42	141.88	121.96	250.8	147.4	192.7	171.9	202.4	176.8	374.9	270.1	301.2	158.5	198.9	214.9	134	165	152	9
Os03g0419200	6.12	5.59	8.62	5.747	8.136	9.516	2.35	0.63	0.69	1.724	1.096	2.822	0	4.6673	0	0	5.153	5.18	6.503	6.45	4.311	5.176	4.988	5.056	0	0	13.16	1.85	0.96	0	11
Os03g0335200	118	124	154	105.9	184.4	34.6	284	402	176	72.42	43.86	49.86	29.39	32.671	48.31	59.7	36.07	37.3	55.74	66.92	12.93	41.41	174.6	56.34	16.9	7.543	26.32	7.4	16.3	29.2	18
Os03g0214200	467	577	503	163.4	153.7	179.1	171	170	194	514.7	544.9	538.1	494.19	539.53	615.36	1039	653.4	606.1	209	296.7	295.7	236.6	285.8	207.3	653.9	604.4	451.7	764	541	692	13
Os03g0379300	361	254	295	91.13	71.41	84.77	82.2	81.6	88.5	263.8	275.2	289.8	260.16	287.5	259.76	394.1	349.4	326.4	92.9	113.7	98.29	84.29	96.21	111.9	233.5	302.7	346.5	224	234	246	3
Os03g0232200	0	0	0	0	0	0	0	0	0	0	0	0	0	0	0	0	0	0	0	0	0	0	0	0	0	0	0	0.93	0	0	1
Os03g0566700	0	2.79	1.08	0	1.808	0	1.57	2.53	1.38	0	0	0	0	0	1.5839	0	0	0	0	0.806	0	3.697	0.713	0	0	0	10.96	0	0.96	0	11
Os03g0205500	276	274	278	282.4	323.6	318.3	301	307	283	241.4	268.6	242.7	265.6	286.57	272.44	609	257.6	255.9	347.4	300.7	320.7	284.7	298.6	294	250.4	225.3	274.1	251	274	294	6
Os03g0850100	20.4	14	11.9	32.02	23.5	50.17	14.9	15.2	26.3	14.66	14.25	15.99	11.974	14.002	15.047	35.82	15.46	11.4	33.44	30.64	32.76	48.06	39.91	28.17	20.07	15.09	15.35	11.1	25.8	14.2	10
Os03g0454100	1081	1024	1101	905.5	992.6	931.7	953	901	859	848.4	970.3	986.9	1011.2	1004.4	897.3	1314	1017	961.4	962.4	942.5	896.7	753.4	806.7	860.9	984.5	955.1	969.3	957	785	1017	13
Os03g0848700	47.9	58.7	39.9	247.9	271.2	238.8	63.4	44.3	53.2	19.83	44.95	36.69	25.036	34.538	24.551	71.65	35.04	22.79	187.6	216.9	188	275.1	114	205.1	44.37	36.77	37.28	50	48.8	67.5	10
Os03g0216000	3.06	6.52	6.47	18.88	22.6	14.71	16.4	27.2	30.4	3.449	9.868	0	3.2656	0.9335	5.5438	11.94	0	6.216	13.01	18.54	12.07	31.79	34.21	23.83	3.169	1.886	2.193	5.55	1.91	10.8	12
Os03g0784100	21.4	13	9.7	17.24	7.232	22.49	48.5	30.4	32.5	23.28	9.868	13.17	10.885	19.602	11.879	0	22.67	14.5	22.29	12.09	25.87	31.79	40.62	46.22	16.9	20.74	30.7	15.7	15.3	10	18
Os03g0572300	458	508	514	598.5	612	505.2	577	652	594	561.3	605.2	645.4	578	520.87	562.3	704.5	548.2	511.8	606.6	664.4	594.1	538.3	516.7	538.8	520.8	590.2	530.7	582	558	629	6
Os03g0616500	47.9	28.9	32.3	115.8	88.59	91.7	40.7	59.5	46.3	39.66	30.7	33.87	53.337	49.473	34.847	143.3	38.13	33.15	87.32	95.14	77.6	63.59	65.56	69.34	36.97	30.17	41.67	56.4	44.9	46.7	6
Os03g0151300	1298	1531	1367	1301	1373	1311	1246	1475	1211	1119	1230	1053	1357.4	1194.8	1269.5	2484	1466	1393	1199	1430	1441	1474	1368	1217	1316	1312	1066	1450	1467	1502	6
Os03g0292100	1327	1670	1016	268.5	202.5	218.9	305	348	377	449.2	393.6	354.7	403.84	308.04	404.69	1564	1942	1149	215.5	239.5	428.5	466.6	513.8	1299	458.5	482.7	434.2	336	345	494	14
Os03g0300200	222	225	218	330.9	337.2	315.7	107	112	104	153.5	121.7	159.9	174.16	198.83	181.36	203	177.3	202	290.8	332.2	301.8	170.1	190.3	144.4	150	171.6	166.7	172	193	153	10
Os03g0294200	453	366	397	231.5	198	243.1	171	252	227	329.4	304.8	202.3	314.58	278.17	352.42	489.6	303	407.2	168.1	185.4	187.1	353.4	382	301.9	294.7	269.7	320.2	431	436	385	13

Os03g0192000	270	224	234	226.6	226.9	193.8	171	173	182	196.6	193	212.6	188.31	218.43	175.02	394.1	205.1	182.3	252.7	254	249.2	159.7	160.3	153.1	221.8	208.4	206.1	183	197	172	6
Os03g0386500	31.6	15.8	22.6	9.852	10.85	7.785	4.7	15.2	11.7	81.91	55.92	84.67	35.921	56.007	72.069	59.7	40.19	24.86	6.503	9.675	6.036	14.05	21.38	15.17	99.3	59.4	39.47	47.2	42.1	57.5	3
Os03g0640100	773	800	830	1259	1268	1135	1396	1378	1385	913.9	823.4	911.7	936.13	943.72	930.56	1457	741	772.9	1244	1134	1187	980.4	1063	994.5	855.7	818.4	771.9	822	845	876	12
Os03g0673600	317	299	330	279.1	273	290.7	323	309	355	618.2	577.8	792.2	658.55	534.87	519.53	823.9	591.5	513.9	480.3	382.2	520.8	234.4	193.1	299.7	656	692	609.6	414	458	366	3
Os03g0165300	191	342	204	429.4	492.7	357.3	255	365	249	159.5	230.2	76.21	138.24	131.62	177.4	179.1	165.9	178.2	113.3	140.3	128.5	523.5	499.6	352.5	210.2	200.8	129.4	268	194	342	17
Os03g0145150	1.02	1.86	1.08	3.284	0.904	1.73	3.91	0	0	0	0	0	2.177	0	0	0	1.031	0	0	1.613	2.587	2.218	2.851	0	0	0.943	0	0.93	1.91	0.83	2
Os03g0857000	59.1	76.4	53.9	151.9	151.9	129.8	305	275	258	57.77	47.14	106.3	109.94	70.942	89.492	95.53	94.81	85.99	144	139.5	200.9	207	171.7	278.8	50.71	56.57	67.98	67.5	70.8	75	18
Os03g0228800	5.1	12.1	6.47	3.284	9.944	7.785	46.2	14.5	35.2	4.311	7.675	8.467	10.885	4.6673	5.5438	11.94	2.061	3.108	0.929	2.419	3.449	11.09	9.264	14.44	3.169	4.714	13.16	0.93	1.91	1.67	18
Os03g0334700	1832	1694	1739	1053	1010	1064	1412	1711	1576	1818	1899	1602	1715.5	1562.6	1865.9	2269	1878	1657	998.6	1048	936.4	1553	1588	1576	1836	1852	1640	1822	1798	1924	13
Os03g0399532	999	1086	1130	476.2	507.1	485.3	265	393	359	650.1	768.6	575.8	1042.8	833.57	1027.2	1827	910	959.4	333.5	372.5	309.5	467.3	436.8	493.3	826.1	667.5	550.4	916	842	990	13
Os03g0180600	2.04	4.66	0	0	0	0	0.78	0	0	0	0	0	0	0	0	0	0	0	0.929	0	0.862	0.739	0	0	0	0.943	0	0	0	0.83	14
Os03g0194350	0	1.86	0	0	0.904	2.595	0.78	1.27	2.76	0.862	2.193	2.822	0	0	0	0	3.092	1.036	0.929	0	1.724	0	0.713	0.722	0	0	0	1.85	0	0.83	15
Os03g0822100	92.8	75.4	59.3	116.6	141.9	147.9	83	75.9	91.9	70.7	78.94	107.3	93.613	94.279	80.781	143.3	73.17	103.6	129.1	137.9	182.8	153.8	145.4	117	68.66	84.86	78.95	93.4	99.5	95	10
Os03g0801500	286	308	281	477	550.5	518.2	806	827	810	413.8	445.1	429	355.95	359.38	384.1	322.4	261.8	318.1	551.8	583.7	612.2	660.3	558	607.4	387.7	420.5	379.4	281	327	323	18
Os03g0432000	15.3	26.1	23.7	16.42	14.46	10.38	29.7	19.6	16.6	39.66	31.8	27.28	13.062	20.536	21.383	59.7	22.67	29.01	27.87	33.86	19.83	10.35	30.64	18.78	36.97	16.03	15.35	29.6	31.6	15	6
Os03g0858200	5.1	10.2	5.39	6.568	4.52	5.19	12.5	10.1	5.53	7.76	8.771	10.35	7.6196	5.6007	3.9598	11.94	3.092	4.144	4.645	0	0.862	1.479	3.563	6.5	12.68	17.91	17.54	2.78	4.78	9.16	11
Os03g0646200	26.5	34.5	22.6	151.1	189.8	160	57.9	46.8	83.6	37.94	20.83	32.93	26.124	40.138	20.591	0	27.82	29.01	184.9	156.4	159.5	54.71	54.16	52	9.507	23.57	24.12	11.1	23	24.2	10
Os03g0672700	1065	1042	983	1053	964.5	1036	963	1007	1039	1124	1161	1467	1355.2	1143.5	1127.8	1552	1069	1098	1322	1333	1322	862.9	896.5	915.1	1132	1242	1158	1107	1137	1121	16
Os03g0642900	5.1	9.31	4.31	5.747	6.328	6.92	28.2	10.1	12.4	5.173	0	19.76	2.177	6.5342	3.9598	0	5.153	6.216	12.08	3.225	2.587	6.654	7.839	3.611	1.056	2.829	6.579	7.4	9.56	2.5	15
Os03g0850000	408	465	460	658.4	678	556.2	1017	1047	1056	590.6	570.1	589.9	478.95	448.99	516.36	477.6	471	430	640	669.2	498.4	797.1	801.7	840.7	567.3	514.8	491.2	384	458	491	18
Os03g0758950	2.04	1.86	1.08	0.821	1.808	2.595	0	0	3.46	0.862	1.096	0	2.177	0	0.792	0	1.031	3.108	3.716	4.031	3.449	0	0	0.722	1.056	0	0	0.93	2.87	3.33	16
Os03g0761000	18.4	13	4.31	194.6	221.5	184.3	90.8	47.4	51.1	12.07	12.06	11.29	11.974	12.135	7.1277	11.94	4.122	9.324	189.5	276.6	218.1	28.1	19.95	31.78	6.338	6.6	6.579	4.63	5.74	6.66	10
Os03g0742800	13.3	11.2	9.7	11.49	12.66	7.785	1.57	10.8	5.53	6.897	5.482	11.29	4.3541	12.135	14.255	23.88	9.275	12.43	4.645	16.13	5.173	20.7	21.38	10.83	12.68	8.486	17.54	16.7	22	18.3	8
Os03g0345300	0	0	0	0	0	0	0	0	0	0	0	0	0	0	0.792	0	0	1.036	0	0	0	0	0	0	0	0	0	0	0	0	4
Os03g0855700	3231	3617	3797	2260	2449	2625	1676	1867	1799	3045	3075	2648	3474.6	3205.5	3291.4	8239	3414	3908	2023	2468	2158	2337	1970	1874	3377	2949	2603	4217	4563	4483	13
Os03g0830800	0	0	1.08	0.821	0.904	0	0	0.63	2.07	0.862	1.096	0	0	0	0	0	0	1.036	1.858	1.613	0	0.739	0.713	0.722	0	0	0	0	1.91	0	12
Os03g0151600	50	36.3	51.7	97.7	94.01	76.99	41.5	57.6	63.6	45.7	51.53	33.87	45.718	44.806	47.518	11.94	34.01	30.05	76.17	77.4	82.77	40.67	31.36	46.95	30.63	18.86	39.47	29.6	41.1	36.7	10
Os03g0133850	4.08	2.79	2.16	3.284	1.808	1.73	0	0.63	2.76	2.587	2.193	0.941	0	0	1.5839	0	1.031	8.288	1.858	1.613	0	0.739	1.425	0.722	2.113	2.829	2.193	5.55	0	0	19
Os03g0757900	389	377	452	192.1	169	269	250	276	255	416.4	206.1	185.3	162.19	158.69	194.03	203	252.5	194.8	198.8	229	214.7	179.7	235.9	273.7	124.7	112.2	208.3	130	203	111	14
Os03g0826800	4.08	5.59	2.16	548.4	645.4	415.2	392	487	372	11.21	4.386	8.467	4.3541	11.201	14.255	11.94	11.34	11.4	279.6	274.9	229.3	539	532.3	426.1	14.79	8.486	8.772	16.7	9.56	8.33	12
Os03g0371350	486	604	524	756.9	790.1	689.4	93.9	155	133	563.9	703.9	454.4	966.61	618.88	791.97	1051	546.2	551.2	431	501.5	371.6	161.2	182.4	134.3	672.9	429	298.2	553	624	861	13
Os03g0743400	2621	2711	2538	3404	3531	3264	4678	4550	4610	3368	3138	3699	3230.7	2941.3	2870.9	3212	2333	2323	3628	3211	3458	3270	3523	3613	2964	2885	3031	2175	2012	2399	18
Os03g0752100	895	1039	918	1632	1729	1561	1231	1359	1198	910.5	967	1085	998.17	861.58	909.97	931.4	905.8	948	1340	1396	1489	1295	1191	1327	1057	975.8	1066	928	837	971	10
Os03g0146600	185	154	142	174.9	149.2	179.1	384	380	321	192.3	203.9	230.5	152.39	146.55	175.02	262.7	126.8	147.1	159.8	162.9	159.5	195.2	214.5	242	187	175.4	193	135	112	128	18
Os03g0668900	79.5	52.2	67.9	305.4	312.8	322.7	105	99.9	106	73.29	51.53	113.8	101.23	90.545	53.854	59.7	56.68	70.45	346.5	394.3	369	65.07	81.95	62.11	43.31	80.14	96.49	49	54.5	52.5	10
Os03g0811550	0	0	0	0.821	0	0	0	0.63	0	0	0	0	0	2.8004	0.792	0	1.031	0	0.929	0.806	0	0.739	1.425	0	0	2.829	2.193	1.85	0.96	0	11
Os03g0670800	3.06	3.73	5.39	0.821	5.424	1.73	4.7	3.16	8.98	1.724	1.096	1.882	5.4426	4.6673	2.3759	0	4.122	2.072	0.929	1.613	1.724	5.176	4.988	0.722	3.169	0.943	2.193	8.33	0.96	6.66	1
Os03g0131400	54	55.9	45.3	79.63	75.03	70.07	135	106	130	68.11	104.2	68.68	64.223	81.21	44.35	59.7	39.16	36.26	115.2	85.47	100.9	76.9	79.82	83.06	54.93	49.03	50.44	37	52.6	49.2	15
Os03g0187400	56.1	51.2	63.6	36.94	23.5	29.41	27.4	27.8	42.2	56.9	33.99	31.05	32.656	27.07	39.598	35.82	61.83	40.41	43.66	26.61	43.97	48.06	43.47	35.39	38.03	35.83	35.09	61.1	30.6	53.3	14
Os03g0127100	1.02	0	1.08	0	0	0	0	0	1.38	0	0	3.763	0	0.9335	0	0	0	0	0.929	0	0	0	0	0.722	0	0.943	8.772	0.93	2.87	2.5	11
Os03g0329700	695	940	972	659.2	731.3	492.2	1055	717	1041	940.6	826.7	455.4	721.69	668.35	696.14	1564	600.8	744.9	430.1	626.5	340.6	675.8	648.5	629.8	764.8	508.2	570.2	841	1040	870	6
Os03g0782900	7.14	6.52	3.23	7.389	1.808	3.46	3.13	8.86	6.91	6.035	8.771	6.586	8.7082	7.4676	13.463	0	7.214	6.216	3.716	12.09	5.173	3.697	4.276	5.778	5.282	8.486	6.579	8.33	7.65	10	4
Os03g0732200	691	811	737	1048	1120	1002	1621	1720	1597	869.9	971.4	935.2	1152.7	1033.3	1072.3	1385	775	1006	1004	1103	1234	1323	1214	1244	960.2	850.4	835.5	995	1070	1160	18
Os03g0205400	263	305	280	345.6	420.3	331.3	278	321	330	194.9	242.3	166.5	293.9	237.1	280.36	358.2	226.7	240.4	278.7	294.3	275	383	426.2	305.5	274.7	260.2	263.2	307	284	299	17
Os03g0701700	43.8	38.2	46.3	64.86	62.37	70.93	124	81.6	91.9	52.59	72.36	96.9	74.019	65.342	43.558	35.82	55.65	42.48	103.1	82.24	117.3	80.59	76.25	88.11	94.02	69.77	78.95	57.4	56.4	45	15
Os03g0764600	2446	1380	2051	931.8	582.2	844.3	160	572	732	2576	2561	2547	1747.1	1196.7	1935.6	3200	1932	1734	1136	1197	1280	593.7	809.6	571.3	2305	2856	2228	970	1299	1035	3
Os03g0255500	862	1334	1280	1316	1027	1792	9333	7522	####	1768	1934	1358	1396.6	1422.6	1876.2	883.6	898.6	936.6	1079	1703	1086	11998	13870	10985	1296	1229	1138	1619	1692	1355	18
Os03g0298600	182	145	209	229.9	190.7	165.2	398	373	371	281.1	291.6	271	261.24	221.23	239.97	238.8	203	189.6	245.2	252.4	289.7	224	265.8	316.3	210.2	281.9	296	229	193	228	5
Os03g0302700	127	98.7	124	160.1	130.2	134.9	155	84.1	91.9	150	131.6	127	199.2	168.02	171.06	214.9	117.5	110.9	151.4	189.5	190.5	104.3	91.22	85.95	132	114.1	94.3	130	121	132	16
Os03g0137600	1303	981	1130	921.1	965.4	1062	2449	2037	1733	970	801.5	1845	1341.1	1604.6	1106.4	1827	1960	1351	2124	2276	2201	1216	1182	2230	1301	1483	1314	1444	1597	1262	15
Os03g0140100	190	279	216	525.4	455.6	400.5	164	239	220	190.5	205	188.2	227.5	252.97	218.58	429.9	238.1	227.9	300.1	433.8	413.9	347.5	523.8	312	276.8	265.9	271.9	294	276	288	10
Os03g0145775	2.04	0	0	0	0	0.865	0.78	0.63	0	0	0	0	0	0	0	0	0	0	0	2.419	0.862	0	2.138	0	0	0	0	1.85	0	0	19
Os03g0235300	199	234	236	204.4	168.1	197.2	138	173	233	187.1	193	204.2	202.46	249.23	242.34	406	232.9	230	183.9	203.2	204.3	227.7	191.7	219.6	209.2	226.3	225.9	254	238	215	13
Os03g0847500	366	345	376	416.2	390.5	401.4	198	222	189	335.4	284	211.7	334.18	297.77	373.81	465.7	341.1	367.8	296.3	371.7	267.3	428.1	359.9	296.8	360.2	321.5	355.3	402	400	432	8
Os03g0180300	5273	4854	5500	1622	1576	1629	1281	1440	1421	3772	4319	3443	5241.2	5557.8	6053	12132	5988	5655	1745	1764	1697	1628	1705	1491	4762	4761	4259	6137	5969	6077	13
Os03g0397400	141	95.9	114	96.05	139.2	83.04	84.6	99.9	146	155.2	108.5	144.9	138.24	90.545	97.412	155.2	87.6	126.4	78.03	74.18	67.25	194.5	146.1	156	112	107.5	151.3	92.5	95.6	94.1	9
Os03g0704700	444	472	388	494.2	430.3	446.4	297	338	296	422.5	447.3	317.1	351.59	417.25	364.3	489.6	552.4	503.5	371.6	420.1	419	295	327.1	324.3	389.8	401.7	394.7	474	466	394	8
Os03g0738400	####	####	####	14612	13413	14116	####	####	####	83013	98014	79769	98262	96552	95281	#####	80213	75858	22191	18219	19267	44848	45187	58844	97066	#####	90035	####	####	####	3
Os03g0644700	22.4	27.9	23.7	40.23	42.49	35.47	33.7	52.5	51.8	15.52	24.12	17.88	8.7082	16.802	10.296	0	14.43	20.72	40.87	30.64	54.32	65.07	45.61	70.06	7.395	6.6	8.772	13	10.5	5.83	12
Os03g0422300	0	0	0	0	0	0	0	1.9	4.15	0	0	0	1.0885	0	0	0	2.061	0	0.929	0	1.724	0.739	0	0	1.056	0	0	0	0	0	18
Os03g0130500	1190	1195	1126	1203	1265	1155	1322	1311	1233	875.1	889.2	849.6	931.77	892.38	897.3	1588	1080	926.2	905.7	899	886.4	1187	1135	1161	942.3	897.6	947.3	946	941	1001	6
Os03g0145600	54	70.8	37.7	28.73	18.98	25.95	16.4	13.3	12.4	52.59	44.95	86.56	14.151	39.205	29.303	35.82	63.89	55.95	17.65	12.9	13.8	10.35	4.276	17.33	21.13	105.6	92.1	18.5	37.3	27.5	20
Os03g0857600	65.2	44.7	34.5	4.926	2.712	4.325	39.9	12	36.6	18.11	27.41	133.6	25.036	26.137	15.047	95.53	127.8	84.96	25.08	7.257	22.42	46.58	36.34	96.06	68.66	209.3	217.1	25.9	38.3	25.8	11
Os03g0267000	96.9	35.4	72.2	10.67	13.56	14.71	6.26	6.96	0.69	66.39	52.63	83.73	91.436	100.81	76.821	35.82	23.7	37.3	3.716	8.063	8.622	3.697	6.414	4.333	32.75	32.06	50.44	25	20.1	24.2	3
Os03g0288400	332	322	398	714.2	718.7	647.9	997	926	974	475.9	449.5	638.8	555.15	443.39	415.78	632.9	367.9	383.3	942	961.9	975.2	593	527.3	621.9	369.7	395.1	326.7	342	347	332	12
Os03g0678100	54	36.3	42	37.76	39.77	48.44	77.5	70.8	67	81.91	61.4	97.85	47.895	50.406	57.022	107.5	41.22	50.77	65.03	46.76	86.22	44.36	42.76	57.78	90.85	85.8	94.3	69.4	41.1	53.3	20
Os03g0177000	466	440	498	417.1	468.3	508.6	539	698	683	386.3	498.9	415.8	379.89	276.3	344.51	334.3	344.2	407.2	234.1	338.6	359.5	646.2	627.8	629.1	407.8	380.9	320.2	264	252	318	18
Os03g0790000	1.02	0	0	1.642	1.808	1.73	0	0	0	2.587	0	0	0	0	0	0	1.031	0	0	4.031	0	1.479	0	0	1.056	0	0	0	0	1.67	2
Os03g0294600	11.2	6.52	1.08	5.747	9.04	9.516	14.1	5.69	7.6	19.83	8.771	8.467	8.7082	6.5342	7.9197	47.76	2.061	5.18	9.29	12.9	11.21	4.436	7.839	10.83	11.62	4.714	6.579	5.55	6.69	7.5	6
Os03g0332533	2.04	0.93	4.31	0	0.904	0.865	0.78	0.63	0	0	0	3.763	2.177	0.9335	0	23.88	0	0	0	3.225	0.862	2.218	0	14.44	2.113	0	0	0.93	0	1.67	6
Os03g0281201	0	0	0	0	0	0.865	0	1.27	0	0	0	0	0	0	0	0	0	0	0	0	0	0.739	1.425	1.444	0	0.943	2.193	0	0	0	11
Os03g0182600	1167	952	1017	1419	1483	1512	1578	1321	1589	1309	964.8	1875	1082	1208.8	796.72	1337	867.7	1015	1611	1340	1809	1116	1123	1252	980.3	1201	1278	773	973	724	15
Os03g0574300	198	234	184	128.1	155.5	117.6	75.2	91.1	65.6	137.9	186.4	145.8	152.39	164.29	218.58	274.6	259.7	267.3	110.5	140.3	136.2	167.8	165.3	96.78	210.2	248.9	166.7	281	202	246	13
Os03g0310633	64.2	30.7	70	103.4	126.6	131.5	25.8	22.1	23.5	23.28	12.06	33.87	60.957	55.074	49.894	179.1	88.63	69.41	124.5	94.33	132.8	40.67	36.34	27.45	42.25	42.43	37.28	54.6	52.6	48.3	16
Os03g0593200	653	719	736	1484	1576	1337	514	617	549	563	615.1	479.8	881.7	642.21	779.29	991.1	531.8	654.8	737.6	828.1	743.2	666.9	663.5	574.9	533.5	445	311.4	613	660	761	2
Os03g0221200	16.3	27.9	38.8	32.84	10.85	32.87	15.7	31	29	70.7	20.83	7.527	23.947	32.671	34.847	23.88	8.244	5.18	6.503	15.32	10.35	31.79	76.25	23.83	24.3	10.37	13.16	5.55	44.9	16.7	17
Os03g0724500	130	91.3	122	262.7	316.4	230.1	151	214	122	88.81	72.36	53.63	210.08	206.29	179.78	167.2	79.35	119.1	174.6	220.1	210.4	114.6	173.9	135.8	90.85	63.17	63.59	82.3	114	90.8	10
Os03g0844450	134	95.9	87.3	220.8	249.5	254.3	132	141	106	117.3	88.81	141.1	100.14	117.61	90.284	131.4	93.78	82.88	247.1	224.1	219.9	141.2	137.5	121.3	118.3	123.5	140.3	116	121	100	10
Os03g0333200	673	610	657	449.1	436.6	417.8	522	559	624	512.1	476.9	521.2	514.87	471.39	557.54	692.6	517.3	529.4	394.8	384.6	399.2	662.5	632.1	711.4	517.6	577	443	457	494	518	9
Os03g0386600	22.4	29.8	28	13.14	33.45	14.71	15.7	15.2	11.1	13.79	26.31	11.29	4.3541	16.802	15.047	0	16.49	21.76	17.65	11.29	13.8	18.48	8.552	10.11	12.68	6.6	4.386	14.8	22	22.5	14
Os03g0708000	0	0	0	2.463	0	0.865	0	1.27	4.15	0	0	0	0	0	0	0	1.031	0	1.858	0	0	0.739	1.425	0.722	0	0	0	0	0.96	0	18
Os03g0839200	42.8	33.5	34.5	27.91	28.93	21.63	90.8	75.3	53.2	92.25	70.17	157.1	47.895	38.272	65.733	143.3	68.02	46.62	51.09	35.48	52.6	61.37	47.03	83.06	83.45	85.8	52.63	55.5	35.4	53.3	20
Os03g0146300	121	90.4	101	620.7	453.8	781.1	125	123	105	129.3	107.4	162.8	96.878	120.42	90.284	238.8	120.6	90.14	1119	647.4	967.4	93.9	91.22	133.6	97.19	128.2	157.9	105	85.1	97.5	10
Os03g0255000	997	1007	1014	834.9	767.5	841.7	629	805	701	833.7	944	700.9	1040.6	843.84	1082.6	1636	879	984.2	604.7	723.2	653.6	758.6	897.2	794.5	951.1	909.8	743.4	1164	995	1087	13
Os03g0728600	16.3	29.8	19.4	94.41	104.9	93.43	57.9	73.4	70.5	38.8	25.22	16.93	20.682	26.137	26.135	107.5	24.73	31.08	105.9	87.88	86.22	70.98	73.4	70.06	12.68	15.09	17.54	34.2	23	32.5	10
Os03g0761500	51	40.1	57.1	13.14	9.944	17.3	3.13	8.22	2.76	30.18	18.64	23.52	7.6196	12.135	6.3357	23.88	58.74	45.59	46.45	34.67	61.22	1.479	1.425	3.589	26.41	19.8	15.35	28.7	17.2	17.5	16
Os03g0585600	1.02	2.79	3.23	4.105	0	0.865	1.57	5.69	3.46	0	4.386	0	1.0885	2.8004	0.792	23.88	6.183	3.108	1.858	3.225	1.724	2.218	1.425	1.444	0	3.771	0	6.48	2.87	4.17	6
Os03g0119500	2005	2520	2211	2114	2196	2243	1996	2097	1804	1654	1758	1459	1681.8	1783.8	1896.8	3630	2239	2042	1549	1663	1671	2555	2951	2176	2067	1796	1730	2614	2408	2264	6
Os03g0161000	882	1005	1084	885.8	922	958.5	975	834	858	947.5	982.4	967.2	987.29	1011.9	944.02	1075	853.3	948	764.5	794.3	860.9	1099	981.3	860.9	1156	905.1	861.8	930	926	1174	8
Os03g0115200	406	446	332	432.7	475.5	424.7	637	571	717	436.3	371.7	679.3	455	409.79	358.76	382.1	460.6	383.3	389.2	387.8	389.7	625.5	468.9	563.4	395.1	515.7	515.3	320	370	296	18
Os03g0206400	79.5	99.7	57.1	128.1	89.49	173	336	262	435	177.6	152.4	236.1	161.1	172.69	163.14	191.1	98.93	103.6	162.6	165.3	231.9	319.4	330.7	325.7	200.7	216.9	320.2	158	124	150	18
Os03g0124200	229	151	205	73.07	64.18	95.16	143	119	162	308.7	197.4	208.9	146.95	189.49	195.62	191.1	235	196.8	145.8	95.95	131.1	136.8	180.3	172.6	178.5	159.3	190.8	216	202	183	3
Os03g0390600	3730	3217	3078	2356	2318	2424	3716	3077	3482	3981	4045	4251	3919.8	3750.6	3498.1	4717	4063	3904	3745	4152	4441	3105	3193	3801	4239	4883	4802	3860	4230	3845	3
Os03g0735000	1193	1176	1240	913.7	1048	951.6	1367	1553	1545	1057	1288	1419	1105.9	1305.9	1178.4	1122	1261	1293	970.8	924.8	1161	1247	1097	1382	1111	1270	1162	1076	1230	1204	5
Os03g0348900	2.04	0.93	1.08	0.821	0.904	2.595	3.91	5.06	4.84	1.724	1.096	4.704	1.0885	0.9335	2.3759	0	3.092	0	4.645	2.419	4.311	5.176	2.138	7.945	1.056	4.714	6.579	0.93	2.87	1.67	9
Os03g0192800	57.1	71.7	61.4	73.07	76.84	90.83	20.4	15.2	19.3	27.59	28.51	13.17	42.452	39.205	50.686	191.1	50.5	63.2	19.51	44.35	31.9	41.41	47.75	18.78	38.03	39.6	21.93	70.3	44	76.6	6
Os03g0741700	153	146	120	124.8	113.9	119.4	79.9	113	92.6	99.15	115.1	97.85	137.15	133.48	133.84	155.2	131.9	137.8	151.4	112.1	106.1	118.3	119	106.9	113	80.14	92.1	112	121	127	8
Os03g0756200	1699	1622	1747	1497	1528	1478	1647	1696	1665	1809	1846	1356	1617.5	1703.5	1859.5	3236	1641	1566	1213	1484	1336	2085	2274	1797	1789	1662	1585	1802	1718	1743	6
Os03g0562000	1621	1676	1805	1348	1308	1404	1239	1378	1324	1137	1550	1055	1314.9	1360	1500	3332	1493	1558	1200	1670	1370	1927	1384	1450	1505	1447	1294	1669	1642	1776	6
Os03g0390400	931	754	822	572.2	600.2	616.8	868	704	797	919.1	827.8	1258	870.82	1040.8	870.37	955.3	830.6	712.8	903.9	754.7	793.2	528.7	587.2	613.9	796.5	1022	1213	705	809	674	20
Os03g0128866	15.3	10.2	20.5	9.031	10.85	10.38	4.7	12.7	4.15	17.24	21.93	9.408	8.7082	11.201	11.088	11.94	9.275	25.9	2.787	13.71	8.622	13.31	8.552	7.945	10.56	7.543	6.579	14.8	24.9	18.3	1
Os03g0198500	298	325	359	253.7	240.5	281.1	452	439	439	271.6	330	292.6	359.21	336.04	330.25	453.8	392.6	369.9	312.1	275.7	274.2	375.6	342.8	398.7	325.4	358.3	318	319	329	313	5
Os03g0167400	7.14	3.73	0	1.642	3.616	8.65	3.13	0	0	0.862	1.096	0	5.4426	2.8004	2.3759	0	5.153	3.108	0	0.806	2.587	2.958	2.851	4.333	5.282	0.943	0	7.4	5.74	0.83	8
Os03g0124500	64.2	103	62.5	82.1	76.84	68.34	510	258	379	150.9	202.8	113.8	90.347	112.01	60.189	143.3	81.41	83.92	49.23	30.64	60.36	93.9	54.16	91	104.6	96.17	142.5	37.9	45.9	68.3	5
Os03g0741400	1086	733	937	541.8	471.9	513	896	859	760	1074	787.2	1107	601.95	691.69	637.53	1552	795.6	898.2	556.4	479.7	552.7	645.5	673.4	760.5	597.9	810.8	907.9	643	806	725	13
Os03g0763000	659	552	709	612.4	681.6	664.4	477	435	412	676.8	689.6	838.3	763.05	800.9	791.97	1170	823.4	807.1	794.3	899	956.2	429.6	361.3	373.4	668.7	826.9	743.4	723	766	813	13
Os03g0731800	0	1.86	0	0	0	0	0	0	0	0	1.096	0	0	0.9335	0	0	0	0	0	0	0	0	0	0	0	0	0	0	0	0.83	14
Os03g0676300	272	230	246	190.5	145.5	184.3	164	209	365	144.8	140.3	101.6	89.259	61.608	85.532	155.2	97.9	76.67	157.9	106.4	56.04	117.6	138.3	216.7	90.85	33.94	67.98	111	67.9	70	18
Os03g0302500	127	171	170	218.4	249.5	191.2	558	427	495	338	287.3	278.5	199.2	209.09	181.36	286.6	148.4	162.7	229.5	287.8	204.3	330.5	371.3	385.7	262	218.7	230.3	210	173	200	18
Os03g0653100	0	0	0	0	0	1.73	0	0.63	0	0	0	0	0	0.9335	0	0	1.031	0	0	0.806	0	0.739	0	1.444	0	0	2.193	0	0.96	0	11
Os03g0397300	250	257	242	269.3	266.7	251.7	323	273	307	250.9	265.3	211.7	216.62	230.56	258.18	453.8	240.1	262.1	271.3	280.6	227.6	282.4	261.5	237.6	247.2	248	228.1	248	208	258	6
Os03g0655300	142	114	122	137.9	175.4	153.1	156	159	131	121.6	140.3	106.3	120.83	151.22	135.43	167.2	145.3	161.6	177.4	178.2	202.6	88.73	94.07	101.8	110.9	159.3	160.1	160	166	153	16
Os03g0423850	6745	6705	7151	6904	7215	7474	5407	6094	5441	5837	7026	6252	7152.7	5720.2	6779.2	12657	6588	7654	6366	7979	6443	8907	6987	6421	7139	6479	5506	7314	7301	8090	6
Os03g0159900	791	829	753	1346	1428	1421	764	674	682	859.6	944	1148	837.07	1004.4	818.1	1433	1348	1224	1750	1421	1901	795.6	610.7	816.9	925.4	1057	962.7	908	899	1106	16
Os03g0374901	0	2.79	1.08	0.821	0	1.73	0	0	0.69	0	0	0	1.0885	0	1.5839	0	1.031	0	0	1.613	4.311	0.739	2.138	0.722	1.056	0	0	2.78	1.91	0.83	16
Os03g0340500	111	113	103	243	229.6	209.3	335	288	323	175	159	136.4	130.62	144.68	134.63	322.4	97.9	89.1	262.9	294.3	232.8	142	190.3	168.3	109.9	93.34	125	86	101	92.5	12
Os03g0151900	709	700	654	514.8	530.6	509.5	662	667	623	734.6	868.3	729.1	761.96	853.17	769.79	764.2	773.9	702.4	514.6	475.7	490.6	531.6	625	653.6	845.1	804.2	1002	774	620	707	3

Os03g0328400	93.8	82.9	106	66.5	65.99	68.34	175	144	131	136.2	91	122.3	108.85	86.811	79.197	203	127.8	90.14	95.68	91.92	96.57	77.64	84.09	91.73	82.4	106.5	94.3	82.3	124	96.6	6
Os03g0712900	208	173	194	252.9	268.5	282.9	165	177	182	230.2	223.7	146.8	219.88	212.83	220.17	286.6	215.4	195.8	255.5	337	267.3	194.5	205.2	181.3	249.3	208.4	225.9	282	274	284	16
Os03g0610900	157	121	140	299.7	301	339.1	199	199	195	202.6	186.4	251.2	182.87	220.29	186.11	131.4	243.2	191.7	272.2	296.7	335.4	211.5	171.7	210.2	300	270.6	285.1	249	130	192	16
Os03g0680566	2.04	0	0	1.642	1.808	4.325	3.91	1.27	0	0.862	0	5.645	1.0885	0	2.3759	23.88	3.092	1.036	7.432	0	4.311	0.739	2.138	2.167	1.056	0.943	0	0.93	5.74	0.83	6
Os03g0806800	1.02	1.86	1.08	0	6.328	3.46	2.35	0.63	0	12.07	1.096	6.586	3.2656	0	1.5839	0	0	2.072	0.929	0.806	4.311	0	2.851	7.945	0	10.37	15.35	0	7.65	0.83	11
Os03g0158700	46.9	64.3	42	96.88	102.1	70.07	51.7	52.5	47	32.76	54.82	32.93	54.426	37.338	41.974	95.53	72.14	51.8	85.46	82.24	62.94	36.97	49.88	27.45	57.04	34.89	24.12	37.9	46.9	50.8	10
Os03g0843500	3318	3235	3464	2739	3102	3208	2137	2303	2195	2701	3167	2652	3365.7	3283.9	3293.8	5290	3714	3907	2704	2945	2819	3040	2461	2235	3317	3274	2974	3844	3669	3965	13
Os03g0685600	447	387	433	363.7	364.3	428.2	507	439	458	487.1	462.7	485.5	474.59	540.47	502.9	692.6	498.8	450.7	433.8	375.7	480.3	415.5	401.2	382.1	521.8	517.6	418.8	461	478	458	13
Os03g0112600	87.7	107	87.3	77.99	68.7	59.69	12.5	29.7	18	29.31	39.47	18.82	39.187	40.138	47.518	35.82	46.37	82.88	13.01	32.25	15.52	99.82	89.08	51.28	44.37	61.28	21.93	87	82.2	95.8	8
Os03g0711600	129	99.7	134	65.68	55.14	38.93	86.1	75.9	87.8	119.8	85.52	116.7	100.14	84.011	83.948	179.1	99.96	134.7	71.53	74.98	78.46	93.9	82.67	93.89	106.7	102.8	103.1	95.3	79.4	101	13
Os03g0842501	33.6	17.7	9.7	20.52	14.46	33.74	47	28.5	33.2	15.52	23.02	25.4	25.036	34.538	21.383	0	18.55	23.83	34.37	27.41	36.21	28.1	21.38	28.89	20.07	24.51	35.09	13	13.4	10.8	15
Os03g0797000	233	207	212	181.4	179	190.3	233	262	279	180.2	202.8	225.8	243.83	252.97	280.36	429.9	267.9	253.8	183	195.1	208.7	330.5	292.2	214.5	257.8	272.5	258.8	256	221	262	6
Os03g0844050	0	0	0	0.821	0	0	0	0	0	0	0	0	0	0	0	0	0	0	0	0	0	0	0	0	0	0	0	0	0	0	2
Os03g0215800	404	329	393	348.1	326.3	371.1	337	336	346	365.6	357.4	262.5	321.11	314.57	393.61	549.3	339	347.1	322.3	358.8	313	496.9	391.9	411.7	335.9	369.6	394.7	421	364	352	6
Os03g0203200	1786	2494	1701	2441	2258	2426	3936	4843	4512	2808	2744	2897	2477.5	2284.2	2837.6	2723	2250	2336	1756	1802	1712	2992	4043	2860	2361	2334	1932	1847	1454	2127	18
Os03g0745000	16.3	7.45	10.8	4.105	3.616	5.19	0	0.63	0	26.73	13.16	21.64	46.806	27.07	39.598	0	2.061	2.072	2.787	2.419	0	0.739	0.713	0	8.451	1.886	2.193	5.55	4.78	7.5	4
Os03g0752700	1993	2207	2187	1527	1506	1479	2117	2178	1951	1941	2135	1945	2124.8	2058.3	2279.3	3881	2775	2492	1502	1685	1493	2354	2144	2147	2283	2528	2217	2509	2704	2544	13
Os03g0689400	18.4	12.1	12.9	25.45	23.5	58.82	25.1	31	24.2	15.52	12.06	17.88	4.3541	16.802	19.799	11.94	26.79	11.4	31.58	19.35	25	57.67	53.45	60.67	22.18	27.34	30.7	33.3	17.2	20.8	9
Os03g0283500	28.5	23.3	44.2	125.6	142.8	122.8	57.2	63.9	56.7	10.35	17.54	1.882	14.151	10.268	14.255	23.88	16.49	13.47	78.03	106.4	67.25	59.89	59.86	36.83	26.41	11.31	4.386	23.1	16.3	20	10
Os03g0758250	0	0	0	0	0	0	2.35	0	0	0.862	0	0.941	0	0	0	0	0	1.036	0	0	0	0.739	0	0.722	0	0	0	0	0	0	5
Os03g0118400	52	61.5	71.1	56.65	47.01	50.17	40.7	32.3	19.3	24.14	21.93	23.52	34.833	30.804	30.887	71.65	53.59	37.3	34.37	17.74	29.32	50.28	42.76	43.33	43.31	46.2	28.51	43.5	32.5	60	8
Os03g0651201	0	0	0	0	0	0	0	0	0	0	0	0	0	0	0	0	0	0	0	0	1.724	0	0	0	0	0	0	0	0	0	16
Os03g0370800	8.16	11.2	5.39	13.14	11.75	11.25	29.7	26.6	26.3	22.42	17.54	24.46	13.062	14.002	15.047	0	11.34	10.36	15.79	16.13	11.21	25.88	16.39	15.17	9.507	8.486	24.12	12	17.2	16.7	5
Os03g0801800	227	194	237	404.7	437.5	410	311	297	267	202.6	211.6	222	262.33	256.7	205.91	441.8	204	225.9	358.6	416	370.8	227.7	245.9	239.8	156.3	190.5	133.8	170	203	210	10
Os03g0310800	9820	8062	8945	768.4	571.3	499.1	189	287	218	5818	6674	6123	5986.9	5248.8	5635.6	9732	9822	6365	1249	1116	1279	182.6	281.5	728.7	6568	6285	5908	4835	5085	6205	3
Os03g0773600	136	58.7	81.9	14.78	12.66	25.95	23.5	50.6	37.3	64.66	43.86	42.34	37.01	44.806	56.23	167.2	84.5	82.88	22.29	10.48	12.93	20.7	23.52	46.22	41.2	30.17	35.09	59.2	62.2	43.3	13
Os03g0248200	16.3	11.2	8.62	35.3	28.02	10.38	41.5	44.3	25.6	9.484	17.54	23.52	32.656	28.937	22.175	35.82	17.52	12.43	47.38	72.57	48.28	14.05	26.37	9.389	26.41	15.09	2.193	17.6	30.6	15	16
Os03g0174800	372	207	322	112.5	109.4	81.31	197	207	220	320.7	239	319.9	203.55	196.96	232.05	489.6	242.2	217.6	139.3	125	124.2	127.2	157.5	176.2	196.5	198.9	219.3	162	152	157	13
Os03g0214900	583	634	642	627.2	628.3	648.8	570	595	560	604.4	650.2	469.5	636.78	574.07	650.2	847.8	609	690	589	599.9	608.7	598.2	677	652.9	650.7	631.7	614	620	621	670	6
Os03g0602300	205	184	165	211	244.1	167	173	426	117	94.84	89.9	107.3	146.95	153.09	161.56	585.1	215.4	224.8	189.5	234.6	113.8	224.8	466.8	229.7	168	135.8	155.7	284	305	313	6
Os03g0812300	40.8	45.6	44.2	20.52	23.5	17.3	18.8	3.16	24.2	54.32	47.14	47.98	45.718	45.739	41.182	11.94	39.16	34.19	38.09	36.28	24.14	19.22	9.977	14.44	30.63	30.17	30.7	38.9	31.6	25.8	3
Os03g0798101	1.02	2.79	1.08	7.389	2.712	0.865	2.35	4.43	0	2.587	4.386	2.822	0	5.6007	0.792	0	2.061	3.108	7.432	0	4.311	0	0	0.722	0	0.943	4.386	1.85	1.91	0	16
Os03g0326000	160	177	153	122.3	110.3	89.1	145	163	146	201.8	131.6	96.9	105.59	96.146	129.88	155.2	82.44	80.81	85.46	127.4	47.42	181.9	208.1	155.3	104.6	61.28	74.56	98.1	79.4	107	17
Os03g0804100	71.4	56.8	55	61.57	87.69	72.66	66.5	64.5	78.1	51.73	72.36	49.86	48.983	93.345	76.821	71.65	49.47	53.87	68.74	68.53	62.08	90.21	68.41	63.56	48.59	70.71	43.86	83.3	76.5	65.8	2
Os03g0738000	55.1	48.4	83	149.4	142.8	147.1	180	154	135	97.43	82.23	79.03	120.83	128.82	85.532	95.53	60.8	52.84	300.1	285.4	364.7	81.33	114.7	98.95	65.49	66	70.17	58.3	86.1	69.1	10
Os03g0389700	26.5	30.7	39.9	42.69	18.08	46.71	16.4	4.43	51.8	75.87	46.05	29.17	25.036	25.203	26.135	0	20.61	13.47	54.81	46.76	50.87	11.83	12.83	10.11	10.56	22.63	46.05	21.3	29.6	11.7	7
Os03g0288500	172	172	153	127.3	182.6	145.3	116	125	104	150.9	139.2	132.7	139.33	143.75	159.98	214.9	159.7	152.3	145.8	123.4	168.1	156	153.2	111.2	184.9	136.7	142.5	194	205	178	13
Os03g0830400	15.3	40.1	21.6	27.09	26.22	9.516	44.6	26.6	44.9	27.59	26.31	30.11	20.682	23.336	23.759	11.94	14.43	21.76	14.86	9.675	16.38	55.45	64.14	46.95	15.85	18.86	24.12	28.7	18.2	20	17
Os03g0192500	####	####	####	1599	1837	1781	306	399	280	11452	13739	11975	17993	18536	16513	29040	17710	15866	2214	2024	1959	402.2	375.6	437	15516	14421	11666	####	####	####	13
Os03g0267600	529	495	434	215.1	184.4	242.2	232	250	194	405.2	458.3	513.7	248.18	314.57	339.75	513.5	449.3	426.8	172.8	159.6	196.6	224	265.8	274.5	340.1	479	453.9	379	405	371	3
Os03g0663900	0	0	0	0	0	0	0	0	0	0	0	0	0	0	0	0	0	0	0	0	0	0	0	0	0	0	0	0.93	0	0	1
Os03g0825500	961	1028	1143	1479	1720	1597	850	997	850	881.2	1047	1176	1550.1	1262	1094.5	1325	865.6	962.5	1619	2072	1693	595.2	597.2	797.4	873.6	743.9	633.8	791	796	931	10
Os03g0124100	0	0.93	0	0	0	0	0	0	0	0	0	0	0	0	0	0	0	0	0	0	0	0	0	0	0	0.943	0	0	0	0.83	1
Os03g0654500	279	303	279	356.3	358.9	322.7	361	301	315	327.6	339.9	276.6	316.76	274.43	346.09	394.1	254.5	233.1	282.4	331.4	338	324.6	308.6	252.8	300	303.6	361.8	297	340	303	6
Os03g0108000	96.9	132	115	160.9	136.5	122.8	312	295	292	169	133.8	146.8	105.59	103.61	110.08	59.7	106.1	92.21	164.4	150.8	181.9	210	224.5	250.6	107.7	100.9	85.52	86	102	89.1	18
Os03g0654700	0	0	0	0	0	0	0	0	0	0	0	0	0	0	0	0	0	0	0	0	0	0	0	0.722	0	0	0	0	0	0	9
Os03g0160900	532	622	643	871.9	974.5	879.8	860	833	788	770.8	701.7	702.8	653.11	622.61	595.56	812	537.9	556.4	1042	932.9	907.1	686.9	626.4	777.9	693	620.4	592.1	664	615	613	10
Os03g0781100	23.4	52.2	17.2	80.46	68.7	62.28	17.2	12.7	18	6.897	10.96	2.822	17.416	10.268	10.296	23.88	19.58	22.79	28.8	34.67	15.52	45.1	50.6	28.17	11.62	8.486	0	25.9	20.1	25.8	2
Os03g0353701	79.5	73.6	66.8	113.3	106.7	111.6	114	161	139	77.6	114	87.5	95.79	87.744	102.16	179.1	93.78	107.7	115.2	140.3	142.3	108	117.6	117.7	88.73	100.9	107.5	104	89.9	93.3	6
Os03g0749800	331	373	376	307.9	338.1	305.4	287	280	258	306.1	332.2	313.3	424.52	336.04	380.14	429.9	341.1	390.6	247.1	268.5	252.6	261	298.6	254.2	312.7	327.2	293.9	400	387	341	13
Os03g0593700	200	183	186	327.6	356.2	256.9	147	198	147	159.5	156.8	169.3	121.91	196.96	158.39	322.4	228.8	243.5	172.8	288.6	204.3	265.4	158.9	174.1	174.3	227.2	228.1	230	217	270	2
Os03g0833100	0	5.59	0	0.821	0	0	2.35	0.63	0	0	0	2.822	0	0.9335	0	0	1.031	0	0	0	0	4.436	0.713	5.056	0	0	0	0	0	0.83	9
Os03g0336100	2.04	0.93	0	0.821	0	0	0	0	0	0	2.193	0	0	0	0	0	1.031	0	0	0	0.862	0	0	0	0	0	0	0	0	0	19
Os03g0840350	239	254	231	276.7	277.5	327	321	361	318	250	273	340.6	289.55	320.17	270.85	358.2	293.7	298.4	375.3	437	455.3	242.5	287.9	278.1	265.1	243.3	315.8	187	206	216	15
Os03g0624000	0	5.59	0	3.284	0.904	8.65	1.57	1.9	8.29	0	1.096	0	1.0885	3.7338	3.1679	11.94	3.092	7.252	0	3.225	5.173	5.915	5.701	5.056	1.056	2.829	0	2.78	1.91	9.16	6
Os03g0110500	5.1	8.38	2.16	4.926	15.37	6.92	14.1	2.53	1.38	5.173	7.675	2.822	2.177	4.6673	1.5839	0	4.122	1.036	3.716	2.419	2.587	0	0	5.056	4.225	0	2.193	2.78	0	1.67	2
Os03g0588800	0	0	0	0	0	0	0	0.63	0	0	0	0	0	0	0	0	0	0	0	0	0	0	0	0	0	0	0	0	0	0	18
Os03g0856400	788	930	838	957.3	897.6	874.6	953	1073	936	998.4	907.8	1007	937.22	827.97	986	1158	915.1	840.2	972.6	924.8	952.7	803.7	942.1	909.3	991.9	1010	905.7	814	913	940	6
Os03g0170000	0	0	0	0	0	0	0	0	0	0	0	0	0	0	0	0	0	0	0	0	0	0	0	0	0	0	0	0	0.96	0	1
Os03g0381300	1755	1456	1895	818.5	829.8	921.3	640	590	504	2412	2025	2574	2056.2	2059.2	2053.6	2137	1291	1227	1303	1174	1035	495.4	619.3	564.8	2019	1744	2061	1363	1282	1175	3
Os03g0120200	47.9	63.3	73.3	73.89	64.18	73.53	66.5	89.8	59.4	81.91	89.9	98.79	59.869	100.81	88.7	107.5	120.6	98.42	81.75	82.24	117.2	79.11	83.38	99.67	134.2	202.7	197.4	104	102	99.1	11
Os03g0301400	464	460	606	396.5	395	384.1	363	416	393	488.9	538.3	426.2	718.42	620.75	706.43	1218	557.5	537.7	442.2	433.8	382	417	352	430.5	610.6	517.6	519.7	676	637	718	13
Os03g0667500	0	2.79	1.08	4.926	0	3.46	1.57	0.63	2.07	0.862	3.289	4.704	1.0885	1.8669	0	0	1.031	3.108	1.858	0	0.862	0.739	2.851	1.444	0	2.829	0	2.78	1.91	0.83	2
Os03g0111300	3.06	2.79	3.23	0	1.808	1.73	0	0	0.69	0	0	0	6.5311	0.9335	3.9598	0	0	2.072	0.929	0	0	0	0	0	0	0	0	0	0	0	4
Os03g0757200	745	498	667	47.62	46.1	83.91	175	134	101	1147	1159	1799	1066.7	1135.1	1091.3	1099	1626	1105	347.4	173.4	319	63.59	61.29	229	1306	2073	1820	920	1122	978	3
Os03g0333601	0	0	0	0	1.808	0	0	0	0	0	0	0	0	0.9335	0	0	0	0	0	0	0.862	0	0	0	0	0	0	0	0.96	0.83	2
Os03g0272300	324	327	330	259.4	238.6	231	297	274	252	338.8	378.3	384.8	360.3	462.06	409.45	537.3	340.1	337.7	353	280.6	288	241.8	272.2	225.3	356	363	449.5	347	351	377	13
Os03g0434800	0	0	0	1.642	0	0	1.57	0.63	0	0	0	0	0	0	0	0	0	0	0	0	0	0.739	0	0	0	0	0	0	0	0	18
Os03g0211500	81.6	56.8	64.7	99.34	139.2	145.3	52.5	82.9	87.8	89.67	72.36	112	47.895	81.21	47.518	59.7	64.92	96.35	113.3	71.76	50.87	84.29	94.07	70.78	84.51	111.3	168.9	76.8	81.3	66.6	11
Os03g0149700	0	0	0	3.284	1.808	1.73	2.35	3.8	0	0	0	0.941	0	3.7338	0.792	0	2.061	2.072	1.858	0	3.449	0	0.713	2.889	0	4.714	10.96	0	0.96	0.83	11
Os03g0641700	1029	1239	1068	1584	1806	1498	2806	3089	2953	1234	1426	1456	1248.5	1108	1089.7	2520	1120	1213	1808	1793	1777	1775	1922	1952	1258	1121	1057	968	1041	1114	18
Os03g0258500	0	0	0	0	0	0	0	0	0.69	0	0	0	0	0	0	0	0	0	0	0	0	1.479	0.713	0.722	1.056	1.886	0	0.93	0	0	9
Os03g0789466	117	183	144	191.3	185.3	145.3	41.5	62.6	47	75.87	110.7	61.15	93.613	89.611	103.75	179.1	107.2	127.4	103.1	109.7	91.4	136.8	144	102.6	108.8	111.3	116.2	195	176	166	8
Os03g0787100	59.1	60.5	52.8	46.8	29.83	39.79	29.7	24	25.6	31.9	37.28	26.34	37.01	38.272	41.182	35.82	61.83	67.34	39.94	49.18	28.45	53.98	62.71	36.83	49.65	49.97	21.93	58.3	66	47.5	8
Os03g0750800	1760	1698	1733	1546	1425	1504	1386	1529	1446	1814	1787	1813	1944.1	1755.8	1955.4	2973	1724	1736	1305	1469	1454	1523	1673	1473	1969	1964	1965	1849	1662	1868	13
Os03g0761300	6.12	3.73	2.16	53.36	69.61	68.34	31.3	45.5	36.6	6.897	1.096	2.822	9.7967	3.7338	5.5438	0	3.092	1.036	39.94	45.96	56.04	47.32	38.48	34.67	6.338	0.943	0	3.7	7.65	0.83	12
Os03g0185950	3.06	5.59	3.23	4.105	6.328	3.46	10.2	15.8	11.7	0.862	4.386	6.586	5.4426	2.8004	7.1277	11.94	7.214	8.288	1.858	0	1.724	8.133	7.839	12.28	4.225	6.6	6.579	13	4.78	4.17	18
Os03g0208600	9.18	6.52	3.23	47.62	57.85	57.96	47.8	41.1	24.2	2.587	4.386	1.882	2.177	5.6007	2.3759	11.94	0	4.144	48.31	54.02	80.19	22.18	36.34	30.33	2.113	8.486	6.579	9.25	3.83	1.67	10
Os03g0679100	90.7	73.6	100	39.41	41.58	36.33	45.4	55.7	32.5	60.35	81.13	112.9	51.16	90.545	65.733	95.53	85.53	89.1	77.1	62.08	60.36	93.16	91.22	102.6	78.17	125.4	98.68	135	120	70	1
Os03g0321700	7.14	1.86	11.9	5.747	13.56	5.19	5.48	8.86	2.76	6.035	1.096	3.763	7.6196	3.7338	3.9598	0	4.122	9.324	7.432	5.644	4.311	6.654	9.264	5.778	4.225	2.829	8.772	4.63	3.83	8.33	2
Os03g0710700	277	172	234	45.15	34.35	51.9	29	32.3	26.9	137.9	126.1	110.1	115.38	106.41	125.13	334.3	287.5	224.8	84.53	72.57	101.7	39.93	37.06	58.5	128.9	167.8	162.3	155	90.9	117	13
Os03g0606100	21.4	25.1	23.7	28.73	22.6	30.28	40.7	36.7	46.3	14.66	25.22	26.34	19.593	19.602	17.423	11.94	26.79	29.01	31.58	20.16	60.36	42.88	39.91	52.72	29.58	24.51	70.17	16.7	24.9	27.5	11
Os03g0322200	58.1	44.7	59.3	48.44	42.49	51.9	11.7	20.9	23.5	36.21	30.7	18.82	30.479	28.937	47.518	35.82	45.34	44.55	41.8	41.93	28.45	29.58	42.76	31.78	26.41	19.8	24.12	25	35.4	27.5	8
Os03g0622600	147	142	124	214.3	200.7	204.2	127	83.5	110	106	74.55	139.2	136.06	125.08	89.492	107.5	105.1	141.9	211.8	238.7	281.9	88.73	93.36	68.61	122.5	146.1	131.6	131	139	98.3	16
Os03g0827700	8464	8014	8850	14110	15658	12948	####	####	####	8291	7809	10821	11421	9886.2	8568.3	15452	9492	10190	14360	14581	14571	6789	6987	7487	8034	8639	7552	7687	8690	8354	10
Os03g0699200	900	895	861	1577	1614	1356	1989	1855	1780	909.6	871.6	963.4	975.31	1001.6	844.24	1576	718.3	869.2	1247	1197	1198	1463	1335	1342	776.4	748.6	809.2	668	753	687	12
Os03g0587250	2.04	12.1	6.47	34.48	50.62	39.79	41.5	39.2	42.8	11.21	6.578	12.23	11.974	3.7338	10.296	0	8.244	6.216	26.01	29.83	47.42	26.62	22.09	37.56	4.225	1.886	8.772	2.78	3.83	1.67	12
Os03g0710550	7.14	12.1	4.31	9.852	9.944	6.92	0	0	3.46	13.79	14.25	4.704	13.062	18.669	19.007	0	13.4	13.47	15.79	17.74	25.87	3.697	2.851	8.667	9.507	13.2	4.386	25	12.4	6.66	16
Os03g0569000	3.06	2.79	0	2.463	2.712	3.46	3.91	7.59	3.46	9.484	17.54	18.82	7.6196	4.6673	7.9197	0	11.34	5.18	6.503	7.257	6.036	9.612	6.414	6.5	14.79	21.69	10.96	22.2	5.74	11.7	20
Os03g0748900	187	169	190	125.6	120.2	113.3	117	173	155	84.49	99.77	82.79	84.905	126.02	132.26	274.6	196.8	170.9	93.82	93.53	73.29	193.7	182.4	150.9	99.3	118.8	136	142	136	138	6
Os03g0350100	806	653	698	1754	1697	1711	939	914	862	769.9	531.8	922.9	697.74	910.12	592.39	1134	765.7	726.3	1818	1823	1755	639.6	747.6	573.5	485.9	725	763.1	660	654	548	10
Os03g0285300	111	192	168	143.7	158.2	76.12	172	261	223	238	178.7	284.1	90.347	147.49	74.445	167.2	72.14	77.7	75.25	58.86	36.21	40.67	77.68	45.5	117.3	87.68	72.37	29.6	32.5	25.8	7
Os03g0110600	458	360	352	118.2	105.8	172.1	108	104	138	195.7	169.9	193.8	211.17	220.29	221.75	214.9	270	319.1	143.1	151.6	167.3	159.7	198.1	166.8	235.6	219.7	219.3	215	186	152	14
Os03g0395700	169	221	209	158.4	177.2	250.9	362	261	290	200.9	185.3	192.9	199.2	193.22	247.09	346.3	156.6	174.1	236	186.3	155.2	208.5	236.6	264.3	178.5	188.6	162.3	211	171	192	18
Os03g0680200	0	0.93	1.08	0	0.904	0.865	0.78	3.16	2.07	2.587	0	0	0	0	0.792	0	0	0	2.787	0	0.862	0	0	2.167	2.113	4.714	0	0	0	0.83	15
Os03g0307000	2051	1615	1499	288.2	337.2	362.5	386	372	255	480.2	457.2	416.8	412.55	477.93	416.57	1481	1368	1159	278.7	317.7	257.8	241.8	309.3	292.5	416.2	394.1	489	425	394	474	14
Os03g0622500	1675	1841	1599	1152	1273	1279	696	805	807	837.2	891.4	482.6	781.56	841.04	1235.5	2412	1838	1771	654	912.7	871.7	1820	1916	936	1356	1090	1072	2209	1515	1584	8
Os03g0213500	348	387	374	449.9	479.1	444.6	484	540	467	300	384.8	350.9	362.48	332.31	463.3	441.8	388.5	380.2	389.2	491.8	446.6	413.3	363.4	408.1	368.7	330.9	241.2	337	365	391	12
Os03g0774400	355	355	381	376.8	369.7	409.2	301	347	328	306.9	335.5	375.4	289.55	336.98	257.39	716.5	385.4	443.4	455.2	449.1	382.8	324.6	207.4	322.1	348.6	369.6	353.1	262	339	302	6
Os03g0660300	0	0.93	0	0	0	0	0	0	0	0	0	0	1.0885	0	0	0	0	0	0	0	0	0.739	0	0.722	0	0	0	0	0	0	9
Os03g0215900	139	109	123	280.8	385.1	262.1	289	326	312	171.6	184.2	190	191.58	145.62	163.94	250.8	78.32	98.42	330.7	355.6	297.5	178.2	178.2	237.6	151.1	142.4	125	104	128	122	12
Os03g0257700	0	0	0	2.463	0	0	0	0	0	0	0	0	0	0	0	0	0	0	0	0	0	0.739	1.425	1.444	0	0	0	0	0	0	17
Os03g0844000	169	270	242	458.9	536.1	437.7	317	438	391	206.1	307	229.6	240.56	132.55	169.48	262.7	107.2	160.6	310.3	495.1	332.8	430.3	367.7	336.6	197.5	76.37	54.82	96.2	103	142	12
Os03g0350300	854	896	749	1591	1638	1644	1798	1774	1772	903.6	777.3	1326	1038.4	1151.9	759.5	1660	828.5	799.8	1608	1372	1484	1156	1249	1067	745.8	890	980.2	754	793	652	12
Os03g0685800	127	167	150	174	182.6	176.5	114	176	130	134.5	128.3	104.4	127.36	122.28	137.01	226.9	120.6	143	114.3	96.75	109.5	114.6	130.4	144.4	133.1	113.1	103.1	136	140	164	6
Os03g0390200	637	608	714	393.2	418.5	335.6	522	642	558	464.7	558.1	457.2	653.11	612.34	611.4	1015	678.1	676.5	496.1	440.2	461.3	440.7	535.9	516.4	587.3	479.9	464.9	657	617	690	13
Os03g0130400	987	913	935	803.7	784.6	891.9	1484	1253	1389	973.4	990	1322	805.5	1159.3	860.08	1493	1158	933.5	1060	984.5	1123	1436	1291	1427	962.3	1188	1425	940	923	887	5
Os03g0560050	0	1.86	2.16	0	0	0	1.57	0	1.38	0.862	0	0	0	0	0	0	0	0	0	0	0	0.739	1.425	1.444	0	0.943	0	0	0	0	9
Os03g0387900	####	####	####	2506	2076	2946	18.8	16.4	18.7	15499	18481	14056	19443	18421	21922	34605	18935	18503	3177	3372	3148	30.31	37.77	25.28	19668	18565	16193	####	####	####	13
Os03g0810800	83.6	69.9	63.6	63.22	70.51	64.01	110	118	65.6	84.49	97.58	62.09	57.692	78.41	68.901	47.76	82.44	63.2	83.61	62.08	110.4	56.93	74.83	74.39	82.4	62.23	87.72	71.2	76.5	70.8	15
Os03g0651100	347	303	300	406.4	448.4	409.2	253	223	238	264.7	281.8	186.3	297.17	313.64	331.83	370.2	311.2	270.4	406	334.6	388.9	252.9	282.9	239.8	245.1	264.9	256.6	318	316	355	10
Os03g0675000	6.12	6.52	3.23	3.284	6.328	3.46	5.48	5.69	12.4	4.311	8.771	12.23	5.4426	4.6673	13.463	11.94	7.214	3.108	0.929	1.613	5.173	5.915	5.701	5.778	6.338	11.31	0	3.7	1.91	11.7	5
Os03g0801300	2.04	6.52	3.23	16.42	14.46	16.44	11.7	13.9	22.8	4.311	2.193	0.941	4.3541	6.5342	1.5839	11.94	2.061	1.036	13.01	12.9	36.21	7.394	2.851	10.11	2.113	1.886	0	2.78	6.69	3.33	12
Os03g0764150	1.02	2.79	0	0	0	0.865	0	0.63	0	0	0	0	0	0	0	0	1.031	0	0	0	0	0	0.713	0	0	0	0	0	0	0	14
Os03g0152600	879	904	1064	475.3	427.6	547.6	1264	1438	1440	1384	1352	1240	1766.7	1684.9	1664.7	1875	1471	1298	642.8	620	633.7	1047	1150	1373	1876	1648	1450	1550	1362	1795	3
Os03g0748000	2145	1774	2205	2623	2669	2530	1258	1224	1341	1930	1884	2075	2874.8	2629.5	2423.4	3738	2444	2220	2218	2417	2606	1379	1378	1133	2118	2186	1910	2116	2117	2261	13
Os03g0758400	289	309	335	514.8	604.8	433.4	466	398	446	304.4	365.1	395.1	407.11	300.57	292.24	537.3	310.2	375	573.2	587.8	535.4	355.6	290.8	352.5	299	339.4	388.1	277	251	287	10

Os03g0314800	245	177	242	142	110.3	117.6	99.4	79.7	74.6	180.2	129.4	179.7	340.71	302.44	277.98	585.1	313.3	258	150.5	197.5	155.2	96.86	91.22	108.3	243	246.1	197.4	311	296	303	13
Os03g0328450	238	222	234	184.7	166.3	167.8	193	195	171	194.9	218.2	106.3	231.85	179.22	256.6	250.8	202	188.6	159.8	159.6	131.1	172.3	198.1	193.6	178.5	138.6	160.1	234	281	279	8
Os03g0617500	7.14	14	15.1	58.29	39.77	32.01	33.7	20.9	13.1	28.45	26.31	38.57	6.5311	11.201	8.7116	11.94	5.153	5.18	31.58	37.9	34.49	32.53	23.52	52.72	8.451	24.51	32.89	7.4	10.5	7.5	12
Os03g0111700	440	536	487	522.1	499.9	460.2	276	343	279	324.2	417.7	293.5	415.81	378.98	408.65	680.6	432.8	476.6	374.4	456.4	319	430.3	329.2	335.1	403.5	341.3	293.9	560	528	542	8
Os03g0782500	3543	4191	3830	995.8	881.4	906.6	137	101	176	7032	9333	9719	7314.9	6497.8	8057.5	6221	3896	4677	1050	858.7	1032	146.4	127.6	108.3	9396	9191	7467	5707	6293	6600	3
Os03g0203700	86.7	59.6	56	29.56	14.46	52.77	33.7	29.1	40.8	55.18	27.41	47.04	19.593	32.671	18.215	131.4	99.96	58.02	20.44	20.16	7.76	43.62	57.01	38.28	41.2	54.68	100.9	21.3	40.2	25.8	13
Os03g0659266	0	0	1.08	0	0	0	0	1.27	0	0	0	0	0	0	0	0	0	0	0	0	0	0	0	0	0	0	0	0	0	0	5
Os03g0143300	801	920	915	883.4	967.2	835.6	870	949	926	865.6	882.6	826	866.46	909.18	883.83	1409	789.4	846.4	894.6	753.1	778.6	892.4	1075	878.2	876.8	940	868.4	882	853	976	6
Os03g0305550	0	0	0	0	0	1.73	0	1.9	0.69	0	0	0	0	0	0.792	0	0	0	0	0	1.724	1.479	0.713	0	0	0	0	0.93	0.96	0	17
Os03g0366700	22.4	26.1	25.9	32.02	38.87	27.68	30.5	27.2	26.9	29.31	32.89	24.46	30.479	18.669	29.303	83.59	22.67	42.48	28.8	36.28	31.9	36.23	24.23	36.11	42.25	36.77	39.47	28.7	35.4	24.2	6
Os03g0743800	0	0	0	1.642	0	0	0	1.27	0	0	0	0	1.0885	0	0	0	0	1.036	0	0.806	2.587	1.479	1.425	1.444	0	0	0	0	0	0	9
Os03g0366000	373	221	307	344.8	341.7	357.3	137	119	130	352.6	267.5	394.2	449.56	491	351.63	191.1	256.6	211.4	521.1	499.1	545.8	106.5	96.21	79.45	219.7	285.7	331.1	228	270	210	16
Os03g0299200	94.8	117	77.6	7.389	5.424	5.19	5.48	12.7	13.1	13.79	13.16	8.467	4.3541	1.8669	10.296	35.82	65.95	30.05	5.574	11.29	12.07	19.22	19.24	57.78	6.338	1.886	21.93	4.63	4.78	5	14
Os03g0297900	0	0.93	0	0	0	0	0	0	0	0	0	0	0	0	0	0	0	0	0	0	0	0	0	0	0	0	0	0	0	0	14
Os03g0762900	17.3	23.3	22.6	65.68	68.7	83.04	153	148	142	6.035	14.25	9.408	17.416	15.869	15.839	35.82	26.79	36.26	101.3	82.24	93.12	73.94	94.78	98.95	16.9	17.91	28.51	21.3	16.3	20.8	12
Os03g0648250	1.02	0	0	0	3.616	0.865	1.57	0	1.38	0	2.193	0	1.0885	1.8669	0	0	1.031	1.036	0.929	0	0	0.739	0	0	1.056	0.943	0	0.93	0	0	2
Os03g0335600	311	360	314	356.3	358	373.7	404	357	348	247.4	293.8	292.6	230.77	301.5	260.56	429.9	338	347.1	396.7	348.3	385.4	380.8	346.3	356.1	293.7	317.7	331.1	285	341	293	12
Os03g0152100	187	125	180	83.74	65.09	86.5	62.6	67.7	71.9	108.6	115.1	93.14	150.22	216.56	149.68	214.9	204	209.3	103.1	91.11	104.3	102	84.09	69.34	145.8	104.7	160.1	175	149	164	13
Os03g0114200	147	167	176	174	188	190.3	311	290	372	214.7	156.8	164.6	174.16	199.76	190.07	274.6	197.9	187.5	244.3	228.2	165.5	289.1	289.3	234.7	155.3	147.1	195.2	175	145	163	18
Os03g0729800	0	2.79	0	0	0.904	0	0.78	1.27	0	0	0	0.941	0	0	0.792	0	1.031	1.036	0.929	0	0.862	1.479	0.713	0	1.056	0	0	1.85	0.96	0	1
Os03g0416400	5.1	2.79	1.08	2.463	0.904	0.865	10.2	3.16	6.22	0.862	2.193	8.467	0	2.8004	0	0	5.153	7.252	7.432	5.644	0	9.612	9.264	10.11	1.056	1.886	2.193	7.4	1.91	2.5	9
Os03g0293000	1053	749	1027	97.7	87.69	125.4	41.5	63.9	43.5	1336	1400	1153	794.62	663.68	1027.2	1505	667.8	627.8	197.9	184.6	124.2	36.97	62	57.78	1281	974.9	1300	797	737	612	3
Os03g0406900	8.16	14	3.23	0	1.808	0.865	1.57	0	2.07	8.622	6.578	3.763	14.151	8.4011	4.7518	0	3.092	9.324	0	0	0	3.697	2.138	1.444	3.169	14.14	6.579	10.2	14.3	5.83	3
Os03g0861400	2.04	3.73	8.62	3.284	0	0.865	0	0	0	0.862	6.578	7.527	0	0	0	0	3.092	4.144	2.787	0	0	0	0	2.167	2.113	1.886	0	0	0.96	0	14
Os03g0717700	1576	1216	1545	728.2	685.2	689.4	427	433	356	1208	1328	1457	1581.6	1478.6	1491.3	2806	1569	1508	818.4	774	833.8	471	478.2	577.8	1492	1410	1340	1526	1426	1554	13
Os03g0217200	433	455	433	503.3	510.7	551.9	485	421	426	375.9	316.9	326.5	433.23	389.25	431.62	764.2	465.8	420.6	427.3	394.3	419.9	512.4	479.6	505.6	460.6	375.3	438.6	426	427	496	6
Os03g0120800	0	0	2.16	2.463	0.904	1.73	3.13	0.63	0	0	0	0	0	1.8669	0	0	0	0	0	2.419	2.587	1.479	1.425	3.611	0	3.771	0	0	0	0	9
Os03g0769900	427	346	346	299.7	245	300.2	421	438	493	315.6	262	318.9	412.55	340.71	345.3	441.8	386.4	330.5	316.8	264.5	281.9	301.7	302.9	380.6	296.8	317.7	342.1	286	269	333	5
Os03g0623100	0	0	0	3.284	1.808	1.73	0	0	0	0	0	0	1.0885	0.9335	0	0	0	0	3.716	0	0	0	0	0.722	0	0	0	0	0	0	10
Os03g0102600	38.7	47.5	52.8	348.9	416.7	404	6.26	8.22	8.29	42.25	18.64	31.05	40.275	34.538	26.927	59.7	43.28	46.62	282.4	305.6	351.8	5.176	3.563	2.889	17.96	23.57	17.54	30.5	35.4	30.8	10
Os03g0598900	1097	1192	1296	871.1	932	846.9	841	958	831	1302	1312	1092	1062.4	1051.1	1174.5	1349	1204	1176	998.6	947.4	887.2	855.5	1015	906.4	1133	1044	1210	1162	1131	1117	3
Os03g0136900	1255	1182	1013	1350	1422	1420	1426	1287	1623	1150	988.9	1417	942.66	1102.4	959.07	1528	995.5	1004	1717	1455	1532	1151	1303	1176	999.3	1028	1340	989	1040	873	12
Os03g0730800	633	667	636	716.7	706.9	861.6	616	626	624	538	580	505.2	533.37	524.6	510.03	1063	558.5	595.7	585.2	727.3	705.3	771.2	672	668.8	527.1	524.2	464.9	572	594	671	6
Os03g0664400	0	0	0	0	0	0	0	0.63	0	0	0	0	0	0	0	0	0	0	0	0	0	0	0	0	0	0	0	0	0	0	18
Os03g0192400	1389	1215	1349	862	841.6	903.1	889	805	916	1245	1140	1649	1463	1586.9	1317.8	2042	1606	1439	1195	1092	1209	699.5	768.9	821.2	1386	1482	1515	1234	1389	1244	13
Os03g0218500	680	375	526	156	130.2	147.1	105	81	104	550.9	474.7	481.7	1246.4	822.37	742.86	632.9	303	408.2	78.96	109.7	138.8	58.41	73.4	41.89	508.1	354.5	480.2	299	303	297	3
Os03g0140650	21.4	10.2	16.2	32.02	39.77	18.17	29	23.4	26.9	11.21	21.93	12.23	51.16	32.671	37.222	83.59	13.4	18.65	13.01	11.29	26.73	15.53	10.69	24.56	9.507	15.09	26.32	18.5	10.5	14.2	6
Os03g0130700	93.8	96.9	95.9	88.67	105.8	109.9	70.5	64.5	69.1	93.12	97.58	65.86	85.993	106.41	72.861	119.4	99.96	89.1	48.31	92.72	95.71	113.1	81.95	91	68.66	96.17	105.3	118	97.5	111	8
Os03g0314200	427	389	395	292.3	269.4	359.9	255	264	256	285.4	285.1	248.4	226.41	262.3	281.15	382.1	349.4	359.5	223.9	210.4	217.3	343.8	401.9	331.5	306.3	316.8	372.8	301	315	324	14
Os03g0701900	2377	2178	2251	2431	2409	2576	2878	2722	2609	2338	2453	2622	2473.1	2552.1	2331.5	3976	2362	2537	2849	2953	2627	3106	2632	2550	2559	2476	2467	2406	2690	2359	6
Os03g0222500	0	0	0	0	0.904	0	1.57	1.27	0	0	0	0.941	0	0	0	0	0	2.072	0.929	0.806	0	0.739	2.138	0	2.113	0	0	2.78	0	0	1
Os03g0800200	2.04	3.73	2.16	13.96	9.944	5.19	0	4.43	2.07	1.724	4.386	0	1.0885	0	3.1679	0	5.153	1.036	0	3.225	1.724	2.218	5.701	3.611	8.451	2.829	4.386	12	10.5	6.66	2
Os03g0718300	11.2	7.45	4.31	12.31	4.52	18.17	0.78	4.43	1.38	6.035	4.386	0	1.0885	8.4011	7.1277	11.94	3.092	2.072	3.716	6.45	0.862	7.394	7.839	2.889	7.395	4.714	0	3.7	7.65	5	8
Os03g0254250	552	552	581	67.32	51.53	66.61	144	120	57.4	620.8	699.5	416.8	708.63	689.82	577.34	1397	1013	701.4	188.6	212.1	144.9	39.19	53.45	61.39	662.3	820.3	813.6	933	901	949	13
Os03g0366100	0	0	0	0	0	0	0	0	0	0	0	0	0	0	0	0	0	0	0	0	0	0	0	0.722	0	0	0	0	0	0	9
Os03g0195450	127	98.7	117	59.11	44.29	39.79	107	50.6	55.3	139.7	110.7	185.3	77.285	84.011	87.116	83.59	72.14	120.2	114.3	95.14	67.25	79.85	57.01	93.17	116.2	116.9	186.4	69.4	61.2	70	20
Os03g0620400	62.2	62.4	52.8	60.75	63.28	58.82	50.1	43.6	38	43.97	49.34	39.51	38.098	55.074	47.518	83.59	45.34	68.38	48.31	60.47	56.91	79.11	77.68	64.28	44.37	52.8	65.79	72.2	73.6	51.7	8
Os03g0737000	1021	1500	888	3129	3648	2831	4312	4178	3895	2008	2236	2057	1280.1	1057.6	1082.6	871.7	840.9	969.7	2318	2273	2404	3198	2745	3096	1474	1176	1485	639	598	851	12
Os03g0265300	318	354	307	353	281.1	401.4	291	276	239	407.8	316.9	292.6	262.33	327.64	330.25	382.1	354.5	322.2	390.2	426.5	477.7	383	483.2	506.3	340.1	386.6	464.9	395	296	295	9
Os03g0387100	681	720	653	894	898.5	901.4	1254	1113	1204	902.7	853	898.5	755.43	857.84	738.9	1182	713.1	672.4	1075	787.7	932.9	838.5	865.1	936	788	826.9	739	636	602	640	18
Os03g0135100	0	0	0	0	0	0	0	0	0	0	0	0	0	0	0	0	0	0	0.929	0	0	0	0	0	0	0	0	0	0	0	16
Os03g0370200	58.1	81	69	78.81	91.3	67.47	47	55	47	71.56	72.36	42.34	92.524	59.741	87.116	191.1	72.14	70.45	25.08	28.22	35.35	99.08	83.38	75.11	63.38	55.63	35.09	54.6	79.4	70	6
Os03g0261800	273	213	291	135.5	120.2	220.6	228	337	240	177.6	137	212.6	213.35	361.25	264.52	465.7	355.5	302.5	157.9	166.1	114.7	225.5	269.4	187.1	142.6	210.3	254.4	372	432	337	13
Os03g0678950	9.18	4.66	7.54	4.105	3.616	11.25	5.48	5.06	8.29	7.76	3.289	14.11	14.151	12.135	10.296	35.82	8.244	8.288	5.574	8.063	6.898	8.873	1.425	5.778	15.85	8.486	6.579	13.9	11.5	6.66	13
Os03g0328000	578	870	735	611.6	652.7	636.7	398	347	253	524.2	629.3	418.7	652.02	568.47	599.52	1111	640	653.7	339.1	401.5	364.7	223.3	292.9	209.5	630.6	569.5	401.3	824	779	861	13
Os03g0859700	274	266	297	126.4	150.1	129.8	38.4	58.2	53.9	143.1	93.19	99.73	222.06	176.42	194.82	692.6	150.5	185.5	51.09	51.6	58.63	88.73	81.24	59.95	160.6	109.4	122.8	188	143	202	13
Os03g0848400	44.9	42.8	17.2	22.17	25.31	48.44	43.8	34.8	25.6	12.93	32.89	29.17	15.239	35.471	19.799	0	27.82	23.83	41.8	31.44	51.73	25.88	51.31	52.72	16.9	41.49	46.05	28.7	24.9	20	15
Os03g0344232	0	4.66	0	4.926	1.808	4.325	5.48	11.4	8.29	0	7.675	0	2.177	0	0	0	0	1.036	1.858	3.225	2.587	2.958	3.563	5.778	4.225	0	0	1.85	0	0	18
Os03g0626700	153	143	140	30.38	23.5	77.85	19.6	26.6	15.2	190.5	139.2	139.2	131.71	90.545	190.86	83.59	94.81	155.4	25.08	7.257	13.8	15.53	134	28.17	140.5	135.8	129.4	104	151	136	3
Os03g0842400	53	47.5	59.3	77.99	74.13	96.02	50.1	67.7	71.2	41.38	55.92	39.51	47.895	62.541	44.35	167.2	45.34	53.87	78.03	87.08	45.7	76.9	65.56	57.78	34.86	27.34	41.67	44.4	88	49.2	6
Os03g0137800	41.8	30.7	43.1	52.54	48.81	32.87	33.7	39.8	36.6	66.39	39.47	60.21	40.275	30.804	27.719	0	22.67	27.97	49.23	62.08	39.66	25.88	30.64	42.61	24.3	28.29	72.37	24.1	24.9	23.3	15
Os03g0371300	433	571	488	646.1	731.3	538.9	74.4	91.1	90.5	455.2	463.8	216.4	695.56	368.71	570.22	597	341.1	399.9	254.5	348.3	246.6	134.6	136.1	80.17	393	254.6	190.8	427	421	656	8
Os03g0856000	221	311	223	454	427.6	483.6	170	170	191	119.8	159	135.5	119.74	152.15	167.9	429.9	225.7	240.4	377.2	400.7	375.1	732	888.7	821.2	217.6	190.5	261	328	327	284	17
Os03g0852800	0	0	0	0	0.904	0.865	0	0	0	0	0	0	0	0	0	0	0	0	0	0	0	0	0	1.444	0	0.943	0	0.93	0	0.83	9
Os03g0231900	349	287	331	435.9	417.6	435.1	491	447	533	520.8	560.3	444.1	521.4	478.86	505.27	549.3	445.2	416.5	557.4	518.4	545.8	380	430.4	450.7	491.2	436.5	467.1	448	418	437	15
Os03g0249100	198	184	142	32.02	24.41	38.93	39.9	32.3	24.2	65.53	59.21	57.39	56.603	60.674	53.854	214.9	296.8	173	26.01	39.51	33.63	59.15	88.37	114.8	106.7	116.9	155.7	132	109	132	13
Os03g0720500	6.12	2.79	4.31	3.284	0.904	5.19	6.26	0.63	5.53	3.449	12.06	3.763	6.5311	2.8004	1.5839	11.94	7.214	3.108	8.361	14.51	2.587	3.697	2.851	6.5	12.68	1.886	0	7.4	4.78	1.67	6
Os03g0339300	0	0	0	0	0	0	0	0	0	0	0	0	0	0	0	0	0	0	1.858	0	0	0	0	0	0	0	0	0	0	0	16
Os03g0159800	243	218	206	201.1	265.8	235.3	314	307	342	207.8	258.7	239	243.83	243.63	252.64	429.9	225.7	229	249.9	228.2	290.6	264.7	230.9	247	226.1	226.3	236.8	238	213	194	6
Os03g0406200	576	621	503	1011	1137	1030	1058	1037	1097	525.1	398	553.2	506.16	659.02	449.84	573.2	491.6	611.3	813.8	805.5	976	905.7	859.4	875.4	413	538.4	548.2	420	432	388	12
Os03g0379700	0	0	0	0	0	0	0	0.63	0	0	0	0	0	0	0	0	0	0	0	0	0	0.739	0	0	0	0	0	0	0	0	9
Os03g0131300	164	205	207	119.9	142.8	129.8	65	73.4	65	128.5	144.7	87.5	167.63	177.36	224.13	238.8	194.8	151.3	85.46	90.3	84.5	101.3	103.3	64.28	165.8	143.3	157.9	212	161	254	13
Os03g0824000	40.8	38.2	35.6	27.91	27.12	33.74	51.7	49.3	52.5	43.97	53.72	32.93	42.452	20.536	38.014	83.59	30.92	22.79	33.44	42.73	46.56	35.49	33.49	38.28	43.31	42.43	50.44	36.1	45.9	38.3	6
Os03g0123600	594	552	548	424.4	374.2	436	846	675	679	799.2	551.5	812.9	500.72	631.95	562.3	1421	531.8	469.3	525.8	417.7	428.5	600.4	632.8	658	572.5	603.4	767.5	538	526	475	6
Os03g0841700	233	198	200	406.4	472.8	381.5	300	253	269	212.1	217.1	220.2	229.68	286.57	226.5	561.2	304	190.6	392.9	391.9	390.6	227	325.7	229.7	217.6	217.8	263.2	219	240	204	10
Os03g0242100	140	119	142	167.5	198.9	181.7	106	105	88.5	115.5	108.5	111	154.57	155.89	156.81	298.5	161.8	158.5	197.9	200	193.1	89.47	111.9	97.5	114.1	97.11	105.3	145	145	112	6
Os03g0138700	132	244	214	977	743.1	1035	548	638	546	269	235.7	241.8	79.462	192.29	172.65	107.5	174.2	176.1	732	720.8	929.5	1839	1808	1912	171.1	270.6	418.8	250	213	176	9
Os03g0803500	80.5	45.6	76.5	20.52	26.22	26.82	83	50.6	101	79.32	63.59	75.27	31.567	39.205	46.726	95.53	47.4	49.73	27.87	33.86	12.93	304.6	136.8	172.6	45.42	49.97	54.82	32.4	46.9	40.8	9
Os03g0592500	####	####	####	16002	14064	13243	131	142	178	65150	67555	93413	49251	46670	52263	59800	45161	53100	20609	17165	22750	43.62	74.83	41.17	53593	63872	49909	####	####	####	3
Os03g0827500	469	506	447	516.4	508	470.6	1033	825	1016	453.5	474.7	376.3	399.49	389.25	444.29	609	468.9	411.3	483.1	484.6	482.8	799.3	642.1	691.2	452.1	418.6	407.9	416	421	498	18
Os03g0412200	793	733	809	885.8	932	813.1	1056	1039	1005	959.6	855.2	813.8	1201.7	913.85	869.58	1242	815.2	838.2	910.4	915.1	869.1	825.2	739	787.2	899	760.9	791.6	797	809	781	6
Os03g0740600	132	143	129	146.1	153.7	172.1	178	158	160	138.8	143.6	176.9	140.42	162.42	160.77	262.7	163.9	163.7	178.4	171.7	180.2	153.1	144	152.4	160.6	175.4	184.2	139	176	163	6
Os03g0117100	####	####	####	11439	12167	10990	####	####	####	17932	19870	18903	20036	18073	19207	24789	16023	15334	12486	11617	13020	17611	15354	20657	18707	17944	16359	####	####	####	5
Os03g0594900	2.04	0.93	3.23	0	0	0	0	0	0	8.622	4.386	0	1.0885	0.9335	1.5839	0	2.061	0	0	0	0	0	0.713	0	2.113	8.486	2.193	1.85	0.96	3.33	7
Os03g0769050	77.5	75.4	77.6	114.9	132	148.8	116	108	132	98.29	97.58	128	91.436	86.811	113.25	250.8	112.3	84.96	125.4	126.6	131.9	125	148.9	101.8	99.3	102.8	120.6	98.1	87	93.3	6
Os03g0278900	####	8959	####	7113	6831	7261	####	####	####	20204	21465	26434	18162	15578	17965	21876	13950	12524	13647	12134	14899	7331	7347	11361	16986	19344	16250	####	####	####	20
Os03g0355100	99.9	119	80.8	140.4	174.5	170.4	89.2	95.5	73.9	103.5	83.33	96.9	118.65	95.212	117.21	238.8	116.5	108.8	97.54	129	125	145.7	131.1	117.7	72.89	110.3	43.86	125	107	134	6
Os03g0586800	905	741	797	1391	1518	1426	828	858	842	757.9	628.2	850.5	666.17	903.58	661.29	847.8	745.1	936.6	1306	1201	1461	863.6	938.5	788	685.6	754.3	782.9	673	647	594	10
Os03g0712800	0	0	0	0	0	0.865	0	0	0	0	0	0	0	0	0	0	1.031	0	0	0	0	0	0.713	1.444	0	0	0	0	0	0	9
Os03g0812400	6.12	4.66	3.23	6.568	11.75	3.46	7.83	26.6	8.98	35.35	12.06	14.11	4.3541	7.4676	11.088	0	3.092	9.324	8.361	2.419	0	3.697	16.39	8.667	6.338	17.91	10.96	0.93	10.5	8.33	7
Os03g0331900	482	369	408	274.2	286.6	281.1	279	290	257	388.8	365.1	414	496.37	431.25	468.05	704.5	464.8	381.3	315.8	274.9	327.6	241.8	245.9	252.1	415.2	435.6	462.7	391	430	428	13
Os03g0321100	48.9	38.2	30.2	23.81	17.18	24.22	21.1	20.2	9.67	56.9	59.21	114.8	52.249	70.942	68.109	0	60.8	35.23	65.03	28.22	62.08	5.176	7.839	17.33	76.06	100.9	96.49	37	23	45.8	20
Os03g0116700	40.8	64.3	35.6	177.3	184.4	233.6	456	481	501	50.01	61.4	66.8	53.337	80.277	87.908	238.8	55.65	74.59	130.1	99.98	93.12	454.7	404.8	333	57.04	80.14	76.75	56.4	75.5	90.8	18
Os03g0226200	0	4.66	2.16	0.821	0.904	0	0	0	0	0	0	2.822	0	0.9335	0	0	0	0	0	0	0	0	0.713	0	0	0	0	0	0	0	14
Os03g0431600	1.02	6.52	7.54	13.14	13.56	5.19	9.39	1.27	1.38	7.76	21.93	20.7	0	1.8669	5.5438	0	3.092	4.144	2.787	8.869	4.311	4.436	3.563	4.333	6.338	21.69	39.47	4.63	14.3	20	11
Os03g0128800	1573	1604	1698	440.9	367	458.5	2418	2853	2723	3306	3462	4005	2802.9	2423.2	2863	3129	2441	2276	858.4	700.7	633.7	1556	1576	2136	3384	3488	2910	2158	2325	2384	5
Os03g0624600	1.02	0.93	0	0	6.328	2.595	0	0	0.69	0	2.193	0.941	0	0	0.792	0	0	0	0.929	0.806	0.862	0.739	1.425	0.722	0	3.771	0	0	0.96	2.5	2
Os03g0115300	104	143	128	229.1	256.7	202.4	153	209	194	62.08	126.1	82.79	129.53	99.879	103.75	143.3	62.86	126.4	130.1	203.2	149.2	213.7	141.1	171.2	96.13	69.77	52.63	84.2	108	125	12
Os03g0112800	78.5	65.2	74.4	115.8	132.9	119.4	159	185	155	70.7	70.17	45.16	33.744	42.939	49.102	143.3	54.62	54.91	91.04	105.6	95.71	177.5	213.1	153.1	71.83	56.57	41.67	56.4	66.9	54.2	18
Os03g0165900	3.06	5.59	0	16.42	14.46	22.49	1.57	6.96	2.76	9.484	0	7.527	3.2656	8.4011	16.631	0	4.122	1.036	7.432	9.675	10.35	2.218	3.563	0	8.451	20.74	10.96	3.7	10.5	5.83	2
Os03g0719400	95.8	76.4	124	96.88	43.39	162.6	30.5	23.4	57.4	125.9	125	130.8	90.347	45.739	67.317	83.59	41.22	61.13	108.7	112.9	130.2	133.1	144	106.9	109.9	98.06	149.1	106	88.9	74.1	11
Os03g0619800	4.08	5.59	1.08	20.52	56.05	10.38	14.9	18.3	40.1	4.311	1.096	1.882	26.124	7.4676	6.3357	23.88	8.244	9.324	10.22	17.74	18.11	7.394	4.988	5.778	6.338	0	0	3.7	3.83	10	12
Os03g0344700	2109	2161	2217	1545	1688	1610	1743	2051	1809	1840	2020	1534	2611.4	2205.7	2486	3964	2132	2129	1325	1643	1438	1951	2035	1909	2468	1938	1621	2248	2468	2434	13
Os03g0210900	111	95	106	74.71	85.88	72.66	170	163	160	137.1	115.1	148.6	133.89	127.88	104.54	83.59	162.8	103.6	167.2	135.5	169.9	111.6	89.79	152.4	107.7	144.3	153.5	74	103	95.8	15
Os03g0262700	44.9	46.6	35.6	27.91	27.12	22.49	86.9	36.1	67.7	75.87	47.14	95.02	40.275	56.941	41.974	47.76	63.89	43.51	18.58	23.38	37.08	56.19	44.9	57.78	41.2	51.86	72.37	43.5	60.2	25.8	5
Os03g0669100	12.2	8.38	6.47	106.7	126.6	148.8	182	132	111	8.622	21.93	2.822	8.7082	20.536	12.671	23.88	11.34	7.252	175.6	153.2	152.6	67.28	81.95	89.56	7.395	3.771	2.193	4.63	8.61	5	12
Os03g0660400	0	1.86	1.08	0	0	0	0	0	0	0	0	0	0	0	0	0	0	0	2.787	0	0	11.83	2.138	6.5	0	0	0	0.93	0	0	9
Os03g0702700	257	202	204	181.4	226.9	198.1	189	157	207	181.1	154.6	191	170.9	163.35	152.85	322.4	206.1	192.7	163.5	187.1	165.5	189.3	148.9	177.7	158.5	185.7	184.2	179	162	176	6
Os03g0131900	18.4	15.8	18.3	32.02	35.25	26.82	29	27.8	52.5	30.18	21.93	32.93	26.124	12.135	17.423	11.94	9.275	32.12	28.8	29.03	26.73	25.88	26.37	33.95	26.41	9.428	19.74	13	14.3	11.7	12
Os03g0726700	0	1.86	0	2.463	0	3.46	0	1.9	0	0	2.193	0.941	3.2656	0	1.5839	0	2.061	0	0	0	0.862	1.479	0.713	0	0	0	0	0.93	0	0	4
Os03g0361100	0	2.79	0	1.642	1.808	5.19	4.7	4.43	3.46	0	0	0	1.0885	0	0	0	3.092	1.036	0	3.225	3.449	1.479	6.414	3.611	2.113	0	0	0	0.96	0	12
Os03g0307400	292	268	365	544.3	617.4	587.4	249	254	281	311.2	288.4	278.5	429.97	373.38	375.39	1302	361.7	396.8	458	630.5	576	224.8	233.7	179.8	312.7	228.2	197.4	343	332	336	6
Os03g0338400	0	0	0	0	0.904	0	0	0.63	0	1.724	0	0	1.0885	0	0	0	0	0	0	1.613	0	0	0.713	0	0	0	0	0	0	0.83	7
Os03g0213200	54	75.4	57.1	123.1	137.4	144.5	107	130	111	49.14	65.78	31.05	59.869	68.142	74.445	35.82	34.01	44.55	120.8	120.1	112.1	94.64	118.3	93.89	41.2	40.54	32.89	56.4	43	45	12
Os03g0437600	719	209	280	372.7	379.7	246.5	68.1	117	350	657	549.3	525.9	686.86	679.55	592.39	656.8	822.4	416.5	310.3	382.2	392.3	369	379.8	392.2	201.8	215.9	526.3	253	612	798	13
Os03g0779500	31.6	34.5	36.6	41.87	39.77	31.14	10.2	22.8	20	32.76	38.37	34.81	27.213	14.935	30.887	23.88	41.22	35.23	27.87	24.99	26.73	36.23	28.51	23.83	33.8	25.46	28.51	36.1	41.1	38.3	8
Os03g0129100	670	675	362	333.3	251.3	366.8	474	581	572	197.4	224.8	253.1	191.58	305.24	243.13	346.3	525.6	477.6	426.4	425.7	523.4	1426	1660	1570	284.2	391.3	557	450	365	367	9

Os03g0341100	34.7	11.2	18.3	14.78	14.46	21.63	12.5	10.1	24.9	25	21.93	34.81	11.974	22.403	7.1277	0	8.244	10.36	26.01	31.44	24.14	13.31	9.264	5.778	16.9	13.2	32.89	12	9.56	9.16	20
Os03g0255900	127	101	85.1	12.31	17.18	12.11	56.4	47.4	52.5	83.63	59.21	42.34	52.249	35.471	45.934	71.65	68.02	68.38	17.65	26.61	12.07	102.8	91.22	98.95	36.97	33.94	30.7	24.1	43	25.8	14
Os03g0431800	1352	1452	1498	1068	1206	1167	756	977	804	1047	1214	879.7	1385.7	1273.2	1415.2	2615	1462	1591	901.1	1013	943.3	1211	1052	944.7	1264	1264	984.6	1702	1500	1757	13
Os03g0215400	90.7	63.3	63.6	18.06	22.6	12.98	61.8	68.9	63.6	73.29	63.59	126.1	76.196	56.007	64.149	23.88	74.2	88.06	13.01	13.71	13.8	25.88	37.06	43.33	77.11	80.14	107.5	71.2	28.7	57.5	20
Os03g0119966	9.18	7.45	11.9	12.31	6.328	13.84	7.05	5.69	5.53	2.587	5.482	5.645	2.177	6.5342	1.5839	35.82	10.31	5.18	9.29	10.48	11.21	15.53	14.25	16.61	6.338	7.543	6.579	15.7	6.69	8.33	6
Os03g0169600	289	591	426	550.9	618.3	583.9	28.2	22.8	20	529.4	632.6	448.8	738.02	748.63	768.21	680.6	539	568.8	476.6	562.8	463	24.4	22.8	18.78	641.2	601.5	530.7	734	697	803	3
Os03g0861800	1778	1840	1869	1578	1574	1557	1610	1524	1556	2280	2339	2243	2431.8	2236.5	2400.4	3021	2093	1965	1594	1719	1671	1550	1398	1613	2291	2216	2246	2192	2096	2119	13
Os03g0297400	374	481	552	409.7	495.4	444.6	575	584	624	262.1	276.3	329.3	352.68	405.12	266.1	656.8	278.2	297.3	483.1	424.9	494.9	408.1	406.2	546.7	244	205.5	309.2	220	277	237	12
Os03g0178066	647	541	577	104.3	52.43	91.7	238	291	256	1073	1003	1469	1014.5	967.99	997.88	597	885.2	783.2	202.5	129.8	170.7	166.4	163.2	256.4	1202	1534	1700	739	895	805	3
Os03g0795400	132	160	131	160.1	125.7	108.1	48.5	75.9	89.1	106.9	104.2	76.21	141.51	113.88	148.1	214.9	131.9	131.6	110.5	107.2	69.84	144.2	128.3	133.6	126.8	95.23	57.02	155	143	185	8
Os03g0751400	364	482	405	1147	1335	1037	1268	1392	1239	272.5	346.5	461	316.76	220.29	192.45	191.1	233.9	352.3	1024	1248	883.8	863.6	706.9	1078	187	186.7	127.2	181	253	241	12
Os03g0698500	1056	1002	990	1304	1353	1208	1435	1595	1343	949.3	910	937.1	1038.4	911.98	1060.4	2149	911	990.5	1393	1388	1356	1153	1140	1242	1031	876.8	881.6	1002	975	1031	6
Os03g0257500	3.06	0.93	0	0	0.904	0	3.13	2.53	0	0	0	10.35	0	0	0	0	0	1.036	0	0	0.862	0	0.713	0.722	0	0.943	4.386	0	0.96	0	20
Os03g0596800	13.3	19.6	18.3	28.73	22.6	11.25	113	112	113	49.14	47.14	31.05	34.833	35.471	31.679	11.94	18.55	29.01	19.51	25.8	26.73	70.98	66.28	93.89	27.47	26.4	28.51	28.7	24.9	35.8	18
Os03g0337200	1121	1038	1178	746.3	800.9	764.7	945	880	1003	1035	1228	1144	1348.7	1150	1336.8	1624	1139	1109	645.6	821.6	656.1	1561	1255	1131	1217	1310	1191	1235	1082	1325	13
Os03g0746600	93.8	103	89.4	146.1	162.7	164.4	149	209	140	103.5	100.9	99.73	80.55	89.611	87.116	167.2	89.66	97.39	129.1	130.6	140.5	136.8	134.7	124.2	106.7	91.46	74.56	114	131	130	12
Os03g0243300	1409	1474	1387	1404	1390	1486	1833	1763	1793	1497	1421	1419	1219.1	1376.8	1445.3	1684	1294	1390	1366	1163	1267	1612	1600	1526	1513	1385	1610	1262	1255	1368	18
Os03g0193000	575	628	639	568.9	593.9	555.4	382	392	336	555.2	525.2	450.7	581.27	456.46	568.63	1134	625.5	667.2	510	518.4	399.2	400.7	398.4	388.6	602.1	480.8	500	604	663	716	13
Os03g0646900	1164	1221	1153	1067	1029	1071	1217	1100	1115	1054	1133	998.2	1156	1048.3	1186.4	1743	1187	1100	982.8	1026	1005	1162	1093	1262	1213	1085	1143	1211	1147	1185	6
Os03g0643250	0	3.73	3.23	2.463	0	1.73	0.78	1.27	1.38	0	0	2.822	1.0885	0.9335	1.5839	0	0	1.036	0	1.613	2.587	2.218	1.425	0	1.056	0	6.579	5.55	0	0	11
Os03g0129900	656	635	683	575.5	555.9	470.6	556	609	641	748.4	712.7	763.9	767.41	663.68	675.55	429.9	510.1	525.3	489.6	367.7	453.5	427.4	426.9	515.7	595.8	612.8	651.3	505	482	527	20
Os03g0184550	7.14	0.93	0	2.463	1.808	0.865	0	0.63	2.07	0	2.193	0	2.177	0.9335	10.296	0	3.092	1.036	0	0	0	5.176	0	0	2.113	0.943	0	3.7	0	3.33	4
Os03g0765400	12.2	11.2	8.62	4629	4979	4394	1838	2158	1957	26.73	36.18	23.52	26.124	25.203	12.671	11.94	6.183	8.288	1909	1927	2141	1465	1348	1379	10.56	8.486	2.193	4.63	8.61	5.83	10
Os03g0643700	0	0	0	0	0	0	0	0	0	0.862	0	0	0	0	0	0	0	0	0	0	0	0	0	0	0	0	0	0	0	0	7
Os03g0224700	596	516	617	363.7	335.4	410.9	314	298	368	378.5	400.2	453.5	537.73	487.26	514.78	1111	608	483.8	384.6	401.5	450.1	442.9	418.3	377	476.4	494	421	425	421	416	13
Os03g0663500	0	0.93	0	3.284	1.808	1.73	3.91	3.16	3.46	6.035	0	0.941	0	0	0.792	0	0	0	0	3.225	1.724	4.436	0.713	2.167	0	0.943	0	0	0	0.83	18
Os03g0368900	0	0	0	0	0	0	0	1.27	0.69	0	0	1.882	0	0	0	0	0	1.036	0	0.806	0	0	0	0.722	0	0	0	0	0	0	20
Os03g0405200	0	3.73	0	0	0	0	3.13	3.8	2.76	0	1.096	0.941	1.0885	0	0.792	0	0	2.072	0	0	0	2.218	2.851	2.889	0	0	0	0	0	0	18
Os03g0734100	12.2	3.73	6.47	2.463	2.712	4.325	2.35	6.33	5.53	4.311	5.482	4.704	4.3541	0.9335	0.792	0	1.031	14.5	0.929	0.806	5.173	2.218	15.68	6.5	0	5.657	8.772	5.55	14.3	4.17	19
Os03g0332100	799	527	400	475.3	256.7	860.7	14.1	23.4	48.4	723.4	760.9	1215	396.22	541.4	416.57	453.8	855.3	690	1060	645	1199	128.7	79.1	105.4	1089	1516	1208	585	672	731	20
Os03g0775000	1112	933	1149	504.9	432.1	506.9	1383	1612	1788	1200	1286	1504	1793.9	1465.5	1349.5	3248	1653	1531	961.5	930.4	924.3	990	1142	1425	1463	1526	1425	1457	1573	1550	13
Os03g0852400	0	1.86	4.31	1.642	1.808	10.38	0	0	0	2.587	0	0.941	0	0.9335	2.3759	0	1.031	0	3.716	0.806	8.622	0	0	0	0	1.886	4.386	0	0	0.83	16
Os03g0689100	2599	3126	2878	811.9	875	733.6	2418	2629	2739	2270	2645	2414	2504.7	2224.4	2358.5	3845	3316	2862	730.2	759.5	688	2685	2391	2621	3043	3221	3138	3303	3035	3697	13
Os03g0374100	388	411	390	486	493.6	480.1	571	613	615	455.2	474.7	446.9	402.75	439.66	488.64	537.3	365.8	367.8	420.8	487.8	507.8	528.7	551.6	574.2	482.8	423.3	386	431	356	377	18
Os03g0214100	2.04	11.2	2.16	0.821	0.904	2.595	0	0	3.46	0	1.096	4.704	3.2656	0	0.792	0	0	1.036	1.858	6.45	4.311	1.479	1.425	2.889	0	1.886	2.193	1.85	3.83	3.33	14
Os03g0247300	172	217	177	265.2	275.7	288.9	492	501	366	272.5	209.4	192.9	249.27	240.83	240.76	334.3	224.7	204.1	226.7	228.2	221.6	379.3	424	358.2	229.2	204.6	206.1	296	262	296	18
Os03g0773000	24.5	18.6	40.9	16.42	7.232	12.11	13.3	22.8	20.7	16.38	9.868	19.76	18.505	10.268	10.296	0	40.19	18.65	24.15	12.09	18.11	9.612	10.69	23.11	19.01	28.29	28.51	10.2	5.74	7.5	14
Os03g0186600	587	633	648	670.7	654.5	632.4	734	736	567	635.4	756.5	535.3	766.32	807.44	876.71	1314	603.9	601.9	667.9	690.2	557	508	638.5	392.9	625.4	438.4	535.1	772	851	876	6
Os03g0412900	591	710	627	828.4	877.8	771.6	734	922	766	514.7	541.6	447.8	716.25	598.34	631.99	979.2	484.3	585.4	688.4	865.1	688	565.6	678.4	543.9	536.6	478	438.6	637	598	653	6
Os03g0423300	64.2	18.6	15.1	13.96	21.7	18.17	0.78	0.63	0.69	198.3	70.17	21.64	321.11	108.28	152.85	35.82	24.73	16.58	5.574	3.225	0	0	0.713	0	33.8	9.428	0	20.4	23	22.5	4
Os03g0797400	59.1	28.9	19.4	307	37.06	370.2	25.8	436	347	119	146.9	14.11	183.96	22.403	22.175	23.88	28.85	31.08	266.6	123.4	265.6	486.5	69.84	114.8	22.18	31.11	149.1	10.2	19.1	8.33	12
Os03g0333000	473	420	445	303.8	264	317.5	215	231	218	331.1	334.4	316.1	323.29	416.32	356.38	788.1	397.8	444.5	317.7	383.8	361.3	221.1	238.7	182	364.4	348.9	377.2	369	361	388	13
Os03g0773300	87.7	66.1	109	37.76	24.41	38.93	27.4	38	29	20.69	10.96	11.29	16.328	14.002	26.927	47.76	18.55	25.9	7.432	12.09	8.622	105.7	96.92	60.67	10.56	10.37	6.579	15.7	20.1	20	17
Os03g0305100	125	143	126	184.7	141	152.2	108	120	106	134.5	155.7	141.1	261.24	168.02	268.48	143.3	134	172	113.3	105.6	111.2	202.6	166	130	171.1	151.8	100.9	184	128	186	4
Os03g0370250	27.5	34.5	28	60.75	38.87	49.31	62.6	64.5	88.5	63.8	73.46	55.51	46.806	42.939	54.646	107.5	50.5	41.44	83.61	74.98	104.3	38.45	39.91	54.89	52.82	45.26	43.86	46.3	44	40	6
Os03g0296200	1.02	0	0	0	1.808	0.865	0	0	0	0	0	0	0	0	0.792	0	1.031	1.036	0.929	0.806	0.862	0	0.713	1.444	0	0	0	0.93	0.96	1.67	1
Os03g0708250	110	105	110	141.2	176.3	127.2	186	174	211	184.5	176.5	202.3	141.51	123.22	148.1	226.9	186.5	127.4	247.1	225.8	219.9	68.02	94.78	116.3	104.6	124.5	142.5	110	124	112	15
Os03g0625800	42.8	32.6	25.9	43.51	58.76	31.14	75.9	58.8	65.6	37.07	24.12	25.4	17.416	33.604	34.847	83.59	29.89	32.12	39.94	43.54	31.9	49.54	64.85	39.72	49.65	30.17	57.02	39.8	26.8	46.7	6
Os03g0209800	0	0	1.08	0	0	1.73	3.13	0.63	0	0.862	1.096	0	0	0.9335	0	0	0	0	0	0	0	0.739	1.425	0	0	0	0	0	0.96	1.67	5
Os03g0568400	0	0	0	0	0	0	0	0	0	0	1.096	0	0	0	0	0	0	0	0	0	1.724	0	0	0	0	0	0	0	0	0	16
Os03g0765000	17.3	4.66	17.2	9.031	11.75	19.03	9.39	5.06	0.69	6.035	3.289	11.29	9.7967	9.3345	8.7116	23.88	22.67	16.58	14.86	20.16	37.08	4.436	5.701	8.667	12.68	16.97	13.16	17.6	15.3	12.5	16
Os03g0363200	39.8	41.9	29.1	49.26	57.85	52.77	172	119	135	61.22	46.05	28.22	48.983	45.739	39.598	59.7	30.92	45.59	66.88	44.35	50.87	79.11	99.06	91.73	29.58	40.54	52.63	26.8	32.5	45	18
Os03g0163100	40.8	30.7	39.9	65.68	99.44	88.23	188	147	180	53.46	53.72	15.05	40.275	41.072	35.638	119.4	48.43	34.19	100.3	95.95	113.8	108	107.6	120.6	28.52	35.83	21.93	27.8	39.2	65.8	18
Os03g0357200	0	5.59	2.16	8.21	17.18	17.3	34.4	39.2	19.3	10.35	16.45	4.704	14.151	10.268	5.5438	11.94	2.061	6.216	6.503	3.225	2.587	16.27	5.701	11.56	3.169	3.771	2.193	3.7	1.91	6.66	18
Os03g0216700	0	0.93	0	0.821	0.904	0	0	0	0	0	0	0	2.177	4.6673	3.1679	0	0	2.072	0.929	0	0	0	0	0	0	0	0	1.85	1.91	0	4
Os03g0647400	11.2	22.4	22.6	61.57	48.81	51.9	22.7	14.5	18.7	11.21	18.64	25.4	13.062	13.068	8.7116	23.88	15.46	7.252	46.45	53.21	47.42	13.31	14.97	17.33	9.507	21.69	17.54	13.9	13.4	10.8	10
Os03g0387300	3.06	7.45	5.39	5.747	7.232	4.325	9.39	11.4	6.22	13.79	4.386	5.645	4.3541	2.8004	5.5438	11.94	6.183	0	4.645	3.225	2.587	5.915	5.701	5.056	3.169	0	6.579	11.1	3.83	4.17	7
Os03g0358975	66.3	72.7	58.2	80.46	73.22	83.91	59.5	60.7	47	27.59	32.89	29.17	38.098	38.272	48.31	35.82	55.65	68.38	34.37	45.15	40.52	98.34	86.94	67.89	48.59	56.57	32.89	74	72.7	66.6	17
Os03g0411000	488	614	614	714.2	791	624.6	484	560	502	555.2	639.2	530.6	579.09	546.07	573.38	1015	501.9	559.5	582.5	566	557.9	530.9	464.6	390.7	494.4	479	429.8	583	507	561	6
Os03g0395000	629	672	659	927.7	1003	754.3	511	653	555	622.5	725.8	920.1	914.36	631.95	529.83	656.8	446.2	616.4	938.2	973.2	1005	485	451.1	459.3	489.1	446.9	399.1	355	382	438	10
Os03g0288000	0	0	1.08	0	0	2.595	0	0.63	0.69	0	0	0	0	0	0	0	0	0	0	0	0	0	0	0	1.056	0	0	0	0	0	2
Os03g0746900	32.6	17.7	22.6	113.3	46.1	197.2	73.6	108	255	20.69	26.31	9.408	44.629	50.406	56.23	71.65	31.95	38.33	149.6	83.05	185.4	170.8	200.3	202.2	32.75	23.57	85.52	37	62.2	50.8	12
Os03g0668033	52	71.7	74.4	103.4	75.93	96.89	131	157	98.8	108.6	115.1	95.96	88.17	91.478	99.788	107.5	71.11	89.1	87.32	87.08	58.63	98.34	103.3	104.7	76.06	70.71	76.75	96.2	96.6	90	18
Os03g0264000	312	257	264	155.2	135.6	160.9	131	131	126	163.8	138.1	147.7	157.84	152.15	156.02	453.8	241.1	188.6	160.7	124.2	134.5	145.7	130.4	135.1	132	127.3	162.3	171	160	164	13
Os03g0196600	324	230	281	147	129.3	184.3	74.4	99.9	83.6	244	304.8	359.4	370.1	268.83	360.34	310.5	457.6	404.1	256.4	336.2	431.1	62.11	78.39	137.9	322.2	525.2	421	227	186	218	3
Os03g0102333	3.06	0	3.23	4.926	5.424	7.785	1.57	1.9	0	0.862	0	0.941	1.0885	1.8669	0	0	1.031	0	0	0.806	4.311	0	2.851	8.667	5.282	2.829	0	1.85	0.96	3.33	2
Os03g0843900	193	208	224	167.5	160.9	189.4	223	229	226	231.1	333.3	274.7	292.81	257.63	261.35	274.6	205.1	205.1	176.5	185.4	211.2	190	209.5	194.3	216.6	247	271.9	235	228	261	3
Os03g0786000	1107	973	996	458.1	466.4	490.5	741	569	726	759.6	705	701.9	780.47	756.1	787.21	525.4	694.6	785.3	480.3	453.1	442.3	657.3	632.1	624	738.4	691.1	653.5	662	550	602	14
Os03g0424500	1630	1351	1369	1756	1803	1950	1836	1539	2060	1729	1589	2478	1776.5	1806.2	1360.6	1660	1376	1428	1990	1900	1908	1692	1607	1735	1514	1774	2079	1169	1324	1101	15
Os03g0118500	2.04	3.73	4.31	3.284	6.328	0.865	8.61	8.86	11.7	4.311	4.386	0.941	5.4426	3.7338	4.7518	0	2.061	5.18	7.432	6.45	7.76	2.958	3.563	1.444	1.056	3.771	10.96	6.48	7.65	11.7	15
Os03g0294800	190	188	221	87.84	72.32	118.5	114	116	149	275	282.9	291.7	296.08	281.9	275.6	525.4	377.2	301.5	118.9	122.6	184.5	181.9	183.1	196.5	412	389.4	302.6	411	305	355	13
Os03g0723600	151	181	150	325.9	321.8	305.4	258	280	238	216.4	218.2	236.1	280.84	237.1	238.38	226.9	187.6	169.9	261	265.3	306.9	263.2	235.9	249.2	209.2	159.3	168.9	236	202	246	10
Os03g0131200	####	####	####	30778	29196	34690	####	####	####	53715	49217	54476	70732	68815	61030	#####	60254	50097	47200	44486	50200	14627	16465	22219	51045	46450	41179	####	####	####	13
Os03g0816000	341	313	300	286.5	318.2	292.4	403	380	316	476.8	571.2	387.6	437.59	429.39	463.3	632.9	350.4	402	373.4	372.5	375.1	319.4	268.7	306.2	419.4	476.1	361.8	488	427	441	13
Os03g0235400	94.8	106	99.1	117.4	111.2	106.4	40.7	36.1	26.3	35.35	70.17	18.82	67.488	44.806	83.948	179.1	68.02	94.28	36.23	44.35	46.56	120.5	88.37	44.78	75	38.66	46.05	122	85.1	124	8
Os03g0341600	79.5	132	105	108.4	9.04	142.7	48.5	5.69	4.15	30.18	19.74	112.9	5.4426	7.4676	29.303	322.4	185.5	115	13.93	29.83	4.311	229.2	53.45	187.8	108.8	282.9	453.9	54.6	176	10.8	11
Os03g0834050	104	82.9	79.7	59.11	35.25	62.28	68.9	108	121	75.87	85.52	89.38	43.541	59.741	63.357	191.1	86.56	141.9	155.1	111.3	39.66	303.9	372.7	199.3	84.51	204.6	342.1	53.7	132	88.3	17
Os03g0355200	6.12	5.59	5.39	4.926	3.616	116.8	142	11.4	3.46	2.587	12.06	7.527	6.5311	147.49	6.3357	0	3.092	5.18	102.2	34.67	6.036	7.394	3.563	2.889	4.225	13.2	4.386	39.8	9.56	5.83	15
Os03g0579000	1418	1216	1560	1486	1560	1359	2000	2228	2238	1634	1711	1792	2861.7	2165.6	2129.6	2293	1608	1381	1785	1929	1816	1223	1298	1678	1786	1530	1436	1544	1553	1598	4
Os03g0101200	3.06	8.38	3.23	2.463	0.904	5.19	3.13	1.9	3.46	3.449	5.482	5.645	7.6196	2.8004	3.1679	0	1.031	3.108	0	0.806	0	2.218	1.425	2.889	5.282	6.6	6.579	5.55	4.78	2.5	20
Os03g0744600	1809	1441	1781	1747	1461	1952	1014	992	1227	1934	1562	2332	1617.5	1909.8	1781.9	3881	2682	2296	2247	2031	2444	1173	1323	1270	2302	2325	2309	2169	1910	1753	13
Os03g0822400	0	0.93	0	0	0.904	0	0	1.27	0	0	0	0	0	0	0	0	4.122	1.036	0	0	0	0	0	0.722	1.056	0	0	0.93	0	0	1
Os03g0131800	0	0	0	0	0	0	0	0	0	0	0	0	1.0885	0	0	0	0	0	0	0	0	0	0	0	0	0	0	0	0	0	4
Os03g0271800	0	0	1.08	0	0	0	0	0	0	0	2.193	0	0	0.9335	0	0	0	0	0	0	0.862	0.739	0	0	1.056	0	0	1.85	0	0	1
Os03g0672400	1.02	0	0	0	0	0	0	0	0	0	0	0	0	0	0	0	1.031	2.072	0	0	0	0	0	0	0	1.886	0	0	0	0	19
Os03g0366200	1349	1302	1171	1074	1068	1202	1424	1293	1363	1170	1151	1232	1277.9	1251.8	1338.4	2388	1465	1288	1097	1325	1287	1309	1330	1406	1339	1311	1215	1434	1416	1410	6
Os03g0554950	63.2	121	110	89.49	93.11	84.77	14.9	16.4	13.8	17.24	39.47	14.11	53.337	55.074	57.022	119.4	52.56	75.63	13.01	39.51	16.38	92.42	80.53	45.5	36.97	31.11	17.54	109	71.7	119	8
Os03g0301200	519	514	371	36.94	19.89	33.74	11	11.4	18.7	34.49	9.868	36.69	30.479	34.538	27.719	155.2	364.8	238.3	28.8	29.83	28.45	62.85	49.17	85.22	21.13	36.77	39.47	38.9	35.4	35.8	14
Os03g0862300	37.7	46.6	35.6	54.18	45.2	71.8	54.8	29.7	38	41.38	39.47	39.51	39.187	43.872	45.142	23.88	47.4	69.41	33.44	48.38	40.52	43.62	34.21	46.95	42.25	44.31	46.05	56.4	48.8	39.2	2
Os03g0281466	0	0.93	1.08	0	0	1.73	0	0	0	0	6.578	2.822	1.0885	0	2.3759	0	2.061	2.072	0.929	2.419	1.724	0	2.138	0	0	1.886	0	1.85	0	1.67	20
Os03g0313000	996	946	981	880.1	1071	978.4	973	902	891	1003	871.6	1025	1045	1128.5	852.16	1851	945	928.3	1135	946.6	1003	703.2	694.8	847.2	903.2	876.8	1079	780	804	841	6
Os03g0769800	132	125	112	246.3	379.7	261.2	687	510	618	80.18	123.9	178.8	84.905	76.543	66.525	107.5	80.38	105.7	252.7	287.8	247.5	382.3	258	395.1	92.96	80.14	111.8	59.2	44	53.3	18
Os03g0435900	1.02	0	0	2.463	0.904	0.865	3.13	2.53	3.46	0	1.096	1.882	2.177	0	1.5839	0	1.031	1.036	1.858	0	3.449	5.176	2.138	2.889	0	1.886	0	1.85	0.96	1.67	18
Os03g0390900	324	352	336	369.4	386	401.4	287	367	340	387.1	410.1	373.5	380.98	357.51	385.69	704.5	468.9	450.7	463.5	491	481.1	456.2	359.2	376.3	468	476.1	458.3	526	429	455	13
Os03g0297700	122	124	124	208.5	167.2	178.2	361	327	370	172.4	110.7	191.9	133.89	124.15	131.47	191.1	107.2	139.9	214.6	171.7	211.2	178.9	194.5	234.7	135.2	145.2	111.8	125	136	94.1	18
Os03g0764100	1501	2263	1149	209.3	75.03	119.4	354	289	379	647.5	444	514.6	419.08	249.23	285.11	2054	2547	1459	167.2	237.9	463	797.8	420.5	2551	364.4	403.5	493.4	253	350	706	14
Os03g0206150	18.4	21.4	38.8	21.35	29.83	38.06	12.5	6.96	5.53	8.622	14.25	15.05	8.7082	17.736	12.671	35.82	16.49	22.79	28.8	20.96	12.07	6.654	4.988	8.667	8.451	6.6	19.74	21.3	31.6	28.3	8
Os03g0766000	419	515	453	610.8	508	606.4	669	569	694	609.6	505.4	509.9	458.27	463.93	402.32	620.9	404	358.5	589	531.3	485.4	676.5	614.3	630.5	454.2	385.6	506.6	389	438	377	18
Os03g0844800	16.3	13	12.9	6.568	6.328	10.38	0	10.8	12.4	19.83	12.06	13.17	8.7082	11.201	12.671	11.94	14.43	14.5	21.37	17.74	5.173	2.958	4.276	10.11	10.56	12.26	21.93	15.7	14.3	9.16	3
Os03g0788600	0	1.86	2.16	1.642	0.904	6.92	3.13	0	0.69	1.724	1.096	0	0	4.6673	1.5839	0	2.061	0	3.716	1.613	5.173	2.218	0	3.611	0	2.829	6.579	1.85	0.96	2.5	11
Os03g0687800	0	0	0	0.821	0	0.865	0	0	0	0	0	0	0	0	0.792	0	0	0	0	0.806	0	1.479	0	0	0	0.943	0	0	0	0	2
Os03g0408300	1523	1221	1253	1666	1635	1688	2271	1586	2241	2131	1708	3197	1915.8	1777.3	1325.8	2937	1109	1250	2432	2062	2243	1090	1177	1442	1502	1872	2079	1033	1199	861	15
Os03g0854600	14.3	22.4	22.6	19.7	8.136	25.95	18	34.8	13.8	12.93	16.45	12.23	18.505	28.937	22.175	11.94	23.7	12.43	7.432	6.45	7.76	24.4	21.38	29.61	12.68	16.97	26.32	26.8	18.2	20	9
Os03g0122100	0	0	0	0	0	0	0	0	0.69	0	0	0	0	0	0	0	0	0	0	0	0	0	0	0	0	0	0	0	0	0	18
Os03g0354500	4.08	6.52	0	0	0.904	0.865	0.78	0	1.38	3.449	3.289	2.822	0	0.9335	1.5839	0	8.244	5.18	1.858	6.45	4.311	1.479	5.701	0	7.395	9.428	4.386	0.93	3.83	2.5	1
Os03g0144700	16.3	33.5	21.6	27.91	40.68	35.47	45.4	51.2	38	22.42	23.02	11.29	26.124	28.004	31.679	71.65	24.73	25.9	27.87	29.03	16.38	38.45	32.07	24.56	19.01	22.63	13.16	25.9	31.6	28.3	6
Os03g0731900	####	####	####	5592	4920	7094	4284	5408	4794	52916	60273	53190	64873	65423	68396	77150	47383	45929	9040	6878	8726	5637	4354	4966	57064	55350	50029	####	####	####	3
Os03g0822000	0	3.73	1.08	0	0.904	0	2.35	1.9	2.07	3.449	0	0	0	0	0.792	0	2.061	0	1.858	0	0.862	0.739	0	0	0	0.943	4.386	1.85	0	1.67	11
Os03g0187550	0	0.93	0	0	0	0	0	0	0	0	0	0	0	0	0	0	0	0	0	0	0	0	0	0	0	0	0	0	0	0	14
Os03g0591000	842	764	897	769.3	857	872	568	657	547	633.7	718.1	723.5	781.56	738.36	690.59	1349	897.6	926.2	720.9	911.9	813.9	893.9	701.2	660.9	867.3	899.5	743.4	970	927	1026	13
Os03g0321800	6.12	11.2	2.16	9.031	9.944	5.19	21.9	11.4	21.4	9.484	5.482	14.11	5.4426	7.4676	5.5438	11.94	3.092	3.108	9.29	12.09	8.622	11.83	6.414	13	14.79	6.6	6.579	1.85	7.65	3.33	18
Os03g0699400	316	296	294	292.3	316.4	320.9	381	380	365	360.4	323.4	406.4	369.01	370.58	348.47	358.2	330.8	302.5	373.4	337	347.5	273.6	292.9	299	316.9	306.4	432	269	258	234	15
Os03g0592100	1.02	0	0	0.821	0	0	0.78	0.63	0	0	0	0.941	0	0.9335	0	0	0	0	0.929	0.806	0	0.739	0	0	0	0	0	0	0	0	15
Os03g0817200	19.4	18.6	6.47	10.67	20.79	9.516	7.83	9.49	18	23.28	12.06	39.51	17.416	12.135	18.215	11.94	12.37	11.4	7.432	5.644	11.21	19.96	19.24	19.5	5.282	19.8	13.16	4.63	1.91	0.83	20
Os03g0663700	175	247	178	512.3	575.8	547.6	135	206	104	121.6	136	190	127.36	118.55	116.42	226.9	197.9	180.3	380.9	328.2	398.3	129.4	210.9	162.5	151.1	156.5	144.7	149	110	123	10
Os03g0310666	20.4	17.7	19.4	18.06	11.75	14.71	14.9	6.33	15.2	10.35	12.06	31.05	29.39	12.135	13.463	71.65	22.67	22.79	22.29	7.257	17.24	10.35	19.95	10.11	16.9	21.69	10.96	25	17.2	19.2	6
Os03g0180900	116	149	75.4	289	43.39	279.4	19.6	81.6	106	20.69	35.08	11.29	54.426	12.135	23.759	131.4	66.98	58.02	94.75	104	106.9	266.2	528.1	327.9	23.24	61.28	217.1	39.8	75.5	33.3	17

Os03g0183300	1144	1468	1181	905.5	791.9	720.6	2263	1719	3023	1276	1135	1205	1246.4	990.39	1168.1	1552	1627	1344	796.1	679.7	1086	3196	3136	3410	1052	1055	927.6	921	969	1215	9
Os03g0263700	1889	1525	1879	763.5	811.8	770.8	979	880	858	1941	1520	1552	1482.6	1627.9	1618.8	2591	1176	1259	969.8	831.3	736.3	664.7	860.9	721.5	1564	1295	1395	1202	1113	1006	13
Os03g0339400	0	0.93	5.39	0	0	0.865	0	0	5.53	0	0	0	0	1.8669	0.792	0	2.061	0	0	0.806	0	0	0	0	0	0	0	0	1.91	0	14
Os03g0211900	194	170	167	431	405.9	493.9	495	400	441	153.5	114	120.4	96.878	136.28	81.572	286.6	139.1	173	442.2	353.2	419.9	432.5	464.6	485.3	94.02	134.8	195.2	186	171	122	12
Os03g0802400	0	0	0	0	0.904	0	0	0	0.69	0	1.096	0	0	0	0	0	0	0	0	0	0	0	0	0	1.056	0	0	0	0	0	7
Os03g0142600	0	0	0	0	0	0	0	0	0	0	0	0	0	0	0	0	0	0	0	0	0	0.739	0	0	0	0	0	0	0	0	9
Os03g0324300	1899	1895	1599	1897	1816	1834	3294	2713	3294	2340	2507	2215	2090	2008.8	2177.1	1767	1916	1921	2241	2164	2472	3460	3249	3546	2206	2421	2649	1955	1917	2108	18
Os03g0618800	1.02	0	0	0.821	2.712	2.595	2.35	0	2.07	2.587	0	0	0	0.9335	0	0	0	3.108	0.929	0	0.862	1.479	0	5.056	2.113	0	2.193	2.78	1.91	2.5	9
Os03g0192100	0	0	0	0	0	0	0	0.63	0	0	0	0	0	0	0.792	0	0	0	0	0.806	0	0.739	0	0	1.056	0.943	0	0	2.87	0	1
Os03g0105000	0	0	0	0	0	0	0	0	0	0.862	0	2.822	0	0.9335	0	0	0	0	1.858	0	2.587	0	0	0	0	0	0	0	0.96	0	16
Os03g0270600	70.3	93.1	115	36.12	50.62	35.47	30.5	47.4	38	56.04	49.34	31.05	65.311	56.941	64.149	59.7	64.92	60.09	25.08	26.61	17.24	68.76	72.69	62.11	73.95	49.97	41.67	67.5	53.6	87.5	14
Os03g0582100	55.1	60.5	46.3	182.3	170.9	192.9	70.5	58.2	58	55.18	61.4	50.8	43.541	57.874	45.142	167.2	41.22	67.34	141.2	158.8	184.5	65.81	62	59.95	54.93	51.86	39.47	47.2	51.6	45.8	10
Os03g0254000	150	162	134	33.66	24.41	47.58	31.3	20.2	57.4	200	216	191	156.75	125.08	145.72	107.5	114.4	82.88	59.45	41.93	37.08	27.36	36.34	55.61	198.6	114.1	155.7	166	112	194	3
Os03g0777500	87.7	63.3	79.7	193.8	171.8	243.9	220	203	196	49.14	58.11	62.09	59.869	50.406	38.014	47.76	68.02	53.87	196.9	181.4	188	167.1	205.2	245.6	32.75	55.63	111.8	70.3	70.8	45.8	12
Os03g0298100	0	0	0	0.821	0	0	1.57	0.63	0	1.724	1.096	0	0	0	0	0	0	0	0.929	0	0	0.739	0.713	0	0	0.943	0	0	0.96	0	7
Os03g0701800	1777	1798	1711	1588	1497	1665	608	439	488	1623	1599	1629	1503.2	1543.9	1663.1	2699	2169	2067	1601	1669	1972	635.1	697	627.6	1973	1948	1726	2129	1812	1928	13
Os03g0158200	531	327	365	202	156.4	178.2	192	161	187	271.6	273	303.9	299.34	333.24	298.57	477.6	325.6	357.4	280.5	249.9	248.3	144.2	148.2	166.1	277.8	264.9	247.8	310	292	247	13
Os03g0809700	140	144	113	129.7	132.9	132.4	134	175	117	158.6	144.7	132.7	174.16	138.15	172.65	298.5	120.6	139.9	104	100.8	93.12	191.5	158.9	150.9	181.7	158.4	118.4	147	180	173	6
Os03g0276700	199	127	149	293.1	297.4	282.9	260	263	294	166.4	159	213.6	215.53	224.03	163.94	322.4	113.4	136.8	358.6	329.8	319	190	213.8	226.8	131	148	129.4	108	106	92.5	10
Os03g0844900	3388	3222	3689	1604	1583	1567	2118	2196	2240	4769	5166	6365	5329.4	4886.6	4947.4	5361	4385	4030	2218	2102	2284	1524	1445	1947	5074	5698	4844	3751	4017	3715	3
Os03g0263900	1380	1338	1274	438.4	358.9	359.9	608	700	673	1472	1468	1715	1288.8	1403	1462	2137	1557	1651	435.7	443.5	461.3	537.5	558.7	743.2	1614	2037	1840	1315	1303	1398	3
Os03g0241600	449	443	477	66.5	61.47	63.15	135	111	144	551.8	573.4	475.1	470.24	428.45	492.6	740.3	482.3	488	71.53	104.8	104.3	143.4	208.8	188.5	529.2	559.1	605.2	540	489	518	3
Os03g0607800	101	85.7	94.8	72.25	79.55	79.58	57.9	50	58.7	107.8	86.62	93.14	129.53	84.944	80.781	167.2	103.1	102.6	72.46	76.6	81.91	59.89	59.15	73.67	82.4	125.4	133.8	87.9	97.5	102	13
Os03g0829200	1.02	0.93	0	0.821	2.712	3.46	8.61	10.8	8.29	1.724	2.193	2.822	2.177	1.8669	0.792	11.94	0	2.072	0	2.419	0	0.739	2.138	2.167	2.113	0	2.193	0.93	0	0	6
Os03g0269300	9.18	3.73	3.23	0	0	0.865	0.78	0	0	0.862	1.096	2.822	0	2.8004	2.3759	0	4.122	1.036	1.858	0	0.862	1.479	2.138	2.167	4.225	4.714	6.579	5.55	0.96	5	19
Os03g0824400	300	262	308	284.1	272.1	311.4	478	450	435	355.2	358.5	337.8	250.36	302.44	308.87	382.1	245.3	285.9	308.4	306.4	298.3	411.8	384.1	392.9	329.6	358.3	372.8	278	318	303	18
Os03g0201100	639	646	580	683.1	733.1	686	575	631	599	649.2	631.5	693.4	646.58	621.68	654.16	1134	679.1	682.7	668.8	614.4	613.9	611.5	647.1	572.7	664.5	711.8	732.4	664	690	725	6
Os03g0126700	0	0	0	0	0	0	0	0	0	0	0	0	0	0	0	0	0	0	0.929	0	0	0	0	0	0	0	0	0	0	0	16
Os03g0364400	604	816	864	1586	1886	1064	2102	2340	2014	1937	1618	901.3	1373.7	939.05	1562.5	1505	558.5	647.5	1086	1388	933.8	1165	1360	1286	1135	656.2	443	723	624	914	18
Os03g0710600	4997	5253	6051	4875	5132	4729	6933	6861	6078	8668	9533	10039	9522.4	7512.4	8148.5	11583	6502	6260	6787	7569	6802	3180	3371	4255	7702	7876	6741	5766	6676	6214	13
Os03g0177900	5072	4086	4071	5097	5122	5587	6626	5037	6339	5466	4661	7790	4632.7	5212.4	3888.6	6866	3862	4414	5916	4777	5473	4691	4616	4747	4335	5530	5919	3318	4015	3098	15
Os03g0325000	0	0.93	1.08	0.821	0	0.865	0	0.63	1.38	0	0	0	0	0	3.1679	0	0	1.036	0.929	0.806	0	0	0	0	0	0	0	0	0	0	4
Os03g0680400	0	0	0	0	0	0	0.78	0	0	0	0	0	0	0	0	0	2.061	0	0	0	0	0	0	0	0	0	0	0	0	0	1
Os03g0599800	3044	2485	2846	1925	1851	2655	2258	2228	2645	2218	1472	1625	1377	1704.5	1591.9	2723	2121	1943	1800	1591	1668	2269	2585	2549	1448	1380	2066	1527	1480	1381	14
Os03g0682200	0	0	1.08	0	0.904	0.865	0	0	0	0	0	0	0	0	0	0	0	1.036	0	0	0	0	0	0	1.056	0	2.193	0	0	0	11
Os03g0353300	1.02	0	0	0	0.904	0	0	0	0	0	0	0	0	0	0.792	0	0	0	0	0.806	0	0	1.425	0	0	0	0	0	0	0	17
Os03g0137200	414	437	478	385	436.6	359.9	867	824	855	486.3	400.2	376.3	433.23	399.52	426.87	370.2	414.3	449.6	346.5	366.1	321.6	1196	962.1	1019	404.6	373.4	475.9	407	454	396	18
Os03g0100400	462	528	481	600.1	562.3	633.2	600	619	646	582.8	629.3	616.2	736.93	585.27	739.7	931.4	522.5	482.8	560.2	548.3	562.2	675.8	701.2	638.5	705.7	658.1	607.4	665	602	643	6
Os03g0276500	####	####	####	875.2	535.1	1284	261	438	424	10859	9627	11105	5381.6	4988.4	6927.3	10341	9643	7490	2337	1633	1383	539.8	597.9	699.9	9300	8694	9309	5357	5105	4299	3
Os03g0263400	646	543	635	282.4	246.8	258.6	352	340	385	587.1	686.3	770.5	789.18	828.9	691.39	1325	915.1	866.1	379	358	358.7	318.7	310	304.8	821.9	905.1	868.4	645	654	695	13
Os03g0332700	1507	1465	1736	1583	1600	1522	1181	1209	1365	1735	1792	1999	2337.1	2045.2	1988.6	1672	1286	1436	1338	1384	1417	1196	1102	1301	1537	1431	1399	1267	1317	1357	4
Os03g0858100	1494	1568	1609	1594	1652	1617	760	760	675	1038	1065	920.1	1250.7	1330.2	1260.8	2197	1544	1525	1226	1404	1510	1129	1002	863.8	1182	1120	984.6	1539	1518	1615	8
Os03g0735200	3.06	0.93	1.08	9.852	21.7	17.3	16.4	12.7	8.98	3.449	2.193	3.763	6.5311	2.8004	1.5839	0	4.122	7.252	15.79	24.99	23.28	5.915	2.851	14.44	2.113	5.657	4.386	0.93	6.69	2.5	10
Os03g0269601	6.12	3.73	6.47	6.568	3.616	4.325	0.78	0	0	12.07	4.386	10.35	1.0885	9.3345	6.3357	47.76	22.67	18.65	0	7.257	3.449	0.739	0	0.722	23.24	22.63	4.386	11.1	12.4	14.2	13
Os03g0245800	3.06	0.93	0	0.821	0.904	2.595	0	0	0	0	0	0	1.0885	1.8669	0	0	0	0	0.929	0	0	0	1.425	0	0	0	0	0	0	1.67	19
Os03g0343300	314	366	301	469.6	462.8	387.5	210	261	301	267.3	308.1	343.4	342.88	305.24	313.62	752.3	274.1	325.3	350.2	383	341.4	323.1	322.8	259.3	338	335.7	302.6	307	320	307	6
Os03g0776400	12.2	14	22.6	7.389	7.232	4.325	23.5	28.5	29	36.21	20.83	31.99	10.885	15.869	11.088	35.82	8.244	9.324	3.716	12.9	9.484	16.27	22.09	15.89	12.68	9.428	26.32	9.25	5.74	7.5	5
Os03g0638800	1257	1216	1217	1079	1115	1085	1568	1437	1564	1480	1433	1586	1560.9	1417.9	1458	1493	1245	1154	1202	962.7	1005	1174	1209	1251	1344	1458	1272	1216	1184	1126	5
Os03g0221150	54	64.3	77.6	79.63	46.1	62.28	115	81	89.1	106.9	120.6	141.1	100.14	107.35	76.029	71.65	89.66	71.49	101.3	113.7	118.1	62.11	71.26	91.73	90.85	95.23	85.52	95.3	101	66.6	20
Os03g0778400	2.04	0.93	0	0	0	0	0	0	0	0	0	0	0	0	0	0	0	0	0	0	0	0	0	0	1.056	0.943	0	0	0	0	19
Os03g0194100	160	158	194	314.4	289.3	331.3	486	311	393	177.6	132.7	199.5	83.816	139.08	106.12	179.1	164.9	180.3	374.4	312.8	275.9	340.1	456.8	416.7	129.9	173.5	186.4	130	181	133	12
Os03g0637900	138	165	94.8	64.86	46.1	105.5	37.6	44.9	36.6	83.63	86.62	108.2	70.754	96.146	59.397	370.2	162.8	122.3	78.96	69.34	62.08	57.67	94.07	119.9	116.2	156.5	138.2	146	112	146	13
Os03g0786700	187	155	189	217.6	255.8	222.3	172	163	162	212.1	159	291.7	199.2	213.76	153.64	310.5	190.6	174.1	284.3	253.2	266.4	152.3	165.3	130	121.5	196.1	208.3	165	182	148	16
Os03g0256400	162	170	139	306.2	335.4	292.4	375	336	296	128.5	125	130.8	114.29	124.15	97.412	179.1	103.1	146.1	297.3	243.5	279.4	244	300	257.1	100.4	121.6	155.7	86	104	74.1	12
Os03g0807000	6.12	15.8	4.31	266.8	277.5	252.6	83	106	107	4.311	8.771	2.822	13.062	1.8669	17.423	0	2.061	6.216	222.9	212.9	210.4	102	118.3	99.67	7.395	3.771	6.579	3.7	1.91	4.17	10
Os03g0718500	0	0	2.16	0	0	0	0	0	0	0.862	0	0	0	0	0	0	0	0	0	0	0	0	0	0	0	0	0	0	0	0	7
Os03g0248600	1471	1650	1742	1616	1934	1559	1822	1879	1445	1133	1210	1034	1090.7	1265.8	1310.7	1994	1180	1224	1627	1260	1424	1149	1378	1219	1085	748.6	831.1	1328	1317	1140	6
Os03g0843000	86.7	86.6	75.4	52.54	60.57	55.36	50.9	61.4	40.1	86.22	108.5	47.98	71.842	82.144	96.62	298.5	79.35	94.28	57.6	62.89	59.49	99.08	80.53	83.78	84.51	70.71	48.24	125	120	100	13
Os03g0811700	945	936	934	1012	1043	942	942	967	971	1054	1044	1003	1210.4	1106.1	1153.9	1063	914.1	823.6	1054	836.9	925.2	825.9	813.1	801	966.6	897.6	1018	941	815	1036	4
Os03g0789600	64.2	51.2	40.9	49.26	52.43	64.88	52.5	66.4	64.3	31.04	30.7	40.46	45.718	49.473	49.102	107.5	52.56	61.13	52.02	62.08	43.97	79.85	86.23	73.67	47.54	35.83	28.51	58.3	47.8	67.5	6
Os03g0250000	1082	924	927	2834	2365	3144	1956	1947	2103	707	782.8	807.2	843.6	916.65	1062.8	1803	1248	1166	3032	3373	3536	2773	2561	2554	1181	1090	1318	1629	1366	1450	10
Os03g0725800	118	106	125	115.8	115.7	134.1	121	63.3	78.1	90.53	86.62	83.73	83.816	112.95	95.828	214.9	109.2	110.9	133.8	104	108.6	93.9	84.09	110.5	85.57	103.7	195.2	126	138	118	6
Os03g0740700	535	518	559	504.9	546.9	542.4	521	552	451	411.3	468.2	422.4	556.23	479.79	565.46	597	597.7	582.3	433.8	489.4	438.9	510.9	514.5	472.3	582.1	481.8	451.7	532	517	635	8
Os03g0755100	90.7	80.1	93.7	81.28	76.84	95.16	231	209	181	141.4	126.1	148.6	105.59	98.946	122.75	119.4	78.32	89.1	143.1	149.2	132.8	99.82	140.4	122.1	94.02	99	125	93.4	127	96.6	18
Os03g0161500	628	595	439	288.2	240.5	246.5	450	345	550	406.1	445.1	640.7	318.94	318.31	312.83	286.6	455.5	398.9	236	204	181.9	559.7	573	613.2	498.6	426.2	443	474	285	412	5
Os03g0266300	561	230	445	77.99	67.8	59.69	28.2	20.9	15.2	716.5	432	904.1	941.57	659.95	659.71	274.6	177.3	197.9	79.89	86.27	68.12	14.79	29.93	15.89	426.8	259.3	372.8	253	244	213	3
Os03g0154100	82.6	75.4	81.9	123.1	120.2	99.48	348	355	418	93.12	77.84	77.15	42.452	56.007	48.31	23.88	30.92	60.09	94.75	132.2	87.08	153.1	156.1	148.8	41.2	52.8	30.7	40.7	47.8	37.5	18
Os03g0758300	242	329	292	206.1	270.3	134.1	285	322	285	220.7	267.5	158.1	297.17	222.16	226.5	179.1	196.8	204.1	115.2	120.9	87.95	105.7	146.1	158.2	187	129.2	85.52	118	178	205	4
Os03g0267500	41.8	34.5	22.6	31.2	9.944	31.14	25.1	10.8	24.9	23.28	17.54	31.99	22.859	22.403	20.591	47.76	72.14	31.08	10.22	20.16	17.24	36.97	85.52	54.17	35.92	54.68	57.02	61.1	35.4	40	1
Os03g0565825	1.02	0	0	0.821	0	0	0	0	0	0	1.096	0.941	0	0	0.792	0	0	0	0	0	0	0	0	1.444	0	0	0	0	0	0	19
Os03g0856500	731	830	533	4401	4115	4445	4834	4677	7731	2647	3287	3696	1034.1	584.34	755.54	513.5	345.2	496.3	2538	2437	2575	6872	4260	6947	1456	1250	1610	181	124	217	18
Os03g0159150	3.06	1.86	0	0	0	0	0	0	0	0	0	0	0	0.9335	0.792	0	0	1.036	1.858	0.806	0	0	0	0	1.056	0	0	0	0	0.83	19
Os03g0767900	232	168	196	252	221.5	234.4	233	161	168	221.6	179.8	277.5	217.7	222.16	211.45	286.6	230.8	219.6	314	261.2	271.6	147.1	139	174.8	233.5	163.1	239	238	201	176	16
Os03g0253701	1.02	1.86	1.08	0.821	1.808	0.865	0	1.27	0	6.035	3.289	0	0	0.9335	1.5839	0	0	1.036	0.929	0.806	3.449	0.739	0	0	1.056	0.943	2.193	1.85	0.96	3.33	7
Os03g0319300	6184	4782	5596	2185	1906	2721	2233	2893	3069	4830	3828	3364	3560.5	3540.6	3705.6	7929	5751	5035	2498	3125	2570	3259	3599	3384	4397	3882	3971	4104	3692	3645	13
Os03g0651300	192	251	207	263.5	302.8	303.6	297	292	241	130.2	138.1	121.4	175.25	140.02	167.1	262.7	180.3	181.3	183.9	207.2	256.1	258	240.9	267.2	191.2	149	164.5	191	175	204	12
Os03g0326100	34.7	47.5	42	39.41	29.83	24.22	14.9	15.8	12.4	29.31	23.02	5.645	14.151	18.669	23.759	83.59	21.64	27.97	17.65	11.29	3.449	68.76	67.7	29.61	22.18	15.09	10.96	31.5	20.1	45	8
Os03g0189100	1362	1297	1016	318.5	301	238.8	427	493	390	542.3	460.5	340.6	438.67	373.38	456.17	788.1	964.6	823.6	265.7	287	292.3	499.1	457.5	607.4	407.8	356.4	370.6	426	424	467	14
Os03g0701300	119	110	131	96.88	129.3	134.1	164	182	139	146.6	142.5	212.6	165.46	168.95	159.19	107.5	150.5	130.5	154.2	170.9	197.4	119.8	121.1	158.2	188	208.4	155.7	189	153	152	20
Os03g0656800	4.08	1.86	1.08	3.284	6.328	5.19	7.05	5.06	13.1	0	1.096	4.704	2.177	2.8004	1.5839	0	3.092	4.144	1.858	0	7.76	8.873	2.138	10.11	5.282	1.886	10.96	0	8.61	3.33	9
Os03g0135300	688	854	897	1938	2138	1675	3571	3455	3439	1096	1218	1415	1169.1	809.3	787.21	1027	511.1	556.4	1554	1615	1472	1945	1915	2573	734.2	623.2	677.6	361	459	500	18
Os03g0208700	2.04	0.93	0	0.821	2.712	1.73	0	0	0.69	3.449	1.096	0	0	0	0.792	0	0	4.144	2.787	2.419	0	1.479	1.425	2.167	0	0	2.193	0	0.96	0.83	7
Os03g0127600	21.4	29.8	22.6	50.9	45.2	47.58	10.2	10.8	8.98	16.38	16.45	6.586	10.885	9.3345	8.7116	0	18.55	26.94	22.29	14.51	19.83	24.4	27.79	12.28	11.62	15.09	8.772	18.5	23	19.2	2
Os03g0784000	2364	2172	2358	1689	1768	1579	1102	1495	1157	1509	1669	1689	1840.7	1721.3	2086	3475	2335	2296	1368	1736	1283	1704	1524	1345	1908	1871	1820	2478	2207	2351	13
Os03g0697833	78.5	79.2	89.4	50.9	64.18	58.82	75.2	55	44.9	101.7	85.52	82.79	71.842	72.809	95.036	107.5	57.71	70.45	38.09	33.06	36.21	53.98	53.45	46.95	78.17	65.06	70.17	74	51.6	77.5	13
Os03g0743500	467	312	445	335	336.3	252.6	535	823	757	356.9	199.5	227.7	168.72	198.83	258.97	131.4	249.4	296.3	275.9	248.3	188	703.9	912.9	710	225	196.1	274.1	133	156	156	18
Os03g0760600	448	489	463	500	478.2	503.5	566	542	548	473.3	500	537.2	567.12	533	511.61	919.5	508.1	434.1	570.4	478.1	430.2	524.2	456.1	444.9	511.3	488.4	521.9	534	449	537	6
Os03g0190301	0	0	0	0	0	0	0	0	2.07	0	0	0	0	0	0	0	0	0	0	0	0	0	0	0	0	0	0	0	0	0	18
Os03g0115000	0	0.93	1.08	0.821	2.712	1.73	3.13	1.9	2.07	3.449	0	3.763	2.177	0.9335	3.9598	0	1.031	0	5.574	0.806	0.862	0.739	1.425	0.722	0	0.943	2.193	0.93	0	0.83	15
Os03g0616700	674	653	663	660.1	721.4	641	560	676	548	673.4	637	599.3	690.12	702.89	752.37	812	608	674.5	554.6	627.3	557	610.7	599.3	525.1	713	653.4	675.4	747	731	668	13
Os03g0784800	658	725	707	876.8	892.2	895.3	1062	1088	1009	856.2	897.9	779.9	817.48	841.04	911.55	1194	614.2	624.7	785.9	885.3	861.4	933.1	959.9	947.6	870.4	747.7	802.6	707	759	742	18
Os03g0836300	161	168	172	177.3	185.3	146.2	353	368	313	303.5	224.8	182.5	163.28	155.89	163.94	274.6	155.6	135.7	132.8	150	121.6	300.2	370.6	277.3	165.8	95.23	120.6	91.6	129	119	18
Os03g0786900	748	903	814	850.5	881.4	808.8	828	874	878	684.6	805.9	571.1	726.04	700.09	797.51	1027	837.8	798.8	628	730.5	701	1012	1038	914.4	807.1	727.9	730.2	896	847	924	17
Os03g0143900	0	1.86	3.23	0.821	4.52	4.325	7.05	1.9	3.46	0	0	0	2.177	0.9335	3.9598	11.94	1.031	2.072	0.929	0.806	0	5.176	6.414	2.889	0	0	2.193	1.85	0	0.83	6
Os03g0307900	35.7	56.8	44.2	128.9	126.6	93.43	50.1	74	49.8	41.38	38.37	51.75	58.78	43.872	40.39	23.88	18.55	39.37	98.47	102.4	82.77	66.54	61.29	62.84	34.86	37.71	26.32	16.7	35.4	35	10
Os03g0802500	42.8	40.1	55	113.3	115.7	67.47	229	173	368	62.08	53.72	51.75	65.311	65.342	46.726	250.8	55.65	25.9	140.3	123.4	71.56	93.16	163.9	83.78	30.63	27.34	28.51	28.7	55.5	19.2	18
Os03g0862100	1144	1322	1224	1777	2035	1684	1582	1524	1502	1314	1176	1224	1326.9	1174.3	1209.3	1588	877	1051	1239	1380	1293	1344	1348	1210	1084	1028	1068	1069	1093	1107	12
Os03g0425200	1822	2035	2004	2444	2663	2266	2746	3309	3065	2147	2323	2238	2226	2229.1	2284	3021	1956	1951	2341	2279	2235	3097	2935	3155	2322	2068	2022	2059	1840	2160	18
Os03g0793100	54	59.6	42	38.59	46.1	53.63	85.3	75.3	69.1	49.14	60.3	42.34	31.567	45.739	53.062	71.65	55.65	46.62	40.87	36.28	37.08	138.3	94.07	115.6	59.16	46.2	37.28	62	86.1	65.8	9
Os03g0314700	1525	1391	1801	1282	1332	1215	975	913	1077	2381	2395	2535	2769.2	2589.4	2562	3893	2016	1767	2108	1711	1956	573	679.9	768.5	2204	1950	1752	1724	1850	1787	13
Os03g0183100	319	462	394	973.7	1251	876.3	1137	1379	1345	590.6	691.8	545.7	394.04	285.64	407.07	537.3	254.5	316	574.1	729.7	612.2	1037	1021	1033	509.2	390.3	322.4	233	237	316	12
Os03g0787000	194	183	135	34.48	47.01	47.58	118	97.4	100	92.25	74.55	60.21	55.515	42.939	47.518	95.53	225.7	148.2	26.94	28.22	52.6	126.4	117.6	219.6	52.82	44.31	24.12	37.9	37.3	60	14
Os03g0762400	0	0	2.16	1.642	3.616	0.865	3.13	1.9	0	1.724	1.096	4.704	0	0	0.792	0	0	0	0	0	0	4.436	3.563	0.722	1.056	0.943	0	0	0	0	17
Os03g0399800	1011	587	748	287.3	205.2	789.8	358	275	142	462.1	288.4	799.7	562.76	1293.8	673.17	1875	1938	1177	987.5	782.9	1255	323.9	808.1	1371	798.6	1042	1445	1391	992	841	13
Os03g0183800	0	3.73	0	0.821	0	0	0	0	1.38	0.862	4.386	0	0	0	0	0	1.031	0	0	0.806	0	0.739	2.138	1.444	0	0	0	0	0.96	0	7
Os03g0757750	4.08	1.86	3.23	0	0	0.865	4.7	4.43	0	1.724	0	0	2.177	1.8669	1.5839	11.94	5.153	1.036	4.645	1.613	0	2.218	1.425	0	0	0	2.193	1.85	3.83	0.83	6
Os03g0810100	13.3	10.2	12.9	0	0	1.73	0.78	2.53	2.07	31.9	19.74	62.09	5.4426	12.135	4.7518	0	18.55	12.43	0	0	0.862	1.479	0	0	17.96	32.06	13.16	10.2	11.5	10	20
Os03g0276300	12.2	19.6	18.3	8.21	11.75	12.11	5.48	6.96	0.69	18.11	9.868	24.46	18.505	15.869	12.671	23.88	19.58	23.83	13.93	6.45	16.38	8.873	7.126	10.83	24.3	12.26	15.35	10.2	20.1	10	13
Os03g0231950	97.9	116	114	67.32	95.82	91.7	205	214	144	220.7	197.4	253.1	125.18	154.95	178.19	179.1	154.6	126.4	81.75	87.88	78.46	133.1	146.8	122.8	216.6	213.1	212.7	156	186	152	5
Os03g0349000	643	673	677	824.3	864.2	839.1	797	830	765	637.2	651.3	666.1	690.12	743.03	785.63	1505	618.3	704.5	821.2	730.5	832	664.7	739	622.6	646.5	668.5	646.9	652	663	635	6
Os03g0209000	79.5	67.1	84.1	62.39	71.41	89.1	111	97.4	85	74.15	75.65	108.2	95.79	65.342	60.981	394.1	98.93	80.81	91.04	85.47	119.8	48.8	67.7	80.17	90.85	77.31	74.56	61.1	71.7	93.3	6
Os03g0561400	1.02	6.52	0	6.568	0.904	9.516	4.7	6.33	2.76	2.587	3.289	2.822	1.0885	0	0.792	11.94	2.061	0	3.716	0.806	0.862	2.218	7.126	2.167	0	0	0	1.85	0.96	4.17	6
Os03g0418700	106	150	123	166.7	229.6	149.7	68.9	116	72.6	89.67	88.81	43.28	131.71	112.95	114.84	238.8	83.47	115	83.61	134.6	82.77	200.4	138.3	116.3	94.02	74.48	35.09	132	191	146	8
Os03g0746500	256	241	202	298.8	329	322.7	330	348	294	178.5	225.9	188.2	262.33	261.37	267.68	525.4	239.1	261.1	352.1	354.8	339.7	374.9	338.5	335.8	251.4	230.1	210.5	346	322	260	6
Os03g0320000	331	254	265	307.9	306.4	340.8	188	254	211	230.2	268.6	214.5	304.79	335.11	284.32	549.3	359.7	315	494.2	341.1	378.5	209.2	190.3	205.8	263	290.4	296	285	259	301	16
Os03g0700400	29.6	6.52	24.8	23.81	18.98	19.9	7.05	16.4	17.3	12.07	7.675	6.586	7.6196	13.068	10.296	47.76	37.1	32.12	9.29	8.063	13.8	11.83	17.1	8.667	5.282	14.14	6.579	19.4	8.61	15	6
Os03g0563300	2664	2888	2989	3417	3704	3297	3670	3442	3881	3592	3846	6059	3007.6	2940.4	2533.5	4705	2965	3679	3681	3518	4245	2663	2306	3208	3506	3879	3287	2095	2695	2404	20

Os03g0686100	247	234	232	215.9	186.2	211.9	225	259	239	270.7	264.2	224.9	256.89	260.43	270.85	561.2	264.8	258	198.8	217.7	229.3	300.9	233.7	228.2	273.6	279.1	307	311	314	287	13
Os03g0293100	2538	2741	3222	2268	2042	1885	5389	5932	5894	3743	5016	3632	4245.2	3125.2	4719.3	5851	3743	3710	2860	2856	3001	3895	3505	4328	3940	3475	2774	3309	3562	4097	5
Os03g0247750	0	0	0	0	0	0	0	0	0	0	0	0	0	0	0	0	0	0	0	0	0	0	0	0.722	0	0	2.193	0	0	0	11
Os03g0378000	464	528	503	486.8	511.6	508.6	691	689	709	435.4	470.4	416.8	503.98	497.53	591.6	967.2	543.1	514.9	495.1	508.8	526.8	634.4	531.6	610.3	527.1	527	447.4	512	501	504	6
Os03g0185400	3.06	0	0	0	2.712	0	3.13	0.63	0	0	0	2.822	1.0885	0	1.5839	0	0	2.072	1.858	0.806	2.587	0	1.425	5.778	0	0	0	0	0	0	9
Os03g0724800	2.04	0	0	0	0.904	1.73	0	0	1.38	0	0	0	0	0	0	0	1.031	1.036	0	0	3.449	0	0	0	0	0	2.193	0	1.91	2.5	16
Os03g0417800	48.9	40.1	56	34.48	32.54	37.2	26.6	17.1	22.1	54.32	40.57	80.91	32.656	60.674	36.43	59.7	44.31	23.83	36.23	24.99	29.32	19.22	22.09	19.5	46.48	37.71	50.44	34.2	47.8	30	20
Os03g0158500	1560	2251	1879	2314	2453	2019	1895	2652	2265	1333	1399	815.7	1716.6	1221	1854.8	2018	1392	1667	1139	1210	1046	2790	2950	2258	1558	1233	1026	1603	1455	2017	17
Os03g0711200	876	978	877	835.8	842.5	845.2	665	786	692	732.9	816.8	577.7	828.36	762.63	856.12	1314	701.8	766.7	594.5	654.7	684.6	781.5	720.5	779.3	819.7	787.3	789.5	804	808	852	13
Os03g0397700	89.7	75.4	71.1	163.4	154.6	100.3	211	243	245	55.18	43.86	35.75	32.656	49.473	60.981	47.76	69.05	68.38	90.11	118.5	76.74	167.8	234.5	134.3	50.71	74.48	87.72	74	77.5	70.8	18
Os03g0287900	336	401	343	304.6	343.5	345.2	727	600	697	538.9	530.7	527.8	346.15	428.45	424.49	609	383.4	344	412.5	382.2	389.7	549.4	554.4	589.4	465.9	504.4	565.8	354	357	329	5
Os03g0231600	11.2	9.31	17.2	0.821	0.904	0.865	12.5	8.86	29.7	5.173	6.578	12.23	15.239	20.536	24.551	23.88	24.73	23.83	6.503	1.613	2.587	2.958	9.977	12.28	14.79	9.428	19.74	34.2	21	24.2	13
Os03g0431550	2.04	0	0	0	0	0	0	0	0	0	1.096	0	0	0	0	0	0	0	0	0	0	0.739	0	0	0	0	0	0	0	0	19
Os03g0140700	1045	963	1091	692.1	810	738.8	1668	2006	2141	610.4	734.6	470.4	956.81	856.91	841.07	1994	863.6	675.5	1191	941.7	1242	1250	1296	1626	626.4	495.9	605.2	960	1164	1072	18
Os03g0356300	1726	2107	2087	4497	5295	4125	5412	5574	5481	2003	2469	2984	2291.3	1767	1453.3	1791	1154	1586	4250	4479	4168	3222	3094	4263	1483	1143	940.8	847	988	1020	12
Os03g0100020	0	4.66	1.08	0	0	1.73	0	0	0	0	2.193	1.882	0	0	0	0	1.031	0	0	0	0.862	0	0.713	0	1.056	0.943	0	2.78	2.87	0	1
Os03g0329900	1263	1220	1126	1098	1071	1063	1088	1074	1120	1137	1249	1164	1188.7	1252.7	1338.4	2078	1225	1223	1161	1178	1098	1072	1182	1132	1250	1262	1342	1300	1184	1235	13
Os03g0364500	68.3	125	123	178.2	178.1	123.7	153	162	135	103.5	116.2	57.39	62.046	86.811	110.88	191.1	63.89	87.03	100.3	148.4	103.5	87.25	111.9	98.23	85.57	64.11	61.4	51.8	66	78.3	12
Os03g0168900	185	191	176	247.9	266.7	224.9	501	376	387	279.3	197.4	305.8	234.03	238.96	199.58	238.8	166.9	176.1	328.8	367.7	325.1	269.9	263	322.8	203.9	225.3	236.8	190	163	152	15
Os03g0192600	36.7	35.4	37.7	13.96	12.66	16.44	14.1	14.5	13.1	30.18	19.74	49.86	15.239	9.3345	12.671	11.94	39.16	25.9	26.94	8.869	24.14	21.44	22.8	37.56	15.85	33	46.05	27.8	33.5	31.7	20
Os03g0729200	2942	2480	2931	2376	2221	2587	2956	3083	2716	3658	3665	3852	3822.9	4385.4	3885.4	7403	4070	3547	3488	3582	3844	2023	2375	2505	3831	4140	3785	3722	3803	3527	13
Os03g0217400	221	187	179	180.6	179	193.8	206	176	217	238.8	229.1	159	176.34	190.42	180.57	179.1	232.9	201	197.9	219.3	239.7	257.3	241.6	201.5	305.3	252.7	291.7	266	300	258	1
Os03g0781000	638	627	662	1415	1685	1531	1572	1724	1893	442.3	572.3	843.9	547.53	450.86	327.08	835.9	420.5	543.9	1365	1611	1706	1281	1030	1398	425.7	337.5	375	304	340	311	12
Os03g0119700	21.4	28.9	28	41.87	37.06	39.79	35.2	24.7	22.8	15.52	27.41	17.88	19.593	20.536	14.255	47.76	22.67	33.15	26.94	45.15	43.97	22.92	37.06	39.72	24.3	16.03	17.54	45.3	25.8	26.7	10
Os03g0693000	777	648	680	447.4	367.9	378	418	557	578	623.4	509.8	623.8	531.2	529.27	617.73	1302	544.1	595.7	426.4	388.6	394	490.2	535.2	407.3	572.5	512.9	508.8	519	458	416	13
Os03g0824600	0	0	0	0	0	0	0	0	0	0	0	0	0	0	0	0	0	0	0	0	0	2.218	0	0	0	0	0	0	0	0	9
Os03g0355300	159	230	166	199.5	236.8	205	178	160	187	186.2	167.7	164.6	200.29	170.82	191.66	298.5	183.4	220.7	227.6	226.6	178.5	187.1	181	183.5	183.8	176.3	162.3	167	234	261	6
Os03g0297300	467	438	379	501.6	665.3	632.4	901	719	1124	472.5	447.3	637.9	583.45	506.86	366.68	573.2	324.6	358.5	812.8	685.3	658.7	519	690.5	606.7	368.7	449.7	644.7	435	316	279	15
Os03g0681800	274	247	263	246.3	278.4	244.8	231	254	224	284.5	260.9	262.5	264.51	248.3	269.27	501.5	328.7	264.2	256.4	270.1	243.1	298	233.7	282.4	305.3	296.1	241.2	265	274	310	6
Os03g0700500	0	0	0	1.642	0	0	0	0	0	0	0	0	0	0	0.792	0	0	0	0	0	0	0	0	0	0	0	0	0	0	0	2
Os03g0424200	753	851	856	811.1	810.9	771.6	539	543	512	635.4	706.1	530.6	861.02	588.07	718.31	1493	717.2	845.4	664.2	740.2	698.4	704.6	670.6	509.9	688.7	645.8	609.6	859	921	936	13
Os03g0157400	3311	3294	3677	3289	4046	3430	2170	2100	2069	2771	2578	1722	2850.8	2925.4	3019.8	8609	3902	4263	2666	3604	2869	2355	2746	1928	3035	2954	2651	4868	4301	4534	13
Os03g0684100	250	253	224	235.6	222.4	246.5	243	206	189	250.9	247.8	241.8	277.57	281.9	245.51	286.6	249.4	284.9	205.3	244.3	224.2	195.2	218.1	211.6	263	254.6	269.7	266	214	239	13
Os03g0719100	203	198	212	254.5	253.1	253.5	211	195	209	229.3	268.6	334	226.41	212.83	200.37	370.2	260.7	231	299.1	323.3	303.5	186.3	199.5	216.7	241.9	269.7	285.1	187	254	255	16
Os03g0110400	550	591	579	636.3	678	611.6	608	589	588	601.8	604.1	774.3	534.46	615.14	597.14	812	574	540.8	649.3	626.5	610.4	476.2	510.2	505.6	623.3	619.4	554.8	535	595	555	6
Os03g0840900	299	234	251	236.4	224.2	246.5	334	262	261	354.4	331.1	433.7	348.33	304.31	280.36	226.9	313.3	251.8	390.2	311.2	371.6	225.5	231.6	257.1	281	342.3	315.8	276	293	248	20
Os03g0584400	10.2	8.38	4.31	10.67	16.27	5.19	79.1	83.5	40.1	41.38	24.12	22.58	9.7967	19.602	8.7116	0	10.31	11.4	19.51	19.35	4.311	25.88	45.61	11.56	17.96	16.03	26.32	6.48	7.65	11.7	18
Os03g0135600	914	1013	985	995.8	1120	1095	723	817	754	945	971.4	891.9	1394.4	1186.4	1140.4	1684	1018	1038	1053	1191	1279	678	678.4	813.2	1079	948.5	837.7	918	1060	1038	13
Os03g0747300	772	830	841	669.1	729.5	672.1	568	655	735	776	855.2	594.6	927.42	757.96	975.7	1445	626.6	620.6	652.1	662	539.7	779.3	771.1	715	813.4	700.5	745.6	788	787	791	13
Os03g0716900	25.5	15.8	23.7	29.56	33.45	30.28	20.4	22.8	24.2	14.66	18.64	21.64	21.77	13.068	15.047	35.82	32.98	12.43	50.16	42.73	24.14	8.133	17.1	7.945	16.9	8.486	10.96	13	11.5	15	10
Os03g0645200	28.5	12.1	16.2	18.88	9.944	12.11	32.1	13.3	32.5	45.7	18.64	22.58	22.859	7.4676	19.007	23.88	20.61	17.61	12.08	23.38	24.14	12.57	16.39	20.22	22.18	30.17	28.51	23.1	17.2	12.5	7
Os03g0356596	2.04	1.86	0	1.642	2.712	1.73	0.78	1.27	0	1.724	0	0	1.0885	2.8004	0	11.94	3.092	2.072	2.787	0	4.311	1.479	1.425	3.611	0	4.714	0	0.93	0	0.83	6
Os03g0229801	17.3	16.8	19.4	22.17	28.02	22.49	65	51.2	33.9	9.484	8.771	22.58	8.7082	8.4011	7.9197	35.82	11.34	14.5	19.51	28.22	16.38	14.05	22.09	47.67	14.79	11.31	6.579	1.85	7.65	7.5	18
Os03g0232500	761	859	886	662.5	773.8	664.4	1255	1166	1149	826.8	960.4	862.7	927.42	882.11	738.11	1361	809	826.8	808.2	1032	929.5	689.1	573	770.6	622.2	630.8	548.2	610	706	599	6
Os03g0144100	22.4	15.8	25.9	31.2	30.73	36.33	41.5	27.2	22.1	12.93	19.74	6.586	7.6196	16.802	14.255	47.76	32.98	18.65	21.37	18.54	16.38	42.15	29.93	30.33	19.01	20.74	15.35	20.4	24.9	22.5	6
Os03g0306100	0	0	0	0	0	0	0	0	0	0	0	0	0	0	0	0	0	0	0	0	0	1.479	0	0	0	0	0	0	0	0	9
Os03g0321400	626	613	641	577.1	567.7	640.1	902	805	774	756.1	660	844.9	694.48	742.09	696.93	632.9	674	600.9	732	697.4	689.8	675.8	656.3	751.1	659.2	719.4	743.4	626	615	597	15
Os03g0838100	750	797	766	571.4	474.6	482.7	773	678	762	810.5	753.2	594.6	484.39	575.94	646.24	991.1	708	674.5	438.5	507.2	512.2	1037	1018	947.6	840.9	899.5	776.3	811	719	766	9
Os03g0349200	51	53.1	59.3	237.3	257.6	194.6	290	293	273	56.04	62.49	51.75	66.4	78.41	57.814	59.7	39.16	68.38	237.8	267.7	222.5	191.5	223.1	268	61.27	49.97	39.47	61.1	67.9	67.5	12
Os03g0581200	154	165	194	192.9	216	221.5	232	206	206	155.2	190.8	118.5	166.54	169.89	161.56	214.9	129.8	151.3	205.3	210.4	173.3	165.6	176.7	173.3	132	120.7	131.6	154	182	162	12
Os03g0784300	209	184	191	143.7	162.7	163.5	188	106	171	204.3	224.8	194.7	166.54	226.83	214.62	346.3	217.4	232.1	133.8	133.8	169.9	176	146.8	144.4	244	234.8	197.4	271	259	241	13
Os03g0237250	0	0	0	0	0	0	0	0	0	0	0	0	0	0	0	0	0	0	0	0	0	0	0	0	0	0	0	0	0.96	0	1
Os03g0126300	582	585	573	823.4	1026	734.4	760	946	778	488	667.7	665.2	599.77	522.73	496.56	931.4	445.2	524.2	1026	1101	945	613	569.4	686.9	476.4	382.8	414.5	431	449	416	10
Os03g0764550	4.08	5.59	0	0	2.712	2.595	0.78	1.27	2.76	0.862	4.386	6.586	6.5311	4.6673	4.7518	0	5.153	1.036	3.716	1.613	2.587	2.958	0.713	2.889	1.056	1.886	2.193	6.48	2.87	0.83	4
Os03g0260100	10.2	3.73	6.47	7.389	5.424	8.65	9.39	9.49	4.84	12.93	12.06	16.93	18.505	17.736	6.3357	0	6.183	9.324	14.86	12.09	13.8	12.57	8.552	6.5	16.9	7.543	26.32	6.48	8.61	9.16	11
Os03g0322700	35.7	34.5	36.6	71.42	91.3	48.44	29	29.7	21.4	29.31	44.95	20.7	28.302	24.27	29.303	35.82	22.67	36.26	43.66	41.93	39.66	16.27	8.552	25.28	36.97	40.54	43.86	30.5	21	40	2
Os03g0426350	320	391	304	571.4	565.9	615.9	532	421	511	574.2	514.2	600.2	592.15	454.59	510.82	704.5	315.3	366.8	681.9	646.6	645.8	482.8	388.4	518.6	461.6	438.4	423.2	377	355	403	10
Os03g0422200	6.12	9.31	4.31	4.105	7.232	4.325	9.39	8.22	6.22	3.449	1.096	0.941	5.4426	2.8004	3.9598	0	6.183	4.144	1.858	4.838	1.724	2.218	5.701	5.778	2.113	3.771	4.386	1.85	0	0.83	18
Os03g0322000	0	0	0	4.105	0.904	0.865	0	1.27	3.46	0	0	2.822	0	0	0	0	0	1.036	2.787	0.806	9.484	0	2.138	0	2.113	0	2.193	3.7	0	0	16
Os03g0399600	517	507	605	322.6	340.8	308.8	211	249	205	247.4	325.6	307.6	415.81	427.52	466.47	1075	525.6	624.7	194.2	300.7	228.5	329.8	284.3	210.2	483.8	462.9	390.3	617	630	671	13
Os03g0225500	250	299	268	515.6	525.2	546.7	204	211	193	189.7	209.4	132.7	240.56	222.16	229.67	728.4	230.8	307.7	326.1	415.2	402.7	324.6	319.3	237.6	189.1	199.9	162.3	287	264	304	6
Os03g0165400	1.02	0.93	0	0.821	0	3.46	3.13	0.63	0	0	0	0	2.177	2.8004	0	0	1.031	1.036	2.787	0.806	0.862	0	3.563	10.11	0	1.886	8.772	3.7	1.91	0.83	11
Os03g0186500	957	921	1019	685.5	717.8	667.8	1139	977	979	1038	1101	965.3	1091.8	1037.1	1113.5	1672	951.2	911.7	695.8	648.3	704.4	930.9	927.1	926.6	1186	1184	989	1062	1149	1110	13
Os03g0727600	13.3	9.31	1.08	0	0	0.865	9.39	0	3.46	1.724	0	0	0	0	1.5839	0	9.275	3.108	0	0	0	11.09	2.851	31.06	1.056	0.943	0	1.85	0	0	9
Os03g0799600	629	546	620	500.8	509.8	599.5	260	245	226	560.4	596.4	606.8	683.59	660.88	662.88	680.6	549.3	673.4	693	649.9	783.8	292.1	305	314.2	583.1	625.1	638.1	614	601	646	16
Os03g0259100	0	6.52	5.39	1.642	1.808	9.516	2.35	1.27	0	3.449	1.096	2.822	5.4426	1.8669	0	0	1.031	1.036	0.929	0	2.587	0	0.713	0	1.056	0	0	1.85	2.87	2.5	2
Os03g0391000	344	392	377	419.5	381.5	390.1	351	385	339	431.1	439.7	447.8	362.48	404.18	354.01	823.9	368.9	361.6	455.2	474.1	475.1	364.5	343.5	377	375	476.1	399.1	441	408	442	6
Os03g0383100	47.9	41	58.2	46.8	57.85	39.79	84.6	87.9	89.1	30.18	41.66	44.22	41.364	39.205	40.39	59.7	70.08	45.59	56.67	66.92	50.01	48.06	47.75	54.17	55.99	44.31	19.74	51.8	35.4	39.2	18
Os03g0411900	295	346	356	851.4	875	725.8	581	697	670	530.2	475.8	420.5	538.82	469.53	499.73	943.3	255.6	406.1	608.5	678.1	522.5	740.9	620.7	502.7	402.5	365.8	318	453	468	496	12
Os03g0401951	1.02	1.86	1.08	0	2.712	0.865	0	0	0	1.724	0	2.822	1.0885	0	0	0	3.092	0	0	0	2.587	0.739	0.713	1.444	0	1.886	0	0.93	0	0	20
Os03g0810500	11.2	8.38	7.54	12.31	17.18	18.17	4.7	7.59	2.76	4.311	13.16	5.645	25.036	12.135	15.047	23.88	22.67	14.5	21.37	12.9	21.56	14.05	11.4	17.33	26.41	17.91	24.12	22.2	20.1	26.7	1
Os03g0407000	37.7	53.1	45.3	38.59	62.37	44.98	132	109	112	119	92.1	71.5	79.462	66.275	72.861	0	42.25	42.48	65.96	75.79	44.84	62.85	83.38	73.67	63.38	59.4	67.98	46.3	60.2	50	5
Os03g0253800	224	209	178	348.1	382.4	393.6	354	302	267	206.1	209.4	199.5	188.31	223.09	163.14	370.2	191.7	214.5	420.8	355.6	364.7	250.7	243	242.7	176.4	232.9	166.7	177	175	195	10
Os03g0833700	537	449	419	709.3	683.4	674.7	808	727	878	576.8	518.6	630.3	643.32	644.08	578.93	561.2	542.1	535.6	710.6	616	753.6	823.7	696.2	700.6	513.4	650.6	664.5	510	483	459	12
Os03g0717600	1059	977	917	1246	1352	1271	1287	1384	1377	1050	1004	1122	1415.1	1259.2	1283	1337	976.9	1040	1250	1093	1318	1158	1135	1101	1138	1156	1116	1063	929	987	12
Os03g0227000	1402	1499	1484	1434	1445	1582	1285	1083	1164	1163	1124	1104	1171.2	1260.2	1186.4	2842	1416	1328	1177	1125	1198	1413	1477	1481	1243	1130	1193	1485	1345	1296	6
Os03g0187200	114	92.2	86.2	78.81	76.84	87.37	300	233	217	228.5	210.5	297.3	174.16	155.89	144.14	83.59	107.2	116	175.6	162.1	178.5	122	120.4	164.7	169	203.7	274.1	135	147	117	15
Os03g0140200	52	73.6	30.2	504.1	660.8	603.8	359	249	408	50.01	62.49	34.81	18.505	25.203	11.088	47.76	38.13	40.41	364.1	312.8	445.8	841.4	706.9	609.6	52.82	65.06	190.8	44.4	34.4	25	12
Os03g0265900	38.7	52.2	43.1	5413	5992	5902	218	182	268	31.04	33.99	27.28	46.806	40.138	38.014	35.82	61.83	40.41	5005	5134	5439	312	290.8	291.1	43.31	19.8	6.579	34.2	49.7	34.2	10
Os03g0396100	5.1	9.31	6.47	15.6	22.6	16.44	11.7	8.22	11.7	9.484	4.386	4.704	13.062	4.6673	7.1277	23.88	11.34	8.288	17.65	24.99	31.04	7.394	9.264	7.222	7.395	10.37	8.772	6.48	12.4	8.33	10
Os03g0227800	####	####	####	32662	32660	32298	####	####	####	43681	48096	41343	44744	44915	49879	89473	56454	57353	35245	41890	33606	41752	34903	31227	51595	51394	51498	####	####	####	13
Os03g0581650	372	303	365	209.3	229.6	211.9	110	133	108	331.1	309.2	334.9	487.66	411.65	472.01	561.2	304	249.7	161.6	219.3	181.9	161.2	134.7	127.8	380.3	302.7	320.2	356	290	364	13
Os03g0107600	149	184	133	126.4	129.3	117.6	79.1	105	96.7	318.1	144.7	91.26	103.41	91.478	129.09	107.5	146.3	139.9	76.17	90.3	47.42	153.8	148.9	111.9	118.3	119.7	103.1	166	146	158	7
Os03g0162200	1.02	2.79	1.08	0.821	0	0	0	0.63	0	0	0	0	0	0	0	0	0	2.072	0	0	3.449	1.479	0	3.611	4.225	1.886	2.193	0	3.83	0	1
Os03g0244000	2216	1827	2131	678.1	696.1	737	1420	1323	1522	2196	2540	4199	2634.2	2301	2003.7	3081	3010	2455	1415	1078	1103	1073	917.2	1523	2840	3497	3511	1720	1841	1910	3
Os03g0374500	0	0	0	0	0	0	0	0.63	0	0	0	0	0	0	0	0	0	0	0	0.713	0	0	0	0	0	0.943	0	0	0	0	17
Os03g0663400	7.14	27.9	29.1	37.76	41.58	28.55	198	123	233	92.25	39.47	26.34	17.416	28.004	31.679	11.94	18.55	14.5	27.87	25.8	34.49	77.64	99.77	66.45	5.282	11.31	8.772	0	15.3	7.5	18
Os03g0388800	0	1.86	1.08	0	0	0	0	0	0.69	0	1.096	0.941	1.0885	0	0.792	0	0	0	0	0	0	0	0	0	0	0	2.193	0	0	0	11
Os03g0604500	0	0	0	0	0	0	0	0.63	0	0	0	0.941	0	0	0	0	0	0	0	0	0	0	0	0	0	0	0	0	0	0	20
Os03g0289200	383	285	273	293.1	224.2	275.1	347	306	287	378.5	387	371.6	341.8	315.51	311.24	597	384.4	335.7	316.8	324.1	331.1	264.7	310.7	283.8	295.8	338.5	357.4	288	303	275	6
Os03g0774200	1440	1334	1348	1285	1463	1367	1643	1486	1560	1430	1356	1693	1504.3	1637.3	1321	1708	1450	1382	1550	1407	1610	1292	1222	1463	1368	1520	1629	1277	1222	1246	15
Os03g0768900	0	0	0	0	0	0	0	0	0	0.862	0	0	0	0	0	0	0	0	0	0.806	0	0	0	0	0	0.943	0	0	0	0	7
Os03g0809200	2.04	4.66	1.08	3.284	3.616	7.785	11.7	12.7	2.76	4.311	5.482	10.35	5.4426	12.135	8.7116	11.94	4.122	5.18	3.716	2.419	6.898	8.873	4.276	13.72	8.451	1.886	0	7.4	9.56	5.83	5
Os03g0639600	572	570	539	528.7	515.3	549.3	737	665	720	544.9	526.3	553.2	459.36	507.8	527.45	1087	591.5	514.9	564.8	551.5	561.3	666.2	756.1	794.5	503.9	491.2	611.8	451	456	509	6
Os03g0835800	659	719	749	642.8	653.6	632.4	499	533	538	524.2	609.6	464.8	549.7	521.8	681.88	1099	644.1	627.8	489.6	599.1	480.3	742.3	687	580.7	658.1	556.3	568	797	716	716	13
Os03g0114800	87.7	79.2	89.4	219.2	245	329.6	226	257	249	76.73	58.11	42.34	68.577	80.277	76.821	83.59	37.1	61.13	184.9	165.3	173.3	244	314.3	200.8	58.1	55.63	116.2	49	67.9	63.3	12
Os03g0416200	5.1	2.79	1.08	0.821	2.712	2.595	1.57	0.63	0	0.862	6.578	6.586	1.0885	2.8004	1.5839	23.88	4.122	0	2.787	3.225	0	0	0.713	0.722	0	7.543	15.35	0.93	3.83	3.33	6
Os03g0619600	52	36.3	35.6	27.91	17.18	38.06	12.5	2.53	15.9	30.18	17.54	23.52	22.859	32.671	28.511	322.4	222.6	167.8	76.17	109.7	131.9	139	165.3	137.9	172.2	165	149.1	315	179	218	13
Os03g0851400	64.2	63.3	50.6	73.07	98.53	65.74	32.9	28.5	31.1	24.14	28.51	15.99	31.567	28.004	33.263	23.88	36.07	43.51	37.16	54.02	15.52	48.06	47.75	49.11	29.58	25.46	17.54	28.7	38.3	53.3	2
Os03g0852101	4.08	1.86	0	0	0	0	0.78	0	0	0	0	0.941	0	0.9335	0	0	0	2.072	0.929	1.613	6.036	0	1.425	2.167	4.225	0	0	0	0	2.5	19
Os03g0675700	376	348	375	481.1	514.4	455.9	405	355	361	396.6	402.4	290.7	396.22	366.85	404.69	847.8	319.5	288	539.7	537.8	548.4	245.5	265.1	275.9	410.9	313	375	318	321	317	6
Os03g0720400	22.4	29.8	19.4	41.05	39.77	51.9	36	41.7	37.3	31.9	28.51	22.58	35.921	36.405	30.887	11.94	24.73	29.01	42.73	38.7	41.39	43.62	52.73	64.28	24.3	21.69	8.772	26.8	39.2	23.3	12
Os03g0287100	676	529	647	201.1	186.2	191.2	247	327	186	645.8	592.1	827	672.71	734.63	818.1	1230	893.5	747	262	302.4	260.4	176.7	281.5	230.4	959.2	867.4	815.8	834	670	834	13
Os03g0857500	58.1	56.8	37.7	29.56	33.45	47.58	11.7	8.22	19.3	40.52	54.82	128.9	34.833	37.338	26.927	23.88	86.56	49.73	64.1	62.08	69.84	16.27	11.4	23.11	68.66	116	78.95	16.7	39.2	25.8	20
Os03g0100050	297	281	262	288.2	297.4	294.1	288	312	346	325.9	354.1	356.6	313.49	322.04	297.78	274.6	249.4	287	274	294.3	281.1	298	295	295.4	288.4	285.7	307	305	220	303	7
Os03g0427100	78.5	75.4	74.4	99.34	145.5	98.62	184	149	163	125	115.1	116.7	81.639	98.946	135.43	155.2	103.1	94.28	82.68	110.5	98.29	197.4	176.7	197.9	138.4	100.9	103.1	124	109	131	18
Os03g0284600	2739	2549	2971	792.2	772.9	975.8	1869	2188	1760	4099	4394	4552	4674.1	4397.5	4531.6	5875	4119	3448	1587	1496	1631	1131	1316	1398	4533	4648	4105	3707	3642	3925	3
Os03g0751800	478	482	517	500.8	476.4	516.4	322	333	325	428.5	394.7	470.4	463.71	501.26	491.81	776.2	510.1	558.4	445.9	491	471.6	588.6	467.5	378.5	560.9	613.8	535.1	663	562	660	13
Os03g0739500	0	0	0	0	0	1.73	3.13	0	0	0	0	0	5.4426	0	0	0	0	0	0	0	0	3.697	0	0	0	0	0	0	0	0	4
Os03g0274300	1967	2341	2120	598.5	564.1	664.4	2596	3063	2781	1595	1784	1675	2100.8	1920.1	2074.2	3929	2025	2256	693	721.6	643.2	2879	2888	3187	2063	2050	2057	2190	2354	2667	5
Os03g0114100	14.3	19.6	17.2	2.463	2.712	2.595	19.6	6.96	41.5	5.173	3.289	7.527	7.6196	6.5342	4.7518	0	12.37	17.61	0.929	9.675	12.07	116.1	34.92	111.2	7.395	12.26	6.579	8.33	9.56	8.33	9
Os03g0817800	8.16	3.73	3.23	13.14	12.66	7.785	2.35	2.53	1.38	33.63	10.96	23.52	6.5311	6.5342	7.1277	0	7.214	4.144	1.858	7.257	2.587	4.436	0.713	0.722	9.507	6.6	6.579	7.4	6.69	2.5	7
Os03g0187800	0	0	1.08	0	0	0	0	0	0	0	1.096	0	0	0	0	0	0	1.036	0	0.806	0	0	1.425	5.056	0	0	0	0	0	0	9
Os03g0638400	4.08	1.86	0	2.463	0	2.595	1.57	3.16	0.69	1.724	1.096	0	0	1.8669	1.5839	0	0	0	0	0	0	2.218	9.977	1.444	0	0.943	0	1.85	0.96	0	17
Os03g0123300	315	364	329	330.9	340.8	360.7	398	479	418	285.4	292.7	236.1	244.92	265.1	270.85	513.5	301.9	304.6	328.8	364.4	293.2	422.2	391.9	388.6	282	238.5	256.6	266	301	311	6
Os03g0125800	850	943	885	680.6	627.4	595.2	613	607	546	621.6	690.7	589	709.71	785.03	857.7	1290	807.9	729.4	568.5	528.1	491.5	774.9	738.3	658	892.6	787.3	824.5	1096	965	995	13
Os03g0337900	637	584	558	376	352.5	423.9	390	397	540	657.8	548.2	633.2	505.07	482.59	534.58	1027	522.5	560.5	395.7	333	368.2	486.5	526.6	458.6	497.5	495.9	539.5	537	568	472	13

Os03g0199000	121	115	90.5	110.8	132	100.3	162	164	155	136.2	112.9	180.6	146.95	131.62	116.42	107.5	112.3	105.7	231.3	242.7	185.4	96.86	111.9	127.1	128.9	114.1	179.8	88.8	120	107	15
Os03g0610400	23.4	46.6	36.6	1.642	1.808	5.19	16.4	23.4	18.7	12.93	10.96	22.58	17.416	11.201	3.9598	23.88	40.19	17.61	14.86	6.45	1.724	25.14	28.51	32.5	11.62	3.771	15.35	7.4	16.3	17.5	14
Os03g0811300	0	0.93	0	0.821	2.712	0	1.57	0	0	0	0	0	2.177	0	0	0	0	0	0	0.806	0	0.739	0.713	2.167	0	0	0	0	0	0	2
Os03g0212400	208	162	212	385.9	354.4	300.2	389	459	351	228.5	198.4	137.4	215.53	153.09	205.12	274.6	183.4	202	390.2	497.5	428.5	315.7	336.4	288.9	108.8	102.8	89.91	191	140	177	12
Os03g0591300	1.02	0	0	0	0	0	11.7	4.43	2.76	0	0	0	0	0	0	0	0	0	0	0	0	10.35	2.138	9.389	0	0	0	0	0	0	9
Os03g0216300	17.3	24.2	24.8	341.5	332.7	290.7	50.9	62	56	8.622	9.868	13.17	5.4426	27.07	15.839	35.82	15.46	18.65	225.7	243.5	225	90.94	101.2	50.56	6.338	14.14	4.386	23.1	14.3	20.8	10
Os03g0114700	33.6	29.8	44.2	52.54	43.39	38.06	76.7	89.8	76	30.18	38.37	33.87	22.859	16.802	30.095	23.88	17.52	25.9	27.87	41.12	41.39	59.15	48.46	66.45	23.24	32.06	24.12	15.7	35.4	19.2	18
Os03g0853500	0	1.86	0	4.105	0.904	3.46	3.13	0	2.76	0	0	0	0	4.6673	1.5839	11.94	3.092	4.144	2.787	0.806	0	1.479	0.713	2.889	0	1.886	0	1.85	0.96	0.83	6
Os03g0244200	61.2	64.3	28	82.92	59.66	92.56	236	99.9	172	78.46	51.53	66.8	47.895	40.138	26.927	11.94	26.79	30.05	47.38	49.99	48.28	130.1	79.1	157.4	24.3	40.54	39.47	25.9	20.1	13.3	18
Os03g0114400	41.8	28.9	48.5	13.96	11.75	16.44	52.5	20.2	28.3	43.97	40.57	64.92	37.01	35.471	34.847	35.82	37.1	39.37	30.66	34.67	18.11	45.1	39.91	75.11	28.52	59.4	61.4	36.1	23	35	20
Os03g0333400	3957	3165	3442	2178	2181	2131	2310	1929	2367	4345	4768	9987	3348.3	3723.5	3070.5	6400	5019	4788	3828	3024	3872	1834	1315	1949	4869	6611	5954	2923	3376	3214	20
Os03g0188100	46.9	18.6	38.8	2.463	3.616	2.595	1.57	2.53	5.53	31.04	38.37	15.05	148.04	120.42	107.71	286.6	79.35	38.33	0	0	0	0.739	4.276	1.444	44.37	22.63	32.89	65.7	72.7	62.5	13
Os03g0356484	474	452	463	356.3	374.2	346.9	420	413	395	461.3	410.1	391.4	375.54	365.91	422.91	704.5	380.3	399.9	369.7	325.7	344.9	404.4	459.6	380.6	415.2	379	427.6	378	348	342	6
Os03g0759125	0	0	0	0.821	0	0.865	0	0	0	0	0	0	0	0	0	0	0	0	0	0	0	0	0	0	0	0	0	0	0	0	2
Os03g0280800	19.4	4.66	18.3	11.49	9.04	8.65	29.7	25.3	15.9	7.76	15.35	9.408	15.239	9.3345	14.255	11.94	18.55	14.5	7.432	13.71	6.898	12.57	9.977	4.333	14.79	12.26	6.579	13.9	3.83	11.7	5
Os03g0830300	1.02	0	1.08	4.105	1.808	0	6.26	10.1	4.84	0.862	0	0	2.177	0	1.5839	0	0	0	0	0	0.862	7.394	0.713	1.444	0	1.886	0	0.93	2.87	4.17	18
Os03g0342900	4706	6210	4610	5635	5451	6309	5771	4568	6060	6786	8009	6277	5983.6	5309.5	6420.5	6162	3987	4763	2866	3186	3603	5525	4539	4730	5626	5015	5114	3486	3192	4388	7
Os03g0815200	1197	1538	1671	1280	1560	1252	655	942	1004	2390	1563	1012	1233.3	1314.3	1816.8	1361	714.2	778.1	756.2	741	553.5	848.1	1218	743.9	1200	856.1	953.9	947	1170	1061	7
Os03g0387500	289	284	286	400.6	369.7	308.8	279	249	206	200	200.6	249.3	254.71	200.69	244.72	382.1	282.4	267.3	315.8	402.3	317.3	299.5	265.8	250.6	300	251.7	245.6	302	320	297	10
Os03g0775300	232	254	258	493.4	504.4	477.5	267	259	271	237.1	245.6	259.7	287.37	302.44	248.68	394.1	226.7	274.5	402.2	378.1	455.3	329.8	277.2	270.8	264.1	218.7	252.2	280	230	243	10
Os03g0168300	0	5.59	0	2.463	7.232	0	0	0	0.69	0.862	0	0	0	0	0	0	0	0	0	3.225	0	0.739	2.138	1.444	1.056	1.886	0	0	0	0	2
Os03g0791200	17.3	16.8	10.8	2.463	3.616	6.055	19.6	19.6	20	10.35	8.771	12.23	11.974	10.268	7.9197	23.88	30.92	13.47	3.716	12.09	4.311	25.88	27.08	22.39	26.41	19.8	13.16	17.6	13.4	16.7	5
Os03g0650850	65.2	40.1	37.7	174.9	149.2	168.7	155	123	93.3	79.32	40.57	60.21	72.931	75.61	66.525	71.65	62.86	50.77	243.4	238.7	313	87.99	72.69	90.28	62.33	76.37	96.49	58.3	56.4	61.7	10
Os03g0684700	359	335	329	236.4	215.1	256.9	324	466	504	448.3	508.7	830.7	213.35	295.9	272.44	214.9	449.3	453.8	275	292.7	362.1	317.2	356.3	528	476.4	730.7	633.8	176	284	242	20
Os03g0656201	36.7	38.2	39.9	68.14	72.32	60.55	121	93	120	27.59	23.02	35.75	40.275	37.338	30.095	35.82	28.85	22.79	87.32	79.82	75.88	93.16	59.86	103.3	21.13	16.97	24.12	16.7	31.6	27.5	12
Os03g0253500	464	367	455	410.5	381.5	388.4	334	336	322	422.5	409	394.2	416.9	436.86	450.63	394.1	329.8	436.2	335.4	329.8	275	423.7	410.5	341.6	547.2	537.4	445.2	453	454	536	3
Os03g0189400	373	355	359	214.3	187.1	179.1	493	343	630	311.2	373.9	272.8	327.64	378.05	431.62	668.7	343.2	313.9	210.9	196.7	123.3	482.1	515.9	481.7	359.2	321.5	300.4	391	354	342	5
Os03g0197300	163	117	172	34.48	56.05	19.9	99.4	48.7	42.8	256.1	167.7	295.4	193.76	187.62	270.85	334.3	135	143	95.68	104	32.76	23.66	32.78	23.83	104.6	58.46	195.2	241	405	186	13
Os03g0731500	1921	2221	2093	1545	1586	1606	1248	1545	1300	1782	1900	1677	2278.3	1950	2139.9	3523	2147	2188	1459	1578	1490	1545	1510	1593	2035	1919	1739	2201	2031	2470	13
Os03g0287750	0	0	0	0	0	0	0.78	0	0	0	0	0	0	0	0.792	0	0	0	0	0	0.862	0	0	0	2.113	0	2.193	0	0	0	11
Os03g0201600	543	442	523	581.3	568.6	567.5	660	631	570	482	446.2	428.1	533.37	578.74	560.71	835.9	432.8	438.2	706.9	685.3	640.6	471.7	525.2	490.4	474.3	446.9	589.9	472	453	458	6
Os03g0734900	616	578	657	408	444.8	448.1	542	649	589	600.1	699.5	589.9	698.83	744.89	818.1	1493	845	758.4	585.2	634.5	607.9	720.9	650.6	723	754.2	765.6	646.9	894	977	1011	13
Os03g0612600	290	328	316	674	654.5	609.9	835	798	767	398.3	354.1	392.3	422.35	349.11	331.83	525.4	262.8	337.7	783.1	807.1	744.1	519	536.6	601.6	297.9	279.1	269.7	281	312	323	12
Os03g0769700	2.04	0	2.16	0.821	0	1.73	37.6	15.2	22.8	1.724	5.482	11.29	0	2.8004	4.7518	0	7.214	3.108	6.503	1.613	4.311	14.79	7.839	39.72	1.056	3.771	2.193	0	1.91	4.17	18
Os03g0290300	9695	8126	8122	612.4	415.8	542.4	142	347	223	6633	6293	9734	6928.4	5744.5	6243.1	12514	9353	7964	1427	861.9	846.7	315	357	392.2	8280	10004	9666	5280	4659	5399	3
Os03g0111500	691	576	603	538.6	607.5	529.4	744	700	776	584.6	639.2	742.3	643.32	719.69	640.7	859.7	708	641.3	739.4	610.4	656.1	787.4	739.7	777.9	708.8	783.5	708.3	649	600	706	5
Os03g0198600	0	0	0	0	0	0	0	0.63	2.76	0	0	0	0	0	0	0	0	0	0	0	0	5.915	5.701	5.056	0	0	0	0	0	0	17
Os03g0240800	199	224	239	293.9	309.2	369.4	400	275	315	225.9	243.4	242.7	257.98	292.17	243.13	489.6	230.8	234.1	317.7	374.1	322.5	286.1	301.4	289.6	227.1	281.9	278.5	214	240	261	6
Os03g0745600	61.2	36.3	46.3	18.06	18.08	18.17	11	17.1	13.1	47.42	42.76	47.04	48.983	41.072	51.478	35.82	42.25	30.05	10.22	7.257	16.38	10.35	19.95	13.72	43.31	34.89	24.12	46.3	42.1	32.5	3
Os03g0210883	4.08	1.86	2.16	1.642	5.424	0.865	3.91	6.33	1.38	4.311	2.193	0.941	3.2656	6.5342	0.792	0	1.031	9.324	3.716	2.419	4.311	4.436	4.276	1.444	3.169	5.657	8.772	0	0.96	8.33	11
Os03g0281600	1955	2185	2134	2852	2974	2686	2216	2275	2318	2036	1950	1515	2201	2003.2	2215.9	2675	1782	1808	1886	2053	2155	2500	2384	2015	1901	1680	1570	1952	1805	2008	2
Os03g0791300	0	0	0	0	0	0	0	0	0	0	0	0	0	0	0	0	0	1.036	0	0	0	0	0	0	0	0	0	0	0	0	1
Os03g0719900	40.8	95.9	51.7	144.5	153.7	136.7	172	119	182	93.12	108.5	63.98	72.931	70.009	75.237	23.88	42.25	64.23	90.11	81.43	73.29	90.21	109	94.61	61.27	75.43	76.75	56.4	41.1	62.5	12
Os03g0682100	357	380	546	95.23	101.2	84.77	74.4	65.1	47	616.5	369.5	481.7	283.02	369.65	292.24	537.3	290.6	317	201.6	212.1	222.5	67.28	99.06	89.56	386.6	282.9	239	381	313	314	3
Os03g0157500	523	577	586	545.1	592.1	607.3	634	701	690	599.2	638.1	557.9	668.35	640.35	666.04	883.6	559.6	598.8	640	629.7	766.5	652.1	575.1	661.6	674	630.8	620.6	672	653	711	6
Os03g0263300	0	0	1.08	0	0	0	0	0	0	0	0	0	0	0	0	0	0	0	0	0	0	0	0	0	0	0	0	0	0	0	14
Os03g0154700	474	300	363	477	460.1	579.6	884	614	762	490.6	516.4	647.3	512.69	614.21	352.42	382.1	329.8	412.3	759	703.9	752.7	463.6	499.6	528	453.2	518.6	600.9	276	357	272	15
Os03g0259900	464	428	398	206.9	190.7	205	134	168	158	278.5	279.6	306.7	291.72	370.58	283.52	561.2	493.6	391.6	182.1	191.1	176.8	278.7	260.8	281	368.7	331.9	403.5	469	374	414	13
Os03g0621400	22.4	17.7	25.9	55.01	40.68	44.12	14.1	28.5	24.9	18.97	15.35	15.99	25.036	10.268	28.511	11.94	25.76	37.3	44.59	39.51	37.94	42.15	29.93	34.67	19.01	27.34	28.51	27.8	25.8	17.5	10
Os03g0633900	534	582	603	2380	2445	2574	922	948	910	753.6	757.6	796.9	764.14	812.1	581.3	740.3	473	508.7	2745	2569	3128	638.8	642.1	663	572.5	588.3	537.3	396	556	578	10
Os03g0792900	1236	1065	1226	1085	1172	1035	1081	1056	930	1229	1061	1027	1126.6	1144.4	1167.4	1361	997.6	893.1	1130	1052	944.1	795.6	900.1	956.3	1050	1041	1153	1051	1067	998	13
Os03g0678800	42.8	82.9	17.2	3.284	0	5.19	0	1.27	0	0.862	0	0	0	0	0	23.88	47.4	36.26	0	0.806	6.898	0.739	9.264	52.72	0	1.886	4.386	0	0	0	14
Os03g0791800	1274	1268	1127	692.9	700.6	633.2	2050	1774	1909	1990	1657	2410	1119	1198.6	1257.6	859.7	1186	1047	928	849.8	934.6	1290	1222	1534	1429	1379	1585	1007	903	845	5
Os03g0330000	99.9	91.3	121	101	98.53	102.9	74.4	48.1	52.5	62.08	64.69	67.74	52.249	59.741	64.941	131.4	99.96	104.6	65.96	64.5	67.25	70.24	54.16	75.11	68.66	75.43	125	70.3	89.9	80	13
Os03g0586900	704	800	710	931.8	1029	891	1051	1068	1170	625.1	749.9	773.4	787	725.29	679.51	1302	703.9	710.7	955	1022	989.8	912.4	909.3	988.8	685.6	608.1	616.2	702	732	709	12
Os03g0262900	888	815	957	495	485.4	468	405	493	427	654.4	603	425.3	626.99	605.81	682.67	1182	666.8	771.8	406.9	437.8	429.4	459.9	505.3	450.7	540.9	544	539.5	585	603	547	13
Os03g0778000	569	422	605	449.1	392.3	458.5	411	414	346	316.4	258.7	248.4	403.84	364.98	342.13	704.5	515.3	526.3	535.1	513.6	543.2	386.7	424.7	369.8	307.4	293.2	296	446	401	407	6
Os03g0756500	12.2	14	5.39	19.7	21.7	21.63	5.48	3.8	2.07	5.173	5.482	3.763	8.7082	7.4676	5.5438	11.94	2.061	4.144	15.79	19.35	25.87	9.612	14.25	14.44	7.395	5.657	4.386	10.2	6.69	6.66	10
Os03g0233500	491	594	539	517.2	491.8	436.8	497	438	396	590.6	570.1	602.1	539.91	545.14	591.6	1134	534.8	538.7	455.2	489.4	501.8	484.3	510.2	512.8	633.8	656.2	629.4	698	717	652	13
Os03g0164200	11.2	31.7	25.9	22.17	12.66	18.17	9.39	21.5	40.1	29.31	27.41	45.16	18.505	21.469	34.055	11.94	3.092	1.036	3.716	15.32	0	5.915	12.11	12.28	29.58	3.771	13.16	17.6	14.3	16.7	7
Os03g0790600	1.02	0	1.08	0	0	0	0	0	0	0	0	0	0	0	1.5839	0	3.092	0	0	0	0	0	0	0	0	0	2.193	3.7	0	0.83	1
Os03g0165100	141	179	153	183.1	169	171.3	85.3	106	83.6	142.3	149.1	141.1	140.42	146.55	135.43	131.4	113.4	157.5	121.7	125.8	113.8	161.9	136.8	116.3	144.7	163.1	116.2	204	201	202	8
Os03g0323200	####	####	####	7551	6681	6032	1228	2196	1632	30939	30140	40831	23648	22999	28417	38999	22927	29212	9172	8308	9690	1250	1375	1151	30621	31498	23416	####	####	####	3
Os03g0231000	2.04	6.52	6.47	1.642	4.52	0	8.61	10.1	8.98	7.76	5.482	3.763	4.3541	5.6007	1.5839	0	5.153	3.108	1.858	0.806	1.724	1.479	12.11	2.167	6.338	2.829	2.193	4.63	3.83	7.5	5
Os03g0851000	438	307	325	471.2	540.6	552.8	400	357	430	344.9	293.8	527.8	372.27	375.25	264.52	394.1	279.3	317	587.1	539.4	515.6	314.2	313.6	296.8	288.4	423.3	418.8	244	330	230	10
Os03g0400901	2.04	0	1.08	0	0	1.73	0	0.63	2.07	0	0	0	1.0885	0.9335	0	0	3.092	1.036	0	0	0	2.958	0	0	0	0.943	0	0	2.87	0	19
Os03g0427000	470	480	401	482.7	508	455	625	612	630	431.1	500	452.5	610.66	593.68	590.81	620.9	624.5	509.7	465.4	435.4	482.8	944.2	704.1	701.3	514.4	540.2	497.8	532	489	678	9
Os03g0780900	89.7	99.7	97	229.9	212.4	196.4	115	103	77.4	80.18	83.33	95.02	108.85	112.95	118.79	286.6	124.7	131.6	210.9	200	191.4	162.7	133.3	115.6	102.5	113.1	111.8	97.1	131	127	10
Os03g0655100	44.9	29.8	28	2.463	0	1.73	0	0	0.69	15.52	15.35	33.87	37.01	61.608	23.759	59.7	66.98	44.55	1.858	2.419	0	3.697	0.713	1.444	26.41	42.43	43.86	39.8	64.1	39.2	3
Os03g0772300	2136	2274	2386	2198	2213	2360	2384	2555	2421	2143	2627	2600	2473.1	2588.5	2477.3	3379	2586	2854	2195	2662	2687	3085	2298	2466	2790	2840	2695	2658	2722	2881	13
Os03g0415200	13.3	8.38	18.3	3.284	9.04	9.516	17.2	12	8.98	31.04	8.771	7.527	10.885	10.268	12.671	11.94	4.122	11.4	10.22	9.675	6.898	18.48	42.76	28.89	8.451	8.486	28.51	10.2	13.4	8.33	17
Os03g0632800	220	219	199	134.6	116.6	120.2	192	185	190	213.8	208.3	158.1	229.68	223.09	252.64	274.6	232.9	236.2	157	150.8	139.7	156	161.8	153.8	208.1	186.7	225.9	216	207	179	13
Os03g0113200	2.04	0	0	0	0	0	1.57	0	2.07	0	0	0	0	0	0	0	0	0	0	0	0	0	0	0	0	0	0	0	0	0	19
Os03g0135700	0	0	0	0	0	0.865	0	0	0	0	0	0	0	0	0	0	0	0	0	0	0	0	0	0	0	0	0	0	0	0	2
Os03g0152900	50	60.5	50.6	101	79.55	97.75	37.6	46.8	40.8	57.77	43.86	51.75	56.603	60.674	76.029	119.4	59.77	67.34	62.24	70.15	54.32	74.68	81.24	52	86.62	43.37	41.67	70.3	85.1	71.6	8
Os03g0228500	260	211	297	116.6	119.3	111.6	143	193	132	208.6	228.1	232.4	210.08	238.03	211.45	406	221.6	279.7	120.8	120.1	105.2	110.2	112.6	101.8	194.4	236.7	182	216	232	199	13
Os03g0438000	55.1	53.1	61.4	29.56	42.49	42.39	67.3	50	54.6	56.9	48.24	78.09	81.639	57.874	58.605	23.88	61.83	62.16	46.45	29.03	36.21	41.41	32.07	41.17	48.59	54.68	57.02	56.4	39.2	49.2	20
Os03g0620500	0	0.93	2.16	0	1.808	0	0	0	0	1.724	0	0	0	0	0.792	0	0	0	1.858	0.806	0.862	2.218	5.701	2.167	2.113	3.771	0	0.93	2.87	1.67	17
Os03g0815800	1251	1127	1218	1366	1299	1278	1935	1713	1521	1790	1721	1859	1468.4	1459.9	1478.6	1815	1374	1313	1933	1916	1819	1287	1470	1593	1571	1713	2024	1436	1455	1345	15
Os03g0711250	135	194	177	168.3	146.4	147.1	72.8	76.5	62.9	73.29	74.55	63.03	111.03	73.743	114.04	286.6	108.2	130.5	61.31	72.57	68.12	165.6	150.4	101.8	88.73	78.26	43.86	179	150	159	8
Os03g0343900	1775	1804	2394	2008	1309	1323	2693	3061	3143	4176	5089	7038	2580.9	2090	2574.7	3224	3005	3600	3214	2204	3496	2081	1772	2564	4090	4367	3566	1581	2043	2126	20
Os03g0218300	198	241	221	308.7	338.1	311.4	88.5	157	70.5	78.46	116.2	38.57	156.75	121.35	182.94	203	132.9	203.1	65.96	99.17	95.71	230.7	222.3	153.8	120.4	93.34	57.02	239	180	310	8
Os03g0107500	1.02	0	1.08	2.463	1.808	2.595	0.78	0	2.07	2.587	0	0	3.2656	0.9335	0	0	1.031	1.036	1.858	0	0	0.739	0	2.889	1.056	0.943	0	1.85	0	1.67	2
Os03g0153500	64.2	82.9	80.8	23.81	17.18	39.79	6.26	8.22	5.53	117.3	62.49	41.4	48.983	57.874	49.102	71.65	25.76	32.12	7.432	12.09	13.8	7.394	15.68	7.945	95.07	50.91	111.8	96.2	150	117	3
Os03g0236300	0	0	1.08	0	0.904	0	0.78	0	0	0	0	0	0	0	0	0	1.031	0	0	0	0	0	0	2.167	0	0	0	0	0	0	9
Os03g0310500	560	572	570	562.4	538.8	492.2	475	503	534	666.5	616.2	583.3	630.25	693.55	672.38	752.3	680.2	547	575	541.8	569.9	530.9	549.4	530.1	646.5	643	627.2	632	567	513	13
Os03g0820900	294	208	278	134.6	110.3	159.2	218	172	169	296.6	246.7	338.7	271.04	274.43	267.68	382.1	284.4	223.8	196.9	198.3	244.9	138.3	145.4	159.6	287.3	277.2	331.1	226	242	249	3
Os03g0601500	0	0	0	0	0	0	0	0	0	0	0	0	0	0	0	0	0	0	0	0	0	0	0	0.722	0	0	0	0	0	0	9
Os03g0782400	13.3	6.52	7.54	1.642	2.712	1.73	0	0	0	26.73	26.31	27.28	22.859	16.802	15.839	71.65	9.275	17.61	1.858	2.419	0	0	0	0	17.96	15.09	30.7	9.25	18.2	8.33	13
Os03g0608200	140	139	214	190.5	208.8	161.8	103	149	122	122.4	109.6	95.02	130.62	134.42	133.05	226.9	144.3	198.9	140.3	132.2	132.8	164.1	136.8	124.9	146.8	146.1	89.91	142	208	195	8
Os03g0578900	1.02	0	0	0	0	0.865	0	3.16	2.76	0	0	0	0	0	0	0	0	0	0.929	1.613	0	0.739	0.713	1.444	0	0	0	0	0	0	18
Os03g0659233	0	0	0	0	0.904	0	0	0	0.69	0	0	0	0	0	0	0	0	0	1.858	0	1.724	0	0	0	0	0	0	0	0	0	16
Os03g0737600	237	244	304	269.3	289.3	263	240	252	230	278.5	275.2	249.3	254.71	287.5	296.99	346.3	243.2	269.4	242.5	270.9	271.6	253.6	230.9	203	252.5	287.6	313.6	261	274	263	13
Os03g0370400	1.02	0	0	3.284	0	10.38	0	0	0	0	0	0	1.0885	0	0	0	8.244	0	0	0	0.862	0.739	0	0	0	0	0	0	0	0	2
Os03g0839100	522	443	508	532	542.4	461.9	461	503	415	430.2	447.3	482.6	459.36	511.53	522.7	847.8	499.8	442.4	436.6	473.3	435.4	487.3	550.2	470.2	524	562.9	513.1	528	504	479	6
Os03g0109900	5794	5527	6534	4326	4451	3715	5139	6163	5316	5998	7522	5684	6044.6	5043.4	6127.4	8072	5571	6954	4357	4776	3889	5922	4190	5358	4485	6073	4180	6466	6034	6057	13
Os03g0211600	224	251	248	572.2	622.8	568.3	359	471	398	210.4	219.3	237.1	280.84	226.83	226.5	358.2	141.2	247.6	387.4	427.3	430.2	337.9	312.1	322.8	219.7	192.3	125	248	253	287	10
Os03g0803900	380	393	356	218.4	169.9	186	403	293	336	414.7	430.9	348.1	365.74	429.39	395.19	346.3	321.5	299.4	198.8	227.4	218.1	386.7	347.1	343.8	459.5	393.2	427.6	404	319	372	3
Os03g0655600	710	741	742	986.8	899.4	917	911	1025	1063	891.5	908.9	816.6	915.45	638.48	875.91	1146	578.1	634.1	769.2	811.1	733.7	1020	922.9	964.9	798.6	734.5	690.8	636	689	796	18
Os03g0378200	11.2	4.66	9.7	9.031	3.616	2.595	7.05	4.43	4.15	3.449	3.289	12.23	2.177	5.6007	11.879	0	5.153	6.216	1.858	2.419	3.449	5.915	6.414	1.444	3.169	9.428	6.579	11.1	1.91	8.33	19
Os03g0174300	130	125	136	261.1	291.1	281.1	131	137	124	100	137	100.7	96.878	116.68	147.31	238.8	169	143	228.5	221.7	268.1	162.7	191.7	186.3	135.2	139.5	96.49	137	118	146	10
Os03g0351500	3402	2865	3513	564	452	890.1	950	558	511	5430	4111	8021	4349.7	5341.2	3854.5	4932	3836	3703	1476	1220	1375	612.2	629.3	831.3	4123	5718	6066	4220	4226	3501	3
Os03g0805100	164	146	121	43.51	33.45	64.01	98.6	50	36.6	69.84	87.71	60.21	60.957	77.476	40.39	191.1	158.7	114	41.8	16.93	49.15	53.98	34.21	52	85.57	110.3	127.2	71.2	88.9	69.1	13
Os03g0392600	0	0	3.23	0.821	3.616	0.865	2.35	0	0.69	1.724	0	0	0	0.9335	0	0	0	0	0	0	0.862	0	1.425	0	1.056	0	0	0	0	0	2
Os03g0221800	461	414	469	389.1	355.3	301.9	453	502	417	344.9	366.2	250.3	364.65	361.25	363.51	597	462.7	438.2	319.6	322.5	315.6	435.5	434.7	444.2	381.3	381.9	302.6	371	383	437	6
Os03g0263000	1.02	0	1.08	0	0	0	0	0	0.69	0	0	0	0	0	0	0	0	0	0	0	0	0	0	0	0	0	0	0	0	0	19
Os03g0112400	900	836	925	710.1	767.5	773.4	1011	957	916	1060	961.5	1052	1047.2	1030.5	1126.2	1791	975.9	862	976.3	950.6	857.9	705.4	798.9	746.1	885.2	941	1064	921	946	876	13
Os03g0201901	6.12	10.2	8.62	9.852	2.712	7.785	19.6	17.7	24.2	35.35	21.93	17.88	18.505	22.403	19.007	35.82	11.34	14.5	9.29	8.063	11.21	19.22	43.47	22.39	19.01	41.49	13.16	29.6	39.2	32.5	1
Os03g0163200	109	99.7	114	167.5	180.8	195.5	261	392	466	148.3	185.3	109.1	169.81	105.48	129.09	167.2	88.63	101.5	105.9	137.1	113.8	287.6	217.4	251.3	116.2	81.08	59.21	94.4	78.4	103	18
Os03g0125000	2120	2446	2276	4025	4391	3980	7002	6494	7117	2736	3320	4721	2653.8	1902.4	1799.3	3045	1535	1948	4287	4371	4721	3536	3386	5195	2044	2029	1789	1016	1346	1294	12
Os03g0223550	3.06	4.66	8.62	7.389	5.424	1.73	6.26	14.5	15.9	2.587	6.578	8.467	7.6196	1.8669	3.1679	0	7.214	12.43	0.929	9.675	4.311	9.612	6.414	7.945	2.113	0.943	8.772	5.55	8.61	5.83	18
Os03g0570800	485	656	454	111.7	65.09	96.02	58.7	56.9	44.2	145.7	205	176.9	126.27	149.35	144.93	453.8	324.6	373	32.51	64.5	38.8	255.8	113.3	89.56	178.5	160.3	160.1	425	377	388	14
Os03g0444900	179	174	212	271.7	275.7	250.9	338	393	286	204.3	235.7	213.6	236.21	231.5	258.18	346.3	195.8	219.6	199.7	216.1	222.5	295	285.8	323.6	196.5	205.5	186.4	218	249	226	18
Os03g0827600	8.16	13	8.62	17.24	15.37	18.17	16.4	10.8	8.98	2.587	10.96	3.763	5.4426	22.403	12.671	11.94	8.244	11.4	11.15	9.675	3.449	24.4	16.39	20.22	12.68	12.26	4.386	12	8.61	8.33	9
Os03g0144150	82.6	98.7	84.1	120.7	124.7	83.04	63.4	79.7	62.9	40.52	64.69	46.1	48.983	66.275	68.109	71.65	79.35	100.5	73.39	95.95	60.36	157.5	116.9	75.84	59.16	63.17	50.44	95.3	137	85.8	8
Os03g0345901	144	215	161	152.7	156.4	149.7	87.7	158	140	128.5	150.2	111	115.38	112.01	149.68	155.2	183.4	134.7	85.46	124.2	105.2	227.7	232.3	216	159.5	205.5	146.9	227	187	226	1
Os03g0339150	52	51.2	64.7	65.68	67.8	77.85	68.1	50	52.5	43.11	36.18	42.34	28.302	68.142	29.303	95.53	46.37	46.62	80.82	82.24	100.9	62.85	57.01	65	41.2	33.94	65.79	55.5	44.9	45.8	10

Os03g0144951	0	1.86	0	0	0	0	0	0	0	0.862	0	0	0	0	0	0	1.031	0	0	0	0	0.739	0.713	1.444	0	0.943	0	0	0	0	9
Os03g0180100	290	251	353	140.4	79.55	141	37.6	95.5	63.6	275	350.8	214.5	217.7	267.9	397.57	286.6	189.6	255.9	140.3	162.1	123.3	131.6	233.7	180.6	347.5	320.6	361.8	340	314	362	3
Os03g0325400	52	41.9	49.6	56.65	48.81	56.23	55.6	80.3	95.4	56.9	59.21	54.57	72.931	92.412	69.693	47.76	65.95	47.66	54.81	86.27	69.84	67.28	57.01	76.56	51.76	60.34	63.59	56.4	58.3	70	15
Os03g0795500	964	762	806	633.8	712.3	760.4	1044	879	1064	931.2	912.2	1153	871.9	961.46	885.42	1206	962.5	871.3	887.2	749	850.1	728.3	735.4	891.3	969.7	1107	1118	754	776	775	20
Os03g0212600	2.04	1.86	2.16	1.642	0	0	0	0.63	0	0.862	0	2.822	0	0	0.792	0	0	0	1.858	0	0	0	1.425	2.167	0	1.886	0	0	0	0	19
Os03g0326600	368	384	422	262.7	264	248.3	232	221	214	469	517.5	438.4	624.81	590.87	557.54	1134	459.6	478.6	227.6	295.1	233.7	223.3	213.8	190	501.8	523.3	453.9	538	586	559	13
Os03g0793500	673	683	636	582.1	642.7	528.5	668	626	671	669.1	726.9	778.1	660.73	708.49	654.96	871.7	812.1	710.7	732	615.2	647.5	602.6	574.4	671.7	726.8	720.3	934.2	643	701	662	13
Os03g0296400	788	669	700	834.1	860.6	844.3	819	703	793	933.7	745.5	1106	801.15	830.77	819.68	1266	844	700.4	972.6	810.3	904.5	615.9	616.4	725.1	752.1	915.5	870.6	659	694	627	6
Os03g0138900	248	279	251	355.5	400.5	361.6	398	392	361	403.5	468.2	541	453.91	318.31	326.29	358.2	276.2	246.6	430.1	483	469.9	245.5	277.9	359.7	369.7	339.4	304.8	156	243	228	15
Os03g0448700	1340	1526	1487	1457	1494	1441	2090	2397	2016	1625	1798	1815	1424.9	1349.8	1585.5	1493	1519	1483	1428	1688	1351	2162	2074	1872	1709	1713	1822	1472	1445	1590	18
Os03g0336500	442	400	447	551.7	530.6	487.9	528	569	600	444	436.4	380.1	493.1	547	401.53	453.8	368.9	422.7	525.8	513.6	546.6	581.9	642.1	563.4	364.4	321.5	392.5	352	366	372	12
Os03g0797700	537	533	532	509.8	558.7	519	825	853	854	638.9	628.2	669.9	683.59	635.68	672.38	1099	660.6	532.5	537.9	548.3	499.2	627.7	669.2	693.4	670.8	595.9	521.9	538	513	623	6
Os03g0208900	671	609	693	710.1	607.5	741.3	957	902	965	688	581.1	734.8	612.84	707.56	680.3	1314	617.3	652.7	849.1	724	757.9	860.6	911.5	897	631.7	674.1	616.2	661	695	578	6
Os03g0794900	1715	1931	1727	1951	1885	1846	5788	4404	5053	2554	2276	2857	2176	2041.5	2200.1	4096	1820	1949	2108	1948	2264	3595	3122	3846	2365	2414	2610	1920	1770	1969	18
Os03g0390000	1560	1353	1253	1686	1725	1741	1642	1361	1670	1625	1312	2275	1366.1	1601.8	1166.6	2496	1181	1301	1645	1389	1652	1425	1445	1405	1182	1757	1855	974	1203	883	6
Os03g0427900	890	836	1002	902.3	1016	918.7	709	793	677	888.9	856.3	756.4	851.22	879.31	936.1	1457	892.4	884.8	709.7	801.4	749.3	865.8	877.3	806.7	852.5	853.3	719.3	936	882	829	6
Os03g0761900	36.7	42.8	40.9	46.8	54.24	64.88	36.8	46.2	35.2	32.76	44.95	52.69	33.744	27.07	22.967	35.82	32.98	27.97	46.45	27.41	30.18	50.28	36.34	41.89	13.73	18.86	35.09	20.4	14.3	12.5	2
Os03g0762100	0	0	0	0	0	0	0	0	0.69	0	0	0	0	0	0	0	0	0	0	0	0	3.697	15.68	13	0	0	0	0	0.96	0	17
Os03g0136800	436	400	408	307.9	270.3	319.2	384	402	417	544.9	510.9	598.4	545.35	553.54	541.7	573.2	548.2	453.8	400.4	370.9	449.2	348.3	330.7	384.2	521.8	615.7	585.5	432	490	440	3
Os03g0325600	0	0	0	6.568	0	7.785	0.78	0	2.07	0.862	0	0	0	0	0	0	0	0	0.929	1.613	1.724	0	19.95	9.389	0	1.886	2.193	0	0	0	17
Os03g0301500	0	0	1.08	0	0	0	0	0	0	0	0	0	0	0	0	0	0	0	0	0	0	0	0	0.722	0	0	0	0	0	0	9
Os03g0687700	0	0	0	0	0	2.595	0	0	0	0	0	0	0	0.9335	0	0	2.061	0	0	0	0	0	2.138	0	0	0	0	0	0	0	17
Os03g0721300	46.9	41	38.8	54.18	70.51	72.66	96.3	78.4	88.5	51.73	48.24	63.98	27.213	31.737	34.847	11.94	42.25	45.59	60.38	47.57	49.15	63.59	58.44	111.9	50.71	61.28	70.17	46.3	44	42.5	18
Os03g0114900	350	191	266	99.34	94.01	132.4	79.9	76.5	86.4	191.4	136	249.3	218.79	191.36	186.9	203	271	182.3	221.1	184.6	223.3	78.37	78.39	80.89	177.5	228.2	322.4	170	146	146	3
Os03g0268300	1328	1843	1434	1377	1548	1190	2743	2933	2791	1739	1896	1557	1407.5	1381.5	1831	2340	1420	1501	1261	1330	1267	2846	2463	2680	1847	1691	1636	1709	1590	1742	18
Os03g0815400	1866	2032	2179	3189	3379	3064	5283	4557	5304	2171	2577	3387	2504.7	1871.6	1672.6	2066	1489	1704	3703	3919	3507	3010	3010	4379	1853	1368	1298	1072	1335	1279	12
Os03g0428200	0	0	0	0	0	0	0	0	0	0	0	0	0	0	0	0	0	0	0	0	0	0	0	0	0	0	0	0	0	0.83	1
Os03g0731100	1008	1526	1336	1673	1927	1527	3763	3724	3559	1514	1778	1851	1280.1	1282.6	1176.9	2436	1340	1327	1807	1464	1624	2599	2389	2958	1570	1408	1401	888	962	1256	18
Os03g0681900	490	441	460	561.5	531.5	471.5	443	481	378	390.6	449.5	371.6	358.12	349.11	409.45	740.3	412.2	435.1	446.8	538.6	431.1	416.3	440.4	405.2	460.6	367.7	320.2	466	466	540	6
Os03g0385100	0	0	0	1.642	0	1.73	1.57	0	0.69	0	0	0	2.177	0	0	0	0	0	0	2.419	0	0	0	0	0	0	2.193	0	0	0	15
Os03g0621900	0	0	0	0	0	1.73	0	0	0	0	0	0	0	0	0	0	0	0	0	0	0	0	0	0	0	0	0	0	0	0	2
Os03g0324900	66.3	91.3	75.4	41.87	50.62	31.14	40.7	59.5	60.1	75.01	76.75	33.87	77.285	51.34	84.74	83.59	26.79	30.05	42.73	24.99	20.69	22.18	38.48	30.33	44.37	14.14	21.93	27.8	47.8	45	7
Os03g0442800	0	0	0	0	1.808	0	0.78	0	2.07	0	2.193	0	0	0.9335	0.792	0	0	0	0.929	0	0	0	0.713	0.722	0	0	0	0	0	0	18
Os03g0356540	0	0	0	0	0.904	0	0.78	0	0	0	0	0	0	0	0	0	1.031	1.036	0	0	0	0	2.851	0	0	0.943	0	0.93	0	0	17
Os03g0202200	0	0	0	0.821	0.904	0.865	0.78	0	0.69	0	0	4.704	0	0	0	0	3.092	0	0	0	0	0	0	0	0	0	0	0	0.96	0	20
Os03g0798300	8.16	5.59	12.9	17.24	8.136	20.76	9.39	10.1	11.7	12.07	9.868	12.23	15.239	8.4011	9.5036	23.88	16.49	4.144	11.15	16.93	11.21	14.79	13.54	5.778	16.9	17.91	15.35	20.4	27.7	15.8	1
Os03g0603300	377	410	399	378.5	382.4	380.6	444	449	460	457	445.1	388.6	436.5	437.79	483.89	632.9	389.5	377.1	421.7	424.9	447.5	375.6	426.9	408.8	399.3	446.9	361.8	389	397	378	6
Os03g0295866	19.4	17.7	21.6	50.08	54.24	47.58	34.4	37.3	40.8	40.52	18.64	31.99	35.921	41.072	14.255	11.94	37.1	32.12	40.87	43.54	67.25	24.4	38.48	42.61	26.41	40.54	21.93	15.7	23.9	17.5	10
Os03g0249300	366	371	349	560.7	569.5	551	409	401	402	415.6	393.6	441.2	537.73	463.93	447.46	764.2	359.7	436.2	637.3	637.8	702.7	408.1	410.5	387.8	386.6	432.8	449.5	404	400	435	16
Os03g0133400	1086	734	947	863.7	653.6	1039	314	399	502	642.3	485.7	702.8	248.18	462.99	538.54	644.8	647.2	678.6	752.5	944.2	867.4	453.2	659.2	561.2	443.7	552.5	800.4	323	383	260	19
Os03g0187350	0	0	0	0	0	0	0	0	0.69	0	0	0.941	0	0	0	0	1.031	0	0	0.806	0	0	2.851	0.722	0	0	0	1.85	0	0	17
Os03g0200600	176	229	221	309.5	311.9	314.9	239	257	246	154.3	194.1	118.5	148.04	140.95	204.33	370.2	206.1	187.5	263.8	242.7	266.4	252.9	277.2	234.7	203.9	166.9	193	229	217	192	6
Os03g0234350	1733	1572	1603	2144	2489	2066	2367	2219	2411	1680	1505	2273	1844	2007.9	1396.2	1182	1291	1525	1930	1408	1781	1644	1773	1720	1242	1548	1458	953	1148	1106	12
Os03g0133000	391	315	413	109.2	104.9	92.56	171	224	129	384.5	189.7	200.4	168.72	209.09	228.09	489.6	410.2	362.6	74.32	108.8	108.6	224	258	272.3	164.8	212.1	230.3	164	156	149	14
Os03g0800700	1024	1056	1317	433.5	400.5	514.7	700	700	578	1536	1337	1330	1417.3	1632.6	1519	1397	1025	953.2	639.1	541.8	543.2	414.1	512.4	425.4	1211	1059	1180	1170	1255	1091	3
Os03g0802300	442	552	530	502.4	526.1	510.4	784	820	776	510.4	491.2	449.7	643.32	564.74	666.84	979.2	472	396.8	488.6	449.9	435.4	570.1	562.3	564.1	548.3	513.8	469.3	504	493	530	6
Os03g0755000	355	308	315	286.5	292.9	341.7	308	313	324	309.5	333.3	316.1	299.34	333.24	373.02	489.6	418.4	351.2	335.4	316.1	282.8	317.9	349.9	291.8	344.4	374.3	326.7	379	327	325	13
Os03g0142500	6.12	13	3.23	9.031	4.52	5.19	15.7	8.86	15.2	7.76	6.578	5.645	10.885	14.673	10.296	0	3.092	4.144	0.929	1.613	0.862	29.58	44.9	22.39	1.056	6.6	2.193	2.78	4.78	7.5	17
Os03g0794500	150	122	103	184.7	267.6	232.7	266	238	177	144	72.36	161.8	85.993	79.343	74.445	107.5	74.2	78.74	225.7	152.4	130.2	154.5	158.2	188.5	91.9	237.6	471.5	61.1	142	97.5	11
Os03g0679700	556	711	617	33135	40723	32692	8456	8025	8590	307.8	282.9	255.9	419.08	399.52	342.92	812	534.8	725.2	28858	26644	32498	7185	6061	6497	262	295.1	328.9	565	566	586	10
Os03g0164400	241	223	213	268.5	329	263.8	270	281	196	201.8	211.6	276.6	273.22	266.03	263.72	609	231.9	273.5	317.7	305.6	273.3	267.7	290.8	270.8	228.2	273.4	184.2	235	256	253	6
Os03g0413000	546	574	533	416.2	320	377.2	427	448	457	553.5	553.7	976.6	664	477.93	537.74	680.6	668.8	651.7	467.3	419.3	598.4	313.5	274.4	400.1	581	578.9	611.8	345	373	450	20
Os03g0352500	1820	2096	1976	1676	1691	1682	1152	1313	1251	1749	1964	1609	2186.8	1779.2	2012.4	3785	1847	1931	1331	1509	1367	1474	1450	1423	2070	1891	1708	1997	2001	2094	13
Os03g0758600	1380	1465	1404	1230	1190	1225	858	869	807	1436	1488	1247	1389	1238.7	1550.7	2890	1544	1388	1288	1308	1156	879.1	997.7	983	1473	1428	1298	1751	1632	1498	13
Os03g0718650	0	0	0	0	1.808	0	0	0	0	0	0	0	0	0	0	0	0	0	0	0	0	0	0	0	0	0	0	0	0	0	2
Os03g0755600	39.8	24.2	30.2	16.42	13.56	28.55	19.6	12.7	32.5	39.66	38.37	22.58	40.275	61.608	44.35	59.7	66.98	31.08	64.1	27.41	28.45	28.84	28.51	26	32.75	40.54	61.4	52.7	55.5	46.7	13
Os03g0156500	704	679	698	587.8	543.3	574.4	929	974	904	905.3	858.5	1026	858.84	696.35	713.56	1516	755.4	645.5	596.4	593.4	480.3	798.5	713.3	806.7	801.8	859.9	964.9	715	644	710	6
Os03g0795000	976	967	1061	535.3	550.5	560.6	745	784	710	883.7	1002	906	1366.1	1176.1	1351.1	1815	1290	1112	646.6	641.8	644.1	586.3	641.4	571.3	1211	1157	853	1200	1195	1280	13
Os03g0661600	0	2.79	1.08	1.642	1.808	0	0	3.16	0.69	0	0	6.586	4.3541	1.8669	0.792	0	1.031	10.36	0	1.613	0	3.697	5.701	0	0	2.829	2.193	2.78	2.87	5	1
Os03g0363400	205	254	240	573.9	586.7	538.9	456	331	465	198.3	240.1	225.8	286.28	262.3	217.79	429.9	264.8	260	560.2	720	676	401.5	348.5	395.1	197.5	191.4	193	225	267	232	10
Os03g0775200	134	110	125	182.3	173.6	224.8	149	127	149	119	78.94	122.3	107.76	94.279	112.46	143.3	116.5	95.32	207.2	195.9	144	106.5	124.7	182	91.9	125.4	291.7	143	119	142	11
Os03g0731025	55.1	59.6	52.8	43.51	42.49	46.71	57.9	58.2	54.6	87.08	71.27	77.15	66.4	84.944	57.022	71.65	68.02	73.56	63.17	40.31	61.22	27.36	44.18	41.89	64.44	82.97	78.95	73.1	59.3	54.2	3
Os03g0338600	0	0	2.16	1.642	0	0	0	0	1.38	0.862	0	0	0	0.9335	0.792	0	1.031	0	1.858	0.806	0	0	1.425	0	0	0	0	0	0	0	7
Os03g0429800	2546	2843	2649	3036	3253	2389	2613	3022	2701	2997	3317	2274	3028.3	2362.6	2914.4	3129	2171	2420	2237	2409	2162	2138	2572	2269	2553	2009	2068	2073	2044	2347	7
Os03g0787150	2.04	0	0	0	0	0	0	0	3.46	0.862	1.096	0	0	0	0	0	0	2.072	2.787	0	0	0	0.713	3.611	0	0	0	0	0	0	9
Os03g0685300	266	248	248	36.12	21.7	27.68	12.5	20.2	9.67	244.9	302.6	287	279.75	303.37	309.66	274.6	242.2	207.2	30.66	12.09	7.76	11.83	22.8	20.22	256.7	289.5	346.5	202	198	262	3
Os03g0131100	1302	1792	1538	1043	949.2	1139	1484	1696	1534	1609	1914	1705	1729.7	1506.6	1593.4	2412	1376	1518	746.9	807.1	728.6	1528	1578	1489	1750	1752	1489	1643	1780	2022	13
Os03g0683866	425	443	431	425.3	410.4	373.7	409	429	396	412.1	410.1	373.5	411.46	408.85	505.27	788.1	458.6	498.3	337.2	331.4	340.6	395.6	434.7	374.8	425.7	380.9	377.2	500	495	577	13
Os03g0805600	8409	8859	9968	2580	2693	2652	2370	2695	2466	12093	13362	11833	12615	12145	14133	20168	10981	10731	3717	3691	4057	1712	1673	2213	12031	11916	10515	####	####	####	13
Os03g0708200	32.6	32.6	28	23.81	31.64	21.63	49.3	27.2	50.4	42.25	41.66	47.04	46.806	42.005	50.686	95.53	54.62	30.05	46.45	43.54	66.39	30.31	34.92	28.89	49.65	49.97	43.86	52.7	35.4	40.8	6
Os03g0784500	898	985	876	828.4	779.2	716.3	1457	1591	1323	1002	1036	954	861.02	958.65	1203.8	1445	1067	940.7	640	753.1	770.8	1496	1709	1611	1352	1300	1414	1373	1074	1211	5
Os03g0123500	0	0	0	0.821	0.904	0	1.57	2.53	3.46	0.862	2.193	0.941	0	0.9335	0	0	0	0	0	0	0	1.479	0	0	0	0	0	0	0	0	18
Os03g0733000	322	286	312	338.2	405	347.7	453	399	435	389.7	407.9	424.3	366.83	429.39	405.49	441.8	368.9	296.3	419.9	403.1	481.1	353.4	344.9	351.7	342.3	369.6	480.2	322	360	310	15
Os03g0737200	783	802	792	1020	1098	996.5	722	666	664	786.3	707.2	747	886.06	846.64	803.85	1146	767.7	884.8	986.5	924	965.7	735.7	834.5	624	734.2	724.1	752.2	822	849	764	10
Os03g0798200	151	176	173	198.7	176.3	196.4	279	210	216	189.7	196.3	171.2	164.37	197.89	193.24	214.9	194.8	172	162.6	191.9	186.2	217.4	224.5	223.9	211.3	183.9	212.7	207	153	217	18
Os03g0327600	487	283	290	202.8	240.5	308.8	102	62.6	87.1	195.7	143.6	258.7	140.42	286.57	140.97	179.1	230.8	259	332.6	200	269.9	79.85	84.09	78.72	166.9	158.4	201.7	128	166	125	19
Os03g0747100	4.08	3.73	1.08	5.747	4.52	4.325	32.1	18.3	31.1	6.897	14.25	9.408	1.0885	9.3345	0	11.94	6.183	7.252	13.93	13.71	16.38	29.58	12.11	26.72	5.282	8.486	17.54	5.55	9.56	3.33	18
Os03g0126850	1.02	5.59	0	1.642	0	0	0	1.9	0	0.862	0	0	1.0885	0	0	0	0	0	0	1.613	0	0	2.851	0	0	0.943	2.193	0.93	0	0	14
Os03g0582200	597	635	668	1009	1018	984.4	897	943	864	718.2	622.8	524	447.38	518.07	580.51	513.5	626.6	583.3	812.8	834.5	795	998.9	1149	836.4	565.2	469.5	592.1	582	522	517	12
Os03g0726800	99.9	142	136	147	142.8	162.6	106	119	124	138.8	143.6	128.9	138.24	165.22	141.76	298.5	127.8	154.4	134.7	171.7	175.9	180.4	172.5	145.9	159.5	143.3	114	171	147	168	6
Os03g0236983	10.2	1.86	3.23	12.31	5.424	19.03	35.2	15.8	22.1	12.93	15.35	13.17	7.6196	6.5342	6.3357	11.94	5.153	19.68	18.58	16.93	21.56	17.01	14.97	13	3.169	3.771	6.579	11.1	4.78	5	15
Os03g0271400	350	469	420	1144	1161	1002	341	378	354	438.9	599.7	505.2	587.8	376.18	502.11	620.9	315.3	356.4	915.9	1016	872.6	402.2	344.9	312	432.1	345.1	315.8	386	413	498	10
Os03g0309000	528	517	496	480.3	535.1	397.9	776	743	900	562.1	500	502.4	464.8	536.73	549.62	489.6	568.9	477.6	453.3	416.8	350.9	846.6	778.9	625.5	586.3	402.6	495.6	479	431	487	18
Os03g0298800	21.4	16.8	16.2	33.66	33.45	27.68	64.2	29.7	52.5	32.76	19.74	43.28	10.885	25.203	14.255	0	44.31	33.15	28.8	40.31	34.49	24.4	29.22	35.39	31.69	32.06	28.51	19.4	17.2	25	15
Os03g0399400	0	0.93	0	0	0	1.73	1.57	0	1.38	0	0	0	0	0	0	0	3.092	0	2.787	0	0.862	1.479	0.713	4.333	0	0	0	0	1.91	0	9
Os03g0215200	3.06	0.93	1.08	0.821	0	0	0	0	0	0	0	0	1.0885	3.7338	0	0	2.061	2.072	0	0	2.587	0	0	0	1.056	0	0	0	0.96	0	19
Os03g0639800	32.6	24.2	18.3	28.73	26.22	46.71	31.3	17.1	26.3	49.14	21.93	52.69	28.302	36.405	34.055	59.7	35.04	30.05	26.01	30.64	31.04	24.4	23.52	28.17	30.63	30.17	50.44	32.4	37.3	18.3	13
Os03g0270700	210	236	289	740.5	706.9	634.9	1228	1159	1025	348.3	255.5	334.9	299.34	407.92	354.8	441.8	319.5	207.2	791.5	786.1	874.3	659.5	880.8	782.2	357.1	361.1	267.5	378	399	338	12
Os03g0327100	278	248	200	315.3	257.6	363.3	168	226	216	80.18	70.17	12.23	137.15	93.345	104.54	525.4	398.8	231	191.4	282.2	262.1	233.6	332.1	244.1	79.23	50.91	57.02	105	117	110	6
Os03g0293900	291	290	239	443.3	358	344.3	189	208	216	297.5	274.1	335.9	372.27	319.24	336.59	573.2	298.9	325.3	366.9	499.1	547.5	258	233	174.8	236.6	248	278.5	304	318	260	16
Os03g0340001	117	142	144	166.7	203.4	210.2	77.5	87.3	87.1	96.56	115.1	104.4	87.082	128.82	114.84	143.3	144.3	128.5	200.7	212.1	214.7	110.2	100.5	71.5	125.7	140.5	118.4	125	130	130	16
Os03g0410700	282	364	339	477	582.2	538.1	888	938	873	431.1	484.6	526.9	480.04	383.65	405.49	489.6	374.1	366.8	589.9	599.9	650.1	583.4	543.7	699.1	416.2	436.5	359.6	327	367	337	18
Os03g0274000	4166	3063	3986	2900	2802	3325	2560	2663	3123	2662	2171	1702	2301.1	2388.7	2534.3	3893	2609	2934	2814	2922	2851	2711	3030	2438	2159	1973	2116	2021	2070	2072	14
Os03g0234500	0	0	0	0	0	0	0	0	0	0	0	0	0	0	0	0	0	0	0	0	0	2.958	0	2.167	0	0	0	0	0	0	9
Os03g0338200	365	423	454	347.3	340.8	327.9	281	269	250	279.3	360.7	234.3	376.63	350.98	404.69	668.7	381.3	426.8	248	288.6	256.9	503.5	419	358.2	399.3	354.5	348.7	499	478	499	13
Os03g0713400	1411	1358	1221	1043	1164	1121	914	938	972	1236	1129	1427	1144	1312.4	1191.1	1731	1338	1328	1091	972.4	1004	841.4	1061	1016	1245	1239	1406	1157	1190	1178	13
Os03g0800900	51	48.4	39.9	59.11	65.99	60.55	64.2	83.5	65	50.87	48.24	65.86	53.337	50.406	47.518	47.76	56.68	45.59	85.46	70.15	60.36	65.81	61.29	54.17	42.25	54.68	54.82	62	41.1	34.2	12
Os03g0129300	####	####	####	14969	14009	14952	####	####	####	65709	66516	81959	60576	58742	63048	81915	58514	53745	26188	20455	23423	10538	10516	15946	61661	67646	60020	####	####	####	3
Os03g0370500	108	66.1	72.2	330.9	330.9	319.2	114	95.5	135	106.9	48.24	127	94.701	102.68	67.317	203	60.8	74.59	321.4	297.5	297.5	130.9	120.4	89.56	55.99	106.5	105.3	50.9	79.4	40.8	10
Os03g0100030	0	0	0	0	1.808	1.73	0	0	0	0	0	0	0	0	2.3759	119.4	0	0	0	0	0.862	0	1.425	0	1.056	0	0	1.85	0	0	6
Os03g0302800	87.7	57.7	50.6	4.105	7.232	6.92	0	1.27	0	4.311	9.868	2.822	6.5311	4.6673	5.5438	35.82	72.14	55.95	3.716	4.838	7.76	0	1.425	7.945	3.169	2.829	0	0.93	0.96	2.5	14
Os03g0816700	6.12	3.73	2.16	0	0	0	0	0	0	0	0	0	0	0	0	0	5.153	1.036	0	0	0	1.479	0	0	0	0	0	0	0	0	14
Os03g0329200	0	0.93	2.16	1.642	0	0.865	6.26	0.63	5.53	0	1.096	0	0	0	0	0	0	0	0.929	3.225	3.449	0.739	0.713	5.778	0	0	6.579	0	1.91	0	15
Os03g0369600	0	0	2.16	1.642	0	1.73	0.78	1.9	3.46	0	4.386	0	0	0	0.792	0	1.031	1.036	0.929	8.869	1.724	1.479	0	3.611	0	0.943	0	0.93	1.91	0.83	16
Os03g0279000	2877	2330	2613	1639	1571	1577	2529	2560	2611	2718	2731	2674	2721.3	2204.8	2692.7	3260	2458	2376	1978	1914	1820	1794	1924	1893	2364	2273	2031	2399	2660	2864	13
Os03g0346900	290	289	304	211.8	191.6	194.6	200	201	167	264.7	237.9	263.4	292.81	304.31	279.56	800	310.2	290.1	228.5	222.5	190.5	211.5	213.8	198.6	319	338.5	300.4	342	300	351	13
Os03g0127950	219	190	209	87.84	91.3	82.18	8.61	12	9.67	146.6	138.1	67.74	180.69	125.08	171.86	226.9	201	178.2	58.52	64.5	43.11	42.15	36.34	33.95	170.1	115	100.9	253	186	237	13
Os03g0700800	1886	1953	1975	2147	2274	1951	695	747	717	1276	1293	911.7	1858.1	1491.7	1571.3	3045	1560	1615	1410	1577	1526	876.9	1025	788.7	1330	1109	975.8	1613	1623	1720	8
Os03g0659200	344	412	397	790.6	799.1	675.6	1556	1240	1501	433.7	595.3	588	433.23	238.03	227.29	465.7	248.4	337.7	1006	938.5	1076	899.1	778.2	1339	318	243.3	221.5	171	203	223	12
Os03g0222700	8.16	14.9	9.7	13.14	20.79	10.38	8.61	17.1	8.98	12.93	12.06	3.763	7.6196	9.3345	16.631	11.94	3.092	10.36	16.72	8.063	10.35	14.05	10.69	15.17	11.62	2.829	0	7.4	12.4	10	2
Os03g0705800	165	105	97	62.39	42.49	78.72	90.8	111	104	65.53	59.21	139.2	74.019	79.343	77.613	47.76	106.1	122.3	104	83.05	93.12	125.7	133.3	179.1	50.71	140.5	182	60.1	70.8	81.6	11
Os03g0399900	55.1	33.5	37.7	10.67	6.328	42.39	14.1	9.49	4.84	16.38	5.482	27.28	26.124	82.144	50.686	35.82	134	84.96	62.24	39.51	90.53	34.01	81.24	131.4	31.69	77.31	116.2	108	62.2	59.2	1
Os03g0559900	6.12	9.31	3.23	9.852	11.75	15.57	2.35	5.06	4.84	13.79	9.868	14.11	7.6196	7.4676	6.3357	11.94	12.37	9.324	8.361	14.51	18.11	11.83	6.414	17.33	12.68	21.69	26.32	11.1	9.56	5	11
Os03g0604600	1.02	6.52	3.23	13.96	11.75	4.325	4.7	3.8	1.38	3.449	3.289	5.645	0	1.8669	1.5839	0	1.031	0	0	1.613	3.449	5.176	8.552	3.611	2.113	0.943	2.193	1.85	0.96	1.67	2
Os03g0214600	648	659	687	865.3	884.1	881.5	827	954	872	757	700.6	750.8	716.25	734.63	714.35	1039	716.2	585.4	928	807.1	789.8	685.4	834.5	746.8	664.5	670.4	719.3	600	698	674	12
Os03g0578500	41.8	60.5	47.4	8.21	1.808	6.055	1.57	0	2.07	102.6	92.1	85.61	43.541	61.608	58.605	95.53	71.11	45.59	7.432	13.71	16.38	0.739	0	0	73.95	69.77	39.47	58.3	41.1	40	3
Os03g0861100	28.5	51.2	28	39.41	33.45	48.44	26.6	35.4	28.3	27.59	36.18	27.28	17.416	22.403	25.343	35.82	25.76	24.86	26.94	20.16	26.73	32.53	29.22	31.78	23.24	22.63	17.54	31.5	25.8	24.2	2

Os03g0226300	54	68	50.6	4.105	12.66	7.785	32.1	32.9	19.3	87.94	145.8	147.7	32.656	32.671	63.357	143.3	50.5	40.41	14.86	5.644	10.35	11.83	13.54	34.67	69.72	101.8	94.3	37	34.4	35	20
Os03g0712600	119	123	111	77.99	65.99	68.34	58.7	35.4	47	115.5	102	128.9	136.06	150.29	139.39	214.9	146.3	133.6	60.38	90.3	83.64	60.63	44.9	20.95	169	111.3	92.1	117	113	110	13
Os03g0282232	1.02	1.86	0	7.389	5.424	2.595	0.78	3.16	0	2.587	4.386	0.941	0	0.9335	5.5438	0	2.061	8.288	1.858	2.419	0	2.218	3.563	0.722	3.169	1.886	0	0	1.91	2.5	2
Os03g0161100	41.8	50.3	40.9	41.05	34.35	44.12	22.7	17.7	26.3	71.56	62.49	37.63	43.541	55.074	57.814	47.76	52.56	36.26	37.16	50.8	44.84	39.19	36.34	33.95	61.27	59.4	50.44	65.7	62.2	71.6	3
Os03g0223100	359	468	447	643.6	748.5	533.7	1019	1366	1013	799.2	778.4	436.5	587.8	455.52	733.36	453.8	262.8	355.4	605.7	577.3	332	587.1	931.4	650.7	517.6	386.6	245.6	419	429	509	18
Os03g0102200	82.6	76.4	74.4	103.4	93.11	70.07	150	135	123	99.15	97.58	123.2	75.108	93.345	77.613	71.65	128.8	118.1	130.1	97.56	181.9	138.3	110.5	115.6	89.79	127.3	164.5	110	119	102	15
Os03g0429900	0	0	0	0	0	0	2.35	0	0	0	0	0	0	0	1.5839	0	0	0	0	0	0.862	0	0	0	0	0	0	0	0	0	5
Os03g0625700	841	956	891	802.9	825.3	769.9	817	758	826	739.8	692.9	535.3	732.57	731.83	760.29	1039	868.7	734.6	668.8	670.8	661.3	926.5	873	827.7	872.6	738.2	780.7	869	777	871	8
Os03g0565600	428	382	448	715.1	853.3	705.9	1167	1135	1280	384.5	464.9	510.9	450.65	456.46	452.21	609	385.4	462.1	617.8	682.9	582	1380	1135	1065	526.1	399.8	458.3	514	450	531	18
Os03g0847900	34.7	23.3	20.5	18.06	28.02	17.3	39.9	34.2	38	41.38	37.28	68.68	9.7967	27.07	30.095	0	34.01	21.76	10.22	13.71	18.11	17.75	24.23	28.17	35.92	36.77	41.67	17.6	13.4	11.7	20
Os03g0629900	595	661	693	512.3	527.9	555.4	525	600	486	532	538.3	591.8	532.29	575.01	588.43	1146	820.3	747	576.9	659.5	619.1	581.2	515.9	584.3	627.5	606.2	620.6	748	917	788	13
Os03g0707600	526	524	519	504.9	513.5	461.1	783	664	818	638.9	571.2	694.3	465.89	434.99	450.63	1039	555.5	472.4	489.6	575.7	538.9	665.4	662.8	707.1	508.1	600.6	521.9	501	504	552	6
Os03g0733200	0	0	0	0.821	0	0.865	0	1.9	0	0	0	0	0	0	0	0	0	0	0	0	0	0	3.563	2.167	5.282	0	0	3.7	0.96	0.83	1
Os03g0181600	199	218	167	269.3	308.3	261.2	132	142	124	186.2	138.1	137.4	218.79	194.16	200.37	334.3	159.7	157.5	174.6	204	178.5	218.1	206.7	201.5	195.4	219.7	160.1	230	155	207	8
Os03g0283800	248	301	274	312.8	303.7	303.6	247	215	250	263.8	293.8	317.1	314.58	304.31	315.99	513.5	294.7	329.5	259.2	271.7	269.9	327.5	364.9	275.2	285.2	325.3	309.2	320	273	364	6
Os03g0681201	4.08	5.59	16.2	13.96	10.85	8.65	1.57	1.9	1.38	0.862	15.35	3.763	1.0885	3.7338	3.9598	11.94	9.275	12.43	3.716	7.257	4.311	6.654	9.264	2.167	9.507	5.657	0	13	6.69	5	8
Os03g0372600	1.02	1.86	4.31	6.568	1.808	8.65	5.48	2.53	2.76	2.587	3.289	2.822	2.177	0.9335	0.792	0	3.092	2.072	0.929	3.225	3.449	9.612	12.83	8.667	7.395	6.6	4.386	4.63	0.96	7.5	17
Os03g0356652	733	609	689	463.9	489	493.9	499	528	487	657	593.1	643.5	771.76	727.16	715.94	1003	827.5	693.1	579.7	570	615.6	416.3	426.2	403	751.1	617.6	703.9	760	645	704	13
Os03g0669300	141	97.8	90.5	82.1	72.32	79.58	79.1	50.6	116	62.94	60.3	123.2	31.567	62.541	41.182	59.7	106.1	109.8	85.46	74.98	67.25	103.5	94.78	142.3	65.49	81.08	87.72	47.2	48.8	48.3	19
Os03g0802200	88.7	37.3	71.1	19.7	28.93	22.49	19.6	22.8	34.6	48.28	28.51	47.04	35.921	54.14	63.357	59.7	43.28	50.77	15.79	17.74	19.83	39.93	40.62	56.34	29.58	38.66	63.59	24.1	37.3	37.5	19
Os03g0667800	215	202	194	307	308.3	257.8	499	373	481	286.2	224.8	298.2	261.24	249.23	202.74	489.6	165.9	185.5	427.3	331.4	394.9	237.3	301.4	290.3	201.8	191.4	203.9	133	187	136	12
Os03g0237000	1378	1456	1395	1545	1595	1755	1148	1130	1402	1232	1133	1043	997.08	1012.8	1080.2	1911	1213	1200	1301	1263	1342	1460	1589	1512	1250	1122	1377	1133	1114	1091	6
Os03g0758100	694	849	987	2800	2963	3437	202	180	182	385.4	361.8	252.1	1303	1785.7	993.92	1361	696.6	880.6	2739	1821	2433	87.25	57.01	51.28	281	239.5	274.1	1298	1622	1139	10
Os03g0307800	258	279	291	311.1	289.3	286.3	116	159	115	170.7	164.5	92.2	195.93	180.16	255.01	394.1	252.5	285.9	167.2	202.4	142.3	207	214.5	139.4	234.5	194.2	166.7	285	297	322	8
Os03g0168700	0	4.66	1.08	0.821	0	0.865	1.57	0.63	0.69	0.862	2.193	0.941	2.177	0.9335	0	0	0	3.108	5.574	0	0	2.958	2.138	5.778	8.451	1.886	0	0.93	0.96	2.5	9
Os03g0135800	4.08	0	5.39	28.73	38.87	40.66	9.39	13.9	15.9	0	2.193	2.822	4.3541	0.9335	0	0	0	1.036	20.44	23.38	36.21	17.01	22.09	16.61	0	0	0	1.85	0.96	0.83	10
Os03g0712100	0	0	0	0	0	0	0	0	0	0	0	0	0	0	0	0	0	0	0	0	0	0	0	0.722	0	2.829	6.579	1.85	0	0	11
Os03g0608700	592	785	705	527.1	548.7	708.5	636	524	645	549.2	618.4	599.3	576.92	611.41	623.28	955.3	896.6	894.1	688.4	669.2	647.5	899.8	744.7	959.1	676.1	697.7	997.8	848	756	815	1
Os03g0356582	270	219	289	183.9	203.4	193.8	195	211	196	246.6	291.6	252.1	338.53	312.71	362.72	429.9	388.5	396.8	218.3	304.8	309.5	159	151.1	160.3	320.1	300.8	221.5	368	298	354	13
Os03g0282700	1.02	1.86	0	0	0.904	0	0.78	0	0	1.724	0	1.882	0	0	0	0	0	0	0	0	0	0	0	0	0	0	0	0	0	0	7
Os03g0227700	296	189	239	431	368.8	565.7	270	433	359	305.2	263.1	411.1	293.9	340.71	272.44	179.1	350.4	300.5	1026	808.7	990.7	327.5	304.3	397.2	345.4	345.1	484.6	241	335	318	16
Os03g0572900	3046	2227	2953	404.7	388.7	380.6	279	431	283	3076	3448	3597	3987.2	4006.4	4032.7	7475	4673	3926	859.3	848.2	666.5	275.1	358.5	254.2	4064	4322	3678	3972	4472	4313	13
Os03g0836600	380	360	444	427.7	458.3	413.5	252	269	284	371.6	398	444.1	537.73	465.79	412.61	692.6	446.2	444.5	458	452.3	498.4	315.7	280.8	302.6	450	496.9	482.4	384	452	442	13
Os03g0750300	22.4	26.1	11.9	22.17	23.5	29.41	25.1	15.2	27.6	19.83	8.771	11.29	10.885	12.135	11.088	11.94	28.85	11.4	19.51	26.61	29.32	22.92	23.52	32.5	20.07	15.09	13.16	18.5	6.69	26.7	12
Os03g0744800	1124	1081	1119	925.2	980.8	1011	908	942	935	955.3	970.3	986.9	1048.2	1052.9	1070.7	2388	1121	1050	1051	913.5	857.9	919.1	984.1	918.7	1147	964.5	1136	1100	1013	1052	6
Os03g0727200	0	0	0	0	0	0	0	0	1.38	0	0	0.941	0	0	0	11.94	2.061	0	2.787	1.613	0.862	0.739	0	0	0	0	0	1.85	1.91	0	6
Os03g0343800	0	0	1.08	2.463	0	0.865	0.78	1.27	1.38	0	0	1.882	0	0	0	0	2.061	0	0.929	0.806	0	0	2.138	1.444	0	0	2.193	4.63	3.83	0.83	1
Os03g0856300	74.4	80.1	84.1	96.88	87.69	93.43	91.6	58.2	77.4	78.46	67.98	122.3	62.046	76.543	47.518	143.3	71.11	60.09	85.46	84.66	97.43	40.67	28.51	66.45	52.82	74.48	122.8	44.4	65	60	6
Os03g0762700	255	269	254	257	207	243.9	267	219	262	213.8	218.2	182.5	197.02	236.16	273.23	406	246.3	264.2	206.2	220.9	293.2	309.8	293.6	309.8	258.8	232.9	230.3	243	244	255	6
Os03g0100300	89.7	68.9	88.4	41.87	33.45	66.61	64.2	45.5	44.2	125.9	104.2	148.6	92.524	69.075	71.277	155.2	71.11	65.27	70.6	41.12	53.46	56.93	69.84	85.95	109.9	98.06	78.95	71.2	89.9	45	13
Os03g0858400	423	464	383	664.2	713.2	592.6	907	838	786	490.6	481.3	491.1	361.39	439.66	424.49	656.8	394.7	432	591.7	587	486.3	663.2	662.8	691.9	452.1	448.8	478.1	395	377	427	18
Os03g0633400	883	826	948	558.3	577.6	602.9	925	887	850	851	902.3	1000	1249.6	1080.9	1132.5	1994	1200	1111	770.1	770	782	684.7	677	825.5	998.3	1087	986.8	1053	1051	1056	13
Os03g0748700	586	568	658	381.8	406.8	403.1	243	290	220	541.5	624.9	620	657.47	600.21	737.32	680.6	608	472.4	370.7	384.6	371.6	295.8	324.2	319.2	840.9	682.6	585.5	748	661	728	3
Os03g0278566	43.8	38.2	40.9	33.66	51.53	42.39	57.2	44.9	38	58.63	51.53	32.93	47.895	45.739	42.766	95.53	56.68	40.41	39.02	45.15	40.52	49.54	38.48	47.67	35.92	48.08	32.89	56.4	52.6	67.5	6
Os03g0381100	94.8	118	109	128.1	151	153.1	50.1	60.1	71.9	85.36	95.39	65.86	102.32	100.81	100.58	167.2	83.47	111.9	88.25	111.3	128.5	99.08	78.39	80.17	98.24	66.94	59.21	94.4	108	87.5	8
Os03g0713200	38.7	40.1	32.3	161.7	183.5	144.5	116	182	115	50.87	55.92	71.5	41.364	27.07	39.598	0	23.7	26.94	119.8	154.8	137.1	70.24	85.52	104.7	47.54	45.26	35.09	20.4	11.5	15.8	12
Os03g0392400	468	467	483	417.9	412.2	423.9	493	411	486	578.5	551.5	592.7	603.04	518.07	538.54	752.3	477.1	465.2	437.5	434.6	398.3	342.3	387	453.6	496.5	487.4	526.3	486	496	433	13
Os03g0668500	62.2	121	95.9	76.35	52.43	72.66	157	131	207	154.3	122.8	167.5	141.51	140.02	167.1	95.53	117.5	96.35	73.39	77.4	94.84	175.2	182.4	178.4	199.7	159.3	197.4	148	155	167	5
Os03g0271200	771	775	725	1607	1897	1492	668	676	583	559.6	609.6	525	771.76	791.57	607.44	1433	486.4	666.2	1125	1122	990.7	618.1	583.6	551.1	464.8	409.2	530.7	451	579	579	10
Os03g0841900	2759	2335	2764	1971	1958	2012	1483	1998	1671	1872	2162	2194	3851.2	3212	3166.3	5624	3368	3220	2014	2424	2131	1777	1762	2056	2745	2670	2070	3327	3264	3371	13
Os03g0227750	0	0.93	0	0	0	0	0	0	0	0	0	0	0	0	0	0	0	0	0	0	0	0	0	0	0	0	0	0	0	0	14
Os03g0155300	0	0	0	0	0	0	0	0	0	0	4.386	0	1.0885	0	0	0	0	0	0	0	0	0	0	0	0	0	0	0	0	2.5	7
Os03g0103800	2742	3415	3345	2821	3080	2567	3379	3841	3594	2669	3148	2463	2589.6	2152.5	2733.9	5111	2847	3034	2112	2606	1933	5542	4476	3868	2946	2803	2588	3031	2799	3181	17
Os03g0799500	277	264	263	278.3	294.7	250	324	318	346	294	280.7	306.7	256.89	297.77	282.73	406	243.2	280.8	290.8	291.9	316.4	345.3	300	306.2	321.1	341.3	342.1	273	206	294	6
Os03g0147001	19.4	42.8	21.6	19.7	29.83	20.76	27.4	21.5	31.1	25.87	44.95	37.63	30.479	38.272	41.182	83.59	26.79	29.01	15.79	26.61	23.28	22.18	29.22	20.95	43.31	35.83	30.7	25.9	36.3	20	13
Os03g0142200	0	0	1.08	0.821	0	0	0	0	1.38	0	3.289	0	0	0	0	0	0	0	0	2.419	0	1.479	2.851	0	0	0	2.193	0	0	0	17
Os03g0280700	103	127	125	252	203.4	215.4	202	152	182	104.3	89.9	69.62	91.436	87.744	77.613	179.1	112.3	105.7	114.3	162.9	162.1	188.5	215.2	199.3	83.45	86.74	61.4	115	95.6	103	12
Os03g0315800	7515	7882	8073	8245	9117	8059	####	####	####	7535	7851	11102	8961.8	8279.7	6984.3	11416	9290	9466	10646	10039	11136	8795	8044	11857	7560	7275	6780	6706	7108	7204	18
Os03g0782200	1878	1397	560	0	0	0	1.57	0	0	41.38	33.99	1203	701.01	184.82	15.839	167.2	3497	1367	16.72	2.419	2.587	0	0	0	370.8	3385	4993	297	865	177	11
Os03g0734400	67.3	55	40.9	53.36	56.05	59.69	50.9	46.2	49.8	60.35	40.57	64.92	43.541	54.14	45.142	47.76	55.65	34.19	69.67	62.89	71.56	45.84	42.76	44.06	50.71	64.11	111.8	43.5	41.1	40	11
Os03g0767800	999	920	941	750.4	790.1	828.7	1098	1053	929	1008	971.4	1048	784.82	835.44	749.99	1314	840.9	820.5	839.8	707.1	683.7	761.6	850.9	820.5	882.1	857	1094	838	768	811	6
Os03g0152800	378	449	374	430.2	457.4	474.9	357	397	398	397.5	405.7	352.8	366.83	363.11	387.27	656.8	385.4	359.5	340.9	379.8	369.9	445.1	462.5	389.3	423.6	396	412.3	433	415	443	6
Os03g0265666	19.4	23.3	16.2	18.88	18.08	19.03	8.61	3.16	6.22	4.311	15.35	6.586	8.7082	12.135	9.5036	11.94	8.244	14.5	5.574	4.031	12.07	25.14	24.23	7.945	8.451	5.657	8.772	21.3	6.69	22.5	8
Os03g0735100	0	0.93	0	0.821	1.808	0	0	0	0	0	1.096	0	0	0.9335	0.792	0	0	0	2.787	0.806	0.862	0	0	0	1.056	0	0	0	1.91	0.83	16
Os03g0705550	0	0	1.08	0	0	0	0	0	0	0	0	0	0	0	0	0	0	0	0	0	0	0	0	0	0	0	0	0	0	0	14
Os03g0570300	459	399	427	369.4	411.3	305.4	266	244	271	390.6	346.5	477.9	408.19	384.58	401.53	453.8	379.2	359.5	301	323.3	293.2	218.1	282.2	248.5	425.7	341.3	379.4	334	361	339	3
Os03g0603800	135	125	145	208.5	223.3	211.9	211	227	200	173.3	143.6	168.4	167.63	147.49	141.76	262.7	109.2	130.5	228.5	245.1	275	147.9	183.1	187.1	173.2	183.9	171	137	141	146	10
Os03g0799900	551	602	627	381.8	313.7	353.8	132	159	162	466.4	583.3	526.9	893.67	561	681.88	1158	493.6	679.6	263.8	342.7	227.6	238.8	180.3	192.1	616.9	480.8	495.6	621	607	756	13
Os03g0247200	32.6	48.4	40.9	39.41	27.12	19.9	18.8	29.7	36.6	48.28	39.47	50.8	51.16	57.874	44.35	35.82	46.37	33.15	18.58	20.16	26.73	33.27	23.52	27.45	49.65	56.57	28.51	50.9	44	40	3
Os03g0146900	0	0	0	0	0	0	0	0	0	0	0	0	0	0	0	0	0	0	0	0	0	2.958	0	0	0	0	0	0	0	0.83	9
Os03g0314100	1416	1461	1418	1247	1241	1328	820	878	859	1226	1310	1328	1483.7	1374	1413.7	2687	1503	1550	1003	1332	1193	1200	1060	944	1477	1502	1292	1742	1646	1665	13
Os03g0839700	2.04	1.86	0	3.284	0	4.325	2.35	0.63	0	2.587	2.193	2.822	0	3.7338	0	0	2.061	0	0.929	0	1.724	3.697	1.425	0	0	8.486	6.579	0.93	5.74	0.83	11
Os03g0230300	59.1	56.8	71.1	45.15	33.45	46.71	21.9	30.4	15.2	38.8	26.31	44.22	39.187	40.138	71.277	119.4	64.92	66.31	31.58	28.22	17.24	30.31	69.84	39.72	53.87	58.46	72.37	70.3	86.1	59.2	13
Os03g0741050	722	400	491	886.7	521.6	1112	144	432	228	289.7	225.9	63.98	216.62	227.76	525.07	752.3	451.4	582.3	344.6	751.5	429.4	2207	1607	584.3	389.8	386.6	412.3	915	785	801	17
Os03g0438200	22.4	12.1	10.8	9.031	9.04	20.76	4.7	3.16	2.76	17.24	3.289	14.11	5.4426	8.4011	7.9197	0	13.4	10.36	16.72	14.51	10.35	3.697	6.414	3.611	9.507	7.543	10.96	10.2	12.4	5.83	19
Os03g0634000	774	770	808	1949	2012	1931	2150	1660	2000	1415	1467	1836	1102.7	854.11	818.1	871.7	659.5	769.8	2635	2783	2703	1165	961.3	1340	1167	1146	1105	571	607	678	15
Os03g0762500	98.9	116	73.3	55.01	16.27	32.87	34.4	20.2	22.8	25.87	23.02	47.04	19.593	21.469	14.255	95.53	152.5	76.67	40.87	44.35	70.7	46.58	31.36	123.5	15.85	51.86	35.09	22.2	29.6	46.7	14
Os03g0172100	28.5	30.7	22.6	270.1	117.5	369.4	408	618	509	12.93	12.06	24.46	46.806	32.671	26.927	11.94	24.73	14.5	221.1	154.8	199.2	476.2	334.2	482.5	15.85	11.31	32.89	23.1	17.2	15.8	18
Os03g0843300	997	991	859	598.5	609.3	747.4	462	466	522	691.5	648	620.9	443.03	681.42	545.66	1063	906.9	825.7	547.2	447.5	531.1	499.8	519.5	548.2	667.6	697.7	879.4	827	730	670	13
Os03g0360700	2345	2249	2345	2134	2204	2082	2627	2575	2276	3072	2671	3394	2843.2	3329.6	2649.1	6544	2807	2416	3221	3111	2832	1464	2030	2148	2923	2496	2890	2736	2398	2420	6
Os03g0257900	105	124	106	160.9	195.3	185.1	185	149	125	109.5	116.2	112.9	115.38	104.55	122.75	334.3	86.56	127.4	196	224.1	181.1	118.3	133.3	141.6	107.7	95.23	100.9	111	94.7	121	6
Os03g0130750	118	87.6	103	146.1	160.9	180.8	193	187	200	111.2	93.19	100.7	120.83	110.15	111.67	191.1	72.14	76.67	151.4	162.1	183.7	144.9	164.6	148.8	87.68	79.2	109.6	86	110	98.3	12
Os03g0815900	1310	1437	1320	1113	1141	1038	2057	1937	2070	1465	1429	1489	1574	1387.1	1534	1636	1334	1198	1082	955.4	1042	1604	1430	1522	1358	1261	1320	1263	1170	1241	5
Os03g0681700	343	434	412	458.1	499.9	388.4	543	578	549	505.2	487.9	459.1	470.24	453.66	450.63	573.2	344.2	371.9	412.5	358	350.1	351.9	393.4	424.7	441.6	391.3	418.8	354	385	443	5
Os03g0570775	1.02	1.86	0	0	0	0	0	0	0	1.724	2.193	2.822	1.0885	0	1.5839	0	0	1.036	2.787	0.806	0	1.479	0	0.722	0	0	0	2.78	0.96	0	20
Os03g0666700	83.6	97.8	100	104.3	97.63	118.5	112	96.1	84.3	107.8	125	89.38	66.4	79.343	64.941	47.76	86.56	75.63	82.68	75.79	73.29	85.03	75.54	112.7	76.06	92.4	109.6	91.6	66	81.6	7
Os03g0193075	177	194	187	142	169.9	142.7	336	317	287	233.7	232.4	197.6	194.85	202.56	213.83	441.8	179.3	160.6	195.1	158.8	183.7	222.6	193.1	242.7	158.5	178.2	219.3	158	180	189	6
Os03g0389900	992	1002	1058	1032	1014	1087	705	862	750	838	845.3	637.9	802.24	855.04	858.49	1648	958.4	1020	854.6	883.7	863.1	1125	970.6	905	924.3	772.2	756.6	1028	965	886	6
Os03g0169400	28.5	28.9	31.2	164.2	151	146.2	153	152	149	71.56	91	50.8	71.842	44.806	52.27	35.82	21.64	26.94	196	206.4	157.8	82.81	103.3	104	59.16	37.71	28.51	25.9	39.2	30	12
Os03g0352400	1946	2175	2057	2114	2046	1859	3838	3854	3530	2313	2198	2341	1805.9	1867.8	1808.9	3654	1722	1892	1920	1847	1783	2644	2527	2742	1773	1877	1899	1589	1567	1516	18
Os03g0667400	164	161	168	153.5	151	161.8	144	135	168	136.2	141.4	88.44	140.42	159.62	132.26	214.9	95.84	128.5	151.4	163.7	152.6	139	160.3	135.1	131	122.6	100.9	111	117	134	6
Os03g0752000	8.16	26.1	15.1	27.09	33.45	33.74	13.3	10.8	8.29	11.21	17.54	24.46	5.4426	13.068	7.1277	35.82	6.183	15.54	7.432	20.16	17.24	16.27	28.51	17.33	19.01	13.2	15.35	22.2	17.2	15.8	2
Os03g0430000	439	460	428	569.8	642.7	646.2	420	441	319	363	422.1	395.1	411.46	404.18	411.82	620.9	455.5	459	582.5	652.3	557.9	380	362	327.2	449	366.8	326.7	476	459	445	10
Os03g0811400	12.2	10.2	12.9	87.02	30.73	140.1	83	25.3	67	13.79	14.25	20.7	4.3541	4.6673	10.296	35.82	19.58	9.324	83.61	65.31	87.95	310.5	134.7	580.7	7.395	33	54.82	13.9	25.8	10	9
Os03g0316900	635	468	542	422.8	386	474	462	491	493	386.3	429.8	432.8	537.73	634.75	559.13	871.7	846.1	612.3	533.2	436.2	438.9	414.1	544.5	545.3	512.3	479	493.4	617	453	518	13
Os03g0278300	1638	1538	1433	932.6	812.7	1011	568	645	628	809.6	698.4	702.8	875.17	847.57	841.07	1230	1250	1290	827.7	767.6	938.1	822.9	807.4	766.3	776.4	811.8	901.3	836	828	903	14
Os03g0775700	16.3	35.4	20.5	22.99	21.7	22.49	22.7	17.1	24.2	30.18	25.22	32.93	8.7082	6.5342	22.967	11.94	15.46	13.47	6.503	8.063	4.311	13.31	10.69	16.61	23.24	19.8	13.16	9.25	7.65	9.16	7
Os03g0563100	12.2	8.38	11.9	3.284	3.616	1.73	42.3	35.4	21.4	1.724	10.96	2.822	13.062	14.935	6.3357	11.94	16.49	4.144	9.29	7.257	15.52	8.873	4.276	29.61	9.507	13.2	15.35	10.2	15.3	13.3	5
Os03g0766500	8.16	14	5.39	132.2	207	125.4	8.61	3.8	6.91	11.21	14.25	6.586	11.974	5.6007	7.9197	35.82	11.34	8.288	84.53	105.6	68.12	8.133	3.563	1.444	7.395	9.428	2.193	16.7	13.4	10	10
Os03g0685200	28.5	36.3	40.9	39.41	39.77	59.69	3.13	10.8	2.76	28.45	23.02	23.52	31.567	26.137	10.296	83.59	30.92	38.33	35.3	54.83	47.42	8.133	12.11	7.945	12.68	26.4	15.35	27.8	33.5	24.2	16
Os03g0145700	759	632	697	811.9	865.1	806.2	462	563	473	438.9	362.9	467.6	377.72	454.59	389.65	1099	653.4	718	699.5	770	694.1	833.3	648.5	567.7	453.2	595.9	585.5	626	580	496	6
Os03g0284400	1231	1401	1347	2551	2719	2340	5838	4945	5563	2180	2536	3317	1668.7	1150.9	1147.6	1158	868.7	974.9	2903	2881	2911	2634	2495	3913	1360	1276	1303	575	659	781	18
Os03g0823000	119	101	117	150.2	126.6	133.2	233	180	240	194.9	186.4	117.6	62.046	74.676	81.572	167.2	80.38	99.46	135.6	139.5	87.08	283.2	246.6	230.4	150	132.9	217.1	133	125	101	18
Os03g0589400	0	0	0	0	0	0	0	0	0	0	2.193	1.882	1.0885	0.9335	0	0	1.031	0	0	0	0	0	0	0	0	0	0	0	2.87	0.83	20
Os03g0811200	16.3	14	14	18.06	10.85	19.03	8.61	10.1	23.5	6.897	12.06	3.763	16.328	8.4011	20.591	23.88	6.183	11.4	3.716	10.48	8.622	22.18	19.95	14.44	10.56	9.428	8.772	6.48	12.4	11.7	17
Os03g0238700	171	131	156	138.7	104.9	158.3	157	111	112	137.1	140.3	126.1	149.13	132.55	146.51	71.65	192.7	163.7	200.7	193.5	215.6	137.5	144.7	129.3	139.4	128.2	177.6	195	257	145	16
Os03g0733400	0	0	0	0.821	0	0	0	0	0	0	0	0	0	0	0	0	0	0	0.929	0.806	0	0	0	0	0	0	0	0	0.96	0	16
Os03g0766600	31.6	41	17.2	403.1	530.6	282.9	91.6	70.8	143	5.173	12.06	0	11.974	5.6007	3.1679	47.76	34.01	19.68	245.2	284.6	404.4	255.1	57.01	261.5	2.113	2.829	8.772	1.85	6.69	4.17	10
Os03g0758000	132	125	155	174	188	168.7	153	244	147	108.6	97.58	52.69	169.81	103.61	126.71	274.6	107.2	137.8	92.9	89.5	77.6	94.64	146.8	86.67	66.55	54.68	81.14	136	140	110	6
Os03g0666800	7.14	5.59	9.7	22.99	25.31	28.55	14.1	16.4	13.8	9.484	8.771	4.704	8.7082	3.7338	7.1277	35.82	7.214	7.252	29.73	14.51	25.87	15.53	7.126	19.5	6.338	5.657	0	5.55	10.5	5	10
Os03g0769000	187	160	154	85.38	96.72	84.77	45.4	66.4	60.8	62.94	59.21	92.2	69.665	94.279	99.788	346.3	182.4	146.1	120.8	113.7	160.4	127.9	94.07	104.7	114.1	132	146.9	129	111	142	13
Os03g0130100	16.3	10.2	10.8	10.67	10.85	8.65	0.78	0.63	0.69	2.587	2.193	2.822	1.0885	4.6673	1.5839	0	0	4.144	5.574	4.838	5.173	1.479	1.425	2.889	1.056	0.943	4.386	1.85	0.96	0	19
Os03g0106300	0	1.86	0	0.821	2.712	0	2.35	1.27	2.07	0	1.096	0.941	1.0885	0.9335	0	0	0	3.108	2.787	1.613	0	1.479	0.713	1.444	1.056	1.886	0	0	2.87	0.83	15
Os03g0282100	0	0.93	0	2.463	0.904	3.46	1.57	1.27	4.84	0.862	1.096	0.941	0	0	0.792	0	0	0	0	0	0.862	2.958	4.988	9.389	0	2.829	0	0	0	0	9
Os03g0448200	0	0.93	0	0.821	0	0	0	0	0	0	0	0	1.0885	0	0	0	0	0	0	0	0	2.958	0.713	0	0	0	6.579	0	1.91	0	11
Os03g0107800	4233	4939	5865	13581	13412	15310	9831	7609	8721	3973	4097	3175	6302.5	7750.4	6723.8	13374	5667	5712	14755	16309	16030	6511	6850	7402	4109	3619	3774	7547	8448	7510	10
Os03g0855600	707	690	749	828.4	1020	809.7	1350	1320	1464	953.6	877.1	1119	1052.6	924.12	859.28	1063	902.7	791.5	1067	1049	1122	920.5	981.3	1134	910.6	952.3	879.4	693	760	726	18

Os03g0665800	137	104	99.1	236.4	229.6	244.8	157	175	171	93.98	128.3	116.7	119.74	132.55	98.204	191.1	113.4	123.3	239.7	214.5	246.6	181.9	158.9	162.5	96.13	99	138.2	101	123	85	10
Os03g0742300	0	2.79	0	0	0	0	0	1.27	0	0.862	0	0	0	0	0.792	0	0	0	0	0.806	0	0	0	0	0	0	0	0	0	0	14
Os03g0182400	2991	3328	2989	1724	1692	1679	2129	2620	2300	2959	3332	2703	2974.9	3086	3666.8	3833	3305	3120	1590	1605	1692	2135	2533	2124	3196	3189	3068	3273	3473	3503	3
Os03g0725100	793	858	837	841.5	848.8	788.9	763	830	807	904.4	936.3	832.6	1035.2	911.05	998.67	1481	889.3	860.9	602.9	682.9	619.1	708.3	821	794.5	1101	945.7	844.3	1047	870	999	13
Os03g0710000	609	627	514	193.8	138.3	142.7	153	172	173	270.7	296	282.2	232.94	253.9	285.9	597	791.4	490	93.82	150.8	120.7	450.3	469.6	615.4	483.8	513.8	526.3	476	342	473	14
Os03g0202300	194	173	175	217.6	207	205	141	201	119	169.9	180.9	145.8	188.31	168.95	137.8	417.9	163.9	192.7	182.1	215.3	205.2	137.5	136.1	129.3	170.1	173.5	186.4	144	158	157	6
Os03g0229500	244	115	144	107.5	116.6	141.9	39.9	68.9	60.1	85.36	109.6	255.9	92.524	183.89	104.54	238.8	213.3	323.2	132.8	75.79	62.94	61.37	49.17	44.78	163.7	211.2	324.6	172	212	208	3
Os03g0136600	28.5	43.8	16.2	26.27	23.5	22.49	64.2	43.6	69.8	42.25	44.95	38.57	19.593	41.072	34.847	11.94	30.92	35.23	26.01	22.58	21.56	47.32	44.9	52.72	30.63	25.46	52.63	12	25.8	21.7	5
Os03g0861000	0	0	0	0.821	0.904	0	0	0	0	1.724	0	0.941	0	0	0	0	0	0	0	0	0.862	0.739	0	0	0	0	0	0	0	0	7
Os03g0145500	713	844	679	573	575.8	628.9	421	446	388	567.3	650.2	576.7	621.54	639.41	669.21	1039	844	674.5	502.6	560.4	545.8	530.9	493.9	462.2	623.3	703.4	712.7	751	668	738	13
Os03g0794000	0	2.79	1.08	0	0	0	0.78	0	0	0	0	0	0	0	0	0	0	0	0	0	0	0	0	0	0	0	0	0	0	0.83	14
Os03g0219900	1291	1319	1401	2362	2552	2076	4668	4283	4432	1519	1799	2505	1459.7	1132.3	1010.5	1600	931.6	1103	2723	2733	2828	2359	2186	3439	1071	992.8	811.4	625	860	751	18
Os03g0840500	211	246	259	299.7	363.4	297.6	97.1	167	100	108.6	148	110.1	189.4	124.15	171.06	226.9	169	187.5	163.5	162.1	148.3	240.3	238.7	170.4	122.5	127.3	92.1	160	130	180	2
Os03g0627300	192	249	223	232.3	193.4	219.7	245	223	276	212.1	284	245.6	251.45	215.63	211.45	477.6	240.1	241.4	229.5	212.1	219.9	248.4	268	238.3	231.3	281	190.8	227	247	242	6
Os03g0241100	846	981	849	794.7	838.9	692.9	842	876	850	813.9	866.2	677.4	815.3	860.64	987.58	1421	894.5	909.6	580.6	657.9	655.3	961.2	907.9	860.9	832.4	754.3	782.9	874	927	1000	13
Os03g0703200	898	1018	1091	1621	1671	1629	3370	3589	3168	1123	1017	1308	928.51	932.52	1052.5	907.5	827.5	886.8	1459	1376	1511	4049	3843	3750	1147	1102	1197	1057	785	900	18
Os03g0696300	2979	3146	2921	1134	1152	1026	1827	1467	1656	2592	2850	2948	2828	2704.2	2775.8	3320	3531	3129	1142	1180	1280	1824	1846	1671	2978	3802	3763	3181	2989	3443	3
Os03g0291800	134	117	76.5	55.83	41.58	42.39	87.7	79.7	124	68.11	111.8	50.8	80.55	82.144	68.109	167.2	94.81	84.96	39.94	27.41	37.08	104.3	87.65	121.3	78.17	85.8	105.3	102	104	106	13
Os03g0659300	195	191	228	195.4	144.6	144.5	171	156	167	355.2	330	309.5	272.13	343.51	294.61	322.4	253.5	217.6	264.8	269.3	207.8	105.7	102.6	139.4	292.6	287.6	307	277	298	193	3
Os03g0692300	0	0.93	0	0	0	0	0	0	0	0	0	0	0	0	0	0	0	0	0	0	0	0	0	0	0	0	0	0	0	0	14
Os03g0635800	71.4	46.6	34.5	46.8	51.53	33.74	54	27.2	16.6	25.87	23.02	34.81	17.416	37.338	12.671	107.5	56.68	45.59	51.09	43.54	56.04	28.84	19.95	31.06	39.09	37.71	21.93	33.3	39.2	33.3	6
Os03g0219200	3003	2930	3041	1548	1595	1970	3415	3106	3290	3863	3960	5307	4059.1	4410.6	3821.2	4597	4114	3981	2192	2340	2390	3686	2916	3422	4349	4386	4316	3466	3345	3568	3
Os03g0645250	222	184	189	198.7	221.5	164.4	124	176	143	163.8	148	181.6	176.34	151.22	163.14	226.9	178.3	187.5	118.9	103.2	104.3	215.2	180.3	134.3	164.8	149.9	125	163	157	190	8
Os03g0583900	814	1000	919	963	944.6	1066	1232	1360	990	1089	969.2	875	820.74	1031.5	887.79	1015	736.8	759.4	653.1	1088	607.9	1405	1547	955.5	959.2	885.3	866.2	1036	965	895	17
Os03g0126100	320	366	322	330	292	333	637	487	600	327.6	339.9	280.4	342.88	413.52	458.55	573.2	337	373	288	283.8	312.1	500.6	444	427.6	367.6	380.9	368.4	403	360	403	18
Os03g0121800	1424	1403	1304	830.8	781.9	827.9	651	674	701	1067	1193	1215	1159.3	1183.6	1413.7	2508	1532	1492	818.4	874.8	820.8	1102	900.8	928.8	1301	1377	1246	1521	1443	1461	13
Os03g0317000	1115	983	1042	716.7	685.2	804.5	738	829	809	770.8	747.7	838.3	1145.1	1013.7	1050.9	1779	1408	1122	628	761.1	662.2	1189	1088	1114	1086	996.6	850.9	1150	1167	1212	13
Os03g0272900	328	370	343	168.3	169	95.16	802	644	679	448.3	591	417.7	262.33	223.09	277.98	370.2	286.5	310.8	126.3	185.4	143.1	216.6	292.9	307.7	414.1	370.5	348.7	182	243	273	5
Os03g0278800	128	77.3	108	189.6	185.3	172.1	101	109	67	87.08	91	89.38	160.01	121.35	107.71	119.4	102	90.14	218.3	187.1	192.3	66.54	80.53	87.39	79.23	69.77	89.91	75.9	88.9	79.1	10
Os03g0854400	56.1	27	36.6	82.92	90.4	61.42	70.5	44.9	73.2	50.01	40.57	54.57	22.859	34.538	37.222	35.82	45.34	41.44	75.25	49.18	51.73	47.32	64.14	65	34.86	51.86	78.95	41.6	40.2	22.5	12
Os03g0216500	0	0.93	0	0	0	0	0	0	0	0	0	0	0	0	0	0	0	0	0	0	0	0	0	0	0	0	0	0	0	0	14
Os03g0401366	0	0	0	0	0	0	0	0	0	0	0	0	0	0	0	0	0	0	0	0.806	0	0	0	0	0	0	0	0	0	0	16
Os03g0701200	90.7	144	113	110.8	81.36	182.5	13.3	32.9	28.3	61.22	81.13	53.63	53.337	55.074	62.565	143.3	93.78	93.24	132.8	99.17	145.7	17.01	15.68	15.17	76.06	51.86	131.6	71.2	118	93.3	16
Os03g0267400	0	0	0	0	0	0	0	0	1.38	0	0	0	0	0	0	0	0	0	0	0	0	0	0	0	0	0	0	0	0	0	18
Os03g0785200	442	625	584	1227	1249	1103	358	398	350	1003	1061	977.5	880.61	736.49	867.99	728.4	587.4	773.9	1253	1288	1498	259.5	235.9	268	939.1	749.6	596.5	551	595	713	16
Os03g0575700	271	289	351	50.08	27.12	44.98	135	195	119	410.4	348.7	427.1	460.44	406.05	437.96	573.2	383.4	311.8	86.39	41.12	68.98	108.7	145.4	168.3	458.5	337.5	348.7	324	359	349	3
Os03g0847800	79.5	71.7	83	43.51	75.93	61.42	11	39.8	23.5	47.42	53.72	25.4	60.957	41.072	85.532	131.4	69.05	71.49	23.22	24.99	18.11	56.93	65.56	39	75	60.34	67.98	85.1	66.9	102	13
Os03g0308000	824	949	856	995	941.9	981	1111	1211	1173	1003	958.2	853.3	810.95	780.37	941.65	1027	757.4	822.6	952.2	842.6	888.1	1106	1099	1249	903.2	863.6	1088	879	836	911	18
Os03g0690700	3.06	1.86	1.08	1.642	1.808	0.865	3.13	2.53	0.69	0.862	1.096	0.941	4.3541	0.9335	1.5839	0	1.031	5.18	1.858	4.031	1.724	3.697	7.839	1.444	2.113	0.943	0	4.63	0.96	2.5	17
Os03g0609200	0	0	0	0	0	0	0	0	0	0	0	0	0	0	0	0	0	1.036	0	0	0	0	0	0	0	0	0	0	0	0.83	1
Os03g0603200	0	3.73	6.47	5.747	5.424	11.25	0	0	0.69	1.724	4.386	4.704	1.0885	1.8669	2.3759	11.94	4.122	2.072	0.929	0	1.724	1.479	3.563	0	1.056	11.31	10.96	2.78	4.78	0.83	11
Os03g0244700	1.02	0	0	0.821	0	3.46	14.9	12.7	26.9	0	4.386	0.941	2.177	3.7338	1.5839	0	2.061	1.036	4.645	3.225	2.587	19.22	11.4	15.17	1.056	0	0	1.85	1.91	3.33	18
Os03g0124800	0	2.79	1.08	2.463	0	1.73	8.61	1.9	6.91	5.173	2.193	0	0	0.9335	1.5839	0	0	3.108	1.858	1.613	0	9.612	2.851	19.5	0	0	0	1.85	2.87	0	9
Os03g0735400	226	209	238	207.7	173.6	202.4	205	204	175	270.7	268.6	329.3	239.47	354.71	254.22	238.8	314.3	353.3	233.2	261.2	329.4	242.5	204.5	174.8	296.8	380.9	322.4	282	358	262	3
Os03g0295700	1062	947	1083	568.1	607.5	578.7	577	765	681	890.6	880.4	802.5	1159.3	997.86	1113.5	1397	1069	1125	451.5	539.4	525.1	777.1	741.1	567	1051	959.8	789.5	1103	1047	1085	13
Os03g0239400	557	573	591	576.3	585.8	596	535	581	573	501.8	482.4	390.4	510.52	520.87	602.69	1385	629.7	545	500.7	536.2	464.7	649.2	622.1	591.5	548.3	539.3	521.9	577	629	614	6
Os03g0353333	0	0	0	0	0	0	0	0	2.07	0	0	0.941	0	0	0	0	0	0	0	0.806	0.862	0	0	0	0	0	0	0	0	0	15
Os03g0268200	0	0.93	0	0	0.904	4.325	5.48	2.53	3.46	3.449	0	0	0	1.8669	0	0	0	2.072	2.787	4.838	3.449	1.479	1.425	1.444	4.225	0	0	0.93	1.91	0	15
Os03g0670200	10.2	6.52	4.31	6.568	8.136	12.98	7.83	15.2	12.4	16.38	13.16	20.7	7.6196	13.068	19.007	11.94	14.43	15.54	16.72	17.74	26.73	8.873	12.83	11.56	15.85	8.486	4.386	8.33	5.74	5.83	16
Os03g0723000	25.5	16.8	30.2	14.78	11.75	29.41	14.1	27.2	15.2	23.28	9.868	29.17	19.593	26.137	10.296	23.88	19.58	20.72	14.86	12.09	14.66	22.18	20.67	16.61	19.01	6.6	24.12	17.6	14.3	17.5	19
Os03g0750100	1217	931	1228	385	371.5	354.7	405	390	373	1461	1505	1486	2327.3	2006.9	2167.6	1421	978	1034	411.5	363.6	428.5	286.1	269.4	283.1	1439	1229	1303	1348	1185	1288	3
Os03g0825600	137	143	112	45.15	22.6	34.6	12.5	15.8	6.91	138.8	143.6	149.6	71.842	151.22	134.63	226.9	170	192.7	17.65	41.12	47.42	62.11	83.38	65	333.8	365.8	315.8	356	176	232	3
Os03g0280000	1865	2067	2111	1623	1667	1526	1334	1747	1674	1382	1406	1174	1657.8	1417.9	1793	2591	1770	1899	1116	1371	1285	2258	2240	1836	1533	1491	1333	1863	1872	1890	8
Os03g0799801	0	0.93	0	0	0	0	0.78	0	0	0.862	0	0	1.0885	0.9335	0	0	0	3.108	0	2.419	0.862	0	0	0	0	0	0	0	0.96	1.67	1
Os03g0269900	1703	1350	1692	660.1	593	667.8	1599	1338	1687	2013	1476	1611	1380.2	1349.8	1515.8	3021	1983	1674	1052	1213	1191	1429	1573	1333	1975	1939	2013	1918	1919	1562	13
Os03g0851600	437	410	457	287.3	258.5	250	312	294	285	470.8	447.3	477	445.2	462.06	462.51	597	451.4	439.3	239.7	315.3	259.5	327.5	312.8	299	512.3	567.6	519.7	466	418	462	3
Os03g0704400	6.12	14.9	7.54	1.642	5.424	3.46	2.35	4.43	7.6	3.449	3.289	3.763	4.3541	3.7338	3.1679	11.94	12.37	8.288	12.08	3.225	2.587	4.436	9.977	3.611	5.282	6.6	6.579	5.55	8.61	3.33	14
Os03g0785900	15.3	7.45	11.9	9.852	9.944	7.785	7.05	14.5	5.53	2.587	10.96	6.586	1.0885	7.4676	3.1679	35.82	12.37	7.252	13.01	2.419	3.449	8.873	13.54	18.78	3.169	4.714	13.16	1.85	10.5	3.33	6
Os03g0854250	0	0	0	0.821	0	0	0	0	0	0	0	0	6.5311	0	0	0	0	0	0	0	0	0	0	0	0	0	0	0	1.91	0	4
Os03g0360800	28.5	14	22.6	3.284	3.616	6.055	6.26	5.69	3.46	18.97	13.16	11.29	8.7082	6.5342	11.879	47.76	22.67	32.12	4.645	3.225	4.311	10.35	14.97	13	20.07	27.34	15.35	13	10.5	19.2	13
Os03g0619400	515	447	411	510.6	572.2	564	465	352	375	427.6	394.7	461.9	426.7	448.06	417.37	620.9	407.1	403	486.8	463.6	593.2	454	439.7	353.9	404.6	416.7	506.6	402	443	340	10
Os03g0105800	6.12	3.73	3.23	3.284	2.712	5.19	3.13	1.27	6.22	1.724	3.289	0	2.177	17.736	7.1277	0	9.275	6.216	3.716	5.644	10.35	4.436	13.54	13	7.395	2.829	8.772	12	5.74	8.33	1
Os03g0678400	419	482	414	275.8	250.4	320.9	204	283	321	332.8	333.3	271.9	275.4	204.43	246.3	310.5	256.6	214.5	236	211.2	217.3	282.4	253.7	299.7	324.3	316.8	350.9	227	194	194	14
Os03g0831100	332	315	291	566.5	620.1	571.8	320	312	301	281.9	258.7	365	234.03	340.71	255.81	632.9	308.1	333.6	557.4	469.3	528.5	298	270.8	255.7	185.9	280	280.7	279	316	225	10
Os03g0352200	1793	1683	1869	1501	1475	1511	665	794	764	1711	1886	1707	2420.9	2156.3	2139.9	3749	2307	2235	1758	1742	1759	1040	845.2	754.7	2208	1985	2046	2606	2266	2671	13
Os03g0633100	95.8	129	114	120.7	75.93	76.12	67.3	82.9	97.4	90.53	98.68	63.98	131.71	101.75	121.96	155.2	157.7	155.4	49.23	54.83	54.32	102.8	126.8	91.73	117.3	119.7	78.95	191	124	166	8
Os03g0128300	3.06	0.93	0	0.821	0.904	3.46	2.35	3.16	0	0	0	2.822	0	0	0	0	0	0	0.929	7.257	2.587	1.479	2.851	0.722	0	0	4.386	0	0.96	1.67	15
Os03g0435200	40.8	30.7	45.3	72.25	75.03	76.99	39.9	50.6	73.2	45.7	47.14	40.46	51.16	46.673	50.686	47.76	45.34	35.23	66.88	99.17	39.66	57.67	51.31	59.95	42.25	34.89	32.89	40.7	44	45	10
Os03g0747200	1657	1994	1940	1607	1694	1426	2227	2287	1925	2040	1990	1865	1717.7	1774.5	1925.3	2340	1667	1680	1576	1562	1563	1536	1677	1769	1952	1839	1982	1604	1579	1654	5
Os03g0308800	6004	5016	5820	2151	2225	2563	1166	1529	1233	5921	6073	6728	8274.9	8287.2	7785.8	12825	8471	7214	3140	3161	3428	1202	1179	1358	7467	7818	6421	7741	6993	7376	13
Os03g0821300	1136	845	755	120.7	75.93	102.9	191	209	233	422.5	354.1	449.7	290.63	308.04	324.71	394.1	786.3	651.7	220.2	188.7	196.3	199.6	234.5	289.6	334.9	371.5	478.1	287	266	292	14
Os03g0742400	920	924	929	759.4	755.7	801	1304	1030	1195	1167	1044	1267	950.28	1028.7	911.55	1087	982.1	839.2	1015	917.5	967.4	1009	994.1	1217	1010	1037	1178	871	857	831	5
Os03g0595600	0	0	0	0	0	0	0	0	0	0	0	0	0	0	0	0	0	1.036	0	0	0	0	0	0	0	0	0	0	0	0	1
Os03g0336700	372	355	362	259.4	212.4	317.5	146	199	209	346.6	345.4	347.2	387.51	365.91	440.33	609	329.8	349.1	258.2	312.8	294.9	259.5	282.9	273.7	477.5	396	405.7	388	399	447	13
Os03g0195200	303	328	309	331.7	380.6	411.8	451	357	575	267.3	315.8	271	352.68	363.11	342.13	489.6	343.2	349.1	323.3	419.3	344.9	428.8	399.1	357.5	321.1	330	307	356	362	319	18
Os03g0683900	34.7	24.2	35.6	16.42	29.83	10.38	3.13	8.86	2.76	7.76	9.868	7.527	14.151	9.3345	11.088	47.76	19.58	20.72	9.29	6.45	15.52	11.83	19.24	6.5	17.96	12.26	6.579	25	30.6	34.2	8
Os03g0266700	334	354	374	384.2	377.9	339.1	492	633	550	317.3	336.6	268.1	291.72	282.84	314.41	752.3	292.7	329.5	365.1	341.1	321.6	539.8	525.9	541.7	346.5	296.1	276.3	363	278	302	6
Os03g0290500	828	925	794	692.9	599.3	668.7	762	813	683	782	810.2	735.7	842.51	753.3	955.11	1922	884.2	922.1	561.1	658.7	637.2	964.2	1032	727.3	996.2	912.7	947.3	945	886	949	13
Os03g0605300	509	464	345	322.6	245	305.4	197	294	265	493.2	442.9	494.9	312.41	401.38	442.71	692.6	554.4	400.9	263.8	257.2	205.2	388.2	466.8	410.2	649.7	681.7	635.9	658	570	533	13
Os03g0769100	1318	1376	1476	2082	2357	2091	3339	3348	3450	1529	1887	2199	1758	1306.8	1112.7	1493	1158	1294	2735	3011	3115	1539	1473	2339	1208	1102	1061	848	1033	915	12
Os03g0247900	247	215	175	119	83.17	128	146	140	149	81.05	47.14	63.98	67.488	56.007	68.109	250.8	175.2	108.8	134.7	146.7	113.8	164.1	200.3	173.3	52.82	53.74	107.5	89.7	103	72.5	14
Os03g0795100	1362	1556	1628	495	548.7	468.9	1226	1438	1244	2080	2070	1901	1958.2	1731.6	1839.7	2305	1462	1494	620.5	668.4	594.9	1029	1127	1181	1933	1687	1621	1481	1730	1799	3
Os03g0156600	3.06	5.59	3.23	0	0	0	0	0	0	0	0	0	0	0	0.792	0	1.031	1.036	0	0	0	0	0	0.722	1.056	0.943	0	0.93	0	0	14
Os03g0708800	65.2	30.7	35.6	21.35	20.79	20.76	29	32.3	33.2	50.01	39.47	56.45	71.842	55.074	45.934	71.65	50.5	54.91	26.01	24.19	31.04	19.96	24.23	18.78	50.71	28.29	37.28	40.7	38.3	45.8	13
Os03g0373500	31.6	16.8	11.9	4.926	12.66	13.84	6.26	5.69	5.53	7.76	1.096	8.467	5.4426	5.6007	10.296	0	29.89	19.68	9.29	5.644	7.76	14.79	28.51	17.33	3.169	2.829	10.96	9.25	9.56	7.5	19
Os03g0147150	3.06	4.66	5.39	11.49	11.75	6.92	7.05	8.22	14.5	11.21	7.675	8.467	2.177	6.5342	5.5438	0	9.275	9.324	4.645	8.869	9.484	14.05	14.25	7.222	7.395	13.2	15.35	8.33	18.2	20	1
Os03g0796400	36.7	54	50.6	27.91	27.12	38.06	51.7	74	52.5	50.01	62.49	36.69	41.364	68.142	47.518	35.82	54.62	66.31	16.72	30.64	38.8	70.24	66.28	54.17	64.44	99	43.86	62.9	60.2	69.1	1
Os03g0609500	19.4	22.4	26.9	4.105	2.712	8.65	11	6.33	8.98	43.97	48.24	65.86	11.974	6.5342	25.343	11.94	24.73	24.86	4.645	8.063	7.76	8.873	12.11	7.945	23.24	54.68	35.09	12	9.56	16.7	20
Os03g0311000	24.5	96.9	15.1	4.926	2.712	3.46	4.7	10.8	9.67	11.21	15.35	23.52	11.974	10.268	9.5036	11.94	21.64	10.36	13.01	14.51	12.07	0.739	4.988	10.11	5.282	18.86	21.93	8.33	7.65	9.16	14
Os03g0381200	464	472	492	519.7	520.7	525.1	554	514	509	514.7	588.8	612.5	619.37	606.74	568.63	1087	594.6	554.3	538.8	514.4	573.4	490.2	467.5	502.7	596.8	592.1	585.5	656	581	599	6
Os03g0322500	2373	2222	2207	2366	2609	2079	4448	4563	4688	2816	2784	3498	2017	2287	2202.5	2496	2114	1943	2197	2202	2029	3351	3528	3984	2824	2683	2860	2073	1721	1835	18
Os03g0252800	571	515	514	769.3	755.7	795.8	931	1075	1008	612.2	654.5	593.7	620.46	572.21	630.4	764.2	562.7	532.5	662.3	616.8	688	936.8	948.5	886.2	612.7	625.1	598.7	533	514	546	18
Os03g0266200	294	296	302	329.2	299.2	310.6	351	388	415	346.6	333.3	288.8	346.15	378.05	376.18	537.3	244.2	265.2	316.8	270.1	249.2	334.9	384.8	353.2	324.3	347	302.6	299	282	253	6
Os03g0151100	112	109	140	105.9	114.8	80.45	94.7	115	71.9	68.97	71.27	66.8	127.36	98.946	132.26	226.9	105.1	163.7	79.89	94.33	91.4	119.8	101.9	83.06	100.4	92.4	67.98	109	105	127	13
Os03g0228400	411	448	496	298.8	276.6	286.3	288	257	276	582	581.1	581.4	514.87	480.73	526.66	716.5	503.9	483.8	331.6	330.6	357	194.5	198.1	225.3	566.2	611	565.8	432	529	508	3
Os03g0712700	1801	2164	2030	1007	1125	968	851	1183	1105	1504	1454	1457	1330.2	1610.2	1566.5	1767	1639	1850	658.6	450.7	631.1	1100	968.5	954.8	1488	1534	1197	1434	1457	1849	3
Os03g0680600	0	0	0	0	0	1.73	1.57	0	0	1.724	1.096	0	0	0	0	0	0	0	0	1.425	0	0	0	0	0	0	0	0	0	0	7
Os03g0251700	2.04	9.31	11.9	73.89	82.26	84.77	150	122	142	2.587	6.578	4.704	18.505	11.201	8.7116	23.88	13.4	12.43	40.87	53.21	57.77	99.82	106.9	109.8	16.9	16.03	13.16	7.4	17.2	12.5	18
Os03g0281100	6.12	7.45	2.16	9.031	4.52	5.19	22.7	10.8	13.8	4.311	2.193	2.822	8.7082	1.8669	1.5839	0	4.122	1.036	14.86	4.838	4.311	4.436	11.4	13.72	5.282	6.6	0	0	5.74	2.5	18
Os03g0812000	240	259	263	1639	1933	1578	880	854	570	208.6	187.5	140.2	264.51	277.24	146.51	417.9	184.5	263.2	1221	1467	1411	479.9	547.3	514.2	126.8	109.4	83.33	158	214	197	10
Os03g0174200	0	2.79	1.08	0	0	0	0	0	0	0	0	0	0	0	0	0	0	0	0	0	1.724	0	0	1.444	0	1.886	2.193	0.93	0	0	11
Os03g0850900	0	0	0	13.14	1.808	51.9	0.78	0	4.15	3.449	0	0.941	0	0	0	0	0	0	10.22	0	31.9	3.697	84.8	41.89	3.169	2.829	4.386	0	4.78	3.33	17
Os03g0423200	524	471	435	458.1	507.1	537.2	669	618	609	561.3	557	723.5	640.05	637.55	544.87	632.9	545.2	543.9	642.8	600.7	687.2	542.7	574.4	617.5	553.5	615.7	684.2	480	537	485	15
Os03g0773700	86.7	57.7	78.7	82.1	78.65	84.77	39.9	27.2	35.9	35.35	39.47	63.98	29.39	36.405	37.222	59.7	63.89	39.37	68.74	64.5	120.7	25.14	12.11	25.28	20.07	59.4	52.63	19.4	31.6	25.8	16
Os03g0609800	107	210	159	393.2	350.7	357.3	575	564	516	170.7	190.8	126.1	145.86	122.28	186.9	167.2	127.8	104.6	250.8	194.3	186.2	890.2	711.2	621.9	131	132.9	155.7	138	158	137	17
Os03g0131500	1224	1277	1412	1351	1448	1442	1438	1386	1321	1711	1730	1836	1980	1784.8	1672.6	2066	1409	1487	1708	1865	1753	1144	1033	1325	1691	1665	1419	1357	1397	1494	16
Os03g0703600	0	0	0	0	0	0.865	0	0	0	0	0	0	0	0	0.792	0	0	0	0	0	0	0	0	0	0	0	0	0	0	0	4
Os03g0795200	192	232	223	448.3	478.2	353.8	629	613	544	241.4	336.6	282.2	262.33	200.69	225.71	179.1	161.8	182.3	391.1	347.5	355.2	434	384.1	450	220.8	198	133.8	130	159	222	12
Os03g0125650	0	0	1.08	0	0	0	0	0	0	0	1.096	0	0	0	0	0	1.031	0	0	0	0	0.739	0.713	0	0	0	0	0	0	0	17
Os03g0168600	0	0	0	1.642	0.904	4.325	0.78	1.9	3.46	2.587	0	0.941	0	2.8004	0	0	1.031	0	0	0	0.862	3.697	5.701	3.611	3.169	0.943	0	0	2.87	0	17
Os03g0608000	0	0.93	1.08	0	0	0	0	0	0	0.862	0	0	0	0	0	0	0	0	0	0	0	0	0	0	0	0	0	0	0	0	14
Os03g0852300	56.1	44.7	53.9	147	158.2	173	93.9	69.6	77.4	64.66	74.55	43.28	77.285	81.21	80.781	83.59	46.37	77.7	141.2	126.6	108.6	80.59	82.67	83.06	63.38	58.46	48.24	77.7	62.2	51.7	10
Os03g0574600	44.9	30.7	32.3	2.463	10.85	18.17	25.1	24.7	19.3	31.04	39.47	70.56	22.859	55.074	30.887	59.7	86.56	56.98	37.16	29.83	48.28	8.873	3.563	21.67	49.65	140.5	122.8	51.8	48.8	36.7	11
Os03g0817900	158	174	196	226.6	217	246.5	524	532	516	135.4	116.2	157.1	133.89	163.35	129.88	417.9	323.6	215.5	248	272.5	302.6	417	352.8	510.6	155.3	216.9	274.1	212	275	157	18
Os03g0100500	251	292	239	341.5	386.9	358.1	338	381	316	204.3	164.5	252.1	222.06	215.63	189.28	417.9	233.9	230	389.2	318.5	362.1	290.6	374.8	338	217.6	188.6	201.7	213	197	193	12
Os03g0728100	749	703	698	725.7	741.3	654.8	409	359	391	680.3	741.2	758.3	1029.7	881.18	940.86	1278	930.6	731.4	714.4	727.3	727.7	349.7	352.8	380.6	817.6	746.7	677.6	765	722	808	13
Os03g0102000	99.9	139	145	178.2	163.6	160	234	202	228	158.6	126.1	167.5	111.03	127.88	108.5	203	111.3	105.7	187.6	154	136.2	210	191.7	200.8	102.5	108.4	149.1	97.1	110	105	18

Os03g0398600	30.6	28.9	22.6	126.4	135.6	105.5	68.1	72.1	74.6	27.59	35.08	13.17	22.859	32.671	22.967	11.94	30.92	32.12	125.4	79.82	93.12	66.54	88.37	67.17	35.92	29.23	30.7	43.5	39.2	40.8	10
Os03g0426800	1897	1639	1875	452.4	432.1	442	1262	1169	1113	2073	2162	2533	2213	1983.6	2039.3	4681	2156	1960	695.8	590.2	582.9	1037	885.1	1127	2250	2293	2344	1843	2275	2274	13
Os03g0694600	5.1	2.79	11.9	10.67	11.75	12.11	8.61	6.96	8.98	12.93	3.289	15.99	15.239	11.201	11.879	11.94	5.153	9.324	11.15	8.063	10.35	14.05	12.11	10.11	7.395	7.543	2.193	13	5.74	14.2	4
Os03g0598700	10.2	20.5	14	27.09	22.6	17.3	13.3	9.49	10.4	18.11	14.25	15.05	10.885	12.135	11.088	35.82	25.76	12.43	20.44	18.54	14.66	11.83	18.53	16.61	15.85	18.86	17.54	25.9	12.4	18.3	6
Os03g0579200	1.02	2.79	0	6.568	0.904	2.595	3.91	1.9	0.69	4.311	1.096	0.941	2.177	0.9335	1.5839	0	0	7.252	1.858	0	1.724	5.915	3.563	5.778	3.169	3.771	8.772	6.48	4.78	3.33	11
Os03g0686700	89.7	110	119	110	108.5	99.48	123	107	108	110.4	132.7	69.62	126.27	69.075	105.33	191.1	71.11	92.21	68.74	74.18	58.63	153.1	116.9	147.3	161.6	124.5	67.98	98.1	96.6	131	6
Os03g0294700	89.7	79.2	92.7	110	122	99.48	75.2	67.7	63.6	63.8	71.27	47.04	48.983	46.673	49.102	143.3	76.26	82.88	107.8	146.7	94.84	105.7	114.7	132.9	60.21	60.34	70.17	104	61.2	75.8	10
Os03g0694500	0	4.66	2.16	6.568	2.712	2.595	0	2.53	2.07	4.311	2.193	2.822	1.0885	5.6007	0	0	1.031	1.036	3.716	0.806	1.724	1.479	4.276	0.722	1.056	0	0	4.63	2.87	1.67	2
Os03g0106100	1.02	0.93	1.08	2.463	5.424	4.325	0	0	0	0	0	0	0	0	0	0	0	0	0	0.806	0	2.958	3.563	0	0	0	2.193	1.85	0	2.5	2
Os03g0148300	0	0	1.08	0	0	0	0	0	0	4.311	6.578	0	1.0885	0	0	0	0	0	0	0	0	0	0	0.722	1.056	0	2.193	0.93	0.96	0	7
Os03g0183950	2.04	0	0	0	0	0	0	0	0	0	0	0	0	0	0	0	0	0	0	0	0	0	1.425	0	0	0	0	0	0	0	19
Os03g0116000	137	120	122	325.1	305.5	334.8	141	163	190	136.2	92.1	99.73	148.04	185.76	124.34	238.8	116.5	121.2	350.2	325.7	344.9	203.3	170.3	182	104.6	107.5	107.5	146	147	125	10
Os03g0854100	578	587	575	489.3	454.7	535.5	568	562	704	489.7	392.5	432.8	380.98	439.66	480.72	788.1	530.7	462.1	470.1	461.2	427.7	596.7	635	715	406.7	462	585.5	472	506	446	6
Os03g0729300	2016	1705	2103	1044	1012	1165	1410	1619	1740	2598	2545	2790	3175.2	2832.1	2967.5	3057	2492	2229	1844	1716	1735	1220	1357	1356	2898	2984	2732	2508	2469	2510	3
Os03g0834466	0	0	0	0	0	2.595	10.2	1.9	0.69	5.173	0	0	0	0	2.3759	11.94	1.031	0	5.574	0	0	2.218	0.713	7.222	3.169	0.943	2.193	0.93	2.87	2.5	6
Os03g0225700	125	153	145	134.6	135.6	125.4	277	295	314	195.7	130.5	157.1	156.75	110.15	175.82	203	102	107.7	118.9	114.5	89.67	183.4	212.4	203	131	102.8	89.91	99.9	97.5	95.8	18
Os03g0197800	27.5	29.8	24.8	87.02	74.13	65.74	35.2	34.8	41.5	32.76	23.02	8.467	34.833	19.602	38.014	47.76	36.07	24.86	79.89	62.08	86.22	63.59	52.73	44.78	27.47	35.83	15.35	40.7	65	42.5	10
Os03g0842700	797	912	795	807.8	824.4	793.2	937	890	883	894.9	945.1	943.6	829.45	785.03	843.44	1708	973.9	994.6	811	965.9	871.7	907.2	818.8	774.2	901.1	970.2	962.7	959	953	952	6
Os03g0790700	54	86.6	91.6	58.29	93.11	78.72	67.3	109	111	43.11	64.69	31.05	83.816	65.342	53.062	191.1	68.02	75.63	68.74	79.02	52.6	163.4	120.4	161.1	59.16	48.08	41.67	116	70.8	122	6
Os03g0397500	2.04	0.93	3.23	0	5.424	4.325	3.91	0	0	6.035	2.193	4.704	0	2.8004	1.5839	0	2.061	1.036	3.716	3.225	4.311	1.479	1.425	0.722	1.056	1.886	2.193	1.85	0.96	0.83	16
Os03g0784900	186	168	184	155.2	154.6	191.2	168	175	167	194	149.1	207	148.04	179.22	160.77	310.5	158.7	156.4	144.9	125.8	156.1	150.8	172.5	158.9	131	151.8	214.9	141	167	170	6
Os03g0333700	648	618	592	442.5	481.8	432.5	1028	924	830	801.8	639.2	1179	601.95	644.08	605.85	991.1	668.8	770.8	565.7	566	599.2	874.7	714.1	606.7	838.8	846.7	1004	779	695	682	5
Os03g0826900	1.02	1.86	0	1.642	0	0	3.91	18.3	1.38	0	0	0	0	0.9335	0	0	0	0	1.858	0	0.862	0.739	0.713	0	0	0	0	4.63	0	0	18
Os03g0748200	564	670	645	611.6	593	579.6	659	648	674	672.5	715.9	669.9	667.26	635.68	693.76	1349	725.5	680.7	603.8	641	612.2	726.1	734.7	693.4	643.3	727.9	758.7	704	699	761	6
Os03g0319000	15.3	34.5	34.5	114.1	132.9	30.28	75.9	99.3	67	66.39	46.05	36.69	34.833	28.937	69.693	0	42.25	32.12	79.89	99.17	88.81	34.01	71.26	41.17	30.63	35.83	30.7	36.1	21	35	12
Os03g0297000	753	671	692	380.1	374.2	288.9	421	441	529	653.5	505.4	616.2	472.42	427.52	536.16	740.3	557.5	552.2	362.3	329.8	311.3	525	644.2	483.2	458.5	404.5	445.2	457	466	492	14
Os03g0279801	4.08	6.52	1.08	3.284	3.616	6.92	0.78	0.63	4.84	0	2.193	0	1.0885	0.9335	0	11.94	2.061	2.072	0.929	6.45	3.449	3.697	5.701	1.444	7.395	0	0	2.78	1.91	2.5	6
Os03g0639300	766	663	647	396.5	350.7	394.5	139	132	123	920	969.2	947.4	938.3	1163.1	1004.2	1051	927.5	811.2	460.8	454.7	460.4	113.1	110.5	98.95	1292	1395	1276	1332	1336	1317	3
Os03g0145900	38.7	14.9	20.5	316.1	289.3	408.3	47.8	18.3	49.8	11.21	7.675	18.82	21.77	12.135	7.9197	59.7	50.5	46.62	438.5	399.9	494.1	59.89	40.62	63.56	28.52	26.4	24.12	19.4	17.2	27.5	10
Os03g0740833	177	108	115	189.6	100.3	210.2	49.3	123	79.5	92.25	58.11	41.4	80.55	85.878	163.94	95.53	118.5	153.3	105.9	150	136.2	257.3	233.7	91.73	76.06	92.4	122.8	148	125	149	17
Os03g0736400	1304	1539	1477	3795	4624	3761	3204	3220	2745	1386	1694	1823	2275	1792.2	1211.7	1946	1163	1439	4009	4481	4430	1724	1796	2089	1161	976.8	857.4	1010	1146	1126	10
Os03g0248300	0	0	0	7.389	14.46	6.055	12.5	8.22	11.7	0.862	1.096	0	1.0885	0	0	0	0	2.072	6.503	4.031	12.07	1.479	7.126	4.333	1.056	0.943	0	0.93	0	0	12
Os03g0391700	452	414	412	729	634.6	769	606	767	761	319.9	271.9	270	203.55	183.89	249.47	250.8	243.2	294.2	507.2	624.1	538	957.5	949.9	807.5	245.1	306.4	258.8	206	262	217	12
Os03g0699300	627	581	682	1204	1385	985.3	1769	1741	1627	455.2	475.8	845.8	726.04	632.88	529.83	967.2	566.8	552.2	1312	1203	1542	918.3	855.9	1347	499.7	422.4	475.9	303	487	397	12
Os03g0851900	880	858	884	754.5	777.4	772.5	903	1008	811	738	793.8	893.8	962.25	1031.5	1016.1	1636	1176	935.5	760.8	924	708.7	831.8	960.6	923.8	1053	999.4	991.2	1155	1057	1089	13
Os03g0396900	161	161	159	174.9	188.9	201.6	123	121	115	151.7	148	111	156.75	146.55	134.63	286.6	151.5	124.3	156.1	188.7	151.8	150.1	160.3	121.3	81.34	127.3	120.6	118	116	136	6
Os03g0845700	436	414	387	462.2	489	485.3	732	659	624	553.5	510.9	628.5	522.49	440.59	460.13	656.8	407.1	442.4	546.2	499.1	572.5	602.6	502.4	558.3	484.9	514.8	510.9	426	429	443	18
Os03g0715500	606	645	570	659.2	584	659.2	429	482	434	554.4	502.1	510.9	647.67	554.47	620.9	955.3	607	615.4	465.4	462.8	439.7	544.9	548	528.7	644.4	568.5	445.2	713	637	700	13
Os03g0746700	55.1	73.6	62.5	199.5	215.1	157.4	240	324	300	87.94	108.5	75.27	82.728	95.212	103.75	95.53	72.14	64.23	103.1	127.4	140.5	238.8	296.5	205.1	80.28	100.9	87.72	127	91.8	117	18
Os03g0277700	1.02	0.93	0	18.06	0	6.055	0.78	1.9	5.53	0.862	0	0	0	0	0	0	0	0	0	0	0	3.697	66.28	5.778	1.056	0	2.193	0	0.96	0	17
Os03g0247400	0	0	0	0	0	0	0	0	0	0	0	0	0	0	0	0	0	1.036	0	0	0	0	0	0	0	0	0	0	0	0	1
Os03g0104800	118	96.9	129	136.3	170.9	187.7	113	129	135	104.3	130.5	131.7	215.53	203.49	175.02	214.9	176.2	179.2	126.3	154.8	160.4	90.94	87.65	49.11	125.7	110.3	89.91	118	118	155	4
Os03g0422800	48.9	64.3	76.5	95.23	127.5	75.26	110	154	131	44.83	33.99	84.68	28.302	24.27	42.766	119.4	28.85	53.87	78.03	109.7	61.22	156	244.4	105.4	26.41	25.46	48.24	20.4	44.9	32.5	17
Os03g0212700	1557	1556	1546	1073	1078	1164	1087	1035	1162	1313	1204	1408	1190.8	1361	1197.5	1827	1404	1402	1163	936.1	1090	1059	1051	1176	1349	1381	1410	1215	1368	1181	13
Os03g0243600	470	570	469	568.1	501.7	477.5	1229	1429	1110	514.7	497.8	534.4	389.69	426.59	502.11	823.9	459.6	463.1	494.2	402.3	385.4	1060	1382	1202	524	533.6	625	570	524	482	18
Os03g0818300	164	203	202	192.9	229.6	195.5	406	361	413	237.1	239	182.5	185.05	212.83	217.79	155.2	158.7	222.7	216.4	197.5	218.1	332.7	313.6	322.1	232.4	212.1	146.9	147	158	199	18
Os03g0300300	221	166	152	435.1	451.1	487.9	236	202	261	185.4	109.6	219.2	151.3	210.96	151.27	262.7	165.9	185.5	482.1	490.2	467.3	250.7	239.4	189.2	141.6	208.4	188.6	131	196	113	10
Os03g0337500	600	688	670	50.9	57.85	70.07	196	247	299	533.7	541.6	518.4	859.93	659.02	705.64	812	623.5	550.1	31.58	41.12	25.87	330.5	272.9	359	680.3	597.8	464.9	739	601	809	3
Os03g0186950	50	51.2	62.5	108.4	145.5	143.6	203	194	206	88.81	74.55	108.2	103.41	106.41	63.357	131.4	40.19	54.91	198.8	169.3	189.7	115.3	109	135.8	50.71	68.83	59.21	51.8	54.5	52.5	12
Os03g0582900	141	138	107	107.5	124.7	108.1	321	356	278	200.9	194.1	283.2	194.85	189.49	185.32	143.3	166.9	168.9	119.8	131.4	132.8	174.5	160.3	273	217.6	165.9	179.8	134	148	152	5
Os03g0574900	4414	3852	3575	939.2	687	1090	216	583	301	1881	1923	993.5	2148.7	2230.9	2403.6	5803	3977	3511	1103	1068	738.9	656.6	1035	686.9	3376	3185	3189	4456	3894	4216	13
Os03g0637800	45.9	27.9	25.9	13.96	12.66	21.63	7.83	7.59	4.15	32.76	25.22	38.57	25.036	29.87	31.679	59.7	63.89	43.51	25.08	20.16	25.87	11.09	15.68	11.56	57.04	62.23	87.72	62	44.9	45	3
Os03g0394001	0	0	0	0.821	3.616	0	0	0	0	0	0	0	0	0	0	0	0	0	0	2.419	0.862	0	0	0	0	0	0	0	0	0	10
Os03g0402000	303	310	330	353	332.7	426.5	528	423	528	323.3	307	404.6	265.6	310.84	295.4	441.8	242.2	304.6	426.4	391	392.3	330.5	403.4	324.3	238.7	237.6	274.1	271	270	257	12
Os03g0640000	12.2	13	5.39	0.821	0	0	0	0	0	0	0	0	0	0	0	0	4.122	4.144	0	0	0	0.739	0	2.167	0	0	0	0	0.96	0	14
Os03g0163300	1596	1558	1707	1837	2112	1827	1891	1913	1915	1462	1619	1753	1848.3	1795	1621.2	2866	1660	1601	1882	2056	1922	1473	1416	1711	1571	1613	1395	1634	1698	1575	6
Os03g0807900	5174	4180	5135	637.1	567.7	794.1	1682	1790	2006	5658	6196	5719	8136.7	8584	8105	9756	6946	5845	1732	1476	1558	1264	1712	1725	6807	7503	7636	6482	6676	6249	3
Os03g0204900	1.02	2.79	1.08	5.747	6.328	5.19	6.26	1.27	7.6	6.035	3.289	10.35	5.4426	0	1.5839	0	1.031	1.036	3.716	1.613	2.587	3.697	5.701	6.5	1.056	0.943	8.772	2.78	3.83	0	15
Os03g0138400	286	280	217	238.1	265.8	268.2	329	288	337	339.7	367.3	408.3	288.46	284.7	259.76	346.3	309.2	306.7	299.1	288.6	281.9	264.7	259.4	293.2	304.2	387.5	344.3	276	281	273	20
Os03g0405550	0	0	0	0.821	0	0	0	0	0	0	0	0	0	0	0	0	0	0	0	0	0	0	0	0	0	0	0	0	0	0	2
Os03g0385900	503	535	476	488.5	499.9	503.5	740	727	913	611.3	540.5	652.9	628.08	674.89	549.62	644.8	457.6	502.5	542.5	475.7	517.3	578.9	563.7	673.1	525	545.9	587.7	412	426	432	18
Os03g0709300	112	80.1	75.4	9.031	1.808	44.98	24.3	7.59	26.9	87.94	35.08	133.6	44.629	84.011	20.591	143.3	77.29	64.23	28.8	4.838	34.49	11.83	71.98	29.61	29.58	51.86	125	30.5	69.8	25.8	13
Os03g0687200	1403	1294	1526	1413	1564	1396	1448	1487	1485	1285	1460	1392	1611	1342.3	1464.3	2317	1469	1590	1219	1507	1416	1579	1447	1337	1562	1405	1200	1650	1500	1705	6
Os03g0765200	2165	2093	2383	1413	1664	1505	932	1167	907	1903	1964	1757	2220.6	1953.7	2173.2	2854	2214	2030	1692	1985	1751	905	1006	1067	1989	2055	1590	2173	2308	2392	13
Os03g0244800	1.02	0	0	1.642	0	2.595	0	0.63	3.46	1.724	0	0.941	3.2656	1.8669	0	0	1.031	0	1.858	3.225	1.724	0	1.425	2.167	0	0	4.386	0	0.96	1.67	11
Os03g0841100	58.1	79.2	61.4	81.28	75.93	63.15	32.9	39.8	44.2	30.18	49.34	16.93	40.275	29.87	40.39	95.53	50.5	56.98	34.37	37.09	33.63	101.3	54.87	43.33	48.59	35.83	19.74	52.7	57.4	75	8
Os03g0428000	28.5	21.4	22.6	82.92	96.72	87.37	93.9	63.9	85.7	35.35	35.08	48.92	35.921	29.87	35.638	107.5	22.67	37.3	112.4	110.5	136.2	87.99	69.84	67.17	15.85	38.66	32.89	39.8	44.9	30	10
Os03g0404500	1.02	2.79	1.08	9.031	11.75	9.516	21.1	21.5	20.7	0	0	3.763	3.2656	5.6007	0	0	2.061	3.108	13.01	3.225	11.21	5.915	7.839	10.83	0	0	4.386	1.85	0	1.67	12
Os03g0374575	761	570	735	289.8	330.9	318.3	225	269	254	638.9	812.4	932.4	844.69	743.03	852.95	632.9	547.2	502.5	403.2	388.6	395.8	235.9	190.3	192.1	849.3	828.8	929.8	653	595	602	3
Os03g0395801	96.9	98.7	83	87.02	95.82	89.1	163	124	127	110.4	144.7	138.3	109.94	100.81	102.16	107.5	138.1	141.9	130.1	136.3	116.4	110.9	94.78	123.5	135.2	160.3	153.5	116	112	110	5
Os03g0337700	305	301	316	418.7	500.8	386.7	440	404	377	369.9	387	409.3	406.02	392.05	381.73	370.2	342.1	316	427.3	392.7	452.7	334.2	332.1	354.6	375	347	309.2	340	354	355	10
Os03g0441500	686	677	643	764.3	724.1	746.5	757	702	828	747.5	660	918.2	632.43	720.62	590.01	1134	640	668.2	703.2	590.2	710.5	699.5	763.9	728	696.1	871.2	811.4	552	591	517	6
Os03g0130300	10.2	8.38	1.08	32.84	11.75	93.43	8.61	6.96	6.22	75.01	17.54	44.22	5.4426	28.937	22.175	0	1.031	4.144	38.09	6.45	14.66	0.739	3.563	1.444	0	5.657	17.54	3.7	4.78	5	7
Os03g0805300	772	764	795	585.4	565.9	692.9	734	630	636	787.2	777.3	805.3	781.56	721.56	731.78	1684	869.8	852.7	716.2	721.6	729.4	605.6	592.2	664.5	862	851.4	752.2	810	836	826	13
Os03g0143800	564	584	565	486.8	516.2	524.2	272	281	188	299.2	360.7	333.1	382.07	434.99	426.87	716.5	632.7	510.8	362.3	391.9	419	298.7	391.2	359.7	376.1	408.3	436.4	596	538	507	8
Os03g0308900	36.7	38.2	30.2	88.67	80.45	77.85	10.2	12	8.98	12.07	10.96	8.467	33.744	24.27	12.671	71.65	24.73	32.12	25.08	35.48	54.32	31.05	29.22	16.61	8.451	16.03	8.772	23.1	33.5	20	2
Os03g0799000	2374	1710	1655	571.4	687	430.8	2075	1838	1784	2106	2453	3128	1094	971.72	907.59	3952	2696	2012	1269	1178	1107	1091	1185	1349	1466	2523	2230	1412	1661	1604	13
Os03g0279832	2.04	4.66	5.39	1.642	3.616	3.46	2.35	2.53	2.76	12.07	13.16	0.941	9.7967	6.5342	5.5438	0	3.092	3.108	4.645	3.225	0.862	5.176	3.563	1.444	4.225	2.829	4.386	12	3.83	5	7
Os03g0136200	769	610	725	1024	987.1	997.4	218	213	188	676.8	677.6	771.5	637.87	749.56	605.85	1719	1154	794.6	1702	1492	1658	175.2	217.4	266.5	916.9	1164	1132	748	939	833	16
Os03g0319400	9860	8294	####	1871	2078	1790	2018	2541	2448	7833	8771	7294	12086	9897.4	8529.5	33626	10694	9132	2484	3457	2694	1886	2305	1857	8390	8472	5717	####	9181	8424	13
Os03g0856700	21.4	33.5	10.8	32.02	30.73	44.98	29	21.5	56.7	26.73	32.89	63.98	56.603	44.806	37.222	35.82	46.37	31.08	17.65	20.16	37.08	68.02	86.94	66.67	85.57	88.63	74.56	87	44	75.8	1
Os03g0416500	313	226	332	70.6	85.88	64.01	21.9	36.1	29.7	171.6	178.7	251.2	241.65	264.17	285.11	334.3	261.8	249.7	57.6	66.12	52.6	53.24	50.6	52.72	222.9	204.6	265.3	282	257	217	3
Os03g0852700	171	194	176	255.3	288.4	266.4	266	262	267	257.8	246.7	240.8	234.03	227.76	240.76	477.6	298.9	262.1	441.3	354	473.4	181.9	193.1	248.5	252.5	297.9	285.1	257	261	294	16
Os03g0246500	157	97.8	112	58.29	34.35	118.5	40.7	36.7	31.1	61.22	43.86	86.56	26.124	70.009	36.431	95.53	158.7	172	108.7	53.21	75.88	53.98	66.28	72.95	60.21	91.46	111.8	78.6	72.7	65	19
Os03g0836200	1767	1933	1959	4165	4413	3926	1606	1464	1526	1678	1573	1482	1546.8	1595.3	1594.2	2245	1802	1926	3447	3263	3473	1771	1839	1523	1779	1712	1570	1864	1810	1820	10
Os03g0716200	15.3	7.45	15.1	18.06	17.18	12.98	12.5	1.27	10.4	6.897	6.578	15.05	4.3541	9.3355	7.1277	35.82	9.275	8.288	11.15	16.93	25	6.654	7.839	10.83	1.056	6.6	17.54	3.7	5.74	2.5	6
Os03g0193225	182	213	206	217.6	240.5	228.4	289	250	261	279.3	274.1	312.4	291.72	292.17	249.47	310.5	248.4	207.2	269.4	258.8	291.4	193	179.6	186.3	232.4	240.4	274.1	210	217	263	20
Os03g0284100	1918	1804	2009	1016	1043	1058	110	106	101	1914	1931	2439	2030.1	1932.2	2024.3	1815	2125	2129	1188	1166	1174	85.03	57.72	70.06	2046	2248	2283	1936	1900	1952	3
Os03g0760000	0	0	0	371.9	608.4	247.4	15.7	30.4	13.8	0	0	0	0	0	0	0	0	0	8.361	4.031	9.484	2.218	8.552	0	0	0	2.193	0	0	0	2
Os03g0786600	41.8	44.7	45.3	59.93	47.91	38.93	39.9	51.2	44.2	62.08	74.55	68.68	43.541	66.275	53.854	83.59	34.01	29.01	52.95	52.41	42.25	44.36	34.92	31.06	62.33	48.08	37.28	37.9	42.1	44.2	6
Os03g0656075	0	0	0	0	0	0	0.78	0	0	0	0	0	0	0	0	0	0	0	0	0	0	0	0	0	0	0	0	0	0	0	5
Os03g0318600	909	880	976	902.3	852.4	856.4	1050	1068	1171	1008	1027	939.9	1084.2	1092.1	1168.9	1170	947.1	923.1	887.2	1002	1018	1306	1345	1193	1075	1038	962.7	1112	1024	1158	17
Os03g0286100	0	0	0	1.642	0.904	0	0	0	0	0	0	0.941	0	0	0	0	0	0	0.929	0.806	0	0	0	0	0	0	0	0	0	0	10
Os03g0222401	1.02	4.66	0	0.821	0	0.865	3.13	0	3.46	3.449	1.096	1.882	0	0.9335	0.792	0	2.061	2.072	0	0	1.724	4.436	5.701	2.889	3.169	0	0	1.85	0	0.83	17
Os03g0759000	195	156	131	294.7	276.6	260.4	89.2	111	78.1	153.5	184.2	186.3	107.76	74.676	130.67	119.4	176.2	187.5	275	291.1	275.9	201.1	219.5	229.7	128.9	179.1	138.2	141	148	131	10
Os03g0665000	2.04	0.93	2.16	9.031	7.232	4.325	8.61	6.33	15.9	10.35	5.482	2.822	8.7082	2.8004	6.3357	0	8.244	7.252	11.15	5.644	11.21	11.83	5.701	10.83	7.395	3.771	2.193	3.7	2.87	1.67	15
Os03g0182350	0	0	0	0	0	0	0	0	0	0	0	0	0	0	0	0	0	1.036	0	0	0	0	0	0	0	0	0	0	0	0	1
Os03g0583000	66.3	77.3	70	68.96	53.33	70.93	34.4	41.7	45.6	55.18	77.84	76.21	64.223	53.207	50.686	119.4	76.26	63.2	57.6	66.12	54.32	48.8	50.6	53.45	105.6	75.43	78.95	87	92.8	91.6	13
Os03g0164901	0	0	0	3.284	5.424	1.73	2.35	1.27	4.84	0	0	0.941	1.0885	0	0	11.94	0	0	0	0.806	0.862	6.654	8.552	6.5	0	0	0	0	0.96	0	6
Os03g0837000	7.14	6.52	6.47	10.67	14.46	13.84	6.26	3.8	0	3.449	1.096	5.645	9.7967	11.201	3.1679	0	1.031	8.288	12.08	12.09	25	8.873	4.276	7.222	5.282	6.6	2.193	9.25	13.4	2.5	16
Os03g0810750	1.02	2.79	1.08	18.88	14.46	10.38	9.39	8.22	4.15	2.587	3.289	1.882	2.177	9.3345	4.7518	11.94	1.031	0	6.503	13.71	2.587	8.133	10.69	7.945	6.338	5.657	4.386	5.55	1.91	7.5	2
Os03g0572250	240	311	241	401.5	466.4	341.7	611	579	535	169.9	237.9	327.4	200.29	188.56	141.76	358.2	239.1	281.8	360.4	285.4	291.4	431.8	337.1	530.1	241.9	241.4	203.9	197	176	197	18
Os03g0207200	30.6	19.6	24.8	6.568	7.232	18.17	25.8	7.59	20	22.42	18.64	24.46	8.7082	14.002	11.088	35.82	25.76	12.43	8.361	19.35	20.69	8.133	13.54	22.39	17.96	40.54	24.12	15.7	12.4	20	13
Os03g0240500	989	1042	964	1344	1466	1171	1096	1168	1182	994.1	980.2	1166	1182.1	1136.9	953.53	1481	1018	945.9	1269	1327	1273	1031	898.6	1022	998.3	979.6	879.4	769	893	847	10
Os03g0833600	3.06	11.2	3.23	9.031	9.944	12.11	10.2	10.1	6.22	9.484	24.12	10.35	10.885	18.669	15.047	35.82	5.153	6.216	7.432	3.225	6.898	17.01	7.126	9.389	7.395	6.6	10.96	6.48	5.74	4.17	6
Os03g0438100	1303	963	955	811.9	758.4	1004	592	566	717	1048	811.3	1057	555.15	595.54	555.96	1254	838.9	773.9	1032	820.8	1083	644	666.3	764.9	605.3	792	1340	610	745	668	11
Os03g0693400	249	190	217	204.4	176.3	186	308	300	373	405.2	435.3	414	328.73	233.36	346.88	250.8	186.5	205.1	248	209.6	181.1	253.6	243.7	210.2	313.7	318.7	263.2	206	214	208	5
Os03g0655700	123	170	177	258.6	289.3	274.2	391	330	375	204.3	144.7	192.9	191.58	165.22	146.51	238.8	128.8	128.5	393.9	462	451.8	198.9	219.5	265.8	144.7	159.3	151.3	117	140	100	12
Os03g0109600	2076	1827	1905	2281	2514	2326	3576	3232	3528	2202	2015	2322	2153.1	2160.9	1755.8	2293	2006	1937	3268	3115	2934	2143	2132	2514	1972	2202	2351	1589	1589	1475	15
Os03g0353500	363	318	311	340.7	321.8	291.5	492	498	466	424.2	385.9	459.1	339.62	367.78	315.99	286.6	319.5	305.6	419.9	367.7	337.1	309.1	323.5	317.8	327.5	344.1	309.2	314	286	258	15
Os03g0788300	607	612	544	540.2	599.3	609	611	517	554	538.9	540.5	573.9	600.86	559.14	624.07	812	624.5	667.2	527.6	519.2	533.7	637.4	563.7	574.9	595.8	672.2	576.7	674	609	655	13
Os03g0424000	421	440	417	967.9	1030	740.5	466	579	434	379.4	403.5	370.7	499.63	421.92	433.21	955.3	360.7	473.5	700.4	965.1	660.5	671.4	535.9	423.4	350.7	355.5	315.8	478	520	583	10
Os03g0620800	412	369	411	423.6	449.3	429.1	468	459	556	394	348.7	345.3	301.52	299.64	350.84	382.1	320.5	305.6	349.3	343.5	338.9	411.8	467.5	469.5	353.9	316.8	339.9	327	325	316	18
Os03g0626100	0	0	0	1.642	1.808	2.595	3.91	17.1	3.46	0.862	0	0	0	0	0.792	0	0	0	0.929	0.806	0	2.958	2.138	4.333	0	0	0	0.93	0	0	18
Os03g0798400	31.6	23.3	18.3	18.06	9.944	42.39	46.2	33.5	28.3	47.42	53.72	62.09	40.275	42.939	38.014	83.59	48.43	41.44	39.02	29.83	46.56	27.36	25.65	39.72	48.59	59.4	48.24	43.5	40.2	51.7	13
Os03g0627500	541	560	545	474.5	520.7	497.4	557	496	542	569.9	586.6	545.7	622.63	590.87	652.58	740.3	578.1	511.8	491.4	447.5	448.4	469.5	420.5	465.1	576.8	561.9	486.8	609	597	622	13
Os03g0287500	5.1	6.52	4.31	4.105	7.232	0	3.91	1.9	2.07	1.724	5.482	3.763	0	0	3.9598	0	6.183	4.144	0.929	2.419	2.587	8.873	1.425	7.222	3.169	4.714	6.579	4.63	3.83	3.33	9

Os03g0745100	1.02	0	0	2.463	0.904	1.73	1.57	1.27	3.46	0	0	0	0	0	0	0	0	0	0	0.806	3.449	2.218	2.851	0	0	0	0	0	0	0	12
Os03g0563400	0	0	0	0	0.904	0	0	0	0	0	0	0	0	0	0	0	0	0	0	0	0	0	0	0	0	0	0	0	0	0	2
Os03g0769450	0	0	0	0	0	0	0.78	0.63	0	1.724	0	0	1.0885	2.8004	4.7518	11.94	0	1.036	2.787	3.225	3.449	0	2.138	0.722	0	0	2.193	0	0.96	0.83	6
Os03g0760200	2296	1979	2108	532.8	346.2	590	341	283	392	2076	1763	3245	1380.2	1831.4	1640.2	1170	1848	1599	715.3	495.9	579.4	488.7	598.6	548.9	1581	1821	1910	1429	1020	1236	3
Os03g0119400	0	0	0	0	0	0	0	0	0	0	0	0	0	0	0	0	0	0	0	0	0	0	0.713	0	0	0	0	0	0	0	17
Os03g0831000	0	0	1.08	0	0.904	0.865	0	0	0.69	0.862	0	0	0	0	0.792	0	0	0	0	2.419	0.862	0.739	0	6.5	0	0	0	0.93	0.96	1.67	9
Os03g0279600	477	493	461	1057	1194	1091	466	326	375	569	525.2	595.5	726.04	684.22	504.48	967.2	496.7	512.8	1174	1175	1213	261.7	311.4	255.7	504.9	432.8	394.7	372	448	501	10
Os03g0165050	41.8	38.2	30.2	29.56	19.89	22.49	31.3	34.2	31.1	42.25	47.14	56.45	50.072	37.338	45.142	47.76	37.1	32.12	45.52	34.67	49.15	31.79	27.08	40.45	39.09	53.74	67.98	37	43	22.5	20
Os03g0818700	9.18	23.3	12.9	10.67	12.66	7.785	93.2	110	91.9	31.9	24.12	49.86	25.036	18.669	22.967	23.88	10.31	21.76	9.29	8.869	12.07	49.54	67.7	79.45	21.13	22.63	35.09	16.7	10.5	10	18
Os03g0832800	0	1.86	0	0	0.904	0	0	1.27	0	0	0	0	0	0	0	0	0	0	0	0	0	0	2.851	2.889	0	0	0	0	0	2.5	17
Os03g0715332	136	123	157	91.13	77.74	90.83	159	145	159	138.8	159	194.7	188.31	122.28	130.67	191.1	164.9	145	125.4	113.7	103.5	125	144.7	145.9	164.8	162.2	190.8	133	158	162	5
Os03g0287400	0	0.93	0	0.821	1.808	2.595	0	0.63	0	1.724	0	0	1.0885	0	0	0	0	0	0.929	0.806	0	0.739	1.425	0	0	0	0	0	0	0	2
Os03g0686050	0	0	1.08	3.284	0.904	3.46	0	0	0	0	0	0	1.0885	0	0	0	2.061	1.036	0	0.806	2.587	0.739	0	2.167	1.056	0.943	0	3.7	2.87	1.67	1
Os03g0802600	0	3.73	3.23	13.96	18.98	10.38	19.6	13.9	11.7	0.862	0	2.822	0	0	0	0	1.031	2.072	6.503	4.031	0.862	3.697	5.701	4.333	0	0	0	0.93	2.87	0	12
Os03g0722200	199	266	213	385	363.4	357.3	668	615	658	305.2	383.7	474.2	448.47	298.7	285.11	274.6	236	250.7	402.2	443.5	389.7	478.4	408.3	586.5	352.8	290.4	247.8	170	207	266	18
Os03g0382100	4.08	8.38	4.31	0	0	0.865	2.35	1.27	14.5	4.311	2.193	1.882	9.7967	0	5.5438	0	0	0	0.929	0	0	8.873	10.69	16.61	2.113	1.886	0	5.55	0.96	2.5	9
Os03g0120000	69.3	95	64.7	19.7	12.66	21.63	19.6	18.3	6.22	63.8	41.66	65.86	33.744	45.739	22.967	71.65	55.65	44.55	13.01	17.74	18.11	23.66	8.552	19.5	25.35	38.66	41.67	34.2	42.1	36.7	14
Os03g0804200	265	338	212	114.9	67.8	69.2	56.4	44.9	53.9	257.8	288.4	361.3	290.63	209.09	198.78	394.1	269	238.3	85.46	61.28	65.53	90.94	109	106.2	640.2	482.7	436.4	522	264	419	3
Os03g0167200	85.6	134	135	112.5	128.4	122	99.4	127	110	68.11	106.4	63.98	112.12	81.21	117.21	167.2	126.8	111.9	70.6	131.4	63.8	165.6	116.2	120.6	95.07	84.86	54.82	140	155	157	8
Os03g0820100	521	564	567	513.1	508	521.6	557	572	518	550.9	529.6	594.6	568.21	552.6	580.51	800	578.1	558.4	540.7	458	513	561.9	532.3	567	537.7	577	657.9	569	508	524	6
Os03g0267300	7540	7991	9143	5458	5552	5439	####	####	####	10844	12087	11728	10903	10872	11498	17099	10443	9223	8903	6935	9050	9134	9164	12944	10550	11044	10256	8166	9928	####	5
Os03g0594700	117	142	121	145.3	179	178.2	128	129	137	109.5	129.4	94.08	136.06	166.15	133.05	274.6	137.1	131.6	106.8	140.3	167.3	150.8	144.7	167.6	120.4	134.8	100.9	136	112	167	6
Os03g0346800	247	225	232	262.7	291.1	261.2	198	230	212	245.7	221.5	204.2	272.13	265.1	305.7	465.7	276.2	260	274	285.4	247.5	163.4	178.9	217.4	249.3	243.3	252.2	238	281	228	6
Os03g0779000	119	129	114	106.7	101.2	101.2	84.6	62.6	96.7	142.3	149.1	141.1	115.38	124.15	144.93	417.9	153.5	161.6	105	107.2	123.3	152.3	135.4	154.6	153.2	187.6	182	203	142	178	13
Os03g0693900	1.02	0	0	0	0	0	1.57	0	15.2	0	2.193	0.941	2.177	0	0	0	0	0	0	0	0	0	0	0	0	0	0	0	0	0	18
Os03g0211100	320	346	335	285.7	358	346.9	468	430	484	369.9	342.1	345.3	366.83	391.12	412.61	835.9	372	331.5	306.6	283.8	347.5	550.1	466.1	437	388.7	402.6	346.5	360	323	390	6
Os03g0843600	480	441	387	466.3	527.9	489.6	312	341	384	363.8	391.4	362.2	421.26	404.18	441.13	394.1	426.6	465.2	402.2	456.4	387.1	514.6	419.7	426.1	403.5	398.8	386	429	431	435	8
Os03g0725300	159	162	142	318.5	310.1	286.3	235	232	218	146.6	140.3	139.2	115.38	151.22	145.72	238.8	155.6	155.4	341.9	299.9	277.6	254.3	233.7	184.2	157.4	133.9	131.6	164	146	168	10
Os03g0144000	264	309	287	444.1	431.2	433.4	318	354	292	281.1	304.8	256.8	303.7	274.43	293.82	632.9	271	313.9	314.9	330.6	330.2	346.8	374.1	286	254.6	266.8	162.3	222	253	263	6
Os03g0389400	23.4	19.6	15.1	4.926	6.328	6.92	7.05	13.9	4.84	27.59	18.64	41.4	7.6196	22.403	19.799	59.7	42.25	32.12	15.79	11.29	9.484	5.176	2.851	8.667	32.75	25.46	59.21	24.1	15.3	13.3	13
Os03g0122000	260	349	306	888.3	918.4	970.6	1047	1091	1168	469.9	510.9	492	441.94	353.78	374.6	441.8	309.2	284.9	719	721.6	815.7	1221	1045	1052	527.1	410.1	296	315	221	409	12
Os03g0125100	150	91.3	100	31.2	37.97	41.52	79.9	64.5	71.9	75.87	75.65	112.9	60.957	100.81	83.156	238.8	113.4	127.4	84.53	66.12	68.98	68.02	72.69	60.67	87.68	89.57	100.9	96.2	60.2	86.6	13
Os03g0251800	139	92.2	89.4	75.53	50.62	95.16	79.9	83.5	91.9	80.18	89.9	84.67	85.993	86.811	65.733	59.7	141.2	96.35	86.39	65.31	73.29	207	220.9	175.5	133.1	118.8	164.5	159	164	153	9
Os03g0213700	36.7	25.1	29.1	18.88	21.7	27.68	22.7	26.6	18.7	33.63	30.7	8.467	17.416	23.336	27.719	23.88	30.92	26.94	26.94	21.77	27.59	28.1	28.51	23.11	21.13	22.63	19.74	24.1	23.9	25.8	19
Os03g0627100	0	0	0	0	0	0	0	0	0	0	0	0	0	0	0	0	0	1.036	0	0	0	0	0	0	0	0	0	0	0	0	1
Os03g0105300	401	315	290	377.6	318.2	409.2	383	359	426	392.3	404.6	529.7	359.21	382.72	365.89	609	331.8	431	372.5	365.2	432	425.9	421.9	422.5	410.9	468.6	489	368	368	359	6
Os03g0758900	330	373	378	1050	1195	1038	940	962	1175	325.9	335.5	376.3	545.35	339.78	239.97	525.4	246.3	329.5	878.8	907.9	914	822.2	804.6	905	255.6	215.9	239	228	274	282	12
Os03g0738300	727	641	648	621.5	567.7	617.6	1032	929	879	1023	970.3	1110	1006.9	1080.9	984.41	1445	834.7	857.8	942.9	947.4	932.1	732	719.8	797.4	967.6	1130	1210	809	828	775	13
Os03g0256800	3.06	1.86	1.08	22.17	16.27	19.03	6.26	3.8	11.1	1.724	2.193	3.763	1.0885	0	3.1679	0	6.183	2.072	9.29	13.71	7.76	24.4	26.37	19.5	2.113	3.771	2.193	5.55	1.91	0	17
Os03g0175800	4.08	0	2.16	9.852	7.232	6.055	6.26	0	2.07	0	1.096	1.882	0	2.8004	0.792	0	1.031	13.47	3.716	0.806	4.311	0	1.425	2.889	1.056	2.829	0	0	3.83	1.67	2
Os03g0711525	69.3	98.7	74.4	120.7	128.4	114.2	36	53.1	68.4	39.66	39.47	20.7	51.16	38.272	79.989	107.5	68.02	49.73	27.87	28.22	50.87	171.5	103.3	74.39	62.33	29.23	43.86	87.9	73.6	100	8
Os03g0210200	3.06	15.8	18.3	37.76	41.58	31.14	70.5	87.9	92.6	6.897	8.771	5.645	9.7967	9.3345	14.255	0	5.153	4.144	32.51	22.58	25	37.71	47.03	59.95	12.68	1.886	10.96	4.63	15.3	6.66	18
Os03g0124900	2.04	9.31	5.39	27.09	18.98	25.95	9.39	12.7	16.6	9.484	4.386	15.05	5.4426	9.3345	3.9598	0	10.31	11.4	10.22	33.06	31.9	9.612	13.54	5.778	6.338	7.543	2.193	3.7	11.5	11.7	10
Os03g0781501	1617	1164	1209	1743	1782	1978	1932	1527	2081	1639	1540	2941	1500	2083.5	1332.9	1648	1187	1391	2315	2047	2527	1369	1536	1429	1622	2029	2533	1134	1181	838	15
Os03g0832200	63.2	52.2	56	25.45	23.5	32.01	14.9	36.7	16.6	110.4	76.75	128	38.098	81.21	45.934	23.88	43.28	36.26	53.88	23.38	24.14	10.35	9.977	6.5	26.41	54.68	65.79	36.1	42.1	33.3	20
Os03g0682666	32.6	29.8	34.5	26.27	37.97	25.09	21.9	18.3	30.4	22.42	37.28	31.05	27.213	35.471	28.511	47.76	25.76	31.08	37.16	28.22	23.28	33.27	33.49	26.72	39.09	21.69	21.93	35.2	32.5	30	6
Os03g0169300	12.2	6.52	4.31	42.69	38.87	37.2	8.61	10.1	13.8	6.035	9.868	0	6.5311	0.9335	7.9197	11.94	5.153	3.108	21.37	21.77	22.42	21.44	16.39	15.89	10.56	2.829	0	7.4	7.65	8.33	10
Os03g0275100	0	0	0	0.821	0.904	0.865	1.57	3.16	1.38	0	0	0	0	0	0	0	1.031	0	0	0	2.587	9.612	2.138	4.333	0	0	0	0.93	0	0	9
Os03g0371400	574	651	625	788.1	870.5	678.2	253	300	273	1040	902.3	983.2	1211.5	893.31	998.67	680.6	742	705.5	805.4	851.4	682	173.8	238.7	135.8	834.5	688.3	671	605	714	863	4
Os03g0605400	47.9	27	23.7	28.73	25.31	31.14	25.1	20.9	8.98	20.69	25.22	7.527	11.974	25.203	15.839	11.94	25.76	16.58	33.44	33.06	36.21	23.66	19.95	14.44	17.96	17.91	28.51	28.7	14.3	35.8	19
Os03g0134300	3226	2835	3505	2786	2856	2868	1675	1800	1971	2765	2648	3521	3924.1	3934.5	3122.7	4561	3451	3385	2919	2781	3644	1385	1417	1523	2393	2393	2270	2200	2462	2293	13
Os03g0136500	163	191	138	64.04	61.47	56.23	72	90.4	78.1	100.9	76.75	75.27	65.311	57.874	91.868	95.53	114.4	112.9	86.39	53.21	71.56	90.94	108.3	156	89.79	59.4	59.21	60.1	65	46.7	14
Os03g0254200	369	444	431	32.84	73.22	50.17	32.9	46.8	35.9	248.3	346.5	155.2	371.19	352.84	374.6	1349	578.1	436.2	65.96	58.05	48.28	31.79	27.08	34.67	361.3	341.3	258.8	634	661	877	13
Os03g0265200	155	132	141	136.3	138.3	105.5	83.8	90.4	84.3	98.29	122.8	119.5	187.23	118.55	140.18	262.7	138.1	137.8	127.3	125.8	156.1	94.64	84.8	109.1	154.2	120.7	105.3	125	152	168	13
Os03g0791366	0	0	0	0.821	0	0	0	0	0	0	0	0	0	0	0	0	0	0	0	0	0	0	0	0	0	0	0	0	0	0	2
Os03g0747900	592	272	458	179.8	163.6	233.6	86.1	109	84.3	220.7	166.7	326.5	198.11	336.98	259.76	441.8	532.8	439.3	210.9	162.9	208.7	166.4	164.6	182.7	319	381.9	535.1	424	366	367	3
Os03g0425300	343	375	304	415.4	458.3	320.1	333	307	328	275.9	302.6	210.7	306.96	228.7	311.24	716.5	399.8	316	361.4	368.5	358.7	434.8	425.4	421.1	352.8	375.3	390.3	483	364	495	6
Os03g0190000	120	88.5	116	165.8	151	118.5	198	216	249	131.1	121.7	150.5	109.94	99.879	110.88	131.4	135	87.03	178.4	197.5	235.4	125.7	121.9	186.3	98.24	111.3	85.52	81.4	77.5	120	12
Os03g0759600	1.02	1.86	0	1.642	2.712	1.73	0	3.16	0	0	3.289	1.882	1.0885	2.8004	0	0	2.061	0	0	4.031	2.587	2.958	2.138	4.333	0	0.943	0	0.93	0	1.67	9
Os03g0824200	257	309	305	454.8	404.1	437.7	658	552	688	378.5	418.8	422.4	363.57	347.24	344.51	358.2	266.9	265.2	453.3	433.8	444.9	584.9	498.8	584.3	369.7	425.2	414.5	294	280	296	18
Os03g0341200	260	277	266	264.4	268.5	288.1	402	383	324	295.7	355.2	333.1	347.24	323.91	353.22	549.3	336	278.7	417.1	389.4	385.4	256.6	241.6	294.7	395.1	384.7	407.9	329	341	306	6
Os03g0395900	4457	3917	4848	1997	1827	2138	1783	1899	1787	4211	4722	4770	5654.9	5113.4	5158.9	6806	4862	4572	2345	2298	2109	2240	1961	2049	5068	4883	5024	5085	4723	4981	3
Os03g0723700	1.02	0.93	1.08	1.642	0.904	0	0	0	0	0.862	1.096	0	0	0	0.792	0	0	1.036	0	0	0	0	0	0	0	0.943	0	0	0	0.83	8
Os03g0826400	307	332	312	312.8	346.2	315.7	415	338	352	380.2	328.9	404.6	377.72	357.51	339.75	382.1	386.4	367.8	409.7	354.8	369.9	273.6	280.8	297.6	359.2	409.2	372.8	358	480	383	20
Os03g0358200	3.06	0.93	0	7.389	3.616	8.65	2.35	4.43	4.84	2.587	0	0	1.0885	4.6673	0.792	0	4.122	2.072	7.432	1.613	1.724	5.915	32.78	12.28	1.056	1.886	2.193	0.93	2.87	0	17
Os03g0162000	27.5	19.6	7.54	233.2	268.5	263.8	168	223	152	3.449	12.06	1.882	11.974	5.6007	3.1679	23.88	9.275	7.252	126.3	191.9	100	298	220.9	174.1	4.225	1.886	8.772	7.4	13.4	10.8	12
Os03g0338000	0	0.93	0	1.642	0	0	0	0	0	2.587	0	0	0	0	1.5839	0	0	1.036	0	0	0	0	0	0	0	0	0	1.85	0.96	1.67	7
Os03g0659350	2.04	0.93	1.08	0	0	0	0	0	0.69	0	3.289	2.822	2.177	6.5342	3.1679	11.94	3.092	5.18	0	0	0	0	0	1.444	3.169	3.771	4.386	4.63	3.83	1.67	13
Os03g0809900	2.04	9.31	1.08	10.67	11.75	12.11	24.3	31.6	11.1	2.587	6.578	5.645	4.3541	3.7338	1.5839	0	9.275	5.18	15.79	8.063	13.8	14.79	10.69	17.33	1.056	3.771	2.193	4.63	1.91	5.83	12
Os03g0826500	50	37.3	55	370.3	429.4	332.2	380	372	312	32.76	29.6	37.63	33.744	46.673	40.39	11.94	31.95	47.66	251.7	277.4	214.7	293.5	258.7	281	30.63	32.06	24.12	15.7	38.3	21.7	12
Os03g0113700	303	195	247	68.96	75.03	84.77	61.1	51.2	48.4	447.5	309.2	582.4	404.93	358.45	365.1	203	216.4	202	138.4	121.7	130.2	39.19	29.93	41.89	325.4	285.7	348.7	259	220	184	3
Os03g0111600	308	308	355	330	351.6	336.5	262	309	327	288.8	279.6	258.7	359.21	345.38	333.42	346.3	359.7	299.4	350.2	373.3	304.4	309.8	296.5	239.1	334.9	334.7	346.5	396	364	352	8
Os03g0439700	3720	3653	4058	939.2	945.6	900.5	1505	1639	1590	4861	5458	5337	5892.2	5033.2	5398.8	6914	5014	4166	1301	1330	1119	973.8	1003	1186	5243	5307	4318	4235	4477	4609	3
Os03g0755625	26.5	18.6	22.6	18.88	21.7	19.03	24.3	17.1	20	21.55	21.93	56.45	28.302	28.937	25.343	59.7	32.98	21.76	24.15	20.16	31.04	19.96	7.839	12.28	39.09	65.06	67.98	31.5	33.5	30	20
Os03g0648600	16.3	14	17.2	0	0	1.73	0.78	4.43	1.38	1.724	0	5.645	0	0	3.9598	35.82	8.244	11.4	4.645	0	0	5.915	6.414	5.778	0	1.886	4.386	2.78	2.87	0	6
Os03g0275900	3.06	3.73	0	0	0	0.865	0	0.63	0	0	1.096	2.822	1.0885	0.9335	0	0	0	0	0	0	0	0	0.713	0	2.113	0	0	0	0	1.67	19
Os03g0284700	0	0	0	0	0	0	0	0	0	0	0	0	0	0	0	0	1.031	0	0	0	0	0	0.713	0	0	0	0	0.93	0	0	1
Os03g0674900	1.02	0	1.08	0	4.52	1.73	24.3	18.3	13.1	0	0	0.941	0	0	1.5839	0	2.061	1.036	0	1.613	4.311	11.09	20.67	9.389	4.225	2.829	2.193	2.78	3.83	0.83	18
Os03g0639700	597	703	712	871.1	889.5	945.5	931	908	896	795.8	900.1	770.5	973.14	852.24	799.89	1122	666.8	665.1	906.7	991.7	1080	664.7	699.1	749.7	799.7	774.1	699.5	652	755	737	6
Os03g0636600	189	217	212	221.7	191.6	186.9	252	256	240	223.3	251.1	189.1	259.07	219.36	267.68	513.5	216.4	196.8	200.7	236.2	219.9	187.1	210.2	214.5	223.9	262.1	230.3	246	284	277	6
Os03g0411100	2142	2699	2419	2656	3036	2448	2439	1706	1826	2043	1979	2430	2038.8	2141.3	1720.2	2746	2236	2137	2425	2595	2867	1714	1693	1863	2244	2594	2697	2315	1968	2454	16
Os03g0698800	951	738	831	524.6	450.2	500.9	618	645	618	723.4	633.7	897.5	488.75	481.66	506.86	1290	705.9	596.8	476.6	514.4	574.2	706.9	644.2	763.4	722.6	735.4	892.5	539	576	518	6
Os03g0343500	930	767	779	1066	1152	1148	1152	934	1191	928.6	937.4	1615	989.46	1053.9	687.43	1039	735.8	789.5	1233	1096	1146	904.3	846.6	912.9	780.7	1087	1186	595	710	487	15
Os03g0793700	0	0	0	0	0	0	0	0.63	0	0	1.096	1.882	3.2656	0	0	0	1.031	0	0	0	0.862	0	0	0.722	0	0.943	0	2.78	0	0	4
Os03g0281500	39.8	41	30.2	32.02	20.79	37.2	34.4	36.1	32.5	60.35	36.18	54.57	29.39	45.739	43.558	71.65	36.07	36.26	39.02	33.06	56.91	35.49	29.93	38.28	42.25	37.71	54.82	30.5	31.6	17.5	6
Os03g0803300	1.02	2.79	0	0.821	1.808	5.19	6.26	3.16	7.6	1.724	2.193	3.763	2.177	2.8004	2.3759	0	1.031	0	1.858	4.838	6.036	2.958	4.276	4.333	1.056	0	6.579	4.63	0.96	1.67	15
Os03g0177100	552	520	579	417.9	427.6	442.9	363	397	347	489.7	468.2	350.9	460.44	494.73	520.32	835.9	584.3	596.8	332.6	435.4	333.7	560.5	406.9	418.9	490.2	468.6	475.9	666	645	602	13
Os03g0581100	112	141	125	182.3	195.3	141	129	114	122	106.9	123.9	118.5	125.18	132.55	130.67	179.1	124.7	158.5	187.6	178.2	142.3	117.6	134.7	136.5	135.2	112.2	149.1	149	159	150	10
Os03g0571700	584	609	494	834.1	900.1	780.3	359	357	220	510.4	440.8	534.4	228.59	404.18	268.48	501.5	480.2	449.6	714.4	584.6	779.4	382.3	334.9	483.2	299	377.1	342.1	391	463	376	10
Os03g0812500	1317	1285	1567	2346	2556	2442	172	130	126	819.9	873.8	552.3	1111.4	1177.1	1074.7	1994	1124	1354	2462	2430	2603	164.1	198.1	124.2	770.1	580.8	530.7	970	912	1148	16
Os03g0153400	299	306	310	247.1	220.6	243.9	369	395	321	576.8	603	602.1	377.72	483.53	417.37	429.9	357.6	296.3	475.6	333	363.9	147.9	207.4	140.8	398.2	399.8	510.9	359	416	411	20
Os03g0283300	848	727	913	739.7	677.1	778.5	1149	882	1053	1047	1039	1408	1095.1	1177.1	994.71	1421	1201	1074	1172	1024	1183	807.4	698.4	833.5	1121	1272	1338	990	1004	954	20
Os03g0707300	937	898	955	652.7	660.8	718	641	603	700	749.2	814.6	748	975.31	850.37	911.55	1266	985.2	922.1	677.2	722.4	748.4	800.8	743.3	684.7	883.1	821.2	822.3	993	936	1007	13
Os03g0820400	57.1	167	45.3	2.463	0.904	3.46	0	2.53	0.69	2.587	0	0	2.177	0	0	95.53	52.56	55.95	0	1.613	0	2.218	7.839	23.11	0	0	0	0	3.83	0	14
Os03g0121200	6.12	7.45	8.62	0.821	2.712	3.46	4.7	2.53	4.84	11.21	3.289	13.17	2.177	4.6673	1.5839	11.94	9.275	15.54	0	2.419	3.449	1.479	2.138	4.333	3.169	12.26	17.54	5.55	1.91	5	20
Os03g0596700	0	0	0	1.642	0	0.865	0	0	0	0	0	0	5.4426	0	0	0	0	1.036	0	0	0	0.739	1.425	0	0	0	0	0.93	2.87	0	4
Os03g0306400	3.06	0	1.08	18.88	25.31	12.11	0	0.63	1.38	0.862	2.193	5.645	1.0885	1.8669	0	23.88	2.061	7.252	3.716	12.09	7.76	0	0.713	0	0	0	0	2.78	0	4.17	10
Os03g0270800	4.08	0	2.16	2.463	0.904	2.595	3.91	3.16	1.38	0	1.096	0.941	0	0	3.1679	0	2.061	1.036	4.645	0.806	3.449	1.479	9.264	5.778	2.113	0	0	0.93	0.96	0.83	17
Os03g0804800	1018	972	922	1033	1168	1099	922	860	905	904.4	901.2	1056	944.84	987.59	847.4	1218	907.9	911.7	1035	828.1	924.3	814.8	890.8	770.6	901.1	1066	1039	796	830	846	6
Os03g0239000	0	0	0	0	0	0	1.57	3.16	3.46	0	0	0	1.0885	0	0	0	1.031	0	0.929	0	0	4.436	4.276	6.5	0	2.829	2.193	0	0	0	9
Os03g0115800	0	0.93	0	4.105	1.808	0	0	0	0.69	0	0	1.882	0	0	0	0	0	0	0	2.419	0	0	0	0	0	0	0	0	0.96	0	2
Os03g0382350	6471	6845	7015	5392	5708	6101	6666	7215	7444	7279	8953	8051	8122.5	8520.5	8395.6	9911	9151	8901	5949	6863	6935	9143	6442	6993	10330	10110	9890	8220	7521	8907	3
Os03g0664700	232	246	256	302.1	287.5	331.3	268	243	245	271.6	260.9	231.4	272.13	262.3	243.93	322.4	205.1	207.2	229.5	257.2	232.8	271.4	275.8	252.1	207	164.1	203.9	239	234	292	2
Os03g0209500	80.5	55.9	65.7	38.59	57.85	53.63	88.5	60.1	82.2	47.42	38.37	50.8	60.957	76.543	49.102	83.59	98.93	55.95	27.87	48.38	72.43	65.81	52.02	95.34	33.8	50.91	85.52	47.2	63.1	69.1	9
Os03g0190400	33.6	35.4	29.1	1.642	2.712	0	5.48	1.27	8.29	12.07	14.25	9.408	14.151	7.4676	16.631	47.76	25.76	30.05	4.645	1.613	2.587	11.09	5.701	5.778	9.507	19.8	15.35	25	15.3	9.16	13
Os03g0216800	1.02	0	0	0	0	0	0	0	0	0	0	0	0	0	0	0	0	0	0	0	0	0	0	0	0	0	0	0	0	0	19
Os03g0646100	12.2	13	5.39	20.52	14.46	28.55	39.9	13.9	26.3	14.66	8.771	9.408	2.177	4.6673	6.3357	0	9.275	5.18	18.58	21.77	26.73	8.133	4.988	15.17	5.282	8.486	15.35	4.63	0.96	1.67	15
Os03g0343400	1925	1613	2014	1611	1533	1388	1650	1812	1987	2390	2357	2938	2587.4	2608.1	2535.1	3391	2244	2061	2271	1850	2168	1077	1106	1427	2260	2301	2184	1688	2272	1815	13
Os03g0838900	5799	4916	5942	1736	1846	1867	2021	2473	2137	4926	5243	5655	6742.3	7426.5	6675.5	11929	7428	6448	2685	2335	2593	1908	2157	2228	6174	6286	5546	6231	6278	6262	13
Os03g0820700	352	272	303	203.6	226.9	259.5	303	288	276	239.7	247.8	248.4	323.29	238.03	274.81	585.1	289.6	283.9	224.8	258.8	236.2	221.1	282.2	256.4	255.6	269.7	289.5	241	227	234	6
Os03g0309800	1170	1331	1317	2200	2442	2244	2136	2214	2226	1098	1420	1387	1402	1206	1227.5	2137	1152	1302	1971	2249	2177	1976	1693	2127	1218	1104	975.8	1059	1180	1186	12
Os03g0366900	9.18	9.31	8.62	7.389	6.328	4.325	11.7	8.22	7.6	10.35	5.482	6.586	5.4426	2.8004	7.9197	0	5.153	9.324	0.929	0.806	2.587	12.57	12.83	3.611	8.451	4.714	13.16	12	12.4	15	1
Os03g0776150	443	355	353	449.9	469.2	495.7	576	704	639	388.8	425.4	402.7	584.54	372.45	505.27	620.9	392.6	502.5	477.5	492.6	505.3	543.4	501	578.5	418.3	443.1	379.4	457	493	540	18
Os03g0689688	10.2	5.59	6.47	13.96	6.328	4.325	3.91	3.8	8.98	1.724	1.096	1.882	1.0885	0	4.7518	0	3.092	6.216	3.716	0.806	1.724	19.96	19.95	13	4.225	4.714	0	10.2	2.87	5	17
Os03g0719300	536	641	619	739.7	682.5	706.7	598	601	612	978.6	1010	1224	879.52	787.83	902.84	740.3	443.1	539.8	525.8	567.6	543.2	698.7	669.2	582.9	902.1	836.3	693	520	615	709	20
Os03g0106400	3.06	1.86	1.08	8.21	4.52	5.19	12.5	4.43	17.3	3.449	1.096	3.763	0	2.8004	0	23.88	0	3.108	10.22	9.675	8.622	5.915	4.988	13	1.056	3.771	0	0.93	0.96	0.83	6
Os03g0130800	922	904	886	683.9	656.3	646.2	914	903	889	891.5	957.2	955.9	1080.9	885.85	1070.7	1767	965.6	1001	614	657.1	688.9	908	840.9	898.5	1011	1061	964.9	981	958	1075	13

Os03g0219100	11.2	8.38	5.39	0	0	0.865	6.26	3.16	2.76	9.484	4.386	8.467	2.177	1.8669	12.671	11.94	14.43	5.18	0.929	1.613	0	8.873	2.851	23.11	3.169	7.543	4.386	2.78	6.69	0	9
Os03g0340700	378	357	373	350.6	318.2	419.5	403	362	451	280.2	275.2	300.1	249.27	292.17	286.69	549.3	390.6	275.6	252.7	287	332.8	312.8	317.8	368.3	332.8	257.4	287.3	335	271	332	6
Os03g0208800	3.06	3.73	3.23	20.52	28.93	14.71	0.78	9.49	2.07	2.587	0	2.822	3.2656	0.9335	0	0	0	4.144	11.15	21.77	9.484	6.654	8.552	2.889	2.113	1.886	6.579	1.85	6.69	1.67	10
Os03g0769500	0	4.66	0	3.284	1.808	1.73	0.78	8.22	0.69	0	1.096	0.941	0	0.9335	0	0	0	0	3.716	3.225	4.311	1.479	0.713	2.167	2.113	0	2.193	0.93	0	0.83	12
Os03g0556200	319	309	383	357.1	413.1	370.2	240	369	314	206.1	239	256.8	300.43	268.83	261.35	429.9	322.6	378.2	310.3	388.6	338	426.6	295.7	346.7	284.2	263.1	300.4	328	394	352	8
Os03g0126800	2.04	0.93	0	3.284	0.904	0.865	0	0	0	0	1.096	0	1.0885	1.8669	0.792	0	3.092	3.108	0	0.806	0	0.739	6.414	0	2.113	1.886	2.193	5.55	0.96	4.17	1
Os03g0373601	0	0	0	4.926	1.808	0.865	0	0	0.69	0	0	0	0	0	0	0	0	0	0	0	3.449	0	0	0	1.056	0	0	0	0	0	2
Os03g0296600	0	1.86	0	0.821	0	0.865	1.57	1.27	0	0	0	1.882	0	0.9335	0.792	0	0	0	1.858	0.806	0	0.739	0	2.167	1.056	0	2.193	0	2.87	0	11
Os03g0727100	93.8	99.7	97	127.3	110.3	98.62	205	183	182	156.9	171	196.6	134.98	150.29	133.84	191.1	124.7	120.2	182.1	203.2	174.2	105.7	119.7	158.9	154.2	172.5	155.7	111	128	139	15
Os03g0315400	143	178	114	47.62	35.25	62.28	31.3	19.6	39.4	37.07	18.64	8.467	14.151	7.4676	6.3357	35.82	41.22	56.98	13.01	22.58	22.42	19.96	44.18	44.06	10.56	2.829	8.772	10.2	11.5	6.66	14
Os03g0218200	67.3	89.4	67.9	107.5	108.5	112.5	14.9	25.3	35.2	22.42	20.83	11.29	28.302	36.405	28.511	95.53	43.28	53.87	49.23	57.25	60.36	108.7	79.82	60.67	36.97	26.4	26.32	64.8	42.1	55.8	2
Os03g0304500	31.6	24.2	73.3	3.284	4.52	10.38	2.35	5.06	7.6	11.21	2.193	8.467	5.4426	19.602	7.9197	83.59	37.1	56.98	10.22	2.419	0.862	5.176	3.563	1.444	6.338	7.543	10.96	16.7	12.4	3.33	13
Os03g0775500	790	739	721	605.9	642.7	600.3	899	903	939	984.6	936.3	1240	884.97	695.42	841.07	1003	690.5	666.2	563.9	558.8	676	836.2	813.8	933.9	852.5	924	975.8	632	681	656	5
Os03g0200400	1.02	0	0	0	0	0	0	0	0	0	0	0	0	0	0	0	0	0	0	0	0	0	0	0	1.056	0.943	0	0	0	0	19
Os03g0151800	923	900	930	1289	1335	1382	1338	1137	1093	1260	1160	1482	1137.5	1252.7	1053.3	1134	994.5	975.9	1707	1594	1578	776.4	908.6	954.8	1048	1033	1162	1013	1041	969	16
Os03g0819100	760	844	883	1022	1210	981.8	989	1074	983	805.3	745.5	824.2	781.56	755.16	736.53	931.4	671.9	682.7	1074	937.7	939.8	848.8	868	951.9	784.9	681.7	627.2	690	734	778	12
Os03g0725050	96.9	91.3	59.3	78.81	104.9	98.62	150	114	142	106.9	108.5	112.9	104.5	93.345	125.13	155.2	70.08	103.6	88.25	89.5	123.3	97.6	109	113.4	92.96	78.26	76.75	89.7	86.1	93.3	6
Os03g0296300	221	218	164	225.8	210.6	191.2	168	151	151	213	224.8	266.3	234.03	252.97	207.5	394.1	269	213.4	239.7	237.9	189.7	181.9	191	195.7	206	276.3	271.9	194	279	232	13
Os03g0170300	299	282	323	431.8	495.4	434.3	424	400	443	340.6	337.7	374.4	422.35	409.79	350.05	310.5	255.6	311.8	508.1	486.2	545.8	324.6	339.2	342.3	351.8	346	350.9	254	308	300	10
Os03g0103700	933	1104	1089	1014	1144	1011	715	732	639	802.7	984.6	743.2	674.88	647.82	732.57	1719	812.1	875.5	862.1	1143	914.8	1280	970.6	913.6	794.4	776.9	673.2	826	872	944	6
Os03g0195900	0	0.93	0	2.463	1.808	0	0.78	1.9	3.46	0	4.386	2.822	0	0.9335	0	23.88	0	2.072	1.858	0.806	2.587	2.218	2.851	1.444	1.056	0	0	0	0	0	6
Os03g0159400	18.4	21.4	15.1	17.24	28.02	19.03	11	10.1	22.8	28.45	23.02	24.46	23.947	33.604	20.591	23.88	19.58	10.36	37.16	45.96	27.59	22.18	12.83	17.33	30.63	10.37	21.93	20.4	23	27.5	16
Os03g0573001	0	0	0	0	0	0	0	0	0	0	0	0	0	0	0	0	0	2.072	0	0	0	0	0	0	0	0	0	0.93	0	0	1
Os03g0181675	9.18	5.59	2.16	4.105	4.52	0	0	1.9	2.76	0	1.096	0	6.5311	1.8669	1.5839	0	2.061	4.144	0	1.613	0.862	2.218	1.425	0.722	4.225	0	2.193	2.78	0	1.67	19
Os03g0636800	3.06	4.66	5.39	2.463	1.808	10.38	0.78	3.8	0.69	6.035	4.386	10.35	7.6196	2.8004	3.1679	0	11.34	4.144	9.29	4.838	2.587	3.697	6.414	5.778	10.56	11.31	8.772	3.7	5.74	3.33	20
Os03g0305700	158	139	152	206.1	186.2	202.4	138	105	176	190.5	162.3	110.1	150.22	149.35	169.48	322.4	158.7	136.8	148.6	194.3	174.2	160.4	173.9	169.7	179.6	193.3	142.5	215	216	193	6
Os03g0831200	300	254	266	221.7	173.6	249.1	307	225	282	275.9	268.6	293.5	223.15	216.56	282.73	585.1	339	253.8	276.8	275.7	281.9	275.1	323.5	322.1	310.6	313	326.7	295	307	254	6
Os03g0221600	2.04	7.45	0	0	4.52	0	1.57	2.53	8.98	1.724	2.193	0	1.0885	5.6007	1.5839	0	2.061	3.108	1.858	4.031	5.173	3.697	5.701	4.333	5.282	0.943	0	1.85	0	5	17
Os03g0334000	1.02	1.86	1.08	1.642	0	0.865	0	0.63	0	0.862	1.096	0	0	0	1.5839	0	2.061	3.108	1.858	1.613	0	5.915	5.701	0	2.113	1.886	4.386	0.93	0.96	1.67	17
Os03g0643611	0	1.86	4.31	8.21	2.712	1.73	12.5	12	11.7	0.862	2.193	1.882	3.2656	2.8004	3.1679	0	1.031	1.036	3.716	6.45	6.036	2.218	9.264	5.056	0	0.943	0	0	0	0.83	18
Os03g0686900	437	411	430	313.6	275.7	261.2	290	373	390	506.1	539.4	616.2	567.12	466.73	518.74	728.4	506	500.4	394.8	349.1	383.7	284.7	280.8	325.7	557.8	528.9	471.5	444	452	436	13
Os03g0287700	8.16	13	6.47	7.389	6.328	7.785	15.7	8.86	7.6	21.55	6.578	15.99	5.4426	14.935	3.9598	0	13.4	5.18	12.08	13.71	12.93	6.654	5.701	9.389	10.56	17.91	13.16	11.1	9.56	9.16	20
Os03g0621650	0	0	0	0	0	0	0	0	0	0	0	0	0	0	0	0	1.031	0	0	0	0	0	0	0	0	0	0	0	0	0	1
Os03g0599600	449	373	454	619	567.7	618.5	191	216	203	434.5	497.8	341.5	636.78	548.87	633.57	549.3	352.4	396.8	564.8	566	526.8	250.7	265.8	215.2	470.1	385.6	429.8	574	469	538	16
Os03g0134900	0	0	0	0	0	0	0	0	0	0.862	0	0	0	0	0	0	0	0	0	0	0	0	0	0	0	0	0	0	0	0	7
Os03g0109000	0	0.93	0	1.642	0.904	0	0	0	0	0	0	0	1.0885	1.8669	0	0	0	1.036	0	0	0	0	0	0	0	0	0	0	0	0	4
Os03g0786100	9512	####	####	11005	11778	11004	####	####	####	16230	17221	17880	16508	13199	12891	11320	11026	11078	13639	13478	13889	15214	15832	20897	16187	15873	13574	8509	####	####	18
Os03g0429000	260	155	248	104.3	57.85	141	293	376	357	328.5	193	143	124.09	156.82	147.31	203	140.2	164.7	73.39	98.37	81.91	286.9	307.1	246.3	119.4	141.4	133.8	104	108	73.3	18
Os03g0187000	268	262	242	471.2	499.9	543.3	301	257	323	292.3	197.4	329.3	326.56	361.25	257.39	370.2	218.5	283.9	492.3	478.9	474.2	299.5	230.2	249.2	180.6	301.7	263.2	199	239	281	10
Os03g0268900	697	715	694	1221	1206	1165	1017	975	1068	676.8	828.9	692.4	982.93	877.44	727.02	704.5	568.9	654.8	1277	1359	1249	706.1	695.5	855.9	599	572.3	436.4	492	576	602	10
Os03g0823200	205	180	207	194.6	181.7	171.3	95.5	120	168	116.4	151.3	128.9	187.23	122.28	152.85	310.5	152.5	167.8	92.9	106.4	86.22	257.3	230.9	156.7	190.1	151.8	149.1	157	172	157	8
Os03g0745550	258	198	238	149.4	151	154.8	277	439	284	162.1	185.3	206	271.04	233.36	237.59	406	256.6	173	245.2	159.6	233.7	190.8	176	234.7	177.5	177.3	162.3	184	188	187	6
Os03g0830500	0	1.86	1.08	3.284	0	0	0	2.53	6.91	0	0	0	0	0	0	0	0	0	0	0	0	0	0	0	0	0	0	0	0	0	18
Os03g0734500	41.8	67.1	35.6	8.21	0	3.46	1.57	1.27	4.84	9.484	1.096	0	8.7082	1.8669	0	59.7	75.23	64.23	3.716	4.838	25	11.83	11.4	72.22	1.056	5.657	6.579	0	6.69	2.5	14
Os03g0160000	102	108	103	93.59	106.7	109.9	116	106	118	98.29	84.42	101.6	69.665	79.343	79.989	107.5	120.6	180.3	91.97	103.2	138	181.1	144	124.9	112	124.5	100.9	109	77.5	75.8	9
Os03g0643800	0	0	0	0	0	0	0	0	0	0	0	0	0	0	0	0	0	0	0.929	0	0	0	0	0	0	0	0	0.93	0	0	16
Os03g0705433	1.02	0	0	0	0	0	1.57	0	0	0	0	0	0	0	0	0	0	0	0	0	0	0	0.713	0.722	0	0	2.193	0	0	0	11
Os03g0229600	1797	1827	1930	2628	2734	2727	2163	2326	2003	1614	1745	1863	2469.9	2172.1	1986.3	4048	2257	2145	2795	3020	2906	1993	1875	1813	1953	1984	1912	2310	2325	2362	6
Os03g0670700	####	####	####	50841	51498	51047	####	####	####	12572	12321	11992	9644.3	7618.8	8051.1	16586	9702	9716	47150	49638	50591	38379	35963	45220	8365	7497	7673	5617	6008	5755	12
Os03g0803100	1.02	0	1.08	0.821	0	1.73	0	0	3.46	9.484	2.193	0.941	2.177	2.8004	10.296	11.94	1.031	5.18	0	0	0	0	0	0	5.282	2.829	13.16	3.7	2.87	6.66	13
Os03g0657400	7.14	5.59	8.62	30.38	23.5	36.33	39.1	20.9	44.2	38.8	19.74	13.17	9.7967	24.27	17.423	0	8.244	3.108	28.8	58.86	30.18	25.88	38.48	21.67	11.62	15.09	15.35	0.93	3.83	10.8	12
Os03g0833900	633	787	676	820.2	823.5	771.6	1215	975	1136	795.8	778.4	760.2	698.83	747.69	660.5	692.6	597.7	670.3	863	705.5	710.5	931.6	949.2	1047	650.7	629.8	796	642	538	582	18
Os03g0194400	176	146	182	169.1	137.4	128	298	252	269	261.2	216	239	223.15	200.69	219.37	226.9	197.9	182.3	201.6	171.7	191.4	241	242.3	247.7	218.7	253.6	162.3	181	215	181	5
Os03g0852600	188	200	172	197.9	245	208.5	229	221	272	129.3	134.9	146.8	186.14	168.02	156.81	214.9	148.4	188.6	225.7	204	200.9	271.4	237.3	238.3	191.2	149.9	100.9	187	168	192	12
Os03g0140400	3.06	4.66	6.47	2.463	1.808	6.92	3.91	4.43	2.07	5.173	7.675	6.586	2.177	2.8004	0.792	0	2.061	5.18	1.858	5.644	3.449	2.958	0.713	2.889	6.338	9.428	8.772	3.7	4.78	4.17	20
Os03g0793800	3.06	16.8	16.2	4.105	1.808	6.92	0.78	0.63	0	0	1.096	0	5.4426	4.6673	3.1679	0	1.031	0	5.574	4.031	0.862	2.218	0.713	2.889	1.056	0	2.193	0.93	0.96	2.5	14
Os03g0424800	2308	1907	1897	2201	2384	2560	2771	2181	3032	2569	2298	3917	2611.4	2864.8	1875.4	3129	1875	2132	3175	2883	3051	2098	2118	2381	2329	2662	3171	1678	1952	1458	15
Os03g0109650	0	0	0	0	0	0	0	0	0	0	0	0	0	0	0	0	0	0	0	0	0	0	0	0	0	0	0	0	0	1.67	1
Os03g0328100	21.4	26.1	23.7	24.63	15.37	23.36	40.7	32.9	25.6	23.28	26.31	24.46	7.6196	21.469	6.3357	47.76	14.43	23.83	18.58	23.38	19.83	31.05	14.25	31.06	10.56	21.69	8.772	6.48	12.4	15.8	6
Os03g0195475	358	298	289	148.6	113.9	153.1	78.3	56.9	78.1	171.6	200.6	247.4	79.462	112.95	114.04	214.9	246.3	200	101.3	68.53	103.5	193.7	97.63	247.7	181.7	185.7	217.1	113	128	135	14
Os03g0219700	981	998	1010	1883	2047	1861	1888	2065	1774	1070	961.5	1251	1289.9	1464.6	1188.7	2890	1250	1260	1902	1746	2067	1289	1485	1415	1156	1181	1215	1168	1177	1147	6
Os03g0206300	284	250	320	159.3	164.5	168.7	116	136	138	223.3	282.9	303.9	374.45	348.18	376.18	644.8	294.7	361.6	203.4	211.2	170.7	148.6	134	114.8	292.6	285.7	243.4	318	334	373	13
Os03g0385400	542	420	554	821	956.4	812.3	1185	968	1119	627.7	708.3	894.7	870.82	729.03	692.97	1099	550.3	493.2	875.1	872.4	1045	542	558	781.5	604.2	662.8	721.5	508	638	517	15
Os03g0821700	7.14	22.4	10.8	55.83	71.41	66.61	29	45.5	38	16.38	16.45	7.527	10.885	8.4011	7.1277	23.88	7.214	17.61	45.52	54.83	38.8	35.49	34.21	33.22	10.56	15.09	8.772	19.4	18.2	11.7	10
Os03g0816200	158	152	178	120.7	153.7	149.7	92.4	71.5	92.6	129.3	174.3	208.9	171.99	230.56	159.98	262.7	192.7	222.7	161.6	158	175.9	181.9	151.1	127.1	165.8	277.2	243.4	238	260	200	13
Os03g0581701	99.9	61.5	105	54.18	50.62	59.69	59.5	50.6	56.7	166.4	133.8	130.8	180.69	150.29	146.51	143.3	99.96	112.9	101.3	117.7	81.05	50.28	62	38.28	146.8	146.1	151.3	106	99.5	100	3
Os03g0266800	876	851	981	948.2	786.5	968.9	880	906	837	549.2	507.6	564.5	766.32	720.62	616.15	1516	731.7	684.8	971.7	809.5	730.3	564.2	642.8	845	485.9	465.8	559.2	683	820	696	6
Os03g0347400	22.4	25.1	23.7	29.56	18.08	28.55	11.7	7.59	3.46	16.38	10.96	9.408	16.328	14.002	10.296	11.94	10.31	24.86	8.361	10.48	4.311	13.31	24.23	11.56	13.73	18.86	10.96	28.7	24.9	31.7	8
Os03g0707200	1492	1272	1587	564	496.3	613.3	564	643	820	1229	1571	1294	1864.6	1768.9	2071	3260	1717	1480	772	766	734.6	640.3	666.3	621.1	1823	1795	1476	1783	1794	1915	13
Os03g0294900	4.08	7.45	5.39	13.14	17.18	21.63	3.13	4.43	0	1.724	5.482	0.941	3.2656	0.9335	2.3759	0	6.183	5.18	2.787	10.48	7.76	19.96	9.977	5.778	3.169	1.886	0	7.4	4.78	6.66	2
Os03g0225100	275	232	232	213.5	180.8	200.7	186	118	236	397.5	301.5	396.1	226.41	222.16	230.46	238.8	243.2	267.3	293.5	295.9	303.5	250.7	188.1	281	230.3	218.7	315.8	192	194	276	20
Os03g0286500	0	0.93	1.08	0	0	0	1.57	1.27	0.69	0	1.096	0	0	0	1.5839	0	2.061	1.036	0.929	0.806	0	1.479	2.138	4.333	0	0	8.772	0.93	1.91	0	11
Os03g0179100	1.02	4.66	4.31	15.6	9.04	12.11	11.7	5.06	5.53	0.862	13.16	11.29	3.2656	3.7338	2.3759	11.94	3.092	4.144	25.08	16.13	29.32	11.09	12.11	9.389	5.282	1.886	4.386	9.25	13.4	11.7	10
Os03g0726900	81.6	102	90.5	135.5	138.3	122.8	168	137	160	94.84	115.1	86.56	112.12	124.15	131.47	226.9	140.2	115	144.9	145.1	172.4	150.8	119.7	122.8	140.5	97.11	76.75	130	128	120	6
Os03g0748300	122	200	137	2148	2217	1867	3507	3280	3428	328.5	378.3	480.8	309.14	171.76	157.6	191.1	143.2	165.8	2601	2483	2937	1467	1623	2032	195.4	190.5	157.9	90.7	95.6	122	12
Os03g0177300	464	352	475	312	307.3	315.7	382	403	337	411.3	460.5	431.8	559.5	471.39	476.76	644.8	511.1	481.8	333.5	402.3	319.9	298.7	354.9	335.1	452.1	408.3	429.8	386	415	390	13
Os03g0725400	31.6	20.5	15.1	137.1	135.6	134.1	28.2	20.9	44.2	19.83	16.45	23.52	20.682	33.604	16.631	23.88	15.46	27.97	126.3	154	167.3	21.44	26.37	15.89	5.282	7.543	8.772	13.9	14.3	15.8	10
Os03g0305200	27.5	31.7	39.9	3.284	1.808	6.055	44.6	25.9	26.9	26.73	14.25	20.7	29.39	16.802	17.423	0	11.34	14.5	4.645	4.838	4.311	50.28	32.07	49.83	12.68	12.26	6.579	3.7	3.83	9.16	9
Os03g0747700	309	412	363	485.2	556.8	436	812	813	865	319	463.8	474.2	372.27	270.7	314.41	513.5	346.3	390.6	775.7	804.7	677.7	528.7	500.3	721.5	284.2	195.2	184.2	256	312	337	12
Os03g0843800	5005	6323	4958	1102	885.9	1454	159	321	169	6351	7894	6006	4383.5	8321.7	6941.6	11057	6011	6369	888.1	1706	1371	652.1	863.7	868.9	10781	9229	6550	####	6365	8582	3
Os03g0796600	242	370	267	220	203.4	255.2	389	478	333	342.3	426.5	436.5	369.01	375.25	384.1	788.1	453.4	366.8	198.8	316.9	238.8	478.4	543.7	356.8	551.4	538.4	576.7	640	623	548	13
Os03g0754500	14.3	8.38	7.54	18.06	11.75	29.41	3.13	2.53	8.29	10.35	7.675	0	10.885	2.8004	5.5438	0	16.49	5.18	9.29	12.09	14.66	11.83	37.06	27.45	1.056	11.31	35.09	18.5	30.6	2.5	11
Os03g0788500	53	43.8	48.5	9.031	9.944	15.57	19.6	6.96	19.3	24.14	15.35	16.93	11.974	14.002	11.879	0	29.89	35.23	11.15	10.48	10.35	56.93	41.33	67.89	21.13	31.11	15.35	21.3	22	21.7	9
Os03g0824350	258	223	245	243.8	217	209.3	172	203	205	220.7	235.7	202.3	237.3	222.16	234.42	465.7	266.9	306.7	213.7	226.6	229.3	309.1	244.4	237.6	246.1	276.3	236.8	308	254	281	13
Os03g0586500	4354	4073	5113	35723	38453	37039	####	####	####	5753	5274	5475	7921.2	7913.8	7347.1	8538	6764	5503	44365	42335	49467	7009	8197	8013	5684	5331	4607	5996	5854	6889	10
Os03g0363800	85.6	74.5	89.4	137.1	153.7	128	68.9	82.2	82.2	84.49	96.48	109.1	115.38	112.95	86.324	226.9	127.8	101.5	139.3	139.5	125	79.11	54.16	78	97.19	111.3	114	98.1	87	109	6
Os03g0191800	0	0	0	0	0	0	0	0	0	0	0	0	0	0	0	0	0	0	0	0	0	0	0	0	1.056	0	0	0	0	0	1
Os03g0355700	11.2	18.6	11.9	13.96	14.46	10.38	27.4	28.5	29	6.035	21.93	15.99	9.7967	4.6673	9.5036	0	7.214	19.68	22.29	22.58	13.8	21.44	15.68	23.83	7.395	19.8	26.32	12	12.4	15	18
Os03g0804400	1685	1505	1844	1043	1063	1183	912	890	832	1293	1340	1149	1971.3	1885.6	2055.9	3451	1589	1559	1022	1214	1134	973.8	942.8	833.5	1473	1308	1210	1874	1812	1818	13
Os03g0237600	568	668	579	729.8	861.5	729.2	561	620	439	462.1	547.1	477.9	571.47	630.08	563.88	823.9	497.7	612.3	635.4	639.4	699.3	512.4	533.8	585.7	543	455.4	554.8	566	606	655	10
Os03g0786400	413	631	550	515.6	451.1	548.4	689	556	684	906.2	1082	1160	569.3	491.93	555.17	919.5	507	477.6	721.8	588.6	716.5	331.2	325	573.5	641.2	870.2	881.6	380	462	388	20
Os03g0729700	112	76.4	116	282.4	342.6	286.3	207	231	205	76.73	97.58	91.26	169.81	111.08	87.116	226.9	95.84	120.2	276.8	312	234.5	161.2	159.6	175.5	73.95	63.17	46.05	49	88.9	90	10
Os03g0795600	5.1	5.59	2.16	2.463	3.616	9.516	0	1.27	3.46	0.862	0	0	2.177	0.9335	6.3357	0	8.244	7.252	0.929	1.613	1.724	1.479	2.851	5.778	4.225	0	13.16	1.85	2.87	1.67	11
Os03g0681400	843	806	793	779.9	766.6	804.5	792	723	762	945.8	917.7	1040	933.95	896.11	903.63	1027	902.7	882.7	1001	903	893.3	751.2	669.9	780	886.3	957	896.9	835	862	866	20
Os03g0407400	0	0	0	0	0	0	0	0	0	0.862	0	0	0	0	0	0	2.061	0	0	0.806	0.862	0	0	0	0	0	0	0	0	0.83	16
Os03g0141200	7316	6109	6603	532.8	578.5	700.7	1364	1596	1343	4223	4326	3887	6325.4	4281.7	5215.1	6436	4783	4784	775.7	895	759.6	997.4	1106	1145	4142	3294	3112	3787	3556	4086	3
Os03g0791432	31.6	41	20.5	16.42	15.37	21.63	67.3	60.7	60.1	37.94	41.66	48.92	30.479	30.804	33.263	23.88	37.1	33.15	30.66	30.64	48.28	28.84	32.07	44.78	28.52	39.6	35.09	25.9	26.8	20.8	5
Os03g0821275	16.3	17.7	11.9	32.84	54.24	37.2	45.4	54.4	57.4	20.69	18.64	26.34	26.124	18.669	16.631	35.82	21.64	17.61	40.87	51.6	54.32	36.97	47.75	44.06	25.35	28.29	0	18.5	21	16.7	12
Os03g0752500	25.5	59.6	26.9	0	0	1.73	18.8	13.3	17.3	18.11	70.17	47.04	69.665	59.741	39.598	11.94	90.69	27.97	0.929	0	0	3.697	11.4	41.17	66.55	174.4	120.6	62.9	168	143	3
Os03g0229575	304	297	328	243.8	258.5	252.6	193	219	173	260.4	271.9	208.9	231.85	245.5	228.88	597	298.9	272.5	267.5	280.6	242.3	184.1	201.7	188.5	247.2	255.5	210.5	269	318	301	13
Os03g0440900	3483	3260	3529	2662	2563	2352	4919	3932	4925	5124	3991	4869	3242.7	3628.3	3815.7	4764	2740	2953	3387	2899	2638	4767	4803	5492	3654	3425	4283	2646	2624	2371	5
Os03g0236200	93.8	104	50.6	40.23	25.31	29.41	16.4	17.7	27.6	64.66	42.76	48.92	17.416	16.802	65.733	35.82	87.6	49.73	26.01	54.02	18.97	43.62	33.49	33.95	9.507	16.03	28.51	17.6	23	8.33	14
Os03g0269000	888	823	930	713.4	838	792.4	691	641	641	707	679.8	871.2	912.18	887.71	852.95	1051	888.3	844.4	791.5	766	732	681.7	620	711.4	787	830.6	701.7	769	776	803	13
Os03g0231800	937	657	722	448.3	365.2	412.6	219	299	263	941.5	1091	1013	806.59	1052.9	960.65	656.8	883.2	672.4	685.6	749	688.9	294.3	417.6	472.3	1098	1298	1333	1046	954	835	3
Os03g0432100	3.06	5.59	4.31	4.926	5.424	2.595	6.26	4.43	4.84	0	0	1.882	0	5.6007	0	11.94	3.092	3.108	3.716	2.419	6.036	28.84	4.276	3.611	2.113	4.714	4.386	1.85	1.91	3.33	9
Os03g0115700	0	0	0	0.821	0.904	2.595	0.78	2.53	0	3.449	0	0	0	0	0	0	6.183	4.144	0	0	0.862	1.479	0	2.889	1.056	0.943	4.386	4.63	1.91	0.83	1
Os03g0131950	0	0.93	0	0	0	3.46	0	3.16	0	2.587	1.096	2.822	1.0885	0	0	0	3.092	3.108	0	0	0.862	0	0	0	0	1.886	0	4.63	1.91	0.83	1
Os03g0767600	2.04	2.79	1.08	3.284	6.328	11.25	1.57	3.16	2.07	0	1.096	0	1.0885	4.6673	0	0	2.061	3.108	1.858	6.45	0.862	9.612	7.839	3.611	2.113	5.657	0	2.78	1.91	1.67	2
Os03g0839900	1003	715	800	708.5	577.6	665.2	485	528	786	546.6	598.6	421.5	682.5	702.89	776.92	1708	797.6	653.7	689.3	830.5	843.2	1253	1292	1015	756.4	860.8	984.6	1067	1106	918	6
Os03g0402400	342	305	296	470.4	505.3	519.9	557	529	527	290.6	278.5	382.9	248.18	331.38	194.03	322.4	240.1	258	479.3	330.6	448.4	367.5	405.5	374.8	179.6	266.8	311.4	201	226	193	12
Os03g0265800	257	216	245	121.5	124.7	147.1	58.7	62	68.4	185.4	160.1	203.2	268.86	252.97	163.14	238.8	342.1	233.1	188.6	175	196.6	48.8	64.14	78.72	183.8	228.2	122.8	227	196	203	3
Os03g0109400	90.7	130	100	112.5	90.4	107.3	215	163	155	143.1	148	190	111.03	137.22	127.51	71.65	134	104.6	118	137.1	127.6	142.7	124.7	157.4	136.3	161.2	193	144	144	130	5
Os03g0168000	2.04	0.93	1.08	2.463	3.616	1.73	3.91	5.06	0	0	1.096	0	0	0	0	0	0	1.036	0	2.419	1.724	4.436	13.54	3.611	2.113	0.943	0	1.85	0	0	17
Os03g0164100	525	532	504	394.1	376.1	392.7	361	378	356	363	423.2	329.3	306.96	335.11	460.92	776.2	559.6	527.3	345.6	396.7	349.2	520.5	441.1	389.3	428.9	397.9	443	514	548	555	13
Os03g0170600	1.02	1.86	1.08	2.463	0	6.92	0	0	0	0	0	3.763	0	4.6673	0	0	5.153	2.072	0.929	0	0	0	0	0	0	1.886	4.386	0	0	0	11
Os03g0189300	21.4	16.8	16.2	4.105	3.616	4.325	17.2	6.96	2.07	11.21	9.868	27.28	1.0885	0.9335	6.3357	11.94	21.64	9.324	6.503	0	2.587	8.873	9.264	6.5	3.169	5.657	6.579	7.4	5.74	3.33	14
Os03g0641200	17.3	21.4	19.4	13.14	17.18	9.516	21.1	37.3	21.4	22.42	17.54	13.17	8.7082	25.203	30.095	11.94	21.64	18.65	11.15	17.74	22.42	56.19	62	71.5	28.52	28.29	32.89	49	23	19.2	9

Os03g0183200	70.3	61.5	64.7	18.88	11.75	17.3	456	235	799	88.81	70.17	103.5	87.082	83.077	101.37	71.65	61.83	51.8	11.15	12.09	14.66	919.1	1023	871	59.16	53.74	67.98	57.4	59.3	55.8	17
Os03g0292900	37.7	47.5	25.9	156	141.9	153.1	35.2	49.3	21.4	31.9	29.6	30.11	45.718	43.872	29.303	47.76	25.76	49.73	107.8	133.8	102.6	26.62	34.21	15.17	25.35	28.29	50.44	32.4	38.3	30.8	10
Os03g0268000	738	884	828	620.7	637.3	569.2	462	579	557	582.8	711.6	507.1	813.12	597.41	825.23	716.5	646.1	748	366	374.1	379.4	692.8	668.5	615.4	684.5	577	502.2	670	635	820	8
Os03g0296800	945	1065	1043	1132	1087	999.1	913	980	865	1377	1412	1164	1583.8	1501	1625.1	2926	1395	1153	1084	1258	1406	633.7	815.3	622.6	1673	1248	1059	1653	1609	1567	13
Os03g0334200	148	89.4	91.6	39.41	35.25	21.63	68.9	99.9	59.4	35.35	26.31	31.05	21.77	38.272	27.719	23.88	63.89	52.84	38.09	35.48	40.52	66.54	71.98	91	19.01	32.06	46.05	17.6	29.6	18.3	19
Os03g0787600	116	159	177	399.8	369.7	431.7	426	408	397	231.1	197.4	346.2	332	284.7	278.77	274.6	194.8	252.8	609.4	505.5	627.7	219.6	216.6	361.1	189.1	176.3	144.7	160	189	210	10
Os03g0169000	249	248	236	218.4	226	183.4	173	192	202	219	294.9	204.2	243.83	282.84	228.09	358.2	257.6	254.9	241.5	227.4	243.1	178.2	195.3	205.8	264.1	276.3	256.6	236	278	271	13
Os03g0111800	291	343	244	587.8	547.8	584.8	278	242	254	213	218.2	189.1	237.3	273.5	222.54	489.6	271	296.3	476.6	545.9	461.3	339.4	361.3	213.1	187	244.2	193	349	260	297	10
Os03g0703400	1996	1951	2141	671.6	676.2	696.4	828	901	908	1120	1093	879.7	1141.9	1044.5	1161	1970	1229	1443	530.4	591.8	506.1	1128	1071	1000	1071	1060	1079	1126	971	1171	14
Os03g0835300	0	4.66	5.39	3.284	4.52	4.325	4.7	7.59	12.4	5.173	2.193	2.822	7.6196	2.8004	1.5839	11.94	3.092	7.252	7.432	4.838	6.898	3.697	3.563	5.056	6.338	3.771	2.193	3.7	7.65	1.67	6
Os03g0628800	57.1	68.9	43.1	154.3	190.7	154	211	151	252	36.21	41.66	37.63	28.302	41.072	30.887	23.88	62.86	24.86	84.53	95.14	120.7	102	66.28	167.6	41.2	49.97	21.93	34.2	18.2	20.8	12
Os03g0775400	43.8	49.4	31.2	206.1	207	188.6	153	149	143	53.46	42.76	36.69	56.603	56.007	49.102	131.4	37.1	45.59	139.3	151.6	151.8	108.7	106.2	91.73	34.86	39.6	35.09	33.3	54.5	33.3	12
Os03g0831700	25.5	31.7	29.1	30.38	9.04	22.49	27.4	22.8	20.7	30.18	57.01	27.28	25.036	16.802	22.175	47.76	15.46	14.5	22.29	20.16	13.8	27.36	28.51	41.17	22.18	49.97	24.12	20.4	34.4	18.3	7
Os03g0588100	4.08	3.73	3.23	0.821	1.808	3.46	0	1.9	1.38	15.52	1.096	7.527	3.2656	13.068	15.047	35.82	12.37	9.324	1.858	8.869	6.036	0.739	0	4.333	14.79	6.6	15.35	12	8.61	19.2	13
Os03g0676866	0	0	0	0.821	0.904	0.865	0	0	0	0	0	0	0	0	0	0	0	0	1.858	0	0	0	0	0	0	0	0	0	0	0	10
Os03g0421800	39.8	14.9	22.6	17.24	14.46	18.17	21.9	22.1	18	27.59	17.54	38.57	19.593	21.469	11.088	11.94	16.49	13.47	22.29	9.675	15.52	14.79	14.97	21.67	20.07	28.29	21.93	20.4	25.8	12.5	20
Os03g0844700	325	292	274	523	465.5	538.1	342	491	442	383.7	354.1	277.5	396.22	357.51	394.4	406	262.8	284.9	434.8	357.2	391.4	449.5	403.4	399.4	294.7	276.3	307	273	312	315	12
Os03g0722800	324	333	350	289.8	286.6	324.4	258	266	261	295.7	234.6	223.9	284.1	338.84	336.59	847.8	391.6	324.3	321.4	268.5	312.1	337.9	305.7	269.4	294.7	238.5	232.4	385	342	347	6
Os03g0285166	11.2	16.8	19.4	14.78	6.328	12.98	3.91	9.49	4.84	17.24	14.25	35.75	11.974	23.336	14.255	23.88	16.49	18.65	11.15	8.869	14.66	4.436	1.425	5.778	19.01	16.97	21.93	9.25	22	15	3
Os03g0823301	6.12	3.73	8.62	20.52	13.56	9.516	1.57	0.63	10.4	0.862	4.386	4.704	2.177	0	0.792	0	7.214	6.216	7.432	2.419	0.862	17.01	19.24	7.222	8.451	4.714	0	4.63	4.78	8.33	17
Os03g0160600	48.9	74.5	47.4	27.91	30.73	32.87	40.7	36.7	23.5	58.63	51.53	35.75	28.302	42.939	58.605	47.76	40.19	52.84	2.787	24.19	26.73	34.75	17.1	23.83	50.71	31.11	28.51	37	28.7	39.2	14
Os03g0164800	13.3	6.52	10.8	49.26	48.81	69.2	38.4	40.5	50.4	1.724	2.193	7.527	1.0885	2.8004	5.5438	0	8.244	4.144	42.73	67.73	41.39	54.71	61.29	41.17	1.056	4.714	10.96	7.4	7.65	4.17	12
Os03g0288300	1087	964	985	508.2	562.3	524.2	808	673	797	883.7	817.9	925.8	849.05	797.17	860.08	1302	1024	927.3	594.5	565.2	513	592.2	579.4	665.2	1104	990	1039	751	741	730	13
Os03g0710500	0	0	0	0	0.904	0	0	0	0	0.862	0	0	0	0	2.3759	0	0	0	0	0	0	0	0	0	0	0	0	1.85	0	0	4
Os03g0232900	259	296	341	221.7	223.3	199	256	196	186	195.7	189.7	194.7	179.61	172.69	193.24	465.7	226.7	254.9	211.8	266.1	240.6	237.3	240.2	198.6	178.5	188.6	217.1	267	310	234	6
Os03g0122300	58.1	55.9	64.7	64.86	52.43	97.75	184	131	185	157.8	87.71	79.97	79.462	90.545	59.397	11.94	38.13	55.95	77.1	20.96	29.32	115.3	198.8	121.3	64.44	50.91	118.4	122	170	80.8	5
Os03g0558700	22.4	20.5	19.4	4.926	14.46	8.65	19.6	20.9	20	29.31	49.34	39.51	23.947	13.068	18.215	11.94	21.64	18.65	6.503	12.9	4.311	16.27	33.49	20.95	33.8	18.86	48.24	19.4	18.2	19.2	20
Os03g0289100	174	142	180	209.3	186.2	150.5	141	115	107	174.2	151.3	123.2	244.92	236.16	223.33	238.8	128.8	162.7	222.9	212.9	110.4	131.6	118.3	83.06	162.7	104.7	144.7	175	153	177	4
Os03g0607400	98.9	56.8	91.6	2.463	1.808	7.785	9.39	1.27	3.46	75.87	109.6	70.56	120.83	135.35	152.85	191.1	120.6	123.3	7.432	8.869	16.38	6.654	1.425	3.611	99.3	153.7	111.8	182	122	133	3
Os03g0320100	170	170	143	200.3	203.4	154.8	204	239	232	130.2	133.8	102.5	111.03	164.29	169.48	191.1	92.75	96.35	111.5	103.2	118.1	354.9	355.6	328.6	154.2	132.9	116.2	106	114	93.3	17
Os03g0367000	954	1028	1081	905.5	929.3	914.4	1226	1280	1220	1270	1122	1122	957.9	1050.1	1151.5	1815	851.2	942.8	920.6	921.6	923.4	1091	1124	1035	1049	1001	1031	1091	973	970	6
Os03g0200200	10.2	16.8	7.54	30.38	53.33	32.01	20.4	26.6	15.2	50.87	28.51	13.17	11.974	19.602	7.9197	0	0	5.18	13.93	7.257	8.622	27.36	51.31	33.22	14.79	8.486	15.35	14.8	14.3	14.2	17
Os03g0566500	3.06	0.93	4.31	0	2.712	1.73	0	1.27	0	1.724	0	4.704	0	3.7338	1.5839	11.94	0	5.18	0	1.613	0	0	0	0	1.056	3.771	0	0.93	2.87	4.17	13
Os03g0660050	23.4	39.1	22.6	91.95	96.72	85.64	91.6	139	130	15.52	29.6	17.88	35.921	29.87	49.894	107.5	32.98	63.2	48.31	62.89	58.63	168.6	134.7	110.5	39.09	40.54	10.96	46.3	70.8	38.3	18
Os03g0102100	1091	1190	1184	1680	1717	1626	1264	1334	1438	959.6	1129	1061	1509.8	1255.5	1144.4	1708	1205	1356	1581	1847	1641	1463	1285	1294	1236	980.6	826.7	1339	1266	1505	10
Os03g0794300	31.6	61.5	26.9	28.73	28.93	34.6	64.2	55.7	70.5	75.87	81.13	73.38	64.223	62.541	60.981	35.82	47.4	62.16	29.73	28.22	24.14	42.15	36.34	52.72	53.87	63.17	70.17	47.2	41.1	61.7	5
Os03g0363900	527	505	505	660.9	684.3	617.6	700	723	715	613	527.4	595.5	572.56	543.27	548.83	1337	542.1	466.2	695.8	694.2	609.6	717.2	647.8	631.2	632.8	495	513.1	573	501	547	6
Os03g0335300	3104	3392	3167	4275	3516	4925	2522	2634	3061	3539	3265	3003	3027.2	3151.3	2989.7	3702	2745	2831	4299	4094	4181	3206	3275	2942	3285	3111	3570	2962	3070	3011	10
Os03g0305800	6.12	4.66	3.23	4.926	2.712	3.46	4.7	3.16	3.46	5.173	12.06	4.704	8.7082	2.8004	3.9598	0	5.153	2.072	1.858	4.838	1.724	3.697	3.563	9.389	5.282	3.771	2.193	2.78	0.96	3.33	7
Os03g0836000	18.4	9.31	18.3	13.14	21.7	6.92	7.05	6.96	4.15	2.587	8.771	2.822	7.6196	7.4676	5.5438	0	5.153	11.4	8.361	6.45	4.311	7.394	4.988	12.28	5.282	2.829	0	5.55	4.78	6.66	2
Os03g0184000	3546	3126	3614	4099	4424	4197	3882	4352	3707	3242	3471	3482	3940.4	4216.4	3868.8	7212	4256	3717	4558	4535	4634	3338	3663	3644	3648	3684	3204	3707	3914	3744	6
Os03g0409100	53	136	66.8	1171	1466	1315	1747	1807	1640	140.5	166.7	138.3	164.37	141.88	183.74	71.65	87.6	81.85	881.6	803.1	893.3	1249	1191	1317	92.96	82.03	89.91	83.3	66	89.1	12
Os03g0146500	621	852	828	435.1	434.8	429.1	1304	1525	1313	999.3	1424	1036	1157.1	956.79	1191.1	1373	1022	867.2	459.8	450.7	376.8	828.1	971.3	1042	1177	1191	1145	860	948	1100	5
Os03g0106200	6.12	7.45	9.7	33.66	30.73	30.28	8.61	21.5	4.84	4.311	2.193	0.941	5.4426	0.9335	3.9598	0	1.031	6.216	7.432	16.13	20.69	29.58	24.23	25.28	4.225	10.37	0	13.9	14.3	5.83	2
Os03g0320900	428	359	421	670.7	761.1	666.1	247	254	269	302.6	299.3	421.5	476.77	497.53	430.04	609	396.8	464.1	774.7	891.7	760.5	235.1	220.2	247	252.5	418.6	432	360	352	403	16
Os03g0778100	####	####	####	10628	9062	8861	####	####	####	19690	20129	32023	16260	14476	14845	20861	14552	15147	17604	14524	18804	6164	5712	9584	18790	20589	17947	####	####	####	20
Os03g0347900	34.7	58.7	10.8	8.21	7.232	16.44	5.48	19.6	7.6	47.42	8.771	36.69	6.5311	11.201	4.7518	0	6.183	10.36	11.15	9.675	13.8	11.09	15.68	13	12.68	7.543	0	1.85	6.69	10	14
Os03g0565100	583	508	485	553.3	604.8	633.2	657	543	637	607	600.8	648.2	531.2	594.61	499.73	895.6	473	501.4	680	662	619.1	538.3	606.5	538.1	550.4	526.1	616.2	467	517	477	6
Os03g0172000	579	582	515	880.1	989.8	846	701	702	643	463.9	473.6	445.9	641.14	593.68	521.91	835.9	451.4	439.3	611.3	707.1	723.4	692.8	679.9	645	442.6	492.2	434.2	502	450	460	10
Os03g0723400	3.06	1.86	2.16	0	0	0.865	0	0	0	4.311	0	0.941	1.0885	0	0.792	59.7	26.79	13.47	0	0	0.862	0	0	0	2.113	0	0	10.2	0.96	1.67	6
Os03g0425000	725	972	897	2001	2053	1687	1442	1669	1396	790.6	1010	960.6	1114.6	677.69	685.05	1087	571.9	771.8	1290	1588	1586	1360	1251	1517	710.9	648.7	410.1	388	521	667	12
Os03g0806900	518	551	603	792.2	857.9	891.9	908	727	931	552.7	474.7	623.8	457.18	557.27	460.92	406	516.3	462.1	945.7	832.9	927.7	655.8	683.4	753.3	472.2	479	563.6	356	459	416	12
Os03g0584050	265	253	302	275	303.7	360.7	176	196	189	195.7	240.1	183.5	259.07	238.96	231.25	358.2	290.6	294.2	241.5	275.7	247.5	332	268	222.5	296.8	293.2	217.1	319	406	388	8
Os03g0596400	0	0	0	1.642	0	0	2.35	9.49	0	0	0	0	0	0	0	0	0	0	0	0	0	0.739	0	0.722	0	0	0	0	0	0	18
Os03g0217000	186	299	212	611.6	658.1	590.8	590	605	759	295.7	288.4	217.3	296.08	238.03	305.7	358.2	188.6	204.1	605.7	639.4	688	458.4	481	535.2	312.7	171.6	186.4	155	202	186	12
Os03g0359100	641	587	547	506.5	516.2	425.6	711	607	497	589.7	562.5	702.8	586.71	626.35	540.91	704.5	526.6	504.5	520.2	470.1	488.9	486.5	521.6	569.9	529.2	537.4	730.2	531	532	469	20
Os03g0702600	9.18	8.38	18.3	10.67	16.27	4.325	18	20.9	18.7	28.45	13.16	10.35	6.5311	15.869	7.9197	11.94	6.183	7.252	11.15	11.29	9.484	14.05	17.1	10.83	14.79	9.428	4.386	18.5	10.5	5	7
Os03g0417900	434	579	469	552.5	568.6	521.6	552	574	587	416.4	398	434.7	350.5	444.32	438.75	752.3	417.4	480.7	438.5	422.5	472.5	587.1	549.4	536.6	402.5	399.8	460.5	501	430	489	6
Os03g0769400	13.3	23.3	20.5	46.8	69.61	39.79	11.7	17.1	15.2	12.07	19.74	4.704	27.213	28.937	25.343	47.76	22.67	29.01	14.86	15.32	14.66	27.36	36.34	14.44	16.9	11.31	6.579	27.8	23.9	20	2
Os03g0687900	2.04	1.86	6.47	16.42	27.12	20.76	29	23.4	18	6.035	7.675	5.645	11.974	6.5342	4.7518	0	1.031	2.072	13.93	4.031	5.173	15.53	10.69	22.39	4.225	1.886	8.772	1.85	1.91	0	12
Os03g0320800	0	0	0	0	0	0	0.78	0	1.38	1.724	1.096	0	0	0	0	0	1.031	1.036	0	0	0	0	0	0	0	0	0	0	1.91	0.83	7
Os03g0659700	0	0	0	2.463	1.808	2.595	0	2.53	4.15	0	2.193	0	3.2656	0	0.792	0	0	0	3.716	3.225	0	2.958	3.563	0	0	0.943	8.772	2.78	0	0	11
Os03g0243700	0	0	0	2.463	0	0	8.61	3.16	2.07	0	0	0.941	0	0	0	0	0	0	0	0	3.449	2.958	1.425	3.611	0	1.886	0	0	1.91	0	18
Os03g0101100	1565	1899	1753	2196	2531	2199	3444	3122	3399	1799	1998	2017	1530.5	1632.6	1511.9	2543	1476	1574	2051	1983	1757	2754	2588	2913	1826	1740	1748	1417	1327	1612	18
Os03g0343225	0	0	0	0	0	0	0	0	0	0	0	0	0	0	0	0	0	0	0	0	0	0	0	0	2.113	0	0	0	0	0	1
Os03g0761200	39.8	35.4	42	56.65	56.95	46.71	24.3	37.3	36.6	29.31	37.28	52.69	56.603	51.34	41.974	47.76	24.73	25.9	74.32	61.28	68.12	35.49	21.38	33.95	40.14	20.74	19.74	31.5	27.7	27.5	16
Os03g0844100	565	442	487	388.3	386	354.7	483	577	520	479.4	426.5	480.8	474.59	452.72	450.63	573.2	451.4	437.2	347.4	271.7	433.7	505	528.1	498.3	501.8	481.8	559.2	415	428	490	5
Os03g0245100	740	648	885	1192	1299	1038	857	841	688	626.8	582.2	591.8	724.95	757.03	676.34	1039	678.1	707.6	1252	1265	1207	707.6	580.8	709.2	455.3	460.1	361.8	532	563	654	10
Os03g0318500	2.04	3.73	8.62	13.14	10.85	12.11	21.9	25.9	8.29	0	9.868	0	6.5311	0.9335	1.5839	0	0	10.36	10.22	16.93	18.11	13.31	17.1	7.945	2.113	0.943	0	2.78	4.78	4.17	12
Os03g0660700	203	172	235	222.5	211.5	208.5	506	397	490	281.1	224.8	244.6	261.24	208.16	220.17	346.3	171.1	155.4	237.8	285.4	216.4	354.2	379.1	402.3	179.6	178.2	160.1	168	221	236	18
Os03g0118800	194	138	129	102.6	119.3	128	42.3	51.2	53.2	115.5	107.4	136.4	74.019	126.02	68.109	214.9	179.3	214.5	68.74	66.92	51.73	70.24	58.44	70.06	159.5	179.1	241.2	189	151	212	13
Os03g0138000	333	292	295	327.6	350.7	297.6	323	485	457	240.6	229.1	159	238.39	224.96	343.71	644.8	365.8	304.6	275	308	303.5	494.6	493.9	471.6	267.3	197.1	232.4	343	370	401	6
Os03g0787400	41.8	107	24.8	1.642	2.712	0	11.7	2.53	6.91	11.21	7.675	7.527	9.7967	5.6007	11.879	11.94	10.31	12.43	8.361	1.613	8.622	1.479	1.425	12.28	11.62	6.6	2.193	5.55	6.69	12.5	14
Os03g0387000	676	763	694	981.1	1071	850.3	682	761	747	733.7	765.3	732	867.55	737.43	839.48	1337	790.4	760.5	898.3	867.6	881.2	628.5	690.5	678.9	747.9	749.6	725.9	793	753	724	6
Os03g0302000	117	103	128	101	117.5	134.1	110	133	150	206.1	142.5	226.7	163.28	165.22	152.06	167.2	139.1	143	150.5	177.4	131.1	91.68	124.7	144.4	169	133.9	171	91.6	133	125	20
Os03g0782950	10.2	11.2	9.7	23.81	26.22	19.03	0	4.43	4.15	6.897	12.06	3.763	11.974	1.8669	4.7518	0	0	11.4	1.858	10.48	9.484	1.479	4.276	5.056	9.507	4.714	0	9.25	6.69	13.3	2
Os03g0239200	2179	1878	1766	817.7	726.8	766.4	1058	1121	1037	1026	887	987.9	931.77	876.51	944.02	1122	1197	1413	934.5	778.9	810.5	964.9	1045	1049	878.9	902.3	991.2	764	770	801	14
Os03g0812200	2084	2357	2338	2327	2309	2165	2252	2501	2049	2400	2138	1758	1857	1741.8	2135.1	2746	1884	2010	1741	1957	1792	2311	2640	2079	2217	1920	1866	2188	2199	2260	17
Os03g0168550	0	1.86	2.16	18.88	13.56	12.98	13.3	9.49	11.7	0	1.096	0	0	0	0	0	0	1.036	14.86	9.675	9.484	10.35	15.68	13	0	0	0	0	0	0	12
Os03g0635100	9.18	12.1	3.23	13.14	13.56	22.49	34.4	17.1	18.7	11.21	8.771	5.645	7.6196	12.135	10.296	11.94	12.37	6.216	13.93	8.869	9.484	34.01	19.24	20.22	15.85	14.14	4.386	18.5	15.3	8.33	18
Os03g0185000	0	0	0	0.821	0	0	0	0	0.69	0	0	0	0	0	0	0	0	0	0	0	0.862	3.697	3.563	0	1.056	0	0	0	0	0	17
Os03g0124000	190	230	195	168.3	136.5	127.2	107	91.7	110	240.6	190.8	194.7	249.27	130.68	209.87	417.9	244.2	264.2	173.7	213.7	185.4	181.9	198.8	142.3	266.2	175.4	179.8	248	257	296	13
Os03g0101500	63.2	68.9	59.3	30.38	45.2	51.04	86.1	48.7	41.5	54.32	51.53	97.85	35.921	43.872	40.39	35.82	44.31	41.44	50.16	52.41	40.52	50.28	43.47	52.72	50.71	52.8	52.63	41.6	44	39.2	20
Os03g0240400	123	133	91.6	170.8	145.5	170.4	135	145	147	103.5	117.3	87.5	120.83	139.08	137.01	167.2	85.53	133.6	149.6	117.7	99.15	138.3	154.6	132.9	104.6	116	76.75	104	111	118	2
Os03g0606600	181	178	124	134.6	122.9	88.23	283	224	176	187.1	278.5	237.1	109.94	82.144	93.452	167.2	135	159.5	97.54	58.86	93.98	337.2	210.2	275.9	179.6	165.9	210.5	125	115	134	5
Os03g0748400	761	752	839	665.8	678.9	686	446	431	493	553.5	649.1	505.2	764.14	754.23	774.54	1218	813.1	766.7	658.6	566	583.7	646.2	566.5	535.9	752.1	716.6	771.9	886	842	903	13
Os03g0130200	6.12	3.73	3.23	5.747	5.424	2.595	4.7	8.22	1.38	10.35	6.578	7.527	1.0885	4.6673	0.792	0	4.122	2.072	8.361	1.613	1.724	0	1.425	0.722	1.056	0	2.193	0	0.96	1.67	7
Os03g0285800	1069	1355	763	163.4	118.4	118.5	166	196	239	188	85.52	111	101.23	142.82	120.38	465.7	632.7	503.5	124.5	179.8	128.5	357.1	343.5	476.7	96.13	111.3	105.3	82.3	120	81.6	14
Os03g0238300	381	487	488	211.8	206.1	295.8	134	101	143	690.6	683.1	788.4	583.45	589.94	585.26	453.8	563.7	462.1	419.9	276.6	443.2	229.2	153.9	235.5	840.9	712.8	693	707	514	601	3
Os03g0179200	14.3	14.9	22.6	26.27	18.08	21.63	16.4	19.6	9.67	12.07	15.35	23.52	21.77	16.802	22.175	11.94	20.61	18.65	16.72	16.93	12.07	14.79	13.54	18.78	20.07	20.74	24.12	26.8	13.4	35	1
Os03g0135200	4.08	4.66	3.23	8.21	17.18	15.57	8.61	9.49	2.07	9.484	3.289	6.586	10.885	9.3345	7.1277	23.88	0	2.072	5.574	6.45	10.35	9.612	12.83	6.5	6.338	4.714	0	9.25	6.69	7.5	6
Os03g0297100	1439	1126	1199	1520	1803	1738	2241	2024	2469	1328	1398	1962	1492.4	1607.4	1143.6	1564	993.4	1200	1793	1591	1832	1590	1650	1660	1065	1491	1743	884	1028	876	15
Os03g0225900	11.2	31.7	17.2	0	0	2.595	1.57	3.8	10.4	1.724	2.193	0	1.0885	0	2.3759	0	1.031	2.072	0	0.806	0	20.7	14.25	18.06	1.056	0	0	3.7	1.91	1.67	14
Os03g0625300	114	102	123	43.51	20.79	39.79	1.57	7.59	1.38	244	198.4	256.8	214.44	177.36	144.14	95.53	196.8	174.1	105	129	124.2	1.479	1.425	2.167	245.1	180.1	142.5	210	158	203	3
Os03g0825300	10.2	12.1	6.47	8.21	10.85	18.17	32.1	20.2	13.1	6.897	20.83	21.64	8.7082	5.6007	6.3357	0	13.4	4.144	9.29	5.644	7.76	10.35	9.264	15.17	2.113	14.14	15.35	5.55	2.87	10.8	15
Os03g0809100	328	419	376	532	501.7	538.1	500	574	466	293.1	303.7	214.5	291.72	282.84	298.57	573.2	330.8	296.3	466.3	554.7	525.1	554.5	624.3	531.6	305.3	278.1	219.3	356	349	315	12
Os03g0337800	2062	2094	2105	2350	2631	2412	2289	2391	2540	2000	1593	2539	2291.3	2282.3	1858	1851	1822	1715	1994	1495	1846	2112	2242	2040	1848	1952	1875	1450	1760	1730	18
Os03g0258900	0	0	0	0	0	0	0	0	0	0	0	0	0	0	0	0	0	0	0	0	0	0	0	0.722	0	0	0	0	0	0	9
Os03g0645900	12.2	17.7	12.9	7.389	6.328	11.25	4.7	3.16	4.15	2.587	2.193	4.704	4.3541	0.9335	5.5438	71.65	31.95	7.252	16.72	12.9	4.311	2.218	2.851	2.167	9.507	5.657	13.16	0	3.83	5	6
Os03g0676400	43.8	66.1	19.4	9.852	3.616	6.055	2.35	17.1	20.7	7.76	0	1.882	4.3541	0	0.792	0	6.183	10.36	11.15	2.419	3.449	7.394	6.414	11.56	0	0	0	0	0.96	1.67	14
Os03g0225300	145	161	167	147.8	170.9	141	197	233	229	162.1	176.5	147.7	201.38	146.55	175.82	406	138.1	143	137.5	147.5	143.1	152.3	190.3	177.7	168	202.7	149.1	127	149	142	6
Os03g0651000	540	527	595	1038	1231	971.4	1172	1113	1100	542.3	767.5	826	726.04	649.68	476.76	859.7	449.3	564.6	1279	1275	1330	785.2	788.9	1005	502.8	484.6	491.2	327	389	376	12
Os03g0211700	322	302	253	541	447.5	660.9	310	234	211	203.5	183.1	159.9	202.46	238.96	185.32	477.6	327.7	291.1	516.5	519.2	630.3	293.5	317.8	385	202.8	260.2	311.4	285	304	276	10
Os03g0326300	138	102	159	121.5	104.9	119.4	215	152	170	209.5	195.2	225.8	215.53	195.09	171.06	71.65	170	99.46	151.4	153.2	184.5	102.8	106.2	150.9	176.4	142.4	157.9	144	148	151	20
Os03g0253200	0	0	0	0	0	0	0	0	0	0	0	0	0	0	0	0	0	0	0.929	0.806	0.862	0	0	0.722	0	1.886	2.193	0	0	0	11
Os03g0582000	44.9	53.1	61.4	1644	1694	1770	117	103	113	50.01	38.37	30.11	42.452	49.473	44.35	47.76	36.07	32.12	1038	971.6	1305	149.4	124	148.1	28.52	46.2	43.86	62.9	44.9	35.8	10
Os03g0168800	1.02	1.86	2.16	0	4.52	2.595	0.78	0	0.69	0.862	3.289	0	1.0885	0.9335	0	35.82	1.031	0	0	3.225	0	5.915	2.138	0	0	0	0	0.93	0.96	1.67	6
Os03g0802900	56.1	31.7	59.3	39.41	46.1	32.87	20.4	31.6	20.7	20.69	17.54	18.82	16.328	24.27	27.719	71.65	27.82	46.62	29.73	45.96	28.45	20.7	14.25	18.06	24.3	25.46	21.93	17.6	33.5	28.3	6
Os03g0125300	1469	1614	1333	1716	1826	1631	2783	2756	2915	1589	1492	1776	1445.6	1510.3	1241	2269	1366	1532	1418	1305	1363	2338	2367	2428	1411	1666	1487	1167	1139	1008	18
Os03g0351800	1381	1419	1324	604.2	568.6	632.4	1599	1539	1499	1438	1390	1318	1505.4	1463.7	1473.8	2006	1575	1570	566.7	554.7	526	1279	1265	1431	1458	1457	1638	1328	1358	1509	5
Os03g0305500	560	531	579	991.7	1036	1051	825	863	1043	569.9	414.4	526.9	499.63	502.2	416.57	728.4	285.5	387.5	819.3	637.8	762.2	658.1	799.6	717.2	324.3	324.3	320.2	264	338	270	12
Os03g0107000	376	400	429	483.6	508.9	510.4	294	369	357	381.9	428.7	402.7	492.01	383.65	392.82	609	377.2	440.3	378.1	461.2	381.1	331.2	371.3	309.8	417.3	426.2	335.5	474	462	415	6
Os03g0323600	227	386	332	432.7	289.3	249.1	1386	1678	1792	842.4	866.2	615.3	784.82	364.05	920.26	453.8	249.4	310.8	362.3	140.3	135.4	1371	1406	1086	535.6	376.2	300.4	334	393	472	18
Os03g0758551	71.4	101	91.6	71.42	88.59	90.83	19.6	13.3	11.1	37.07	28.51	31.05	44.629	39.205	64.941	143.3	57.71	77.7	37.16	27.41	23.28	67.28	65.56	39	47.54	37.71	21.93	94.4	75.5	112	8
Os03g0307100	2727	2937	2707	4772	5609	4688	1659	1605	1595	2464	2580	2679	2743.1	2726.6	2563.6	3379	2649	2629	4216	4264	4449	1480	1687	1631	2354	2521	2436	2488	2446	2401	10
Os03g0337600	1568	1901	1717	2222	2362	1990	2301	2589	2464	2124	2374	3413	1668.7	1484.2	1564.9	1899	1422	1769	1710	1850	1921	1948	1792	2190	2092	2165	1976	1131	1160	1294	15
Os03g0669200	674	670	663	786.5	839.8	820.9	999	845	1055	769.9	764.2	669.9	755.43	701.96	784.05	1158	651.3	594.7	809.1	718.4	810.5	918.3	850.2	1008	667.6	637.4	750	619	621	645	18

Os03g0708900	143	140	133	408.8	425.8	443.8	136	133	96.1	81.91	82.23	100.7	145.86	171.76	95.828	262.7	131.9	157.5	441.3	359.6	405.2	119.8	112.6	90.28	79.23	115	153.5	147	96.6	138	10
Os03g0579800	30.6	46.6	42	22.99	29.83	27.68	21.1	13.3	24.2	28.45	14.25	33.87	31.567	33.604	20.591	23.88	25.76	32.12	14.86	16.93	18.11	17.01	20.67	10.11	20.07	30.17	24.12	45.3	39.2	30	8
Os03g0698900	663	611	677	477	483.6	522.5	489	490	489	472.5	494.5	442.2	422.35	501.26	497.35	668.7	637.9	557.4	492.3	466	499.2	508	464.6	473.8	498.6	557.2	532.9	550	471	582	14
Os03g0106000	815	866	961	1496	1512	1410	2479	2706	2555	1117	1128	1104	1356.3	1140.7	1060.4	2018	932.6	906.5	1409	1571	1561	1610	1623	1815	968.7	958.9	949.5	893	887	998	18
Os03g0140500	56.1	77.3	50.6	90.31	85.88	100.3	73.6	84.1	83.6	48.28	80.04	64.92	63.134	59.741	54.646	59.7	52.56	61.13	75.25	70.95	72.43	73.2	62.71	93.89	71.83	41.49	41.67	39.8	58.3	56.7	12
Os03g0276800	0	0	0	0	0	0	0	0	0	0	0	0	0	0	0	0	0	0	0	0	0	0	0	0	1.056	0	0	0	0	0	1
Os03g0612400	0	0	0	0	0	0	0	0	0	0	0	0	0	0	0	0	0	0	0	0	0	0	0	0	0	0	0	0	0	1.67	1
Os03g0656100	9.18	1.86	1.08	2.463	0	2.595	1.57	3.8	2.76	0.862	2.193	3.763	1.0885	1.8669	0.792	0	6.183	3.108	3.716	1.613	4.311	0	0.713	2.167	1.056	5.657	26.32	0	1.91	2.5	11
Os03g0622300	45.9	52.2	47.4	119	64.18	64.88	54	58.8	64.3	62.94	73.46	50.8	78.373	53.207	57.814	143.3	50.5	88.06	103.1	98.37	87.08	65.07	54.87	54.89	43.31	60.34	74.56	49	56.4	40.8	6
Os03g0861700	1604	1891	2041	2122	2405	2029	1891	2099	2061	1549	1784	1916	2167.2	1720.4	1636.2	3140	1647	1855	1969	2261	2032	1559	1583	1725	1618	1473	1167	1541	1872	1971	6
Os03g0141700	9.18	14.9	6.47	2.463	6.328	4.325	7.83	2.53	13.8	10.35	12.06	11.29	4.3541	16.802	7.1277	35.82	17.52	9.324	7.432	13.71	4.311	11.83	14.25	20.95	13.73	16.97	35.09	7.4	14.3	7.5	11
Os03g0169900	449	619	624	1112	1124	1043	791	737	798	847.5	964.8	748	894.76	769.16	906.01	1182	448.3	460	1259	1269	1191	705.4	779.6	632	880	713.7	592.1	554	643	802	10
Os03g0363700	38.7	59.6	50.6	112.5	122.9	84.77	32.9	38	33.2	26.73	36.18	26.34	63.134	42.005	51.478	59.7	19.58	46.62	34.37	56.44	75.01	70.98	91.93	50.56	40.14	32.06	19.74	63.8	41.1	54.2	2
Os03g0387800	97.9	115	106	69.78	55.14	71.8	21.9	38.6	29.7	55.18	80.04	47.98	64.223	61.608	64.941	71.65	71.11	67.34	48.31	25.8	44.84	48.8	47.75	41.89	69.72	75.43	32.89	90.7	66	102	8
Os03g0726100	1559	1388	1321	1649	1326	2260	1159	877	977	1348	1093	1580	1186.5	1257.4	1096.9	1970	1657	1274	2107	1783	2350	869.5	830.2	1129	1186	1239	1393	1111	1146	1118	16
Os03g0389501	14.3	13	9.7	3.284	3.616	2.595	2.35	6.33	0.69	9.484	3.289	16.93	9.7967	9.3345	3.9598	11.94	7.214	7.252	3.716	6.45	10.35	4.436	2.138	2.889	12.68	8.486	10.96	12	2.87	7.5	3
Os03g0264150	293	255	225	68.14	58.76	89.96	127	99.3	135	137.9	115.1	112.9	109.94	152.15	129.88	71.65	207.1	215.5	97.54	75.79	108.6	113.9	109	117	105.6	98.06	114	129	96.6	113	14
Os03g0185200	98.9	102	106	151.1	141	157.4	81.4	91.1	76.7	79.32	87.71	86.56	123	105.48	104.54	191.1	99.96	106.7	122.6	154	112.1	126.4	114.7	96.78	89.79	75.43	67.98	124	117	108	6
Os03g0374400	383	341	403	169.9	166.3	184.3	229	209	179	280.2	246.7	314.2	280.84	323.91	301.74	441.8	412.2	354.3	196.9	218.5	200	188.5	234.5	211.6	277.8	276.3	247.8	287	318	307	13
Os03g0760500	113	41.9	40.9	41.87	18.98	58.82	60.3	72.1	78.1	192.3	138.1	266.3	76.196	82.144	98.996	59.7	58.74	63.2	42.73	16.93	13.8	167.1	107.6	130.7	107.7	173.5	318	99.9	107	90	20
Os03g0858600	14.3	4.66	7.54	9.852	5.424	1.73	5.48	1.27	1.38	15.52	6.578	6.586	2.177	5.6007	0.792	11.94	9.275	11.4	2.787	4.031	5.173	4.436	4.276	20.95	4.225	2.829	4.386	4.63	4.78	4.17	19
Os03g0569800	279	237	295	315.3	311	320.9	52.5	65.8	70.5	253.5	273	390.4	296.08	275.37	213.83	370.2	294.7	361.6	468.2	449.1	497.5	53.24	49.17	59.95	239.8	291.3	285.1	247	272	279	16
Os03g0152000	552	926	307	142	60.57	101.2	26.6	36.7	37.3	67.25	78.94	53.63	79.462	52.273	94.244	441.8	471	307.7	92.9	127.4	156.9	73.2	105.5	287.5	104.6	98.06	74.56	84.2	87	142	14
Os03g0743200	149	131	120	173.2	139.2	183.4	186	241	249	196.6	187.5	136.4	263.42	159.62	203.54	191.1	123.7	139.9	151.4	176.6	122.4	235.1	256.5	196.5	181.7	134.8	146.9	139	162	165	18
Os03g0740800	72.4	63.3	61.4	101	84.77	84.77	65	65.1	87.1	56.9	47.14	68.68	52.249	47.606	57.022	59.7	43.28	71.49	101.3	79.82	103.5	62.11	75.54	72.22	33.8	47.14	76.75	43.5	35.4	55	10
Os03g0124300	2314	1831	2062	1291	1156	1260	1429	1328	1265	1397	1239	1450	1160.4	1259.2	1214.1	2114	1612	1773	1123	1120	1230	1233	1345	1468	1331	1425	1520	1190	1202	1111	14
Os03g0400200	2.04	0	0	0	0.904	0	4.7	0	2.07	3.449	0	2.822	0	1.8669	2.3759	0	2.061	1.036	0	0.806	0	2.218	0.713	0	0	0	0	0	1.91	0	5
Os03g0269500	14.3	5.59	6.47	4.926	8.136	18.17	29	10.8	28.3	18.97	16.45	21.64	29.39	21.469	15.047	0	19.58	18.65	21.37	29.83	34.49	20.7	2.851	23.83	14.79	18.86	35.09	21.3	19.1	16.7	15
Os03g0755900	4.08	4.66	3.23	2.463	4.52	2.595	7.05	6.96	5.53	1.724	3.289	3.763	1.0885	2.8004	6.3357	0	5.153	0	8.361	4.838	6.898	9.612	3.563	5.778	7.395	6.6	0	1.85	5.74	5	9
Os03g0737900	717	776	766	907.2	933.8	942	766	793	794	762.2	726.9	742.3	826.19	830.77	818.89	1994	632.7	750.1	796.1	816.8	867.4	689.8	701.9	663.7	843	758	673.2	759	710	811	6
Os03g0837100	79.5	81	85.1	105.1	75.93	160	53.2	52.5	51.1	45.7	17.54	41.4	34.833	47.606	30.095	83.59	78.32	62.16	67.81	63.7	95.71	76.16	77.68	108.3	22.18	45.26	81.14	46.3	59.3	34.2	2
Os03g0133300	87.7	95	85.1	37.76	50.62	68.34	55.6	113	105	127.6	100.9	159.9	114.29	87.744	106.92	71.65	71.11	55.95	27.87	23.38	23.28	62.11	59.15	71.5	133.1	89.57	89.91	56.4	88.9	96.6	20
Os03g0270500	18.4	10.2	16.2	5.747	4.52	0.865	18.8	19.6	10.4	21.55	10.96	15.05	7.6196	14.002	19.799	0	10.31	9.324	4.645	8.063	10.35	7.394	12.11	6.5	11.62	4.714	13.16	9.25	12.4	11.7	5
Os03g0109800	937	941	1015	108.4	67.8	105.5	189	212	159	1211	1517	1416	1182.1	1028.7	1303.6	2448	1338	1226	170	158	130.2	130.1	138.3	172.6	1461	1666	1382	1234	1445	1331	13
Os03g0281800	172	154	137	222.5	186.2	202.4	139	117	113	163.8	140.3	170.3	241.65	193.22	190.86	179.1	173.1	195.8	215.5	208.8	205.2	161.9	156.1	114.8	202.8	194.2	140.3	201	210	235	16
Os03g0588600	2.04	1.86	2.16	3.284	2.712	6.92	1.57	0	4.15	3.449	3.289	0.941	0	0	1.5839	23.88	1.031	3.108	1.858	0.806	2.587	5.176	6.414	6.5	2.113	9.428	8.772	0	4.78	5.83	6
Os03g0845300	2.04	1.86	1.08	3.284	0.904	2.595	0.78	0	2.07	0	0	0	1.0885	0	3.1679	0	1.031	0	5.574	0.806	1.724	0	3.563	2.167	0	1.886	0	3.7	1.91	0	2
Os03g0691400	226	251	232	125.6	106.7	128	294	392	359	281.9	285.1	236.1	237.3	261.37	246.3	370.2	216.4	252.8	119.8	138.7	95.71	222.6	240.2	296.8	227.1	250.8	245.6	272	253	278	5
Os03g0841600	8.16	5.59	6.47	50.9	26.22	51.04	14.9	17.1	19.3	0	6.578	3.763	13.062	12.135	29.303	47.76	21.64	14.5	48.31	46.76	39.66	21.44	51.31	29.61	14.79	1.886	8.772	15.7	31.6	22.5	10
Os03g0232400	4525	4485	5342	2365	2399	2480	320	353	566	7360	6757	7616	5539.5	6823.5	6209	7320	6514	4934	4859	3686	5208	303.1	272.9	274.5	9339	7338	6770	7109	4889	5729	3
Os03g0788700	2.04	7.45	4.31	0	0	0.865	0.78	2.53	0	1.724	2.193	2.822	1.0885	1.8669	0.792	0	1.031	2.072	1.858	0.806	3.449	4.436	1.425	1.444	4.225	2.829	2.193	1.85	0.96	2.5	14
Os03g0201400	319	344	381	317.7	339	358.1	220	224	207	319.9	305.9	375.4	355.95	379.91	383.31	453.8	336	392.7	301	329	366.4	262.5	257.3	227.5	386.6	414.9	396.9	394	388	402	13
Os03g0811500	485	480	450	527.1	558.7	527.7	579	621	626	517.3	583.3	499.6	634.61	519.93	593.18	800	491.6	533.6	510.9	573.3	503.5	536.1	585.8	532.3	557.8	573.2	491.2	568	541	585	6
Os03g0206201	2.04	2.79	3.23	0	0	1.73	0	0	0	0.862	0	0	4.3541	1.8669	1.5839	0	3.092	0	2.787	0	2.587	0	0	0.722	0	4.714	2.193	2.78	1.91	0	4
Os03g0757550	36.7	35.4	36.6	33.66	31.64	29.41	12.5	17.7	8.98	22.42	31.8	15.05	38.098	23.336	42.766	47.76	36.07	38.33	23.22	54.02	28.45	29.58	22.8	24.56	23.24	23.57	19.74	52.7	39.2	45	8
Os03g0789900	15.3	16.8	20.5	16.42	12.66	19.03	21.9	17.7	19.3	32.76	17.54	48.92	9.7967	18.669	17.423	11.94	18.55	16.58	24.15	20.16	19.83	16.27	9.977	20.95	19.01	15.09	26.32	17.6	14.3	10.8	20
Os03g0579300	335	279	288	304.6	324.5	366.8	398	304	466	368.2	314.7	536.3	304.79	378.98	280.36	394.1	245.3	269.4	484.9	456.4	454.4	289.8	286.5	283.8	259.9	385.6	392.5	237	275	182	15
Os03g0191100	1376	1134	1367	409.7	387.8	411.8	333	471	549	1061	1113	1126	1489.1	1438.4	1479.4	1767	1480	1477	419	406.4	387.1	520.5	478.9	512.8	1410	1407	1395	1405	1368	1519	3
Os03g0165266	39.8	58.7	25.9	54.18	39.77	35.47	42.3	50	64.3	60.35	72.36	52.69	31.567	36.405	60.981	35.82	15.46	15.54	27.87	33.86	22.42	36.97	34.21	48.39	48.59	36.77	43.86	39.8	32.5	39.2	7
Os03g0699000	1.02	0	0	0	0	0	0	0	0	0	0	0	0	0	0	0	0	0	0	0	0	0	0	0	0	0	0	0	0	0	19
Os03g0240600	28.5	28.9	25.9	1.642	0.904	0.865	0	0.63	0	14.66	2.193	7.527	1.0885	1.8669	2.3759	0	15.46	14.5	0.929	0	0	5.915	4.988	7.222	3.169	1.886	0	0	2.87	1.67	14
Os03g0334800	435	366	419	268.5	273	269.9	377	342	342	544	506.5	573	443.03	447.12	475.18	955.3	407.1	441.4	451.5	385.4	360.4	262.5	274.4	278.1	463.7	466.7	592.1	440	479	408	13
Os03g0738600	1.02	0	0	9.031	0	6.055	0	3.16	0.69	0.862	0	0	1.0885	0.9335	0	0	0	0	0	0	0	0	0	0	0	0	0	0	0	0	2
Os03g0601100	403	438	387	435.1	414.9	403.1	360	460	365	372.5	395.8	383.9	335.26	345.38	422.91	967.2	401.9	342.9	372.5	402.3	419.9	391.9	478.2	499.8	384.5	373.4	414.5	430	416	418	6
Os03g0233900	1.02	0	0	0.821	0	4.325	0	0	1.38	3.449	0	3.763	0	0	0	0	0	0	2.787	0	0	0.739	0.713	0.722	0	0	0	0	4.78	0	7
Os03g0201800	0	0.93	0	0.821	1.808	0	0.78	1.9	1.38	0	2.193	0	1.0885	0.9335	0	0	0	1.036	0.929	0	1.724	3.697	4.276	6.5	2.113	0.943	0	1.85	0.96	0.83	9
Os03g0852200	108	106	99.1	86.2	93.11	95.16	66.5	62.6	60.1	58.63	53.72	44.22	89.259	70.009	57.022	35.82	60.8	75.63	42.73	45.15	40.52	110.9	82.67	92.45	59.16	61.28	65.79	74	64.1	72.5	8
Os03g0283100	5769	4863	5747	3061	3363	2687	1954	1939	1696	4105	4276	4826	5353.3	5996.5	6116.4	8036	6168	5301	3875	3547	3724	1270	1549	1689	5066	4571	5346	5327	5269	5248	13
Os03g0743650	202	183	178	179	170.9	204.2	365	300	365	235.4	208.3	193.8	188.31	210.96	206.7	250.8	194.8	204.1	205.3	180.6	222.5	271.4	263	304.1	188	175.4	144.7	200	201	179	18
Os03g0352700	0	0	0	0.821	0	0	0.78	0	2.07	0	0	0	0	0	0	0	1.031	0	0	0	2.587	1.479	0	0	0	0	0	0	0	0	15
Os03g0729500	0	0	1.08	1.642	1.808	5.19	0	0	0	0	0	0	0	0	0	0	0	0	1.858	1.613	5.173	0	0	0	0	0	0	0	0	1.67	10
Os03g0184700	238	253	278	348.1	332.7	311.4	207	263	236	285.4	322.3	285.1	386.42	294.97	305.7	561.2	316.4	302.5	284.3	349.1	313.8	256.6	253	223.2	331.7	322.5	256.6	303	294	290	6
Os03g0391100	0	0	0	0	0	0	0.78	0	0	0.862	1.096	0	0	0	1.5839	0	0	0	0	0	0	0	0	0	0	0.943	0	0	0	0	7
Os03g0616450	0	0	0	1.642	0.904	3.46	0	0	0	0	0	0	1.0885	0	0	0	0	0	0.929	0	6.036	0	0	0	0	0	0	0	0	0	16
Os03g0850800	289	297	305	293.1	311	273.4	406	343	329	283.7	264.2	345.3	185.05	260.43	176.61	298.5	222.6	231	366.9	315.3	293.2	214.4	228	321.4	188	252.7	228.1	177	171	127	15
Os03g0683800	2.04	0	0	0.821	0	0	0	1.9	0	0	2.193	0	1.0885	0	0.792	0	0	0	0	0	1.724	0.739	0	2.167	0	0	0	2.78	0	3.33	1
Os03g0432900	1842	1596	1826	1797	1669	2040	2568	2858	3056	2247	2321	2678	2455.7	2315.9	2374.3	4120	2050	2208	2440	2684	2690	2409	2324	2387	2321	2430	2289	1806	2111	1855	6
Os03g0183500	120	107	52.8	113.3	11.75	152.2	29	53.8	74.6	9.484	6.578	1.882	43.541	18.669	3.1679	59.7	27.82	41.44	47.38	37.9	58.63	119	179.6	93.17	19.01	23.57	76.75	20.4	33.5	25.8	17
Os03g0661700	7.14	8.38	3.23	1.642	4.52	7.785	11	5.06	5.53	3.449	4.386	0.941	3.2656	2.8004	1.5839	0	4.122	2.072	1.858	10.48	2.587	5.176	3.563	10.11	3.169	3.771	2.193	1.85	2.87	3.33	9
Os03g0163500	92.8	86.6	77.6	66.5	71.41	74.39	71.2	60.7	51.1	61.22	66.88	93.14	58.78	74.676	66.525	167.2	115.4	96.35	97.54	99.98	116.4	82.07	91.22	93.17	86.62	109.4	127.2	126	117	114	13
Os03g0298300	236	267	214	180.6	177.2	176.5	524	402	417	189.7	177.6	247.4	203.55	228.7	189.28	250.8	240.1	217.6	190.4	190.3	230.2	228.5	268.7	284.6	171.1	236.7	265.3	179	191	167	18
Os03g0221700	33.6	41	28	74.71	91.3	48.44	61.1	78.4	76	39.66	54.82	57.39	19.593	37.338	46.726	23.88	37.1	40.41	71.53	76.6	54.32	111.6	84.8	135.1	43.31	65.06	98.68	50.9	37.3	31.7	9
Os03g0341300	142	126	127	65.68	53.33	80.45	61.8	55.7	41.5	167.3	162.3	201.3	99.055	143.75	152.85	191.1	148.4	158.5	97.54	87.08	152.6	57.67	128.3	83.78	164.8	204.6	271.9	205	166	157	3
Os03g0322900	0	0.93	0	0	0	0	0	0	0.69	5.173	0	0	0	0	0.792	0	1.031	0	0.929	0	0.862	0	0	0	0	3.771	0	0	0	0	7
Os03g0161900	####	7824	9325	360.4	237.7	354.7	166	200	183	8898	10637	12591	4736.2	4334.9	6528.2	10281	9124	7180	1119	828.9	931.2	162.7	109.7	190.7	12988	12358	11908	5546	5365	5119	3
Os03g0327800	129	250	162	297.2	348.9	118.5	608	867	355	296.6	434.2	191	568.21	336.04	674.76	513.5	160.8	232.1	215.5	150.8	112.1	292.1	593.6	217.4	292.6	172.5	149.1	371	435	521	5
Os03g0418000	0	0	0	1.642	1.808	0.865	0.78	1.27	3.46	15.52	7.675	3.763	1.0885	0.9335	0	0	0	1.036	0	0	0	0.739	0	0	0	0	0	0	1.91	0	7
Os03g0234900	0	0.93	0	0	0	0.865	0	0	0	0	0	0	1.0885	0	0	0	0	0	0	0	1.724	0	0	0	0	0.943	4.386	0	0	0.83	11
Os03g0797800	0	0	0	0	0	0	0	0	0	0	0	0	1.0885	0	0	0	0	0	0	0	0	0	0	0	0	0	0	0.93	0	0	4
Os03g0857750	333	314	306	383.4	367	340.8	769	581	670	572.5	478	577.7	413.64	358.45	399.94	191.1	312.3	326.4	426.4	409.6	413.9	404.4	367	554.7	404.6	446	416.7	265	292	267	15
Os03g0186800	415	328	355	237.3	309.2	269	269	304	312	278.5	268.6	255.9	253.63	257.63	279.56	453.8	333.9	295.3	256.4	286.2	312.1	232.2	289.3	247.7	234.5	298.9	256.6	255	238	276	6
Os03g0586400	71.4	56.8	76.5	2017	2054	1877	240	204	247	37.07	29.6	32.93	59.869	63.475	52.27	131.4	85.53	88.06	1940	2066	1899	266.2	224.5	218.8	42.25	34.89	30.7	94.4	99.5	97.5	10
Os03g0212200	436	389	446	472.9	442.9	462.8	364	382	390	433.7	399.1	365	416.9	460.19	453	632.9	379.2	392.7	501.6	503.9	575.1	359.3	374.8	374.8	351.8	392.2	368.4	358	367	449	6
Os03g0695400	1.02	0.93	0	0.821	1.808	1.73	4.7	0.63	4.84	0	0	0	0	0	0.792	0	0	1.036	8.361	3.225	3.449	2.218	0.713	3.611	0	0.943	0	0.93	1.91	0	12
Os03g0725200	4.08	0.93	6.47	0.821	1.808	0	5.48	4.43	1.38	8.622	7.675	20.7	2.177	4.6673	6.3357	11.94	5.153	5.18	1.858	0	0	2.958	2.851	5.778	8.451	11.31	6.579	1.85	1.91	1.67	20
Os03g0323950	1.02	0	1.08	0.821	0.904	0.865	0	1.27	2.76	1.724	2.193	0	4.3541	0.9335	2.3759	0	2.061	3.108	2.787	0	1.724	0	1.425	2.167	2.113	1.886	2.193	0	0	1.67	4
Os03g0597200	1175	1482	1451	2812	3191	2401	2111	2271	1965	1405	1645	1339	1478.2	1244.3	1196.7	2209	1073	1364	1847	2314	2001	2026	1757	1912	1523	1211	980.2	1180	1399	1490	12
Os03g0730200	0	0	0	0	0	0	0	0.63	0	0	0	1.882	0	0	0	0	0	0	0	0	0	0	0	0	0	0	0	0	0	0	20
Os03g0283200	90.7	94.1	129	22.17	9.944	14.71	14.1	13.3	11.7	80.18	58.11	112	34.833	56.941	40.39	155.2	156.6	146.1	69.67	28.22	45.7	9.612	8.552	74.39	125.7	99	136	108	122	85.8	13
Os03g0167800	282	292	300	284.9	247.7	282	248	195	202	263.8	287.3	227.7	263.42	302.44	281.15	609	314.3	262.1	208.1	242.7	281.9	287.6	229.5	229	291.6	281.9	267.5	334	307	263	13
Os03g0758050	21.4	31.7	34.5	32.02	27.12	32.01	25.8	34.8	21.4	21.55	14.25	12.23	18.505	25.203	25.343	35.82	16.49	29.01	13.93	20.96	14.66	16.27	32.78	10.83	10.56	6.6	15.35	26.8	17.2	18.3	8
Os03g0765800	564	569	587	703.6	706.9	741.3	460	522	520	574.2	588.8	504.3	480.04	474.19	610.61	1075	541	519.1	634.5	687	641.5	673.6	717.6	639.9	687.7	675.1	631.6	690	680	646	6
Os03g0254700	3042	3650	3166	3964	4147	3689	4041	4311	4154	3144	3014	2856	3219.8	3028.1	3289	3893	3005	2942	3044	3038	3192	4091	4133	3970	3197	2972	2838	2976	2845	3197	18
Os03g0821800	284	310	254	303.8	311	373.7	399	408	404	324.2	285.1	244.6	262.33	320.17	289.07	477.6	263.8	242.4	336.3	378.1	414.7	400.7	404.1	361.8	285.2	293.2	289.5	274	250	246	12
Os03g0722600	638	521	575	500	524.3	505.2	434	463	528	424.2	449.5	524	506.16	582.47	552.79	620.9	568.9	581.2	472.8	429.7	496.6	468	496	434.1	495.4	520.4	583.3	518	462	496	13
Os03g0333800	766	712	710	714.2	706.9	724.9	889	932	964	848.4	853	995.4	935.04	983.86	942.44	1337	989.3	913.8	850.9	870	911.4	772.7	775.3	805.3	995.1	1050	1024	804	803	886	13
Os03g0736700	210	229	177	120.7	99.44	113.3	114	98	110	150.9	129.4	179.7	108.85	149.35	125.13	226.9	212.3	231	161.6	109.7	103.5	136	128.3	137.9	163.7	176.3	256.6	144	183	150	13
Os03g0641250	72.4	64.3	60.3	34.48	37.97	31.14	15.7	32.9	20	25	29.6	21.64	40.275	55.074	51.478	107.5	78.32	82.88	27.87	48.38	42.25	108.7	127.6	89.56	62.33	50.91	52.63	118	82.2	96.6	8
Os03g0452300	1394	1556	1434	3211	3651	3069	4260	4563	4776	1742	1956	2265	1617.5	1169.6	1093.7	1087	833.7	1148	2866	2809	2967	2681	2465	3380	1133	974.9	732.4	594	763	967	12
Os03g0400600	258	230	307	151.1	168.1	165.2	214	195	216	249.2	218.2	230.5	266.69	281.9	274.81	585.1	277.2	274.5	247.1	205.6	200.9	128.7	174.6	157.4	193.3	218.7	168.9	215	230	217	13
Os03g0111000	5.1	5.59	7.54	3.284	4.52	4.325	11.7	0	4.84	0	2.193	0	3.2656	6.5342	3.9598	0	1.031	12.43	4.645	0.806	4.311	0.739	1.425	1.444	3.169	1.886	0	4.63	0.96	1.67	19
Os03g0111100	798	714	711	802.9	822.6	737.9	944	930	1028	685.4	667.7	787.5	664	818.64	741.28	1457	827.5	732.5	863.9	821.6	854.5	1020	804.6	967.1	683.5	710	646.9	776	878	788	6
Os03g0762200	0	0	2.16	0.821	0	0.865	1.57	0	0	2.587	3.289	2.822	0	1.8669	3.1679	0	2.061	0	0	0	0	0.739	2.138	1.444	1.056	0.943	0	0.93	1.91	1.67	7
Os03g0210600	1470	1352	1561	1302	1165	1369	1040	950	1009	1641	1181	1559	1416.2	1496.3	1375.6	1624	1255	1118	1538	1331	1301	610	919.3	751.9	1349	1218	1579	926	1211	1045	20
Os03g0561283	0	0.93	0	0	0	0	0	0	0	0	0	0	0	0	0	0	0	0	0	0	0	0	1.425	0	0	0	0	0	0	0	17
Os03g0205000	464	469	398	486	522.5	513.8	446	473	492	456.1	504.3	423.4	585.62	491	548.04	812	475.1	397.8	415.2	495.9	429.4	481.3	545.2	451.4	494.4	497.8	464.9	505	516	584	6
Os03g0654900	0	0.93	0	1.642	2.712	0.865	2.35	3.8	2.76	0.862	3.289	0	1.0885	2.8004	0.792	0	0	1.036	6.503	0.806	1.724	2.958	2.851	5.056	0	0	0	3.7	0	0.83	6
Os03g0398900	0	0	0	0	0	0	0	0	0	0	0	0	0	0	0	0	0	0	0	0	0	0	0	0.722	0	0	0	0	0	0	9
Os03g0409600	4.08	0.93	1.08	0	3.616	3.46	4.7	0	4.84	1.724	0	0	2.177	1.8669	0	0	0	2.072	3.716	0	0.862	4.436	0	2.889	1.056	1.886	0	0	1.91	0	9
Os03g0703900	407	319	346	124	135.6	156.6	250	202	209	330.2	343.2	294.5	265.6	278.17	274.02	250.8	274.1	283.9	192.3	185.4	135.4	236.6	273.7	280.2	250.4	272.5	337.7	257	292	222	3
Os03g0353000	21.4	14.9	12.9	13.96	13.56	12.98	18.8	13.3	18	15.52	7.675	42.34	15.239	11.201	11.088	11.94	10.31	14.5	15.79	10.48	11.21	15.53	8.552	25.28	12.68	12.26	15.35	10.2	19.1	17.5	20
Os03g0385301	810	680	751	557.4	621	628	785	762	719	701	824.5	766.8	794.62	920.38	834.73	835.9	814.1	777	713.4	739.4	762.2	595.9	625.7	661.6	829.2	825	745.6	772	797	807	3
Os03g0276900	623	449	509	316.9	369.7	328.7	380	424	429	301.8	367.3	382.9	364.65	420.99	375.39	776.2	530.7	569.8	370.7	414.4	415.6	405.9	393.4	360.4	388.7	417.7	416.7	363	395	405	13
Os03g0322800	517	469	517	340.7	295.6	376.3	248	309	303	348.3	299.3	301.1	273.22	326.71	354.8	394.1	363.8	347.2	303.8	304.8	278.5	431.8	461.8	326.5	378.2	351.7	396.9	327	341	301	14
Os03g0308500	286	326	287	333.3	332.7	340	365	352	397	384.5	313.6	412.1	391.87	367.78	353.22	441.8	305	312.9	329.8	304.8	299.2	357.9	379.8	364.7	354.9	435.6	407.9	346	334	342	5
Os03g0851300	1755	1984	1898	1853	1966	1850	2619	2564	2798	2003	2033	2044	2041	2041.5	1994.2	3690	1969	1863	1775	1659	1758	2126	2258	2203	1894	1836	1914	1687	1803	1889	6
Os03g0401100	3463	4368	3474	1622	1512	1757	983	1132	893	4351	4380	3067	3139.3	3292.3	4067.5	7690	4820	4477	2058	2042	2507	1228	1631	1406	5719	4579	4235	5826	4461	5272	13

Os03g0257600	33.6	27	26.9	55.83	61.47	44.98	83.8	83.5	66.3	51.73	28.51	46.1	55.515	74.676	48.31	59.7	58.74	36.26	56.67	43.54	36.21	77.64	117.6	96.06	52.82	40.54	96.49	50	61.2	60	17
Os03g0668000	154	197	184	245.5	209.7	205.9	243	244	211	178.5	186.4	148.6	199.2	166.15	188.49	203	169	138.8	202.5	199.2	206.9	223.3	235.9	203.7	197.5	193.3	186.4	193	195	231	12
Os03g0151700	266	305	292	636.3	587.6	601.2	546	553	551	269.9	220.4	250.3	259.07	306.17	319.95	597	244.2	310.8	454.3	483	475.9	476.9	495.3	442.7	213.4	272.5	214.9	226	276	208	12
Os03g0415500	3.06	4.66	1.08	4.926	7.232	9.516	10.2	3.16	6.22	0.862	1.096	1.882	2.177	0.9335	0.792	0	2.061	0	9.29	0.806	0.862	6.654	5.701	2.889	0	0.943	2.193	0	1.91	1.67	12
Os03g0843400	1762	1936	1916	3382	3394	3243	5609	4838	5503	2334	2671	3667	2261.9	1665.3	1373.3	1708	1421	1715	4041	4242	4476	2941	2794	4215	1723	1770	1531	923	1193	1151	12
Os03g0355500	0	0	0	0.821	0	0	0	0	0	0	0	0	0	0	1.5839	0	0	0	0	4.031	0.862	0.739	0	0.722	3.169	0	0	0	0	0	16
Os03g0209600	1589	1693	1942	2183	2425	2160	2259	2385	2262	1517	1624	1671	1873.3	1729.7	1585.5	2508	1628	1859	2321	2496	2486	1701	1626	1978	1380	1494	1248	1383	1597	1463	12
Os03g0607600	1.02	2.79	0	9.031	6.328	0.865	5.48	3.16	7.6	1.724	1.096	0	1.0885	0	3.1679	11.94	0	5.18	11.15	2.419	5.173	5.915	5.701	3.611	4.225	0	2.193	0	0	0.83	6
Os03g0183900	0	0	0	0	0	0	0.78	0	0	0	0	0	0	0	0	0	0	0	0	0	0	0	0	0.722	0	0	0	0	0	0	9
Os03g0441101	31.6	28.9	33.4	59.11	42.49	74.39	62.6	42.4	42.8	50.01	37.28	51.75	40.275	42.005	33.263	11.94	30.92	32.12	66.88	56.44	72.43	59.15	46.32	54.17	39.09	26.4	70.17	44.4	32.5	37.5	15
Os03g0250200	11.2	10.2	4.31	9.852	10.85	10.38	13.3	16.4	11.7	15.52	7.675	7.527	7.6196	8.4011	6.3357	23.88	16.49	3.108	8.361	11.29	9.484	8.873	9.264	10.11	26.41	5.657	10.96	9.25	7.65	8.33	6
Os03g0430400	2203	2289	2136	1991	2085	1932	979	1107	1036	1587	1883	1610	2026.8	1975.2	2086	3439	2370	2446	1531	1846	1720	1736	1556	1389	2136	2108	2026	2527	2332	2524	13
Os03g0178600	7.14	12.1	5.39	29.56	35.25	28.55	29.7	26.6	21.4	12.93	7.675	5.645	5.4426	7.4676	3.1679	71.65	5.153	7.252	26.94	41.93	32.76	29.58	36.34	31.78	9.507	8.486	6.579	11.1	2.87	13.3	6
Os03g0659900	1066	1266	1144	1921	2358	2097	2009	2356	3107	1259	1593	1036	1373.7	1246.2	1637	3546	1167	1358	1573	2006	1952	4578	3287	2995	1701	1577	962.7	1304	1646	1543	17
Os03g0104000	190	203	226	224.9	258.5	217.1	461	456	473	300.9	285.1	329.3	177.43	163.35	197.2	155.2	211.3	225.9	278.7	273.3	240.6	356.4	324.2	339.5	263	225.3	265.3	200	158	182	18
Os03g0353400	5925	7685	5092	4287	3627	3744	2015	2234	1847	2681	2405	1869	2533	2225.3	2691.1	7069	5611	4730	3246	3637	4939	2373	2407	4637	1995	1702	1871	2168	2255	2980	14
Os03g0317100	119	108	135	152.7	147.3	158.3	60.3	83.5	58.7	65.53	81.13	61.15	114.29	88.678	87.908	179.1	145.3	129.5	117	126.6	107.8	96.12	106.2	83.78	87.68	90.51	76.75	135	118	98.3	8
Os03g0248500	0	0	0	0	0	0	0	1.9	0	0	0	0	0	0	0	0	0	0	0	0	0	0	0.713	0	0	0	0	0	0	0	18
Os03g0324200	653	674	600	759.4	732.2	668.7	734	817	876	623.4	724.7	501.5	612.84	510.6	666.84	11.94	606	639.2	526.7	506.3	570.8	1174	1111	899.9	592.6	599.6	589.9	670	646	727	17
Os03g0108600	1521	2079	1881	4447	5275	4512	3866	4484	3884	1546	1926	1563	2383.9	1651.3	1633	2090	1066	1547	2703	3236	3241	3483	2897	3345	1330	985.3	651.3	1045	1245	1458	12
Os03g0747800	338	347	307	448.3	468.3	433.4	488	370	475	293.1	227	357.5	350.5	337.91	267.68	286.6	312.3	304.6	420.8	377.3	423.3	366.7	403.4	427.6	230.3	252.7	372.8	316	277	293	12
Os03g0679800	6.12	4.66	3.23	151.9	181.7	187.7	110	74.6	65	3.449	2.193	7.527	0	0	1.5839	0	8.244	2.072	448.7	337.8	351.8	56.19	36.34	55.61	2.113	0.943	17.54	3.7	2.87	2.5	10
Os03g0851500	273	303	311	356.3	341.7	371.1	336	378	332	445.8	388.1	376.3	309.14	377.11	300.95	573.2	270	313.9	575	403.1	430.2	341.6	320	313.5	364.4	374.3	386	364	401	348	6
Os03g0273800	2034	1684	1976	172.4	104.9	154.8	121	132	191	1456	1529	1661	1008	995.06	1046.2	3917	2483	2158	460.8	305.6	366.4	208.5	267.2	187.1	1856	1908	1838	1429	1706	1773	13
Os03g0411500	751	927	899	1543	1809	1497	1555	1637	1392	694.1	880.4	858	1017.8	925.05	759.5	1922	606	792.6	1486	1818	1549	1048	895.1	1202	709.9	571.4	460.5	606	741	676	12
Os03g0279950	225	362	235	1757	1996	1799	7864	8868	8447	271.6	292.7	619.1	200.29	166.15	135.43	179.1	191.7	188.6	1418	1412	1543	6309	5261	7228	247.2	209.3	254.4	104	116	155	18
Os03g0185800	18.4	10.2	9.7	9.031	15.37	6.92	28.2	41.1	38.7	28.45	15.35	16.93	9.7967	17.736	16.631	35.82	26.79	18.65	17.65	12.9	11.21	20.7	27.79	39	10.56	23.57	17.54	13	12.4	18.3	18
Os03g0141000	1176	868	964	1112	1168	1138	1358	1039	1384	1215	1044	1817	1091.8	1239.6	894.92	1087	878	967.7	1332	1121	1217	1036	964.2	1034	1028	1347	1221	718	815	695	15
Os03g0275700	1.02	0	1.08	0	0	0	0.78	1.27	0.69	0	0	3.763	0	0	0	0	2.061	1.036	0	0	0	1.479	2.138	6.5	0	0.943	0	0	0	0.83	9
Os03g0251425	9.18	9.31	6.47	23.81	36.16	53.63	29.7	27.8	19.3	10.35	10.96	30.11	14.151	11.201	18.215	0	14.43	4.144	26.01	28.22	26.73	18.48	6.414	22.39	10.56	8.486	4.386	7.4	8.61	9.16	12
Os03g0838800	6.12	0.93	6.47	4.926	2.712	1.73	0	1.9	3.46	5.173	1.096	2.822	3.2656	1.8669	0.792	11.94	2.061	3.108	3.716	4.838	2.587	2.958	5.701	0.722	0	0.943	2.193	0	1.91	1.67	6
Os03g0765900	4729	4865	4649	4998	5071	4971	####	####	####	5459	6371	11912	5712.6	5747.3	4715.4	5433	6711	5757	8681	6638	9387	8622	7597	11755	6518	7821	7370	3665	4122	4201	18
Os03g0581600	271	261	248	315.3	320.9	332.2	406	364	438	341.4	321.2	391.4	339.62	341.64	304.11	441.8	287.5	226.9	449.6	317.7	427.7	274.3	342.8	356.1	266.2	347	315.8	261	277	237	15
Os03g0363600	1.02	1.86	1.08	133.8	144.6	153.1	0.78	7.59	6.91	0.862	1.096	1.882	0	4.6673	3.1679	0	1.031	1.036	61.31	56.44	54.32	3.697	5.701	3.611	5.282	0.943	0	0.93	1.91	3.33	10
Os03g0811800	679	576	624	665.8	724.1	724.9	637	618	631	550.1	599.7	753.6	691.21	658.08	635.95	1397	608	567.7	790.5	578.1	660.5	516.8	580.1	606	592.6	694.9	712.7	529	529	551	6
Os03g0227900	78.5	65.2	72.2	31.2	28.02	53.63	6.26	12.7	8.98	35.35	25.22	35.75	30.479	39.205	37.222	179.1	109.2	61.13	45.52	42.73	39.66	39.19	35.63	34.67	55.99	77.31	87.72	74.9	51.6	51.7	13
Os03g0808100	27.5	27.9	20.5	390.8	413.1	316.6	135	133	111	7.76	18.64	3.763	16.328	6.5342	1.5839	0	13.4	20.72	121.7	154.8	115.5	170.8	186.7	147.3	7.395	5.657	10.96	5.55	19.1	6.66	2
Os03g0226600	1289	1315	1194	2423	2832	2181	1343	1713	1659	959.6	1125	777.1	1297.5	999.73	1570.5	3809	1477	1462	1523	2021	1876	2159	2177	1506	1949	1083	828.9	1733	1872	2203	6
Os03g0810900	201	272	205	350.6	339.9	302.8	312	351	305	220.7	267.5	158.1	224.24	231.5	252.64	429.9	237	217.6	253.6	298.3	279.4	325.3	379.8	306.2	266.2	247	217.1	229	279	283	6
Os03g0681300	337	348	315	362	334.5	346.9	593	615	584	495.8	463.8	462.9	414.73	404.18	418.95	346.3	408.1	316	490.5	457.2	532.9	433.3	431.9	507.7	440.5	403.5	515.3	405	354	356	15
Os03g0154300	2.04	3.73	3.23	1.642	4.52	2.595	1.57	1.9	0.69	2.587	4.386	0.941	2.177	0.9335	3.1679	11.94	1.031	2.072	0	0	0	1.479	4.988	0.722	2.113	0	0	4.63	6.69	4.17	6
Os03g0835600	725	792	740	708.5	715	785.5	806	832	860	759.6	679.8	677.4	623.72	738.36	661.29	979.2	754.3	620.6	702.3	598.3	664.8	637.4	684.8	705.6	705.7	694.9	721.5	645	656	604	6
Os03g0843100	438	370	384	193.8	202.5	182.5	199	188	150	422.5	350.8	345.3	329.82	318.31	327.87	167.2	375.1	319.1	216.4	178.2	171.6	185.6	204.5	174.8	294.7	330.9	353.1	293	288	307	3
Os03g0213600	8.16	3.73	1.08	9.852	15.37	13.84	9.39	10.8	14.5	5.173	13.16	1.882	19.593	7.4676	8.7116	0	1.031	3.108	9.29	3.225	8.622	18.48	8.552	15.17	7.395	13.2	15.35	13	6.69	4.17	9
Os03g0197100	5015	4839	4391	1234	1040	1162	636	417	381	4694	4422	5455	2836.7	4622.5	3785.6	5899	5908	4812	2386	1927	2237	406.7	448.2	458.7	6340	8804	9340	6971	5014	5466	3
Os03g0703000	1308	904	733	305.4	233.2	572.7	450	204	277	1085	847.5	1528	501.81	972.66	456.17	656.8	962.5	767.7	743.2	355.6	518.2	449.5	238.7	574.2	705.7	959.8	1642	728	707	542	20
Os03g0707900	551	630	577	743.8	883.2	775.1	764	843	750	580.3	644.7	576.7	596.51	535.8	553.58	1003	567.8	536.7	643.8	685.3	651.8	649.2	627.8	673.9	573.6	597.8	543.8	494	498	527	6
Os03g0714400	919	876	873	905.5	908.5	925.6	1175	1100	1174	1006	1144	1075	1090.7	1150.9	1097.7	1600	1024	967.1	1217	1047	1253	987.8	1061	1117	989.8	1113	1042	895	931	1021	6
Os03g0198200	30.6	14.9	21.6	9.031	5.424	6.92	11	3.16	13.1	23.28	24.12	49.86	16.328	44.806	13.463	47.76	24.73	34.19	10.22	1.613	18.97	17.75	6.414	14.44	28.52	63.17	39.47	21.3	26.8	7.5	3
Os03g0409400	0	0	0	0	0	0	2.35	0	0	0	0	0	0	0	0	0	0	0	0	0	0	0	0	0	0	0	0	0	0	0	5
Os03g0167600	0	1.86	0	0	0	3.46	3.13	1.27	2.07	0.862	0	3.763	0	0	0.792	0	2.061	0	0	0.806	0	4.436	0	0.722	3.169	4.714	0	1.85	1.91	3.33	1
Os03g0683700	357	339	306	352.2	334.5	365.1	244	245	193	213	200.6	257.8	174.16	247.36	219.37	549.3	410.2	346	249.9	245.9	300.1	292.8	290.8	269.4	259.9	267.8	217.1	361	285	258	6
Os03g0192700	1101	1009	1375	21.35	39.77	37.2	83.8	86	159	767.3	689.6	506.2	857.75	887.71	1127	1278	1076	730.4	30.66	15.32	12.07	101.3	121.1	75.11	743.7	445	405.7	825	571	860	3
Os03g0746800	379	447	390	793.1	768.4	850.3	404	431	490	421.6	371.7	452.5	487.66	481.66	376.18	895.6	333.9	474.5	868.6	854.7	839.8	425.1	482.5	373.4	348.6	372.4	377.2	350	379	350	10
Os03g0102300	1.02	0.93	1.08	2.463	0.904	2.595	0	0	0	2.587	0	0	0	0.9335	0	0	0	2.072	0	1.613	0	1.479	2.138	0	2.113	1.886	0	0	0.96	0.83	2
Os03g0381500	17.3	7.45	7.54	18.06	15.37	24.22	62.6	61.4	66.3	37.07	33.99	29.17	10.885	28.937	30.887	0	34.01	18.65	26.01	31.44	33.63	32.53	29.22	41.89	30.63	14.14	24.12	21.3	23.9	23.3	18
Os03g0418733	18.4	18.6	14	20.52	22.6	19.03	14.9	17.1	10.4	2.587	5.482	6.586	11.974	2.8004	16.631	0	6.183	11.4	8.361	16.93	14.66	24.4	17.82	5.778	14.79	3.771	4.386	25.9	25.8	16.7	2
Os03g0643100	0	0	0	0	0	0	0	0	0	0	0	0	0	0	0	0	0	1.036	0	0	0	0	0	0	0	0	0	0	0	0	1
Os03g0660500	2.04	2.79	1.08	0	0	0.865	0	0	0.69	1.724	0	0	1.0885	0	0	0	0	0	0	0	0	12.57	0.713	8.667	1.056	0	0	1.85	0	2.5	9
Os03g0824100	450	734	504	3109	3580	2919	1273	1541	1210	568.2	686.3	573.9	720.6	463.93	422.91	740.3	270	406.1	2163	2658	2556	1105	1077	1052	403.5	271.5	271.9	233	229	352	10
Os03g0383900	0	0	0	0	0	0	2.35	0.63	1.38	0	0	0	0	0	0	0	0	0	0	0	0.862	2.218	0	0	0	0	0	0	0	0	18
Os03g0313100	0	0	0	0	0	0	0	0	0	0	0	0	0	0	0	0	0	0	0	0	0	0	0	0.722	0	0	0	0	0	0	9
Os03g0835100	2770	3871	3526	499.2	526.1	556.2	2735	3454	3029	3511	4377	3336	4476	3483.6	4469.9	5373	2888	3311	417.1	433	300.9	2736	2598	2911	4596	4118	3094	3454	3785	4230	3
Os03g0658800	0	0	0	0	0	0.865	0	0	0	0	2.193	1.882	0	0	0.792	0	0	0	1.858	0.806	0	0	0	0	0	0	0	0	0.96	0.83	20
Os03g0208750	0	0	0	0	0	1.73	0	1.27	0	0.862	0	0	0	0.9335	0	0	1.031	0	2.787	0.806	1.724	0	0.713	1.444	0	1.886	0	0	0	0	16
Os03g0271600	393	361	335	214.3	191.6	179.1	301	328	281	313.8	327.8	301.1	288.46	290.3	312.03	585.1	386.4	385.4	247.1	264.5	266.4	334.2	357.7	444.9	353.9	336.6	396.9	292	325	315	13
Os03g0137350	0	0	0	0.821	0	0.865	2.35	5.69	6.91	12.93	8.771	5.645	4.3541	0.9335	0	23.88	16.49	10.36	7.432	2.419	8.622	1.479	1.425	0.722	3.169	8.486	15.35	15.7	8.61	5	13
Os03g0406250	144	117	99.1	195.4	202.5	226.6	316	212	267	144	146.9	200.4	158.92	174.56	113.25	203	99.96	120.2	243.4	214.5	262.1	153.8	164.6	182	104.6	125.4	116.2	104	89.9	91.6	12
Os03g0857900	382	387	398	469.6	552.3	528.5	2259	2257	2211	642.3	573.4	572	546.44	454.59	483.89	740.3	360.7	394.7	615.9	768.4	641.5	1820	1836	2040	637	451.6	416.7	379	461	518	18
Os03g0405900	454	385	458	364.5	333.6	371.1	278	250	240	391.4	313.6	521.2	308.05	369.65	275.6	489.6	383.4	351.2	350.2	317.7	279.4	216.6	248.7	213.1	314.8	316.8	357.4	233	286	196	20
Os03g0275800	0	0.93	0	0	0	0	0.78	0	1.38	0	0	0	0	0.9335	0	0	2.061	0	0	0	0	0	2.138	1.444	0	0.943	0	0	0	0.83	17
Os03g0103950	314	381	389	470.4	471	397.1	608	621	517	423.3	424.3	376.3	294.99	289.37	342.92	382.1	289.6	285.9	354.9	445.1	304.4	642.5	470.3	512.8	349.7	349.8	414.5	306	331	296	18
Os03g0398250	1.02	0	2.16	0	1.808	2.595	5.48	5.69	3.46	0	0	0	2.177	0	1.5839	0	0	0	0	3.225	0.862	2.218	1.425	0	0	1.886	0	0	0	0.83	18
Os03g0288800	118	125	107	335	356.2	317.5	261	272	211	110.4	123.9	108.2	125.18	123.22	111.67	382.1	111.3	122.3	295.4	243.5	265.6	235.1	239.4	223.2	79.23	118.8	94.3	115	131	108	12
Os03g0818200	9.18	14	20.5	18.88	22.6	16.44	47	33.5	22.1	24.14	19.74	16.93	46.806	12.135	19.799	0	11.34	15.54	14.86	13.71	15.52	31.05	12.11	32.5	6.338	3.771	4.386	10.2	23.9	2.5	18
Os03g0694800	195	179	223	201.1	228.7	202.4	178	179	146	153.5	139.2	115.7	181.78	190.42	140.97	203	157.7	180.3	201.6	213.7	200	149.4	134	120.6	151.1	140.5	107.5	163	184	179	10
Os03g0210700	4518	4028	4722	5470	5740	5532	3189	3424	2845	4476	4575	5462	5875.8	5437.4	4808	10556	5149	4671	7891	8653	7870	1769	1919	2183	4671	3935	3792	4341	4498	4521	16
Os03g0183600	2.04	0	2.16	3.284	0.904	10.38	0.78	3.8	2.76	0	1.096	0	0	4.6673	2.3759	0	2.061	2.072	10.22	6.45	1.724	11.09	4.988	11.56	0	3.771	6.579	0	2.87	1.67	9
Os03g0860900	709	742	686	677.3	687.9	757.8	706	740	687	613.9	672.1	618.1	755.43	675.82	790.38	1099	652.3	713.8	640	707.1	551.8	808.1	782.5	728.7	704.6	679.8	710.5	847	769	805	6
Os03g0336600	176	196	207	128.1	146.4	124.6	177	182	186	191.4	174.3	148.6	205.73	206.29	200.37	226.9	198.9	173	147.7	162.1	147.4	193.7	194.5	159.6	193.3	182.9	236.8	165	142	172	13
Os03g0141800	0	0	0	0	0	0	0	0	0	0	0	0	0	0	0.792	0	0	0	0	1.613	0	0	0	0	0	0	0	0.93	0	0	16
Os03g0284800	104	83.8	65.7	58.29	61.47	58.82	71.2	59.5	50.4	54.32	63.59	134.5	69.665	64.408	60.981	107.5	117.5	93.24	69.67	55.63	63.8	82.07	74.11	91	60.21	98.06	107.5	62.9	82.2	83.3	20
Os03g0380700	0	0.93	0	0	0	0	0	0	0	0	0	0	0	0	0	0	0	0	0	0	0	0	0	0	0	0	0	0	0	0	14
Os03g0708750	8.16	3.73	6.47	3.284	0.904	2.595	2.35	0	1.38	8.622	4.386	13.17	6.5311	13.068	10.296	23.88	5.153	18.65	0.929	1.613	5.173	1.479	2.138	2.167	7.395	14.14	8.772	10.2	8.61	5.83	13
Os03g0427300	17.3	27.9	22.6	3.284	9.944	5.19	1.57	3.16	4.15	8.622	16.45	12.23	14.151	11.201	11.879	35.82	12.37	14.5	0	2.419	3.449	5.176	0.713	5.056	20.07	25.46	10.96	15.7	15.3	20	13
Os03g0373300	2.04	0	0	0	0	0.865	0	0	0	0	0	0	0	0	0	0	0	0	0	0	0	0	0	0	0	0	0	0	0	0	19
Os03g0362200	13.3	29.8	14	204.4	241.4	204.2	185	185	153	28.45	33.99	26.34	42.452	31.737	55.438	35.82	17.52	10.36	104	190.3	100	99.82	128.3	114.8	32.75	16.03	24.12	17.6	27.7	30.8	12
Os03g0347200	607	658	650	862.8	900.4	907.4	479	527	500	604.4	533.9	507.1	687.94	660.88	596.35	1111	618.3	644.4	732.9	747.4	761.3	509.4	565.8	480.3	503.9	610	535.1	661	557	623	6
Os03g0296500	1.02	3.73	3.23	3.284	0	4.325	2.35	3.8	1.38	6.035	4.386	8.467	2.177	0	3.1679	0	5.153	3.108	2.787	2.419	5.173	0.739	2.138	1.444	1.056	4.714	4.386	2.78	2.87	5	20
Os03g0148700	649	484	686	738.1	808.1	610.7	702	763	683	478.5	458.3	245.6	374.45	448.06	480.72	429.9	402.9	454.8	498.8	587.8	585.4	851.8	853	760.5	414.1	452.6	425.4	456	408	413	12
Os03g0611100	680	697	900	167.5	133.8	246.5	9.39	13.9	3.46	418.2	553.7	667	677.06	435.92	696.93	537.3	841.9	686.9	229.5	224.1	603.6	9.612	4.276	13.72	896.9	465.8	633.8	421	268	709	3
Os03g0120501	0	0	0	0	0	0	0	0.63	0	0	0	0	0	1.8669	0	0	1.031	0	0	0	0	0	0	0	0	0	0	0	0	1.67	4
Os03g0421250	10.2	7.45	7.54	7.389	2.712	13.84	5.48	1.27	0.69	8.622	10.96	8.467	6.5311	3.7338	7.9197	11.94	10.31	11.4	9.29	8.869	6.036	12.57	11.4	9.389	5.282	8.486	4.386	3.7	7.65	8.33	19
Os03g0832300	403	346	392	520.5	424.9	449.8	651	546	612	499.2	512	589	423.43	479.79	407.07	979.2	461.7	466.2	705.1	590.2	656.1	554.5	460.4	546	503.9	501.6	561.4	377	419	363	6
Os03g0766900	285	294	280	478.6	528.8	449	427	392	352	269.9	253.3	255	275.4	238.96	233.63	346.3	288.5	247.6	653.1	582.1	604.4	455.5	382	398	253.5	240.4	315.8	251	245	239	10
Os03g0775600	84.6	129	111	96.88	71.41	91.7	18.8	32.3	38.7	83.63	73.46	67.74	125.18	102.68	139.39	119.4	109.2	138.8	35.3	32.25	52.6	45.84	67.7	53.45	113	77.31	76.75	110	86.1	147	8
Os03g0173900	16.3	28.9	15.1	24.63	27.12	12.98	17.2	22.8	10.4	20.69	7.675	16.93	13.062	10.268	11.879	83.59	25.76	23.83	13.01	10.48	8.622	9.612	7.839	14.44	8.451	19.8	13.16	21.3	16.3	20	6
Os03g0704000	2690	2915	2981	5351	6210	4904	7373	7177	6701	2477	3144	3468	3269.9	2819	2440	3690	1793	2549	5340	5468	5244	4723	4180	6182	2422	1644	1397	1536	1777	1961	12
Os03g0383800	2762	2817	2830	2637	2774	2576	1478	1739	1562	2508	2538	2591	2599.4	2544.6	2883.5	4179	2788	3022	2133	2387	2391	2100	2130	1758	2879	2822	2585	3192	2872	3046	13
Os03g0132800	436	356	361	378.5	334.5	371.1	658	686	677	360.4	301.5	333.1	387.51	432.19	419.74	597	380.3	387.5	268.5	258.8	337.1	571.5	562.3	562.6	363.4	360.2	449.5	394	360	391	18
Os03g0719500	1051	1114	1088	976.1	962.7	938.6	797	884	766	898.4	931.9	764.9	1046.1	958.65	1131.7	1648	1094	1083	791.5	776.4	891.5	982.6	916.4	966.4	1012	969.2	912.3	1083	1093	1197	13
Os03g0229000	152	183	150	128.1	133.8	134.9	89.2	94.2	78.1	138.8	140.3	155.2	124.09	130.68	138.59	203	198.9	174.1	108.7	158	160.4	93.9	94.07	110.5	172.2	182	127.2	167	168	204	13
Os03g0383600	1.02	0	0	0.821	0	0	0.78	1.27	2.07	0	0	0	1.0885	0	0	0	0	0	0	0	0	1.479	0	0.722	0	1.886	0	0	0.96	1.67	5
Os03g0365900	1.02	0	0	0	0	0	0	0	1.38	0	0	0	0	0	0	0	0	0	0	0	0	0	0	0	0	0	0	0	2.87	0	1
Os03g0174100	312	413	355	361.2	391.4	393.6	484	519	525	466.4	416.6	440.3	424.52	377.11	397.57	501.5	412.2	409.2	407.8	393.5	423.3	437.7	428.3	471.6	457.4	452.6	401.3	420	399	407	5
Os03g0785300	602	584	577	486.8	496.3	487	746	712	737	735.4	707.2	771.5	615.01	714.09	731.78	1373	671.9	713.8	670.7	644.2	710.5	610.7	597.9	667.4	707.8	797.6	809.2	650	748	657	6
Os03g0806700	33.6	40.1	21.6	15.6	13.56	20.76	11	11.4	15.9	30.18	37.28	44.22	30.479	41.072	39.598	23.88	52.56	29.01	30.66	23.38	26.73	6.654	4.276	15.17	24.3	49.03	24.12	47.2	33.5	25	3
Os03g0153900	3.06	4.66	20.5	21.35	19.89	1.73	22.7	67.7	26.3	12.07	5.482	6.586	11.974	8.4011	13.463	35.82	2.061	7.252	18.58	8.063	0	12.57	39.19	11.56	9.507	0	0	3.7	27.7	27.5	18
Os03g0231150	97.9	95.9	83	38.59	33.45	33.74	135	104	106	50.01	66.88	63.03	57.692	58.807	50.686	95.53	65.95	96.35	23.22	30.64	28.45	96.86	94.78	102.6	60.21	85.8	63.59	47.2	36.3	50	5
Os03g0254800	1162	1060	1119	904.7	843.4	889.3	759	858	1210	1086	973.6	1097	981.84	1077.2	1116.7	907.5	1189	1112	785	668.4	665.6	945.7	1078	980.1	951.8	1058	1180	917	932	862	3
Os03g0757500	10.2	20.5	19.4	18.88	9.944	12.11	3.13	1.9	6.22	10.35	8.771	3.763	4.3541	8.4011	13.463	11.94	3.092	13.47	5.574	7.257	11.21	8.133	2.851	6.5	11.62	13.2	10.96	18.5	16.3	16.7	8
Os03g0821100	1628	1339	1568	1152	1048	1385	686	735	661	1698	1500	2083	1742.7	1912.6	1632.2	4120	1856	1988	1901	1629	1750	412.6	490.3	654.4	1855	1948	1778	1511	1604	1382	13
Os03g0283750	6.12	6.52	5.39	3.284	5.424	3.46	0.78	1.27	1.38	0.862	3.289	0	3.2656	0	4.7518	23.88	3.092	2.072	0.929	2.419	4.311	4.436	3.563	2.167	5.282	1.886	0	4.63	4.78	1.67	6
Os03g0157700	1.02	0	0	1.642	0	0.865	0.78	0.63	2.76	0	0	0	0	0	2.3759	0	0	0	0.929	4.838	4.311	2.958	2.851	2.167	0	0	0	0	0	0	12
Os03g0784700	453	497	539	473.7	511.6	492.2	294	314	357	438	315.8	369.7	242.74	305.24	350.84	704.5	377.2	359.5	458.9	314.4	364.7	315.7	463.2	340.2	329.6	355.5	436.4	372	387	355	6
Os03g0344100	1892	1921	2034	2410	2559	2538	1372	1425	1382	1815	1959	1661	2378.4	2318.7	2117.7	4084	2325	2427	2442	2859	2765	2124	1865	1522	1966	1982	1776	2668	2430	2705	6
Os03g0742900	184	235	289	597.7	693.3	419.5	47	58.2	43.5	100.9	134.9	93.14	250.36	226.83	258.18	214.9	171.1	193.7	349.3	309.6	357.8	58.41	71.26	49.11	162.7	91.46	65.79	158	155	187	10

Os03g0568900	1102	1308	1061	1717	1671	1478	2174	2024	2142	1410	1470	1243	1067.8	1158.4	1070.7	1564	951.2	1014	1381	1451	1298	1717	1931	1880	1237	1189	1208	1109	1026	1049	18
Os03g0607500	5634	5482	6056	2977	2995	2638	5566	6028	5558	6978	8018	7925	9528.9	8527.1	9255.7	9708	7759	6983	3894	3737	4089	3536	3613	4349	8117	8563	7228	6830	7185	7149	3
Os03g0661900	12.2	14.9	18.3	26.27	31.64	27.68	13.3	13.3	15.2	9.484	1.096	9.408	18.505	24.27	12.671	23.88	10.31	13.47	16.72	27.41	14.66	9.612	20.67	11.56	20.07	8.486	2.193	13.9	14.3	8.33	2
Os03g0186900	0	0	0	11.49	11.75	12.11	0	0	0	0	0	0	0	0	0	0	0	0	17.65	8.869	9.484	0	0	0.722	0	0	0	0	0	0	10
Os03g0355900	2546	1904	2648	252.9	207	284.6	257	262	218	2492	2426	2756	2842.1	3325	2882.8	3272	3443	2557	552.7	566.8	588	162.7	182.4	220.3	2401	2966	2960	2269	2382	2034	3
Os03g0689833	22.4	12.1	9.7	26.27	17.18	18.17	9.39	11.4	13.1	13.79	14.25	7.527	15.239	10.268	15.047	11.94	17.52	16.58	14.86	8.063	5.173	44.36	33.49	37.56	13.73	17.91	15.35	29.6	15.3	29.2	9
Os03g0104400	488	441	462	505.7	503.5	489.6	557	531	555	500.1	461.6	508	475.68	432.19	410.24	597	420.5	502.5	507.2	497.5	471.6	530.9	540.9	457.2	499.7	538.4	550.4	438	462	432	6
Os03g0422900	5.1	10.2	16.2	26.27	30.73	22.49	13.3	31.6	33.9	8.622	9.868	3.763	6.5311	2.8004	13.463	23.88	10.31	10.36	16.72	15.32	18.97	29.58	37.77	22.39	12.68	9.428	4.386	13	13.4	15.8	12
Os03g0648900	408	371	456	393.2	411.3	447.2	455	412	464	457	400.2	509.9	477.86	553.54	441.13	979.2	483.3	476.6	586.2	557.9	653.6	329.8	357.7	348.8	464.8	453.5	388.1	389	408	340	6
Os03g0789500	323	319	279	384.2	366.1	353.8	253	276	293	314.7	369.5	349	253.63	282.84	328.67	429.9	267.9	274.5	370.7	437	375.9	269.9	342.1	273.7	372.9	315.9	379.4	370	384	327	16
Os03g0760900	10.2	8.38	16.2	0.821	2.712	5.19	11	9.49	6.91	5.173	0	11.29	9.7967	5.6007	9.5036	11.94	14.43	3.108	9.29	7.257	1.724	7.394	4.276	3.611	4.225	11.31	10.96	5.55	2.87	5.83	14
Os03g0181750	13.3	20.5	8.62	23.81	28.02	22.49	4.7	17.7	12.4	12.07	21.93	8.467	7.6196	17.736	12.671	0	7.214	18.65	17.65	13.71	7.76	8.133	15.68	13.72	20.07	19.8	4.386	14.8	20.1	18.3	2
Os03g0123100	808	683	637	613.3	609.3	607.3	996	834	960	752.7	699.5	984.1	648.76	831.71	697.72	1111	717.2	699.3	675.3	614.4	588.9	704.6	770.4	929.5	758.5	844.8	1011	718	640	562	5
Os03g0441400	39.8	52.2	52.8	149.4	154.6	150.5	57.9	82.2	81.5	31.04	29.6	29.17	32.656	23.336	46.726	47.76	31.95	39.37	125.4	95.95	70.7	89.47	91.22	57.06	30.63	29.23	21.93	50	45.9	51.7	10
Os03g0300900	9.18	3.73	3.23	2.463	2.712	13.84	15.7	8.86	2.76	4.311	0	1.882	5.4426	5.6007	0.792	35.82	9.275	1.036	13.01	6.45	26.73	3.697	7.126	11.56	2.113	3.771	32.89	0.93	7.65	3.33	6
Os03g0197000	6894	5473	6936	164.2	99.44	133.2	124	111	108	5689	6928	5650	6059.8	5401.9	6832.3	9839	6293	5773	242.5	193.5	230.2	135.3	146.1	187.8	7298	7661	7436	5695	5877	6319	3
Os03g0216900	0	3.73	1.08	9.031	6.328	8.65	11.7	8.86	4.15	6.035	4.386	0	2.177	1.8669	3.9598	0	2.061	1.036	8.361	15.32	10.35	3.697	0.713	2.889	1.056	0.943	0	1.85	0.96	0.83	12
Os03g0748600	19.4	46.6	19.4	26.27	19.89	23.36	29.7	27.2	27.6	18.11	20.83	43.28	11.974	12.135	19.799	11.94	22.67	31.08	43.66	35.48	40.52	42.15	35.63	39.72	20.07	24.51	32.89	15.7	17.2	10	9
Os03g0595300	2888	2719	3051	1924	1986	1908	4335	3912	4161	4026	4179	6781	3689	3576.1	3300.1	4621	4486	3749	3315	2454	2968	2382	2165	3265	4364	5294	4971	2625	2961	2816	20
Os03g0683833	13.3	11.2	19.4	10.67	10.85	14.71	21.9	26.6	28.3	13.79	20.83	21.64	19.593	14.935	18.215	47.76	15.46	18.65	14.86	21.77	25.87	14.05	13.54	18.78	15.85	31.11	30.7	16.7	12.4	13.3	6
Os03g0804300	673	668	727	600.1	648.1	612.5	716	798	732	724.2	558.1	559.8	655.29	635.68	621.69	919.5	586.4	639.2	558.3	640.2	527.7	675.8	672.7	712.1	607.4	509.1	546	635	549	556	6
Os03g0207300	219	255	265	459.7	474.6	444.6	300	278	309	245.7	247.8	237.1	223.15	212.83	268.48	226.9	252.5	270.4	329.8	355.6	405.2	295	239.4	270.8	265.1	223.5	232.4	235	258	272	10
Os03g0712400	247	145	228	133	143.7	124.6	120	136	96.7	168.1	173.2	227.7	225.32	251.1	239.97	513.5	306.1	250.7	157	167.7	181.9	95.38	105.5	129.3	253.5	273.4	203.9	258	210	212	13
Os03g0828100	902	1207	1027	3261	3767	2882	5100	5194	4705	1170	1495	1900	966.61	673.95	545.66	812	521.4	661	2847	3286	3260	2730	2368	3986	723.6	605.3	585.5	291	361	371	12
Os03g0624800	346	319	334	350.6	331.8	331.3	379	346	372	338.8	364	360.3	388.6	366.85	326.29	740.3	404	386.4	315.8	354.8	413.9	357.1	336.4	347.4	365.5	425.2	348.7	382	406	409	6
Os03g0188200	3.06	4.66	10.8	0	0	2.595	0.78	0.63	2.76	6.035	1.096	14.11	1.0885	4.6673	0.792	11.94	6.183	12.43	1.858	0	0	0	0.713	0.722	9.507	1.886	0	0.93	0.96	0	13
Os03g0210100	1.02	0	0	4.105	4.52	0	2.35	0.63	2.07	0	0	0	1.0885	0	0	0	0	0	0	0	1.724	0	1.425	4.333	0	0	0	0	0	2.5	2
Os03g0320600	19.4	17.7	21.6	29.56	29.83	26.82	19.6	26.6	23.5	18.11	36.18	16.93	15.239	23.336	15.839	23.88	29.89	13.47	17.65	32.25	26.73	35.49	29.93	41.89	11.62	16.97	19.74	16.7	19.1	12.5	9
Os03g0386000	658	572	683	335	200.7	260.4	168	87.9	252	325	345.4	161.8	628.08	358.45	524.28	1911	785.3	563.6	300.1	320.9	199.2	258	295	259.3	403.5	130.1	151.3	513	422	463	13
Os03g0324600	3.06	8.38	11.9	10.67	6.328	6.055	9.39	2.53	13.1	14.66	3.289	2.822	8.7082	0.9335	3.1679	0	4.122	5.18	6.503	4.031	13.8	16.27	9.264	13	6.338	7.543	4.386	2.78	2.87	6.66	9
Os03g0356700	1427	1562	1351	834.1	791.9	955.9	622	636	511	819.9	879.3	893.8	805.5	1053.9	921.06	1934	1582	1350	723.7	709.5	723.4	943.5	928.6	790.1	1094	1036	1289	1538	1197	1300	13
Os03g0405400	0	2.79	0	0	0.904	3.46	0	1.27	0.69	0	0	0.941	0	0	1.5839	23.88	2.061	1.036	0.929	1.613	1.724	1.479	4.276	5.056	2.113	0	0	4.63	8.61	3.33	6
Os03g0578200	669	686	734	777.5	913	731	987	983	963	664.7	655.6	757.4	730.4	687.95	643.87	895.6	634.8	628.9	856.5	861.9	931.2	672.8	644.2	808.2	637	672.2	629.4	522	579	551	12
Os03g0837800	309	262	295	380.1	394.1	374.6	75.2	51.2	64.3	237.1	257.7	170.3	346.15	358.45	327.08	525.4	266.9	285.9	344.6	401.5	419.9	80.59	104	103.3	231.3	236.7	236.8	286	265	257	16
Os03g0342850	30.6	39.1	35.6	65.68	48.81	57.96	123	137	106	37.94	37.28	63.98	58.78	28.004	29.303	71.65	51.53	25.9	84.53	78.21	86.22	82.81	66.99	117	25.35	28.29	59.21	24.1	39.2	33.3	18
Os03g0170400	467	466	529	523	540.6	493.1	258	280	247	306.9	252.2	262.5	299.34	304.31	295.4	346.3	492.6	453.8	409.7	420.9	450.9	346	349.2	323.6	269.4	293.2	296	405	357	426	8
Os03g0115350	10.2	8.38	20.5	13.14	32.54	7.785	9.39	15.8	15.9	16.38	7.675	14.11	13.062	8.4011	18.215	35.82	12.37	10.36	26.01	41.93	18.97	15.53	20.67	13	17.96	9.428	4.386	16.7	9.56	20	6
Os03g0313300	88.7	54	57.1	9.852	9.04	17.3	9.39	4.43	7.6	15.52	13.16	15.05	10.885	27.07	15.047	203	83.47	70.45	25.08	16.13	16.38	27.36	39.19	53.45	11.62	32.06	46.05	22.2	30.6	20.8	13
Os03g0237500	202	127	162	19.7	8.136	12.11	3.13	5.69	19.3	215.5	189.7	207.9	87.082	65.342	106.12	71.65	72.14	77.7	39.94	18.54	23.28	15.53	8.552	12.28	174.3	119.7	140.3	64.8	66	54.2	3
Os03g0685750	36.7	29.8	35.6	36.12	18.08	32.87	81.4	55.7	66.3	59.49	52.63	47.04	41.364	38.272	34.847	35.82	30.92	39.37	58.52	38.7	45.7	35.49	27.79	41.89	43.31	49.03	43.86	29.6	45.9	33.3	15
Os03g0827900	127	120	103	53.36	47.91	57.96	154	102	125	151.7	162.3	180.6	111.03	125.08	78.405	59.7	165.9	114	78.96	64.5	79.32	102.8	121.1	137.9	169	164.1	208.3	151	119	128	20
Os03g0597800	3.06	0	5.39	0	0	0	6.26	0	1.38	2.587	0	1.882	4.3541	0	1.5839	0	0	2.072	1.858	1.613	3.449	0.739	0	1.444	3.169	2.829	2.193	0.93	0.96	0	5
Os03g0196450	1.02	0.93	0	0	0	0	1.57	0	0.69	0	1.096	0.941	0	0	0	0	0	0	0	0	0	0.739	0	0	0	1.886	2.193	0	0	0.83	11
Os03g0710800	2944	2665	3012	1504	1518	1843	3396	2800	3128	3541	3412	3926	2982.5	3705.8	3147.3	3821	3517	3204	2610	2509	2544	2562	2863	3286	3680	3984	4528	3488	3094	2853	3
Os03g0162400	0	0.93	0	0.821	0	0	0	0	0	0	0	0	3.2656	0	0	0	0	1.036	0	0	0	0	0.713	0	2.113	0	0	0.93	0	0	4
Os03g0826600	5746	5498	5534	6045	6015	5799	4888	5810	4431	6032	5419	5755	5641.8	6267.2	6666.8	8717	6527	6042	6322	7486	6338	4135	5659	4982	6690	6269	6081	7808	6872	6406	13
Os03g0757400	13.3	15.8	24.8	7.389	16.27	17.3	14.9	11.4	13.8	23.28	33.99	24.46	16.328	29.87	15.839	11.94	12.37	23.83	23.22	17.74	15.52	18.48	14.97	8.667	17.96	14.14	8.772	18.5	23	15.8	7
Os03g0816100	1471	1438	1432	870.2	800.9	891	819	875	818	1329	1440	1064	1195.2	1257.4	1269.5	2388	1283	1342	789.6	969.2	809.6	1077	1194	983	1333	1384	1476	1535	1552	1537	13
Os03g0235900	509	490	633	370.3	438.4	359	344	410	370	308.7	402.4	284.1	548.61	479.79	499.73	883.6	331.8	426.8	262.9	293.5	246.6	356.4	348.5	331.5	368.7	310.2	254.4	440	419	486	13
Os03g0711800	1067	1196	1134	1184	1197	1134	1314	1265	1394	1393	1254	1248	1369.4	1295.6	1265.6	1755	1106	1079	990.3	1138	1017	1327	1256	1165	1253	1101	1112	1139	1077	1171	6
Os03g0791500	526	469	538	445.8	409.5	423	479	440	512	491.4	509.8	521.2	604.13	568.47	694.55	943.3	463.7	394.7	417.1	387.8	383.7	440.7	370.6	452.1	581	533.6	554.8	530	498	528	13
Os03g0834300	2.04	0	1.08	0	0	0	7.05	3.8	1.38	0.862	0	0	1.0885	1.8669	0.792	0	1.031	2.072	0	0	0.862	1.479	1.425	2.889	2.113	0	6.579	0	2.87	0	5
Os03g0837900	546	363	527	179	195.3	224	36.8	17.7	43.5	582	603	725.4	833.81	728.09	710.39	776.2	628.6	589.5	334.4	348.3	388.9	28.84	24.23	28.89	724.7	785.4	651.3	678	615	634	3
Os03g0279500	0	0	0	0	0	0.865	0	0	0	0	0	0.941	0	0	0	0	0	0	0	0	0	0	0	0	0	0	0	0	0	0.83	20
Os03g0857400	####	####	####	14232	12938	15405	3388	3598	3182	27699	28728	29708	28429	27822	27806	38247	23078	20991	18568	17669	22179	1908	1953	2313	27801	29583	25449	####	####	####	3
Os03g0300000	46.9	41.9	50.6	22.99	16.27	32.01	14.1	41.7	25.6	18.11	19.74	35.75	28.302	35.471	26.135	59.7	53.59	54.91	17.65	17.74	18.11	20.7	37.06	33.95	27.47	44.31	43.86	32.4	44.9	28.3	13
Os03g0821900	313	318	364	293.9	304.6	295	277	341	289	251.8	254.4	216.4	351.59	283.77	309.66	370.2	293.7	326.4	190.4	217.7	212.1	320.2	281.5	277.3	278.9	284.7	182	278	263	315	8
Os03g0656900	1685	1997	1775	5343	5765	4916	4112	4333	3737	1325	1942	1773	2408.9	1898.6	1132.5	2436	1213	1938	5992	6949	6898	3018	2996	3516	1048	799.5	824.5	1171	1235	1267	10
Os03g0756400	1534	1515	1595	1138	1021	1155	1494	1288	1236	1939	2031	2009	2133.5	2018.1	2014	2364	1628	1676	1549	1514	1561	1125	1114	1333	1945	1894	1882	1579	1430	1575	3
Os03g0609400	51	41	50.6	64.86	55.14	46.71	39.1	38	40.1	44.83	43.86	33.87	30.479	37.338	36.43	47.76	37.1	96.35	54.81	49.18	91.4	41.41	37.77	37.56	42.25	43.37	35.09	50	43	63.3	16
Os03g0110900	3261	3254	3471	4528	4423	4714	189	249	254	3986	4594	4485	5026.8	4627.1	4749.4	7845	4213	4144	5591	6014	6424	187.8	210.9	206.6	5154	4959	3919	4428	4651	4776	16
Os03g0822200	3278	2846	3562	2179	2344	2383	1699	2032	1520	3874	3741	4270	5565.6	5225.5	4620.3	4824	3196	2908	2696	2633	2452	1370	1626	1459	3496	3252	3467	3105	3238	3421	3
Os03g0286900	35.7	21.4	35.6	8.21	4.52	2.595	1.57	10.8	6.22	9.484	14.25	3.763	83.816	17.736	23.759	59.7	13.4	10.36	3.716	1.613	2.587	0.739	5.701	4.333	12.68	0.943	6.579	11.1	7.65	8.33	13
Os03g0794400	2.04	1.86	0	1.642	3.616	3.46	3.13	3.16	4.84	2.587	2.193	0.941	4.3541	3.7338	5.5438	0	2.061	2.072	2.787	0	0.862	0.739	1.425	4.333	0	0.943	2.193	5.55	2.87	1.67	4
Os03g0808600	304	273	298	282.4	296.5	274.2	611	452	437	569	409	424.3	355.95	426.59	392.02	119.4	293.7	270.4	424.5	408.8	346.6	329	409.1	372.7	402.5	358.3	375	306	381	327	15
Os03g0850300	95.8	83.8	99.1	107.5	88.59	91.7	113	114	133	151.7	137	158.1	126.27	101.75	126.71	191.1	84.5	90.14	111.5	115.3	120.7	67.28	85.52	82.34	134.2	108.4	136	86	104	84.1	6
Os03g0118900	108	156	170	156.8	202.5	173.9	167	140	147	145.7	164.5	188.2	160.01	115.75	147.31	179.1	121.6	111.9	188.6	217.7	216.4	120.5	119.7	154.6	138.4	161.2	160.1	136	154	112	16
Os03g0206700	526	645	599	184.7	219.7	196.4	520	707	674	582.8	768.6	663.3	827.28	722.49	836.32	883.6	595.6	791.5	205.3	204.8	197.4	573.8	538	561.9	761.6	586.4	565.8	555	588	814	3
Os03g0281900	0	5.59	9.7	0.821	0.904	3.46	0	0	0	0	1.096	0	6.5311	0.9335	0	0	0	0	6.503	1.613	0	0	1.425	0	0	0	0	0	0	2.5	14
Os03g0576600	57.1	63.3	45.3	27.09	40.68	29.41	7.05	18.3	4.15	63.8	69.07	196.6	64.223	142.82	70.485	167.2	126.8	91.17	52.02	37.09	76.74	9.612	11.4	5.778	73.95	113.1	114	81.4	102	65	13
Os03g0205800	18.4	19.6	23.7	16.42	19.89	15.57	7.83	9.49	6.91	14.66	6.578	16.93	17.416	17.736	12.671	47.76	18.55	18.65	18.58	34.67	25	8.873	9.264	5.778	12.68	23.57	6.579	14.8	22	10.8	6
Os03g0227500	138	114	124	137.1	141	115.1	152	161	138	128.5	103.1	113.8	112.12	106.41	135.43	131.4	107.2	100.5	153.3	150	136.2	125	118.3	142.3	184.9	119.7	98.68	140	135	130	12
Os03g0190500	0	0	0	0	0	1.73	0	0	0.69	2.587	0	0	0	0	0	0	0	0	0	0	0	5.915	3.563	7.222	0	0	0	0	0	0	9
Os03g0173500	513	445	530	538.6	563.2	602.1	228	306	304	482	497.8	430.9	610.66	603.01	460.13	823.9	528.7	581.2	415.2	525.7	485.4	515.4	421.9	307	514.4	594.9	464.9	636	712	642	13
Os03g0148400	101	124	86.2	21.35	16.27	23.36	17.2	20.2	8.29	24.14	26.31	31.99	27.213	26.137	20.591	95.53	94.81	68.38	26.94	20.96	20.69	24.4	28.51	30.33	41.2	41.49	37.28	27.8	20.1	26.7	14
Os03g0648800	1.02	0.93	1.08	0	0	0	0	0	0	0	0	4.704	0	0	0	0	1.031	0	0	0	0	0	0	0	0	0.943	2.193	2.78	0.96	0.83	20
Os03g0717900	1490	996	1574	507.4	487.2	667.8	103	116	115	2002	1923	1822	3075.1	2754.6	2951.7	1672	1713	1281	1196	1401	1173	86.51	108.3	71.5	1684	1428	1364	1917	1782	1703	3
Os03g0411800	249	332	378	523.8	498.1	274.2	2003	1762	1754	1048	660	605.9	494.19	339.78	655.75	417.9	156.6	222.7	441.3	479.7	375.9	1179	1349	1088	442.6	341.3	355.3	268	359	324	18
Os03g0261100	1068	788	1045	374.4	535.1	546.7	158	161	292	687.2	788.3	875.9	892.59	1131.3	852.16	1337	1159	953.2	589	462.8	714.8	136.8	72.69	185.6	724.7	725	622.8	542	677	591	13
Os03g0249400	256	168	149	298	299.2	332.2	294	211	285	166.4	140.3	350.9	164.37	193.22	118	537.3	177.3	165.8	409.7	379.8	417.3	173	172.5	227.5	147.9	221.6	195.2	135	156	120	6
Os03g0599400	0	0.93	1.08	0	0	0	0	0	0	0	0	0	0	0	0	0	0	0	0	0	0	0	0	0	0	0	0	0	0	0	14
Os03g0110800	9.18	8.38	4.31	1.642	5.424	2.595	11	2.53	2.76	6.897	1.096	13.17	3.2656	4.6673	4.7518	11.94	7.214	7.252	3.716	4.838	0.862	2.958	2.138	3.611	5.282	16.97	6.579	5.55	2.87	8.33	20
Os03g0655500	0	0	0	0	0	0	0	0	0	1.724	2.193	0	0	1.8669	0	0	0	0	0	0	0	0	0	0	0	1.886	0	0	0.96	0	7
Os03g0767500	2388	2397	2994	2886	3104	2997	3244	3913	3626	3215	3562	2515	5132.4	4362	4583.1	5612	2797	2617	3262	3485	3104	3335	3433	3552	3459	2869	2888	3425	3178	3930	6
Os03g0829100	563	499	571	556.6	595.7	575.3	784	769	815	631.1	584.4	700	637.87	561	582.89	740.3	563.7	595.7	689.3	669.2	704.4	542.7	604.3	606.7	620.1	562.9	625	565	533	521	15
Os03g0263500	460	453	497	511.5	507.1	487.9	464	454	462	502.7	410.1	490.2	473.51	402.32	425.29	883.6	431.8	477.6	465.4	431.4	450.9	465.8	406.9	496.2	474.3	479	414.5	395	389	452	6
Os03g0849800	156	183	163	75.53	79.55	77.85	65.8	64.5	47	101.7	95.39	85.61	102.32	86.811	73.653	298.5	198.9	153.3	61.31	60.47	60.36	113.1	113.3	78.72	144.7	95.23	98.68	179	164	197	13
Os03g0309250	0	0	0	0	0	0	0.78	0	0	0	0	0	0	0	0	0	0	0	0	0	0	0	0	0	0	0	0	0	0	0	5
Os03g0724700	244	228	246	137.1	95.82	121.1	91.6	72.7	69.1	250	234.6	178.8	215.53	229.63	230.46	274.6	185.5	172	177.4	177.4	140.5	119	152.5	130.7	279.9	202.7	269.7	225	213	204	3
Os03g0736300	484	376	383	476.2	322.7	899.6	204	189	187	331.9	291.6	294.5	242.74	304.31	270.85	585.1	475.1	324.3	406	292.7	501.8	385.2	542.3	705.6	303.2	436.5	943	356	516	334	11
Os03g0797902	0	0	0	1.642	2.712	1.73	0	5.06	0	0.862	0	0	0	0	0	0	0	0	0	0	0	0	0	2.167	0	0	0	0	0	0	2
Os03g0166800	691	651	687	701.9	713.2	721.5	647	603	549	651	667.7	632.2	716.25	697.29	696.93	1325	742	640.3	654.9	748.2	753.6	691.3	623.6	656.5	681.4	645.8	745.6	695	686	712	6
Os03g0394900	30.6	29.8	17.2	38.59	42.49	38.93	99.4	96.8	65	37.94	33.99	19.76	28.302	18.669	34.847	23.88	16.49	24.86	35.3	43.54	31.9	56.19	68.41	44.06	22.18	22.63	41.67	24.1	27.7	10.8	18
Os03g0759375	0	0	0	0	0	0	0	0	0	0.862	0	0	0	0	0	0	0	0	0	0	0	0.739	0	0	0	0	0	0.93	0	0	7
Os03g0639200	154	221	195	172.4	173.6	170.4	55.6	55.7	54.6	68.97	117.3	46.1	134.98	97.079	132.26	262.7	131.9	130.5	46.45	63.7	50.87	121.3	114	78.72	115.1	91.46	67.98	180	156	206	8
Os03g0568600	0	0.93	0	0.821	0	0.865	0	0	2.07	0	0	0	0	0	0	0	0	0	0	5.644	0.862	0	0.713	0	0	0	0	2.78	0	0.83	16
Os03g0339100	888	888	820	967.9	1065	997.4	1235	1172	1304	935.5	1024	986	1194.1	1056.7	1026.4	1612	948.1	849.6	1008	1042	1083	1145	1014	1156	1010	1006	1156	889	865	892	6
Os03g0849600	388	401	414	574.7	528.8	627.2	520	463	547	438	381.5	396.1	421.26	451.79	436.37	668.7	396.8	412.3	533.2	549.1	550.1	475.4	467.5	528.7	375	394.1	473.7	451	471	407	10
Os03g0204300	283	281	280	241.4	219.7	251.7	450	310	431	435.4	390.3	382	322.2	301.5	293.03	274.6	349.4	366.8	461.7	316.1	407	279.5	270.1	317.1	353.9	368.7	386	301	335	319	15
Os03g0817100	22.4	25.1	28	20.52	22.6	19.03	110	125	131	49.14	29.6	26.34	39.187	35.471	52.27	23.88	27.82	13.47	27.87	28.22	19.83	151.6	125.4	158.9	34.86	30.17	70.17	36.1	28.7	31.7	18
Os03g0568500	671	713	642	766.8	759.3	796.7	820	984	1053	887.2	689.6	883.4	758.7	717.82	749.99	823.9	601.8	611.3	773.8	658.7	674.3	941.2	1047	889.1	764.8	738.2	747.8	564	598	751	18
Os03g0607700	220	243	237	206.9	209.7	194.6	187	202	216	204.3	206.1	302.9	329.82	299.64	296.99	298.5	248.4	305.6	226.7	179	256.1	188.5	150.4	221	138.4	237.6	258.8	249	187	285	13
Os03g0844500	337	367	357	316.1	329	326.1	301	326	288	322.5	339.9	279.4	342.88	353.78	392.02	585.1	344.2	366.8	249	310.4	272.5	354.2	332.1	329.3	346.5	302.7	339.9	417	359	452	13
Os03g0173700	916	1037	876	1221	1370	1111	475	593	526	855.3	835.5	665.2	880.61	853.17	811.77	1648	1012	978	775.7	778.1	901	610	505.3	442.7	834.5	843.8	528.5	853	884	947	8
Os03g0839800	3.06	0	2.16	2.463	0	3.46	0	0	0	1.724	2.193	0	1.0885	0	0	0	1.031	0	0	0.806	2.587	0.739	0.713	0.722	1.056	0	10.96	0	1.91	4.17	11
Os03g0334100	279	324	358	632.2	678.9	562.3	838	895	775	376.8	367.3	499.6	490.92	370.58	426.08	489.6	329.8	385.4	692.1	724.8	695.8	607	623.6	609.6	428.9	343.2	372.8	355	442	407	12
Os03g0125600	333	289	295	364.5	318.2	355.5	536	545	648	332.8	357.4	437.5	370.1	339.78	374.6	226.9	314.3	356.4	317.7	273.3	297.5	463.6	480.3	544.6	359.2	423.3	410.1	278	303	285	18
Os03g0238600	393	377	391	301.3	233.2	317.5	324	280	176	195.7	243.4	138.3	237.3	259.5	277.19	322.4	382.3	306.7	326.1	268.5	271.6	260.3	322.8	314.9	269.4	230.1	250	564	540	472	8
Os03g0673700	234	237	353	273.4	124.7	184.3	376	346	390	289.7	268.6	348.1	254.71	278.17	292.26	262.7	376.1	306.7	415.2	237	356.1	259.5	301.4	350.3	253.5	236.7	271.9	261	239	274	15
Os03g0396300	117	105	127	46.8	38.87	42.39	63.4	56.3	44.2	63.8	82.23	79.97	106.67	61.608	77.613	274.6	87.6	70.45	15.79	26.61	21.56	77.64	79.82	39	66.55	35.83	52.63	54.6	88.9	91.6	13
Os03g0586700	1298	1243	1326	1631	1738	1595	1417	1729	1535	1098	1027	945.5	1239.8	1063.2	1177.7	2054	1147	1195	1351	1661	1355	1587	1469	1491	1043	886.3	885.9	1316	1209	1330	6
Os03g0853700	379	421	363	402.3	342.6	361.6	287	299	291	390.6	410.1	442.1	443.03	381.78	410.24	585.1	419.4	427.9	312.1	321.7	360.4	366	377	322.8	470.1	529.9	458.3	402	406	451	13
Os03g0838400	34.7	51.2	66.8	30.38	29.83	38.06	42.3	89.2	26.3	158.6	71.27	78.09	63.134	45.739	116.42	95.53	42.25	51.8	46.45	77.4	27.59	28.84	62	26.72	95.07	64.11	57.02	46.3	94.7	58.3	7
Os03g0223700	1258	1338	1288	1283	1334	1349	1238	1359	1214	1129	1176	1104	1206.1	1232.2	1296.2	1516	1376	1339	1078	1142	1128	1596	1474	1452	1308	1255	1208	1448	1369	1421	8
Os03g0808300	97.9	112	78.7	150.2	153.7	164.4	896	618	799	207.8	143.6	184.4	94.701	121.35	103.75	83.59	106.1	106.7	176.5	209.6	214.7	413.3	475.3	454.3	92.96	106.5	142.5	79.6	105	90	18
Os03g0741100	4100	6598	3025	1109	590.3	1300	1058	1842	1336	913.1	695.1	486.4	720.6	729.03	953.53	1337	1262	1776	1423	1861	1593	2583	2203	1625	801.8	732.6	1465	994	1057	851	14
Os03g0728900	282	308	274	334.1	318.2	261.2	243	262	244	302.6	337.7	245.6	205.73	230.56	283.52	310.5	347.3	323.2	239.7	293.5	250	437.7	612.2	362.6	494.4	435.6	399.1	547	347	384	1

Os03g0165375	1.02	3.73	5.39	26.27	26.22	18.17	29.7	39.2	38	6.897	3.289	0	3.2656	7.4676	3.9598	0	2.061	5.18	20.44	17.74	14.66	14.79	17.82	15.89	9.507	0	0	0	7.65	5	12
Os03g0831300	179	174	177	192.1	217	181.7	170	166	149	196.6	182	183.5	216.62	201.63	197.2	346.3	208.2	237.3	182.1	225	213.8	180.4	158.2	169.7	197.5	210.3	184.2	218	224	227	13
Os03g0323900	5.1	6.52	10.8	5.747	11.75	7.785	14.9	13.9	15.9	8.622	9.868	7.527	14.151	9.3345	7.1277	11.94	24.73	11.4	11.15	4.031	11.21	19.96	12.11	9.389	11.62	14.14	19.74	5.55	9.56	9.16	5
Os03g0180000	429	412	411	351.4	341.7	314.9	263	288	335	363.8	358.5	239	370.1	292.17	369.85	573.2	332.9	337.7	258.2	296.7	257.8	356.4	384.8	354.6	365.5	297	318	323	333	392	13
Os03g0682600	1.02	0	1.08	0	0	0	0	0	0	0	0	0	0	0	0	0	1.031	0	0	0	0	0	0	0	0	0	0	0	0.96	0	19
Os03g0570100	0	0	0	0	0	0	0	0	0	0	0	0.941	0	0.9335	1.5839	0	0	0	0	0	0	0	0	0	0	0	0	0.93	0	0	4
Os03g0109500	195	162	149	219.2	191.6	250.9	239	214	298	248.3	220.4	457.2	225.32	202.56	184.53	406	146.3	151.3	289.8	196.7	294.9	163.4	178.2	194.3	180.6	256.5	291.7	99	164	127	20
Os03g0370600	25.5	23.3	12.9	64.04	63.28	73.53	6.26	7.59	14.5	30.18	7.675	23.52	30.479	27.07	16.631	47.76	26.79	29.01	64.1	59.66	51.73	37.71	16.39	10.11	12.68	27.34	39.47	11.1	12.4	17.5	10
Os03g0251600	0	3.73	1.08	1.642	3.616	6.92	1.57	3.8	9.67	2.587	2.193	0	0	2.8004	4.7518	0	4.122	1.036	2.787	2.419	1.724	5.915	6.414	2.167	2.113	2.829	2.193	3.7	1.91	1.67	17
Os03g0839400	0	0	0	0	0.904	0.865	0	0	0	0	0	0.941	0	1.8669	0.792	0	1.031	1.036	3.716	0	0.862	0	0.713	2.167	0	0	0	0	0	0	16
Os03g0213400	1213	1251	1223	1516	1553	1405	1357	1519	1347	1304	1226	1257	1173.4	1154.7	1265.6	1731	1119	1109	1318	1325	1263	1393	1472	1255	1261	1224	1088	1283	1262	1216	6
Os03g0283600	1612	1351	1597	994.2	942.8	1019	389	283	363	1342	1341	1561	1545.7	1508.5	1528.5	2937	1959	1868	1580	1499	1349	332	280.8	369.1	1489	1444	1647	1632	1641	1520	13
Os03g0836800	5.1	11.2	8.62	4.105	1.808	1.73	1.57	4.43	4.15	4.311	14.25	11.29	13.062	8.4011	15.047	0	24.73	12.43	6.503	6.45	8.622	3.697	1.425	2.889	17.96	16.03	24.12	6.48	8.61	5.83	3
Os03g0828600	0	0	0	0	0	0	0	0	0	0	0	0	0	0	0	0	0	0	0	0	0	0	0	1.444	0	0	0	0	0	0	9
Os03g0191400	196	150	184	142.8	116.6	141.9	157	144	168	234.5	194.1	195.7	150.22	196.02	216.21	274.6	178.3	151.3	167.2	126.6	130.2	214.4	240.2	182.7	157.4	187.6	252.2	202	187	211	13
Os03g0248000	921	952	900	837.4	893.1	792.4	846	959	781	745.8	762	686.8	963.34	862.51	892.55	1696	1031	1007	820.3	950.6	829.5	774.9	815.3	728	803.9	653.4	655.7	1069	1010	1144	6
Os03g0652100	0	0	0	0	0	0	0	0	0	0	0	0	0	0	0	0	0	0	0	0	0.862	0	0	0	0	0	0	0	0	0	16
Os03g0571900	835	647	778	293.9	277.5	301	210	182	202	313.8	371.7	414.9	417.99	614.21	503.69	859.7	739.9	658.9	445.9	352.3	338.9	407.4	421.2	353.2	461.6	439.4	491.2	781	709	647	13
Os03g0214000	68.3	87.6	90.5	130.5	150.1	93.43	54.8	96.1	88.5	44.83	59.21	35.75	81.639	49.473	65.733	95.53	50.5	50.77	57.6	64.5	51.73	97.6	90.5	77.28	41.2	41.49	67.98	75.9	73.6	77.5	2
Os03g0721900	109	56.8	78.7	40.23	43.39	33.74	37.6	32.3	40.1	93.12	86.62	148.6	66.4	58.807	59.397	143.3	63.89	48.69	75.25	58.05	71.56	32.53	34.21	39	47.54	76.37	85.52	45.3	58.3	40	20
Os03g0726400	795	958	812	468	579.4	502.6	531	722	600	859.6	983.5	825.1	859.93	813.97	960.65	1039	681.2	895.1	503.5	497.5	452.7	609.3	699.1	660.1	866.2	858	666.6	696	724	853	3
Os03g0785500	343	338	380	321.8	313.7	333.9	375	393	437	352.6	361.8	263.4	360.3	338.84	445.08	573.2	388.5	332.6	302.8	280.6	299.2	464.3	432.6	443.5	316.9	310.2	361.8	358	405	450	6
Os03g0650900	29.6	14.9	17.2	15.6	21.7	29.41	41.5	33.5	42.2	21.55	17.54	19.76	14.151	17.736	18.215	11.94	7.214	9.324	24.15	10.48	22.42	28.1	30.64	21.67	8.451	7.543	19.74	15.7	12.4	15	18
Os03g0402200	2.04	1.86	4.31	3.284	1.808	2.595	0	1.27	0.69	0	2.193	1.882	5.4426	4.6673	4.7518	0	4.122	3.108	3.716	1.613	0	2.218	5.701	0.722	8.451	0	0	3.7	5.74	1.67	4
Os03g0783700	343	390	369	433.5	389.6	478.4	475	407	520	485.4	453.9	446.9	485.48	473.26	437.17	704.5	400.9	364.7	558.3	519.2	543.2	341.6	376.3	378.5	426.8	400.7	497.8	376	390	380	6
Os03g0588400	0	0	0	0.821	0	0	0	0	0	0	0	0	0	0	0	0	0	0	0	0	0	0	0	0	0	0	0	0	0	0	2
Os03g0802100	0	0	0	0.821	0	0	0	0	0	0	0	0	0	0	0	0	0	0	0	0	0	0	0	0	0	0	0	0	0	0	2
Os03g0136700	109	120	130	363.7	346.2	343.4	153	159	131	175.9	178.7	179.7	206.82	184.82	152.06	167.2	170	135.7	466.3	562	670.8	93.9	92.64	150.9	134.2	158.4	116.2	101	122	137	16
Os03g0807500	0	0	1.08	4.926	9.04	2.595	6.26	0	0	0.862	0	0.941	2.177	0	0	0	5.153	0	2.787	0	0	0	0	0	0	0	0	0	0.96	0	2
Os03g0758500	320	205	254	97.7	69.61	109	33.7	23.4	12.4	87.94	66.88	67.74	74.019	124.15	70.485	155.2	344.2	204.1	93.82	103.2	156.1	28.84	37.77	48.39	73.95	82.97	87.72	69.4	73.6	80	14
Os03g0198900	511	519	552	515.6	582.2	532	492	481	424	464.7	557	619.1	582.36	530.2	511.61	835.9	553.4	551.2	510.9	591.8	559.6	573.8	539.5	505.6	502.8	509.1	403.5	512	581	626	6
Os03g0308200	284	347	349	266.8	239.6	353.8	321	328	283	256.1	222.6	268.1	211.17	254.83	222.54	477.6	332.9	380.2	275.9	297.5	318.2	372.7	411.2	413.1	239.8	287.6	322.4	428	337	346	6
Os03g0343700	277	232	251	399.8	432.1	423	330	269	282	301.8	285.1	436.5	346.15	340.71	307.28	549.3	251.4	276.6	496.1	540.2	549.2	266.2	255.1	253.5	238.7	310.2	335.5	281	323	241	16
Os03g0243350	42.8	54	45.3	55.83	55.14	44.98	163	115	128	64.66	58.11	55.51	58.78	46.673	47.518	59.7	45.34	43.51	50.16	66.12	54.32	108.7	85.52	79.45	53.87	49.03	57.02	37.9	45.9	30	18
Os03g0775650	84.6	107	105	45.15	46.1	50.17	47.8	43	54.6	112.1	89.9	175	132.8	154.02	145.72	95.53	134	158.5	87.32	74.98	102.6	46.58	48.46	54.89	127.8	175.4	164.5	96.2	112	106	3
Os03g0720300	640	675	657	1944	1176	2514	273	237	508	512.1	396.9	506.2	284.1	406.05	390.44	597	759.5	808.1	1018	1535	1385	1033	1655	1081	716.2	732.6	885.9	942	743	886	2
Os03g0107700	331	435	231	55.01	34.35	51.04	119	96.1	92.6	80.18	81.13	51.75	43.541	52.273	65.733	143.3	254.5	187.5	52.02	54.02	59.49	126.4	114	294	42.25	37.71	48.24	26.8	58.3	51.7	14
Os03g0415351	1.02	0.93	0	0.821	0	4.325	0	0	0	1.724	1.096	0	5.4426	1.8669	0	0	0	0	0	0	0	0.739	0	1.444	0	0	0	0.93	0	4.17	4
Os03g0219500	484	486	482	377.6	383.3	428.2	329	288	354	599.2	553.7	662.3	619.37	589.01	597.93	1206	694.6	712.8	451.5	420.9	578.5	366.7	383.4	310.6	709.9	627.9	596.5	629	715	697	13
Os03g0147700	238	246	216	355.5	331.8	359.9	257	303	261	202.6	172.1	119.5	222.06	158.69	242.34	477.6	188.6	235.2	246.2	298.3	223.3	333.5	380.5	261.5	170.1	163.1	162.3	228	225	216	6
Os03g0587600	0	0	0	0	0	0	0	1.27	0	0	0	0	0	0	0	0	0	0	0	0	0	0	0	0	0	0	0	0	0	0	18
Os03g0566600	0	3.73	0	6.568	5.424	0.865	4.7	0	2.07	0.862	0	0.941	0	0.9335	0	0	1.031	0	5.574	4.031	2.587	2.218	1.425	0.722	1.056	0	0	0.93	0	0	10
Os03g0129800	155	211	216	244.7	198	222.3	324	412	384	231.9	279.6	189.1	327.64	252.97	312.83	394.1	217.4	232.1	185.8	166.9	167.3	316.5	344.2	251.3	255.6	164.1	175.4	226	196	287	18
Os03g0161200	3.06	4.66	3.23	0	0	0	0	0	0	1.724	2.193	9.408	3.2656	0	0	0	4.122	0	0	0	0.862	0	0.713	0	1.056	1.886	0	0.93	0.96	0	20
Os03g0160100	1840	1815	1740	1641	1645	1697	1272	1377	1390	1662	1508	1219	1435.8	1444	1549.1	2137	1437	1574	1355	1550	1393	1769	1659	1574	1496	1455	1366	1488	1419	1442	8
Os03g0720800	44.9	43.8	36.6	31.2	18.98	34.6	15.7	11.4	18.7	33.63	39.47	36.69	32.656	28.937	22.175	59.7	35.04	36.26	32.51	27.41	20.69	87.25	45.61	70.06	33.8	67.88	63.59	35.2	31.6	22.5	9
Os03g0857200	401	414	427	266.8	289.3	317.5	160	209	173	459.5	403.5	475.1	338.53	455.52	415.78	644.8	494.7	451.7	337.2	334.6	363	212.2	228	213.1	442.6	602.5	561.4	515	534	495	13
Os03g0816300	337	387	351	526.2	507.1	423	372	341	375	334.5	392.5	251.2	420.17	320.17	369.85	537.3	312.3	309.8	327.9	402.3	278.5	390.4	473.9	401.6	335.9	329.1	296	474	374	361	2
Os03g0728500	19.4	15.8	23.7	144.5	113.9	91.7	224	278	248	25.87	31.8	21.64	42.452	32.671	41.974	23.88	16.49	31.08	67.81	87.88	74.15	105	122.6	121.3	33.8	28.29	10.96	20.4	23	20.8	18
Os03g0626000	0	0	1.08	0	0	0	0	0.63	0	0	0	0	0	0	0	0	0	0	0.929	0.806	3.449	0.739	0	0	0	0	0	0	0.96	0	16
Os03g0345100	87.7	115	81.9	135.5	147.3	154	203	238	190	74.15	65.78	54.57	93.613	77.476	80.781	131.4	68.02	93.24	135.6	125.8	116.4	214.4	196.7	203	64.44	82.97	81.14	83.3	109	85.8	18
Os03g0129200	1213	1199	1210	1050	1069	1127	835	892	855	963.9	1088	1021	1344.3	1237.8	1239.4	3117	1385	1246	945.7	1003	1041	1082	1070	1025	1286	1214	1121	1371	1229	1268	13
Os03g0117300	13.3	11.2	12.9	13.14	9.04	14.71	17.2	12.7	22.8	6.035	5.482	14.11	9.7967	14.935	13.463	0	13.4	10.36	20.44	4.838	9.484	13.31	19.95	20.95	3.169	6.6	15.35	7.4	15.3	9.16	18
Os03g0116400	82.6	66.1	71.1	9.852	8.136	12.11	14.1	13.9	23.5	42.25	47.14	88.44	28.302	27.07	26.927	35.82	53.59	48.69	9.29	6.45	11.21	21.44	17.1	26	33.8	35.83	43.86	33.3	30.6	21.7	14
Os03g0225600	358	318	284	341.5	339.9	360.7	535	473	491	396.6	391.4	331.2	280.84	359.38	323.91	334.3	337	329.5	387.4	324.1	426.8	408.1	379.1	452.8	353.9	351.7	427.6	301	307	295	18
Os03g0333300	1151	995	1068	1044	1102	1052	832	836	981	1233	1122	1598	1136.4	1264.8	1015.3	1385	1071	1050	1163	940.9	1172	831.8	772.5	780	1206	1385	1366	977	1005	861	20
Os03g0179900	531	512	432	600.1	584.9	544.1	295	305	211	403.5	323.4	408.3	242.74	294.04	319.16	1099	499.8	395.8	441.3	482.2	451.8	408.1	503.1	468.7	285.2	459.2	460.5	486	532	445	6
Os03g0699700	21.4	25.1	18.3	31.2	17.18	30.28	17.2	20.2	18	21.55	21.93	9.408	14.151	23.336	16.631	11.94	17.52	14.5	26.94	30.64	23.28	26.62	19.95	25.28	5.282	16.97	24.12	28.7	44.9	30	2
Os03g0187300	474	510	573	336.6	386	327	409	391	395	459.5	461.6	398.9	563.85	446.19	539.33	919.5	424.6	430	289.8	362	306.1	377.1	421.9	359	484.9	410.1	421	599	564	582	13
Os03g0734200	5.1	6.52	1.08	4.105	1.808	5.19	0.78	0	3.46	2.587	3.289	4.704	3.2656	0	1.5839	0	1.031	1.036	6.503	2.419	0	1.479	5.701	0.722	5.282	1.886	0	4.63	1.91	0	19
Os03g0859300	454	426	460	525.4	566.8	518.2	386	420	446	483.7	457.2	516.5	669.44	613.28	555.17	764.2	429.7	446.5	476.6	594.2	448.4	464.3	476.8	382.1	513.4	536.5	429.8	495	506	491	6
Os03g0576900	652	785	681	808.7	781.9	741.3	557	605	520	647.5	680.9	454.4	576.92	571.27	719.9	1194	646.1	599.9	618.7	624.9	650.1	577.5	585.1	574.9	672.9	557.2	497.8	694	678	759	6
Os03g0608800	494	597	613	555	599.3	556.2	354	302	352	435.4	484.6	454.4	583.45	405.12	482.31	704.5	451.4	438.2	478.4	507.2	447.5	349	311.4	315.6	367.6	404.5	432	452	480	384	13
Os03g0776100	295	199	200	235.6	210.6	189.4	445	478	443	370.7	285.1	410.2	283.02	264.17	321.54	370.2	325.6	245.5	434.8	417.7	427.7	260.3	285.1	409.5	312.7	397.9	447.4	210	373	269	15
Os03g0423000	178	187	192	261.1	252.2	211.1	232	211	234	194	185.3	174.1	227.5	228.7	209.87	429.9	166.9	186.5	188.6	262.8	238	219.6	213.8	203	237.7	170.7	173.2	229	227	219	6
Os03g0822900	358	439	418	503.3	488.1	450.7	265	361	323	600.9	611.8	619.1	581.27	444.32	518.74	525.4	383.4	394.7	420.8	435.4	343.2	392.6	394.1	359	679.2	655.3	703.9	461	423	493	20
Os03g0729900	195	237	213	268.5	282.9	243.9	256	308	240	202.6	190.8	183.5	229.68	186.69	239.97	286.6	238.1	212.4	207.2	222.5	201.8	280.2	288.6	275.2	246.1	206.5	195.2	232	209	234	17
Os03g0841800	95.8	84.8	89.4	116.6	125.7	77.85	160	245	149	68.97	74.55	96.9	67.488	92.412	69.693	191.1	53.59	85.99	88.25	85.47	106.1	119	126.8	145.2	80.28	77.31	94.3	85.1	89.9	74.1	18
Os03g0710100	139	166	138	184.7	212.4	201.6	127	158	124	140.5	159	112	151.3	177.36	142.55	262.7	157.7	156.4	203.4	196.7	201.8	154.5	168.2	141.6	177.5	172.5	173.2	218	173	187	6
Os03g0807400	1077	1480	1260	1832	1998	1677	1152	1404	1278	844.9	1442	907	1259.4	1011.9	1012.1	1433	997.6	1112	1303	1689	1308	1346	1196	1236	1049	802.4	785.1	904	993	1119	10
Os03g0643300	451	434	519	481.9	564.1	513	522	591	487	505.2	480.2	479.8	684.68	541.4	677.13	704.5	427.7	456.9	530.4	512	444.9	378.6	458.2	416.7	472.2	389.4	432	494	536	548	4
Os03g0754200	773	678	720	445.8	479.1	468.9	307	305	313	649.2	714.8	754.5	735.84	748.63	811.77	1027	794.5	775	446.8	517.6	516.5	431.1	383.4	373.4	784.9	859.9	807	761	762	755	3
Os03g0820500	227	160	149	15.6	20.79	21.63	6.26	8.22	5.53	172.4	114	126.1	72.931	120.42	114.84	191.1	177.3	135.7	10.22	19.35	31.9	6.654	9.264	18.78	131	150.9	186.4	183	129	119	3
Os03g0367101	21.4	15.8	45.3	4.105	5.424	4.325	19.6	11.4	6.91	41.38	33.99	35.75	23.947	15.869	15.839	0	15.46	8.288	13.01	8.869	0.862	11.09	12.83	12.28	21.13	17.91	10.96	12	19.1	12.5	7
Os03g0650800	125	185	142	534.5	559.6	477.5	165	223	203	89.67	109.6	85.61	109.94	129.75	140.97	167.2	155.6	135.7	407.8	472.5	500.1	236.6	223.8	249.2	124.7	159.3	142.5	192	147	182	10
Os03g0661500	89.7	82	91.6	45.97	41.58	43.25	157	124	136	157.8	91	96.9	77.285	62.541	91.076	107.5	93.78	81.85	73.39	54.02	68.12	149.4	173.9	145.2	97.19	96.17	111.8	122	98.5	67.5	5
Os03g0332600	602	482	523	464.7	432.1	525.9	615	516	613	762.2	787.2	981.3	994.91	985.72	824.44	656.8	670.9	673.4	916.9	934.5	988.1	379.3	413.3	518.6	713	786.3	1002	592	610	547	20
Os03g0852500	172	179	149	210.2	233.2	256.9	182	144	211	112.9	118.4	113.8	106.67	149.35	116.42	334.3	154.6	146.1	223.9	195.1	227.6	169.3	196.7	180.6	117.3	124.5	160.1	139	133	113	6
Os03g0686300	274	341	303	259.4	238.6	250.9	92.4	131	98.8	89.67	162.3	57.39	198.11	203.49	217.79	406	227.7	260	57.6	92.72	86.22	416.3	391.9	210.2	210.2	195.2	114	391	305	372	8
Os03g0785100	134	144	129	124.8	116.6	130.6	272	189	189	208.6	189.7	264.4	154.57	229.63	176.61	143.3	129.8	145	164.4	116.9	174.2	135.3	141.1	183.5	141.6	173.5	153.5	171	134	152	5
Os03g0808400	0	0	0	0	0	0	0	0.63	0	0	0	0	0	0	0	0	0	0	0	0	0	0.739	2.851	0	0	0	0	0	0	0	17
Os03g0148000	0	0	1.08	1.642	3.616	0	0.78	0	0	0.862	0	0	5.4426	0.9335	0	0	1.031	0	2.787	1.613	0	0.739	2.138	1.444	0	0.943	0	0	1.91	0.83	4
Os03g0379400	31.6	33.5	40.9	52.54	56.05	37.2	47.8	46.2	56.7	46.56	51.53	43.28	38.098	47.606	36.43	95.53	42.25	33.15	60.38	33.86	62.94	34.75	32.07	39.72	55.99	43.37	37.28	37.9	45.9	42.5	6
Os03g0610700	1.02	0	1.08	1.642	0	4.325	0.78	0.63	0.69	0	0	0.941	0	0	0	0	0	0	0.929	0.806	2.587	0	0	0.722	0	0	2.193	0	0	0	10
Os03g0171900	291	299	406	656	757.5	438.6	1188	1107	949	353.5	263.1	359.4	438.67	354.71	349.26	262.7	227.7	244.5	869.5	693.4	656.1	530.9	590.1	811.8	326.4	284.7	453.9	325	293	319	12
Os03g0690000	387	453	499	1251	1492	1322	438	331	397	381.9	341	260.6	284.1	464.86	342.92	549.3	349.4	390.6	1401	1228	1251	329.8	347.1	353.9	289.4	243.3	201.7	252	261	256	10
Os03g0850600	3881	3548	4066	3424	3405	3824	3125	2631	2897	6210	6588	7658	6238.3	5827.5	5694.2	6591	4956	4860	6224	6103	6683	1675	1699	2258	5832	6349	6129	4261	4456	3952	20
Os03g0655200	339	346	330	276.7	266.7	288.1	420	385	352	299.2	310.3	292.6	379.89	361.25	444.29	417.9	386.4	304.6	253.6	268.5	271.6	309.8	316.4	316.3	304.2	318.7	342.1	387	340	352	5
Os03g0764000	93.8	185	235	68.14	87.69	57.96	101	145	105	250	285.1	209.8	777.2	499.6	813.35	441.8	216.4	260	132.8	72.57	113.8	98.34	89.08	138.7	265.1	283.8	197.4	366	489	403	4
Os03g0115100	485	620	533	1439	1589	1352	698	787	642	556.1	560.3	372.6	499.63	450.86	447.46	812	399.8	493.2	1054	1098	964.8	887.3	999.1	728	514.4	453.5	383.8	562	527	559	10
Os03g0336900	0	0	1.08	0	0	1.73	0	0	0	3.449	0	0.941	0	0.9335	1.5839	0	2.061	1.036	1.858	0	0.862	0	0	0.722	4.225	0.943	4.386	0.93	3.83	1.67	11
Os03g0406100	3.06	1.86	8.62	4.105	2.712	11.25	9.39	13.3	6.22	4.311	4.386	0	1.0885	6.5342	2.3759	0	2.061	0	12.08	4.838	1.724	6.654	16.39	20.22	5.282	0.943	32.89	13.9	31.6	10	11
Os03g0773800	####	####	####	18076	19572	18862	####	####	####	34399	36977	38658	33554	31986	30927	38975	28703	26227	24845	20929	24584	28283	28643	37478	34951	36118	33635	####	####	####	5
Os03g0758800	3159	2818	2879	2882	3287	3675	3931	3419	3886	2925	2693	3521	2920.5	3061.7	2406	3666	2549	2762	4377	3955	4694	3222	3573	3625	2747	3080	3193	1945	2246	2195	15
Os03g0577150	0	0	0	0	0	0.865	0	0	0	0	0	0	0	0	0	0	0	0	0	0	0	0	0	0	0	0	0	0	0	0	2
Os03g0852000	37.7	33.5	33.4	86.2	87.69	88.23	72.8	59.5	46.3	78.46	55.92	76.21	44.629	43.872	47.518	47.76	31.95	34.19	75.25	84.66	65.53	62.11	51.31	52.72	27.47	45.26	61.4	20.4	22	26.7	10
Os03g0204500	0	0	1.08	0.821	0	0	0	0	2.07	1.724	0	0	0	0	1.5839	0	0	0	0.929	0	0	2.218	1.425	2.889	0	0	0	0	0.96	0.83	9
Os03g0265500	845	948	894	1434	1520	1295	1003	1035	1103	1043	1084	1131	1154.9	1059.5	1024	2221	1010	1063	1485	1517	1562	947.9	985.6	1075	1221	1199	1123	1081	1092	1161	6
Os03g0567100	37.7	40.1	46.3	169.9	216	154	66.5	47.4	62.2	29.31	28.51	28.22	32.656	32.671	23.759	47.76	20.61	38.33	222	204.8	242.3	27.36	23.52	39	25.35	19.8	28.51	23.1	23.9	28.3	10
Os03g0615300	34.7	35.4	24.8	24.63	18.98	38.93	9.39	17.7	27.6	34.49	36.18	32.93	28.302	29.87	30.095	95.53	58.74	71.49	31.58	37.9	47.42	54.71	42.05	31.78	63.38	44.31	57.02	71.2	63.1	44.2	13
Os03g0251350	778	1208	879	1886	1610	2045	1661	1655	1832	1825	1912	2234	1189.8	1032.4	1108.8	1051	1127	1435	1576	1358	1953	1362	1125	1427	1686	1774	1577	738	686	1101	15
Os03g0625933	0	0	0	0.821	0	0	0.78	0	0	0	3.289	0	0	0	0	0	2.061	0	0	0	0	0	0	0	3.169	0	8.772	0	2.87	0	11
Os03g0199200	0	1.86	3.23	2.463	0.904	4.325	4.7	1.9	2.76	2.587	1.096	3.763	0	0	0	0	0	3.108	0.929	4.031	0.862	2.958	4.988	5.056	1.056	0	2.193	2.78	0.96	0	17
Os03g0395200	0	0	0	2.463	0	0	0	0	0	0	0	2.822	0	0	5.5438	0	1.031	3.108	1.858	0.806	0	0.739	0	0	2.113	1.886	0	0.93	0	0.83	4
Os03g0425800	2788	2521	2541	1300	1197	1383	1481	1521	1699	2111	2054	1652	1860.3	1875.3	2219.9	4096	2277	2251	1244	1403	1399	1765	1755	1717	2243	2285	2011	2021	2040	2019	13
Os03g0340900	5678	5356	6461	4322	4523	4251	4566	5301	5348	4527	5603	4616	6841.3	5877	6261.3	11308	6486	6406	4171	4679	4072	5417	4673	5030	6223	5772	4620	5985	6417	6673	13
Os03g0557900	8.16	3.73	8.62	5.747	6.328	1.73	2.35	0.63	1.38	10.35	3.289	7.527	8.7082	9.3345	11.088	11.94	0	5.18	1.858	3.225	0.862	7.394	6.414	0.722	9.507	0	0	10.2	23	6.66	8
Os03g0596101	0	1.86	1.08	0.821	0.904	0	0.78	0	3.46	0	0	0	0	2.8004	0	0	0	0	0	0	0	0	0	0	0	0	0	0	0	0.83	4
Os03g0860000	303	340	337	329.2	348.9	321.8	289	417	308	263	246.7	275.7	302.61	260.43	295.4	489.6	361.7	339.8	205.3	303.2	250	349	359.9	312.7	314.8	274.4	265.3	368	359	333	6
Os03g0565500	1231	1219	1079	1629	1690	1599	1467	1397	1329	1223	1181	1182	1254	1289.1	1039.9	1648	1155	1224	1632	1459	1482	1329	1343	1278	1068	1121	1281	1142	1007	1031	10
Os03g0137400	368	364	360	422	414.9	399.7	391	470	458	308.7	342.1	306.7	302.61	339.78	406.28	561.2	380.3	328.4	325.1	361.2	349.2	429.6	461.8	444.9	348.6	396.9	287.3	386	353	372	6
Os03g0824500	763	686	745	666.6	777.4	664.4	763	658	715	704.4	787.2	762.1	784.82	890.51	792.76	1672	681.2	702.4	785.9	820.8	711.3	609.3	655.6	622.6	749	694.9	881.6	698	642	743	6
Os03g0650700	262	277	279	171.6	144.6	137.5	106	83.5	106	242.3	298.2	290.7	310.23	266.03	324.71	358.2	207.1	205.1	160.7	121.7	169.9	126.4	133.3	106.2	281	296.1	271.9	270	197	198	3
Os03g0217801	163	95	109	61.57	43.39	63.15	78.3	49.3	64.3	105.2	71.27	101.6	96.878	95.212	89.492	131.4	137.1	153.3	77.1	65.31	94.84	66.54	50.6	52.72	95.07	93.34	155.7	109	95.6	112	3
Os03g0798000	0	0	0	0	0	0.865	0	0	0	0	0	0	0	0	0.792	0	0	0	0	0	0	0	0	0	0	0	0	0	0	0.83	4
Os03g0800000	1257	1194	1499	767.6	352.5	683.4	660	417	731	1711	2048	1423	1346.5	1141.6	1332.1	1827	1267	1340	860.2	418.5	425.1	1345	1135	2298	1839	1959	2147	1856	2007	1962	3
Os03g0264600	0	0	1.08	0	0	0	1.57	1.27	3.46	0	0	0	0	0	0	0	0	0	0	0	0.862	0	1.425	0	0	0	0	0	0	0.83	18

Os03g0709000	1318	1074	1570	550.1	540.6	606.4	235	285	158	970	974.7	901.3	1261.6	1343.2	1465.1	1946	1047	999.8	1117	972.4	744.1	139.7	203.8	175.5	956	682.6	787.3	866	1034	957	13
Os03g0208500	547	558	573	1093	1157	1080	1105	1180	1135	632	544.9	657.6	661.82	648.75	638.32	979.2	563.7	583.3	1111	1226	1134	1349	1462	1325	597.9	643	804.8	677	708	651	12
Os03g0830200	23.4	18.6	23.7	22.17	10.85	28.55	117	68.9	107	56.04	29.6	43.28	14.151	14.935	19.799	0	16.49	13.47	40.87	30.64	23.28	124.2	168.9	147.3	26.41	4.714	24.12	31.5	16.3	15	17
Os03g0584300	68.3	64.3	80.8	94.41	86.78	88.23	141	102	123	76.73	74.55	75.27	37.01	47.606	61.773	47.76	40.19	58.02	91.04	83.05	94.84	84.29	91.93	110.5	39.09	61.28	61.4	49	42.1	46.7	12
Os03g0819400	92.8	82	47.4	67.32	72.32	73.53	21.1	20.2	11.7	64.66	73.46	49.86	67.488	88.678	71.277	35.82	98.93	73.56	92.9	76.6	106.1	11.09	17.1	8.667	60.21	72.6	54.82	62	79.4	76.6	16
Os03g0405000	552	626	630	656	765.7	659.2	732	854	872	506.1	491.2	456.3	495.28	525.53	529.03	967.2	530.7	547	565.7	585.4	549.2	810.4	761.8	740.3	481.7	417.7	543.8	557	475	553	18
Os03g0772100	0	0	0	0	0	0	0	0	0	0	0	0	0	0.9335	0.792	0	0	0	0	0	0	0	0	0	0	0	0	0	0.96	0	4
Os03g0305000	959	657	915	63.22	63.28	64.88	110	132	87.1	707.9	862.9	895.7	979.67	1030.5	1013.7	1230	1236	1063	130.1	99.98	87.08	82.81	100.5	100.4	950.7	1027	853	847	873	956	3
Os03g0122500	25.5	21.4	23.7	22.99	28.02	41.52	61.1	32.3	51.1	44.83	36.18	47.04	31.567	38.272	24.551	35.82	22.67	20.72	37.16	54.02	40.52	60.63	34.21	55.61	32.75	34.89	39.47	24.1	53.6	23.3	15
Os03g0628900	575	548	553	527.9	616.5	580.4	711	616	675	518.2	583.3	625.6	544.26	622.61	559.92	680.6	508.1	541.8	686.5	478.9	547.5	520.5	518.1	543.1	511.3	502.5	539.5	485	415	465	15
Os03g0558400	2.04	10.2	0	13.14	24.41	16.44	2.35	8.22	2.76	0	0	0	2.177	3.7338	1.5839	0	1.031	2.072	12.08	4.838	11.21	15.53	24.23	13.72	2.113	2.829	0	1.85	6.69	5	2
Os03g0722400	43.8	32.6	33.4	174.9	171.8	217.1	152	130	131	59.49	38.37	45.16	48.983	59.741	45.142	119.4	58.74	71.49	170.9	191.9	188.8	176.7	121.1	140.1	39.09	39.6	50.44	41.6	64.1	44.2	12
Os03g0274400	4.08	2.79	2.16	2.463	1.808	2.595	7.05	4.43	4.84	9.484	3.289	8.467	1.0885	0.9335	2.3759	23.88	2.061	12.43	0.929	4.031	1.724	7.394	4.988	1.444	0	4.714	8.772	1.85	6.69	1.67	6
Os03g0722500	0	2.79	1.08	2.463	0	1.73	3.91	3.8	2.76	3.449	6.578	0	1.0885	0	1.5839	0	1.031	1.036	1.858	0	3.449	0.739	2.138	1.444	1.056	0	0	0	0.96	0.83	7
Os03g0242900	97.9	87.6	77.6	36.94	49.72	25.95	104	106	58	83.63	77.84	149.6	67.488	66.275	61.773	71.65	76.26	75.63	43.66	44.35	27.59	62.11	54.16	83.78	52.82	53.74	103.1	52.7	69.8	64.2	20
Os03g0639400	1.02	0.93	1.08	0.821	1.808	2.595	1.57	3.16	0.69	0	0	2.822	0	4.6673	0.792	0	0	1.036	4.645	2.419	0	0	0	2.167	4.225	7.543	6.579	5.55	0.96	1.67	11
Os03g0816800	87.7	61.5	61.4	41.87	33.45	30.28	57.2	65.1	60.1	47.42	36.18	29.17	19.593	29.87	36.43	71.65	62.86	63.2	68.74	39.51	39.66	51.76	78.39	65.72	30.63	33	24.12	27.8	38.3	25.8	14
Os03g0219400	221	246	241	242.2	276.6	216.3	383	316	310	206.1	234.6	215.4	275.4	196.02	227.29	417.9	205.1	272.5	219.2	222.5	231.1	223.3	228.8	269.4	241.9	177.3	182	261	240	315	6
Os03g0171300	0	0	0	0	1.808	0	0	0	0	0	0	0	0	0	0	0	0	0	0	0	0	0	0	0	0	0	0	0.93	0	0	2
Os03g0664800	37.7	26.1	42	54.18	39.77	33.74	121	104	160	127.6	48.24	58.33	39.187	54.14	54.646	23.88	18.55	29.01	52.95	49.99	41.39	89.47	101.9	130	51.76	28.29	46.05	32.4	23	21.7	18
Os03g0227601	12.2	15.8	7.54	23.81	20.79	23.36	12.5	35.4	8.98	12.93	21.93	13.17	21.77	15.869	25.343	11.94	12.37	13.47	35.3	34.67	30.18	24.4	25.65	13.72	16.9	15.09	13.16	21.3	27.7	25	16
Os03g0250400	1.02	2.79	0	2.463	1.808	3.46	0	5.69	0	0	0	0	0	0	2.3759	0	0	0	0	0	0	0	0	0.722	0	0	0	0	0	0	2
Os03g0757100	77.5	97.8	83	48.44	34.35	40.66	4.7	22.1	6.91	128.5	111.8	103.5	190.49	178.29	186.9	214.9	115.4	114	46.45	31.44	45.7	25.14	21.38	31.78	126.8	153.7	120.6	123	133	172	3
Os03g0223000	284	288	298	285.7	298.3	285.5	345	338	256	308.7	294.9	309.5	271.04	272.57	266.1	561.2	277.2	276.6	390.2	323.3	355.2	316.5	311.4	293.2	347.5	334.7	355.3	280	277	276	6
Os03g0133875	1.02	0	0	0.821	0	0.865	0	0	2.07	0.862	0	0.941	1.0885	0	3.1679	11.94	0	0	0	1.613	0.862	0	0	2.167	0	0.943	0	0	0	0	6
Os03g0712733	0	0	0	0	0	0	0	0	0	0	0	0	0	0	0	0	1.031	0	0	0	0	0	0	0	0	0	0	0	0	0	1
Os03g0722700	173	170	213	246.3	245	249.1	287	253	337	239.7	257.7	305.8	234.03	259.5	201.95	334.3	189.6	212.4	279.6	220.9	243.1	237.3	259.4	231.1	153.2	216.9	182	204	219	142	6
Os03g0207400	214	294	152	58.29	61.47	69.2	116	125	99.5	74.15	58.11	21.64	42.452	48.539	34.055	191.1	164.9	187.5	43.66	81.43	74.15	160.4	176.7	171.9	46.48	46.2	57.02	56.4	44.9	45	14
Os03g0710900	534	659	630	751.2	800	787.2	668	761	759	601.8	640.3	391.4	559.5	566.61	618.53	859.7	574	560.5	631.7	637	575.1	632.2	613.6	613.2	555.6	456.3	440.8	582	569	682	6
Os03g0154000	120	91.3	97	67.32	65.99	70.07	47.8	48.7	44.9	62.08	48.24	89.38	55.515	77.476	63.357	167.2	73.17	63.2	64.1	35.48	48.28	31.79	35.63	35.39	109.9	66	57.02	74.9	68.9	94.1	13
Os03g0210800	914	894	887	895.7	829.8	833	822	867	842	857.9	856.3	705.6	955.72	846.64	926.6	1731	948.1	864.1	783.1	890.9	746.7	853.3	882.2	890.5	858.8	876.8	833.3	928	984	1005	6
Os03g0831500	154	136	120	526.2	568.6	520.8	307	269	275	118.1	119.5	142.1	117.56	176.42	86.324	179.1	97.9	118.1	515.6	492.6	601	266.9	290	291.1	84.51	139.5	151.3	99.9	114	108	10
Os03g0222800	121	152	122	152.7	143.7	138.4	173	187	167	114.7	114	144.9	112.12	114.81	141.76	179.1	132.9	152.3	162.6	176.6	144	142	130.4	170.4	108.8	154.6	100.9	115	112	107	12
Os03g0439800	562	538	518	244.7	241.4	232.7	644	620	639	540.6	557	660.5	511.6	504.06	590.01	823.9	761.6	681.7	301	311.2	322.5	417	424.7	517.8	651.8	710.9	581.1	570	598	637	13
Os03g0800100	37.7	38.2	38.8	31.2	28.02	27.68	83	49.3	52.5	55.18	54.82	62.09	62.046	85.878	49.102	35.82	42.25	43.51	57.6	54.02	69.84	45.1	42.05	36.11	66.55	52.8	85.52	47.2	41.1	47.5	15
Os03g0133800	43.8	23.3	31.2	21.35	18.98	32.87	14.9	36.1	19.3	12.07	12.06	17.88	13.062	16.802	12.671	95.53	39.16	51.8	10.22	13.71	15.52	19.22	27.79	16.61	20.07	37.71	21.93	31.5	29.6	42.5	13
Os03g0712200	13.3	10.2	15.1	105.9	136.5	122.8	86.1	98	80.8	6.035	4.386	6.586	2.177	9.3345	7.1277	11.94	3.092	5.18	84.53	109.7	113	93.9	81.24	81.61	9.507	1.886	15.35	7.4	4.78	6.66	12
Os03g0114300	774	803	913	637.9	604.8	650.5	1070	910	1095	977.7	958.2	967.2	976.4	914.78	935.31	1684	758.5	692.1	608.5	680.5	679.4	1222	1006	1163	1038	1035	1085	1034	761	840	6
Os03g0279900	232	270	247	314.4	308.3	382.4	362	391	366	251.8	271.9	291.7	330.91	306.17	295.4	322.4	311.2	276.6	303.8	287.8	364.7	334.9	344.2	356.8	325.4	255.5	318	290	257	276	18
Os03g0436400	2.04	3.73	3.23	3.284	6.328	4.325	0.78	1.27	0.69	0.862	1.096	2.822	3.2656	0.9335	3.1679	0	5.153	5.18	1.858	4.838	3.449	0.739	2.138	4.333	1.056	2.829	2.193	2.78	4.78	3.33	2
Os03g0259001	4.08	4.66	2.16	3.284	8.136	8.65	1.57	1.9	7.6	0.862	2.193	2.822	1.0885	0.9335	0	11.94	5.153	6.216	9.29	5.644	3.449	1.479	0.713	7.222	1.056	1.886	6.579	2.78	2.87	1.67	6
Os03g0118450	374	158	298	56.65	52.43	60.55	64.2	58.8	62.2	468.2	299.3	534.4	246.01	412.59	323.12	274.6	281.3	295.3	209.9	91.92	133.6	26.62	43.47	54.89	297.9	358.3	390.3	289	268	234	3
Os03g0858700	402	379	363	964.6	1080	833.9	1265	923	907	381.9	437.5	629.4	502.9	464.86	340.55	573.2	367.9	436.2	1059	1168	1227	669.1	575.8	943.3	283.1	297	381.6	218	276	282	12
Os03g0832900	858	841	810	1279	1337	1375	727	838	791	834.6	792.7	730.1	942.66	962.39	894.13	1409	893.5	920	1575	1570	1520	719.4	828.8	802.4	838.8	708.1	717.1	1007	888	879	10
Os03g0859850	0	0	0	0	0	0	0	0	0	0	0	0	0	0	0	0	0	0	0	0	0	0	0.713	0	0	0	0	0	0	0	17
Os03g0147400	3291	3415	3115	1979	1748	1856	172	469	220	1455	1426	537.2	2525.4	1746.5	3047.5	5242	2737	3279	663.3	1102	557.9	2017	2438	1048	2696	2257	1452	4897	4844	4855	8
Os03g0415600	0	0	0	0	0	0	0.78	0	0	0	0	0	0	0	0	0	0	0	0	0	0	0	0	0	0	0	0	0	0	0	5
Os03g0323100	375	346	303	344	348.9	273.4	265	300	330	465.6	491.2	917.3	489.83	404.18	382.52	931.4	434.9	621.6	451.5	461.2	487.2	352.7	269.4	272.3	600	529.9	440.8	380	319	376	13
Os03g0236050	0	0	0	0	0	2.595	3.13	5.06	2.07	0	0	0	0	0	0	0	0	0	0	0.806	1.724	0.739	2.138	2.167	0	0	0	0	0	0	18
Os03g0321300	34.7	29.8	28	22.17	22.6	24.22	41.5	12.7	42.2	30.18	50.43	67.74	46.806	41.072	49.894	119.4	34.01	34.19	26.94	24.19	27.59	68.02	52.73	64.28	33.8	57.51	70.17	41.6	39.2	32.5	6
Os03g0153000	431	400	425	398.2	377.9	393.6	384	385	408	521.6	453.9	508	634.61	577.81	525.87	597	448.3	467.3	482.1	494.3	440.6	361.6	310	342.3	562	477.1	631.6	474	465	447	3
Os03g0737800	388	480	419	403.9	449.3	453.3	510	490	489	435.4	361.8	366.9	374.45	424.72	461.72	788.1	349.4	375	349.3	371.7	364.7	408.1	455.4	453.6	395.1	342.3	377.2	358	335	373	6
Os03g0286300	3360	2685	2695	1737	1531	1936	958	910	1041	1186	1051	1111	1170.2	1207.9	1263.2	2006	2503	2200	2055	1959	2108	1183	1303	1360	1231	1177	1401	1265	1306	1222	14
Os03g0651950	1.02	0	3.23	0	0	0	0	0	0	0	0	0	0	0	0	0	0	2.072	0	0	0	0.739	0	0	1.056	0	0	0	0	0.83	14
Os03g0410100	155	110	114	151.9	114.8	172.1	117	136	153	104.3	69.07	155.2	118.65	184.82	136.22	143.3	123.7	114	198.8	223.3	226.8	158.2	125.4	161.8	115.1	180.1	190.8	134	121	98.3	16
Os03g0441000	259	309	318	325.9	285.7	281.1	189	285	220	213.8	255.5	175.9	333.09	269.77	320.75	585.1	298.9	312.9	192.3	211.2	217.3	258.8	300	257.1	302.1	295.1	225.9	347	325	417	13
Os03g0254900	1938	1699	1950	1022	822.6	941.2	1089	1213	1074	1427	1406	1168	1823.3	1632.6	1572.1	2436	1624	1615	910.4	990.9	860.5	981.2	912.9	981.5	1402	1298	1088	1645	1677	1825	13
Os03g0769025	15.3	14.9	6.47	8.21	6.328	10.38	0	1.27	4.84	1.724	3.289	5.645	2.177	3.7338	6.3357	11.94	11.34	12.43	0.929	2.419	4.311	8.873	14.25	16.61	7.395	0	6.579	14.8	8.61	10.8	8
Os03g0288700	398	310	336	157.6	133.8	186	311	243	236	238.8	237.9	245.6	227.5	234.3	226.5	561.2	317.4	294.2	225.7	224.1	194.9	187.1	203.8	234.7	222.9	213.1	293.9	271	227	224	13
Os03g0117900	0	1.86	0	2.463	0.904	1.73	2.35	3.16	1.38	0	1.096	4.704	2.177	1.8669	3.1679	0	8.244	5.18	3.716	2.419	13.8	0.739	2.851	1.444	0	6.6	0	0	5.74	1.67	16
Os03g0823800	5.1	7.45	7.54	0	0	0	1.57	0	2.07	0	4.386	1.882	0	1.8669	0	0	4.122	1.036	0	0	0	2.218	0	0.722	0	0	0	0.93	0	0	14
Os03g0132300	47.9	31.7	44.2	92.77	74.13	68.34	98.6	125	151	56.04	69.07	66.8	48.983	52.273	60.189	83.59	39.16	39.37	67.81	63.7	70.7	108.7	84.8	129.3	49.65	50.91	54.82	42.6	53.6	36.7	18
Os03g0713100	124	117	105	195.4	186.2	242.2	284	185	202	126.7	99.77	141.1	108.85	139.08	109.29	214.9	87.6	84.96	204.4	202.4	226.8	130.9	122.6	164.7	75	107.5	136	71.2	65	65.8	12
Os03g0702900	5.1	3.73	0	4.105	0.904	2.595	2.35	3.16	3.46	5.173	1.096	5.645	5.4426	0.9335	2.3759	35.82	8.244	13.47	2.787	2.419	0	7.394	4.276	5.778	3.169	1.886	0	5.55	5.74	3.33	6
Os03g0344650	0	2.79	1.08	1.642	0	1.73	7.83	2.53	8.29	1.724	0	4.704	3.2656	2.8004	0.792	0	0	3.108	0	4.838	1.724	2.218	3.563	4.333	2.113	2.829	0	0	0.96	0	18
Os03g0640300	324	372	293	263.5	302.8	243.1	238	249	218	286.2	273	327.4	326.56	385.52	346.09	835.9	327.7	331.5	248	320.1	281.1	297.2	275.1	262.2	281	358.3	348.7	418	401	486	13
Os03g0396000	1.02	0.93	0	0	0	1.73	3.13	0	3.46	0	0	0	0	0	0	0	0	0	0	0	3.449	0	0.713	0	0	0	2.193	0	0	0	15
Os03g0219800	869	914	907	882.5	990.8	980.1	712	701	614	922.5	880.4	856.1	1110.3	975.46	932.94	1182	1082	1067	895.5	995.8	980.3	860.6	884.4	758.4	974	966.4	888.1	1112	1055	1176	13
Os03g0113100	285	296	356	555	560.5	532	629	596	575	272.5	251.1	287.9	341.8	307.11	254.22	406	196.8	279.7	551.8	533.8	651.8	346	436.8	437.7	216.6	221.6	318	171	233	197	12
Os03g0831800	413	452	448	604.2	712.3	642.7	618	534	603	451.8	416.6	360.3	360.3	406.05	403.11	513.5	355.5	322.2	600.1	531.3	501.8	309.8	372	383.5	357.1	337.5	311.4	297	361	342	10
Os03g0276600	261	251	264	338.2	334.5	357.3	224	236	258	234.5	198.4	244.6	275.4	212.83	207.5	406	207.1	233.1	301	350.7	368.2	264.7	275.8	193.6	208.1	242.3	219.3	253	269	256	10
Os03g0375717	0	0	0	0	0	0	0	0	0	0	2.193	0	0	0	0	0	0	0	0	0	0	0	0	0	1.056	0	0	0	0	0	7
Os03g0621600	3.06	1.86	4.31	4.926	2.712	3.46	8.61	0.63	1.38	0	4.386	1.882	0	0	0	0	1.031	2.072	2.787	0.806	0	5.176	4.276	5.056	1.056	3.771	0	4.63	0.96	1.67	9
Os03g0299900	1613	1708	1691	2622	3016	2427	2818	2823	2934	1142	1254	1379	1194.1	1276	1051.7	1493	1012	1340	2598	2355	2793	2285	2118	2654	1047	957.9	1250	886	974	849	12
Os03g0234600	408	372	448	344.8	361.6	392.7	471	409	451	488	503.2	554.1	502.9	535.8	501.31	692.6	516.3	443.4	467.3	462.8	476.8	359.3	333.5	468	492.3	579.8	480.2	443	481	488	13
Os03g0812800	0	0	0	0	0	0	0	0	0	0	1.096	0	0	0	1.5839	0	1.031	0	0	0	0	0	0	0	0	0	0	0	0	0	4
Os03g0197400	551	531	513	613.3	609.3	642.7	733	658	625	636.3	615.1	750.8	582.36	653.42	592.39	644.8	549.3	564.6	628	596.6	646.7	474.7	560.8	516.4	616.9	629.8	708.3	487	518	481	15
Os03g0279400	250	333	341	330	354.4	338.2	346	239	297	256.1	222.6	192.9	151.3	229.63	160.77	262.7	140.2	178.2	291.7	244.3	225.9	187.1	238	258.6	181.7	109.4	166.7	156	146	134	12
Os03g0680800	5628	5511	5553	3165	2923	3319	2576	3278	3024	4877	5031	4715	6210	5914.3	6554.3	10556	6309	6005	2902	3004	3019	3499	3994	3868	6189	5895	5039	6401	5712	6657	13
Os03g0301700	2.04	0	1.08	2.463	7.232	4.325	0	1.27	0	2.587	1.096	2.822	0	0	1.5839	0	2.061	0	1.858	0	1.724	0	1.425	0.722	0	3.771	0	0	0	0	2
Os03g0407100	25.5	19.6	22.6	51.72	23.5	25.09	45.4	40.5	57.4	39.66	27.41	32.93	9.7967	14.935	13.463	11.94	32.98	34.19	48.31	41.12	62.08	63.59	108.3	80.89	23.24	22.63	48.24	34.2	38.3	25.8	17
Os03g0835400	112	123	100	115.8	115.7	156.6	246	221	243	161.2	121.7	180.6	87.082	140.95	119.59	59.7	93.78	77.7	117	108.8	107.8	148.6	171.7	188.5	141.6	134.8	151.3	114	115	114	18
Os03g0850200	29.6	30.7	31.2	112.5	62.37	78.72	79.1	166	186	62.08	54.82	25.4	20.682	27.07	49.894	59.7	11.34	18.65	62.24	68.53	44.84	204.8	197.4	182.7	45.42	45.26	67.98	46.3	47.8	45.8	17
Os03g0180500	1567	1393	1664	1121	1125	1285	1134	1199	1090	1599	1774	1817	1766.7	1769.8	1759	2723	2037	1859	1204	1363	1324	1547	1289	1288	1969	2159	1906	2096	2132	2065	13
Os03g0648400	795	1198	804	837.4	892.2	630.6	1039	1216	1157	676	695.1	507.1	573.65	413.52	696.93	1099	644.1	661	406	347.5	321.6	1355	1325	1189	649.7	611.9	508.8	588	565	789	17
Os03g0139200	385	401	399	311.1	292	300.2	323	240	286	393.2	292.7	454.4	313.49	368.71	275.6	597	343.2	415.5	241.5	238.7	201.8	408.1	381.3	445.6	264.1	362.1	368.4	383	445	382	13
Os03g0211850	0	0	1.08	0	0	0	0	0	0	0	0	0	0	0.9335	0.792	0	0	0	0	0	0	0	0.713	0	0	0	0	0	0	0.83	4
Os03g0345200	1868	1533	1490	2103	2336	2462	3171	2337	3214	2013	1784	3247	1940.8	2216.9	1465.9	3929	1352	1579	3055	2383	3101	1670	1742	1983	1577	2070	2423	1039	1350	1001	15
Os03g0275500	2229	1924	1950	1549	1453	1555	1484	1416	1470	1553	1436	1563	1396.6	1437.5	1629.1	2412	2055	1686	1433	1283	1497	1366	1383	1603	1465	1555	1507	1259	1363	1345	14
Os03g0120300	39.8	48.4	35.6	36.94	62.37	51.04	42.3	41.1	34.6	56.04	49.34	47.04	32.656	58.807	37.222	11.94	45.34	46.62	47.38	36.28	41.39	56.19	43.47	36.11	45.42	49.97	61.4	50.9	46.9	37.5	11
Os03g0773100	281	305	270	229.9	203.4	244.8	245	245	240	257.8	267.5	205.1	181.78	244.56	269.27	453.8	280.3	268.3	183	207.2	238	331.2	344.2	298.3	293.7	277.2	250	372	358	332	13
Os03g0151000	1.02	0.93	0	0	0	0	0	0	0.69	2.587	1.096	0	2.177	0	0	0	3.092	2.072	0	0	0	0	0	0.722	2.113	0	0	0	1.91	0.83	1
Os03g0209400	474	497	495	545.9	566.8	536.3	611	590	540	563.9	549.3	426.2	453.91	434.99	458.55	573.2	484.3	451.7	545.3	502.3	500.1	502	515.2	536.6	509.2	540.2	456.1	498	516	456	12
Os03g0726500	4044	3482	4053	1524	924.8	1831	321	535	748	2236	2352	2076	5040.9	3962.5	5030.6	9732	6239	4375	3602	2488	2913	985.6	673.4	695.5	2904	3572	3195	5394	4062	4665	13
Os03g0839000	417	320	386	224.1	262.2	249.1	357	378	340	391.4	328.9	324.6	309.14	333.24	408.65	406	320.5	312.9	226.7	229	220.7	334.9	394.1	354.6	230.3	264	276.3	296	280	287	5
Os03g0138600	1.02	1.86	0	22.17	7.232	12.98	28.2	17.7	18	7.76	3.289	3.763	1.0885	5.6007	1.5839	0	4.122	6.216	24.15	9.675	22.42	62.11	36.34	76.56	2.113	3.771	10.96	7.4	11.5	2.5	9
Os03g0737701	5.1	8.38	8.62	8.21	11.75	6.055	4.7	3.8	9.67	11.21	5.482	3.763	10.885	9.3345	9.5036	0	10.31	8.288	5.574	6.45	1.724	6.654	11.4	5.056	9.507	5.657	10.96	3.7	5.74	3.33	4
Os03g0598800	1105	907	879	1250	1284	1412	1528	1197	1635	1255	926.5	1984	1168	1208.8	873.54	1397	843	882.7	1419	1064	1388	978.9	980.6	1083	937	1227	1316	655	878	577	15
Os03g0326500	5414	5767	6565	1449	1489	1629	1130	1160	978	7523	8735	6816	8526.4	8045.4	8300.6	7594	5947	6504	1483	1715	1551	710.6	791	793.7	6694	6343	5557	5698	5603	6663	3
Os03g0824300	151	173	133	227.4	218.8	261.2	121	133	150	130.2	145.8	112.9	160.01	160.55	161.56	250.8	123.7	160.6	190.4	225.8	252.6	160.4	156.1	132.9	136.3	129.2	116.2	192	211	175	10
Os03g0724100	404	388	336	297.2	251.3	367.6	331	238	314	515.6	387	587.1	380.98	425.65	408.65	310.5	426.6	379.2	388.3	333.8	314.7	319.4	443.3	372.7	440.5	506.3	581.1	484	458	397	20
Os03g0725000	427	455	450	475.3	485.4	531.1	707	535	608	479.4	559.2	546.6	482.21	538.6	505.27	573.2	448.3	493.2	581.5	522.5	587.2	492.4	434.7	471.6	413	495	506.6	428	435	459	15
Os03g0108800	24.5	34.5	36.6	46.8	55.14	45.85	33.7	47.4	42.2	35.35	37.28	20.7	30.479	17.736	43.558	83.59	27.82	29.01	20.44	24.19	11.21	46.58	57.01	33.22	42.25	24.51	6.579	43.5	44	38.3	6
Os03g0301800	13.3	10.2	3.23	7.389	13.56	19.03	3.91	2.53	5.53	0.862	5.482	14.1	6.5311	11.201	3.1679	0	20.61	10.36	4.645	7.257	5.173	8.873	4.988	2.889	1.056	7.543	4.386	9.25	2.87	3.33	19
Os03g0616300	92.8	124	111	298.8	343.5	285.5	186	195	161	119.8	144.7	132.7	121.91	134.42	137.01	191.1	154.6	150.2	218.3	218.5	234.5	204.1	189.6	192.1	142.6	126.3	127.2	153	133	148	10
Os03g0711300	390	467	447	434.3	452	416.1	463	440	555	490.6	426.5	468.5	502.9	463.93	529.03	823.9	517.3	450.7	412.5	406.4	392.3	453.2	432.6	460.1	543	509.1	502.2	452	508	499	6
Os03g0220100	106	85.7	75.4	22.17	9.04	25.09	47.8	45.5	65	139.7	94.29	187.2	68.577	74.676	57.814	11.94	61.83	48.69	43.66	37.9	38.8	57.67	29.93	61.39	143.7	98.06	103.1	72.2	45.9	74.1	20
Os03g0845500	1161	1222	1348	1236	1249	1140	1251	1459	1180	1147	1196	1286	1469.5	1400.2	1361.4	2054	1308	1377	1262	1208	1277	901.3	911.5	958.4	1215	1166	1123	1271	1216	1282	6
Os03g0369700	1.02	0.93	0	27.09	28.02	28.55	10.2	13.3	21.4	0	3.289	0.941	0	0	0.792	11.94	2.061	2.072	26.01	24.19	18.11	10.35	19.24	11.56	0	0	0	0	0	0	10
Os03g0564200	73.4	103	102	238.9	312.8	197.2	604	679	675	207.8	200.6	95.96	179.61	93.345	115.63	131.4	30.92	49.73	169.1	158.8	111.2	326.8	320.7	325	86.62	53.74	57.02	37.9	52.6	57.5	18
Os03g0212800	5.1	6.52	6.47	2.463	3.616	1.73	15.7	3.16	3.46	2.587	1.096	0.941	4.3541	1.8669	2.3759	0	5.153	4.144	0	0.806	1.724	8.873	4.276	6.5	4.225	8.486	8.772	0	1.91	5.83	5
Os03g0168400	413	445	435	474.5	466.4	475.8	269	299	321	401.8	398	359.4	409.28	393.92	401.53	477.6	392.6	451.7	350.2	408.8	358.7	405.9	420.5	300.5	386.6	348.9	348.7	525	491	539	8
Os03g0152400	577	607	599	477	413.1	423.9	667	737	606	794.9	717	789.3	695.56	794.37	788.8	1039	530.7	655.8	513.7	403.1	389.7	539	684.8	609.6	698.3	631.7	774.1	686	587	561	13
Os03g0700700	284	171	238	55.83	45.2	56.23	22.7	36.1	27.6	119.8	116.2	63.03	106.67	119.48	105.33	238.8	159.7	162.7	31.58	23.38	20.69	104.3	106.2	106.9	156.3	146.1	96.49	151	177	145	13
Os03g0666850	276	244	287	318.5	348.9	304.5	207	257	240	257.8	292.7	271	230.77	229.63	295.4	477.6	272.1	292.2	232.2	290.3	238.8	303.1	306.4	249.9	344.4	302.7	326.7	258	302	260	6
Os03g0853900	153	162	155	192.1	169	173	181	157	223	172.4	168.8	185.3	191.58	209.09	180.57	238.8	171.1	204.1	187.6	182.2	193.1	251.4	188.1	206.6	165.8	197.1	217.1	185	204	177	6
Os03g0200000	1.02	0	1.08	0	0	3.46	2.35	0	2.76	0	0	0	0	0	0	0	0	0	0	3.225	0	0	0.713	0	0	0	0	0.93	2.87	0	2

Os03g0159100	896	661	705	1933	1710	1521	166	340	542	607.8	377.2	630.3	401.66	437.79	505.27	1039	606	548.1	1479	1426	771.7	597.4	712.6	450.7	458.5	522.3	554.8	750	877	919	10
Os03g0184400	0	0	0	1.642	2.712	0	0	0	0	0.862	2.193	0	0	0	1.5839	0	5.153	2.072	0	0.806	0	0.739	0	0	1.056	4.714	2.193	2.78	6.69	1.67	1
Os03g0137050	0	0.93	1.08	0	0	0	0	0	0	0	0	0	0	0	0	0	0	0	0	0	0	2.218	0	0	0	1.886	0	0	0	0	9
Os03g0300400	55.1	50.3	78.7	36.12	28.93	51.04	31.3	11.4	262	99.15	48.24	169.3	18.505	79.343	30.095	23.88	18.55	31.08	77.1	45.96	31.9	34.01	89.79	76.56	28.52	33.94	85.52	76.8	190	39.2	20
Os03g0100010	0	0	0	4.926	0	0.865	1.57	0	1.38	0	0	0	0	0	0	0	0	0	0	0.806	1.724	0	2.851	0	0	0	0	0	0	0	2
Os03g0749500	3.06	14	17.2	3.284	2.712	2.595	2.35	3.16	3.46	6.035	9.868	26.34	9.7967	6.5342	12.671	0	11.34	14.5	3.716	1.613	9.484	3.697	7.126	2.889	12.68	17.91	26.32	9.25	9.56	13.3	20
Os03g0323500	171	268	283	239.7	263.1	257.8	438	491	487	244.9	332.2	149.6	249.27	220.29	323.12	417.9	203	185.5	152.3	179	181.1	641	545.9	465.1	216.6	176.3	177.6	264	277	342	17
Os03g0709200	25.5	37.3	22.6	26.27	18.98	45.85	20.4	13.9	15.2	31.9	19.74	24.46	5.4426	28.004	22.175	23.88	30.92	20.72	24.15	32.25	40.52	36.23	58.44	41.89	26.41	33	26.32	42.6	41.1	30	17
Os03g0364000	324	346	306	349.7	386	360.7	479	412	500	414.7	422.1	416.8	384.25	329.51	354.8	322.4	296.8	338.8	359.5	474.9	391.4	376.3	428.3	377.7	326.4	347	256.6	315	320	280	15
Os03g0283000	30.6	14	48.5	44.33	37.06	12.11	4.7	40.5	8.29	6.897	7.675	6.586	56.603	44.806	75.237	238.8	63.89	47.66	49.23	91.11	9.484	7.394	41.33	2.889	33.8	1.886	4.386	58.3	74.6	57.5	6
Os03g0247600	59.1	84.8	52.8	181.4	214.2	183.4	306	293	305	63.8	87.71	102.5	83.816	70.009	58.605	35.82	50.5	74.59	135.6	167.7	187.1	151.6	117.6	192.1	47.54	71.66	54.82	36.1	56.4	40	12
Os03g0759700	0	1.86	0	0.821	0.904	1.73	3.13	1.27	9.67	1.724	1.096	0	0	0	0.792	0	0	1.036	1.858	0	0	2.218	3.563	2.167	0	0	0	0	0.96	0	18
Os03g0672500	11.2	12.1	4.31	12.31	2.712	11.25	35.2	7.59	18.7	22.42	27.41	23.52	9.7967	9.3345	7.1277	0	3.092	3.108	13.93	5.644	6.898	17.75	9.264	14.44	11.62	1.886	2.193	8.33	7.65	5.83	5
Os03g0178300	3.06	11.2	0	0	3.616	4.325	0.78	0	0	5.173	2.193	2.822	1.0885	1.8669	3.1679	0	6.183	1.036	0.929	0.806	0	2.218	0	1.444	4.225	6.6	8.772	3.7	5.74	5.83	11
Os03g0259400	0	0	0	0	0	0	0	0	0	0	0	0	1.0885	0	0.792	0	0	0	0	0	0	0	0	0	0	0	0	0.93	0	4.17	1
Os03g0681750	104	77.3	101	82.1	75.03	87.37	139	126	151	138.8	121.7	176.9	114.29	112.01	90.284	131.4	92.75	80.81	113.3	112.9	114.7	89.47	74.11	134.3	116.2	105.6	131.6	99.9	82.2	77.5	15
Os03g0685900	1446	1461	1419	1244	1313	1240	1365	1198	1289	1636	1442	1956	1550.1	1526.2	1367.7	1743	1395	1291	1459	1420	1442	951.6	1078	1169	1320	1395	1579	1134	1274	1289	20
Os03g0332400	1188	1259	1332	1430	1584	1343	920	1042	867	1196	1160	980.3	1249.6	1225.6	1007.4	1421	914.1	1063	1447	1384	1174	767.5	880.1	826.2	890.5	861.8	956.1	908	904	886	10
Os03g0711100	2489	2865	2525	870.2	632.8	687.7	103	111	122	4745	5277	5216	3977.5	3352	3529.8	3511	3375	3826	1408	1334	1680	156	94.78	87.39	4630	6109	5647	3047	3452	3287	3
Os03g0854800	3132	3607	3201	3112	3385	2961	4020	4451	4094	3255	3404	2871	2980.4	2728.5	3389.6	4502	3261	3432	2605	2932	2616	5382	5032	4641	3501	3466	3068	3505	3433	3551	17
Os03g0805200	1627	1882	1862	2288	2589	2272	795	968	1069	1395	1481	1220	1560.9	1537.4	1647.3	2711	1687	1708	1863	1909	2034	1006	1100	1002	1597	1308	1114	1558	1765	1728	16
Os03g0817700	214	174	164	141.2	169.9	138.4	139	154	126	165.5	114	136.4	103.41	160.55	156.02	238.8	233.9	247.6	183	231.4	162.1	206.3	195.3	117.6	209.2	238.5	261	237	212	212	1
Os03g0413100	924	707	727	215.1	185.3	252.6	279	277	278	532.8	617.3	625.6	542.08	578.74	522.7	1552	856.4	822.6	447.8	397.5	550.1	319.4	298.6	405.2	626.4	899.5	962.7	675	766	715	13
Os03g0144200	251	280	302	253.7	260.3	301	225	240	253	213.8	236.8	252.1	252.54	306.17	291.44	250.8	286.5	323.2	280.5	327.4	289.7	350.5	284.3	255.7	291.6	296.1	287.3	320	295	312	1
Os03g0299700	3.06	0.93	0	0.821	1.808	1.73	0.78	0.63	0	4.311	0	0	1.0885	0	0	0	1.031	0	0	0	0	1.479	0.713	0.722	0	2.829	0	0	0	1.67	7
Os03g0188500	57.1	75.4	83	4.105	2.712	19.03	1.57	15.2	6.22	25.87	7.675	9.408	3.2656	0.9335	6.3357	155.2	309.2	173	13.93	4.838	9.484	12.57	24.23	42.61	14.79	13.2	10.96	14.8	10.5	14.2	14
Os03g0599000	197	238	153	451.5	350.7	330.4	203	266	257	137.9	96.48	76.21	129.53	83.077	148.1	214.9	99.96	106.7	227.6	249.1	156.9	366.7	474.6	316.3	110.9	87.68	57.02	123	117	156	17
Os03g0310701	182	168	181	57.47	75.93	59.69	22.7	43	22.8	179.3	190.8	196.6	215.53	196.96	209.87	179.1	197.9	158.5	89.18	100.8	83.64	18.48	18.53	37.56	158.5	192.3	162.3	167	206	167	3
Os03g0780500	456	422	393	1741	1886	1632	564	577	545	424.2	392.5	579.5	466.98	462.99	299.36	1122	297.8	443.4	1738	1767	1664	516.1	438.3	471.6	246.1	306.4	304.8	308	335	307	10
Os03g0718800	80.5	42.8	39.9	366.2	94.92	954.1	30.5	8.86	17.3	119	33.99	76.21	18.505	33.604	26.927	71.65	73.17	54.91	654.9	111.3	560.4	30.31	75.54	117	17.96	82.97	736.8	46.3	195	35	16
Os03g0206800	0	0.93	3.23	0.821	0.904	0	0	0	0	0	0	2.822	0	0	1.5839	11.94	0	0	0	0	0	0	0	0	0	0	2.193	0	0	0	6
Os03g0143200	1.02	0.93	1.08	0	6.328	0	0	0.63	0.69	0.862	1.096	1.882	1.0885	0	0.792	0	2.061	3.108	0	1.613	0	1.479	0	2.889	0	0	0	0.93	1.91	2.5	2
Os03g0807100	6.12	4.66	6.47	14.78	22.6	16.44	2.35	8.22	5.53	2.587	5.482	2.822	4.3541	3.7338	8.7116	11.94	17.52	2.072	9.29	19.35	11.21	9.612	2.851	9.389	5.282	3.771	10.96	3.7	6.69	9.16	10
Os03g0831400	11.2	6.52	9.7	11.49	11.75	15.57	23.5	24	21.4	9.484	6.578	12.23	4.3541	19.602	11.088	11.94	14.43	15.54	13.01	12.9	18.97	17.01	12.83	13	8.451	7.543	2.193	9.25	12.4	10	18
Os03g0853800	284	298	282	556.6	576.7	532	580	453	456	335.4	284	302	370.1	258.57	232.84	441.8	260.7	299.4	576	521.7	621.7	376.3	376.3	442.7	231.3	286.6	296	225	281	267	12
Os03g0275300	2.04	0	0	252.9	273	293.3	339	405	364	4.311	2.193	0	0	0	0.792	0	0	0	166.3	125	138	259.5	222.3	264.3	1.056	3.771	2.193	0	0	0	12
Os03g0108200	64.2	70.8	65.7	80.46	90.4	88.23	43.8	41.1	39.4	43.97	41.66	40.46	95.79	66.275	63.357	71.65	55.65	59.05	65.03	56.44	56.04	64.33	60.57	52.72	51.76	54.68	41.67	51.8	84.2	88.3	8
Os03g0646400	40.8	37.3	38.8	30.38	26.22	32.01	54	40.5	45.6	47.42	37.28	35.75	26.124	46.673	45.142	47.76	44.31	21.76	41.8	33.06	37.56	28.1	37.06	37.56	42.25	33	59.21	39.8	36.3	26.7	5
Os03g0820600	2276	2075	2083	1656	1715	1747	1787	1680	1761	1981	1717	2336	1803.7	2274.8	1986.3	3320	1908	1783	1973	1684	1981	1556	1686	1871	2041	2070	2441	1900	1790	1691	13
Os03g0304400	173	115	165	151.1	131.1	161.8	51.7	75.3	75.3	90.53	74.55	63.98	74.019	102.68	129.88	310.5	141.2	130.5	113.3	124.2	105.2	148.6	195.3	117	127.8	131.1	107.5	171	175	168	8
Os03g0282900	345	308	321	357.9	324.5	333	295	297	277	356.1	309.2	475.1	374.45	441.52	310.45	561.2	336	308.7	419.9	366.9	421.6	275.1	299.3	257.1	302.1	358.3	381.6	280	296	294	6
Os03g0196100	0	0.93	1.08	0.821	0	1.73	0.78	0	0.69	0.862	0	0	0	0	1.5839	11.94	1.031	1.036	0	2.419	0.862	0.739	0.713	0	0	0	0	0	0.96	2.5	6
Os03g0858800	14.3	16.8	10.8	9.031	13.56	0	2.35	4.43	0.69	11.21	13.16	19.76	3.2656	4.6673	5.5438	0	3.092	9.324	0	2.419	0	1.479	0	0	1.056	5.657	4.386	0.93	3.83	2.5	20
Os03g0239300	41.8	42.8	52.8	87.02	67.8	76.99	160	152	131	63.8	57.01	125.1	42.452	33.604	20.591	23.88	42.25	17.61	85.46	32.25	50.87	114.6	70.55	127.1	26.41	53.74	81.14	20.4	38.3	25	18
Os03g0145800	478	468	456	625.6	660.8	646.2	451	394	477	543.2	503.2	633.2	523.58	553.54	479.93	632.9	463.7	442.4	685.6	702.3	701.8	433.3	468.2	395.8	571.5	548.7	614	408	411	422	16
Os03g0425100	196	200	245	203.6	236.8	211.9	134	128	108	239.7	225.9	309.5	316.76	280.97	241.55	429.9	231.9	287	244.3	246.7	226.8	204.8	143.2	135.1	296.8	264	289.5	260	290	242	13
Os03g0377700	438	543	524	1018	1123	910.9	337	348	278	433.7	433.1	321.8	501.81	451.79	467.26	632.9	419.4	456.9	843.5	990.1	1070	240.3	230.9	191.4	381.3	331.9	276.3	402	381	446	10
Os03g0203100	303	298	255	944.1	985.3	939.4	225	331	243	217.3	182	183.5	247.09	302.44	198.78	370.2	249.4	318.1	854.6	911.9	926.9	250.7	316.4	242.7	150	190.5	164.5	208	244	214	10
Os03g0787300	5704	4958	6071	1154	1165	1235	734	854	722	4097	4010	3221	2718	2932	3223.3	3952	3307	3097	1053	875.6	960.5	891	901.5	819.7	3809	2940	2750	2539	2244	2707	3
Os03g0202500	283	276	207	467.1	499	444.6	210	268	285	232.8	258.7	301.1	237.3	239.9	248.68	489.6	254.5	273.5	451.5	423.3	496.6	281.7	267.2	268.7	240.9	270.6	203.9	283	316	246	10
Os03g0371000	4.08	5.59	2.16	2.463	0	2.595	0	0	0.69	2.587	2.193	1.882	4.3541	0	7.1277	0	5.153	0	1.858	1.613	0.862	0	7.126	0.722	2.113	0	0	0	5.74	4.17	4
Os03g0321500	1389	1219	1295	893.2	874.1	892.7	1147	1236	908	1112	1047	1085	991.64	1076.3	1141.2	1469	1336	1296	872.3	948.2	856.2	1034	1174	948.3	1008	1087	1125	1124	1029	1082	13
Os03g0796000	339	265	365	336.6	327.2	282	541	713	492	402.6	343.2	396.1	566.03	472.33	467.26	465.7	307.1	310.8	292.6	312.8	227.6	302.4	345.6	359.7	381.3	247	315.8	369	267	330	5
Os03g0803200	1.02	0	2.16	5.747	4.52	2.595	7.83	15.2	2.07	0.862	2.193	0.941	0	0	1.5839	0	0	0	0.929	1.613	0.862	3.697	3.563	2.889	2.113	0.943	6.579	0	1.91	0	18
Os03g0104901	0	0	0	0.821	0	0	0	0	0	0	0	0	0	0	0	0	0	0	0	0	0	0	0	0	0	0	0	0	0	0	2
Os03g0287800	8.16	4.66	5.39	0.821	0.904	6.055	0	0.63	0.69	9.484	1.096	2.822	2.177	2.8004	2.3759	0	6.183	8.288	1.858	0	3.449	0.739	0	1.444	6.338	0.943	10.96	3.7	2.87	5	11
Os03g0590700	80.5	65.2	49.6	82.1	90.4	94.29	145	118	149	95.7	60.3	110.1	58.78	72.809	87.908	107.5	78.32	58.02	88.25	113.7	112.1	127.2	101.2	124.2	63.38	72.6	83.33	80.5	76.5	82.5	18
Os03g0199100	13.3	8.38	8.62	7.389	5.424	19.03	14.1	9.49	22.8	10.35	9.868	9.408	6.5311	7.4676	5.5438	11.94	16.49	9.324	14.86	15.32	16.38	18.48	39.19	29.61	6.338	12.26	2.193	6.48	3.83	7.5	17
Os03g0209700	4.08	9.31	4.31	5.747	4.52	4.325	4.7	1.27	4.84	1.724	1.096	1.882	3.2656	0.9335	0.792	0	7.214	5.18	5.574	4.031	5.173	4.436	4.276	2.889	3.169	4.714	0	0.93	4.78	3.33	14
Os03g0170800	0	0	1.08	0	0	0	0	0	0	0	0	0	0	0	0	0	0	0	0	0	0	0	0	0	0	0	0	0	0	0	14
Os03g0646300	0	0	0	0	0.904	0.865	0	1.9	4.15	0	0	0.941	1.0885	0	1.5839	0	0	0	0	1.613	0	0	0.713	0	2.113	0.943	0	0	0	0	18
Os03g0293600	1.02	0.93	1.08	1.642	0.904	0	0.78	0.63	2.76	1.724	0	0.941	0	0	0.792	0	1.031	4.144	1.858	1.613	1.724	0.739	2.851	1.444	0	2.829	0	4.63	1.91	0.83	1
Os03g0593600	0	1.86	0	7.389	18.98	7.785	3.13	0	0	0	0	0	2.177	0	0	0	0	1.036	12.08	8.063	12.93	0	0	0.722	0	0	0	0	0	0	10
Os03g0805766	0	0.93	0	0.821	0	0	1.57	0.63	0	0	2.193	0	0	0	0	0	0	3.108	0	1.613	0	0	1.425	0	0	0	0	2.78	0	0	1
Os03g0138100	954	1020	971	985.2	911.2	895.3	868	917	892	819.9	905.6	750.8	943.75	828.9	981.25	1672	856.4	948	698.6	807.9	780.3	987.1	937.8	845	894.7	827.8	809.2	1001	923	983	6
Os03g0342100	125	141	53.9	60.75	0.904	268.2	51.7	3.16	7.6	25	9.868	124.2	0	1.8669	24.551	35.82	93.78	78.74	29.73	41.93	16.38	228.5	97.63	227.5	28.52	135.8	495.6	53.7	144	17.5	11
Os03g0399466	122	115	119	40.23	49.72	44.12	97.9	45.5	91.2	101.7	153.5	132.7	128.45	159.62	101.37	429.9	143.2	132.6	87.32	74.98	67.25	47.32	52.02	67.17	145.8	136.7	208.3	96.2	94.7	82.5	13
Os03g0213800	47.9	65.2	45.3	184.7	222.4	190.3	134	124	89.1	64.66	35.08	55.51	43.541	68.142	41.182	83.59	48.43	61.13	188.6	211.2	250	97.6	134	87.39	24.3	28.29	67.98	37.9	64.1	30	10
Os03g0439500	55.1	14.9	23.7	190.5	149.2	266.4	54.8	30.4	51.8	12.07	7.675	16.93	33.744	15.869	11.879	11.94	18.55	19.68	280.5	185.4	317.3	62.11	50.6	113.4	12.68	42.43	184.2	41.6	63.1	20	10
Os03g0440200	2.04	0	0	1.642	0.904	0.865	3.13	4.43	1.38	3.449	3.289	2.822	2.177	0.9335	2.3759	0	3.092	5.18	0.929	0	5.173	2.958	4.988	0.722	2.113	5.657	2.193	4.63	3.83	1.67	1
Os03g0103400	17.3	19.6	11.9	11.49	20.79	13.84	14.9	25.9	20	12.93	6.578	17.88	4.3541	14.935	5.5438	0	4.122	5.18	13.01	3.225	5.173	19.22	12.11	14.44	7.395	12.26	8.772	1.85	7.65	6.66	18
Os03g0412300	3.06	0.93	0	1.642	1.808	3.46	3.91	3.16	0.69	3.449	3.289	1.882	1.0885	3.7338	2.3759	0	5.153	2.072	0	0.806	7.76	1.479	8.552	12.28	0	4.714	17.54	2.78	3.83	0.83	11
Os03g0784850	1.02	0	0	2.463	0	6.92	0	0	0	0	0	0	0	0.9335	0	0	0	0	0.929	0	2.587	2.958	2.138	0.722	0	0.943	0	0	0.96	1.67	2
Os03g0833800	246	235	192	224.1	217	278.5	288	289	236	136.2	205	194.7	179.61	196.02	162.35	394.1	244.2	206.2	209	262.8	231.1	312	297.9	279.5	226.1	257.4	217.1	348	276	285	6
Os03g0826000	3.06	4.66	6.47	0	1.808	0	2.35	0	0	5.173	2.193	3.763	2.177	7.4676	0.792	11.94	6.183	9.324	1.858	4.031	1.724	1.479	1.425	5.056	7.395	4.714	10.96	11.1	8.61	11.7	13
Os03g0116500	712	629	626	640.4	681.6	650.5	496	517	471	664.7	668.8	653.9	646.58	675.82	715.94	1337	775	763.6	658.6	641	610.4	633.7	585.8	517.8	699.3	723.2	671	778	755	777	13
Os03g0363100	0	0	0	0	0	0	0	0	0	1.724	0	0	0	0	0	0	0	0	0	0	0	0	0.713	0.722	0	0	0	0	0	0	7
Os03g0376100	3364	2997	3232	2800	2622	3047	1721	1904	1714	3729	3568	5225	3380.9	3623.7	3346.1	5099	3331	3591	3844	3500	3638	1768	1850	2263	3494	4141	4053	2774	2994	2722	20
Os03g0306800	3488	3334	4235	2701	3186	2189	1296	1311	1303	4742	5130	4518	5578.7	4101.6	4747.8	5744	3075	3345	3137	3075	2902	593.7	592.9	646.4	3230	2135	1649	2253	2842	2908	13
Os03g0134500	0	0.93	3.23	4.105	0.904	1.73	1.57	5.06	1.38	1.724	2.193	1.882	3.2656	0	3.9598	0	0	0	0.929	0	0	0.739	0.713	0	0	0.943	0	1.85	0.96	0	4
Os03g0265100	1084	1088	1015	490.1	369.7	532.9	507	318	454	1547	1657	1753	1340	1430	1366.9	2436	1509	1315	910.4	879.7	937.2	349	292.9	465.8	1555	1832	1844	1190	1307	1106	3
Os03g0284000	80.5	82	62.5	59.93	66.89	72.66	91.6	115	118	70.7	53.72	89.38	97.967	78.41	60.981	107.5	49.47	44.55	86.39	68.53	71.56	68.76	88.37	63.56	52.82	55.63	67.98	61.1	67.9	52.5	18
Os03g0153100	116	43.8	103	32.84	12.66	44.12	1.57	4.43	2.76	101.7	55.92	97.85	45.718	99.879	85.532	191.1	229.8	128.5	53.88	48.38	62.94	37.71	77.68	42.61	239.8	314.9	688.6	264	432	230	11
Os03g0565300	530	555	634	652.7	620.1	590.7	754	927	734	627.7	655.6	524	768.5	672.09	779.29	1027	566.8	524.2	555.5	693.4	528.5	794.8	921.4	668.8	707.8	630.8	576.7	792	803	753	6
Os03g0360300	0	0	2.16	0.821	4.52	6.055	4.7	1.9	7.6	0.862	2.193	0	5.4426	1.8669	0.792	0	4.122	3.108	2.787	0.806	2.587	2.218	2.851	3.611	4.225	1.886	0	2.78	1.91	2.5	18
Os03g0216400	0	0.93	2.16	18.06	27.12	22.49	16.4	13.3	17.3	0	0	0	0	0	0	0	0	0	17.65	14.51	14.66	2.958	7.126	7.945	0	0	0	0	0.96	0	12
Os03g0712500	5.1	13	4.31	11.49	9.04	6.92	3.13	7.59	1.38	6.035	7.675	6.586	10.885	4.6673	11.088	23.88	11.34	17.61	2.787	1.613	6.036	8.133	9.264	4.333	7.395	18.86	8.772	10.2	10.5	17.5	13
Os03g0737300	91.8	105	116	22.17	33.45	25.09	52.5	70.8	56.7	108.6	55.92	86.56	60.957	79.343	57.814	107.5	91.72	103.6	40.87	26.61	26.73	101.3	148.2	80.17	70.78	65.06	59.21	69.4	38.3	57.5	14
Os03g0310900	73.4	400	61.4	1.642	1.808	0.865	0.78	1.27	0.69	21.55	6.578	11.29	25.036	18.669	27.719	35.82	57.71	65.27	4.645	5.644	13.8	2.218	0.713	5.056	20.07	13.2	21.93	37	38.3	38.3	14
Os03g0188400	0	0	0	0	0	0	0	0	0	0	0	0	1.0885	0.9335	0	0	0	0	0	0	0	0.739	0	0	0	0	0	0	0	0	4
Os03g0129000	282	265	240	316.1	297.4	287.2	307	229	314	331.1	291.6	326.5	280.84	251.1	304.11	298.5	231.9	282.8	379	343.5	327.6	295.8	277.2	271.6	308.5	342.3	342.1	328	291	292	16
Os03g0201200	193	152	172	199.5	233.2	231	204	194	176	182.8	188.6	210.7	198.11	216.56	172.65	441.8	190.6	193.7	264.8	300.7	274.2	193	172.5	162.5	180.6	204.6	252.2	173	167	160	6
Os03g0792600	99.9	138	157	220.8	228.7	202.4	168	146	142	155.2	168.8	150.5	154.57	163.35	125.13	322.4	155.6	176.1	211.8	207.2	238	131.6	154.6	102.6	133.1	150.9	129.4	141	154	148	6
Os03g0690600	701	855	763	378.5	426.7	412.6	385	415	352	574.2	650.2	585.4	641.14	518.07	812.56	812	790.4	747	320.5	365.2	285.4	553.1	610.7	478.8	771.1	749.6	587.7	933	770	956	3
Os03g0607200	18.4	10.2	16.2	4.926	8.136	11.25	4.7	3.16	2.07	5.173	21.93	5.645	14.151	9.3345	7.9197	0	10.31	13.47	10.22	4.838	6.036	2.958	4.276	1.444	13.73	10.37	37.28	9.25	5.74	6.66	11
Os03g0859500	83.6	86.6	69	212.6	216	179.9	235	248	223	72.42	98.68	47.04	76.196	88.678	117.21	83.59	60.8	95.32	156.1	159.6	199.2	271.4	213.8	205.8	70.78	55.63	87.72	99.9	103	88.3	12
Os03g0745400	3116	2571	3253	2157	2386	2429	1516	1979	1622	1899	2198	1886	2763.8	2518.5	2659.4	5612	3417	3257	1909	2520	1955	2536	2430	1865	2834	2783	2682	3286	3055	3282	13
Os03g0831900	790	776	776	633	617.4	608.1	853	856	929	912.2	924.3	1133	899.12	888.65	840.28	1481	917.2	882.7	791.5	730.5	780.3	722.4	687	798.1	985.6	1148	1028	832	799	897	13
Os03g0177400	7111	5767	5867	6938	7085	7586	7589	6646	8120	7321	6202	10103	6927.3	7153	5277.7	7726	5192	5985	7609	6714	7534	5816	6243	6614	5904	7208	7879	4375	5305	4141	15
Os03g0222100	0	0	1.08	0	0	0	0	0	0	0	0	0	0	0	0	0	0	0	0	0	0.862	0	0	0	0	0	0	0	0	0	16
Os03g0611200	1347	1261	1395	1506	1684	1374	1747	2066	1919	1223	1316	1704	1562	1488.9	1340	2639	1541	1415	1437	1601	1574	1510	1373	1694	1356	1351	1204	1211	1498	1275	6
Os03g0100800	1.02	0	2.16	0.821	0	2.595	0.78	2.53	2.76	0.862	2.193	0	0	0.9335	0	0	0	1.036	0	2.419	5.173	2.218	1.425	4.333	2.113	0.943	0	0	0	0.83	9
Os03g0715900	3.06	0	0	1.642	0.904	0	0	1.27	0	0	0	0.941	0	0	2.3759	0	0	0	0	0	0	0	0	0	0	0	0	0	0	0	19
Os03g0684000	763	726	680	552.5	490.9	520.8	651	703	663	788.9	833.3	827	840.34	838.24	912.34	2078	1013	868.2	709.7	717.6	662.2	668.4	684.1	705.6	961.3	1013	842.1	1091	956	1061	13
Os03g0243800	98.9	84.8	65.7	96.88	91.3	117.6	128	145	152	87.94	89.9	98.79	62.046	48.539	88.7	119.4	112.3	94.28	93.82	88.69	68.12	113.1	146.1	132.2	68.66	65.06	133.8	74	80.3	83.3	18
Os03g0753100	2.04	0.93	2.16	0	0.904	0	0	0	1.38	5.173	14.25	2.822	0	3.7338	5.5438	11.94	4.122	4.144	0	0.806	1.724	0	0	0	3.169	7.543	2.193	1.85	3.83	0.83	13
Os03g0684300	52	59.6	59.3	47.62	46.1	50.17	43.1	32.9	53.9	66.39	59.21	63.98	78.373	71.876	55.438	35.82	56.68	41.44	40.87	46.76	37.08	31.79	35.63	20.95	58.1	76.37	41.67	47.2	61.2	66.6	3
Os03g0195400	53	73.6	64.7	38.59	51.53	48.44	36	60.1	32.5	26.73	38.37	21.64	65.311	51.34	64.941	131.4	41.22	60.09	35.3	23.38	16.38	47.32	48.46	34.67	42.25	36.77	32.89	54.6	64.1	55.8	13
Os03g0679000	498	480	458	439.2	440.2	486.2	568	602	587	556.1	549.3	620.9	525.76	497.53	523.49	656.8	476.1	447.6	478.4	515.2	556.1	579.7	548	625.5	594.7	613.8	660.1	406	413	432	5
Os03g0388100	910	662	869	319.4	259.4	301.9	265	292	263	746.7	837.6	1447	1088.5	1073.5	1053.3	2197	1808	1254	698.6	582.9	624.2	244.7	306.4	319.2	1283	1731	1689	1102	1206	1048	13
Os03g0159600	10.2	15.8	18.3	11.49	9.04	15.57	4.7	3.8	10.4	6.897	14.25	10.35	7.6196	7.4676	7.9197	23.88	8.244	6.216	13.93	16.93	1.724	1.479	4.276	5.778	7.395	3.771	4.386	2.78	4.78	0	6
Os03g0176100	0	0.93	2.16	1.642	2.712	2.595	0	3.16	0	1.724	1.096	1.882	0	0	2.3759	0	0	4.144	0.929	0	0	0	1.425	0.722	1.056	0	0	1.85	1.91	0.83	2
Os03g0117200	313	318	314	305.4	255.8	274.2	342	336	386	435.4	403.5	373.5	398.4	389.25	392.02	346.3	298.9	321.2	288	297.5	263	283.2	280.1	275.9	330.6	287.6	280.7	270	301	294	7
Os03g0819900	473	512	462	617.4	622.8	607.3	1358	1281	1338	539.7	474.7	464.8	457.18	516.2	522.7	764.2	446.2	469.3	565.7	538.6	550.1	930.9	870.1	922.3	420.4	447.8	572.4	381	385	396	18
Os03g0133500	32.6	25.1	11.9	50.08	28.02	36.33	18	19	24.2	25	31.8	19.76	13.062	16.802	9.5036	11.94	12.37	20.72	20.44	33.06	37.08	27.36	32.78	26.72	23.24	19.8	8.772	18.5	24.9	20	2
Os03g0203800	1179	873	1040	324.3	292.9	335.6	391	406	435	685.4	707.2	803.5	919.8	830.77	887	1803	1371	1062	340	402.3	348.3	477.6	470.3	533.7	891.6	963.6	905.7	964	918	928	13
Os03g0575500	439	471	413	475.3	611.1	474.9	1246	1239	1321	578.5	592.1	725.4	735.84	608.61	624.07	597	492.6	536.7	530.4	463.6	501.8	642.5	611.4	832.7	481.7	412	434.2	407	378	457	18

Os03g0719850	0	0	0	0	0	0	0	0	0	0	0	0	1.0885	0	0.792	0	0	0	0	0	0.862	0	0	0	0	1.886	0	0	0	0.83	4
Os03g0809800	138	138	95.9	172.4	159.1	139.3	266	250	232	161.2	172.1	168.4	153.48	143.75	151.27	155.2	111.3	106.7	145.8	158	151.8	238.1	231.6	207.3	133.1	146.1	96.49	125	116	121	18
Os03g0644400	547	635	571	1534	1533	1551	538	643	531	735.4	651.3	716.9	862.11	758.9	809.39	1469	927.5	852.7	1956	2054	2359	775.6	612.9	624.7	782.8	1044	1037	896	900	1054	16
Os03g0719800	8.16	19.6	8.62	8.21	1.808	4.325	6.26	10.8	9.67	20.69	12.06	4.704	6.5311	15.869	13.463	0	12.37	14.5	1.858	8.869	0	19.22	7.126	11.56	17.96	12.26	13.16	16.7	27.7	25	1
Os03g0717000	226	119	156	116.6	105.8	126.3	24.3	45.5	30.4	53.46	50.43	47.98	64.223	61.608	54.646	119.4	136	103.6	184.9	129	153.5	40.67	37.77	35.39	47.54	64.11	76.75	43.5	63.1	40.8	16
Os03g0374600	15.3	22.4	11.9	22.99	27.12	19.9	21.1	19	18	20.69	14.25	15.99	15.239	33.604	17.423	35.82	28.85	23.83	24.15	26.61	31.04	28.1	25.65	39.72	19.01	20.74	43.86	26.8	19.1	18.3	11
Os03g0825400	345	319	342	483.6	480.9	529.4	345	313	393	395.7	302.6	449.7	399.49	419.12	313.62	573.2	366.9	318.1	562	541.8	627.7	284.7	322.8	275.9	306.3	365.8	425.4	289	307	254	16
Os03g0724300	101	65.2	101	53.36	61.47	39.79	28.2	32.9	31.1	79.32	75.65	79.03	89.259	70.009	83.948	167.2	131.9	102.6	76.17	91.92	54.32	28.84	29.22	23.11	70.78	59.4	72.37	87.9	70.8	80.8	13
Os03g0318400	5.1	6.52	16.2	4.105	3.616	6.055	1.57	5.06	0	2.587	3.289	5.645	3.2656	1.8669	1.5839	0	1.031	4.144	9.29	2.419	1.724	4.436	4.276	2.889	9.507	2.829	0	0.93	3.83	1.67	14
Os03g0839300	871	970	825	1052	1118	1010	1722	1532	1752	1163	1076	1354	1001.4	886.78	846.61	1278	743	817.4	1060	1143	1125	1147	1091	1392	899	899.5	958.3	724	714	702	18
Os03g0290400	58.1	41.9	43.1	29.56	29.83	37.2	47	53.8	42.8	55.18	63.59	63.03	51.16	51.34	54.646	35.82	59.77	53.87	41.8	38.7	56.04	30.31	33.49	33.22	55.99	89.57	107.5	50	52.6	51.7	11
Os03g0686800	4.08	6.52	0	4.105	6.328	5.19	15.7	2.53	12.4	7.76	2.193	15.05	8.7082	6.5342	3.9598	11.94	9.275	4.144	5.574	4.838	0.862	7.394	13.54	5.778	4.225	2.829	8.772	9.25	0	5.83	5
Os03g0262500	925	797	1028	728.2	678	683.4	593	579	583	625.9	562.5	515.6	620.46	660.88	699.31	1278	925.4	816.4	648.4	709.5	703.6	1099	825.2	686.9	740.5	787.3	802.6	928	943	916	13
Os03g0183000	43.8	31.7	32.3	99.34	84.97	91.7	35.2	12.7	73.9	15.52	15.35	17.88	14.151	31.737	23.759	71.65	79.35	95.32	106.8	52.41	29.32	153.1	106.2	105.4	45.42	18.86	37.28	41.6	28.7	45.8	9
Os03g0346500	4.08	9.31	5.39	18.88	10.85	13.84	5.48	1.27	1.38	4.311	6.578	9.408	8.7082	9.3345	5.5438	0	13.4	6.216	27.87	28.22	23.28	2.218	3.563	1.444	13.73	13.2	15.35	21.3	6.69	10.8	16
Os03g0242300	782	360	658	280	169.9	307.1	42.3	34.8	39.4	719.9	508.7	1059	761.96	804.64	704.85	1970	971.8	921	808.2	733.7	716.5	40.67	55.59	52.72	1122	825	875	768	536	706	13
Os03g0657000	628	547	568	621.5	640	577	698	664	654	664.7	610.7	570.1	623.72	654.35	673.17	1349	615.2	613.3	745.9	746.6	644.1	709.8	605.7	629.1	653.9	584.6	655.7	646	605	638	6
Os03g0772600	2093	1990	2239	1937	1861	1388	808	722	858	2274	1748	1567	1365	1243.4	1553	2066	1486	1436	1589	1814	1574	739.4	959.2	972.1	1503	1410	1421	1273	1237	1149	14
Os03g0143100	0	0	0	0	0	0	0	0	0	0	0	0	0	0	0	0	0	0	0	0	0	0	0	0	0	0	0	0	0.96	0	1
Os03g0182000	0	0	0	1.642	0	0	0	2.53	0	0	0	0	0	0	0.792	0	0	0	0	0	0	0.739	0	0	0	0	0	0.93	0	0	18
Os03g0307600	0	0	0	0	0	0.865	0	0.63	0	0	0	0	0	0	0	0	0	0	0	0	0	0	0	0	0	0.943	0	0	0	0	2
Os03g0248250	0	0	1.08	3.284	4.52	4.325	18	11.4	6.91	0	0	0	0	0	1.5839	0	1.031	0	0	0.806	2.587	11.09	4.988	0.722	0	0	0	0.93	0	0	18
Os03g0748500	5.1	2.79	1.08	0	0	0	0	1.9	1.38	1.724	0	0.941	1.0885	0	2.3759	0	2.061	3.108	0.929	2.419	0	1.479	1.425	7.945	1.056	0.943	4.386	0.93	0.96	1.67	9
Os03g0351100	2023	2009	2137	1594	1525	1533	2245	2492	2213	2513	2307	2531	3668.3	3112.1	3456.9	3320	2889	2399	1901	1963	2017	1355	1615	1543	2879	2765	2651	2843	2617	2579	3
Os03g0856200	982	1049	1078	1351	1438	1412	1514	1496	1350	1049	992.2	964.3	1022.1	1015.6	1059.7	1158	1083	1018	1510	1226	1505	1087	1171	1171	1014	921.2	991.2	956	961	1008	12
Os03g0597600	108	105	87.3	52.54	41.58	26.82	65.8	43	33.9	87.94	99.77	69.62	54.426	77.476	52.27	95.53	120.6	109.8	78.96	32.25	50.87	22.18	22.8	45.5	72.89	49.03	89.91	95.3	82.2	96.6	3
Os03g0123200	201	174	164	237.3	226.9	218	160	133	151	195.7	140.3	223.9	140.42	181.09	144.93	310.5	159.7	137.8	228.5	179.8	225.9	147.9	146.1	130.7	166.9	196.1	166.7	151	152	144	6
Os03g0602600	234	319	323	1385	1626	1283	448	552	406	247.4	300.4	216.4	305.87	274.43	183.74	358.2	126.8	235.2	1002	1383	1246	477.6	489.6	422.5	135.2	100.9	105.3	147	190	219	10
ENSRNA04947228	0	0	0	0	0	0	0	0	0	0	0	0	0	0	0	0	0	0	0	3.225	0	0	0	0	0	0	0	0	0	0.83	16
ENSRNA04947235	0	0	0	0	0	0	0	0	0	0	0	0.941	0	0	0	0	0	0	0	0	0	0	0	0.722	0	0	0	0	0	0	20
ENSRNA04947070	0	0	0	0.821	0	0	0	0.63	0	0	1.096	0	0	0.9335	0	0	0	0	0	0	0	0	0	0	0	0	0	0	0	0	7
ENSRNA04947074	2.04	0	0	0	0	0	0	0	0.69	0	0	2.822	2.177	0	0.792	0	0	0	0	0	0	0	1.425	0.722	1.056	0	0	2.78	0	0	20
ENSRNA04947076	0	0	0	0	0	0	0.78	0	0	0	0	0	0	0	0	0	0	0	0	0	0	0	0.713	0.722	0	0	0	0.93	0	0	17
ENSRNA04946915	0	0	0	0	0	3.46	0	0	0	0	0	0	0	0	0	0	0	0	0	0	0	0	0	0	3.169	0	0	0.93	1.91	0	1
ENSRNA04946916	0	0	1.08	0.821	0	0	0	0	0	0.862	0	0	0	0	0	0	0	1.036	0	1.613	0.862	0.739	0.713	0	0	0	0	0	1.91	0	16
ENSRNA04946918	3.06	0	0	4.926	0.904	1.73	0	0.63	0	0	0	0	0	0	0	0	0	1.036	1.858	0.806	0.862	0	0	0	0	0	0	0	1.91	0	2
ENSRNA04946926	2.04	4.66	4.31	0.821	2.712	0	0	1.9	0.69	1.724	4.386	3.763	5.4426	4.6673	5.5438	0	5.153	7.252	5.574	4.031	2.587	4.436	2.851	3.611	3.169	2.829	0	4.63	2.87	6.66	1
ENSRNA04947092	0	1.86	0	1.642	0	0.865	0.78	0.63	0	0	0	0.941	1.0885	0.9335	2.3759	0	1.031	1.036	0	0.806	0	0	2.138	0.722	1.056	0	13.16	0	0.96	0.83	11
ENSRNA04947099	2.04	0.93	5.39	0	0	0	0	0	0	3.449	0	0.941	1.0885	4.6673	0.792	0	3.092	2.072	0.929	5.644	0	0.739	0.713	0.722	0	5.657	0	1.85	0.96	0	1
ENSRNA04947100	1.02	1.86	0	3.284	1.808	4.325	2.35	1.9	2.76	0.862	0	0.941	0	0	3.1679	0	2.061	1.036	0	0	0.862	2.218	3.563	0.722	0	0.943	2.193	4.63	1.91	4.17	2
ENSRNA04947104	10.2	24.2	9.7	4.105	11.75	6.055	20.4	6.96	11.1	33.63	21.93	31.05	35.921	24.27	24.551	11.94	24.73	21.76	11.15	10.48	8.622	15.53	4.988	11.56	26.41	22.63	13.16	25	15.3	25	3
ENSRNA04947109	71.4	88.5	55	38.59	39.77	33.74	45.4	41.7	28.3	80.18	70.17	93.14	138.24	112.01	100.58	71.65	90.69	70.45	55.74	41.93	35.35	46.58	34.21	47.67	82.4	80.14	54.82	102	48.8	65.8	3
ENSRNA04947110	####	####	####	13982	15396	12757	####	####	####	27686	20989	20376	16768	19868	17472	12789	24973	23975	11813	11576	20272	14190	10915	11711	20131	18081	30359	####	####	####	3
Os02g0607500	0	0	0	0	0.904	0	0	0	0	0	0	0	0	0	0.792	0	0	0	0	0	0	0	0	0	0	0	0	0	0	0	4
Os02g0749900	201	171	157	144.5	141	156.6	134	143	158	180.2	208.3	207	217.7	186.69	195.62	226.9	231.9	183.4	194.2	167.7	188.8	127.9	126.1	120.6	191.2	199.9	206.1	166	176	180	3
Os02g0832400	370	550	445	509.8	528.8	561.4	107	198	162	135.4	143.6	99.73	207.91	149.35	221.75	429.9	296.8	335.7	116.1	125.8	97.43	595.2	560.8	325	188	205.5	96.49	399	348	436	8
Os02g0298100	86.7	134	93.7	308.7	400.5	261.2	402	481	453	86.22	130.5	123.2	131.71	70.942	78.405	143.3	84.5	99.46	175.6	308	189.7	521.3	453.2	468	91.9	82.03	67.98	82.3	80.3	95	12
Os02g0671400	510	522	541	197	226	171.3	166	192	178	324.2	387	324.6	312.41	340.71	404.69	668.7	511.1	511.8	155.1	169.3	149.2	258	214.5	145.2	367.6	462.9	447.4	483	543	531	13
Os02g0119400	681	659	697	428.5	452	444.6	669	734	724	706.1	673.2	724.4	704.27	649.68	735.74	310.5	675	662	421.7	428.9	451.8	652.1	656.3	698.4	814.5	699.6	640.3	628	658	722	5
Os02g0128800	132	86.6	85.1	86.2	60.57	95.16	90.8	88.6	91.9	83.63	73.46	76.21	100.14	82.144	82.364	83.59	77.29	94.28	75.25	76.6	64.67	102	108.3	104.7	83.45	88.63	96.49	104	66	80.8	19
Os02g0177800	1406	1802	1568	2087	2197	2113	1809	2094	1975	1561	1667	1180	1792.8	1731.6	2016.3	2090	1822	1738	1815	1871	1729	2382	2159	2043	1867	1686	1502	2043	1912	2341	17
Os02g0571300	27.5	27	8.62	16.42	26.22	22.49	30.5	37.3	52.5	18.11	28.51	24.46	2.177	10.268	14.255	0	31.95	21.76	13.93	13.71	20.69	68.76	40.62	67.89	24.3	34.89	17.54	28.7	17.2	10	9
Os02g0106966	15.3	29.8	9.7	22.17	19.89	20.76	5.48	5.06	0.69	2.587	10.96	4.704	3.2656	6.5342	7.1277	0	2.061	11.4	2.787	7.257	4.311	18.48	23.52	5.056	8.451	0.943	4.386	20.4	15.3	19.2	8
Os02g0698100	78.5	99.7	64.7	31.2	35.25	32.01	44.6	47.4	49.8	61.22	46.05	57.39	31.567	48.539	34.055	35.82	68.02	65.27	40.87	45.96	31.04	57.67	55.59	50.56	68.66	68.83	50.44	62.9	53.6	53.3	14
Os02g0208675	0	0	1.08	0	0	0	0	0.63	0	0	1.096	0.941	0	0	0	0	0	0	0.929	0	0	0.739	0	0	2.113	0.943	0	0.93	0	0.83	1
Os02g0694800	514	814	724	1172	1297	1087	1919	2072	2039	821.7	934.1	982.2	1009.1	886.78	759.5	1087	568.9	728.3	1003	1193	1170	1763	1668	1837	837.7	657.2	714.9	640	822	835	18
Os02g0744650	21.4	15.8	14	9.852	9.04	6.92	7.83	12	10.4	8.622	7.675	7.527	7.6196	5.6007	7.1277	11.94	16.49	15.54	11.15	11.29	3.449	23.66	22.8	18.06	15.85	8.486	19.74	5.55	12.4	7.5	9
Os02g0582400	571	564	578	727.4	710.5	652.2	999	1004	984	780.3	730.2	725.4	684.68	605.81	645.45	525.4	567.8	598.8	735.7	788.5	736.3	757.9	760.4	851.5	627.5	663.8	568	580	565	612	18
Os02g0749300	1874	2390	2277	2687	2688	2552	1401	1823	2103	3163	2854	3360	3217.7	2761.1	3152.8	2424	2031	2490	2517	2948	3345	1789	1928	1674	2623	2785	2224	1914	1999	2174	16
Os02g0753000	1.02	0	0	4.105	0.904	1.73	1.57	0	0.69	0	0	0	0	0	0	0	2.061	2.072	1.858	0	0	0	0	0.722	0	0	2.193	0	0	0.83	2
Os02g0164400	0	0	0	0	0	0	0	0	0	0	0	0	0	0	0	0	0	0	0	0	0	0	0	0	0	0.943	0	0	0	0	1
Os02g0719700	38.7	13	20.5	30.38	31.64	33.74	25.1	16.4	13.8	25.87	20.83	43.28	40.275	23.336	22.967	11.94	14.43	18.65	38.09	33.06	29.32	14.79	8.552	2.889	13.73	19.8	24.12	14.8	20.1	13.3	16
Os02g0805400	7.14	4.66	5.39	2.463	5.424	6.055	3.13	2.53	2.07	7.76	5.482	4.704	1.0885	0	0.792	0	8.244	1.036	0.929	0.806	2.587	3.697	4.276	2.889	4.225	6.6	10.96	9.25	1.91	6.66	11
Os02g0621850	2.04	0	0	3.284	5.424	3.46	0	0	0	3.449	2.193	1.882	1.0885	0.9335	0	0	0	0	1.858	5.644	1.724	0.739	0	0	0	0	0	0	0.96	0	10
Os02g0669700	12.2	12.1	16.2	9.852	7.232	19.9	17.2	15.8	10.4	14.66	4.386	6.586	14.151	13.068	9.5036	0	19.58	8.288	21.37	10.48	18.11	10.35	17.1	11.56	15.85	12.26	28.51	14.8	29.6	10.8	11
Os02g0689550	0	1.86	0	1.642	0.904	1.73	0	0	0	0.862	2.193	0.941	0	0	0	0	1.031	1.036	0	0	2.587	0.739	1.425	1.444	1.056	2.829	0	2.78	0	0.83	1
Os02g0282600	69.3	91.3	88.4	284.1	349.8	261.2	171	203	201	45.7	60.3	47.98	55.515	57.874	63.357	95.53	53.59	70.45	208.1	233.8	177.6	214.4	138.3	231.8	51.76	39.6	37.28	55.5	66	68.3	12
Os02g0118400	103	128	88.4	114.1	75.93	130.6	93.2	96.1	107	96.56	94.29	95.96	57.692	90.545	72.861	95.53	109.2	78.74	97.54	111.3	103.5	116.1	86.23	107.6	78.17	102.8	109.6	92.5	113	102	9
Os02g0551900	1099	1226	1242	1121	1129	1004	1582	1715	1624	1450	1551	1483	1667.6	1448.7	1516.6	1170	1076	1119	1066	1122	1015	1176	1234	1281	1372	1307	1285	1166	1274	1279	5
Os02g0737300	0	0	0	0.821	0	0	0	0	0	0.862	0	0	0	0	0	0	0	1.036	0	0	0	0	0.713	0	0	0	0	0	0	0	7
Os02g0213100	0	0	1.08	1.642	7.232	0.865	11	2.53	22.8	1.724	0	0	1.0885	0	0	0	0	0	2.787	6.45	0	65.07	27.08	80.89	1.056	4.714	2.193	0	0.96	0	9
Os02g0118200	761	631	765	433.5	429.4	429.9	585	588	547	551.8	468.2	424.3	462.62	479.79	602.69	716.5	557.5	501.4	327	350.7	310.4	629.2	678.4	562.6	536.6	517.6	493.4	523	501	541	14
Os02g0176900	6.12	8.38	7.54	7.389	3.616	4.325	4.7	2.53	6.91	4.311	1.096	2.822	1.0885	7.4676	3.9598	0	12.37	8.288	3.716	2.419	0.862	13.31	12.83	8.667	5.282	5.657	2.193	12	15.3	5.83	1
Os02g0763900	466	399	455	335.8	319.1	289.8	385	410	468	334.5	318	274.7	387.51	374.31	342.92	453.8	345.2	344	314	294.3	266.4	468	406.2	439.1	339.1	302.7	298.2	278	307	317	9
Os02g0208500	335	193	178	101.8	56.95	205.9	168	163	82.9	100	89.9	184.4	124.09	285.64	126.71	71.65	571.9	265.2	182.1	156.4	332.8	380	688.4	1279	147.9	403.5	559.2	433	370	232	9
Os02g0638200	2.04	4.66	2.16	2.463	4.52	0	2.35	0.63	4.84	1.724	2.193	0.941	0	2.8004	1.5839	11.94	1.031	2.072	2.787	0.806	12.07	8.873	4.988	5.056	1.056	0	2.193	2.78	3.83	0	6
Os02g0125566	55.1	57.7	58.2	28.73	23.5	17.3	3.13	7.59	4.84	43.11	31.8	26.34	27.213	34.538	50.686	47.76	35.04	39.37	22.29	16.13	20.69	14.79	11.4	13	59.16	33.94	28.51	49	42.1	57.5	3
Os02g0316800	0	1.86	1.08	0	2.712	0	4.7	0	0	2.587	5.482	0	0	2.8004	0.792	0	1.031	1.036	2.787	4.031	7.76	5.176	0.713	5.778	0	0.943	0	0.93	0	0.83	9
Os02g0733300	0	0	0	0	0	0	0	0	0.69	0	0	0	0	0	0	0	0	0	0	0	0	0	0	0	0	0	0	0	0	0	18
Os02g0691800	176	181	223	230.7	221.5	201.6	258	248	231	241.4	208.3	275.7	207.91	217.49	219.37	250.8	188.6	227.9	217.4	225.8	190.5	209.2	213.1	226.1	299	235.7	269.7	238	231	202	5
Os02g0330200	5.1	2.79	8.62	4.926	2.712	11.25	3.91	0.63	1.38	6.897	4.386	5.645	2.177	0.9335	5.5438	0	4.122	5.18	2.787	7.257	6.036	1.479	3.563	3.611	2.113	1.886	0	1.85	1.91	3.33	16
Os02g0687200	81.6	93.1	61.4	12.31	13.56	6.92	63.4	27.2	25.6	73.29	44.95	33.87	20.682	29.87	21.383	11.94	77.29	53.87	11.15	7.257	6.898	8.133	22.09	32.5	42.25	50.91	70.17	13.9	25.8	16.7	14
Os02g0234700	6.12	1.86	2.16	1.642	3.616	5.19	15.7	3.8	11.1	2.587	4.386	5.645	6.5311	7.4676	1.5839	0	17.52	5.18	19.51	14.51	26.73	4.436	8.552	4.333	8.451	2.829	19.74	4.63	6.69	5	15
Os02g0697700	911	1012	962	525.4	502.6	539.8	1195	935	992	1486	1512	1383	1279	1336.7	1293.3	1051	1140	1042	678.1	606.3	613	806.7	787.5	856.6	1473	1412	1487	1377	1279	1370	3
Os02g0179100	864	784	808	839	899.4	967.1	1217	1111	1260	852.7	854.1	1207	997.08	1000.7	908.38	1887	1008	939.7	1212	1297	1330	888.7	873.7	1072	991.9	1145	1028	857	951	856	6
Os02g0165100	1031	986	875	42.69	50.62	51.04	17.2	32.3	36.6	58.63	21.93	23.52	64.223	58.807	71.277	143.3	201	361.6	40.87	31.44	31.9	113.1	101.2	78.72	51.76	48.08	54.82	62	62.2	70.8	14
Os02g0528900	251	247	241	167.5	180.8	87.37	31.3	52.5	53.9	103.5	85.52	38.57	348.33	160.55	276.4	191.1	84.5	110.9	65.96	41.93	26.73	14.79	42.05	10.11	69.72	27.34	17.54	130	154	155	4
Os02g0203000	1051	633	1001	497.5	471	545.8	345	375	208	891.5	910	1188	1186.5	1240.6	1050.9	2161	1481	1014	921.5	1092	775.1	259.5	383.4	330.1	1315	1373	1353	1081	918	1092	13
Os02g0162766	78.5	86.6	72.2	64.04	49.72	57.09	14.1	12.7	23.5	15.52	15.35	17.88	38.098	32.671	36.43	71.65	43.28	69.41	22.29	25.8	12.07	72.46	57.72	29.61	33.8	24.51	37.28	80.5	63.1	94.1	8
Os02g0814100	509	472	511	350.6	349.8	331.3	249	265	242	394.9	411.1	375.4	558.41	555.4	540.12	453.8	567.8	497.3	366.9	428.1	431.1	343.1	294.3	307.7	480.6	437.5	447.4	623	564	583	3
Os02g0472700	347	412	482	208.5	176.3	270.8	402	354	381	790.6	633.7	582.4	511.6	639.41	585.26	549.3	602.9	505.6	313.1	339.4	350.1	424.4	456.1	403	809.2	706.2	642.5	844	596	606	3
Os02g0206833	7.14	8.38	15.1	7.389	10.85	5.19	6.26	5.06	11.1	10.35	15.35	17.88	14.151	12.135	8.7116	47.76	20.61	18.65	3.716	11.29	7.76	5.176	2.138	2.167	12.68	15.09	4.386	22.2	9.56	17.5	13
Os02g0281150	284	265	249	202	204.3	231.8	189	199	212	222.4	237.9	263.4	251.45	247.36	258.97	382.1	286.5	324.3	222.9	219.3	178.5	266.2	249.4	196.5	256.7	262.1	269.7	291	272	241	13
Os02g0235050	11.2	11.2	16.2	10.67	11.75	13.84	1.57	3.16	1.38	0	6.578	4.704	10.885	3.7338	11.879	23.88	12.37	10.36	1.858	8.063	6.898	7.394	15.68	5.056	3.169	5.657	0	17.6	19.1	20	8
Os02g0474000	980	1501	1120	2052	1871	1800	2515	2423	2685	1083	1349	966.2	876.26	841.04	1018.5	1433	957.4	1050	1340	1695	1436	3321	3211	2757	997.2	1113	1081	1035	1210	1121	17
Os02g0833000	0	0	0	0.821	0	0	0	0	0	0	1.096	0	0	0	0	0	0	2.072	0	0.806	0	1.479	2.138	1.444	1.056	0	0	2.78	0.96	0	1
Os02g0606800	364	371	388	449.9	440.2	425.6	495	497	497	404.4	369.5	331.2	352.68	324.84	391.23	537.3	306.1	307.7	458.9	437.8	428.5	723.9	652.1	675.3	331.7	353.6	311.4	439	396	408	17
Os02g0128600	110	119	116	128.1	137.4	115.9	162	135	135	172.4	180.9	191.9	127.36	132.55	150.47	143.3	117.5	111.9	152.3	152.4	150	117.6	91.93	120.6	164.8	139.5	186.4	135	112	120	20
Os02g0529900	220	255	232	431.8	456.5	426.5	429	445	446	217.3	203.9	239.9	314.58	268.83	258.97	322.4	292.7	305.6	440.3	493.4	507	414.1	360.6	445.6	251.4	286.6	214.9	257	300	283	12
Os02g0729500	18.4	22.4	21.6	39.41	27.12	16.44	34.4	36.1	37.3	25.87	47.14	31.99	17.416	15.869	19.007	0	23.7	22.79	20.44	24.19	19.83	28.84	28.51	30.33	20.07	21.69	6.579	25	11.5	25	18
Os02g0469900	268	343	260	177.3	162.7	204.2	658	712	538	325.9	393.6	428.1	384.25	398.58	359.55	310.5	425.6	367.8	296.3	220.9	225.9	466.6	419.7	515	396.1	521.4	550.4	383	416	448	5
Os02g0586400	830	871	865	856.3	847	878.9	1066	1103	1250	786.3	678.7	632.2	746.72	782.23	874.33	907.5	783.2	771.8	762.7	763.5	728.6	916.1	1021	1077	762.7	685.4	881.6	742	779	708	18
Os02g0595400	388	374	349	419.5	432.1	383.2	975	758	813	534.6	510.9	638.8	458.27	393.92	357.97	525.4	333.9	399.9	557.4	560.4	608.7	411.8	439	558.3	345.4	474.2	429.8	269	299	200	15
Os02g0297132	9.18	9.31	4.31	10.67	16.27	5.19	11	29.7	10.4	13.79	25.22	11.29	6.5311	6.5342	14.255	0	11.34	8.288	12.08	7.257	8.622	17.75	27.08	21.67	20.07	8.486	26.32	13.9	17.2	30	17
Os02g0608400	1134	1201	1112	1473	1585	1389	1155	1275	1088	1300	1191	1244	1203.9	1251.8	1215.7	1552	1273	1226	1362	1311	1342	1150	1305	1106	1213	1245	1186	1236	1223	1179	10
Os02g0477100	745	795	867	660.1	771.1	641.9	516	538	511	537.1	732.4	508	632.43	638.48	698.51	919.5	793.5	908.6	532.3	699.9	611.3	830.3	621.4	531.6	792.3	665.6	574.5	947	966	1063	8
Os02g0116000	1167	1007	1151	947.4	1044	1004	795	727	725	1209	1073	1449	1258.3	1323.6	1047	1373	1002	1235	995.8	1142	1167	961.2	820.2	794.5	1129	1291	1278	1190	1222	1058	3
Os02g0270900	1.02	0.93	0	0	0	0	0.78	0	2.07	1.724	2.193	9.408	1.0885	0.9335	0	0	0	1.036	0	0	0	0	0	0	0	3.771	0	0	1.91	0	20
Os02g0108302	28.5	39.1	35.6	56.65	39.77	44.98	25.1	35.4	23.5	15.52	29.6	10.35	19.593	19.602	18.215	11.94	38.13	20.72	13.01	17.74	12.07	53.98	47.75	35.39	27.47	16.03	21.93	15.7	27.7	44.2	2
Os02g0608300	653	651	631	450.7	459.2	525.9	532	588	563	623.4	635.9	607.8	748.9	731.83	668.42	1063	763.6	701.4	568.5	592.6	590.6	656.6	663.5	607.4	699.3	801.4	754.4	757	743	717	13
Os02g0125400	19.4	19.6	1.08	0	0.904	2.595	3.91	0	0	1.724	1.096	0.941	2.177	0	0	0	0	3.108	0.929	0.806	2.587	0	0.713	1.444	0	0	0	0	1.91	1.67	14
Os02g0800800	0	0	0	0	0	0	0	0	0	0	0	5.645	1.0885	0	0.792	0	0	0	0	0	0	0	0	0	0	0	0	1.85	0	0	20
Os02g0714200	89.7	89.4	67.9	128.1	150.1	199.8	75.2	62	91.9	81.05	80.04	113.8	65.311	126.02	79.989	83.59	110.3	84.96	211.8	170.9	281.1	65.07	46.32	81.61	78.17	129.2	153.5	60.1	108	60	16
Os02g0616800	91.8	71.7	88.4	107.5	138.3	126.3	50.1	70.8	75.3	81.05	76.75	47.04	117.56	96.146	127.51	131.4	69.05	82.88	81.75	67.73	84.5	100.6	99.06	82.34	67.61	91.46	43.86	91.6	90.9	95.8	8
Os02g0314925	0	0	0	0	0	0	0.78	0	0	0	0	0	0	0	1.5839	0	3.092	0	2.787	0	0	0	0	0	0	0	0	0	0	0	16
Os02g0198300	54	67.1	49.6	47.62	47.01	52.77	31.3	39.2	38.7	44.83	50.43	30.11	43.541	32.671	48.31	35.82	78.32	39.37	36.23	34.67	18.97	55.45	54.87	41.17	35.92	38.66	32.89	64.8	79.4	52.5	8
Os02g0798400	0	0.93	0	2.463	0	0	0	0.63	0	0	1.096	0	0	0	0.792	0	1.031	0	0.929	0	0	1.479	1.425	0	0	0	0	0	1.91	0	17

Os02g0779500	92.8	101	97	106.7	95.82	123.7	90.8	77.8	122	113.8	111.8	156.2	83.816	110.15	93.452	71.65	107.2	120.2	124.5	120.9	144	116.8	113.3	106.2	137.3	120.7	125	89.7	85.1	99.1	20
Os02g0603000	7.14	11.2	16.2	9.031	12.66	8.65	8.61	18.3	3.46	19.83	33.99	29.17	11.974	14.002	15.047	23.88	19.58	17.61	17.65	21.77	26.73	11.09	7.839	12.28	31.69	28.29	32.89	25.9	10.5	17.5	20
Os02g0602400	771	861	849	1170	1106	1158	1812	1982	1899	954.4	1059	1005	1005.8	959.59	1117.5	1015	856.4	936.6	1104	1204	1335	1655	1396	1701	1032	1097	824.5	876	931	990	18
Os02g0814200	0	0	1.08	0	0	0	0	0	1.38	0	0	0	0	0	0	0	0	0	0	0	0	0.739	0	1.444	0	0	0	0	0	0	9
Os02g0809700	0	0	0	0	0	0	0	0.63	0	0	0	0.941	0	0.9335	0	0	1.031	1.036	0	1.613	0	0	0	0	2.113	1.886	0	0	0.96	0	1
Os02g0551100	510	509	453	1138	920.2	1265	551	528	692	470.8	376.1	381	379.89	443.39	459.34	513.5	532.8	510.8	1179	1159	1148	940.5	839.5	629.1	496.5	411.1	506.6	525	503	488	10
Os02g0537200	101	115	106	184.7	226	167	276	269	276	100	114	93.14	109.94	74.676	102.16	191.1	78.32	89.1	145.8	129	135.4	174.5	156.1	178.4	82.4	108.4	89.91	82.3	103	91.6	18
Os02g0768100	6.12	13	11.9	12.31	11.75	15.57	10.2	12	15.9	18.11	12.06	14.11	16.328	17.736	13.463	11.94	7.214	11.4	23.22	23.38	21.56	11.83	4.988	16.61	17.96	18.86	15.35	13	14.3	12.5	16
Os02g0216200	0	0	0	0	0	0	0	0	0	0	1.096	0	0	0	0	0	0	1.036	0	0	0	0	0	0	0	0	0	0	0	0	7
Os02g0168800	453	560	502	981.9	1088	829.6	646	762	750	521.6	578.9	551.3	492.01	485.39	423.7	406	393.7	475.5	768.2	882.1	843.2	642.5	614.3	702	447.9	415.8	381.6	377	386	384	10
Os02g0479100	0	0	0	0	0	0	0	0.63	0	0	0	0	0	0	0	0	0	0	0	0	0	1.479	0	0	0	0	0	0	0	0	9
Os02g0656200	242	255	248	243.8	221.5	257.8	187	245	247	219	243.4	181.6	242.74	271.63	284.32	274.6	240.1	250.7	189.5	211.2	186.2	336.4	352	309.1	236.6	183.9	199.6	312	289	284	17
Os02g0296800	274	258	221	345.6	408.6	287.2	391	417	368	242.3	271.9	261.5	268.86	270.7	216.21	226.9	220.5	210.3	374.4	374.9	405.2	328.3	320	365.5	233.5	220.6	203.9	212	236	200	12
Os02g0481700	273	329	272	378.5	343.5	350.3	250	322	329	219	233.5	172.2	327.64	266.03	323.91	406	237	256.9	255.5	324.9	269.9	303.9	302.9	269.4	260.9	267.8	199.6	256	261	261	6
Os02g0175800	1298	1375	1367	1241	1312	1267	994	1163	1203	1108	1205	1231	1254	1221	1157.1	1505	1351	1275	1186	1009	1164	1184	1143	1259	1068	1096	1129	1051	1135	1314	14
Os02g0285900	146	140	193	105.9	126.6	113.3	80.6	134	97.4	174.2	198.4	220.2	389.69	339.78	348.47	238.8	187.6	192.7	90.11	175	56.91	86.51	80.53	66.45	226.1	213.1	94.3	245	252	247	4
Os02g0173800	836	752	901	802.1	823.5	706.7	685	679	697	665.6	586.6	433.7	522.49	603.01	676.34	955.3	537.9	618.5	832.3	818.4	691.5	789.7	826.7	768.5	503.9	434.7	583.3	590	593	544	6
Os02g0668000	25.5	7.45	34.5	3.284	0.904	1.73	0	5.69	1.38	13.79	15.35	15.99	15.239	10.268	16.631	11.94	20.61	23.83	1.858	9.675	0.862	2.958	0.713	0	13.73	13.2	15.35	32.4	17.2	12.5	3
Os02g0752300	1.02	1.86	0	0.821	1.808	3.46	3.91	1.9	6.91	1.724	1.096	0	3.2656	0.9335	0	0	3.092	2.072	1.858	0.806	1.724	0	1.425	4.333	1.056	3.771	2.193	2.78	1.91	0.83	18
Os02g0805200	177	147	194	256.1	264	245.7	344	282	303	161.2	165.6	162.8	211.17	179.22	166.31	143.3	168	179.2	254.5	203.2	248.3	256.6	218.8	255	170.1	191.4	162.3	117	150	124	12
Os02g0731400	0	0	1.08	4.105	0.904	3.46	4.7	2.53	4.15	0	1.096	2.822	0	0	0	11.94	3.092	1.036	0	0.806	0.862	3.697	2.138	0.722	1.056	0	8.772	0	0	0	6
Os02g0761200	28.5	30.7	22.6	48.44	39.77	62.28	27.4	39.8	54.6	36.21	21.93	33.87	27.213	36.405	7.9197	11.94	21.64	14.5	36.23	21.77	28.45	60.63	46.32	36.83	27.47	28.29	26.32	28.7	18.2	16.7	12
Os02g0117700	11.2	4.66	6.47	11.49	6.328	4.325	16.4	5.06	8.98	12.07	1.096	2.822	7.6196	5.6007	3.1679	23.88	6.183	1.036	9.29	8.063	10.35	7.394	7.126	5.778	2.113	2.829	4.386	2.78	9.56	9.16	6
Os02g0214633	2.04	0	0	0	0.904	0	0	0	0	1.724	0	0	0	0	0	0	0	1.036	0	0	0	0	0	0	0	0	0	1.85	0	0	19
Os02g0791400	0	0	0	0	0	0	0	0	0	0	0	0	0	0	0	0	0	0	0	0	0	1.479	0.713	1.444	0	0	0	0	0	0	9
Os02g0795000	376	442	402	406.4	441.1	384.1	232	299	248	339.7	330	388.6	299.34	303.37	311.24	370.2	359.7	337.7	338.1	419.3	393.2	283.2	278.6	244.1	308.5	282.9	254.4	364	348	372	16
Os02g0649000	107	88.5	109	80.46	101.2	88.23	231	227	166	119	93.19	98.79	91.436	121.35	125.92	286.6	121.6	124.3	99.4	153.2	119	142.7	141.1	158.2	155.3	140.5	166.7	157	155	106	6
Os02g0489525	169	187	205	107.5	87.69	115.1	163	112	128	247.4	174.3	261.5	225.32	182.02	186.11	71.65	206.1	218.6	190.4	158	164.7	152.3	127.6	125.7	221.8	261.2	263.2	212	164	159	20
Os02g0533200	1232	971	1120	1871	2093	1934	214	255	167	1137	949.5	1282	1560.9	1701.7	1462	1588	1818	1525	2288	2135	2909	133.8	177.4	148.8	1223	1317	1252	1304	1208	1231	16
Os02g0179800	499	675	608	756.1	758.4	676.5	919	1094	918	553.5	603	470.4	705.36	619.81	650.2	585.1	525.6	582.3	657.7	705.5	676.8	771.2	865.1	790.9	635.9	539.3	451.7	702	623	715	18
Os02g0668400	108	111	110	246.3	257.6	215.4	131	142	119	113.8	99.77	120.4	104.5	82.144	110.88	119.4	80.38	108.8	223.9	234.6	270.7	161.2	131.8	156	121.5	108.4	129.4	101	103	85	10
Os02g0725600	124	117	74.4	227.4	235	269	207	154	212	129.3	121.7	154.3	133.89	133.48	83.948	203	65.95	116	278.7	288.6	303.5	192.2	215.9	178.4	94.02	118.8	171	95.3	104	79.1	10
Os02g0823400	207	195	224	167.5	222.4	192	748	699	737	331.9	377.2	366	279.75	228.7	273.23	274.6	254.5	204.1	175.6	136.3	146.6	369.7	392.7	528.7	254.6	284.7	289.5	172	160	205	18
Os02g0688800	36.7	37.3	47.4	1.642	0	0.865	57.9	20.2	36.6	68.11	59.21	47.04	42.452	47.606	36.43	47.76	53.59	38.33	3.716	5.644	1.724	14.79	22.8	23.11	31.69	49.97	61.4	31.5	43	34.2	3
Os02g0272600	351	339	365	388.3	377.9	373.7	577	549	500	428.5	453.9	467.6	396.22	436.86	419.74	525.4	343.2	381.3	449.6	408	500.1	477.6	459.6	436.2	455.3	412	486.8	377	347	369	18
Os02g0217800	2.04	0.93	1.08	4.105	4.52	1.73	3.91	5.69	4.84	5.173	3.289	7.527	2.177	3.7338	3.9598	0	6.183	2.072	8.361	9.675	6.898	5.176	6.414	3.611	3.169	3.771	4.386	4.63	2.87	5	15
Os02g0531900	1.02	0	0	0	0	0	0	0	0	1.724	0	0	0	0	0	0	0	0	0	0	0	0	0	0	0	0	0	0	0	0	7
Os02g0503400	1259	1204	1029	1207	1337	1190	3092	2386	2994	1519	1341	2021	1268.1	1214.4	944.02	907.5	887.3	940.7	1600	1500	1549	1799	1778	2087	1046	1179	1362	748	772	667	18
Os02g0486500	1.02	0	5.39	2.463	0	3.46	3.91	1.9	2.76	0	1.096	2.822	0	0	0	0	1.031	1.036	1.858	0.806	2.587	1.479	0.713	3.611	3.169	7.543	0	0	0.96	0	15
Os02g0826500	1443	1640	1475	1722	1793	1721	2580	2445	2526	1624	1595	1737	1415.1	1579.4	1433.5	1863	1467	1471	1766	1528	1689	1913	1910	2022	1467	1546	1610	1217	1324	1215	18
Os02g0622500	1540	1074	1596	219.2	207	237	162	214	144	1245	1275	1179	1662.2	1300.3	1685.3	1421	989.3	969.7	322.3	320.9	327.6	151.6	146.1	134.3	1337	1054	1228	1118	1135	1136	3
Os02g0184200	151	139	122	115.8	101.2	109.9	199	143	165	212.1	219.3	423.4	176.34	166.15	160.77	71.65	165.9	172	206.2	137.9	224.2	88.73	64.85	118.4	203.9	232.9	221.5	118	131	122	20
Os02g0734101	0	0	0	0	0	0	0	0	0	0	0	0	0	0	0.792	0	0	0	0	0	0	0	0	0	0	0	0	0	0	0	4
Os02g0156700	1.02	0	0	1.642	2.712	3.46	3.91	2.53	0	2.587	0	0.941	1.0885	0.9335	0.792	0	0	2.072	1.858	0	0	0	0.713	0.722	0	1.886	0	0	1.91	0.83	2
Os02g0566500	291	296	248	300.5	284.8	305.4	332	362	361	316.4	333.3	257.8	314.58	277.24	301.74	298.5	360.7	305.6	288.9	294.3	297.5	303.1	344.9	311.3	370.8	334.7	291.7	336	313	362	5
Os02g0309500	1.02	0	0	0	0	0	0	0	0	0	0	0	0	0	0	0	0	0	0	1.613	0	0	0	0.722	0	0	0	0	0	0	19
Os02g0168600	334	392	345	354.7	341.7	381.5	492	429	399	389.7	395.8	301.1	426.7	499.4	455.38	394.1	426.6	408.2	466.3	463.6	479.4	349.7	441.8	444.2	391.9	394.1	416.7	430	405	401	4
Os02g0121600	127	155	147	230.7	286.6	218	436	427	417	227.6	227	219.2	194.85	153.09	224.92	119.4	89.66	135.7	262	214.5	197.4	295	255.8	260	181.7	187.6	140.3	148	137	142	18
Os02g0825650	1.02	0	0	0	0	0	0	0	0	0	1.096	0	0	0	0.792	0	0	0	0	0	0	1.479	0.713	0	0	0	0	0	0.96	0	17
Os02g0497500	216	197	219	570.6	698.8	609.9	301	329	243	156.9	132.7	151.5	106.67	120.42	117.21	286.6	177.3	180.3	320.5	424.1	336.3	305.4	364.9	258.6	113	132	142.5	160	178	179	10
Os02g0282301	57.1	31.7	43.1	7.389	9.944	19.03	5.48	15.8	14.5	48.28	37.28	78.09	21.77	15.869	19.007	0	49.47	32.12	26.94	16.13	24.14	12.57	13.54	20.95	29.58	47.14	76.75	25	30.6	21.7	20
Os02g0796700	522	582	567	704.4	631.9	623.7	1004	1030	908	585.4	632.6	560.7	696.65	618.88	685.05	668.7	571.9	605	607.5	624.9	590.6	684.7	778.2	768.5	650.7	562.9	539.5	657	611	632	18
Os02g0818700	44.9	40.1	35.6	34.48	46.1	26.82	32.9	33.5	31.8	18.11	32.89	19.76	32.656	19.602	30.095	59.7	37.1	26.94	16.72	36.28	23.28	44.36	29.22	23.11	36.97	24.51	17.54	37	53.6	39.2	8
Os02g0125000	489	478	492	726.6	891.3	666.1	952	934	1041	587.1	767.5	825.1	720.6	637.55	629.61	859.7	526.6	562.6	955.9	1050	867.4	717.9	600	895.6	641.2	656.2	552.6	482	557	517	12
Os02g0167850	0	0	0	0	0	0	0	0	0	0	0	0	0	0	0	0	0	0	0	0	0	0	0	0	0	0	0	0	0	0.83	1
Os02g0787300	203	136	192	396.5	279.3	359	424	548	566	166.4	119.5	145.8	146.95	110.15	112.46	131.4	147.4	163.7	368.8	277.4	424.2	495.4	441.1	440.6	97.19	165	228.1	125	133	124	12
Os02g0521700	116	121	134	264.4	264	216.3	442	455	448	159.5	143.6	128	134.98	109.21	110.08	95.53	96.87	76.67	286.1	298.3	216.4	269.9	257.3	338	128.9	77.31	118.4	95.3	127	120	18
Os02g0631100	18.4	8.38	19.4	13.14	9.04	6.055	5.48	13.3	6.22	19.83	12.06	6.586	4.3541	8.4011	4.7518	71.65	7.214	5.18	14.86	9.675	0.862	16.27	23.52	12.28	6.338	1.886	2.193	8.33	6.69	0.83	6
Os02g0293900	0	0	0	0	0	0	0	0	0	0	0	0	0	0	0	0	0	0	0	0	0.862	0	0	0	0	0	0	0	0	0	16
Os02g0822200	429	498	482	781.6	841.6	734.4	978	1047	977	501.8	536.1	620	569.3	477.93	477.56	680.6	455.5	490	699.5	683.7	655.3	783	664.9	768.5	557.8	515.7	506.6	433	449	480	12
Os02g0491600	0	3.73	1.08	0	0	0	0	0	0	0	0	0	2.177	0	0	0	2.061	0	0	0	0	0.739	1.425	0	0	0	0	0	0	0	14
Os02g0584300	0	0.93	0	0	0	0.865	0	0	0	0	0	0	0	0	1.5839	0	0	0	0	0	0	0	0	0	0	0	0	0	0	0	4
Os02g0207300	0	0	0	0	1.808	0.865	0	0	0.69	0	1.096	0	0	0	0	0	1.031	0	1.858	0.806	0	0	0	0	0	0	0	0	0	0	10
Os02g0117550	0	0.93	0	0	0	0	0.78	0	0	0	0	0	0	0	0	0	0	0	0	0	0	0	0	0	0	0	0	0	0	0	14
Os02g0741500	2245	2479	2290	1963	1942	1915	1495	1834	1524	1775	2010	1672	2411.1	2156.3	2467.8	2866	1992	2174	1396	1546	1426	1647	1728	1473	2030	1962	1531	2436	2205	2454	13
Os02g0833600	399	397	419	325.1	346.2	316.6	560	504	522	444	544.9	532.5	502.9	532.07	524.28	453.8	395.7	332.6	460.8	445.1	342.3	360.1	409.8	411.7	453.2	435.6	451.7	349	373	382	5
Os02g0100050	0	0	1.08	0	0	0	0	0	0	0	0	0	0	0	0	0	0	0	0	0	0	0	0	0	0	0	0	0	0	0	14
Os02g0754300	780	1050	820	3226	3477	2728	5364	4696	4895	877.7	1282	1781	830.54	510.6	434	477.6	447.3	583.3	2980	3303	2926	2924	2681	4253	590.5	581.7	456.1	248	237	262	12
Os02g0640500	0	0	0	0	0	0	0	0	0	0	0	0	0	0	0	0	0	0	0	0.806	0	0	0	0	0	0	0	0	0	0	16
Os02g0155900	161	115	144	77.99	33.45	89.1	43.1	46.8	24.2	133.6	106.4	307.6	169.81	245.5	212.25	95.53	197.9	183.4	115.2	108	202.6	46.58	34.92	53.45	216.6	255.5	214.9	189	150	124	3
Os02g0167300	38.7	15.8	24.8	19.7	29.83	43.25	63.4	35.4	44.9	28.45	23.02	53.63	23.947	26.137	15.047	0	19.58	23.83	33.44	18.54	37.08	10.35	15.68	32.5	16.9	15.09	19.74	10.2	14.3	15.8	15
Os02g0125300	545	535	512	132.2	132	145.3	96.3	101	117	203.5	179.8	183.5	216.62	210.96	224.13	179.1	224.7	299.4	239.7	237.9	201.8	92.42	104	100.4	176.4	165	142.5	136	150	109	14
Os02g0606000	615	550	577	691.3	745.8	640.1	651	681	708	614.7	697.3	831.7	659.64	533	518.74	632.9	541	505.6	656.8	742.6	767.4	516.8	463.9	548.2	566.2	538.4	594.3	374	449	450	15
Os02g0739100	1758	1444	1320	417.9	391.4	389.3	326	371	362	263	253.3	101.6	299.34	247.36	266.1	632.9	822.4	877.5	304.7	408	373.3	398.5	371.3	358.2	191.2	194.2	208.3	299	280	325	14
Os02g0265300	8.16	14.9	4.31	0.821	0.904	2.595	0	1.27	0	23.28	9.868	15.05	5.4426	4.6673	11.879	0	9.275	3.108	2.787	2.419	3.449	0.739	0	4.333	5.282	17.91	6.579	11.1	3.83	2.5	20
Os02g0186800	38.7	63.3	44.2	173.2	171.8	139.3	62.6	59.5	59.4	85.36	70.17	88.44	67.488	51.34	49.102	47.76	57.71	47.66	157	156.4	184.5	59.15	74.11	59.22	35.92	44.31	13.16	47.2	45.9	36.7	10
Os02g0217250	0	0	0	0	0	0	0	0.63	0	0	0	0.941	0	0	0	0	0	0	0	0	0	0	0	0	0	0	0	0	0	0	20
Os02g0518800	251	203	255	170.8	166.3	136.7	114	152	113	134.5	159	114.8	127.36	116.68	141.76	167.2	209.2	233.1	128.2	153.2	109.5	209.2	169.6	150.2	148.9	139.5	140.3	222	222	198	8
Os02g0564400	2950	3623	3477	4084	4231	3934	1953	1982	1680	3960	4605	3807	4357.3	4168.8	4122.2	4263	4240	3885	4728	4876	4926	1390	1546	1753	4781	4362	4048	4082	3935	4780	16
Os02g0484900	0	2.79	0	0.821	0.904	2.595	0.78	1.27	2.07	1.724	5.482	0	2.177	0	0.792	0	2.061	1.036	0.929	2.419	3.449	2.958	0.713	1.444	3.169	0	0	0.93	0.96	0.83	7
Os02g0225000	2033	2256	2364	2814	3167	2842	3659	4235	3786	2111	2376	2136	2572.2	2469	2539.8	2006	2028	2128	2641	2355	2783	2933	2772	3136	2094	2229	1952	1773	2029	2082	18
Os02g0730500	334	253	325	329.2	395	422.1	366	359	435	313.8	277.4	375.4	390.78	407.92	328.67	394.1	287.5	315	513.7	463.6	509.6	304.6	297.9	304.1	376.1	348.9	322.4	249	321	222	16
Os02g0528550	0	0.93	0	0	0.904	1.73	0	1.27	0.69	3.449	1.096	0	0	0.9335	3.9598	11.94	1.031	1.036	1.858	2.419	0	0	0.713	0	0	0	0	0	0.96	4.17	6
Os02g0533000	3261	3082	3527	2428	2386	2369	1447	2039	1678	2906	3464	3162	4683.9	4020.4	4529.3	5827	4526	3946	2735	3078	2839	1454	1621	1657	4099	4047	3272	4342	3974	4484	13
Os02g0172850	0	0	0	1.642	0	0	0.78	0	0	0	0	0	0	0	0	0	0	0	0	2.419	6.036	1.479	0	0	0	0	0	0	0	0	16
Os02g0799000	678	360	609	989.3	887.7	1433	73.6	128	104	383.7	348.7	300.1	542.08	570.34	533.79	573.2	448.3	472.4	1553	1821	1840	181.1	218.1	156.7	378.2	348.9	298.2	469	497	466	16
Os02g0188400	3.06	9.31	11.9	6.568	5.424	4.325	11.7	5.06	9.67	3.449	0	2.822	2.177	3.7338	1.5839	0	6.183	6.216	2.787	3.225	3.449	13.31	19.24	57.78	3.169	1.886	0	4.63	8.61	5	9
Os02g0269000	202	183	198	173.2	182.6	199	240	268	229	181.9	146.9	158.1	168.72	182.02	183.74	119.4	236	223.8	198.8	179	188.8	216.6	176.7	211.6	182.8	167.8	258.8	185	193	205	18
Os02g0734300	614	629	603	266	258.5	172.1	544	656	457	662.2	585.5	588	632.43	505.93	602.69	513.5	700.8	553.2	205.3	214.5	372.5	408.9	486.7	719.4	544	432.8	427.6	439	372	522	5
Os02g0768200	390	471	378	544.3	537	546.7	813	706	697	444.9	480.2	406.4	369.01	443.39	472.01	752.3	386.4	391.6	566.7	513.6	464.7	686.9	720.5	668.8	476.4	485.6	576.7	488	497	432	18
Os02g0820700	952	859	933	721.6	744	734.4	777	790	722	1036	926.5	1072	1058	1030.5	1149.1	704.5	825.5	833	851.9	778.1	792.4	844.4	757.5	759.1	997.2	1018	1068	1044	947	936	3
Os02g0697200	153	165	151	304.6	285.7	327	300	302	300	161.2	151.3	126.1	173.07	200.69	175.02	286.6	157.7	187.5	269.4	254	341.4	253.6	281.5	240.5	135.2	138.6	142.5	153	160	155	12
Os02g0608700	886	777	953	674	701.5	717.1	673	612	734	1007	1010	973.7	965.52	983.86	1023.2	752.3	764.7	839.2	904.8	786.9	741.5	567.1	614.3	642.1	885.2	888.2	1035	814	817	741	3
Os02g0118950	0	0	0	0	0	0	0	0	0	0	0	0	0	0	0	0	0	0	0	0	0	0	0	0	0	0	0	0	0.96	0	1
Os02g0672100	809	938	947	1116	990.8	1074	1761	1781	2098	1012	1303	828.9	1145.1	1003.5	1237.8	1612	960.5	1087	1232	1224	1055	1812	1575	1639	1013	1041	1075	913	1133	1201	18
Os02g0132200	75.4	102	95.9	110.8	112.1	121.1	180	204	221	71.56	108.5	97.85	142.6	131.62	140.18	143.3	122.6	122.3	86.39	81.43	105.2	164.9	157.5	145.9	107.7	108.4	118.4	80.5	121	93.3	18
Os02g0226300	390	353	398	388.3	328.1	393.6	316	329	374	275	296	212.6	363.57	299.64	374.6	298.5	343.2	326.4	280.5	321.7	300.9	462.9	438.3	416	273.6	267.8	258.8	352	342	365	17
Os02g0496100	241	325	310	481.9	468.3	441.2	500	492	509	408.7	394.7	350	370.1	351.91	394.4	394.1	324.6	263.2	476.6	516.8	475.1	408.9	459.6	442.7	380.3	300.8	348.7	337	307	326	12
Os02g0119000	0	0	0	0.821	0	0	0	0	0	0.862	0	0	0	0	0	0	0	0	0	0	0.862	0	0	0	0	0	0	0	0	0	7
Os02g0802700	1277	964	1262	424.4	441.1	359.9	478	525	245	450.9	414.4	341.5	593.24	556.34	567.84	1254	1008	898.2	577.8	553.9	552.7	324.6	394.1	339.5	485.9	482.7	502.2	895	786	666	13
Os02g0435200	195	252	200	286.5	326.3	292.4	420	381	373	235.4	228.1	224.9	236.21	197.89	259.76	334.3	244.2	241.4	311.2	333.8	326.8	357.9	387.7	378.5	240.9	200.8	217.1	226	200	247	12
Os02g0221600	368	332	361	316.9	331.8	311.4	335	385	352	350	320.1	219.2	266.69	307.11	320.75	417.9	308.1	304.6	217.4	271.7	211.2	502.8	531.6	434.1	327.5	320.6	328.9	261	325	300	17
Os02g0119200	8.16	2.79	8.62	10.67	9.04	3.46	3.91	15.8	6.91	5.173	13.16	5.645	3.2656	3.7338	11.879	23.88	13.4	12.43	6.503	4.031	0.862	5.915	9.264	9.389	10.56	8.486	17.54	8.33	9.56	10.8	13
Os02g0719100	643	572	653	459.7	418.5	479.2	301	301	287	469.9	540.5	507.1	624.81	559.14	685.05	931.4	742	598.8	427.3	483.8	469.9	299.5	320	286.7	562	540.2	552.6	689	637	627	13
Os02g0110600	62.2	44.7	22.6	60.75	26.22	113.3	38.4	51.2	31.1	57.77	59.21	72.44	26.124	81.21	44.35	11.94	98.93	42.48	44.59	41.12	121.6	76.9	114.7	163.2	77.11	190.5	361.8	90.7	175	70.8	11
Os02g0667500	27.5	18.6	21.6	14.78	28.02	19.03	25.8	29.1	15.9	50.01	58.11	30.11	63.134	51.34	86.324	47.76	62.86	56.98	10.22	16.93	22.42	20.7	24.23	21.67	67.61	61.28	52.63	68.5	39.2	50	3
Os02g0798800	65.2	80.1	66.8	88.67	82.26	72.66	97.9	48.1	56.7	75.87	87.71	81.85	101.23	54.14	58.605	47.76	72.14	104.6	53.88	104	90.53	150.8	89.79	109.1	72.89	80.14	87.72	62	82.2	65.8	9
Os02g0597800	1116	1223	1209	1078	1168	1035	1318	1498	1285	1210	1298	1030	1183.2	1196.7	1286.2	1516	1348	1248	1077	1117	1171	1254	1152	1189	1319	1232	1230	1311	1359	1459	13
Os02g0496500	121	199	166	1054	1248	976.6	315	244	206	145.7	125	103.5	151.3	149.35	116.42	226.9	114.4	125.4	784	922.4	854.5	256.6	272.9	208.7	96.13	82.03	96.49	99	140	109	10
Os02g0137000	346	386	356	415.4	353.5	430.8	362	340	404	406.1	292.7	315.2	363.57	323.91	365.89	346.3	315.3	352.3	314.9	388.6	351.8	351.2	394.8	374.8	377.1	356.4	291.7	423	375	360	2
Os02g0754100	572	602	587	556.6	522.5	627.2	442	541	549	568.2	557	562.6	722.78	578.74	622.49	859.7	682.2	721.1	546.2	540.2	579.4	590.8	548.7	558.3	643.3	664.7	646.9	686	624	677	13
Os02g0159850	0	0.93	0	1.642	0.904	5.19	0	1.27	1.38	0.862	1.096	0	1.0885	0	1.5839	0	0	0	0	0.806	0	11.83	4.988	3.611	0	1.886	0	0	0	0	9
Os02g0297200	23.4	36.3	10.8	9.852	16.27	0.865	2.35	2.53	9.67	13.79	15.35	2.822	17.416	15.869	16.631	47.76	16.49	54.91	18.58	18.54	7.76	19.96	41.33	23.83	45.42	38.66	39.47	116	84.2	50	1
Os02g0549700	1766	1511	1658	1263	1171	1426	1448	1468	1462	1766	1730	1626	1771	1833.3	1924.5	1911	1614	1733	1502	1504	1633	1236	1189	1173	1616	1654	1686	1631	1879	1876	3
Os02g0196100	0	0	0	0	0.904	0	0	0	0	0	0	0	0	0	0	0	0	0	0	0	0	0	0	0	0	0	0	0	0	0	2
Os02g0565000	480	438	490	504.1	505.3	484.4	507	503	440	672.5	685.2	779	770.67	629.15	675.55	800	651.3	560.5	680	691.8	762.2	323.1	307.1	375.6	627.5	775	660.1	612	584	588	20
Os02g0730100	4.08	7.45	6.47	2.463	6.328	2.595	10.2	4.43	6.91	8.622	4.386	7.527	3.2656	3.7338	8.7116	0	4.122	12.43	8.361	12.09	10.35	8.133	7.839	7.945	14.79	15.09	6.579	2.78	6.69	9.16	1

Os02g0756100	13.3	14.9	11.9	5.747	8.136	4.325	45.4	44.3	38.7	10.35	8.771	10.35	4.3541	0.9335	3.1679	0	9.275	10.36	1.858	3.225	4.311	13.31	14.25	27.45	2.113	0.943	6.579	4.63	4.78	0	18
Os02g0215100	1.02	7.45	8.62	34.48	37.97	38.06	21.1	25.9	20	1.724	0	0.941	7.6196	0.9335	0.792	0	2.061	5.18	48.31	45.96	43.11	18.48	15.68	23.11	4.225	0	2.193	0.93	3.83	0	10
Os02g0471100	22.4	22.4	16.2	8.21	12.66	10.38	12.5	8.22	11.1	13.79	33.99	23.52	13.062	11.201	16.631	23.88	13.4	10.36	4.645	13.71	6.036	22.18	12.83	23.83	25.35	30.17	26.32	20.4	19.1	25.8	3
Os02g0730700	456	440	440	505.7	558.7	481.8	1348	1051	1073	857.9	798.2	788.4	547.53	503.13	495.77	334.3	347.3	370.9	635.4	636.2	469	681	776.8	780.7	549.3	495	570.2	401	324	312	18
Os02g0815000	419	372	365	408	423.1	489.6	698	509	574	482	422.1	660.5	401.66	487.26	334.21	203	342.1	419.6	566.7	564.4	509.6	466.6	473.2	495.5	415.2	540.2	550.4	340	367	312	15
Os02g0528100	0	0	0	0	0	0	0	0	0	0	0	0	1.0885	0	0	0	0	0	0	0	0	0	0	0	0	0	0	0	0	1.67	4
Os02g0752701	3014	2666	3032	1442	1319	1076	712	770	811	2110	2372	1334	1761.2	1463.7	2179.5	1851	1985	2672	650.3	878.8	665.6	1894	2307	1533	2620	2127	1607	2507	2814	2885	8
Os02g0813800	349	378	362	172.4	150.1	158.3	106	144	126	234.5	290.5	280.4	296.08	281.9	304.11	358.2	272.1	300.5	168.1	170.1	135.4	98.34	122.6	140.1	284.2	314	331.1	365	334	366	3
Os02g0631500	452	455	469	487.7	464.6	460.2	329	347	354	488	435.3	383.9	583.45	510.6	563.09	441.8	473	520.1	360.4	445.1	365.6	388.9	382	361.1	526.1	483.7	407.9	542	581	562	4
Os02g0552400	2.04	0	1.08	0.821	0.904	0	0	0	0.69	0	1.096	0	0	0.9335	2.3759	11.94	1.031	1.036	0.929	0	1.724	0	1.425	1.444	1.056	0	0	0	0	0.83	6
Os02g0178400	908	850	874	922	964.5	1057	1570	1224	1692	1169	967	935.2	845.78	904.51	913.93	1493	781.1	776	1061	1028	1017	1062	1169	1036	874.7	785.4	760.9	656	696	628	18
Os02g0748800	7.14	0.93	4.31	1.642	0	0	7.05	6.33	5.53	0.862	6.578	0.941	4.3541	3.7338	0.792	0	3.092	1.036	0	1.613	4.311	8.873	17.82	13	4.225	1.886	0	2.78	0.96	0.83	17
Os02g0763000	19.4	15.8	20.5	25.45	36.16	27.68	32.1	24.7	25.6	37.07	14.25	23.52	7.6196	15.869	10.296	11.94	11.34	15.54	32.51	12.09	22.42	20.7	14.97	15.17	7.395	11.31	10.96	0.93	8.61	8.33	12
Os02g0137100	412	443	388	783.2	766.6	656.6	849	951	764	407	413.3	458.2	417.99	398.58	428.45	525.4	438	423.7	605.7	620	609.6	974.5	806.7	822.6	507.1	411.1	375	489	395	502	12
Os02g0581900	24.5	34.5	22.6	36.12	37.97	35.47	67.3	72.1	69.8	71.56	73.46	78.09	78.373	50.406	56.23	35.82	35.04	39.37	40.87	41.93	61.22	44.36	44.9	67.17	60.21	67.88	65.79	42.6	29.6	50.8	5
Os02g0189500	29.6	25.1	34.5	23.81	33.45	42.39	40.7	36.1	34.6	52.59	30.7	36.69	30.479	41.072	42.766	47.76	32.98	39.37	38.09	37.9	32.76	29.58	25.65	25.28	40.14	37.71	41.67	32.4	41.1	31.7	7
Os02g0307800	906	876	922	1544	1645	1458	1924	1861	1931	1157	1483	1677	1512	1114.5	994.71	1087	918.2	927.3	2157	2162	2198	1099	1127	1406	1142	1045	960.5	695	849	756	15
Os02g0522300	300	267	228	229.1	259.4	270.8	420	437	358	231.1	262	390.4	391.87	293.1	248.68	250.8	358.6	305.6	418	323.3	466.5	258	223.8	369.8	294.7	321.5	293.9	200	248	260	15
Os02g0656500	479	619	532	482.7	495.4	505.2	800	761	756	407.8	414.4	341.5	398.4	366.85	475.18	597	473	447.6	369.7	384.6	346.6	797.1	797.4	787.2	465.9	413	339.9	458	451	461	18
Os02g0201800	8.16	10.2	11.9	8.21	8.136	9.516	14.1	10.1	17.3	9.484	5.482	10.35	6.5311	10.268	7.1277	0	20.61	13.47	3.716	6.45	8.622	7.394	13.54	10.83	10.56	21.69	4.386	20.4	10.5	2.5	1
Os02g0812500	4.08	0.93	1.08	3.284	3.616	3.46	0	2.53	0	2.587	0	5.645	1.0885	0.9335	0.792	0	1.031	0	1.858	9.675	2.587	0	1.425	0.722	7.395	2.829	0	1.85	5.74	3.33	16
Os02g0206400	44.9	48.4	51.7	394.1	382.4	244.8	44.6	60.1	57.4	56.04	50.43	21.64	83.816	42.939	95.036	0	43.28	51.8	256.4	294.3	201.8	102	105.5	70.06	45.42	49.03	28.51	98.1	112	107	10
Os02g0293500	120	95	110	278.3	283.8	251.7	172	201	185	101.7	114	77.15	131.71	91.478	118.79	107.5	91.72	94.28	170	186.3	158.6	159	140.4	171.2	99.3	105.6	89.91	108	92.8	92.5	12
Os02g0600650	68.3	44.7	57.1	17.24	12.66	16.44	29.7	27.8	31.1	38.8	43.86	54.57	67.488	56.941	50.686	47.76	72.14	46.62	35.3	33.06	23.28	19.22	24.94	36.11	48.59	34.89	28.51	30.5	41.1	40.8	3
Os02g0126900	217	156	149	183.9	198	216.3	224	252	261	188.8	168.8	233.3	189.4	183.89	182.15	250.8	202	187.5	314	345.9	359.5	142	148.9	192.1	195.4	208.4	230.3	155	172	147	16
Os02g0593400	81.6	79.2	95.9	91.13	65.99	95.16	104	81	104	62.94	71.27	77.15	55.515	64.408	57.814	143.3	81.41	104.6	97.54	92.72	94.84	136	141.1	107.6	82.4	74.48	122.8	121	114	84.1	17
Os02g0121200	421	323	335	297.2	257.6	270.8	229	273	276	282.8	324.5	352.8	390.78	405.12	443.5	441.8	418.4	370.9	343.7	287.8	341.4	240.3	268	221.7	387.7	345.1	355.3	407	403	412	13
Os02g0597200	212	125	167	25.45	25.31	19.03	10.2	12.7	16.6	16.38	27.41	20.7	31.567	26.137	24.551	23.88	52.56	78.74	26.01	20.96	18.11	19.22	25.65	28.17	22.18	13.2	28.51	27.8	26.8	31.7	14
Os02g0472500	16.3	28.9	23.7	18.06	10.85	15.57	3.13	1.9	5.53	19.83	25.22	3.763	15.239	8.4011	20.591	35.82	19.58	29.01	5.574	5.644	4.311	26.62	33.49	10.11	23.24	13.2	24.12	39.8	26.8	50	8
Os02g0708200	783	790	804	740.5	750.3	738.8	778	812	855	696.6	765.3	943.6	689.03	773.83	723.86	728.4	1062	768.7	819.3	715.2	753.6	917.6	897.2	776.4	1047	1168	1138	847	783	751	11
Os02g0813350	619	647	583	1153	1073	1099	489	415	416	666.5	557	804.4	499.63	571.27	464.09	382.1	615.2	574	1052	954.6	1099	337.2	355.6	409.5	613.7	642.1	570.2	508	536	568	10
Os02g0613100	0	0	1.08	4.105	0.904	1.73	0.78	1.27	0	0	1.096	0.941	0	0	1.5839	0	0	0	0	0.806	0.862	0	0.713	0	0	0	0	0	0	0.83	2
Os02g0721700	618	428	626	108.4	105.8	159.2	79.1	87.3	98.1	223.3	169.9	188.2	229.68	294.97	205.91	417.9	495.7	463.1	113.3	130.6	123.3	131.6	145.4	101.1	247.2	285.7	232.4	222	236	193	14
Os02g0685900	0	0.93	0	0	0	0	0	0.63	0.69	0	1.096	0	0	0	0	0	0	0	0	0	0	0	0	0	0	0	0	0	0	0	7
Os02g0316200	155	178	138	140.4	150.1	162.6	431	447	435	241.4	264.2	241.8	206.82	223.09	228.09	107.5	157.7	162.7	155.1	118.5	121.6	315	291.5	311.3	222.9	211.2	269.7	204	164	220	5
Os02g0811101	23.4	20.5	35.6	39.41	45.2	32.01	33.7	39.8	38	27.59	26.31	13.17	30.479	29.87	25.343	23.88	18.55	16.58	34.37	72.57	70.7	29.58	29.22	30.33	12.68	23.57	19.74	11.1	23	21.7	10
Os02g0720600	50	41.9	51.7	45.97	27.12	38.06	60.3	41.7	310	42.25	73.46	13.17	29.39	27.07	24.551	11.94	19.58	17.61	27.87	33.06	21.56	162.7	144	126.4	17.96	16.97	48.24	21.3	23	9.16	18
Os02g0120500	16.3	2.79	1.08	6.568	4.52	11.25	1.57	8.86	3.46	5.173	4.386	10.35	7.6196	13.068	3.1679	0	4.122	5.18	13.01	4.838	12.93	1.479	0.713	4.333	7.395	13.2	24.12	6.48	13.4	10.8	11
Os02g0128200	370	438	371	306.2	319.1	259.5	737	759	752	583.7	645.8	878.7	407.11	300.57	373.02	346.3	415.3	413.4	249	241.1	199.2	564.9	533.8	609.6	560.9	596.8	543.8	256	261	334	5
Os02g0814800	165	178	218	208.5	189.8	257.8	452	400	369	293.1	337.7	350.9	388.6	326.71	262.93	298.5	215.4	201	286.1	285.4	311.3	208.5	204.5	293.2	276.8	260.2	225.9	229	258	225	15
Os02g0742900	0	0	0	0	0.904	0	0	0	0	0	2.193	0	0	0	0	0	0	0	1.858	0	0	0	0.713	0.722	0	0	0	0.93	0.96	1.67	1
Os02g0694600	23.4	35.4	57.1	47.62	56.95	34.6	56.4	38.6	48.4	38.8	44.95	45.16	38.098	37.338	36.43	131.4	61.83	69.41	63.17	80.63	43.97	87.25	65.56	68.61	52.82	61.28	52.63	89.7	71.7	83.3	6
Os02g0749500	420	431	421	413	536.1	494.8	493	547	520	324.2	330	258.7	334.18	368.71	395.19	644.8	404	411.3	411.5	395.1	413	547.1	588.6	469.5	343.3	335.7	353.1	417	360	407	6
Os02g0680500	66.3	74.5	74.4	261.1	287.5	261.2	211	210	209	63.8	85.52	50.8	84.905	74.676	57.814	59.7	58.74	61.13	222	211.2	207.8	130.1	137.5	151.7	64.44	58.46	67.98	58.3	44	48.3	12
Os02g0638300	2326	2167	2748	1757	1511	1843	3255	3130	4072	4258	4551	3936	4224.5	3690.9	3652.5	5457	3485	3379	2380	2624	2814	2508	2600	2931	4648	4165	3485	3232	3152	3373	13
Os02g0163400	21.4	15.8	10.8	11.49	17.18	34.6	37.6	26.6	25.6	13.79	16.45	8.467	1.0885	7.4676	2.3759	0	4.122	10.36	31.58	9.675	26.73	13.31	12.83	34.67	4.225	10.37	4.386	2.78	12.4	0	12
Os02g0573500	7.14	14.9	6.47	18.88	10.85	6.055	43.1	6.33	15.2	12.93	6.578	8.467	21.77	10.268	20.591	11.94	8.244	12.43	10.22	14.51	9.484	51.02	42.05	62.84	10.56	15.09	10.96	25	12.4	13.3	9
Os02g0462401	124	110	109	102.6	153.7	117.6	93.9	128	104	61.22	83.33	70.56	127.36	97.079	104.54	298.5	109.2	144	82.68	93.53	83.64	105.7	107.6	95.34	75	65.06	54.82	105	143	171	6
Os02g0249300	14.3	9.31	5.39	151.1	147.3	172.1	21.9	22.1	35.9	18.97	21.93	27.28	18.505	23.336	25.343	23.88	19.58	19.68	171.9	131.4	172.4	22.92	26.37	25.28	31.69	18.86	28.51	18.5	10.5	19.2	10
Os02g0608500	640	723	657	883.4	932.9	989.6	556	619	703	919.1	862.9	923.9	849.05	718.76	844.24	1039	690.5	575	832.3	811.1	752.7	745.3	649.2	699.9	901.1	849.5	695.2	669	634	764	6
Os02g0726833	0	5.59	4.31	1.642	2.712	2.595	1.57	0	0	3.449	4.386	1.882	7.6196	0.9335	1.5839	0	1.031	4.144	0	0	3.449	9.612	4.988	2.167	0	1.886	4.386	1.85	5.74	1.67	9
Os02g0214900	4.08	4.66	3.23	0.821	0.904	0.865	0	2.53	0	7.76	3.289	3.763	5.4426	8.4011	11.879	0	4.122	2.072	6.503	2.419	6.898	1.479	4.276	2.167	5.282	6.6	8.772	5.55	9.56	0.83	4
Os02g0478050	291	258	270	182.3	220.6	168.7	117	154	143	207.8	190.8	250.3	222.06	236.16	220.96	358.2	307.1	333.6	204.4	229.8	221.6	232.2	203.8	182.7	264.1	325.3	274.1	328	352	357	13
Os02g0245100	172	142	184	213.5	215.1	204.2	266	221	276	242.3	217.1	249.3	232.94	208.16	165.52	322.4	172.1	179.2	243.4	254.8	232.8	150.8	168.9	199.3	193.3	185.7	171	170	160	141	6
Os02g0834800	29.6	49.4	40.9	41.87	53.33	44.98	64.2	57.6	85	66.39	37.28	43.28	62.046	52.273	34.055	23.88	63.89	51.8	72.46	81.43	78.46	66.54	64.85	85.22	55.99	49.97	54.82	39.8	41.1	40.8	15
Os02g0685750	0	0	0	0.821	2.712	0.865	0	0	0	0	0	0	0	0	0	0	0	0	0	0.806	0	0	0	0	1.056	0	0	0	0	0	2
Os02g0678300	4.08	15.8	0	2.463	0.904	0.865	0	0.63	0	0	0	0	0	0.9335	0.792	0	3.092	6.216	0.929	1.613	3.449	2.218	0.713	0	0	0	0	0	1.91	0.83	14
Os02g0770800	11.2	14.9	12.9	9.852	0.904	21.63	0	0.63	0	25.87	21.93	44.22	11.974	14.002	15.047	23.88	42.25	9.324	29.73	49.18	19.83	0	1.425	0	7.395	10.37	19.74	53.7	26.8	21.7	16
Os02g0289700	59.1	45.6	40.9	75.53	79.55	72.66	87.7	70.8	92.6	61.22	67.98	119.5	68.577	74.676	54.646	47.76	55.65	64.23	86.39	74.18	98.29	59.15	62.71	50.56	57.04	60.34	96.49	60.1	79.4	51.7	15
Os02g0806700	1657	2052	1885	1213	1134	977.5	3164	3387	2829	2251	2254	2101	2164	2262.7	2531.1	2627	2074	1960	1408	1288	1290	2857	3329	3151	2735	2566	2274	2507	2465	2576	5
Os02g0659500	5.1	4.66	7.54	4.105	1.808	0.865	7.83	2.53	1.38	2.587	2.193	0.941	1.0885	1.8669	0.792	0	0	0	0.929	4.031	3.449	0.739	2.138	5.056	1.056	1.886	2.193	0	0.96	0	14
Os02g0716500	6365	4978	4917	851.4	659.9	930.8	1188	1030	1495	5947	5873	12548	5165	3997	4602.1	6138	5861	5894	2000	1326	1590	1559	1134	2014	5903	8652	8193	3623	4488	3852	3
Os02g0701700	156	140	123	71.42	80.45	57.09	163	199	149	166.4	208.3	128.9	216.62	191.36	234.42	406	150.5	148.2	73.39	74.18	38.8	137.5	173.2	124.9	189.1	149	129.4	235	263	250	13
Os02g0589900	0	0	0	0	0	0.865	0	0	0	1.724	1.096	0	0	0	0.792	11.94	0	1.036	0	0	0.862	0	1.425	1.444	0	2.829	6.579	4.63	1.91	0.83	6
Os02g0758800	5.1	0.93	4.31	7.389	2.712	8.65	0	7.59	3.46	7.76	5.482	0.941	0	5.6007	0	0	0	9.324	5.574	1.613	0	0	0.713	0	0	4.714	0	1.85	0.96	3.33	7
Os02g0102400	362	319	338	572.2	575.8	525.1	377	323	448	387.1	296	451.6	370.1	443.39	379.35	537.3	308.1	354.3	582.5	514.4	550.1	351.2	373.4	307	305.3	384.7	436.4	272	344	250	10
Os02g0199900	364	341	366	711	751.2	755.2	599	480	499	452.6	467.1	514.6	427.79	448.99	466.47	704.5	446.2	357.4	1152	1063	1141	410.4	411.9	442.7	461.6	494	495.6	390	383	369	10
Os02g0470600	72.4	72.7	57.1	63.22	52.43	64.01	19.6	22.1	6.91	38.8	17.54	25.4	50.072	43.872	34.847	83.59	41.22	46.62	34.37	57.27	44.84	36.23	37.77	31.78	34.86	33	15.35	74	42.1	55.8	8
Os02g0251900	69.3	59.6	57.1	24.63	8.136	7.785	32.9	31.6	37.3	61.22	18.64	8.467	10.885	16.802	35.638	59.7	71.11	40.41	9.29	32.25	15.52	70.98	53.45	119.2	8.451	19.8	10.96	13	12.4	5.83	9
Os02g0636400	335	355	345	407.2	436.6	296.7	167	191	163	275.9	250	196.6	367.92	367.78	351.63	346.3	296.8	292.2	424.5	445.9	384.5	194.5	163.2	196.5	282	231	252.2	289	293	294	16
Os02g0188000	800	734	978	353	354.4	248.3	777	838	826	901.8	948.4	844.9	1150.6	830.77	1077.9	1158	813.1	691	429.2	362	370.8	373.4	543	491.1	717.3	581.7	489	669	752	831	13
Os02g0123300	67.3	66.1	69	85.38	94.01	96.02	15.7	31.6	30.4	34.49	30.7	30.11	45.718	39.205	53.062	47.76	60.8	55.95	20.44	29.03	38.8	63.59	70.55	31.06	39.09	29.23	19.74	50	39.2	43.3	8
Os02g0105300	1.02	0	0	0	0	0	0	0	0	0	0	0	0	0	0	0	0	0	0	0	0	0	0	0	0	0	0	0	0	0	19
Os02g0166200	1587	1845	2154	1055	969.1	808.8	766	913	981	1732	2016	1063	1735.1	1585	2338.7	2866	1432	2089	884.4	1155	966.5	1500	1281	977.9	2281	1841	1485	2666	2561	2736	13
Os02g0299200	1.02	0.93	0	5.747	5.424	4.325	0	0	0	0.862	0	0	0	0	0	0	2.061	0	16.72	6.45	16.38	0	0.713	0	0	0	0	0.93	0.96	0.83	16
Os02g0657775	0	0	0	0	0	0	0	0	0	0	0	0	0	0	0	0	0	0	0.929	0	0	0	0	0	0	0	0	0	0	0	16
Os02g0180900	7.14	7.45	2.16	4.105	0	0	1.57	2.53	2.76	0.862	5.482	0.941	1.0885	0.9335	5.5438	0	5.153	5.18	6.503	5.644	0.862	1.479	2.851	5.778	5.282	0.943	0	8.33	6.69	5	1
Os02g0592200	161	128	191	309.5	319.1	270.8	238	224	198	104.3	98.68	83.73	185.05	119.48	137.01	262.7	198.9	129.5	248	283	233.7	143.4	173.2	179.8	79.23	70.71	67.98	152	168	133	10
Os02g0694700	6.12	7.45	4.31	9.852	6.328	17.3	31.3	35.4	39.4	9.484	20.83	11.29	13.062	9.3345	2.3759	0	5.153	4.144	13.01	15.32	11.21	26.62	20.67	28.17	4.225	5.657	13.16	7.4	16.3	10	18
Os02g0105800	80.5	87.6	52.8	78.81	70.51	83.91	59.5	46.8	49.1	41.38	44.95	68.68	60.957	64.408	43.558	83.59	52.56	67.34	58.52	67.73	81.05	81.33	79.1	46.22	46.48	95.23	72.37	50	65	32.5	2
Os02g0819500	4600	3775	4612	1765	1720	1659	3093	3199	3615	4485	4805	5510	5827.9	5615.6	5722	6508	5551	4999	2082	2358	2120	3644	3404	3686	6517	6199	6149	5465	5320	5863	3
Os02g0317700	203	146	158	215.9	191.6	230.1	284	279	296	227.6	217.1	257.8	223.15	205.36	203.54	191.1	181.4	176.1	249	305.6	241.4	247.7	221.6	225.3	209.2	196.1	232.4	185	180	177	15
Os02g0168400	42.8	30.7	29.1	43.51	40.68	32.87	33.7	49.3	50.4	20.69	26.31	8.467	28.302	33.604	28.511	59.7	28.85	36.26	31.58	37.9	44.84	68.76	47.03	46.95	23.24	16.97	30.7	33.3	25.8	32.5	17
Os02g0686100	91.8	47.5	74.4	21.35	15.37	10.38	3.13	6.33	6.22	28.45	27.41	47.98	20.682	28.004	19.799	119.4	161.8	71.49	78.96	47.57	45.7	12.57	7.839	21.67	57.04	84.86	111.8	49	25.8	56.7	13
Os02g0689500	135	156	103	66.5	47.01	76.12	142	103	182	163.8	103.1	158.1	121.91	85.878	113.25	107.5	159.7	122.3	82.68	106.4	134.5	203.3	198.8	227.5	150	129.2	217.1	99.9	72.7	80	9
Os02g0755500	66.3	77.3	73.3	376.8	624.6	142.7	955	1975	837	225	199.5	135.5	269.95	104.55	344.51	11.94	40.19	71.49	177.4	89.5	109.5	198.9	391.9	254.2	112	67.88	48.24	55.5	38.3	81.6	18
Os02g0193200	41.8	54	38.8	28.73	18.08	17.3	18.8	20.2	16.6	32.76	18.64	19.76	39.187	36.405	45.142	83.59	168	67.34	18.58	45.15	58.63	15.53	22.09	34.67	35.92	25.46	30.7	28.7	35.4	51.7	13
Os02g0174300	69.3	65.2	66.8	280.8	293.8	261.2	376	294	328	70.7	62.49	63.98	93.613	96.146	58.605	47.76	56.68	62.16	250.8	237	256.9	173.8	158.9	213.8	69.72	75.43	72.37	68.5	75.5	70	12
Os02g0261100	265	213	280	114.9	128.4	143.6	150	156	138	296.6	269.7	261.5	226.41	254.83	259.76	59.7	184.5	172	189.5	155.6	210.4	94.64	129.7	137.9	221.8	188.6	271.9	176	211	155	20
Os02g0189400	0	0	1.08	0	0	0.865	0	0.63	0	0	0	0	1.0885	0	0	0	1.031	1.036	0	0	0	2.218	0	1.444	0	1.886	2.193	0	0	0	11
Os02g0180700	586	436	403	165	128.4	186.9	150	207	248	261.2	172.1	189.1	178.52	233.36	216.21	250.8	248.4	264.2	170	171.7	128.5	209.2	245.1	202.2	233.5	179.1	254.4	271	234	217	14
Os02g0507100	0	0.93	1.08	7.389	7.232	5.19	4.7	1.9	2.76	2.587	5.482	0	2.177	0.9335	2.3759	0	2.061	1.036	4.645	7.257	9.484	5.176	3.563	6.5	0	3.771	4.386	0.93	1.91	5.83	10
Os02g0572050	1.02	0	0	0.821	0	0	0	0.63	0	1.724	0	0	0	0.9335	0.792	0	0	0	1.858	0	0	0	0	0	0	0	0	0	0	0	7
Os02g0193732	1.02	0.93	2.16	0.821	0	0.865	0	0	0.69	5.173	0	0.941	4.3541	2.8004	4.7518	0	2.061	1.036	0	0	0	0	0	0	1.056	0	0	0.93	2.87	0.83	4
Os02g0727300	303	347	348	300.5	346.2	309.7	100	132	121	133.6	211.6	90.32	259.07	189.49	230.46	501.5	260.7	376.1	129.1	235.4	181.9	357.1	314.3	153.8	211.3	195.2	72.37	398	389	422	8
Os02g0110101	0	0	0	0.821	0	0	0.78	0.63	0	0	0	0	0	0	0	0	0	0	0	0	0	1.479	0	0	0	0	0	0	0	0	18
Os02g0593900	305	288	292	302.1	272.1	281.1	348	313	256	346.6	286.2	397	303.7	367.78	308.87	394.1	308.1	266.3	320.5	337	369.9	307.6	300.7	280.2	313.7	313	355.3	257	265	258	20
Os02g0661400	0	0	0	0	0	0	0	0	0	0	0	0	0	0	0	0	0	0	0	0	3.449	0	0	0	0	0	0	0	0	0	16
Os02g0506500	637	691	600	574.7	639.1	593.4	837	858	892	691.5	749.9	728.2	582.36	641.28	658.12	979.2	578.1	445.5	513.7	519.2	511.3	760.1	697	723	532.4	582.7	657.9	607	478	577	18
Os02g0227000	404	372	441	374.4	433.9	452.4	175	240	173	342.3	423.2	353.7	655.29	567.54	638.32	883.6	443.1	402	350.2	435.4	403.5	239.6	219.5	208	415.2	324.3	333.3	489	529	486	13
Os02g0556600	426	426	421	416.2	424.9	428.2	501	510	547	401.8	395.8	367.9	469.15	404.18	527.45	692.6	440	420.6	346.5	416	437.1	495.4	520.2	453.6	426.8	395.1	372.8	477	405	547	6
Os02g0778200	3305	3643	3728	4302	4683	4218	4358	4432	4635	2810	3121	3146	4201.7	3686.2	3525	4144	3158	3316	3757	3856	4160	4573	3906	4562	2873	2981	2546	2976	3178	3194	12
Os02g0829500	1580	1576	1843	1459	1436	1497	1599	1991	1810	1646	1784	1893	1658.9	1697	1563.3	1576	1395	1458	1831	1560	1692	1354	1516	1530	1725	1481	1634	1425	1481	1283	15
Os02g0642850	1.02	0	1.08	0	0.904	0	0	0	0	1.724	0	0	0	0	0.792	0	0	0	0	0.806	0	0.739	0	0	0	0	0	2.78	2.87	0	1
Os02g0549401	13.3	13	19.4	41.05	43.39	24.22	50.1	34.2	20	13.79	20.83	0.941	10.885	10.268	9.5036	11.94	14.43	25.9	21.37	47.57	64.67	44.36	32.78	41.89	9.507	20.74	13.16	18.5	17.2	18.3	12
Os02g0727500	1293	1202	1220	1392	1483	1436	2494	2228	2246	1549	1560	1618	1286.6	1417.9	1355.8	1158	1225	1268	1629	1446	1538	1635	1631	1823	1358	1500	1447	1163	1251	1171	18
Os02g0696700	760	599	792	631.3	570.4	741.3	557	605	618	469	458.3	840.8	749.99	748.63	788.8	1075	876	834	1107	911.9	929.5	539	489.6	679.6	506	632.6	741.2	756	938	830	16
Os02g0806000	1040	999	1173	1204	1454	1279	828	715	701	1119	1205	981.3	1359.6	1293.8	1195.1	1254	1110	1114	1488	1600	1598	521.3	533.1	634.1	1013	998.5	1035	980	1053	1097	16
Os02g0485800	0	0	1.08	0	0	0	0	0	0	0	1.096	0.941	0	2.8004	0	0	0	1.036	2.787	0	0	0	0	0	0	1.886	2.193	0	0	1.67	11
Os02g0735100	366	379	376	227.4	215.1	233.6	210	246	216	356.9	358.5	344.3	459.36	361.25	403.9	298.5	377.2	321.2	247.1	239.5	238	166.4	201.7	218.1	360.2	362.1	339.9	330	366	317	3
Os02g0564000	1686	1554	1627	865.3	885.9	785.5	1455	1420	1372	1724	1935	1946	1774.3	1879	1928.4	1636	1766	1579	1058	974	933.8	1152	1213	1138	1831	1879	1952	1716	1524	1749	3
Os02g0232900	79.5	75.4	83	37.76	9.04	38.06	31.3	15.2	87.8	36.21	32.89	36.69	13.062	21.469	27.719	35.82	15.46	23.83	7.432	15.32	11.21	32.53	91.22	52	16.9	10.37	28.51	13	52.6	20	14
Os02g0679700	1.02	0	1.08	0	1.808	0	0	0.63	0	0	0	0	0	0	0	0	0	0	2.787	0	2.587	0	0	0	0	0	0	0.93	0	2.5	16
Os02g0300700	528	383	392	177.3	191.6	168.7	179	268	199	341.4	287.3	426.2	218.79	433.12	353.22	286.6	414.3	509.7	170.9	137.9	157.8	181.9	193.1	233.9	304.2	392.2	513.1	256	308	313	3
Os02g0273100	112	145	101	168.3	149.2	158.3	441	313	574	110.4	137	120.4	100.14	103.61	63.357	83.59	77.29	69.41	113.3	89.5	104.3	289.1	175.3	424.7	58.1	85.8	103.1	30.5	43	58.3	18
Os02g0231000	0	0	0	0	0.904	0	0	0	0	0.862	0	0	0	0	0	0	0	0	0	1.613	2.587	0	0	0	0	1.886	0	0.93	0	1.67	16
Os02g0690000	12.2	7.45	6.47	22.17	18.08	9.516	49.3	60.7	29.7	6.897	13.16	17.88	15.239	8.4011	18.215	23.88	8.244	6.216	15.79	5.644	13.8	33.27	23.52	35.39	9.507	6.6	4.386	3.7	8.61	12.5	18

Os02g0174000	31.6	27.9	22.6	33.66	33.45	24.22	17.2	20.2	22.1	25	40.57	40.46	29.39	36.405	24.551	35.82	35.04	26.94	22.29	12.09	18.11	15.53	21.38	23.11	41.2	36.77	65.79	22.2	36.3	44.2	11
Os02g0221900	30.6	26.1	33.4	52.54	47.91	54.5	485	316	217	44.83	53.72	25.4	23.947	36.405	41.974	35.82	74.2	49.73	226.7	155.6	144	196.7	207.4	509.9	54.93	33	67.98	73.1	63.1	63.3	18
Os02g0583700	0	0.93	0	0	0	0	0	0	0	0	0	0	0	0	0	0	0	0	0	0	0	0	0	0	0	0	0	0	0	0	14
Os02g0577400	72.4	89.4	78.7	145.3	141	151.4	141	125	135	149.2	166.7	211.7	168.72	132.55	143.35	83.59	111.3	98.42	175.6	203.2	178.5	90.21	94.07	110.5	137.3	95.23	105.3	77.7	68.9	84.1	15
Os02g0322400	1.02	1.86	1.08	0.821	0	0.865	3.13	0.63	1.38	0.862	1.096	3.763	1.0885	1.8669	0.792	0	2.061	1.036	2.787	0.806	2.587	1.479	0.713	2.889	2.113	0.943	2.193	0.93	2.87	3.33	20
Os02g0727700	152	129	157	47.62	44.29	46.71	134	143	158	143.1	149.1	107.3	85.993	85.878	100.58	131.4	106.1	94.28	37.16	69.34	55.18	89.47	118.3	115.6	95.07	79.2	127.2	70.3	72.7	96.6	5
Os02g0491300	0	0	2.16	0.821	0.904	0	1.57	0	1.38	0.862	2.193	2.822	0	0	1.5839	0	1.031	0	1.858	0	0.862	2.218	0.713	2.889	0	3.771	2.193	0	0.96	0	20
Os02g0739600	1409	1472	1522	958.1	1108	894.5	686	863	1009	1172	1130	1259	1208.3	1319.9	1290.9	1851	1609	1516	962.4	675.7	891.5	708.3	797.4	825.5	1247	1501	1088	1267	1326	1372	13
Os02g0140101	363	331	388	247.1	247.7	308	301	362	271	411.3	315.8	478.9	360.3	352.84	373.81	668.7	428.7	349.1	411.5	372.5	408.7	320.2	280.8	309.1	391.9	478	578.9	380	358	338	13
Os02g0751300	5.1	5.59	9.7	3.284	2.712	6.055	6.26	4.43	2.76	6.897	5.482	0.941	1.0885	10.268	5.5438	0	1.031	6.216	4.645	9.675	0	3.697	6.414	2.889	4.225	1.886	0	8.33	0.96	6.66	7
Os02g0209400	11.2	20.5	16.2	40.23	33.45	38.06	8.61	15.2	26.3	16.38	4.386	3.763	7.6196	16.802	8.7116	11.94	5.153	12.43	55.74	67.73	36.21	6.654	19.95	17.33	1.056	2.829	6.579	8.33	5.74	7.5	10
Os02g0286891	51	29.8	45.3	41.87	37.97	45.85	59.5	73.4	39.4	35.35	80.04	59.27	54.426	70.009	48.31	131.4	74.2	62.16	63.17	49.99	63.8	49.54	49.88	62.11	98.24	45.26	65.79	58.3	75.5	72.5	13
Os02g0817800	52	64.3	64.7	61.57	83.17	95.16	73.6	64.5	60.8	79.32	85.52	95.02	105.59	87.744	89.492	119.4	70.08	85.99	44.59	50.8	62.08	79.11	71.26	65.72	69.72	70.71	67.98	81.4	85.1	105	13
Os02g0834050	19.4	31.7	31.2	50.9	42.49	51.9	54.8	52.5	45.6	19.83	31.8	28.22	32.656	29.87	37.222	107.5	29.89	35.23	61.31	40.31	57.77	39.93	19.95	57.06	19.01	34.89	28.51	29.6	16.3	40	6
Os02g0193600	0	0.93	3.23	0	0	2.595	0	0	0	0	3.289	1.882	1.0885	5.6007	0.792	0	2.061	2.072	0.929	0.806	1.724	0	0	1.444	0	4.714	4.386	0	1.91	0	11
Os02g0290400	688	599	575	588.6	621	541.5	431	436	424	594	622.8	773.4	726.04	869.98	684.26	704.5	669.8	664.1	761.7	677.3	748.4	442.2	456.8	392.2	641.2	732.6	725.9	662	627	620	3
Os02g0119700	50	37.3	30.2	61.57	62.37	41.52	57.9	44.3	39.4	34.49	33.99	36.69	42.452	43.872	48.31	71.65	43.28	38.33	64.1	57.25	49.15	45.84	52.73	43.33	30.63	48.08	37.28	32.4	36.3	30	6
Os02g0820600	138	151	137	154.3	116.6	150.5	199	161	180	169.9	149.1	188.2	156.75	173.62	171.86	274.6	116.5	117.1	151.4	153.2	151.8	152.3	141.8	174.1	165.8	187.6	166.7	143	142	133	6
Os02g0730600	26.5	13	34.5	8.21	3.616	4.325	2.35	12	6.22	27.59	24.12	16.93	26.124	17.736	31.679	47.76	21.64	29.01	6.503	0.806	2.587	2.958	3.563	2.889	26.41	18.86	10.96	10.2	16.3	6.66	13
Os02g0750200	1910	1704	1821	1697	1601	1868	2759	2644	2579	2036	2187	3297	2386	2183.3	2158.1	1994	2746	2552	3121	2995	3460	1942	1730	2625	2673	3105	3256	1956	2164	2101	15
Os02g0687900	321	193	206	63.22	63.28	107.3	104	86	76.7	122.4	85.52	190	116.47	294.97	175.02	191.1	387.5	285.9	157.9	122.6	169.9	79.11	134.7	156	150	216.9	311.4	291	200	190	3
Os02g0105200	382	379	360	265.2	311	282.9	438	392	471	451.8	423.2	414	383.16	394.85	361.14	453.8	438	349.1	315.8	329	327.6	340.1	364.2	353.9	391.9	397.9	401.3	395	384	363	5
Os02g0602900	168	166	173	195.4	168.1	172.1	107	88.6	74.6	177.6	172.1	159	134.98	196.02	186.11	262.7	277.2	237.3	158.9	132.2	183.7	156	101.9	115.6	200.7	154.6	188.6	273	250	321	13
Os02g0814400	462	524	500	699.5	745.8	673.9	1466	1451	1717	719.9	856.3	926.7	568.21	463.93	509.23	585.1	489.5	538.7	793.3	909.5	946.7	918.3	886.5	1143	577.8	622.3	631.6	334	393	384	18
Os02g0797600	5945	4234	4968	1658	1458	1610	1754	1764	1942	4887	4568	5096	3545.3	3861.7	3680.3	5660	5655	4827	2255	2308	2555	1924	2124	2834	4883	5060	5186	3913	4270	4007	3
Os02g0657000	2.04	2.79	2.16	0.821	0	0.865	0.78	0	0.69	2.587	3.289	1.882	1.0885	1.8669	0	11.94	20.61	13.47	1.858	4.838	1.724	0.739	1.425	10.83	3.169	5.657	13.16	1.85	13.4	8.33	13
Os02g0635700	387	293	345	271.7	227.8	262.1	323	242	245	389.7	351.9	399.8	526.84	435.92	486.27	477.6	418.4	359.5	328.8	324.9	354.4	219.6	168.9	206.6	369.7	374.3	353.1	470	452	447	3
Os02g0298900	80.5	59.6	72.2	188	189.8	186	212	192	212	75.87	82.23	59.27	57.692	47.606	57.814	11.94	32.98	82.88	255.5	278.2	225	136	143.2	188.5	46.48	38.66	98.68	38.9	71.7	50	12
Os02g0131600	76.5	49.4	71.1	74.71	94.92	79.58	91.6	86	97.4	71.56	87.71	138.3	57.692	94.279	74.445	71.65	52.56	68.38	102.2	96.75	96.57	55.45	71.98	55.61	62.33	62.23	70.17	58.3	71.7	53.3	15
Os02g0144300	277	301	336	242.2	254.9	257.8	268	237	236	267.3	234.6	230.5	228.59	308.04	220.96	429.9	311.2	298.4	236	270.9	312.1	346	280.1	309.8	292.6	292.3	219.3	347	276	306	6
Os02g0564200	1174	1193	1123	713.4	696.1	810.6	1257	1023	1211	1443	1293	1775	1260.5	1267.6	1154.7	919.5	1261	1154	1075	832.9	939	805.9	812.4	1015	1363	1434	1401	995	1056	998	20
Os02g0141300	478	591	482	686.3	748.5	628.9	708	853	634	530.2	551.5	442.2	424.52	371.51	505.27	406	416.3	435.1	427.3	423.3	391.4	656.6	887.9	642.8	545.1	506.3	486.8	451	464	527	17
Os02g0469200	1654	1954	1899	1800	1909	1741	1651	1651	1687	1483	1456	1256	1933.2	1748.4	1778	2436	1850	1806	1598	1740	1463	1968	1795	1664	1625	1439	1401	2122	1980	2252	8
Os02g0537950	0	0	0	0	0	0	0	0	0	0	0	0	0	0	0	0	0	0	0	0	0.862	0	0	0	0	0	0	0	0	0	16
Os02g0696900	45.9	68.9	50.6	26.27	28.93	20.76	36.8	47.4	53.2	43.97	44.95	37.63	44.629	43.872	72.861	83.59	57.71	66.31	15.79	17.74	16.38	53.98	50.6	54.17	35.92	51.86	35.09	55.5	65	74.1	13
Os02g0520000	0	0.93	0	0	0	0	0	0	0	0	0	0	0	0	0	0	0	0	0	0	0	0	0	0	0	0	0	0	0	0	14
Os02g0489800	1.02	8.38	2.16	6.568	8.136	3.46	3.91	5.06	0.69	0.862	2.193	0	4.3541	0.9335	0.792	0	6.183	4.144	7.432	6.45	4.311	2.218	4.988	2.167	1.056	3.771	13.16	1.85	0.96	0.83	11
Os02g0639100	156	189	129	233.2	225.1	259.5	108	67	117	237.1	202.8	283.2	74.019	143.75	96.62	59.7	172.1	127.4	241.5	144.3	229.3	269.9	126.1	210.9	194.4	261.2	285.1	125	127	115	11
Os02g0226801	2.04	5.59	4.31	3.284	1.808	0.865	3.91	7.59	6.91	3.449	4.386	3.763	1.0885	2.8004	1.5839	0	11.34	4.144	6.503	0.806	1.724	2.218	6.414	7.222	8.451	12.26	28.51	0.93	6.69	6.66	11
Os02g0631200	837	673	656	429.4	429.4	412.6	235	309	234	374.2	491.2	595.5	361.39	511.53	475.18	847.8	1161	936.6	524.9	338.6	439.7	360.1	343.5	343.8	741.6	817.4	934.2	835	791	741	3
Os02g0106800	897	913	861	702.8	676.2	685.1	1881	1751	1904	1021	1046	1296	1030.8	976.39	972.53	1182	1002	897.2	824.9	738.6	801.9	1116	1105	1431	1069	1220	1410	877	854	864	5
Os02g0693700	11.2	1.86	4.31	0	0	0.865	0	0	0	0	0	0	1.0885	4.6673	3.9598	0	17.52	12.43	3.716	0	0.862	0	2.851	0.722	0	7.543	6.579	11.1	6.69	2.5	1
Os02g0136933	4.08	2.79	7.54	6.568	5.424	6.92	0.78	1.9	1.38	0	1.096	0	0	0	2.3759	0	4.122	4.144	0	1.613	1.724	0.739	2.851	2.889	2.113	1.886	2.193	8.33	1.91	4.17	8
Os02g0738900	315	346	294	334.1	354.4	339.1	346	327	283	260.4	228.1	228.6	205.73	245.5	251.05	358.2	267.9	244.5	231.3	235.4	222.5	305.4	359.9	301.2	229.2	197.1	243.4	382	291	275	17
Os02g0686300	122	110	95.9	70.6	60.57	70.93	75.2	79.7	96.7	93.98	93.19	79.03	83.816	91.478	95.828	83.59	107.2	129.5	48.31	80.63	78.46	113.1	113.3	114.1	115.1	131.1	87.72	129	120	111	1
Os02g0440900	7.14	0.93	3.23	5.747	9.944	6.055	3.13	14.5	6.91	6.897	6.578	7.527	0	3.7338	4.7518	0	8.244	5.18	3.716	6.45	2.587	5.176	7.126	5.778	6.338	3.771	0	3.7	6.69	3.33	18
Os02g0136000	5.1	8.38	7.54	14.78	3.616	4.325	7.05	9.49	8.98	6.035	4.386	4.704	5.4426	1.8669	7.1277	0	0	2.072	0.929	2.419	1.724	5.915	4.988	10.11	1.056	5.657	0	8.33	5.74	5	18
Os02g0204000	48.9	46.6	36.6	79.63	45.2	85.64	97.9	63.3	81.5	72.42	59.21	76.21	47.895	60.674	67.317	35.82	60.8	49.73	121.7	128.2	113	42.15	51.31	59.95	62.33	66	103.1	50	77.5	51.7	15
Os02g0326500	255	254	276	231.5	244.1	258.6	218	244	220	291.4	296	271.9	232.94	218.43	248.68	561.2	303	297.3	201.6	246.7	236.2	407.4	302.2	350.3	313.7	337.5	285.1	327	311	332	6
Os02g0188600	0	0	0	0	0.904	0	0	0	0	0	0	0	0	0	0	0	0	0	0	0	0	0.739	0	0	1.056	0	0	0	0	0	2
Os02g0814900	289	340	319	295.6	296.5	333.9	251	293	316	288.8	369.5	287	283.02	377.11	306.49	322.4	360.7	364.7	342.8	326.5	397.5	359.3	344.9	315.6	320.1	330	311.4	377	388	326	1
Os02g0550900	186	178	211	269.3	254	204.2	263	262	245	205.2	189.7	122.3	189.4	210.96	235.21	262.7	171.1	218.6	200.7	208.8	187.1	247	232.3	199.3	229.2	170.7	175.4	220	186	217	18
Os02g0527600	696	564	587	249.6	245.9	256.1	546	315	1139	550.9	546	624.7	641.14	608.61	700.89	632.9	824.4	679.6	355.8	286.2	320.7	2858	1744	2057	696.1	868.4	574.5	764	717	769	9
Os02g0822100	783	1012	679	811.9	816.3	805.4	399	428	325	868.2	709.4	888.1	621.54	744.89	731.78	656.8	859.5	789.5	686.5	805.5	762.2	233.6	245.9	344.5	816.6	842	850.9	916	686	854	3
Os02g0508000	244	221	224	252.9	202.5	248.3	240	213	299	265.6	269.7	338.7	225.32	277.24	216.21	262.7	212.3	235.2	302.8	252.4	289.7	204.1	230.9	237.6	222.9	291.3	269.7	217	225	196	20
Os02g0798100	182	146	171	119	119.3	96.89	186	200	218	272.5	211.6	195.7	160.01	135.35	179.78	203	207.1	189.6	118	116.1	61.22	260.3	215.9	239.1	231.3	203.7	182	195	197	167	5
Os02g0627800	30.6	15.8	18.3	7.389	8.136	6.92	7.83	3.16	6.22	12.93	17.54	18.82	15.239	4.6673	13.463	0	20.61	18.65	11.15	0.806	11.21	9.612	5.701	17.33	13.73	4.714	2.193	22.2	20.1	7.5	19
Os02g0110000	35.7	29.8	47.4	31.2	45.2	53.63	84.6	55.7	62.9	67.25	66.88	88.44	57.692	47.606	56.23	71.65	55.65	48.69	78.03	60.47	76.74	36.97	32.78	48.39	54.93	62.23	39.47	37.9	64.1	40	15
Os02g0662700	241	212	242	105.1	107.6	147.1	240	233	182	280.2	232.4	315.2	271.04	306.17	247.09	238.8	250.4	261.1	138.4	135.5	122.4	150.8	186.7	187.1	269.4	324.3	342.1	254	249	188	3
Os02g0639600	3.06	0.93	1.08	2.463	0.904	15.57	3.91	0.63	4.15	0	6.578	0	1.0885	0.9335	3.9598	0	1.031	7.252	1.858	0.806	6.036	4.436	1.425	0.722	0	0.943	0	1.85	4.78	2.5	2
Os02g0767150	169	188	177	186.4	194.4	204.2	139	120	155	188.8	169.9	153.4	164.37	193.22	190.07	322.4	185.5	185.5	170	161.3	178.5	156.7	151.1	162.5	188	187.6	190.8	195	178	189	13
Os02g0828533	0	0	0	0.821	0	0	0	0	0	0	0	0	0	0	0	0	1.031	0	0	0	0	0.739	0	0.722	0	0	2.193	0.93	0	0.83	11
Os02g0831800	161	138	138	344	363.4	262.1	338	347	409	269	166.7	290.7	153.48	231.5	156.02	179.1	132.9	121.2	456.1	349.1	337.1	266.9	329.2	294	164.8	149	188.6	118	124	102	12
Os02g0584900	96.9	111	90.5	42.69	49.72	29.41	87.7	55.7	87.1	59.49	38.37	90.32	43.541	61.608	59.397	23.88	63.89	77.7	17.65	32.25	35.35	70.98	62	82.34	71.83	74.48	52.63	61.1	29.6	28.3	14
Os02g0133200	0	0.93	0	0	0	3.46	4.7	1.27	4.15	0	0	0	0	0.9335	0.792	11.94	2.061	0	5.574	1.613	0	2.958	2.851	1.444	0	0	0	0	1.91	0.83	6
Os02g0702700	28.5	15.8	24.8	11.49	10.85	25.95	29.7	21.5	26.9	27.59	19.74	20.7	15.239	22.403	19.007	23.88	35.04	15.54	17.65	24.99	27.59	25.88	15.68	26	28.52	21.69	26.32	20.4	24.9	15.8	5
Os02g0499000	4314	3808	5062	1265	1153	1548	2324	2424	2512	4092	4559	3863	5796.4	5555.9	5360	9087	5629	4996	1946	1933	1979	2411	2226	2705	5155	5078	4384	4839	4892	5599	13
Os02g0155700	3.06	2.79	5.39	1.642	0	6.92	1.57	2.53	1.38	3.449	0	1.882	0	0.9335	1.5839	11.94	3.092	5.18	0	0	0.862	8.873	0.713	5.056	1.056	4.714	0	11.1	5.74	0.83	6
Os02g0709000	173	86.6	246	13.96	10.85	155.7	0	1.27	1.38	131.9	122.8	236.1	65.311	212.83	175.02	131.4	243.2	276.6	65.03	59.66	128.5	3.697	1.425	2.167	405.6	451.6	502.2	74	20.1	28.3	3
Os02g0771400	3.06	0.93	1.08	0	0.904	2.595	1.57	1.27	0	0	0	0	1.0885	0	1.5839	0	2.061	0	0	0	0	1.479	0.713	0	0	0.943	0	0.93	0	1.67	19
Os02g0531600	0	0	0	0	0	0	0	0	0	0	0	0.941	1.0885	0	0	0	3.092	3.108	0	0	0	0	0	0	0	0	0	0	0	0.83	1
Os02g0200300	24.5	47.5	32.3	125.6	145.5	169.5	250	307	277	43.97	55.92	53.63	47.895	31.737	54.646	23.88	52.56	39.37	138.4	81.43	124.2	290.6	279.4	271.6	46.48	51.86	59.21	48.1	29.6	28.3	18
Os02g0226200	20.4	20.5	5.39	1.642	0.904	6.055	7.83	0	6.22	8.622	4.386	11.29	8.7082	14.935	9.5036	23.88	6.183	10.36	0	0	0.862	7.394	8.552	5.778	5.282	25.46	39.47	13.9	12.4	26.7	11
Os02g0314300	2277	2610	2817	1704	2048	1878	400	380	478	2900	3084	2665	3366.8	3321.2	3221.7	4609	3683	3577	1473	2075	1979	550.1	589.3	533	3300	3592	3002	4099	3882	4056	3
Os02g0672200	508	835	684	588.6	668.9	509.5	515	571	565	682	762	652.9	588.89	453.66	552.79	752.3	476.1	636.1	320.5	325.7	364.7	737.2	671.3	610.3	714.1	617.6	440.8	448	460	557	7
Os02g0603800	8.16	13	20.5	3.284	0.904	2.595	0.78	1.9	0	5.173	8.771	3.763	7.6196	9.3345	11.879	0	13.4	7.252	0	0	0	5.915	1.425	4.333	7.395	6.6	4.386	16.7	17.2	15	1
Os02g0165500	0	0	2.16	1.642	0.904	0	0.78	2.53	4.84	0	0	0	1.0885	0	0	0	0	0	3.716	0	0	5.915	0.713	6.5	0	0	0	0	0	0	9
Os02g0684800	335	351	325	394.9	378.8	367.6	539	544	577	342.3	387	320.8	327.64	350.98	342.92	453.8	290.6	313.9	340	417.7	375.9	448.8	443.3	449.2	330.6	305.5	359.6	339	362	312	18
Os02g0738300	2.04	12.1	9.7	2.463	9.04	2.595	11.7	8.22	13.1	18.97	7.675	1.882	5.4426	9.3345	8.7116	0	0	5.18	0	5.644	0	18.48	12.83	10.83	3.169	1.886	4.386	4.63	2.87	1.67	17
Os02g0615500	193	244	391	298.8	366.1	293.3	98.6	134	108	146.6	132.7	55.51	145.86	159.62	224.92	1099	138.1	190.6	104	183.8	98.29	298.7	255.1	114.1	203.9	98.06	74.56	268	238	269	6
Os02g0832500	1764	1885	1849	1821	1718	1632	2382	2605	2354	1693	1714	1977	1748.2	1719.4	1630.7	1887	1920	1832	1642	1694	1669	2413	2326	2427	1862	1888	1934	1746	1904	1820	18
Os02g0287900	80.5	87.6	75.4	55.01	58.76	47.58	18.8	26.6	14.5	80.18	69.07	78.09	88.17	111.08	88.7	203	110.3	95.32	49.23	35.48	62.08	33.27	41.33	29.61	114.1	78.26	50.44	125	99.5	81.6	13
Os02g0728700	294	286	283	320.2	433	366.8	239	230	222	302.6	325.6	328.3	314.58	345.38	357.97	441.8	341.1	322.2	393.9	363.6	377.7	225.5	259.4	221	268.3	361.1	405.7	320	308	341	16
Os02g0144200	0	0	1.08	0	0	0	0	0	0	0	0	0.941	0	0	0	0	1.031	1.036	0	2.419	0.862	0	2.851	0.722	4.225	0	0	0	1.91	0.83	1
Os02g0518400	91.8	76.4	98.1	43.51	28.02	67.47	80.6	88.6	83.6	55.18	61.4	96.9	25.036	70.942	56.23	609	416.3	439.3	202.5	212.9	222.5	323.9	201	255.7	401.4	355.5	399.1	534	495	421	13
Os02g0830801	76.5	104	112	82.92	96.72	107.3	21.9	8.86	18	33.63	42.76	17.88	33.744	36.405	55.438	107.5	53.59	82.88	30.66	28.22	31.9	105.7	89.08	52.72	47.54	32.06	50.44	104	85.1	103	8
Os02g0146500	1796	1490	1582	649.4	588.5	679.9	1060	944	889	1211	1191	1252	1322.6	1423.5	1427.1	1516	1655	1595	843.5	851.4	826	938.3	881.5	933.9	1235	1406	1325	1361	1280	1251	3
Os02g0749800	514	465	580	792.2	896.7	716.3	1162	1132	1073	640.6	672.1	654.8	744.55	547	558.34	1134	675	620.6	1032	1026	945.9	497.6	588.6	797.4	605.3	515.7	515.3	496	554	543	12
Os02g0195000	112	125	111	184.7	158.2	170.4	103	169	155	125	131.6	155.2	116.47	108.28	102.16	107.5	83.47	111.9	132.8	137.9	103.5	160.4	171.7	187.8	129.9	134.8	107.5	93.4	98.5	76.6	12
Os02g0637000	9.18	8.38	4.31	9.031	6.328	12.11	12.5	5.69	10.4	18.97	12.06	9.408	13.062	11.201	8.7116	11.94	10.31	8.288	26.01	16.93	27.59	9.612	7.126	7.222	2.113	7.543	21.93	8.33	9.56	7.5	16
Os02g0114150	0	0	1.08	2.463	0	0	0	0	0	0	0	0	2.177	0	0	11.94	0	0	0	0	0	0.739	0	2.167	0	0	0	0	0	0	6
Os02g0797400	0	0	0	1.642	3.616	5.19	2.35	0	1.38	0.862	0	0.941	0	0	0	0	1.031	0	1.858	7.257	4.311	2.218	0	0.722	0	4.714	0	0.93	0.96	0	10
Os02g0608200	2998	2243	2796	2032	1945	2170	1584	2016	1766	2301	2263	2682	3079.4	3022.5	2893.1	4717	3378	3081	3054	3419	3063	2801	2634	2165	2880	3206	3186	3525	3402	3489	13
Os02g0561900	13.3	16.8	32.3	71.42	92.2	24.22	21.1	29.1	24.2	11.21	16.45	4.704	8.7082	8.4011	30.095	23.88	14.43	15.54	60.38	53.21	40.52	8.873	13.54	11.56	14.79	0	4.386	8.33	16.3	14.2	10
Os02g0209201	45.9	77.3	42	54.18	66.89	83.04	34.4	25.3	30.4	17.24	39.47	9.408	30.479	45.739	34.055	59.7	37.1	48.69	22.29	24.19	14.66	66.54	71.26	41.17	39.09	16.97	2.193	38.9	29.6	40	8
Os02g0628600	182	166	158	53.36	42.49	63.15	92.4	84.1	93.3	170.7	202.8	196.6	115.38	144.68	106.12	334.3	212.3	206.2	79.89	64.5	98.29	39.93	48.46	104.7	182.8	223.5	177.6	172	147	157	13
Os02g0173100	320	360	289	1747	2036	1741	1538	1829	1988	313.8	480.2	346.2	300.43	238.96	248.68	334.3	201	295.3	1183	1496	1172	2194	1800	1822	352.8	232.9	252.2	268	198	262	12
Os02g0766600	829	884	996	1009	1172	811.4	1325	1219	986	1433	1275	1245	1080.9	989.46	1127	716.5	847.1	831.9	1144	1026	1036	669.9	764.7	745.4	1071	814.6	936.4	816	664	798	7
Os02g0749100	39.8	32.6	29.1	63.22	75.03	47.58	16.4	21.5	12.4	18.97	20.83	8.467	20.682	16.802	25.343	11.94	34.01	37.3	18.58	9.675	15.52	45.84	21.38	19.5	45.42	21.69	19.74	23.1	29.6	27.5	2
Os02g0759400	1396	797	1446	7.389	1.808	12.98	12.5	8.22	13.1	333.7	227	287.9	128.45	106.41	120.38	238.8	798.7	543.9	3.716	13.71	1.724	62.85	41.33	91	279.9	296.1	278.5	90.7	92.8	98.3	14
Os02g0689700	206	164	210	256.1	325.4	241.3	254	251	209	181.9	235.7	273.8	259.07	196.02	163.94	358.2	166.9	198.9	304.7	272.5	279.4	166.4	178.2	224.6	165.8	143.3	175.4	121	164	167	10
Os02g0713500	1.02	0	0	0	0	0	0	0.63	0	0	0	0	0	0	0	0	0	0	0	0	0	0	0	0	0	0	0	0	0	0	19
Os02g0826300	3.06	10.2	7.54	18.06	10.85	15.57	20.4	36.1	42.8	18.11	16.45	8.467	7.6196	3.7338	8.7116	11.94	5.153	7.252	13.01	14.51	18.97	11.09	25.65	41.89	8.451	10.37	32.89	1.85	2.87	7.5	18
Os02g0700000	572	594	635	479.4	401.4	444.6	424	536	492	544	549.3	501.5	660.73	625.41	662.08	835.9	680.2	642.3	426.4	436.2	511.3	515.4	585.1	598	610.6	574.2	587.7	666	557	657	13
Os02g0810100	456	509	504	504.1	475.5	477.5	611	600	602	448.3	421	380.1	525.76	475.13	568.63	1230	472	425.8	402.2	316.9	338	464.3	558	489.7	509.2	468.6	432	467	461	507	6
Os02g0722650	1.02	2.79	0	0	0.904	0.865	3.13	8.86	5.53	2.587	1.096	2.822	0	4.6673	0.792	0	1.031	8.288	0.929	0	2.587	0.739	5.701	3.611	2.113	0.943	2.193	2.78	2.87	1.67	5
Os02g0121000	764	899	880	2030	2200	1942	1682	1830	1762	872.5	1006	1065	928.51	812.1	638.32	776.2	499.8	675.5	1673	1808	1661	1327	1089	1430	699.3	492.2	519.7	419	498	554	12
Os02g0286200	189	237	211	352.2	388.7	373.7	351	316	376	210.4	257.7	147.7	219.88	246.43	217	406	202	185.5	309.3	341.9	298.3	346	340.6	286	221.8	169.7	199.6	205	183	250	12
Os02g0596500	296	317	302	362.9	378.8	352.9	473	541	453	343.2	322.3	384.8	297.17	343.51	311.24	298.5	316.4	285.9	446.8	297.5	399.2	339.4	350.6	385	289.4	293.2	348.7	265	255	272	18
Os02g0742100	1885	1914	1902	1615	1611	1638	1828	1827	1860	1839	1888	1653	2109.6	1924.8	2116.9	2949	2060	2053	1540	1613	1639	1902	2016	1863	2014	1972	1822	2137	2000	2093	13
Os02g0178800	111	128	129	41.87	37.06	50.17	29	25.3	102	26.73	31.8	21.64	10.885	4.6673	14.255	47.76	47.4	22.79	26.01	28.22	10.35	25.14	32.07	72.95	15.85	13.2	10.96	21.3	17.2	16.7	14
Os02g0639500	0	0	0	0.821	0	0	0	0	0	0	0	0	0	0	0	0	0	0	0	0	0	0	0	0	0	0	0	0	0	0	2
Os02g0663800	3.06	6.52	10.8	105.1	118.4	89.1	113	114	96.7	5.173	6.578	4.704	7.6196	7.4676	9.5036	0	1.031	3.108	117	111.3	67.25	85.03	94.78	69.34	2.113	1.886	2.193	8.33	8.61	4.17	12
Os02g0175400	61.2	54	61.4	69.78	77.74	95.16	16.4	29.1	18	50.01	47.14	51.75	15.239	42.005	26.927	35.82	48.43	47.66	72.46	62.08	66.39	28.1	33.49	36.11	39.09	59.4	74.56	31.5	30.6	36.7	16
Os02g0289300	30.6	25.1	33.4	36.12	46.1	29.41	30.5	27.2	29	18.11	25.22	20.7	10.885	24.27	15.047	47.76	27.82	30.05	41.8	29.83	42.25	41.41	24.94	47.67	25.35	26.4	24.12	29.6	29.6	29.2	6
Os02g0463500	1.02	0	2.16	0	0	0	0	0	0	1.724	0	0	0	0	0.792	0	0	0	0	0	0	0.739	1.425	0	0	0	0	1.85	0.96	0	1
Os02g0293800	269	311	330	376.8	354.4	341.7	347	357	375	396.6	410.1	379.2	369.01	382.72	379.35	525.4	399.8	406.1	377.2	379.8	377.7	374.1	342.1	364	394	445	355.3	411	405	421	13

Os02g0654500	463	438	514	406.4	483.6	411.8	449	416	446	408.7	466	562.6	538.82	523.67	510.82	716.5	488.5	524.2	499.8	430.6	540.6	369.7	306.4	360.4	437.3	491.2	616.2	458	427	461	13
Os02g0699900	10.2	9.31	7.54	15.6	11.75	10.38	21.9	14.5	21.4	12.07	19.74	15.99	20.682	19.602	11.088	23.88	8.244	8.288	25.08	16.13	18.97	12.57	15.68	15.17	7.395	27.34	8.772	8.33	12.4	9.16	15
Os02g0153900	200	160	238	182.3	206.1	154.8	46.2	54.4	71.9	162.1	131.6	113.8	150.22	158.69	149.68	119.4	90.69	136.8	178.4	160.5	131.1	70.98	79.1	75.11	105.6	110.3	74.56	143	158	139	8
Os02g0620100	121	103	106	140.4	150.1	159.2	112	109	87.1	173.3	94.29	159	175.25	171.76	146.51	179.1	189.6	179.2	225.7	186.3	225.9	116.8	131.8	118.4	203.9	248.9	236.8	188	162	158	16
Os02g0260200	122	143	142	73.07	77.74	76.99	74.4	76.5	101	185.4	190.8	243.7	237.3	187.62	237.59	143.3	148.4	170.9	58.52	56.44	57.77	94.64	81.95	88.11	245.1	206.5	160.1	179	163	205	3
Os02g0218550	4.08	2.79	2.16	2.463	1.808	6.92	4.7	0.63	2.07	0	4.386	0	1.0885	0.9335	0	0	11.34	3.108	0.929	0.806	0.862	12.57	19.24	15.89	3.169	6.6	0	3.7	5.74	2.5	17
Os02g0701600	1800	2126	2319	2259	2349	2437	3981	3487	4137	2748	2857	2639	3156.7	3217.6	2561.2	2830	2126	2033	3080	2620	3269	2042	2052	2932	2300	2317	2160	1962	2342	2164	15
Os02g0564700	5.1	10.2	6.47	7.389	9.04	8.65	22.7	15.2	11.1	20.69	8.771	10.35	10.885	2.8004	6.3357	0	3.092	2.072	5.574	9.675	11.21	7.394	9.264	6.5	11.62	4.714	19.74	3.7	3.83	2.5	15
Os02g0286933	80.5	86.6	85.1	65.68	83.17	62.28	90.8	73.4	110	55.18	42.76	58.33	65.311	69.075	49.102	47.76	63.89	67.34	58.52	53.21	63.8	221.1	186.7	270.8	62.33	78.26	94.3	88.8	62.2	61.7	9
Os02g0182900	840	865	817	812.8	732.2	718.9	914	880	975	833.7	924.3	791.2	1181	1088.4	1131.7	1111	720.3	773.9	683.7	716	740.6	841.4	807.4	801.7	907.4	831.6	822.3	872	900	968	4
Os02g0832100	6.12	9.31	12.9	14.78	15.37	21.63	11	22.1	9.67	18.97	13.16	19.76	8.7082	13.068	3.1679	0	18.55	19.68	15.79	8.063	18.97	19.22	11.4	21.67	10.56	16.97	13.16	22.2	17.2	17.5	1
Os02g0612100	70.3	65.2	66.8	78.81	93.11	70.07	175	120	159	81.91	76.75	94.08	55.515	60.674	64.149	95.53	48.43	48.69	118	77.4	80.19	90.94	108.3	96.06	57.04	49.97	50.44	62.9	72.7	61.7	18
Os02g0529800	0	0	1.08	1.642	0	0.865	0	2.53	1.38	0	0	0	0	0	0	0	0	2.072	0	1.613	0	0.739	1.425	0	0	0	2.193	1.85	0	0	17
Os02g0749400	96.9	95	125	102.6	98.53	134.1	147	136	168	77.6	59.21	112.9	94.701	89.611	102.16	107.5	89.66	105.7	96.61	106.4	111.2	167.8	112.6	144.4	67.61	69.77	65.79	106	119	117	18
Os02g0580983	0	0	0	0.821	0	0	0	0.63	0.69	0	0	0	0	0	0	0	0	1.036	0	1.613	0.862	0	0	0.722	0	0	0	0	0	0	12
Os02g0563000	0	0	0	137.9	112.1	183.4	1.57	6.96	7.6	6.035	7.675	1.882	5.4426	6.5342	1.5839	0	1.031	1.036	222	161.3	215.6	14.79	13.54	7.945	1.056	8.486	6.579	4.63	10.5	5	10
Os02g0573300	691	596	645	574.7	682.5	590	360	378	368	493.2	470.4	484.5	569.3	601.14	537.74	477.6	765.7	623.7	631.7	574.9	663.9	423.7	453.9	441.3	570.4	570.4	638.1	550	576	593	16
Os02g0776700	1254	1313	1291	1481	1495	1501	1455	1517	1469	1349	1273	1196	1342.1	1218.2	1293.3	1397	1248	1263	1254	1420	1342	1358	1397	1386	1345	1224	1178	1356	1339	1430	12
Os02g0294700	396	376	329	477	464.6	434.3	434	417	435	358.7	385.9	356.6	357.03	394.85	342.92	417.9	434.9	397.8	469.1	492.6	425.1	437.7	466.1	446.3	341.2	417.7	392.5	351	437	353	12
Os02g0709400	1924	2097	2038	1736	1796	1758	1275	1290	1374	1395	1597	1310	1729.7	1569.1	1856.4	2125	1766	1914	1241	1330	1227	2078	1846	1596	1817	1719	1335	2028	1851	2094	8
Os02g0461600	128	114	116	128.9	132.9	144.5	138	99.3	129	135.4	116.2	124.2	146.95	122.28	132.26	214.9	147.4	151.3	144.9	156.4	186.2	117.6	117.6	120.6	144.7	146.1	149.1	146	177	140	6
Os02g0162200	1.02	3.73	1.08	3.284	0.904	8.65	9.39	0	2.07	6.897	3.289	0	3.2656	0.9335	0.792	0	5.153	4.144	0	0.806	5.173	0.739	4.988	5.056	6.338	0.943	2.193	4.63	0.96	1.67	5
Os02g0244600	34.7	31.7	34.5	24.63	13.56	19.9	10.2	12	21.4	44.83	28.51	9.408	28.302	24.27	34.055	226.9	89.66	80.81	15.79	11.29	11.21	16.27	27.08	16.61	58.1	99	140.3	91.6	80.3	46.7	13
Os02g0590200	8.16	13	6.47	10.67	9.04	26.82	7.05	1.27	8.29	10.35	6.578	10.35	10.885	3.7338	9.5036	11.94	12.37	14.5	11.15	20.96	15.52	16.27	29.93	33.95	8.451	33	59.21	12	23.9	3.33	11
Os02g0716800	1.02	2.79	2.16	0	0	3.46	0	0	0	0.862	0	2.822	0	1.8669	0.792	0	7.214	3.108	1.858	0.806	4.311	0	0.713	0	0	2.829	2.193	2.78	0.96	2.5	1
Os02g0459001	0	0	0	0	0	0	0	0	0	0	0	0	0	0	0	0	0	0	0	0	0	0	0	0	1.056	0	0	0	0	0	1
Os02g0732700	1416	1526	1588	1553	1605	1581	1363	1386	1287	1255	1252	1193	1490.2	1391.8	1421.6	1767	1519	1498	1275	1456	1375	1527	1590	1353	1395	1328	1257	1746	1552	1612	8
Os02g0777800	248	257	199	174	94.92	250	128	83.5	122	206.1	180.9	187.2	322.2	444.32	398.36	322.4	471	311.8	156.1	195.1	329.4	545.7	447.5	533	408.8	443.1	447.4	422	342	522	1
Os02g0468400	2200	2697	2367	339.9	324.5	442.9	2783	3313	3360	3087	3455	3305	3962.2	3872	4020.8	4215	3159	3055	432.9	358.8	377.7	2334	2122	2624	3778	3532	3009	2972	2960	3241	3
Os02g0598800	2.04	38.2	9.7	9.031	22.6	40.66	0.78	13.9	5.53	6.035	7.675	0.941	0	28.004	7.9197	11.94	3.092	16.58	33.44	5.644	2.587	16.27	17.82	10.11	2.113	8.486	13.16	6.48	25.8	9.16	2
Os02g0654300	14.3	0.93	4.31	11.49	3.616	25.09	3.91	1.27	4.15	7.76	4.386	15.99	2.177	6.5342	3.1679	0	9.275	5.18	7.432	8.063	7.76	22.92	39.91	23.11	27.47	20.74	35.09	7.4	12.4	8.33	11
Os02g0731200	160	247	258	229.9	259.4	223.2	212	361	232	205.2	196.3	131.7	231.85	227.76	292.24	441.8	218.5	320.1	170.9	225.8	174.2	297.2	335.7	264.3	274.7	203.7	100.9	330	361	451	8
Os02g0805600	868	838	940	1028	1194	1042	1322	1389	1401	912.2	928.6	780.9	905.65	955.85	922.64	1087	827.5	839.2	1064	938.5	971.7	1262	1229	1341	875.7	811.8	907.9	775	737	873	18
Os02g0208600	972	1074	1082	1282	1379	1201	1007	1190	1056	934.6	978	956.8	1172.3	953.05	1069.2	1540	1014	1138	849.1	926.4	894.1	1281	1161	960.6	1105	916.4	706.1	1060	1095	1246	8
Os02g0167100	935	963	1344	643.6	663.5	545	1401	1416	1421	891.5	902.3	888.1	1468.4	1349.8	1237.1	1911	1260	1090	732	728.9	771.7	924.2	776.1	1007	1018	909.8	914.4	1044	1129	1188	13
Os02g0634500	3476	3122	3913	6494	6889	6407	4713	4331	5022	5406	5396	5522	7420.4	5768.7	5554.8	9206	5642	4759	9649	9947	9934	3076	3478	3958	5233	4771	4145	5069	5293	5058	16
Os02g0578500	0	0	0	0	0	0	0	0	0	0	0	1.882	0	0	0	0	0	0	0	0	0	0	0	0	0	0	0	0	0	0	20
Os02g0117900	53	48.4	43.1	71.42	65.09	61.42	58.7	74	61.5	47.42	85.52	54.57	43.541	56.941	60.189	23.88	44.31	48.69	70.6	82.24	72.43	64.33	75.54	69.34	62.33	49.03	98.68	49	64.1	62.5	15
Os02g0567850	82.6	64.3	83	103.4	113	102.1	54	60.7	104	66.39	66.88	93.14	76.196	67.209	69.693	83.59	95.84	102.6	65.03	49.99	76.74	99.08	77.68	65	57.04	77.31	54.82	87.9	95.6	106	8
Os02g0780200	0	0	0	0	0.904	0	0	0	0	0	0	0	0	0	0	0	0	0	0	0	0	0	0	0	0	0	0	0	0	0	2
Os02g0315400	210	121	150	192.1	216	250	285	253	300	268.1	235.7	337.8	180.69	219.36	194.03	119.4	161.8	136.8	268.5	217.7	282.8	191.5	176.7	242.7	146.8	192.3	265.3	162	138	117	15
Os02g0108500	12.2	17.7	20.5	23.81	28.02	18.17	11	12	11.1	16.38	8.771	4.704	8.7082	12.135	13.463	0	15.46	15.54	13.01	16.13	5.173	31.05	19.95	12.28	9.507	12.26	13.16	27.8	9.56	40.8	8
Os02g0717500	43.8	68	49.6	64.04	73.22	83.04	65.8	81	58	32.76	50.43	37.63	44.629	41.072	45.934	83.59	46.37	35.23	67.81	43.54	42.25	76.16	91.22	67.89	38.03	49.97	41.67	30.5	38.3	60.8	17
Os02g0832800	891	992	959	857.9	823.5	920.4	871	966	845	990.7	1027	906	993.82	931.58	1022.4	1325	944	986.3	846.3	794.2	796.7	1049	1063	981.5	1099	970.2	940.8	1046	1072	1101	13
Os02g0740400	1.02	0	0	0	0	0	0	0	0	0	0	0	2.177	0.9335	0	0	1.031	0	0	0	1.724	0	0.713	0	3.169	0	2.193	0	0	0	11
Os02g0148700	161	183	206	264.4	314.6	270.8	321	285	270	193.1	205	246.5	223.15	149.35	199.58	155.2	166.9	173	329.8	359.6	308.7	276.5	235.2	305.5	181.7	193.3	125	153	186	189	12
Os02g0127000	1084	971	1115	808.7	852.4	838.2	1102	1129	1200	1310	1125	1296	1257.2	1255.5	1203	1815	1264	1159	1087	896.6	1001	990	1059	1077	1263	1295	1471	1131	1119	1164	13
Os02g0621700	1586	1496	1442	1288	1474	1431	1648	1492	1779	1552	1419	1627	1317.1	1745.6	1443	1696	1619	1403	1772	1475	1606	1345	1464	1463	1438	1624	1866	1312	1358	1322	15
Os02g0813166	9.18	13	9.7	16.42	22.6	23.36	73.6	38	90.5	26.73	16.45	9.408	13.062	6.5342	11.088	23.88	13.4	10.36	26.01	19.35	18.11	53.24	34.21	42.61	12.68	19.8	8.772	16.7	14.3	9.16	18
Os02g0121700	0	0	1.08	21.35	8.136	12.11	2.35	14.5	6.91	9.484	17.54	0	9.7967	0	0.792	0	0	0	12.08	6.45	2.587	3.697	1.425	0.722	0	0	0	0	0	0	2
Os02g0202900	95.8	164	121	140.4	168.1	126.3	75.9	53.1	43.5	142.3	131.6	128	78.373	103.61	104.54	155.2	113.4	122.3	144	153.2	156.1	70.24	89.08	57.78	140.5	151.8	146.9	158	166	156	16
Os02g0204500	414	421	425	156	186.2	166.1	186	211	170	369	436.4	357.5	372.27	430.32	433.21	453.8	508.1	456.9	171.9	158	160.4	274.3	230.2	217.4	501.8	541.2	418.8	568	569	547	3
Os02g0478900	0	0	2.16	1.642	0.904	0.865	1.57	0.63	0	0	1.096	0	0	0	0	0	2.061	1.036	0.929	5.644	0.862	3.697	7.126	1.444	1.056	0.943	6.579	0.93	1.91	0.83	17
Os02g0612800	2072	1967	2248	1852	1822	2008	2396	2528	2256	1764	1901	2115	1970.2	1915.4	2071.8	2782	2102	2187	1859	2034	1715	2192	2148	2151	2089	2131	2149	2021	2168	2053	6
Os02g0699400	4954	4354	5040	3698	4137	3834	2616	3013	2600	3671	4262	3906	6890.3	7294	6474.3	7917	6309	5610	5005	5013	5035	2163	2335	2472	4775	4515	4572	6415	5529	5943	13
Os02g0548700	37.7	22.4	17.2	4.105	1.808	0.865	4.7	4.43	10.4	4.311	2.193	6.586	3.2656	6.5342	9.5036	0	11.34	14.5	2.787	1.613	3.449	5.915	3.563	12.28	2.113	5.657	6.579	2.78	1.91	0	14
Os02g0799400	4.08	2.79	2.16	9.031	11.75	7.785	2.35	3.16	3.46	6.035	4.386	0	1.0885	0.9335	3.1679	11.94	1.031	0	9.29	3.225	0	8.133	9.264	2.889	1.056	0	0	8.33	2.87	2.5	2
Os02g0627700	5.1	3.73	3.23	2.463	0.904	0	0.78	0	2.76	2.587	4.386	4.704	5.4426	7.4676	1.5839	11.94	10.31	11.4	2.787	3.225	3.449	0	0	3.611	11.62	3.771	8.772	4.63	3.83	4.17	13
Os02g0623400	1.02	0	0	1.642	1.808	0.865	2.35	0	2.07	0	0	0	2.177	0.9335	0	0	0	0	0	0	0	1.479	0	0	0	0	0	0	0	0	2
Os02g0192500	5.1	4.66	0	46.8	37.97	70.07	48.5	17.1	22.8	0.862	5.482	6.586	0	0.9335	0.792	0	2.061	2.072	74.32	50.8	81.91	18.48	38.48	75.84	2.113	2.829	21.93	3.7	13.4	7.5	10
Os02g0628900	18.4	21.4	9.7	12.31	19.89	10.38	23.5	12.7	7.6	27.59	20.83	12.23	13.062	10.268	17.423	11.94	4.122	20.72	7.432	8.869	10.35	21.44	29.22	33.95	13.73	18.86	19.74	19.4	23.9	19.2	9
Os02g0606600	3.06	1.86	1.08	0.821	0	1.73	0.78	2.53	2.07	0	0	4.704	0	0	0	0	1.031	2.072	0.929	0.806	1.724	2.958	0.713	0.722	0	0.943	2.193	0	1.91	1.67	19
Os02g0232300	0	0	0	0	0	0.865	0	0	0	0	0	0	0	0	0	0	0	0	0	0	0	0	0	0	0	0	0	0	0	0	2
Os02g0752200	0	0	0	0	0	0	0	0	0.69	0	0	0	1.0885	0	0	0	0	0	1.858	0	2.587	0	0	0.722	0	0	0	0	0	0	16
Os02g0827100	443	412	454	228.2	236.8	199.8	338	251	361	411.3	421	496.8	334.18	308.04	277.98	346.3	415.3	398.9	243.4	266.9	278.5	403	270.1	377	365.5	417.7	414.5	293	339	319	20
Os02g0582300	98.9	91.3	83	171.6	211.5	171.3	96.3	113	101	61.22	59.21	55.51	50.072	67.209	70.485	35.82	78.32	75.63	127.3	160.5	151.8	139	168.9	122.1	66.55	81.08	85.52	94.4	67.9	70	10
Os02g0270100	0	0	0	0.821	2.712	0	0	2.53	0	0	0	0	0	0	0	0	0	0	0	0	0	0.739	9.264	0.722	0	0	0	0	0	0	17
Os02g0494600	132	123	131	169.9	185.3	188.6	245	250	275	111.2	104.2	162.8	96.878	109.21	83.948	191.1	155.6	119.1	302.8	239.5	213.8	155.3	188.8	123.8	88.73	99	103.1	99.9	107	105	12
Os02g0477300	2287	2031	2159	2107	2071	2245	1852	1757	1837	2337	2226	2990	2402.4	2628.6	2051.2	2293	2300	2507	2512	2596	2685	2311	1884	1709	2276	2633	2807	2125	2347	1905	20
Os02g0169900	1552	1406	1633	1961	2019	2188	2362	2161	2394	1565	1649	2291	1962.6	2000.4	1660	2293	1902	1722	2738	2137	3116	1582	1397	1996	1769	1890	2070	1400	1491	1593	15
Os02g0750400	232	318	327	527.9	659	503.5	460	534	444	252.6	384.8	180.1	330.91	280.04	233.63	226.9	278.2	345	506.3	480.5	557.9	342.3	296.5	403.7	236.6	237.6	212.7	178	207	195	12
Os02g0177400	8.16	12.1	9.7	13.96	15.37	14.71	7.05	21.5	15.9	12.07	18.64	13.17	14.151	9.3345	13.463	11.94	18.55	16.58	16.72	24.99	14.66	23.66	21.38	24.56	19.01	25.46	26.32	15.7	15.3	20.8	9
Os02g0637200	4.08	14.9	8.62	13.14	10.85	13.84	21.9	24.7	34.6	22.42	5.482	9.408	8.7082	5.6007	1.5839	0	1.031	4.144	7.432	8.063	5.173	14.05	12.11	19.5	3.169	2.829	24.12	3.7	2.87	5	18
Os02g0220500	1797	1667	1619	1959	2094	2190	1524	1561	1755	1507	1495	1778	1719.9	1841.7	1446.9	2006	1496	1679	1611	1473	1736	1853	1767	1684	1479	1711	1702	1296	1385	1328	2
Os02g0139200	178	181	179	433.5	459.2	424.7	178	213	179	177.6	146.9	136.4	173.07	153.09	159.98	155.2	140.2	164.7	330.7	311.2	361.3	230.7	254.4	195	148.9	152.7	142.5	170	144	169	10
Os02g0770300	435	413	478	394.9	487.2	469.7	363	477	384	336.3	382.6	348.1	403.84	420.99	437.17	585.1	465.8	474.5	354.9	433	402.7	563.4	507.4	402.3	462.7	418.6	344.3	500	523	474	8
Os02g0569000	7.14	10.2	10.8	7.389	2.712	4.325	11.7	1.27	3.46	9.484	12.06	26.34	11.974	14.002	11.879	0	2.061	3.108	5.574	5.644	4.311	8.133	4.276	7.945	17.96	14.14	24.12	2.78	10.5	10	20
Os02g0270200	0	0	0	0	1.808	1.73	0	0.63	0	0	0	0	0	0	0	0	0	0	0	0	0	2.958	0.713	0.722	0	0	0	0	0	0	9
Os02g0307900	0	2.79	0	2.463	0.904	0	0.78	0.63	1.38	0	0	0	0	0	0	11.94	0	0	0	0.806	3.449	0.739	1.425	0	3.169	0	0	1.85	0	0	6
Os02g0486400	724	649	631	490.9	549.6	556.2	299	334	336	513.9	554.8	489.2	555.15	578.74	564.67	620.9	714.2	720	395.7	496.7	466.5	666.9	424.7	430.5	669.7	637.4	688.6	756	668	726	3
Os02g0142750	1.02	0	1.08	0	0	0	0.78	0	0	0	0	0	0	0	0	0	3.092	0	0	0	0	0	0	0	0	0	0	0	0	0	14
Os02g0718100	2.04	3.73	5.39	19.7	31.64	33.74	54	40.5	32.5	43.11	13.16	12.23	3.2656	9.3345	7.9197	0	3.092	5.18	48.31	25.8	22.42	8.873	31.36	10.11	9.507	0	4.386	4.63	15.3	13.3	12
Os02g0700700	18.4	11.2	8.62	8.21	18.08	16.44	13.3	7.59	13.8	36.21	28.51	31.05	25.036	26.137	27.719	23.88	24.73	18.65	26.94	23.38	13.8	4.436	8.552	5.778	41.2	41.49	21.93	47.2	27.7	20.8	3
Os02g0202950	38.7	21.4	32.3	25.45	21.7	25.09	4.7	8.86	7.6	25	32.89	51.75	43.541	50.406	34.055	107.5	46.37	29.01	22.29	53.21	35.35	9.612	10.69	10.83	39.09	34.89	32.89	44.4	38.3	67.5	13
Os02g0815900	1.02	6.52	5.39	0.821	2.712	4.325	3.13	1.9	1.38	3.449	1.096	1.882	6.5311	3.7338	1.5839	11.94	1.031	0	5.574	1.613	9.484	4.436	1.425	1.444	5.282	5.657	8.772	0.93	5.74	5	6
Os02g0724600	555	428	525	469.6	497.2	481	498	518	464	563.9	548.2	729.1	632.43	648.75	617.73	453.8	549.3	563.6	574.1	545	469.9	388.2	478.2	384.2	626.4	687.3	600.9	604	591	600	3
Os02g0583500	0	0	0	0	0	0	0	0	0	0	0	0	0	0	0	0	0	0	0	1.613	0	0	0	0	0	0	0	0	0	0	16
Os02g0654000	25.5	29.8	17.2	17.24	8.136	6.92	30.5	11.4	11.1	21.55	23.02	24.46	11.974	7.4676	14.255	47.76	15.46	11.4	11.15	23.38	12.07	12.57	13.54	16.61	14.79	17.91	19.74	24.1	18.2	25	6
Os02g0603700	0	3.73	0	0.821	0.904	1.73	0.78	0	1.38	1.724	1.096	0	0	0	3.1679	0	0	1.036	0.929	0	0	8.873	6.414	7.945	1.056	0	0	0.93	0.96	0.83	9
Os02g0646400	498	489	468	472.1	461.9	507.8	431	328	375	483.7	432	519.3	448.47	498.46	510.03	716.5	530.7	518	520.2	550.7	639.8	539.8	469.6	447.1	483.8	492.2	524.1	525	515	542	13
Os02g0653900	1253	1163	942	268.5	187.1	306.2	215	233	264	619.9	592.1	704.7	414.73	425.65	445.08	704.5	886.3	768.7	388.3	383.8	508.7	419.2	444.7	521.5	696.1	626	587.7	649	524	780	14
Os02g0120800	14.3	9.31	10.8	3.284	1.808	8.65	2.35	1.27	0.69	7.76	3.289	5.645	5.4426	13.068	7.9197	0	19.58	10.36	1.858	3.225	6.036	4.436	4.276	1.444	12.68	26.4	19.74	11.1	14.3	16.7	3
Os02g0668500	67.3	139	84.1	474.5	539.7	533.7	1779	2211	1888	244.9	248.9	204.2	167.63	134.42	229.67	131.4	48.43	94.28	408.7	350.7	310.4	1465	1527	1432	107.7	83.91	81.14	96.2	93.7	94.1	18
Os02g0739900	29.6	18.6	15.1	11.49	13.56	8.65	25.1	20.9	19.3	11.21	14.25	31.05	10.885	24.27	8.7116	35.82	31.95	29.01	26.94	24.19	43.11	5.176	9.977	13	20.07	24.51	30.7	16.7	23	12.5	16
Os02g0753900	5.1	5.59	8.62	0	2.712	0	5.48	3.16	6.91	13.79	13.16	22.58	11.974	4.6673	7.9197	0	8.244	5.18	0	1.613	2.587	2.218	0.713	2.889	7.395	12.26	6.579	7.4	3.83	3.33	20
Os02g0175600	663	442	542	647.7	660.8	629.8	983	765	1075	776	680.9	1237	780.47	617.01	551.21	955.3	499.8	503.5	859.3	815.2	822.6	592.2	613.6	764.9	614.8	805.2	782.9	391	515	383	15
Os02g0460200	####	####	8339	2124	1660	1682	1808	855	1381	11170	11018	10478	5381.6	6838.5	7651.2	4203	6724	5915	2201	2061	2201	846.6	825.9	1348	6547	6732	8096	5675	3345	5906	3
Os02g0492600	1.02	0	0	3.284	0.904	0.865	2.35	0	2.07	1.724	1.096	0	0	5.6007	3.1679	0	0	0	1.858	0	1.724	0.739	0.713	0.722	0	0	0	0	0	0	4
Os02g0244300	453	554	454	488.5	519.8	511.2	401	374	357	421.6	494.5	374.4	348.33	445.26	441.13	513.5	501.9	537.7	408.7	494.3	514.7	493.2	526.6	388.6	466.9	575.1	541.7	546	500	521	8
Os02g0197900	79.5	83.8	100	74.71	61.47	55.36	140	140	132	96.56	117.3	132.7	129.53	109.21	123.55	83.59	96.87	103.6	91.04	95.14	119.8	116.8	77.68	101.1	90.85	106.5	114	83.3	92.8	73.3	5
Os02g0462000	35.7	37.3	33.4	75.53	62.37	96.89	77.5	62.6	66.3	8.622	14.25	22.58	13.062	18.669	19.799	11.94	29.89	30.05	46.45	45.15	72.43	109.4	64.85	84.5	17.96	38.66	15.35	36.1	29.6	14.2	12
Os02g0648900	15.3	14	10.8	4.105	16.27	19.03	21.1	15.8	22.8	28.45	19.74	13.17	18.505	27.07	22.967	11.94	13.4	18.65	22.29	15.32	14.66	11.83	7.126	21.67	13.73	16.97	10.96	19.4	14.3	14.2	7
Os02g0656300	86.7	97.8	92.7	101.8	137.4	102.9	57.2	57.6	62.2	83.63	44.95	47.04	85.993	56.941	106.12	131.4	45.34	83.92	76.17	75.79	81.91	79.11	71.26	98.95	87.68	82.97	65.79	96.2	77.5	92.5	8
Os02g0124300	0	0	0	0	0	0	0	0	0	0	0	0	0	0	0	0	0	0	0	0	0	0	0.713	0	0	0	0	0	0	0	17
Os02g0826600	0	0	0	0	0	0	0	0	0	0	0	0	0	0	0	95.53	55.65	55.95	81.75	91.11	77.6	72.46	63.42	91.73	71.83	62.23	74.56	37	47.8	44.2	9
Os02g0633700	0	0	0	0	0	0	0	1.9	0	0	0	0	0	0	0	0	0	0	0	0	0	0	0	0	0	0	0	0	0	0	18
Os02g0514150	0	0	1.08	3.284	0.904	5.19	6.26	5.69	1.38	0	0	0.941	1.0885	0.9335	0.792	0	1.031	0	8.361	3.225	5.173	2.958	1.425	3.611	0	0	0	0	0	0	12
Os02g0823600	742	819	907	845.6	820.8	782.9	983	848	926	1036	958.2	1096	808.77	919.45	856.12	823.9	749.2	738.7	881.6	823.2	844.1	859.9	906.5	872.5	902.1	914.6	901.3	843	843	843	20
Os02g0741800	12.2	4.66	2.16	3.284	0	8.65	10.2	5.06	5.53	1.724	3.289	1.882	2.177	6.5342	2.3759	11.94	6.183	6.216	11.15	4.031	6.898	3.697	1.425	9.389	4.225	3.771	17.54	16.7	8.61	8.33	11
Os02g0284500	66.3	53.1	59.3	82.1	66.89	69.2	62.6	43	50.4	61.22	50.43	99.73	76.196	71.876	66.525	35.82	62.86	61.13	70.6	66.12	47.42	53.24	59.15	32.5	73.95	54.68	70.17	56.4	59.3	48.3	20
Os02g0257400	921	544	677	321	217.9	260.4	562	520	545	736.3	637	1967	696.65	867.18	618.53	620.9	1213	1126	1065	774.8	1320	229.9	189.6	437.7	1018	1774	2180	562	718	505	20
Os02g0636700	307	261	281	311.1	308.3	224	100	157	122	283.7	239	256.8	248.18	334.18	380.14	262.7	286.5	227.9	248	264.5	256.1	123.5	154.6	117	326.4	269.7	366.2	379	343	317	3
Os02g0687500	871	771	930	620.7	608.4	497.4	547	739	644	734.6	759.8	703.7	1094	834.51	909.18	1170	1192	982.2	564.8	541.8	622.5	489.5	511	486.8	888.4	740.1	723.7	895	869	959	13
Os02g0178200	23.4	12.1	12.9	9.031	14.46	10.38	18.8	17.1	15.9	7.76	20.83	27.28	38.098	20.536	19.007	0	17.52	25.9	26.01	14.51	24.14	5.915	7.839	10.11	19.01	19.8	21.93	18.5	23.9	20.8	4
Os02g0650300	0	0	0	0	0	0	0	0	0	0	0	0	0	0	0	0	0	0	0	0	2.587	0	0	0	0	0	0	0	0	0	16
Os02g0559300	9.18	7.45	4.31	24.63	11.75	11.25	12.5	8.86	4.84	5.173	4.386	5.645	1.0885	2.8004	1.5839	0	0	12.43	7.432	11.29	6.036	17.75	11.4	17.33	0	6.6	0	10.2	11.5	7.5	2
Os02g0777100	1682	1786	1800	977	947.4	985.3	1086	1353	1157	1648	1909	1614	2001.8	1868.8	2193.7	2591	1809	1935	842.6	975.6	896.7	1378	1370	1284	1964	1967	1732	1996	1928	2070	13

Os02g0321600	1.02	1.86	0	4.926	0.904	1.73	3.91	5.06	1.38	4.311	2.193	5.645	1.0885	3.7338	0	0	3.092	0	7.432	5.644	4.311	1.479	1.425	2.167	1.056	6.6	0	1.85	0	5	15
Os02g0100800	22.4	21.4	18.3	30.38	21.7	26.82	57.2	43	30.4	38.8	20.83	21.64	14.151	21.469	24.551	47.76	24.73	11.4	46.45	24.19	52.6	31.05	29.93	47.67	26.41	38.66	30.7	25	18.2	29.2	18
Os02g0213450	37.7	27.9	44.2	13.96	23.5	31.14	25.1	32.9	23.5	18.11	23.02	31.99	45.718	37.338	44.35	0	36.07	38.33	16.72	17.74	28.45	31.79	27.79	36.83	36.97	50.91	30.7	38.9	54.5	55	1
Os02g0297600	301	366	317	316.1	347.1	288.9	238	250	254	173.3	196.3	123.2	195.93	207.23	213.83	143.3	231.9	250.7	169.1	201.6	175.9	374.9	406.9	301.2	211.3	200.8	151.3	246	274	292	17
Os02g0123100	141	123	121	85.38	79.55	59.69	149	133	140	112.9	85.52	112	89.259	95.212	65.733	95.53	99.96	92.21	72.46	63.7	55.18	71.72	81.24	86.67	54.93	68.83	67.98	65.7	78.4	67.5	5
Os02g0293400	90.7	106	78.7	120.7	121.1	109	86.1	138	91.2	56.9	67.98	46.1	78.373	50.406	89.492	119.4	91.72	93.24	65.03	59.66	53.46	127.9	141.1	106.9	68.66	45.26	65.79	69.4	59.3	94.1	17
Os02g0245301	2.04	0.93	1.08	0	0	1.73	0	0	0	1.724	1.096	0	0	0	1.5839	0	1.031	0	0	0	0.862	0.739	0.713	0	4.225	1.886	2.193	4.63	0.96	0.83	1
Os02g0634900	1760	1854	1817	2157	2397	2222	3628	3090	3551	2137	1992	1938	1955	2379.4	2010.8	2734	1860	1677	2439	1971	2364	2524	2675	2653	1970	1841	2377	1721	1760	1727	18
Os02g0101300	18.4	12.1	12.9	8.21	10.85	4.325	49.3	25.3	43.5	22.42	16.45	59.27	27.213	16.802	13.463	23.88	21.64	37.3	13.93	4.031	10.35	28.84	16.39	30.33	35.92	36.77	13.16	17.6	23.9	13.3	5
Os02g0655200	119	55	83	22.99	12.66	11.25	7.05	15.8	25.6	43.11	59.21	61.15	42.452	19.602	31.679	47.76	47.4	27.97	22.29	14.51	4.311	19.96	28.51	28.89	115.1	92.4	81.14	85.1	44.9	69.1	3
Os02g0714600	443	394	367	917.8	1019	993.1	611	530	679	353.5	322.3	434.7	503.98	498.46	327.08	788.1	373.1	418.6	1085	885.3	1127	514.6	488.9	542.4	278.9	318.7	359.6	299	319	299	10
Os02g0601600	114	83.8	76.5	146.1	144.6	154.8	121	145	141	128.5	153.5	150.5	144.77	132.55	118.79	238.8	122.6	90.14	150.5	144.3	136.2	128.7	123.3	139.4	154.2	164.1	168.9	122	106	119	6
Os02g0199600	72.4	101	81.9	179.8	154.6	179.9	122	113	133	138.8	117.3	157.1	167.63	135.35	133.84	95.53	117.5	136.8	208.1	207.2	249.2	122	124.7	184.2	144.7	142.4	116.2	130	150	107	16
Os02g0131000	7.14	17.7	9.7	33.66	37.06	19.9	32.1	26.6	39.4	25.87	17.54	24.46	42.452	24.27	13.463	71.65	14.43	19.68	19.51	13.71	22.42	31.79	22.8	28.89	32.75	23.57	8.772	23.1	15.3	25.8	6
Os02g0241300	552	524	533	648.6	610.2	674.7	296	319	330	465.6	458.3	603.1	425.61	467.66	491.81	776.2	529.7	578.1	617.8	616	558.7	573	554.4	437	517.6	620.4	557	548	571	507	16
Os02g0620400	390	233	287	72.25	65.09	64.01	63.4	39.8	56	196.6	144.7	224.9	137.15	203.49	169.48	250.8	409.1	298.4	151.4	129	174.2	51.76	58.44	86.67	264.1	244.2	359.6	208	174	169	3
Os02g0811600	0	0	1.08	0	3.616	0	0	0	1.38	0	0	1.882	0	0	0	0	0	3.108	0	0	0	0	0	0	0	0	0	0	0	0	2
Os02g0289500	3.06	6.52	1.08	5.747	4.52	9.516	3.13	5.06	8.98	3.449	4.386	4.704	2.177	3.7338	1.5839	0	4.122	4.144	5.574	3.225	10.35	8.133	13.54	5.778	4.225	5.657	8.772	2.78	1.91	1.67	17
Os02g0299300	90.7	47.5	37.7	576.3	666.2	543.3	22.7	27.8	22.8	12.93	6.578	24.46	22.859	30.804	15.839	107.5	76.26	66.31	436.6	548.3	644.1	116.1	133.3	99.67	11.62	30.17	19.74	25.9	25.8	31.7	10
Os02g0725000	201	182	135	55.01	61.47	88.21	16.4	12.7	9.67	124.2	117.3	114.8	82.728	132.55	102.96	286.6	245.3	176.1	64.1	117.7	140.5	58.41	86.23	101.8	222.9	332.8	324.6	308	230	235	13
Os02g0318100	0	0	2.16	28.73	45.2	22.49	2.35	3.8	2.76	0.862	0	0	0	4.6673	4.7518	0	1.031	2.072	13.01	26.61	34.49	2.958	10.69	5.056	2.113	0	2.193	3.7	0.96	3.33	10
Os02g0799600	145	114	119	188	185.3	216.3	79.9	91.1	98.8	125.9	171	112	142.6	106.41	152.85	167.2	122.6	125.4	214.6	160.5	211.2	95.38	126.8	88.11	138.4	102.8	92.1	130	89.9	137	16
Os02g0135900	708	657	849	443.3	428.5	401.4	305	455	351	600.9	692.9	599.3	677.06	883.98	818.1	644.8	787.3	682.7	381.8	268.5	349.2	275.1	278.6	308.4	661.3	685.4	543.8	664	720	684	3
Os02g0633100	960	958	913	977.8	1002	1101	586	679	550	697.5	760.9	647.3	839.25	870.91	830.77	991.1	818.2	850.6	754.3	828.9	772.5	768.2	759	646.4	705.7	668.5	695.2	990	937	1004	8
Os02g0786000	735	906	853	695.4	750.3	775.1	538	523	527	571.6	604.1	510.9	589.98	617.94	623.28	1373	737.9	747	551.8	576.5	634.6	637.4	629.3	533.7	599	544	644.7	779	813	786	13
Os02g0534000	8.16	6.52	9.7	4.926	8.136	6.92	9.39	12.7	13.8	10.35	5.482	10.35	3.2656	5.6007	4.7518	47.76	8.244	9.324	9.29	6.45	29.32	5.915	18.53	20.22	4.225	5.657	13.16	5.55	8.61	11.7	6
Os02g0502500	356	341	316	328.4	347.1	330.4	305	280	256	288.8	325.6	299.2	342.88	356.58	381.73	250.8	332.9	309.8	236	255.6	250.9	252.9	297.2	265.1	352.8	335.7	302.6	334	343	374	4
Os02g0767300	0	0	0	0	0	0	0	0	0	0	0	0	0	0	0	0	0	0	0	0	0	0	0	0.722	0	0	0	0	0	0	9
Os02g0121800	306	265	270	287.3	301.9	265.6	342	322	323	328.5	309.2	365	223.15	280.04	249.47	262.7	237	262.1	413.4	276.6	392.3	227	220.9	262.2	228.2	272.5	258.8	179	239	197	15
Os02g0714100	140	156	139	76.35	62.37	56.23	184	153	189	169.9	139.2	151.5	127.36	152.15	140.18	143.3	82.44	79.77	55.74	54.83	43.97	120.5	132.6	151.7	120.4	113.1	98.68	102	75.5	85	5
Os02g0543000	2655	2887	2606	828.4	941	751.7	1146	1416	1034	1760	1855	2215	1085.3	1219.1	1340.8	1337	1535	1375	760.8	719.2	445.8	917.6	1270	930.3	1622	1237	1316	1513	1022	1126	14
Os02g0636600	34.7	37.3	46.3	85.38	80.45	99.48	0.78	4.43	1.38	26.73	46.05	5.645	48.983	96.146	102.16	95.53	47.4	25.9	51.09	88.69	88.81	12.57	2.851	1.444	42.25	30.17	41.67	54.6	43	86.6	16
Os02g0777001	6.12	7.45	1.08	3.284	0	6.92	4.7	0.63	6.91	7.76	10.96	15.05	7.6196	9.3345	17.423	0	12.37	3.108	3.716	1.613	6.036	5.176	2.851	5.778	20.07	13.2	10.96	10.2	3.83	12.5	3
Os02g0674400	0	0	0	12.31	24.41	6.92	0	0	0	0	0	0	0	0	0	0	0	0	12.08	16.93	17.24	0.739	0	0	0	0	0	0	0	0	10
Os02g0602600	155	112	161	116.6	127.5	106.4	97.1	101	127	111.2	136	114.8	132.8	126.02	144.93	167.2	135	144	105.9	120.9	122.4	108.7	95.49	117	154.2	160.3	114	164	166	171	13
Os02g0781400	3.06	0.93	4.31	26.27	27.12	12.11	33.7	22.8	28.3	5.173	6.578	2.822	3.2656	0	0	11.94	3.092	1.036	17.65	19.35	22.42	14.79	4.988	26.72	3.169	2.829	0	1.85	1.91	0	12
Os02g0294000	0	0	0	0	0	0	0	0	0	0	0	0	0	0	0	0	0	0	0	0	0	0	2.851	0	0	0	0	0	0	0	17
Os02g0800500	41.8	119	58.2	36.12	208.8	42.39	29.7	32.9	48.4	112.9	109.6	62.09	53.337	308.04	72.861	143.3	56.68	46.62	41.8	103.2	35.35	70.24	62	33.95	94.02	66	74.56	194	73.6	75	4
Os02g0821100	0	0	0	0	0	0	0	0	0	0	0	0	0	0	0	0	0	0	0	0	0	0.739	0	0.722	2.113	0	0	0	0.96	0	1
Os02g0224800	29.6	33.5	34.5	55.83	75.03	51.04	245	175	165	43.11	54.82	65.86	37.01	37.338	44.35	11.94	26.79	34.19	108.7	102.4	93.98	99.82	105.5	156	47.54	51.86	89.91	24.1	38.3	35	18
Os02g0150600	196	246	228	307	319.1	258.6	287	334	310	268.1	256.6	239	311.32	247.36	246.3	179.1	225.7	242.4	279.6	329	254.4	369	330.7	284.6	289.4	243.3	223.7	266	253	272	12
Os02g0799100	11.2	23.3	22.6	22.17	37.97	22.49	15.7	17.1	15.9	15.52	21.93	21.64	18.505	18.669	22.967	11.94	24.73	25.9	17.65	23.38	11.21	24.4	29.22	18.78	23.24	17.91	17.54	34.2	16.3	24.2	8
Os02g0172800	0	0	1.08	0	0	0	0	1.9	2.07	0	1.096	0	1.0885	0.9335	0	0	0	1.036	0.929	0	0.862	1.479	4.988	0.722	1.056	1.886	0	0	1.91	1.67	17
Os02g0245800	517	510	377	114.1	84.97	106.4	29	39.8	26.3	249.2	286.2	191.9	180.69	336.04	349.26	250.8	462.7	377.1	166.3	183.8	179.3	68.76	82.67	85.22	376.1	372.4	350.9	501	400	535	3
Os02g0201401	27.5	26.1	33.4	27.09	37.06	26.82	49.3	62.6	54.6	51.73	46.05	72.44	41.364	45.739	32.471	23.88	58.74	32.12	44.59	40.31	57.77	48.06	52.02	55.61	39.09	34.89	52.63	31.5	37.3	31.7	15
Os02g0725100	1719	1766	1653	1348	1498	1337	1252	1283	1319	1580	1537	1388	1437.9	1857.6	1511.9	3057	1861	1714	1356	1275	1457	1281	1442	1254	1797	1798	1897	1835	1906	1716	13
Os02g0158500	20.4	14	28	34.48	28.93	19.9	45.4	53.8	38	31.04	30.7	17.88	51.16	17.736	36.43	83.59	24.73	18.65	26.94	45.96	18.11	35.49	29.22	23.11	36.97	20.74	30.7	22.2	34.4	38.3	6
Os02g0652300	532	540	515	505.7	446.6	485.3	455	420	410	555.2	584.4	604	562.76	603.94	610.61	620.9	628.6	601.9	568.5	628.9	591.5	417	424	411	639.1	684.5	594.3	616	542	639	3
Os02g0121900	38.7	46.6	38.8	77.17	117.5	107.3	53.2	57.6	84.3	32.76	62.49	41.4	29.39	32.671	35.638	71.65	46.37	44.55	99.4	70.15	99.15	82.81	66.28	69.34	41.2	36.77	37.28	62.9	49.7	53.3	10
Os02g0100300	297	236	262	186.4	168.1	212.8	181	154	160	400.9	323.4	379.2	386.42	391.12	349.26	394.1	253.5	225.9	305.6	304	332	108.7	116.9	136.5	283.1	318.7	416.7	229	207	213	3
Os02g0109400	20.4	17.7	18.3	23.81	19.89	22.49	18.8	14.5	34.6	13.79	14.25	14.11	18.505	16.802	11.088	11.94	14.43	12.43	19.51	22.58	16.38	31.79	37.06	39.72	27.47	23.57	17.54	23.1	36.3	24.2	17
Os02g0178100	3533	3336	3207	2194	2119	2027	406	660	630	4945	6040	4870	5143.3	4591.6	5170.7	4669	4349	4099	2425	2908	2796	846.6	1149	855.9	5571	5437	4427	4876	3849	4544	3
Os02g0668100	885	897	1033	1104	1044	1021	923	913	870	1075	1146	1248	1275.7	1086.5	1074.7	1003	905.8	957.3	1421	1515	1348	622.6	612.9	742.5	1152	1036	938.6	838	935	894	16
Os02g0175950	130	138	94.8	1.642	4.52	4.325	31.3	17.7	65.6	106	75.65	128	117.56	97.079	98.204	35.82	170	122.3	12.08	20.96	21.56	198.9	62	163.2	99.3	117.9	87.72	87.9	94.7	121	1
Os02g0129600	221	218	207	233.2	229.6	205	158	188	196	237.1	235.7	186.3	209	231.5	248.68	262.7	192.7	187.5	200.7	250.8	261.3	232.2	248.7	187.8	202.8	208.4	225.9	273	238	260	8
Os02g0713400	520	506	554	427.7	457.4	453.3	744	620	844	622.5	588.8	572	437.59	582.47	544.87	668.7	510.1	483.8	484.9	462.8	450.9	739.4	706.9	811.8	574.7	586.4	682	551	517	474	5
Os02g0793900	1076	1186	1198	1153	1203	1012	1096	1309	1160	1129	1156	1071	1310.6	1243.4	1252.1	1946	1189	1240	1069	1102	989	1143	1168	1089	1307	1088	1002	1378	1287	1429	13
Os02g0762100	386	455	486	935.1	1118	705.9	1852	2064	1817	500.1	549.3	493.9	612.84	414.45	452.21	728.4	423.5	662	925.2	807.9	839.8	979.7	1016	1208	508.1	245.1	258.8	344	416	577	18
Os02g0723700	398	420	394	381.8	335.4	364.2	415	425	408	379.4	502.1	396.1	476.77	459.26	454.59	609	451.4	359.5	423.6	475.7	450.1	380.8	389.1	405.9	438.4	463.9	517.5	470	439	427	13
Os02g0458900	0	0	0	0	0	0	0	0	0	0	0	0	0	0	0	0	1.031	0	0.929	0.806	0	0	0	0	0	0	0	0	0.96	0	16
Os02g0147600	29.6	32.6	37.7	45.15	50.62	49.31	75.9	62.6	65	31.9	62.49	47.98	45.718	48.539	47.518	35.82	28.85	24.86	61.31	50.8	70.7	31.79	47.75	63.56	31.69	36.77	26.32	25	30.6	23.3	15
Os02g0573400	644	637	696	627.2	601.1	576.1	471	540	479	593.2	547.1	449.7	674.88	657.15	725.44	704.5	734.8	734.6	603.8	578.1	510.4	717.9	636.4	637.7	577.8	560	532.9	813	699	899	8
Os02g0828566	42.8	48.4	39.9	45.97	71.41	51.04	32.1	46.8	32.5	55.18	52.63	49.86	47.895	46.673	34.055	47.76	38.13	47.66	43.66	41.93	48.28	34.01	43.47	48.39	47.54	40.54	30.7	38.9	49.7	49.2	2
Os02g0751900	0	0.93	0	1.642	0	1.73	0	1.27	0	0	0	0	0	0	0	0	0	1.036	0.929	2.419	3.449	0	0	0	0	0	0	0	0	0	16
Os02g0169000	775	712	739	1085	1199	1110	1051	1200	1131	796.7	953.9	916.4	1205	877.44	948.78	1027	720.3	756.3	1188	1339	1360	782.3	795.3	965.6	812.3	717.5	831.1	675	681	652	10
Os02g0218400	51	62.4	51.7	70.6	56.95	63.15	11	24	15.2	26.73	38.37	31.05	44.629	36.405	45.934	71.65	57.71	71.49	18.58	48.38	26.73	52.5	39.19	20.95	59.16	57.51	28.51	70.3	61.2	86.6	8
Os02g0598300	364	449	367	492.6	505.3	431.7	165	219	219	200	245.6	121.4	296.08	230.56	302.53	394.1	263.8	332.6	174.6	228.2	177.6	431.1	455.4	320	277.8	236.7	212.7	385	382	399	8
Os02g0571900	2.04	0.93	3.23	4.105	0.904	0	3.91	4.43	2.76	2.587	2.193	5.645	3.2656	2.8004	4.7518	0	1.031	0	1.858	0.806	0	0.739	0.713	0	2.113	0.943	0	5.55	0.96	1.67	4
Os02g0673100	1.02	1.86	0	0	0	0	0	0	0	5.173	0	5.645	0	0	0	0	0	0	0	0	0	0	0	0	0	0	0	0	0	0	20
Os02g0529400	877	783	886	628	683.4	660.9	891	960	1069	1002	1209	1423	1297.5	1309.6	1161	1528	1261	1006	956.8	982.1	942.4	694.3	748.3	788	1267	1229	1274	991	873	1036	3
Os02g0775300	201	166	209	186.4	161.8	212.8	131	157	104	195.7	184.2	271	163.28	244.56	221.75	477.6	287.5	272.5	318.6	229	319.9	128.7	117.6	122.8	398.2	292.3	403.5	251	179	221	13
Os02g0762300	285	253	252	257	264	314	278	308	311	233.7	294.9	302	318.94	275.37	321.54	382.1	244.2	325.3	351.1	365.2	344.9	396.3	320.7	296.8	337	363.9	337.7	301	356	327	6
Os02g0197400	91.8	78.2	65.7	71.42	63.28	71.8	155	123	133	108.6	108.5	150.5	89.259	90.545	79.989	107.5	110.3	132.6	145.8	118.5	113	90.94	94.78	119.9	96.13	109.4	127.2	66.6	93.7	83.3	15
Os02g0795800	3.06	6.52	1.08	10.67	8.136	6.92	5.48	1.27	4.15	4.311	6.578	0.941	2.177	3.7338	3.1679	0	1.031	3.108	2.787	1.613	0.862	0.739	1.425	2.889	1.056	0	0	5.55	3.83	0	2
Os02g0670400	470	398	429	348.9	301.9	436.8	358	210	242	446.6	338.8	550.4	342.88	442.46	394.4	823.9	563.7	508.7	445	393.5	491.5	246.2	320.7	290.3	409.9	512	576.7	486	442	360	13
Os02g0105450	2.04	1.86	2.16	1.642	1.808	0.865	3.91	0.63	2.07	1.724	3.289	3.763	3.2656	3.7338	2.3759	0	1.031	0	2.787	0.806	5.173	7.394	2.138	5.778	0	0	0	1.85	0.96	2.5	9
Os02g0670700	251	202	269	181.4	172.7	193.8	103	147	120	150	177.6	132.7	114.29	151.22	153.64	358.2	210.2	264.2	143.1	175	150.9	194.5	188.8	129.3	158.5	156.5	118.4	260	240	240	8
Os02g0519300	424	487	525	454	421.2	473.2	244	301	266	348.3	398	382	469.15	459.26	453	632.9	475.1	468.3	434.8	446.7	396.6	345.3	325	284.6	374	437.5	436.4	588	535	611	13
Os02g0329300	15.3	24.2	10.8	18.06	14.46	11.25	10.2	12	11.7	8.622	9.868	8.467	5.4426	7.4676	8.7116	23.88	18.55	9.324	7.432	5.644	2.587	17.75	7.126	8.667	8.451	7.543	4.386	11.1	3.83	14.2	14
Os02g0120900	396	370	416	790.6	791	788.1	517	519	457	322.5	336.6	315.2	399.49	602.08	340.55	620.9	426.6	466.2	1017	991.7	1070	473.9	496.7	448.5	284.2	381.9	425.4	328	428	361	10
Os02g0247800	342	319	353	247.1	203.4	254.3	94.7	94.2	106	293.1	278.5	253.1	229.68	332.31	289.86	525.4	426.6	365.7	210.9	204.8	190.5	225.5	290.8	217.4	374	330.9	331.1	340	252	322	13
Os02g0123400	28.5	20.5	24.8	25.45	22.6	25.95	43.8	38.6	33.2	35.35	25.22	26.34	28.302	22.403	24.551	11.94	32.98	39.37	28.8	36.28	24.14	38.45	35.63	38.28	26.41	45.26	52.63	30.5	26.8	20.8	11
Os02g0498700	378	293	288	377.6	292.9	436.8	525	494	623	340.6	312.5	332.1	304.79	301.5	268.48	573.2	342.1	306.7	340.9	307.2	381.1	526.4	595.1	663	299	363	532.9	278	351	295	18
Os02g0202500	453	490	491	1091	1230	1079	1327	1213	1298	395.7	457.2	471.4	503.98	633.81	403.11	489.6	441.1	533.6	1361	1255	1269	992.3	926.4	1056	380.3	405.4	421	375	454	363	12
Os02g0286500	1.02	0.93	0	3.284	0.904	0	1.57	1.27	0	0	0	0.941	0	0	0.792	0	0	0	0	1.613	0	0	0.713	2.889	0	0.943	0	0	0	1.67	2
Os02g0474300	328	334	353	433.5	465.5	419.5	460	595	477	336.3	396.9	386.4	301.52	332.31	354.8	167.2	289.6	381.3	380.9	395.9	419	414.8	411.2	454.3	351.8	350.7	339.9	272	262	337	12
Os02g0154600	26.5	14	18.3	17.24	14.46	25.95	16.4	16.4	8.29	3.449	13.16	6.586	10.885	8.4011	6.3357	0	9.275	9.324	14.86	16.13	20.69	18.48	17.82	7.222	7.395	14.14	2.193	13	17.2	2.5	2
Os02g0104100	0	0	0	0	0	0	0	0	0	0	0	0	0	0	0.792	0	0	0	0	0	0	0	0	0	0	0	0	0	0	0	4
Os02g0739700	2.04	3.73	4.31	6.568	5.424	3.46	14.9	5.69	8.29	5.173	5.482	2.822	2.177	2.8004	1.5839	47.76	8.244	3.108	11.15	10.48	7.76	3.697	4.988	7.222	3.169	5.657	8.772	2.78	0.96	2.5	6
Os02g0707100	4726	5108	5321	2480	2683	2240	4383	5688	5088	5268	6208	5381	6865.3	6534.2	6900.4	5947	5323	5367	2242	2171	2118	4295	4025	4525	6395	5591	4741	5440	5154	6199	3
Os02g0215650	0	1.86	3.23	7.389	1.808	3.46	11.7	9.49	6.91	6.897	4.386	4.704	0	0	4.7518	0	2.061	1.036	0.929	4.031	6.898	11.83	10.69	7.945	1.056	0	2.193	3.7	0.96	0	18
Os02g0178300	1.02	0	1.08	4.926	3.616	2.595	3.13	5.06	8.98	5.173	4.386	7.527	3.2656	5.6007	3.9598	11.94	3.092	0	3.716	1.613	7.76	4.436	4.988	2.889	2.113	2.829	8.772	1.85	1.91	1.67	6
Os02g0121100	1536	1292	1323	1410	1473	1436	931	894	906	1313	1235	2313	1091.8	1339.5	1190.3	1218	1565	1408	1301	1119	1243	865.1	876.5	845.7	1617	2004	2123	1072	1258	1161	20
Os02g0750701	3.06	1.86	4.31	3.284	3.616	4.325	0	1.27	1.38	1.724	2.193	0	4.3541	7.4676	1.5839	11.94	2.061	5.18	0	0	0.862	3.697	2.138	0.722	0	0.943	2.193	3.7	1.91	6.66	13
Os02g0523800	187	144	182	73.89	75.93	62.28	234	237	266	202.6	161.2	188.2	102.32	92.412	123.55	47.76	141.2	120.2	114.3	134.6	78.46	153.1	188.1	193.6	100.4	106.5	107.5	99	97.5	83.3	5
Os02g0326700	6175	8250	7385	3129	3573	3060	2230	2500	2981	8642	11156	8605	7298.5	7302.4	8793.2	8383	6579	7613	1737	1912	1795	2838	2100	1893	9403	8914	6506	6047	6041	7761	3
Os02g0611800	698	780	636	186.4	202.5	191.2	334	308	333	506.1	385.9	450.7	364.65	325.77	430.83	394.1	484.3	521.1	170	154	133.6	603.3	620	481	274.7	260.2	337.7	294	386	327	14
Os02g0122700	0	0	0	0	0	0	0	0	0	0	0	0	0	0	0	0	0	0	0	0	0	0	0	1.444	0	0	0	0	0	0	9
Os02g0207800	0	0	0	0	2.712	0	0	0	0	0	0	0	1.0885	0	0.792	0	0	0	0	0	0.862	0	0	0	0	0	0	0	0	0	2
Os02g0314600	339	288	288	45.15	31.64	30.28	16.4	25.3	22.1	97.43	46.05	64.92	53.337	53.207	50.686	167.2	303	219.6	30.66	37.9	38.8	61.37	109	128.6	61.27	69.77	50.44	66.6	40.2	59.2	14
Os02g0537900	1037	1106	1140	1093	1059	1169	1174	1001	999	1040	976.9	1033	917.62	992.26	964.61	1600	903.8	963.5	921.5	907.1	824.3	1114	1033	1052	972.9	1009	1035	944	946	971	6
Os02g0754900	1486	1378	1421	1217	1250	1282	1771	1686	1711	1404	1430	1614	1484.7	1326.4	1365.3	2926	1450	1536	1568	1496	1585	1473	1405	1512	1422	1488	1460	1283	1261	1300	6
Os02g0768300	44.9	19.6	25.9	1.642	5.424	7.785	1.57	1.9	0	7.76	12.06	19.76	11.974	45.739	6.3357	0	23.7	21.76	8.361	3.225	2.587	0.739	0	0.722	4.225	26.4	52.63	28.7	23	27.5	3
Os02g0458100	0	0	0	0	0	0	1.57	0	0	0	0	0	0	0	0.792	0	0	0	0	0	0	0	0	0	1.056	0	6.579	0	0	0	11
Os02g0303200	7.14	4.66	1.08	4.105	5.424	6.055	11.7	8.86	9.67	18.97	2.193	16.93	6.5311	10.268	2.3759	0	12.37	8.288	1.858	5.644	8.622	6.654	7.126	5.056	5.282	7.543	26.32	8.33	7.65	7.5	11
Os02g0221100	9.18	2.79	14	3.284	4.52	6.055	9.39	8.22	7.6	21.55	10.96	11.29	9.7967	13.068	17.423	0	6.183	10.36	5.574	11.29	6.036	25.14	6.414	13	8.451	18.86	13.16	10.2	2.87	11.7	7
Os02g0205400	138	126	117	119	144.6	143.6	172	128	153	152.6	134.9	198.5	109.94	147.49	102.96	71.65	132.9	119.1	148.6	109.7	143.1	113.1	101.9	123.5	142.6	163.1	138.2	95.3	126	119	15
Os02g0215700	28.5	29.8	30.2	58.29	50.62	37.2	90	125	81.5	61.22	30.7	31.05	14.151	18.669	22.175	23.88	36.07	16.58	22.29	19.35	21.56	139.7	158.9	114.8	43.31	46.2	59.21	20.4	33.5	17.5	17
Os02g0195800	178	233	210	251.2	271.2	272.5	236	252	258	275.9	291.6	242.7	252.54	266.03	297.78	274.6	206.1	248.6	338.1	275.7	308.7	236.6	248.7	242	293.7	270.6	254.4	286	259	271	16
Os02g0288200	449	424	477	593.6	606.6	613.3	534	458	462	435.4	367.3	492	484.39	411.65	439.54	489.6	383.4	452.7	540.7	557.9	582.9	468	473.9	492.6	457.4	441.2	429.8	467	413	387	10
Os02g0180000	251	262	200	849.7	835.3	699.8	134	213	149	100.9	159	73.38	97.967	111.08	118.79	107.5	216.4	215.5	563.9	571.7	589.8	269.9	335.7	203.7	129.9	160.3	155.7	174	128	194	10
Os02g0701900	178	148	181	216.7	223.3	205.9	273	223	275	167.3	165.6	162.8	139.33	164.29	166.3	131.4	147.4	112.9	234.1	174.2	208.7	282.4	268.7	329.3	133.1	174.4	149.1	128	123	117	13
Os02g0816700	39.8	39.1	32.3	18.06	18.08	12.11	42.3	25.9	49.8	52.59	39.47	28.22	41.364	28.937	36.43	95.53	47.4	46.62	26.01	24.99	21.56	22.92	24.23	27.45	48.59	44.31	43.86	37.9	44	34.2	13
Os02g0100200	261	239	222	224.1	254.9	205.9	126	167	147	211.2	188.6	173.1	257.98	221.23	244.72	214.9	233.9	216.5	138.4	137.9	144	224	214.5	192.1	254.6	248.9	153.5	246	237	324	8
Os02g0258250	0	0	0	0.821	1.808	0.865	0	0	2.07	0	0	0	0	0	0	0	1.031	0	0	3.225	0	0.739	0	0	1.056	0	0	0	0	0	10
Os02g0146900	59.1	69.9	62.5	92.77	105.8	132.4	204	133	185	84.49	88.81	74.32	76.196	64.408	90.284	95.53	93.78	65.27	159.8	104.8	156.1	111.6	110.5	169.7	90.85	85.8	87.72	77.7	66	70.8	12
Os02g0592300	197	230	185	610.8	645.4	568.3	406	392	350	175.9	138.1	136.4	182.87	177.36	181.36	286.6	175.2	195.8	416.2	541	425.1	278	311.4	250.6	112	116	133.8	182	173	208	10

Os02g0826000	0	0	0	0	0	0	0	0	0	0	0	0	0	0	0	0	0	0	0	0	0	0.739	0	0	0	0	2.193	0	0	0	11
Os02g0655300	1745	1692	1724	1724	1690	1548	2222	1862	1883	1640	1687	1832	2046.4	2007.9	1585.5	2173	1573	1651	2158	2015	2327	1293	1310	1686	1424	1533	1561	1292	1440	1339	15
Os02g0164300	626	581	602	316.1	230.5	387.5	326	318	298	494	486.8	499.6	330.91	457.39	436.37	1122	656.4	586.4	335.4	329	295.7	442.2	439.7	442.7	428.9	500.6	546	597	601	557	13
Os02g0523500	2508	2549	2528	2442	2279	2664	2662	2428	2685	2468	2028	2099	1921.2	2118	2204.8	2902	2107	2183	2027	1953	1774	2502	2522	2391	1894	1865	2213	1946	1800	1959	6
Os02g0832301	0	0	0	0	0	0	0	0	0	0.862	0	0	0	0	0	0	0	0	0	0	0	0	0	0	0	0	0	0	0	0	7
Os02g0749150	678	657	690	874.3	951	852.1	921	803	851	783.7	822.3	780.9	932.86	911.05	866.41	883.6	728.6	726.3	1015	1060	1099	619.6	601.5	684	770.1	815.6	743.4	722	755	720	16
Os02g0727966	76.5	55.9	62.5	71.42	65.09	83.04	93.2	94.2	65.6	99.15	78.94	119.5	119.74	125.08	72.861	47.76	77.29	58.02	118.9	105.6	80.19	43.62	59.15	70.06	106.7	90.51	100.9	87	86.1	65	20
Os02g0506400	5.1	1.86	3.23	4.926	3.616	4.325	1.57	0.63	2.76	8.622	14.25	5.645	2.177	12.135	8.7116	0	6.183	4.144	2.787	10.48	14.66	1.479	7.126	3.611	11.62	14.14	2.193	7.4	8.61	6.66	16
Os02g0137600	65.2	64.3	60.3	248.8	258.5	239.6	177	211	148	45.7	66.88	84.67	57.692	52.273	49.894	119.4	71.11	77.7	290.8	298.3	284.5	139	138.3	170.4	54.93	54.68	48.24	44.4	74.6	44.2	10
Os02g0602500	108	148	129	168.3	207.9	141	215	209	231	177.6	148	222	145.86	154.95	154.43	83.59	139.1	128.5	159.8	160.5	204.3	163.4	191	195.7	192.3	192.3	219.3	139	147	132	15
Os02g0596100	236	225	292	260.2	224.2	253.5	207	256	205	213	260.9	214.5	290.63	237.1	275.6	620.9	211.3	243.5	189.5	260.4	230.2	250.7	242.3	232.6	254.6	228.2	236.8	258	259	265	6
Os02g0206100	604	760	725	1730	1704	1688	1157	1102	1179	993.2	1080	848.6	1526.1	1164.9	1231.5	1385	873.9	817.4	1819	1961	1801	829.6	855.9	936.8	1103	990	802.6	1187	1347	1178	10
Os02g0736100	5.1	8.38	6.47	2.463	4.52	8.65	3.91	3.16	2.07	6.897	4.386	0.941	2.177	4.6673	1.5839	11.94	1.031	2.072	5.574	5.644	1.724	2.218	3.563	5.778	6.338	4.714	2.193	8.33	2.87	6.66	6
Os02g0312600	16.3	19.6	22.6	8.21	6.328	9.516	14.9	16.4	12.4	5.173	12.06	19.76	3.2656	6.5342	4.7518	0	18.55	15.54	10.22	17.74	12.93	20.7	13.54	21.67	2.113	16.03	13.16	10.2	15.3	7.5	9
Os02g0512000	3.06	10.2	3.23	0	0	1.73	0.78	0	0	0	0	0	5.4426	1.8669	5.5438	0	0	0	4.645	0	0	0	3.563	1.444	0	0	0	0.93	1.91	1.67	14
Os02g0814000	3061	2411	2494	173.2	85.88	211.1	84.6	73.4	87.1	1544	1341	1354	574.74	640.35	783.25	1779	2202	1463	409.7	331.4	350.1	76.9	84.8	145.9	1438	1413	1752	806	941	880	14
Os02g0265200	10.2	16.8	21.6	4.105	1.808	5.19	5.48	4.43	8.98	11.21	3.289	9.408	8.7082	12.135	17.423	0	16.49	5.18	16.72	6.45	9.484	7.394	4.276	10.11	5.282	10.37	13.16	11.1	9.56	10	14
Os02g0464700	0	0	0	0	0	1.73	0	0	0	0	0	0	0	0	0	0	0	0	0	0	0	0	0	0	0	0	0	0	0	0	2
Os02g0234750	0	0	0	0	1.808	0	0.78	0	0	0	0	0	0	0	0	0	1.031	0	0	0	0.862	0	0	0	1.056	0	0	0	0	0	2
Os02g0287700	168	108	190	188.8	207.9	169.5	153	176	175	139.7	145.8	134.5	186.14	140.95	148.1	214.9	147.4	162.7	165.4	208	178.5	180.4	152.5	162.5	169	132	120.6	193	156	193	6
Os02g0819100	275	289	258	344	301.9	308.8	392	349	354	311.2	275.2	351.9	354.86	316.44	324.71	429.9	348.3	320.1	391.1	369.3	374.2	346	268.7	323.6	368.7	361.1	285.1	324	352	332	6
Os02g0137400	777	800	751	1024	1094	1061	924	830	841	763	857.4	796.9	810.95	883.05	903.63	895.6	735.8	871.3	993.1	961.1	948.4	925	932.8	871	944.4	935.3	894.7	864	785	783	10
Os02g0661600	21.4	34.5	25.9	13.14	10.85	18.17	23.5	16.4	23.5	33.63	47.14	48.92	32.656	44.806	30.887	71.65	30.92	11.4	16.72	12.9	25	17.01	7.839	30.33	29.58	41.49	43.86	23.1	23	35	13
Os02g0562400	342	324	355	339.1	306.4	316.6	364	380	359	411.3	436.4	415.8	435.41	433.12	456.96	274.6	374.1	350.2	359.5	337.8	321.6	353.4	342.8	335.8	435.2	363.9	416.7	388	420	415	4
Os02g0736300	20.4	27.9	33.4	17.24	17.18	17.3	31.3	38	23.5	12.93	3.289	3.763	7.6196	5.6007	10.296	0	7.214	10.36	9.29	11.29	13.8	22.18	42.76	28.17	10.56	2.829	2.193	3.7	5.74	15.8	17
Os02g0215801	3.06	0.93	0	0.821	2.712	2.595	3.13	1.9	1.38	0	2.193	1.882	1.0885	0	0.792	0	0	2.072	0.929	0	0	0	0	0	0	0.943	0	0	0	5	19
Os02g0243300	33.6	15.8	19.4	7.389	5.424	7.785	0.78	1.27	8.98	24.14	24.12	43.28	9.7967	11.201	6.3357	11.94	49.47	53.87	32.51	17.74	40.52	27.36	19.24	41.89	68.66	113.1	146.9	40.7	44	37.5	11
Os02g0261000	6.12	5.59	2.16	0.821	0.904	6.055	0	1.9	1.38	1.724	0	0	1.0885	6.5342	0	0	6.183	4.144	6.503	0	1.724	3.697	1.425	0.722	1.056	4.714	0	0.93	4.78	1.67	19
Os02g0575000	736	785	446	72.25	49.72	76.12	86.9	79.1	70.5	80.18	65.78	81.85	87.082	82.144	75.237	274.6	427.7	296.3	119.8	83.05	194.9	79.85	84.8	138.7	80.28	113.1	103.1	72.2	72.7	69.1	14
Os02g0461100	1119	1123	1297	944.9	1062	959.3	384	463	359	732	846.4	667	965.52	948.39	883.83	1588	1272	1428	818.4	1007	925.2	730.5	615.7	523.6	1019	918.3	717.1	1279	1177	1332	13
Os02g0592500	451	465	442	454	469.2	411.8	712	686	670	491.4	519.7	436.5	457.18	434.05	428.45	716.5	377.2	440.3	498.8	460.4	469	544.9	555.1	584.3	488	352.6	421	390	389	421	18
Os02g0822400	0	0	0	0	0	0	0	0.63	0	0	0	0	0	0	0	0	0	0	0	0	0	0	0	0	1.056	0	0	1.85	0.96	0	1
Os02g0723400	21.4	12.1	16.2	5.747	1.808	6.055	21.9	13.9	9.67	13.79	18.64	19.76	16.328	20.536	19.799	0	21.64	18.65	8.361	0.806	11.21	64.33	62	73.67	40.14	33.94	48.24	25.9	17.2	27.5	9
Os02g0484975	15.3	12.1	23.7	32.84	22.6	24.22	5.48	4.43	8.29	18.11	25.22	13.17	18.505	9.3345	21.383	11.94	68.02	34.19	19.51	15.32	37.94	22.92	28.51	24.56	48.59	33	35.09	52.7	27.7	40.8	1
Os02g0811800	0	0.93	1.08	0	0	0	0	0	0	0	0	0	0	0.9335	0	0	0	0	0	0	0	0	0	0	0	1.886	0	0	0	0	14
Os02g0721600	1294	1320	1263	1106	1109	1106	1728	1632	1690	1356	1354	1299	1490.2	1515	1550.7	1290	1275	1289	995.8	982.9	1036	1346	1288	1421	1408	1402	1180	1280	1237	1375	5
Os02g0599200	1.02	0.93	1.08	1.642	0.904	2.595	2.35	0.63	4.15	0	3.289	1.882	0	3.7338	0.792	0	2.061	0	4.645	0.806	1.724	6.654	2.851	3.611	2.113	2.829	4.386	0	4.78	0	9
Os02g0157000	0	0	1.08	0	3.616	0.865	0	0	0.69	3.449	0	3.763	1.0885	1.8669	0.792	0	1.031	1.036	1.858	0.806	4.311	0	0.713	3.611	1.056	3.771	0	0	0	0.83	20
Os02g0579000	9.18	14	10.8	206.1	107.6	62.28	35.2	78.4	29	10.35	10.96	10.35	23.947	5.6007	16.631	11.94	6.183	6.216	75.25	33.86	4.311	23.66	32.78	13	5.282	0.943	6.579	17.6	19.1	18.3	2
Os02g0681000	655	568	649	257	318.2	286.3	132	235	191	375.1	509.8	354.7	488.75	536.73	543.29	1111	758.5	827.8	209	221.7	295.7	194.5	179.6	168.3	498.6	488.4	421	702	666	702	13
Os02g0722600	4.08	4.66	0	4.105	3.616	0	1.57	0	0	1.724	0	0.941	1.0885	2.8004	0.792	0	0	0	0	0.806	0	1.479	3.563	0	0	0	0	0	0.96	1.67	2
Os02g0301000	218	148	163	182.3	162.7	206.7	186	191	193	211.2	252.2	217.3	266.69	212.83	219.37	370.2	175.2	212.4	204.4	210.4	211.2	156.7	157.5	203	206	220.6	214.9	197	197	212	6
Os02g0182600	5.1	1.86	7.54	2.463	2.712	6.92	18.8	11.4	24.2	6.035	7.675	8.467	4.3541	6.5342	4.7518	0	10.31	6.216	8.361	1.613	10.35	15.53	15.68	14.44	11.62	11.31	19.74	4.63	4.78	3.33	5
Os02g0686600	262	271	301	637.9	665.3	613.3	478	555	527	311.2	253.3	265.3	320.02	364.98	292.24	322.4	289.6	330.5	693	710.3	722.5	440.7	454.7	411	251.4	221.6	245.6	248	316	242	10
Os02g0127900	254	207	236	232.3	230.5	210.2	182	150	137	205.2	217.1	233.3	217.7	223.09	209.87	214.9	223.6	188.6	269.4	269.3	287.1	126.4	166	138.7	198.6	160.3	203.9	190	237	207	16
Os02g0467600	0	0	0	4.926	0	0.865	0.78	0.63	8.98	1.724	0	0	4.3541	0	0.792	0	0	0	0	0	0.862	0	4.276	0	0	0	0	0	0	0.83	18
Os02g0168700	332	384	387	348.9	401.4	314	466	514	568	444.9	515.3	474.2	486.57	425.65	464.88	549.3	356.6	351.2	436.6	436.2	428.5	278.7	339.9	396.5	501.8	379	353.1	340	369	382	5
Os02g0697800	481	525	537	336.6	370.6	391	579	567	499	572.5	538.3	398.9	480.04	471.39	515.57	668.7	557.5	518	399.4	324.9	349.2	438.5	485.3	437.7	563	482.7	603.1	541	461	565	13
Os02g0712000	1499	1595	1583	2657	2799	2539	878	916	843	1268	1239	1003	1369.4	1302.2	1245	1433	1369	1408	1966	2351	2112	1113	1053	979.4	1178	1138	1081	1467	1380	1450	10
Os02g0760500	276	262	266	245.5	279.3	232.7	251	298	249	223.3	167.7	241.8	182.87	208.16	175.82	274.6	258.7	197.9	269.4	198.3	235.4	251.4	319.3	262.9	208.1	219.7	223.7	206	180	228	17
Os02g0664350	0	0	0	0	0	0.865	2.35	4.43	2.76	0	0	0	0	0	0.792	0	0	0	0	0	0.862	0	1.425	0	0	0	0	0	0	0	18
Os02g0209100	1041	1046	1020	1041	1040	1191	914	945	1089	951	836.5	636.9	906.74	931.58	994.71	1349	850.2	935.5	880.6	916.7	901	1176	1258	1095	857.8	824	855.2	922	840	866	6
Os02g0789100	6.12	13	12.9	18.06	10.85	12.11	21.9	25.3	26.3	9.484	6.578	12.23	10.885	7.4676	8.7116	23.88	11.34	14.5	13.01	10.48	15.52	23.66	19.24	18.06	14.79	7.543	10.96	13.9	9.56	11.7	18
Os02g0750800	12.2	23.3	10.8	7.389	10.85	13.84	3.13	6.33	4.84	0.862	4.386	1.882	1.0885	1.8669	0.792	35.82	17.52	22.79	12.08	11.29	30.18	2.218	4.276	3.611	3.169	3.771	10.96	1.85	7.65	2.5	6
Os02g0518100	536	463	459	282.4	205.2	314.9	476	497	618	506.1	473.6	380.1	401.66	444.32	520.32	716.5	602.9	554.3	353.9	361.2	422.5	611.5	633.5	668.1	540.9	615.7	668.8	595	634	587	1
Os02g0704966	153	193	172	179.8	196.2	164.4	132	202	117	137.9	195.2	186.3	160.01	168.95	213.83	298.5	163.9	187.5	148.6	190.3	133.6	173	148.9	153.1	201.8	225.3	193	152	176	180	13
Os02g0497700	413	360	412	312	293.8	341.7	181	168	147	281.9	265.3	288.8	311.32	280.97	268.48	537.3	455.5	400.9	293.5	309.6	306.1	189.3	176	187.8	285.2	314.9	412.3	284	292	326	13
Os02g0168100	2051	1610	2149	540.2	517.1	569.2	686	789	731	2612	2870	3229	4053.6	3111.2	3529	2699	2594	2341	1197	1266	1067	396.3	600.8	581.4	2962	2869	2491	2481	2359	2385	3
Os02g0141201	0	0.93	0	0.821	0	0	0	0	0	0	0	0	0	0	0	0	0	0	0	0	0	0	0	0	0	0	0	0	0	0	14
Os02g0744000	572	917	731	2625	2746	2195	####	####	####	868.2	1340	1424	507.25	389.25	521.11	406	541	586.4	2182	2477	2241	8015	7043	10202	820.8	792	778.5	362	416	467	18
Os02g0145600	395	439	445	563.2	602	587.4	319	244	262	427.6	432	425.3	444.12	370.58	381.73	334.3	370	378.2	525.8	614.4	547.5	406.7	278.6	287.5	341.2	327.2	289.5	455	423	356	16
Os02g0711400	506	438	499	234	167.2	247.4	364	331	470	636.3	607.4	888.1	369.01	368.71	427.66	262.7	341.1	319.1	379.9	191.1	242.3	336.4	384.8	537.4	599	437.5	539.5	426	302	386	20
Os02g0664100	78.5	134	89.4	91.95	103.1	68.34	15.7	35.4	22.8	25	42.76	4.704	52.249	27.07	38.014	95.53	37.1	68.38	16.72	22.58	6.036	98.34	107.6	67.17	35.92	23.57	26.32	97.1	64.1	87.5	8
Os02g0196000	393	436	442	407.2	414.9	335.6	376	377	330	468.2	452.8	423.4	445.2	472.33	486.27	489.6	441.1	461	426.4	405.6	469	373.4	361.3	363.3	475.4	466.7	493.4	479	540	525	3
Os02g0157600	304	313	287	202.8	172.7	186.9	400	418	366	414.7	453.9	425.3	396.22	357.51	456.96	429.9	418.4	404.1	254.5	267.7	300.9	352.7	413.3	450.7	556.7	456.3	392.5	373	426	502	5
Os02g0242900	3.06	1.86	2.16	0	0	0.865	4.7	0.63	0.69	1.724	0	0	2.177	0	0.792	0	0	0	1.858	0	0	18.48	13.54	18.06	1.056	0	0	7.4	2.87	0.83	9
Os02g0581400	524	601	547	722.5	903.1	763.8	88.5	178	167	282.8	338.8	172.2	452.82	276.3	390.44	680.6	322.6	443.4	247.1	433.8	266.4	451.8	361.3	212.3	359.2	203.7	120.6	404	381	560	8
Os02g0810400	109	117	135	84.56	99.44	94.29	41.5	53.1	59.4	72.42	76.75	39.51	69.665	66.275	79.197	71.65	83.47	105.7	48.31	27.41	32.76	142.7	116.9	93.89	75	50.91	30.7	120	96.6	107	8
Os02g0137700	####	7339	####	746.3	800	627.2	50.1	67	62.9	7477	8961	6687	11259	9258	10789	12299	11267	8950	1238	1326	986.4	66.54	100.5	94.61	8963	8924	8039	8454	7220	9193	3
Os02g0253502	0	1.86	0	0.821	1.808	1.73	0	0	9.67	0.862	3.289	0	4.3541	0.9335	0	0	0	1.036	0	0	1.724	0	0	4.333	0	0	0	0	0	0	18
Os02g0244700	136	124	123	214.3	180.8	219.7	525	364	402	231.9	242.3	284.1	199.2	191.36	215.41	59.7	146.3	157.5	323.3	314.4	307.8	190.8	282.9	308.4	166.9	201.8	304.8	201	158	147	15
Os02g0720300	141	176	204	169.9	175.4	168.7	201	224	236	173.3	256.6	145.8	171.99	182.02	215.41	238.8	148.4	162.7	166.3	143.5	173.3	250.7	206.7	245.6	221.8	192.3	177.6	186	172	172	9
Os02g0601800	3631	3812	3249	2836	2736	3068	2185	2523	2513	2175	2391	1906	2611.4	2403.6	2981.8	2949	2563	2750	2236	2715	2498	3497	3203	2768	2552	2013	2088	2581	2597	2685	8
Os02g0741300	958	1107	1040	756.1	829.8	729.2	629	629	629	803.6	1103	929.5	1249.6	1079.1	1144.4	1839	1167	1212	813.8	935.3	874.3	683.2	627.8	665.2	1203	1094	943	1123	1068	1199	13
Os02g0255300	0	0	0	0	0	3.46	0	0	0	0	0	0	0	0	0	0	0	0	0	0	0	0	0	0	0	0	0	0	0	0	2
Os02g0103100	1.02	0.93	1.08	0	0	0	0	0	0	0.862	0	0.941	1.0885	0	2.3759	0	0	0	0	0	0	0	0	0	0	2.829	0	0.93	2.87	1.67	1
Os02g0301800	345	415	380	220	220.6	231.8	242	233	197	339.7	342.1	337.8	297.17	333.24	319.16	406	334.9	386.4	196	216.9	212.1	270.6	241.6	252.8	382.4	404.5	289.5	441	409	422	3
Os02g0823300	456	499	480	364.5	318.2	340	412	485	435	464.7	560.3	615.3	637.87	506.86	594.77	692.6	490.5	556.4	313.1	358	349.2	402.2	393.4	464.4	649.7	690.2	638.1	562	539	602	3
Os02g0171700	0	0	0	0	0	0.865	0	0	0	0	0	0	0	0.9335	0	0	0	0	0	0	0	0	0	0	0	0	0	0	0	0	4
Os02g0649700	0	0	0	0	0	0	0	0	0	0	0	0	0	0	0	0	0	0	0	2.419	0	0	0	0	0	0	0	0	0	0	16
Os02g0105400	5.1	3.73	4.31	7.389	5.424	8.65	54.8	44.9	42.2	25.87	20.83	10.35	11.974	10.268	15.047	11.94	2.061	3.108	7.432	10.48	6.036	21.44	32.78	38.28	8.451	6.6	13.16	6.48	6.69	3.33	18
Os02g0543900	17.3	6.52	9.7	27.09	28.02	13.84	32.9	16.4	47.7	7.76	5.482	10.35	2.177	2.8004	2.3759	0	4.122	3.108	8.361	7.257	5.173	3.697	2.138	7.222	2.113	0	0	1.85	2.87	0.83	12
Os02g0533900	1152	1088	1034	958.9	1107	985.3	1159	1236	1367	1440	1282	1647	1170.2	1228.4	1184	764.2	983.1	1013	1124	832.1	885.5	1142	1155	1216	1236	1260	1368	937	948	886	20
Os02g0830300	129	131	84.1	94.41	95.82	70.07	49.3	76.5	65.6	75.01	72.36	73.38	64.223	57.874	50.686	179.1	84.5	92.21	59.45	66.12	53.46	96.12	95.49	85.22	58.1	82.03	57.02	95.3	95.6	113	6
Os02g0829200	2669	2480	2744	1282	1243	1183	869	1076	967	2962	2856	2837	3375.5	2846.1	3255	4621	2805	2705	1513	1609	1338	902.1	989.1	1095	2925	2923	2439	2675	2587	2640	13
Os02g0317300	143	174	161	193.8	197.1	181.7	164	190	183	150.9	152.4	190	152.39	148.42	163.94	226.9	183.4	161.6	161.6	162.9	142.3	236.6	193.1	187.8	168	140.5	118.4	178	181	186	6
Os02g0136966	14.3	13	20.5	7.389	9.04	12.98	6.26	5.06	6.22	12.07	0	9.408	5.4426	4.6673	14.255	0	12.37	9.324	8.361	12.09	4.311	14.79	10.69	17.33	4.225	6.6	21.93	13.9	7.65	10	11
Os02g0664300	1677	1800	1925	2927	3163	2831	1646	1858	1793	1600	1691	1499	1803.7	1758.6	1729.7	2149	1716	1766	2519	2528	2638	1507	1559	1455	1622	1514	1570	1718	1837	1815	10
Os02g0102850	202	183	182	206.1	194.4	207.6	173	211	196	181.1	186.4	175	210.08	156.82	161.56	203	152.5	220.7	236	216.1	194	191.5	197.4	197.9	220.8	239.5	199.6	179	243	202	16
Os02g0578300	0	0	0	0	0	0	0	0	0	0	0	0	0	0	0	0	0	0	0	0	0	0	0	0.722	0	0	0	0	0	0	9
Os02g0810200	14.3	19.6	16.2	9.852	11.75	8.65	5.48	7.59	6.91	21.55	13.16	23.52	11.974	13.068	11.088	11.94	11.34	17.61	8.361	4.838	1.724	5.915	3.563	6.5	9.507	10.37	10.96	0.93	14.3	14.2	3
Os02g0785200	0	0	0	0	0	0	0	0	0	0	0	0	0	0	0	0	0	1.036	0	0	0	0	0	0	0	0	0	0	0	0.83	1
Os02g0787600	277	387	357	329.2	320.9	280.3	566	578	376	807.9	555.9	315.2	414.73	273.5	359.55	274.6	241.1	290.1	249.9	231.4	223.3	229.2	444	221	434.2	220.6	188.6	174	223	258	7
Os02g0175200	0	0	0	0	0	0	0	1.27	0	0	0	0	0	0	0	0	0	1.036	0	0	1.724	0	0	0	0	2.829	0	0	0	0	16
Os02g0770000	834	829	834	832.5	947.4	891.9	914	812	868	918.2	849.7	948.3	890.41	902.65	912.34	1039	792.5	833	964.3	673.2	813.1	655.1	743.3	699.9	834.5	907	936.4	779	697	743	20
Os02g0193933	1.02	0.93	0	0	3.616	0.865	0	0.63	6.22	1.724	0	0	0	0	0	0	0	0	1.858	0	0.862	2.218	1.425	0	2.113	0	0	0	0	0	18
Os02g0199666	36.7	44.7	72.2	137.1	139.2	135.8	72.8	71.5	104	136.2	125	115.7	115.38	61.608	63.357	59.7	61.83	83.92	173.7	149.2	190.5	88.73	79.82	96.06	112	80.14	81.14	113	71.7	87.5	16
Os02g0690800	893	935	928	894.9	864.2	840	800	831	893	951.9	828.9	929.5	831.63	995.06	824.44	1373	878	875.5	923.4	779.7	811.3	722.4	723.3	660.1	812.3	816.5	879.4	833	753	788	6
Os02g0122800	756	1081	964	2005	2136	2029	1105	942	907	1092	1100	993.5	1215.9	1124.8	1197.5	1087	1172	1119	1859	1871	1912	923.5	968.5	840.7	1014	1061	1033	1178	1118	1101	10
Os02g0164900	850	666	695	412.1	408.6	522.5	699	514	647	663.9	597.5	654.8	482.21	563.8	522.7	656.8	802.8	716.9	425.5	437.8	538.9	839.9	728.3	773.5	751.1	841	673.2	715	526	603	9
Os02g0174200	204	211	251	257	264	243.9	420	364	363	287.1	255.5	313.3	249.27	229.63	251.85	226.9	244.2	236.2	333.5	341.9	325.1	319.4	317.8	290.3	304.2	293.2	328.9	238	212	218	15
Os02g0801300	13.3	12.1	10.8	6.568	8.136	9.516	3.91	8.22	23.5	8.622	2.193	12.23	6.5311	1.8669	68.109	23.88	16.49	16.58	4.645	7.257	5.173	9.612	8.552	2.167	7.395	29.23	4.386	18.5	3.83	15.8	4
Os02g0281000	2097	2341	2343	1065	1236	1058	1398	1529	1427	1619	1885	1721	1993.1	1740	2046.4	2842	2202	2377	1161	1019	906.2	1675	1766	1640	1960	2010	1695	2402	2402	2639	13
Os02g0136150	86.7	64.3	95.9	21.35	21.7	38.93	41.5	25.9	27.6	79.32	98.68	90.32	111.03	78.41	80.781	107.5	77.29	109.8	36.23	31.44	54.32	44.36	43.47	43.33	102.5	101.8	157.9	83.3	88.9	86.6	3
Os02g0608600	893	789	807	1028	974.5	1075	995	1020	1084	656.1	481.3	1248	487.66	541.4	388.06	931.4	965.6	818.5	1274	911.1	1473	1170	810.3	1587	577.8	1266	1454	438	645	432	15
Os02g0635300	46.9	30.7	57.1	27.09	45.2	46.71	27.4	32.9	28.3	30.18	32.89	18.82	27.213	41.072	39.598	47.76	21.64	44.55	19.51	17.74	31.04	45.84	28.51	23.83	59.16	40.54	35.09	60.1	65	53.3	8
Os02g0734800	5.1	3.73	6.47	5.747	7.232	12.98	4.7	3.16	2.76	2.587	2.193	0	3.2656	8.4011	0.792	11.94	4.122	6.216	11.15	10.48	5.173	5.176	6.414	3.611	2.113	0	10.96	2.78	1.91	2.5	10
Os02g0717900	52	58.7	48.5	43.51	23.5	34.6	61.8	48.1	53.9	52.59	48.24	43.28	38.098	49.473	53.854	47.76	45.34	54.91	39.94	33.86	33.63	44.36	39.91	43.33	44.37	39.6	54.82	47.2	47.8	49.2	5
Os02g0448000	6.12	9.31	7.54	1.642	0.904	3.46	0	3.8	0	14.66	1.096	15.99	10.885	14.002	8.7116	0	5.153	8.288	0.929	8.869	4.311	5.176	4.276	5.778	2.113	6.6	15.35	4.63	8.61	6.66	20
Os02g0712600	0	0	0	0	0	0	0	0	2.76	0	0	0	0	0	0	0	0	0	0	0	0	7.394	2.138	5.778	0	0	0	0	0	0	9
Os02g0134300	1325	1135	1232	1189	1231	1162	1951	1490	1505	1561	1434	1351	2233.6	2092.8	1800.1	1934	1067	1099	1292	1437	1167	1315	1472	1204	1376	1085	1193	1730	1382	1337	4
Os02g0651300	7304	8308	7293	5873	6233	6121	6428	6797	6781	12577	14506	18341	11864	10011	10566	7833	8391	7969	5300	5182	5275	5145	4805	6102	13484	15282	13741	6425	4961	7116	20
Os02g0121500	1096	1444	1370	2054	2017	2051	431	577	432	1112	1255	1216	1713.3	1656.9	1581.6	2746	1849	1704	1982	1963	2390	425.1	386.2	378.5	1586	1772	1263	1694	1760	2159	16
Os02g0733001	64.2	41.9	70	75.53	50.62	64.01	54.8	29.1	42.8	94.84	78.94	95.02	137.15	71.876	65.733	119.4	70.08	54.91	106.8	90.3	98.29	48.06	55.59	52	83.45	79.2	76.75	71.2	53.6	59.2	16
Os02g0228151	2.04	3.73	2.16	4.105	4.52	7.785	24.3	16.4	14.5	13.79	13.16	5.645	7.6196	7.4676	4.7518	0	3.092	2.072	10.22	15.32	1.724	7.394	9.264	7.222	12.68	1.886	13.16	8.33	4.78	5.83	5
Os02g0656100	3.06	0	0	0	0	0	0	0	0	0	0	0	0	0	0	0	0	0	0	0	0	0	0	0.722	0	0	6.579	0	0	0	11
Os02g0650900	18.4	27	18.3	22.17	19.89	22.49	29.7	29.1	30.4	22.42	20.83	36.69	29.39	21.469	25.343	11.94	13.4	29.01	21.37	24.99	31.9	23.66	32.78	31.78	30.63	18.86	32.89	25	23.9	15	15

Os02g0194100	735	723	735	544.3	562.3	573.5	368	397	428	657.8	535	605.9	545.35	764.5	628.82	680.6	735.8	710.7	584.3	589.4	491.5	568.6	577.9	532.3	657.1	693	684.2	719	682	728	3
Os02g0208200	1214	1223	1290	1221	1278	1218	1097	982	917	1304	1139	1317	1517.4	1440.3	1359.8	1302	1345	1419	1510	1363	1449	1023	1030	945.4	1292	1319	1454	1373	1343	1410	16
Os02g0829600	2971	3302	3399	882.5	852.4	854.7	3180	4040	2986	5359	5171	4706	4014.5	4061.4	5044.8	3427	3673	3400	946.6	1019	978.6	1942	2798	2037	4418	4399	3568	2931	3157	3460	3
Os02g0210800	265	277	283	257	229.6	295.8	234	288	312	244.9	294.9	259.7	341.8	336.98	291.44	286.6	292.7	296.3	251.7	275.7	294	406.7	329.2	298.3	325.4	330.9	315.8	311	334	329	1
Os02g0142060	52	56.8	52.8	55.01	43.39	62.28	29.7	29.7	36.6	32.76	25.22	25.4	17.416	39.205	34.055	83.59	35.04	32.12	26.94	45.96	37.94	90.21	54.87	50.56	36.97	34.89	32.89	70.3	32.5	65	8
Os02g0591850	0	0	1.08	0	3.616	0	0	0	0	1.724	0	0	0	1.8669	0	0	0	0	0	0.806	1.724	0	0	0	0	1.886	0	0	0	0	16
Os02g0260500	101	102	78.7	144.5	151	152.2	100	94.2	94	86.22	106.4	57.39	117.56	82.144	91.868	107.5	86.56	107.7	88.25	94.33	104.3	105	101.2	96.06	66.55	66.94	89.91	89.7	101	90	2
Os02g0569900	3.06	2.79	5.39	18.88	18.08	10.38	38.4	32.9	27.6	10.35	14.25	17.88	7.6196	9.3345	8.7116	0	6.183	4.144	18.58	21.77	4.311	36.23	28.51	46.22	2.113	8.486	13.16	4.63	1.91	5.83	18
Os02g0665800	3.06	0.93	4.31	0.821	2.712	0.865	0	0	0	1.724	1.096	5.645	4.3541	0	0.792	0	3.092	2.072	0	0	0.862	0.739	2.851	1.444	4.225	0	0	0.93	0	3.33	20
Os02g0527650	0	0.93	0	0.821	0	0	3.13	0	1.38	0	0	0	0	0	0	0	1.031	1.036	0	0	0	10.35	4.276	3.611	1.056	0	0	0	0	2.5	9
Os02g0595100	1287	1390	1439	2332	2173	2948	1453	2042	1719	1136	899	1351	1316	1386.2	1470.7	1170	1561	1628	2188	2058	2764	1723	1612	1963	1140	1367	1171	1549	1420	1557	10
Os02g0704262	5.1	3.73	12.9	0.821	5.424	6.92	0	1.9	2.76	7.76	13.16	22.58	7.6196	14.002	7.9197	11.94	7.214	13.47	13.93	9.675	9.484	5.915	3.563	4.333	13.73	21.69	10.96	18.5	22	15	3
Os02g0819700	401	316	349	356.3	289.3	392.7	445	467	523	481.1	460.5	495.8	494.19	542.34	451.42	585.1	409.1	336.7	484	504.7	520.8	389.7	384.1	396.5	470.1	492.2	489	387	424	354	15
Os02g0815100	0	0.93	0	0	0	0	0	0	0	0	0	0	0	0	0	0	0	0	0	0	0	0	0	0	0	0	0	0	0	0	14
Os02g0733900	46.9	31.7	28	1.642	1.808	16.44	0.78	0	0	0	0	0.941	9.7967	2.8004	0	0	12.37	17.61	2.787	0.806	0.862	0	6.414	10.11	2.113	2.829	0	0	6.69	3.33	14
Os02g0116300	0	0	0	0	0	0	0	0	0	0	0	0	0	0	0	0	0	0	0	0	0	0	0	0	1.056	0.943	0	0	1.91	0	1
Os02g0793500	3.06	7.45	4.31	2.463	4.52	7.785	3.13	0.63	0	7.76	10.96	15.05	3.2656	2.8004	0	35.82	5.153	5.18	8.361	10.48	4.311	8.873	6.414	9.389	5.282	9.428	19.74	1.85	14.3	5	6
Os02g0611000	73.4	70.8	99.1	50.9	60.57	62.28	57.9	64.5	61.5	64.66	97.58	85.61	60.957	73.743	83.948	167.2	96.87	109.8	61.31	69.34	67.25	73.94	90.5	65.72	92.96	100.9	63.59	137	137	124	13
Os02g0674233	0	0	0	202.8	57.85	89.1	10.2	6.33	6.91	0	0	0	0	0	0	11.94	2.061	0	278.7	170.9	338	4.436	0.713	7.945	0	0	0	0	0	0.83	10
Os02g0240100	0	0	1.08	15.6	13.56	8.65	49.3	35.4	78.8	0.862	4.386	0	0	0	0	0	0	0	4.645	9.675	7.76	47.32	19.95	52.72	0	0	0	0	0	0.83	18
Os02g0716100	422	353	443	423.6	429.4	560.6	307	396	273	332.8	339.9	335.9	360.3	409.79	432.41	417.9	443.1	457.9	371.6	354	406.1	441.4	362	325	407.8	446.9	440.8	442	508	575	8
Os02g0491900	0	0.93	0	0	0	0	0	1.27	0	0	0	0	0	0	0	0	0	0	0	0	0	0.739	0	0	0	0	0	0	0	0	18
Os02g0329600	740	709	835	610	617.4	650.5	568	610	538	616.5	677.6	592.7	863.2	783.17	822.06	788.1	817.2	840.2	642.8	697.4	734.6	818.5	731.9	656.5	709.9	796.7	728	888	851	918	1
Os02g0291000	3.06	2.79	4.31	8.21	4.52	5.19	65.8	19	45.6	3.449	3.289	10.35	1.0885	0.9335	6.3357	0	4.122	3.108	0.929	0.806	0	14.79	9.264	25.28	5.282	7.543	6.579	0	1.91	5.83	18
Os02g0617300	170	160	124	160.9	193.4	201.6	243	211	217	123.3	110.7	156.2	78.373	88.678	53.854	47.76	130.9	123.3	106.8	136.3	191.4	182.6	146.1	243.4	90.85	117.9	114	57.4	60.2	67.5	12
Os02g0516400	15.3	12.1	10.8	4.926	9.04	3.46	12.5	3.16	2.76	10.35	8.771	21.64	8.7082	6.5342	5.5438	0	17.52	20.72	13.93	6.45	4.311	3.697	0.713	3.611	5.282	7.543	10.96	7.4	10.5	5	20
Os02g0521300	1290	1167	1377	869.4	838.9	884.9	990	1106	1115	963.1	950.6	892.8	1232.2	1261.1	1383.6	1564	1257	1304	871.4	951.4	858.8	1294	1297	1135	1204	1038	1031	1269	1288	1182	13
Os02g0680700	3729	3189	4014	509.8	460.1	535.5	1275	1543	1365	4025	4285	4333	4513	4194	4399.4	7391	4740	4402	882.5	815.2	737.2	1169	1318	1361	4673	4878	4292	4620	4498	4683	13
Os02g0519900	9.18	4.66	2.16	9.852	11.75	7.785	0	0.63	0.69	3.449	4.386	0	5.4426	1.8669	2.3759	0	7.214	9.324	1.858	4.031	1.724	8.133	6.414	1.444	4.225	14.14	2.193	5.55	8.61	10.8	8
Os02g0534800	1040	848	874	960.5	1022	1129	1094	863	1084	1058	999.9	1750	1149.5	1267.6	824.44	1242	964.6	934.5	1338	1235	1428	934.6	910	958.4	1024	1194	1531	726	881	596	20
Os02g0229600	4.08	2.79	3.23	1.642	0.904	6.055	0	1.27	1.38	1.724	1.096	0.941	0	0	1.5839	0	1.031	1.036	1.858	2.419	0	0.739	2.851	2.889	1.056	1.886	0	1.85	0	0.83	19
Os02g0510100	89.7	72.7	79.7	59.11	81.36	83.04	98.6	114	85.7	103.5	104.2	112.9	87.082	93.345	106.92	310.5	96.87	77.7	105.9	116.1	114.7	87.25	73.4	81.61	89.79	85.8	107.5	68.5	51.6	57.5	6
Os02g0703600	67.3	50.3	42	0	0	5.19	25.1	8.22	34.6	5.173	2.193	0	0	5.6007	6.3357	0	46.37	35.23	13.01	1.613	4.311	147.1	63.42	91.73	0	2.829	8.772	0.93	6.69	1.67	9
Os02g0824100	0	0.93	0	0.821	0	0	0	0	0	0	0	0	0	0	0	0	0	1.036	0	0	0	0	0	0	0	0	0	0	0	0	14
Os02g0769700	0	0	0	5.747	9.04	8.65	4.7	6.96	2.07	0	0	0	0	0	1.5839	0	0	0	0.929	5.644	7.76	9.612	4.988	3.611	0	0	0	0	0	0	12
Os02g0543300	0	0	1.08	0	0	1.73	0	0.63	0	1.724	0	2.822	0	1.8669	0	0	0	0	0	0	0	1.479	1.425	0	1.056	0	0	0	0	0	7
Os02g0272350	9.18	17.7	11.9	10.67	12.66	8.65	22.7	6.96	17.3	17.24	7.675	17.88	15.239	5.6007	22.967	11.94	26.79	8.288	14.86	12.9	15.52	11.83	14.97	20.95	12.68	15.09	13.16	13.9	9.56	9.16	5
Os02g0117500	25.5	34.5	23.7	76.35	72.32	94.29	79.1	112	126	28.45	40.57	4.704	25.036	10.268	30.887	23.88	17.52	20.72	16.72	22.58	16.38	127.9	131.8	96.06	26.41	16.03	26.32	22.2	22	20.8	18
Os02g0290900	3.06	7.45	2.16	16.42	23.5	24.22	22.7	11.4	16.6	4.311	2.193	2.822	4.3541	3.7338	3.9598	0	6.183	7.252	29.73	37.09	27.59	24.4	19.24	17.33	11.62	9.428	8.772	13	6.69	8.33	12
Os02g0668700	73.4	53.1	36.6	69.78	46.1	52.77	65	52.5	58	50.87	69.07	76.21	56.603	62.541	67.317	143.3	70.06	56.98	58.52	51.6	97.43	74.68	66.99	70.06	63.38	56.57	85.52	80.5	73.6	62.5	6
Os02g0720100	30.6	18.6	15.1	3.284	3.616	6.92	21.1	15.2	9.67	22.42	13.16	20.7	9.7967	19.602	19.007	23.88	34.01	15.54	13.01	16.93	16.38	10.35	9.264	13.72	20.07	18.86	24.12	16.7	23.9	30	3
Os02g0113800	595	573	626	576.3	551.4	508.6	612	604	642	847.5	767.5	824.2	760.88	757.03	779.29	907.5	679.1	634.1	591.7	607.1	669.9	493.9	549.4	546	814.5	864.6	763.1	753	711	690	3
Os02g0733400	1.02	0.93	2.16	5.747	5.424	4.325	6.26	7.59	11.1	1.724	3.289	0.941	2.177	4.6673	1.5839	0	4.122	2.072	2.787	2.419	0.862	2.218	2.138	5.056	0	0	4.386	2.78	0.96	0.83	18
Os02g0797500	4395	4511	4471	3700	3918	3960	4863	4725	4999	4370	3836	4793	3759.7	4365.8	3803.8	5815	3612	3996	3845	3311	3620	4277	3805	4779	3810	3871	4134	3120	3399	3339	6
Os02g0761300	2.04	0	0	0.821	0	0	0.78	0	0	0	0	0	0	0	0	0	0	0	2.787	0	0	1.479	0.713	0.722	1.056	0	0	0.93	0	0	19
Os02g0248440	0	0	0	10.67	14.46	12.98	0	0	0	0	0	1.882	0	0	3.1679	0	0	0	12.08	15.32	38.8	0.739	0	0	0	0	0	0	0	1.67	10
Os02g0735200	####	####	####	2414	2994	2037	2623	2429	1703	11918	9331	12840	7301.8	7850.3	7187.1	9433	10610	9804	2576	2150	2570	2313	3040	3103	8995	9594	11429	####	8689	9835	3
Os02g0116400	91.8	82.9	85.1	13.14	18.08	9.516	11	17.7	1.38	62.08	115.1	92.66	65.311	70.009	92.66	107.5	103.1	105.7	13.93	12.9	22.42	26.62	30.64	29.61	166.9	182	129.4	159	96.6	140	3
Os02g0286850	5.1	13	15.1	8.21	4.52	16.44	3.91	1.9	0	0.862	6.578	7.527	4.3541	4.6673	3.9598	0	2.061	2.072	2.787	1.613	2.587	16.27	16.39	6.5	1.056	2.829	4.386	9.25	6.69	5.83	17
Os02g0735800	344	481	411	1011	1115	877.2	723	835	707	370.7	475.8	399.8	527.93	436.86	346.09	513.5	315.3	415.5	719.9	916.7	878.6	570.8	584.4	792.3	396.1	221.6	241.2	248	285	310	12
Os02g0793700	4478	4477	4874	2098	2053	2476	1638	1707	1636	4936	4743	5945	4183.2	4962.2	3486.2	4824	5118	4548	4047	2608	4580	1177	1387	1574	4171	5472	5245	3008	4044	3577	3
Os02g0738100	18.4	14.9	11.9	17.24	32.54	19.9	4.7	22.1	13.8	45.7	28.51	33.87	2.177	19.602	36.43	23.88	29.89	2.072	16.72	32.25	39.66	11.09	27.79	21.67	19.01	11.31	17.54	12	8.61	5	7
Os02g0177900	1122	1261	1140	1074	1280	995.7	1939	1939	1863	1318	1459	1333	1249.6	1221.9	1332.1	1349	1257	1182	1173	1177	1187	1533	1540	1623	1386	1386	1186	1206	1377	1284	18
Os02g0692700	732	570	708	849.7	846.1	942	1287	896	1018	1112	862.9	1039	601.95	591.81	593.18	549.3	561.6	589.5	1157	1001	1087	1045	1191	1132	792.3	679.8	760.9	848	721	689	15
Os02g0745800	56.1	56.8	53.9	48.44	65.09	42.39	58.7	80.3	74.6	41.38	61.4	43.28	39.187	44.806	27.719	47.76	43.28	52.84	76.17	68.53	74.15	59.15	46.32	47.67	26.41	37.71	30.7	62.9	73.6	42.5	12
Os02g0170200	556	370	476	347.3	337.2	408.3	535	510	578	552.7	555.9	736.7	531.2	662.75	519.53	453.8	617.3	455.9	487.7	420.9	434.6	418.5	404.1	443.5	589.5	648.7	754.4	498	451	421	20
Os02g0197700	194	202	223	107.5	90.4	122.8	24.3	29.1	36.6	170.7	225.9	212.6	185.05	186.69	167.9	489.6	170	217.6	175.6	139.5	182.8	25.14	18.53	36.83	235.6	202.7	162.3	223	214	214	13
Os02g0470400	923	1002	850	912.9	909.4	915.2	672	882	747	811.3	895.8	829.8	1008	807.44	940.86	1075	967.7	869.2	667	865.9	810.5	868	836.6	798.8	885.2	876.8	890.3	923	942	887	13
Os02g0578800	884	926	913	899.8	1069	849.5	1094	1077	928	947.5	983.5	1117	1083.1	967.06	986	1337	848.1	933.5	929	923.2	868.3	721.6	747.6	663.7	830.3	717.5	708.3	786	850	807	6
Os02g0293300	0	0	0	0	0	0	0	0.63	0	0	0	0	0	0	0	0	0	0	0	0	0	0	0	0	0	0	0	0.93	0.96	0	1
Os02g0730900	39.8	49.4	32.3	211.8	249.5	176.5	88.5	124	86.4	36.21	36.18	21.64	46.806	32.671	38.014	35.82	27.82	36.26	121.7	185.4	161.2	66.54	61.29	87.39	35.92	30.17	6.579	25.9	26.8	27.5	10
Os02g0775400	82.6	88.5	94.8	114.1	94.92	113.3	60.3	84.8	80.2	59.49	69.07	50.8	40.275	56.941	60.189	83.59	63.89	88.06	91.97	91.92	96.57	84.29	89.08	99.67	42.25	49.97	41.67	50.9	43	61.7	10
Os02g0117400	585	624	573	947.4	898.5	903.1	1031	898	1023	699.2	664.4	715	538.82	643.15	593.18	597	570.9	583.3	896.4	907.9	778.6	882.8	841.6	830.6	629.6	625.1	633.8	580	590	615	12
Os02g0541500	0	0	0	0	0	0	0	0	0	0	1.096	0	0	0	0	0	0	0	0	0	0	0	0	0.722	0	0	0	0	0	0	7
Os02g0826200	373	421	421	670.7	777.4	696.4	412	467	457	321.6	401.3	313.3	374.45	379.91	350.05	597	499.8	451.7	706	771.6	656.1	432.5	339.9	364.7	304.2	308.3	282.9	330	374	337	10
Os02g0171100	3.06	0	1.08	0	0	0	4.7	0	0	0.862	0	1.882	0	1.8669	1.5839	0	1.031	3.108	0	0	4.311	0	0	0.722	1.056	0	0	0	0.96	0.83	19
Os02g0259100	2217	2042	2228	1671	1628	1758	885	942	1083	1988	1944	2039	2013.8	1887.4	1996.5	2090	1898	1964	1620	1668	1965	1167	1417	1121	1987	2227	1947	1758	1786	1920	3
Os02g0161500	0	0	0	0	0.904	0	1.57	1.27	0	0.862	0	0	0	0	0.792	0	0	0	0	0	0	0.739	0	0	0	1.886	0	0	0	0	5
Os02g0194400	9.18	3.73	1.08	0.821	1.808	1.73	3.13	0	5.53	1.724	0	3.763	1.0885	0.9335	2.3759	0	7.214	6.216	2.787	4.031	9.484	5.176	3.563	5.778	3.169	4.714	2.193	2.78	1.91	2.5	19
Os02g0204300	845	894	920	1044	1035	979.2	1087	1061	966	947.5	837.6	948.3	975.31	864.38	898.88	979.2	949.1	952.1	969.8	1040	965.7	877.7	955.6	910	900	807.1	850.9	969	972	934	12
Os02g0149800	4193	4477	4724	793.1	763	780.3	2472	2653	2755	4135	4519	4571	4394.4	4473.1	4743.1	5696	5176	4926	1325	1167	952.7	2915	2777	2852	4838	5089	4460	5124	5216	5588	3
Os02g0122600	152	156	164	401.5	395	308.8	557	693	536	353.5	232.4	165.6	158.92	133.48	174.23	203	129.8	139.9	406.9	379	326.8	340.1	534.5	376.3	162.7	142.4	149.1	110	136	141	12
Os02g0271000	7.14	3.73	0	1.642	0.904	0	0.78	5.06	0	6.035	0	6.586	0	0.9335	3.1679	0	2.061	4.144	0.929	0	0	5.176	7.126	1.444	1.056	1.886	0	1.85	1.91	2.5	19
Os02g0814700	1718	1344	1455	2065	2185	2309	2213	1719	2339	1878	1698	2901	1867.9	2147.9	1375.6	2078	1225	1471	2636	2180	2446	1680	1762	1677	1600	1892	2215	1136	1294	995	15
Os02g0696500	79.5	41	40.9	8.21	2.712	21.63	11.7	10.1	12.4	15.52	10.96	25.4	8.7082	14.002	7.9197	23.88	74.2	53.87	6.503	4.031	12.07	9.612	5.701	14.44	7.395	28.29	32.89	4.63	7.65	11.7	14
Os02g0202400	0	0	0	0	0	0	0.78	0	0	0	0	0	0	0	0	0	0	0	0	0	0	0	0	0	0	0	0	0	0	0	5
Os02g0489700	1.02	0	0	2.463	0	4.325	0	0	0.69	0	0	0	0	0	0	0	0	0	0	0	0	0	1.425	0	0	0	0	0	5.74	1.67	2
Os02g0491400	3202	2517	3826	746.3	476.4	805.4	152	136	274	3789	3472	3795	5875.8	4966	6449.8	2866	2138	2015	1157	979.6	834.6	317.2	369.1	354.6	3418	2617	3053	3392	3015	2795	3
Os02g0620800	88.7	75.4	67.9	130.5	160	154	87.7	89.8	71.2	55.18	47.14	66.8	107.76	92.412	78.405	83.59	89.66	83.92	140.3	164.5	96.57	76.9	52.73	78.72	77.11	62.23	52.63	95.3	73.6	87.5	10
Os02g0187600	200	185	202	287.3	312.8	230.1	208	190	229	203.5	195.2	189.1	280.84	229.63	185.32	119.4	109.2	132.6	214.6	173.4	224.2	113.9	116.2	139.4	146.8	136.7	175.4	130	133	150	2
Os02g0781300	7.14	4.66	5.39	0	0	0	0	0	0	0	0	0	0	0	0	0	0	2.072	0	0	0	0	0	0	0	0	0	0	0	0	14
Os02g0160800	415	355	365	165.8	122.9	120.2	27.4	31.6	23.5	145.7	182	152.4	127.36	180.16	178.19	226.9	410.2	358.5	129.1	167.7	218.1	36.97	59.86	33.22	189.1	238.5	171	373	274	299	3
Os02g0554800	257	362	343	417.9	408.6	325.3	532	577	520	334.5	396.9	267.2	428.88	361.25	383.31	513.5	343.2	378.2	429.2	525.7	518.2	304.6	323.5	404.5	403.5	226.3	263.2	340	309	379	12
Os02g0743600	0	0	1.08	1.642	1.808	2.595	1.57	0	2.76	0	0	2.822	0	0.9335	0	0	3.092	0	4.645	9.675	7.76	0.739	0	1.444	0	0	0	1.85	0	0.83	16
Os02g0565400	432	401	434	633.8	739.4	560.6	554	641	520	492.3	564.6	689.6	678.15	517.13	515.57	609	387.5	521.1	638.2	777.3	703.6	420	458.9	512.1	463.7	429	381.6	419	427	405	10
Os02g0766700	1347	1260	1072	342.3	223.3	485.3	592	367	450	632	595.3	541.9	527.93	575.01	590.81	883.6	1038	1005	406	520.1	511.3	406.7	451.8	384.2	582.1	688.3	745.6	704	730	629	14
Os02g0606100	398	431	386	503.3	515.3	454.2	469	496	558	462.1	475.8	566.4	511.6	486.33	430.83	764.2	388.5	374	517.4	558.8	466.5	454	451.1	455.7	483.8	475.2	526.3	408	405	387	6
Os02g0325401	0	0	0	0	0	0	0	0	0	0	0	0	0	0	0	0	0	0	0	0	0	0.739	0	0	0	0	0	0	0	0	9
Os02g0561500	0	0	0	0.821	0	0.865	0.78	0.63	0	0.862	0	0	1.0885	0.9335	0.792	0	4.122	1.036	0	0	0	0	0	0	0	0	0	0	0.96	1.67	1
Os02g0236800	17.3	35.4	23.7	130.5	128.4	92.56	283	219	220	17.24	39.47	36.69	15.239	15.869	11.879	0	18.55	14.5	123.6	100.8	110.4	146.4	100.5	205.8	16.9	25.46	24.12	13	23	14.2	18
Os02g0307000	987	984	989	1118	1084	1145	705	848	683	868.2	775.2	884.4	940.48	977.32	918.68	1278	1007	971.8	760.8	828.1	890.7	781.5	897.9	866	1013	848.6	835.5	1115	978	1033	8
Os02g0453000	233	319	280	305.4	330.9	343.4	212	240	178	166.4	175.4	125.1	191.58	195.09	191.66	298.5	211.3	262.1	218.3	260.4	186.2	264.7	297.9	252.8	219.7	170.7	263.2	297	230	259	8
Os02g0669000	18.4	20.5	10.8	12.31	19.89	13.84	32.9	39.8	27.6	23.28	10.96	30.11	20.682	26.137	6.3357	0	21.64	22.79	5.574	4.031	9.484	19.22	16.39	22.39	11.62	16.03	17.54	9.25	8.61	2.5	5
Os02g0759000	12.2	5.59	4.31	1.642	0	0	3.91	5.06	8.29	25.87	17.54	12.23	11.974	15.869	19.799	35.82	24.73	10.36	0	1.613	2.587	2.958	4.988	6.5	41.2	46.2	19.74	14.8	20.1	12.5	3
Os02g0571100	292	202	199	105.1	109.4	216.3	160	102	144	214.7	242.3	324.6	77.285	196.02	181.36	191.1	416.3	374	155.1	143.5	241.4	503.5	295	370.5	277.8	393.2	357.4	384	263	285	1
Os02g0733200	199	126	140	151.9	161.8	164.4	169	136	139	163	152.4	165.6	139.33	136.28	121.96	107.5	148.4	165.8	231.3	175	219.9	107.2	119	127.1	128.9	158.4	168.9	128	128	105	16
Os02g0722800	607	628	620	648.6	618.3	570.1	454	469	482	570.8	538.3	494.9	630.25	506.86	665.25	812	519.4	552.2	496.1	466.8	454.4	601.1	600	507.7	538.7	540.2	438.6	609	567	660	8
Os02g0480200	327	344	284	351.4	352.5	388.4	304	350	368	316.4	341	370.7	392.96	353.78	346.88	406	357.6	403	444	405.6	429.4	293.5	281.5	322.1	372.9	374.3	377.2	362	396	353	16
Os02g0694400	659	687	691	634.6	605.1	545	445	482	521	539.7	560.4	420.5	666.17	696.35	822.85	1158	702.8	740.8	527.6	619.2	476.8	720.2	617.1	499.1	667.6	632.6	614	903	768	855	13
Os02g0543100	32.6	20.5	26.9	18.06	20.79	25.95	28.2	38	26.3	26.73	36.18	27.28	23.947	21.469	22.967	23.88	40.19	33.15	26.94	32.25	42.25	41.41	23.52	34.67	25.35	21.69	19.74	23.1	38.3	28.3	9
Os02g0198400	5.1	11.2	5.39	11.49	5.424	7.785	15.7	13.3	11.1	4.311	6.578	4.704	3.2656	5.6007	3.1679	0	0	13.47	4.645	2.419	6.898	14.79	19.24	15.17	12.68	7.543	6.579	5.55	5.74	10.8	17
Os02g0824500	22.4	10.2	18.3	10.67	18.08	6.92	10.2	9.49	9.67	11.21	18.64	11.29	26.124	51.34	12.671	23.88	20.61	15.54	10.22	3.225	6.898	5.915	10.69	10.83	19.01	19.8	8.772	40.7	25.8	26.7	3
Os02g0527800	1.02	0.93	1.08	0	1.808	0	0	0.63	4.15	0	0	0	0	0.9335	0	0	0	0	0	1.613	0	0.739	4.276	3.611	0	0	0	0	0	0	17
Os02g0817400	0	0	0	0	0.904	0	0.78	0	0	0.862	0	0	0	0	0	0	3.092	0	0	1.613	0	0	1.425	0	1.056	0	0	0	0.96	0.83	1
Os02g0814600	23.4	31.7	26.9	36.12	17.18	32.87	13.3	17.1	11.1	5.173	16.45	10.35	11.974	18.669	21.383	71.65	21.64	14.5	21.37	18.54	23.28	24.4	10.69	16.61	9.507	12.26	19.74	14.8	28.7	15	6
Os02g0478600	299	200	235	260.2	231.4	304.5	482	329	444	306.1	274.1	572	275.4	286.57	212.25	95.53	168	200	405	372.5	378.5	258.8	255.8	242.7	202.8	344.1	425.4	168	227	123	15
Os02g0737401	2.04	11.2	7.54	1.642	2.712	1.73	6.26	4.43	0.69	26.73	5.482	11.29	3.2656	14.002	5.5438	0	5.153	12.43	0	1.613	3.449	3.697	4.988	4.333	10.56	5.657	8.772	0.93	0.96	0.83	7
Os02g0123500	938	1100	914	1489	1804	1347	1858	1946	2012	1029	991.1	1124	1112.5	1070.7	946.4	764.2	777	788.4	1417	1299	1348	1253	1363	1426	941.2	781.6	879.4	760	826	906	12
Os02g0215400	8.16	25.1	12.9	13.14	16.27	13.84	33.7	15.2	22.8	25	27.41	19.76	16.328	20.536	21.383	23.88	12.37	19.68	24.15	21.77	19.83	27.36	15.68	13	28.52	22.63	13.16	29.6	17.2	12.5	5
Os02g0613900	13.3	2.79	12.9	33.66	11.75	18.17	2.35	5.06	11.1	39.66	17.54	44.22	10.885	7.4676	4.7518	11.94	10.31	19.68	147.7	30.64	34.49	41.41	28.51	41.17	10.56	33.94	74.56	10.2	14.3	6.66	16
Os02g0768600	4118	3933	3548	3928	4411	3651	7258	6419	6764	4464	4489	6002	3277.5	3857	3148.9	4764	3639	3849	4920	4247	5058	3894	3818	5470	3590	4336	4776	2412	2582	2484	15
Os02g0110100	1.02	1.86	0	0	0	0	0	0	0	2.587	0	0	0	0	0	0	0	1.036	0	0	0	0	0	0	0	0	0	0	0	0	7
Os02g0127600	93.8	80.1	83	156.8	138.3	121.1	54.8	82.9	80.8	64.66	41.66	68.68	59.869	54.14	56.23	131.4	99.96	82.88	113.3	85.47	89.67	88.73	111.9	135.8	66.55	90.51	83.33	73.1	86.1	98.3	2
Os02g0258000	73.4	77.3	83	82.1	50.62	87.37	83	81.6	86.4	121.6	116.2	160.9	83.816	122.28	122.75	95.53	205.1	119.1	133.8	95.95	196.6	58.41	84.8	68.61	233.5	187.6	203.9	164	120	137	20
Os02g0185200	416	428	417	197.9	169	222.3	35.2	62.6	27.6	325	309.2	255.9	379.89	354.71	402.32	429.9	339	327.4	111.5	130.6	91.4	127.2	200.3	176.9	573.6	437.5	552.6	593	475	557	3
Os02g0590700	7.14	20.5	10.8	18.06	41.58	39.79	25.8	24	21.4	6.897	15.35	13.17	5.4426	6.5342	14.255	35.82	4.122	13.47	13.01	19.35	22.42	31.79	20.67	19.5	5.282	10.37	2.193	5.55	10.5	14.2	12
Os02g0622300	36.7	23.3	20.5	13.96	17.18	23.36	15.7	10.1	24.2	20.69	17.54	38.57	28.302	40.138	17.423	0	24.73	30.05	39.94	27.41	32.76	22.92	12.11	16.61	14.79	34.89	35.09	13	12.4	7.5	20

Os02g0508100	3.06	0.93	3.23	0	0	0	0	0	0	0	0	0	3.2656	1.8669	0.792	0	8.244	2.072	0.929	0	0	0	1.425	0	1.056	3.771	8.772	0.93	2.87	2.5	11
Os02g0622100	249	318	216	303.8	314.6	308	347	386	334	264.7	271.9	216.4	272.13	208.16	300.95	298.5	221.6	248.6	274	283.8	225	269.9	299.3	327.9	264.1	172.5	199.6	250	274	239	18
Os02g0529600	311	264	279	258.6	259.4	289.8	305	360	286	271.6	257.7	259.7	201.38	205.36	229.67	214.9	259.7	219.6	255.5	262	223.3	289.1	302.2	333	240.9	236.7	296	228	241	225	18
Os02g0121300	####	####	####	31368	30804	36184	9482	8903	9374	37625	30876	51706	30010	42156	31797	35560	44236	39593	41003	31589	40460	7636	8661	7687	34293	42913	43494	####	####	####	3
Os02g0666300	648	680	696	604.2	711.4	508.6	604	774	705	493.2	551.5	439.4	690.12	481.66	511.61	644.8	495.7	601.9	452.4	555.5	494.9	615.2	535.9	542.4	468	414.9	368.4	475	545	572	18
Os02g0683500	1.02	1.86	2.16	4.105	5.424	0.865	0	0.63	0	0.862	1.096	0	0	0.9335	1.5839	0	0	3.108	0.929	0	0	8.873	2.851	2.167	1.056	1.886	0	0.93	0	3.33	17
Os02g0193425	115	136	165	15.6	11.75	6.92	11	17.7	10.4	130.2	231.3	169.3	216.62	207.23	221.75	179.1	185.5	153.3	21.37	18.54	11.21	5.915	7.126	5.056	197.5	158.4	103.1	123	142	157	3
Os02g0205500	339	483	297	62.39	50.62	49.31	42.3	67.7	38.7	119.8	54.82	58.33	83.816	70.942	85.532	83.59	107.2	165.8	81.75	95.14	56.91	51.76	84.8	49.83	51.76	33.94	37.28	51.8	76.5	65.8	14
Os02g0187000	2.04	0.93	1.08	9.852	5.424	4.325	4.7	3.16	1.38	0.862	0	0	0	0	0	0	2.061	1.036	4.645	5.644	4.311	5.915	5.701	5.056	0	0.943	0	0	1.91	0	12
Os02g0755900	0	2.79	8.62	26.27	18.98	36.33	0.78	5.69	5.53	12.07	3.289	10.35	19.593	22.403	13.463	0	5.153	2.072	7.432	7.257	0.862	3.697	4.276	0	7.395	15.09	10.96	4.63	5.74	4.17	2
Os02g0831500	19.4	6.52	12.9	8.21	10.85	7.785	7.83	20.9	8.29	4.311	7.675	16.93	13.062	9.3345	11.088	0	15.46	7.252	9.29	7.257	16.38	16.27	4.276	16.61	13.73	16.03	28.51	12	9.56	10.8	11
Os02g0700300	132	107	85.1	32.84	21.7	32.01	13.3	9.49	10.4	27.59	29.6	41.4	32.656	42.939	34.055	95.53	106.1	98.42	33.44	18.54	22.42	19.96	32.07	20.95	75	89.57	65.79	52.7	59.3	78.3	14
Os02g0780600	172	200	214	267.6	264.9	302.8	263	254	250	263	206.1	187.2	265.6	243.63	225.71	441.8	188.6	208.2	236	300.7	291.4	224.8	233.7	200.8	216.6	198.9	217.1	213	202	240	6
[truncated: 4,694,001 more chars]
